# Identification of immune subtypes associated with CD8+ T cell-related genes providing new treatment strategies of esophageal carcinoma

**DOI:** 10.3389/fimmu.2025.1512230

**Published:** 2025-02-27

**Authors:** Youyi Wu, Chen Lin, Yuchen Qian, Xiaowei Huang, Yajing Xu, Jiayi Li, Youdi He, Congying Xie, Huafang Su

**Affiliations:** ^1^ Department Oncology Radiotherapy, The Third Affiliated Hospital of Wenzhou Medical University, Rui’an People Hospital, Ruian, Zhejiang, China; ^2^ Department of Radiation Oncology, The First Affiliated Hospital of Wenzhou Medical University, Wenzhou, China; ^3^ Zhejiang Key Laboratory of Intelligent Cancer Biomarker Discovery and Translation, First Affiliated Hospital of Wenzhou Medical University, Wenzhou, China; ^4^ Department of Radiation Oncology Wenzhou Central Hospital Theorem Hospital Affiliated of Wenzhou Medical University, Wenzhou, China

**Keywords:** immune subtype, CD8+ T cell, CHMP7, WGCNA, esophageal carcinoma

## Abstract

**Background:**

CD8+ T lymphocytes greatly affect the efficacy of immunotherapy, displaying promising potential in various tumors. Here, we aimed to identify immune subtypes associated with CD8+ T cell-related genes to predict the efficacy of treatment in esophageal cancer (ESCA).

**Methods:**

We obtained 13 immune cell-related datasets from the Gene Expression Omnibus (GEO) database and removed batch effects. Weighted correlation network analysis (WGCNA) and co-expression analysis were performed to identify highly correlated CD8+ T cell genes. Cox analysis was used to process ESCA clinical information, and the immune clusters (ICs) were constructed through consensus cluster analysis. Furthermore, we constructed an immune risk score model to predict the prognosis of ESCA based on these CD8+ T cell genes. This model was verified using the IMvigor210 dataset, and we functionally validated the immune risk score model *in vitro*.

**Results:**

The results revealed significant correlations between CD8+ T cell-related genes and immune-related pathways. Three ICs were identified in ESCA, with IC3 demonstrating the most favorable prognosis. The final 6-gene prognostic risk model exhibited stable predictive performance in datasets across different platforms. Compared with that in normal esophageal epithelial (HEEC cells), CHMP7 in the 6-gene prognostic risk model was upregulated in KYSE150 and TE-1 cells. Si-CHMP7 transfection led to a decrease in tumor cell migration, invasion, and proliferation, accompanied by an accelerated apoptotic process.

**Conclusions:**

Collectively, we identified the immune subtypes of CD8+ T cell-related genes with different prognostic significance. We designated CHMP7 in the 6-gene prognostic risk model as a potential target to improve tumor cell prognosis. These insights provide a strong basis for improving prognosis and facilitating more personalized and accurate treatment decisions for the immunotherapy of ESCA.

## 1 Introduction

Esophageal cancer (ESCA) exhibits a poor prognosis and high mortality rate, ranking seventh in terms of incidence and sixth in mortality overall according to global cancer statistics in 2020 (1). The incidence rate of ESCA is high in Africa, Southeast Asia (especially in China), and South America (1). Successful treatment of early ESCA can be achieved through endoscopic resection (2), while locally advanced cases require surgery combined with radiotherapy and chemotherapy (3). Late diagnosis is a common issue, with approximately 70–80% of resected specimens in North America showing metastases in regional lymph nodes (4). To improve the overall survival of advanced or metastatic ESCA, which currently has a median survival of less than one year (5), new treatments for ESCA are urgently needed.

Immune checkpoint blockers (ICBs), programmed cell death protein 1 (PD-1)/programmed cell death ligand 1(PD-L1) antibodies (trastuzumab, ramucirumab, and pembrolizumab), have been effective in enhancing the survival of patients with ESCA. However, PD-L1 expression has been observed in only about 40% of patients with ESCA (6). A more comprehensive understanding of the heterogeneity of the immune response is essential for selecting the most suitable immunotherapy for ESCA.

Existing studies have analyzed transcriptomics, epigenetics, and immunohistochemistry data of ESCA, leading to the identification of subtypes of ESCA related to the prognosis of patients (7, 8). Xie Y et al. used unsupervised learning to identify immune subtypes (ISs) of ESCA in publicly available data, revealing the potential for targeted immunotherapy based on different ISs (9). Further studies have shown that increased PD-L1 expression in ESCA correlates with a decrease in the number of tumor-infiltrating lymphocytes (10). The primary manifestation of lymphopenia is observed in CD8+ T cells, leading to diminished patient survival rates (11). Hence, we aimed to explore the relationship between CD8+ T cells and ESCA immunotherapy.

In this study, we selected CD8+ T cell-related genes from the immune cell data set. Subsequently, we used the single-sample gene set enrichment analysis (ssGSEA) method to evaluate immune characteristics based on the CD8+ T cell-related genes and classified ESCA into different ISs through ConsensusClusterPlus. Finally, the ESCA risk model was constructed based on CD8+ T cell genes and verified using the immunotherapy dataset IMvigor210.

## 2 Materials and methods

### 2.1 Data source and processing

**Figure 1:**
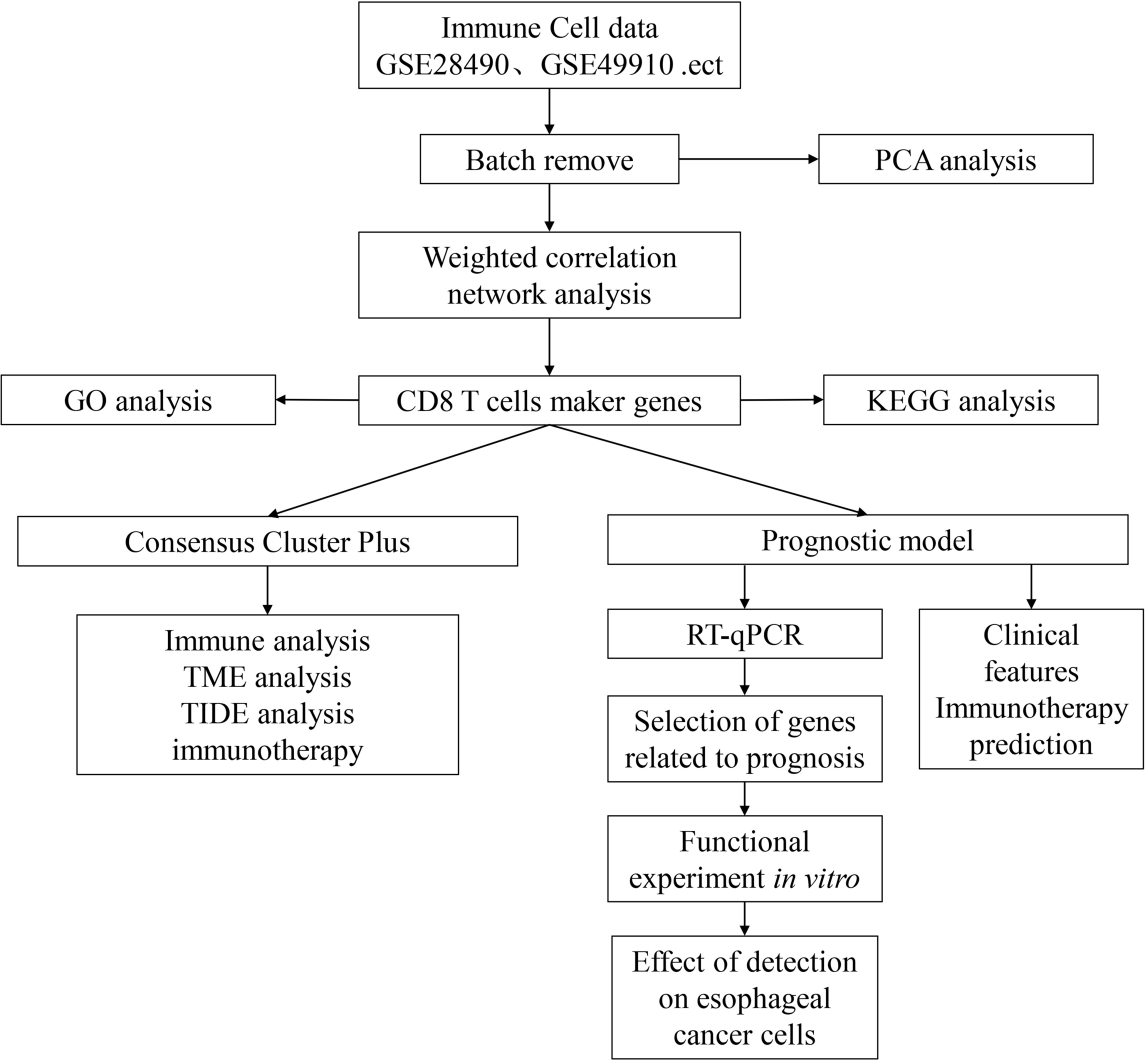
Technology roadmap of this study.

The gene expression profile, tumor mutation burden (TMB), and clinical follow-up information data of the 160 ESCA cancer samples were downloaded from The Cancer Genome Atlas (TCGA) database (https://portal.gdc.cancer.gov). The validation cohort (n = 70) was obtained from the GEO database (GSE54993). The clinical characteristics of TCGA-ESCA and GSE54993 are presented in **Table 1** and **Supplementary Table S1**, and TCGA training and validation sets are shown in **Supplementary Table S2**. The IMvigor210 cohort containing transcriptome data was downloaded from the website (http://research‐pub.gene.com/IMvigor210CoreBiologies). The immune cell-related datasets were derived from the NCBI Gene Expression Omnibus (GEO) data portal (https://www.ncbi.nlm.nih.gov/geo/query/acc.cgi), including GSE13906, GSE23371, GSE27291, GSE27838, GSE28490, GSE28726, GSE37750, GSE39889, GSE42058, GSE49910, GSE59237, GSE6863, and GSE8059. These datasets included gene expression data for 14 immune cells (**Supplementary Table S3**). The data were processed using the “RMA” function in the R package “affy” (12), followed by the application of the function “removeBatchEffect” in the R package “limma” (13). The flow chart of this article is shown in **Figure 1**.

**Table 1:** Detail clinical pathological features for TCGA-ESCA and GSE54993 cohorts.

Clinical Features	TCGA-ESCA	GSE54993
OS		
0	97	34
1	63	36
T Stage		
T1	25	4
T2	41	2
T3	87	64
T4	5	
TX	2	
N Stage		
N0	64	32
N1	70	24
N2	9	11
N3	5	3
NX	12	
M Stage		
M0	128	70
M1	15	
MX	17	
Stage		
I	16	5
II	71	29
III	55	36
IV	14	
X	4	
Gender		
Male	137	57
Female	23	13
Age		
≤ 60	82	44
>60	78	26

### 2.2 Analytical methods

The WGCNA (14) package in R software was applied to construct an immune-related gene co-expression network to identify significant gene modules. The clusterProfiler (15) package in R software was used to implement the Kyoto Encyclopedia of Gene and Genomes (KEGG) pathway and gene ontology (GO) analysis. The ConsensusClusterPlus (16) package in R software was used for consensus cluster analysis to determine the number of subtypes in ESCA samples. The rationality of clustering was verified through the resampling method. We finally determined the optimal number of clusters by considering the cumulative distribution function (CDF) and delta region graphs. The ssGSEA method was performed to quantify the infiltration levels of immune cell types, functions, and pathways in the cancer samples, using the GSVA package (17) in R software. Additionally, the Cell-type Identification by Estimating Relative Subsets of RNA Transcripts (CIBERSORT) algorithm was applied to explore the infiltration degrees of 22 immune cell types in esophageal carcinoma (18). The microenvironment cell populations counter (MCPcounter) analysis method was used to determine the level of tumor-infiltrating immune cells with the MCPcount package (19) in R software (20). The correlation analysis used the corr.test() function in R, when adjust=“bonferroni”, output the validated P value.

### 2.3 Construction and validation of a prognostic risk model

The tumor immune dysfunction and exclusion (TIDE) score was computed online (http://tide.dfci.harvard.edu/). The two main mechanisms of tumor immune evasion were the induction of T cell dysfunction in tumors with high cytotoxic T lymphocyte (CTL) infiltration, and the prevention of T cell infiltration in tumors with low CTL levels. Identify genes that affect cytotoxic T cell function on patient survival outcomes based on immune escape mechanisms. The z-score for each gene was the interaction coefficient d divided by its standard error. The accessible data from patients in GSE78220 treated with immunotherapies was used to predict the clinical response using the subclass mapping method. Additionally, the Genomics of Drug Sensitivity in Cancer (GDSC; https://www.cancerrxgene.org/) was used to predict the efficacy of the three subtypes with chemotherapeutic drugs. The univariate Cox regression analysis was used to screen for prognostic differential genes with a significance level of p<0.05, employing the “coxph” function of the “survival” package in R. Subsequently, a multivariate Cox regression analysis, utilizing stepwise regression in our study, was applied to further select significant genes using the “stepAIC” function of MASS package (21) in R. This process started with the most complex model and then successively deleted variables to reduce AIC based on AIC Akaike information guidelines. The Kaplan–Meier survival curve was used to analyze the survival difference between the low- and high-risk groups using the “survminer” R package. The predictive accuracy of the risk model was evaluated using the “timeROC” package in R. The “timeROC” package had a built-in function, when adjusted=TRUE, output the validated P value (22). TIMER2.0 (http://timer.cistrome.org/) was used to explore the correlation of CHMP7 with immune invasion in esophageal cancer.

### 2.4 Cell culture

Normal esophageal epithelial (HEEC cells) and ESCA (KYSE150 and TE-1 cells) cell lines were purchased from the cell bank of the Chinese Academy of Sciences (Shanghai, China). They were cultured in RPMI1640 medium containing 10% FBS and 1% penicillin-streptomycin, maintained in a humidified incubator containing 5% CO_2_ at 37°C. For each experiment, cells in the logarithmic phase were used.

### 2.5 Cell transfection

We used Lipofectamine2000 (Invitrogen, USA) to transfect CHMP7-siRNA (Si-CHMP7#1: GGAGGTGTATCGTCTGTAT; Si-CHMP7#2: CAAGGTCTCTCCAGTCAAT; Si-CHMP7#3: GAGTGAACAGCTTCTCTCA); pcDNA3.1-CHMP7(RiboBio, China) and NC-siRNA (RiboBio, China) into cells. The transfection efficiency was assessed using RT-qPCR 48 h after transfection.

### 2.6 RT-qPCR

The cells were collected, and the total RNA was extracted using Trizol (Invitrogen, USA). Subsequently, 1 µg of total RNA was reverse transcribed into cDNA using the HiScript II Q RT SuperMix for qPCR kit (Vazyme, China). RT-qPCR reaction was conducted on different samples using the Taq Pro Universal SYBR qPCR Master Mix kit (Vazyme) for a total of 40 cycles. The reaction conditions included a 95°C initial denaturation for 10 s, followed by 30 s at 10°C, using the QuantStudio5 real-time fluorescence quantitative PCR detection system. The sequences of all primers are listed in **Table 2**.

**Table 2:** Sequences of all primers.

Genes	Primer	Sequences (5’-3’)
GAPDH	Sense	GGTGGTCTCCTGTGACTTCAA
	Antisense	CCACCCTGTTGCTGTAGCC
PIK3R1	Sense	TGGAAGCAGCAACCGAAACAAAG
	Antisense	CCACCACTACAGAGCAGGCATAG
RCAN3	Sense	GCGAATAGAACTCCACGAAACAGAC
	Antisense	GCGGCAGGAGATAGGACTTGTC
CHMP7	Sense	TGAAGCCTCTCAAGTGGACTCTTTC
	Antisense	GATACAGACGATACACCTCCTCAGC

### 2.7 Scratch test

When the cell fusion degree reached 90%, a straight line was drawn in the cell petri dish with the 1000 µl pipette. It was rinsed with PBS, and 1640 medium containing 2% FBS was added. Images under the microscope were observed and captured at 0 and 24 h.

### 2.8 Transwell assay

The cells were collected, and 300 µl serum-free medium was added to the upper chamber with matrix glue. Medium containing 10% FBS was added to the lower chamber, with 50,000 cells in each well. After 24 h of culture, the medium was discarded, and the cells were fixed with 4% paraformaldehyde for 30 min. They were stained with 2% crystal violet for 10 min, followed by rinsing with PBS several times, drying, observing under a microscope, and capturing the images.

### 2.9 CCK8 assay

After collecting cells, 1000 cells were added to each well of four 96-well plates. At 0, 24, 48, and 72 h after cell attachment, 10 μl of CCK8 reagent was added. After 4 h of incubation, the OD value was measured using an enzyme labeling instrument. The daily OD values were compared with the OD value obtained at 0 h, and the curve was obtained to evaluate the cell proliferation rate.

### 2.10 Western blot analysis

The total cell protein was extracted, and the protein concentration was determined. The protein was separated using 10% SDS-PAGE and transferred to a PVDF membrane. After 2 h of incubation at room temperature, diluted antibody was added and allowed to incubate overnight at 4°C. Following the removal of the primary antibody and washing the next day, the corresponding secondary antibody was added and incubated at room temperature for 1 h. The protein band images were captured using a gel imaging system with ECL, and ImageJ was used for quantitative analysis. The protein indicators tested were BCL2 (Proteintech, China), BAX (Proteintech), and β-actin (Proteintech).

### 2.11 Statistical analysis

The statistical analysis was primarily conducted using the R programming language. The chi-squared test was used to compare differences between groups for categorical variables. For non-normally distributed variables, the Wilcoxon rank-sum test was conducted. ANOVA and Kruskal–Wallis test were selected for comparing more than two groups. The log-rank test was performed to evaluate the statistical differences in overall survival among different groups in the Kaplan–Meier survival analysis. The statistical significance was set at p<0.05 or p<0.01.

## 3 Results

### 3.1 Identification of marker genes for CD8+ T cells

**Figure 2:**
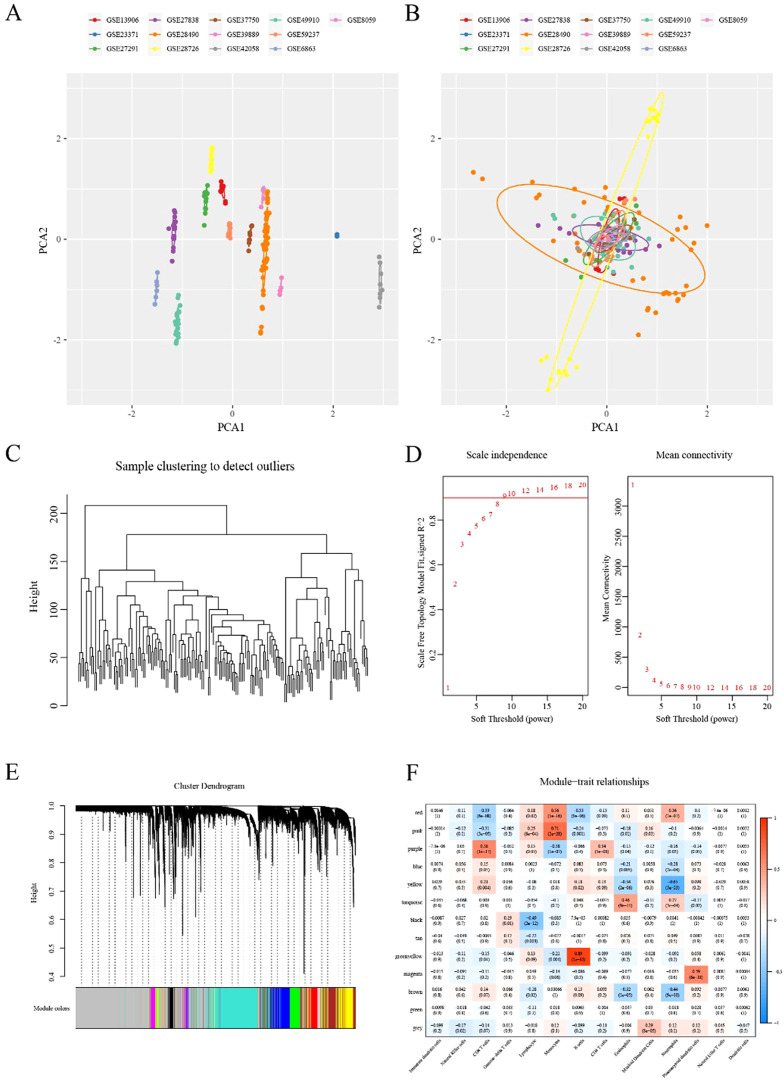
Identification of the marker genes of CD8+ T cells in ESCA **(A)** PCA scatter plot of immune datasets before removing batch effects. **(B)** PCA scatter plot of immune datasets after removing batch effects. **(C)** Cluster analysis on 179 expression profiles of immune cell dataset. **(D)** Analysis of network topology for various soft-thresholding powers. **(E)** Gene dendrogram and corresponding module colors. **(F)** Correlation heatmap of 13 modules and various clinical phenotypes.

Given the expression of CD8+ T cells may influence the survival of esophageal carcinoma patients, we investigated the CD8+ T cell-related prognosis in esophageal carcinoma. Thirteen immune cell datasets were merged to form a single dataset with batch effects eliminated. Data before and after normalization were explored using principal component analysis (PCA). The transition from scattered datasets to a mixed state indicated the successful application of the “removeBatchEffect” function (**Figure 2A, B**). Following the normalization of the gene expression profile, hierarchical clustering was performed on samples based on the 179 expression profile data of the immune cell datasets (**Figure 2C**). The Pearson correlation coefficient was then used to calculate the distance between each gene. Using the aforementioned WGCNA method, we constructed a scale-free network with a determined soft threshold of 9 (**Figure 2D**), resulting in the identification of 13 gene modules (**Figure 2E**). The grey module comprised genes that could not be aggregated into other modules. We further analyzed the correlations between each module and immune cells (**Figure 2F**). The results indicated that the purple module, containing 346 genes, was the most positively correlated with CD8+ T cells and had little correlation with other immune cells. The genes of all the modules are listed in **Supplementary Table S2**.

Subsequently, we performed functional enrichment analysis on CD8+ T cell-related genes belonging to the purple module. For GO analysis, 103 biological processes (BP) showed significant differences (FDR<0.05; **Supplementary Table S3**), with the top ten BP displayed in **Supplementary Figure S1A**. Eight cellular components (CC) demonstrated significant differential enrichment (FDR<0.05; **Supplementary Figure S1B**, **Supplementary Table S3**), and 12 molecular function (MF) signatures were significantly differentially enriched (FDR<0.05; **Supplementary Table S3**), with the top ten presented in **Supplementary Figure S1C**. In KEGG analysis, eight pathways were significantly differentially enriched (FDR<0.05; **Supplementary Figure S1D**, **Supplementary Table S3**). The findings suggested that these genes were closely related to immune functions and pathways.

### 3.2 Molecular subtyping based on CD8+ T cell-related genes

**Figure 3:**
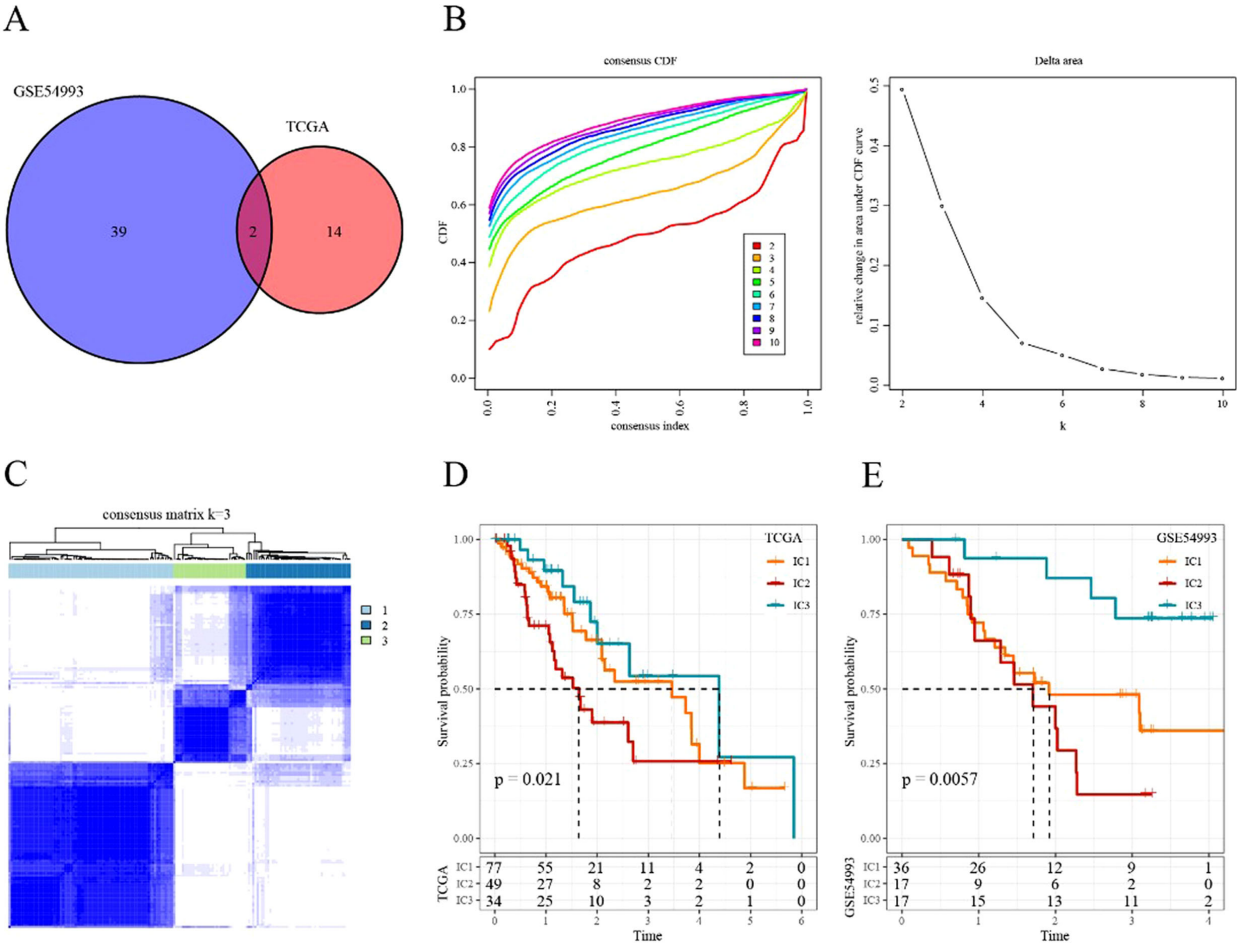
Immune cluster in ESCA **(A)** Intersectional Venn diagram of CD8+ T cell-related genes with significant prognosis in two cohorts (TCGA and GEO). **(B)** CDF curve and CDF Delta area curve of consensus clustering in TCGA cohort. The CDF Delta area curve indicates the relative change in the area under the CDF curve for each category number k compared with that for k-1. **(C)** Sample clustering heatmap when consensus k = 3. **(D)** Kaplan–Meier survival curve of the three immune subtypes in TCGA cohort. **(E)** Kaplan–Meier survival curve of the three immune subtypes in the GSE54993 cohort.

We conducted univariate analysis separately on genes related to CD8+ T cells in TCGA-ESCA and GSE54993 datasets. The results revealed 16 and 41 genes related to prognosis in TCGA (**Supplementary Table S4**) and GSE54993 (**Supplementary Table S5**), respectively. Only two genes were common to both cohorts (**Figure 3A**), indicating limited consistency in CD8+ T cell-related genes among datasets of different platforms. Therefore, we selected 55 CD8+ T cell-related genes, identified as prognostic genes in two datasets, for further analysis (p<0.05).

We applied consensus clustering to categorize 160 patients from TCGA-ESCA cohort. The clustering results remained relatively stable with three clusters (**Figure 3B**). We selected k = 3 as the optimal number, leading to three CD8+ T cell-related immune clusters (ICs; **Figure 3C**, **Supplementary Table S6**). Analyzing the prognostic signature of these three subtypes revealed significant differences (**Figure 3D**), with IC2 displaying the poorest outcomes and IC3 demonstrating generally favorable outcomes. Similar results were observed in the GSE54993 cohort (**Figure 3E**, **Supplementary Table S7**), suggesting the transplantability of the three molecular subtypes based on CD8+ T cell-related genes across different cohorts.

We compared the distribution of various clinical features (survival events, TNM stages, stage, age, and gender) among the three immune subtypes in TCGA-ESCA cohort to investigate the differences among them during analysis. The results indicated a significant difference in the proportion of T stages among the three subtypes, with the IC2 group having the highest proportion of T3, indicating a poor prognosis. No other significant clinical differences were observed among the three immune subtypes (**Supplementary Figure S2A-G**).

### 3.3 Comparative genomic profiling of immune subtypes

**Figure 4:**
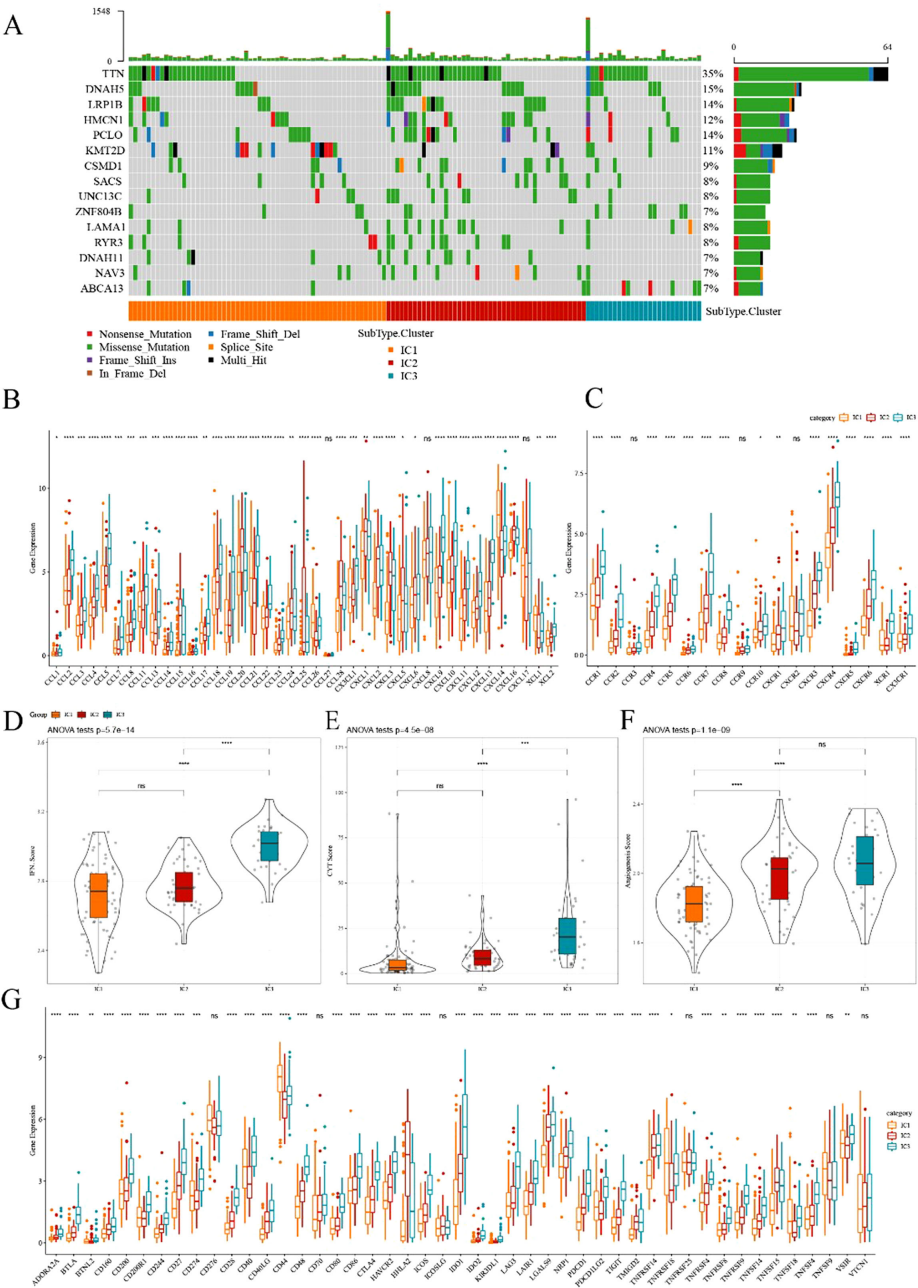
Comparison of mutational analysis of ESCA immune subtypes **(A)** Mutational landscape of the top 15 significantly mutated genes in samples of various immune subtypes. **(B, C)** Distribution of expression levels of chemokines and chemokine receptors across three immune subtypes in TCGA cohort, respectively. **(D–F)** Distribution of IFN-γ score, immune T cell lysis activity, and angiogenesis score in three ICs, respectively. **(G)** Differences in the expression levels of immune checkpoint genes in TCGA cohort. The significance was statistically tested through ANOVA analysis; *p<0.05, **p<0.01, ***p<0.001, ****p<0.0001. ns, no significance.

No significant difference was observed in TMB and the number of mutated genes among the three subgroups (**Supplementary Figure S3A, B**). Furthermore, we screened 1224 genes with a mutation frequency greater than three (**Supplementary Table S8**). Then, 124 genes with high mutation frequency in each subtype were identified (p<0.05; **Supplementary Table S9**). The mutation signatures of the top 15 genes are depicted in the mutation heatmap in **Figure 4A**.

Previous research has suggested that chemokines play a pivotal role in tumorigenesis and tumor development (23). Chemokines can attract various immune cells into the tumor microenvironment, providing T cells access to the tumor and influencing both tumor immunity and therapeutic effects. In this study, we analyzed whether there were differences in the expression distribution of chemokines among immune subtypes based on TCGA-ESCA cohort. Thirty-eight out of 41 chemokines significantly differed in expression among subtypes (**Figure 4B**), indicating variations in the level of immune cell infiltration among different immune subtypes. These differences could contribute to distinctions in tumor progression and immunotherapy efficacy. Additionally, the expression of chemokine receptor genes was compared, and 15 out of 18 (83.88%) significantly differed among immune subtypes (**Figure 4C**).

CD8+ T cells in the tumor microenvironment can produce interferon-γ (IFN-γ), which stimulates the upregulation of PD-1/PD-L1 and IDO1 (24). We obtained the Th1/IFN-γ gene signatures from previous research (25) and calculated the IFN-γ score of each patient using the ssGSEA method. We found significant differences among immune subtypes in IFN-γ scores. IC3 subtype exhibited a relatively high IFN-γ score, whereas the opposite trend was observed for IC1 and IC2 groups (**Figure 4D**). Moreover, intra-tumoral immune T cell lysis activity was assessed using the average of *GZMA* and *PRF1* expression values, according to a previous study (26). Significant differences were observed among the three subgroups (**Figure 4E**). IC1 and IC2 exhibited relatively lower immune T cell lysis activity, whereas IC3 displayed the opposite trend.

Similarly, the angiogenesis score of each patient was evaluated based on an angiogenesis-related dataset derived from a previous study (27). The results suggested that different immune subtypes differed in angiogenesis score, with IC2 and IC3 exhibiting higher scores than IC1 (**Figure 4F**). Moreover, we obtained 47 immune checkpoint-related genes from prior research, and a significant difference was observed in 41 genes (**Figure 4G**) through further exploration. Immune checkpoint-related genes such as *LAG3*, *CTLA4*, *PDCD1*, *PDCD1LG2*, and *IDO1* were highly expressed in IC3. These results indicated that different subgroups may exhibit varying responses to immunotherapy.

### 3.4 Immune and pathway signatures in different immunotypes

**Figure 5:**
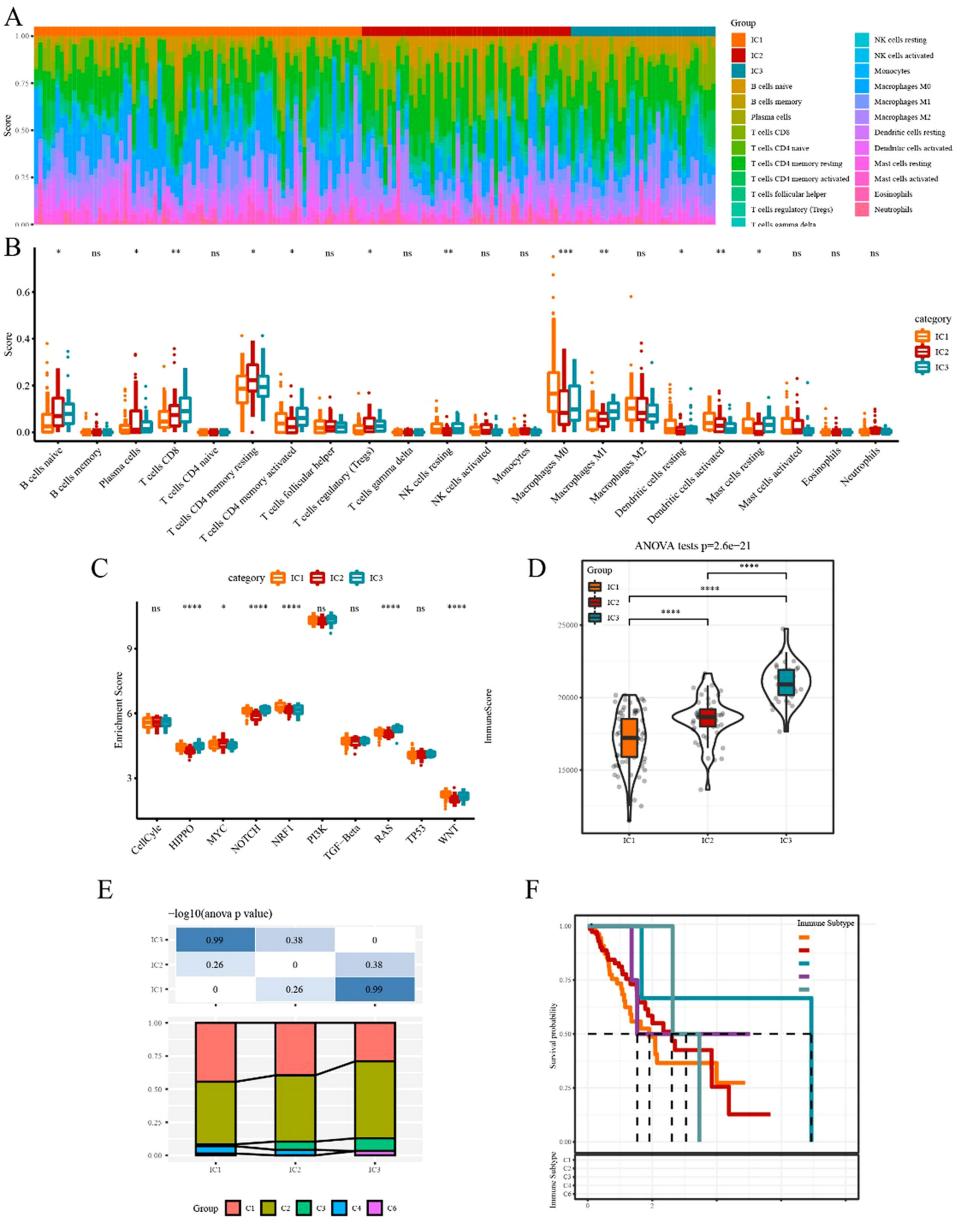
Immune and pathway signatures in different immune subtypes **(A)** Proportion of 22 immune cells in samples of different subtypes. **(B)** Differences in 22 immune cell components among different immune subgroups. **(C)** Comparison of enrichment scores of ten oncogenic pathways among immune subtypes. **(D)** Difference in immune infiltration scores among different subgroups. **(F)** Comparison of our molecular subtypes with the previous six pan-cancer immune subtypes. **(F)** Kaplan–Meier survival curve of the six pan-cancer immune subtypes. *p<0.05, **p<0.01, ***p<0.001, ****p<0.0001. ns, no significance.

The CIBERSORT method was used to evaluate the infiltration scores of 22 immune cells in each TCGA-ESCA dataset sample. Overall, significant differences in immune signatures were observed among different subgroups. Additionally, CD8+ T cells, resting memory CD4+ T cells, and M0, M1, and M2 macrophages were highly expressed in each subtype, suggesting their potential role in ESCA (**Figure 5A, B**). By analyzing the differences in ten oncogenic pathways proposed in a previous study (28), six pathways were found to be significantly different between immune subtypes (**Figure 5C**). Subsequently, the immune infiltration analysis revealed that IC3 exhibited the highest immune microenvironment infiltration score, whereas IC1 had the lowest score (**Figure 5D**). Moreover, most of the immune checkpoint-related genes had the highest expression in IC3, possibly contributing to the favorable outcome of IC3.

To explore the relationship between our immune subtypes and the six pan-cancer immunotypes studied previously, we collected the data of molecular subtypes from previous research (29) for comparison. Our subgroups significantly differed from the six immunophenotype groups in the previous study (**Figure 5E, F**). The three subtypes we defined could serve as a supplement to the existing six subtypes.

### 3.5 Comparative response of immune subtypes to immunotherapy or chemotherapy

**Figure 6:**
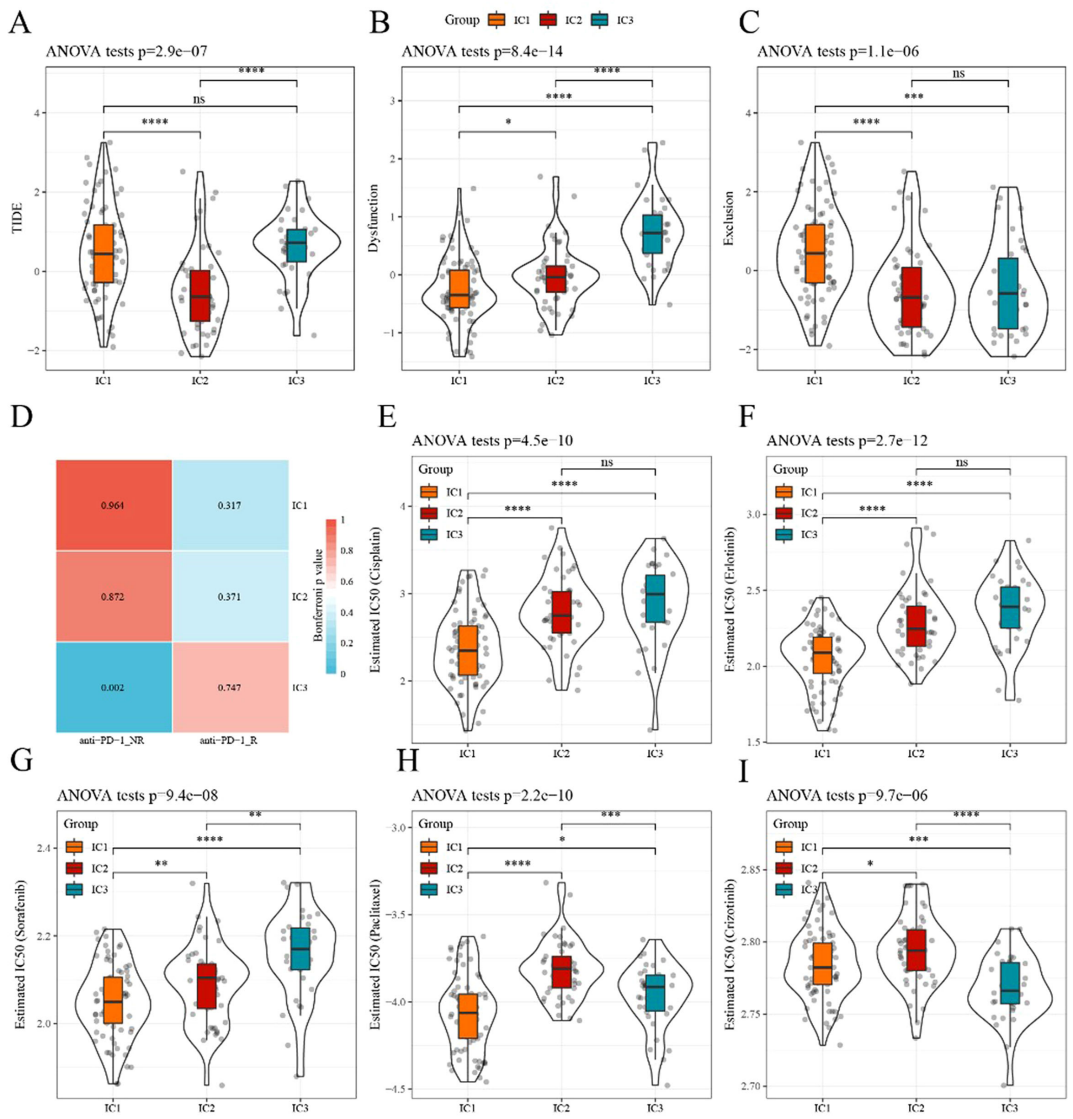
Differences in TIDE scores and therapeutic treatments in immune subtypes **(A–C)** Differences in TIDE, T cell dysfunction, and T cell rejection scores among different immune subtypes of TCGA dataset, respectively. **(D)** TCGA submap analysis revealed that IC3 could be more sensitive to anti-PD-1 (Bonferroni-corrected p<0.05). **(E–I)** Box plots of the estimated IC50 for cisplatin, erlotinib, sorafenib, paclitaxel, and crizotinib, respectively. *p<0.05, **p<0.01, ***p<0.001, ****p<0.0001. ns, no significance.

Here, we applied the TIDE software to evaluate the potential clinical effects of immunotherapy. In TCGA dataset, the TIDE score of IC1 or IC3 was significantly higher than that of IC2 (**Figure 6A**), suggesting that immunotherapy had a greater impact on IC2 than on IC1 or IC3. The predictive T cell dysfunction scores of IC1 were relatively lower, whereas those of IC3 were higher (**Figure 6B**). However, for the predictive T cell rejection scores, the score of IC1 was significantly higher than that of IC3 (**Figure 6C**), potentially explaining why IC1 exhibited a poor prognosis while IC3 demonstrated a better outcome.

Subsequently, the subclass mapping method was applied to compare the similarity of the three defined subtypes with patients treated with immunotherapy from the available dataset, GSE78220. A lower p-value indicated a higher similarity. The IC3 subtype showed similarity to anti-PD-1 non-resistance (anti-PD-1 NR) in TCGA dataset (**Figure 6D**), indicating that the IC3 group patients were more likely to respond to anti-PD-1 agents.

We investigated the sensitivity of different subtypes to conventional chemotherapy drugs using the same approach. A lower IC50 value indicated a higher sensitivity. The results revealed that IC1 was more sensitive than other subtypes to these chemotherapeutic drugs (**Figure 6E-I**).

### 3.6 Construction and validation of a prognostic risk model based on CD8+ T cell-related genes

**Figure 7:**
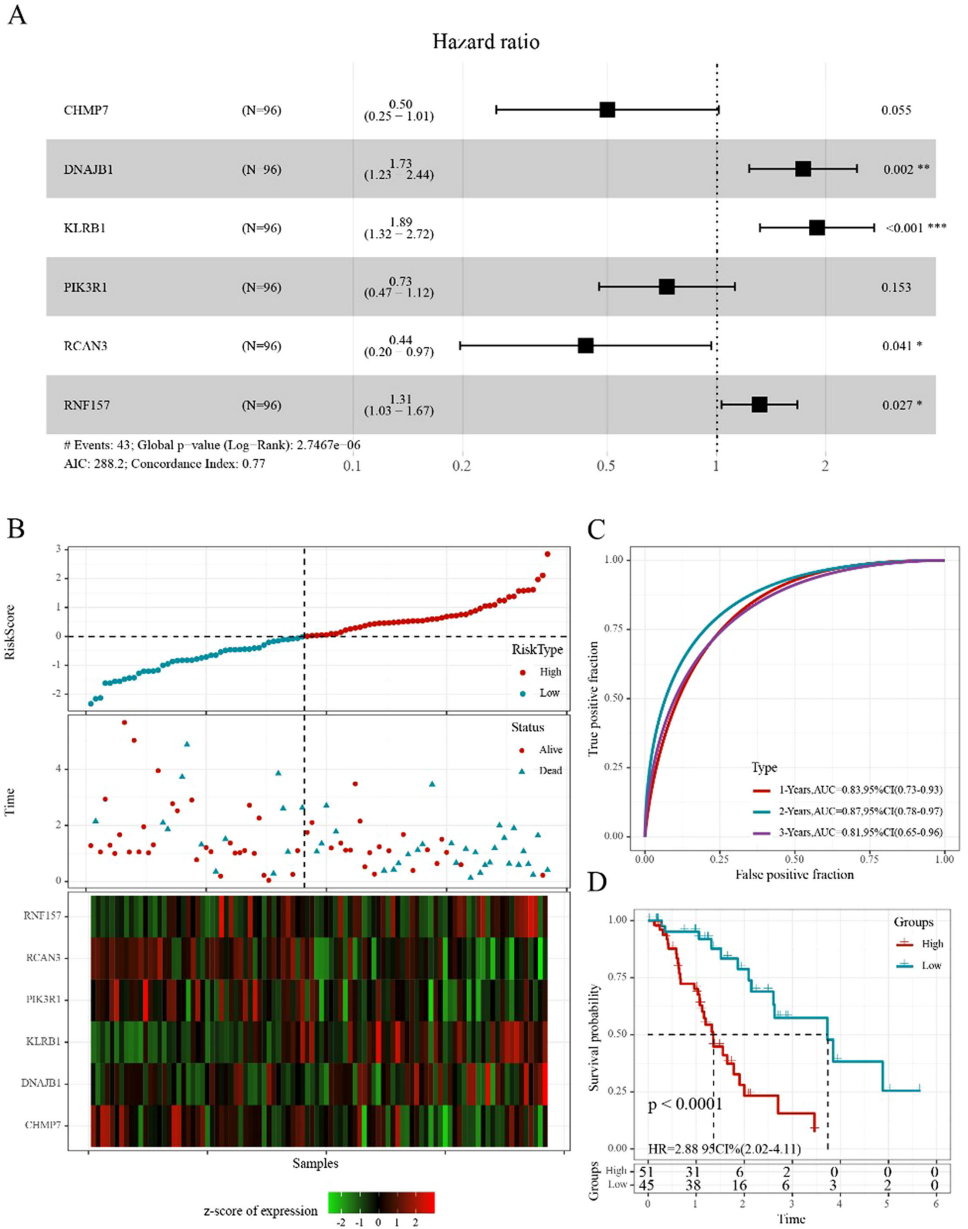
Construction of the prognostic risk score model **(A)** Forest diagram of multivariate Cox analysis showed six prognostic immune-related genes. **(B)** Distribution of risk score, survival time, and expression of six genes for each patient in the training cohort. **(C)** ROC curve based on the 6-gene signature for 1-, 2-, and 3-year OS predictions in TCGA training cohort. **(D)** Kaplan–Meier survival curve based on the risk score of the 6-gene signature in TCGA training cohort. *p<0.05, **p<0.01, ***p<0.001.

Ninety-six samples were eventually included in the training cohort, and 64 samples were included in the validation cohort, with the clinicopathological characteristics of patients listed in **Table 3**. The grouped results were tested for rationality, and no significant difference existed between them (p>0.05). By applying univariate Cox regression on the training set, we identified ten genes with prognostic significance (p<0.05; **Supplementary Table S10**). Based on multivariate Cox regression, six genes were selected to construct a prognostic risk model to avoid overfitting (**Figure 7A**).

**Table 3:** Details of TCGA training and validation sets.

Clinical Features	TCGA-ESCA train	TCGA-ESCA test	P
OS			
0	53	44	0.1206
1	43	20	
T Stage			
T1	17	8	0.4644
T2	22	19	
T3	51	36	
T4	4	1	
TX	2	0	
N Stage			
N0	39	25	0.9841
N1	41	29	
N2	5	4	
N3	3	2	
NX	8	4	
M Stage			
M0	82	46	0.1053
M1	7	8	
MX	7	10	
Stage			
I	11	5	0.1148
II	45	26	
III	33	22	
IV	7	7	
X	0	4	
Gender			
Male	80	57	0.4342
Female	16	7	
Age			
≤ 60	50	32	0.9228
>60	46	32	

The following six genes were selected: *CHMP7*, *DNAJB1*, *KLRB1*, *PIK3R1*, *RCAN3*, and *RNF157*. The coefficients of these genes were -0.691, 0.550, 0.638, -0.314, -0.829, and 0.273, respectively. Ultimately, the complete risk score was calculated using the following formula: Risk score = (-0.691 * gene expression value of *CHMP7*) + (-0.550 * gene expression value of *DNAJB1*) + (-0.638 * gene expression value of *KLRB1*) + (-0.314 * gene expression value of *PIK3R1*) + (-0.829 * gene expression value of *RCAN3*) + (0.273 * gene expression value of *RNF157*).

We determined the risk score of each sample derived from TCGA training set based on their gene expression level and plotted the risk score distribution. The distribution showed that the risk scores and mortality of the sample in the low-risk cohort were lower than those in the high-risk cohort (**Figure 7B**). A time-dependent ROC analysis was conducted to assess the predictive power of the 6-gene-based model. The area under the curve (AUC) of 1-, 2-, and 3-year predictions were 0.83, 0.87, and 0.81, respectively, indicating the high prognostic diagnostic competence of the 6-gene signature (**Figure 7C**). Additionally, the Kaplan–Meier plots indicated that the overall survival probability in the low-risk group was significantly better than that in the high-risk group (p<0.0001; **Figure 7D**).

Based on the risk score formula, similar results were obtained using the aforementioned validation methods on TCGA validation cohort (**Supplementary Figure S4A-C**). Furthermore, the conclusion drawn from the entire TCGA-ESCA cohort suggested these six genes were prognostic for ESCA and that the 6-gene-based model was effective (**Supplementary Figure S4D-F**). To further examine the extrapolation capability of the 6-gene-based model to external populations, we validated its predictive power using the independent validation cohort, GSE54993 (**Supplementary Figure S4G-I**).

### 3.7 Correlation between risk score model and clinical features

**Figure 8:**
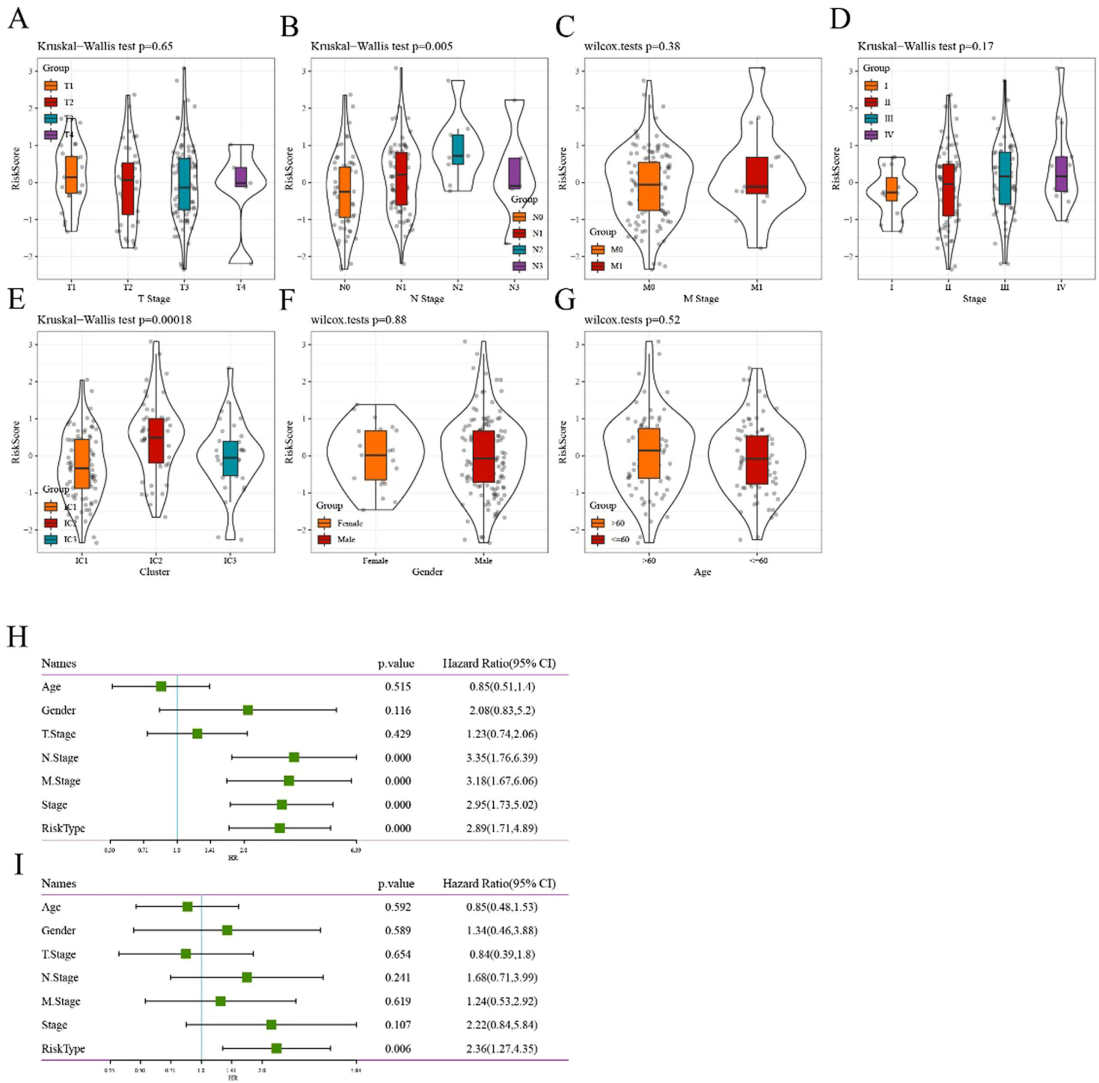
Correlation between risk score model and clinical features **(A–G)** Comparison of the distribution of risk score among T stage, N stage, M stage, stage, immune cluster, gender, and age grouping, respectively. **(H)** Univariate Cox analysis showed that the risk type and clinical features, including N stage, M stage, and stage, were significantly related to OS in TCGA cohort. **(I)** Multivariate Cox analysis demonstrated that the 6-gene signature was an independent prognostic factor in TCGA-ESCA cohort.

When comparing the distribution of risk scores in TCGA-ESCA dataset among different groups based on clinical features (age, gender, TNM stage, and stage), the results indicated the following: (1) Significant differences were observed in terms of risk scores among the different N staging groups and immune subtypes (p<0.05). The risk score increased with a higher N stage. Particularly, the IC2 subtype, associated with the worst prognosis in various subtypes, exhibited the highest risk score, whereas the IC3 subtype, associated with the best prognosis, showed the opposite trend. (2) Additionally, no significant differences in risk scores were observed among the different groups based on the clinical characteristics (**Figure 8A-G**).

To identify the 6-gene signature as an independent prognostic factor for clinical features, both univariate and multivariate Cox regression were performed. The results from the univariable analysis indicated that the risk type was of prognostic significance, with a hazard ratio (95% CI) of 2.89 (1.71, 4.89) and p<0.0001 (**Figure 8H**). Similarly, the corresponding hazard ratio (95% CI) in the multivariable analysis was 2.36 (1.71, 4.89; p = 0.006; **Figure 8I**), suggesting that the risk type was significantly related to survival. Overall, these results revealed the independent prognostic value of the 6-gene-based risk type for patients with ESCA.

### 3.8 Efficacy prediction of immunotherapy using our risk model

**Figure 9:**
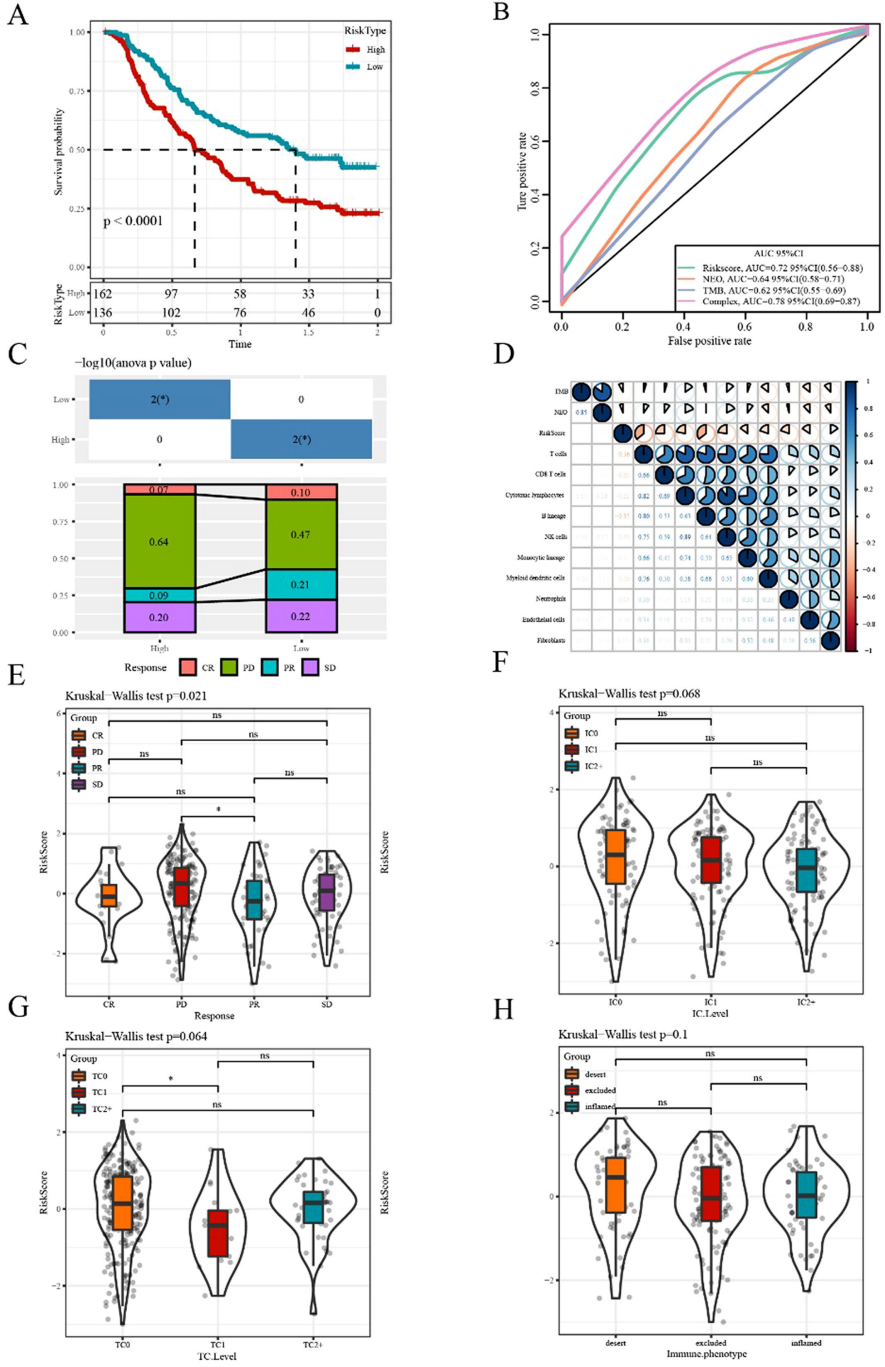
Efficacy prediction of immunotherapy using our risk model **(A)** Kaplan–Meier survival curve based on the 6-gene model in the Imvigor210 dataset. **(B)** ROC curve of the Imvigor210 dataset was used to evaluate the predictive value of the risk model based on the 6-gene signature for immunotherapy efficacy, compared with NEO and TMB. **(C)** Stacked graphs of the proportion of clinical response statuses (CR, complete response; PR, partial response; PD, progressive disease; SD, stable disease) to immunotherapy in high- and low-risk groups of the Imvigor210 dataset. **(D)** Correlations between the risk score and immune cells, TMB, and NEO in the Imvigor210 dataset. **(E–H)** Differences in risk scores among different immunotherapy clinical response statuses, immune cell levels, tumor cell levels, and immune phenotypes, respectively. *p<0.05. ns, no significance.

Currently, constrained by the lack of effective biomarkers for predicting the clinical benefits of immunotherapy, the identification of new predictive biomarkers is essential for advancing precision immunotherapy. The immunotherapy dataset (Imvigor210) was used to explore whether the 6-gene model can predict the efficacy of immunotherapy. The Kaplan–Meier curve showed that in metastatic urothelial carcinoma (mUC) patients treated with immunotherapy, higher risk scores corresponded to worse survival rates (**Figure 9A**). The AUC curves indicated the AUC of our risk model was greater than that of previously published signatures, including TMB and immunogenic neoantigen (NEO; **Figure 9B**). The high- and low-risk groups were assigned as previously mentioned. Significant differences between the two groups were observed in responders and non-responders to immunotherapy (**Figure 9C**). The MCPcounter analysis method was applied to calculate the immune-cell infiltrating level, followed by determining the correlation between the risk score and TMB, NEO, and immune cells. The results suggested a negative correlation between the risk score and immune cell score (**Figure 9D**).

Additionally, we compared the differences in the risk scores among different groups and found significant differences in risk scores among the effectiveness of immunotherapy groups. However, no significant difference in risk score was observed among other immune characteristics grouping, including immune cells, tumor cells, and immunophenotypic grouping (**Figure 9E-H**).

### 3.9 Effect of CHMP7 on phenotype and apoptosis in ESCA cells

**Figure 10:**
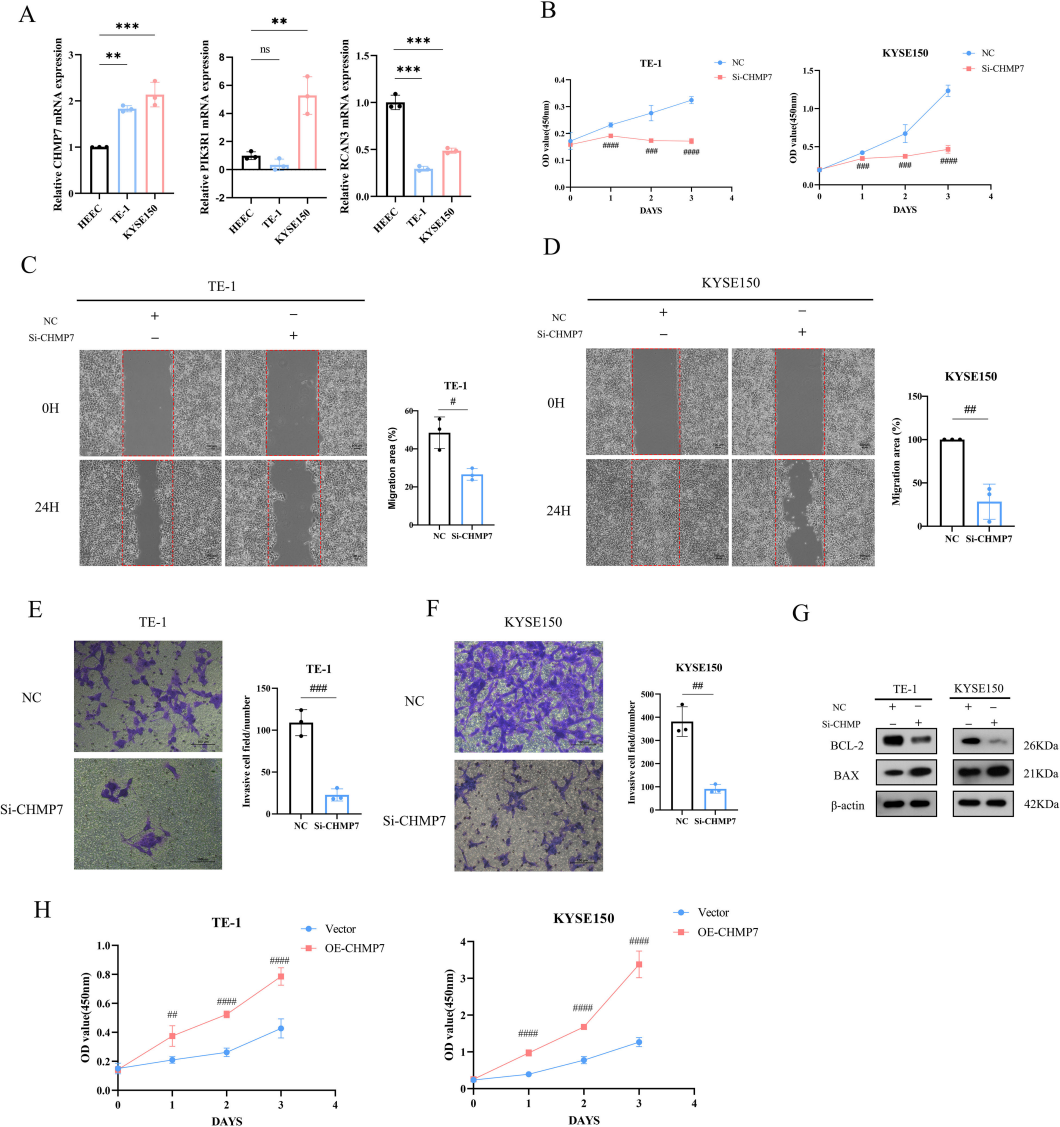
*CHMP7* can affect the phenotype and apoptosis process of ESCA cells **(A)** Expression of *CHMP7*, *PIK3R1*, and *RCAN3* in TE-1 and KYSE150 cells. **(B)** After transfection of Si-CHMP7, the cell proliferation ability of TE-1 and KYSE150 cells was evaluated. **(C–F)** After transfection of Si-CHMP7, the migration and invasion ability of TE-1 and KYSE150 cells was evaluated. **(G)** Expression of apoptosis-related proteins after Si-CHMP7 transfection. **(H)** Assessment of cell proliferation activity after overexpressing CHMP7 *p<0.05, **p<0.01, ***p<0.001 *vs*. HEEC; #p<0.05, #p<0.01, ###p<0.001, ####p<0.0001 *vs*. NC. ns, no significance.

Based on the aforementioned results, we can conclude that the 6-gene prognostic risk model exhibits a strong ability to predict prognostic risk. Further analysis of the related genes in the 6-gene prognostic risk model revealed that *PIK3R1*, *RCAN3*, and *CHMP7* were associated with poor prognosis in patients with ESCA. Subsequently, we conducted RT-qPCR analysis, revealing that only *CHMP7* was overexpressed in both TE-1 and KYSE150 cells (**Figure 10A**). Given that upregulated genes are easier to manipulate than downregulated genes in biological and therapeutic systems, we focused on the function of *CHMP7* in ESCA. After the transfection of Si-CHMP7 into TE-1 and KYSE150 cells to inhibit its expression (**Supplementary Figure S5A, B**), we observed a significant inhibition in terms of cell migration, invasion, and proliferation (**Figure 10B-F**). Western blot analysis indicated a downregulation of BCL2 and an upregulation of BAX (**Figure 10G**). In addition, we upregulated the expression of CHMP7 in esophageal cancer cells by transfecting overexpressing plasmids (**Supplementary Figure S5C, D**), and subsequently observed a significant increase in cell proliferation (**Figure 10H**). At the same time, in order to further explore the relevant mechanism of CHMP7 regulating tumor growth, we also obtained the signaling pathways that interact with CHMP7 through online analysis software (https://www.genecards.org/) (**Table 4**). From these findings, we infer that CHMP7 possesses the ability to influence the phenotype and apoptosis of ESCA cells.

**Table 4:** Signal pathways related to CHMP7.

GENE_ID	Reactome pathways
CHMP7	Autophagy
	Budding and maturation of HIV virion
	Cell Cycle
	Cell Cycle, Mitotic
	Disease
	Early SARS-CoV-2 Infection Events
	Endosomal Sorting Complex Required For Transport (ESCRT)
	HCMV Infection
	HCMV Late Events
	HIV Infection
	HIV Life Cycle
	Infectious disease
	Late endosomal microautophagy
	Late Phase of HIV Life Cycle
	M Phase
	Macroautophagy
	Membrane Trafficking
	Mitotic Anaphase
	Mitotic Metaphase and Anaphase
	Nuclear Envelope (NE) Reassembly
	Programmed Cell Death
	Pyroptosis
	Regulated Necrosis
	SARS-CoV Infections
	SARS-CoV-1 Infection
	SARS-CoV-2 Infection
	Sealing of the nuclear envelope (NE) by ESCRT-III
	Translation of Replicase and Assembly of the Replication Transcription Complex
	Translation of Replicase and Assembly of the Replication Transcription Complex
	Vesicle-mediated transport
	Viral Infection Pathways

### 3.10 Correlation of CHMP7 with immune invasion

**Figure 11:**
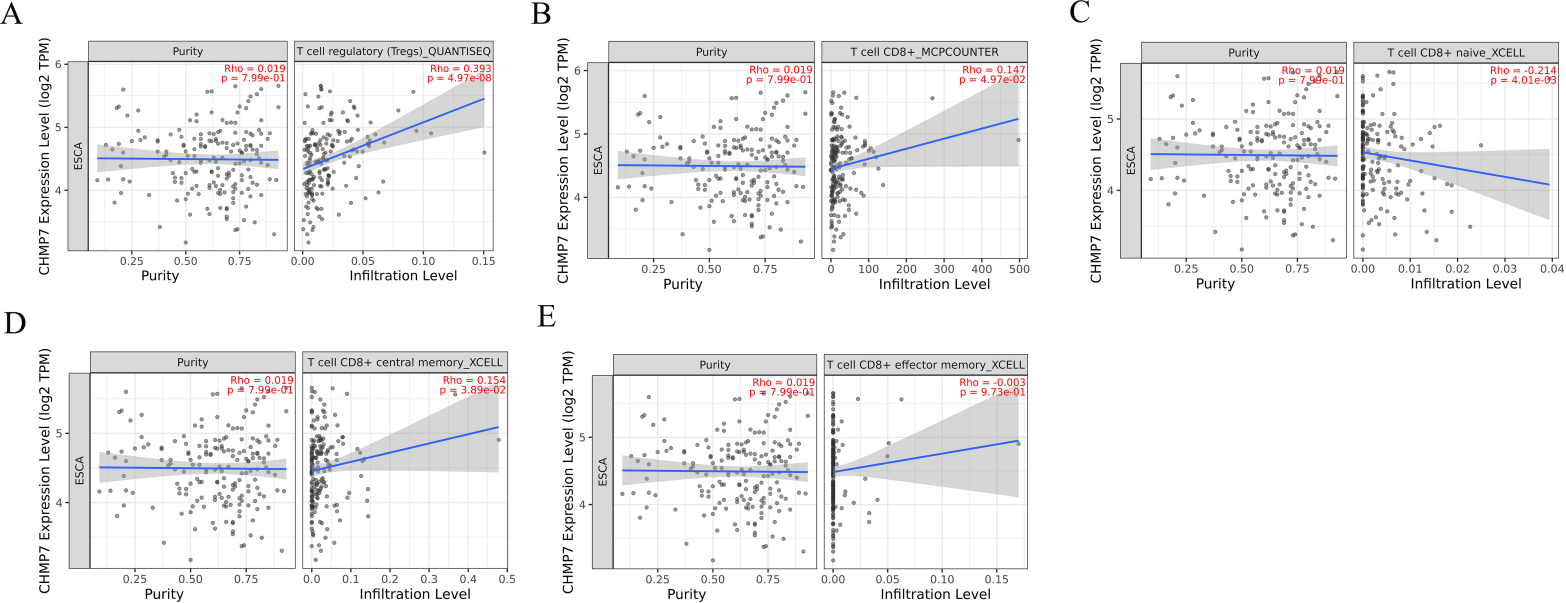
The correlation of CHMP7 with immune invasion in esophageal cancer **(A)** Treg cells **(B)** CD8+ T cells **(C)** naive CD8+ Tcells **(D)** central memory CD8+ Tcells **(E)** effector memory CD8+ Tcells.

Further investigating the role of CHMP7 in immune invasion of esophageal cancer, we ultimately found a positive correlation with Treg cells (0.393;p=4.97e-08; **Figure 11A**) and CD8+ T cells (0.147; p=4.97e-2; **Figure 11B**), when CHMP7 was analyzed for correlation with different immune cells. Subsequent analysis showed that CHMP7 was negatively correlated with naive CD8+ Tcells (-0.124; p=4.01e-03; **Figure 11C**) and positively correlated with central memory CD8+ Tcells (0.154; P=3.89e-02; **Figure 11D**). In addition, CHMP7 was not significantly correlated with effector memory CD8+ Tcells (-0.003; P=9.73e-01). It indicating that CHMP7 may interact with immune cells to affect the occurrence and development of esophageal cancer.

## 4 Discussion

To enhance the treatment effect and prognosis of ESCA patients, we investigated the role of CD8+ T cells in treatment response and its predictive value for prognosis in the present study. The evidence supporting the role of the CD8+ T cell subset in tumor control is compelling (30). A high number of CD8+ T cells could indicate a good clinical prognosis for most tumors, whereas it correlated with a poor prognosis in a small number of tumors such as melanoma (31, 32). There was a correlation between pre-treatment infiltrating CD8+ T cell numbers and the response to PD-1 blockade (33). These findings indicate the vital role of CD8+ T cells in anti-tumor immunotherapy. However, multiple immune escape mechanisms and the complex tumor microenvironment inhibit the anti-tumor effect of CD8+ T cells (34). Thus, studying the regulation of CD8+ T cells and the mechanism of killing tumor cells is crucial to improving the immunotherapy effect of ESCA. We identified marker genes related to CD8+ T cells in ESCA, constructed molecular subtypes based on these marker genes, and successfully constructed a prognostic risk model, which holds clinical significance.

In this study, the clustering of CD8+ T cell-related genes revealed significant differences in the immune characteristics of ESCA, providing new treatment ideas. Thorsson V et al. implemented a pan-cancer classification identifying six immune subtypes: wound healing, IFN-γ dominant, inflammatory, lymphocyte depleted, immunologically quiet, and TGF-β dominant. These subtypes might play a critical role in predicting disease outcomes, as opposed to relying solely on features specific to individual cancer types (29). Two main strategies exist for improving anti-tumor immunity in ESCA: those designed to increase the initiation of tumor-killing immune responses (e.g., vaccination and adoptive T cell therapies) and those aimed at rescuing existing anti-tumor immune responses suppressed in tumors (e.g., immune checkpoint blockade) (35). In recent preclinical studies, neoantigen-targeted cancer vaccines have shown anti-tumor efficacy against ESCA, but clinical trials are limited (36). In a clinical trial involving ten patients with recurrent ESCA who received *MAGEA4* with TCR-T cell transfer, seven patients exhibited tumor progression within 2 months after treatment. Three patients with minimal tumor lesions at baseline survived for over 27 months (37). Some immune checkpoint inhibitors have achieved promising results in ESCA patients, but others have caused serious adverse events (38, 39). These results indicate that the existing ESCA immune subtypes may not sufficiently predict immunotherapy response. Compared with the six subtypes identified in the previous study, three new immune subtypes of ESCA were identified in our study.

The subtype analysis of ESCA revealed distinct patterns of chemokines and immune checkpoint genes. IC2 displayed the poorest survival, whereas IC3 demonstrated the best survival. The IFN-γ score in the IC3 subgroup was higher than that of other groups. CD8+ T cells in the tumor microenvironment can produce IFN-γ, stimulating the upregulation of PD-1/PD-L1 and *IDO1* (40). *IDO1*, an immune checkpoint-related gene, is positively correlated with poor prognosis and tumor progression and metastasis (41). Activation of the aryl hydrocarbon receptor (*AhR*) by the *IDO1* product kynurenine (KYN) through the metabolic pathway led to the generation of immune-tolerant dendritic and regulatory T cells, resulting in immune cell dysfunction (42, 43). We observed that *IDO1*, *LAG3*, *CTLA4*, *PDCD1*, *PDCD1LG2*, and other immune checkpoint-related genes were highly expressed in IC3, indicating that IC3 may respond favorably to ICBs. Additionally, IC3 exhibited the highest immune T cell lysis activity. Cytolytic (CYT) activity was associated with counter-regulatory immune responses and improved prognosis, as evaluated using the average expression levels of *GZMA* and *PRF1* (26). CYT serves as a new immunotherapy biomarker indicative of anti-tumor immune activity involving cytotoxic T cells and macrophages (44). Approximately 63% of ESCA patients expressed *MAGEA4*, contributing to tumor cell lysis when recognized by cytolytic T lymphocytes (45). Moreover, significant differences in the expression of genes related to chemokines, chemokine receptors, and angiogenesis scores were observed among the three subtypes, with IC3 exhibiting the highest expression. Chemokines and chemokine receptors can mediate T cell infiltration into tumors, influencing tumor immunity and therapeutic effects (46). Lymphotoxin-α (LT-α) secreted by activated T cells promotes abnormal angiogenesis of head and neck squamous cell carcinoma through the NF-κB pathway (47). Both approaches were beneficial in increasing the number of tumor-infiltrating T cells, and chemokines were implicated in inducing angiogenesis and lymphangiogenesis (48, 49). These findings collectively support the notion that IC3 maintains high immune activity, suggesting a favorable response to immunotherapy.

Furthermore, these immune subgroups exhibit diverse immune and pathway characteristics. Immune cell groups, including CD8+ T cells, resting memory CD4+ T cells, and M0, M1, and M2 macrophages were significantly highly expressed in ESCA. Multivariate analysis indicated that CD8+ T cell infiltration was an independent prognostic factor, and the presence of CD8+ T cell infiltration in ESCA was identified as a favorable prognostic factor (50). Animal studies have shown that blocking the *CCL2*-*CCR2* axis greatly reduces the incidence of tumors by hindering the recruitment of tumor-associated macrophages. M2 macrophage polarization led to immune evasion and tumor promotion through the PD-1 signaling pathway (51). Consistent with these results, IC3 exhibited a higher proportion of CD8+ T cells and a lower proportion of macrophages. Among the ten oncogenic signaling pathways, six showed significant differences in various subtypes, indicating variations in infiltrating immune cell components, tumorigenesis, and distinct escape mechanisms (52).

We examined the correlation between immune subtypes and the response to immunotherapy and chemotherapy. The IC1 subtype exhibited greater sensitivity to chemotherapy drugs (cisplatin, erlotinib, sorafenib, paclitaxel, and crizotinib). Moreover, the IC3 subtype demonstrated similarity to anti-PD-1 NR, suggesting a more favorable immunotherapy effect. Studies have shown that innate anti-PD-1 resistance weakens the effect of PD-1/PD-L1 inhibitors in melanoma (53). However, despite the TIDE score, IC3 demonstrated the least benefit from immunotherapy. Upon further comparing the differences between T cell dysfunction and exclusion scores, we found that IC3 exhibited lower T cell exclusion scores, potentially contributing to the better prognosis of IC3. Immunosuppressive factors may impede T cells from infiltrating tumors (54). Studies have indicated that tumor-intrinsic Wnt/β-catenin pathway activation primarily causes T cell exclusion, resulting in non-T cell inflammation in the tumor microenvironment in melanoma (55). These findings provide a strong basis for ESCA patients who opt for systemic treatment options. Additionally, there was no significant difference in the number of mutant genes among the three subtypes.

We successfully constructed an ESCA risk model based on CD8+ T cell-related genes, comparing clinical characteristics and molecular subtypes between groups with high and low scores. The IC3 subtype, associated with the best prognosis, exhibited a lower risk score. In the N stage, higher risk scores were observed in the late stage, with the IC2 subtype associated with the worst prognosis having the highest risk score. Conversely, the IC3 subtype with a lower risk score demonstrated the best prognosis. The 6-gene signature model was independent in clinical applications. Single-factor Cox regression analysis identified the RiskScore model, and multi-factor Cox regression analysis found that RiskType (hazard ratio = 2.36, 95% CI = 1.27–4.35, p = 0.006) significantly correlated with survival. These results validate the predictive performance and clinical application value of our 6-gene signature model. A negative correlation was observed between risk score and immune cell scores. Significant differences were noted in the effectiveness of risk score and immunotherapy groups as well as differences between risk score and tumor cells.

Finally, we identified six gene signatures; *PIK3R1*, *RCAN3*, and *CHMP7* were negatively correlated with the prognosis of patients with ESCA, whereas *DNAJB1*, *KLRB1*, and *RNF157* showed positive correlations. We selected the genes *PIK3R1*, *RCAN3*, and *CHMP7*, negatively associated with prognosis, for further investigation. The experimental results of RT-qPCR indicated that compared with that in HEEC cells, only CHMP7 was upregulated in both KYSE150 and TE-1 cells. Furthermore, upon knocking down the expression of CHMP7, we observed reduced migration, invasion, and proliferation of ESCA cells, along with an accelerated apoptosis process. Therefore, we posit that CHMP7 serves not only as a risk factor associated with the immune index but also as a potential molecular target for immunotherapy in ESCA.

## 5 Conclusion

In this study, we identified three immune subtypes of ESCA based on CD8+ T cell-related genes, established a 6-gene model associated with prognosis, and conducted *in vitro* functional experiments to identify CHMP7 as a prognostic potential biomarker. Overall, our research contributes valuable insights for personalized ESCA treatment.

## Data Availability

The original contributions presented in the study are included in the article/**Supplementary Material**. Further inquiries can be directed to the corresponding authors.

## Supplementary material

Supplementary Figure 1 Functional enrichment plot of the purple module, including biological processes **(A)**, cellular components **(B)**, molecular functions **(C)**, and KEGG analysis **(D)**.

Supplementary Figure 2 Comparison of the distribution of different clinical characteristics, comprising survival events **(A)**, T stage **(B)**, N stage **(C)**, M stage **(D)**, stage **(E)**, age **(F)**, and gender **(G)**, among the three immune subtypes in TCGA cohort.

Supplementary Figure 3 Distribution of tumor mutational burden **(A)** and the number of mutated genes **(B)** among different immune subtypes.

Supplementary Figure 4 Validation of the prognostic risk score model **(A)** Distribution of risk score, survival time, and expression of the six genes for each patient in the validation cohort. **(B)** ROC curve based on the 6-gene signature for 1-, 2-, and 3-year OS probability in TCGA validation cohort. **(C)** Kaplan–Meier survival curve based on the risk score of the 6-gene signature in TCGA validation cohort. **(D–F)** Validation of the prognostic risk score model based on the 6-gene signature using the entire TCGA dataset. **(G–I)** Validation of the prognostic risk score model based on the 6-gene signature using the independent validation dataset, GSE54993.

Supplementary Figure 5 Evaluation of Si-CHMP7 knocking efficiency **(A, B)** Evaluation of knocking efficiency of TE-1 and KYSE150 cells using three different sequences of Si-CHMP7.

Supplementary Table 1 Statistical information of the expression of 14 immune cells in 13 GEO datasets.

Supplementary Table 2 Genes included in all modules and their correlation with immune cells.

Supplementary Table 3 Functional enrichment analysis of the purple gene module.

Supplementary Table 4 Univariate analysis of 16 prognostic CD8+ T cell-related genes in TCGA-ESCA cohort.

Supplementary Table 5 Univariate analysis of 41 prognostic CD8+ T cell-related genes in the GSE54993 cohort.

Supplementary Table 6 Immune clustering of the samples of TCGA cohort.

Supplementary Table 7 Immune clustering of the samples of the GSE54993 cohort.

Supplementary Table 8 1224 genes with a mutation frequency greater than 3 in immune subtypes.

Supplementary Table 9 124 genes with a significantly high frequency of mutations in each subtype.

Supplementary Table 10 Univariate analysis of ten prognostic CD8+ T cell-related genes in TCGA training cohort.

## References

[1] Sung H Ferlay J Siegel RL Laversanne M Soerjomataram I Jemal A. Global cancer statistics 2020: GLOBOCAN estimates of incidence and mortality worldwide for 36 cancers in 185 countries. CA Cancer J Clin. (2021) 71:209–49. doi: 10.3322/caac.21660 33538338

[2] Fitzgerald RC di Pietro M Ragunath K Ang Y Kang JY Watson P. British Society of Gastroenterology guidelines on the diagnosis and management of Barrett’s oesophagus. Gut. (2014) 63:7–42. doi: 10.1136/gutjnl-2013-305372 24165758

[3] Lordick F Mariette C Haustermans K Obermannova R Arnold D Committee EG. Oesophageal cancer: ESMO Clinical Practice Guidelines for diagnosis, treatment and follow-up. Ann Oncol. (2016) 27:v50–7. doi: 10.1093/annonc/mdw329 27664261

[4] Ajani JA D’Amico TA Bentrem DJ Chao J Corvera C Das P. Esophageal and esophagogastric junction cancers, version 2.2019, NCCN clinical practice guidelines in oncology. J Natl Compr Canc Netw. (2019) 17:855–83. doi: 10.6004/jnccn.2019.0033 31319389

[5] Smyth EC Lagergren J Fitzgerald RC Lordick F Shah MA Lagergren P. Oesophageal cancer. Nat Rev Dis Primers. (2017) 3:17048. doi: 10.1038/nrdp.2017.48 28748917 PMC6168059

[6] Kelly RJ. Emerging multimodality approaches to treat localized esophageal cancer. J Natl Compr Canc Netw. (2019) 17:1009–14. doi: 10.6004/jnccn.2019.7337 31390584

[7] Bornschein J Wernisch L Secrier M Miremadi A Perner J MacRae S. Transcriptomic profiling reveals three molecular phenotypes of adenocarcinoma at the gastroesophageal junction. Int J Cancer. (2019) 145:3389–401. doi: 10.1002/ijc.32384 PMC6851674 31050820

[8] Ma M Chen Y Chong X Jiang F Gao J Shen L. Integrative analysis of genomic, epigenomic and transcriptomic data identified molecular subtypes of esophageal carcinoma. Aging (Albany NY). (2021) 13:6999–7019. doi: 10.18632/aging.202556 33638948 PMC7993659

[9] Xie Y Shi X Chen Y Wu B Gong X Lu W. The intra-class heterogeneity of immunophenotyping and immune landscape in oesophageal cancer and clinical implications. Ann Med. (2021) 53:626–38. doi: 10.1080/07853890.2021.1912385 PMC8078926 33860722

[10] Vigano S Alatzoglou D Irving M Menetrier-Caux C Caux C Romero P. Targeting adenosine in cancer immunotherapy to enhance T-cell function. Front Immunol. (2019) 10:925. doi: 10.3389/fimmu.2019.00925 31244820 PMC6562565

[11] King RJ Qiu F Yu F Singh PK. Metabolic and immunological subtypes of esophageal cancer reveal potential therapeutic opportunities. Front Cell Dev Biol. (2021) 9:667852. doi: 10.3389/fcell.2021.667852 34307352 PMC8295652

[12] Gautier L Cope L Bolstad BM Irizarry RA. affy–analysis of Affymetrix GeneChip data at the probe level. Bioinformatics. (2004) 20:307–15. doi: 10.1093/bioinformatics/btg405 14960456

[13] Ritchie ME Phipson B Wu D Hu Y Law CW Shi W. limma powers differential expression analyses for RNA-sequencing and microarray studies. Nucleic Acids Res. (2015) 43:e47. doi: 10.1093/nar/gkv007 25605792 PMC4402510

[14] Langfelder P Horvath S. WGCNA: an R package for weighted correlation network analysis. BMC Bioinf. (2008) 9:559. doi: 10.1186/1471-2105-9-559 PMC2631488 19114008

[15] Yu G Wang LG Han Y He QY. clusterProfiler: an R package for comparing biological themes among gene clusters. OMICS. (2012) 16:284–7. doi: 10.1089/omi.2011.0118 PMC3339379 22455463

[16] Wilkerson MD Hayes DN. ConsensusClusterPlus: a class discovery tool with confidence assessments and item tracking. Bioinformatics. (2010) 26:1572–3. doi: 10.1093/bioinformatics/btq170 PMC2881355 20427518

[17] Hanzelmann S Castelo R Guinney J. GSVA: gene set variation analysis for microarray and RNA-seq data. BMC Bioinf. (2013) 14:7. doi: 10.1186/1471-2105-14-7 PMC3618321 23323831

[18] Newman AM Liu CL Green MR Gentles AJ Feng W Xu Y. Robust enumeration of cell subsets from tissue expression profiles. Nat Methods. (2015) 12:453–7. doi: 10.1038/nmeth.3337 PMC4739640 25822800

[19] Becht E Giraldo NA Lacroix L Buttard B Elarouci N Petitprez F. Estimating the population abundance of tissue-infiltrating immune and stromal cell populations using gene expression. Genome Biol. (2016) 17:218. doi: 10.1186/s13059-016-1070-5 27765066 PMC5073889

[20] Zhang Z Bao S Yan C Hou P Zhou M Sun J. Computational principles and practice for decoding immune contexture in the tumor microenvironment. Brief Bioinform. (2021) 22. doi: 10.1093/bib/bbaa075 32496512

[21] Venables WN Ripley BD Venables WN. Modern applied statistics with S. 4th. New York: Springer (2002). p. 495.

[22] Blanche P Dartigues JF Jacqmin-Gadda H. Estimating and comparing time-dependent areas under receiver operating characteristic curves for censored event times with competing risks. Stat Med. (2013) 32:5381–97. doi: 10.1002/sim.5958 24027076

[23] Nagarsheth N Wicha MS Zou W. Chemokines in the cancer microenvironment and their relevance in cancer immunotherapy. Nat Rev Immunol. (2017) 17:559–72. doi: 10.1038/nri.2017.49 PMC5731833 28555670

[24] Takikawa O Tagawa Y Iwakura Y Yoshida R Truscott RJ. Interferon-gamma-dependent/independent expression of indoleamine 2,3-dioxygenase. Studies with interferon-gamma-knockout mice. Adv Exp Med Biol. (1999) 467:553–7. doi: 10.1007/978-1-4615-4709-9_68 10721099

[25] Danilova L Ho WJ Zhu Q Vithayathil T De-Jesus-Acosta A Azad NS. Programmed cell death ligand-1 (PD-L1) and CD8 expression profiling identify an immunologic subtype of pancreatic ductal adenocarcinomas with favorable survival. Cancer Immunol Res. (2019) 7:886–95. doi: 10.1158/2326-6066.CIR-18-0822 PMC6548624 31043417

[26] Rooney MS Shukla SA Wu CJ Getz G Hacohen N. Molecular and genetic properties of tumors associated with local immune cytolytic activity. Cell. (2015) 160:48–61. doi: 10.1016/j.cell.2014.12.033 25594174 PMC4856474

[27] Masiero M Simoes FC Han HD Snell C Peterkin T Bridges E. A core human primary tumor angiogenesis signature identifies the endothelial orphan receptor ELTD1 as a key regulator of angiogenesis. Cancer Cell. (2013) 24:229–41. doi: 10.1016/j.ccr.2013.06.004 PMC3743050 23871637

[28] Sanchez-Vega F Mina M Armenia J Chatila WK Luna A La KC. Oncogenic signaling pathways in the cancer genome atlas. Cell. (2018) 173:321–337 e310. doi: 10.1016/j.cell.2018.03.035 29625050 PMC6070353

[29] Thorsson V Gibbs DL Brown SD Wolf D Bortone DS Ou Yang TH. The immune landscape of cancer. Immunity. (2018) 48:812–830 e814. doi: 10.1016/j.immuni.2018.03.023 29628290 PMC5982584

[30] van der Leun AM Thommen DS Schumacher TN. CD8(+) T cell states in human cancer: insights from single-cell analysis. Nat Rev Cancer. (2020) 20:218–32. doi: 10.1038/s41568-019-0235-4 PMC7115982 32024970

[31] Fridman WH Pages F Sautes-Fridman C Galon J. The immune contexture in human tumours: impact on clinical outcome. Nat Rev Cancer. (2012) 12:298–306. doi: 10.1038/nrc3245 22419253

[32] Barnes TA Amir E. HYPE or HOPE: the prognostic value of infiltrating immune cells in cancer. Br J Cancer. (2017) 117:451–60. doi: 10.1038/bjc.2017.220 PMC5558691 28704840

[33] Tumeh PC Harview CL Yearley JH Shintaku IP Taylor EJ Robert L. PD-1 blockade induces responses by inhibiting adaptive immune resistance. Nature. (2014) 515:568–71. doi: 10.1038/nature13954 PMC4246418 25428505

[34] Jiang X Xu J Liu M Xing H Wang Z Huang L. Adoptive CD8(+) T cell therapy against cancer:Challenges and opportunities. Cancer Lett. (2019) 462:23–32. doi: 10.1016/j.canlet.2019.07.017 31356845

[35] Huang TX Fu L. The immune landscape of esophageal cancer. Cancer Commun (Lond). (2019) 39:79. doi: 10.1186/s40880-019-0427-z 31771653 PMC6878621

[36] Forghanifard MM Gholamin M Moaven O Farshchian M Ghahraman M Aledavood A. Neoantigen in esophageal squamous cell carcinoma for dendritic cell-based cancer vaccine development. Med Oncol. (2014) 31:191. doi: 10.1007/s12032-014-0191-5 25178937

[37] Kageyama S Ikeda H Miyahara Y Imai N Ishihara M Saito K. Adoptive transfer of MAGE-A4 T-cell receptor gene-transduced lymphocytes in patients with recurrent esophageal cancer. Clin Cancer Res. (2015) 21:2268–77. doi: 10.1158/1078-0432.CCR-14-1559 25855804

[38] Janjigian YY Bendell J Calvo E Kim JW Ascierto PA Sharma P. CheckMate-032 study: efficacy and safety of nivolumab and nivolumab plus ipilimumab in patients with metastatic esophagogastric cancer. J Clin Oncol. (2018) 36:2836–44. doi: 10.1200/JCO.2017.76.6212 PMC6161834 30110194

[39] Zhao Q Yu J Meng X. A good start of immunotherapy in esophageal cancer. Cancer Med. (2019) 8:4519–26. doi: 10.1002/cam4.2336 PMC6712478 31231980

[40] Garcia-Diaz A Shin DS Moreno BH Saco J Escuin-Ordinas H Rodriguez GA. Interferon receptor signaling pathways regulating PD-L1 and PD-L2 expression. Cell Rep. (2017) 19:1189–201. doi: 10.1016/j.celrep.2017.04.031 PMC6420824 28494868

[41] Kiyozumi Y Baba Y Okadome K Yagi T Ishimoto T Iwatsuki M. IDO1 expression is associated with immune tolerance and poor prognosis in patients with surgically resected esophageal cancer. Ann Surg. (2019) 269:1101–8. doi: 10.1097/SLA.0000000000002754 31082908

[42] Cheong JE Sun L. Targeting the IDO1/TDO2-KYN-ahR pathway for cancer immunotherapy - challenges and opportunities. Trends Pharmacol Sci. (2018) 39:307–25. doi: 10.1016/j.tips.2017.11.007 29254698

[43] Zhai L Ladomersky E Lenzen A Nguyen B Patel R Lauing KL. IDO1 in cancer: a Gemini of immune checkpoints. Cell Mol Immunol. (2018) 15:447–57. doi: 10.1038/cmi.2017.143 PMC6068130 29375124

[44] Jiang X Jiang Z Xiang L Chen X Wu J Jiang Z. Identification of a two-gene prognostic model associated with cytolytic activity for colon cancer. Cancer Cell Int. (2021) 21:95. doi: 10.1186/s12935-021-01782-6 33557848 PMC7869500

[45] Duffour MT Chaux P Lurquin C Cornelis G Boon T van der Bruggen PA. MAGE-A4 peptide presented by HLA-A2 is recognized by cytolytic T lymphocytes. Eur J Immunol. (1999) 29:3329–37. doi: 10.1002/(SICI)1521-4141(199910)29:10<3329::AID-IMMU3329>3.0.CO;2-7 10540345

[46] Dangaj D Bruand M Grimm AJ Ronet C Barras D Duttagupta PA. Cooperation between constitutive and inducible chemokines enables T cell engraftment and immune attack in solid tumors. Cancer Cell. (2019) 35:885–900 e810. doi: 10.1016/j.ccell.2019.05.004 31185212 PMC6961655

[47] Yang JG Wang WM Xia HF Yu ZL Li HM Ren JG. Lymphotoxin-alpha promotes tumor angiogenesis in HNSCC by modulating glycolysis in a PFKFB3-dependent manner. Int J Cancer. (2019) 145:1358–70. doi: 10.1002/ijc.32221 30785217

[48] Grasso CS Tsoi J Onyshchenko M Abril-Rodriguez G Ross-Macdonald P Wind-Rotolo M. Conserved interferon-gamma signaling drives clinical response to immune checkpoint blockade therapy in melanoma. Cancer Cell. (2020) 38:500–515 e503. doi: 10.1016/j.ccell.2020.08.005 32916126 PMC7872287

[49] Korbecki J Grochans S Gutowska I Barczak K Baranowska-Bosiacka I. CC chemokines in a tumor: A review of pro-cancer and anti-cancer properties of receptors CCR5, CCR6, CCR7, CCR8, CCR9, and CCR10 ligands. Int J Mol Sci. (2020) 21. doi: 10.3390/ijms21207619 PMC7590012 33076281

[50] Schumacher K Haensch W Roefzaad C Schlag PM. Prognostic significance of activated CD8(+) T cell infiltrations within esophageal carcinomas. Cancer Res. (2001) 61:3932–6. 11358808

[51] Hugo W Zaretsky JM Sun L Song C Moreno BH Hu-Lieskovan S. Genomic and transcriptomic features of response to anti-PD-1 therapy in metastatic melanoma. Cell. (2016) 165:35–44. doi: 10.1016/j.cell.2016.02.065 26997480 PMC4808437

[52] Zhang Z Yan C Li K Bao S Li L Chen L. Pan-cancer characterization of lncRNA modifiers of immune microenvironment reveals clinically distinct *de novo* tumor subtypes. NPJ Genom Med. (2021) 6:52. doi: 10.1038/s41525-021-00215-7 34140519 PMC8211863

[53] Jiang P Gu S Pan D Fu J Sahu A Hu X. Signatures of T cell dysfunction and exclusion predict cancer immunotherapy response. Nat Med. (2018) 24:1550–8. doi: 10.1038/s41591-018-0136-1 PMC6487502 30127393

[54] Spranger S Gajewski TF. Tumor-intrinsic oncogene pathways mediating immune avoidance. Oncoimmunology. (2016) 5:e1086862. doi: 10.1080/2162402X.2015.1086862 27141343 PMC4839364

[55] Chen L Yang L Yao L Kuang XY Zuo WJ Li S. Characterization of PIK3CA and PIK3R1 somatic mutations in Chinese breast cancer patients. Nat Commun. (2018) 9:1357. doi: 10.1038/s41467-018-03867-9 29636477 PMC5893593

