# Supplementary figures and images for: Identification of immune subtypes associated with CD8+ T cell-related genes providing new treatment strategies of esophageal carcinoma
Source: Front Immunol. 2025 Feb 27;16:1512230. doi: 10.3389/fimmu.2025.1512230 (PMC11903738; doi:10.3389/fimmu.2025.1512230)

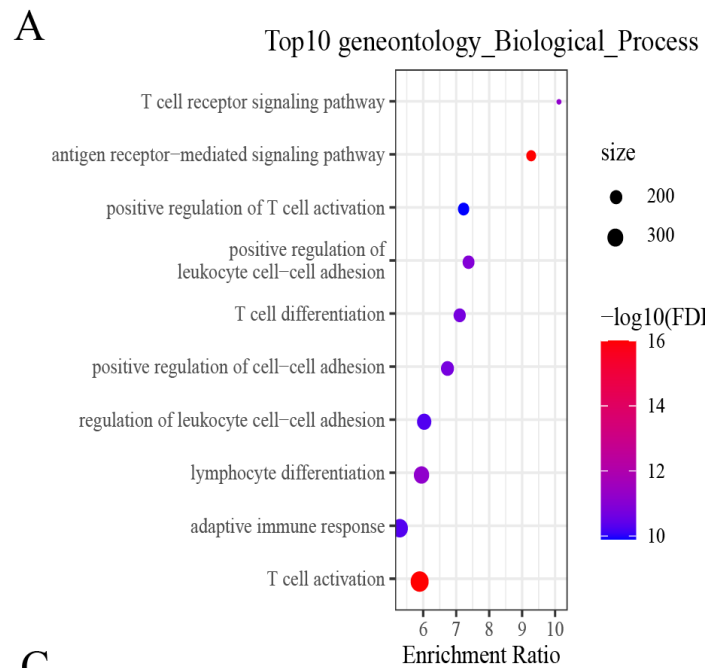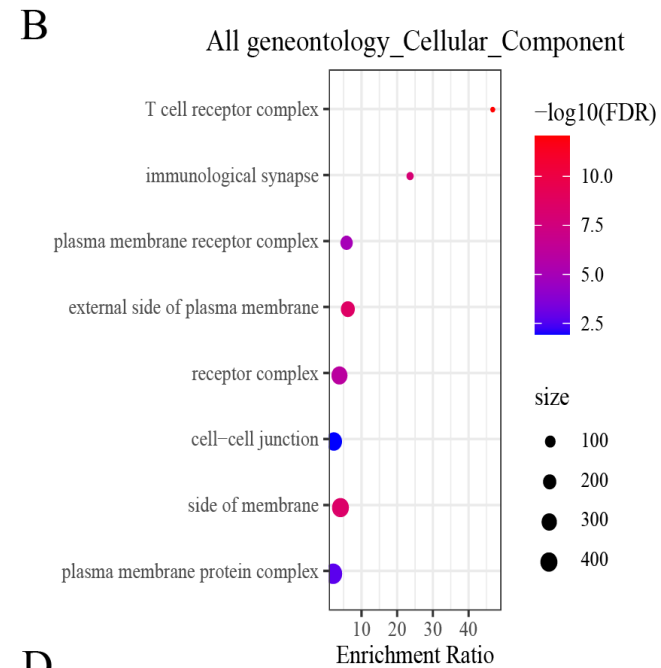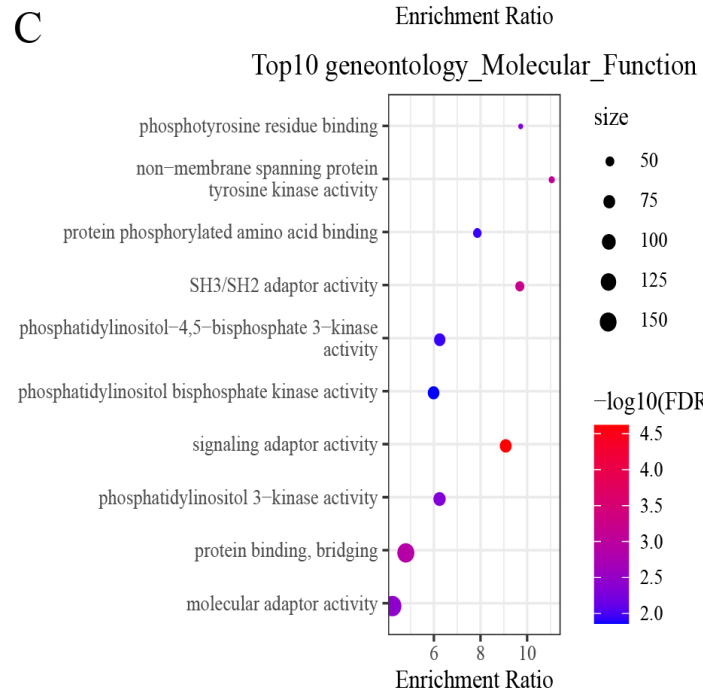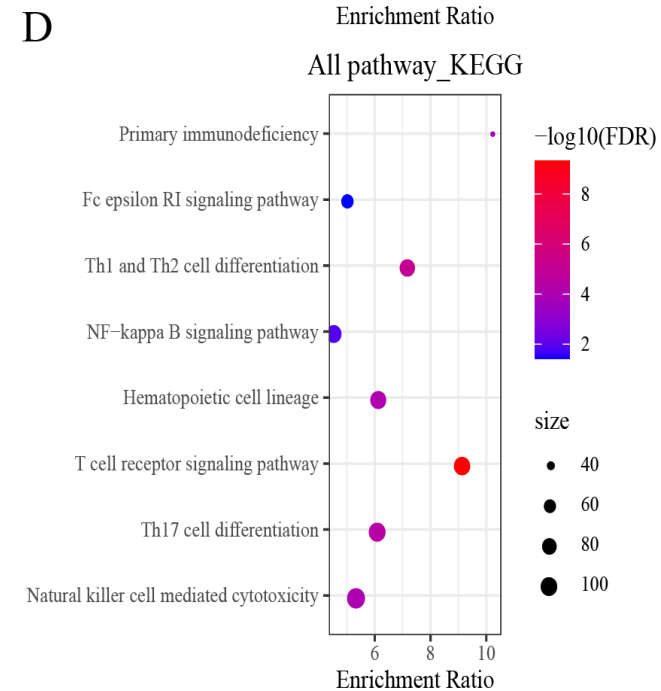

Supplement: Supplementary Figure 1 — Functional enrichment plot of the purple module, including biological processes (A), cellular components (B), molecular functions (C), and KEGG analysis (D). [file Image1.pdf]

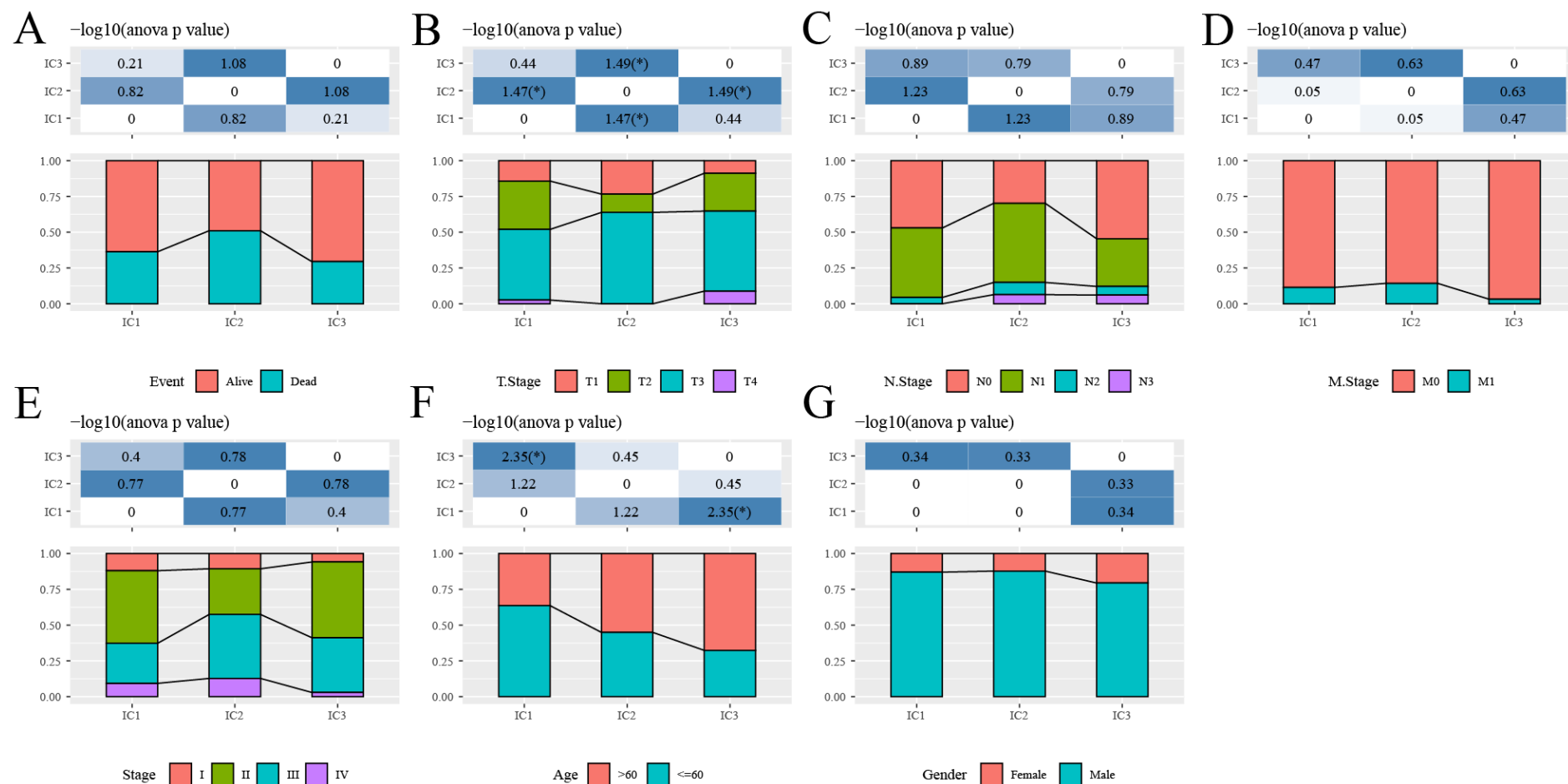

Supplement: Supplementary Figure 2 — Comparison of the distribution of different clinical characteristics, comprising survival events (A), T stage (B), N stage (C), M stage (D), stage (E), age (F), and gender (G), among the three immune subtypes in TCGA cohort. [file Image2.pdf]

A

ANOVA tests  $p=0.098$ 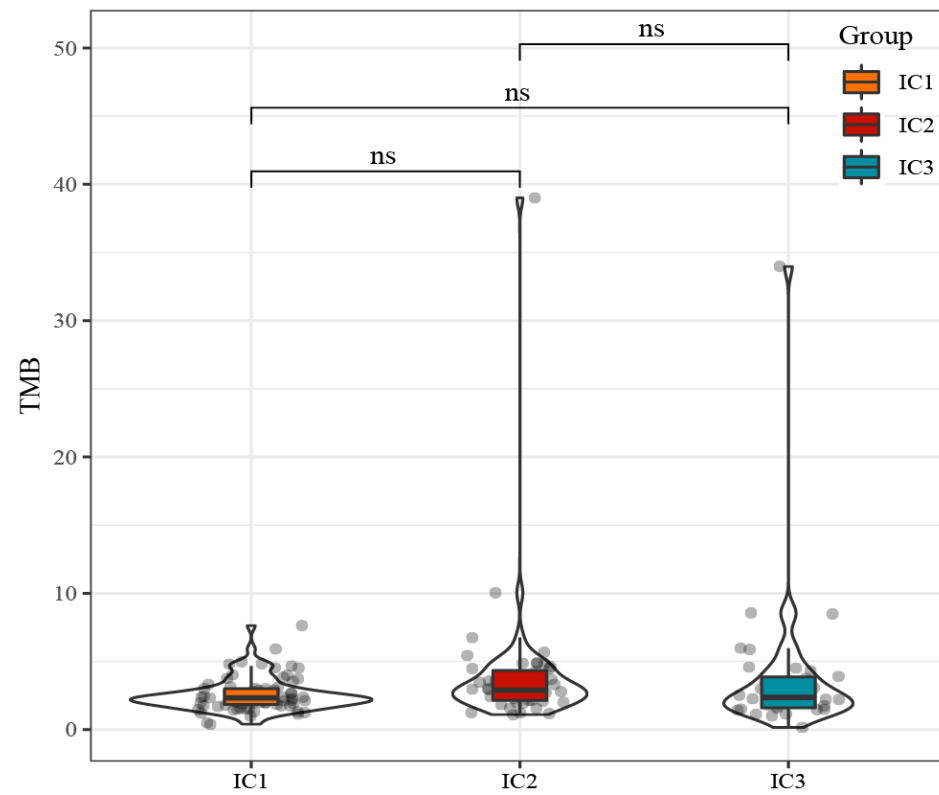

B

ANOVA tests  $p=0.1$ 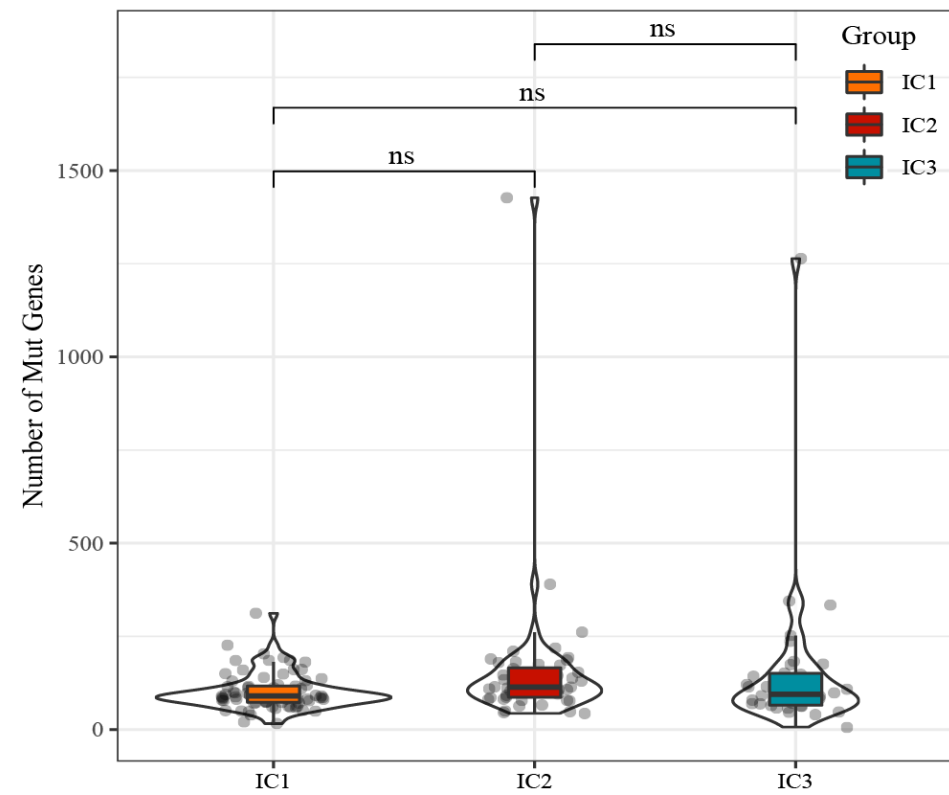

Supplement: Supplementary Figure 3 — Distribution of tumor mutational burden (A) and the number of mutated genes (B) among different immune subtypes. [file Image3.pdf]

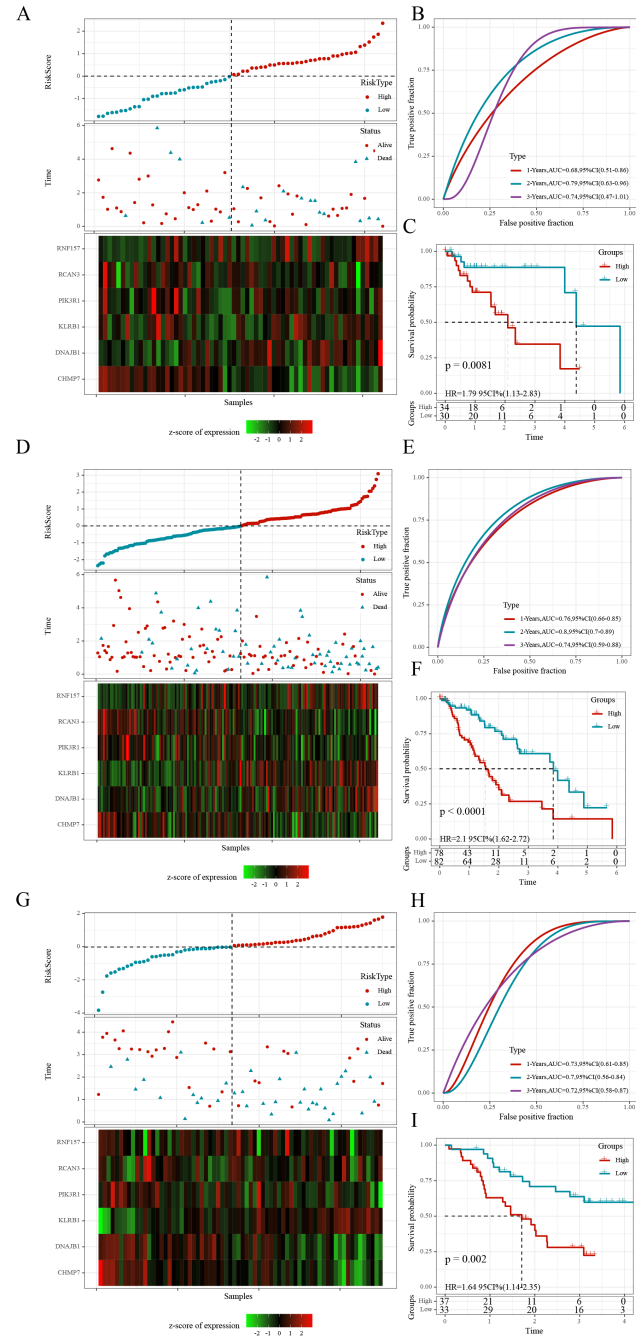

Supplement: Supplementary Figure 4 — Validation of the prognostic risk score model (A) Distribution of risk score, survival time, and expression of the six genes for each patient in the validation cohort. (B) ROC curve based on the 6-gene signature for 1-, 2-, and 3-year OS probability in TCGA validation cohort. (C) Kaplan–Meier survival curve based on the risk score of the 6-gene signature in TCGA validation cohort. (D–F) Validation of the prognostic risk score model based on the 6-gene signature using the entire TCGA dataset. (G–I) Validation of the prognostic risk score model based on the 6-gene signature using the independent validation dataset, GSE54993. [file Image4.pdf]

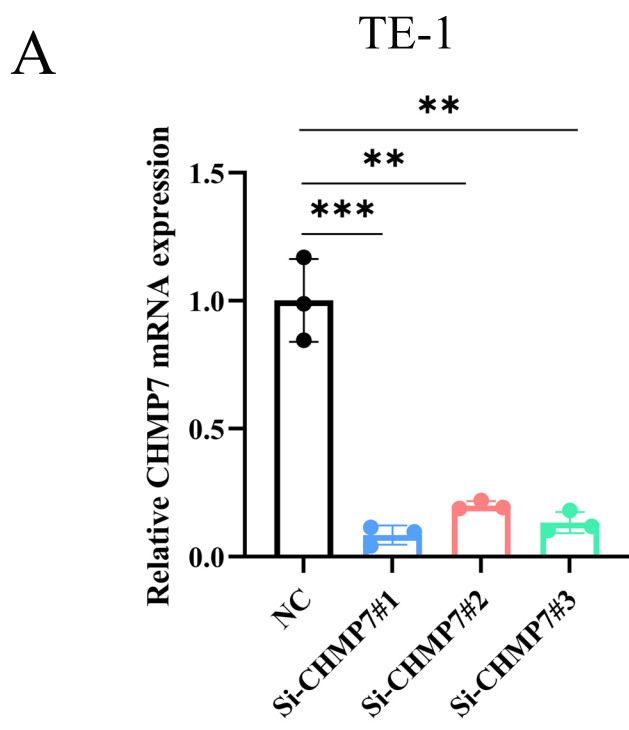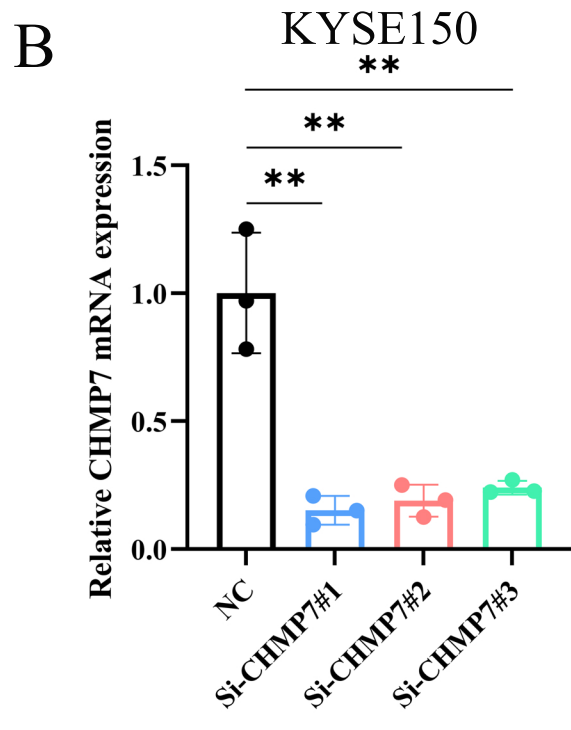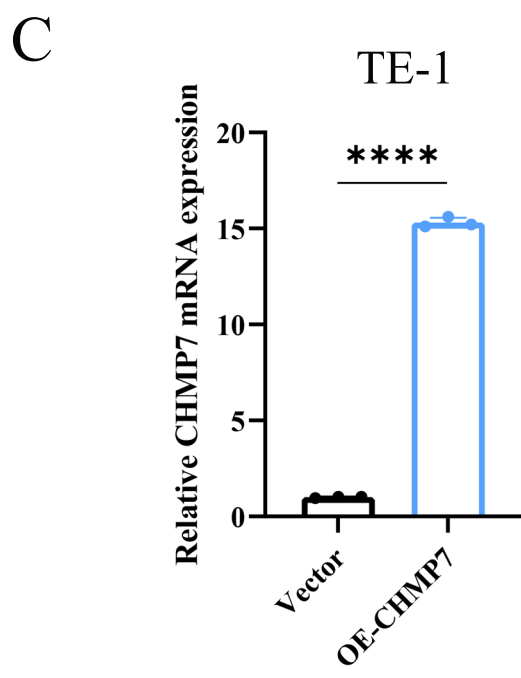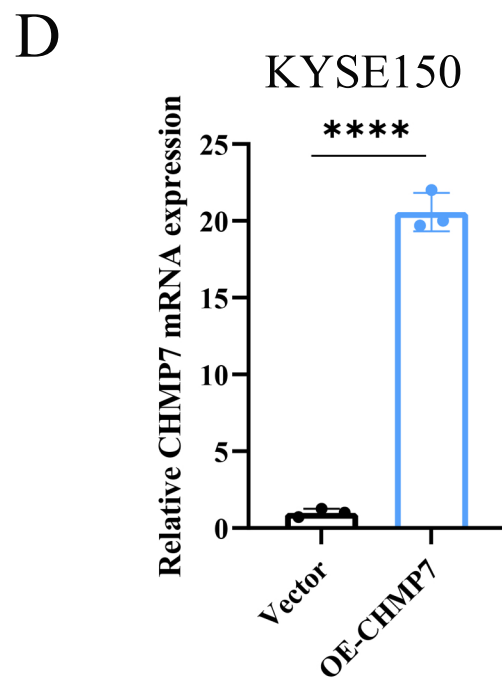

Supplement: Supplementary Figure 5 — Evaluation of Si-CHMP7 knocking efficiency (A, B) Evaluation of knocking efficiency of TE-1 and KYSE150 cells using three different sequences of Si-CHMP7. [file Image5.pdf]
