# Supplementary material for: Identification of immune subtypes associated with CD8+ T cell-related genes providing new treatment strategies of esophageal carcinoma
Source: Front Immunol. 2025 Feb 27;16:1512230. doi: 10.3389/fimmu.2025.1512230 (PMC11903738; doi:10.3389/fimmu.2025.1512230)
Supplement: Supplementary Table 1 — Statistical information of the expression of 14 immune cells in 13 GEO datasets. [file SupplementaryFile1.pdf]

ail clinical pathological features for TCGA-ESCA and GSE5

Clinical Features	TCGA-ESCA	GSE54993
**OS**		
0	97	34
1	63	36
**T Stage**		
T1	25	4
T2	41	2
T3	87	64
T4	5	
TX	2	
**N Stage**		
N0	64	32
N1	70	24
N2	9	11
N3	5	3
NX	12	
**M Stage**		
M0	128	70
M1	15	
MX	17	
**Stage**		
I	16	5
II	71	29
III	55	36
IV	14	
X	4	
**Gender**		
Male	137	57
Female	23	13
**Age**		
≤ 60	82	44
＞60	78	26

Table S2:Details of TCGA training set and validation set

Clinical Features	TCGA-ESCA train	TCGA-ESCA test	P
**OS**			
0	53	44	0.1206
1	43	20	
**T Stage**			
T1	17	8	0.4644
T2	22	19	
T3	51	36	
T4	4	1	
TX	2	0	
**N Stage**			
N0	39	25	0.9841
N1	41	29	
N2	5	4	
N3	3	2	
NX	8	4	
**M Stage**			
M0	82	46	0.1053
M1	7	8	
MX	7	10	
**Stage**			
I	11	5	0.1148
II	45	26	
III	33	22	
IV	7	7	
X	0	4	
**Gender**			
Male	80	57	0.4342
Female	16	7	
**Age**			
≤ 60	50	32	0.9228
＞60	46	32	

Table S3: The statistical information of the expression of 14 immune cells in 13 GEO datas

	B cells	CD4 T ce	CD8 T ce	Dendriti	Eosinoph	Gamma-de	Immature	Lymphocy
GSE13906	0	0	0	0	0	8	0	2
GSE23371	0	0	0	0	0	0	3	0
GSE27291	0	0	0	0	0	12	0	0
GSE27838	0	0	0	0	0	0	0	0
GSE28490	5	5	5	0	4	0	0	0
GSE28726	0	8	0	0	0	0	0	0
GSE37750	0	0	0	0	0	0	0	0
GSE39889	0	0	0	0	0	0	0	0
GSE42058	0	0	0	0	0	0	0	0
GSE49910	4	3	6	0	0	0	0	0
GSE59237	0	0	0	18	0	0	0	0
GSE6863	0	0	0	0	0	0	6	0
GSE8059	0	0	1	0	0	0	0	0
	9	16	12	18	4	20	9	2

;ets.

Monocyte:	Myeloid [	Natural [	Natural]	Neutroph:	Plasmacytoid dendritic cells	
0	0	0	0	0	0	
0	0	0	0	0	0	
0	0	0	0	0	0	
0	0	16	0	0	0	
10	5	5	0	3	5	
0	0	0	12	0	0	
0	0	0	0	0	8	
0	0	0	0	4	0	
0	8	0	0	0	0	
6	0	0	0	3	0	
0	0	0	0	0	0	
0	0	0	0	0	0	
0	0	4	0	0	0	
16	13	25	12	10	13	179

Table S4: Genes included in all modules and their correlation with immune cells

Genes	GS.Immature de	GS.Natural Killer cells	GS.CD8 T	GS.Gamma-	GS.Lympho	GS.Monocy
A1BG	-0.003902023	-0.211102778	0.177402	-0.00263	0.035888	0.153885
A1BG-AS1	-0.00804462	-0.117515393	-0.01221	-0.01077	0.037326	0.100421
A1CF	-0.053312357	-0.032247009	0.132143	0.006067	-0.03562	-0.05097
A2M	-0.015843278	-0.038780461	-0.04761	-0.00151	-0.03072	-0.10217
A2M-AS1	0.002167902	0.104948114	0.355472	-0.0369	0.12302	-0.24148
A2ML1	-0.058088633	0.019706026	0.033818	0.002818	0.006832	-0.03211
A2MP1	-0.02450794	-0.026352768	0.045507	-0.01113	0.008152	-0.13166
A4GALT	-0.013008981	-0.070529474	-0.03272	-0.01846	-0.03657	-0.12586
A4GNT	-0.04456679	-0.039619436	0.117977	0.006792	0.016972	0.097928
AA06	-0.034142328	-0.079780566	-0.07414	-0.00401	0.002837	0.089099
AAAS	0.008774774	-0.087071015	0.030301	0.05751	-0.23697	-0.09305
AACS	0.017436824	0.026027857	0.072937	0.085769	-0.24718	-0.10806
AACSP1	-0.053603502	-0.04882004	-0.08233	0.020832	-0.05122	-0.04978
AADAC	-0.040519106	-4.04E-05	-0.01299	0.003837	0.013619	-0.12374
AADACL2	-0.06823933	-0.020065176	-0.06682	-0.00157	-0.0045	0.033358
AADACP1	-0.037771346	-0.096683771	0.026266	0.010102	-0.02003	-0.10546
AADAT	-0.041459083	-0.035088646	-0.01312	0.008703	-0.00588	-0.0703
AAED1	0.020858663	0.004082649	-0.06609	-0.11106	0.282118	0.227005
AAGAB	0.024728999	-0.010595128	0.033867	-0.13126	-0.08822	-0.00307
AAK1	-0.037360277	0.087147453	0.282261	-0.01427	-0.08623	-0.1535
AAMDC	0.00422723	-0.065390431	-0.02443	0.024042	-0.18841	0.282961
AAMP	0.049420519	-0.017133815	-0.03623	0.041984	-0.15143	0.104187
AANAT	-0.037413968	-0.023097045	-0.14142	-0.02196	0.096986	0.149997
AAR2	0.042217835	0.005944766	0.121378	0.034996	-0.12682	0.101194
AARS	0.026476425	0.055293901	0.053341	0.079594	-0.25422	-0.16406
AARS2	0.031611399	-0.026912042	0.259193	0.073398	-0.25092	-0.19739
AASDH	0.013564346	0.047701079	0.190512	0.013831	-0.01423	0.043002
AASDHPPT	0.035355423	0.142908537	0.106222	0.053056	-0.16016	0.108715
AASS	-0.026351242	0.054051899	0.185171	-0.01626	0.055829	-0.26761
AATF	0.066464844	0.036381919	0.024288	0.098531	-0.26755	-0.05178
AATK	-0.000217454	0.025892742	-0.06226	-0.03244	0.017353	0.092749
AB488780	0.0029185	-0.007600454	0.12159	0.010893	-0.00218	0.02974
ABAT	0.028398014	0.025907152	-0.28132	-0.06076	0.278783	0.250284
ABCA1	-0.007245572	-0.123020971	-0.26588	-0.08999	0.213709	0.145025
ABCA11P	-0.012395506	0.015574756	0.137239	0.005038	0.01764	-0.22816
ABCA12	-0.042575415	-0.033125726	-0.0358	0.069701	-0.13828	0.006524
ABCA13	-0.066696369	-0.057248374	0.100335	0.227218	-0.04953	-0.02371
ABCA17P	-0.025338231	-0.063218891	0.08155	0.060718	-0.16457	-0.03568
ABCA2	-0.014801005	-0.066560663	0.083845	0.014355	-0.01416	-0.05412
ABCA3	-0.004596867	0.000154957	0.14681	0.057498	-0.18135	-0.0688
ABCA4	-0.042685147	-0.04726208	-0.04982	0.047889	-0.12712	-0.04968
ABCA5	-0.008868935	0.073203706	0.185653	-0.06919	0.180129	-0.41971
ABCA6	-0.020691765	-0.047982801	-0.11761	0.002795	0.013421	0.050628
ABCA7	0.013918751	-0.115535957	-0.03906	-0.04264	0.10839	-0.17051
ABCA8	-0.063671256	-0.082765824	-0.10286	0.079712	-0.04747	-0.11938
ABCA9	-0.039230082	0.027375108	-0.10259	-0.03691	0.017991	0.114976
ABCB1	-0.001860126	0.260572454	0.277266	-0.03639	0.119875	-0.2695
ABCB10	0.030540864	0.052156776	-0.03651	0.06717	-0.16275	0.090212

ABCB11	-0.066693686	-0.078037998	-0.05669	-0.01719	0.052884	0.099838
ABCB4	-0.00999837	-0.080016094	-0.10242	-0.02241	0.062755	-0.17368
ABCB5	-0.069798452	-0.115588364	0.0199	-0.02183	-0.05954	-0.07198
ABCB6	-0.011619921	-0.066150928	-0.09197	0.015926	-0.0838	0.002374
ABCB7	0.029158012	-0.024060072	0.010491	0.010672	-0.01544	0.267336
ABCB8	-0.024527887	-0.019100694	0.119432	0.05483	-0.17452	-0.06135
ABCB9	-0.100624318	-0.043376286	-0.04057	0.029271	0.020586	0.051538
ABCC1	0.024978599	-0.133469832	0.096572	0.061281	-0.18518	-0.01193
ABCC10	0.019682835	0.052944198	0.193739	-0.00934	0.001463	0.055206
ABCC11	-0.041527347	-0.051067963	-0.0024	-0.00417	-0.03229	-0.05825
ABCC12	-0.062961084	-0.047223608	0.037365	0.025046	-0.05534	-0.06053
ABCC13	-0.00706147	-0.09455078	0.1309	0.013941	0.012383	-0.04552
ABCC2	-0.02773951	-0.006439673	-0.00551	0.004628	0.012406	-0.11911
ABCC3	-0.02992945	-0.091187209	-0.12702	-0.19119	0.284012	0.490501
ABCC4	-0.016081673	0.001410096	0.074218	-0.00482	0.041925	-0.03464
ABCC5	0.012059849	0.002952755	0.046725	-0.03131	0.052593	-0.03017
ABCC6P1	-0.059048153	-0.04485253	-0.00442	0.003826	0.009739	0.070749
ABCC8	-0.044226132	-0.127619826	-0.04913	0.02821	-0.07401	0.00026
ABCC9	-0.002667177	0.072357124	-0.11682	0.079003	-0.04009	-0.13873
ABCD1	-0.011758006	-0.121125191	-0.13167	-0.06158	0.125068	0.336593
ABCD2	-0.011254755	-0.081744881	0.371348	0.028073	-0.0476	-0.20384
ABCD3	0.011252435	0.104801702	0.129383	0.0249	-0.06869	0.151184
ABCD4	0.015572605	0.014683902	0.172992	-0.00165	-0.01842	-0.10779
ABCE1	0.02195185	0.025658053	0.159802	0.063491	-0.1683	-0.03213
ABCF1	0.051755671	0.142324869	0.051441	0.083649	-0.23991	-0.07708
ABCF2	0.000654657	-0.037564447	0.13837	-0.00154	-0.14533	-0.16808
ABCF3	0.024374762	-0.005511111	0.060303	0.024764	-0.11763	-0.05217
ABCG1	-0.022942328	-0.075590404	0.060852	-0.08314	0.142272	-0.19929
ABCG2	-0.017583327	0.041649178	0.028533	0.029568	-0.10172	-0.0788
ABCG4	-0.027911764	-0.072196465	-0.08438	-0.01302	-0.01718	0.010642
ABCG5	-0.039284724	-0.036458768	0.018449	0.000607	0.02625	-0.16834
ABCG8	-0.030060219	-0.059202173	-0.01905	0.007911	0.004086	0.129488
ABHD1	-0.064226706	-0.021898	0.042873	0.009436	-0.03072	0.032172
ABHD10	0.02470396	-0.030553619	-0.06038	0.017366	-0.05542	0.046236
ABHD11	0.129691962	-0.053164824	-0.09695	0.14285	-0.09924	-0.00318
ABHD12	-0.01683846	0.091008305	-0.00104	0.067556	-0.17416	0.114153
ABHD13	-0.002143836	-0.04174143	-0.16129	-0.01013	0.064379	0.167046
ABHD14B	0.022566664	-0.038055554	0.24645	0.024961	-0.0464	-0.017
ABHD15	0.010312286	0.083946672	-0.01097	-0.00041	0.016496	-0.20268
ABHD16A	0.030607776	-0.030066213	-0.24146	-0.01879	0.056166	0.18396
ABHD16B	-0.028148698	-0.009708406	-0.1606	0.019401	-0.02141	0.10245
ABHD17A	0.02739354	0.260711058	0.236749	-0.03294	0.082238	-0.16944
ABHD17B	-0.010463211	0.099447426	0.033912	0.02075	-0.12363	-0.23585
ABHD17C	-0.010733981	0.062226209	0.093549	0.082152	-0.24786	-0.184
ABHD2	0.035249559	0.120199904	-0.30172	-0.02046	0.087634	0.093792
ABHD3	0.045208834	0.051346853	-0.03832	0.026918	-0.05163	-0.02056
ABHD4	0.012268928	0.058979671	-0.11313	-0.03408	0.052419	-0.05507
ABHD5	-0.007342614	-0.015512355	-0.27524	-0.00379	0.026589	0.31964
ABHD6	0.005542965	-0.009960136	-0.15713	0.008771	0.006311	0.14128
ABHD8	-0.02144382	0.012023122	-0.06344	-0.01642	0.071622	0.261223
ABI1	0.06984861	0.141683356	-0.11769	-0.05241	0.150424	-0.22969

ABI2	0.05878488	-0.010705025	0.136391	0.074317	-0.1578	-0.22849
ABI3	0.030053185	0.14755788	-0.00205	-0.02161	0.007908	0.187838
ABI3BP	-0.089860418	-0.038398739	0.116806	-0.00345	-0.00695	-0.0413
ABL1	0.024895303	0.074823268	0.025672	0.017488	-0.01926	0.038367
ABL2	-0.014740752	0.005604155	0.122684	0.038445	-0.08156	-0.06859
ABLIM1	0.008509819	0.070731851	0.372725	-0.0559	0.141255	-0.37738
ABLIM2	-0.038007482	-0.085121261	-0.02396	0.00932	-0.04427	-0.1106
ABLIM3	-0.010683624	-0.076409437	-0.15434	-0.18281	0.501996	0.172465
ABO	-0.036853214	-0.078969711	-0.1574	0.000478	-0.01882	0.031053
ABR	0.025002922	0.018159939	-0.20959	-0.04495	0.090693	0.147832
ABRA	-0.075201718	-0.011649657	-0.01794	-0.00065	0.011122	-0.05063
ABRACL	0.044946646	0.063397591	0.245941	0.013337	-0.04303	0.200116
ABT1	0.044187276	0.094451842	0.138642	0.02379	-0.11309	-0.13483
ABTB1	-0.040129126	0.021636549	-0.05204	-0.08719	0.101877	-0.09692
ABTB2	-0.009798887	-0.055619524	-0.05127	0.011316	-0.06313	-0.0636
AC000111.	-0.164621663	-0.071714518	-0.00661	0.020103	0.084175	0.005781
AC002059.	-0.027419021	-0.055735764	########	0.020514	-0.09058	-0.09361
AC002064.	-0.033294066	-0.03880268	0.021923	0.002314	0.010048	-0.04651
AC003973.	-0.050650141	-0.025119833	0.005434	-0.00834	0.049362	-0.06478
AC003989.	-0.035857971	-0.055917946	-0.04525	0.021039	-0.04122	-0.04901
AC004692.	0.017099807	-0.105151787	0.2809	0.042105	-0.1462	-0.18922
AC004941.	0.028532669	0.033769307	-0.11284	0.067021	-0.22187	-0.13446
AC005162.	-0.02959327	-0.061404028	-0.01933	-0.0098	-0.00441	-0.02466
AC005224.	-0.018937133	0.020177704	0.178899	0.025155	-0.05937	-0.14492
AC005256.	-0.019442954	-0.112629187	0.108088	0.008727	-0.0308	-0.04845
AC005306.	-0.002902817	-0.117013556	-0.06893	-0.01906	0.039497	-0.11076
AC005498.	-0.034037227	-0.046962177	0.072862	-0.02659	0.10565	0.114931
AC005523.	0.010753248	0.004387553	0.029299	-0.00772	-0.02589	0.081053
AC005523.	-0.027274627	-0.038145553	-0.03484	0.026449	-0.09084	0.016878
AC005537.	-0.032328139	-0.018039942	0.090984	0.02967	-0.05913	-0.13747
AC005592.	-0.023085494	-0.035800718	0.075721	-0.00556	-0.02091	-0.05697
AC005606.	-0.033559341	-0.02482095	-0.04511	-0.05648	0.179171	0.037292
AC005785.	-0.028752019	-0.095669394	0.005894	0.037318	-0.11448	0.001905
AC005838.	-0.027938173	-0.016551216	0.085048	0.00719	-0.01151	-0.01899
AC006026.	-0.023353033	-0.035926084	0.069313	-0.07148	0.200533	-0.11238
AC006129.	-0.004553217	0.069548607	0.328333	-0.04419	0.136516	-0.4796
AC006538.	-0.040115678	-0.030749492	-0.14586	0.031661	-0.06508	0.04011
AC007292.	-0.011718928	-0.024663754	0.06305	-0.00665	-0.0075	0.044202
AC007349.	-0.03620298	0.100704071	-0.02283	0.000393	0.009526	0.019402
AC007362.	-0.043608455	0.034201842	-0.20347	0.105124	-0.05533	0.000301
AC007365.	-0.026906833	-0.066331808	-0.06067	-0.04947	0.117915	0.050071
AC007389.	-0.022657672	-0.027831469	0.069426	0.011318	-0.05995	-0.01105
AC007401.	-0.012048319	-0.001786148	-0.06167	-0.01924	0.074071	0.057196
AC007680.	-0.033303843	-0.02903315	-0.12312	0.029961	-0.07422	-0.18112
AC007787.	-0.034823821	-0.087027329	-0.0816	0.017497	-0.04918	0.024813
AC008088.	-0.036975454	-0.028209196	-0.08676	-0.00631	0.045472	-0.12972
AC008746.	-0.019916236	0.045058034	0.081986	0.018869	-0.02713	-0.16007
AC008753.	-0.02095517	-0.044153736	0.09305	0.021321	-0.10829	0.02301
AC009120.	0.032071766	-0.006370326	0.008457	0.000432	-0.02828	-0.14139
AC009133.	-0.019643451	-0.109563441	0.068485	0.00876	0.001443	0.044833
AC009502.	-0.027025839	-0.036581291	-0.00605	-0.02555	-0.00268	-0.10147

AC010145.	-0.027523709	-0.058730847	0.028229	-0.0203	0.049341	0.069517
AC010524.	-0.022335909	-0.063080316	0.105525	0.018319	-0.03154	0.016837
AC012065.	-0.017324789	-0.086471605	-0.09943	-0.01346	0.019999	-0.21202
AC012360.	-0.025093407	-0.053514357	0.010636	0.0485	-0.12867	-0.00222
AC012499.	-0.045955129	-0.066275306	-0.02229	-0.02942	0.103718	-0.00487
AC012531.	-0.025310184	-0.050427898	0.010268	0.000485	-0.02453	-0.09006
AC013463.	-0.030384881	-0.065656689	-0.09767	0.056784	-0.14577	-0.06079
AC013733.	-0.075828969	-0.066255907	0.032823	-0.01745	0.037101	0.01417
AC016831.	-0.023151591	-0.048936849	0.069138	0.02224	-0.04956	0.044859
AC016999.	-0.030659224	-0.106048587	0.078415	0.016625	-0.05048	0.087058
AC017002.	-0.002924242	0.029909253	-0.01309	0.010097	-0.06704	-0.05853
AC017104.	0.003925337	0.038060182	-0.00776	-0.00868	-0.08391	-0.22144
AC018755.	0.006220684	0.022052703	0.040089	-0.0123	-0.02897	0.013967
AC018755.	-0.049645855	-0.044678377	-0.11035	-0.02044	0.053987	-0.05975
AC018766.	-0.034251748	0.00983708	-0.00425	0.012485	-0.02149	-0.12256
AC018816.	-0.013683761	0.022516319	0.118561	0.011133	-0.11318	-0.12305
AC024560.	-0.022225003	-0.066143228	0.044028	0.044636	-0.1238	-0.0315
AC025442.	-0.030978949	-0.093294039	0.064947	0.010585	0.002239	0.050471
AC064852.	-0.049054942	-0.048427115	-0.09177	0.005137	0.015385	-0.00293
AC067956.	-0.05134147	-0.061058313	-0.0587	0.020885	-0.07157	0.066051
AC068039.	-0.016218162	0.002893337	0.07635	-0.02199	0.065396	-0.10382
AC068138.	-0.02448877	-0.054947223	0.116329	0.006923	-0.0339	-0.15508
AC068831.	-0.027162051	-0.017875043	-0.06998	0.020131	-0.02343	0.082574
AC073283.	-0.004428625	-0.094964325	-0.11059	-0.0007	0.013906	0.052438
AC073321.	-0.03244727	-0.046449207	-0.05814	0.026382	-0.06534	0.001504
AC074212.	-0.041851794	-0.123396788	-0.00766	0.00604	-0.0265	0.054129
AC079305.	-0.012878781	-0.044442331	-0.00773	0.004505	-0.00368	0.023195
AC079741.	-0.037016853	-0.075493257	0.055598	0.025962	-0.0615	0.011352
AC079767.	-0.004642583	-0.058095867	-0.11189	-0.04825	0.138365	-0.07793
AC079807.	-0.051050529	-0.009939829	-0.00132	-0.07541	0.238962	0.04302
AC083843.	-0.024436122	-0.004717648	-0.03682	-0.0296	0.03344	0.102632
AC083949.	0.004105583	-0.048891644	0.224628	0.009921	0.01485	-0.15094
AC084219.	-0.039363718	-0.020997308	0.125248	0.008566	0.004569	0.043448
AC087501.	-0.050759247	0.018561515	-0.16402	0.02157	-0.05878	-0.04593
AC090627.	-0.029422372	0.02196659	-0.01244	-0.00944	0.033254	-0.07734
AC091133.	0.003136281	-0.011764815	0.037779	-0.02093	0.023894	-0.05456
AC091633.	-0.034082099	-0.010547547	0.051659	-0.01999	0.044547	-0.0007
AC092192.	-0.019150393	-0.029644206	0.068717	-0.00931	0.017231	-0.08334
AC092620.	-0.001242628	-0.088048139	-0.3531	-0.01853	0.065996	0.07666
AC092660.	-0.026876353	-0.011003516	0.065178	-0.00068	-0.01355	-0.09012
AC092667.	-0.040196234	-0.045413118	0.066269	-0.0089	0.044893	-0.04322
AC100830.	-0.019827424	-0.059084208	0.244499	-0.03298	0.102062	0.122493
AC104667.	-0.036930127	-0.033118527	-0.00262	-0.0269	0.066652	0.050565
AC106801.	-0.025385002	-0.040964266	0.01837	-0.00263	-0.06322	-0.06906
AC108056.	-0.046009743	-0.023783085	-0.05662	0.002435	0.008194	0.013522
AC112198.	-0.031533962	-0.058612046	-0.04796	0.052357	-0.13564	0.031403
AC114730.	-0.055746034	-0.019617551	-0.0065	-0.01013	0.042046	0.143514
AC114752.	-0.048085017	0.014309719	0.00151	-0.03579	0.117104	0.018956
AC124997.	-0.059870755	-0.021656857	-0.14427	-0.0248	0.061088	-0.03729
AC136289.	-0.046242933	-0.009144249	-0.07038	-0.00328	0.005207	-0.01853
AC139100.	-0.046392197	-0.049386115	0.058781	-0.0136	0.048158	0.118619

AC141928.	-0.039954469	-0.077064538	-0.03945	0.015182	-0.09785	-0.04726
AC144652.	-0.004986484	0.021393404	0.059088	0.001537	-0.03404	-0.14289
AC145343.	-0.030842758	-0.015933853	-0.06424	-0.00906	0.035937	0.048982
ACAA1	0.044120818	-0.071560527	-0.19027	-0.01625	0.057873	0.102848
ACAA2	-0.01179374	0.039816822	0.030922	0.071186	-0.23077	-0.05521
ACACA	0.002304559	0.030463734	0.016559	0.077488	-0.24663	0.038202
ACACB	0.000289553	-0.115846947	0.12057	-0.05741	0.12779	-0.15216
ACAD10	-0.022143588	-0.070933951	0.046128	-0.01328	0.069126	0.119528
ACAD8	0.016084755	0.049612494	0.084392	0.030243	-0.04239	0.059379
ACAD9	0.024360453	0.045241648	-0.06322	0.053089	-0.14062	0.223235
ACADL	-0.062023083	-0.021513936	0.170892	0.036888	-0.07128	-0.05114
ACADM	0.024177784	0.034075032	0.083985	0.027797	-0.08359	-0.03209
ACADS	0.010963147	-0.034253888	-0.07027	-0.03634	0.020856	0.107458
ACADSB	0.001999692	0.0577984	0.189278	-0.00755	0.054161	-0.1434
ACADVL	0.059843327	0.013822738	-0.2006	0.02661	-0.06483	0.281557
ACAN	-0.054144129	-0.074798132	-0.11005	-0.00357	-0.08705	0.078455
ACAP1	0.009656494	-0.007160453	0.228399	0.025685	-0.09612	-0.2974
ACAP2	0.021887769	0.002521137	-0.23409	-0.01814	0.045146	0.135266
ACAP3	-0.006778605	-0.103855515	0.023907	-0.00139	0.000204	0.308159
ACAT1	-0.063636057	-0.037683049	0.152006	-0.05084	-0.03	-0.15446
ACAT2	0.028030009	0.06862913	0.129543	0.096262	-0.29537	-0.2533
ACBD3	0.037409802	0.053175259	-0.15411	0.029114	-0.08368	0.025464
ACBD4	-0.005925739	-0.078908439	0.241642	6.45E-05	-0.06046	-0.12098
ACBD5	0.027821588	0.184895029	-0.07105	0.012882	0.000363	0.220407
ACBD6	0.02536275	-0.026195683	0.095437	0.116752	-0.31158	0.008085
ACCS	0.011752541	-0.121074846	-0.0973	-0.08803	0.229249	0.059789
ACD	0.038604548	0.085602952	0.136811	0.044807	-0.1591	-0.25439
ACE	-0.017362199	-0.094834462	0.005817	-0.02546	0.06795	0.016778
ACE2	-0.058868391	-0.050495956	-0.02009	-0.00425	0.021714	-0.10833
ACER1	-0.029426231	-0.11489896	0.113274	0.03634	-0.07845	-0.04959
ACER3	0.00651549	-0.074364655	-0.26229	-0.00563	0.045216	0.561459
ACHE	-0.0192193	-0.134546836	0.008384	-0.01311	0.027536	-0.01466
ACIN1	0.034030525	-0.008143717	0.061336	0.03471	-0.12692	-0.20834
ACKR1	-0.035285705	-0.055947011	-0.05554	-0.00021	-0.019	-0.07544
ACKR2	0.013018698	-0.060029177	0.03181	-0.05849	-0.07702	-0.07759
ACKR3	-0.010221694	0.115715676	0.137441	-0.05649	0.172991	-0.13739
ACKR4	-0.021092019	-0.040251815	0.040535	-0.00803	0.014464	-0.06245
ACLY	0.055165319	0.109940707	-0.08335	0.046891	-0.21445	-0.05405
ACMSD	-0.0549706	-0.032376125	0.05068	0.006578	-0.00458	0.023976
ACN9	0.006603522	0.01662852	0.138954	0.039824	-0.08913	-0.01445
ACO1	-0.007970456	0.076021383	0.059638	0.004609	-0.00463	0.216108
ACO2	0.026466869	0.024843487	-0.10092	0.062734	-0.16647	0.213266
ACOT11	-0.041284309	-0.085263751	-0.05093	0.038526	-0.12077	-0.05608
ACOT12	-0.025364268	-0.016704912	-0.03716	0.121792	-0.11292	0.008426
ACOT13	0.022983849	0.032771563	0.049995	0.089571	-0.23689	0.165109
ACOT4	-0.003569449	0.250651704	0.117805	0.049666	-0.13169	-0.22482
ACOT6	-0.040100979	-0.05047409	-0.07504	0.007477	-0.00966	0.070156
ACOT7	0.004895137	0.128061457	-0.02022	0.15963	-0.44634	-0.21568
ACOT8	0.006273152	0.050331971	-0.07918	0.057308	-0.1656	0.281407
ACOT9	0.044180871	-0.029009948	-0.23474	-0.03943	0.10609	0.31547
ACOX1	0.003656909	0.0410211	-0.31869	-0.00878	0.08838	0.298751

ACOX2	-0.017565033	-0.067811005	-0.18509	0.026674	-0.05214	0.501185
ACOX3	0.000757361	-0.060457529	-0.1599	-0.0022	-0.05162	0.182216
ACOXL	-0.057684671	-0.023004055	-0.11027	0.026754	-0.08219	0.180293
ACP1	0.032508575	0.116310049	0.234333	0.042575	-0.15424	-0.04934
ACP2	0.021782274	0.037582198	-0.10254	-0.03137	0.017598	0.420402
ACP5	0.018311969	-0.10329784	-0.10326	-0.07994	0.182486	0.002626
ACP6	-0.002201224	-0.084067245	0.233427	-0.03585	0.069398	-0.2322
ACPP	0.023021281	-0.104531064	-0.30268	-0.00851	0.108477	0.3178
ACPT	-0.012786769	-0.053122705	0.033647	-0.01918	-0.06879	-0.07005
ACR	-0.033079445	-0.04218427	0.053516	0.008873	-0.02264	-0.04301
ACRBP	-0.060429784	-0.114817811	-0.16062	-0.05211	0.682092	0.187913
ACRC	0.009450413	0.086454198	0.212378	-0.01536	0.06206	-0.25164
ACRV1	-0.020255052	-0.030008441	0.059131	-0.01761	-0.02695	-0.06314
ACSBG1	-0.02420264	-0.069940854	-0.0623	-0.11268	0.338386	-0.03392
ACSBG2	-0.028870924	-0.018001114	0.085403	0.042977	-0.10145	-0.02208
ACSF2	0.006486829	-0.11478439	-0.09483	0.006216	-0.01943	0.009556
ACSF3	-0.118920807	0.049853755	-0.02551	-0.08703	0.071075	0.006183
ACSL1	0.014706578	-0.052360313	-0.38278	0.002112	0.006831	0.357534
ACSL3	0.026118403	0.051168478	0.042001	0.018138	-0.04204	-0.12136
ACSL4	-0.016563634	-0.0850389	-0.22866	-0.02049	-0.06469	0.080464
ACSL5	0.03860678	0.0904006	0.124294	0.011752	-0.04361	-0.13079
ACSL6	-0.025804731	-0.083955773	0.407114	-0.04514	0.131676	-0.18639
ACSM1	-0.049981549	-0.100731501	-0.07814	-0.00531	0.049898	0.035641
ACSM2A	-0.059648813	-0.037888806	0.119008	0.01213	-0.02763	0.013173
ACSM2B	-0.049079741	-0.083458561	0.119585	0.002491	0.011883	-0.03566
ACSM3	-0.013540706	-0.05602097	0.265918	-0.01113	0.065173	-0.12698
ACSM5	-0.042435086	-0.053998455	0.096725	0.006513	-0.05075	-0.11485
ACSS1	-0.031632511	-0.015075997	-0.01779	-0.01922	-0.04836	0.049983
ACSS2	0.015464856	-0.164382739	-0.32296	-0.04463	0.106993	0.397788
ACSS3	-0.030750858	-0.024031194	0.026123	-0.00507	-0.01396	-0.08133
ACTA1	-0.032174906	-0.001908624	-0.05511	0.015356	-0.015	0.027842
ACTA2	0.004721235	0.065551699	0.01047	0.075477	-0.21902	0.193179
ACTA2-AS:	-0.024915685	-0.039995563	-0.08955	-0.01681	0.057884	-0.05296
ACTB	0.059877232	0.142332638	-0.32561	0.117887	-0.07168	0.156783
ACTC1	-0.030342368	-0.012451001	-0.04432	0.007792	-0.07961	0.171921
ACTG2	-0.046814023	-0.04620089	-0.05018	0.022631	-0.0677	0.019837
ACTL10	-0.010747095	-0.023642694	-0.06512	-0.00085	-0.05464	-0.02671
ACTL6A	0.01559011	0.055940428	0.054373	0.045087	-0.12622	0.109046
ACTL6B	-0.043365679	-0.039532538	-0.09539	0.007145	0.007189	0.056256
ACTL7A	-0.032814631	-0.039444381	-0.09526	0.034729	-0.08263	0.075557
ACTL7B	-0.032780188	-0.091240295	0.001211	0.026133	-0.06191	-0.05075
ACTL8	-0.026309748	-0.055783344	0.035901	0.001766	-0.07789	-0.07625
ACTL9	-0.0344156	-0.026564427	0.072247	0.015199	-0.02468	0.132585
ACTN1	-0.004177106	-0.143964053	0.147044	-0.11088	0.247396	0.11368
ACTN2	-0.058719828	-0.050966952	0.079318	-0.00797	-0.027	-0.1887
ACTN3	-0.033273666	-0.078226532	0.00998	-0.00619	0.043174	0.102646
ACTN4	0.049320107	0.201505474	-0.15039	0.033053	-0.08295	0.015848
ACTR10	0.09125574	0.176743282	0.022644	0.02644	-0.04749	-0.09642
ACTR1A	0.056416913	0.130294879	-0.31384	0.024989	-0.09663	0.312766
ACTR1B	0.042544178	-0.020615557	0.186178	0.034132	-0.12382	-0.11959
ACTR2	0.071088265	0.121638059	-0.17928	-0.01588	0.024552	0.059879

ACTR3	0.078814427	0.192488107	-0.24518	0.053611	-0.10069	-0.07042
ACTR3B	-0.007110576	-0.012436901	0.093415	0.019622	-0.04793	0.003383
ACTR3BP2	-0.019163812	-0.017019845	0.167985	0.00021	-0.01669	-0.11322
ACTR3C	-0.026898078	0.037483044	0.193347	0.042223	-0.09187	-0.16413
ACTR5	0.013694582	0.042092732	0.137082	0.036448	-0.10304	-0.09022
ACTR6	0.010912291	0.072948565	0.244349	0.032343	-0.08869	-0.2292
ACTR8	0.023137083	0.071092497	0.013007	0.072984	-0.27832	-0.17899
ACTRT2	-0.036182697	0.014131566	-0.01946	0.038297	-0.08686	0.009299
ACTRT3	-0.029564593	-0.015533463	-0.02994	0.065308	-0.17157	-0.02694
ACVR1	0.01301439	-0.157581387	-0.00946	-0.0319	0.042766	0.25811
ACVR1B	-0.049366984	-0.112462554	0.03468	-0.05599	0.073077	0.216063
ACVR1C	-0.015331859	-0.107183106	0.447198	-0.07325	0.212493	-0.20894
ACVR2A	0.008827837	0.082444053	-0.03055	-0.05051	0.159675	0.186246
ACVR2B	-0.000475245	-0.070798065	0.276796	0.006389	0.138206	-0.1872
ACVR2B-AS	-0.020983284	-0.026555878	0.044666	-0.00109	-0.04982	-0.10296
ACVRL1	-0.034624612	-0.151759248	-0.0824	-0.00121	0.01578	0.127228
ACY1	-0.030212497	-0.057029715	0.012843	########	0.006528	-0.01614
ACY3	-0.037145103	-0.091666554	-0.14983	0.034764	-0.06677	0.019817
ACYP1	0.014360868	0.060334998	0.197115	0.050915	-0.14157	-0.18069
ADA	0.018303593	0.115736524	0.104204	0.027354	-0.08167	-0.10729
ADAD1	-0.04676358	-0.080935985	0.086912	0.009125	-0.0153	-0.09997
ADAD2	-0.019956727	-0.055562909	-0.09065	0.029031	-0.12027	-0.05827
ADAL	-0.104866934	0.010128928	0.060975	0.060235	-0.17716	-0.10207
ADAM10	0.014307106	0.121877323	-0.27994	-0.04145	0.088312	0.237097
ADAM11	-0.036620177	-0.066226702	-0.07096	0.023521	-0.07111	-0.02355
ADAM12	-0.00895582	0.010590805	0.160258	-0.01866	0.159706	-0.12747
ADAM15	-0.002870828	-0.064272025	-0.09639	-0.02868	0.018674	0.232516
ADAM17	0.050483355	0.024255247	-0.3048	0.00122	0.00119	0.171757
ADAM18	-0.046547308	-0.056843345	0.030087	-0.00406	0.029436	-0.05392
ADAM19	0.006773688	-0.168158225	-0.25768	0.044698	-0.14529	-0.12606
ADAM2	-0.069328591	-0.045643381	-0.05159	-0.0313	0.072104	0.055903
ADAM20	-0.040023088	-0.087548355	-0.02142	0.017305	-0.05204	-0.03881
ADAM21	-0.042431576	0.005136134	-0.0176	0.018498	-0.0438	-0.10882
ADAM22	-0.007423695	-0.056621208	0.055639	0.116788	-0.21724	-0.11143
ADAM23	-0.014527132	-0.06640029	0.092902	-0.03052	-0.04537	-0.11831
ADAM28	-0.003794296	-0.014435326	-0.31231	-0.06806	0.117415	-0.13638
ADAM30	-0.105308397	-0.097924228	-0.06484	0.024469	0.027169	-0.16617
ADAM32	-0.050514176	-0.082957627	0.003826	0.04971	-0.1296	0.000488
ADAM33	0.019154356	-0.065983951	0.004381	-0.01296	0.012953	-0.19375
ADAM3A	-0.052829131	-0.020740035	-0.01569	-0.01909	0.074042	0.136071
ADAM5	-0.047385474	-0.001864674	0.065228	0.025197	-0.05482	-0.03177
ADAM6	-0.030561254	-0.027235309	-0.01129	-0.00306	-0.00283	-0.11382
ADAM7	0.043821766	-0.027579069	-0.06373	0.058183	-0.04559	-0.11354
ADAM8	0.012400388	0.082499256	-0.17077	-0.04782	0.113169	-0.11435
ADAM9	-0.014501815	-0.046168964	-0.18956	0.022967	-0.05512	0.181423
ADAMDEC1	-0.017582055	-0.07157059	-0.05036	0.004555	-0.01304	0.167808
ADAMTS1	-0.007307206	0.339651143	-0.10015	-0.0141	0.031278	-0.10801
ADAMTS10	-0.015613025	-0.039675792	0.134883	-0.02306	0.060515	-0.00125
ADAMTS12	-0.038801303	-0.053993973	0.034661	-0.00462	-0.02879	-0.08773
ADAMTS13	-0.035358586	-0.05254405	-0.02133	0.015546	-0.12244	-0.13223
ADAMTS14	-0.028771165	-0.082743096	0.003853	0.009825	-0.07899	-0.09687

ADAMTS15	-0.039982211	-0.064914142	-0.11201	0.011465	-0.01487	0.024272
ADAMTS16	-0.026791379	-0.050225542	-0.01742	0.018642	-0.08038	0.003434
ADAMTS17	-0.008253974	0.014675096	0.208953	0.033548	0.01446	-0.15386
ADAMTS18	-0.04284444	0.000539925	-0.06311	0.010811	-0.00621	-0.01978
ADAMTS19	-0.147494256	-0.106629347	0.025476	0.004269	0.067382	-0.0144
ADAMTS2	-0.032980218	-0.059345172	-0.02509	-0.01075	0.018227	-0.10828
ADAMTS20	-0.034738719	-0.06237934	0.060022	-0.01351	0.02488	-0.15106
ADAMTS3	-0.032923036	-0.075399001	4.29E-06	-0.02343	0.068359	-0.11036
ADAMTS4	-0.036247489	-0.051710534	-0.01656	-0.00031	-0.02841	-0.03382
ADAMTS5	-0.011618971	-0.082353008	-0.13194	-0.11608	0.362578	0.697807
ADAMTS6	-0.033997237	-0.041826008	0.275073	0.153613	0.004106	-0.20332
ADAMTS7	-0.042237287	-0.080690324	-0.08916	0.001161	-0.02575	0.054153
ADAMTS8	-0.022289124	0.011875007	-0.08517	0.025239	-0.00171	-0.00789
ADAMTS9	-0.057665242	-0.075612273	0.115601	0.007126	-0.07841	-0.20152
ADAMTS9-/	-0.082222866	-0.002048529	0.145744	-0.02948	0.01158	-0.16099
ADAMTSL1	-0.114009914	-0.102438928	-0.11645	0.081834	-0.0526	0.094597
ADAMTSL2	-0.030249596	-0.04356868	-0.07126	0.002404	0.021893	0.12382
ADAMTSL3	-0.043599503	-0.112076361	-0.10406	0.004324	-0.0356	0.056429
ADAMTSL4	-0.014563942	-0.063001532	-0.19213	-0.13791	0.355122	0.52827
ADAMTSL5	-0.034614446	-0.117171941	0.02468	0.023235	-0.09697	-0.06979
ADAP1	0.022305667	0.063827667	-0.12764	-0.02792	0.054614	0.336414
ADAP2	0.016809418	-0.114114051	-0.27281	-0.0911	0.256294	0.579345
ADAR	0.06465497	0.108507873	-0.07856	0.055265	-0.1225	-0.28951
ADARB1	0.020976431	-0.080231194	0.037808	0.029083	-0.07347	-0.06593
ADARB2	-0.048688241	-0.013107289	0.003998	0.022074	-0.05609	-0.01885
ADARB2-AS	-0.029558826	-0.041585215	-0.06036	-0.01618	-0.05301	-0.08732
ADAT1	0.022539434	-0.009722094	0.211621	0.027721	-0.08069	-0.17602
ADAT2	-0.011944959	-0.019746414	0.186559	0.020044	-0.04873	-0.1873
ADAT3	-0.010491227	-0.064153742	-0.00797	0.022474	-0.11837	-0.10842
ADCK1	0.014370938	-0.103887471	-0.01905	-0.03167	0.062942	0.172578
ADCK2	0.006321088	-0.054028579	0.051257	0.041724	-0.10213	-0.04416
ADCK3	0.027694174	-0.065344544	-0.00956	-0.03124	0.102617	-0.02343
ADCK4	0.000490381	-0.004036061	-0.01197	-0.00625	-0.03276	-0.00926
ADCK5	-0.005900908	-0.061640778	-0.00172	0.019914	-0.01779	0.166607
ADCY1	-0.026167119	-0.11815482	0.0551	0.091686	-0.07679	-0.0738
ADCY10	-0.063595274	-0.026699943	0.056814	0.007484	0.00564	-0.10141
ADCY10P1	-0.004839701	-0.080471862	0.166047	-0.08471	0.265302	-0.21732
ADCY2	-0.018090267	-0.08310361	-0.0239	-0.01302	-0.07489	-0.01689
ADCY3	0.006800615	-0.161963303	0.106435	0.092005	-0.25213	-0.2179
ADCY4	0.000188352	-0.088420602	-0.05972	0.010387	-0.07215	-0.03214
ADCY5	-0.042180865	-0.054050822	-0.00169	-0.00496	-0.01484	-0.08953
ADCY6	-0.030764037	-0.047301947	-0.09251	-0.03755	0.040323	-0.05075
ADCY7	0.025016496	0.117519697	-0.07008	-0.03036	0.093607	0.108044
ADCY8	-0.027632436	0.023038055	-0.03701	0.018042	-0.02192	0.050581
ADCY9	-0.002982712	0.077677287	-0.01602	-0.02724	0.083457	0.183828
ADCYAP1	-0.054277991	-0.067772052	-0.05875	0.027594	-0.04184	-0.00325
ADCYAP1R	-0.046456147	-0.033627198	-0.02412	8.45E-05	0.01272	0.058449
ADD1	0.037910045	0.097181625	0.012992	-0.01493	0.081664	-0.25905
ADD2	-0.011856266	-0.08664974	0.034586	-0.06101	-0.07653	-0.1049
ADD3	0.030036296	0.089045673	0.123174	-0.04902	0.115968	-0.29025
ADD3-AS1	0.004020693	0.000734496	0.109702	-0.09503	0.110072	0.050103

ADGB	-0.076081736	-0.036508638	0.018341	-0.05168	0.106453	0.095378
ADH1A	-0.040320652	-0.06206745	-0.23372	-0.01525	0.07184	0.159123
ADH1B	-0.075851148	-0.074468534	0.006419	0.00946	-0.03094	0.042751
ADH1C	-0.039687372	-0.07248281	-0.12921	-0.02287	0.079666	0.091844
ADH4	-0.098634496	-0.084711744	-0.06432	-0.0251	-0.00269	-0.02393
ADH5	0.025519071	0.092203409	0.166211	0.029694	-0.06727	-0.09778
ADH6	-0.042459953	-0.096301921	-0.00135	0.027162	-0.06361	-0.12445
ADH7	-0.04865203	-0.046188646	-0.04748	-0.00505	0.046552	-0.00771
ADHFE1	0.013612955	0.119587368	0.256342	-0.08158	0.218522	0.02476
ADI1	0.03522001	-0.038636104	-0.28035	-0.05837	0.131061	0.182779
ADIG	-0.027515072	-0.036557492	0.018241	-0.0241	-0.03813	-0.12615
ADIPOQ	-0.023821349	-0.043913629	-0.00139	-0.00345	0.009337	-0.07542
ADIPOR1	0.054971495	-0.059274924	-0.35586	0.006563	-0.00478	0.13339
ADIPOR2	0.067108874	0.271878546	-0.15851	0.027293	-0.07986	0.077351
ADIRF	-0.024197031	-0.104321329	0.000486	-0.00224	0.026959	0.069339
ADIRF-AS	-0.024836776	-0.136291838	0.109493	0.024354	-0.07814	0.118624
ADK	0.012940958	-0.106556851	-0.08087	0.025879	-0.06162	0.06129
ADM	0.002262663	-0.063843692	-0.22731	-0.0365	0.112192	0.295791
ADM2	-0.009438616	-0.089036868	-0.08032	-0.02169	-0.04772	-0.06989
ADM5	-0.017465556	-0.124155479	0.070933	0.062099	-0.15429	-0.08842
ADNP	0.075401022	0.129454642	0.095287	0.051795	-0.13396	-0.30231
ADNP2	0.034746648	0.030680637	0.255106	0.055143	-0.18639	-0.22606
ADO	0.019671326	0.096505107	-0.05044	0.038012	-0.11307	0.20054
ADORA1	-0.027496049	-0.12645402	-0.07896	-0.02876	0.011473	-0.10296
ADORA2A-/	-0.041442962	-0.056794394	-0.08695	0.038393	-0.09914	-0.02123
ADORA2B	-0.00666299	-0.058423057	-0.12951	-0.06528	0.173117	0.266189
ADORA3	-0.010489802	-0.086611676	-0.18906	-0.02717	0.052638	0.139939
ADPGK	0.044026274	-0.050199984	-0.30544	-0.02585	0.085692	0.159298
ADPRH	0.010743344	-0.00455398	-0.3077	-0.13558	0.340455	0.275763
ADPRHL1	-0.040607952	0.004733867	-0.01286	0.00497	-0.06932	0.012113
ADPRHL2	0.05713771	0.001927794	-0.16136	0.024204	-0.07162	0.144403
ADPRM	0.01223283	-0.112610405	0.387712	-0.0351	0.081908	-0.17664
ADRA1A	-0.085452467	-0.096335054	-0.03311	0.023021	0.053175	0.003694
ADRA1B	-0.036185043	-0.002388568	-0.05764	0.004513	0.009349	0.084754
ADRA1D	-0.053931559	-0.059494492	-0.06827	0.015093	-0.00923	0.092461
ADRA2A	-0.041854536	-0.023740463	-0.01575	-0.1595	0.481894	0.008122
ADRA2B	-0.008131086	-0.090716366	-0.07542	0.01928	-0.08404	0.065111
ADRA2C	-0.025039856	-0.10250592	-0.10463	-0.00492	-0.02743	-0.07254
ADRB1	-0.021569056	0.154240593	-0.0934	-0.01015	0.211208	0.207169
ADRB2	0.016612762	0.211221244	0.100588	-0.10707	0.285739	-0.06218
ADRB3	-0.047131965	-0.042469745	-0.07262	0.003368	-0.01331	-0.09025
ADRBK1	0.063910584	-0.016292619	-0.10002	-0.01785	0.00987	0.099937
ADRBK2	0.030240007	-0.175661551	-0.38926	-0.06883	0.145212	0.403003
ADRM1	0.054267958	0.058034241	-0.2226	0.109054	-0.2954	0.219336
ADSL	0.041938054	-0.022032444	0.264299	0.050465	-0.13935	0.055533
ADSS	0.020193669	0.132625695	0.00571	0.052638	-0.14155	0.14708
ADSSL1	-0.030359056	-0.084839338	0.039294	0.007567	-0.01144	0.286376
ADTRP	-0.009832165	-0.087008499	0.100102	-0.04497	0.11128	-0.16713
AEBP1	0.002120627	-0.088452393	0.007636	-0.02262	0.03443	-0.28736
AEBP2	0.008059345	0.114473567	0.23078	0.069033	-0.20537	-0.10903
AEN	0.013940247	-0.047257875	0.127029	0.078071	-0.26931	-0.26526

AES	0.028901997	0.098479502	0.263338	0.008862	-0.02476	-0.28625
AF001548.	-0.001753869	-0.040772577	0.01917	-0.02754	0.069259	-0.08642
AF007147	0.016826966	0.003392539	0.015518	-0.01626	0.030531	-0.24417
AF067845.	-0.04866229	-0.005978082	-0.05096	-0.00751	0.06574	0.010798
AF070581	-0.044565971	-0.083554442	0.012659	-0.02691	0.042009	-0.0658
AF086184	-0.029652964	-0.043829961	-0.10135	-0.0136	0.051692	-0.03869
AF090939	-0.010470174	-0.02668338	0.090312	0.029268	-0.07417	-0.07867
AF131215.	-0.013745855	0.099077677	0.171814	-0.01872	0.062446	-0.19151
AF131215.	-0.033316134	0.056454979	0.058134	-0.03025	0.102241	-0.11838
AF186192.	-0.033352136	-0.034318894	-0.02811	0.032286	-0.07579	-0.05352
AF198444	-0.042552702	-0.026316496	0.046202	-0.01701	0.043687	0.06914
AF213884.	-0.015404145	-0.051684688	0.03791	-0.00793	-0.08956	-0.0727
AF279780	-0.043650782	-0.044424672	0.012179	-0.00071	0.020265	-0.09273
AF289551	0.004280676	0.242488072	0.113701	-0.01381	0.074604	-0.23257
AF520793	0.001081905	0.017257739	-0.09238	-0.04627	0.14917	-0.18102
AFAP1	-0.020755305	-0.05120902	0.052363	-0.04201	0.09887	0.014816
AFAP1-AS	-0.019636708	-0.027596652	-0.0152	0.054141	-0.12185	-0.00104
AFAP1L1	-0.048912383	-0.050821923	-0.00629	0.013356	-0.03854	0.029311
AFAP1L2	-0.014081051	0.00620887	0.084102	0.082445	-0.22764	-0.1072
AFF1	-0.042180625	0.050552597	-0.22807	-0.00592	-0.09243	-0.14918
AFF2	-0.056248993	-0.085241598	-0.07955	0.00883	0.002747	0.010775
AFF3	-0.014671235	-0.098643635	-0.17387	-0.0217	0.081607	-0.15096
AFF4	-0.015375748	-0.047937386	-0.10148	0.026276	0.01196	-0.06738
AFG3L1P	0.010102494	0.012140305	0.028033	0.008184	-0.07153	0.015066
AFG3L2	0.045658475	0.02125069	0.088046	0.084244	-0.20316	-0.01764
AFM	-0.04788829	-0.095773103	0.023266	0.03791	-0.09951	0.133153
AFMID	-0.019870868	-0.089103545	-0.12243	0.027293	-0.07037	0.342604
AFP	-0.040199072	-0.028700421	-0.00194	0.033397	-0.06279	-0.00751
AFTPH	0.086389194	0.007356731	-0.34463	0.005702	0.025156	-0.15282
AGA	-0.010265534	-0.017650852	0.177882	-0.0183	-0.05389	0.127569
AGAP1	-0.003263219	0.310343293	0.274169	-0.04258	0.114404	-0.22794
AGAP11	-0.048461902	-0.022169107	0.143293	-0.00436	0.047118	-0.09185
AGAP2	-0.008273222	0.030066541	0.168111	0.053785	-0.14097	-0.25346
AGAP2-AS	-0.030829756	-0.037341458	0.000216	0.008751	-0.02365	-0.03305
AGAP3	0.001711858	-0.040802518	-0.04532	0.001526	-0.08866	-0.01787
AGAP4	-0.010814654	0.035917456	0.009241	-0.03419	0.049424	-0.06907
AGAP6	-0.019218526	0.027548589	0.181146	-0.02322	0.009	-0.17491
AGAP9	-0.007805526	0.031862623	0.117051	-0.00636	0.046769	0.012635
AGBL1	-0.031632448	-0.049725289	0.021277	0.014632	-0.03037	-0.10566
AGBL2	-0.016074284	0.002108731	0.216071	-0.01404	0.052556	-0.26253
AGBL3	-0.080563429	-0.057688347	-0.02402	0.038031	0.045943	-0.13719
AGBL4	-0.047359934	-0.078495314	-0.0294	0.020572	-0.03882	0.032218
AGBL5	0.009222639	-0.06140894	0.155823	0.0917	-0.1871	-0.03858
AGER	-0.012473008	-0.152516376	-0.04302	-0.01068	0.028215	-0.05055
AGFG1	0.023921202	-0.102032745	-0.13469	-0.06326	0.140393	0.360527
AGFG2	-0.018078328	-0.012065614	0.080151	-0.05088	-0.28228	-0.04991
AGGF1	0.005184486	0.060228067	-0.05156	-0.03663	0.176803	0.069481
AGK	0.006380939	0.203567252	0.101953	0.056495	-0.14396	-0.09202
AGL	0.022571871	0.089305669	0.199138	0.00553	-0.03161	-0.34952
AGMAT	0.014368344	-0.094083906	0.354966	0.023694	3.74E-05	-0.28285
AGO1	-0.00315189	-0.007223476	-0.05372	-0.06671	0.088607	0.286782

AGO2	0.022175017	0.051621031	-0.07951	0.033837	-0.096	0.045895
AGO3	-0.006534005	0.035634231	0.105547	-0.01294	0.029175	-0.03413
AGO4	0.004983021	0.000367864	-0.2543	-0.06955	0.195994	0.264577
AGPAT1	0.035072311	-0.067160746	-0.01309	-0.02972	0.069217	-0.1735
AGPAT2	0.010342001	-0.12576266	-0.17266	-0.02785	-0.00756	0.15407
AGPAT3	-0.014040655	-0.104025921	0.047235	0.031815	-0.11907	0.428009
AGPAT4	0.005682193	0.19595748	-0.0502	-0.00272	0.024901	0.226103
AGPAT4-I	0.006493438	0.1356364	0.047255	0.02174	-0.03963	0.082498
AGPAT5	0.009496967	-0.019380175	-0.05445	0.02611	-0.05877	-0.05932
AGPAT6	-0.014373414	-0.013752473	0.172062	-0.02933	0.084169	-0.01204
AGPAT9	0.013127424	-0.119348636	-0.20978	-0.03846	0.095577	0.367147
AGPS	0.028119347	0.115425487	-0.07689	0.040199	-0.1493	0.421293
AGR2	-0.043694255	-0.007099571	-0.08891	-0.00515	0.037612	-0.00372
AGR3	-0.072305101	0.010221722	-0.06684	-0.00485	-0.00407	0.021815
AGRN	-0.016624031	-0.088919004	0.012747	0.000773	-0.1252	-0.04591
AGRP	-0.039936151	-0.051602156	0.056451	0.025924	-0.10216	0.004248
AGT	-0.028605572	-0.05747339	0.026275	0.058437	-0.1592	0.017594
AGTPBP1	0.025045185	0.079277791	-0.12377	-0.03619	0.12275	0.299225
AGTR1	-0.055909778	-0.056225793	-0.02128	0.031531	-0.06341	-0.09557
AGTR2	-0.062876318	-0.057369032	-0.14595	0.006139	0.058706	0.056576
AGTRAP	0.009142515	0.066705152	-0.19899	-0.04806	0.148257	0.4823
AGXT	-0.027518074	-0.051528197	-0.08886	0.083937	-0.18032	0.061343
AGXT2	-0.033649588	0.035436953	-0.00063	0.046175	-0.12491	0.059755
AHCTF1	0.020779974	0.016068271	-0.00243	0.036662	-0.09594	-0.30836
AHCY	0.01812494	-0.114097492	-0.00361	0.096039	-0.25664	0.135751
AHCYL1	0.065260022	0.055864657	-0.29235	0.061382	-0.1617	0.348704
AHCYL2	-0.002824319	-0.03619065	-0.00384	0.058506	-0.18093	0.179675
AHDC1	0.027124204	0.120199342	-0.01681	0.017306	-0.06236	-0.16793
AHI1	0.014833184	0.016247392	0.002953	0.077684	-0.2393	-0.089
AHNAK	-0.038107881	0.007028789	-0.06171	-0.10071	0.212496	0.448146
AHNAK2	-0.034884587	-0.102901738	-0.101	-0.02602	0.096033	0.074813
AHR	0.019266098	-0.085237812	-0.17272	-0.0035	0.017397	0.462266
AHRR	-0.012755092	0.017427818	-0.06443	-0.00564	-0.04717	-0.01088
AHSA1	0.049253503	0.105420043	0.059871	0.087597	-0.23838	-0.1184
AHSA2	0.02180002	0.024364733	0.174898	-0.05273	0.161287	-0.21648
AHSG	-0.055711992	-0.07652959	-0.07715	0.02321	-0.031	-0.00541
AHSP	-0.029416432	-0.035297524	-0.01996	-0.03028	0.070862	0.074012
AICDA	-0.023490107	-0.033387553	0.031204	0.031136	-0.08132	-0.02839
AIDA	0.028073994	0.043258671	0.104632	0.013339	-0.03076	-0.2522
AIF1	0.019620228	-0.164079002	-0.16557	-0.03933	0.107354	0.503954
AIF1L	-0.037559119	-0.033981232	-0.05249	0.032519	-0.11476	-0.02295
AIFM1	0.028290847	0.046122495	0.028641	0.062321	-0.18042	0.060738
AIFM2	-0.010180144	0.023445227	0.012883	-0.00878	-0.06583	-0.07869
AIFM3	-0.010689533	-0.114640252	-0.18846	-0.01968	0.066251	0.362433
AIG1	-0.029562603	-0.040957808	0.034704	0.093404	0.073506	0.003068
AIM1	0.037022807	0.008358379	0.124956	0.038643	-0.08669	0.090009
AIM1L	-0.026569249	-0.074533784	0.04954	0.015231	-0.05056	-0.09821
AIM2	0.00385401	-0.120282779	-0.17322	0.00655	-0.03879	0.09984
AIMP1	0.014547488	-0.014880092	0.148445	0.016937	-0.0585	0.124911
AIMP2	0.035995172	0.073980001	-0.02781	0.089943	-0.20814	0.284748
AIP	0.041316458	0.019205392	0.297781	0.03313	-0.11962	-0.02293

AIPL1	-0.037946856	-0.085415286	0.017604	0.007113	-0.05544	-0.09828
AIRE	-0.027027917	-0.113728275	0.072605	0.016555	-0.03829	-0.00833
AJAP1	-0.001223844	-0.08843997	-0.05908	0.008278	0.03559	-0.15368
AJUBA	-0.039959041	-0.020041369	0.088423	0.095624	-0.1048	-0.11559
AK000798	-0.023352634	0.224194919	0.158432	-0.01412	0.04557	-0.12654
AK021537	-0.022974979	-0.056090884	0.167089	0.009474	-0.06402	-0.15138
AK021804	-0.046564413	-0.047126635	0.09024	0.007953	0.002011	-0.09442
AK021933	-0.026616552	0.001381112	-0.01428	0.027819	-0.09605	-0.05262
AK021977	-0.014984961	-0.028607718	0.089334	0.005971	-0.0265	-0.10573
AK024936	-0.049530095	-0.014006149	0.103419	-0.01603	0.045551	-0.02341
AK025288	-0.003884103	-0.031943434	-0.10879	0.02615	-0.06094	0.179593
AK026905	-0.005667931	-0.015077603	-0.19942	-0.00235	-0.00266	0.234787
AK054988	-0.047112311	-0.047282157	-0.09172	0.003737	-0.00551	0.025603
AK055458	-0.032986334	-0.013670251	0.144709	0.017467	-0.05306	-0.16674
AK055981	-0.00094897	0.071089638	0.177245	-0.00869	0.039135	-0.15933
AK056098	-0.039586207	-0.105000563	0.02634	0.00257	0.006391	-0.06801
AK056982	-0.015296221	-0.053638232	0.006821	-0.00156	-0.01637	-0.19301
AK074476	-0.115263115	-0.054564609	0.153213	-0.00657	0.016084	-0.06567
AK090844	0.010484571	-0.018765056	-0.03797	-0.03663	0.034415	-0.22427
AK091729	-0.019821165	-0.019432943	-0.03995	0.013146	-0.02875	-0.12406
AK093205	-0.068204274	-0.099367364	0.223032	0.003313	0.018319	-0.14677
AK094644	-0.027121548	-0.016039323	0.017262	0.003247	-0.00738	-0.15621
AK096159	-0.022934484	-0.014041623	0.044566	-0.02119	0.054469	-0.10245
AK097370	-0.025652394	0.069994769	-0.22858	-0.02379	0.081878	-0.00809
AK097453	-0.020281676	-0.036660053	-0.11996	-0.01143	0.005641	-0.18585
AK098263	-0.094131555	-0.047148181	0.012308	0.00494	-0.00347	0.015298
AK1	0.009708271	0.128188914	0.341359	0.010121	-0.03076	-0.35009
AK130486	-0.032790557	-0.020672843	0.111137	0.011475	-0.03463	-0.11292
AK131021	-0.027860883	-0.03680087	-0.09396	-0.01937	0.055383	-0.0401
AK2	0.032842491	0.045561169	-0.05135	0.040263	-0.19315	0.167638
AK3	0.020557197	0.087272588	0.313819	0.048912	-0.1197	-0.33533
AK5	-0.002322162	0.12686924	0.241024	-0.06973	0.219346	-0.21135
AK6	0.025891438	0.060654077	0.144623	0.062404	-0.17535	-0.01168
AK7	-0.064313977	0.02870458	0.010316	-0.00763	-0.03249	-0.05405
AK8	-0.020615665	-0.042682388	-0.00569	0.00473	-0.09957	-0.07829
AK9	-0.035547222	0.091616291	-0.01943	0.073911	0.003401	-0.15143
AKAP1	0.025300815	-0.091101295	0.287429	0.02635	-0.06914	-0.08953
AKAP10	0.022163879	-0.02786026	-0.15068	-0.08888	0.127744	0.055543
AKAP11	0.017348287	0.015046347	0.158836	-0.00442	0.001532	-0.17506
AKAP12	0.071021233	-0.044897373	-0.0044	0.015999	0.059128	-0.02183
AKAP13	0.016708205	0.142854074	-0.05652	-0.05287	0.172716	0.061542
AKAP14	-0.076950414	-0.049126971	-0.02245	-0.03395	0.114607	0.0849
AKAP17A	0.012528238	-0.012646177	0.079699	-0.06419	0.143295	-0.17544
AKAP3	-0.0340176	-0.077151838	0.086732	0.004807	-0.00582	-0.06792
AKAP4	-0.040669532	-0.033541459	0.056654	0.009582	0.001827	-0.02886
AKAP5	-0.010945252	0.327267811	-0.13068	0.009772	-0.01981	-0.07133
AKAP6	-0.046399392	-0.06972235	0.04149	0.057061	-0.1397	-0.07225
AKAP7	0.005912068	0.049676419	0.235668	-0.07085	0.16734	-0.06372
AKAP8	0.053053702	-0.004581746	0.126089	-0.00724	-0.0269	-0.10868
AKAP8L	-0.052690633	-0.07402159	0.051173	0.01742	0.019748	0.053155
AKAP9	-0.003101297	-0.045522622	0.042478	-0.03485	0.131464	-0.04773

AKIP1	0.020089546	-0.05742436	0.054565	0.015399	-0.0729	0.201759
AKIRIN1	0.051217859	0.007825478	0.088745	0.050985	0.001448	-0.17794
AKIRIN2	0.036506281	-0.069694741	-0.38645	0.070479	-0.17033	0.222308
AKNA	0.033612124	0.120454569	0.088685	-0.01757	0.070172	-0.16376
AKNAD1	-0.078106704	-0.106998502	-0.09112	0.092657	0.060033	0.001027
AKR1A1	0.034990116	-0.018891446	-0.05324	0.031542	-0.07861	0.357134
AKR1B1	0.037169953	0.104368755	0.2645	0.052993	-0.14283	-0.01861
AKR1B10	-0.037137683	-0.101608696	0.100754	-0.02817	0.109411	-0.11366
AKR1C1	-0.05688505	-0.046186036	0.003746	-0.03856	-0.0262	-0.06989
AKR1C3	0.007043968	0.304717931	-0.01682	-0.03238	0.088005	-0.19896
AKR1C4	-0.044404913	0.034641355	-0.08874	0.008594	-0.00487	-0.02264
AKR1C6P	-0.037815846	-0.02292056	-0.09852	-0.01378	0.041101	0.141546
AKR1D1	-0.060433195	-0.027925228	0.119068	-0.00554	-0.00038	-0.05845
AKR1E2	-0.024325413	-0.074533689	-0.09921	0.005647	-0.00526	0.085936
AKR7A2	0.025744952	0.044994488	-0.0199	0.043942	-0.10292	0.131582
AKR7A3	0.00781779	-0.01761336	0.01996	0.072086	-0.20578	0.129035
AKT1	0.033534752	0.075262219	-0.16874	0.054299	-0.13325	0.060938
AKT1S1	-0.021134129	-0.08894694	0.048267	0.050177	-0.11689	0.341068
AKT2	-0.010554945	-0.016716226	0.226622	-0.00144	-0.00841	-0.28064
AKT3	-0.051049083	0.232471618	0.178789	-0.03785	0.188387	-0.19046
AKTIP	0.006701885	0.031573195	0.301072	-0.00897	0.000375	-0.2519
AL022341.	-0.023448723	-0.116252398	-0.03853	0.020215	-0.0696	0.084618
AL022344.	-0.019653638	-0.042607672	0.024496	-0.01429	-0.00196	-0.04881
AL109706	-0.034970241	0.019972507	-0.12961	0.014031	-0.01907	-0.06143
AL132709.	-0.044346188	-0.00239192	-0.06209	0.004734	0.001154	0.09122
AL133493.	-0.020267539	-0.05667945	-0.00932	0.113212	-0.19859	-0.02352
AL590762.	-0.017023727	-0.102487168	-0.04887	0.012131	-0.05798	0.169236
AL832163	-0.088138418	-0.019095813	-0.06225	-0.03947	0.086537	0.015149
AL833181	-0.005938776	-0.138721108	-0.22717	-0.03818	0.105831	-0.04077
AL928742.	-0.028272801	-0.057200851	0.0077	0.02731	-0.10768	-0.04842
ALAD	0.011978803	-0.018021609	0.012416	-0.01563	0.056596	0.185593
ALAS1	0.034919892	0.050347982	-0.25848	0.082701	-0.23537	0.199503
ALAS2	-0.033036079	-0.039816382	-0.02685	-0.01232	0.038304	-0.04531
ALB	-0.097918848	-0.048304908	0.026585	-0.01977	0.012814	-0.01952
ALCAM	0.00650037	-0.19429939	-0.27718	-0.0093	0.002298	0.038391
ALDH16A1	0.018608517	-0.064203129	-0.01489	0.028399	-0.09655	0.083506
ALDH18A1	0.009455015	0.103634955	0.298452	0.056863	-0.20278	-0.44123
ALDH1A1	0.000508418	-0.073135073	-0.24238	-0.1118	0.325931	0.776535
ALDH1A2	-0.032955075	-0.088576928	0.079605	-0.00425	0.005519	0.067577
ALDH1A3	-0.049093437	0.04471461	0.013838	0.004725	0.007899	0.036314
ALDH1B1	-0.021705678	-0.04845402	-0.04434	0.115131	-0.38683	-0.07039
ALDH1L1	-0.029009737	-0.047910791	-0.03934	0.02956	-0.14599	-0.13139
ALDH1L1-/	-0.033893157	-0.080986328	-0.17174	-0.00111	-0.00756	0.086309
ALDH1L2	-0.049936218	-0.093187226	-0.1657	-0.02757	0.007557	0.042604
ALDH2	0.016352686	-0.178601329	-0.38991	-0.09095	0.250151	0.535853
ALDH3A1	-0.027403212	-0.028005394	-0.10345	0.008763	-0.00045	0.02591
ALDH3A2	0.014439559	0.011919831	0.030711	-0.11326	0.241849	0.256496
ALDH3B1	-0.053723339	-0.153309172	-0.23672	-0.0758	0.165506	0.448246
ALDH3B2	-0.060432582	-0.049146099	-0.08791	-0.01198	0.06883	-0.07688
ALDH4A1	-0.019073204	-0.071184713	-0.1157	0.027066	-0.15494	0.068287
ALDH5A1	4.22E-07	0.036996385	0.26767	-0.02903	0.096045	-0.47812

ALDH6A1	0.014724754	0.000269224	0.01103	0.030967	-0.03692	-0.00597
ALDH7A1	-0.012194291	-0.094789169	-0.06299	-0.00288	0.082358	-0.18273
ALDH8A1	-0.011868291	0.051239898	0.127472	0.053306	-0.15258	0.014431
ALDH9A1	0.09078308	0.150887088	-0.01111	0.06515	-0.15345	-0.15257
ALDOA	0.056934735	-0.011555168	-0.18727	0.118158	-0.27511	0.330225
ALDOAP2	-0.030916575	-0.030933903	0.050705	-0.00059	0.021673	-0.0381
ALDOB	-0.004631645	-0.110650381	-0.13384	0.00396	0.103126	0.17715
ALDOC	0.002645652	0.026272459	0.203052	0.032344	-0.12529	-0.2745
ALG1	-0.001616696	0.08236727	0.017563	0.043505	-0.10269	0.173224
ALG10	-0.021475476	-0.001248273	-0.01623	0.09703	-0.2815	-0.05299
ALG12	-0.001414999	-0.036972683	-0.06713	-0.00709	0.021661	0.077079
ALG13	-0.034603033	-0.029389382	0.127368	0.011946	-0.07487	0.130245
ALG14	-0.000273836	-0.019107764	0.026883	0.023103	-0.02501	0.185282
ALG2	0.021053646	0.044686901	-0.0695	0.021187	-0.08963	-0.0123
ALG3	0.023250424	-0.061624657	0.006237	0.063864	-0.21412	0.196502
ALG5	-0.006109914	0.018266884	0.028731	0.050804	-0.12961	0.081941
ALG6	0.024753077	0.072756795	0.034421	0.043267	-0.13813	-0.02631
ALG8	0.024573963	0.001152894	0.095003	0.069102	-0.18375	0.104067
ALG9	0.00571824	0.041776137	0.178968	0.092646	-0.1955	-0.18872
ALK	-0.037285478	-0.086720999	-0.03274	-0.01461	0.017576	0.035526
ALKBH1	0.004078368	0.028429095	0.027072	0.060149	-0.20898	0.005038
ALKBH2	-0.005435352	-0.042734239	0.145364	0.075852	-0.1946	-0.0385
ALKBH3	0.019276406	0.077638144	0.109535	-0.02635	0.094797	0.071195
ALKBH3-AS	-0.047913576	-0.051622121	-0.04767	0.002229	0.015507	0.055945
ALKBH4	0.003370286	0.024954096	0.08471	0.022928	-0.02264	0.080954
ALKBH5	0.029295112	0.022223397	0.160382	0.016498	-0.08641	-0.18719
ALKBH6	0.013264262	0.066634892	0.059153	0.036074	-0.07879	-0.01323
ALKBH7	0.014462524	-0.037678455	0.22716	0.052819	-0.0474	0.165338
ALKBH8	0.000582764	0.111270294	0.117982	0.015957	-0.058	-0.09999
ALLC	-0.036962938	-0.123674558	0.036633	0.019005	-0.03315	0.07246
ALMS1	0.010654579	0.030820275	0.085455	0.009658	6.31E-05	-0.07776
ALMS1-IT	-0.016245502	0.015536872	-0.04578	0.067592	-0.19018	0.050973
ALMS1P	-0.034405441	-0.0284174	0.062419	0.018256	-0.0389	-0.21365
ALOX12	-0.011769642	-0.003218463	-0.08821	-0.2195	0.622982	0.168763
ALOX12B	-0.030412944	-0.032044941	0.101346	0.110396	-0.29146	0.000134
ALOX12P2	-0.025865121	-0.015169944	-0.04112	-0.00598	-0.02927	-0.17365
ALOX15	-0.017383773	-0.052124533	-0.11349	-0.01037	0.012787	-0.17733
ALOX15B	-0.012835543	-0.025118751	-0.05053	-0.01477	0.050401	0.1855
ALOX5	-0.004826423	-0.169826376	-0.33773	-0.11139	0.298941	0.351622
ALOX5AP	0.024012118	0.074752984	-0.09761	0.069731	-0.17433	-0.26598
ALOXE3	-0.05091398	-0.125597637	0.059999	0.007194	-0.05063	-0.00754
ALPI	-0.035272788	-0.030380939	-0.14948	0.011554	-0.00523	0.027701
ALPK1	0.030394374	-0.117755268	-0.23188	-0.01383	-0.05738	0.15021
ALPK2	-0.021221528	-0.036315966	0.044743	0.006107	-0.05641	-0.03292
ALPK3	-0.045091353	-0.00242864	-0.03997	0.014959	-0.01304	-0.03016
ALPL	-0.015847233	-0.035530871	-0.19533	0.002794	-0.00336	-0.03723
ALPP	-0.03182972	-0.004402231	0.008328	0.010106	-0.00232	0.100129
ALPPL2	-0.028726924	-0.123761197	0.024427	-0.00523	-0.04355	0.001674
ALS2	-0.022705291	0.092850329	-0.00309	0.076015	-0.21592	0.009574
ALS2CL	-0.025460286	-0.151000052	0.230689	-0.0456	0.07363	-0.04235
ALS2CR11	-0.08726445	-0.022571435	0.097604	-0.02863	0.0615	-0.12337

ALS2CR12	-0.033420922	0.054329768	0.200638	-0.05322	0.183965	-0.12002
ALX1	-0.024752193	0.006396944	-0.00067	-0.00096	-0.02738	-0.15696
ALX3	-0.02975366	-0.090231789	-0.04436	0.02339	-0.06099	-0.0936
ALX4	-0.031174153	-0.041625567	-0.04412	-0.00162	0.00662	0.07177
ALYREF	0.037219806	0.067967133	-0.09135	0.116562	-0.32347	0.140183
AMACR	-0.039277148	-0.003906883	-0.01561	0.017834	-0.09409	0.015698
AMBN	-0.019070582	-0.021872709	-0.03181	0.04352	-0.11277	0.000781
AMBP	-0.05195622	-0.094495727	-0.02564	0.038847	-0.10266	-0.03038
AMBRA1	-0.024253341	0.035584086	0.11451	0.034034	-0.18782	-0.14943
AMD1	0.048440984	-0.019450011	-0.10657	0.02858	-0.05885	-0.06373
AMDHD1	-0.018669424	0.146622495	0.115561	0.025498	-0.04791	0.141592
AMELX	-0.045849308	-0.008342977	-0.08166	-0.00242	0.048025	-0.03493
AMELY	-0.04047069	-0.056476008	-0.02316	-0.00868	0.054993	0.0436
AMER1	-0.027778405	-0.068495529	-0.01604	0.05714	-0.13727	0.111322
AMER2	-0.093222353	-0.009413786	0.050081	-0.05732	-0.03721	0.001253
AMER3	-0.041079569	-0.041200801	0.024736	0.045863	-0.08022	-0.06826
AMFR	0.012824759	-0.074341813	-0.13051	-0.00344	0.009715	0.13881
AMHR2	-0.028448064	-0.093197561	0.028569	0.013865	-0.09512	0.019339
AMICA1	0.017384339	-0.187359287	-0.13179	-0.0659	0.181714	0.200391
AMIGO1	-0.003586126	0.020510756	0.528746	-0.05299	0.160471	-0.24771
AMIGO2	0.002172192	0.022537548	0.293199	-0.07186	0.192722	-0.31419
AMMECR1	-0.00019971	0.09163523	0.181926	0.02667	-0.05553	-0.12675
AMMECR1L	0.029290793	0.078723189	-0.01789	0.01097	-0.06597	-0.1637
AMN	-0.018331118	-0.145732792	0.222852	-0.05257	0.11136	-0.19931
AMN1	-0.00157743	-0.001195675	0.073413	-0.05637	0.164688	-0.36521
AMOT	-0.038759754	0.036623464	0.026548	-0.02002	0.080788	-0.11892
AMOTL1	-0.003563921	0.030087655	-0.07749	-0.00749	0.000384	0.041318
AMOTL2	-0.033471351	-0.075690235	0.021976	-0.00892	0.048402	0.018529
AMPD1	-0.046107356	-0.002216601	-0.04046	0.011641	-0.00024	0.019952
AMPD2	0.029605019	-0.079479211	-0.23791	-0.02591	0.083136	0.226066
AMPD3	0.012203132	-0.142982113	-0.12142	-0.05869	0.143635	0.045644
AMPH	-0.032073992	-0.053976279	-0.00598	-0.00258	0.006632	0.086719
AMT	0.003064061	-0.015247514	0.118298	-0.06586	0.173056	-0.12684
AMZ1	-0.024332546	-0.074503088	-0.02718	0.014817	-0.13416	-0.04667
AMZ2	0.065980434	0.206695346	0.23643	0.100588	-0.27157	-0.38534
AMZ2P1	0.022301983	0.133548259	0.091546	0.076862	-0.22098	0.011779
ANAPC10	0.007340382	0.079208907	0.107672	0.053878	-0.10453	0.111958
ANAPC11	0.046165603	0.068941734	-0.0671	0.100054	-0.27551	0.317773
ANAPC13	0.049164177	-0.032309909	-0.01735	0.025587	-0.04364	0.046587
ANAPC15	0.032836627	-0.126822575	0.055874	0.002225	-0.0557	0.252239
ANAPC16	0.067511062	0.052995737	0.289591	-0.02583	-0.034	-0.316
ANAPC2	0.01112153	-0.032407492	0.03391	0.041863	-0.11499	0.106259
ANAPC4	0.052972172	0.059659453	0.140453	0.005606	-0.01257	-0.21
ANAPC5	0.030547926	0.063304055	0.176694	0.010196	-0.00166	-0.04225
ANAPC7	0.044204913	0.077649749	0.063104	0.057332	-0.15306	-0.26319
ANG	-0.009495777	-0.095854601	-0.22948	-0.05002	0.153848	0.700768
ANGEL1	0.015823972	-0.066943993	0.258965	0.026252	-0.11642	-0.13383
ANGEL2	0.008401123	0.037387074	-0.06242	0.088436	-0.19992	-0.10333
ANGPT1	-0.020824958	-0.0632194	-0.14365	0.017095	0.031398	0.172844
ANGPT2	-0.025156657	-0.113459214	-0.08264	0.008321	0.107135	-0.07593
ANGPT4	-0.025843014	0.026129825	0.083151	-0.01497	0.002037	-0.09595

ANGPTL1	-0.037669261	-0.055427448	-0.00536	-0.05724	-0.01478	-0.04329
ANGPTL2	-0.00420994	-0.059333998	-0.06117	0.018439	-0.08212	0.098296
ANGPTL3	-0.089794453	-0.11504052	0.051491	-0.00114	0.071135	-0.02152
ANGPTL4	-0.02196056	-0.072648049	0.003962	0.004822	-0.05154	-0.03901
ANGPTL6	-0.03394092	0.067922665	0.012952	0.009507	-0.05675	-0.07313
ANGPTL7	-0.035580363	-0.035982011	0.016264	0.031079	-0.07392	-0.09223
ANHX	-0.010291777	-0.045031137	-0.04186	-0.02433	-0.00201	-0.12626
ANK1	-0.006752957	-0.087466048	0.102653	-0.01359	0.095206	-0.1579
ANK2	-0.042530657	0.00387249	-0.10573	0.077055	-0.18347	0.014938
ANK3	-0.028484	-0.066461739	0.14001	0.10197	-0.04841	-0.22539
ANKAR	-0.06191016	0.016438098	-0.00506	-0.01021	0.046491	-0.05078
ANKDD1A	-0.00592406	0.03020125	0.052318	0.010659	-0.01692	0.033342
ANKEF1	-0.011874235	0.075497063	0.178035	-0.02582	0.100618	-0.2819
ANKFN1	-0.02549302	-0.050598946	-0.00903	0.074377	-0.21838	-0.10442
ANKFY1	-0.012571248	0.002287525	-0.05024	-0.09908	0.210677	0.111237
ANKH	-0.0361718	0.030892328	0.194021	-0.03381	0.046981	-0.17232
ANKHD1	-0.011668167	-0.024059639	-0.00635	-0.00026	0.043907	-0.04281
ANKIB1	0.026129992	0.118040032	0.128859	0.031569	-0.02519	-0.06996
ANKLE1	-0.018597214	-0.092253682	-0.07212	-0.01098	0.044644	0.145715
ANKLE2	0.006294599	-0.008247733	0.001507	0.038124	-0.07477	-0.05751
ANKMY1	-0.069670855	-0.127773189	-0.0118	0.010584	0.014096	-0.00614
ANKMY2	0.036412268	0.136396446	0.116203	-0.03246	0.067218	-0.35326
ANKRA2	0.027989391	0.053275164	0.1651	0.008011	-0.03523	-0.18461
ANKRD1	-0.049323051	-0.055489129	-0.06876	0.003118	0.015342	0.03358
ANKRD10	0.051770731	0.02560388	-0.01832	-0.00686	-0.0489	-0.19799
ANKRD10-I	0.042953486	0.01442319	-0.03161	0.012803	-0.02486	-0.18746
ANKRD11	0.058480654	0.02910409	0.124927	-0.06557	-0.14671	-0.31756
ANKRD12	0.053760622	0.04846792	0.109689	-0.0354	0.060117	-0.50893
ANKRD13A	0.02550105	-0.027849655	-0.20245	-0.00589	0.013636	-0.09508
ANKRD13B	-0.014847119	-0.084489944	-0.02743	0.045466	-0.1746	-0.0475
ANKRD13C	-0.029639206	0.085916124	0.077137	0.023538	-0.04557	-0.10818
ANKRD13D	0.017272273	-0.078518527	-0.08933	-0.04072	0.094778	0.190963
ANKRD16	-0.018791613	-0.047662045	0.126232	0.014989	-0.03438	-0.09109
ANKRD17	0.058342944	0.167962226	0.158234	0.043255	-0.11899	-0.08256
ANKRD18A	-0.029625858	-0.063500512	0.077597	0.053793	-0.16971	-0.10626
ANKRD18B	-0.027959457	-0.003710243	-0.05268	0.090416	-0.24715	0.00575
ANKRD19P	-0.006184464	-0.103018007	-0.06518	0.00531	-0.04641	-0.05622
ANKRD2	-0.024812091	-0.052586051	-0.00809	0.010065	-0.00924	0.134847
ANKRD20A1	0.010963413	0.160691913	0.064813	-0.00684	-0.01847	-0.35154
ANKRD20A1	-0.022537311	-0.046866349	0.093512	0.016833	-0.07148	-0.11106
ANKRD22	-0.008290981	-0.066796507	-0.15822	-0.01165	0.00865	0.404559
ANKRD23	-0.003626527	0.020415052	0.136786	-0.00778	0.051686	-0.16854
ANKRD24	-0.004731206	-0.114856021	-0.05054	0.083656	-0.02568	0.068822
ANKRD26	-0.019829054	-0.012131953	0.102808	0.076254	-0.20102	-0.07557
ANKRD26P3	-0.02875808	-0.050752218	-0.00211	-0.00297	0.01016	-0.072
ANKRD27	0.055393789	0.127437513	0.029552	0.078212	-0.23302	-0.11511
ANKRD28	-0.004066834	-0.004022578	0.144527	-0.00844	0.070515	-0.29635
ANKRD29	-0.098159164	-0.046272127	-0.11386	-0.06638	0.170717	0.115489
ANKRD30A	-0.052754533	-0.055572956	-0.16668	0.000798	0.008162	0.137204
ANKRD30B	-0.057332881	-0.014093275	-0.13043	-0.01668	0.035032	0.013195
ANKRD30BI	-0.050609745	0.031911919	0.044866	0.013468	-0.00217	0.166515

ANKRD30BI	0.014661698	-0.038264889	0.018129	0.097422	-0.06916	-0.14244
ANKRD31	-0.025159466	-0.087365061	0.08011	0.026497	-0.05124	0.022522
ANKRD32	-0.005717024	0.040950997	0.069427	0.079139	-0.20644	-0.12287
ANKRD33	-0.030441015	-0.04405354	-0.11181	0.013156	-0.03115	0.079967
ANKRD33B	-0.005386135	-0.03231664	-0.03172	-0.00968	-0.03172	-0.13826
ANKRD34A	-0.022879217	-0.050470552	0.174868	0.051478	-0.13615	-0.1688
ANKRD34C	-0.030354556	0.006327037	-0.07354	0.018466	-0.03618	-0.09204
ANKRD35	-0.013790676	-0.03809901	0.080134	0.007437	-0.01624	0.287251
ANKRD36	-0.007489451	0.093817537	0.363354	0.010766	-0.01253	-0.33048
ANKRD36B	0.011245192	0.072528377	0.384	-0.00025	0.002703	-0.41556
ANKRD36BI	-0.065600284	-0.125621173	0.119279	-0.07499	0.205257	-0.11673
ANKRD36C	-0.01788	-0.018598411	0.053485	0.033783	-0.08643	-0.31411
ANKRD37	-0.006737939	-0.016330452	-0.00442	0.052413	-0.15859	-0.01584
ANKRD39	0.009471704	0.080826051	0.181216	0.062064	-0.14274	-0.05979
ANKRD40	0.014007641	0.040326647	-0.0316	0.070785	-0.24605	0.077654
ANKRD44	-0.035339615	-0.013700367	-0.0185	0.011366	0.051953	-0.13996
ANKRD44-I	-0.018972656	-0.025707743	-0.14743	-0.03226	0.107645	-0.15117
ANKRD45	-0.055170155	-0.025325251	-0.02092	-0.02652	0.087683	-0.0523
ANKRD46	0.015914727	0.127167644	0.113165	0.010873	-0.02479	-0.18882
ANKRD49	0.05427726	0.080307044	0.158997	-0.03117	0.081361	-0.35654
ANKRD50	0.038550688	0.003342593	-0.32325	-0.00892	0.160374	0.348256
ANKRD52	0.019924974	-0.014919543	0.081035	0.006043	-0.12351	-0.24682
ANKRD53	-0.017312666	-0.100456813	-0.02063	-0.01409	-0.00595	-0.13089
ANKRD54	-0.003365324	-0.017763179	0.101511	0.069133	-0.22777	-0.05097
ANKRD55	-0.010352327	-0.08866727	-0.12239	-0.07936	0.206972	-0.20317
ANKRD6	-0.020166593	0.033804919	-0.00713	-0.06952	0.175874	0.035214
ANKRD60	-0.034428921	-0.070472687	-0.03253	0.004729	0.005034	0.009684
ANKRD65	-0.037718978	-0.047675216	-0.12264	-0.01329	0.0058	-0.11575
ANKRD7	-0.033159455	-0.087516003	0.110393	0.009726	-0.04464	-0.08383
ANKRD9	0.03884607	-0.031252965	-0.10455	-0.1405	0.005841	0.106785
ANKS1A	0.023032397	-0.051217262	-0.23319	0.005037	-0.00307	0.227505
ANKS1B	-0.002492366	-0.004259782	-0.24867	0.077915	-0.01072	0.081282
ANKS3	-0.00593563	-0.05809083	0.123992	0.006273	-0.01398	-0.02205
ANKS4B	-0.023931597	0.015276592	-0.07038	0.020819	-0.03479	0.029337
ANKS6	-0.047282216	-0.088180742	0.243843	0.070241	0.042448	-0.16679
ANKUB1	-0.042111503	0.038193189	0.067391	0.003106	-0.00743	-0.06032
ANKZF1	0.018110279	-0.04887964	0.235914	-0.02202	0.033293	-0.23849
ANLN	-0.016154377	0.000181071	0.018056	0.132511	-0.25473	-0.01333
ANO1	-0.056641238	-0.082921081	-0.11048	-0.00645	-0.00664	-0.05894
ANO10	0.008833273	-0.069646409	-0.16487	0.012035	-0.05716	0.413171
ANO2	-0.030111916	-0.029500298	-0.04291	0.078249	-0.20742	0.020916
ANO3	-0.027219982	-0.067726736	-0.01183	0.170881	-0.19544	-0.05017
ANO4	-0.040559186	-0.118986628	0.025065	0.012722	0.036561	0.040774
ANO5	-0.022452566	-0.042920388	-0.12358	-0.02135	0.063134	0.252929
ANO6	0.022619642	0.031254346	-0.14564	-0.05816	0.164786	0.545392
ANO7	-0.015125383	-0.065143413	0.011711	-0.01953	-0.0549	-0.03735
ANO8	0.001422159	-0.041949324	0.02464	0.003817	0.006463	0.049516
ANO9	-0.002307554	-0.125916313	0.357317	-0.05482	0.155051	-0.3117
ANP32A	0.117836687	-0.02942656	-0.2988	0.049218	-0.13094	0.103814
ANP32A-I'	-0.00885125	-0.084783142	-0.24428	-0.0182	0.064265	0.183427
ANP32B	0.070982966	0.031255509	0.214987	0.076117	-0.1464	-0.02086

ANP32C	-0.048462294	-0.049798806	0.031981	0.051983	-0.12257	-0.01757
ANP32D	-0.047317228	0.011337348	-0.07162	-0.01454	0.053474	0.055589
ANP32E	0.059891818	0.094318006	0.196699	0.081973	-0.17224	-0.03221
ANPEP	-0.035176827	-0.006955113	-0.1298	0.003826	0.008389	-0.00185
ANTXR1	-0.02805108	-0.022506923	-0.09629	0.029756	-0.05387	-0.02364
ANTXR2	0.011735855	-0.176245939	-0.27128	0.025409	-0.12508	0.035984
ANXA1	0.014634121	0.020876536	0.064498	-0.02491	0.086399	0.414278
ANXA10	-0.02682341	-0.010820498	0.027964	0.067526	-0.18687	-0.1302
ANXA11	0.032935464	-0.004342189	0.01691	-0.04978	0.117104	-0.22297
ANXA13	-0.032502694	-0.038917143	-0.08816	0.015037	-0.02742	-0.04799
ANXA2	0.033838082	0.04348524	-0.09178	0.020538	-0.03263	0.452222
ANXA2P1	-0.017924725	0.002783883	-0.18702	0.008623	-0.00904	0.290994
ANXA2P2	0.049481551	0.016799973	-0.14619	0.003603	-0.0241	0.434161
ANXA2P3	-0.006797094	-0.030949	-0.2428	0.009001	-0.01221	0.36052
ANXA2R	0.014674646	0.053891344	0.392089	-0.03611	0.093441	-0.30374
ANXA3	-0.002028397	-0.055483664	0.048311	-0.09131	0.27178	-0.13476
ANXA4	0.031709077	0.107263942	-0.2417	-0.00615	0.005608	0.279252
ANXA5	0.037454829	-0.224391857	-0.17513	0.023256	-0.06831	0.501194
ANXA6	0.01456438	0.135378955	0.209104	0.017769	-0.04478	-0.11698
ANXA7	0.100075798	-0.004923379	-0.05964	0.063974	-0.15418	0.07057
ANXA9	-0.032750056	-0.115311177	-0.14979	0.050016	-0.1222	0.153189
AOAH	0.016526616	0.114460407	-0.18473	-0.03943	0.111603	0.40374
AOC1	-0.02206268	-0.066543302	-0.13482	0.025548	-0.07119	-0.02635
AOC2	-0.017671781	-0.016298091	-0.13426	0.004686	-0.02732	-0.12916
AOC3	-0.019022304	-0.074351294	-0.04418	-0.00757	0.004871	-0.08145
AOC4P	-0.030999298	-0.091660658	0.006205	-0.01563	-0.05024	-0.07585
AOX1	-0.041639703	-0.011726969	-0.0592	0.021808	-0.03437	-0.04011
AOX2P	-0.029808608	-0.032375271	-0.0468	-0.00677	0.024737	-0.06807
AP000230.	-0.02720137	-0.057786268	-0.01803	0.022615	-0.09376	-0.01914
AP000253.	-0.043474855	-0.011557745	0.073923	0.040164	-0.11211	0.043085
AP000265.	-0.04510207	-0.040899448	0.039874	-0.01451	0.040608	-0.00134
AP000347.	-0.000957649	0.007021855	0.022568	0.012803	-0.0176	-0.12606
AP000462.	-0.041137084	-0.029785058	-0.15483	0.002332	0.013806	0.066559
AP000473.	-0.023884104	-0.025135393	0.044001	-0.00992	-0.00662	-0.05533
AP000525.	-0.018609207	-0.03439926	0.069832	-0.01257	-0.04091	-0.13717
AP000696.	-0.03324997	-0.043075428	-0.04256	0.00946	-0.05506	0.026552
AP001063.	-0.037296096	-0.111053535	-0.07211	-0.00155	-0.00102	0.078645
AP001171.	-0.028111112	-0.040189034	0.055442	0.027012	-0.0817	-0.08519
AP001189.	-0.019505354	-0.031767287	-0.03839	-0.25415	0.720105	0.025673
AP001462.	-0.003959015	0.063699363	0.160351	-0.00173	0.051894	-0.08663
AP001605.	-0.027729346	-0.040441313	0.028973	0.000269	-0.047	-0.07467
AP001630.	-0.034377547	-0.115553661	-0.1283	0.028508	-0.0881	-0.00196
AP006216.	-0.034917358	-0.065435989	0.144602	-0.00575	0.037198	0.007608
AP006222.	-0.013986412	-0.097958559	-0.18869	0.004918	0.006055	0.147915
AP006547.	-0.021133552	-0.093086689	-0.00201	-0.01896	-0.08083	-0.11999
AP1AR	-0.054442105	-0.007558727	0.022999	-0.02922	-0.01245	0.020624
AP1B1	0.026515558	-0.09374915	-0.23448	-0.00132	-0.05495	0.322781
AP1G1	0.036289311	0.093106305	-0.14439	0.039137	-0.13995	-0.11967
AP1G2	0.021277329	-0.048973932	0.111021	0.002376	-0.01933	-0.31602
AP1M1	0.034851321	-0.029043857	0.089641	0.000231	-0.03296	0.158326
AP1M2	-0.034391468	-0.063558403	0.018738	-0.0422	0.065701	-0.09314

AP1S1	-0.020809601	-0.064077632	-0.01765	0.137569	-0.4179	0.185539
AP1S2	0.006633772	-0.118450138	-0.36239	-0.04934	0.170063	0.630211
AP1S3	-0.033841232	0.019624679	-0.15469	0.132686	-0.18404	-0.10957
AP2A1	0.033966108	-0.058986789	-0.02297	-0.00069	-0.0107	0.118458
AP2A2	0.057349233	-0.007800979	0.035373	0.006237	0.00177	0.175927
AP2B1	0.038758919	0.095791572	-0.01994	0.051572	-0.1723	0.007189
AP2M1	0.053699048	0.045634659	-0.09584	0.034077	-0.0717	0.230895
AP2S1	0.026937483	-0.037991874	-0.14472	0.026719	-0.08934	0.518717
AP3B1	0.039160309	-0.006082044	-0.34087	0.032713	-0.08888	-0.01789
AP3B2	-0.053780739	0.071756445	-0.00485	-0.0684	0.235368	-0.07832
AP3D1	0.061780036	0.093123382	-0.06421	0.034183	-0.08	0.090919
AP3M1	0.040560719	0.162340635	-0.05988	0.017358	-0.03715	0.086799
AP3M2	0.015205848	-0.021882047	0.114235	0.042238	-0.13403	-0.39665
AP3S1	0.119198679	0.012098005	-0.18599	0.104122	-0.21004	-0.00695
AP4B1	-0.063024187	-0.02690778	0.006208	-0.10528	0.203147	-0.02945
AP4E1	-0.055471429	-0.014796275	0.046056	0.013402	-0.02271	0.104894
AP4M1	-0.010415326	-0.051026762	-0.00247	0.023128	-0.06435	0.213294
AP4S1	-0.015415827	-0.013497534	0.156171	0.030401	-0.10484	-0.11557
AP5B1	-0.005031697	-0.099152389	-0.2391	-0.02304	0.053311	0.366586
AP5M1	0.006958869	-0.026375137	0.148065	-0.02087	0.081612	-0.173
AP5S1	0.029736346	0.034734448	0.061545	0.047782	-0.15749	0.183255
APAF1	0.014534152	0.010013152	-0.26882	-0.00559	0.035269	0.254174
APBA1	-0.019023076	-0.055595326	-0.086	0.176962	-0.38067	-0.0246
APBA2	0.002726807	0.084141145	0.557859	-0.09559	0.258528	-0.29006
APBA3	0.025461968	-0.037150703	0.149581	0.050442	-0.20719	-0.14996
APBB1	-0.004512673	-0.030047813	0.347576	0.019022	-0.02601	-0.15681
APBB1IP	-0.010064877	-0.006742185	-0.02235	-0.00618	0.32594	-0.09434
APBB2	-0.039576997	-0.040990005	-0.00874	0.234404	-0.07946	-0.02826
APC	-0.002465749	0.043131348	-0.09641	-0.03226	0.088711	0.003729
APC2	-0.03824081	-0.142867856	0.047681	0.031648	-0.09728	0.058004
APCDD1	-0.007486317	-0.093362044	-0.17978	-0.04285	0.140175	0.230835
APCDD1L	-0.035728582	-0.090504001	-0.00288	0.030816	-0.1084	0.015267
APCDD1L-/	-0.078429076	-0.119277982	0.070822	-0.09048	0.015878	-0.0385
APCS	-0.036266559	-0.125618985	0.045373	-0.01412	0.031523	0.026228
APEH	0.016260111	0.015937867	0.07609	0.041697	-0.10012	0.154505
APELA	-0.069736309	-0.082897496	0.079274	-0.02719	0.09892	0.069988
APEX1	0.034570134	0.010231766	0.169519	0.020228	-0.04268	0.043996
APEX2	0.014133313	0.071557036	-0.10426	0.010759	-0.04734	0.155456
APH1A	0.013606369	-0.011751274	0.078855	0.055985	-0.21653	-0.0354
APH1B	0.008063877	-0.124668991	-0.12058	-0.07238	0.143508	0.336182
API5	0.012091244	0.006848449	0.02272	0.057821	-0.1068	-0.07803
APIP	0.019090359	0.006113705	-0.02626	0.041526	-0.16379	0.20066
APLF	-0.036689062	-0.058958119	-0.0581	0.057858	-0.15234	0.040251
APLN	-0.023994122	-0.078192286	0.026866	0.008937	-0.07059	-0.05699
APLNR	-0.025285134	-0.011868636	-0.11502	0.00083	-0.01321	-0.13195
APLP1	-0.024515266	-0.020006285	0.062218	0.123279	-0.36265	-0.02941
APLP2	0.03309635	-0.02015479	-0.47456	-0.06999	0.186186	0.548437
APMAP	0.054989038	0.333506487	0.064308	0.021707	-0.03213	-0.19498
APOA1	-0.03592224	-0.09008163	-0.08011	0.016944	-0.02051	0.001926
APOA1BP	0.03591806	0.090661912	0.131596	0.095859	-0.26905	-0.05917
APOA2	-0.051905378	-0.017134905	-0.14933	0.022139	-0.02915	0.012494

APOA4	-0.018928286	-0.14221239	0.056379	0.021361	-0.0584	-0.04098
APOA5	-0.025451927	-0.109835862	-0.03791	-0.00283	-0.09888	-0.00628
APOB	-0.049253693	-0.036253692	-0.01271	-0.00234	0.039083	0.09225
APOBEC1	-0.040488333	-0.078281921	-0.01552	0.002892	0.015985	-0.12795
APOBEC2	-0.025477183	-0.079328486	0.014805	0.007679	-0.04867	-0.08608
APOBEC3B	-0.003081499	0.016239671	-0.06455	0.047193	-0.07726	0.134517
APOBEC3C	0.030899632	0.15358448	0.072457	0.039637	-0.136	-0.05605
APOBEC3D	-0.033894992	-0.035203028	-0.04391	0.023843	-0.08319	-0.03724
APOBEC3F	-0.01695465	-0.064563302	-0.09515	-0.01779	-0.03927	-0.01027
APOBEC3G	0.029928316	0.231234111	0.181939	0.02797	-0.10798	-0.14284
APOBEC4	-0.023806516	-0.023168491	0.036476	-0.00118	########	-0.10538
APOBR	0.023991192	0.0704806	-0.29722	-0.06556	0.173502	0.271057
APOC1	0.003090522	-0.112394885	0.076319	-0.01328	-0.05518	-0.13305
APOC2	-0.040807093	-0.107352471	-0.0047	0.016066	-0.06252	-0.07046
APOC3	-0.039923841	-0.018879724	-0.09918	0.029124	-0.05641	-0.03787
APOD	-0.033389414	-0.05763231	-0.07008	0.037385	-0.11283	0.021643
APOE	-0.031382304	-0.007751565	-0.08497	0.02369	-0.14927	0.045124
APOF	-0.044440876	0.020830362	-0.02596	-0.00958	0.017397	-0.07414
APOH	-0.041970271	-0.018748774	-0.02594	0.015064	-0.0547	-0.09881
APOL1	0.016491718	0.13744864	-0.0774	0.038872	-0.14063	-0.02341
APOL2	0.031800555	0.025612102	-0.02766	0.023414	-0.11538	-0.06775
APOL3	0.016388205	0.009624352	0.162111	-0.00614	-0.00545	0.210623
APOL4	-0.101503276	-0.037735972	-0.14068	0.063094	-0.2313	0.122585
APOL5	-0.034676673	-0.043265014	-0.10137	0.013798	-0.04278	-0.12961
APOL6	-0.005897242	0.148089976	0.000774	0.040577	-0.12833	0.142114
APOLD1	-0.011365699	-0.001805117	0.015628	0.104978	-0.30141	-0.06836
APOM	-0.014554537	-0.146586915	0.008538	0.0966	-0.24997	0.2765
APOO	0.023873478	0.04378446	0.143285	0.113172	-0.30637	-0.03531
APOOL	0.011101996	-0.022725836	0.012663	-0.05436	0.186663	0.230664
APOPT1	0.065389308	0.025074477	0.063065	0.055396	-0.19384	-0.04993
APP	-0.002096578	-0.169007208	-0.15713	-0.05829	0.245243	0.253174
APPBP2	0.005150798	0.066732508	0.039476	0.004041	0.010716	0.007723
APPL1	0.014945803	0.04994407	-0.06668	0.015294	-0.01356	0.07379
APPL2	0.010502502	0.072280667	0.006577	0.015395	0.014484	-0.1712
APRT	0.041526048	-0.027462343	0.233423	0.057118	-0.16655	0.124015
APTR	-0.01157682	-0.020111871	-0.08995	0.051322	-0.12831	-0.03833
APTX	0.050036376	0.008601619	-0.0403	0.066391	-0.28741	0.161887
AQP1	-0.04519294	-0.029037963	0.049696	-0.00844	0.037473	-0.14991
AQP10	-0.026974981	0.011267994	-0.01289	-0.21054	0.614841	-0.00052
AQP11	-0.021626455	0.057428389	0.061017	0.008507	-0.04043	-0.11994
AQP2	-0.004440563	-0.08501415	-0.05147	-0.00432	-0.02879	-0.06711
AQP3	0.025084449	-0.070621725	0.35834	-0.11216	0.159068	-0.25749
AQP4	-0.103468056	-0.042538716	-0.04177	-0.00515	0.010592	0.073867
AQP4-AS1	-0.086612477	0.015136276	-0.05049	-0.00446	0.027125	0.028028
AQP5	-0.033212824	-0.060577796	-0.05984	-0.01978	0.012716	-0.07549
AQP6	-0.035121829	-0.064706048	0.053504	-0.00265	-0.03619	-0.04712
AQP8	-0.032722777	-0.053516562	-0.00117	0.034575	-0.10234	-0.02314
AQP9	0.004830585	-0.061937045	-0.27049	-0.06471	0.178505	0.360824
AQPEP	-0.037262974	-0.022076545	0.000811	0.010656	-0.02568	-0.11597
AQR	0.020020851	0.067169515	0.117123	-0.03501	0.14995	-0.06699
AR	0.004050254	-0.049584828	-0.07409	0.029458	0.061019	-0.20807

ARAF	0.021868977	0.049961718	-0.07117	0.025092	-0.04946	0.027514
ARAP1	0.023053086	-0.016773718	-0.31973	-0.06167	0.16058	0.291308
ARAP3	0.001796359	0.00284971	-0.19353	-0.04025	0.093819	0.20125
ARC	-0.013017009	-0.019271052	-0.03432	-0.00266	0.018254	0.045362
ARCN1	0.078035213	0.198724495	0.001129	0.063802	-0.16841	-0.20119
AREG	-0.03306442	0.141775934	-0.05866	-0.03257	0.001458	-0.13902
AREL1	-0.051021755	-0.061402976	-0.04329	0.194121	-0.02156	-0.06275
ARF1	0.038472286	-0.055490196	-0.08985	-0.02397	0.056997	-0.09872
ARF3	0.053867634	-0.047293166	-0.28995	0.015726	-0.06751	0.33125
ARF4	0.069297153	0.094826554	-0.21636	0.07987	-0.18822	0.181059
ARF5	0.05014013	-0.032377452	-0.25392	0.013269	-0.02115	0.274177
ARF6	0.12625141	0.213949363	-0.09496	0.102036	-0.18581	-0.19558
ARFGAP1	0.019994488	-0.055368571	0.045885	0.024323	-0.14741	-0.01611
ARFGAP2	0.038363722	0.003130507	-0.02161	0.006066	-0.03295	0.071801
ARFGAP3	0.06563626	0.18746809	-0.17283	0.013857	-0.02466	-0.09321
ARFGEF1	0.036715292	0.081180321	0.065296	-0.01856	-0.02501	-0.07236
ARFGEF2	-0.004514763	-0.002194096	0.094577	0.044076	-0.07412	0.017848
ARFIP1	0.011013302	0.069844351	-0.31693	-0.00334	0.005518	0.253748
ARFIP2	0.03541296	0.008554576	0.159009	0.065023	-0.19109	0.005189
ARFRP1	0.010461854	-0.044300156	0.200046	0.003633	-0.09793	-0.0543
ARG1	0.066982381	0.005634352	-0.18641	0.1491	0.042191	-0.25839
ARG2	-0.012556476	0.000694779	-0.07648	-0.00126	-0.01802	0.021135
ARGLU1	0.049529259	0.03609101	0.232594	-0.02959	-0.0185	-0.15042
ARHGAP1	-0.014886035	-0.062877318	0.064984	-0.08289	0.121187	-0.14828
ARHGAP10	0.017474572	0.002226725	0.218408	0.004845	-0.09309	-0.09568
ARHGAP11/	-0.020916002	-0.022233285	0.004209	0.147783	-0.28007	-0.05174
ARHGAP12	0.007955367	0.060821477	0.12605	-0.02775	0.132998	-0.07421
ARHGAP17	0.043402469	-0.062288994	-0.18239	-0.02156	0.045951	-0.10185
ARHGAP18	0.034979151	0.085535133	-0.17664	-0.0311	0.015191	0.131887
ARHGAP19	-0.010023563	-0.021974966	-0.16943	0.059849	-0.13473	0.080354
ARHGAP20	-0.029757253	-0.055587651	-0.09198	0.003934	-0.02668	-0.05268
ARHGAP21	0.004210068	0.020972989	-0.09175	-0.04861	0.14371	0.217615
ARHGAP22	-0.020252874	-0.08525929	-0.1046	-0.0313	0.084362	0.13438
ARHGAP22-	-0.046794589	0.024301153	-0.0475	0.007697	0.003438	0.026717
ARHGAP23	-0.031309872	-0.083944877	-0.04598	0.047061	-0.14634	0.008851
ARHGAP24	-0.020505898	-0.143166132	-0.27027	-0.05338	0.127589	0.190716
ARHGAP25	0.042483275	0.107223594	0.018426	-0.02209	0.084936	-0.30256
ARHGAP26	0.008729747	0.083911583	-0.13206	-0.03926	0.119209	0.349141
ARHGAP26-	-0.032467672	0.015472014	-0.05482	0.009859	-0.0057	0.091007
ARHGAP27	-0.046060348	-0.014759122	-0.21571	-0.08222	0.132893	0.361453
ARHGAP28	-0.077977115	-0.086911764	0.035637	-0.01376	-0.00292	0.052317
ARHGAP29	-0.005564735	-0.016773033	-0.07202	0.076169	0.011343	0.19323
ARHGAP30	0.039592184	0.050190506	-0.02739	-0.00775	0.022851	0.01942
ARHGAP31	-0.004395657	-0.062716603	-0.19331	-0.01042	0.040162	0.179013
ARHGAP32	-0.015155811	-0.097700756	-0.04126	-0.00987	0.086247	0.083752
ARHGAP33	-0.038104097	-0.085004352	-0.02369	0.00913	-0.07432	-0.0795
ARHGAP35	0.002227493	0.04912177	0.002362	0.041166	-0.10343	0.132975
ARHGAP36	-0.063227844	-0.049789532	-0.0117	-0.01331	0.034112	0.03992
ARHGAP39	-0.044598881	-0.066288743	0.02397	0.017206	-0.01507	0.125821
ARHGAP4	0.037042293	-0.079166426	0.068777	-0.05121	0.119392	-0.06678
ARHGAP42	-0.026439317	0.063020885	-0.10483	9.11E-05	0.049988	-0.18411

ARHGAP44	-0.044033558	-0.07701633	0.049082	-0.01179	0.007713	-0.02123
ARHGAP5	-0.041682693	-0.071776773	0.031492	-0.03002	0.05881	-0.22722
ARHGAP5-/	-0.020114861	-0.089996401	0.046861	-0.01852	0.043677	-0.07432
ARHGAP6	-0.023146287	-0.058384292	-0.07638	-0.20816	0.611951	0.184666
ARHGAP9	0.039140555	0.068162629	-0.12332	-0.02515	0.079435	-0.13092
ARHGDIA	0.034942248	-0.02551128	0.066717	0.056905	-0.17081	-0.00483
ARHGDIB	0.021027675	0.039921423	0.048861	-0.0073	0.024955	-0.04224
ARHGDIG	-0.031025357	-0.094855686	-0.05591	0.019406	-0.07142	0.003345
ARHGEF1	0.034734204	0.006302246	0.087286	-0.01395	0.021338	-0.1465
ARHGEF10	-0.02931558	0.092463224	0.015786	0.003966	0.037425	-0.08196
ARHGEF10I	-0.002778893	-0.106401962	-0.19199	-0.0697	0.217061	0.694823
ARHGEF11	-0.003072008	-0.116439163	-0.11355	-0.09183	0.389573	0.297868
ARHGEF12	-0.05058219	-0.063008562	-0.09773	0.033997	-0.01648	-0.02219
ARHGEF15	-0.031593633	-0.072416901	0.023274	-0.02484	-0.01374	-0.07609
ARHGEF16	-0.015967288	-0.026226019	0.006407	-0.01375	-0.07185	-0.08742
ARHGEF17	-0.00634202	-0.057333366	-0.00489	-0.00673	-0.08141	-0.06311
ARHGEF18	0.035965801	-0.026334602	0.153951	-0.05463	0.150775	-0.19881
ARHGEF19	-0.003638521	0.024550491	0.180896	-0.04964	0.059494	-0.22611
ARHGEF2	0.029014532	-0.055526091	-0.11174	-0.01731	0.004001	0.058034
ARHGEF25	-0.027875756	-0.019481824	0.138223	0.00351	-0.08074	-0.13799
ARHGEF26	-0.019418043	0.080021558	-0.04462	-0.10115	-0.19895	0.086842
ARHGEF26-	-0.06276233	-0.040296449	0.043405	-0.0236	0.092568	-0.09486
ARHGEF28	-0.001004624	0.048795064	0.067183	0.006996	0.0563	-0.18248
ARHGEF3	0.02959077	0.218045766	0.254285	-0.00962	0.030891	0.031506
ARHGEF33	-0.05652631	-0.033304779	-0.11233	-0.04977	0.120829	0.051604
ARHGEF37	-0.038480644	-0.083821541	-0.07608	0.013867	-0.04278	-0.0133
ARHGEF38	-0.035006835	-0.007948334	0.008799	0.010926	-0.0269	0.023927
ARHGEF39	-0.025142689	0.043444696	-0.06138	0.173768	-0.50723	-0.03271
ARHGEF4	-0.035129959	-0.055624412	0.009711	0.024675	-0.0744	-0.00318
ARHGEF40	0.027116672	-0.082092389	-0.28229	-0.08355	0.140428	0.337276
ARHGEF5	-0.028980839	-0.03728433	0.139411	-0.01731	0.007643	-0.19895
ARHGEF6	0.03580753	0.004073822	0.027497	-0.03672	0.096177	-0.03519
ARHGEF7	-0.047670145	-0.00984633	0.072348	-0.06659	-0.01736	0.006101
ARHGEF7-/	-0.076936484	-0.035603307	-0.02022	-0.01083	0.004868	0.037721
ARHGEF7-]	-0.037465296	-0.019654674	0.118974	-0.00926	0.049956	-0.09833
ARHGEF9	0.00411981	0.156003269	0.1549	0.035679	-0.07825	-0.1059
ARID1A	0.06651926	0.055646622	0.001686	-0.01893	0.092855	-0.04666
ARID1B	-0.025917022	-0.025459957	0.120734	0.006365	0.10659	-0.37859
ARID2	0.013550728	0.073205701	-0.02115	0.048144	-0.05377	-0.05403
ARID3A	0.010515102	-0.113130025	-0.30831	-0.01649	0.028254	0.234407
ARID3B	0.023644223	-0.012017475	0.110222	0.026498	-0.14198	-0.25542
ARID4A	0.010489917	0.008754145	-0.08115	-0.01056	0.052029	-0.06238
ARID4B	0.013565572	0.065151387	0.105781	-0.00141	-0.01542	-0.28893
ARID5A	0.034008457	0.075130595	0.026858	0.097676	-0.25946	-0.17179
ARID5B	0.000554026	0.079071227	0.011314	-0.02381	0.000844	-0.13374
ARIH1	0.053451838	0.056545287	-0.00388	0.038342	-0.08037	-0.0358
ARIH2	-0.003279423	0.104395112	0.220843	-0.04043	-0.12773	-0.13325
ARIH2OS	-0.007314282	0.065880608	0.207625	-0.03494	0.12937	-0.06717
ARL1	0.077616192	0.095460805	0.102742	0.027418	-0.1376	-0.00715
ARL10	0.051405848	0.109554827	-0.06178	-0.13506	0.073891	-0.3337
ARL11	-0.012472643	-0.145399311	-0.43159	-0.14003	0.396469	0.425871

ARL13B	-0.002245527	0.05634404	-0.03034	0.054137	-0.15466	0.026473
ARL14	-0.055290854	-0.025341319	-0.10496	0.019878	-0.06162	0.01531
ARL14EP	0.036066614	0.072921642	0.218801	0.027051	-0.06063	-0.05764
ARL15	0.009791684	0.066315685	0.012669	-0.04865	0.14968	0.21687
ARL16	0.023674648	-0.010352612	0.166118	0.0458	-0.1665	-0.11857
ARL2	0.021475769	-0.033139505	0.240353	0.096957	-0.25451	-0.04536
ARL2BP	0.043941424	0.098460893	0.112906	0.021447	-0.07482	0.096988
ARL3	-0.043939868	-0.025495626	0.102515	-0.07754	-0.04384	-0.14777
ARL4A	0.029335906	-0.123746342	-0.03669	0.051615	-0.17001	0.169821
ARL4C	0.028916276	0.237876557	0.348418	0.016266	-0.03222	-0.48895
ARL4D	-0.039003117	-0.106152639	0.051769	-0.01055	-0.03168	-0.10957
ARL5B	0.004945341	0.023200509	-0.04644	0.018026	-0.06701	-0.04654
ARL5C	-0.03832814	-0.053636871	-0.06168	0.012871	-0.08059	0.053011
ARL6	-0.021099683	0.053687343	0.124465	0.001311	-0.00689	-0.10645
ARL6IP1	0.076859075	0.212520108	0.055792	0.087726	-0.20505	-0.29603
ARL6IP4	0.049479344	-0.039841145	0.07657	0.041771	-0.13182	0.128318
ARL6IP5	0.052454073	0.107281526	0.059693	0.012835	-0.0148	0.004126
ARL6IP6	-0.005202213	-0.051699836	-0.13875	0.109552	-0.20359	-0.06832
ARL8A	0.029599538	-0.009429584	-0.31266	0.009776	-0.06456	0.152503
ARL8B	0.040903318	0.053789796	-0.18032	0.02011	-0.0529	0.305125
ARL9	-0.027735829	-0.074559754	-0.17406	-0.01007	0.020675	0.127436
ARMC1	0.032263101	0.105576746	0.158083	0.046383	-0.12215	-0.01553
ARMC10	0.036603064	-0.019767575	-0.08785	0.021122	-0.08216	0.176043
ARMC12	-0.033602552	-0.094821974	0.071419	-0.01404	0.059449	-0.07659
ARMC2	-0.029316977	0.000511876	0.101835	-0.03389	0.116335	-0.08301
ARMC3	-0.046681095	-0.065358579	0.087233	-0.04502	0.144753	-0.00782
ARMC4	-0.049261814	-0.080500945	-0.07503	-0.00956	-0.04183	-0.11672
ARMC5	0.005594907	-0.081230847	-0.05704	-0.01454	-0.06413	-0.15298
ARMC6	0.002169352	-0.04531951	0.047318	0.029859	-0.15397	0.017718
ARMC7	0.011974509	-0.017057705	-0.10224	-0.01133	-0.06249	0.042205
ARMC8	-0.050061792	-0.025859735	0.040434	-0.02288	-0.1298	-0.01916
ARMC9	-0.020642315	-0.034048438	0.026012	-0.00816	-0.00421	-0.02577
ARMCX1	-0.002695471	-0.150156347	0.081101	-0.08297	0.250315	0.225417
ARMCX2	-0.005322957	-0.058049616	0.273723	-0.13114	0.378357	-0.25065
ARMCX3	0.02241953	0.048816023	0.001493	-0.10513	0.276755	0.066388
ARMCX4	-0.082342878	-0.044506156	0.3203	0.005867	0.13277	-0.10989
ARMCX5	0.005105781	0.014962372	0.040944	-0.09032	0.295322	0.179634
ARMCX6	0.029470449	0.034258252	0.124073	-0.09572	0.23354	-0.12069
ARNT	0.023070495	0.027963265	-0.17126	-0.00732	0.085446	0.219915
ARNT2	-0.036124556	-0.050650996	-0.05383	0.00061	0.023242	0.093889
ARNTL	0.020333395	0.198273824	0.048396	-0.008	0.026862	-0.15831
ARNTL2	-0.026585334	-0.029735945	-0.02577	0.118734	-0.26672	0.010277
ARNTL2-AS	-0.048025565	-0.004328429	0.003269	0.052779	-0.10258	0.023451
ARPC1A	0.043768199	-0.026761876	-0.17334	0.05436	-0.15784	0.197089
ARPC1B	0.054259352	-0.026417134	-0.12004	-0.0014	0.036745	0.320615
ARPC2	0.095960096	0.122448604	-0.33156	0.058236	-0.15233	0.02555
ARPC3	0.080658236	-0.026918709	-0.34955	0.037758	-0.06678	0.259963
ARPC4	0.065226823	0.075357899	-0.19073	0.021233	-0.05212	0.100902
ARPC5	0.048380915	0.086427775	-0.12147	0.016278	-0.01916	0.098199
ARPC5L	0.036587006	0.254986619	0.178062	0.078182	-0.27332	-0.38942
ARPIN	0.002102286	-0.134585896	-0.15639	-0.10717	0.151066	0.589374

ARPP19	0.041126288	0.143604854	-0.06527	0.056833	-0.16345	-0.20587
ARPP21	-0.040714832	-0.09446392	-0.05447	0.003838	0.016987	-0.18709
ARR3	-0.018316485	-0.039186694	-0.02811	0.0068	-0.11724	-0.1332
ARRB1	0.008703389	0.014768749	-0.21866	-0.08184	0.25749	0.426842
ARRB2	0.030959837	-0.030860424	-0.09205	-0.01124	0.052722	0.275914
ARRDC1	-0.003065803	-0.064875845	-0.19892	-0.06897	0.154466	0.196924
ARRDC2	0.045866633	-0.034065963	0.067452	-0.07638	0.193018	0.027799
ARRDC3	0.01639796	0.065673965	0.084297	-0.01969	0.058725	-0.1584
ARRDC3-AS	-0.030484464	-0.067156729	-0.0263	0.03579	-0.11989	-0.01864
ARRDC4	0.000272236	-0.02228615	-0.40353	-0.11752	0.318939	0.503701
ARSA	-0.003617909	-0.090632457	-0.11134	-0.02238	0.042656	0.075058
ARSB	-0.004841619	-0.058995526	-0.29405	0.010291	-0.05307	0.36993
ARSD	-0.006715969	-0.01441977	-0.26097	-0.06929	0.210891	0.422653
ARSE	-0.035993664	-0.075353959	0.075084	0.030133	-0.06421	-0.13103
ARSF	-0.032979555	-0.013594832	-0.03492	-0.01152	0.011907	-0.13371
ARSG	0.001111671	0.103005113	0.010749	-0.02489	0.059043	0.142555
ARSI	-0.037457315	-0.03710666	-0.03334	0.020381	-0.02927	0.11932
ARSJ	-0.051486252	0.001648409	-0.0141	-0.04521	0.109809	-0.06721
ARSK	0.037595028	0.129732289	0.212389	0.049862	0.000804	-0.25035
ART1	-0.013257166	-0.008360612	-0.00344	0.050053	-0.1563	-0.07155
ART3	-0.035218403	-0.037132083	0.058208	0.069893	-0.15817	-0.03751
ART4	-0.054215198	-0.082860679	-0.00392	0.009725	-0.01311	0.041915
ART5	-0.027864206	-0.071592739	-0.05677	-0.02841	-0.00254	-0.0693
ARTN	-0.056160949	0.036220259	-0.22581	0.013819	-0.07186	0.274887
ARV1	0.012410352	0.019114269	0.159552	0.059873	-0.15725	-0.02923
ARVCF	0.020668615	-0.036110068	0.014363	0.009399	-0.04809	-0.12705
ARX	-0.041872513	-0.080836913	-0.02329	-0.0219	0.014194	0.065321
AS3MT	-0.02180161	-0.028418402	0.196564	-0.02821	0.09333	0.021053
ASAH1	0.017187622	-0.018410529	-0.31251	-0.08249	0.250591	0.380181
ASAH2B	-0.022552762	0.00764823	0.20117	0.025165	-0.05951	-0.31153
ASAP1	0.024360768	-0.077367492	-0.22577	-0.02009	0.06439	0.168123
ASAP1-IT1	-0.011662947	-0.0414822	-0.16913	-0.00073	-0.01972	0.090627
ASAP1-IT2	-0.005056597	-0.065811947	-0.17327	-0.00547	0.01204	0.147954
ASAP2	-0.002821733	-0.093578605	-0.16115	-0.12625	0.371129	0.161325
ASAP3	-0.031686769	-0.046600771	0.037259	-0.03189	-0.00235	-0.09846
ASB1	-0.015714059	0.16796945	0.096123	-0.00232	0.026958	-0.15983
ASB10	-0.036920981	-0.024951509	-0.04937	0.01574	-0.05621	0.058387
ASB11	-0.042262007	-0.025781639	0.009084	0.018489	-0.02772	0.12844
ASB12	-0.014379313	-0.040129074	0.020124	-0.00379	-0.03308	-0.03186
ASB13	0.003626273	-0.123671684	0.001444	-0.06989	0.200115	0.443338
ASB14	-0.047893079	-0.085435334	0.031407	0.013423	-0.03984	-0.07024
ASB15	-0.03155655	-0.007237124	-0.03555	0.038119	-0.1201	-0.01777
ASB16	-0.016882689	-0.026865368	0.037183	-0.01123	-0.06206	-0.09436
ASB16-AS1	-0.003807689	-0.035767759	0.041614	0.018131	-0.03755	-0.16641
ASB17	-0.062768129	-0.040054721	0.077248	0.002495	0.002587	0.172602
ASB2	-0.00373112	-0.018148892	-0.04157	0.096865	-0.26366	-0.08928
ASB4	-0.034487504	-0.048642285	0.006708	-0.0301	-0.1651	-0.01907
ASB5	-0.178906898	-0.07262079	0.009377	-0.00554	0.064692	0.053941
ASB6	0.024756804	0.003671084	0.090286	-0.00599	-0.01357	-0.06235
ASB7	0.023971068	-0.021104721	-0.1019	0.038401	-0.10778	0.040858
ASB8	0.022471957	0.05065487	-0.02267	-0.00733	0.012551	-0.05299

ASB9	-0.036604868	-0.046108187	-0.0099	0.021565	-0.04675	0.186441
ASCC1	0.023759458	0.098967681	0.012859	0.080538	-0.25456	-0.16307
ASCC2	0.024930128	-0.027129578	-0.10765	0.027957	-0.05825	0.234272
ASCC3	0.017798922	0.061951117	0.072018	0.102452	-0.25623	0.093982
ASCL1	-0.034365672	-0.050696569	-0.0121	0.049854	-0.02475	0.073687
ASCL2	-0.01472348	0.17621685	-0.06999	-0.07857	0.249108	0.253761
ASCL3	-0.025422829	-0.042331494	0.024836	-0.01354	0.005016	-0.07214
ASCL5	-0.036928271	-0.052761723	-0.02801	0.030192	-0.06158	0.017751
ASF1A	0.021787149	0.036707573	0.351467	0.016549	-0.05741	-0.19694
ASF1B	-0.007414185	0.018566148	-0.1127	0.113976	-0.39212	0.019585
ASGR1	-0.003630409	-0.091366121	-0.25008	-0.0583	0.177836	0.7799
ASGR2	0.003301728	-0.086676822	-0.22124	-0.05677	0.181351	0.649691
ASH1L	0.041191173	0.037877254	0.055285	-0.00581	0.01944	-0.19017
ASH1L-AS	0.01572064	-0.037830063	0.234848	0.035746	-0.13927	-0.04754
ASH2L	0.085420471	0.07355577	-0.21081	0.061187	-0.15494	-0.094
ASIC1	-0.032214906	-0.075237575	0.215767	-0.01517	0.001353	-0.17362
ASIC2	-0.033887293	-0.062942778	-0.0471	0.03901	-0.09982	0.116254
ASIC4	-0.034706775	-0.106008107	-0.05718	0.027709	-0.08481	-0.01381
ASIC5	-0.036722911	-0.123938141	0.022036	-0.00027	0.024831	-0.02867
ASIP	-0.009281659	-0.074631061	-0.13608	0.009801	-0.0036	-0.11822
ASL	0.010594965	0.150717557	-0.26368	0.037014	-0.11095	0.357367
ASMT	-0.046977325	-0.00075576	-0.17202	0.002419	0.02018	-0.00412
ASMTL	0.03955936	0.059550569	-0.12627	-0.00258	-0.08443	-0.01572
ASMTL-AS	0.046618091	-0.06673845	-0.03926	0.067695	0.027981	0.118663
ASNA1	0.029352333	0.072212014	-0.16621	0.028918	-0.09314	0.320099
ASNS	0.005223594	0.04186647	0.209711	-0.06142	0.137946	-0.38559
ASNSD1	0.073429487	0.087872841	0.263622	0.067047	-0.16479	-0.37678
ASPA	-0.035905182	-0.020277381	0.002299	-0.00936	-0.00391	-0.05576
ASPDH	-0.003814922	-0.039045196	-0.12516	0.017982	-0.09803	0.016699
ASPG	-0.049899369	-0.01786097	-0.08499	-0.00136	0.007745	0.095924
ASPH	0.009071136	-0.110762406	-0.14412	-0.08177	0.091546	0.04898
ASPHD1	-0.04111331	-0.076089256	-0.07385	0.015295	-0.09555	-0.04687
ASPHD2	0.013713512	-0.104718346	-0.21904	0.021574	-0.0833	0.203528
ASPM	0.010893493	0.026340793	0.020007	0.055134	-0.27767	-0.03437
ASPN	-0.05127617	-0.041417884	0.055127	0.024089	-0.04888	-0.00903
ASPRV1	-0.011335448	-0.049166853	-0.0616	-0.01269	-0.01196	-0.19063
ASPSCR1	0.012197066	0.003337867	0.030078	0.023355	-0.03512	0.165649
ASRGL1	-0.00081158	-0.093209749	-0.19711	-0.03647	0.084113	0.458748
ASS1	-0.009058166	0.01704632	0.07054	-0.01558	-0.00029	-0.15249
ASTE1	0.021060096	0.065345979	0.100719	-0.00486	0.059691	-0.08629
ASTN1	-0.053806185	-0.061269091	-0.02731	-0.01865	0.069314	0.036773
ASTN2	-0.030050486	0.036193772	0.121355	0.079165	-0.20857	-0.06749
ASUN	0.028149605	0.103306527	0.235325	0.054846	-0.14654	-0.08704
ASXL1	0.036593811	-0.03650028	0.211476	0.070028	-0.19122	-0.2656
ASXL2	-0.036740085	-0.051266079	-0.01521	0.042243	0.058273	-0.03872
ASXL3	-0.05972407	-0.003069644	0.000563	-0.03111	-0.08137	-0.00369
ASZ1	-0.084304046	-0.087658645	-0.00364	-0.01775	0.047607	-0.08374
ATAD1	0.021066858	0.134044181	0.275622	0.032483	-0.07777	-0.37678
ATAD2	0.005337527	0.081409914	0.075537	0.121488	-0.31216	-0.05109
ATAD2B	-0.032106109	0.019718661	-0.07795	-0.01463	0.027157	0.09599
ATAD3A	0.016292911	-0.041275537	0.030386	0.040066	-0.17881	-0.12896

ATAD3B	0.027211253	-0.040853199	0.048462	0.03544	-0.12039	-0.15797
ATAD3C	-0.019619126	-0.04716781	0.009792	0.007027	-0.00351	-0.03495
ATAD5	-0.005794464	0.047472974	0.127349	0.145717	-0.40347	-0.08471
ATAT1	-0.026518426	-0.081070025	0.095655	0.01277	-0.03809	-0.08358
ATCAY	-0.03185605	-0.015043501	-0.05933	0.023234	-0.09005	0.045258
ATE1	0.007918363	0.022367008	0.070682	-0.03841	-0.02481	-0.08068
ATF1	0.023069111	0.08704577	-0.00406	0.020503	-0.02629	-0.06024
ATF2	-0.006075645	0.073571756	-0.01709	0.046468	-0.12024	-0.10983
ATF3	0.007483045	-0.00143169	0.027198	0.104756	-0.29936	-0.05299
ATF4	0.078383809	0.006212413	-0.06597	0.11029	-0.20697	-0.03262
ATF5	-0.030644401	-0.107736693	-0.10837	-0.00899	-0.0724	0.139998
ATF6	0.064411886	-0.011924441	-0.14728	-0.00816	0.016914	0.331926
ATF6B	-0.105272422	-0.060675248	0.014796	-0.09402	-0.03898	0.098403
ATF7	0.076491669	0.01988266	-0.15382	0.088077	-0.01871	0.339329
ATF7IP	0.042372627	0.089475786	0.001924	-0.01989	0.045538	-0.30869
ATF7IP2	0.014174968	-0.044418748	0.433835	-0.05134	0.121417	-0.38428
ATG10	0.030333481	0.036492642	0.088092	0.041798	-0.16281	0.102515
ATG101	0.025649506	-0.001834397	-0.20625	0.042377	-0.13187	0.022252
ATG12	0.046553706	0.037949978	-0.03075	0.052235	-0.20603	0.131702
ATG13	0.019876467	0.092937339	-0.04376	-0.06957	-0.02273	-0.12626
ATG14	0.031208945	0.032459109	0.153699	-0.0162	0.078898	0.033083
ATG16L1	0.007297464	0.041459862	0.281573	0.00043	-0.00024	-0.2503
ATG16L2	0.023934282	-0.058955967	-0.31493	-0.04809	0.147405	0.152322
ATG2A	0.034732329	0.017903476	-0.04198	0.005833	0.002943	-0.12066
ATG2B	-0.020578851	0.12264106	0.075112	0.022976	-0.02543	-0.26566
ATG3	0.034184882	-0.04798233	-0.34676	-0.0204	0.07803	0.385235
ATG4A	0.02222156	0.131262689	0.061443	0.01838	-0.06639	-0.02272
ATG4B	0.046585865	0.002933184	-0.10318	0.034962	-0.10341	0.094538
ATG4C	0.005732757	-0.033029789	-0.13712	-0.0149	0.049153	0.104984
ATG4D	0.028384665	0.110080688	0.06021	0.040173	-0.15069	-0.28332
ATG5	0.011537331	0.120946015	0.016354	0.031521	-0.09229	0.031034
ATG7	0.014507325	-0.046397393	-0.30713	-0.02773	0.065928	0.612171
ATG9A	0.035604405	0.022231427	-0.06251	0.031777	-0.11845	0.090633
ATG9B	-0.031252018	-0.091139824	0.218112	-0.0661	0.187571	-0.22208
ATHL1	0.01731217	0.000488368	0.303642	-0.05001	0.135254	-0.30751
ATIC	0.025475649	0.025942374	0.239872	0.063878	-0.16638	-0.13544
ATL1	-0.00288362	-0.03903367	0.08937	-0.14082	0.401578	-0.20966
ATL2	-0.023088553	-0.026004668	0.032459	0.064222	-0.22292	-0.17472
ATL3	0.016996514	0.007960349	-0.17483	0.00655	-0.0323	0.283177
ATM	-0.006948591	0.094808795	0.306023	-0.06852	0.145193	-0.22007
ATMIN	0.096721766	0.201956672	0.100116	-0.02625	-0.00787	0.008291
ATN1	-0.030545596	-0.085286753	0.022983	-0.01252	-0.06875	-0.10354
ATOH1	-0.025247156	0.016669431	-0.0214	0.012865	-0.00043	0.181818
ATOH7	-0.104034839	-0.072478531	0.021328	0.0144	0.019713	-0.09559
ATOH8	-0.011923169	-0.071187109	-0.06083	0.014547	0.002125	0.174929
ATOX1	0.044705796	0.041515296	-0.28577	0.05998	-0.169	0.501007
ATP10A	-0.00942209	-0.016561819	0.045691	0.018811	-0.02906	-0.03909
ATP10B	-0.03289319	-0.064234208	-0.08631	0.002088	-0.03417	0.019787
ATP10D	0.019097155	0.065470861	-0.23793	-0.03571	0.102508	0.17131
ATP11A	-0.018448814	-0.148799711	-0.19566	-0.03994	-0.00407	0.480204
ATP11A-AS	-0.034087809	-0.017864207	-0.05302	0.016336	-0.08216	0.041261

ATP11B	-0.012030577		0.069638253	-0.13579	0.056135	-0.23849	-0.04281
ATP11C	0.010393201		0.023297951	0.139103	0.009549	-0.03746	-0.13276
ATP12A	-0.025093367		-0.055569916	-0.06783	0.001578	-0.07514	-0.03823
ATP13A1	0.039211217		-0.097851652	0.086314	-0.01314	0.046694	0.125083
ATP13A2	-0.000452553		-0.060594281	-0.07746	0.016698	-0.02778	0.083994
ATP13A3	0.017992638		0.031592591	0.003078	0.057344	-0.17882	0.0346
ATP13A4	-0.04547526		-0.0331922	-0.12197	0.007744	-0.02232	0.071389
ATP13A4-A	-0.091383179		-0.067798016	0.069212	-0.03905	0.101143	0.002647
ATP13A5	-0.05178936		-0.090987275	-0.05578	0.013927	-0.01854	0.075506
ATP1A1	0.037317357		-0.007640182	0.241395	0.009395	-0.02426	0.058313
ATP1A1-AS	-0.020896338		-0.052766633	0.054022	0.018545	-0.05491	-0.11319
ATP1A2	-0.047356528		-0.060492126	0.014644	-0.00548	0.037645	-0.02163
ATP1A4	-0.066241765		0.064968893	-0.0265	-0.02507	0.092255	-0.0202
ATP1B1	0.012020988		0.041822616	-0.10236	-0.03608	0.088038	0.099551
ATP1B2	-0.026087773		-0.072635306	0.091438	0.018356	-0.07748	-0.06446
ATP1B3	-0.038273515		0.002870665	-0.0823	-0.01524	-0.06497	-0.00328
ATP1B4	-0.069259363		-0.037019084	0.066644	0.002036	0.008469	-0.0301
ATP2A1	-0.044341549		-0.070305834	0.000264	0.069182	-0.15528	0.06409
ATP2A2	0.04028836		0.034161257	0.02589	-0.00081	-0.1658	0.294632
ATP2A3	-0.056497092		-0.044732128	0.097836	-0.02092	0.006203	-0.22685
ATP2B1	0.056233294		-0.030156649	-0.24904	-0.03981	0.119735	0.186118
ATP2B2	-0.047668638		-0.069514918	-0.13046	-0.0435	-0.02246	0.036239
ATP2B3	-0.058821991		-0.017228933	-0.04628	-0.04901	0.061369	0.244693
ATP2B4	0.029266839		0.197306506	0.138449	0.03019	0.000122	-0.08037
ATP2C1	0.024759824		0.04478403	-0.0743	0.052833	-0.12403	0.104281
ATP2C2	-0.080435969		-0.122036899	-0.11794	0.010849	-0.09616	0.06392
ATP4A	-0.034366908		-0.076167589	-0.12597	0.042397	-0.10801	-0.00841
ATP4B	-0.037498371		-0.090523551	-0.13979	-0.00255	0.028443	-0.03724
ATP5A1	0.043176317		0.07858811	0.073092	0.07095	-0.15052	0.080915
ATP5B	0.079355681		0.059556188	-0.01101	0.118045	-0.27909	0.16489
ATP5C1	0.07463309		0.04264906	0.0335	0.072256	-0.15745	0.187086
ATP5D	0.045723338		-0.045059531	0.142294	0.086265	-0.25546	0.167312
ATP5E	0.064066129		-0.017920751	-0.05915	-0.01595	0.122433	0.13395
ATP5F1	0.052730083		0.186193065	0.027467	0.060338	-0.09859	0.153045
ATP5G1	0.0418266		-0.015968733	0.091245	0.087271	-0.24447	0.123907
ATP5G2	0.033458419		0.003203905	0.123552	0.059844	-0.16455	0.127156
ATP5G3	0.048629566		0.060387619	0.120744	0.073196	-0.18306	0.216962
ATP5H	0.052380516		-0.038457027	0.209309	0.065701	-0.19127	0.105983
ATP5I	0.096021497		0.097605352	0.036247	0.118007	-0.28117	0.101797
ATP5J	0.08672123		0.080270748	0.065669	0.123955	-0.30719	0.140458
ATP5J2	-0.030416786		-0.043689445	-0.06962	0.033751	-0.11298	0.038424
ATP5L	0.082573121		0.058223235	0.137089	0.101297	-0.20676	0.07255
ATP5O	0.073244403		-0.03523958	0.178605	0.069878	-0.17936	-0.01116
ATP5S	-0.022265237		0.036707897	0.042484	-0.00231	0.087475	0.116647
ATP5SL	0.051313382		0.016615329	-0.06093	0.047801	-0.16699	0.141552
ATP6	0.056169463		0.09861855	-0.01434	0.088428	-0.11242	0.033821
ATP6AP1	0.058356906		-0.006553191	-0.22432	-0.00146	0.034197	0.234375
ATP6AP1L	-0.028112138		-0.03410135	0.031472	0.065807	-0.17375	-0.10933
ATP6AP2	0.064383498		0.087503487	-0.16922	-0.01485	0.073129	0.449889
ATP6V0A1	-0.003910807		-0.127786156	-0.24829	-0.0521	0.066406	0.164007
ATP6V0A2	-0.032031913		-0.003583385	0.04094	0.034947	-0.00811	-0.30874

ATP6V0A4	-0.02403778	-0.016856109	-0.04898	0.083465	-0.22611	0.046529
ATP6V0B	0.045893643	-0.062749133	-0.45516	0.015365	-0.01641	0.352166
ATP6V0C	0.061103626	-0.048005015	-0.20913	0.002215	0.029465	0.435293
ATP6V0D1	0.037136896	0.02371699	-0.44087	0.004616	0.025076	0.379418
ATP6V0D2	-0.022610191	-0.015316658	0.013495	-0.01494	-0.00898	-0.10332
ATP6V0E1	0.074804402	-0.034599088	-0.15417	-0.06114	0.121658	0.061706
ATP6V0E2	0.010984989	0.009373003	0.36345	0.040547	-0.12615	-0.23712
ATP6V0E2-	-0.01952898	-0.105833939	0.367587	-0.00596	0.042698	-0.12075
ATP6V1A	0.034244772	0.021345762	-0.45731	-0.00047	0.025558	0.367077
ATP6V1B1	-0.048554839	-0.108464768	0.032519	0.034994	-0.05767	0.054827
ATP6V1B2	0.031094704	-0.031128581	-0.38669	-0.00691	0.02357	0.361772
ATP6V1C1	0.039633277	0.072346506	-0.29716	0.010176	-0.03502	0.292652
ATP6V1C2	-0.049275251	-0.009043123	0.114	0.017867	-0.07196	0.001868
ATP6V1D	0.027863378	0.240505159	-0.31331	0.026017	-0.04468	0.282488
ATP6V1E1	0.123460312	0.109868503	-0.30071	0.0627	-0.10255	0.093997
ATP6V1E2	0.003507474	-0.047764923	0.106576	-0.01472	0.056036	0.042531
ATP6V1G1	0.060570993	-0.045371795	0.013742	-0.05543	0.158993	0.0594
ATP6V1G2	-0.022964762	-0.028827366	0.153221	0.028653	-0.06595	-0.17947
ATP6V1G3	-0.036258129	-0.029758659	-0.06463	0.017359	-0.02778	-0.09664
ATP6V1H	0.001738656	0.037509566	0.053903	0.002132	0.025368	-0.16201
ATP7A	0.010348748	0.087297721	-0.10629	0.008942	-0.02051	-0.08625
ATP7B	-0.025293141	-0.009419866	-0.0502	0.060646	-0.16213	-0.08078
ATP8A1	-0.013529926	0.058030238	0.048359	0.021934	-0.05412	-0.21688
ATP8A2	-0.022216982	-0.060169727	0.22256	0.159178	-0.3913	-0.09657
ATP8B1	0.022118677	0.01349255	0.038155	-0.01011	0.02627	-0.19363
ATP8B2	0.004467129	0.001391118	0.399424	-0.03152	0.044224	-0.41271
ATP8B3	0.05824072	0.077829314	-0.09684	0.100963	-0.26605	0.146024
ATP8B4	0.003344875	0.179151903	-0.2022	0.072689	-0.20434	0.239612
ATP8B5P	-0.075743695	-0.106443239	0.035532	-0.01521	0.04089	0.099846
ATP9A	-0.008572105	0.143438455	0.00116	-0.05327	0.13646	-0.05711
ATP9B	-0.006577362	-0.030399122	0.098341	-0.0975	0.069192	0.008375
ATPAF1	0.013761836	0.007215763	0.119455	-0.01543	0.034862	0.043713
ATPAF2	-0.003468315	-0.121453318	-0.19138	-0.00458	0.026188	0.236098
ATPIF1	0.038964236	0.063876218	0.188987	0.028615	-0.09889	-0.05212
ATR	0.021150431	0.060668833	0.13749	0.037057	-0.0605	-0.02769
ATRAID	0.053584824	0.028933593	0.188028	0.040476	-0.11332	0.041786
ATRN	0.009256755	-0.003399545	-0.03499	-0.02287	0.094252	0.475248
ATRNL1	-0.017467166	-0.122143998	0.00139	0.012216	0.080119	-0.00656
ATRX	0.022008168	0.106147472	0.148059	-0.0357	0.04298	-0.32686
ATXN1	0.026779503	0.011730681	0.043066	-0.02801	-0.00986	0.114349
ATXN10	0.04245042	0.026901645	0.109063	0.018523	-0.06298	0.070667
ATXN1L	0.07840795	0.132009244	0.010291	-0.02171	0.038936	-0.0273
ATXN2	0.029877338	0.020392542	0.035046	-0.00296	-0.01579	0.059538
ATXN2L	-0.006657038	-0.078755155	0.110188	0.026133	-0.08692	0.093593
ATXN3	-0.023512587	0.028041252	-0.09848	-0.04772	0.021378	0.26982
ATXN3L	-0.056750836	-0.070347654	-0.03363	-0.01408	0.022483	0.034946
ATXN7	0.077364825	0.032977642	0.154953	-0.04836	0.144442	-0.21769
ATXN7L1	-0.040361364	0.015411289	-0.02703	-0.04185	0.057944	0.33288
ATXN7L2	-0.026000202	0.034129448	0.052621	-0.00998	0.054743	0.057357
ATXN7L3	0.041674164	-0.123150704	0.101542	-0.0207	-0.0172	-0.06463
ATXN7L3B	0.035153005	0.185997399	0.253032	-0.00723	0.015824	-0.20655

ATXN8OS	-0.040661538	-0.045830648	0.043378	0.001984	-0.00155	-0.08312
AUH	0.019991424	0.090150552	0.08182	0.032179	-0.12486	-0.11442
AUNIP	-0.022878827	-0.028357135	0.020297	0.144728	-0.46022	-0.0575
AUP1	0.07492521	0.033828404	-0.06046	0.012387	-0.02461	0.103373
AURKA	0.006115173	-0.049881152	-0.05744	0.169982	-0.43073	0.048868
AURKAIP1	-0.011338594	-0.097852885	-0.10654	0.160462	-0.13958	0.285815
AURKB	-0.013096628	-0.012073697	-0.04004	0.172705	-0.40719	0.014533
AURKC	-0.027874234	-0.042664673	-0.04448	0.018935	-0.08586	-0.1564
AUTS2	-0.018458265	-0.068528705	-0.14577	-0.0407	0.007692	-0.19746
AVEN	0.020287224	0.075211177	0.012301	0.081556	-0.22754	-0.06291
AVIL	-0.056428852	-0.080209718	-0.07301	-0.09445	0.060856	-0.0894
AVL9	-0.03274351	-0.083013033	-0.09839	0.035259	-0.16136	0.002872
AVP	-0.031934564	-0.008143233	0.021773	0.00282	0.004493	0.015597
AVPI1	0.005728564	-0.061436083	0.010614	-0.01657	0.021009	0.035988
AVPR1A	-0.085430778	-0.012604758	-0.02606	-0.12105	0.451666	-0.05185
AVPR1B	-0.014121209	-0.058324844	-0.00871	-0.01331	-0.06898	-0.00375
AVPR2	-0.034107067	-0.05808763	-0.11658	0.007002	-0.033	0.037915
AWAT1	-0.038982183	-0.050690701	0.038654	-0.01711	0.084311	-0.04876
AX746627	-0.046982683	0.004690141	0.031388	-0.02174	0.064069	0.041986
AX746699	-0.03469249	-0.079497989	-0.01173	-0.01753	0.067142	-0.02467
AX746710	-0.060054734	-0.027763719	0.106513	-0.01321	0.025532	-0.07304
AX746823	-0.009828137	-0.037260826	-0.05827	-0.07064	0.187574	0.041028
AX746830	-0.060767696	-0.089680558	-0.01017	-0.02881	0.070416	0.151982
AX746968	-0.023964943	-0.08975578	0.012667	0.004613	-0.04236	-0.06095
AX747031	-0.03210292	0.017472854	0.048419	0.029191	-0.10954	-0.10158
AX747064	-0.019401253	0.053699704	0.084081	-0.0225	0.075599	0.043407
AX747191	-0.041348955	-0.021678555	-0.04473	0.0097	-0.00498	0.134267
AX747250	-0.035841968	-0.029214184	-0.03739	-0.01575	0.069729	-0.09099
AX747261	-0.035067427	-0.075740103	0.00318	0.065018	-0.18356	0.011162
AX747405	-0.029850735	0.071567093	0.064542	-0.00315	0.025086	-0.21831
AX747444	-0.048283307	-0.034113109	-0.07353	-0.00542	0.038625	-0.07649
AX747507	-0.021111357	0.014580794	-0.00335	-0.01623	0.058547	0.159167
AX747630	-0.056821586	-0.04538885	0.02254	0.019439	-0.0686	0.158525
AX747652	-0.014777764	0.004563126	0.115018	0.020879	-0.01954	-0.10918
AX747730	-0.008852913	0.000195892	0.052726	0.04219	-0.11622	-0.03983
AX747826	-0.009424463	-0.153810556	0.127374	0.018979	-0.0388	-0.11344
AX748157	-0.034868414	-0.063230876	0.010033	-0.0098	0.002177	-0.05188
AX748267	-0.014450021	-0.081043712	-0.09627	0.009942	-0.01291	0.165996
AX748273	-0.065412514	-0.011306285	-0.03617	-0.01085	0.04267	0.095401
AX748292	-0.033574948	0.055790394	-0.04713	-0.01993	0.062895	-0.07243
AX748339	0.005090124	-0.058502058	-0.06906	-0.00301	0.007398	-0.08847
AXDND1	-0.037746896	-0.04771994	-0.0228	0.093859	-0.14631	0.044588
AXIN1	0.012962275	0.126346377	0.058979	-0.0383	0.049082	-0.18546
AXIN2	-0.097592612	-0.183313129	0.218216	0.00956	0.134857	-0.17355
AXL	-0.020532333	-0.082977927	-0.09233	-0.02193	0.044559	-0.08816
AY927499	-0.011536171	0.07890212	0.041228	0.006412	0.005517	-0.05962
AY940074	0.016548609	-0.09174703	0.021058	0.031188	-0.08046	-0.16212
AZGP1	-0.038192797	-0.01922243	0.009598	-0.01492	0.049014	-0.07611
AZGP1P1	-0.049356314	-0.038459655	-0.00274	0.024778	-0.03701	-0.12552
AZI2	0.016245134	0.017569199	-0.13178	0.027168	-0.0051	0.603301
AZIN1	0.068531651	0.071793612	-0.19772	0.040582	-0.10422	0.042445

AZIN2	-0.012830091	-0.064048694	0.09436	-0.01634	-0.00557	-0.22063
AZU1	-0.012689398	-0.072801738	0.051979	-0.04474	0.139117	-0.01195
B2M	0.090856792	0.271474024	-0.3	0.191547	-0.03147	-0.11281
B3GALNT1	-0.000122946	0.059258592	-0.0317	-0.0111	0.007045	-0.08895
B3GALNT2	-0.041028755	-0.011210153	-0.12281	0.051993	-0.08104	0.008602
B3GALT1	-0.024505388	0.01436858	0.001443	0.002275	-0.01852	-0.09132
B3GALT2	-0.015862448	-0.010501393	0.092328	-0.01668	0.024221	-0.03868
B3GALT4	0.004869507	0.137189874	0.010621	-0.05271	0.134216	0.042776
B3GALT5	-0.047838957	-0.087704384	-0.0544	0.05342	-0.00698	0.020096
B3GALT6	0.019988001	0.102656022	-0.03738	0.024615	-0.09172	-0.00209
B3GALTL	-0.000706211	0.198605015	0.184518	0.057708	-0.13389	-0.15103
B3GAT1	-0.012299289	0.232665166	0.101581	-0.02887	0.032398	-0.19138
B3GAT2	-0.030365788	-0.026613091	-0.00735	0.000136	0.010033	0.059883
B3GAT3	0.011074819	-0.029490621	0.116672	0.079419	-0.27852	-0.0976
B3GNT1	0.001218417	-0.026938532	0.113846	0.040851	-0.11267	-0.17866
B3GNT2	0.003241732	0.082140988	0.111901	0.040349	-0.1259	-0.32786
B3GNT3	-0.021111184	-0.070106457	-0.0259	-0.01067	-0.04961	-0.01517
B3GNT4	-0.024726397	-0.041475619	0.064268	-0.0133	-0.06085	-0.13112
B3GNT5	-0.002408759	-0.021210479	-0.07095	0.004498	0.011572	-0.03251
B3GNT6	-0.046052358	-0.066465633	-0.01574	0.043109	-0.13075	0.046806
B3GNT7	-0.002129427	0.28293101	-0.21209	-0.00037	0.044949	-0.21955
B3GNT8	-0.018256292	-0.079476426	-0.17726	-0.03342	0.055355	0.155156
B3GNT9	-0.007682311	-0.067455944	0.019591	0.025108	-0.07525	-0.14102
B3GNTL1	-0.005751392	-0.09840438	-0.13207	0.003944	-0.03979	0.139239
B4GALNT1	-0.038533067	-0.059469978	-0.08142	0.048628	-0.15193	0.024617
B4GALNT2	-0.039435348	-0.073241816	0.097122	0.020217	-0.02609	0.090326
B4GALNT3	-0.068659483	-0.133287817	0.029863	0.039737	-0.01661	-0.04786
B4GALNT4	-0.034358299	-0.035824753	-0.0995	0.012167	0.002777	0.254456
B4GALT1	0.05554802	0.059100551	-0.05653	-0.0573	-0.07917	-0.06446
B4GALT2	-0.010845061	-0.018578582	-0.00341	0.05989	-0.2527	-0.08168
B4GALT3	0.023623649	0.050683225	0.275475	0.029962	-0.07401	-0.14349
B4GALT4	0.010043076	0.13589021	-0.09047	0.035028	-0.10901	-0.12181
B4GALT5	0.02610228	0.094502399	-0.11261	0.03702	-0.09821	0.094688
B4GALT6	-0.004601659	0.191388983	-0.10764	-0.10547	0.103968	-0.11014
B4GALT7	0.006766697	-0.080094985	-0.11163	0.054721	-0.1798	0.107689
B9D1	-0.048960383	-0.005095937	-0.05733	0.116428	0.012161	-0.05613
B9D2	0.006551257	-0.107881813	-0.18502	-0.01244	-0.01402	0.096651
BAALC	-0.01705468	0.103931184	-0.04708	-0.0076	0.017562	-0.05208
BAALCOS	-0.052182691	-0.071648504	0.073378	0.008679	-0.01526	-0.00042
BAAT	-0.035473716	-0.132897011	0.009296	0.003296	-0.01073	0.105893
BABAM1	0.052044779	0.068198098	0.095747	0.039771	-0.11254	0.048715
BACE1	0.02364936	-0.111313674	-0.12623	-0.05571	0.122187	0.477233
BACE2	-0.00655965	-0.080604132	-0.14059	-0.04289	0.111116	-0.20647
BACH1	-0.047798506	-0.038969011	-0.06341	-0.03796	-0.02825	0.056032
BACH2	-0.000162752	-0.008765171	0.25618	-0.06771	0.170588	-0.33013
BAD	-0.00713619	-0.023933853	-0.11517	0.038485	-0.12095	0.233848
BAG1	0.057063161	0.04138667	-0.12584	0.049658	-0.06704	0.026432
BAG2	0.021822933	0.040203145	0.28174	0.047323	-0.26528	-0.36344
BAG3	0.006516821	-0.102544114	0.288815	-0.04759	0.118931	-0.02259
BAG4	0.01414823	-0.01041806	-0.26662	0.014682	-0.03957	0.321811
BAG5	0.019603333	0.084523959	0.050493	-0.01066	-0.01423	0.011901

BAG6	0.077744053	-0.023899142	-0.13856	0.07398	-0.13169	0.06414
BAGE	-0.035894458	-0.046804321	-0.04528	-0.00933	0.00287	0.025916
BAHCC1	-0.061438757	-0.029331195	-0.13836	-0.01071	-0.04639	0.120401
BAHD1	0.015069802	0.119173654	0.107286	0.018917	-0.12704	-0.27548
BAI1	-0.030402375	-0.072520255	-0.08809	-0.01372	-0.0079	-0.07366
BAI2	-0.036068484	0.14312748	-0.04848	-0.00482	0.041109	-0.03802
BAI3	-0.039165684	-0.090784959	0.00715	0.064233	-0.09184	-0.04539
BAIAP2	-0.034907096	-0.086465065	-0.15212	-0.03059	0.043998	0.093199
BAIAP2-AS	0.021416824	-0.106975658	-0.22955	-0.06953	0.18728	0.466856
BAIAP2L1	-0.034100505	-0.061028493	-0.00874	0.003478	-0.04831	-0.05011
BAIAP2L2	-0.049419646	-0.068754118	0.061637	0.010382	-0.04897	-0.04361
BAIAP3	-0.011173016	-0.054945498	0.08102	-0.0228	-0.01416	-0.2922
BAK1	0.017559881	-0.012586061	-0.03302	0.078059	-0.29108	0.045111
BAMBI	-0.02005104	-0.085807726	0.054809	-0.03111	0.080991	-0.08893
BANCR	-0.026824132	-0.066844723	0.071236	0.014134	-0.03975	-0.08948
BANF1	0.025731143	0.09322887	0.13065	0.060787	-0.17345	0.109726
BANF2	-0.042099052	-0.07106106	-0.06082	-0.01282	0.062848	-0.07514
BANK1	-0.002234101	-0.109819124	-0.18238	-0.08861	0.249589	-0.11841
BANP	0.069393154	-0.017101165	-0.17536	0.028836	0.011361	-0.11813
BAP1	0.020476261	0.030177014	-0.03897	0.041838	-0.16061	-0.07124
BARD1	-0.018395688	0.063400596	-0.03626	0.146217	-0.30696	-0.16967
BARHL1	-0.020254334	-0.078459327	-0.0102	-0.00555	-0.07513	-0.06259
BARX1	-0.03111369	-0.033004201	-0.03013	-0.00373	0.035906	0.069604
BARX1-AS	-0.030906554	-0.004521585	-0.03147	0.000839	-0.0383	-0.06861
BARX2	-0.027306976	-0.046235117	-0.04814	0.029593	-0.14229	-0.03819
BASP1	0.010320325	-0.176026885	-0.34238	-0.06877	0.186912	0.091045
BATF	0.021055984	0.132830406	0.12303	0.05977	-0.18354	0.027463
BATF2	-0.016458091	-0.09142865	-0.2564	-0.01657	0.032526	0.224693
BATF3	0.006972903	-0.058545839	-0.11782	0.078119	-0.23369	0.093533
BAX	0.008427547	-0.001513884	-0.03957	0.095491	-0.25409	0.216277
BAZ1A	0.053504562	0.060483397	-0.24514	0.015625	-0.01025	0.088079
BAZ1B	-0.008159485	-0.038770355	0.097705	0.028047	-0.11784	-0.06628
BAZ2A	0.034214252	0.006805068	-0.12721	-0.02073	0.016622	-0.05355
BAZ2B	0.021563519	0.019298411	-0.31599	-0.07178	0.236392	0.145088
BBIP1	0.01118756	0.01888659	0.105533	-0.01908	0.093443	-0.05871
BBOX1	-0.04488013	-0.063394822	-0.03732	-0.03626	0.113373	0.025648
BBS1	0.044817624	0.016547066	0.075304	-0.00965	-0.06424	-0.02048
BBS10	-0.005229921	0.039165079	-0.086	0.006586	0.00031	-0.04246
BBS12	-0.008575846	0.050579728	0.01182	0.094673	-0.22311	0.050987
BBS2	0.011113179	-0.014582849	0.222488	0.003113	0.028821	-0.04127
BBS4	0.015295552	0.107771297	0.144233	0.014371	-0.04219	-0.19468
BBS5	-0.049481627	-0.00595945	-0.06116	-0.02438	0.092528	-0.00521
BBS7	-0.010109014	0.075756791	0.070769	0.067659	-0.17366	0.071431
BBS9	-0.083085574	-0.033175546	0.081221	0.03249	-0.04299	-0.00996
BBX	0.057485131	0.085159886	0.208428	0.014998	0.018143	-0.36228
BC010186	-0.009203167	0.019411094	0.031458	0.034224	-0.08392	-0.06979
BC012193	-0.015990689	0.00015553	0.010109	0.015313	-0.02698	0.019339
BC015159	-0.042242497	-0.045318064	0.01103	0.015936	-0.01786	0.10344
BC016361	-0.024297767	-0.04647366	-0.03181	-0.05078	0.089478	-0.02439
BC017209	-0.038382223	-0.098798647	0.14555	0.008542	-0.00107	-0.04325
BC021061	-0.038980911	-0.000127216	-0.04288	0.01842	-0.03091	0.002708

BC022047	0.017533633	-0.033684128	0.168465	-0.02723	0.086391	-0.25189
BC022892	-0.012848238	0.044256352	-0.03504	-0.04157	0.134744	0.058731
BC023201	-0.023437501	-0.058021181	-0.09442	0.037003	-0.07503	0.03206
BC024169	-0.029282054	-0.042924288	-0.03317	0.004347	0.00163	-0.02992
BC027448	-0.032043983	-0.038120991	-0.00155	0.010035	-0.05249	-0.05765
BC028044	-0.054939789	-0.026049514	0.023464	-0.01271	0.041856	0.023649
BC028670	-0.031984337	-0.035981733	-0.13184	0.020743	-0.03909	-0.09032
BC030152	0.00077812	0.072442283	0.010626	0.048425	-0.10467	0.125132
BC032415	-0.01106843	-0.004400302	-0.1873	0.03258	-0.08803	-0.03925
BC033164	-0.023055647	-0.077778321	0.03412	-0.00699	-0.03142	-0.2173
BC033241	-0.051407287	-0.062031074	0.041978	-0.00249	0.000551	0.034368
BC034416	-0.03271701	-0.076358716	0.135885	0.000157	0.02142	-0.09484
BC034444	-0.034599016	-0.05494518	0.089567	0.001086	0.025574	-0.04911
BC034788	-0.032823866	0.012215213	-0.04255	0.002309	-0.02667	-0.06224
BC035400	-0.038118796	0.080067653	-0.06122	0.006872	0.007365	0.016527
BC036209	-0.080435939	-0.035492203	-0.07404	-0.0124	0.012437	0.019577
BC036311	-0.089887087	-0.061188172	-0.05001	-0.02057	0.029139	0.048721
BC038205	-0.04851572	0.036774008	0.064484	0.056443	-0.16829	-0.0516
BC039122	-0.02488975	-0.020685138	0.10111	-0.00155	0.004609	-0.01252
BC039319	-0.051133336	-0.086060036	0.086836	-0.00711	0.027373	-0.12214
BC039487	-0.089129565	0.106687802	-0.11301	-0.00096	0.010502	-0.02809
BC039673	-0.021354159	-0.030063525	-0.00117	-0.00859	-0.0212	-0.05405
BC039686	-0.025113889	-0.116353036	0.089691	-0.02986	0.089298	-0.03679
BC040311	-0.082415428	-0.055215693	0.121693	-0.03278	0.053607	0.009332
BC040734	-0.029715769	-0.045077753	0.004361	-0.01254	0.035506	0.043613
BC040833	-0.036248723	-0.021408198	0.116685	0.007573	-0.00058	-0.06295
BC041025	0.007390849	-0.013888908	0.058179	0.041978	-0.07344	0.084835
BC041363	-0.048624117	0.030617883	0.050561	0.019455	-0.04525	-0.06366
BC041998	-0.027652046	-0.033771795	0.045928	0.038842	-0.10868	-0.02946
BC042029	-0.045689744	-0.011546162	0.073302	0.026299	-0.06674	-0.05155
BC042366	-0.11643814	-0.035661273	0.110586	-0.00568	0.030987	-0.01993
BC042374	-0.054212908	-0.014417862	-0.09726	0.009282	0.001582	-0.04057
BC042590	-0.013476556	-0.023610921	-0.10105	-0.01305	0.044284	0.12773
BC043227	-0.068461215	-0.0475767	-0.00818	0.003333	-0.01877	0.000905
BC043291	-0.030839478	-0.010022982	-0.01719	-0.00652	0.036798	-0.09657
BC043356	-0.01064279	0.089513726	-0.18759	-0.00397	0.027173	-0.09444
BC043540	-0.027240583	-0.056678177	0.052479	0.013125	-0.02777	0.011459
BC044596	0.000383066	-0.088245668	-0.02525	-0.00374	-0.04268	-0.07157
BC044614	-0.051528724	-0.066978403	0.145352	0.014985	-0.02785	-0.09951
BC045559	-0.063298306	-0.077064939	0.099595	-0.01956	0.052934	-0.05109
BC045560	-0.020517198	0.053525961	0.081028	0.006904	-0.01491	-0.08028
BC045779	-0.033378453	-0.026069728	-0.0052	0.03574	-0.07712	-0.00546
BC045784	-0.028718057	-0.054425414	0.076161	-0.00296	0.021121	-0.0916
BC045788	-0.036525903	-0.070042938	0.092885	-0.00379	0.031675	0.012237
BC045789	-0.020562826	-0.005654147	0.090553	-0.01177	0.053943	0.001558
BC045791	-0.044790416	-0.023269244	-0.09303	0.024222	-0.06764	-0.13517
BC045805	-0.035459085	-0.015477096	-0.14787	0.012649	-0.02418	-0.11581
BC047364	-0.02541582	-0.058952763	0.013148	-0.00994	0.029959	-0.15548
BC047484	0.029943761	0.093755113	0.173987	-0.01628	2.30E-05	-0.36998
BC047615	-0.091322707	-0.047001245	0.027794	-0.01594	0.054698	-0.09163
BC047626	-0.042878684	-0.093124369	0.089081	-0.00325	0.040806	-0.01813

BC047644	-0.042591783	-0.044012903	-0.11184	0.016625	-0.04981	0.069084
BC047651	-0.014982601	0.142126094	0.06423	-0.03365	0.110737	0.03341
BC048132	-0.052362121	-0.049635367	0.13285	0.010459	-0.02525	-0.05235
BC048141	-0.028077452	0.038364396	0.200558	0.017709	-0.0455	-0.0851
BC048420	-0.071182676	-0.10794556	0.104938	-0.00488	0.036976	0.141308
BC048997	-0.034121844	-0.046829162	0.068236	-0.00155	0.026587	-0.09275
BC053951	-0.041220553	-0.046214917	0.089297	0.039161	-0.08275	0.022675
BC062753	0.024058218	0.142962012	0.199524	0.007509	-0.01574	-0.02966
BC062763	-0.0135081	-0.010669299	-0.13699	0.079068	-0.23038	0.136684
BC069004	-0.032743411	-0.044415226	-0.06306	-0.03179	0.106599	0.127855
BC069739	-0.029033044	0.002723553	-0.00275	0.030866	-0.07661	-0.0253
BC069756	-0.035087153	-0.030091649	0.172964	-0.01658	0.075625	-0.03713
BC069776	-0.018405907	0.029981746	0.099224	-0.01108	0.058123	0.067652
BC070118	-0.027461804	-0.005765361	-0.00474	-0.00368	-0.03018	-0.0173
BC070490	-0.05848224	-0.029825071	-0.00166	0.016566	-0.02761	0.008943
BC079832	-0.02932844	-0.000692939	-0.0792	0.021658	-0.0564	-0.00082
BC113958	-0.017539571	-0.071431112	-0.15477	-0.02258	0.084781	-0.06136
BC130595	-0.035055933	-0.103977265	-0.04838	-0.01106	-0.02018	-0.05037
BCAM	-0.027002873	-0.084276366	-0.09394	-0.0078	-0.02031	-0.13806
BCAN	-0.040211467	-0.038496625	-0.05455	0.04707	-0.13335	0.035161
BCAP29	-0.035756695	0.076582219	-0.04021	0.117839	-0.22743	0.204954
BCAP31	0.086564518	0.120827905	0.043898	0.056359	-0.11978	-0.09069
BCAR1	-0.044460242	-0.089950848	0.08252	-0.00745	-0.06253	-0.11281
BCAR3	-0.00098641	-0.025140707	-0.08183	-0.00776	0.012555	-0.08428
BCAR4	-0.03190195	-0.030338423	0.080735	0.003498	0.005887	0.044229
BCAS1	-0.023029056	0.026396124	-0.07584	0.025939	-0.07191	-0.04928
BCAS2	0.07400918	0.168176498	0.223209	0.07964	-0.21253	-0.31897
BCAS3	-0.000339426	0.017345405	-0.00371	0.031319	-0.06948	-0.00108
BCAS4	-0.002770644	-0.059701431	0.332036	0.043791	0.042457	-0.38594
BCAT1	-0.001736111	-0.07977234	-0.16444	0.056671	-0.16226	0.248714
BCAT2	0.0015359	-0.075082576	0.053478	0.084861	-0.25789	-0.04601
BCCIP	0.02376878	0.000162505	0.073961	0.02379	-0.09955	0.095092
BCDIN3D	-0.005435687	0.065030213	0.095983	0.014392	-0.10252	-0.1337
BCDIN3D-/	-0.047183979	-0.093125394	-0.06047	0.00538	0.016727	-0.06517
BCHE	-0.024616565	-0.026305583	-0.008	0.136743	-0.09184	-0.00503
BCKDHA	0.040697621	-0.04596391	-0.06905	0.028528	-0.10405	0.310929
BCKDHB	-0.001083763	-0.014856644	0.169622	0.058306	-0.15316	-0.20601
BCKDK	0.013378271	-0.092792997	-0.35411	0.003261	-0.04661	0.492802
BCL10	-0.06015404	-0.044212851	-0.06644	-0.01648	0.086623	-0.06163
BCL11A	0.010479814	-0.154128007	-0.27098	-0.09609	0.258936	-0.05062
BCL11B	0.005803839	0.086735783	0.491209	-0.04046	0.119223	-0.23463
BCL2	0.032840012	0.009514098	0.215338	-0.05432	-0.04362	-0.35344
BCL2A1	0.014584146	-0.077997894	-0.12078	-0.03609	0.093843	0.14124
BCL2L1	-0.029352971	-0.013263474	0.012392	-0.02958	-0.08595	-0.06203
BCL2L10	-0.043716575	-0.0639137	-0.06811	0.022538	-0.03992	0.102091
BCL2L11	0.031019562	-0.040609979	-0.07068	-0.06139	0.008229	0.02864
BCL2L12	-0.004171398	0.033173865	0.058264	0.119417	-0.33374	-0.08905
BCL2L13	7.05E-05	-0.012695857	-0.12021	-0.04524	-0.0488	0.202745
BCL2L14	-0.008394291	-0.020472753	0.111361	0.065956	0.011021	0.012026
BCL2L15	-0.009414563	0.03923782	0.241778	0.031859	-0.00587	-0.1275
BCL2L2	-0.003995508	0.03896443	-0.30769	-0.06007	0.191911	0.429611

BCL6	0.003443103	-0.046065291	-0.23661	0.021457	-0.01107	0.120812
BCL6B	0.002764761	-0.075569777	-0.00537	0.003328	-0.1448	-0.06245
BCL7A	-0.002108441	-0.15074448	-0.05882	-0.02067	0.056953	-0.25189
BCL7B	0.045670515	-0.002745265	0.071775	0.063948	-0.21808	-0.1428
BCL7C	0.041145441	0.055724169	0.157702	-0.02082	-0.01867	-0.09178
BCL9	-0.018452509	0.030613413	0.233972	0.017068	-0.07623	-0.07716
BCL9L	0.006522028	0.113753269	0.328815	-0.05522	0.139684	-0.16692
BCLAF1	0.044427197	0.040615074	0.048303	0.030484	-0.08925	-0.08885
BCMO1	-0.037565763	0.019510499	-0.07313	-0.00211	0.033006	-0.04218
BCO2	-0.029612283	-0.01286217	-0.01818	0.040936	-0.11282	0.035225
BCOR	-0.006445126	0.014953112	-0.05211	0.037711	-0.07532	-0.08069
BCORL1	-0.0413102	-0.090682925	-0.07685	0.016668	-0.07544	-0.01825
BCORP1	-0.024369614	0.023620135	0.240218	-0.03484	0.110762	-0.28545
BCR	0.019534714	0.104851886	0.16502	-0.0483	0.183948	-0.13015
BCRP3	-0.045347133	-0.060340306	-0.10348	0.031834	-0.06773	0.222683
BCS1L	0.019574151	-0.031705284	0.163917	0.052511	-0.1757	-0.12817
BD495725	0.007134824	0.076387446	-0.01737	0.002414	0.027219	0.06899
BDH1	0.00073161	-0.005423243	0.234219	0.088917	-0.24533	-0.29352
BDH2	0.010168978	0.077463623	0.229227	0.025166	-0.05257	-0.10797
BDKRB1	-0.029232088	-0.082498256	-0.04074	0.008874	-0.03582	0.078116
BDKRB2	-0.030289334	-0.040311245	-0.01238	-0.01382	0.040158	-0.11378
BDNF	-0.033789966	0.007887453	-0.0305	-0.0058	-0.02831	-0.10362
BDNF-AS	-0.110911152	-0.048492701	-0.02898	-0.02734	0.084025	-0.07518
BDP1	-0.033332861	0.127880712	0.118194	3.19E-05	-0.06485	-0.01257
BEAN1	-0.046227083	0.033931148	-0.00722	0.001916	0.027612	-0.08963
BECN1	0.074131974	0.085044591	-0.02629	0.055194	-0.11206	0.056095
BEGAIN	-0.020836814	-0.099953019	-0.11479	-0.00699	0.014027	-0.05189
BEND2	-0.018651094	-0.036929793	-0.06871	-0.23819	0.715035	0.096519
BEND3	-0.003426093	0.068719502	-0.01145	0.039467	-0.13781	0.042705
BEND4	-0.020817646	-0.053719649	-0.15249	-0.02907	0.091433	-0.21842
BEND5	-0.013465526	-0.124868804	0.039481	-0.04007	0.120773	-0.36285
BEND6	-0.009787861	-0.053840159	-0.09042	0.005926	0.021319	-0.16194
BEND7	-0.028164163	-0.046363097	-0.0081	0.006294	0.003536	-0.10425
BEST1	-0.007132991	-0.093878637	-0.26161	-0.0421	0.081605	0.344042
BEST2	-0.032760886	-0.002443195	-0.06968	-0.03255	0.098658	0.109136
BEST3	-0.089053649	-0.027446813	-0.02411	0.012285	-0.01116	0.049288
BEST4	-0.028459977	-0.006561429	0.195415	-0.01017	0.048635	-0.15262
BET1	0.021702707	0.057203774	-0.04337	0.012789	-0.05235	-0.00129
BET1L	0.018030594	-0.133455396	0.083307	-0.03058	0.057853	-0.15012
BEX1	-0.019803237	-0.107820596	0.087503	-0.04959	0.108213	-0.09102
BEX2	0.006400523	0.052336124	0.384436	-0.10002	0.281635	-0.47985
BEX4	0.015245846	0.058651052	0.173752	-0.1107	0.307086	-0.04576
BEX5	-0.002395661	0.042596602	0.358932	-0.05795	0.151202	-0.29633
BFAR	0.045008612	0.121169523	0.167076	0.008451	-0.06487	-0.00867
BFSP1	-0.009888205	0.204604863	0.025391	0.006448	0.007041	-0.10963
BFSP2	-0.034357349	-0.065805387	-0.1486	-0.01095	0.060533	0.021359
BFSP2-AS	-0.052720418	-0.081900716	0.175975	0.012357	-0.0012	-0.04778
BGLT3	-0.02702531	-0.049356233	-0.05406	-0.01854	-0.00307	-0.03995
BGN	-0.038506435	-0.09473746	-0.06588	0.060157	-0.08912	-0.02478
BHLHB9	-0.028909299	0.11474356	0.254069	-0.07106	0.231547	-0.18963
BHLHE22	-0.030039712	-0.013003861	0.032607	0.01689	-0.04618	0.017313

BHLHE23	-0.040927363	-0.02548041	-0.11737	0.011508	-0.00059	0.241751
BHLHE40	0.019620416	0.145255244	-0.12293	0.121387	-0.34239	-0.16247
BHLHE41	0.002614885	-0.03363603	-0.14428	-0.04941	0.16931	-0.13239
BHMT	-0.029882472	0.003417377	0.02785	0.018195	-0.04914	0.00159
BHMT2	-0.073022958	0.045847491	-0.00763	-0.04278	0.020049	-0.03516
BICC1	-0.028791921	-0.025	0.055949	0.008264	-0.00262	-0.06442
BICD1	0.021205063	0.059201223	0.085957	0.012027	-0.13528	-0.24916
BICD2	0.038350599	-0.016747489	-0.39798	-0.07288	0.202276	0.270798
BID	0.039362642	0.010288884	-0.31985	-0.10158	0.225699	0.312118
BIK	-0.008263921	-0.12637734	-0.03878	-0.074	0.19248	-0.1805
BIN1	-0.004961076	0.087723549	0.279558	-0.05311	0.087609	-0.39853
BIN2	0.032847449	0.110976017	0.060805	-0.05414	0.145187	-0.00935
BIN3	0.01511511	-0.075190258	-0.03986	-0.01502	-0.01142	-0.05136
BIN3-IT1	-0.013419405	-0.12222092	-0.13065	-0.00161	-0.01758	-0.01013
BIRC2	0.08223188	0.169164641	-0.0171	0.022793	-0.00115	-0.1227
BIRC3	0.010623261	-0.053540946	0.030975	0.0171	-0.05378	-0.18029
BIRC5	-0.013101607	0.004825375	0.033858	0.087682	-0.38538	-0.04204
BIRC6	0.061030271	0.177302559	0.101749	0.029654	-0.06038	-0.25907
BIRC7	-0.014051449	-0.078348821	-0.02659	-0.01221	-0.03766	-0.07218
BIVM	-0.019107928	0.052402654	0.043328	0.095791	-0.10051	-0.11396
BLACAT1	-0.022295043	-0.039721612	-0.00367	0.052018	-0.1357	-0.00146
BLCAP	0.073884646	0.020424687	-0.04725	-0.00549	0.02156	0.004583
BLID	-0.048617361	-0.027639414	-0.1025	-0.00629	0.028013	0.004076
BLK	-0.008804338	-0.085088703	-0.0527	-0.0447	0.125365	-0.15764
BLM	0.011468959	0.110190774	-0.11815	0.049527	-0.1856	-0.12095
BLMH	0.028550108	0.086376773	0.308945	0.064923	-0.17747	-0.00955
BLNK	0.001011555	-0.124089659	-0.18644	-0.08004	0.216631	-0.16441
BLOC1S1	0.054254948	-0.019954538	-0.2559	0.042053	-0.10891	0.25439
BLOC1S2	0.092754218	0.014444972	-0.32861	0.048083	-0.09027	0.046861
BLOC1S3	0.010381737	0.019828335	-0.1117	-0.0385	0.080314	0.086356
BLOC1S4	0.029225023	0.086039741	0.352825	-0.01895	0.007717	-0.15838
BLOC1S6	0.103532243	0.063122173	-0.12204	-0.02296	0.06276	0.137354
BLVRA	0.003559505	-0.000449751	-0.1472	-0.0828	0.247294	0.614998
BLVRB	0.019298031	-0.062440563	-0.31347	-0.0134	0.046053	0.674563
BLZF1	-0.042597011	0.101971579	-0.01232	0.044204	-0.04929	0.00572
BMF	0.006232667	-0.177831489	-0.2447	-0.05464	0.107735	0.294201
BMP1	0.057162086	-0.082765991	-0.06789	0.042019	-0.14137	-0.02075
BMP10	-0.037992768	-0.002098777	-0.06206	0.023922	-0.03584	0.046054
BMP15	-0.033913209	-0.016068886	0.045642	0.001349	0.003951	0.140032
BMP2	-0.015719929	0.028738985	-0.04123	-0.0031	-0.01095	-0.01349
BMP2K	0.015999223	-0.101344885	-0.41584	-0.01825	0.069541	0.389022
BMP3	-0.036954935	-0.044186496	0.074028	0.019241	-0.03789	-0.01332
BMP4	-0.026467698	-0.067911367	0.06624	0.028814	-0.11138	-0.10444
BMP5	-0.044740243	-0.05111912	-0.04467	0.025047	-0.06694	0.065075
BMP6	-0.057939932	-0.040326863	0.022318	-0.04275	0.019033	0.062482
BMP7	-0.038995791	-0.119221019	0.021795	0.053489	-0.1273	0.037952
BMP8A	-0.028024191	-0.033953393	-0.01919	0.004146	0.013777	0.050345
BMP8B	-0.052276399	-0.033073818	-0.00637	0.03077	-0.08396	0.001196
BMPER	-0.07928874	-0.024764989	0.103084	0.020985	-0.0527	-0.08198
BMPR1A	-0.02030903	0.140705178	0.208515	0.081382	-0.06986	-0.17925
BMPR1B	-0.049320993	-0.076191732	-0.00947	-0.00805	-0.03006	-0.01058

BMPR2	0.026349491	0.069218807	0.013267	-0.05659	0.151764	0.187718
BMS1	0.030272041	0.035352489	0.150775	0.045582	-0.14544	-0.11266
BMS1P20	-0.018857589	-0.003521414	-0.12491	0.007697	0.093084	-0.04409
BMS1P5	-0.016483396	0.008807533	0.131715	-0.01782	0.046574	-0.12579
BMS1P6	-0.003993827	-0.025681819	0.147623	0.003685	-0.0883	-0.11348
BMX	-0.01969685	-0.037817209	-0.10992	-0.00527	0.014398	-0.11928
BNC1	-0.079517263	-0.133321027	-0.0105	-0.01644	0.052498	-0.00415
BNC2	-0.027072084	0.419030712	-0.08144	-0.04205	0.072462	-0.12651
BNIP1	-0.04799944	0.034450912	0.091561	0.150107	-0.23318	0.048972
BNIP2	0.087029758	0.041237469	-0.32326	-0.01694	0.080151	0.194273
BNIP3	-0.000142381	0.020859435	0.397463	-0.08371	0.241734	-0.19615
BNIP3L	0.03831118	-0.041503405	-0.22688	-0.02684	0.088985	0.330771
BNIPL	-0.043791099	-0.015367485	0.084126	0.038219	-0.07705	-0.07135
BOC	-0.041884072	-0.073420116	-0.08057	-0.03197	-0.00578	-0.01219
BOD1	0.034697761	0.008761139	0.174341	0.023422	-0.08591	-0.10361
BOD1L1	0.024170928	0.017684105	-0.17041	-0.00277	0.026169	-0.01081
BOD1L2	-0.0374957	-0.05319076	-0.02971	0.018344	-0.02611	0.03792
BOK	-0.007797452	0.389248986	-0.11645	-0.03044	0.107692	-0.09801
BOLA1	-0.002901129	0.089349779	0.080089	0.04277	-0.09012	0.018468
BOLA2	-0.020513879	-0.069076528	0.041603	-0.01106	-0.00831	0.007009
BOLA3	0.024689275	0.08287328	0.341737	0.080844	-0.21577	-0.19533
BOLA3-AS1	-0.032892943	0.002802294	0.076847	-0.07572	0.249956	-0.08404
BOLL	-0.022349987	-0.058864161	0.054064	-0.00806	0.01161	-0.05301
BORA	0.008414803	0.036930821	-0.04127	0.090395	-0.24819	0.060762
BPESC1	-0.068584628	-0.072148555	-0.03317	-0.00058	-0.01764	0.032227
BPGM	0.006249139	0.235357587	-0.10596	0.043767	-0.12496	-0.04678
BPHL	-0.004774279	-0.032222453	0.094647	0.104669	-0.27951	0.007792
BPIFA1	-0.028941215	-0.022560399	0.003933	-0.00119	-0.00554	0.064028
BPIFA2	-0.032796946	-0.016551458	-0.04032	0.013698	-0.01925	-0.02181
BPIFA3	-0.030862603	-0.050661274	0.031746	0.008771	-0.07879	0.029988
BPIFA4P	-0.026283399	-0.017562533	-0.01559	0.009602	-0.03265	-0.11482
BPIFB1	-0.041107798	-0.128740773	0.092717	-0.01298	0.049338	-0.02621
BPIFB2	-0.030865796	-0.018460622	-0.01209	0.014248	-0.09316	-0.02118
BPIFB4	-0.033959445	-0.023263546	-0.13804	0.018812	-0.03085	0.14062
BPIFB6	-0.048397336	-0.037577685	0.032224	0.013884	-0.01102	-0.01931
BPIFC	-0.031786768	0.021117068	0.028295	0.028362	-0.08366	-0.09082
BPNT1	0.031498046	0.060252684	0.044093	0.147424	-0.18495	0.06942
BPTF	0.036286864	0.106293457	0.085081	0.00642	0.011743	-0.30728
BPY2	-0.045803085	-0.112486215	0.060555	-0.00104	0.03141	-0.01969
BRAF	-0.03119878	-0.021395746	-0.05907	0.011636	-0.09324	-0.08158
BRAP	0.046954826	0.080685236	-0.11579	0.022709	-0.05145	-0.00131
BRAT1	0.025304843	-0.024040402	-0.18963	0.013515	-0.05053	0.264223
BRCA1	-0.010596349	0.047422531	-0.0554	0.147192	-0.42285	-0.00564
BRCA2	-0.0308032	-0.027675526	-0.07402	0.215709	-0.42986	-0.07143
BRCC3	0.03701401	0.079724364	-0.04666	0.05563	-0.08962	0.193245
BRD1	0.038308576	0.071830629	0.127084	0.000272	-0.01268	-0.20418
BRD2	0.094213073	0.263503206	0.014214	0.03092	-0.00466	-0.43611
BRD3	0.003321272	0.060062271	-0.13018	-0.02367	0.079544	0.171972
BRD4	-0.015459251	-0.061010345	0.022328	-0.02834	-0.14114	-0.27848
BRD7	-0.011068384	0.144631898	0.013215	0.065692	0.001121	-0.01759
BRD7P3	-0.045785151	-0.028360095	0.102995	-0.01881	0.092697	-0.03385

BRD8	-0.011359913	-0.001111186	0.052155	0.046229	-0.12174	-0.15635
BRD9	0.041839709	-0.034622798	0.099065	0.018244	-0.0998	-0.1526
BRDT	-0.041931694	-0.032904259	-0.06285	-0.00374	0.020996	0.022146
BRE	0.057161062	-0.030957288	0.052022	0.054451	-0.12334	0.186387
BRE-AS1	-0.008549111	-0.025170322	-0.04879	-0.00865	-0.00616	-0.02891
BREA2	-0.024816629	-0.005928174	-0.00989	0.016254	-0.07167	-0.00856
BRF1	-0.010706048	-0.00833709	-0.02777	0.00624	-0.05042	-0.02463
BRF2	0.027074046	-0.008714531	0.087927	0.040568	-0.18643	-0.18947
BRI3	0.037666225	-0.157212874	-0.32485	-0.03293	0.085414	0.470453
BRI3BP	0.027685712	-0.04797503	-0.29716	0.038624	-0.10363	0.058778
BRICD5	-0.011838715	-0.125859451	-0.04077	-0.00303	-0.02792	0.101046
BRINP1	-0.032518894	-0.043942996	0.038893	0.028169	-0.08345	-0.08359
BRINP2	-0.029235452	-0.017066959	0.01865	0.03615	-0.10344	-0.10472
BRINP3	-0.065864244	-0.001925338	-0.07329	-0.03046	0.079322	-0.12954
BRIP1	-0.018850671	0.046930996	0.096187	0.186084	-0.34405	-0.16028
BRIX1	0.01430913	0.003136148	0.126953	0.078157	-0.2079	-0.07279
BRK1	0.077854424	0.065153868	-0.09248	0.000588	-0.02697	0.140576
BRMS1	0.052898894	-0.05005575	-0.11809	0.071591	-0.24013	0.073625
BRMS1L	-0.007803647	-0.025510606	0.059008	0.092609	-0.22806	-0.06172
BROX	-0.073486064	-0.039262153	0.010703	-0.02231	0.074441	0.10234
BRPF1	0.054864618	0.149799079	0.075818	-0.02608	0.036965	-0.11503
BRPF3	-0.017697994	-0.054005594	-0.12587	0.028253	-0.05531	0.093695
BRS3	-0.052107653	-0.023435929	0.039105	0.001451	0.003992	-0.08423
BRSK1	0.00180367	-0.086044952	-0.03484	-0.04081	0.022419	-0.07077
BRSK2	-0.043176768	0.008707178	-0.10398	0.014214	-0.01383	0.006147
BRWD1	0.008436397	0.094052995	0.161486	0.013031	0.018907	-0.09796
BRWD1-IT2	-0.035089731	-0.054138838	0.007841	0.015738	-0.01849	0.103489
BRWD3	0.017061924	0.015103771	-0.22725	0.037006	0.013498	0.182024
BSDC1	0.050902553	0.016341591	-0.00507	-0.05139	0.138409	-0.26921
BSG	0.023869292	0.049966738	-0.07215	0.079743	-0.24026	0.310896
BSN	-0.036801555	-0.017900229	0.068981	0.046094	-0.12284	-0.03059
BSN-AS2	-0.027674703	-0.053415943	0.025632	0.000776	-0.0037	-0.03013
BSND	-0.031034384	-0.059355258	-0.10149	0.007241	-0.04438	0.010862
BSPRY	-0.006587168	0.049516959	-0.0472	0.001105	-0.01598	-0.15081
BST1	-0.00112925	-0.116142303	-0.27196	-0.11477	0.324242	0.56859
BST2	0.031671052	0.076143156	-0.04276	0.042785	-0.1072	0.297498
BTAF1	0.045244367	-0.012104758	0.034686	-0.00941	0.02794	-0.04475
BTBD1	0.090303038	0.155069267	-0.0353	0.054008	-0.13925	-0.07165
BTBD10	0.040967239	0.089809698	-0.25848	0.051846	-0.15509	0.040437
BTBD11	0.000273351	-0.034431102	0.188597	-0.02859	0.119824	0.089107
BTBD16	-0.044509241	-0.057944241	-0.18233	0.022243	-0.04004	0.000426
BTBD17	-0.039421023	-0.018553438	-0.10324	0.012693	0.001338	0.198302
BTBD18	-0.010502564	0.011277126	0.131431	0.001123	0.03492	-0.01431
BTBD19	-0.012062367	-0.097019109	-0.01657	0.000249	-0.01435	-0.07641
BTBD2	-0.019352279	-0.143060461	0.045211	-0.00047	-0.01006	0.075564
BTBD3	0.003336223	-0.053538911	-0.2579	-0.08526	0.259891	0.34966
BTBD6	0.043557184	0.099959234	0.241401	0.053532	-0.16561	-0.35163
BTBD7	0.020684401	0.090775682	-0.13582	-0.03628	0.107066	0.076587
BTBD8	-0.056002283	-0.040953579	0.040232	-0.01164	0.016488	0.044301
BTBD9	-0.012249849	-0.038774196	0.115652	-0.01196	0.004581	-0.0221
BTC	-0.100366289	-0.133465075	0.053356	0.009633	0.021602	-0.1148

BTD	0.020601222	0.037274186	-0.01652	-0.00289	-0.01238	0.192523
BTF3	0.063900309	0.053318762	0.190038	0.063855	-0.13291	0.108596
BTF3L4	0.117446377	-0.049133971	-0.10634	-0.04149	0.082302	0.311776
BTF3P11	-0.034111279	-0.045105609	-0.01639	-0.01016	0.034777	0.108599
BTG1	0.042270035	0.117057849	0.115369	-0.05128	0.143881	-0.33509
BTG2	0.023886527	-0.050036388	-0.1551	-0.03229	0.076852	-0.12724
BTG3	-0.016403154	-0.021895357	0.142692	0.079736	-0.27346	-0.20596
BTG4	-0.027276825	-0.028302531	-0.02112	0.055157	-0.15195	-0.09146
BTK	0.010744897	-0.158851321	-0.45002	-0.11978	0.323251	0.301086
BTLA	0.000327223	-0.104735106	0.052621	-0.06969	0.175863	-0.36067
BTN1A1	-0.026507985	-0.039971309	0.013169	-0.00039	0.015315	-0.1116
BTN2A1	0.041571797	0.037156688	-0.06281	-0.01669	0.05756	-0.20606
BTN2A2	-0.044064387	-0.070008095	-0.19832	0.065536	-0.10884	0.088341
BTN2A3P	-0.036483087	0.003716321	-0.07846	0.007702	-0.01781	0.056175
BTN3A1	0.027655206	0.150992372	0.252677	-0.04305	0.114221	-0.2444
BTN3A2	0.022481829	0.149097627	0.261204	-0.0273	0.062482	-0.16405
BTN3A3	0.018120372	0.121342659	0.258391	-0.01797	0.070877	-0.11073
BTNL2	-0.031615554	-0.037885514	-0.12952	0.023312	-0.05931	0.087768
BTNL3	-0.016781822	-0.037946004	-0.03507	-0.00038	-0.08055	-0.06328
BTNL8	-0.00994781	-0.041586851	-0.16142	-0.00696	0.003295	-0.10948
BTNL9	-0.04359373	-0.143527413	0.010587	-0.02964	0.030783	-0.07593
BTRC	-0.055771759	0.039746404	-0.01193	0.038687	-0.10074	-0.11504
BUB1	0.020929726	-0.000565807	0.037943	0.116127	-0.38042	-0.0833
BUB1B	-0.00389791	-0.011926911	0.02265	0.132811	-0.28645	-0.09444
BUB3	0.034679097	0.11760408	0.388404	0.036986	-0.11558	-0.43282
BUD13	0.038954787	0.080132578	-0.19493	0.040616	-0.18731	-0.05394
BUD31	0.046360155	0.067851779	-0.00253	0.084916	-0.23145	0.102785
BVES	-0.034542594	-0.05419196	-0.06415	0.012067	-0.01313	-0.02494
BVES-AS1	-0.045345306	-0.074699553	-0.04593	-0.00206	0.016887	-0.08782
BX648501	-0.043141414	-0.083228675	0.060648	0.022083	-0.05702	-0.11652
BYSL	0.005440477	0.051347446	-0.09674	0.072105	-0.22467	0.012574
BZRAP1	0.002657592	0.291098216	0.093878	-0.01489	0.038987	-0.19918
BZRAP1-AS	0.014692929	0.118766406	0.330365	-0.03244	0.078976	-0.41976
BZW1	0.060149973	0.198006013	0.016711	0.06304	-0.14658	-0.08008
BZW2	0.037466577	-0.038344278	0.121325	0.063806	-0.18608	0.036331
C10orf10	-0.015072065	-0.029901311	-0.04489	0.077761	-0.1991	0.014138
C10orf10	-0.060249266	-0.06184098	-0.00999	-0.00768	0.009209	-0.04296
C10orf11	0.001537768	-0.084668676	-0.19553	-0.06332	0.187234	0.488147
C10orf11	-0.02689885	0.048776524	-0.07016	-0.00687	0.019561	0.015568
C10orf12	-0.03964002	0.006808912	0.031111	-0.02175	0.006985	-0.11362
C10orf12	-0.115518826	-0.110069525	-0.18924	-0.00902	0.10747	0.050916
C10orf12	0.00734106	0.020845509	-0.05597	0.029831	-0.08978	-0.23738
C10orf2	-0.006667258	-0.017319352	0.178476	0.029376	-0.11339	-0.23211
C10orf25	-0.012851776	0.001844738	0.047179	0.031765	-0.07687	-0.12332
C10orf32	0.034735446	0.028737691	-0.0331	-0.08719	0.229784	0.24772
C10orf35	-0.014552085	-0.118613934	0.323521	-0.05078	0.104249	-0.19721
C10orf40	-0.041736391	-0.069729551	-0.00807	-0.0053	-0.00368	-0.05277
C10orf53	-0.043451023	-0.09273145	-0.11416	-0.00572	-0.0029	0.067886
C10orf54	0.019360102	-0.076030657	-0.1445	-0.07813	0.219007	0.306114
C10orf55	-0.038357724	-0.008773021	-0.09182	-0.03083	0.0821	-0.0153
C10orf62	-0.041253874	-0.050741299	-0.05358	0.0194	-0.0813	-0.08012

C10orf67	0.001472736	-0.097764645	0.156787	0.014819	-0.05776	-0.1093
C10orf71	-0.033260513	-0.071261665	-0.05796	-0.04624	0.165943	0.018296
C10orf71-	-0.03434987	-0.053267424	-0.09067	0.019575	-0.02398	-0.01658
C10orf76	-0.062499381	-0.060283179	-0.13178	0.021772	0.102617	0.187806
C10orf82	-0.028009434	-0.026550628	0.035878	-0.01646	0.058521	0.118735
C10orf85	-0.033628838	-0.040555872	0.008279	-0.00447	-0.02685	-0.0704
C10orf88	0.000691607	0.072507777	0.105755	0.034856	-0.10292	-0.17373
C10orf90	-0.017057537	0.007527224	-0.02976	-0.00685	-0.1014	0.099377
C10orf91	-0.028395735	0.061150919	0.012527	########	0.03583	-0.01534
C10orf95	-0.015809197	-0.029846815	0.116106	-0.00732	0.004646	-0.16438
C10orf99	-0.041539557	-0.025848864	-0.00749	0.042937	-0.10935	-0.08892
C11orf1	0.002687703	-0.056456029	0.335815	0.076918	-0.13089	-0.13035
C11orf16	-0.015028467	-0.075066178	-0.02751	-0.01416	-0.05339	-0.0798
C11orf21	0.010844321	0.066450194	0.095009	-0.08228	0.22138	0.124636
C11orf24	0.014468454	-0.025050197	-0.1921	0.028957	-0.09652	0.01327
C11orf30	-0.044854823	0.071336869	0.033613	-0.00736	-0.11327	-0.11002
C11orf31	0.028290887	-0.012546435	0.121205	-0.00578	0.034494	0.143914
C11orf40	-0.049142099	-0.059469383	0.016278	-0.00963	0.022526	-0.00597
C11orf42	-0.031561082	-0.004943331	-0.00911	0.025605	-0.05972	-0.03912
C11orf44	-0.031583673	-0.073998877	-0.06699	0.016108	-0.02597	-0.02254
C11orf45	-0.043964528	-0.127346125	-0.01566	-0.01488	0.081362	0.19058
C11orf49	0.010457453	0.008099297	0.140722	0.0429	-0.10852	-0.13753
C11orf53	-0.018465957	-0.103938443	0.064096	0.009719	-0.09118	-0.0653
C11orf54	0.027554408	0.026735559	-0.12576	0.007668	-0.00682	0.037845
C11orf57	-0.011029977	0.057343952	-0.0299	0.051925	-0.03676	0.051994
C11orf58	0.04065053	-0.023284968	0.031229	-0.02492	0.079666	0.094032
C11orf63	-0.024550958	-0.069532128	0.175619	-0.04116	0.134456	-0.20295
C11orf65	-0.047672925	-0.054326601	0.053164	-0.01748	0.041271	-0.08573
C11orf68	0.036299923	0.032757405	0.023567	-0.02923	0.043941	-0.1181
C11orf70	-0.073139631	-0.068859371	-0.03818	-0.00502	0.022446	0.032973
C11orf71	0.013821322	-0.023534183	0.033581	0.023686	-0.04608	0.350507
C11orf72	-0.020122379	-0.043716201	0.015751	-0.01449	-0.07545	-0.10483
C11orf73	0.024848659	0.039384989	0.140561	0.055678	-0.1529	0.102833
C11orf74	-0.003539012	-0.082040639	-0.17821	-0.04784	0.156874	0.482912
C11orf80	-0.008062518	-0.001488476	-0.07075	0.017367	-0.0572	-0.23837
C11orf83	0.01527974	0.03842921	-0.05732	0.072025	-0.17313	0.133969
C11orf84	-0.004390824	0.056795422	-0.08234	0.154958	-0.47607	-0.18021
C11orf85	-0.033148879	-0.068867658	0.130831	0.014873	-0.04666	-0.13039
C11orf86	-0.02556985	-0.043337422	-0.03668	0.013819	-0.08532	-0.00158
C11orf87	-0.063314133	-0.124619631	-0.07147	-0.04978	0.06605	0.024439
C11orf88	-0.0423358	-0.015896317	-0.05144	0.00395	0.02565	0.03925
C11orf92	-0.080997788	-0.035216347	-0.03993	-0.0319	0.104272	0.061664
C11orf94	-0.02992231	-0.016566314	-0.14946	0.016548	-0.08371	-0.00925
C11orf95	-0.002177092	-0.026563916	0.148947	0.046238	-0.11188	-0.06622
C11orf96	-0.009543511	-0.013393445	-0.0307	0.002023	0.003609	-0.0061
C11orf97	-0.040075035	0.023233504	-0.0605	0.015043	-0.01234	0.043414
C12orf10	0.020463523	-0.045630202	0.086067	0.039479	-0.09252	0.300757
C12orf29	0.008091929	0.069971546	0.25621	0.05172	-0.12906	-0.22809
C12orf4	0.024290334	0.052657632	-0.1887	0.011917	-0.00803	0.200317
C12orf40	-0.086417562	-0.106476328	-0.01662	-0.04594	0.134287	-0.05735
C12orf42	-0.018228349	-0.105232259	0.036576	-0.00984	0.045382	-0.26661

C12orf43	0.020534081	0.092278385	0.157272	0.021941	-0.062	0.03491
C12orf45	0.020390378	-0.0851718	0.001776	0.033345	-0.09289	0.02036
C12orf49	0.058965584	-0.003369014	0.03658	0.083419	-0.10901	0.052805
C12orf5	0.031754186	0.050591353	-0.27936	0.058102	-0.13554	0.364504
C12orf50	-0.054835756	-0.06340572	-0.05092	-0.03699	0.118511	0.062892
C12orf54	-0.012533875	-0.003764136	-0.11958	0.07991	-0.12017	0.004591
C12orf55	-0.041480958	-0.102960869	-0.05577	0.018892	-0.05334	-0.07744
C12orf56	-0.035509065	-0.024965649	0.086117	0.008456	-0.00801	0.032584
C12orf57	0.018222491	0.043440086	0.433908	0.000795	0.001917	-0.3543
C12orf60	-0.030263512	0.027431639	0.036898	0.090098	-0.22864	-0.04655
C12orf61	-0.043860884	-0.051538737	-0.01912	-0.00225	0.024014	0.041957
C12orf65	0.013097734	0.031172441	0.426556	0.038084	-0.05385	-0.35783
C12orf66	0.048823965	-0.028451202	0.231194	0.078284	-0.06043	-0.05779
C12orf73	0.014712448	0.09538197	0.22456	0.073771	-0.21083	-0.08062
C12orf74	-0.057223745	-0.131072251	-0.0493	0.021004	-0.08206	0.077305
C12orf75	0.012905919	0.15824697	0.340325	0.017787	-0.04889	-0.44411
C12orf76	-0.009602689	-0.070138415	0.077449	-0.10404	0.246926	-0.09399
C12orf77	-0.035314507	-0.022961339	0.05451	-0.01194	0.021738	-0.10232
C12orf79	-0.006245354	-0.026466463	0.224463	-0.00136	0.011002	-0.13023
C12orf80	-0.032236007	-0.086893425	-0.04861	-0.00126	-0.01697	-0.02873
C14orf1	-0.0050704	0.115452951	0.290169	0.081211	-0.2166	-0.33841
C14orf10	-0.047283537	-0.01532925	0.079705	-0.01591	0.053506	-0.11745
C14orf11	0.059562598	0.162448779	-0.01194	0.050567	-0.13105	0.058789
C14orf13	-0.024647877	-0.099894879	0.346926	-0.03121	0.092021	-0.2099
C14orf14	0.021573069	0.088198682	-0.01072	0.050575	-0.1181	0.176364
C14orf15	0.028671651	-0.022714986	-0.10465	0.001309	-0.02366	0.049242
C14orf16	0.055527477	0.031994412	0.171964	0.055829	-0.1465	0.137047
C14orf16	0.035434217	0.111438653	0.25239	0.03803	-0.13765	-0.22634
C14orf17	-0.024674029	-0.058418791	0.034817	0.006625	-0.06769	0.022076
C14orf18	-0.030888961	-0.051918381	-0.09557	0.03025	-0.16363	-0.05959
C14orf18	-0.008756236	0.062568642	0.108533	0.083819	-0.23294	-0.1499
C14orf18	-0.028003151	-0.031359307	0.041724	-0.04777	0.076312	-0.1728
C14orf2	0.064995347	-0.015533031	0.023879	0.065799	-0.1599	0.378401
C14orf23	-0.030172842	-0.025897588	-0.01352	0.01271	-0.04455	-0.06352
C14orf28	-0.006589139	0.070794136	0.182753	-0.0021	0.025007	-0.22041
C14orf37	-0.050000093	0.020420765	-0.09242	-0.01354	0.055298	0.064955
C14orf39	-0.123541707	-0.075557911	-0.01909	-0.00199	0.055812	-0.05014
C14orf64	-0.006574659	-0.12163502	0.493641	-0.08312	0.106384	-0.23327
C14orf79	-0.016642268	-0.095903294	-0.03633	-0.01948	-0.06765	-0.0382
C14orf80	-0.001211596	-0.024946506	-0.13492	0.061845	-0.24515	0.083644
C14orf93	-0.004203714	-0.016741875	0.033565	0.006617	0.010953	0.036108
C15orf26	-0.044233174	-0.011729702	-0.0274	-0.1609	0.484174	0.023516
C15orf27	-0.039350829	-0.025604549	0.063913	0.034518	-0.07548	-0.01348
C15orf32	-0.05613261	-0.032574971	-0.0378	-0.00771	0.041214	0.02728
C15orf37	0.000606283	-0.122987449	-0.16687	-0.04451	0.089216	0.352115
C15orf39	0.037534898	-0.006138957	-0.35963	0.004623	0.091747	0.300378
C15orf40	0.00157219	0.040170223	0.249115	0.012109	-0.01454	-0.12682
C15orf41	-0.010330612	0.0294093	0.038577	0.047687	-0.0802	-0.11726
C15orf43	-0.078664486	-0.069646047	0.076229	-0.00891	0.049084	-0.03525
C15orf45	-0.030557342	0.027281359	-0.02526	0.005624	0.002555	-0.13998
C15orf48	-0.004476175	-0.015403743	-0.05007	0.004231	-0.03771	0.01667

C15orf49	-0.033500633	-0.040596968	-0.08168	-0.00961	0.010115	-0.04092
C15orf52	-0.014552778	-0.083716945	-0.13711	-0.08631	0.257089	0.071464
C15orf54	-0.021224892	-0.040647065	0.016225	-0.14382	0.420308	-0.028
C15orf56	-0.039036614	0.034031442	-0.11803	-0.01063	0.057974	-0.00033
C15orf57	-0.086068573	-0.045037986	-0.02497	-0.08482	-0.00241	-0.17617
C15orf59	-0.04545766	-0.083327214	-0.14277	0.01585	-0.04422	0.042442
C15orf61	0.024857029	0.048824514	0.14857	0.006199	-0.00458	0.101741
C15orf62	-0.0521423	-0.037576281	-0.01693	-0.02899	0.095457	0.095717
C15orf65	-0.029775166	-0.029724224	-0.1552	0.007724	-0.00862	0.26629
C16orf13	-0.03600764	-0.141065793	0.201866	-0.02222	-0.16394	0.055875
C16orf3	-0.031410797	-0.050492995	0.131266	0.036389	-0.08045	0.086658
C16orf45	-0.02191805	-0.059008151	0.136513	0.013221	-0.14146	-0.17248
C16orf46	-0.030671818	-0.013373744	0.014433	0.048603	-0.15276	-0.07574
C16orf54	0.017562992	0.009501974	0.020894	-0.02516	0.072272	-0.06789
C16orf58	0.000663019	-0.039666215	0.191549	0.069828	-0.1856	-0.00063
C16orf59	-0.020865699	-0.031190308	0.002151	0.064078	-0.27916	-0.07851
C16orf62	0.016306085	-0.02892956	-0.07461	-0.02057	0.033611	0.277489
C16orf70	0.011147236	-0.027129503	-0.21691	-0.04168	0.079921	0.453054
C16orf71	-0.032320573	-0.092735077	-0.01761	0.006675	-0.04172	-0.02548
C16orf72	0.022543269	0.069860611	-0.15253	-0.00387	-0.06355	0.017474
C16orf74	-0.009465934	-0.153879642	0.027318	-0.05723	0.148014	0.04182
C16orf78	-0.039067626	-0.00206102	0.062229	0.006202	0.000297	-0.14801
C16orf80	0.053928859	0.105163061	0.230175	0.095827	-0.28574	-0.25394
C16orf82	-0.032609445	0.032993027	-0.12118	-0.0071	0.017117	0.088238
C16orf86	-0.011125664	-0.174781403	0.08248	-0.03789	-0.00143	-0.02848
C16orf87	-0.005608234	0.020932744	-0.13074	0.04766	-0.12499	0.30538
C16orf89	-0.033664586	-0.005280301	-0.03514	0.045745	-0.11741	0.043279
C16orf91	0.025335448	0.035807154	0.028061	0.010962	-0.0931	0.043599
C16orf92	-0.032109254	-0.061960594	-0.03688	0.023051	-0.04518	-0.05159
C16orf93	-0.010186866	-0.110757482	-0.23659	-0.02501	0.070123	0.051484
C16orf95	-0.075484686	-0.044544524	-0.04071	0.303896	-0.04695	-0.07675
C17orf10(	-0.019992737	0.051392334	-0.01574	-0.03436	0.119215	-0.07206
C17orf10(	-0.056609041	-0.104550608	0.080197	-0.02415	-0.05729	-0.02341
C17orf10!	-0.054286578	-0.020326348	-0.2178	-0.00926	0.023079	0.088451
C17orf47	-0.038537398	-0.035443675	0.032602	0.013292	-0.02863	-0.06159
C17orf50	-0.026373459	-0.085621863	0.029797	-0.01143	0.014968	-0.08305
C17orf51	-0.036518614	-0.0599188	0.014341	-0.00556	-0.01491	0.013447
C17orf53	-0.028938846	-0.083517493	-0.1217	0.101679	-0.33632	0.001733
C17orf58	0.002903734	0.100486152	-0.13127	0.078667	-0.26456	0.104542
C17orf59	-0.002083958	0.019073759	-0.00682	-0.02526	0.045034	0.055513
C17orf62	0.034220552	0.008584956	-0.2312	0.000498	0.002243	0.091785
C17orf64	-0.032756237	0.015932205	-0.1441	-0.04225	0.148055	-0.09092
C17orf67	-0.00512894	-0.079018975	0.279915	0.017858	-0.0699	-0.18666
C17orf70	-0.002445133	-0.111806566	-0.06464	-0.03111	-0.0368	-0.0705
C17orf74	-0.05490986	-0.017742908	-0.08354	-0.01057	0.007441	0.052854
C17orf75	-0.000580968	0.079244234	0.054904	0.09495	-0.24308	0.007906
C17orf77	-0.069576975	-0.050870419	0.085101	-0.00584	0.001238	-0.06114
C17orf78	-0.043268763	-0.064100172	-0.01712	0.002661	0.026378	0.028351
C17orf80	-0.016170718	0.045698106	-0.00711	0.029471	-0.06923	-0.08495
C17orf82	-0.014553227	-0.079231048	-0.00357	-0.01972	-0.06267	-0.09071
C17orf85	-0.002729334	0.098101517	-0.21474	-0.04347	-0.10719	0.038782

C17orf89	0.030423869	0.02859611	0.142716	0.09893	-0.26155	0.044624
C17orf96	-0.007089629	-0.017429252	0.026363	0.022052	-0.10387	-0.06164
C17orf97	-0.01378198	-0.024130914	0.012827	0.043904	-0.1367	0.113645
C17orf98	-0.043123452	0.049314546	-0.12053	-0.0111	0.028465	0.08731
C17orf99	-0.019900614	-0.030610506	0.050664	0.006491	-0.01121	-0.03644
C18orf12	-0.033817668	-0.069151436	-0.0116	0.021914	-0.05294	-0.15583
C18orf15	-0.035861165	-0.121865575	0.026883	0.011992	-0.00834	0.042667
C18orf21	0.019721611	0.027826945	0.039201	0.009405	-0.04225	0.23128
C18orf25	-0.024001424	-0.03565145	-0.0482	0.019324	-0.13868	-0.11026
C18orf42	-0.049561487	0.002544178	0.026541	0.013529	-0.02771	0.072375
C18orf54	-0.041790844	0.043336926	0.016644	0.132694	-0.295	0.021501
C18orf61	-0.040502756	-0.062557024	-0.03265	0.048253	-0.13847	-0.13265
C18orf8	-0.001914452	0.028047782	-0.0993	-0.04647	0.126	-0.10787
C19orf10	0.039766055	0.072571195	-0.0077	0.039349	-0.11498	0.333384
C19orf12	0.017355313	0.211868003	0.248209	0.023864	-0.03924	-0.2422
C19orf18	-0.026589928	-0.051361916	0.001412	-0.01176	0.072496	-0.14924
C19orf24	0.024305912	-0.0797872	0.09964	0.059499	-0.17577	0.032397
C19orf25	0.001924317	0.01731709	-0.11478	-0.03045	-0.04617	-0.02593
C19orf26	-0.007858877	-0.101091207	-0.00476	0.028319	-0.02355	-0.01041
C19orf33	-0.00724654	-0.045932827	-0.10595	-0.14005	0.403782	0.08821
C19orf40	-0.000354397	-0.063537499	-0.05025	0.076353	-0.26875	0.050293
C19orf43	0.093124683	0.122428227	0.072341	0.020441	-0.0384	-0.07992
C19orf44	-0.042945082	-0.073309234	-0.00936	0.020105	-0.05471	-0.03562
C19orf45	-0.035848762	-0.055658468	-0.18322	-0.0011	0.000688	0.188892
C19orf47	-0.028663805	-0.108964643	0.06663	0.013455	-0.11631	-0.05329
C19orf52	0.017093894	0.081426491	0.126955	0.045285	-0.0998	0.063467
C19orf53	0.060383402	-0.002340264	0.332404	0.072939	-0.20744	-0.04095
C19orf54	0.0216475	-0.059772815	-0.15379	0.029763	-0.06691	0.347671
C19orf57	-0.01343011	-0.065599801	0.126994	-0.00042	-0.10483	-0.16384
C19orf60	0.033367281	-0.024674722	0.205502	0.042744	-0.11579	0.084507
C19orf66	0.04645175	0.139597247	0.270941	-0.02112	0.061448	-0.09083
C19orf68	-0.007977315	-0.086355487	-0.00623	0.017988	-0.10602	-0.04112
C19orf70	0.029998569	0.030699748	0.194676	0.062217	-0.15525	0.209035
C19orf73	-0.028381507	-0.068101307	0.11833	0.022758	-0.02973	0.129718
C19orf80	-0.032942255	-0.106934111	-0.00713	0.038064	-0.07697	0.03006
C19orf81	-0.026933261	-0.030647859	-0.01251	0.020213	-0.03604	0.040169
C19orf82	-0.01359355	0.015630374	0.118971	0.118192	-0.32669	-0.02047
C19orf83	-0.016320243	0.078473263	0.069557	0.084662	-0.27064	-0.0344
C19orf84	-0.013143592	-0.013791817	0.009947	-0.00441	-0.07085	-0.05797
C1D	0.06192303	0.125095877	-0.09601	0.051889	-0.15387	0.066634
C1GALT1	0.024966829	0.093655566	0.01674	-0.09102	0.077587	-0.12487
C1GALT1C:	0.019102976	0.122043524	-0.17829	0.012557	-0.0322	0.053307
C1orf100	-0.026206719	0.009794732	0.015015	0.017428	-0.04175	0.103194
C1orf101	-0.05038856	-0.045621609	0.191996	-0.02837	0.081496	-0.10632
C1orf105	-0.021463421	-0.035003647	-0.02778	-0.00071	-0.00311	0.012791
C1orf106	-0.017277645	-0.015374662	0.064699	-0.01093	0.010832	-0.03813
C1orf109	0.001792072	0.03043016	0.123638	0.039646	-0.08516	-0.10221
C1orf110	-0.075138193	0.003203422	-0.00058	0.01455	-0.04625	-0.08765
C1orf111	-0.027384364	-0.017448412	-0.03068	0.003872	-0.00297	-0.07759
C1orf112	-0.010293376	0.045368256	-0.06118	0.151047	-0.37863	0.081032
C1orf115	-0.033007164	-0.049256033	-0.05381	-0.0731	0.218042	0.106694

C1orf116	-0.024881563	-0.029268578	-0.08642	-0.024	0.110799	0.014753
C1orf122	0.058768061	0.05954532	-0.01392	0.075569	-0.22677	0.125407
C1orf123	0.046730058	0.053339566	0.205961	0.035966	-0.12181	0.03012
C1orf127	-0.015991866	-0.071443003	-0.01516	-0.02051	-0.0321	-0.01774
C1orf131	0.044498552	0.118276687	0.089342	0.03503	-0.11556	0.045853
C1orf145	-0.052091889	-0.070652175	0.148312	-0.0408	0.140024	-0.18865
C1orf158	-0.040204075	-0.062022538	-0.05503	0.025262	-0.05663	-0.01047
C1orf159	-0.022462011	-0.050803368	0.026278	-0.01011	-0.01409	0.060404
C1orf162	0.02068609	0.024626975	-0.14271	-0.12425	0.363207	0.216359
C1orf167	-0.034254635	-0.075918304	0.013463	0.006446	-0.05347	-0.07342
C1orf168	-0.068557564	-0.146577342	-0.0296	0.006566	-0.01064	0.019549
C1orf174	0.023282139	0.244481039	0.011347	0.029868	-0.07852	0.029477
C1orf177	-0.019841702	0.262699565	0.108215	0.025176	-0.0399	-0.12759
C1orf180	-0.040408529	-0.044166293	0.08554	0.005982	0.00471	-0.01547
C1orf192	-0.029929009	-0.020673201	-0.00034	0.049761	-0.10887	-0.00621
C1orf194	-0.028591929	-0.043175708	-0.01147	-0.00831	0.020513	-0.0437
C1orf198	-0.004772721	-0.033305417	0.075293	-0.09215	0.212636	0.026545
C1orf200	-0.009874952	-0.106771947	-0.05096	-0.02033	-0.04998	-0.10566
C1orf204	-0.025301257	-0.004560121	0.167088	-0.01707	0.074267	-0.05755
C1orf21	-0.076905425	0.306348806	-0.01678	-0.02569	-0.02768	-0.11872
C1orf210	-0.039886397	-0.058676269	0.038603	-0.00305	0.028078	-0.06612
C1orf213	-0.011419489	-0.067833058	0.03569	-0.0015	0.002975	0.160744
C1orf216	0.0104556	0.147497194	-0.06907	-0.00551	-0.05387	0.123975
C1orf220	-0.037891559	-0.009519795	-0.08854	0.001999	-0.00897	-0.18326
C1orf226	-0.037013889	-0.024133713	-0.11942	-0.01445	0.073239	0.185844
C1orf228	0.032314803	-0.198871696	0.200043	-0.11544	0.206441	0.233779
C1orf233	-0.015378987	-0.048307313	-0.0828	-0.05067	0.071152	0.129346
C1orf234	-0.061704202	-0.058934523	0.107999	-0.02481	0.057105	-0.0327
C1orf27	0.018421501	0.00212052	-0.16812	0.013004	-0.01803	0.288686
C1orf35	0.017137935	-0.040753064	0.221724	0.048511	-0.18349	-0.19092
C1orf43	0.040623678	0.04214223	-0.07645	0.055467	-0.14721	0.245118
C1orf50	0.036238073	0.008849351	0.064901	0.014093	-0.03482	0.08724
C1orf52	0.037318016	0.081266001	0.113323	0.002426	0.031168	-0.08456
C1orf53	-0.020336494	-0.03420907	-0.15478	0.130068	-0.24857	0.46519
C1orf54	0.013075548	-0.113217191	-0.10775	-0.03637	0.115403	0.276095
C1orf56	-0.040727638	0.092313429	0.026361	-0.02532	0.103406	0.012402
C1orf61	-0.022172722	-0.028026952	0.033901	0.052258	-0.22038	-0.08887
C1orf64	-0.026533191	-0.076127715	0.053372	0.031403	-0.09523	-0.00049
C1orf68	-0.042001188	-0.114607732	0.03933	0.004292	-0.00545	0.025886
C1orf74	-0.021200129	-0.008734712	0.030596	0.125599	-0.32611	-0.02219
C1orf85	0.017409487	0.051582057	-0.06213	-0.02682	0.082117	0.466378
C1orf86	-0.095368646	-0.069646381	-0.02973	0.03005	-0.04213	-0.10078
C1orf87	-0.037814653	-0.027999125	-0.02823	-0.0257	0.078543	-0.0494
C1orf94	-0.028879337	-0.084943146	0.061949	-0.0139	-0.00136	-0.11153
C1orf95	-0.039228635	-0.060741276	-0.10854	0.006272	-0.0495	-0.03755
C1QA	-0.004553642	-0.083759323	-0.13962	-0.04947	0.146137	0.430909
C1QB	0.001533187	-0.063059117	-0.16812	-0.0309	0.105247	0.514778
C1QBP	0.026673757	0.010936917	0.1778	0.070196	-0.17661	0.013937
C1QC	-0.019937815	-0.078592227	-0.07491	-0.01338	0.013358	0.259707
C1QL1	-0.055970284	-0.08947763	-0.12004	-0.01891	0.048267	0.015588
C1QTNF1	-0.035817819	-0.048229608	-0.13486	0.033513	-0.13203	0.042271

C1QTNF1-/	-0.034617031	-0.051702012	-0.04767	-0.00656	0.04834	0.012644
C1QTNF2	-0.032250083	-0.047894622	-0.00316	0.00586	0.01299	-0.06183
C1QTNF3	-0.02102376	0.075905278	0.218419	-0.01058	0.035	-0.23407
C1QTNF4	-0.039507216	-0.067526369	-0.01802	0.015758	-0.02352	0.142206
C1QTNF6	-0.037628522	-0.108517968	0.155527	0.001305	0.010208	-0.03958
C1QTNF7	-0.066993863	-0.079004521	0.017784	0.020505	-0.04902	-0.06785
C1QTNF9B-	-0.024785725	-0.101202512	0.127272	0.024962	-0.07662	-0.16642
C1R	-0.025176837	0.061452807	0.170645	0.005786	0.010083	-0.12592
C1RL	-0.001551948	-0.037764116	-0.09871	-0.02351	0.056681	0.041274
C1RL-AS1	-0.02609851	0.005300582	-0.05027	-0.00757	-0.02896	-0.13519
C1S	-0.049835522	-0.07830262	0.081565	0.010561	-0.00926	-0.0707
C2	-0.022855231	-0.067000377	-0.07934	-0.02071	0.036802	0.364583
C20orf141	-0.020807582	-0.072963707	0.029884	0.00095	-0.07396	-0.03858
C20orf144	-0.0410213	-0.08276915	-0.07047	-0.01765	0.067704	0.280882
C20orf166	-0.017848847	-0.1023591	0.106571	-0.00065	-0.10511	-0.1713
C20orf166	-0.025710999	-0.069178636	-0.02031	-0.02536	-0.0313	-0.02727
C20orf173	-0.037965806	-0.033714444	-0.04068	0.016548	-0.02982	0.047736
C20orf181	-0.031355272	-0.138519111	-0.0684	0.01971	-0.09823	-0.10213
C20orf194	0.012541282	-0.067588653	-0.03123	-0.09965	0.285565	0.410212
C20orf195	-0.022697632	-0.084804557	-0.01596	-0.01009	0.036268	-0.10073
C20orf196	-0.044879244	0.077450334	-0.00259	0.05779	-0.16513	0.087878
C20orf197	-0.006909236	0.091775151	-0.10554	0.015117	-0.01872	0.04406
C20orf202	-0.042666058	-0.01965724	-0.03706	0.057895	-0.15044	0.084123
C20orf203	-0.024455357	-0.03938465	0.032436	0.002279	-0.0749	-0.13107
C20orf26	0.015600268	0.030997495	-0.04078	0.113187	0.059362	-0.06737
C20orf27	0.006855473	-0.103515886	-0.1435	0.014336	-0.04842	0.530969
C20orf62	-0.010932071	-0.034581901	0.085074	0.042653	-0.07291	-0.06809
C20orf78	-0.035594459	0.038687522	0.038897	-0.01413	0.056551	0.034484
C20orf85	-0.043500675	-0.010815878	-0.10191	-0.00687	0.04094	0.0494
C20orf96	-0.027816052	-0.028138509	0.011826	0.014971	-0.0237	0.14585
C21orf119	-0.006067696	0.054554174	-0.0143	0.020131	-0.05338	0.304866
C21orf128	-0.030546775	-0.011527053	-0.04574	-0.01054	0.043387	-0.04273
C21orf15	-0.016509609	0.176583658	0.03751	0.036602	-0.13548	-0.18956
C21orf2	0.015490333	-0.062600259	0.12163	-0.00962	0.056418	-0.0322
C21orf33	0.028363846	0.022725727	0.084508	0.038336	-0.09485	0.176929
C21orf37	-0.04895572	-0.010396234	-0.00522	0.003273	-0.0009	0.008378
C21orf49	-0.036385902	-0.001242183	0.282633	-0.00906	-0.01524	-0.29553
C21orf58	-0.038789661	-0.08689885	-0.02106	0.130189	-0.18772	0.013164
C21orf59	0.025957776	0.059674451	0.103322	0.040529	-0.10404	0.052958
C21orf62	-0.037584069	-0.035636889	-0.03075	-0.00048	-0.05031	-0.03707
C21orf67	-0.021810928	-0.027761392	0.09126	0.008849	-0.09257	-0.19416
C21orf88	-0.051302682	-0.073681049	-0.05708	0.025992	-0.04807	-0.09475
C21orf90	-0.041357618	-0.066920587	-0.08394	0.027406	-0.13552	-0.01106
C21orf91	0.028739452	0.1263354	0.167176	0.033225	-0.12488	-0.14451
C21orf91-	-0.035267721	-0.064768403	-0.05779	-0.00671	0.018645	-0.01753
C22orf15	-0.028617042	-0.052019468	0.018979	-0.01522	-0.04343	-0.1369
C22orf23	-0.029313835	-0.029518078	-0.0336	0.009305	-0.02632	0.077525
C22orf24	-0.032008411	-0.024162144	0.020836	-0.01069	0.009021	-0.00365
C22orf31	-0.03193303	-0.046980039	-0.00863	0.018521	-0.06174	-0.06046
C22orf34	-0.01587698	-0.022442049	-0.04692	-0.01192	0.002063	0.02975
C22orf39	0.044662593	0.070291103	-0.02082	-0.00549	-0.06418	-0.02221

C22orf42	-0.029066102	-0.039387295	-0.03372	-0.00629	0.015691	0.001584
C22orf46	-0.020684078	-0.041172069	0.143675	0.06653	-0.15629	-0.11653
C2CD2	0.005110102	-0.044329155	0.042132	-0.06937	0.196502	0.197877
C2CD2L	0.005508676	-0.128529641	0.038156	-0.02063	0.027833	0.359315
C2CD3	0.019484643	0.026867116	0.056164	-0.00864	-0.00103	-0.17498
C2CD4A	-0.022701526	-0.047215688	-0.04233	-0.01092	0.033333	-0.00433
C2CD4B	-0.029359135	-0.066308657	-0.10489	0.015643	-0.0758	0.011009
C2CD4C	-0.015796869	-0.121214723	-0.05751	0.004176	-0.09666	-0.11007
C2CD5	0.043316645	0.054892613	0.02522	-0.01021	0.032951	-0.02848
C2orf15	-0.044939517	-0.020376798	0.135983	-0.04969	0.125815	-0.03954
C2orf16	-0.022129873	-0.070837251	0.042083	0.020452	-0.09739	-0.0386
C2orf27A	-0.022007635	-0.043186433	0.088292	-0.0118	-0.01684	-0.09963
C2orf40	-0.010750547	-0.119275892	0.628618	-0.08331	0.251498	-0.2361
C2orf42	0.019688482	0.113210174	0.143469	-0.03113	0.044708	-0.23672
C2orf43	-0.000854617	0.030940503	0.128994	0.047386	-0.10895	-0.20165
C2orf44	-0.006085328	0.121610379	0.12237	0.086439	-0.25527	-0.28701
C2orf47	0.035915017	0.046366561	0.002494	0.103883	-0.30204	0.198627
C2orf49	0.070442939	0.180855308	0.005126	-0.03435	-0.10941	0.112787
C2orf50	-0.032027547	-0.018302422	-0.0888	-0.00269	0.021218	0.065355
C2orf54	-0.03519539	-0.046753722	0.010925	0.00892	-0.01789	-0.19582
C2orf57	-0.025547674	-0.050827401	-0.17541	-0.00968	-0.00686	0.007054
C2orf61	-0.026730375	-0.005026001	0.111465	0.025272	-0.06514	-0.02839
C2orf68	0.002220198	0.004941807	0.019237	-0.07273	0.212045	-0.03005
C2orf69	0.056940397	0.009913242	0.11042	0.065253	-0.1616	-0.04794
C2orf70	-0.033983676	-0.080115489	-0.02866	-0.0184	0.054533	0.116461
C2orf71	-0.031562538	-0.07568968	-0.0406	0.054831	-0.14887	0.072337
C2orf72	-0.058346132	-0.084605282	0.02148	-0.00806	-0.06741	-0.00029
C2orf73	-0.034934455	-0.004481023	-0.10536	-0.00898	0.044089	-0.11624
C2orf76	0.012141541	0.078227652	-0.17902	0.033378	-0.0927	0.342613
C2orf80	-0.049024374	-0.072177084	-0.053	0.033506	-0.07185	-0.02413
C2orf81	-0.002950413	-0.161009089	0.004688	-0.02268	-0.03669	-0.21262
C2orf82	-0.041994922	-0.107291436	-0.0963	0.010247	-0.09257	0.023572
C2orf83	-0.038990163	-0.041803142	0.011439	-0.01571	0.064842	0.005386
C2orf88	-0.006702336	-0.056808427	-0.03122	-0.25695	0.722342	0.049036
C3	-0.003574149	-0.036144495	-0.13312	0.010929	-0.05983	0.243389
C3AR1	0.019485258	0.049768714	-0.2028	-0.04552	0.119135	0.416601
C3orf14	-0.001336052	-0.07004414	-0.07587	-0.05887	0.168915	0.302611
C3orf17	0.031335537	0.092071916	0.0951	0.026605	-0.04209	-0.07903
C3orf18	0.001847757	0.017992156	0.31523	0.046044	-0.18378	-0.32188
C3orf20	-0.034103391	-0.085013062	-0.01321	-0.00436	0.009001	0.039605
C3orf22	-0.034046159	-0.037084851	-0.02959	0.021732	-0.0408	0.09322
C3orf27	-0.026514874	-0.086583524	-0.00498	0.026835	-0.09597	-0.02639
C3orf30	-0.041363089	-0.021256543	0.00244	-0.01904	0.075893	-0.03031
C3orf33	-0.038095447	0.012541427	0.120449	-0.03831	0.104238	-0.02136
C3orf35	-0.031342565	-0.015330014	0.040079	-0.03579	0.130477	-0.13753
C3orf36	-0.034650277	-0.042016627	0.131963	0.024688	-0.03685	-0.06741
C3orf38	0.040643497	0.065320507	-0.14544	0.015393	0.007518	0.265066
C3orf43	-0.03814824	-0.036277684	0.115736	0.014296	-0.02208	-0.15521
C3orf49	-0.036644822	-0.023372974	0.04072	0.043907	-0.12437	-0.02123
C3orf52	-0.019338017	0.014543047	-0.02879	-0.00134	0.007685	-0.04875
C3orf55	-0.061268729	-0.061077713	-0.0193	0.010477	0.004902	-0.06331

C3orf56	-0.05415172	-0.018787022	-0.01973	0.009144	-0.04502	-0.04946
C3orf58	-0.028307785	-0.032913043	0.035589	0.028046	-0.07628	0.029283
C3orf62	-0.024812354	-0.031103766	0.016531	-0.0007	0.010907	0.078738
C3orf65	-0.072087144	-0.000136969	-0.08778	-0.00034	0.058277	-0.108
C3orf67	-0.060188592	-0.053887456	-0.00952	-0.01649	0.038451	0.021982
C3orf70	-0.033678727	-0.009237045	-0.03251	-0.02238	0.017035	-0.03987
C3orf79	-0.068057711	-0.043729354	-0.10123	0.00408	-0.01736	0.004374
C3orf80	-0.081097452	-0.051062852	-0.03027	-0.00748	0.048915	-0.09927
C3P1	-0.03220829	-0.105453669	-0.10737	0.00284	-0.06248	-0.05738
C4BPA	-0.03146072	-0.066193219	-0.11997	-0.01268	-0.03437	-0.16209
C4BPB	-0.028247584	-0.097217455	-0.05725	0.0276	-0.08169	-0.12386
C4orf17	-0.038106112	-0.076041849	0.160934	0.037981	-0.09926	-0.06711
C4orf19	-0.055600124	-0.082703654	0.132208	0.007538	0.002702	-0.02912
C4orf22	-0.075594254	-0.085747053	0.045939	-0.04187	0.093875	0.011438
C4orf26	-0.022626248	-0.023955343	0.01167	0.170707	-0.21855	0.022189
C4orf27	0.011663466	0.022273475	0.058982	0.04072	-0.12529	-0.03551
C4orf29	0.048640683	-0.008420468	0.066717	0.02464	-0.04478	0.067141
C4orf3	0.081598268	0.108904538	0.048338	-0.08962	0.034249	-0.02568
C4orf32	0.004805698	-0.094759092	0.013776	-0.01412	0.049493	0.190901
C4orf33	-0.069829719	-0.024674454	-0.07636	-0.01343	-0.0006	0.042826
C4orf36	-0.055652996	-0.030258675	0.003459	-0.03616	0.099736	-0.05954
C4orf45	-0.037844117	-0.059356	0.028313	0.007367	-0.00288	-0.02859
C4orf46	-0.007878226	0.069222919	-0.05885	0.157702	-0.40018	0.020793
C4orf47	-0.098661249	-0.074760946	-0.00274	0.022075	-0.0485	-0.03687
C4orf48	0.022220638	-0.240387082	-0.08452	0.039887	-0.13675	0.437186
C4orf6	-0.04051728	-0.027195156	-0.01758	0.029641	-0.05382	0.045907
C5	-0.013139473	-0.15830744	-0.15489	-0.0624	0.184138	0.254361
C5AR1	0.005417308	-0.087908129	-0.31629	-0.09344	0.25635	0.461818
C5AR2	-0.021490815	-0.110522455	-0.16254	-0.03115	0.084836	0.153517
C5orf15	0.031414977	0.010171983	-0.22874	0.022018	-0.0668	0.261169
C5orf17	-0.019045755	0.01985542	0.056217	-0.00531	1.35E-05	-0.12012
C5orf22	-0.009983114	0.137306068	-0.06664	0.074559	-0.19796	0.040018
C5orf24	0.042041811	0.167253168	-0.06518	0.041659	-0.07876	0.079158
C5orf27	-0.037697781	-0.0744822	0.075128	0.012546	-0.01477	-0.04116
C5orf28	0.0207861	0.026383144	0.279183	-0.0068	-0.02109	-0.18732
C5orf30	-0.010710368	0.016105844	-0.06435	0.014687	-0.13369	-0.0955
C5orf34	-0.006200602	0.019112145	0.052955	0.090782	-0.23121	-0.19294
C5orf42	-0.030236526	0.053196219	0.05407	-0.00473	0.025427	-0.17257
C5orf45	0.010431574	-0.095880633	0.190926	-0.01029	-0.01179	-0.38897
C5orf46	-0.026093128	0.031890608	-0.14399	0.014356	-0.0355	-0.10417
C5orf47	-0.069011621	-0.037656662	-0.05972	-0.0192	0.060748	-0.05755
C5orf49	-0.032107683	-0.001245162	-0.00433	-0.02397	0.088445	-0.08672
C5orf51	0.016646126	-0.010903166	-0.01974	0.037355	-0.12204	-0.08304
C5orf55	-0.019139963	0.015915839	0.133537	0.040169	-0.08566	0.009136
C5orf56	0.019129434	0.152851515	0.035166	-0.03764	0.101355	-0.25783
C5orf58	-0.080084005	-0.018039213	-0.06035	-0.04182	-0.00047	0.04252
C5orf63	-0.020474473	0.0728241	0.142889	-0.03238	0.150542	-0.39451
C5orf64	-0.009324188	-0.055000764	-0.09485	-0.00402	0.034401	-0.12692
C5orf66	-0.007248962	-0.07851577	-0.00536	0.006685	-0.12022	-0.11076
C5orf66-/	-0.040191179	-0.057592558	-0.04574	0.011383	-0.02109	0.098488
C6	-0.028106351	-0.03578447	0.055789	0.036081	-0.093	-0.03239

C6orf1	0.008846265	-0.001812799	-0.23118	0.035204	-0.14636	0.140226
C6orf10	-0.04727966	-0.016436309	0.075027	-0.00642	0.030134	0.010895
C6orf106	0.00231927	0.065770212	0.085684	-0.00259	-0.17997	-0.12203
C6orf118	-0.059821237	-0.0331081	-0.04781	########	0.026361	0.024193
C6orf120	0.031302282	0.038397039	-0.08917	-0.00945	0.056067	0.389972
C6orf123	-0.031367044	-0.049412352	-0.00956	0.02252	-0.05888	0.007175
C6orf132	-0.045327717	-0.080426549	-0.02498	-0.0046	0.037998	-0.03452
C6orf136	0.029870876	0.105107472	0.235217	0.051494	-0.10318	-0.03512
C6orf141	-0.066445186	-0.12930139	0.075511	0.01204	0.007348	-0.10719
C6orf15	-0.016329844	-0.05949524	0.027025	-0.0141	-0.0775	-0.0541
C6orf163	-0.035571415	0.00766697	0.03563	0.001334	0.019556	-0.17293
C6orf164	-0.032524233	-0.035997582	-0.11894	-0.00225	0.028859	-0.12064
C6orf165	-0.024146762	-0.065620229	-0.02165	-0.0068	-0.07184	-0.0894
C6orf195	-0.038090866	-0.082844176	-0.02922	0.013511	-0.0133	0.008788
C6orf201	-0.052624627	0.022824053	0.005768	-0.01305	0.07308	-0.00457
C6orf203	0.019832891	0.094260588	0.05918	0.029183	-0.05518	0.220477
C6orf211	0.02505387	0.045767857	-0.27643	0.032229	-0.0866	0.243206
C6orf223	-0.012647657	-0.085234201	0.04474	0.000847	-0.08131	-0.20489
C6orf226	0.021487198	0.031381178	0.192962	-0.0274	0.070048	0.104326
C6orf25	-0.025497662	0.005899017	-0.03856	-0.14237	0.431231	0.074816
C6orf47	0.040317869	0.036805098	-0.03017	-0.00563	-0.06159	-0.0879
C6orf48	0.043127404	-0.045703016	0.306904	0.013638	-0.0294	-0.01383
C6orf52	-0.039369689	-0.076456952	-0.0046	0.123817	-0.22099	0.027696
C6orf57	0.005711859	-0.010784396	0.003226	0.068257	-0.15838	0.220281
C6orf58	-0.049445645	-0.043927012	-0.02206	0.091263	-0.23729	0.033372
C6orf62	0.032803053	0.086044129	0.026377	0.011875	-0.01647	-0.21819
C6orf89	0.069531414	0.159208955	0.103216	0.080824	-0.1702	0.046811
C6orf99	-0.044458093	-0.010243255	-0.04603	0.034011	-0.08835	0.054525
C7	-0.050993239	-0.071392542	-0.02901	-0.012	0.048297	-0.07499
C7orf13	-0.022874289	-0.091991767	0.002551	0.000121	-0.06016	-0.17275
C7orf26	0.04925792	0.010336579	0.143009	-0.01844	0.016374	-0.11299
C7orf31	0.007418761	-0.0535756	0.020926	-0.03084	0.128368	0.048144
C7orf33	-0.037470928	-0.096774484	-0.10292	0.019914	-0.02896	0.114832
C7orf34	-0.04749579	-0.054267758	-0.10011	0.001183	0.032773	-0.02584
C7orf43	0.033194389	-0.091526403	-0.04344	-0.00403	-0.0291	-0.13039
C7orf49	0.034530859	0.023939802	-0.00801	0.021731	-0.07118	0.209725
C7orf50	-0.003466877	-0.070137494	0.029686	0.059956	0.014975	0.205151
C7orf55	0.017504233	0.01909955	0.121044	0.053331	-0.14187	0.054095
C7orf57	-0.142193185	-0.032933455	0.046221	-0.01994	0.054416	-0.02333
C7orf60	0.021950583	0.034374529	-0.09437	-0.03353	0.105908	0.058528
C7orf61	-0.020724546	-0.040540131	-0.08214	0.062385	-0.21223	-0.03163
C7orf62	-0.024717572	-0.018930482	0.081746	-0.00416	0.015301	-0.07091
C7orf63	-0.026939081	-0.042874015	0.049047	-0.02164	0.039679	-0.15591
C7orf69	-0.038095304	-0.008592606	0.060304	0.009654	-0.00518	-0.08311
C7orf71	-0.038307194	-0.022202169	-0.0046	-0.01781	0.063291	-0.05495
C7orf73	0.015826429	0.008480864	-0.26456	-0.03936	0.113187	0.386138
C8A	-0.046960963	-0.030221368	-0.12416	-0.00902	0.068114	0.098774
C8B	-0.0332693	-0.066297327	0.035569	0.030444	-0.07537	-0.03777
C8G	-0.026145953	0.100639342	-0.09249	0.011118	-0.00086	0.002974
C8orf12	-0.040917242	-0.11868236	0.106848	0.010742	-0.01248	0.050825
C8orf17	-0.034161575	-0.008164562	0.010355	0.01961	-0.03564	0.071054

C8orf22	-0.041651101	-0.009694564	-0.0922	0.023797	-0.04198	-0.06751
C8orf31	-0.022351368	-0.025825233	0.023477	-0.03806	0.127483	0.139686
C8orf33	0.017638411	0.023145032	0.067613	0.05572	-0.15287	0.024291
C8orf34	-0.089861348	-0.018084258	0.032737	-0.00237	0.077606	0.083091
C8orf37	-0.019298827	-0.066989119	0.105131	0.047838	-0.16757	-0.16066
C8orf4	-0.223984193	-0.125533174	0.028685	0.026268	0.128269	-0.12033
C8orf44	-0.017134645	-0.072001523	-0.03175	0.02996	-0.07223	0.039359
C8orf46	-0.013835248	0.092758882	0.16939	-0.04644	0.086755	0.065624
C8orf48	-0.048090878	-0.02479706	-0.06877	0.029098	-0.09038	-0.12413
C8orf49	-0.059818916	-0.031260314	0.006264	0.010995	-0.05076	0.037191
C8orf58	-0.004726804	-0.145051696	0.025788	-0.03756	0.011332	-0.17397
C8orf59	-0.002769741	0.037595374	0.232858	0.064133	-0.07903	-0.02336
C8orf60	0.000713544	0.004471097	-0.2261	0.001875	-0.03122	0.022472
C8orf66	-0.032917557	-0.083913384	0.053053	0.024488	-0.05796	0.00304
C8orf74	-0.041353461	-0.077427473	-0.09794	0.008576	-0.00829	0.06564
C8orf76	0.027849165	0.0763321	-0.1375	0.073134	-0.21042	0.292712
C8orf82	0.018772315	-0.031992706	-0.1137	0.007797	-0.01197	0.281649
C8orf88	-0.023365451	-0.080399973	0.083035	-0.03142	0.093798	-0.26966
C9	-0.037138535	-0.017795524	-0.00279	-0.01369	0.027456	-0.0256
C9orf114	0.014551026	0.026748089	0.130259	-0.00346	-0.04225	-0.05449
C9orf116	-0.049843293	-0.068340524	-0.02384	0.064621	-0.15771	0.074609
C9orf117	-0.018797539	-0.084238784	0.160662	-0.00397	-0.00553	-0.22348
C9orf131	-0.030413692	0.003347373	0.007808	0.008571	-0.0293	-0.07312
C9orf135	-0.026321521	-0.021287845	0.261131	0.025004	-0.04984	-0.19751
C9orf135-	-0.031880417	-0.067996213	0.046703	0.045946	-0.11517	0.035123
C9orf142	0.021196613	0.109577045	0.239876	-0.00602	0.008114	-0.16429
C9orf152	-0.072692174	-0.091044534	0.002175	-0.05022	0.118276	0.007558
C9orf156	0.041115486	-0.085602332	0.020477	0.020381	0.017409	-0.06548
C9orf16	0.028362573	0.005897158	0.171044	0.014679	-0.0593	0.104511
C9orf163	-0.012404105	-0.050262779	-0.0189	-0.02791	-0.04671	-0.17199
C9orf172	-0.018882199	-0.031071213	-0.0037	0.00785	-0.03311	0.016333
C9orf173	-0.03467723	-0.011929647	-0.06564	0.037573	-0.09114	0.19609
C9orf24	-0.032741052	-0.054106018	0.036595	0.036215	-0.0762	0.186602
C9orf3	-0.017847525	0.021734968	-0.05295	-0.05454	0.106651	-0.02615
C9orf37	0.000202397	-0.035556621	0.109671	0.027713	-0.11291	-0.05676
C9orf38	-0.013234228	-0.031927284	0.053085	0.001537	0.006977	0.03452
C9orf40	-0.005308721	0.026532226	-0.05406	0.100001	-0.32789	-0.19966
C9orf41	-0.000768247	0.107073122	0.285023	0.077339	-0.23088	-0.15443
C9orf43	-0.039466766	-0.080000824	0.039574	-0.01839	0.077637	-0.11418
C9orf47	-0.037087316	-0.07744849	-0.12061	-0.07952	0.254359	0.416672
C9orf50	-0.028792806	-0.081131068	-0.02062	-0.00191	0.021535	0.040867
C9orf53	-0.031077056	-0.00180493	0.043059	0.014375	-0.04869	-0.08377
C9orf57	-0.04561245	-0.068043381	-0.08217	0.001338	0.007241	0.007491
C9orf62	-0.035069351	0.02178656	0.043467	0.025455	-0.11946	-0.06813
C9orf64	0.047231018	0.08357685	-0.09772	-0.06577	0.208136	0.158542
C9orf66	-0.018833954	-0.073583836	-0.22178	0.023576	-0.04987	0.301723
C9orf69	0.023832435	0.146884593	0.065005	-0.00935	-0.01779	0.101508
C9orf72	0.035124415	-0.107351531	-0.23362	-0.0223	0.14517	0.496167
C9orf78	0.054405915	0.109655354	0.390372	-0.02022	0.024941	-0.15206
C9orf84	-0.039927862	-0.057231013	0.059221	0.000724	-0.01399	-0.06531
C9orf85	-0.085330266	0.134485447	0.082114	-0.07231	-0.03957	-0.08297

C9orf89	0.019116334	-0.110407569	-0.13342	0.021666	-0.04259	0.429605
C9orf9	-0.03085835	-0.100020826	-0.07966	0.085844	-0.20493	0.031686
C9orf91	0.012621925	-0.096582297	-0.22716	0.008297	-0.03277	0.208892
CA1	-0.023283034	-0.05920489	-0.04444	0.038285	-0.10983	-0.0734
CA10	-0.064636062	-0.037429498	0.04027	0.003008	-0.00128	-0.05904
CA11	-0.017546461	0.066307022	0.167327	0.012091	-0.02575	-0.207
CA12	-0.005844564	-0.09972974	0.088312	0.065343	-0.07877	-0.23053
CA14	-0.02260242	-0.040009276	-0.00043	-0.00094	-0.05762	-0.05377
CA2	0.001655124	-0.050109977	-0.17221	-0.14863	0.402285	0.257862
CA3	-0.068699095	0.004109115	-0.17675	-0.03899	0.116171	-0.02212
CA4	-0.019609629	-0.054749878	-0.16972	-0.00441	-0.02324	-0.11951
CA5A	-0.029543189	-0.013750009	0.059983	-0.00786	0.039683	-0.04669
CA5B	-0.008361678	0.092515619	0.189435	-0.04711	0.122882	-0.04275
CA5BP1	-0.006110753	-0.028885136	-0.16786	0.020035	-0.02363	0.536387
CA6	-0.028413131	-0.156329879	0.643642	-0.03114	0.085542	-0.15584
CA7	-0.021721305	-0.092031551	-0.00987	0.010948	-0.11518	-0.07121
CA8	-0.049820622	-0.057575144	-0.00275	-0.04192	0.01076	-0.05522
CA9	-0.024540885	-0.054014007	0.072726	0.03617	-0.16786	-0.08038
CAAP1	0.020657667	0.082696983	0.095135	0.060758	-0.14348	0.001706
CAB39	0.047937427	0.063948894	-0.05978	0.025513	-0.05669	-0.00306
CAB39L	0.004056823	0.019822018	-0.01078	0.014894	-0.03702	0.037516
CABIN1	0.022610057	-0.03232974	0.164777	0.004297	-0.04383	-0.19188
CABLES1	-0.014884929	0.010294091	-0.03938	0.017654	-0.06715	-0.10093
CABLES2	0.024425819	0.130255468	-0.00332	0.060931	-0.17834	0.100423
CABP1	-0.066751367	-0.038760228	-0.07069	-0.0415	0.028985	0.10135
CABP2	-0.03389911	-0.084053906	-0.01697	0.012851	-0.00611	-0.09372
CABP4	0.006631854	-0.090348849	0.078647	-0.01083	-0.03172	-0.13055
CABP5	-0.022400349	-0.022753871	-0.03666	-0.23316	0.650181	0.029153
CABP7	-0.039174281	-0.028306577	-0.07343	-0.00658	0.056659	0.042766
CABS1	-0.087706708	-0.077178124	0.017291	-0.02448	0.038865	0.023153
CABYR	-0.029749253	-0.017869595	-0.01067	-0.00818	0.024523	-0.15992
CACFD1	0.019368901	-0.042368514	-0.03558	-0.00677	-0.0409	-0.1335
CACHD1	-0.036836544	-0.161924128	0.450764	-0.04603	0.135186	-0.18332
CACNA1B	-0.051973805	-0.129471608	-0.08842	-0.00226	0.047447	0.036646
CACNA1C	-0.009694468	-0.067402791	-0.01749	0.05146	-0.01783	0.016221
CACNA1D	-0.035003986	-0.034244468	-0.06222	0.044051	-0.07774	-0.01344
CACNA1E	0.026365201	0.018395352	-0.10245	0.044323	-0.19812	-0.05728
CACNA1F	-0.030714105	-0.030517932	-0.01121	0.042116	-0.1484	0.035556
CACNA1G	0.020149496	0.038558381	-0.00332	-0.03012	-0.03182	0.068971
CACNA1G-/	-0.029377395	0.002646594	-0.04059	0.020145	-0.04509	0.0427
CACNA1H	-0.028456572	-0.111714101	0.061671	-0.0115	-0.03836	-0.07784
CACNA1I	-0.069519251	-0.158235889	0.220382	0.011714	0.044879	0.064227
CACNA1S	-0.033273497	-0.157234784	-0.03786	-0.00948	0.024756	0.062944
CACNA2D1	-0.031456058	-0.033292455	-0.01175	0.159562	-0.16196	-0.05139
CACNA2D2	0.001723621	0.283474999	0.248045	-0.05949	0.182286	-0.19715
CACNA2D3	0.003207009	-0.086523109	-0.25113	-0.01583	0.115215	0.486057
CACNA2D4	-0.003981321	0.047146748	0.006748	-0.08976	0.27809	0.443223
CACNB1	-0.031654709	-0.08809679	0.122708	0.009447	-0.05704	0.131559
CACNB2	0.033661076	-0.020897756	0.092857	-0.02953	0.012618	0.007883
CACNB3	-0.01116029	-0.012172543	0.022046	-0.00181	-0.01313	0.051025
CACNB4	-0.034393754	-0.121213843	-0.11716	0.03213	-0.08952	0.05779

CACNG1	-0.033579679	-0.11872964	0.1009	0.009688	-0.05607	-0.02314
CACNG2	-0.045638379	-0.04746426	-0.01154	0.036383	-0.08691	-0.01551
CACNG3	-0.03532582	-0.00300734	-0.05045	0.023601	-0.05749	-0.10064
CACNG4	-0.051427974	-0.118569034	0.001108	-0.0008	0.014604	0.064953
CACNG5	-0.055152743	-0.067273675	0.028853	0.009944	-0.01199	-0.0219
CACNG6	-0.009267385	-0.042275352	0.040683	-0.00644	-0.05988	-0.11457
CACNG7	-0.035196824	-0.059052711	0.035615	0.000796	0.02298	0.014428
CACNG8	-0.018890148	-0.09155687	-0.09821	0.013769	0.053813	0.006179
CACTIN	-0.029930097	-0.03510335	0.213729	-0.04314	0.022332	-0.00449
CACUL1	-0.020715624	-0.057998722	-0.10078	-0.0137	0.024334	0.106543
CACYBP	0.048461345	0.064544378	0.107121	0.093976	-0.26694	-0.03776
CAD	-0.000215908	-0.016825057	0.33582	0.066903	-0.18495	-0.21631
CADM1	-0.018817427	-0.031472052	0.032853	0.038137	-0.05314	-0.11892
CADM2	-0.052945059	-0.024264177	-0.01102	-0.05784	0.164757	-0.01956
CADM2-AS	-0.070929693	-0.039957911	-0.02395	-0.01464	0.042312	-0.09958
CADM3	-0.026846979	-0.035223297	-0.00804	0.015816	-0.06951	-0.03568
CADM3-AS	-0.034575454	-0.018105457	-0.05865	0.016213	-0.03379	0.009315
CADM4	-0.001177402	-0.141036035	-0.04888	0.033945	-0.03247	0.143646
CADPS	-0.055980519	-0.025018867	-0.05348	0.022313	-0.03746	0.036525
CADPS2	-0.021315827	-0.071116283	-0.00989	-0.0105	0.019678	-0.08186
CAGE1	-0.110439685	-0.089023191	-0.0004	-0.01171	0.034643	-0.07099
CAHM	-0.015220761	-0.161143249	-0.08102	-0.0547	0.175618	0.307375
CALB1	-0.058790566	-0.089054387	0.038257	-0.01915	0.041757	-0.08136
CALB2	-0.040389358	-0.013521835	-0.04789	0.046105	-0.12476	-0.13915
CALCA	-0.021444425	-0.038404874	-0.04605	0.018345	0.00772	-0.00295
CALCB	-0.023210757	-0.019267447	-0.02269	0.056446	-0.14479	-0.01377
CALCOCO1	0.027492442	-0.002381049	-0.04149	-0.0661	0.15841	-0.11951
CALCOCO2	0.013155392	-0.059512284	-0.21515	-0.05031	0.098712	0.261162
CALCR	-0.070315775	-0.066562489	-0.00999	0.004251	-0.00231	0.103554
CALCRL	-0.048500416	-0.002375006	-0.04651	-0.04403	0.042234	0.048862
CALD1	-0.019553802	-0.098835923	0.068406	-0.10019	0.546338	0.047387
CALHM1	-0.029918752	-0.021648718	0.002421	0.004947	-0.02061	-0.08056
CALHM2	0.019333527	0.08958019	-0.06448	-0.06435	0.175851	0.369638
CALHM3	-0.031146506	-0.052435441	0.005561	0.026241	-0.06852	-0.09566
CALM1	0.024578502	0.058212042	0.206403	-0.03956	0.052997	-0.09998
CALML3	-0.043830755	-0.12144389	-0.09427	-0.0054	0.003291	-0.04418
CALML4	-0.026711695	-0.076857158	-0.21985	-0.02099	0.148386	0.504459
CALML5	-0.028036269	-0.062423135	-0.05617	-0.00945	0.062893	0.235289
CALML6	-0.038684189	0.003870174	0.040168	-0.01368	0.067966	0.16917
CALN1	-0.030516191	-0.044744887	-0.00572	0.01909	-0.03676	-0.01543
CALR	0.000192393	0.08467353	0.107469	0.035516	-0.10501	0.164371
CALR3	-0.039931336	-0.044053368	-0.12589	-0.00342	0.046576	-0.00576
CALU	0.039792571	0.022466827	-0.09981	0.080471	-0.18233	0.046535
CALY	-0.033620831	-0.066251968	-0.06253	0.003399	0.018876	0.04298
CAMK1	-0.000932396	-0.063674417	-0.21431	-0.06524	0.159393	0.33917
CAMK1D	0.009891858	-0.014453866	-0.14772	-0.02358	0.066011	-0.16241
CAMK1G	-0.033214041	-0.067111553	-0.05806	0.033968	-0.08292	-0.01217
CAMK2A	-0.044742576	-0.076840209	0.027535	-0.00949	-0.03869	-0.08626
CAMK2B	-0.031083363	-0.106735748	-0.12813	0.032334	-0.0266	-0.08674
CAMK2D	0.034775812	0.00813542	0.040417	-0.04079	0.025707	0.063449
CAMK2G	-0.000868772	-0.048732853	0.031686	-0.03387	0.07155	-0.10938

CAMK2N1	-0.03020005	-0.023145299	0.097759	-0.08819	0.058523	-0.0235
CAMK2N2	-0.033661935	-0.0574984	-0.01117	0.132783	-0.37445	0.006174
CAMK4	-0.026517808	-0.128640753	0.554968	-0.01507	-0.00123	-0.22205
CAMKK1	-0.011462712	-0.132513857	-0.10199	-0.0193	-0.02478	-0.03214
CAMKK2	0.000419066	-0.065871347	-0.28146	-0.02151	0.031334	0.382306
CAMKMT	-0.020707674	-0.001277021	0.13404	0.035607	-0.0815	0.125171
CAMKV	-0.029167393	-0.105530468	0.094887	-0.00313	-0.04137	-0.03038
CAMLG	0.081742388	0.062954471	0.228068	0.047503	-0.12044	-0.16821
CAMP	-0.006721241	-0.113856809	0.278119	-0.04048	0.079703	-0.10542
CAMSAP1	-0.059314695	-0.117415347	-0.03964	0.03393	-0.02393	0.102006
CAMSAP2	0.020463073	-0.064527778	0.03747	-0.00361	0.071198	0.18432
CAMSAP3	-0.046193819	-0.050463808	-0.03186	0.023177	-0.03724	0.001394
CAMTA1	-0.044307105	-0.097404701	0.086466	-0.14009	0.294167	-0.11664
CAMTA2	0.007580038	-0.055369792	-0.14225	-0.03702	0.028758	0.213029
CAND1	0.025345181	0.048593894	0.084293	0.057674	-0.04813	-0.08991
CAND1.11	-0.038703528	-0.09720156	-0.06627	-0.00807	0.053595	0.024389
CAND2	-0.032998821	-0.055124732	0.198649	-0.02448	0.073616	-0.24547
CANT1	0.041363449	-0.01913581	-0.18737	0.036379	-0.10159	0.151888
CANX	0.045851756	0.106814001	-0.10902	0.046588	-0.15108	0.334836
CAP1	0.057906523	0.066604928	-0.203	0.026061	-0.00923	-0.08392
CAP2	-0.067741916	-0.034389882	0.049235	0.051724	-0.13245	-0.08336
CAPG	0.019042637	-0.148924024	-0.34276	-0.07687	0.19021	0.361775
CAPN1	0.029025922	-0.009071236	-0.11519	-0.05803	0.120691	0.113902
CAPN10	-0.011754849	-0.009368533	0.110857	-0.05032	0.081627	-0.01984
CAPN10-AS	-0.028481767	-0.051207102	0.011182	0.038907	-0.08444	0.013985
CAPN11	-0.045769555	-0.016609024	-0.03544	-0.01701	0.086495	0.261484
CAPN12	0.002194889	0.15012611	0.130473	-0.01137	-0.00766	-0.2605
CAPN13	-0.060527709	-0.06490067	0.032118	-0.00715	-0.0508	-0.06651
CAPN14	-0.045571926	-0.032676936	0.02895	-0.01732	0.039264	-0.13266
CAPN15	-0.007399038	0.093260258	-0.04402	-0.00274	-0.01959	-0.02019
CAPN2	0.025154412	0.125935913	0.161988	0.002046	-0.00023	0.249845
CAPN3	-0.014057193	-0.111234042	-0.1158	-0.08253	0.205308	0.199044
CAPN5	-0.031637034	0.160619277	0.115954	-0.05554	0.145058	-0.18264
CAPN6	-0.077477093	-0.067483396	0.027335	0.180297	-0.20968	-0.00895
CAPN7	0.023943744	0.008434409	0.217939	0.039138	-0.08316	-0.05096
CAPN8	-0.034539087	-0.042679378	-0.07277	-0.00085	0.025929	0.032007
CAPN9	-0.051502471	-0.062523975	-0.06906	0.024668	-0.04963	0.028531
CAPNS1	0.041718075	0.123083749	-0.10609	0.018505	-0.02424	0.409708
CAPNS2	-0.021638646	-0.116263068	-0.09079	-0.00698	-0.01045	0.032021
CAPRIN1	-0.003034513	0.023784996	0.014021	0.082519	-0.26799	0.001017
CAPRIN2	0.009895357	0.069761776	0.219615	-0.03935	0.102813	-0.3396
CAPS	0.030100624	-0.045882332	0.089547	0.03411	0.036433	-0.02671
CAPS2	-0.053488971	0.075949155	0.203745	-0.04422	0.133646	-0.23055
CAPSL	-0.024563267	-0.01324831	0.095008	0.054878	-0.12844	0.004512
CAPZA1	0.031904748	0.033822356	-0.21154	0.036884	-0.08127	0.043922
CAPZA2	0.051794787	-0.008697457	-0.50834	0.015877	-0.0025	0.340274
CAPZA3	-0.059530037	0.02906551	-0.09095	-0.04899	0.100592	0.036425
CAPZB	0.073973674	0.085517569	0.092122	-0.03983	0.056932	-0.05284
CARD10	-0.053160663	-0.056867886	-0.14649	0.032108	-0.08699	0.139188
CARD11	0.017037916	0.164655922	0.120227	0.008883	-0.05094	-0.49102
CARD14	-0.08364665	-0.04553257	0.095013	0.054901	0.002378	-0.08263

CARD16	0.023657015	0.022311351	-0.24344	-0.03892	0.125464	0.400537
CARD18	-0.031673437	-0.007169503	0.006481	0.024741	-0.04808	-0.14174
CARD6	0.019814469	-0.003610471	-0.29663	-0.08007	0.221977	0.364551
CARD8	0.039286849	0.066994438	0.051219	-0.0639	0.04514	-0.01247
CARF	-0.013657522	0.022395603	0.127836	-0.0216	0.077941	-0.09986
CARHSP1	-0.048075885	-0.003272782	0.133977	0.003768	-0.11717	-0.26825
CARKD	0.031552174	-0.001439296	0.20856	-0.00104	0.019386	0.097385
CARM1	0.019818842	-0.059339689	0.044456	0.041736	-0.0883	0.217666
CARNS1	-0.001537504	0.100301925	0.367398	-0.05895	0.173673	-0.36887
CARS	0.011375184	0.048312895	0.093288	0.074579	-0.24175	0.061549
CARS-AS1	-0.041684085	-0.07384071	-0.00315	0.034233	-0.07867	0.025214
CARS2	0.080201507	-0.044848439	-0.10689	0.031865	0.02512	0.2475
CARTPT	-0.037502872	-0.094228935	0.019982	0.018854	-0.05356	-0.00533
CASC1	-0.049498027	-0.012667993	0.008369	-0.02239	0.07693	-0.095
CASC10	-0.038329975	-0.086998433	0.02952	0.051519	-0.13318	0.072333
CASC11	-0.04224659	-0.014260823	0.023174	0.015013	-0.01959	-0.10981
CASC14	-0.022555668	-0.02933936	0.009007	0.009035	-0.00608	0.038382
CASC15	-0.067984684	-0.047051538	-0.04876	-0.10759	0.102807	0.064801
CASC16	-0.037614229	-0.0219807	0.003433	0.002344	0.029659	0.173549
CASC17	-0.022293484	-0.040882825	0.069984	0.016211	-0.05689	-0.10573
CASC18	-0.044005431	-0.058313347	-0.03804	0.001266	0.010428	0.053572
CASC2	-0.059896956	-0.05446016	-0.00977	-0.02687	-0.04065	-0.05601
CASC22	-0.036606301	-0.026736638	-0.06223	-0.00402	0.033169	0.013099
CASC4	-0.013365534	-0.000756073	0.027015	-0.01396	-0.01975	-0.16872
CASC5	-0.018837745	0.008742354	-0.00723	0.17006	-0.3606	-0.03483
CASC6	-0.032247847	-0.022731604	-0.05985	-0.00895	0.043392	-0.17898
CASC7	-0.006490827	0.019372987	-0.01968	0.028944	-0.09568	-0.04969
CASD1	-0.056355112	0.029065148	-0.0571	0.008164	-0.12538	-0.03359
CASK	-0.011392107	-0.022473683	0.385139	-0.00323	-0.1219	-0.05607
CASKIN1	-0.049912886	-0.098682389	-0.0966	0.063035	-0.0773	0.014155
CASKIN2	-0.047268122	-0.095938367	-0.03795	0.026512	-0.05953	0.17634
CASP1	0.034660072	0.023909738	-0.13041	-0.01099	0.098172	0.343125
CASP10	-0.033590807	0.059521479	0.004055	-0.0739	0.004431	0.02006
CASP12	-0.037183861	-0.071040915	-0.09565	-0.02416	0.091585	-0.0184
CASP14	-0.038011373	-0.083478328	-0.09079	-0.00496	0.04275	0.221545
CASP16	-0.034849911	-0.037368912	-0.16368	-0.01208	0.064494	0.015145
CASP2	-0.018001517	-0.030756431	-0.14556	0.064946	-0.24201	-0.08465
CASP3	0.03089124	0.112001286	-0.1167	0.05365	-0.14871	-0.08107
CASP4	0.023920774	0.080382192	-0.12554	-0.0162	0.074273	0.101681
CASP5	-0.009435584	-0.051821893	-0.23745	-0.03373	0.082451	0.28206
CASP6	0.019232755	-0.062707612	0.225763	0.007687	-0.03001	-0.05414
CASP7	0.028937639	0.088825643	0.101816	0.036472	-0.12957	0.226235
CASP8	0.008839728	0.100680559	0.057618	-0.01104	0.032907	-0.13833
CASP8AP2	-0.001196464	0.047222972	0.147534	0.010432	-0.03009	-0.1728
CASP9	0.005786276	0.009990248	-0.1822	0.020976	-0.12967	-0.04381
CASQ1	-0.035273164	-0.091802225	-0.06186	-0.00705	0.038398	0.143536
CASQ2	-0.024391868	0.004112932	-0.01055	0.011064	-0.0592	-0.10973
CASR	-0.024898289	0.005753847	-0.08599	0.024835	-0.11759	-0.04956
CASS4	-0.01340712	-0.062339733	-0.06293	-0.02466	0.055129	-0.17409
CAST	0.051094152	0.180170445	-0.13666	0.005725	0.002951	0.485562
CASZ1	-0.0804626	0.115065855	0.00682	0.026072	-0.06665	-0.20651

CAT	0.023475643	-0.083098241	-0.30941	-0.02191	0.078386	0.005981
CATIP-AS:	-0.009192739	-0.096286384	-0.20783	-0.06978	0.18617	0.470701
CATR1	-0.08993356	8.68E-05	0.000561	-0.00663	0.011839	-0.0169
CATSPER1	-0.004786949	0.137726775	-0.32417	-0.02245	0.076867	0.656697
CATSPER3	-0.026775653	-0.064688918	-0.00281	0.010251	0.000622	-0.0652
CATSPERB	-0.024782038	-0.006453198	0.134151	-0.03455	0.032722	-0.08223
CATSPERD	-0.063013719	-0.068865688	0.082879	-0.0427	0.101926	0.031883
CATSPERG	-0.038306853	-0.09384264	-0.13281	0.025875	-0.00291	0.075441
CATX-1	-0.050895224	-0.083712799	-0.06532	0.036104	-0.07174	0.0853
CAV1	-0.026357179	-0.056531805	0.121471	-0.02744	0.098085	-0.00276
CAV2	-0.046083979	-0.036690826	-0.01862	-0.09516	0.396094	0.078049
CAV3	-0.027120587	-0.038664166	-0.17215	-0.0031	-0.06958	-0.0369
CBFA2T2	-0.041718874	0.044597418	0.125944	-0.05913	0.056777	-0.1408
CBFA2T3	0.000477736	-0.187543135	-0.40453	-0.08559	0.243318	0.174924
CBFB	0.041393663	0.113415839	0.090527	0.098239	-0.3145	-0.21528
CBL	0.017034356	-0.026962127	-0.09945	-0.02486	0.02353	0.065564
CBLB	0.005503246	0.217904208	0.3144	-0.0098	0.022914	-0.33021
CBLC	-0.040257113	-0.047703781	-0.02556	0.002489	0.00144	0.039168
CBLL1	0.015973911	0.152512028	0.001635	0.013705	-0.02028	-0.11341
CBLN1	-0.036230585	-0.073350289	0.019412	0.016521	-0.02155	0.045761
CBLN2	-0.037028743	-0.04171873	-0.08755	0.019525	-0.03035	-0.08097
CBLN3	-0.006690183	-0.086564488	-0.14962	0.023779	-0.08424	-0.13947
CBLN4	-0.05740953	-0.064228124	-0.04326	-0.01321	0.012436	-0.06557
CBR1	0.019286528	0.025259276	-0.01335	-0.09	0.257259	0.392022
CBR3	0.0029825	-0.079370171	0.234623	-0.01272	-0.01279	-0.19378
CBR3-AS1	-0.023874959	-0.030992882	0.061236	-0.00688	-0.03663	-0.03479
CBR4	0.021785241	0.124290161	0.003147	0.003704	-0.03625	-0.06534
CBS	-0.029540562	0.031657321	-0.02139	-0.07028	-0.06386	-0.0513
CBX1	0.055362494	0.082156058	-0.10176	0.039007	-0.08606	0.102649
CBX2	-0.027570547	-0.091277842	0.058625	0.108781	-0.33296	-0.01509
CBX3	0.057728843	0.045616887	0.160359	0.083748	-0.21354	-0.07509
CBX3P2	-0.029545061	-0.036350012	0.062152	-0.02129	0.048871	-0.10521
CBX4	0.029792721	0.0024276	0.078661	-0.02406	0.096429	-0.12359
CBX5	0.013188926	0.057134592	0.153144	0.104458	-0.28388	-0.18645
CBX6	0.063179389	0.107409156	0.048192	0.079962	-0.20213	-0.18948
CBX7	0.018231386	-0.016858427	0.15347	-0.05383	0.132586	-0.31181
CBX8	-0.021054554	-0.017161758	-0.13169	0.041406	-0.09904	0.054962
CBY1	-0.009726739	-0.056066027	0.197538	0.000308	-0.03927	-0.08805
CBY3	-0.029181264	-0.010895437	-0.09315	0.00336	-0.00222	-0.0338
CC2D1A	0.005253421	0.000992577	-0.0452	0.122296	-0.21237	0.149143
CC2D1B	0.005746929	0.122565109	0.097867	-0.01157	0.025079	-0.148
CC2D2A	-0.048903633	-0.073233809	-0.1095	0.026075	0.00764	0.228499
CC2D2B	-0.018261669	-0.103288764	-0.07376	-0.01952	0.011826	0.046369
CCAR1	0.034646935	0.062356066	0.17265	0.011977	-0.11041	-0.0369
CCAR2	-0.008646956	-0.108282042	0.067632	0.015361	-0.04161	-0.18046
CCBE1	-0.082190579	-0.010048334	-0.03008	0.00561	0.033253	-0.03492
CCBL1	-0.040398748	0.036565442	0.179292	-0.00808	-0.01678	-0.32639
CCBL2	0.058239506	0.019761027	0.039076	0.080228	-0.20639	0.170026
CCDC101	0.040408891	-0.039272526	0.20255	0.004479	-0.0824	-0.18683
CCDC102A	-0.000268749	0.199962555	0.14872	0.077212	-0.19514	-0.29621
CCDC102B	-0.028536583	-0.051826192	-0.06906	-0.01005	0.024314	-0.0967

CCDC104	0.017792542	0.015620183	0.377664	0.007318	-0.00734	-0.32352
CCDC105	-0.029721784	-0.065234797	0.015899	0.007711	-0.03446	0.089624
CCDC106	-0.019302959	-0.121964305	-0.10892	-0.04649	0.148272	0.069192
CCDC107	0.029553647	0.109410107	0.067942	-0.00835	0.004439	-0.11527
CCDC108	0.004063026	-0.145334439	-0.15018	0.14058	-0.11651	0.02921
CCDC109B	0.025297658	-0.029675191	0.26031	-0.0043	0.028899	0.154384
CCDC11	-0.041305975	-0.02482639	-0.16807	0.008261	0.029946	-0.10095
CCDC110	-0.064487403	-0.091252284	0.074582	-0.02882	0.070526	0.061662
CCDC112	-0.00751541	-0.084923995	-0.1052	-0.11192	0.317799	0.118422
CCDC113	-0.046937481	-0.065911198	0.005647	-0.01095	0.008343	-0.12864
CCDC114	-0.02591631	-0.014515626	-0.08791	0.009069	6.14E-05	-0.0061
CCDC115	0.04236883	0.052097632	0.044635	-0.02439	0.05214	0.00356
CCDC116	-0.035907722	-0.118173875	-0.00802	0.004304	-0.04324	-0.14567
CCDC117	0.018055024	0.207625141	-0.14999	0.07252	-0.24831	-0.0093
CCDC12	0.055041775	0.126534299	-0.01288	0.008308	-0.02753	0.156419
CCDC120	-0.038619779	0.060535551	0.077692	-0.00453	0.045639	-0.19222
CCDC121	-0.041127771	-0.047963026	-0.07946	-0.04568	0.115277	-0.00217
CCDC122	-0.039268314	-0.048034154	0.050357	-0.01973	0.03637	0.021307
CCDC124	-0.01881863	-0.018147276	-0.03616	0.093286	-0.24175	0.203837
CCDC125	-0.012862059	-0.040440075	-0.17186	0.063029	-0.18912	-0.06038
CCDC126	-0.00234132	0.027383738	-0.21214	-0.0108	0.00459	-0.00488
CCDC127	0.012977202	0.121795706	0.159399	0.028467	-0.08936	-0.11681
CCDC129	-0.049659946	-0.042418829	-0.02203	0.054917	-0.13053	-0.09823
CCDC13	-0.018580066	-0.077053469	-0.05604	-0.02543	0.061041	0.102935
CCDC13-AS	-0.032511498	-0.05247433	-0.03306	0.018315	-0.02935	-0.09525
CCDC130	0.045855057	-0.019989647	0.203602	-0.04139	0.071044	-0.15299
CCDC132	-0.025355362	0.061421616	-0.014	-0.01164	0.050157	0.270852
CCDC134	0.004187575	0.068936435	0.112254	0.048621	-0.10534	0.019017
CCDC135	-0.020309034	-0.037945621	-0.08252	-0.00462	0.006212	0.041901
CCDC136	-0.024866098	-0.017023247	0.078404	0.038422	-0.07291	-0.15816
CCDC137	0.026615371	0.038300642	-0.01359	0.055089	-0.14872	-0.04048
CCDC138	-0.023515747	-0.053336097	0.092613	0.099968	-0.26581	-0.23261
CCDC14	-0.049390042	-0.021548162	-0.06182	0.012325	-0.03245	-0.13442
CCDC140	-0.045142968	-0.039454332	-0.05193	0.020692	-0.03806	0.13698
CCDC141	-0.04603794	-0.144116593	0.212751	-0.03533	0.111814	-0.1884
CCDC142	-0.009845499	0.108105796	0.053452	-0.02828	0.113351	0.073149
CCDC144A	-0.041059881	-0.030421332	-0.05675	-0.01252	0.038706	0.049144
CCDC144CI	-0.02221761	-0.046788558	0.032955	-0.0002	-0.00279	-0.0984
CCDC146	0.00082994	0.028506645	0.031407	0.022433	-0.05318	-0.28217
CCDC147	-0.025606485	-0.045970702	-0.0537	0.106343	-0.23176	-0.0679
CCDC148	-0.053850249	-0.071128886	-0.1229	0.042284	-0.10592	-0.05389
CCDC148-/	-0.046460523	-0.029366818	-0.07904	0.007921	-0.01782	0.071588
CCDC149	-0.025214337	-0.132030633	-0.25442	-0.0419	0.128836	0.713382
CCDC15	-0.015215956	0.129391515	0.150093	0.025654	-0.05012	-0.10854
CCDC150	-0.031165453	-0.013146051	0.002025	0.185594	-0.26279	-0.08268
CCDC151	-0.009307417	-0.116045905	-0.18083	-0.0058	0.037213	0.641396
CCDC152	0.039009	0.070871692	0.130028	-0.00094	0.036063	-0.37171
CCDC153	-0.019271638	-0.091337735	-0.22565	-0.00103	0.021269	0.227433
CCDC154	-0.028131736	-0.05170763	0.158504	0.004733	-0.03728	0.006114
CCDC155	-0.033945206	-0.066526638	-0.0094	-0.00201	0.025885	0.091411
CCDC157	0.006060678	-0.048148602	0.161214	0.019894	-0.0679	0.060414

CCDC158	-0.053051805	-0.12135474	0.042542	0.01303	-0.00365	-0.0186
CCDC159	0.010669014	-0.050536011	-0.20367	-0.05489	0.111931	0.492811
CCDC160	-0.075830578	-0.034477215	-0.10264	0.01287	-0.03764	0.055183
CCDC163P	-0.003870639	-0.050626966	0.1307	0.032611	-0.17962	-0.22603
CCDC167	0.020868938	0.055475721	0.322862	0.091092	-0.29426	-0.27354
CCDC168	-0.095602598	-0.147593824	0.142781	-0.04412	-0.02762	-0.03663
CCDC169	-0.022601149	-0.050536067	-0.0363	0.014998	0.065215	-0.05356
CCDC17	-0.025262554	-0.051728456	-0.15689	-0.02337	0.056234	0.076757
CCDC170	-0.022768291	-0.087254407	-0.15017	-0.0185	0.052667	0.250707
CCDC171	-0.061162515	-0.03951916	0.000529	0.028865	-0.19318	-0.0824
CCDC172	-0.036534504	-0.020215454	-0.07673	-0.00461	0.032053	0.061532
CCDC173	-0.033648303	-0.072795223	-0.04233	-0.01531	0.051331	-0.0526
CCDC174	0.017662682	-0.001427805	0.033272	-0.04605	0.075765	0.012637
CCDC176	-0.021334264	0.001987924	0.201109	-0.01705	0.036046	-0.33724
CCDC177	-0.034956394	-0.032351484	0.074804	-0.00679	0.036454	-0.02766
CCDC178	-0.021659552	0.023338912	0.104243	0.038527	-0.14144	-0.15815
CCDC18	-0.015213922	0.032096607	-0.07333	0.041395	-0.17786	0.148092
CCDC181	-0.029919961	-0.010925074	-0.09765	0.007452	0.041176	-0.09603
CCDC182	-0.031965007	-0.048891953	-0.04202	-0.02581	0.013548	0.036969
CCDC184	-0.034048041	-0.087066719	0.007075	0.003596	-0.01105	0.002871
CCDC185	-0.030576727	-0.031402141	-0.06125	-0.01385	0.066412	0.040976
CCDC186	0.008529928	0.055180669	0.000309	-0.01524	0.045124	-0.21726
CCDC19	-0.017731049	-0.105679879	-0.13023	0.030494	-0.11026	-0.05304
CCDC22	0.020344944	-0.018191851	-0.00607	0.014518	-0.02266	0.366612
CCDC23	0.032363355	-0.089766042	-0.16018	-0.04914	0.093222	0.168829
CCDC24	-0.025674017	-0.13676079	-0.039	0.045257	-0.16256	0.071816
CCDC25	0.030760856	0.031590605	0.24278	0.034876	-0.10531	-0.17354
CCDC26	-0.024939473	-0.081340223	0.025614	########	0.006913	0.008399
CCDC27	-0.025547628	-0.092542174	-0.10815	0.022407	-0.05059	0.07034
CCDC28A	0.0327106	0.036240476	-0.04232	-0.00062	0.013088	0.002832
CCDC28B	-0.00209865	0.255808062	-0.00589	0.088038	-0.21616	-0.12656
CCDC3	-0.022974486	-0.0149918	0.03912	-0.11124	0.211416	-0.05376
CCDC30	-0.058779312	-0.022243077	0.146172	-0.00267	0.013415	0.035985
CCDC33	-0.032940465	-0.043741531	-0.11113	0.002696	-0.07805	0.049663
CCDC34	-0.016898303	0.061276711	0.21615	0.099253	-0.26054	-0.20988
CCDC36	-0.072417192	-0.08561207	0.015428	0.012277	-0.08722	-0.08805
CCDC37	-0.039050156	-0.088028752	-0.1408	0.023554	-0.05735	0.006522
CCDC37-AS	-0.038888218	-0.011635631	-0.08401	0.004404	0.035647	-0.01781
CCDC38	-0.032708236	-0.060507497	-0.14008	0.012731	-0.00721	0.035306
CCDC40	0.003309032	-0.019570061	-0.04153	0.121631	-0.07565	0.021695
CCDC42	-0.037949888	-0.063822716	-0.06185	-0.01698	0.075604	-0.08298
CCDC42B	-0.013501907	-0.066222132	-0.00583	-0.00771	-0.04584	-0.06672
CCDC43	0.029958054	0.097696105	-0.05461	0.051186	-0.13333	0.305968
CCDC47	0.037860736	0.061489769	-0.24621	0.037215	-0.11297	0.15468
CCDC50	0.003725572	-0.059065489	-0.264	-0.00547	0.015675	-0.07317
CCDC51	0.017776426	0.049632052	0.073077	0.093281	-0.25616	0.037514
CCDC53	0.028584464	-0.013741941	0.088078	0.01314	-0.04202	-0.03733
CCDC54	-0.038085073	-0.009019039	0.024124	0.028775	-0.06874	0.000749
CCDC57	-0.05992533	-0.059465684	0.084056	0.026374	-0.16351	-0.13306
CCDC58	0.00458198	0.002450513	0.15493	0.084095	-0.25516	-0.19398
CCDC59	0.060869617	0.008428852	0.117508	0.051409	-0.13729	-0.04103

CCDC6	0.018639624	-0.013774439	-0.06809	0.031237	-0.06175	0.164887
CCDC60	-0.034525338	-0.055982268	-0.05939	-0.00123	-0.01494	-0.03385
CCDC61	-0.026690159	0.021851467	-0.07217	0.008925	-0.07153	0.016207
CCDC62	-0.025344186	-0.067298753	0.01689	-0.0129	0.032142	-0.21279
CCDC63	-0.037818672	-0.111586782	0.102429	0.071008	-0.19101	0.003841
CCDC64	-0.005680527	-0.011971301	0.23411	-0.01397	-0.00973	-0.18795
CCDC64B	-0.032348599	-0.054210888	0.04041	-0.01065	0.024517	0.034204
CCDC65	-0.054289269	0.15322932	0.366062	-0.06539	0.031536	-0.18244
CCDC66	7.18E-06	-0.00595127	0.378161	-0.00412	0.033834	-0.1501
CCDC67	-0.112881033	-0.07364346	0.027631	-0.00096	0.078483	-0.06218
CCDC68	-0.019147833	-0.049698965	-0.00992	0.000914	0.001211	-0.06508
CCDC69	0.027223631	0.067900149	-0.02446	-0.03402	0.105754	0.106999
CCDC7	0.04320377	-0.039886645	0.175541	0.037642	0.048238	-0.23139
CCDC70	-0.036498055	-0.075233249	-0.01545	0.075729	-0.18531	0.096988
CCDC71	-0.003913869	-0.086857446	0.166745	0.000103	-0.01306	-0.03055
CCDC71L	-0.002533905	-0.015638308	-0.06819	0.071138	-0.12985	-0.20313
CCDC77	-0.009405654	0.025042092	-0.07065	0.026503	-0.0767	0.002023
CCDC78	-0.025576094	0.006979642	0.062721	0.053009	-0.11936	-0.08201
CCDC79	-0.027769403	-0.022815429	-0.02389	0.029339	-0.07101	0.003556
CCDC8	-0.045216136	-0.080023969	0.030581	0.005667	-0.03748	0.042175
CCDC80	-0.047404608	-0.073204818	-0.04187	0.145903	-0.0691	-0.02145
CCDC81	-0.036723259	-0.022553043	-0.04937	0.007612	0.005921	-0.03151
CCDC82	0.013378303	0.081339615	-0.03798	0.018272	-0.03906	-0.14686
CCDC83	-0.105116848	-0.001067739	-0.06063	0.003989	-0.01227	0.019501
CCDC84	0.045920638	-0.011013686	0.276125	-0.00066	0.007339	-0.25078
CCDC85A	-0.037338953	-0.101053752	-0.07261	0.211265	-0.20467	-0.03545
CCDC85B	0.018622249	-0.053204079	0.132768	0.068227	-0.06839	0.041334
CCDC85C	-0.018336187	-0.046423401	0.124246	0.029334	-0.10212	-0.0824
CCDC86	0.013701031	-0.095343474	-0.00522	0.060446	-0.17968	0.054946
CCDC87	-0.030400018	-0.054572395	-0.07868	0.022403	-0.0484	0.060221
CCDC88A	0.045358287	-0.129123463	-0.30452	-0.02849	0.167234	0.335689
CCDC88B	0.055911845	0.004930124	-0.10475	0.007163	-0.02862	-0.03552
CCDC88C	0.001477802	0.138271653	0.232792	-0.00675	0.100468	0.13857
CCDC89	-0.065247929	0.000227418	-0.06857	-0.0546	0.159223	-0.021
CCDC9	-0.007303671	-0.079143112	0.022575	-0.0164	-0.07794	-0.0279
CCDC90B	-0.000792623	0.076617415	0.037168	-0.01312	0.041833	-0.16087
CCDC91	0.007840891	-0.002942501	0.189269	-0.05937	0.166692	-0.08032
CCDC92	0.024271831	0.130217324	0.20248	-0.04556	0.128179	-0.16889
CCDC93	0.018173451	0.038014084	-0.07468	0.030177	-0.0526	0.043803
CCDC94	0.017232679	-0.027724822	0.011271	0.025765	-0.14	-0.03142
CCDC96	-0.023387761	-0.029282876	0.026854	-0.06078	0.189153	0.05547
CCDC97	0.018447646	-0.012127956	0.070513	-0.01791	-0.06888	-0.14963
CCER1	-0.04168705	-0.084485071	-0.19206	-0.01549	0.067391	0.02821
CCHCR1	-0.011267239	-0.024803791	0.113765	0.120865	-0.33633	-0.00295
CCIN	-0.029701323	-0.052402208	0.072475	0.001599	0.011147	-0.0502
CCK	-0.040720764	-0.006595084	-0.16172	0.015386	-0.00222	0.043289
CCKAR	-0.043099401	-0.067770761	0.065659	0.007198	-0.06883	-0.09352
CCKBR	-0.043932341	-0.054729028	-0.05625	0.011197	-0.00594	0.079465
CCL1	-0.018082663	-0.01946441	-0.04418	-0.001	-0.06771	-0.02512
CCL11	-0.04070939	-0.092831909	-0.13485	0.003183	0.013371	0.049971
CCL13	-0.001742148	-0.033588325	-0.02091	-0.00987	-0.00738	-0.0319

CCL16	-0.02810973	-0.042749661	0.183227	0.002381	0.004463	-0.09101
CCL17	-0.017186026	-0.050181562	-0.02451	0.071851	-0.24275	-0.06003
CCL18	-0.000384776	0.002855408	-0.01466	0.065663	-0.18215	-0.00013
CCL19	-0.018614992	-0.032193505	0.016831	-0.01227	-0.00801	-0.03977
CCL2	-0.009069188	-0.044082131	-0.10502	0.034274	-0.0968	0.210487
CCL20	-0.008785653	-0.021113482	-0.00579	-0.01798	0.01651	-0.02073
CCL21	-0.031133363	-0.038719325	-0.12727	-0.00312	-0.01173	-0.03582
CCL22	0.006139381	-0.013601665	-0.01424	0.017568	-0.06554	-0.039
CCL23	0.011716649	-0.035604294	-0.06363	0.011132	-0.04867	-0.05104
CCL24	-0.016581895	-0.027688017	-0.09926	0.017672	-0.05487	-0.02636
CCL25	-0.036207523	-0.002196181	-0.00564	0.027032	-0.0654	0.050543
CCL26	-0.012005705	-0.004878899	-0.04443	0.018429	-0.03959	0.040399
CCL27	-0.027463943	-0.032003568	-0.14466	0.027981	-0.05636	-0.11594
CCL28	0.036047387	0.085365992	0.27635	-0.02167	0.030291	-0.36949
CCL4	0.012281801	0.17085039	0.133314	0.013726	-0.0312	-0.24071
CCL5	0.014113705	0.23470178	0.505287	-0.00922	0.000873	-0.28539
CCL7	-0.015625136	-0.008892948	0.000933	0.01846	-0.0265	0.042638
CCL8	-0.016591375	-0.049408904	-0.11874	-0.06912	0.206028	0.280316
CCM2	0.03353152	0.045904424	0.003285	-0.00464	0.01908	-0.093
CCM2L	-0.020145382	-0.05857039	-0.01599	0.00402	-0.09378	-0.0418
CCNA1	-0.011794665	-0.00192148	0.003321	0.011776	-0.0296	0.01088
CCNA2	-0.001373599	0.010501226	0.114458	0.169036	-0.4077	-0.13607
CCNB1	-0.008544094	0.008875128	-0.00982	0.153797	-0.31769	-0.06845
CCNB1IP1	0.023643538	-0.013203259	0.20457	0.02851	-0.07314	-0.19319
CCNB2	0.024983334	0.015690446	0.045841	-0.31242	-0.24506	-0.07458
CCNB3	-0.034384378	0.146123884	-0.12664	-0.00514	0.037531	0.021446
CCNC	0.022462149	0.050081987	0.176088	0.046149	-0.10388	0.071156
CCND1	-0.0180361	-0.02536087	-0.11598	-0.02158	0.073901	-0.05366
CCND2	-0.010799354	0.144136099	0.203117	0.064569	-0.2439	-0.21577
CCND3	0.031296041	0.069262692	0.179124	0.007468	-0.01667	-0.35178
CCNDBP1	0.071386562	0.114648185	-0.13536	0.025439	-0.03268	-0.1967
CCNE1	-0.013406204	-0.105594433	0.122988	0.082197	-0.27681	-0.13886
CCNE2	-0.015226421	0.040569587	0.015698	0.157439	-0.31242	-0.074
CCNF	-0.008970914	0.031209563	0.002547	0.157682	-0.45689	-0.08592
CCNG1	0.0369759	0.026765484	0.246544	0.052306	-0.13617	-0.13581
CCNG2	0.003881498	-0.050266812	-0.21319	-0.02905	0.053661	-0.12636
CCNH	0.050280451	0.050618059	-0.02056	0.089623	-0.22971	0.039936
CCNI	0.133749297	0.038379873	-0.01531	0.000104	-0.21939	-0.4003
CCNJ	-0.048566197	-0.041956313	-0.13484	0.024899	-0.00295	0.37864
CCNJL	-0.010970758	0.12523788	-0.19604	-0.00274	-0.0009	-0.16453
CCNK	0.061679637	0.140455036	-0.06495	0.085156	-0.19534	-0.05647
CCNL1	0.02326026	-0.054846329	0.043984	-0.01078	0.063007	-0.18739
CCNL2	0.025332698	-0.035941511	0.191617	-0.04824	0.114329	-0.235
CCNO	-0.037638637	-0.088843389	-0.00347	0.014231	-0.01501	0.023134
CCNT1	0.03434364	0.118225665	0.174417	0.043751	-0.15142	-0.27037
CCNT2	0.028849966	0.072567346	0.021108	0.002327	0.040973	-0.01862
CCNT2-AS1	-0.019522524	0.016946271	0.000955	-0.03333	0.077735	-0.16671
CCNY	0.019602515	-0.042028901	-0.24836	-0.05408	0.148365	0.44538
CCNYL1	0.008644331	-0.026524183	-0.17492	-0.01228	0.049502	0.246035
CCNYL2	-0.0481547	-0.025437029	-0.04067	0.010127	-0.01854	0.065976
CCP110	-0.001899793	0.032197018	-0.07129	0.038478	-0.15369	-0.0096

CCPG1	-0.000418511	0.017050058	-0.29448	-0.00147	0.00053	0.25693
CCR1	0.018012999	0.006792438	-0.32645	-0.02194	0.063736	0.429138
CCR10	-0.017851012	-0.14926215	0.053761	-0.07532	0.237799	0.048384
CCR2	0.005226458	-0.084411846	-0.07314	-0.05292	0.159858	0.377763
CCR3	-0.004483053	-0.069359129	-0.10135	-0.00878	0.00828	-0.17255
CCR4	-0.009741007	-0.005067877	0.061846	0.041896	-0.11209	-0.12516
CCR5	0.020193178	-0.046453442	0.239134	0.009089	-0.0456	-0.06398
CCR6	-0.00319843	-0.073884216	-0.03396	-0.02927	0.095272	-0.2593
CCR7	0.008565781	-0.098404564	0.371383	-0.00906	0.03373	-0.31973
CCR8	-0.013584414	-0.027840791	0.093463	-0.00271	-0.0009	-0.07362
CCR9	-0.019396768	-0.106554071	0.135516	-0.0762	0.222459	-0.30686
CCRL2	0.002883772	-0.026525921	-0.12411	-0.01658	0.025328	0.340938
CCRN4L	-0.017155673	-0.009041888	-0.03228	0.05571	-0.16433	0.066848
CCS	0.013937362	-0.073332005	-0.0358	-0.04292	0.089985	0.033965
CCSAP	-0.032458348	-0.039215627	-0.02694	-0.00404	-0.00227	0.028
CCSER1	-0.02322793	-0.027221981	-0.03968	0.001046	-0.00013	-0.01317
CCSER2	0.014891007	0.154146508	0.350113	0.015673	-0.06603	-0.28194
CCT2	0.028941326	0.09296514	0.200215	0.055752	-0.14965	-0.05039
CCT4	0.033815603	0.048635398	0.165249	0.063498	-0.22921	0.009182
CCT5	0.045250458	0.073556158	-0.05391	0.084834	-0.21462	0.102098
CCT6A	0.037335317	0.065131429	0.041305	0.070325	-0.17924	0.156849
CCT6B	-0.008158014	-0.002233611	-0.07643	0.107483	-0.25067	0.110348
CCT7	0.046738624	0.065248911	0.177898	0.086557	-0.25174	-0.09079
CCT8	0.027540402	0.054584802	0.145616	0.062411	-0.15909	0.079267
CCT8L2	-0.038493133	-0.07547188	0.15194	0.02152	-0.03424	-0.08593
CCZ1	-0.024798737	0.005144443	-0.06592	-0.00511	-0.03676	-0.12456
CCZ1B	0.007046851	0.047045595	0.082203	-0.05989	0.1803	0.08226
CD101	-0.010494965	-0.129241945	-0.06533	-0.05665	0.150477	0.386586
CD109	-0.003915571	-0.001914069	-0.01146	-0.00084	0.00273	-0.00956
CD14	0.002941337	-0.086223762	-0.32554	-0.10248	0.304137	0.628118
CD151	0.002400514	-0.023780929	-0.06365	-0.01392	-0.03482	0.173336
CD160	0.000109541	0.265979097	0.274169	-0.03418	0.090552	-0.16768
CD163	0.014063636	-0.077647475	-0.24645	-0.09587	0.283553	0.605359
CD163L1	-0.005810769	-0.062243281	-0.00851	0.005074	-0.00868	0.179088
CD164	0.077660659	0.226846927	-0.12601	0.015438	0.002543	-0.14088
CD177	-0.023042899	-0.04712947	-0.05931	-0.01073	-0.01532	-0.04779
CD180	-0.004533547	-0.140589553	-0.28277	-0.09599	0.256791	0.063029
CD19	-0.005368826	-0.08520766	-0.16068	-0.07382	0.197508	0.003818
CD1A	-0.005103411	-0.103013539	-0.10004	-0.04094	0.089498	0.177684
CD1B	-0.023491641	-0.067161645	-0.02849	-0.04198	0.055979	0.073757
CD1C	0.011978552	-0.113183081	-0.23807	-0.06959	0.186716	0.139462
CD1D	0.004611632	-0.093821924	-0.3041	-0.08173	0.255895	0.494722
CD1E	-0.012273602	-0.072210874	-0.11506	-0.04563	0.139569	0.133578
CD2	0.01255817	0.124542746	0.457072	0.006466	-0.02354	-0.28724
CD200	-0.018097461	-0.065867091	-0.07596	-0.0508	0.07292	-0.1615
CD200R1	-0.010119794	-0.057205585	0.106519	-0.05295	0.120527	-0.14818
CD207	-0.014350184	-0.060460376	-0.10079	0.012416	-0.0305	-0.11238
CD209	-0.117404809	-0.06616528	-0.04702	0.00627	-0.01131	-0.07477
CD22	-0.000356759	-0.087383098	-0.14689	-0.06965	0.222671	-0.12581
CD226	0.005391873	0.258636255	0.131908	-0.00829	-0.02002	-0.22896
CD24	-0.014297794	-0.117348642	0.036562	-0.06699	0.175975	-0.25487

CD244	0.003077371	0.203217878	-0.01422	-0.019	0.042371	0.327584
CD247	0.015497033	0.294954084	0.331676	-0.00543	0.00499	-0.29639
CD248	-0.008502122	-0.192988829	0.614648	-0.04484	0.055272	-0.16549
CD27	0.000192827	-0.111552296	0.51992	-0.07582	0.183776	-0.32943
CD274	0.001866637	0.010183562	0.01749	0.013835	-0.07701	-0.12372
CD276	-0.036925656	-0.035331763	-0.0977	-0.01888	0.041625	0.035153
CD28	0.053105012	-0.027805308	0.381061	-0.05169	0.181759	-0.27204
CD2AP	0.01121258	0.002285918	0.135611	0.035865	-0.09531	-0.29331
CD2BP2	0.077703835	0.187149309	-0.18739	0.037778	-0.12873	-0.21523
CD300A	0.005713457	0.168489183	-0.19788	-0.06383	0.173926	0.183654
CD300C	-0.000327067	-0.010871372	-0.3036	-0.08115	0.230271	0.608918
CD300LB	0.003074261	-0.095665974	-0.2485	-0.12757	0.307498	0.330774
CD300LF	0.008013609	-0.116284081	-0.33101	-0.09686	0.270755	0.405848
CD300LG	-0.093281439	-0.048636592	0.068281	-0.06318	-0.06273	-0.00474
CD320	0.005449783	0.100265397	0.239676	0.107533	-0.32752	-0.35947
CD33	0.007446236	-0.109485625	-0.26997	-0.0791	0.227668	0.573363
CD34	-0.031380893	-0.03087696	0.00266	-0.00094	-0.05409	-0.07604
CD36	-0.011108233	-0.131497501	-0.29617	-0.07952	0.232416	0.669013
CD37	0.010324875	-0.075019352	-0.07433	-0.0527	0.140415	0.037872
CD38	0.00918674	0.206553024	-0.12658	-0.01188	-0.03875	-0.1857
CD3D	0.014182687	-0.045885411	0.631901	0.020708	-0.06308	-0.29458
CD3E	0.005742381	-0.004955166	0.580639	0.015571	-0.08167	-0.32504
CD3EAP	-0.010639549	-0.037294069	-0.0195	0.014811	-0.11961	-0.06056
CD3G	0.006750595	-0.020399537	0.623767	-0.00261	0.001627	-0.28943
CD4	0.007604966	-0.109753386	-0.33291	-0.0853	0.244964	0.351435
CD40	-0.002009117	-0.064494192	-0.2025	-0.03684	0.094192	0.069352
CD40LG	-0.006220646	-0.034843395	0.028475	-0.03132	0.018443	-0.15758
CD44	0.008718535	-0.047264122	0.0749	-0.05958	0.176084	0.255506
CD46	0.027368851	-0.01118857	-0.07809	-0.03344	0.082776	-0.02864
CD47	0.007965688	0.173773332	0.158226	-0.05877	0.09232	-0.31117
CD48	0.03987976	0.063207762	0.116535	-0.00279	-0.00947	0.186723
CD5	0.004856022	-0.143135196	0.374354	-0.07768	0.164428	-0.19661
CD52	0.027490954	-0.025114405	0.10747	-0.04348	0.166095	0.061837
CD53	0.030643631	0.061095916	0.04523	-0.05326	0.144696	-0.26077
CD55	0.04788599	-0.076677778	-0.17988	0.025053	-0.02481	0.127448
CD58	0.027191924	0.10667956	-0.21533	0.018305	-0.02945	0.320934
CD59	0.009591065	0.010256342	0.008901	-0.02676	0.021975	-0.39062
CD5L	-0.034177177	-0.042349231	0.008779	0.009082	-0.02427	-0.00371
CD6	0.001556223	-0.077724772	0.367372	-0.03885	0.190999	-0.21038
CD63	0.045634109	0.162717689	-0.21147	0.061639	-0.12624	0.21363
CD69	0.009831701	0.113602554	0.316209	-0.01505	0.031203	-0.47062
CD7	0.004006237	0.229320982	0.270814	-0.08244	0.209602	-0.2594
CD70	-0.005746555	-0.054653031	0.022474	0.117984	-0.40683	-0.08521
CD72	-0.002602905	0.027461901	-0.11603	-0.05206	0.126698	-0.18399
CD74	0.062945977	-0.169486681	-0.30507	-0.00636	-0.07841	0.273616
CD79A	-0.000259306	-0.11563501	-0.00849	-0.0885	0.230259	-0.14912
CD79B	0.004514211	-0.041666118	-0.03202	0.030488	-0.02464	-0.12658
CD80	0.007928199	0.010557127	-0.02279	0.060239	-0.26557	-0.01192
CD81	0.031042455	0.142281103	0.196622	0.034859	-0.08442	-0.21063
CD81-AS1	-0.029076907	-0.019508256	-0.17405	-0.00392	0.036683	0.070736
CD82	0.012283847	-0.080904795	-0.04864	-0.00122	-0.04605	-0.06486

CD83	0.024204335	-0.035898892	-0.13495	0.027765	-0.06789	-0.02233
CD84	-0.035153769	-0.107476444	0.192428	-0.11542	0.304077	0.120681
CD86	0.025393914	-0.112939065	-0.42704	-0.00669	0.052791	0.524286
CD8A	0.005209664	0.106001662	0.701996	-0.10123	0.263116	-0.22198
CD8B	-0.036486486	-0.079053478	0.198255	0.006153	0.005671	0.036556
CD9	-0.031307275	-0.001088536	-0.04033	0.001893	-0.03943	-0.0226
CD93	0.00688378	-0.031662021	-0.37526	-0.06809	0.195996	0.533441
CD96	0.00220936	0.183445426	0.46065	0.020509	-0.08085	-0.35215
CD97	0.032058306	0.104077573	-0.10923	-0.00644	0.037404	0.115925
CD99	0.032899955	0.184230447	0.21234	-0.01586	0.044339	0.170411
CD99L2	-0.007750651	0.053473553	-0.05153	0.073073	-0.18553	0.183282
CD99P1	-0.000870361	-0.063371145	-0.07131	-0.01421	0.010693	0.203951
CDA	-0.003781616	-0.100693202	-0.31781	-0.07049	0.204346	0.534811
CDADC1	0.003208711	0.077390762	0.065332	0.037452	-0.07204	0.03454
CDAN1	0.008571453	0.047664905	0.080555	0.043115	-0.07369	-0.07774
CDC123	0.066477127	0.047254587	-0.20683	0.073911	-0.1984	-0.33677
CDC14A	0.017428962	-0.016188928	0.262501	-0.03935	0.098449	-0.21239
CDC14B	0.027519767	-0.032570987	0.130644	-0.0305	0.002822	-0.25153
CDC14C	-0.107244931	-0.079676988	0.017885	0.013023	0.0573	-0.10757
CDC16	0.061828093	0.024718114	0.161977	0.013254	0.007172	-0.12882
CDC20	-0.01115282	0.010963353	0.050666	0.151816	-0.33178	-0.06379
CDC20B	-0.060915419	-0.13337455	-0.02797	-0.04353	-0.06729	-0.03296
CDC23	0.009695949	0.112685524	0.062792	0.100283	-0.27551	0.035281
CDC25A	0.018627946	-0.009364782	-0.0141	0.005255	-0.54697	0.00415
CDC25B	0.020109005	0.101177993	0.40072	-0.00212	0.002676	-0.30049
CDC25C	-0.047982337	-0.028589525	0.065126	0.190844	-0.57846	-0.08349
CDC26	0.076456448	0.107553726	0.247209	0.056803	-0.1262	-0.20894
CDC27	0.067594148	0.061473451	0.004253	0.193227	-0.46733	0.009704
CDC34	0.007664626	0.03768255	-0.02941	0.017246	-0.11863	0.00555
CDC37	0.035086965	0.044665146	0.06302	0.036471	-0.10309	0.155695
CDC37L1	-0.01696342	0.027410086	0.27463	-0.00369	-0.01726	-0.21285
CDC40	0.035584003	0.057217825	-0.23398	-0.00218	0.037088	0.120392
CDC42	0.113510574	0.127422338	-0.05177	0.020172	-0.06166	0.106841
CDC42-IT	-0.012445606	-0.031648458	0.02202	-0.06387	0.142391	-0.01377
CDC42BPA	-0.043738344	-0.097588078	-0.08626	0.042844	-0.08572	0.01517
CDC42BPB	-0.02614114	-0.123596894	-0.20696	-0.06587	0.211517	0.457039
CDC42BPG	-0.049484927	-0.011467923	-0.04589	0.054741	-0.14283	-0.06087
CDC42EP1	-0.040460935	-0.063940502	-0.03611	-0.02958	0.062524	0.197678
CDC42EP2	-0.009024609	-0.093718754	-0.23425	-0.03915	0.070673	0.268351
CDC42EP3	0.021408664	0.113692161	-0.05564	-0.05637	0.133394	0.39473
CDC42EP4	-0.019744189	-0.10886751	-0.13292	-0.08894	0.247951	0.342329
CDC42EP5	-0.070855404	-0.034982163	-0.00753	-0.01679	0.031858	0.037903
CDC42SE1	0.046393248	0.141525946	0.044625	0.011717	-0.01175	-0.30224
CDC42SE2	0.059313301	0.061190567	0.297204	-0.00094	-0.05178	-0.49409
CDC45	-0.010959081	0.060876568	-0.03821	0.156904	-0.40602	-0.00962
CDC5L	0.036719852	0.020491612	-0.04761	0.043852	-0.08495	-0.09563
CDC6	-0.017943223	0.035837873	-0.02464	0.17325	-0.31135	-0.09909
CDC7	0.005233424	0.103890468	0.140425	0.07736	-0.19647	-0.12575
CDC73	-0.019305265	0.108447081	-0.04634	0.04222	-0.15798	-0.19965
CDCA2	-0.01892345	-0.018276103	0.019531	0.179547	-0.41535	-0.0561
CDCA3	-0.011909323	-0.011980465	0.002991	0.158692	-0.51101	-0.06028

CDCA4	0.020216975	0.005529563	0.1498	0.132723	-0.41972	-0.11803
CDCA5	-0.008922299	0.011294868	-0.00992	0.143653	-0.42231	-0.04854
CDCA7	-0.002300682	0.0123237	0.098074	0.098099	-0.26753	-0.07585
CDCA7L	0.00603379	-0.148536911	-0.02199	0.019452	-0.07581	-0.32368
CDCA8	-0.011969087	0.032499189	-0.03994	0.150292	-0.45453	-0.0825
CDCP1	-0.00994751	-0.057675274	-0.03661	0.019551	-0.08071	-0.05191
CDH1	-0.012957889	-0.099975753	-0.06768	-0.02711	0.08804	-0.12129
CDH10	-0.039274585	-0.071938358	0.097299	0.039481	-0.09727	-0.06063
CDH11	-0.057750163	-0.062037618	-0.02813	0.013411	-0.05741	-0.01951
CDH12	-0.02156435	-0.027031603	0.170738	0.007363	0.00762	-0.19873
CDH13	-0.056267923	-0.070989594	-0.00449	-0.03452	0.108574	0.026412
CDH15	-0.038675593	-0.100309207	-0.15465	0.026356	-0.09359	-0.06174
CDH16	-0.034008548	-0.016267824	-0.03132	-0.01581	0.058365	0.114823
CDH17	-0.011694347	-0.022978301	0.025656	0.073109	-0.19666	-0.02346
CDH18	-0.020578533	-0.018743979	0.061379	0.059503	-0.17295	-0.07025
CDH19	-0.045327777	-0.039649859	0.049612	-0.06449	-0.00259	-0.02626
CDH2	-0.031384857	-0.029315493	-0.00635	0.008993	-0.04051	-0.20876
CDH20	-0.041917469	-0.045077996	-0.11239	0.008671	0.001518	0.144686
CDH22	-0.004875083	-0.054244634	0.012394	-0.02722	-0.0426	-0.08331
CDH24	-0.03335755	-0.049755744	0.005909	-0.00388	-0.07273	-0.03729
CDH26	-0.040184974	-0.010747472	-0.18047	-0.04864	0.164117	0.402593
CDH3	-0.022142197	-0.021183493	0.004792	-0.01233	-0.03682	-0.07102
CDH4	-0.032698117	-0.103879121	0.112118	-0.01138	-0.06492	0.083642
CDH5	-0.038701411	-0.056789953	-0.06419	-0.02185	0.097661	0.025221
CDH6	0.0218498	0.00179332	-0.00027	0.128091	-0.05215	-0.18037
CDH7	-0.107495201	-0.139292794	-0.01968	0.015074	0.00897	-0.01332
CDH8	-0.079308854	-0.044596189	0.06569	-0.06043	0.066032	0.083071
CDH9	-0.053579371	-0.036392471	-0.00897	-0.03158	0.104566	0.055178
CDHR1	-0.041344014	0.160835215	-0.05899	-0.00434	0.035529	-0.16401
CDHR2	-0.039527479	-0.09469159	-0.0678	-0.03048	0.023548	-0.03697
CDHR3	-0.038736057	-0.030189689	-0.03556	-0.03581	0.12574	-0.11046
CDHR5	0.028236117	-0.047676149	-0.18698	0.017603	-0.0126	0.072227
CDIP1	0.015336622	0.09849799	-0.01115	-0.10121	0.208877	-0.01445
CDIPT	0.076198976	0.10543509	-0.01913	0.013639	-0.01278	-0.00295
CDIPT-AS:	-0.032209866	-0.037531984	-0.06054	0.034454	-0.09739	-0.02103
CDK1	-0.008160938	0.007688432	0.05042	0.236545	-0.32004	-0.11458
CDK10	0.031840299	0.016604926	0.141743	0.017003	0.079879	0.047127
CDK12	-0.033045828	0.096612502	0.148682	0.013361	-0.07366	-0.19372
CDK13	-0.042832755	0.081129033	-0.01694	-0.0403	0.029677	-0.12282
CDK14	0.053293538	-0.057612276	-0.35616	-0.07674	0.024472	-0.04112
CDK15	-0.030782823	-0.074229012	0.019467	0.047194	-0.05886	-0.06162
CDK16	0.00952274	-0.051845895	-0.08773	0.041497	-0.26152	0.083413
CDK17	0.025959198	0.259801376	0.135828	0.020789	-0.02913	-0.35357
CDK18	-0.021050715	-0.089224182	-0.01552	0.015861	-0.05747	0.082612
CDK19	0.019527235	0.03402574	-0.24023	-0.01481	-0.00533	0.022599
CDK2	-0.006414219	0.069341983	0.088478	0.136634	-0.36967	-0.22523
CDK20	-0.027559687	-0.05957055	0.233655	-0.03867	0.052645	-0.08261
CDK2AP1	0.039280749	-0.065918764	-0.36618	0.005691	-0.00836	0.376463
CDK2AP2	0.024406595	0.143697481	-0.03787	0.019163	-0.07058	-0.05592
CDK4	0.026415078	0.05760461	0.116168	0.079511	-0.22731	-0.0344
CDK5	0.003564282	0.001996749	-0.08879	0.041729	-0.09476	0.278181

CDK5R1	-0.006036751	0.06707741	-0.01464	0.013037	0.01331	-0.13387
CDK5R2	-0.028322013	-0.00576518	-0.03769	0.005876	0.005654	-0.15389
CDK5RAP1	0.044283912	0.02197573	0.190845	0.046914	-0.13417	-0.06391
CDK5RAP2	0.01555773	0.062781697	0.072778	0.104097	-0.05544	-0.18753
CDK5RAP3	0.074254592	-0.028128031	-0.00695	-0.00614	0.05417	-0.03167
CDK6	-0.035066729	0.009989041	0.007771	0.134792	-0.24118	-0.10882
CDK7	0.040886014	0.070599853	-0.18465	0.083166	-0.24002	-0.07085
CDK8	0.011251554	0.081061777	0.073706	0.053388	-0.21438	0.002326
CDK9	0.010374468	0.097106595	0.081084	-0.03173	0.052016	-0.00767
CDKAL1	-0.010520409	0.076227764	0.208592	0.035205	-0.08196	-0.09356
CDKL1	-0.018280098	0.094038621	0.008443	-0.03209	0.106375	-0.02293
CDKL2	-0.044592325	-0.024266517	-0.00912	0.012235	-0.0071	0.035389
CDKL3	0.03051179	0.072473709	0.115474	0.081088	-0.19237	-0.0923
CDKL5	-0.03419863	-0.099418727	-0.10783	-0.00896	0.054088	0.078731
CDKN1A	0.029754562	-0.058974762	-0.14639	0.055835	-0.14311	0.194843
CDKN1B	0.051531552	0.085244106	0.21522	-0.02325	0.078774	-0.37761
CDKN1C	-0.001492414	-0.081981918	-0.16023	-0.08191	0.236799	0.522857
CDKN2A	-0.007265268	-0.022699086	0.053163	0.101523	-0.70593	0.036122
CDKN2AIP	0.032866358	0.095110006	0.278853	0.005924	-0.03161	-0.31241
CDKN2AIPI	-0.018163101	0.034466129	0.150944	0.115458	-0.29651	-0.06845
CDKN2B	-0.02332447	-0.042319604	-0.07349	0.037243	-0.04193	0.244404
CDKN2B-AS	-0.043527318	-0.07148553	0.034981	0.005101	0.008618	-0.09497
CDKN2C	0.00710416	0.098342379	0.005645	0.052291	-0.17529	-0.02697
CDKN2D	0.00376629	-0.009815516	-0.11792	-0.07279	0.195644	0.013756
CDKN3	-0.007776437	-0.006322234	0.116458	0.13484	-0.28883	-0.02059
CDNF	-0.060173879	-0.019026117	0.212294	-0.04011	0.089349	-0.08545
CDO1	-0.031100207	-0.037428666	0.057896	-0.00433	0.012456	-0.19974
CDON	-0.024469792	-0.057276628	0.189212	0.013192	-0.14483	-0.12979
CDPF1	0.004086194	0.041895316	0.052193	0.008901	-0.04699	-0.038
CDR1	-0.033634355	-0.018956079	-0.11892	-0.02596	0.073087	-0.10858
CDR2L	-0.040883572	-0.026349138	0.072719	-0.01726	0.03726	0.008029
CDRT1	-0.044993318	-0.073257223	0.035289	-0.01188	0.113329	0.076797
CDRT15	-0.028803431	-0.062997626	-0.11346	0.006322	-0.03404	0.077518
CDRT15L2	-0.034125867	-0.061748252	-0.11373	-0.00075	0.033945	0.032906
CDS1	0.015872966	-0.101278868	-0.04817	-0.02163	0.074726	-0.01752
CDS2	0.011115697	0.034660067	-0.26489	0.083775	-0.16489	0.058635
CDSN	-0.039973796	-0.023997657	0.047165	0.015744	-0.04244	-0.06464
CDT1	-0.012826201	0.011651263	-0.02979	0.200652	-0.48012	0.002154
CDV3	0.052731136	-0.096981196	0.002407	0.002103	-0.01752	0.176374
CDX1	-0.009331386	-0.012539824	0.063831	-0.00592	-0.08752	-0.1305
CDX2	-0.024278113	-0.10268439	-0.01312	-0.01191	0.026727	0.02746
CDX4	-0.029757623	-0.072113097	-0.00018	0.011365	-0.01156	0.151864
CDY1	-0.071406587	-0.080210258	-0.03895	-0.05315	0.133915	-0.04583
CDYL	-0.014901299	0.004697703	-0.03728	-0.01831	-0.05928	-0.06334
CDYL2	-0.000653862	-0.106003497	0.045736	0.127163	-0.40628	0.104739
CEACAM1	-0.008431372	-0.080464272	-0.1146	0.051456	-0.14706	-0.20947
CEACAM19	-0.019641846	-0.04178414	0.05504	-0.01543	-0.05328	-0.02585
CEACAM21	-0.004060823	0.025893824	0.195273	-0.00868	0.031374	-0.1674
CEACAM3	-0.003312494	-0.058568141	-0.22273	-0.01036	0.01784	0.065822
CEACAM4	-0.013637172	-0.108947653	-0.17177	-0.02524	0.040913	0.268209
CEACAM5	-0.043008968	-0.082799396	0.036126	0.00617	-0.07386	-0.13183

CEACAM6	-0.01085657	-0.104840003	0.415089	-0.01325	0.04982	-0.20147
CEACAM7	-0.029598273	-0.072220593	-0.03548	0.024806	-0.04102	0.074175
CEACAM8	-0.01044293	-0.06530365	0.431922	-0.04186	0.136121	-0.19446
CEBPA	0.006089388	-0.053773628	-0.20305	-0.0936	0.257362	0.542602
CEBPA-AS	0.006789544	-0.082144253	0.014824	-0.01454	-0.07318	-0.079
CEBPB	0.021914004	0.004695583	-0.25297	-0.0177	0.05323	0.353627
CEBPD	0.008847383	0.03550463	-0.2863	-0.07139	0.200874	0.463061
CEBPE	-0.014276318	-0.092919518	-0.0402	-0.00405	-0.00995	-0.03411
CEBPG	0.029808939	0.054891031	-0.186	0.029353	-0.0713	0.474153
CEBPZ	0.039725311	0.06884991	0.158162	0.043245	-0.11374	-0.0194
CEBPZOS	-0.011027019	-0.014222881	0.100758	0.1114	-0.34678	-0.00634
CECR1	0.030341867	-0.037628653	-0.05888	-0.04508	0.125268	0.428033
CECR2	0.045006238	-0.104925018	-0.10036	0.042106	-0.06129	0.072122
CECR3	-0.039581585	-0.002945076	0.045853	-0.00192	0.030489	-0.04322
CECR5	0.013058062	-0.050769526	0.049355	-0.01526	0.041127	0.348129
CECR5-AS	-0.04220432	0.007323086	0.023905	0.011406	-0.00962	0.148497
CECR6	-0.014633308	-0.085700738	-0.22722	-0.06732	0.154523	0.433982
CECR7	-0.025072581	-0.031472661	-0.01365	-0.00922	0.043939	0.07717
CECR9	-0.044039796	-0.042763344	-0.25003	-0.02362	0.069444	-0.02098
CEL	-0.032533981	-0.032093207	-0.00169	0.049924	-0.11106	-0.00167
CELA1	-0.015361008	-0.107771575	0.240982	-0.03346	0.110307	0.201582
CELA2B	-0.029004657	-0.072085607	-0.02571	0.001625	0.019075	0.087235
CELA3A	-0.034593161	0.022278055	-0.03617	0.017026	-0.03175	0.043727
CELA3B	-0.02803629	-0.042466779	-0.05961	0.013935	-0.06224	-0.10983
CELF1	-0.009696222	-0.069265294	0.008309	-0.04808	0.007836	-0.06974
CELF2	0.053795893	0.0908178	0.062321	0.008848	0.027533	-0.26704
CELF2-AS	-0.021313628	-0.031247875	0.023395	0.01304	-0.0447	-0.03989
CELF3	-0.023829989	-0.095565706	0.025812	-0.00447	-0.05167	0.037847
CELF4	-0.132334555	-0.026402476	-0.00625	-0.07374	-0.06243	0.030935
CELF5	-0.053307863	-0.026905717	0.035773	0.0196	0.021388	-0.10976
CELF6	-0.014172972	-0.013734179	0.076153	-0.06104	0.192854	-0.2539
CELP	-0.028207542	-0.073667162	-0.0715	0.044235	-0.12369	0.059555
CELSR1	0.017301717	-0.001330538	0.031634	0.066135	0.096796	-0.15905
CELSR2	-0.021504656	0.125923358	0.227582	-0.01613	0.045258	0.039977
CELSR3	-0.008224744	-0.006511458	0.132818	0.073595	-0.19631	-0.02556
CELSR3-AS	-0.037471548	-0.085644378	0.117418	0.006973	-0.00194	0.094438
CEMIP	-0.05573139	-0.030355195	-0.02508	0.047127	-0.10705	0.02645
CEMP1	-0.026093999	-0.09864308	-0.01338	0.039996	-0.11191	0.050402
CEND1	-0.011309073	-0.083108056	0.09708	0.018892	-0.17245	-0.13255
CENPA	-0.023228176	-0.012443894	-0.07679	0.108172	-0.37308	0.014212
CENPB	-0.000745258	-0.015903338	-0.02031	0.034168	-0.0664	0.312999
CENPBD1	0.010940544	0.006087122	-0.17413	-0.03131	0.072316	0.350383
CENPBD1P	-0.049325493	-0.038339681	-0.14144	0.037598	0.025529	0.383586
CENPC	0.018440448	0.118589159	0.044609	0.024304	-0.02597	-0.1463
CENPE	-0.01457001	0.002834296	0.043108	0.188048	-0.37079	-0.09307
CENPF	-0.030118732	-0.038942996	-0.04526	0.211802	-0.23953	-0.03927
CENPH	-0.006213672	0.056306254	0.132508	0.126319	-0.35927	-0.23954
CENPI	-0.025095168	-0.024268683	-0.00083	0.160137	-0.36629	-0.04836
CENPJ	0.020709728	0.098074603	0.049678	0.072253	-0.20502	-0.04702
CENPK	-0.000232198	0.0498826	0.223705	0.047585	-0.13034	-0.2561
CENPL	0.002204382	0.103308548	0.081772	0.125607	-0.33033	-0.15121

CENPM	-0.007646041	-0.002610418	0.040758	0.096801	-0.32469	-0.12941
CENPN	-0.02290769	0.045567586	-0.04668	0.22508	-0.43944	-0.04301
CENPO	-0.016817426	0.037951677	-0.06259	0.128281	-0.40399	-0.11793
CENPQ	-0.014571234	0.058141896	0.012349	0.080257	-0.23774	-0.0262
CENPT	-0.015406172	-0.039751514	0.185808	-0.01781	0.055501	0.011766
CENPU	0.028533076	0.019348224	-0.05719	0.071779	-0.39554	-0.07186
CENPV	0.000727609	-0.00964254	0.140073	0.064832	-0.06478	-0.29092
CENPW	0.015663428	-0.035765007	-0.17011	0.122014	-0.32091	0.428527
CEP104	0.001685514	0.046160615	0.038559	-0.01063	0.048499	0.048258
CEP112	-0.005763507	0.014354476	0.085126	-0.01133	0.061047	-0.24609
CEP120	0.014256918	0.045550791	0.172294	0.017442	-0.02692	-0.30422
CEP128	0.035345994	-0.020725094	0.040256	0.080941	-0.2278	-0.15724
CEP131	-0.009047737	-0.080356557	-0.06709	0.050447	-0.13411	-0.05098
CEP135	0.001704525	-0.012151678	-0.02762	-0.00885	-0.00487	-0.08881
CEP152	-0.059278363	-0.024221918	-0.0331	0.101782	-0.21341	0.054618
CEP162	-0.006497457	0.065780042	-0.064	-0.00913	0.050918	0.111911
CEP164	-0.011149101	-0.088787613	0.017823	-0.03221	-0.00837	-0.03062
CEP170B	-0.017442877	-0.04092098	-0.00453	-0.01963	-0.04748	-0.10211
CEP19	0.006407709	-0.021643871	-0.13541	0.039597	-0.10855	-0.09646
CEP192	0.012700584	0.045859371	0.031862	0.02655	-0.04409	-0.0066
CEP250	0.00925556	0.095228351	0.177989	0.002465	-0.01957	-0.19568
CEP290	0.007529574	0.08186067	0.190297	0.011521	-0.00412	-0.16974
CEP350	0.063786145	0.132549215	0.069196	0.015966	-0.02691	-0.1353
CEP41	-0.010323574	0.058288011	0.347913	-0.05141	-0.02956	-0.18228
CEP44	0.005189581	0.059794967	0.028421	0.04349	-0.09239	-0.0436
CEP55	-0.008754058	0.004965226	0.024691	0.093899	-0.20787	-0.03645
CEP57	0.006181726	0.078891606	0.23159	0.032824	-0.10961	-0.13442
CEP57L1	-0.024384503	-0.038014068	-0.00179	0.138112	-0.2857	-0.00038
CEP63	0.01753258	-0.035448993	-0.21962	0.013789	-0.02504	0.058243
CEP68	-0.013017058	-0.127558393	0.344923	-0.06078	0.153723	-0.24365
CEP70	-0.040194518	0.071706844	0.340196	0.054218	0.000299	-0.24382
CEP72	-0.007166123	0.12260901	0.188605	0.043977	-0.13866	-0.24436
CEP76	0.000905307	0.121460567	0.066094	0.090333	-0.22957	0.021583
CEP78	-0.000116965	0.309490441	0.253477	-0.00733	-0.0031	-0.31664
CEP83	-0.021354128	-0.010954075	-0.00498	0.025859	-0.11917	-0.108
CEP83-AS	-0.031360695	0.007715563	0.088961	0.004547	0.020906	0.072126
CEP85	0.002840773	-0.016485203	-0.0006	0.035763	-0.18979	0.087727
CEP85L	0.02066426	0.014966052	0.241808	-0.03197	0.08926	-0.06328
CEP89	0.009666433	0.05357417	-0.1304	0.024197	-0.08674	0.162399
CEP95	-0.085159032	0.072989542	0.210792	-0.02625	0.101079	-0.15358
CEP97	-0.073190849	-0.004384691	0.179466	0.025752	-0.12833	-0.27614
CEPT1	-0.016040184	-0.067446913	-0.13885	-0.04721	0.207439	0.146416
CER1	-0.031216961	-0.022005318	0.026001	0.042061	-0.11833	-0.09268
CERCAM	-0.037616213	0.027657972	-0.05471	0.006403	-0.0758	-0.05257
CERK	0.036231603	0.069399923	0.205245	-0.04273	0.114377	-0.14363
CERS2	0.080533577	0.02015191	-0.22265	0.073353	-0.15851	0.142911
CERS3	-0.028852103	-0.058904837	0.09506	-0.00573	-0.00962	-0.11032
CERS4	-0.008021653	0.128236284	-0.14984	-0.0079	0.036509	-0.10888
CERS5	0.036665342	0.041673957	-0.08388	0.043699	-0.11108	-0.19999
CERS6	0.00904517	-0.050010359	-0.071	0.035851	-0.09501	0.257729
CES1P1	-0.021366775	-0.045000983	-0.08889	0.035567	-0.1058	0.06411

CES2	0.0170775	-0.076928064	0.039399	-0.0209	-0.05521	0.387868
CES3	-0.010685718	-0.036030233	0.089322	0.000457	-0.07381	-0.03288
CES4A	0.000396199	-0.062131937	0.039281	0.045412	-0.14913	0.066639
CES5A	-0.042877189	0.002775823	-0.04459	0.029658	-0.05659	0.014363
CETN1	-0.029897762	-0.02409197	-0.05918	0.002805	-0.03651	-0.0324
CETN2	0.03330241	0.059338134	-0.02992	0.002194	-0.00656	0.139711
CETN3	0.015158219	0.060266028	0.159748	0.046793	-0.13586	-0.06825
CETP	-0.019127923	-0.078308401	-0.21138	-0.06803	0.219529	0.293668
CFB	-0.019737642	-0.039230622	-0.02207	-0.00174	0.004706	0.043099
CFC1	-0.040828209	-0.051005643	0.081468	0.033624	-0.11004	0.013065
CFC1B	-0.029850495	-0.070120307	0.006625	0.018504	-0.05541	-0.03133
CFD	0.004346924	-0.120719876	-0.23291	-0.08444	0.225624	0.462281
CFDP1	0.015276782	0.015312348	-0.00841	0.012989	-0.02431	0.068793
CFH	-0.009496112	-0.008664633	0.238379	0.161237	-0.32804	-0.11045
CFHR2	-0.032073251	-0.0689354	0.110354	0.000894	0.010657	-0.08935
CFHR3	-0.080716046	-0.070600363	0.108702	0.008126	-0.01876	0.042096
CFHR4	-0.032302939	-0.023122663	0.050047	0.011294	-0.01481	0.009026
CFHR5	-0.066531164	-0.038690707	0.046261	0.026479	-0.07495	-0.01151
CFI	-0.059687931	0.035472522	-0.00872	0.02199	-0.03611	-0.02539
CFL1	0.021082229	0.103425256	0.104442	0.040651	-0.07794	0.06528
CFL2	0.034264525	0.092193151	0.081534	0.020338	-0.07397	-0.09677
CFLAR	0.039512862	0.106748203	-0.17188	-0.0016	0.017381	0.054498
CFLAR-AS1	-0.037979915	0.002209254	-0.09107	0.009661	-0.00766	0.020164
CFP	0.011322565	-0.134299807	-0.31211	-0.08812	0.256157	0.572791
CFTR	-0.037866452	-0.050523491	-0.01358	-0.0186	-0.06837	-0.0182
CGA	-0.034330525	-0.02532968	0.056312	0.007208	-0.02454	-0.06847
CGGBP1	0.012729631	0.083568025	0.098222	-0.01006	-0.06553	-0.2954
CGN	-0.020723279	-0.046405116	0.022169	0.056932	-0.16112	-0.00868
CGREF1	-0.035573599	-0.076596616	-0.05604	0.028762	-0.05882	-0.0277
CGRRF1	0.038565869	0.05075936	0.169155	0.009726	-0.04612	-0.09126
CH25H	-0.006844897	0.001804316	-0.02413	-0.00027	-0.00152	0.020035
CHAC1	-0.014716026	-0.003714195	-0.03383	-0.0029	-0.06373	-0.02917
CHAC2	0.000406794	0.094188328	0.064357	0.094861	-0.26814	-0.22558
CHAD	-0.034495327	-0.055749915	-0.09887	-0.00049	0.003841	0.043986
CHADL	-0.032867042	-0.074430824	-0.12637	-0.01659	0.057064	0.127905
CHAF1A	0.014323696	0.003323903	-0.12406	0.110573	-0.28544	0.158356
CHAF1B	-0.00680179	0.049811252	0.008306	0.103715	-0.35032	-0.12919
CHAMP1	0.02827212	-0.057778439	-0.0082	0.047202	-0.12209	-0.02563
CHAT	-0.039291204	-0.06454172	-0.03378	0.02917	-0.06145	-0.07694
CHCHD1	0.044624594	0.030481943	-0.08029	0.043238	-0.14316	0.395667
CHCHD10	0.031240436	-0.238614457	0.083708	0.045709	-0.13053	0.252566
CHCHD2	0.073847137	0.074197596	0.055831	0.141187	-0.31633	0.044997
CHCHD3	0.016170611	0.037062353	0.00072	0.07264	-0.19848	0.180735
CHCHD4	0.026131529	-0.017021384	-0.05558	0.072446	-0.25024	0.224115
CHCHD5	0.052176603	0.012986187	0.036433	0.037328	0.012274	0.094138
CHCHD6	-0.001764228	-0.065406375	0.258234	0.073522	-0.21073	-0.17404
CHCHD7	0.042402471	0.020558525	0.213743	0.024941	-0.0827	-0.01333
CHD1	0.050411683	0.011885891	-0.0961	0.020306	-0.02314	0.118039
CHD1L	0.018004713	-0.014768192	-0.01392	0.026905	-0.08194	-0.13615
CHD2	-0.004928956	0.041717972	0.084352	-0.05318	0.033061	-0.40964
CHD3	0.029651758	-0.00233823	0.400172	0.001366	-0.04755	-0.32971

CHD4	0.085973336	0.0704349	0.068733	0.061511	-0.12478	-0.16789
CHD5	-0.048067877	-0.087177015	-0.10512	0.034743	-0.12362	0.066358
CHD6	-0.014605245	0.084788779	0.154062	-0.01904	0.037264	-0.14152
CHD7	-0.006316423	-0.036665267	-0.05414	-0.00636	0.003784	-0.37683
CHD8	0.06962351	0.076343174	0.0516	0.004636	-0.05498	-0.26932
CHD9	-0.001874048	-0.070142475	-0.10054	-0.02189	0.030065	0.005483
CHDC2	-0.023344483	-0.022381722	0.065297	-0.01572	0.014819	-0.12004
CHDH	-0.019402198	-0.002421263	-0.04765	0.041791	-0.09345	-0.0748
CHEK1	0.006150096	0.047153432	-0.02668	0.151061	-0.41006	-0.0526
CHEK2	-0.001347336	-0.034758152	-0.09196	0.05713	-0.17317	-0.01941
CHERP	0.014454623	-0.043456421	0.038611	0.011166	-0.08619	-0.16998
CHFR	0.040360884	0.06168396	-0.08573	-0.08083	0.204517	0.021891
CHGA	-0.038349472	-0.03215897	-0.08482	0.016516	-0.02497	0.049022
CHGB	-0.046406312	-0.09112105	0.017219	-0.00085	0.033366	0.024986
CHI3L1	-0.015060829	-0.070436875	0.007972	0.008246	-0.03661	-0.10465
CHI3L2	0.00294744	0.121390692	0.199937	-0.06789	0.191182	-0.23835
CHIA	-0.031690722	-0.033766901	-0.05761	0.012166	-0.02376	0.025256
CHIAP2	-0.041944366	-0.024545624	-0.00537	0.006074	-0.02539	-0.11074
CHIC1	0.029881267	0.105013807	0.177071	0.084358	0.033124	-0.20646
CHIC2	0.033680451	0.103302905	-0.0682	0.025132	-0.06305	0.005697
CHID1	0.016400088	-0.033079487	-0.0318	0.091829	-0.26443	0.193475
CHIT1	-0.024969804	-0.038256739	0.266648	0.047022	-0.09264	-0.07048
CHKA	-0.002945529	-0.064397622	-0.14892	0.004726	-0.0325	0.139631
CHKB	0.058327615	-0.0034899	0.012239	-0.07733	0.21518	-0.06505
CHKB-AS1	-0.02133585	0.001826482	0.05557	0.046977	-0.1142	0.040862
CHL1	-0.054699655	-0.095637201	-0.22721	-0.01936	0.058433	-0.12154
CHL1-AS1	-0.051988227	-0.021953187	0.039223	0.012434	0.000682	-0.11407
CHM	-0.021587721	0.097888612	0.077636	-0.01717	0.049943	0.05366
CHML	-0.036905969	-0.057638238	-0.02797	0.050965	-0.17906	0.048185
CHMP1A	0.067594781	0.063083707	-0.11223	0.046561	-0.15	-0.07619
CHMP1B	0.038372378	-0.026686519	-0.17133	-0.05005	0.162117	0.039192
CHMP2A	0.063685724	0.039206571	-0.33496	0.022225	-0.03794	0.28332
CHMP2B	0.134432352	-0.077699682	-0.25898	0.005224	-0.04534	0.251077
CHMP4B	0.0651188	-0.032167533	-0.23469	-0.01229	0.043765	0.32464
CHMP4C	-0.046179696	0.098302328	-0.00659	-0.03072	0.112266	-0.01488
CHMP5	0.067285237	0.065052674	-0.27675	0.082134	-0.18402	0.104401
CHMP6	0.008920908	0.005692861	-0.09374	-0.01903	-0.02264	0.25865
CHMP7	0.025957091	-0.102794769	0.27771	-0.02834	0.0584	-0.24503
CHN1	-0.017687312	-0.022149695	0.270259	-0.02396	0.060488	-0.25643
CHN2	0.065141843	0.166331211	0.006861	-0.03572	0.104599	0.224154
CHODL	-0.02683346	0.018507551	0.102153	0.031619	-0.09304	-0.12842
CHORDC1	0.025306402	0.085667781	0.071459	0.06255	-0.17303	-0.03065
CHP1	0.037974205	-0.015327585	-0.3937	-0.01053	0.032372	0.516241
CHP2	-0.03418576	-0.074930364	0.040048	0.017713	-0.07176	-0.05029
CHPF	-0.010833875	-0.007100088	-0.01987	0.084417	-0.30527	-0.15413
CHPF2	0.049555211	-0.032505442	-0.19485	-0.01723	0.02136	0.098346
CHPT1	-0.0217012	-0.175820146	-0.2557	-0.01757	0.171389	0.137919
CHRAC1	0.031909409	-0.066578828	-0.18622	0.015227	-0.05947	0.232911
CHRD	-0.042333253	-0.083249547	-0.05485	0.032627	-0.10782	-0.01391
CHRDL1	-0.030503428	-0.094347156	-0.03938	0.01739	-0.03807	-0.02429
CHRDL2	-0.035186597	-0.058662247	0.049895	0.03338	-0.06713	-0.03485

CHRM1	-0.046022931	-0.068160797	-0.10229	-0.00129	0.028743	0.025849
CHRM2	-0.028632825	-0.04982836	0.059041	0.023964	-0.05553	-0.09235
CHRM3	-0.070661806	-0.027872545	0.011687	0.007639	-0.06073	-0.04467
CHRM3-AS1	-0.003069212	-0.072734313	0.380204	-0.03918	0.086976	-0.23628
CHRM4	-0.034666494	-0.034409189	0.005872	0.022549	-0.07063	-0.08226
CHRM5	-0.038249492	-0.001712464	0.070013	0.008439	-0.00181	-0.05789
CHRNA1	-0.054423794	-0.106977678	0.021048	0.049659	-0.11374	-0.02204
CHRNA10	-0.099725891	-0.002063874	0.075573	-0.03415	-0.05546	-0.10414
CHRNA2	-0.017697556	-0.055787528	-0.07557	-0.00551	-0.08565	0.022498
CHRNA3	-0.064508058	-0.089322549	-0.028	-0.04816	-0.00311	0.00788
CHRNA4	-0.084233641	-0.047306278	-0.17088	0.020682	0.003112	0.077813
CHRNA5	-0.025811791	0.011885321	0.108965	0.11823	-0.27329	-0.0581
CHRNA6	-0.027703231	-0.053682808	0.035457	0.076695	-0.20688	-0.03943
CHRNA9	-0.037966041	-0.00024919	-0.05301	0.006607	-0.01302	-0.01772
CHRNB1	-0.003633735	0.149489695	0.133416	-0.01282	0.06773	0.09487
CHRNB2	0.001437832	-0.111900736	0.008868	-0.00135	-0.10564	-0.11236
CHRNB3	-0.052986363	-0.070042685	0.008493	-0.00164	0.035653	0.060461
CHRNB4	-0.052414055	-0.046305054	-0.05886	-0.0029	0.033306	0.014765
CHRND	-0.033560409	-0.049212437	-0.02107	0.002107	0.017469	0.156462
CHRNE	-0.039931941	-0.038620482	0.006471	-0.00765	-0.00421	-0.08287
CHRNG	-0.026810693	-0.056388907	-0.05107	0.004751	-0.05312	0.059038
CHST1	-0.048803531	-0.089221907	0.058583	0.016772	-0.0567	0.093819
CHST10	-0.004769359	0.052051437	0.062377	0.013231	-0.10038	-0.27404
CHST11	0.009231011	0.119007264	-0.24057	0.051927	-0.14035	0.043102
CHST12	0.018696593	0.262232658	0.116141	0.014963	-0.05217	-0.24938
CHST13	-0.010365769	-0.147208106	-0.25122	-0.06509	0.169558	0.459619
CHST14	0.017988937	0.056463503	-0.07387	0.030768	-0.1512	-0.02685
CHST15	0.001657603	-0.162049986	-0.47535	-0.09599	0.266845	0.433505
CHST2	0.005599615	0.352683243	-0.27566	-0.08594	0.237727	0.084946
CHST3	-0.083005654	0.041449644	-0.07091	-0.0485	-0.01338	-0.04084
CHST4	-0.026697699	0.017350971	-0.00755	-0.00122	-0.01318	-0.04562
CHST5	-0.034035559	-0.0441375	0.007913	-0.00549	-0.05301	-0.0462
CHST6	-0.030807453	-0.066161471	0.020613	0.007292	0.004283	-0.07817
CHST7	0.012933958	0.005887532	-0.12406	-0.0325	0.087005	0.242514
CHST8	-0.02569827	-0.041576414	0.005289	0.006263	-0.03689	0.108535
CHST9	-0.064241116	-0.030645409	-0.08006	0.001589	-0.00786	0.044107
CHSY1	0.033791887	0.081495565	-0.13195	0.045402	-0.1142	0.121392
CHSY3	-0.021326104	0.066966531	-0.06207	-0.02251	0.078284	0.046239
CHTF18	0.004927146	0.037785831	0.056136	0.099557	-0.31342	-0.14853
CHTF8	0.079569456	-0.015737574	0.014472	0.048718	-0.13052	-0.00126
CHTOP	0.119557711	0.203698559	0.127519	0.11516	-0.13451	-0.33132
CHUK	0.03068048	0.095274112	-0.26948	0.053352	-0.12372	0.13828
CHURC1	0.028282088	0.025684205	0.090646	-0.06773	0.124175	-0.02225
CIAO1	0.072561143	0.243102728	0.150258	-0.01575	-0.12488	0.075107
CIAPIN1	0.021761516	0.064957387	0.36281	0.040688	-0.13875	-0.06048
CIART	-0.01962371	-0.046700466	0.015202	-0.00137	-0.00476	-0.09242
CIB1	0.054228791	0.050750695	0.118077	-0.01184	0.041828	-0.00076
CIB2	-0.020357669	-0.112016752	0.02636	-0.01801	-0.01065	-0.17081
CIB3	-0.047214743	-0.130153546	-0.05548	0.052689	-0.14834	0.123786
CIB4	-0.048561916	-0.076505356	0.027542	0.002594	0.025163	0.019349
CIC	0.029758677	-0.119696105	0.061098	0.003123	-0.0573	-0.08511

CIDEA	-0.034973383	-0.080327362	-0.06363	0.021107	-0.08323	0.001859
CIDEB	0.021057411	0.030266655	-0.15955	0.008686	-0.01309	0.353844
CIDEC	-0.024967173	-0.014144623	-0.09967	-0.02903	0.049887	0.066778
CIDECP	-0.029216758	-0.033561116	-0.00778	0.016489	-0.03838	0.053302
CIITA	0.031526527	-0.121782533	-0.17344	0.080862	-0.04831	-0.02028
CILP	-0.024858983	-0.017245269	0.041161	-0.02282	-0.00207	-0.0796
CILP2	-0.054660396	-0.072639842	-0.04469	-0.01867	0.094965	0.00214
CINP	0.000530523	0.062561772	-0.07814	0.003398	-0.13504	0.178113
CIPC	0.017106495	0.017571796	-0.21846	0.016497	-0.01677	0.057657
CIR1	0.036977539	0.004819068	-0.16922	-0.01335	0.045336	-0.03239
CIRBP	0.039711385	-0.025587141	0.199533	-0.05679	0.100567	-0.266
CIRBP-AS:	-0.049458914	-0.065322485	-0.11707	0.001905	-0.05896	0.049907
CIRH1A	0.023569289	0.007795425	0.174251	0.062272	-0.15969	-0.02116
CISD1	0.024329278	0.061185455	0.177303	0.053992	-0.15364	0.037321
CISD2	0.00220786	0.036743052	-0.00999	0.081307	-0.24841	0.109763
CISD3	0.010500925	0.161416745	0.010911	0.061096	-0.18614	0.116682
CISH	0.03672283	0.031498753	0.19915	0.092543	-0.17988	-0.14179
CITED1	-0.032814104	-0.027557761	-0.10148	0.01324	-0.03924	0.112245
CITED2	0.040563266	-0.019447739	0.137574	0.008765	0.073565	0.294489
CITED4	-0.01332543	-0.150804909	0.066074	-0.04008	0.056749	0.09642
CIZ1	0.00740931	-0.022039246	0.041006	0.055304	-0.13462	0.054202
CKAP2	-0.041998808	-0.097966412	-0.12853	-0.09169	0.112726	-0.1131
CKAP2L	-0.01573665	-0.019365034	0.025148	0.183769	-0.49094	-0.07568
CKAP4	0.00834871	-0.155296153	-0.12071	-0.08511	0.217238	0.366826
CKAP5	0.023315472	0.116199039	0.074468	0.114093	-0.32176	-0.04892
CKB	-0.007628247	-0.083665103	-0.07001	-0.02792	0.036813	0.029461
CKLF	0.027222142	0.030385022	-0.12246	0.053339	-0.12673	0.150481
CKM	-0.031084026	-0.032757111	-0.07811	-0.00605	0.049393	0.30505
CKMT2	-0.03589887	-0.085367978	0.000754	-0.01143	0.057028	0.011426
CKMT2-AS:	-0.004350727	0.011975446	0.239818	0.014889	-0.01621	-0.22172
CKS1B	0.019152846	0.022425566	0.067902	0.127005	-0.38105	-0.13893
CKS2	0.019735003	0.031015653	-0.11435	0.135428	-0.3867	-0.09383
CLASP1	0.021043036	0.18738362	0.069397	0.033288	-0.11121	-0.14059
CLASP2	-0.027043152	0.07382667	-0.03093	0.042527	-0.10426	0.022398
CLASRP	0.048805182	-0.166065083	0.007294	-0.01972	0.039403	-0.13034
CLC	0.003870118	-0.038068386	-0.07127	-0.04605	0.119669	-0.23088
CLCA1	-0.033189834	-0.054569843	0.023759	0.017743	-0.04214	0.064572
CLCA2	-0.109982106	-0.043792597	-0.06669	-0.01528	0.030579	0.050606
CLCA3P	-0.1174697	-0.108058743	-0.02627	0.015168	0.077792	0.024951
CLCA4	-0.052818311	-0.07574907	-0.02151	-0.02193	0.08128	-0.02397
CLCC1	0.032474368	0.025282565	-0.0347	0.07054	-0.08945	0.176743
CLCF1	-0.004635731	-0.081419132	0.034142	-0.01771	-0.01521	-0.08903
CLCN1	-0.021782347	-0.121619041	-0.08337	0.016029	-0.03181	0.244243
CLCN2	-0.029923775	-0.066049031	0.058943	0.095752	-0.24492	0.059607
CLCN3	0.024457212	0.100266035	-0.01544	-0.00961	0.031004	0.073758
CLCN4	0.014536983	-0.169317708	-0.17024	-0.05773	0.181095	-0.11179
CLCN5	-0.00055471	-0.111734001	-0.21631	-0.03481	0.017707	0.333494
CLCN6	0.010751561	-0.035786456	-0.10428	0.007495	0.014434	0.290054
CLCN7	0.037001757	-0.081749721	0.014805	-0.02666	0.03848	0.070409
CLCNKB	-0.031045867	-0.086877837	-0.10521	0.032655	-0.05442	-0.06985
CLDN1	-0.055466091	-0.032883196	-0.05505	0.043951	-0.10443	0.070398

CLDN10	-0.026056885	-0.077874503	-0.01628	-0.0193	0.055694	-0.11576
CLDN10-AS	-0.049225266	0.014710447	-0.04152	0.047955	-0.10154	-0.02431
CLDN11	-0.077131475	-0.018275026	-0.01834	0.006233	0.00415	0.017154
CLDN12	-0.010885524	-0.014237366	0.108121	-0.0013	0.017737	-0.07234
CLDN14	-0.034191911	-0.104639818	-0.0187	0.036873	-0.07609	0.156981
CLDN15	0.01373807	-0.070747723	0.289665	0.008337	-0.09611	-0.2931
CLDN16	-0.033493429	0.008863989	0.030929	0.011497	-0.08444	-0.14578
CLDN17	-0.027788267	-0.057090616	-0.01865	-0.0049	-0.06096	-0.0472
CLDN18	-0.045517179	-0.020286436	-0.09434	0.031625	-0.05015	0.080499
CLDN19	-0.031782815	-0.051368953	0.107127	0.008311	-0.09625	-0.18171
CLDN2	-0.034683808	-0.063852491	0.061712	0.034035	-0.09942	-0.09441
CLDN20	-0.03153954	0.010535118	-0.10545	-0.02709	0.09638	-0.07987
CLDN23	-0.013691839	-0.091612807	-0.08007	0.002419	0.104395	0.088479
CLDN3	-0.045289819	-0.054580119	-0.10502	0.01891	-0.02847	0.079148
CLDN4	-0.043100822	0.029516341	-0.01546	-0.01683	0.007836	-0.05165
CLDN5	-0.022420609	-0.064984796	0.01157	-0.12853	0.336405	0.003111
CLDN6	-0.032214057	-0.099380828	0.083537	-0.01187	-0.01758	0.061026
CLDN7	-0.031237998	-0.057306839	0.206362	-0.01573	0.038437	0.056169
CLDN8	-0.029805792	-0.041241577	0.064299	-0.00079	-0.01446	-0.03513
CLDN9	-0.029960103	-0.08833741	-0.07458	-0.00037	-0.05982	-0.0436
CLDND1	-0.007805207	0.161410187	0.288241	0.044713	-0.10968	-0.23245
CLDND2	-0.014020322	0.150333053	-0.01552	0.001844	-0.02029	-0.12151
CLEC10A	-0.001907497	-0.083556837	-0.21335	-0.07811	0.22722	0.315946
CLEC11A	-0.020205003	-0.095533755	0.189088	0.021614	0.020298	0.288365
CLEC12A	-0.010999478	-0.101437316	-0.24864	-0.10987	0.298653	0.721915
CLEC12B	-0.002803155	-0.082722631	-0.27456	-0.05318	0.193303	0.609901
CLEC14A	-0.03020636	-0.081223685	-0.0224	-0.01201	0.00525	-0.00765
CLEC16A	-0.005772346	-0.038392811	0.006892	0.045288	-0.11	0.054205
CLEC1A	-0.011993223	-0.1103728	-0.19737	-0.02515	0.051215	0.411164
CLEC1B	-0.008696637	-0.056690136	-0.02019	-0.24904	0.690441	0.127992
CLEC2B	0.023857271	0.129109313	0.112589	-0.05097	0.156603	-0.03674
CLEC2D	-0.001729887	0.02616101	0.374937	-0.01014	0.064836	-0.35888
CLEC2L	-0.032090137	-0.043593476	-0.06381	-0.06272	0.097513	-0.08362
CLEC3A	-0.032900437	-0.027844684	0.014412	0.011336	-0.02899	-0.08559
CLEC4A	0.013392861	-0.131707156	-0.42724	-0.09905	0.298628	0.492217
CLEC4C	-0.005225746	-0.077475236	-0.19015	-0.03578	0.113197	-0.21997
CLEC4D	-0.005631269	-0.084215514	-0.28246	-0.09066	0.25355	0.645539
CLEC4E	-0.002906553	-0.089968604	-0.31102	-0.07367	0.198305	0.493579
CLEC4F	-0.009634909	-0.060779668	-0.08167	0.074788	-0.1869	0.152119
CLEC4G	-0.009285474	-0.060389571	-0.20957	-0.0199	0.077706	0.653295
CLEC4GP1	-0.03433754	-0.029057759	0.014533	-0.02696	0.091065	-0.0401
CLEC4M	-0.054321738	-0.046513953	-0.07525	0.028721	-0.04393	0.088395
CLEC5A	-0.011424581	-0.080762824	-0.13814	-0.07474	0.205032	0.664941
CLEC7A	-0.007118303	-0.13426265	-0.3497	-0.08365	0.167075	0.546902
CLECL1	-0.001618227	-0.112378098	-0.15477	-0.01704	0.041372	0.005984
CLGN	-0.028664042	-0.071261747	0.003673	-0.03415	0.017068	-0.08571
CLHC1	-0.011397562	-0.011230125	0.005858	-0.00191	0.023609	0.030257
CLIC1	0.058009661	0.188812613	-0.32011	0.079792	-0.15917	0.116214
CLIC2	0.001637262	-0.085611346	-0.16917	-0.00036	0.024529	0.21868
CLIC3	0.004330184	0.266438461	0.056567	-0.04198	0.121892	-0.2391
CLIC4	-0.026992491	-0.077333706	-0.15748	-0.12331	0.245019	0.173959

CLIC5	-0.076339316	-0.078363994	0.069905	0.077408	-0.14831	-0.18153
CLIC6	-0.035106294	-0.084316026	0.319377	0.003608	0.000297	-0.15027
CLINT1	0.041632615	0.081554461	0.005384	0.011799	-0.03347	-0.17561
CLIP1	-0.015353579	0.002992676	-0.1063	0.031436	0.021631	0.194473
CLIP1-AS	-0.045739818	-0.067036599	-0.0022	-0.01808	0.092193	0.046503
CLIP2	-0.009961866	0.013827279	-0.20506	-0.06103	0.191874	0.369625
CLIP3	-0.033451211	-0.076466509	0.016039	0.018607	-0.05109	0.014797
CLIP4	0.011863083	0.133462537	0.22485	0.024626	-0.09217	0.328988
CLK1	0.062251288	0.10367922	0.177358	-0.01561	0.088795	-0.42216
CLK2	0.067584714	0.075507317	0.21646	0.017539	-0.04012	-0.16895
CLK3	0.030340837	-0.037980381	0.078765	-0.03229	0.07963	-0.04668
CLK4	0.011052102	0.013790399	0.009556	-0.05098	0.161963	-0.13004
CLMN	-0.014613153	-0.142091319	-0.34059	-0.09441	0.199674	0.649109
CLMP	-0.06723998	-0.014546096	0.0578	-0.01985	0.070837	-0.06466
CLN3	0.039272866	0.042931754	-0.04276	-0.02572	0.049129	0.153984
CLN5	0.007428428	-0.182953887	0.008294	0.029549	-0.00284	0.168186
CLN6	0.026072531	-0.070501906	-0.07958	0.062831	-0.20746	0.152481
CLN8	-0.038275789	-0.095893364	-0.19231	-0.07738	0.203846	0.060299
CLNK	-0.032150428	-0.031319295	-0.10618	-0.02129	0.036878	-0.1575
CLNS1A	0.029678675	-0.020408556	0.167022	0.009553	-0.00417	0.132063
CLOCK	0.026619483	0.142685385	0.179826	0.020047	-0.01291	-0.0178
CLP1	0.027017724	0.008033769	-0.19867	0.057993	-0.16768	0.11538
CLPB	-0.010522366	-0.077352377	-0.17105	0.090957	-0.2556	0.490581
CLPP	0.04383006	-0.027199468	0.351055	0.090608	-0.26859	-0.14605
CLPS	-0.031535003	-0.098762395	0.046716	-0.00765	0.000838	-0.14602
CLPTM1	0.014629837	-0.015414181	-0.00847	-0.00244	-0.02207	0.071767
CLPTM1L	0.056138036	0.137783419	-0.0613	0.120958	-0.14871	-0.05879
CLPX	0.051742823	0.065708753	-0.10154	0.056387	-0.17827	-0.00251
CLRN1	-0.045613896	-0.058712796	0.020049	-0.04759	0.020209	-0.0858
CLRN1-AS	-0.042209348	-0.008505147	-0.0226	-0.00662	0.045233	0.021036
CLRN3	-0.030103382	0.05805474	-0.03741	-0.00577	0.042581	-0.09456
CLSPN	-0.062736286	0.033109295	-0.02287	0.127644	-0.36944	-0.06242
CLSTN1	0.043403852	0.083383397	0.214521	0.01431	-0.03957	-0.16702
CLSTN2	-0.04889907	-0.054585436	-0.02721	0.063586	-0.15491	-0.08836
CLSTN2-AS	-0.034721991	-0.023317194	-0.01319	0.076146	-0.19693	0.056696
CLSTN3	-0.007670293	0.059079628	0.012443	0.05321	-0.21572	-0.18206
CLTA	0.083756007	0.010126688	-0.17985	0.054671	-0.17191	0.270884
CLTB	0.034220284	0.002520403	-0.18155	0.050452	-0.14331	0.119283
CLTC	-0.048252937	-0.019280377	0.000134	-0.09887	0.016316	-0.00422
CLTC-IT1	-0.008292987	0.017092679	-0.1242	0.005575	-0.00237	-0.0209
CLTCL1	-0.013863588	-0.079151701	-0.18841	-0.00769	0.013837	0.4591
CLU	-0.009390992	-0.030547588	-0.05683	-0.02472	0.358578	0.078551
CLUAP1	-0.037345953	0.086541937	0.198438	-0.01878	0.085723	-0.13325
CLUH	0.028737832	-0.017829143	0.095873	0.046964	-0.14871	-0.07451
CLUHP3	0.00844435	-0.000156748	0.083352	0.048251	-0.09542	-0.3745
CLUL1	-0.043434762	-0.063561346	-0.02655	0.021818	-0.04711	-0.05487
CLVS1	-0.031738755	-0.042024799	-0.03196	0.004331	0.001378	-0.04988
CLVS2	-0.050719866	-0.053998124	-0.01124	0.011404	-0.01117	-0.00454
CLYBL	0.027745	0.012352486	0.222813	-0.01758	0.011725	-0.16397
CLYBL-AS	-0.039495799	-0.036744096	0.173991	0.011563	-0.01518	-0.08355
CLYBL-AS	-0.051302455	-0.065833317	0.015439	-0.03511	0.134407	0.046695

CMA1	-0.023549895	-0.040927765	-0.08345	0.046272	-0.12223	0.021598
CMAHP	-0.003449531	0.101928908	0.058095	0.095211	-0.27556	-0.08881
CMAS	0.007030102	0.0253416	0.003065	0.04365	-0.14031	0.083416
CMBL	-0.050447577	-0.018053156	-0.16241	0.009663	0.256324	-0.09248
CMC1	0.020662554	0.240348164	0.41742	0.028428	-0.06497	-0.03984
CMC2	0.049610921	0.089324728	0.15607	0.071616	-0.21574	0.07394
CMC4	0.028400381	0.041300008	0.200521	0.026027	-0.0648	0.02156
CMIP	0.027760708	0.014421992	-0.16235	-0.05773	0.170615	0.189783
CMKLR1	-0.000334098	0.208769682	-0.12231	-0.06824	0.19228	0.198072
CMPK1	0.033886141	0.053816375	0.322782	0.031383	-0.07767	-0.1767
CMPK2	0.019528143	0.027622607	-0.20085	-0.03318	0.087098	0.24018
CMSS1	0.01027739	0.012435205	0.134585	0.067467	-0.20001	-0.22396
CMTM1	-0.020091862	0.004280575	-0.07128	0.008898	-0.02029	0.150721
CMTM2	-0.002662381	-0.068424308	-0.28561	-0.01077	0.037233	0.250959
CMTM3	0.034948364	0.060031483	0.009461	0.02926	-0.0477	0.191307
CMTM4	-0.069158088	-0.097617313	-0.1529	-0.04025	0.090943	0.562556
CMTM5	-0.010146972	-0.03850985	-0.00623	-0.16423	0.436258	0.056776
CMTM6	0.031715421	0.005844296	-0.43731	0.010205	-0.02459	0.194359
CMTM7	0.029011312	-0.297642221	0.085476	-0.07136	0.162478	0.204673
CMTM8	-0.008337912	-0.111140993	0.196668	-0.06979	0.190382	-0.12133
CMTR1	0.044915278	0.059159563	0.103789	-0.00122	-0.04058	-0.12636
CMTR2	0.020633539	0.215953844	-0.02538	0.005677	-0.00781	0.071401
CMYA5	-0.048362341	0.015223109	-0.00988	-0.027	0.060267	-0.0491
CNBD1	-0.05421502	-0.084654094	-0.00733	0.019065	-0.04859	-0.00471
CNBD2	-0.063255463	0.012896317	-0.15026	0.000623	-0.08207	0.01823
CNBP	0.103594656	0.029668094	0.018613	0.046031	-0.10716	0.075126
CNDP1	-0.029413555	-0.005866652	0.012282	0.009302	-0.02168	-0.07711
CNDP2	0.011007255	-0.071460557	0.027413	0.002369	-0.07541	-0.09416
CNEP1R1	0.050781335	0.09000355	-0.21211	0.007212	0.005767	0.260555
CNFN	-0.012389781	-0.015189346	-0.21207	-0.01433	0.056801	0.137067
CNGA1	-0.040126819	-0.096663973	-0.01901	-0.0137	0.061709	0.07539
CNGA3	-0.04779953	-0.000315553	0.007644	-0.01875	0.053916	-0.02354
CNGA4	-0.03214055	0.011941043	-0.10441	0.002326	0.018863	0.077077
CNGB1	-0.036612694	-0.017993611	0.098653	-0.01598	0.080088	0.083005
CNGB3	-0.072670701	-0.078811561	-0.05042	-0.01235	0.053712	0.001594
CNIH1	0.035136544	-0.024989763	0.04902	0.014418	-0.02763	0.317053
CNIH2	-0.022152129	-0.072162384	-0.02833	0.004891	-0.05106	0.036662
CNIH3	-0.044265998	-0.017562211	-0.0005	0.007271	0.006479	-0.00817
CNIH4	-0.053168063	0.056796234	-0.06044	-0.0799	0.046259	0.310462
CNKSR1	-0.024175708	-0.123901308	0.041265	-0.05343	0.158694	-0.05229
CNKSR2	0.002060789	-0.14795172	0.163783	-0.08248	0.179872	-0.36265
CNKSR3	-0.011155242	0.005446279	-0.04168	-0.00112	-0.16423	-0.07419
CNN1	-0.034256239	-0.067031338	-0.06578	-0.02965	0.109512	0.06926
CNN2	0.042614391	-0.095811425	0.082718	0.020331	-0.04258	-0.25264
CNN3	-0.020630669	-0.045974075	0.307426	-0.01323	0.045445	-0.22183
CNNM1	-0.041819423	-0.045818334	-0.10738	0.002621	0.009464	0.051726
CNNM2	-0.046281763	-0.02358829	0.083803	0.037759	-0.14784	-0.02731
CNNM3	0.022615243	0.090548053	0.039339	0.000492	-0.00629	-0.20456
CNNM4	-0.006059941	0.010679446	-0.17299	0.0658	-0.1561	0.039812
CNOT1	0.050055446	-0.041246024	0.129042	0.02901	-0.12768	-0.27127
CNOT10	0.027010519	-0.015946247	-0.0853	0.079047	-0.22974	-0.03637

CNOT11	0.063496809	0.076267135	0.259342	0.068245	-0.21305	-0.3409
CNOT2	-0.004889671	0.079082988	0.085562	-0.00709	-0.02289	-0.13998
CNOT3	-0.016575734	-0.032917765	0.027788	0.009083	0.008327	-0.08422
CNOT4	-0.0608048	0.106567881	-0.00459	0.000267	0.022434	0.008373
CNOT6	0.050629861	0.142697098	-0.00198	0.05862	-0.16154	0.089395
CNOT6L	0.000294036	0.144753687	0.176687	0.002955	0.015563	-0.26635
CNOT7	0.052952569	-0.004420419	0.123083	0.032559	0.145385	-0.15366
CNOT8	0.036974557	-0.005110773	-0.10253	-0.02165	0.079776	-0.22538
CNP	0.022685314	-0.037594729	-0.12483	0.024826	-0.05965	0.055852
CNPPD1	0.04151908	-0.025709342	-0.14285	-0.01767	0.032493	-0.01185
CNPY1	-0.06942843	-0.020134082	-0.06288	-0.05483	0.143053	-0.00865
CNPY2	0.028407412	0.099756569	0.259273	0.047975	-0.13449	0.126732
CNPY3	0.002379436	-0.099384002	-0.28236	-0.06634	0.164235	0.307018
CNPY4	0.004384792	-0.011003785	0.347189	-0.01272	0.011867	-0.1214
CNR1	-0.041613658	-0.081597083	-0.1277	0.127783	0.004322	-0.15263
CNR2	-0.016006489	-0.033153661	-0.08591	0.005381	-0.05594	-0.234
CNRIP1	-0.002570477	-0.051443905	-0.02183	-0.02127	0.06693	-0.11492
CNST	0.01343967	-0.040991426	0.247919	-0.08122	0.219942	-0.33704
CNTD1	-0.030289048	-0.042490866	-0.03836	0.029301	-0.093	-0.03047
CNTD2	-0.003533836	-0.063376249	0.021865	-0.01865	-0.05364	-0.12128
CNTF	-0.036820123	-0.009647967	-0.04175	0.008248	0.001022	-0.13057
CNTFR	-0.024773392	-0.080759605	-0.10779	-0.00522	-0.0881	-0.09228
CNTFR-AS1	-0.057071639	-0.097560092	-0.07112	0.002043	-0.00429	-0.10738
CNTLN	-0.000982191	-0.121365054	-0.2207	-0.02554	0.148874	0.578532
CNTN1	-0.059654158	-0.074105451	-0.06615	-0.00854	0.060213	-0.07284
CNTN2	-0.039076838	-0.087741479	-0.08623	-0.0102	-0.0309	-0.10551
CNTN3	-0.066050038	-0.099424232	0.080385	-0.00568	0.009926	-0.15775
CNTN4	-0.032205333	-0.04217128	-0.0155	0.053174	-0.14112	-0.00023
CNTN5	-0.065406539	-0.076866894	0.020997	0.008303	-0.00435	-0.05948
CNTN6	-0.055622745	-0.05984179	-0.01024	-0.03505	0.085438	-0.01469
CNTNAP1	-0.022359541	-0.082421706	0.047833	-0.0208	0.053509	-0.17513
CNTNAP2	-0.008460452	-0.049041917	-0.12882	0.008439	-0.03134	-0.09976
CNTNAP3	-0.011372344	0.015485765	-0.14271	0.019835	0.033926	-0.16124
CNTNAP3P2	-0.024745089	-0.054310608	-0.08929	0.00882	-0.01499	-0.15782
CNTNAP4	-0.020805765	-0.022890757	-0.0165	0.102073	-0.22174	0.014909
CNTNAP5	-0.053227455	-0.080006188	-0.04823	0.026103	-0.04942	0.063585
CNTRL	0.023894285	-0.056885757	-0.00346	-0.00456	0.006893	-0.03544
CNTROB	-0.02721621	-0.109977947	0.091705	0.038887	-0.17208	-0.0078
COA1	-0.002343896	0.061731217	0.215353	0.013128	0.006189	-0.47923
COA3	0.022853555	0.080083623	0.027044	0.015189	-0.043	0.25736
COA4	0.046471494	0.018879217	0.003293	0.057102	-0.19518	0.139381
COA5	0.041938467	0.054634464	0.145075	0.003099	-0.00487	0.08801
COA6	0.03026026	0.027141377	0.02517	0.05733	-0.16757	0.304011
COA7	0.001613933	-0.017293392	-0.09833	0.068906	-0.20618	0.276106
COASY	0.069503813	-0.018100389	-0.14037	0.028329	-0.09435	0.226443
COBL	-0.013778428	-0.065991204	-0.11869	-0.00056	0.021261	-0.09724
COBLL1	0.015533847	-0.061908364	-0.20403	-0.05055	0.108219	-0.30605
COCH	-0.009611175	-0.061579985	-0.11083	-0.08996	0.246402	-0.13137
COG1	-0.055771001	0.051995059	0.135336	-0.04614	0.094166	-0.16422
COG2	0.01824347	0.0286399	0.077859	0.016115	-0.03817	0.008051
COG3	0.011737792	0.049841344	0.039768	-0.03429	0.114889	-0.05647

COG4	0.024741907	-0.042391335	0.087845	0.004312	-0.02383	0.022046
COG5	0.007890135	0.020060211	-0.06688	0.065293	-0.03216	0.199273
COG6	-0.020636027	0.059630398	0.060141	0.029173	-0.04815	-0.00035
COG7	0.020937895	-0.031819048	0.064959	-0.00568	0.021446	0.066733
COG8	0.020090172	-0.064501862	0.127385	-0.01287	-0.06602	-0.21242
COIL	0.036322846	0.089806675	0.087608	0.051572	-0.13868	0.030073
COL10A1	-0.058084438	-0.039787522	-0.02	-0.0437	0.091762	-0.01323
COL11A1	-0.063219433	-0.09835188	0.057841	-0.02838	0.051462	0.07438
COL11A2	-0.027908179	-0.073214828	0.019469	-0.01926	-0.03922	-0.07815
COL12A1	-0.116540415	-0.124609192	-0.07452	0.033529	0.007489	-0.00912
COL13A1	-0.008946331	0.157984879	-0.12903	-0.00161	0.023446	-0.01691
COL14A1	-0.039560238	-0.029148687	-0.00364	0.013809	0.037239	-0.05212
COL15A1	-0.020956311	0.213918253	0.024307	0.095277	-0.18286	-0.07964
COL16A1	-0.028493768	-0.100277548	0.040464	0.047473	-0.11031	0.079261
COL17A1	-0.031975428	-0.064154807	0.091283	-0.00672	0.023357	0.013712
COL18A1	-0.010338838	-0.121973858	-0.00929	-0.06309	0.135294	-0.15967
COL18A1-/	-0.046862346	-0.080097393	-0.03779	0.020757	-0.02268	0.032214
COL19A1	-0.035869296	-0.101726038	-0.14422	0.032292	-0.07089	-0.048
COL1A1	-0.047831231	-0.107552922	0.006746	0.29366	-0.14195	-0.02707
COL1A2	-0.023295127	-0.034979542	0.019834	-0.01031	0.001872	-0.09963
COL20A1	-0.049675889	-0.005217937	-0.13558	-0.00714	-0.01537	0.115719
COL21A1	-0.021356985	-0.052966608	0.060992	0.033392	-0.11522	-0.07322
COL22A1	-0.019983395	-0.018607553	-0.02959	0.058846	-0.04488	-0.09108
COL23A1	-0.031695277	-0.072672047	0.073947	0.014185	-0.06029	-0.01694
COL24A1	-0.007839825	-0.056910149	-0.06658	-0.00064	0.014676	-0.15252
COL25A1	-0.105501922	-0.028094315	0.124354	-0.11912	0.002521	0.08669
COL26A1	-0.017593259	-0.123476479	-0.07779	0.014692	-0.06343	-0.12152
COL27A1	-0.061413289	-0.080257149	0.011696	0.036104	-0.00888	-0.07754
COL28A1	-0.069243754	-0.025303539	-0.05314	-0.01266	0.068108	-0.02403
COL2A1	-0.055048585	-0.037779885	-0.07564	0.015383	-0.00898	-0.07044
COL3A1	-0.020056456	-0.015823825	-0.01263	0.077148	-0.08847	-0.15941
COL4A1	-0.042806201	-0.046159193	-0.03488	0.034844	-0.07541	0.058495
COL4A2	-0.051498387	-0.065001284	-0.15804	-0.01342	0.076862	0.02288
COL4A3	-0.051809972	-0.090258858	-0.04668	-0.03221	0.092298	0.028183
COL4A3BP	0.067926736	0.096533086	-0.42913	-0.05577	0.195382	0.356139
COL4A4	-0.026160769	-0.076426669	-0.10671	-0.05199	0.14338	-0.16976
COL4A5	-0.064559783	-0.108064402	0.025723	-0.00114	0.029432	0.072152
COL4A6	-0.012776913	-0.052728238	0.064972	0.011269	-0.06366	-0.13036
COL5A1	-0.00318983	-0.062611875	0.019852	0.095983	0.048933	-0.0185
COL5A2	-0.021150924	-0.054423437	0.00533	0.120453	-0.19201	-0.05225
COL5A3	-0.02166014	0.062699907	0.053025	-0.02367	0.040093	-0.10143
COL6A1	-0.045522448	-0.066251852	-0.05841	0.029726	-0.03197	0.073837
COL6A2	-0.013038306	0.044126849	0.263328	-0.05897	0.196823	-0.05448
COL6A3	-0.01334463	-0.078755641	0.277223	0.048726	-0.14039	-0.14692
COL6A5	-0.034910943	-0.037209045	-0.016	0.285067	-0.29616	0.009569
COL6A6	-0.020201297	-0.019486549	-0.01353	0.090266	-0.14905	-0.04978
COL7A1	-0.033479703	-0.080857486	-0.03237	0.012704	-0.10131	0.001876
COL8A1	-0.077949841	-0.03079799	0.048542	-0.0272	0.030575	-0.04919
COL8A2	-0.015368677	-0.112749071	-0.24316	-0.03999	0.128168	0.329842
COL9A1	-0.062381324	-0.079117857	-0.03874	-0.00948	-0.00863	-0.08609
COL9A2	-0.017757843	-0.083212979	-0.1342	0.040108	-0.14587	0.090335

COL9A3	-0.01185506	-0.159127003	-0.20845	-0.04769	0.129161	-0.04611
COLCA1	0.036543742	0.012842878	-0.03413	0.045727	-0.07089	-0.1693
COLCA2	-0.036512639	-0.043454631	0.096483	-0.03264	0.118734	0.117419
COLEC10	-0.031412523	-0.019540731	0.003755	0.016056	-0.03817	-0.02197
COLEC11	-0.034081048	-0.063636426	0.023857	-0.01666	0.045439	0.013907
COLEC12	-0.026317787	-0.029447573	-0.1421	-0.02393	0.083252	0.328228
COLGALT1	0.016567146	-0.098324941	-0.20944	-0.02535	0.048994	0.429418
COLGALT2	-0.017403136	0.254978572	-0.05353	-0.02665	0.075117	-0.13327
COLQ	-0.002793528	0.300627264	0.096724	-0.03228	0.080019	-0.17907
COMMD1	0.032811005	0.036234567	0.02243	0.027166	-0.07838	0.287974
COMMD10	0.051715017	0.044431913	-0.11838	-0.00619	-0.04114	0.375722
COMMD2	0.040654108	0.088544857	-0.11816	0.051387	-0.1526	0.08527
COMMD3	0.04389951	0.035424111	-0.10122	0.043075	-0.12241	0.125816
COMMD4	-0.000390142	0.095556538	-0.03706	0.050593	-0.13178	0.195687
COMMD5	-1.32E-06	0.007607583	-0.16671	0.009488	-0.13337	0.197845
COMMD6	0.042450038	-0.003303259	0.277074	-0.00283	-0.00671	-0.09251
COMMD7	0.033936417	0.103733547	0.097484	0.014759	-0.04826	0.042631
COMMD8	0.050485044	0.077376702	-0.0938	0.041501	-0.10388	0.210566
COMMD9	0.027958431	-0.018975596	-0.15626	-0.01985	0.06472	0.40078
COMP	-0.025088106	-0.053387375	-0.10348	0.033707	-0.078	0.087292
COMT	0.038191964	-0.006275378	-0.17707	0.006295	-0.03272	0.497265
COMTD1	0.012422863	-0.000348313	0.052837	0.065099	-0.21978	0.102052
COPA	-0.020301068	0.001532262	-0.11314	-0.00815	0.003224	0.262389
COPB1	0.099919945	0.182022565	-0.07196	0.089625	-0.2037	-0.13449
COPB2	0.02936255	0.125447292	-0.18081	0.029719	-0.06005	0.154918
COPE	0.031740274	-0.003749435	-0.15945	0.080595	-0.19131	0.365303
COPG1	0.036210616	0.079298872	-0.1748	0.101603	-0.29367	0.049123
COPG2	-0.016503409	-0.046708899	0.09315	0.073759	-0.18371	0.162766
COPG2IT1	-0.025123218	-0.033861632	0.28253	-0.0251	0.101221	-0.07058
COPRS	0.015654845	-0.043658998	-0.13208	0.058859	-0.18311	0.17856
COPS2	0.039821948	0.00567115	0.040646	0.068836	-0.18081	0.10952
COPS3	0.066429186	0.061571698	-0.22795	0.094641	-0.26552	0.078366
COPS4	0.022741135	0.1127425	0.094754	0.026876	-0.06609	0.068082
COPS5	0.089014977	0.170064283	0.054087	0.065232	-0.18331	0.116151
COPS6	0.064867767	0.077625482	0.113394	0.121785	-0.35742	0.150049
COPS7A	0.039231465	-0.022328757	-0.18312	0.003116	-0.04033	0.301133
COPS7B	0.01483507	0.055766913	0.10584	0.028252	-0.24525	-0.11
COPS8	0.004124083	0.103183802	0.158923	0.110514	-0.208	-0.01772
COPZ1	0.054485916	0.085666041	-0.00514	0.016175	-0.06624	0.154509
COPZ2	-0.016455416	0.139164934	-0.0947	-0.01953	-0.03232	0.03116
COQ10A	0.022534423	0.081949829	0.332132	-0.00891	-0.0061	-0.45569
COQ10B	0.033131156	0.10367705	-0.05801	0.05303	-0.1173	-0.15114
COQ2	0.008963367	-0.067493911	-0.24441	-0.00374	0.016209	0.511138
COQ3	0.005331102	0.063342912	0.272743	0.109158	-0.33143	-0.16149
COQ4	0.018490234	0.020373838	0.108769	0.062409	-0.15293	0.023456
COQ5	0.028511443	0.059547142	0.059516	0.041652	-0.12314	0.139556
COQ6	0.024037477	-0.029022372	0.204072	0.072819	-0.17555	0.110356
COQ7	-0.028512101	-0.048505469	-0.01309	0.061417	-0.22778	0.006205
COQ9	0.027129844	0.02415076	0.152436	0.035079	-0.13716	0.12221
CORIN	-0.049040535	-0.057981989	-0.06643	0.027301	-0.08766	0.089865
CORO1A	0.022014673	0.026221887	-0.04929	0.019952	-0.05154	-0.01156

CORO1B	0.019417746	-0.175693381	-0.0754	-0.03675	0.096403	0.310781
CORO1C	0.021361314	-0.04640063	-0.50762	-0.04858	0.131588	0.297434
CORO2A	-0.001331221	-0.092190225	0.227677	-0.00166	0.015158	0.016357
CORO2B	-0.014021447	-0.043542152	-0.106	-0.01131	0.048114	-0.18604
CORO6	-0.022200353	-0.018405958	0.073048	0.003895	-0.02135	-0.11138
CORO7	0.022452755	-0.063470572	-0.01132	-0.03204	0.070143	0.030793
CORT	-0.028420882	0.035705831	-0.09006	0.055278	-0.15142	0.068393
COTL1	0.023457893	-0.184399985	-0.17875	-0.02081	0.058032	0.310759
COX1	0.044198289	0.150866846	-0.0601	0.128559	-0.19157	0.105429
COX10	0.02778071	0.013781578	0.20337	0.058684	-0.2173	0.000665
COX10-AS	-0.017014095	0.015654306	0.091193	0.02204	-0.1087	-0.15487
COX11	0.006806422	0.029395434	0.199071	0.035897	-0.08427	-0.18894
COX14	0.04891533	-0.014746954	0.193274	0.03027	-0.09146	0.156718
COX15	0.017868965	-0.019665513	-0.09591	0.036102	-0.0091	0.16353
COX16	0.030836143	0.063585146	0.13218	0.008815	-0.02012	0.180173
COX17	-0.016541009	0.042530945	-0.07268	-0.02639	0.082548	0.074124
COX18	0.019132431	0.006215634	0.089029	0.040284	-0.08917	0.201902
COX19	0.015326714	-0.107145365	0.148486	-0.06015	0.176453	0.012847
COX2	0.05488949	0.174290366	-0.15424	0.129371	-0.09055	0.168384
COX20	0.016610659	0.058037985	0.113786	0.060033	0.053975	-0.07246
COX4I1	0.064521189	-0.028023906	0.093399	0.030544	-0.05469	0.163349
COX4I2	-0.018343242	-0.084557624	-0.04484	0.027022	-0.14565	0.048466
COX5A	0.04528949	0.043552133	0.065328	0.064431	-0.171	0.206763
COX5B	0.091255587	0.009849902	-0.09251	0.117893	-0.3002	0.346419
COX6A1	0.066025512	0.071810905	-0.10195	0.169165	-0.37757	0.049538
COX6A2	-0.034706307	-0.04512058	0.07773	0.013194	-0.0331	-0.04814
COX6B1	0.089255355	0.01930295	0.018543	0.105976	-0.23132	0.19362
COX6B2	-0.029091885	-0.046236682	-0.02561	0.016141	-0.08115	-0.04843
COX6C	0.046919136	0.081567141	0.194519	0.065662	-0.15127	0.052602
COX7A1	-0.026705697	-0.074465035	-0.13956	0.015762	-0.01466	0.039912
COX7A2	0.088747575	0.047500407	0.108819	0.164984	-0.38938	-0.07225
COX7A2L	0.082869393	0.031361313	0.193123	0.082467	-0.22463	0.038069
COX7B	0.040786839	0.061518896	0.094068	0.055191	-0.10621	0.227985
COX7B2	-0.049118078	0.075540704	-0.10527	0.030402	-0.05125	0.13839
COX7C	0.048942011	0.030640479	0.201693	0.051681	-0.12554	0.101338
COX8A	0.064721081	0.05362017	-0.04705	0.121015	-0.29635	0.310538
COX8C	-0.086131207	-0.095269642	-0.16303	-0.0246	0.045431	0.068332
CP	-0.065306176	0.068235541	-0.07298	0.002343	-0.02208	0.162691
CPA1	-0.052749005	-0.058133152	-0.07486	0.007422	-0.03757	0.091954
CPA2	-0.027676471	-0.058117729	-0.02546	0.00737	-0.0626	-0.14864
CPA3	-0.003074866	-0.035877073	0.063923	-0.07917	0.230678	-0.19414
CPA4	-0.030372971	-0.010063612	-0.04033	-0.01626	-0.01459	-0.03246
CPA5	-0.012899729	-0.134148095	0.603682	-0.00622	0.017163	-0.17913
CPA6	-0.074146429	-0.076040488	0.005377	-0.02388	0.05152	-0.06298
CPAMD8	-0.008822353	-0.0622791	0.020602	-0.01624	0.07342	0.561177
CPB1	-0.011890411	-0.010742589	0.005167	0.090984	-0.19316	-0.01349
CPB2	-0.042688592	0.021352997	0.005324	-0.00179	0.016831	-0.05225
CPD	0.003107638	0.09233367	-0.24652	-0.00175	-0.01193	0.293445
CPE	-0.089475044	-0.131925872	0.055653	5.14E-05	0.055035	0.024599
CPEB1	-0.038639583	-0.062081656	0.289493	-0.01656	0.040293	-0.08987
CPEB2	0.002284575	-0.032088203	-0.12152	0.000371	-0.02477	0.008525

CPEB2-AS:	-0.045799691	-0.025225702	0.006563	0.0074	0.00336	-0.09102
CPEB3	-0.03494931	0.036131579	-0.15842	-0.0334	0.172409	0.267808
CPEB4	-0.011164058	0.022990871	-0.29965	-0.0313	0.067221	0.256743
CPED1	-0.00519179	-0.127077366	-0.26524	-0.03939	0.126319	0.282429
CPLX1	-0.018973667	-0.059360218	-0.0563	-0.00286	0.015118	-0.11404
CPLX2	-0.040278915	-0.020676709	-0.17245	0.012863	-0.00297	0.118776
CPLX3	-0.033791308	-0.072677377	0.005326	0.026811	-0.10178	-0.09675
CPM	-0.025137701	-0.121290415	-0.18412	-0.10166	0.233806	0.644452
CPN1	-0.036093204	-0.031009783	-0.00056	0.020413	-0.05179	0.015394
CPN2	-0.045476787	-0.081520732	-0.10458	0.010168	-0.00373	0.022515
CPNE1	0.043099843	0.055631059	0.13813	0.07062	-0.18614	-0.27183
CPNE2	-0.003390064	-0.150380231	0.265349	-0.11118	0.3002	0.444756
CPNE3	0.023602151	0.017646413	-0.04246	-0.09607	0.257507	-0.08544
CPNE4	-0.072066088	-0.087714752	-0.01787	-0.11829	0.352481	0.058286
CPNE5	-0.007944196	-0.054575628	-0.11698	-0.128	0.340854	-0.08412
CPNE6	-0.031041348	-0.079736283	-0.00317	0.012635	-0.10093	-0.0722
CPNE7	-0.000127576	-0.111233679	0.066563	-0.03333	-0.01379	-0.02322
CPNE8	0.038125071	0.026489639	-0.20323	-0.0836	0.327662	0.502904
CPNE9	-0.026076907	-0.075449177	-0.10602	0.04468	-0.11964	0.111837
CPO	-0.034101443	-0.070185027	-0.05384	0.016916	-0.03351	-0.11223
CPOX	0.022540894	0.077707538	0.064508	0.070703	-0.20291	0.186821
CPPED1	0.023093393	-0.087304814	-0.37817	-0.04683	0.144678	0.39733
CPQ	0.017977118	0.028038918	-0.24302	-0.06065	0.184833	0.337203
CPS1	-0.030197643	-0.037203726	0.034828	0.02376	-0.06871	-0.15498
CPS1-IT1	-0.049749622	-0.060901438	0.010116	0.040754	-0.08425	-0.02402
CPSF1	-0.027552484	-0.066939571	0.15078	-0.00315	0.02371	0.113661
CPSF2	0.046338376	0.113439281	-0.16214	0.06796	-0.15778	0.248641
CPSF3	0.0282436	0.027248004	-0.03945	0.064467	-0.15448	0.1421
CPSF3L	0.009996578	-0.017551429	0.044106	0.047843	-0.16409	0.082284
CPSF4	0.064143722	0.000228214	0.170912	0.102813	-0.29532	-0.17114
CPSF6	0.007395802	-0.021402192	0.253763	0.038007	-0.06181	-0.24652
CPSF7	0.033190233	-0.058420877	-0.03919	0.047058	-0.13661	-0.21466
CPT1A	-0.019255184	0.054719566	-0.1518	0.027416	-0.01929	0.233378
CPT1C	-0.032544997	-0.077816243	0.014651	0.036782	-0.06916	-0.05847
CPT2	0.025045097	-0.067440891	-0.12447	0.01847	-0.05318	0.317708
CPTP	0.017857316	-0.109050527	-0.0536	0.010985	-0.08383	-0.09695
CPVL	0.005754394	-0.116320298	-0.30096	-0.08303	0.25113	0.535569
CPXCR1	-0.078934193	-0.060845642	-0.07874	0.058004	-0.05866	-0.04136
CPXM1	-0.024726705	-0.038323706	0.015264	0.009105	-0.01007	-0.00648
CPXM2	-0.058558631	0.041329187	0.09967	-0.04367	-0.01469	-0.05949
CR1	-0.018068471	-0.168940511	-0.28569	-0.10002	0.203443	0.248373
CR1L	-0.03527994	-0.046390748	-0.19151	-0.0139	0.064072	0.124544
CR2	-0.02187286	-0.118392645	0.138416	-0.0501	0.136526	-0.23893
CR936796	-0.02069277	-0.025039111	0.122317	-0.0067	0.012445	-0.10708
CRABP1	-0.046615574	-0.028182892	0.02398	0.035437	-0.07568	-0.07249
CRABP2	0.001457496	-0.119977163	-0.0346	-0.00935	-0.06279	-0.07365
CRADD	0.008238863	0.077819411	0.004987	0.015347	-0.11103	-0.09084
CRAMP1L	0.000977852	0.259855934	0.010798	-0.04457	0.095109	-0.22872
CRAT	0.008661564	-0.032816677	-0.08032	-0.07921	0.114147	0.126675
CRB1	-0.062657636	-0.105970838	0.028987	0.014935	-0.0402	-0.05803
CRB2	-0.037348821	-0.037908736	-0.08453	0.053853	-0.12228	0.063249

CRB3	-0.017073351	-0.103067259	0.116613	-0.0203	-0.0565	-0.11397
CRBN	0.045674845	0.118298245	0.252586	-0.07109	0.1853	-0.14573
CRCP	0.023165912	0.074660715	0.028917	0.005392	-0.08986	-0.01337
CRCT1	-0.040387805	-0.048110114	-0.02344	0.022387	-0.05999	0.043753
CREB1	0.082644834	0.031166684	-0.12669	-0.03221	0.126488	-0.02198
CREB3	0.043964087	0.053671051	0.018854	0.059235	-0.21787	-0.02945
CREB3L1	-0.041495504	-0.010447631	0.081244	0.016032	-0.06165	-0.1273
CREB3L2	0.009243894	0.112139531	-0.0676	-0.02578	0.059481	0.160337
CREB3L3	-0.024010871	-0.011705375	-0.04583	0.080978	-0.22386	0.004526
CREB3L4	0.003531653	0.074288099	-0.04406	0.024763	-0.04647	-0.09756
CREB5	-0.008533818	-0.087505544	-0.27931	-0.02301	0.052799	0.311796
CREBBP	0.00365485	0.082258389	0.038739	-0.05793	0.130688	-0.15772
CREBL2	0.019631514	-0.061590674	0.137252	-0.09278	0.197372	0.222578
CREBRF	-0.008893013	-0.024286748	0.047546	-0.05991	0.158497	-0.16272
CREBZF	-0.033140724	0.108324733	0.165299	0.048215	-0.06016	-0.18053
CREG1	0.029071848	-0.112886722	-0.30732	-0.00811	0.036711	0.617963
CREG2	-0.041497591	-0.002564749	0.043426	-0.02645	0.079756	-0.07814
CRELD1	-0.000370323	0.080866776	0.15283	-0.01741	-0.00205	-0.09258
CRELD2	0.060320171	0.213124044	0.041697	0.030947	-0.10563	-0.15911
CREM	0.013134946	0.160276295	0.054617	0.081235	-0.17686	-0.19033
CRH	-0.033426774	-0.057682449	-0.03767	0.04041	-0.10541	0.026406
CRHBP	-0.026084064	-0.058495126	0.045448	-0.00497	0.024582	-0.04245
CRHR1	-0.007889002	-0.070505365	-0.06909	0.059675	-0.04052	-0.06255
CRHR2	-0.050946702	-0.053916752	-0.01456	0.031368	-0.0756	0.229431
CRIM1	0.015120538	0.041549918	-0.12922	-0.03391	0.059929	0.01758
CRIP1	0.029582039	0.016316596	0.058557	0.029429	-0.07965	0.15435
CRIP2	-0.011659646	-0.149471692	0.121357	-0.11741	0.351782	-0.1174
CRIP3	-0.01679467	-0.123489741	-0.16972	-0.01337	0.043527	-0.06252
CRIPAK	0.012017009	-0.03351171	0.069738	-0.05643	0.168856	-0.00885
CRIPT	0.023877064	0.10060429	-0.11727	-0.0139	0.031962	-0.11653
CRISP1	-0.079065256	-0.032196678	-0.01737	-0.01613	0.04166	-0.01516
CRISP2	-0.05452515	-0.066164076	0.189266	-0.05101	0.118627	-0.01928
CRISP3	-0.000965925	-0.034844166	0.384173	-0.00126	0.058883	-0.10294
CRISPLD1	-0.033528201	-0.108618229	-0.01534	-0.01895	0.070798	-0.0209
CRISPLD2	-0.003988296	-0.103862854	-0.30193	-0.09091	0.257043	0.555508
CRK	0.031627234	-0.032562178	-0.17115	-0.04322	0.029017	0.098335
CRKL	0.028500884	0.074885469	0.04421	0.030446	-0.11884	-0.19594
CRLF1	-0.028418439	-0.039469841	-0.11283	0.065276	-0.17597	0.094787
CRLF2	-0.013845569	-0.036924202	-0.04055	-0.00518	-0.04883	-0.05237
CRLF3	0.0863933	0.078126091	0.087228	0.000867	0.04612	-0.18732
CRLS1	0.036272611	0.015437736	0.081129	-0.02495	0.227417	0.143496
CRMP1	-0.022980553	-0.055021995	0.027859	0.020235	-0.12673	-0.06219
CRNDE	-0.012395994	-0.020736696	0.001459	0.05644	-0.18541	0.002782
CRNKL1	0.02020998	0.166795607	-0.15275	0.049819	-0.1321	-0.01421
CRNN	-0.038091154	-0.076933692	-0.02196	0.010943	-0.02707	-0.00021
CROCC	-0.010314223	-0.023734657	0.149132	-0.03645	0.131095	-0.07095
CROCCP2	0.007260801	-0.028952081	0.148834	-0.03352	-0.06344	-0.20894
CROCCP3	-0.033975653	0.047594695	0.074481	-0.01546	0.046151	-0.18197
CROT	-0.030978137	0.121306838	0.047539	0.096495	-0.101	-0.1245
CRP	-0.064705701	-0.036879986	-0.06955	0.005222	-0.00026	-0.10244
CRTAC1	-0.033138534	-0.06312905	0.073571	0.039029	-0.11415	-0.03714

CRTAM	-0.004093442	0.035312391	0.359827	-0.05595	0.131665	0.112724
CRTAP	-0.023772245	-0.158638095	-0.1095	-0.04464	0.172054	0.523528
CRTC1	-0.00010046	-0.125047369	0.066851	-0.03462	-0.01301	-0.09111
CRTC2	0.032004411	-0.078410579	0.06894	-0.00021	-0.02805	-0.11389
CRTC3	0.024986229	0.046189636	0.256459	-0.02969	0.087353	-0.11489
CRX	-0.05512592	-0.022720854	0.140914	0.011434	0.003318	0.02742
CRY1	0.018783626	0.168110835	0.240689	0.004844	-0.02771	-0.09419
CRY2	0.013640477	-0.020624085	0.12885	-0.04993	0.102142	-0.15128
CRYAA	-0.027197061	-0.046372661	-0.00132	0.014857	-0.0226	0.093163
CRYAB	-0.030807783	-0.014462214	-0.03875	0.023759	-0.06801	-0.06611
CRYBA1	-0.054383001	-0.049315058	-0.01596	0.022801	-0.06672	0.060659
CRYBA2	-0.038491004	0.014753989	-0.07185	0.009678	-0.00322	0.005895
CRYBA4	-0.027561546	0.037002411	-0.04319	0.05592	-0.13163	0.125113
CRYBB1	-0.028195182	-0.028591454	-0.06654	0.017081	-0.04544	0.138566
CRYBB2	-0.030395778	-0.058960127	-0.09838	0.027333	-0.12655	-0.00828
CRYBB2P1	-0.067669902	-0.171553196	-0.05207	0.005154	-0.02004	-0.03227
CRYBB3	-0.020947919	-0.056479095	0.039608	0.014322	-0.0561	0.011077
CRYBG3	0.005778856	-0.146453281	-0.21713	-0.00797	0.037589	0.349515
CRYGA	-0.032717015	-0.002766642	-0.00128	-0.00168	0.016689	-0.00161
CRYGB	-0.036611123	-0.081350575	-0.0895	0.029802	-0.06539	0.086498
CRYGC	-0.030220888	-0.10670314	0.031314	0.020086	-0.04871	-0.15133
CRYGD	-0.022410502	-0.085685318	-0.07643	0.013098	-0.04639	-0.1058
CRYGEP	-0.026212317	-0.062534213	-0.0587	0.033584	-0.10427	-0.01954
CRYGN	-0.03718208	-0.091005449	0.01521	-0.07136	0.23302	0.153557
CRYGS	-0.009474204	0.096628577	0.165976	-0.0202	0.083591	-0.08131
CRYL1	0.01461811	-0.026027861	-0.00165	-0.06157	0.187843	0.358714
CRYM	-0.01072545	-0.100716532	-0.13779	-0.02442	0.014446	-0.20168
CRYZ	0.012067681	0.096762048	0.145996	0.020007	-0.08341	-0.23635
CRYZL1	0.00244178	0.031433631	0.06872	-0.01255	-0.00036	0.03308
CS	0.08161152	-0.017122371	-0.11448	0.066761	-0.17304	-0.03812
CSAD	-0.009197449	-0.102052594	-0.10473	-0.00214	-0.01128	-0.04237
CSDC2	-0.028428209	-0.095566972	0.060979	0.02211	-0.08016	0.118309
CSDE1	0.047257633	0.058082594	0.115639	0.028906	-0.06881	-0.12785
CSE1L	0.027163466	0.057906124	0.043623	0.134307	-0.35082	-0.00822
CSF1	-0.023957242	-0.046259212	-0.04649	0.020041	-0.12528	-0.01676
CSF1R	0.008789003	-0.0868142	-0.32044	-0.07228	0.204217	0.530165
CSF2	-0.012255049	-0.016693424	-0.05284	0.012158	-0.10172	-0.02288
CSF2RA	-0.007384597	-0.146181261	-0.412	-0.07292	0.203528	0.418191
CSF2RB	0.016669156	-0.13943721	-0.40278	-0.03661	0.091631	0.168417
CSF3	-0.016490026	-0.069712343	-0.01596	-0.00832	-0.04162	0.003797
CSF3R	0.006417684	-0.091416225	-0.3196	-0.08316	0.237044	0.392126
CSGALNAC[illegible]	0.006984925	-0.100066664	-0.00261	-0.06293	0.158559	-0.24666
CSGALNAC[illegible]	0.028960269	0.042854317	-0.18409	0.0071	0.020313	0.409122
CSH1	-0.035821573	0.006615029	-0.04024	0.051318	-0.12377	-0.09775
CSHL1	-0.100092876	-0.136129766	-0.09631	0.051454	0.075557	-0.05472
CSK	0.058912652	0.019027975	-0.21722	0.003239	0.017669	0.178691
CSMD1	-0.061719891	-0.094254519	0.086427	0.021221	0.03497	0.060595
CSMD2	-0.037690626	0.017503283	-0.01339	0.060134	-0.08723	-0.01049
CSMD2-AS[illegible]	-0.025423573	-0.057749783	0.07634	-0.01633	-0.04561	-0.08513
CSMD3	-0.023106341	-0.065939665	0.059502	0.038451	-0.10562	-0.09637
CSN1S1	-0.021058974	-0.026359353	-0.05325	0.117171	-0.20628	-0.10051

CSN1S2AP	-0.054121901	-0.038436916	0.024685	-0.02339	0.089193	0.11591
CSN2	-0.040052628	-0.069248521	-0.01497	0.02908	-0.06566	-0.03845
CSN3	-0.032102478	-0.027691901	-0.00854	-0.02123	0.073456	0.098005
CSNK1A1	0.010462064	0.018219688	0.00498	-0.08864	0.027351	-0.01208
CSNK1A1P	-0.055577846	-0.049753817	-0.07292	-0.01474	0.039026	0.116836
CSNK1D	0.034301647	0.04295118	-0.26207	0.000238	-0.00093	0.221595
CSNK1E	0.009675332	0.009865651	0.09071	0.042411	-0.17864	-0.30382
CSNK1G1	-0.009435288	0.111218575	0.09853	0.061207	-0.15042	-0.04145
CSNK1G2	0.07506084	0.042358227	0.056404	-0.01378	0.051792	-0.21662
CSNK1G2-/	-0.028621723	-0.079993253	0.014346	-0.0053	-0.04832	-0.10411
CSNK1G3	0.043238318	0.146340686	-0.05063	0.028041	-0.04378	-0.14044
CSNK2A1	0.032781511	0.075458996	-0.01804	0.019345	-0.15337	-0.0751
CSNK2A2	0.032600024	0.015860564	0.143946	0.040587	-0.12328	0.080923
CSNK2B	0.089364767	0.091144149	-0.06824	0.10575	-0.26033	0.141237
CSPG4	-0.046341366	-0.034968114	-0.06533	0.03135	-0.06364	0.057989
CSPG4P1Y	-0.036429378	-0.081304325	-0.15252	-0.0065	0.037896	0.213215
CSPG4P5	-0.03244564	0.018885445	0.027876	-0.00257	-0.01321	0.004962
CSPG5	-0.049208391	-0.031532684	0.078542	0.001675	-0.02307	-0.09645
CSPP1	-0.045729852	0.089678358	0.234044	-0.02152	-0.01691	0.01524
CSRNP1	0.020008358	-0.085579398	-0.13408	-0.00126	0.016572	-0.03954
CSRNP2	-0.006212289	0.090008445	0.070653	0.008375	-0.06877	-0.20584
CSRNP3	-0.040604973	-0.0969704	-0.02871	-0.00499	0.004271	-0.06377
CSRP1	0.029096654	-0.041575365	-0.02443	0.070095	-0.2114	0.199017
CSRP2	-0.020359675	-0.038555732	0.030284	0.04595	-0.15305	-0.02727
CSRP3	-0.039653074	-0.012513819	0.018429	0.042195	-0.091	-0.03073
CST1	-0.03094442	-0.05080074	0.016576	0.069909	-0.1775	-0.00493
CST11	-0.051054336	-0.077877901	-0.18333	-0.0161	0.0792	-0.11862
CST13P	-0.053135243	-0.037888224	-0.02977	0.024333	-0.05449	0.134702
CST2	-0.03321175	-0.079336618	-0.12804	0.031814	-0.0703	0.184598
CST3	-0.021544659	-0.124691301	-0.16269	-0.06624	0.125643	0.327746
CST4	-0.006708095	-0.144854137	-0.22537	-0.00328	-0.01399	0.287985
CST5	-0.030253204	-0.045456596	-0.14429	0.026617	-0.04982	0.194612
CST6	-0.012267339	-0.035507627	-0.00702	-0.01306	0.015795	0.004001
CST7	0.019377458	0.277703393	0.39793	0.022108	-0.0529	-0.38477
CST8	-0.031588578	-0.128928484	-0.01514	0.028914	-0.0955	0.031699
CST9	-0.034007466	-0.036768678	-0.1268	0.027288	-0.06249	0.026328
CST9L	-0.044872247	-0.021270948	-0.04019	-0.01155	0.068093	0.020713
CSTA	0.011701551	-0.117320903	-0.29146	-0.06739	0.191282	0.53921
CSTB	0.029018593	0.012168315	-0.00035	-0.03609	0.120487	0.34024
CSTF1	0.005188822	0.084100258	-0.01231	0.068875	-0.21109	-0.14587
CSTF2	0.001785669	0.01657139	-0.03098	0.099279	-0.3108	0.050032
CSTF2T	0.025557836	0.181934027	0.093487	0.019225	-0.07761	-0.11801
CSTF3	0.002238784	-0.090899772	0.23403	0.012273	-0.08384	-0.09197
CSTF3-AS	-0.091912484	-0.119637777	0.023205	-0.00489	0.05888	-0.01757
CSTL1	-0.049892826	-0.022529595	-0.08298	0.021197	-0.03272	0.136931
CT55	-0.02792024	-0.033388301	0.008377	0.007445	-0.01662	-0.07452
CT62	-0.039927891	-0.035428291	-0.00076	0.004686	0.017402	-0.02368
CT83	-0.061076087	-0.051351406	-0.07039	0.008585	-0.02277	0.014113
CTA-246H	-0.033593683	0.001589015	-0.0476	0.02058	-0.08804	-0.0304
CTA-250D	-0.007328967	-0.116607762	########	-0.07046	0.182076	-0.08744
CTA-254O	-0.051959193	-0.09760869	-0.08003	-0.01585	0.076922	-0.00445

CTA-268H	-0.034757161	-0.070255835	0.018089	0.000924	0.013394	-0.05831
CTA-280A	-0.033631853	-0.018074621	-0.06483	0.027991	-0.09322	0.04083
CTA-292E	-0.006365161	0.090820225	0.21471	-0.00841	0.04843	0.024752
CTA-29F1	0.000172059	-0.023918893	0.258001	0.017807	-0.0305	-0.02819
CTA-331P	-0.060260638	-0.029759664	0.00608	0.007524	-0.01829	0.424716
CTA-384D	-0.018353082	-0.065462301	-0.11851	-0.01154	0.017614	0.281888
CTA-390C	-0.016899571	-0.060881254	0.082726	0.004869	-0.00915	-0.12015
CTA-445C	0.01513051	0.260766318	0.278616	-0.0429	0.117912	-0.05718
CTAG2	-0.041211993	-0.029549028	-0.12159	0.007949	0.003284	-0.00078
CTAGE1	-0.04343368	0.00880478	0.034482	0.000125	0.026535	-0.03211
CTAGE11P	-0.033858786	-0.139402698	-0.02574	0.002731	0.002304	-0.01163
CTAGE5	-0.018998435	0.161930503	-0.06995	-0.07508	0.023009	-0.18683
CTB-102L	-0.030269871	-0.068227245	0.045108	0.029039	-0.09902	-0.07478
CTB-113P	-0.024822992	-0.049690518	0.080109	-0.01046	0.003528	-0.07815
CTB-119C	-0.020757555	0.02843194	0.208062	0.004939	-0.00312	-0.35885
CTB-12A1	0.040438799	0.027728379	0.197683	0.0012	-0.04266	0.033833
CTB-1202.	-0.062631325	-0.019133927	-0.04038	-0.00669	0.030666	0.009932
CTB-167B	-0.002360864	-0.044034185	-0.24203	-0.01028	0.054071	0.184188
CTB-174D	-0.030289283	-0.087946123	-0.0046	-0.01367	0.042163	-0.01082
CTB-176F	-0.037353562	-0.025356461	0.030169	0.037508	-0.10125	-0.01132
CTB-181H	-0.016178163	-0.043201905	-0.18206	0.017185	-0.03705	0.065758
CTB-25B1	-0.008456926	-0.067897428	-0.04743	0.031047	-0.09202	0.134269
CTB-3102	0.016735471	0.108468347	0.099225	-0.0384	0.131488	-0.08475
CTB-3102	-0.037165096	-0.074761055	-0.00434	-0.00428	0.040981	0.023085
CTB-43E1	-0.043183361	-0.07333594	-0.04394	0.014198	-0.0109	0.039747
CTB-50L1	-0.004335145	-0.064254709	-0.16967	-0.05146	0.103463	0.133828
CTB-78F1.	-0.034399915	0.016984308	0.027974	-0.01289	0.061093	-0.06347
CTB-92J2	-0.018519123	-0.029021339	0.056556	0.015381	-0.07534	-0.13224
CTBP1	0.040478424	0.288354885	0.036116	0.022978	-0.05843	-0.12016
CTBP1-AS	-0.032412157	0.03388299	-0.07343	0.009753	-0.00102	-0.0497
CTBP1-AS	-0.007372188	-0.03496758	0.105481	0.005413	-0.08828	-0.1949
CTBP2	0.024357927	0.250385535	-0.37551	-0.14117	0.371638	0.258351
CTBS	0.026889717	-0.062123422	-0.38735	0.010333	-0.01215	0.21995
CTC-338M	-0.005331092	-0.012154564	-0.00748	0.041487	-0.10609	0.050293
CTC-360P	-0.031778224	-0.072267353	0.00105	0.017414	-0.08202	-0.04506
CTC-384G	-0.047406229	-0.058468365	0.054045	0.004804	0.057434	-0.15135
CTC-425F	0.0570587	0.102203257	-0.04066	0.012499	-0.04975	-0.08251
CTC-428G	0.014041572	-0.054320871	-0.14198	-0.06609	0.223475	0.115594
CTC-428G	-0.038555947	-0.080594135	-0.1435	-0.06823	0.19551	0.364411
CTC-429P	0.025624632	-0.029972161	0.200512	-0.06089	0.178485	-0.00081
CTC-436K	-0.045153862	-0.025176828	-0.00295	0.026023	-0.04553	-0.11487
CTC-444N	0.009609518	0.097274979	0.12898	0.040851	-0.10077	-0.09455
CTC-459F	-0.000512127	-0.07310541	0.030713	-0.02268	0.009296	-0.00723
CTC-462L	-0.065476621	-0.054501298	0.004341	-0.01069	0.052216	0.047437
CTC-471C	-0.050403815	-0.080128453	-0.11467	0.004146	0.014528	0.00858
CTC-471F	-0.058498471	-0.056656701	0.228639	-0.00487	0.013738	-0.17693
CTC-471J	-0.020203392	0.157910967	0.1149	-0.03672	0.117893	-0.17838
CTC-510F	-0.003922169	-0.116847286	-0.29509	-0.01516	0.034216	0.391506
CTC-523E	-0.00996342	0.284549595	0.112465	-0.0448	0.136783	-0.19948
CTC-527H	-0.033696071	0.008029767	0.117598	0.015889	-0.0507	0.131586
CTC-550B	-0.027761445	0.001952349	0.01626	0.0279	-0.09306	-0.00183

CTC1	-0.033344406	0.013275797	0.296539	-0.07017	0.211168	-0.24623
CTCF	0.077128254	0.144011093	0.049054	0.010627	-0.04592	-0.54627
CTCFL	-0.044427234	-0.032410555	0.027695	0.006368	-0.00959	-0.06285
CTD-2002	-0.045769561	-0.078761704	0.059002	-0.00848	0.034214	-0.08932
CTD-2008I	-0.026567222	-0.039236611	-0.02163	0.008918	-0.10112	0.039741
CTD-2012I	-0.024105807	-0.020889002	-0.11589	-0.00703	0.007247	-0.06892
CTD-2021I	-0.035952457	0.063816147	-0.1449	0.033724	-0.08298	-0.01339
CTD-2033(	-0.036486745	-0.059455167	-0.07241	-0.03921	0.151935	0.09801
CTD-2035I	-0.022092757	-0.060300926	0.113356	-0.01713	0.068002	-0.02939
CTD-2076M	-0.021480811	-0.010083744	0.002919	-0.02591	0.021155	-0.09728
CTD-2083I	-0.026659221	-0.075436315	0.054846	0.005979	-0.00093	0.102766
CTD-2083I	-0.026836974	-0.067768703	0.053078	0.011762	-0.00227	-0.02406
CTD-2118I	-0.068045154	-0.049829496	-0.07302	-0.01172	0.036323	0.153109
CTD-2124I	-0.019817135	0.109582958	0.118826	0.004482	0.003258	-0.17575
CTD-2130(	-0.035177584	-0.070994469	-0.02355	0.038993	-0.09513	0.05502
CTD-2165I	-0.003646909	-0.068544605	-0.03498	-0.01739	0.101486	0.220898
CTD-2194I	-0.048462014	0.036930445	-0.03921	-0.02321	0.079343	0.013447
CTD-2196I	-0.025748038	0.04008676	0.05855	-0.00226	0.021061	0.044733
CTD-2251I	-0.04270373	-0.058101536	-0.0261	0.018227	-0.02728	0.04835
CTD-2256I	-0.022508865	-0.054801609	0.066774	0.07114	-0.20427	0.009942
CTD-2269I	-0.032346393	-0.065607467	-0.20697	0.021934	-0.05007	0.127979
CTD-2281I	-0.044768648	-0.035121779	-0.04115	0.010655	0.011023	-0.04507
CTD-2286I	-0.01934585	0.017677375	-0.01082	0.01246	-0.00787	0.102568
CTD-2287(	-0.032759884	-0.054035139	0.037383	-0.01554	0.025801	-0.07933
CTD-2292M	-0.003161922	0.092499195	0.19342	-0.00265	0.036495	-0.18581
CTD-2293I	-0.02559963	0.023012742	0.116811	-0.01293	0.046194	-0.11488
CTD-2297I	-0.029645645	0.016554478	-0.16622	-0.00568	0.029979	0.020235
CTD-2302I	-0.032867136	-0.023302163	0.077643	0.030447	-0.08063	0.028167
CTD-2310I	-0.081012304	-0.017111205	0.04317	0.008081	-0.03399	-0.02403
CTD-2313	-0.039551857	-0.079813847	-0.05781	0.037123	-0.08618	-0.03163
CTD-2325/	-0.025455473	-0.025871722	0.126132	-0.02312	0.087525	-0.09144
CTD-2350	-0.05574085	-0.069269835	-0.02167	0.02246	-0.07249	0.143014
CTD-2366I	-0.028411912	0.014725655	0.057306	-0.01884	0.051898	-0.13651
CTD-2373	-0.065661752	0.017403672	0.009298	-0.01524	0.026236	-0.14296
CTD-2377I	-0.026674834	-0.080302909	0.185407	0.052199	-0.17602	-0.02919
CTD-2520	-0.043753198	-0.069842504	-0.16109	0.005932	0.013281	0.15796
CTD-2528I	-0.017048768	0.076837705	0.217996	-0.10899	0.315639	-0.23193
CTD-2534	-0.042520025	-0.010467845	0.038822	0.048142	-0.1077	-0.15294
CTD-2537	-0.03072313	-0.00197275	-0.0858	0.004969	-0.05179	0.027568
CTD-2537	-0.034468788	0.008997991	-0.01316	0.002554	0.011256	-0.03431
CTD-2540I	-0.050607282	-0.032151722	-0.02329	-0.02149	0.038503	0.011767
CTD-2541	-0.079377546	-0.08256236	-0.07138	-0.0038	0.045947	-0.18612
CTD-2541M	-0.018558228	-0.080960036	0.016639	-0.11493	0.326399	-0.07301
CTD-2542I	-0.03249756	-0.053388639	-0.01831	0.017827	-0.05886	-0.11788
CTD-2547I	-0.012005203	0.099484094	-0.06506	0.04912	-0.12848	-0.06248
CTD-2553(	-0.016261498	-0.063575795	-0.12209	0.000638	-0.04854	-0.18576
CTD-2554(	-0.038489293	-0.057424676	-0.01107	0.010236	-0.01551	-0.07236
CTD-2561I	-0.025791604	0.022493546	0.054385	0.015275	-0.03001	-0.0471
CTD-2587I	-0.027378109	-0.069155274	-0.00025	-0.00175	-0.00843	0.015575
CTD-2587M	-0.036005223	-0.0488708	-0.06584	-0.00232	0.03483	0.011101
CTD-2611(	-0.030020974	0.032769144	0.088918	-0.00348	0.025675	-0.09274

CTD-2616.	-0.034078583	0.062301987	-0.13221	0.027212	-0.0475	0.096469
CTD-2619.	-0.023416574	-0.046175127	0.055853	-0.00755	-0.06443	-0.11918
CTD-2619.	-0.027041514	0.032240534	0.055666	-0.02564	0.091368	-0.09739
CTD-2639I	-0.027945909	-0.037485872	0.109866	0.037926	-0.08098	0.009669
CTD-3025N	-0.024274018	0.026546112	0.106869	0.065747	-0.13556	-0.08385
CTD-3028N	-0.031622293	-0.070678756	-0.07305	0.011533	-0.01553	0.045852
CTD-3064M	-0.015805807	-0.057756459	-0.02162	-0.01264	-0.06114	0.007529
CTD-3080I	-0.048900076	-0.045191752	-0.11684	0.008055	-0.01282	0.130241
CTD-3092A	0.023814009	0.071708964	0.073247	-0.14785	0.396018	-0.00339
CTD-3126I	-0.021319959	-0.039765568	-0.10154	-0.02212	-0.02903	-0.10435
CTD-3193(	-0.035448613	-0.005501237	-0.08684	0.019206	-0.03506	0.07521
CTDNEP1	0.097681552	-0.025286076	-0.05058	0.061381	-0.12829	-0.05981
CTDP1	0.025024944	-0.019083787	-0.03504	-0.03376	0.050481	0.04037
CTDSP1	0.045518173	0.117534591	-0.05702	-0.0496	0.138858	0.037239
CTDSP2	0.046678101	-0.011845071	-0.0582	-0.06743	0.182791	-0.04331
CTDSPL	-0.00253898	-0.018056447	0.072823	-0.3028	0.400038	0.09021
CTDSPL2	0.029338667	0.08735298	0.082784	0.063907	-0.10828	-0.18291
CTF1	-0.022721639	-0.043010653	-0.05443	-0.0122	-0.01322	0.001444
CTGF	-0.038447853	-0.038530026	-0.13275	-0.03075	0.110507	-0.07338
CTH	-0.008965037	-0.004786846	0.081049	0.09074	-0.23585	-0.07555
CTHRC1	-0.029827893	-0.029621249	-0.01121	-0.02028	-0.01513	-0.04964
CTIF	-0.022772272	-0.084240388	-0.19501	-0.04298	0.038053	0.281035
CTLA4	0.000465879	-0.05424122	0.062182	0.015667	-0.10562	-0.06296
CTNNA1	0.008100793	-0.034843311	-0.37857	-0.07775	0.218675	0.373874
CTNNA2	-0.020479909	-0.068544778	0.059494	-0.00477	-0.00493	-0.05531
CTNNA3	-0.055186702	-0.048989955	-0.02219	0.061959	-0.19566	0.049028
CTNNAL1	0.0015509	0.026479156	0.00177	0.08044	-0.25381	-0.09755
CTNNAP1	-0.036699623	0.040972306	-0.12132	-0.00557	0.047238	0.098202
CTNNB1	0.032689825	0.021249572	-0.07245	-0.01618	0.063191	-0.13227
CTNNBIP1	0.018690745	0.024810422	-0.07059	-0.0513	0.09301	0.114583
CTNNBL1	0.048811775	0.017542128	-0.17828	0.056472	-0.15838	0.057199
CTNND2	-0.029201186	-0.044705728	0.023428	-0.02729	0.025431	0.057058
CTNS	0.038239034	-0.042563506	-0.16726	-0.00136	0.003521	0.039476
CTPS1	0.005960101	0.026055481	-0.00772	0.085057	-0.24542	-0.12388
CTPS2	-0.006581062	-0.006303125	0.09136	0.027779	-0.04699	-0.05609
CTR9	0.066154088	0.202299444	0.104105	0.034979	-0.08312	-0.27102
CTRB2	-0.035478742	-0.058529383	0.012921	0.010738	-0.06363	0.033399
CTRC	-0.022730116	0.087024863	-0.08093	0.001769	-0.00718	0.013399
CTRL	-0.008745772	-0.04691517	0.000392	-0.00081	0.028734	0.027818
CTSA	0.032393712	-0.077371644	-0.10754	-0.05032	0.168292	0.447372
CTSB	0.030051143	-0.096344592	-0.35195	-0.05009	0.167835	0.530719
CTSC	0.042435668	0.177254317	-0.0133	0.061862	-0.1869	0.117779
CTSD	0.019947935	0.090020382	-0.20044	-0.0158	0.026517	0.574963
CTSE	-0.031782649	-0.04217288	0.028537	-0.00297	-0.04439	-0.10696
CTSF	0.006997261	0.059365757	0.273356	-0.01578	0.024317	-0.26269
CTSG	-0.006756924	-0.114622009	0.051245	0.001096	0.003368	-0.13673
CTSH	0.019319021	-0.1508443	-0.28302	-0.0571	0.141968	0.46792
CTSK	0.008670218	-0.084303301	-0.06596	-0.04808	0.141997	0.290929
CTSL	0.018241985	-0.061314504	-0.13576	-0.04341	0.086979	0.284831
CTSLP8	-0.015535318	-0.035480696	-0.14305	-0.00743	0.039785	0.274928
CTSO	0.02214083	0.080765158	0.020631	-0.05661	0.205483	0.217935

CTSS	0.021846157	-0.063211703	-0.39996	-0.10564	0.317465	0.46319
CTSV	-0.010475158	-0.078089789	-0.19085	-0.02759	0.044555	0.001693
CTSW	0.012068444	0.240349226	0.481944	0.002115	-0.03075	-0.31306
CTSZ	0.018803164	-0.148498786	-0.5107	-0.04063	0.093625	0.384574
CTTN	-0.012288675	0.003782539	-0.0538	-0.20096	0.590897	0.063197
CTTNBP2	-0.064401974	-0.060719602	-0.05992	0.012468	-0.02626	0.167286
CTTNBP2NI	-0.02570345	-0.078645836	-0.09314	-0.02023	0.060542	0.114278
CTU2	-0.004699286	-0.10639272	-0.05625	-0.00731	-0.0691	0.090262
CTXN1	-0.033726684	-0.063280402	-0.09478	0.001595	-0.00015	0.139263
CTXN3	-0.041370376	-0.058464609	-0.10191	0.001981	0.003421	-0.15562
CUBN	-0.013538193	-0.083114269	0.328921	-0.00535	0.02752	-0.29266
CUEDC1	-0.003182268	-0.138961115	-0.23864	-0.04021	0.113836	0.174881
CUEDC2	0.040616112	-0.006265029	-0.01438	0.079041	-0.22101	0.262451
CUL1	0.026491441	0.019075557	0.097031	0.030644	-0.09478	-0.0013
CUL2	0.014140086	0.093157038	0.085746	0.027303	-0.02999	0.026133
CUL3	0.002808933	0.102793647	-0.12055	0.006487	0.029781	-0.05562
CUL4A	0.022415383	0.078901065	0.142046	0.077403	-0.13766	-0.05212
CUL4B	0.016772943	-0.019717743	-0.02907	-0.00653	0.010619	-0.11867
CUL5	0.017844594	0.033003012	0.026837	0.026648	-0.0622	0.154426
CUL7	-0.056092348	-0.104141382	0.026549	0.007763	-0.13403	0.03343
CUL9	0.014752491	-0.151848039	0.088535	0.020192	-0.06548	-0.00182
CUTA	0.043099798	0.07502474	0.318701	0.045002	-0.13215	-0.05594
CUTC	0.051450105	0.028696288	0.099715	0.006086	-0.06562	-0.22176
CUX1	0.016881888	-0.053171907	-0.1888	-0.09391	0.233077	0.357113
CUX2	-0.005632543	-0.07631118	-0.06366	-0.00753	0.036729	-0.16343
CUZD1	-0.012027441	0.058755127	0.198586	-0.00571	0.037842	-0.13885
CWC15	0.105139014	0.106610543	0.149246	0.075719	-0.16883	0.036951
CWC22	0.036913239	0.114773935	-0.07026	0.025633	-0.08559	-0.09655
CWC25	0.036983909	0.010734251	-0.15433	0.004657	-0.02622	-0.06163
CWC27	0.033791235	0.060463053	0.131525	0.033708	-0.0828	0.167274
CWF19L1	0.024313292	0.012541792	0.098743	0.024672	-0.13666	-0.09386
CWF19L2	0.013225356	0.091211567	0.051681	-0.00525	-0.00453	-0.15642
CWH43	-0.06758854	-0.075431735	-0.07423	-0.00442	0.017063	-0.02709
CX3CL1	-0.041843117	-0.090503226	0.023055	0.037044	-0.08998	0.010508
CX3CR1	0.00717001	0.248225615	0.080322	-0.09381	0.269665	0.22942
CXADR	-0.063532382	-0.013271559	0.046709	0.132394	-0.14662	-0.06969
CXCL1	-0.010486665	-0.032788504	-0.11336	-0.0169	0.009349	-0.04074
CXCL10	-0.008772659	-0.03272442	-0.17982	0.000193	-0.01406	0.372207
CXCL11	-0.025962687	-0.032332162	-0.03618	-0.04574	0.006931	0.043563
CXCL12	-0.051535284	-0.00021607	-0.05322	-0.0204	0.01677	-0.03594
CXCL13	-0.027367717	-0.046170741	-0.01095	-0.01013	-0.02197	-0.04183
CXCL14	-0.103167279	-0.030166874	0.020824	0.029747	-0.00751	-0.00415
CXCL16	0.008826375	-0.148275627	-0.32232	-0.06842	0.188852	0.357022
CXCL17	-0.040325323	-0.046214261	0.056778	0.032073	-0.08093	-0.05169
CXCL2	-0.027931452	0.000514657	0.083447	0.003373	-0.01151	0.041284
CXCL3	-0.01352309	-0.020709941	-0.02573	0.016984	-0.06945	0.018794
CXCL5	0.025294811	0.004267361	0.028888	-0.31744	0.705582	0.058505
CXCL6	-0.021872348	-0.050853685	-0.10121	-0.01947	0.003898	-0.08317
CXCL8	0.002798469	-0.033697726	-0.16524	0.013667	-0.03176	0.044119
CXCL9	-0.007643119	-0.040814478	-0.1005	-0.00583	0.005734	0.101053
CXCR1	0.00322838	0.039563876	-0.1849	-0.00434	-0.00025	-0.10986

CXCR2	0.004922216	0.122914855	-0.29063	-0.0792	0.221996	0.103329
CXCR3	0.007412476	-0.013943134	0.18757	0.014843	-0.07043	-0.31973
CXCR4	0.028984713	0.000571102	0.09773	0.040769	-0.11204	-0.45946
CXCR5	-0.011684125	-0.088645834	-0.07727	-0.0467	0.107812	-0.14099
CXCR6	-0.000289671	0.023243103	0.309577	-0.01574	0.007661	-0.14952
CXorf21	0.018859772	-0.162560932	-0.33078	-0.02886	0.075098	0.127764
CXorf22	-0.029477069	-0.053868347	-0.04885	0.062735	-0.15753	-0.02354
CXorf23	-0.006368202	0.057767524	-0.09942	0.01625	-0.0165	-0.03152
CXorf24	-0.011782759	-0.009283272	-0.02394	-0.03838	0.125707	0.231322
CXorf30	-0.051908127	-0.018899999	-0.01229	0.046326	-0.11902	0.032035
CXorf31	-0.028148857	-0.058368426	-0.0832	0.022102	-0.04866	-0.17725
CXorf36	-0.029804854	-0.064383325	-0.04203	0.031195	-0.08741	-0.10621
CXorf38	0.054393877	0.095930545	-0.26059	0.005695	-0.03283	0.415146
CXorf40A	0.060722707	0.17029479	0.151346	0.065946	-0.19181	-0.07484
CXorf40B	0.067821546	0.191446504	0.136176	0.056565	-0.1777	-0.10829
CXorf56	-0.004341797	-0.00715945	-0.11631	0.077254	-0.18307	0.171519
CXorf57	-0.019548367	0.013727826	0.254275	-0.02754	0.085515	-0.42938
CXorf58	-0.049689602	-0.022996207	0.035579	-0.01209	0.076009	0.006649
CXorf65	0.006959007	0.115463475	0.310063	-0.02928	0.096774	-0.48723
CXorf67	-0.035143701	-0.047386751	0.015997	0.008721	-0.00618	-0.14494
CXXC1	0.065948712	0.04222163	0.117825	0.024884	-0.07442	-0.15638
CXXC1P1	-0.031454211	-0.063387579	0.01449	-0.00545	0.01563	0.007209
CXXC4	-0.030848493	0.078942728	-0.00882	-0.03892	0.119913	-0.08207
CXXC5	0.025953065	0.176113463	-0.16682	-0.06259	0.175042	-0.05529
CYAT1	-0.037136079	-0.122930385	0.067822	-0.04372	0.128748	-0.07925
CYB561	0.013982258	0.067484238	0.196724	-0.02544	0.136864	-0.00729
CYB561A3	0.025353955	-0.110062342	-0.15556	-0.01372	0.026017	-0.10115
CYB561D1	0.049985921	0.124047741	0.173902	-0.061	0.112621	-0.32274
CYB561D2	0.020102156	0.027881408	0.302119	-0.07011	0.168049	-0.18623
CYB5A	0.017660344	0.038600938	0.187041	-0.04797	0.115562	-0.03461
CYB5B	0.023493198	0.219269542	0.03877	0.087944	-0.17578	-0.08894
CYB5D1	-0.000959334	-0.032240384	0.081867	0.018547	-0.1011	-0.2488
CYB5D2	0.001059693	-0.043491391	0.015181	-0.00044	0.002909	0.175168
CYB5R1	0.017756272	-0.101778932	-0.09365	-0.09867	0.251581	0.160221
CYB5R2	-0.032556088	-0.060183591	-0.17578	-0.01299	0.053886	0.156921
CYB5R3	0.053652102	-0.020269794	-0.22932	-0.03351	0.085963	0.076226
CYB5R4	0.022769684	-0.060355236	-0.41038	0.006016	0.019981	0.24974
CYB5RL	-0.036161768	-0.051675046	-0.04362	0.104966	-0.2797	0.079709
CYBA	0.02885793	0.091291962	-0.11453	0.014614	-0.0338	0.069569
CYBB	0.007641373	-0.200077773	-0.41381	-0.10607	0.321777	0.485933
CYBRD1	0.002256639	0.066933966	-0.32857	-0.09227	0.267642	0.527535
CYC1	0.049562535	0.05886082	-0.05007	0.107604	-0.30861	0.331057
CYCS	-0.018569225	0.042348726	0.250508	0.006593	-0.05728	-0.19135
CYFIP1	0.019682416	-0.084523936	-0.2817	-0.08724	0.239993	0.571873
CYFIP2	0.028136649	0.101317897	0.277187	0.002473	-0.01179	-0.58102
CYGB	-0.015944981	-0.106601232	-0.0002	0.014248	-0.09669	-0.00039
CYHR1	0.029725686	-0.060068569	-0.07394	0.013444	-0.08959	0.174058
CYLC1	-0.028379221	-0.048334004	0.042594	-0.00514	0.047568	-0.00568
CYLC2	-0.028961282	0.021610231	0.071352	0.015778	-0.02728	-0.15208
CYLD	0.034023629	0.001368754	0.261842	-0.03268	0.081246	-0.48533
CYMP	-0.039764033	-0.105348563	0.074077	0.002758	0.017386	0.134488

CYP11A1	-0.034295754	-0.057827687	-0.00529	0.012733	-0.00389	-0.00628
CYP11B1	-0.058765937	-0.162152971	-0.05064	-0.00208	0.05021	0.101797
CYP11B2	-0.021217508	-0.112560981	0.021971	-0.02432	0.008721	0.055227
CYP17A1	-0.058071306	-0.108102292	-0.07681	-0.00337	0.016504	0.014963
CYP19A1	-0.075623637	0.013433823	0.025142	0.080821	-0.04575	-0.12344
CYP1A1	-0.01677236	0.013549984	-0.08096	-0.0158	-0.00769	-0.04429
CYP1A2	-0.022554786	-0.034086708	0.040114	-0.00945	-0.05028	-0.05273
CYP1B1	-0.002427929	-0.05281301	-0.21487	-0.08864	0.208273	0.599319
CYP1B1-AS	-0.046412009	-0.042208011	-0.07729	-0.02317	0.086679	0.349517
CYP20A1	0.026388435	0.02386496	0.157567	-0.00185	5.64E-05	0.010042
CYP24A1	-0.060233724	-0.063101264	-0.01986	-0.01307	0.024649	-0.0426
CYP26A1	-0.016921184	-0.029590198	-0.04074	0.011213	-0.05965	0.000893
CYP26B1	-0.04420823	-0.02112932	0.027152	0.01786	-0.0444	-0.10483
CYP27A1	0.017483976	-0.111351827	-0.14477	-0.06049	0.125205	0.405313
CYP27B1	-0.012713138	-0.026560524	0.019806	0.020749	-0.0781	-0.06084
CYP27C1	-0.064597128	-0.033685413	-0.05473	0.008297	-0.043	0.031449
CYP2A13	-0.025279291	-0.062851979	0.046917	0.01874	-0.06904	0.056597
CYP2A6	-0.05302387	-0.037601296	-0.03536	-0.03831	-0.02722	0.009856
CYP2A7	-0.028186363	-0.005468619	-0.07421	-0.00294	-0.02633	-0.15232
CYP2A7P1	-0.041587028	-0.049733413	-0.04415	0.025741	-0.05995	0.053528
CYP2B6	-0.025429066	-0.056694115	-0.0254	-0.01108	0.020582	-0.06201
CYP2B7P	-0.025416567	-0.032030587	-0.02921	-0.00345	-0.04603	-0.06559
CYP2C18	-0.060720248	-0.10460296	0.059061	0.000281	0.021828	0.018854
CYP2C19	-0.04646939	-0.100507881	-0.10551	0.021735	-0.03699	0.098
CYP2C8	-0.047000277	-0.062750193	-0.09025	-0.02621	0.079559	-0.10586
CYP2C9	-0.029190409	-0.087835423	0.025301	0.062761	-0.01201	-0.0894
CYP2D6	-0.025471795	-0.074283026	-0.05437	-0.01	-0.07454	-0.11436
CYP2E1	0.002838391	-0.004430453	0.05551	0.008661	0.062791	-0.1775
CYP2F1	-0.045004095	-0.013648929	-0.12028	-0.01209	0.064287	0.126697
CYP2J2	-0.02842049	-0.157053067	0.333235	-0.00856	0.034574	-0.10105
CYP2R1	-0.014070112	0.146911074	0.224271	0.01465	-0.02596	-0.21258
CYP2S1	-0.014133979	-0.057407412	-0.20083	-0.07997	0.223372	0.256243
CYP2U1	-0.025705544	-0.024162231	-0.01575	-0.10302	0.223095	-0.14898
CYP2W1	-0.019122027	-0.085627154	-0.13142	-0.00216	-0.03833	-0.0418
CYP39A1	-0.057166648	-0.063704861	0.018514	-0.01545	0.035957	0.022039
CYP3A4	-0.011211773	-0.007582995	0.120576	0.025339	0.007796	-0.12096
CYP3A43	-0.048930569	0.001667733	-0.08683	0.044729	-0.0038	0.009667
CYP3A5	-0.047014776	-0.049679421	0.069615	0.050013	0.045549	0.038187
CYP3A7-C'	-0.049661736	-0.063877735	-0.13664	0.008955	0.008674	0.066877
CYP46A1	-0.008153052	-0.079331937	-0.08415	0.002378	0.0099	-0.1869
CYP4A11	-0.029873304	-0.02536385	0.024152	-0.0076	-0.0566	-0.09633
CYP4B1	-0.060017992	-0.068138018	0.016196	0.047587	-0.15817	0.095274
CYP4F11	-0.051831116	-0.014939053	-0.07004	-0.01706	0.038088	0.012451
CYP4F12	-0.00946584	-0.070138509	-0.02479	0.000464	-0.05644	-0.16699
CYP4F2	-0.016931737	-0.050399761	-0.14144	0.047259	-0.15375	-0.06364
CYP4F22	-0.021297674	-0.044233815	-0.01305	0.011019	-0.02339	0.143705
CYP4F3	-0.004136608	-0.067554848	-0.06749	0.043961	-0.10191	-0.04454
CYP4F30P	-0.062380438	-0.043027785	0.004648	-0.04927	0.132143	0.072348
CYP4F62P	-0.030409813	-0.028988702	-0.10732	0.013279	-0.02496	-0.12256
CYP4F8	-0.034015268	0.00061269	-0.08598	-0.00735	0.03996	0.04523
CYP4V2	0.017639212	0.078257168	0.159577	0.026607	0.065274	-0.0128

CYP4X1	-0.070948961	0.004487349	0.147967	0.033145	-0.06668	-0.02437
CYP4Z1	-0.037726795	-0.039109633	0.10427	-0.00315	0.031171	-0.07157
CYP4Z2P	-0.040712967	-0.053339628	0.089622	0.012458	-0.0338	0.050154
CYP51A1-/	-0.055021957	-0.059575502	0.055449	-0.00383	0.031261	-0.06371
CYP7A1	-0.081078942	-0.041161127	-0.03951	-0.03543	0.059536	-0.05809
CYP7B1	-0.018899536	-0.031250927	-0.00146	-0.01347	0.011887	-0.06223
CYP8B1	-0.026806376	-0.032607994	-0.06293	-0.00266	-0.00939	0.034628
CYR61	-0.046835516	-0.005760397	-0.0178	0.003592	0.020189	-0.02466
CYS1	-0.030696634	0.014003514	-0.01403	0.01002	-0.01454	-0.0353
CYSLTR1	0.000648471	-0.02760632	-0.45579	-0.1025	0.262197	0.125636
CYSLTR2	-0.016834881	-0.032914613	-0.10484	-0.02427	0.03792	0.054194
CYSRT1	-0.025255632	-0.079814053	-0.13227	0.041422	-0.12101	-0.06504
CYSTM1	0.022900038	-0.13056591	-0.30272	0.04105	-0.10261	0.151695
CYTH1	0.027084199	0.058687368	0.094356	-0.05742	0.138002	-0.21507
CYTH2	-0.065148766	-0.10166808	-0.03249	-0.00855	-0.08999	-0.17232
CYTH3	-0.011835722	-0.019429737	0.017288	-0.18281	0.000519	0.043359
CYTH4	-0.035832386	-0.108454042	-0.04328	-0.07839	0.14115	-0.07617
CYTIP	0.052604089	0.122463117	0.136525	0.066284	-0.1598	-0.2557
CYTL1	-0.011427986	0.147221464	-0.03519	-0.02096	0.076445	-0.09161
CYYR1	-0.005376346	-0.074541377	-0.08553	-0.01968	0.069025	-0.16953
D21S2088I	-0.043209368	-0.063040457	0.022588	-0.00776	-0.02289	-0.11413
D21S2090I	-0.04161186	-0.048394089	-0.08413	0.014468	-0.01303	0.223434
D2HGDH	0.020725437	-0.069109535	0.210161	0.016295	-0.06036	-0.07373
DAAM1	-0.031109269	0.018387275	-0.1126	-0.07485	0.121997	-0.08153
DAAM2	-0.021364674	-0.041515046	-0.14407	0.0035	-0.05221	-0.13263
DAB1	-0.018097571	-0.031250543	0.06234	0.084889	-0.20503	-0.02829
DAB1-AS1	-0.052759955	-0.043919728	-0.03446	0.016843	-0.04093	-0.03673
DAB2	-0.007085149	0.129745439	-0.13301	-0.01573	0.055397	-0.17976
DAB2IP	-0.023306584	-0.003415802	-0.01248	-0.00717	-0.25095	0.023843
DACH1	-0.012613402	-0.105056975	-0.14662	-0.03924	0.084308	-0.03398
DACH2	-0.073412927	-0.015289784	0.070396	-0.04041	0.093511	-0.00168
DACT1	-0.00713703	-0.106508723	-0.12097	-0.04968	0.15857	-0.30007
DACT2	-0.031305175	-0.006803007	0.008515	-0.00323	-0.05495	-0.14656
DACT3	-0.043484953	-0.054885415	0.02098	0.022742	-0.09923	0.063003
DACT3-AS	-0.033342449	-0.085546137	-0.05077	0.005392	0.001366	0.102455
DAD1	0.066546432	0.080497745	0.174491	0.113253	-0.29417	-0.05496
DAG1	0.016200309	0.110965548	-0.05914	0.022965	-0.09469	0.070965
DAGLA	-0.028180121	-0.096322382	-0.18769	-0.01023	0.016751	0.273537
DAGLB	-0.000249221	-0.078102411	-0.14879	-0.06163	0.122355	0.552029
DAK	-0.018520604	-0.063024477	-0.06578	0.019988	-0.04484	0.221367
DALRD3	0.021296839	-0.054936265	0.123949	-0.01898	0.046016	-0.14591
DANCR	0.02321171	-0.070854703	0.300479	0.01567	-0.02724	-0.11811
DAND5	-0.028708306	-0.069351707	0.10768	-0.01099	0.043128	-0.06172
DAO	-0.039062444	-0.063625475	0.113434	0.011976	-0.02212	0.072651
DAOA	-0.053210429	-0.087315091	-0.03288	0.017036	-0.026	-0.05672
DAOA-AS1	-0.048736993	-0.082906675	-0.01727	0.001975	-0.01969	-0.07495
DAP	0.038878193	-0.028875911	-0.22216	-0.04914	0.128254	0.22596
DAP3	0.021017659	-0.063885812	0.05489	0.12152	-0.03292	-0.21992
DAPK1	0.00710068	-0.034340183	-0.31348	-0.11999	0.315499	0.306614
DAPK2	-0.002372302	-0.065536651	-0.13737	0.015617	-0.07369	-0.1887
DAPK3	-0.039416589	-0.002475051	-0.01083	-0.02317	0.05413	0.096039

DAPL1	-0.031410149	-0.093152124	-0.00442	0.032498	-0.05533	-0.03334
DAPP1	0.00344361	-0.190924503	-0.43147	-0.0763	0.257574	0.166867
DARS	0.036224737	-0.005195337	0.181111	0.082229	-0.21549	-0.06824
DARS2	-0.013462277	-0.001693336	-0.03434	0.0972	-0.32012	-0.04499
DAW1	-0.038296918	-0.105027325	-0.0092	0.000309	0.028102	0.040659
DAXX	0.043809202	0.084628326	0.174088	-0.02074	-0.01509	-0.38242
DAZAP1	0.061170685	0.113728767	0.177458	0.061857	-0.1483	-0.03164
DAZAP2	0.073486226	0.04415282	-0.11543	-0.0406	0.13245	-0.0127
DAZL	-0.037739133	-0.075144809	0.027378	0.072381	-0.09273	-0.15793
DBF4	0.013680504	0.072571252	0.222818	0.07827	-0.21277	-0.27035
DBF4B	-0.01165523	-0.002977956	0.033509	0.09539	-0.30882	0.029007
DBH	-0.038090239	-0.096474594	-0.02864	-0.0028	0.022939	0.134336
DBH-AS1	-0.024875304	-0.147060203	0.089504	-0.02179	0.048425	-0.03074
DBI	0.07555324	0.204331211	-0.07798	0.113932	-0.27299	0.06333
DBIL5P	-0.029740896	-0.063037506	-0.07769	0.073418	-0.20739	-0.01663
DBIL5P2	-0.028688556	-0.076464427	0.171482	0.009387	-0.011	-0.14684
DBN1	-0.007039154	-0.101260211	0.324205	-0.03356	0.037537	-0.19788
DBNDD1	-0.029018331	-0.07680208	-0.09676	-0.0195	0.04656	-0.10493
DBP	-0.000352441	-0.021186308	0.093731	-0.06288	0.192143	0.040907
Dbpht2	-0.098452665	-0.047290461	0.067639	-0.05626	0.132015	0.030204
DBR1	0.01159421	0.0368311	0.058488	0.015247	-0.10403	-0.02152
DBT	-0.013068589	0.086063345	0.138636	-0.00082	0.020072	-0.06776
DCAF10	0.015370038	0.053417304	0.146907	-0.02072	0.063145	-0.0981
DCAF11	0.034511416	0.04121862	0.043259	0.033871	-0.1657	-0.16282
DCAF12	0.02299026	0.051580996	-0.15438	0.012957	-0.04065	0.274728
DCAF12L1	-0.03968341	-0.0173801	-0.06354	-0.0037	0.040054	0.134265
DCAF12L2	-0.055757458	0.017873815	0.086836	-0.01436	0.039769	-0.00964
DCAF13	0.002554492	0.057782453	-0.02461	0.05099	-0.12933	0.051285
DCAF15	-0.005943582	-0.052658618	0.03362	0.002269	-0.02596	0.029067
DCAF16	-0.0154	-0.000194904	0.183211	-0.02072	0.029829	-0.07501
DCAF17	-0.005683361	0.138399225	0.141355	0.047458	-0.09808	-0.08326
DCAF4	-0.006187235	-0.059386416	0.172422	0.005674	-0.02932	-0.178
DCAF4L1	-0.039453424	-0.009854157	-0.05524	-0.02082	0.064925	-0.04137
DCAF4L2	-0.049154288	-0.014609223	-0.02727	0.003302	0.034927	-0.13862
DCAF5	-0.002360864	0.138624015	0.174672	-0.00231	0.030328	-0.09497
DCAF6	0.034453634	-0.034820488	-0.1029	-0.01661	-0.06576	-0.03584
DCAF7	0.012286982	0.221832191	-0.01818	0.024689	-0.02395	-0.01973
DCAF8	-0.049607252	0.075369806	0.093763	0.141831	-0.1376	-0.13174
DCAKD	-0.026634936	0.000313703	0.041472	0.050708	-0.19099	-0.05247
DCBLD1	-0.00914793	0.072672658	0.050723	-0.03251	0.099509	0.166152
DCBLD2	-0.041150708	-0.101262147	0.035985	-0.08198	0.046271	-0.11171
DCC	-0.021045271	0.005413033	-0.01225	0.027078	-0.05702	0.061529
DCD	-0.03623783	-0.041642214	0.136472	0.009608	-0.01491	-0.03258
DCDC2	-0.050075474	-0.025117169	0.023956	0.030734	-0.0814	-0.02678
DCDC2B	-0.019264388	-0.084264919	-0.08136	0.008939	-0.06842	-0.00847
DCHS1	-0.011560274	-0.194232508	0.437653	-0.05935	0.182361	-0.10298
DCHS2	-0.059319896	-0.095450958	-0.06641	0.004224	-0.0081	-0.02233
DCK	0.021822039	0.026603387	0.081674	0.01038	-0.0221	-0.18404
DCLK1	-0.094373433	-0.04741177	0.052958	-0.00925	0.076381	-0.04921
DCLK2	-0.006856622	-0.134561602	-0.02352	0.004142	-0.01662	-0.16475
DCLK3	-0.057499756	-0.031966118	-0.07996	-0.00878	-0.01048	0.036683

DCLRE1A	-0.004532762	0.118573597	0.113266	0.027903	-0.13187	-0.22919
DCLRE1B	0.006785049	0.022276937	-0.15517	0.042268	-0.13266	0.443316
DCLRE1C	-0.015191657	0.017908259	-0.10089	0.010047	-0.01945	-0.04958
DCN	-0.005893746	-0.074979933	-0.0153	-0.01147	-0.15363	-0.05005
DCP1A	0.035897609	0.055814117	0.171901	0.03029	-0.10602	-0.28996
DCP1B	0.013675936	0.019356483	0.152473	-0.00071	0.026809	-0.00912
DCP2	0.039283251	-0.062470758	-0.27794	0.01526	-0.03322	0.315441
DCPS	0.021102616	0.03444075	-0.03999	0.052364	-0.12163	0.094725
DCST1	-0.035203311	0.026966576	-0.09788	-0.0168	0.058964	0.05958
DCST2	-0.033952095	0.01237617	-0.03898	0.025598	-0.04269	0.074962
DCSTAMP	-0.02379523	-0.025275306	0.006522	-0.00552	-0.00419	-0.0637
DCT	-0.023819336	-0.024697184	0.037372	0.150338	-0.14405	-0.04641
DCTD	0.027368799	0.040077708	0.204136	0.048178	-0.12969	0.035353
DCTN1	0.065504738	0.011405312	-0.0977	0.053016	-0.16964	-0.0283
DCTN1-AS1	-0.05183857	0.0463794	-0.03046	0.002806	-0.01733	-0.0291
DCTN2	-0.057498372	0.033926952	0.067558	-0.01914	-0.07595	-0.00596
DCTN3	0.048154934	0.077194331	-0.04567	0.037956	-0.10476	0.144516
DCTN4	0.011516296	0.043207638	-0.2528	0.016922	-0.02538	0.286117
DCTN5	0.057470187	0.117877978	-0.03697	-0.02118	-0.16397	0.060883
DCTN6	0.04623359	0.01755035	0.138218	0.037388	-0.10511	0.254902
DCTPP1	0.032875488	0.017092905	0.004272	0.088607	-0.26272	-0.15975
DCUN1D1	-0.00453182	0.039750904	-0.24977	0.109665	-0.2279	0.115136
DCUN1D2	0.012153016	0.021125288	-0.13382	0.021118	-0.08464	-0.09186
DCUN1D3	-0.007022836	0.01687609	0.019001	0.04683	-0.21545	-0.04836
DCUN1D4	-0.016396968	0.045044104	0.187543	-0.15583	0.343694	-0.04753
DCUN1D5	-0.030027322	-0.008879003	0.262351	-0.02042	0.037706	-0.25416
DCX	-0.068545413	-0.04066903	0.095963	0.035361	-0.08765	-0.04606
DCXR	0.037876704	0.088775862	0.065965	0.094642	-0.267	0.093671
DDA1	0.047594577	-0.068770465	0.127641	0.06952	-0.11789	-0.01556
DDAH1	-0.033969267	-0.059812133	-0.00588	-0.01017	0.032235	-0.01996
DDAH2	0.025515673	0.000484307	-0.31754	-0.01795	0.021256	-0.11732
DDB1	0.062384982	0.037070375	0.110631	0.090951	-0.27879	-0.07564
DDB2	0.014977	0.103828283	0.136262	0.112899	-0.32649	-0.07887
DDC	-0.033362805	-0.103700649	-0.14152	0.124341	-0.32682	0.031564
DDC-AS1	-0.038830615	-0.069817573	0.028278	0.006413	0.009318	0.07355
DDHD1	0.024801135	0.101338913	0.130521	0.091665	-0.14531	-0.12933
DDHD2	0.013808165	0.085166325	0.238515	0.025363	-0.05155	-0.19551
DDI1	-0.05133299	0.00010469	-0.04081	-0.0014	0.043364	-0.04616
DDIAS	-0.000712829	-0.041943898	-0.24043	0.091534	-0.27737	0.527993
DDIT3	0.037889152	-0.024362415	-0.08061	0.065122	-0.17471	-0.0493
DDIT4	0.035828545	0.192313941	0.073682	0.107026	-0.29466	-0.49385
DDIT4L	-0.016535625	-0.02526035	0.019198	-0.01162	-0.00745	-0.07285
DDN	-0.026085977	-0.064153592	-0.07617	-0.00283	-0.02074	0.055396
DDO	-0.034660349	-0.051404443	-0.0483	-0.0327	0.098535	0.413947
DDOST	0.06367181	0.119804326	0.038279	0.046321	-0.12279	0.138003
DDR1-AS1	-0.041446842	-0.070254665	0.113917	0.022659	-0.08221	-0.10842
DDR2	-0.025546621	0.025752315	0.004185	0.107472	-0.12397	-0.16048
DDRGK1	0.057712839	0.05830557	-0.03093	0.07725	-0.23122	0.170423
DDX1	0.053944529	0.081335225	0.143396	0.062847	-0.17826	-0.10033
DDX10	0.005593851	-0.019774451	0.113753	0.047728	-0.1489	-0.09979
DDX11	-0.016150054	-0.030230881	0.162084	0.033656	-0.16083	-0.28929

DDX11-AS1	-0.076066566	-0.027520584	-0.02936	0.010221	-0.03701	-0.04901
DDX11L2	0.003120935	-0.015117711	-0.05609	-0.23377	0.633714	0.027871
DDX17	0.043722943	0.01746383	0.120999	-0.00471	0.048856	-0.35723
DDX18	0.040790933	0.036275465	0.181115	0.031702	-0.10456	-0.16379
DDX19A	-0.001977792	0.046644288	0.082324	0.015518	-0.04498	0.091958
DDX19B	0.013683202	0.056560725	0.120324	-0.02065	0.103627	0.052828
DDX20	0.018326024	0.043591326	0.020738	0.057275	-0.13365	-0.06192
DDX21	0.062181279	-0.001972933	0.003875	0.060362	-0.14506	0.009337
DDX23	0.048605539	0.094038663	0.087575	0.043967	-0.16859	-0.08225
DDX24	0.050508028	0.043275368	0.336234	0.020004	-0.06225	-0.26382
DDX25	-0.039095144	-0.034160946	-0.08492	0.034429	-0.08705	0.163923
DDX26B	0.022502304	0.021055815	0.120548	-0.02904	0.098919	-0.20822
DDX27	0.063274273	0.011328445	0.063926	0.039063	-0.10933	-0.19332
DDX28	-0.000541702	0.033530798	0.07374	-0.03784	0.106802	0.323903
DDX31	-0.051550296	-0.015666025	0.085301	-0.04737	-0.06964	-0.16855
DDX39A	0.036157783	0.10273968	0.171169	0.061269	-0.174	0.009789
DDX3X	0.027352039	0.044287821	-0.06547	0.03077	-0.08279	-0.03651
DDX3Y	-0.03528111	-0.0121949	0.118489	-0.13715	0.171601	-0.05851
DDX4	-0.024437493	-0.044214328	-0.02599	0.20251	-0.24542	-0.04486
DDX41	0.048100682	0.017338681	-0.1056	0.036984	-0.09645	0.451541
DDX42	-0.002890575	-0.007080224	0.075195	-0.00702	-0.00787	-0.05527
DDX43	-0.008851944	0.05241618	0.032689	-0.02464	0.081591	-0.0267
DDX46	0.066828029	0.145371222	0.064272	0.060502	-0.11954	-0.04795
DDX49	-0.003864713	-0.001348683	-0.08172	0.03524	-0.11548	0.241034
DDX5	0.023459495	0.023353002	0.318654	-0.06119	0.14338	-0.46656
DDX50	-0.006862721	-0.037103641	0.279416	0.017339	-0.03683	-0.35378
DDX51	-0.065578231	-0.062856696	0.024477	-0.03369	-0.07112	-0.06169
DDX52	0.02318817	0.099437761	0.008171	0.09992	-0.20541	0.117459
DDX53	-0.073393293	-0.051598655	-0.00269	-0.00148	0.054828	-0.06771
DDX54	0.02055832	-0.025444612	0.11941	0.047967	-0.09898	0.089205
DDX55	0.011362387	0.031721336	0.104549	0.046173	-0.11955	0.055959
DDX56	0.025799792	-0.042317271	0.168732	0.037259	-0.08906	-0.02908
DDX58	0.028420709	0.044869917	-0.081	-0.02046	0.081616	0.001873
DDX59	0.012034215	0.065475623	-0.16311	0.047702	-0.14899	-0.14357
DDX6	-0.000230524	0.094753522	0.073263	-0.04954	0.045284	-0.12532
DDX60	0.01721098	0.11602662	0.114348	-0.01994	0.06153	0.000711
DDX60L	0.005469612	0.023694006	-0.07453	-0.04298	0.09962	0.143096
DEAF1	-0.011983285	-0.109867232	0.145594	0.010619	0.001478	0.12932
1-Dec	-0.04444862	-0.003410739	-0.00192	-0.01562	0.068311	-0.17356
DECR1	0.046575083	0.040505022	-0.04375	0.01335	-0.02283	0.10852
DECR2	0.009732363	-0.108047103	-0.00981	0.041823	-0.1852	-0.06943
DEDD	-0.004176585	-0.075404244	-0.21749	-0.00174	-0.10243	0.181941
DEDD2	0.051835356	-0.008603189	-0.14495	-0.02913	0.09478	0.084554
DEF6	0.026020115	0.040032026	0.092917	-0.01155	0.021068	-0.14683
DEF8	0.033199885	-0.160274761	-0.40715	-0.02502	0.061428	0.19497
DEFA4	-0.006086597	-0.109140039	0.273752	-0.05636	0.150885	-0.27813
DEFA5	-0.037666132	-0.030364623	-0.0291	0.010492	-0.01386	-0.07149
DEFA6	-0.025896765	-0.008003543	0.02936	-0.02236	-0.00526	-0.10303
DEFB1	-0.031326635	0.004049963	0.01994	0.023143	-0.05733	0.000551
DEFB108B	-0.030185482	-0.002843055	0.081325	-0.01906	0.075423	-0.16178
DEFB114	-0.110708797	-0.115568484	0.080778	-0.01342	0.087506	-0.01508

DEFB118	-0.024020308	-0.013107797	0.05501	-0.01254	-0.03057	-0.09358
DEFB119	-0.062156532	-0.076169652	0.118465	0.018245	-0.01256	-0.01677
DEFB121	-0.042366844	-0.070840527	-0.08333	0.007415	0.010093	-0.08055
DEFB122	-0.035239452	-0.033324228	-0.05165	0.020393	-0.03938	0.04807
DEFB123	-0.030385971	-0.072844479	-0.01087	0.06309	-0.16793	0.084868
DEFB124	-0.022962465	-0.068991893	0.069479	-0.00197	-0.07221	-0.14132
DEFB125	-0.043320487	-0.009738908	0.000441	0.026345	-0.0576	-0.05653
DEFB126	-0.08823158	0.004187966	0.042695	0.010328	-0.02831	0.026571
DEFB127	-0.038861187	-0.043488663	-0.05361	-0.02494	0.084001	0.100083
DEFB129	-0.037019646	-0.09253646	0.035814	0.008864	-0.01117	0.023306
DEFB132	-0.057474254	-0.032082684	0.044288	-0.00282	0.023218	-0.06197
DEGS1	0.056959877	-0.062797086	-0.1975	0.063873	-0.17703	0.064398
DEGS2	-0.02414893	0.025665444	-0.1647	0.049322	-0.14043	-0.0959
DEK	0.102758312	0.246437115	0.021968	0.13305	-0.31315	-0.01551
DENND1A	0.000982055	-0.043012386	-0.24162	0.004718	-0.00819	0.65023
DENND1B	0.01181543	0.092178054	-0.05631	0.024762	-0.06933	-0.17553
DENND1C	0.037857101	0.058577684	0.18097	-0.01386	0.015197	-0.16997
DENND2A	-0.021753843	-0.030253321	0.03071	0.020074	0.020452	-0.09792
DENND2C	-0.032145344	-0.027471241	0.123315	-0.02918	0.075732	-0.09021
DENND2D	0.017789783	0.20589879	0.447921	-0.0153	0.046672	-0.31893
DENND3	0.007966866	0.015191821	-0.33731	-0.02286	0.037219	0.10277
DENND4A	0.011793025	0.048064185	0.017061	0.036099	-0.10084	-0.038
DENND4B	0.080449444	0.066052964	-0.10083	0.025478	-0.03022	-0.08445
DENND4C	0.023054875	0.155964044	0.197991	0.006981	0.009519	-0.1
DENND5A	0.023330106	-0.128295632	-0.11112	-0.04289	0.134005	0.127772
DENND5B	0.057018899	-0.055343818	-0.26093	0.022098	0.131038	-0.31485
DENND6A	0.046979968	0.125497865	0.217688	-0.03222	0.086641	-0.12586
DENND6B	-0.003180608	-0.046011042	-0.12538	-0.02807	-0.0344	0.338813
DENR	0.017515251	0.056658105	0.14656	0.058689	-0.19306	-0.09403
DEPDC1	-0.018995515	-0.006640362	0.041457	0.064247	-0.15424	-0.06203
DEPDC1-AS	-0.049597142	-0.104954295	0.032309	0.016071	-0.04209	0.06492
DEPDC1B	-0.01664191	-0.007169374	0.04243	0.151441	-0.2695	-0.06471
DEPDC4	-0.097775822	-0.061791057	0.094671	0.026132	0.023731	0.065181
DEPDC5	0.001016295	-0.067771338	-0.18009	-0.01517	-0.02488	-0.03359
DEPDC7	-0.023195969	-0.041048323	0.136631	-0.05648	0.159209	-0.07633
DEPTOR	-0.009542283	-0.067313717	0.01497	-0.01585	0.067545	-0.10488
DERA	0.022104122	0.044259116	-0.16612	-0.0481	0.14504	0.263564
DERL1	0.079021234	0.273564964	0.059872	0.059439	-0.12454	-0.20649
DERL2	0.073443024	0.147572712	-0.03692	0.029248	-0.09792	-0.25581
DERL3	-0.00448089	-0.104327294	-0.1059	-0.01563	0.026261	-0.12873
DES	-0.049222197	-0.081480171	-0.08569	-0.00023	-0.07258	-0.10232
DESI1	0.010778188	-0.056332373	0.015562	0.044735	-0.17861	0.220639
DESI2	0.004070922	-0.002421069	-0.04615	0.051978	-0.16757	-0.01779
DET1	-0.004158113	0.001750483	0.049734	-0.02897	0.095673	0.104005
DEXI	0.017370934	0.092425856	0.353519	-0.04075	0.046996	-0.20817
DFFA	0.014333772	0.010872485	0.190492	0.090099	-0.2745	-0.09124
DFFB	0.004807537	0.065480409	0.107141	-0.01192	0.063901	0.017967
DFNA5	-0.007538174	-0.112321327	-0.13518	-0.02247	0.055826	0.051665
DFNB31	-0.025966814	-0.037140742	0.155077	-0.00619	-0.05674	-0.18956
DFNB59	-0.005404326	-0.080001137	0.121682	0.003755	0.009876	-0.18892
DGAT1	0.01399616	-0.072927009	-0.22142	-0.01236	0.04697	0.305234

DGAT2	-0.000815578	-0.0916096	-0.2909	-0.01762	0.028087	0.123418
DGCR10	-0.040648631	-0.030127999	-0.00119	0.00099	0.016571	-0.05517
DGCR11	-0.009528596	-0.052873964	-0.08528	0.012403	-0.04576	0.003844
DGCR12	-0.045164018	-0.123274099	0.027819	0.036652	-0.08735	0.004966
DGCR14	0.010832317	-0.049043063	0.009812	-0.01442	-0.06076	-0.16941
DGCR2	0.015693395	-0.077590457	-0.08958	-0.04776	0.073413	0.039573
DGCR5	-0.062081078	-0.117369473	-0.00508	0.000274	-0.01011	0.076158
DGCR6L	-0.029192546	-0.060822713	0.028613	0.022511	-0.04238	0.024304
DGCR7	-0.047677485	-0.08061011	-0.09442	-0.00479	-0.0003	-0.04205
DGCR9	-0.034733629	-0.033352242	0.005753	-0.01919	-0.00249	-0.10516
DGKA	0.010349744	-0.107262727	0.421928	-0.03884	0.102856	-0.3041
DGKB	-0.08269057	-0.072655366	0.044664	0.008581	-0.03463	-0.01754
DGKD	0.04169578	0.053400146	0.091505	-0.05672	0.146164	-0.21536
DGKE	-0.074152388	-0.004509161	0.018137	-0.04617	-0.08147	-0.12335
DGKG	-0.025280953	-0.140960343	-0.25867	-0.06502	0.197569	0.618507
DGKH	0.04766647	0.067595154	0.033856	0.015622	-0.09971	0.156144
DGKI	-0.026392352	-0.026009045	-0.0353	0.0751	-0.21185	0.028569
DGKK	-0.008061352	0.344564835	-0.07865	-0.02691	0.054748	-0.20589
DGKQ	0.01739472	0.2004617	0.213752	-0.03555	0.105182	-0.01878
DGKZ	0.016344235	0.100811705	0.151359	-0.06417	0.175964	0.046609
DGUOK	0.025524909	0.001812203	0.061917	0.057403	-0.17093	0.03721
DHCR24	-0.00609379	0.122416767	-0.05993	0.068388	-0.23292	-0.10966
DHCR7	-0.005105002	-0.005204333	0.067425	0.153494	-0.43469	-0.2213
DHDDS	0.015264191	-0.000610367	-0.21186	0.05652	-0.25632	0.125452
DHDH	-0.029816015	-0.058460563	0.158142	0.002565	0.002176	-0.10642
DHFR	0.005829652	0.035536622	0.101372	0.093532	-0.27661	-0.08475
DHFRL1	0.011093919	0.099184886	0.079452	0.001408	-0.0039	-0.12139
DHH	-0.039650698	-0.056343853	-0.08999	-0.00939	0.063976	0.137671
DHODH	-0.034236222	-0.029297063	0.184765	0.02758	-0.0624	-0.13966
DHPS	0.035331715	-0.041306457	0.115455	0.071982	-0.21657	0.131583
DHRS1	0.017727269	-0.019239711	-0.05951	-0.0245	0.073124	0.012405
DHRS11	-0.014249389	0.016518807	0.012652	0.074274	-0.22503	0.009328
DHRS12	-0.033656707	-0.06516148	0.050188	-0.00225	-0.00272	0.042526
DHRS13	0.017763555	0.044884464	0.037829	0.044062	-0.12572	-0.25657
DHRS2	-0.014306172	-0.01841198	-0.00556	0.20176	-0.40685	-0.0325
DHRS3	0.009669553	0.215528779	0.255665	-0.04152	0.103209	-0.30186
DHRS4-AS1	-0.002480009	0.03574897	0.024642	0.001366	0.005412	0.083993
DHRS7	-0.024433094	0.07439999	-0.08328	0.016727	-0.00737	-0.02992
DHRS7B	0.003912861	0.000452555	-0.33026	0.02243	-0.06959	0.635794
DHRS7C	-0.0340877	-0.037391179	-0.16645	0.032567	-0.0637	0.119669
DHRS9	-0.009479534	-0.10884541	-0.32218	-0.09526	0.212638	0.149736
DHRSX	0.012665892	-0.027502058	-0.27398	0.023985	-0.08126	0.063476
DHTKD1	0.013428502	-0.078862317	-0.31309	0.007743	-0.03421	-0.08255
DHX15	0.074589035	0.181280619	0.059542	0.095306	-0.22963	-0.17321
DHX16	0.068595067	-0.006027947	-0.04354	0.046242	-0.12542	-0.18695
DHX29	0.034617567	0.079616346	-0.07339	0.042532	-0.07963	0.218671
DHX30	0.051880186	0.104677854	0.090483	0.122807	-0.08074	0.191773
DHX32	0.043595289	0.031859203	-0.02833	-0.07504	0.154446	0.192826
DHX33	0.018923615	0.002581074	0.036577	0.067851	-0.20524	0.136388
DHX34	-0.029389396	0.005009125	-0.1151	0.017407	-0.07316	-0.06204
DHX35	-0.023543693	-0.027048442	0.089512	0.026083	-0.07773	-0.03537

DHX36	0.04383464	0.135833625	0.196393	0.053334	-0.15229	-0.11157
DHX37	0.002578432	-0.061399575	0.052386	0.041495	-0.14281	-0.03887
DHX38	0.02506564	0.01665533	-0.01354	0.030217	-0.1361	-0.18393
DHX40	0.054971659	0.0359063	-0.11646	-0.0022	0.010806	0.044117
DHX57	-0.006013378	-0.053432754	-0.06417	-0.01496	0.062135	0.522632
DHX58	0.011779675	-0.030730316	0.017058	-0.04197	0.091554	0.094864
DHX8	0.051509565	0.00871129	-0.29062	0.045706	-0.05443	0.196188
DHX9	0.029957257	0.043100954	-0.01096	0.032673	-0.08459	-0.01424
DIABLO	0.080628863	0.1584079	-0.00408	0.091655	-0.25238	0.038237
DIAPH1	0.051706331	0.141126275	0.057341	-0.01675	-0.01114	-0.01903
DIAPH2	0.025786539	-0.039026378	-0.21661	-0.03357	0.108216	0.537029
DIAPH2-AS	-0.049770173	-0.071628182	-0.04298	0.000578	0.021845	-0.01853
DIAPH3	-0.050576762	-0.013821764	0.009646	0.127783	-0.20784	-0.02758
DIAPH3-AS	-0.035521815	-0.037138374	0.122732	0.014631	-0.02721	-0.11735
DICER1	-0.015396169	0.013405303	-0.1964	0.043731	0.034551	0.147186
DICER1-AS	-0.031299387	-0.094480502	0.01377	0.020914	-0.02022	-0.16531
DIDO1	-0.035502272	0.069535197	0.155558	0.090069	-0.02932	-0.32104
DIEXF	-0.009617406	0.094568678	0.1392	0.061885	-0.1057	-0.11423
DIMT1	0.024493916	0.012808513	0.196162	0.059132	-0.13592	0.006892
DIO1	-0.058952983	-0.010316322	-0.06403	-0.03773	0.108584	0.229796
DIO2	-0.044884958	-0.055537958	-0.04004	0.086045	-0.18662	-0.05682
DIO2-AS1	-0.038644926	-0.007886332	-0.0013	0.012518	-0.01349	-0.05895
DIO3	-0.026457567	-0.029304078	-0.00815	0.025756	-0.09549	0.029754
DIO3OS	-0.050490501	-0.039587886	-0.12835	0.045835	-0.1097	-0.08168
DIP2A	0.025619586	0.210929585	0.091245	-0.00678	-0.0433	-0.1062
DIP2B	-0.011599178	-0.067273914	-0.00036	-0.01893	0.045922	-0.08451
DIP2C	0.103254847	-0.061994047	0.267644	-0.06477	0.127157	-0.234
DIRAS1	-0.044517234	-0.056127586	-0.10565	-0.00145	0.000946	0.080304
DIRAS2	-0.08881034	-0.106375637	-0.00041	-0.03955	0.110796	-0.01194
DIRAS3	-0.049015378	0.020636082	-0.03579	0.024306	-0.05672	-0.01283
DIRC1	-0.098874963	-0.095454651	0.048809	-0.02509	0.077266	-0.09048
DIRC2	0.011453441	-0.033198513	-0.29441	-0.07335	0.211731	0.462689
DIRC3	-0.020978724	-0.016048472	-0.03186	0.028232	-0.06967	-0.02841
DIS3	0.068487492	0.029814083	0.012363	0.075955	-0.01212	-0.00368
DIS3L	0.026606219	0.046167609	0.168226	0.02581	-0.05038	-0.03627
DIS3L2	0.003187824	-0.026467163	0.230455	-0.05506	0.10503	-0.04935
DISC1	-0.008327092	-0.062931659	-0.02468	-0.03473	0.036665	0.072561
DISP1	-0.01100457	0.050971714	-0.03979	-0.01146	0.05473	-0.10624
DISP2	-0.025763782	-0.036979546	-0.12126	0.01898	-0.04554	-0.04202
DIXDC1	-0.089054276	-0.038243217	0.247579	0.192704	-0.0904	-0.20005
DKFZP434A	-0.025916548	-0.099881026	-0.00275	-0.01087	-0.06663	-0.08239
DKFZp434I	-0.031009101	-0.107730648	-0.05332	0.003408	-0.01348	0.047126
DKFZP434I	-0.032222628	-0.006029368	-0.0127	0.032796	-0.07053	-0.04275
DKFZP434I	-0.031366579	-0.087614661	-0.02568	-0.01979	0.080969	0.06947
DKFZp434J	-0.04194498	0.007526773	0.016771	0.009402	0.00789	-0.00431
DKFZP434I	-0.034600852	-0.088079245	0.031965	-0.0125	-0.04737	-0.01702
DKFZP434I	-0.036280971	0.0655921	0.102295	-0.00912	0.029433	-0.12045
DKFZp434I	-0.0623147	-0.097136726	-0.04648	-0.01453	0.017642	0.032844
DKFZp451I	-0.031786719	0.001150692	-0.01108	0.034375	-0.11638	-0.08882
DKFZp547J	-0.038522192	-0.023960376	-0.01423	0.011787	-0.05427	-0.13146
DKFZp564I	-0.03132761	-0.039659146	0.092385	-0.0079	0.045999	0.052078

DKFZP586I	0.028410314	-0.002500051	-0.1335	-0.03571	0.100239	-0.18322
DKFZp667F	-0.015044481	-0.005288869	-0.08868	0.000512	0.00255	-0.09196
DKFZP761C	-0.036727293	-0.059012232	0.033965	0.129543	-0.09518	-0.02166
DKFZp779M	-0.034154469	-0.038642846	0.058821	0.030869	-0.08159	-0.00151
DKK1	-0.04279582	0.006372819	0.01978	0.036707	-0.08873	-0.04295
DKK2	-0.120094149	-0.005779779	-0.06645	0.112919	-0.11863	0.194907
DKK3	-0.091863973	-0.093456053	0.261235	-0.10645	0.107777	-0.08932
DKK4	-0.030784271	-0.04536494	-0.01936	0.011568	-0.00087	-0.0401
DKKL1	-0.024441665	-0.040699159	0.033059	0.017362	-0.06744	-0.03926
DLAT	0.000342762	0.008320471	0.059681	0.05796	-0.18338	0.025009
DLC1	-0.03824787	-0.015019889	-0.13155	0.090736	-0.17871	0.141451
DLD	0.042275538	0.080175405	-0.06098	0.081629	-0.21217	0.136518
DLEC1	0.007325329	0.037881609	0.097404	-0.01646	0.069708	-0.09138
DLEU1	0.009987506	0.06031868	0.143848	0.032839	-0.10755	-0.49965
DLEU2	-0.010910342	0.109353016	-0.33369	0.004079	-0.10374	0.06708
DLEU2L	-0.038116737	0.007420805	0.041478	0.08787	-0.24218	-0.11883
DLEU7	-0.04292076	-0.035386075	-0.04284	0.03122	-0.07192	0.034698
DLEU7-AS1	-0.029633258	-0.050199815	0.052625	-0.00574	0.017553	-0.11411
DLG1	-0.004443617	0.074050331	0.133073	-0.02246	-0.07916	0.106502
DLG1-AS1	-0.048280352	-0.076989492	0.035426	0.025178	-0.06462	0.003074
DLG2	-0.020255603	-0.010143555	-0.03449	0.072035	-0.19079	0.01428
DLG3	-0.019681712	0.096682859	0.170861	0.000144	-0.09422	-0.20283
DLG3-AS1	-0.027099827	-0.071057373	0.132402	0.032426	-0.09103	0.042443
DLG4	-0.022567756	-0.118142516	0.013312	-0.00532	0.035756	0.319984
DLG5	-0.00304009	0.302857679	0.095909	-0.02139	0.062777	-0.12203
DLG5-AS1	-0.041052385	-0.082372562	-0.02253	-0.00159	-0.02006	-0.10036
DLGAP1	-0.068761654	-0.082001853	0.022444	-0.00651	0.013993	-0.08424
DLGAP1-AS	-0.001396884	0.134484943	0.145428	-0.01516	0.014015	-0.3743
DLGAP1-AS	-0.020660403	0.074952399	0.100957	0.020708	-0.05091	-0.07513
DLGAP1-AS	-0.04226122	-0.026975669	0.026414	-0.02011	0.083927	0.057163
DLGAP2	-0.041050356	-0.055240584	0.080147	0.001317	-0.01048	-0.08747
DLGAP2-AS	-0.04551965	-0.032718677	0.000726	-0.01273	0.052654	-0.16743
DLGAP3	-0.028066498	-0.081967971	-0.06826	0.023751	-0.07806	0.067026
DLGAP4	-0.011940813	-0.073861303	-0.18278	-0.04196	-0.01094	0.002494
DLGAP5	-0.01142559	0.009779401	0.06748	0.14182	-0.27602	-0.04279
DLK1	-0.043861857	-0.054071029	0.044507	0.011605	-0.00861	0.036091
DLK2	-0.025785994	-0.054451542	0.032778	0.010697	-0.11755	-0.10983
DLL1	-0.012119265	0.227447432	0.222112	-0.00705	0.062531	-0.1751
DLL3	-0.02730849	-0.082126001	-0.04853	0.018646	-0.02878	0.093398
DLL4	-0.012220531	-0.040036221	-0.03015	0.018084	-0.13278	-0.01137
DLST	0.041180702	0.009659429	0.117642	0.03816	-0.1437	-0.30054
DLX1	-0.057114549	-0.098707774	0.087023	0.016921	-0.0503	0.021007
DLX2	-0.027875958	-0.005745652	-0.11496	0.015024	-0.04743	-0.02682
DLX3	-0.036343623	-0.086155539	0.08622	-0.00556	0.030991	0.084478
DLX4	-0.042434167	-0.11267519	-0.03259	0.001608	0.032356	0.174302
DLX5	-0.042174305	-0.014864076	-0.05692	0.011524	0.000964	-0.01909
DLX6	-0.046950035	0.029184741	0.003232	-0.0071	0.019563	-0.01778
DLX6-AS1	-0.037847486	0.019421032	-0.08965	0.004219	0.008699	-0.03825
DMAP1	0.013668918	0.039193138	0.104929	0.020939	-0.0283	0.011118
DMBT1	-0.021434796	-0.038418939	-0.02137	0.05285	-0.2035	0.017436
DMBX1	-0.054264125	-0.089713203	-0.17415	0.012401	-0.04386	0.218749

DMC1	-0.02436784	-0.030983438	0.029964	0.115685	-0.26502	-0.05193
DMD	0.034033521	-0.01324123	-0.06246	0.120798	-0.26727	-0.12002
DMGDH	-0.078135679	-0.06057944	-0.09368	-0.00369	-0.00388	0.02829
DMKN	-0.033030147	0.087800667	0.044632	0.010143	-0.00791	-0.12534
DMP1	-0.065051351	-0.061820555	-0.03493	-0.02039	0.033163	0.016845
DMPK	-0.031428902	-0.053639578	0.304497	0.068076	-0.18561	-0.11133
DMRT1	-0.047984163	-0.025058266	0.015967	0.056475	-0.15853	-0.08913
DMRT2	-0.054244577	-0.089752899	0.032448	0.012629	0.000554	-0.01174
DMRT3	-0.061968941	-0.03778414	-0.11456	-0.02514	0.083229	-0.0357
DMRTA1	-0.027336611	-0.015524245	0.006855	-0.01797	0.070949	0.125795
DMRTA2	-0.037468117	-0.043858242	-0.11143	0.011202	-0.02859	-0.00888
DMRTB1	-0.033436576	-0.069882732	0.046153	-0.01597	0.062061	0.110195
DMRTC2	-0.0311883	-0.061567212	0.003505	0.022524	-0.04213	-0.13601
DMTF1	0.048203886	0.095775174	0.100331	0.001702	0.005422	-0.26502
DMTN	-0.015814554	-0.071237755	0.127848	-0.11631	0.238802	-0.18508
DMWD	-0.018052542	-0.066319223	0.104793	0.004155	-0.12685	-0.10962
DMXL1	0.013372863	0.012692767	-0.16458	0.004007	-0.00903	0.057012
DMXL2	0.006763574	-0.135101447	-0.30657	-0.05795	0.171714	0.546105
DNA2	-0.014536261	0.003673197	0.034423	0.135102	-0.41309	-0.08426
DNAAF1	-0.023280239	-0.040933368	-0.05167	-0.00361	-0.04135	-0.0575
DNAAF2	0.001304256	0.07564934	0.047318	0.089538	-0.22298	-0.0657
DNAAF3	-0.020735885	-0.0650171	0.039535	0.076369	-0.19689	-0.04915
DNAH1	-0.040786001	0.028077934	0.006815	-0.03218	0.010818	-0.05586
DNAH10	0.001659301	-0.037607716	0.110855	3.69E-05	-0.01679	0.022496
DNAH11	-0.078975012	-0.093886914	-0.04295	-0.05328	0.044666	-0.09066
DNAH12	-0.058277706	-0.028043022	-0.03046	-0.13428	0.387511	0.092798
DNAH14	-0.105960206	-0.147824096	0.019267	-0.06359	0.073319	-0.03542
DNAH17	-0.048995458	-0.010473927	-0.05044	-0.0022	0.038168	-0.0121
DNAH17-AS	-0.032333672	-0.061885429	-0.04381	0.014089	-0.02098	0.010445
DNAH2	-0.046687162	-0.124815215	-0.05832	0.013953	-0.02223	0.196089
DNAH3	-0.028934979	-0.014306496	0.007619	0.006996	-0.00466	0.146167
DNAH5	-0.039557657	-0.079951356	-0.0762	0.097095	-0.17811	-0.05258
DNAH6	-0.131454248	-0.040484761	0.024095	-0.06159	0.129359	-0.13819
DNAH7	-0.015824542	-0.08238994	-0.09808	0.026748	-0.04762	-0.08191
DNAH8	-0.034200324	-0.020310629	0.104487	0.043761	-0.10034	-0.21642
DNAH9	-0.011093583	-0.061939067	-0.07935	0.020534	-0.00949	-0.01611
DNAI1	-0.042577489	-0.06477557	-0.00363	0.009904	-0.03654	0.075719
DNAI2	-0.03604809	0.002602636	0.123685	-0.00438	0.036476	-0.0422
DNAJA1	0.062030624	0.044455318	-0.14831	0.08791	-0.20603	-0.02879
DNAJA2	0.068449519	0.050338644	0.043539	0.077195	-0.23015	0.117271
DNAJA3	0.033026014	0.004793588	0.284487	0.068019	-0.21083	-0.17246
DNAJA4	0.028119311	-0.021279599	0.117987	-0.12404	0.113411	-0.05315
DNAJB1	0.032270085	-0.007697219	0.35238	0.007984	-0.0182	-0.19565
DNAJB11	0.066911966	0.204629182	-0.16349	0.103244	-0.26586	0.051361
DNAJB12	0.009362507	-0.097488428	-0.29615	0.007699	-0.05712	0.426466
DNAJB13	-0.02622267	-0.030180286	0.007581	-0.00168	-0.001	0.065105
DNAJB14	0.060424434	0.129113409	0.125489	-0.0207	-0.01775	-0.2126
DNAJB2	0.020721343	0.0073324	0.15585	-0.01158	-0.03858	-0.19394
DNAJB4	-0.011078358	0.054834038	-0.00175	0.021399	-0.07893	-0.06867
DNAJB5	-0.019919789	-0.045337618	-0.07041	0.04291	-0.14362	0.075925
DNAJB6	0.026394443	0.116405743	-0.16835	-0.00883	0.01696	0.067529

DNAJB7	-0.060710289	-0.031619078	0.048125	-0.02798	0.068622	0.04974
DNAJB8	-0.032531036	-0.01294955	-0.01726	-0.02635	0.109135	0.042197
DNAJB8-AS	-0.047525162	0.003769102	-0.08372	-0.0103	0.023283	0.022449
DNAJB9	0.021470238	0.025388103	0.013896	-0.00022	-0.06743	-0.20261
DNAJC1	0.087369276	0.285434769	0.092207	-0.02633	0.026819	0.018215
DNAJC10	-0.009207388	-0.005756101	-0.19817	0.022154	-0.02066	0.200299
DNAJC11	0.00841178	-0.050914037	0.119904	0.023236	-0.15389	-0.06737
DNAJC12	-0.012901261	-0.034175936	-0.01106	0.004528	-0.1856	-0.04545
DNAJC13	-0.018245286	-0.012155063	-0.2047	-0.03269	0.11038	0.442486
DNAJC14	0.017869769	0.118491663	-0.08078	0.041354	-0.14268	-0.16028
DNAJC15	0.005895773	0.023486135	-0.0754	-0.03349	0.165616	0.075376
DNAJC16	-0.088627526	0.128820351	0.06959	-0.0354	0.008604	-0.11535
DNAJC17	0.010396105	0.140619389	-0.01133	0.026356	-0.048	0.195304
DNAJC18	-0.019216561	-0.023840031	0.243088	0.108542	-0.30188	-0.11696
DNAJC19	0.033486884	0.032443013	0.300844	0.029522	-0.09153	-0.12356
DNAJC2	0.021155499	0.045226369	0.131231	0.045798	-0.11429	0.070474
DNAJC21	0.071694397	0.011087512	-0.03311	0.109822	-0.05015	0.109546
DNAJC22	-0.028156222	0.058778479	-0.09073	0.02474	-0.06153	-0.05736
DNAJC24	0.008384831	0.06912107	0.224098	0.013534	-0.0312	-0.10611
DNAJC27	-0.026406209	0.11114543	0.202293	-0.00785	0.084019	-0.07506
DNAJC27-/	-0.033028705	-0.035687076	-0.08993	0.02272	-0.04806	0.023213
DNAJC28	-0.037194265	0.026526226	0.076228	-0.03173	0.103345	0.032654
DNAJC3	0.02652389	0.079953204	-0.25239	-0.00559	-0.00938	0.254627
DNAJC3-AS	-0.037413218	-0.02712785	-0.09051	0.036695	-0.08474	-0.01739
DNAJC30	-0.006489755	0.041739355	0.282438	-0.03192	0.101675	-0.14164
DNAJC4	0.071898478	-0.019027583	0.035927	0.085542	0.000961	0.076889
DNAJC5	0.05806362	0.010277534	-0.19764	0.024327	-0.01636	0.384631
DNAJC5B	-0.017369521	-0.03200665	-0.06077	0.103907	-0.27712	-0.0653
DNAJC5G	-0.031377763	-0.077735829	-0.04004	-0.00549	-0.02347	-0.011
DNAJC6	-0.020467006	-0.045240897	0.066074	0.166997	-0.2958	-0.08346
DNAJC7	0.044427668	-0.017511516	-0.03527	0.001477	0.007612	0.083151
DNAJC8	0.061182151	0.095936629	0.017648	0.055773	-0.17889	-0.00362
DNAJC9	0.016630687	0.066967074	0.086629	0.07023	-0.18906	-0.2391
DNAJC9-AS	-0.027020075	-0.047061905	0.01525	0.003659	-0.05455	-0.19061
DNAL1	-0.066414258	0.060392565	0.029262	0.013682	-0.08052	-0.01986
DNAL4	0.006908905	0.067022783	-0.07107	0.011649	-0.04362	0.105304
DNALI1	-0.056997508	-0.119880005	-0.07534	0.012515	0.001275	0.074865
DNASE1	-0.019559998	0.086206483	0.063892	-0.05119	0.045332	-0.13497
DNASE1L1	0.028079935	-0.099355787	-0.18995	-0.04732	0.135879	0.344913
DNASE1L2	-0.02654513	-0.08309536	0.052021	0.021511	-0.05802	-0.04076
DNASE1L3	0.004741971	-0.109156567	-0.18643	-0.02896	0.078484	-0.21505
DNASE2	0.033626606	0.078215552	-0.04943	-0.03678	0.099196	0.294025
DNASE2B	-0.044166303	0.018206605	-0.02698	0.051365	-0.12396	-0.06981
DND1	0.037306877	-0.041799323	0.128928	0.00861	-0.09408	-0.25235
DNER	-0.021862596	-0.063728303	-0.06942	0.035218	-0.10678	-0.02295
DNHD1	-0.018276085	-0.061210738	0.188407	0.010302	-0.01042	-0.0882
DNLZ	0.009131312	0.028637115	0.086919	0.023669	-0.06884	0.024692
DNM1	-0.021341293	-0.119807865	-0.00054	-0.10035	0.285112	0.083108
DNM1L	0.025748038	0.036926479	-0.07145	0.047245	-0.09251	0.204594
DNM1P35	-0.037954498	-0.014642824	-0.01339	0.024804	-0.04175	0.056309
DNM1P41	-0.031836393	-0.057796367	0.037699	-0.00735	-0.00731	-0.04153

DNM1P46	-0.040765556	-0.010774342	0.022922	-0.01181	0.062825	-0.05297
DNM2	0.006311065	0.034869236	-0.05372	0.034906	-0.05864	0.032867
DNM3	0.06138338	0.01907476	-0.00614	-0.30324	0.286875	0.058077
DNM3OS	-0.04562939	-0.058173595	0.021604	-0.01626	0.025283	-0.10565
DNMBP	0.015516005	0.035460892	-0.23263	-0.04196	0.143972	0.315471
DNMT1	0.028703446	0.130644317	0.272811	0.037237	-0.08539	0.001726
DNMT3A	-0.016681095	-0.018172468	0.242407	0.012267	0.190108	-0.10285
DNMT3B	-0.021734316	0.007219854	0.07855	0.02855	-0.09195	-0.0231
DNMT3L	-0.041543225	-0.061462081	-0.02415	0.020939	-0.02922	0.110722
DNPEP	0.020069589	0.022473053	0.048087	0.052678	-0.1368	0.250213
DNPH1	-0.038607375	-0.175119163	0.065278	0.118445	-0.19765	0.195707
DNTT	0.015519978	0.001224777	-0.11483	0.013787	-0.10208	-0.00699
DNTTIP1	0.025995354	-0.084482032	-0.38265	-0.00016	0.011255	0.403583
DNTTIP2	0.066483919	0.116975022	0.062992	0.056764	-0.12655	-0.17346
DOC2A	-0.029325687	-0.035879632	0.032299	0.019758	-0.04491	0.160738
DOC2B	-0.035511663	-0.07110277	-0.09974	0.005445	-0.03672	0.006977
DOCK1	-0.043839083	-0.108392326	-0.07666	-0.06398	0.024069	0.085491
DOCK10	0.014833453	0.058851687	0.243882	0.009078	-0.01795	-0.14367
DOCK11	0.040596009	0.109680262	-0.0312	-0.07656	0.218534	-0.05557
DOCK2	0.031505063	-0.025803071	-0.20393	0.011354	-0.01592	0.145926
DOCK3	-0.018328197	0.000383391	0.246071	0.009094	-0.00642	-0.13933
DOCK4	0.02619834	0.017071544	-0.17651	-0.06244	0.041069	0.087594
DOCK4-AS1	-0.030230222	-0.030472699	-0.03746	0.023859	-0.05854	-0.03601
DOCK5	-0.031657223	0.03560971	-0.34151	-0.03231	0.034905	0.160977
DOCK6	-0.049522551	0.004382681	0.007915	0.05689	-0.07426	-0.0137
DOCK7	2.14E-05	-0.035871716	-0.02563	0.027758	-0.05763	0.112542
DOCK8	0.038036224	-0.001902694	-0.16525	0.016665	-0.0357	0.067732
DOCK9	-0.050969327	0.016418342	0.340202	0.140716	-0.01341	-0.31577
DOCK9-AS2	-0.0398089	-0.007622597	0.203749	0.012857	-0.06669	-0.20242
DOHH	-0.010254215	-0.079332687	-0.00201	0.021965	-0.12248	-0.0146
DOK1	0.023386477	-0.00959889	-0.26829	-0.04263	0.068732	0.295505
DOK2	0.021041845	0.113068544	-0.05507	-0.05661	0.1258	0.330639
DOK3	0.004842149	-0.16432957	-0.38076	-0.0948	0.250087	0.467541
DOK4	-0.016986969	-0.049305417	-0.0119	0.11412	-0.27794	0.051486
DOK5	-0.027020233	-0.04316701	0.094292	0.029691	-0.11701	-0.07495
DOK6	-0.041361398	-0.009476261	0.093908	-0.06879	0.224081	-0.0086
DOLK	0.005858393	0.054461536	0.015457	0.027801	-0.1255	0.139854
DOLPP1	0.023657425	-0.001814935	0.009131	0.019047	-0.15989	-0.11548
DONSON	0.009447781	0.053851423	0.031884	0.14343	-0.3909	-0.06668
DOPEY1	-0.028057603	0.010202223	0.017061	-0.05448	0.125445	-0.0729
DOPEY2	0.001342802	-0.028095568	-0.17188	-0.03944	0.130696	0.040515
DOT1L	-0.004303714	-0.006179873	-0.057	-0.013	-0.11996	-0.00869
DPAGT1	0.0241533	0.008123123	0.011924	0.052818	-0.1529	0.177538
DPCD	-0.000333869	-0.071293966	-0.13898	0.018175	-0.07117	0.489462
DPCR1	-0.046706568	-0.06874513	0.002637	-0.01324	0.069054	0.033423
DPEP1	-0.030522633	-0.064027952	0.012332	-0.01605	-0.01988	0.045087
DPEP2	0.017706744	-0.056190258	-0.18128	-0.0867	0.241399	0.133773
DPEP3	-0.01487353	-0.083953507	-0.17744	-0.01547	0.01373	-0.04201
DPF1	-0.02928744	-0.104581818	0.02939	0.007024	-0.08683	-0.00729
DPF2	0.05561853	0.032677697	-0.16192	0.043822	-0.1286	-0.27518
DPF3	-0.013390727	0.020441955	-0.02949	0.044689	-0.22419	-0.17164

DPH2	0.017406483	-0.022111573	0.101537	0.025777	-0.12129	-0.15636
DPH3	0.028987021	0.076015965	-0.1877	0.03637	-0.10186	0.311199
DPH3P1	-0.032650551	-0.000425729	0.012271	0.066946	-0.164	0.081242
DPH5	0.041116648	-0.017525504	0.277634	0.058202	-0.04291	-0.29633
DPH6	-0.026502591	0.007530112	0.182436	-0.0531	0.113001	-0.08398
DPH6-AS1	-0.034767195	-0.09767689	0.038605	0.005484	-0.02483	-0.09035
DPH7	0.030958176	-0.008730951	0.243928	-0.00392	0.011034	-0.22023
DPM1	0.084689323	0.116821534	0.099932	0.089224	-0.20694	-0.09804
DPM2	0.019732728	0.053600771	0.0174	0.060762	-0.21689	0.186986
DPM3	0.027021736	-0.008319138	0.088906	0.04824	-0.1464	0.252876
DPP10	-0.067767734	-0.082338518	-0.06017	-0.00853	0.008306	-0.044
DPP10-AS	-0.037581097	-0.12423691	-0.03677	0.011762	-0.00092	-0.05872
DPP10-AS	-0.033017525	-0.015676118	-0.0592	0.010095	-0.01179	-0.05781
DPP3	0.035054672	0.0849459	-0.03888	0.083091	-0.23099	0.216395
DPP4	0.025240148	-0.087677547	0.237116	0.126638	-0.26395	-0.33709
DPP6	-0.007445056	-0.043296763	0.041737	0.044576	0.0162	-0.02721
DPP7	-0.065524548	-0.083207986	0.280808	-0.07938	-0.06549	-0.16312
DPP8	0.04109175	0.091981761	0.044853	-0.03179	0.045125	-0.07035
DPP9	0.020823697	-0.066163471	-0.07301	0.04142	-0.14393	-0.04797
DPPA2	-0.039812338	-0.102588848	0.069498	0.009369	-0.00261	0.108431
DPPA4	0.027094443	-0.075697097	-0.05874	0.012904	0.073018	-0.30887
DPPA5	-0.023546029	-0.087179379	0.04202	0.002388	0.006565	-0.16159
DPT	-0.079344915	-0.045772581	0.068124	-0.00496	0.03976	0.081584
DPY19L1	-0.051847282	-0.009093607	-0.00491	-0.00277	-0.1583	-0.02528
DPY19L1P	-0.022248824	-0.043194144	-0.05701	0.018807	-0.04436	-0.11169
DPY19L2	-0.029881648	-0.102388796	0.372068	-0.02452	0.038834	-0.08129
DPY19L2P	-0.014295517	0.057506626	0.249284	-0.01659	0.036005	-0.23155
DPY19L2P	-0.040603113	-0.035593893	-0.06562	-0.02751	0.08768	0.01838
DPY19L2P	-0.021186598	0.009963366	0.00251	-0.00054	0.001766	-0.1203
DPY19L3	0.011206512	0.057992417	-0.17671	0.008751	0.00115	-0.0195
DPY19L4	-0.00824264	0.004250941	0.234654	0.042455	-0.11423	-0.0119
DPY30	0.053512678	0.088262478	0.115901	0.06584	-0.18183	0.004202
DPYD	-0.017437447	0.005606442	-0.17075	-0.00296	-0.05079	0.360648
DPYS	-0.028566827	-0.004473214	0.015829	0.010281	-0.01624	-0.06677
DPYSL2	0.019872451	-0.008380084	-0.24587	0.012035	-0.03172	0.414476
DPYSL3	-0.045349929	-0.027731367	0.07907	-0.02643	0.045292	-0.07978
DPYSL4	-0.041831936	-0.003563302	-0.07208	-0.00535	0.007847	0.043088
DPYSL5	-0.046840331	-0.071099694	-0.06438	0.007344	-0.02827	-0.02057
DQ570096	0.007910793	0.000714029	0.048622	-0.04533	0.096154	0.06268
DQ570835	-0.001146002	-0.011676256	0.181794	-0.02032	0.05139	-0.11857
DQ576800	-0.036669705	-0.005049856	-0.08166	0.035099	-0.08255	-0.03024
DQ576994	-0.039145638	-0.056304689	0.011109	0.017416	-0.03399	0.067576
DQ580846	-0.04216635	-0.044794771	-0.16353	0.000737	0.011145	0.091548
DQ581328	-0.038146304	-0.051662023	0.141766	-0.01616	0.064132	-0.00482
DQ582785	-0.049743504	-0.042688534	0.039111	-0.01313	0.060175	-0.03553
DQ583756	-0.031640362	-0.053559331	0.0083	0.017885	-0.03028	0.110446
DQ586822	-0.019790235	-0.041808668	0.108429	-0.01597	-0.06243	-0.12323
DQ588163	-0.040532916	0.021540358	-0.0898	-0.01828	0.074008	-0.06564
DQ592442	-0.012238714	-0.01516189	0.103871	0.051293	-0.17561	-0.14097
DQ594366	-0.032777768	-0.02083956	-0.00873	0.004918	0.006205	0.065547
DQ599616	-0.022834255	0.038028608	0.025696	-0.0044	0.002074	0.034176

DQX1	-0.037620862	-0.049082866	-0.02774	0.042007	-0.08775	-0.08679
DR1	0.034230997	0.055628303	-0.19175	0.006844	0.034052	0.090334
DRAM1	0.009551506	-0.041193271	-0.2642	0.003937	-0.0151	0.477276
DRAM2	0.024623299	-0.056096641	-0.09779	-0.01062	0.045846	0.20069
DRAP1	0.021992983	0.07458563	0.045302	0.016844	-0.05276	0.083894
DRAXIN	-0.021207001	0.248565906	-0.09934	0.00638	########	-0.10386
DRC1	-0.029763445	-0.038639204	-0.1205	0.005018	-0.06328	0.021463
DRD1	-0.047437526	-0.028577237	-0.02154	-0.00323	0.036417	-0.0512
DRD2	-0.022999173	-0.045489323	-0.01471	0.057265	-0.17779	-0.05481
DRD3	-0.05453784	-0.043168316	-0.07297	0.000398	0.021464	0.02794
DRD4	-0.01234669	-0.065280161	-0.13926	0.023006	-0.03564	-0.04194
DRD5	-0.029372773	0.021766158	0.149901	0.027299	-0.0746	-0.04775
DRG1	0.059068044	0.042358198	0.142053	0.081895	-0.24807	0.044696
DRG2	0.033815683	0.061796037	0.177363	0.049902	-0.21129	0.04426
DRICH1	-0.038721777	-0.13966985	0.024211	0.011833	-0.06081	-0.01022
DROSHA	0.026132694	0.057711203	0.142301	0.03842	-0.09539	-0.13331
DRP2	-0.063030472	0.003835902	-0.03284	0.020659	-0.06386	-0.01285
DSC1	-0.012781636	-0.129209896	0.502563	-0.04176	0.270928	-0.18082
DSC2	-0.022671988	-0.104272866	-0.19489	-0.06132	0.072206	0.198917
DSC3	-0.075114006	-0.013376161	-0.07634	-0.06626	0.008448	-0.04055
DSCAM	-0.052295114	-0.046958149	-0.0301	-0.01369	0.062775	0.046395
DSCAM-AS	-0.032803493	-0.081788634	0.065893	0.013608	-0.02473	-0.01602
DSCAML1	-0.051274863	-0.087432225	0.051208	0.002817	0.027633	-0.03286
DSCC1	-0.022000091	-0.005375713	-0.03975	0.175087	-0.3803	-0.00301
DSCR10	-0.031378398	-0.027640486	-0.0254	-0.02409	-0.01326	-0.13503
DSCR3	0.029295506	0.092689767	-0.06831	-0.0148	0.013618	0.159245
DSCR4	-0.030325563	-0.049335445	0.03668	0.004019	-0.06173	-0.07458
DSCR8	-0.021588682	-0.042007695	-0.00293	0.024895	-0.0929	-0.06835
DSCR9	-0.028298263	0.012216706	-0.01481	0.01522	-0.08965	-0.11059
DSE	-0.026990685	-0.048145692	-0.00386	-0.00503	0.073918	-0.0056
DSEL	-0.066672899	-0.045763936	0.404892	-0.06756	0.033733	-0.11888
DSERG1	-0.002413058	-0.079710465	0.006755	-0.02667	0.066909	-0.10989
DSG1	-0.051707198	-0.046154315	-0.09178	0.004132	0.000634	0.092728
DSG2	-0.019186858	-0.054207623	-0.03853	-0.01342	0.036615	-0.13994
DSG3	-0.07147973	-0.106796111	0.033128	-0.01761	0.031914	0.035847
DSG4	-0.061423611	-0.080342067	0.031025	0.015098	-0.04374	0.115238
DSN1	0.006437485	-0.00055538	-0.09964	0.081163	-0.22099	-0.17037
DSP	-0.01048188	-0.079491541	-0.08088	-0.01669	0.048028	-0.17532
DSPP	-0.016497813	-0.007271214	0.080751	0.021632	-0.04051	0.013346
DST	-0.066450703	-0.08480991	-0.17909	-0.04981	-0.01238	0.163192
DSTN	0.038045881	0.197770213	0.278987	0.021452	-0.07123	-0.13828
DSTNP2	0.009553701	0.002211646	0.082876	0.009684	-0.12292	-0.23665
DSTYK	0.004952867	0.082190124	0.032733	0.013138	0.010814	0.164684
DTD1	0.023107583	0.012631086	0.135889	0.035446	-0.12382	-0.10982
DTD2	0.004070225	0.027309214	0.142567	0.049103	-0.1251	0.037149
DTHD1	-0.003730185	0.275639843	0.127094	0.001056	0.036363	-0.17279
DTL	-0.008994275	0.091360993	0.033446	0.139681	-0.27831	-0.11739
DTNA	-0.05571336	0.013892394	-0.05645	0.048888	0.041508	0.072258
DTNB	-0.040902778	-0.02034938	0.062395	-0.00539	-0.07308	-0.04636
DTNBP1	0.00508574	0.084558118	-0.04333	0.005531	0.156989	-0.04743
DTWD1	0.000607218	-0.006148317	-0.01032	-0.04659	0.037471	0.089068

DTWD2	-0.019007076	-0.066233756	-0.03522	-0.00233	-0.09914	-0.09384
DTX1	-0.013030785	-0.013002923	-0.07635	-0.01365	0.059489	-0.16007
DTX2P1-UI	0.033922415	-0.062638583	0.161304	0.0226	-0.13787	-0.33967
DTX3	-0.053103228	-0.043317938	-0.01471	-0.015	0.048971	-0.04483
DTX3L	0.036822365	0.150878916	-0.0236	0.012025	-0.01448	0.065236
DTX4	-0.001315936	-0.158224304	-0.26729	-0.03704	0.06925	-0.17654
DTYMK	-0.005451767	-0.001256869	-0.0626	0.133649	-0.30126	0.075607
DUOX1	-0.03516655	-0.00848965	-0.04194	-0.03716	0.020785	-0.07293
DUOX2	-0.039558435	0.012491159	-0.08807	-0.00584	0.04952	0.002821
DUOXA1	-0.039484155	-0.08287164	-0.06203	-0.00816	-0.01005	-0.0294
DUOXA2	-0.011062446	-0.05089687	0.01299	-0.00406	-0.07977	-0.03127
DUS1L	0.046349106	0.039185987	0.261746	0.01399	-0.04017	-0.0193
DUS2	0.013942426	-0.029609361	-0.27532	-0.02425	-0.0125	0.093457
DUS3L	-0.011285687	-0.02726389	0.038313	0.037098	-0.08393	0.085697
DUS4L	-0.00379792	-0.006516703	0.109616	0.05633	-0.14003	-0.00193
DUSP1	-0.038465974	-0.059433073	-0.03163	-0.11058	0.180298	0.059226
DUSP10	0.009212664	0.133452074	-0.14619	0.090529	-0.25677	0.281404
DUSP11	0.058701981	0.142851447	0.117551	0.029102	-0.09929	-0.32166
DUSP12	0.046446275	0.121029968	0.230535	0.056441	-0.17078	0.018135
DUSP13	-0.028151848	-0.09415079	-0.11462	0.044614	-0.15521	0.06773
DUSP14	-0.002756049	0.063787174	0.171833	0.082687	-0.26139	-0.27127
DUSP15	-0.024443902	-0.030883373	0.012272	0.012407	-0.02702	-0.03868
DUSP16	-0.015473303	-0.097830627	0.208762	0.032214	-0.08719	-0.30062
DUSP18	0.010167229	-0.08284971	-0.09143	-0.0521	0.0723	0.043618
DUSP19	-0.079783334	-0.016047917	-0.03078	-0.05524	0.050057	-0.02912
DUSP2	0.013649896	0.13194946	0.329562	-0.00512	0.013094	-0.15609
DUSP21	-0.038487259	-0.035723188	-0.04457	0.012597	-0.0061	-0.06058
DUSP22	0.059882292	-0.017749722	-0.37496	-0.03733	0.114764	0.236719
DUSP23	0.016903722	0.010900541	-0.00329	-0.07219	0.170914	0.204372
DUSP26	-0.034457363	-0.105695244	0.107948	-0.01302	-0.00335	-0.10875
DUSP27	-0.037567713	0.00113308	-0.08243	0.001879	0.021847	-0.03881
DUSP28	0.03684854	0.088569911	0.139318	-0.04393	0.107078	-0.0138
DUSP3	0.006195359	-0.070538229	-0.29958	-0.11089	0.28834	0.444746
DUSP4	0.006369534	-0.015061517	-0.00522	0.071705	-0.22168	-0.07772
DUSP5	0.012731799	-0.033490099	-0.10284	0.032839	-0.11208	-0.06556
DUSP5P1	-0.036793342	-0.059193079	-0.07012	0.150157	-0.20734	0.024684
DUSP6	0.010346573	-0.034693081	-0.34891	-0.04798	0.138845	0.41168
DUSP7	0.010348009	0.021996439	-0.01412	-0.02268	0.097325	0.332886
DUSP9	-0.030431805	-0.081564437	-0.09478	0.012968	-0.02876	0.045617
DUT	0.045281355	0.031858582	0.173366	0.078218	-0.21269	-0.13171
DUX1	-0.015457305	-0.085555493	0.064831	-0.00975	-0.08911	-0.1509
DUXAP10	-0.046318762	-0.006852959	0.014042	-0.00131	0.026189	-0.16183
DVL1	0.027783217	-0.035274755	0.212462	0.041978	-0.1107	0.003755
DVL2	0.019521057	-0.011579824	0.28304	0.064971	-0.21519	-0.25944
DVL3	0.024857114	0.054180328	-0.28312	0.088886	-0.2696	0.067289
DXO	0.028373062	-0.07801801	0.030915	0.004841	-0.0117	-0.07452
DYDC1	-0.061102551	-0.049857805	-0.21092	-0.0062	0.008035	0.030506
DYDC2	-0.054635081	0.010587907	0.063387	0.018869	-0.02584	0.054187
DYM	0.006977733	-0.069448882	0.026975	-0.0683	0.194844	0.092902
DYNC1H1	-0.055988805	-0.083354415	0.118981	0.038852	0.003481	0.099283
DYNC1I1	-0.030706507	-0.048748748	0.01427	0.021124	-0.06487	-0.07256

DYNC1I2	0.012674544	0.028283601	0.030746	-0.05953	0.179023	0.142463
DYNC1LI1	0.048591762	-0.007013363	-0.4466	0.025743	-0.04947	0.150817
DYNC1LI2	0.037446115	0.024231666	-0.08647	0.011841	-0.02511	0.262273
DYNC2H1	-0.024799226	0.034984507	0.162226	-0.02436	0.060842	-0.13825
DYNC2LI1	0.010096488	0.029679308	-0.03412	0.025783	0.006957	0.041146
DYNLL1	0.042696242	0.102710235	-0.09364	0.021901	-0.04983	0.202133
DYNLL1-AS	-0.024043223	0.132488296	0.050519	0.078191	-0.2238	-0.09704
DYNLL2	0.008808797	-0.061414636	0.209052	0.019805	-0.08861	0.026341
DYNLRB1	-0.006977751	-0.055336892	0.019652	-0.02128	-0.04961	-0.20609
DYNLRB2	-0.034976681	-0.050620894	0.112226	0.129277	-0.27427	-0.03985
DYNLT1	0.050869908	0.138251692	0.018689	0.030816	-0.04337	0.37838
DYNLT3	0.026973773	0.036807156	0.198409	-0.04215	0.10605	-0.09457
DYRK1A	-0.131831806	0.000979998	0.015966	-0.03334	-0.04609	-0.05883
DYRK1B	-0.022201186	-0.041078824	-0.05582	0.003587	0.008619	0.069878
DYRK2	0.020069158	0.12741407	0.395292	-0.044	0.093542	-0.38033
DYRK3	-0.019422497	-0.057293356	-0.05627	0.004896	-0.05865	-0.08943
DYRK4	0.021351	-0.031066472	0.073369	0.020993	-0.07465	0.002433
DYSF	0.019606826	-0.055427942	-0.18762	-0.02819	0.099217	0.350384
DYX1C1	-0.05927456	-0.113999693	-0.13531	-0.01613	0.052428	0.00843
DZANK1	-0.023419424	0.071552416	0.177444	-0.00251	0.035343	-0.13064
DZIP1	-0.071710254	-0.106809517	0.023614	0.01886	-0.0573	-0.10283
DZIP1L	-0.026874715	-0.092445219	-0.0844	0.026573	-0.07456	0.115503
DZIP3	0.015383712	0.070491055	0.194077	0.049217	-0.09707	-0.21593
E2F1	-0.012968666	-0.018264118	0.004943	0.06877	-0.29457	-0.09232
E2F2	-0.062125012	0.045979707	-0.12354	0.053238	-0.20846	-0.02984
E2F3	0.037890403	0.065843731	0.033342	0.025491	-0.06203	0.119935
E2F4	0.027786054	-0.053460312	-0.02616	0.040746	-0.14005	-0.09158
E2F5	-0.006107538	0.025955962	-0.10589	-0.01322	0.044815	-0.30614
E2F6	0.004213404	0.04942391	0.013796	0.036589	-0.1591	0.044492
E2F7	-0.019869254	-0.016764126	0.008565	0.08647	-0.21683	-0.11844
E2F8	-0.017188899	0.009735135	0.000238	0.160812	-0.27116	-0.01339
E4F1	0.024123351	-0.012791317	-0.07986	-0.01513	-0.00965	-0.06996
EAF1	0.036230341	0.158603327	-0.00712	0.056994	-0.16057	0.185094
EAF2	-0.081360718	-0.093321278	-0.12312	-0.07464	0.034138	0.000243
EAPP	0.069591013	0.051750589	0.014584	-0.02522	0.079198	-0.24297
EARS2	-0.015129954	0.007106249	0.164881	0.103336	-0.18817	-0.1723
EBAG9	0.033722876	-0.009987581	0.215826	0.015948	-0.03127	-0.02551
EBF1	0.012046585	-0.040918996	-0.12277	-0.00333	0.054778	-0.12772
EBF2	-0.033517532	-0.108641253	-0.00996	0.022747	0.013927	-0.1063
EBF3	-0.063154825	-0.061902196	-0.02383	-0.04442	-0.06932	-0.082
EBF4	-0.021687167	-0.013635533	-0.00526	0.001007	-0.02909	-0.17187
EBI3	-0.011240972	-0.039422699	-0.0153	0.063548	-0.22399	-0.05495
EBLN2	0.020738102	-0.052562223	0.011248	-0.01851	0.041691	-0.16784
EBLN3	0.012590414	0.042724183	0.06543	-0.01195	0.0314	-0.34336
EBP	0.016324513	0.165687231	0.153958	0.088676	-0.26251	-0.22906
EBPL	0.024747507	-0.029768161	0.206793	0.062604	-0.16249	-0.03314
ECD	0.036643003	0.079074364	-0.04891	0.040291	-0.14365	0.053702
ECE1	0.007848577	-0.083729882	-0.06995	-0.03573	0.048904	0.091716
ECE2	0.024688607	0.01422996	-0.10283	0.074745	-0.22971	0.100975
ECEL1	-0.033045868	-0.03733938	-0.08621	0.00525	-0.0744	-0.04734
ECH1	0.03713136	0.047192721	0.102467	0.026154	-0.08098	0.120335

ECHDC1	0.040430242	-0.002801808	-0.15136	0.020232	-0.02107	0.22968
ECHDC2	0.011858185	0.055794665	0.302765	-0.07462	0.204087	-0.31081
ECHDC3	-0.012769137	-0.153829498	-0.24284	-0.09021	0.206234	0.329551
ECHS1	0.042417288	0.014275171	0.014206	0.054576	-0.13654	0.197253
ECI1	0.018905508	-0.055126708	-0.06263	0.04286	-0.13956	0.212007
ECI2	0.018864005	0.027673869	0.184054	-0.01788	0.060278	0.002609
ECM1	-0.018249374	-0.09131203	-0.02292	-0.00848	-0.05431	-0.04412
ECM2	-0.041169379	-0.05411188	0.039803	0.041021	-0.09983	-0.00236
ECRP	-0.006404373	-0.100053222	-0.20034	-0.03373	0.108104	0.737153
ECSCR	-0.033879441	0.027889312	-0.07107	-0.00655	-0.11267	-0.14286
ECSIT	0.015258226	-0.07435468	0.050125	0.039874	-0.11038	0.134596
ECT2	0.005011833	0.012856367	0.004487	0.115222	-0.34498	0.079112
EDA	-0.034601185	-0.070015154	0.071875	-0.0193	0.004621	-0.07905
EDA2R	-0.02793674	-0.045521727	-0.05309	0.011116	-0.09399	-0.12989
EDAR	-0.019347881	-0.137952716	0.308865	-0.0601	0.118572	-0.19273
EDARADD	-0.01593523	-0.019256426	0.017326	-0.00883	-0.0005	-0.03306
EDC3	0.013430954	0.01964663	0.151051	0.036745	-0.16459	-0.12794
EDC4	0.028997324	0.014622297	0.141509	0.041811	-0.11669	0.030746
EDDM3A	-0.023357639	-0.007551462	0.038599	-0.08002	0.080372	0.005682
EDDM3B	-0.070230283	0.004586036	0.078837	-0.02931	0.072467	0.092959
EDEM1	0.037732054	-0.056628063	-0.00429	0.041622	-0.11986	-0.32633
EDEM2	0.020065643	-0.044810339	-0.15823	0.014058	-0.03745	0.156963
EDEM3	-0.027208237	-0.017531674	-0.09667	-0.05922	-0.01194	0.204938
EDF1	0.070201098	0.081677846	0.207701	0.068615	-0.18223	0.086967
EDIL3	-0.006784776	-0.08456962	0.001484	0.05473	0.032984	-0.03765
EDN1	-0.011149008	-0.045231709	-0.02181	-0.00379	0.017281	-0.12059
EDN2	-0.035835168	-0.093102219	0.015478	0.029345	-0.10572	-0.06956
EDN3	-0.015435173	0.003282236	-0.00511	0.007121	0.003944	-0.00826
EDNRA	-0.067914055	-0.009326601	-0.02382	-0.00643	-0.10262	-0.02193
EDNRB	0.009088055	-0.0916111	-0.00607	0.039094	0.061497	0.149874
EDNRB-AS1	-0.016804532	-0.058643714	0.081403	0.076565	-0.18586	-0.05865
EDRF1	-0.018672371	-0.035460023	0.005108	0.003962	-0.03491	-0.13912
EEA1	-0.052998924	0.055249794	-0.03475	-0.02795	-0.02572	0.369625
EED	0.012288839	0.027198258	0.103694	0.023932	-0.06985	-0.14189
EEF1A1	0.041880003	0.10807473	0.042639	0.08032	-0.03556	0.03952
EEF1A1P42	-0.004864594	-0.034713804	0.176105	-0.02694	-0.01471	-0.12949
EEF1A2	-0.024306366	-0.037964941	-0.08308	0.033851	-0.10747	0.04044
EEF1D	0.057887883	0.043426791	0.216143	0.027701	-0.06016	-0.16878
EEF1E1	0.009568529	0.021921353	0.131968	0.073048	-0.19396	0.02641
EEF2	0.045430181	0.050095501	0.131554	0.065768	-0.15439	0.121183
EEFSEC	0.006067035	-0.128107508	0.08902	0.03973	-0.18941	0.050562
EEPD1	-0.014792295	-0.127797343	-0.14153	-0.03433	0.038742	0.080448
EFCAB1	-0.079516849	-0.081819244	-0.02085	-0.02818	-0.08209	0.044281
EFCAB11	-0.017285111	0.039991145	-0.13318	0.069306	-0.20332	0.040069
EFCAB12	-0.032497836	-0.052260324	-0.08072	0.016105	-0.01458	0.087654
EFCAB13	0.009409091	-0.131560152	0.087246	-0.08044	0.110067	-0.18004
EFCAB14	0.038889952	-0.014771613	0.081459	0.008439	-0.00415	-0.05885
EFCAB2	-0.016468276	-0.0674762	-0.17344	-0.00775	0.019809	0.179464
EFCAB4A	-0.020623856	-0.057803411	-0.10545	0.031793	-0.13495	-0.00672
EFCAB4B	-0.05133573	0.064889436	0.051136	0.052896	-0.07257	-0.09253
EFCAB5	-0.073063118	-0.043242276	0.082205	0.021343	-0.04296	-0.04957

EFCAB6	-0.029003829	-0.021316703	-0.06401	0.049493	-0.00978	-0.03358
EFCAB7	-0.001752374	0.050769729	0.093845	0.028837	-0.05535	-0.13643
EFCC1	-0.043965075	-0.037944283	0.159791	0.013723	-0.02932	-0.04953
EFEMP1	-0.064898059	-0.058465948	-0.00857	-0.04371	0.053758	-0.02259
EFEMP2	-0.016745977	-0.000704614	0.04664	-0.02708	0.04408	0.18956
EFHB	-0.068142354	-0.102091493	-0.02148	-0.03408	0.124585	0.001673
EFHC1	-0.071403458	-0.025954642	-0.10054	-0.07327	0.047656	-0.1105
EFHC2	-0.015440647	0.170310891	0.126699	-0.11292	0.340105	-0.2279
EFHD1	-0.019702489	-0.062168156	0.362231	-0.01806	0.052825	-0.02809
EFHD2	0.036791969	0.192304302	-0.24785	-0.00565	0.035244	0.224683
EFNA1	-0.027955259	-0.066546259	0.176282	-0.02995	0.067673	-0.18409
EFNA2	-0.057302165	-0.108455822	-0.00368	0.03067	0.041978	0.064434
EFNA3	-0.015532165	-0.059507101	0.021169	0.03191	-0.14051	-0.0361
EFNA4	4.24E-05	0.016064245	-0.20304	-0.00904	0.042874	0.184029
EFNA5	-0.072939387	0.156469205	0.070317	-0.00393	0.024719	-0.1281
EFNB1	-0.033261972	-0.115947695	0.007025	-0.01145	-0.01745	0.016073
EFNB2	-0.025290894	0.013966306	-0.03336	-0.00769	0.026372	-0.10696
EFNB3	-0.030467802	-0.052644947	0.024829	0.002283	-0.07077	-0.00997
EFR3A	0.048320834	0.163117785	0.044623	0.049866	-0.11294	0.107578
EFR3B	-0.032783588	-0.067442032	0.059145	-0.01075	-0.05493	-0.07393
EFS	-0.058961068	-0.121109137	-0.03135	0.001422	-0.01598	-0.10058
EFTUD1	0.028409975	0.054773111	0.008376	0.040351	-0.12477	-0.04018
EFTUD2	0.03961517	0.020683844	-0.13601	0.055421	-0.16492	0.262907
EGF	-0.023519428	-0.017884677	-0.00641	-0.23148	0.673089	-0.02517
EGFEM1P	-0.045398114	-0.061010403	0.095522	-0.01825	0.061512	-0.1137
EGFL6	-0.006201513	0.001484448	0.006295	0.011776	-0.11798	-0.00929
EGFL7	-0.020787449	-0.083284019	-0.00271	-0.03933	0.030441	0.004167
EGFLAM	-0.030156599	-0.014207292	-0.05505	0.015266	-0.06054	-0.00142
EGFLAM-AS	-0.056194815	-0.07612395	-0.13401	-0.00245	0.039836	0.089949
EGFLAM-AS	-0.037683222	-0.022931049	0.029323	0.009453	-0.01285	-0.07457
EGFR	-0.041905109	-0.132517335	0.049035	0.009057	-0.02478	-0.12026
EGLN1	0.005362336	-0.015043283	-0.1607	0.046815	-0.12062	-0.03254
EGLN2	-0.034530737	-0.017545985	-0.04252	-0.00744	0.046785	0.037769
EGLN3	-0.011781442	-0.077492689	-0.06272	-0.02344	0.057487	-0.16644
EGOT	-0.03407032	-0.054437397	-0.01533	0.037066	-0.08656	0.004636
EGR1	-0.01305543	-0.07282718	-0.20257	-0.02863	0.113064	0.335703
EGR2	0.004373047	0.005834331	-0.07791	-0.01965	0.035296	0.042842
EGR3	-0.006582022	-0.031444838	-0.10288	-0.02593	0.047523	-0.0511
EGR4	-0.034325303	-0.090593395	-0.11181	0.039158	-0.05886	0.093544
EHBP1	0.008990499	0.033526159	0.149183	0.026159	-0.05224	-0.08066
EHBP1L1	0.002720507	-0.105647144	-0.03545	-0.06474	0.168512	0.308872
EHD1	0.019296431	0.05358285	0.076599	-0.04046	0.089556	-0.17292
EHD2	-0.028509553	-0.059751873	-0.05561	0.020279	-0.06695	-0.09971
EHD3	-0.005659788	0.012718363	-0.05315	-0.15151	0.493216	-0.0102
EHD4	-0.042819452	-0.034106004	-0.05251	-0.01232	0.0355	0.054185
EHF	-0.045206833	-0.024719104	-0.04229	-0.01925	-0.01336	-0.03299
EHHADH	-0.015797003	0.012914083	0.012214	-0.01013	0.036961	0.128707
EHMT1	-0.038654707	0.046691107	-0.03217	-0.05389	0.106932	0.017402
EHMT1-IT	-0.028572887	-0.047964732	0.020954	-0.00637	-0.01869	-0.06028
EHMT2	0.015262034	-0.006123508	0.228154	0.049809	-0.02545	0.033817
EI24	0.029469974	0.015769172	0.141636	0.1025	-0.28588	0.102621

EID1	0.018572015	0.062254211	0.132003	0.006475	-0.06435	0.190447
EID2	0.033373824	0.093039747	0.260228	0.025179	-0.04395	0.073814
EID2B	-0.000825053	0.134705361	0.126496	0.028872	-0.04956	-0.00628
EID3	-0.005176708	0.088960596	0.253806	-0.04807	0.143792	-0.23682
EIF1	0.079591842	0.026518433	-0.00397	0.055518	-0.12716	-0.26354
EIF1AD	0.0263012	0.067641181	-0.03845	0.036281	-0.13569	0.041789
EIF1AX	0.0154195	0.019016607	0.175261	0.062921	-0.11107	-0.05256
EIF1AY	0.007943442	-0.029178952	-0.08631	-0.08268	0.234489	-0.00274
EIF1B	0.05178524	-0.012634142	-0.06825	0.035546	-0.04749	0.025818
EIF1B-AS	-0.036726569	-0.039789609	-0.00375	-0.00152	-0.04343	-0.13483
EIF2A	0.043196794	0.01728039	0.195552	0.028473	-0.0652	0.144733
EIF2AK1	0.043138639	0.099248455	0.001631	-0.00966	0.029544	0.1057
EIF2AK2	0.041629166	0.070512477	-0.05807	0.043899	-0.05554	-0.05747
EIF2AK3	0.012852801	0.079134727	0.030166	0.008495	-0.02049	-0.11581
EIF2AK4	0.007690869	-0.056519363	-0.08287	-0.02602	0.099433	0.181128
EIF2B1	0.040545489	0.054398284	0.042093	0.019358	-0.04249	0.128119
EIF2B2	0.037994005	0.198490069	-0.0132	0.073904	-0.25237	0.017808
EIF2B3	0.026679047	0.014087075	0.070532	0.092457	-0.28127	0.032195
EIF2B4	0.054835895	0.010611472	0.032709	0.064049	-0.20654	0.121855
EIF2B5	0.019860406	-0.006195952	0.110306	0.041688	-0.11498	-0.0521
EIF2D	0.028060749	-0.059948471	0.217156	0.034354	-0.07337	0.021778
EIF2S1	0.013358959	0.141710824	0.068304	0.020903	-0.09	0.082595
EIF2S2	0.049286355	0.073110349	0.020726	0.087381	-0.2574	0.053607
EIF2S3	0.05659817	-0.016447691	0.109472	0.0359	-0.06677	0.076227
EIF3A	0.092775323	0.116241301	0.067747	0.033335	-0.07653	0.13983
EIF3B	0.042583431	0.044742906	0.088235	0.034897	-0.10079	-0.00897
EIF3C	-0.007562276	0.065131165	-0.02045	-0.01256	0.053535	-0.05515
EIF3D	0.07450935	0.018480893	0.133806	0.056759	-0.12387	-0.10349
EIF3E	0.029506476	-0.018797084	0.15179	0.052159	-0.12071	0.167155
EIF3F	0.003524878	0.019012097	0.117052	-0.03155	0.141212	0.06855
EIF3G	0.050359693	0.16537657	0.195648	0.05016	-0.11401	0.133171
EIF3H	0.030438156	-0.038409034	0.151838	-0.00043	0.033287	0.171578
EIF3I	0.045807519	0.062206194	0.128227	0.068986	-0.20195	0.043225
EIF3J	0.030116485	0.104537612	0.043579	0.051968	-0.13952	0.055576
EIF3J-AS	0.010086952	0.024765206	0.214801	0.008616	########	-0.11514
EIF3K	0.06506528	0.027296622	0.195697	0.034402	-0.07008	0.139095
EIF3L	0.052520364	-0.00914996	0.08808	0.026738	-0.04885	0.140224
EIF3M	-0.016919685	-0.054269797	0.099052	-0.01631	0.088415	0.024918
EIF4A2	-0.045487493	-0.027967829	0.09675	-0.01181	0.01791	0.081959
EIF4A3	0.067944829	0.025363692	-0.16638	0.077179	-0.21745	-0.03277
EIF4B	0.048824971	0.05851464	0.290382	0.023119	-0.02324	-0.17679
EIF4E	0.007645151	0.081341816	-0.0837	0.058104	-0.17842	0.026437
EIF4E2	0.03017964	0.0567881	-0.27633	0.017358	-0.0546	0.473887
EIF4E3	0.013810872	0.071609127	-0.00019	0.007665	0.019865	0.226174
EIF4EBP1	0.013969634	-0.101348852	-0.37165	-0.00889	0.017551	0.536747
EIF4EBP2	0.028012195	0.069157624	-0.03309	-0.04607	0.081392	0.089245
EIF4ENIF	0.014584854	0.034119951	-0.02114	0.006909	-0.02667	-0.10318
EIF4G1	0.025396909	0.057791187	-0.00697	0.084964	-0.22897	0.224902
EIF4G2	-0.022846019	-0.024062059	-0.00605	-0.02328	-0.02364	-0.03521
EIF4G3	-0.017885405	0.11322465	-0.22065	0.076625	-0.11257	0.13211
EIF4H	0.11074109	0.121581466	-0.03139	0.11946	-0.2821	0.187387

EIF5	0.10246788	0.079216713	-0.03713	0.043773	-0.14712	-0.00996
EIF5A	0.045479546	0.036981643	0.1118	0.093428	-0.24953	-0.02054
EIF5A2	-0.014431892	0.269531669	0.211181	-0.04836	0.204776	-0.21183
EIF5B	0.024480172	0.042807626	0.123011	0.015716	-0.10508	-0.00406
EIF6	0.045543523	0.021343407	-0.13211	0.095443	-0.267	0.173901
ELAC1	-0.003408036	0.071687385	0.08461	0.002641	0.022313	-0.02676
ELAC2	0.022097136	0.051194077	0.058558	0.077276	-0.20342	-0.0178
ELANE	-0.008481981	-0.113365102	0.070486	-0.01626	0.058263	-0.08088
ELAVL1	0.000574464	0.039140035	0.067554	0.084439	-0.22364	0.036012
ELAVL2	-0.024237366	-0.048094042	0.041223	0.015179	-0.0697	-0.07825
ELAVL3	-0.032241715	-0.066890978	0.05349	0.000881	0.00016	-0.11883
ELAVL4	-0.004497706	-0.105351582	-0.00088	0.013478	0.033509	-0.02269
ELF1	0.0927917	0.142531433	0.103209	0.002013	0.043778	-0.31797
ELF2	0.083297324	0.003489892	-0.03515	0.037958	0.00837	-0.19423
ELF3	-0.034607344	-0.022829356	0.166676	0.004104	-0.03364	-0.08257
ELF4	0.05561129	0.184791113	-0.22374	-0.03523	0.10077	0.187041
ELF5	-0.051379418	-0.100251438	-0.00398	0.045079	-0.09716	0.0861
ELFN1	-0.021768529	-0.048600716	0.021187	0.036675	-0.09249	-0.04168
ELFN1-AS1	-0.028307234	-0.038214718	-0.01798	0.023454	-0.08225	-0.04555
ELFN2	0.002206176	-0.061299936	0.193472	0.105878	-0.08475	-0.21496
ELK1	-0.023764581	-0.120907703	0.110668	0.027651	-0.16801	-0.11996
ELK2AP	-0.010547041	-0.078558287	-0.0198	0.023023	-0.1681	0.011757
ELK3	0.016236544	0.034900114	0.057651	-0.04998	0.149172	0.260437
ELK4	-0.028423502	0.029851489	0.097099	0.192887	-0.13901	-0.09716
ELL	-0.004123315	-0.090933771	-0.1238	0.011917	-0.04325	0.154839
ELL2	0.000288711	0.015692757	-0.07597	0.094152	-0.22433	0.037439
ELL3	-0.003406291	-0.084464678	-0.0852	-0.01565	0.057193	-0.20848
ELMO1	0.030209898	-0.053714329	-0.22324	0.050566	-0.13547	-0.06287
ELMO2	0.028581587	0.136710113	0.114108	0.002431	-0.00937	-0.12697
ELMO3	-0.018458183	0.027445536	0.031269	0.0068	0.001517	0.087374
ELMOD2	-0.017127836	0.022464535	-0.0496	0.003884	-0.01984	0.090744
ELMOD3	-0.001678666	-0.059680787	0.014066	-0.03431	0.012457	0.092157
ELMSAN1	0.014255596	-0.025707523	-0.19269	0.027298	-0.0472	0.042927
ELN	-0.042624271	-0.07217647	-0.0392	0.02469	-0.10566	-0.03147
ELOF1	0.062274763	-0.014584559	-0.01472	0.042308	-0.17073	0.078603
ELOVL2	-0.043439779	-0.106419788	-0.01014	0.009323	-0.00964	0.01873
ELOVL2-AS	-0.024247973	0.012052505	0.075633	0.006643	-0.01201	-0.08868
ELOVL3	-0.038483512	-0.074524952	-0.14811	-0.01224	0.064233	0.491944
ELOVL4	-0.014175952	-0.036291428	0.240011	0.074375	-0.13567	-0.09076
ELOVL5	-0.004381712	-0.077703462	-0.16348	0.01337	-0.07335	-0.19324
ELOVL6	-0.006866249	0.275299819	0.013741	0.062181	-0.19608	-0.11755
ELOVL7	-0.013894175	-0.047563856	-0.02587	-0.16748	0.41922	0.019833
ELP2	-0.069987926	-0.034564744	0.254006	-0.08514	0.151668	-0.33911
ELP3	0.034158296	0.132860633	0.147216	0.032312	-0.1278	-0.08339
ELP4	0.008639604	-0.002134385	0.038525	0.027536	-0.0782	0.143261
ELP5	0.019701098	0.072999116	0.138189	0.073025	-0.22308	0.028073
ELP6	-0.004146389	0.006466873	0.120217	0.055243	-0.30699	-0.09077
ELSPBP1	-0.038290953	0.026468987	-0.02081	0.010906	-0.02815	0.080793
ELTD1	-0.066800106	-0.11573289	0.157962	0.03981	-0.0803	0.077261
EMB	0.024116709	0.053785207	0.154858	-0.01264	0.054544	-0.08345
EMC1	0.012155507	0.030419847	0.062151	0.023639	-0.05621	0.204089

EMC10	0.01952126	-0.085974788	0.208281	-0.12073	0.29797	0.075628
EMC2	0.035244274	0.111141754	-0.07103	0.045058	-0.13065	0.116275
EMC3	0.04091104	0.076116152	-0.19345	0.028008	-0.10569	0.127779
EMC3-AS1	0.001063897	-0.124373097	0.039523	0.095268	-0.26837	-0.16173
EMC4	0.111231909	0.158230919	0.212546	0.094188	-0.25032	0.027773
EMC6	0.043713078	0.079022656	0.084364	0.051138	-0.16152	0.220728
EMC7	0.055298895	0.074241943	-0.13809	0.091712	-0.23829	0.228398
EMC8	0.058785136	0.046115962	-0.10029	0.101388	-0.24863	0.293317
EMC9	0.019536536	0.034313647	-0.03805	0.126835	-0.39599	-0.00225
EMCN	-0.057428278	-0.032117654	0.105418	-0.00977	0.032934	0.037744
EMD	0.02184338	-0.022462644	0.08647	0.018999	-0.12359	-0.13304
EME1	0.00550514	0.01375171	-0.12364	0.094785	-0.29787	0.102424
EME2	0.022223194	-0.095068783	0.048115	0.059645	-0.06567	-0.04615
EMG1	0.026134857	0.062976276	0.214551	0.022186	-0.06252	-0.0055
EMID1	-0.025572102	-0.062600538	-0.02842	0.00232	-0.06613	-0.02146
EMILIN1	-0.021141004	-0.089853516	-0.07229	0.00864	0.012187	0.119693
EMILIN2	-0.018905631	-0.094814087	-0.2286	-0.18741	0.468414	0.474036
EMILIN3	-0.034967677	-0.012656088	-0.07673	-0.0162	0.040949	-0.04535
EML1	-0.069883066	-0.030743634	-0.02312	-0.00937	0.031472	0.00838
EML2	-0.053680195	0.005711755	0.062817	-0.03502	-0.12641	-0.07262
EML3	0.024791003	-0.008719462	0.148197	0.007987	0.008301	0.052081
EML4	-0.003999282	-0.042524201	0.221664	0.037545	-0.10735	-0.35663
EML5	-0.053974324	-0.077520158	0.136698	-0.01402	0.030872	-0.13689
EML6	-0.012240528	-0.033337234	-0.08294	-0.0035	0.02653	-0.17678
EMP1	-0.005754361	-0.032125004	-0.03871	-0.00911	-0.03134	0.045864
EMP2	-0.03187852	-0.077417172	-0.02834	0.018368	-0.00152	-0.00978
EMP3	0.037475589	0.08233159	0.123854	0.02439	-0.05124	0.239563
EMR1	0.00167538	-0.115926653	-0.24033	-0.05782	0.168234	0.392188
EMR2	-0.000360826	-0.127565532	-0.40945	-0.08226	0.233466	0.401783
EMR3	-0.006194366	-0.078331522	-0.23509	-0.06344	0.163015	0.123795
EMX1	-0.043937933	-0.023007084	-0.01949	-0.00354	0.042157	0.183959
EMX2	-0.057803533	-0.026004941	-0.09314	-0.03317	0.108904	0.013265
EMX2OS	-0.047116874	-0.099792156	0.158673	-0.01226	0.036212	-0.01046
EN1	-0.065257546	0.021420623	0.03658	-0.01008	0.021776	0.078639
EN2	-0.020428864	0.020223205	-0.01792	-0.0055	-0.00336	-0.11075
ENAH	-0.037953211	-0.203318887	-0.08504	0.038963	-0.01973	0.034005
ENAM	-0.026073603	-0.102910506	-0.02521	-0.00019	0.017978	-0.21428
ENC1	0.00253402	0.063704516	-0.07483	-0.04972	0.138192	0.143886
ENDOD1	0.011520537	0.146888374	0.086236	-0.04614	0.110779	-0.18315
ENDOG	0.020126814	0.004701229	0.039226	0.0329	-0.07468	0.208916
ENDOU	-0.046033885	-0.08385621	0.007289	0.01047	0.000451	-0.1087
ENDOV	-0.033545879	-0.085121562	-0.1136	0.009288	-0.08305	0.090963
ENG	-0.005205309	-0.017809579	-0.10785	-0.09986	0.207958	0.310784
ENGASE	0.038381924	-0.079407826	0.235186	-0.07367	0.188531	0.075738
ENHO	-0.004232391	-0.071513301	-0.09755	-0.01497	0.027104	-0.0507
ENKD1	-0.005772173	-0.1322504	-0.08734	0.005887	-0.00336	0.114565
ENKUR	-0.029659986	-0.028937573	0.039473	-0.17524	0.524773	-0.05245
ENO1	0.001227584	0.036704632	0.103132	0.06939	-0.19394	0.088821
ENO1-AS1	-0.017173787	-0.052810702	0.172969	0.076686	-0.19669	-0.02264
ENO2	-0.005736284	-0.009257695	0.443078	-0.03938	0.083229	-0.3757
ENO3	-0.01876979	-0.025327642	0.182488	0.037996	-0.0828	-0.06617

ENOPH1	0.032404021	0.099002351	0.19887	0.052818	-0.13762	0.008342
ENOSF1	0.005746632	0.028597475	0.441777	0.048124	-0.13744	-0.29161
ENOX1	-0.013408073	-0.032208047	-0.02842	0.080285	-0.20615	-0.09729
ENOX2	-0.050700469	-0.020264982	-0.02716	0.03931	0.043316	0.202098
ENPEP	-0.028764499	-0.024875012	-0.05119	0.317429	-0.49699	-0.04769
ENPP1	-0.06452199	-0.112774822	-0.00458	-0.03954	0.040247	-0.15442
ENPP2	-0.000425336	-0.103800134	-0.10295	0.049666	-0.12591	-0.1634
ENPP3	-0.045670048	-0.094061292	0.071193	-0.0282	0.044968	-0.15578
ENPP4	0.005854416	0.236740831	0.197351	0.015517	-0.03037	-0.27793
ENPP5	-0.009327636	0.363882134	0.186631	-0.07291	0.221281	-0.19332
ENPP6	-0.035837448	0.004677453	-0.0077	-0.01743	-0.04778	-0.0206
ENPP7	-0.014733456	-0.005299129	-0.09076	0.01139	-0.09149	-0.08629
ENSA	-0.007809056	0.127055582	0.099489	0.047774	-0.17824	-0.01171
ENTHD1	-0.070713981	-0.062384958	0.04741	-0.03799	0.082941	0.056778
ENTHD2	-0.037782227	-0.108071884	0.004469	-0.03696	-0.04247	-0.14087
ENTPD1	-0.013676505	-0.125930157	-0.36495	-0.03446	0.060392	0.295339
ENTPD1-AS	0.00911983	0.044103537	0.085701	-0.00414	-0.03318	-0.32265
ENTPD2	-0.03307978	-0.04995477	-0.05917	0.001984	-0.03834	-0.03062
ENTPD3	-0.026381405	-0.06704724	-0.02016	0.009646	-0.01187	-0.13088
ENTPD3-AS	-0.031593432	-0.025151354	-0.09878	0.001998	-0.00261	0.028826
ENTPD4	0.006144385	-0.013925443	0.014166	-0.00937	-0.00126	-0.17671
ENTPD5	-0.035134364	0.070556243	0.026597	-0.01035	-0.06573	-0.13672
ENTPD6	0.016625715	-0.151254185	0.041507	-0.02708	0.065205	0.2199
ENTPD7	-0.002123806	0.016990245	-0.07171	-0.00042	-0.03389	0.004845
ENY2	0.022137302	-0.011901285	-0.17932	0.003328	0.019064	0.374198
EOGT	0.007848093	0.002195731	0.091981	0.066392	-0.1318	0.059393
EOMES	0.004526439	0.27294294	0.456118	-0.02694	0.042277	-0.25949
EP300	0.040661176	0.003332186	-0.2744	-0.01798	0.04679	0.101039
EP300-AS	-0.051694551	-0.012309445	0.032658	0.046051	-0.08876	-0.03957
EP400	0.010399297	0.038774154	0.091782	0.031021	-0.09268	-0.05955
EP400NL	0.032641464	-0.052994457	0.0833	0.056025	-0.11699	-0.15457
EPAS1	-0.00414255	0.012181969	-0.09011	0.003413	-0.04637	-0.01033
EPB41	-0.003099485	0.011337656	0.123829	-0.04563	0.158335	-0.28642
EPB41L1	-0.002584674	-0.069553522	-0.07591	0.040313	0.011363	-0.13737
EPB41L2	-0.00856289	-0.108602649	-0.11547	-0.00016	0.099661	0.025766
EPB41L3	0.002944694	-0.114779732	-0.26992	-0.0988	0.270958	0.694001
EPB41L4A	0.024426449	0.166466988	-0.05155	-0.05027	0.047898	-0.03845
EPB41L4A-	0.018137982	0.001875067	0.242759	0.000435	0.021491	0.006168
EPB41L4A-	-0.033859051	0.024232637	-0.10309	-0.01561	0.065379	-0.02901
EPB41L4B	-0.029864023	-0.019962985	0.014579	0.024312	0.047268	-0.17001
EPB41L5	-0.009715811	0.030490408	0.095009	-0.01176	0.066288	-0.0264
EPB42	-0.030564763	-0.053959668	0.025749	-0.03617	0.094132	-0.19505
EPC1	-0.001214627	0.05012203	0.192906	-0.09055	0.138595	-0.32166
EPC2	0.026453906	0.091516029	0.030698	-0.00947	0.009788	-0.14338
EPCAM	-0.019270345	-0.017805831	-0.01903	0.008219	-0.02629	-0.06813
EPDR1	0.000736349	0.051868705	-0.02425	-0.02663	0.080436	-0.10517
EPG5	-0.05151962	0.155280837	-0.02853	-0.01645	0.008162	-0.04505
EPHA1	0.06300771	-0.172097341	0.196644	-0.02564	-0.01662	-0.1478
EPHA1-AS	-0.012670135	-0.139911838	0.327718	-0.03224	0.101505	0.124143
EPHA10	-0.085136597	0.022506418	-0.05265	0.022952	-0.03951	0.018481
EPHA2	-0.007876497	-0.071268259	-0.12038	-0.00089	0.020662	-0.16883

EPHA3	-0.142485169	-0.098486637	0.18761	-0.09658	0.119977	-0.05168
EPHA4	-0.020020392	0.133900622	0.041564	-0.03161	0.139498	-0.2428
EPHA5	-0.051215062	-0.062069399	0.004891	-0.00472	0.023476	0.072191
EPHA5-AS:	-0.026184717	-0.070163639	-0.04535	0.004266	-0.00975	-0.07799
EPHA6	-0.061566522	-0.067978017	-0.05495	0.009803	-0.01769	-0.04024
EPHA7	-0.106370377	-0.029405773	-0.07439	-0.02798	-0.03729	-0.04715
EPHA8	-0.035498573	-0.036540651	-0.0395	-0.00316	-0.04568	-0.05548
EPHB1	-0.039455435	-0.082685248	-0.11195	-0.03312	0.05288	-0.16318
EPHB2	0.000104121	-0.160077886	-0.07283	-0.02456	0.012788	0.414933
EPHB3	-0.034964583	-0.111835734	0.012853	-0.01342	-0.02296	0.068138
EPHB4	-0.007531922	-0.168672781	0.039537	-0.02245	-0.01844	-0.02274
EPHB6	-0.007196731	-0.218845163	-0.0086	-0.07385	0.219536	0.069308
EPHX1	-0.014722172	-0.096564399	-0.06504	-0.17226	0.464341	0.163447
EPHX2	-0.005902976	-0.113250239	0.485863	-0.07095	0.215926	-0.25912
EPHX3	-0.024129076	-0.011549973	-0.10088	0.049863	-0.10672	0.042659
EPHX4	-0.012352635	0.073686431	0.101904	-0.02897	0.072276	-0.06503
EPM2A	-0.047741306	-0.057451279	0.027004	0.131558	-0.11134	-0.13287
EPM2AIP1	0.029612217	-0.008857546	0.076455	0.041426	-0.09482	0.015252
EPN1	0.001398395	-0.097019391	0.005478	-0.00864	-0.0761	0.044411
EPN2-AS1	-0.034644802	-0.046422799	-0.0433	0.008162	-0.00585	-0.0623
EPN3	-0.04834064	-0.007749505	-0.02757	0.017273	-0.02648	0.038661
EPO	-0.039183601	-0.087938418	-0.08252	0.004187	0.009747	0.040701
EPOR	0.000325562	-0.067640264	-0.14246	-0.05872	0.169075	0.186875
EPPK1	-0.029435419	-0.098429131	0.319223	-0.04637	0.257756	-0.15258
EPRS	0.02522862	0.064511474	0.180138	0.078432	-0.1539	-0.02863
EPS15	0.08325764	0.114942725	-0.10399	-0.00504	0.004215	-0.04565
EPS15L1	0.011818	0.069197321	-0.25872	0.016431	-0.06774	-0.05345
EPS8	6.55E-05	-0.066755725	-0.19243	-0.08185	0.241232	0.449099
EPS8L1	0.007526662	0.028411219	-0.109	0.030409	0.003164	0.142298
EPS8L2	-0.038642695	0.005714812	-0.14629	-0.02199	-0.02272	0.045375
EPS8L3	-0.034338421	-0.043759493	0.064777	0.018194	-0.04223	-0.03012
EPSTI1	0.022532475	0.052720861	-0.20745	-0.04538	0.126857	0.096559
EPT1	0.010181992	0.047655492	0.006131	0.081729	-0.18585	-0.02832
EPX	-0.023880467	-0.049824522	-0.02233	0.02542	-0.0577	0.077009
EPYC	-0.08431516	-0.042943334	0.089775	-0.01759	0.040704	-0.04021
EQTN	-0.10652224	-0.062703235	0.010106	-0.02334	0.008513	0.019388
ERAL1	0.01188071	-0.025534754	-0.04978	0.077483	-0.2406	0.138389
ERAP1	-0.019229327	0.106526969	0.118474	-0.10319	0.009284	0.04447
ERAP2	0.004864076	0.113157847	0.130215	-0.03636	0.074954	-0.10731
ERBB2	-0.035159255	-0.019092923	-0.1999	-0.02961	0.096816	-0.00503
ERBB2IP	0.050180103	0.1565999	-0.00914	-0.01924	0.066473	-0.16418
ERBB3	-0.096736296	-0.077053337	0.134823	0.020089	-0.07096	0.022649
ERBB4	-0.037115721	-0.021428809	0.005453	0.086403	-0.18202	-0.04554
ERC1	0.044539917	-0.078984209	0.060508	-0.0429	0.162214	0.172788
ERC2	-0.019757082	0.005109635	0.007939	0.139286	-0.21354	0.007726
ERC2-IT1	-0.018417454	-0.033437975	0.080092	0.073751	-0.19712	-0.00898
ERCC1	0.025478847	-0.051884836	-0.0595	0.0488	-0.07114	0.294187
ERCC2	-0.013496289	-0.127961711	0.00572	0.074713	-0.24812	0.122372
ERCC3	0.036403117	0.074334388	0.098771	0.023352	-0.05851	-0.1895
ERCC4	-0.019658954	0.064613935	0.09654	0.050761	-0.12914	0.08466
ERCC5	0.042343327	0.065666345	0.182993	-0.01888	0.057974	-0.14036

ERCC6	-0.005108108	0.060430289	-0.03827	0.028411	-0.10735	-0.09995
ERCC6L	-0.025198625	-0.001226558	0.026709	0.225965	-0.53524	-0.00012
ERCC6L2	0.005165159	0.0070067	0.24923	0.018142	-0.03038	-0.08351
ERCC8	-0.017658633	-0.001612412	0.051964	0.072723	-0.15683	0.214453
EREG	-0.017693763	-0.099005174	-0.05831	-0.03138	0.040171	0.19648
ERF	-0.011852881	-0.098160633	-0.01109	0.000463	-0.04583	0.083934
ERG	-0.035012414	-0.03895082	0.110306	0.025692	0.066054	0.028646
ERGIC1	0.023193508	0.00524693	-0.19154	-0.01081	0.033957	-0.08891
ERGIC2	0.00905953	0.077347092	0.201435	0.005673	0.048646	-0.26953
ERGIC3	-0.028287178	-0.078812175	-0.0569	0.004312	-0.04127	-0.05684
ERH	0.088717755	0.11025564	0.084801	0.14737	-0.36673	0.075174
ERI1	0.004290171	0.128450623	-0.03987	0.104241	-0.3011	-0.08218
ERI2	-0.072375531	-0.041516767	-0.06428	-0.03036	0.133641	0.039216
ERI3	0.005051551	-0.064128973	0.020363	0.081132	-0.21197	0.102846
ERICH1	-0.013097682	-0.039394984	-0.19032	0.009051	0.076397	0.42229
ERICH1-AS	-0.031056078	-0.032015283	0.036606	0.010906	-0.06825	0.036097
ERICH3	-0.053075815	0.038914973	-0.03191	0.005566	0.000289	-0.00592
ERICH3-AS	-0.038964423	-0.068613382	0.007888	-0.0034	0.035494	0.051985
ERICH4	-0.038161047	-0.011192763	0.002578	0.011566	-0.00767	-0.00617
ERICH5	-0.043032124	-0.070757854	-0.09967	-0.0034	-0.0019	-0.07397
ERICH6	-0.031915037	-0.02043898	0.016408	0.010447	-0.01679	-0.09729
ERLEC1	0.048059827	0.200544029	0.187891	0.059737	-0.13177	-0.28272
ERLIN1	0.019256182	0.020459133	-0.28539	0.00179	-0.00123	0.3472
ERLIN2	-0.021343884	-0.085713277	-0.0293	0.07545	0.080923	0.198501
ERMAP	-0.001990398	-0.05816298	-0.17264	-0.07264	0.222782	0.518807
ERMARD	0.028993559	0.012130933	0.170938	0.026194	-0.06886	-0.11119
ERMN	-0.002873446	-0.002909614	0.046661	0.067772	-0.13126	-0.05885
ERMP1	0.016695586	0.215316005	0.086702	0.046472	-0.15404	-0.1777
ERN1	0.020748739	0.07735635	0.031906	-0.02831	0.037452	-0.21106
ERN2	-0.001230451	-0.115026558	-0.0192	-0.01029	-0.06948	-0.09906
ERO1L	0.015435644	0.003880611	-0.08568	0.134231	-0.37226	0.059283
ERO1LB	0.004091933	-0.031830897	-0.09843	-0.00944	0.057236	-0.10227
ERP27	0.006348959	0.003980813	0.168154	-0.05461	0.183522	0.001047
ERP29	0.051298762	-0.112813464	0.100483	-0.01023	0.030172	0.118099
ERP44	-0.003325428	-0.152813339	-0.16807	-0.0671	0.134335	0.056577
ERRFI1	-0.007569729	0.032052757	0.049773	-0.00831	0.008473	-0.03479
ERV3-1	-0.018928454	-0.011603593	-0.05974	-0.03739	0.108083	-0.10712
ERV3-2	-0.00283659	0.037071922	-0.0596	-0.0098	0.030967	-0.0647
ERV9-1	-0.024315374	-0.047485875	0.047262	0.005001	-0.03124	-0.04369
ERVFH21-1	-0.040602786	-0.080491047	-0.0608	0.015596	-0.0238	0.173615
ERVFRD-1	-0.028157992	0.018782599	-0.01113	0.005328	-0.00352	-0.02588
ERVH-1	0.02796481	-0.061254037	0.074614	0.000735	-0.03904	-0.20858
ERVH-3	-0.036594596	-0.027449661	-0.11308	-0.00722	0.043879	0.063492
ERVH-4	-0.018310538	0.135394234	-0.01746	0.002983	-0.01416	-0.08063
ERVH-6	-0.054097307	-0.085859066	-0.03537	-0.02783	0.00204	0.022363
ERVH48-1	-0.040221386	-0.04627068	0.059816	0.029747	-0.05918	0.026501
ERVK13-1	0.057324909	0.008052294	-0.02756	-0.04898	0.090966	0.075217
ERVK3-2	-0.032497516	-0.002052001	-0.07843	0.007446	0.009456	0.039865
ERVMER34-	-0.055541799	-0.058644855	0.039947	-0.04742	0.169581	0.190366
ERVMER61-	-0.13009786	-0.059794428	0.100789	-0.02732	0.101045	-0.09702
ERVV-1	-0.046158272	-0.083078275	-0.01016	0.00497	0.012459	-0.04087

ERVW-1	-0.0426611	-0.046177654	-0.0165	-0.00632	0.037445	0.103084
ESAM	-0.024041141	-0.055712046	-0.06452	-0.21349	0.553312	-0.00635
ESCO1	0.005979988	0.099181145	0.116742	0.010938	-0.01384	-0.14648
ESCO2	-0.100265062	-0.013569768	0.005691	0.059435	-0.25097	-0.04752
ESD	0.026519543	-0.06653031	0.058209	-0.00109	0.009973	0.2327
ESF1	0.014034531	0.065108867	0.093358	0.041351	-0.10677	0.004393
ESM1	-0.026683056	-0.002317524	0.091559	0.016457	-0.05088	-0.0819
ESPL1	-0.006182329	-0.01911009	-0.00344	0.091688	-0.31475	-0.01065
ESPN	-0.031866598	-0.052789694	-0.08914	0.041596	-0.12091	-0.04237
ESPNL	-0.018189514	-0.070218333	0.008172	-0.00757	-0.09138	-0.17451
ESR1	-0.018619949	-0.077373988	-0.05776	-0.02976	-0.09201	-0.11413
ESR2	-0.016903574	-0.034035469	-0.04571	-0.00445	0.002169	-0.05076
ESRP1	-0.023800284	-0.020892474	0.011049	0.00434	-0.03114	-0.06826
ESRRA	0.020754838	-0.11564247	-0.11329	0.023593	-0.14005	0.247558
ESRRB	-0.024724161	-0.037610441	-0.07377	0.050471	-0.10323	0.003796
ESRRG	-0.057722814	-0.06215743	0.012936	0.000654	0.026782	-0.14251
ESX1	-0.052889925	-0.063551824	-0.03028	0.01876	-0.02765	-0.02476
ESYT1	0.032357712	0.058030959	0.216947	0.013997	-0.03059	-0.30516
ESYT2	0.057677145	0.207780032	0.250344	0.025443	-0.05051	-0.01997
ESYT3	-0.035772021	-0.032066379	-0.11973	-0.01283	0.063072	0.103537
ETAA1	-0.003294941	0.084091031	0.138787	0.03403	-0.05906	-0.04777
ETF1	0.052305559	0.023461126	-0.18917	0.078649	-0.20015	0.192935
ETFA	0.047510647	0.107567099	-0.02632	0.054803	-0.17063	0.288592
ETFB	0.037632971	-0.104759123	0.058652	0.071264	-0.20812	0.148783
ETFDH	0.011223096	0.037626465	-0.10481	0.080582	-0.19313	0.250014
ETHE1	0.02334322	0.011245915	0.111586	-0.0111	0.033248	0.330135
ETNK1	0.004191635	0.121273819	-0.18539	0.11258	-0.23413	0.076097
ETNK2	-0.033891324	-0.129699726	0.027826	0.041099	-0.09413	0.108572
ETNPPL	-0.066220271	-0.042110685	-0.03281	-0.03766	0.106299	0.01674
ETS1	0.004438648	0.147132258	0.378325	-0.03437	0.022175	-0.43195
ETS2	0.012339451	-0.079821098	-0.20963	0.01074	-0.00958	0.273452
ETV1	0.001054718	-0.03722031	-0.06737	0.066706	-0.03669	-0.05218
ETV2	-0.017421259	-0.080926529	-0.02817	0.043147	-0.09333	0.073605
ETV3	0.007866465	-0.02211541	-0.0032	-0.03612	0.085001	-0.07537
ETV4	-0.036041229	-0.064859498	-0.08772	0.022076	-0.10349	0.041698
ETV5	0.079579682	-0.072132867	-0.07385	0.113921	-0.32363	0.028716
ETV6	0.040369859	-0.113397755	-0.50449	-0.02024	0.082882	0.283671
ETV7	-0.019657533	-0.00579794	-0.03159	0.046795	-0.14565	0.012098
EU250746	-0.037564141	0.001731009	0.09776	0.001089	0.009686	-0.06082
EVA1A	-0.022991631	0.0031489	0.02607	0.058391	-0.16235	-0.04821
EVA1B	0.009090799	-0.028684619	-0.10388	-0.03792	0.019149	0.186112
EVA1C	-0.003688173	0.165136408	0.337147	-0.08025	0.240555	-0.19929
EVC	-0.035792201	-0.023934899	0.057379	0.022772	-0.05608	0.018151
EVC2	-0.039646285	-0.07818896	0.002982	0.012028	-0.01225	-0.02194
EVI2A	0.035025425	0.022578444	-0.02353	-0.09326	0.301839	0.241902
EVI2B	0.045783317	-0.105737551	-0.21518	-0.08837	0.300366	0.105869
EVI5	0.00975744	-0.078784694	-0.29935	-0.02064	0.095071	0.611989
EVI5L	-0.032453808	-0.045372208	-0.09366	0.01731	-0.01153	0.083474
EVL	0.02166352	0.123285303	0.31204	-0.02929	0.073641	-0.31063
EVPL	-0.029741306	-0.126331576	0.025697	0.028317	-0.05933	-0.00152
EVPLL	-0.03419824	-0.036365118	-0.01946	-0.01476	0.002169	-0.10946

EVX1	-0.03342432	-0.067382294	-0.07728	-0.02004	0.0207	-0.02695
EWSR1	0.028700743	0.014402436	-0.05347	0.02773	-0.10315	-0.04052
EXD1	-0.06653619	-0.088925961	0.127516	0.002272	-0.00708	0.056278
EXD2	-0.044911995	-0.087987879	0.14498	-0.09617	0.21557	-0.08726
EXD3	-0.012964115	-0.03425177	0.121609	-0.01547	0.101442	0.110254
EXO1	-0.018812462	0.017778335	-0.00566	0.209071	-0.47326	-0.07727
EXO5	-0.00577857	0.109778153	0.02253	-0.01221	-0.00961	-0.19915
EXOC1	0.058697458	-0.032823725	-0.20606	0.006236	0.012309	0.103252
EXOC2	0.013811434	0.097300418	0.202023	0.042783	-0.13335	-0.13711
EXOC3	0.020057522	-0.100879481	-0.09262	-0.01912	0.042765	0.061471
EXOC3L1	-0.030651825	-0.074135178	0.021062	-0.01447	-0.06113	0.018109
EXOC3L2	0.008782999	-0.067446352	0.042159	-0.01485	-0.06113	-0.14993
EXOC3L4	-0.038844493	-0.077944286	-0.08087	0.022943	-0.04391	0.116637
EXOC4	-0.058115615	-0.02108147	0.006892	0.002968	-0.03563	0.096519
EXOC5	0.02186173	0.073823911	-0.0573	0.029642	-0.13976	0.10384
EXOC6	-0.003600374	-0.008271065	-0.30638	0.001328	-0.00829	-0.00976
EXOC6B	-0.024932819	0.034380337	0.189902	0.008179	0.006409	0.121609
EXOC7	0.014495661	0.001234514	-0.0403	-0.09408	0.141467	0.07516
EXOC8	0.032432662	0.057526185	-0.14763	0.042395	-0.1058	-0.04021
EXOG	-0.029827746	-0.103303018	0.044223	0.020065	-0.07866	-0.06055
EXOSC1	-0.015230168	0.157081007	0.113021	-0.03581	0.133202	-0.10368
EXOSC10	0.020594391	0.03140055	0.122787	0.04034	-0.10204	0.021802
EXOSC2	-0.001681614	0.053358106	0.176014	0.065923	-0.19933	-0.04849
EXOSC3	0.01941034	0.029900609	-0.01615	0.083387	-0.26681	0.149392
EXOSC4	0.004003429	0.012223444	-0.13361	0.092783	-0.26197	0.299489
EXOSC5	0.019340693	-0.05517451	0.111677	0.051177	-0.15289	0.167888
EXOSC6	0.007400527	0.010120405	0.13106	0.018063	-0.14445	-0.24579
EXOSC7	0.034074712	0.083203442	0.174714	0.085544	-0.30229	-0.14942
EXOSC8	0.026137399	0.054668633	0.237045	0.067571	-0.18143	-0.11306
EXOSC9	0.046095661	0.071941845	0.205771	0.110255	-0.3425	-0.24421
EXPH5	0.008278968	-0.011370226	0.047834	0.00646	0.081946	-0.09025
EXT1	-0.01788529	-0.044097727	-0.04589	0.00128	0.007554	0.233693
EXT2	0.032849783	0.08495304	0.005752	0.029976	-0.14439	0.162168
EXTL1	-0.031471554	-0.049799089	-0.0284	0.028036	-0.06142	0.032729
EXTL2	-0.009144202	0.084452242	0.103949	0.116534	-0.36357	-0.21799
EXTL3	-0.004960358	0.057086194	-0.18459	-0.00795	-0.04231	0.051566
EXTL3-AS1	-0.027949353	0.060033289	0.015542	0.017841	-0.02455	-0.10852
EYA1	-0.032520078	-0.08049353	-0.08767	-0.00475	0.01768	-0.05359
EYA2	-0.03689808	-0.080636574	0.026669	0.036369	-0.08358	-0.08015
EYA3	-0.014915707	-0.00724005	-0.05229	0.029698	-0.08732	0.049074
EYA4	0.012868588	-0.012015039	-0.05112	0.040006	0.0807	-0.09755
EYS	-0.052294079	-0.02735945	-0.04481	-0.00579	-0.01	0.093065
EZH1	-0.007409871	-0.042566214	0.078784	-0.02502	0.013017	-0.11208
EZH2	0.018062187	0.110735212	-0.0049	0.12816	-0.34898	-0.05824
EZR	0.045620629	0.006986864	0.155812	0.052296	-0.05489	-0.11832
F10	-0.034677125	-0.025904247	0.094747	0.017611	-0.02293	0.116356
F11	-0.154462243	-0.059591501	0.018758	-0.04647	0.087938	0.045377
F11-AS1	-0.055158538	-0.006434006	-0.05208	-0.00381	0.038515	0.048633
F11R	0.020272835	-0.008715328	-0.28744	-0.09985	0.252206	0.092131
F12	-0.016318863	-0.076817311	-0.12451	0.021588	-0.10629	0.023096
F13A1	0.0119219	-0.071629761	-0.23982	-0.13125	0.385411	0.471808

F13B	-0.046548357	-0.028964366	-0.09061	-0.02479	0.064674	-0.03793
F2	-0.029234421	-0.092366784	0.034906	0.03116	-0.09247	0.153439
F2R	0.002004116	0.312817177	0.292887	0.054991	-0.16957	-0.2076
F2RL1	-0.008495294	-0.098680186	-0.18589	-0.10946	0.302416	0.308792
F2RL2	-0.04385967	0.022065018	-0.00765	0.014684	-0.03087	0.044987
F2RL3	-0.037177609	-0.03344586	-0.10188	0.01247	-0.0165	-0.01786
F3	-0.00780629	-0.010085923	-0.02253	-0.00688	-0.0038	-0.0027
F5	0.004161669	-0.095106734	-0.21357	-0.05605	0.124429	0.350153
F7	-0.021406181	-0.086799524	0.000138	-0.00167	-0.08932	-0.10043
F8	0.001734808	-0.009273507	0.132827	0.084145	-0.23737	0.211503
F9	-0.04329193	-0.054018377	-0.02192	0.013666	-0.02645	0.014559
FA2H	-0.036711582	-0.08115802	-0.01934	-0.02525	0.086645	-0.02485
FAAH	-0.011492959	-0.143802051	-0.15845	-0.02645	0.089333	0.238853
FAAH2	-0.017610282	-0.129205012	0.167169	-0.00918	0.009254	-0.30703
FABP1	-0.047673615	-0.034534342	-0.04576	0.010612	-0.03178	0.055475
FABP2	-0.034878423	-0.003381128	0.018926	0.008067	0.00731	0.111659
FABP3	-0.033947289	-0.027382868	-0.06912	0.024901	-0.05652	0.023393
FABP4	-0.03544878	-0.066565364	0.050109	-0.0102	0.007017	-0.03941
FABP5	0.015615429	-0.031715041	0.066845	0.0681	-0.20949	0.063737
FABP6	0.026918537	0.071960554	-0.05429	-0.04943	-0.20949	0.014021
FABP7	0.02684868	-0.015035437	0.049506	0.025449	-0.02093	-0.02815
FADD	0.02483726	0.055570606	-0.01382	0.05384	-0.19616	-0.01131
FADS2	-0.015687268	-0.005867772	-0.01379	0.008304	-0.08681	-0.04147
FADS3	-0.005702856	-0.049677019	-0.1103	0.019268	-0.07021	-0.1231
FAF1	-0.041532565	-0.0357278	0.176939	0.023793	-0.01697	-0.09545
FAF2	0.046520975	0.133289205	-0.02477	0.13101	-0.37561	0.045648
FAH	-0.007142239	-0.032834662	-0.06966	0.064632	-0.16143	0.270658
FAHD1	0.020181335	0.05291312	-0.0216	0.040707	-0.09505	0.302308
FAHD2A	0.007974833	-0.062070645	0.23603	0.12187	-0.29609	-0.14925
FAHD2CP	-0.021521765	-0.000559949	0.177269	0.09295	-0.21435	-0.18277
FAIM	0.006987493	0.132456249	0.088728	-0.03685	0.120342	-0.15481
FAIM2	-0.040876909	-0.071656936	-0.01985	0.006823	-0.0556	-0.00664
FAIM3	0.013287885	0.056286998	0.325176	-0.0986	0.250105	-0.38983
FAM101B	0.020120253	-0.012278475	0.067379	-0.0789	0.220805	0.075278
FAM102A	-0.0003661	-0.086051317	0.35882	-0.04329	0.120609	-0.26832
FAM102B	0.02574634	0.11751579	-0.13722	0.027147	-0.06296	0.28746
FAM103A1	0.061253818	0.128524326	-0.20199	0.061043	-0.17889	-0.05428
FAM104A	0.083389456	0.130890922	0.134963	0.045821	-0.13298	-0.3066
FAM104B	-0.013136176	-0.029943855	0.008011	-0.0057	0.025079	0.115345
FAM105A	0.023680923	0.02426764	-0.17588	-0.03357	0.140194	0.338438
FAM106A	-0.01380805	0.00486494	0.082428	-0.01441	0.056904	-0.01834
FAM107A	-0.034773586	-0.032143955	0.039938	-0.01813	0.003982	-0.07508
FAM107B	0.04312917	0.119604297	0.245469	0.008752	-0.02225	-0.37126
FAM109A	-0.006224576	-0.127040186	-0.02859	-0.03845	0.048556	0.091184
FAM109B	-0.009865795	-0.048952331	-0.15063	-0.07224	0.133641	0.107826
FAM110A	0.013270322	-0.054889723	-0.20605	-0.06181	0.12788	0.335445
FAM110B	-0.029044169	-0.07992285	-0.12616	-0.01419	0.058792	0.231831
FAM110C	-0.00777455	0.227791267	-0.03563	0.00759	1.48E-05	-0.08591
FAM110D	-0.030944831	-0.077609198	0.03778	0.016132	-0.03342	0.016097
FAM111A	0.018733039	0.094664014	0.060972	0.025813	-0.04705	0.06706
FAM111B	-0.014323242	-0.012662838	-0.08835	0.055352	-0.13024	-0.16454

FAM114A1	-0.026788118	-0.082019042	-0.26639	-0.06675	0.188378	0.57454
FAM114A2	0.017048215	0.022993577	-0.0621	0.0293	-0.08722	-0.03147
FAM115A	-0.021600701	0.061233487	0.088045	0.025941	-0.08301	-0.0453
FAM117A	0.032328703	0.131796382	0.245642	-0.0441	0.111456	-0.39489
FAM117B	-0.052395327	-0.186148201	0.067218	-0.09946	0.252687	0.107945
FAM118A	0.00505339	-0.130230013	0.036647	-0.01312	0.03773	0.09601
FAM118B	-0.018729165	-0.009457257	-0.2261	0.005116	0.016063	0.500805
FAM120A	-0.032761688	0.037914483	0.058439	0.065252	-0.02089	-0.04335
FAM120AOS	0.019193322	-0.054347159	0.098161	-0.01366	0.035292	0.117113
FAM120B	0.028361163	0.071675177	-0.0226	-0.02624	0.045382	-0.03512
FAM120C	-0.00037347	-0.021528664	0.113272	0.032811	-0.07136	-0.07062
FAM122A	0.012002524	0.117928864	-0.00809	-0.03384	0.093294	0.022213
FAM122B	0.029272434	0.083282132	0.206597	0.011828	-0.00824	-0.15371
FAM122C	-0.002156318	0.069894958	0.090743	-0.01093	0.092344	-0.0547
FAM124A	-0.042297622	-0.027579927	0.057782	-0.05963	0.005902	-0.11313
FAM124B	-0.02031801	-0.048667688	-0.12512	-0.02398	0.076592	0.205765
FAM126A	0.00225911	0.013681726	-0.12775	0.065704	-0.21116	0.054392
FAM126B	0.016890858	0.074154999	-0.09901	0.011109	-0.01917	-0.08736
FAM127A	0.032307459	0.005349704	-0.02665	-0.12729	0.308941	0.315997
FAM127B	-0.001036615	0.020870703	-0.01161	-0.0722	0.212917	0.31646
FAM129A	0.02647269	0.056893431	-0.0719	0.050356	-0.12087	0.037247
FAM129B	0.000881969	-0.122986375	-0.09651	-0.05158	0.068194	0.405964
FAM129C	-0.022185356	-0.102679142	-0.15126	-0.05348	0.094119	-0.21061
FAM131A	-0.026891411	-0.075386246	-0.02129	-0.0142	-0.10037	-0.0569
FAM131B	-0.028143191	0.003047527	0.023544	0.009422	-0.02076	-0.03946
FAM131C	-0.030123178	-0.085298423	-0.06908	-0.00786	-0.02968	-0.05645
FAM132A	-0.018979801	0.007027597	0.024947	0.041952	-0.10102	-0.00892
FAM132B	-0.016775962	0.284400149	-0.06694	-0.03085	0.09942	-0.09333
FAM133A	-0.073287998	-0.064879685	-0.02697	-0.01052	0.042436	0.105218
FAM134A	0.040318656	0.023322872	0.064263	-0.0027	0.001764	-0.04584
FAM134B	-0.004039917	-0.142330054	0.435985	-0.09286	0.276508	-0.10272
FAM134C	0.047944783	0.138130238	0.142417	-0.01716	0.041643	-0.19665
FAM135A	-0.080161692	-0.075661046	-0.03273	-0.01135	0.012079	0.152652
FAM135B	-0.035920209	0.00044109	-0.06246	0.009814	-0.01802	0.008643
FAM13A	0.030996958	0.052150779	-0.22749	-0.07975	0.180689	0.437954
FAM13A-AS	0.00352359	-0.088680607	-0.12662	-0.075	0.213033	0.069442
FAM13B	0.05511156	0.07672582	0.033921	-0.01029	0.042283	-0.26056
FAM13C	-0.100945949	-0.126197587	0.034541	-0.0275	0.0764	0.098034
FAM149A	-0.023222661	-0.119499102	0.120471	0.03115	-0.05184	-0.16479
FAM149B1	-0.007086063	0.011350848	-0.00868	0.031492	-0.05996	0.080646
FAM150B	-0.010689448	0.306219452	-0.13268	-0.01179	0.06867	-0.14189
FAM151A	-0.036756924	-0.071923867	-0.03754	-0.00695	0.053612	0.033367
FAM153A	-0.027273193	-0.050758183	0.065561	-0.01845	-0.01388	-0.13853
FAM154A	-0.028373382	-0.018729447	0.086828	0.008548	-0.06882	-0.19149
FAM154B	-0.040941304	-0.066329619	0.027538	-0.02107	0.045157	-0.20462
FAM155A	-0.066860776	-0.045755656	0.010452	0.030956	-0.05613	-0.08395
FAM155A-I	-0.074223157	-0.058600494	-0.03522	-0.05378	0.100663	-0.02501
FAM155B	-0.028924296	-0.033922939	-0.02925	-0.00934	-0.03728	-0.0074
FAM159A	-0.00783965	0.031043656	0.317581	-0.05089	0.139137	-0.37266
FAM160A1	-0.022176253	-0.073226248	-0.02983	-0.01022	-0.0344	-0.144
FAM160A2	0.028090662	0.022173618	-0.14015	-0.03283	0.099226	0.330767

FAM160B1	0.026773667	0.103220559	-0.08331	0.013721	-0.01861	-0.04053
FAM160B2	-0.016297357	0.046840715	0.060423	-0.00595	-0.02602	-0.11371
FAM161A	-0.008983859	-0.021980618	0.197962	0.063097	-0.15785	-0.18733
FAM161B	0.042950364	0.103546653	0.028891	-0.00317	0.035152	-0.13782
FAM162A	0.042326711	0.010696843	0.266764	0.084128	-0.20609	-0.14203
FAM162B	-0.020719453	-0.048801323	-0.00691	0.009074	-0.03471	-0.02528
FAM163A	-0.029478814	-0.120430488	-0.06138	-0.01282	0.058909	0.261747
FAM166A	-0.036527486	-0.079495066	-0.08315	0.052948	-0.19169	-0.04035
FAM166B	-0.029533665	0.025856652	0.091077	0.008049	-0.02381	-0.15052
FAM167A	-0.017154207	-0.047419586	-0.07733	0.013363	-0.06432	-0.08522
FAM167B	-0.023969757	-0.064334231	-0.07936	0.018963	-0.04296	0.054898
FAM168A	0.004887878	0.073169502	0.084963	0.000734	0.021192	0.1072
FAM168B	0.039051991	0.036135063	0.037967	0.084304	-0.23526	-0.10834
FAM169A	-0.005081328	0.162977252	0.292859	-0.08168	0.227077	-0.36875
FAM169B	-0.035259712	0.014737196	0.033249	0.007528	-0.00553	-0.08215
FAM170A	-0.031613373	-0.049026444	-0.05144	0.037334	-0.12165	-0.00202
FAM170B	-0.036067869	-0.024756582	-0.10768	0.044338	-0.10418	0.038348
FAM170B-/	-0.044900077	-0.047345603	0.082224	0.009702	0.00554	0.028998
FAM171A1	-0.002760785	-0.030094755	0.496481	-0.11874	0.343948	-0.19317
FAM171A2	-0.029206512	-0.044248183	-0.00506	0.026771	-0.03877	0.175277
FAM171B	-0.012235622	0.035438698	-0.04413	-0.02906	0.024886	-0.02594
FAM172A	0.020812798	0.13113009	0.102103	-0.01474	0.044757	-0.09115
FAM173A	0.013914321	0.03936605	0.206694	0.026615	-0.04804	0.121695
FAM173B	0.002974198	0.063226291	0.003804	0.037741	-0.12325	0.143654
FAM174A	0.018675481	0.0448077	-0.20094	0.0038	-0.00168	0.157694
FAM174B	-0.038106672	-0.096241624	0.000686	-0.01659	0.075427	-0.08681
FAM175A	0.007184903	0.012620903	0.109571	-0.01001	0.062887	0.142674
FAM175B	0.036774594	0.069253052	-0.09297	0.003197	-0.02308	0.02246
FAM178A	0.034288781	0.089224324	-0.01482	0.016344	-0.06469	0.019511
FAM178B	-0.022697093	-0.019446327	-0.00361	-0.00782	-0.02214	-0.01615
FAM179A	0.004022825	0.331202388	0.173478	0.035768	-0.08618	-0.06791
FAM179B	0.014670925	0.082306208	0.007283	-0.03023	0.108513	0.015898
FAM181A	-0.032932569	-0.090513289	-0.06271	-0.00622	0.009527	0.016473
FAM181A-/	-0.043045167	-0.065366422	-0.06843	-0.01177	0.060582	0.111486
FAM181B	-0.039535857	-0.120024292	0.077482	-0.01858	0.048794	0.065933
FAM183CP	-0.035307712	-0.021785793	-0.13617	0.023161	-0.07128	0.086948
FAM184A	-0.022121428	-0.079891557	0.146955	-0.0283	0.051089	-0.18267
FAM184B	-0.007609962	-0.039743871	0.046975	-0.02146	0.050176	0.028116
FAM185A	-0.006172505	-0.088579914	-0.05779	-0.003	-0.0011	-0.16575
FAM186A	-0.063898856	-0.035155988	0.105194	-0.01396	-0.00658	-0.05555
FAM186B	-0.024356876	-0.068939464	-0.08279	0.031164	-0.10732	-0.02503
FAM187B	-0.039229035	-0.015501156	-0.07449	-0.02478	0.095566	0.045226
FAM188A	0.008130423	0.055256703	-0.00013	0.060424	-0.12911	0.013306
FAM189A1	-0.041704296	-0.085035868	-0.02259	0.016643	-0.0236	0.055164
FAM189A2	-0.030623336	-0.083829315	-0.02324	0.004101	-0.04167	-0.01783
FAM189B	0.019068675	-0.094765343	0.078815	0.10726	-0.30203	-0.05568
FAM192A	0.072817222	-0.041722563	-0.13851	0.044284	-0.11279	-0.12218
FAM193A	0.063424742	0.028797602	-0.11352	0.017793	-0.08008	-0.11983
FAM193B	0.022035691	0.054280029	0.06739	-0.03195	0.079186	-0.29863
FAM195A	0.003455274	-0.127160942	0.048133	0.046018	-0.14439	0.297984
FAM195B	0.035915442	0.035837211	-0.00845	-0.00321	-0.09418	-0.02895

FAM196A	-0.028642271	-0.061705896	-0.05475	-0.01426	0.038117	-0.15284
FAM198A	-0.021149148	-0.043370403	0.032592	-0.00143	0.003094	-0.06301
FAM198B	-0.000173253	-0.07718464	-0.2653	-0.11422	0.328259	0.744167
FAM199X	0.069997411	0.256206823	-0.26655	-0.00998	-0.08647	-0.01972
FAM19A1	-0.008297718	-0.052161342	0.513446	-0.08964	0.273777	-0.26945
FAM19A2	-0.022963024	-0.105532827	-0.11252	-0.00827	0.029466	0.169582
FAM19A4	-0.105871742	-0.028227179	-0.01894	-0.0061	0.023659	0.0481
FAM19A5	-0.05480315	-0.065338379	0.217274	0.027623	-0.11714	-0.04282
FAM200A	-0.02340519	0.068131586	-0.01953	0.007399	-0.00718	-0.03387
FAM200B	-0.005578925	-0.047416478	-0.16509	-0.03782	0.074738	0.399352
FAM201A	-0.043755378	-0.084830013	0.136073	-0.01539	0.045213	-0.08494
FAM204A	0.017875452	0.006622076	-0.11061	-0.05221	0.138262	0.240677
FAM205A	-0.044721714	-0.069157994	0.046971	0.031319	-0.05596	0.068333
FAM205B	-0.057228075	-0.098830152	-0.03447	-0.02349	0.073235	0.014995
FAM206A	0.044052909	-0.080757578	-0.16156	0.001728	-0.00589	0.215674
FAM207A	-0.01698205	-0.096232124	-0.11222	0.092095	-0.2927	0.096897
FAM208A	0.095688942	0.144631266	0.183553	0.021478	-0.13848	-0.12986
FAM208B	-0.013261081	-0.031653455	0.089816	-0.00828	-0.00163	-0.13767
FAM209B	-0.004491994	-0.054108675	-0.0545	-0.02998	0.068365	-0.1862
FAM20A	-0.069822796	-0.063279911	0.034534	-0.01763	0.012943	0.131072
FAM20B	0.035817388	0.118916436	0.089118	0.040808	-0.11623	0.09574
FAM20C	0.045759001	-0.054838822	-0.17681	0.029024	-0.00185	0.048528
FAM210A	0.013254394	0.063898467	0.02054	0.07979	-0.18711	0.042368
FAM210B	0.018164655	-0.071548814	0.013607	-0.12723	0.356626	0.209715
FAM212A	-0.014702629	-0.177123317	-0.01147	-0.06235	0.108597	-0.08686
FAM212B-A	-0.030863785	-0.059761025	-0.03496	-0.11209	0.229326	-0.03155
FAM213A	-0.000362445	-0.103558175	0.052985	-0.12955	0.187976	-0.1335
FAM213B	0.016574682	-0.028063721	0.218043	-0.02928	0.092772	0.022784
FAM214A	0.013020291	0.026105046	0.043323	-0.00988	0.040344	-0.30373
FAM214B	0.01985497	-0.017345986	-0.24685	-0.0509	0.119652	0.23523
FAM215A	-0.026204042	-0.039420155	0.248692	-0.00247	0.02853	-0.14695
FAM216A	0.007600973	-0.049345204	0.164492	0.076635	-0.21913	0.088722
FAM216B	-0.054325058	-0.083718804	0.055125	0.007111	-0.01278	0.028413
FAM217A	-0.065987323	0.010409064	-0.06482	-0.00469	0.055721	-0.02917
FAM217B	0.02385471	0.126678513	0.028659	0.013855	-0.03345	0.038894
FAM218A	-0.049633448	-0.023724784	0.16285	-0.0153	0.061748	-0.2161
FAM219A	0.018683442	0.00066535	-0.11132	0.048923	-0.18845	0.057206
FAM219B	-0.002339314	0.096354224	0.078668	-0.03423	0.077479	-0.07575
FAM220A	0.035495503	0.05775587	0.0168	0.010805	-0.0449	-0.19146
FAM221A	-0.006049721	0.018432234	0.029049	-0.07313	0.202827	0.02465
FAM221B	-0.010409429	-0.048990475	-0.10259	0.012137	-0.01443	-0.11512
FAM222A	-0.021167096	0.030043699	-0.03847	0.011445	-0.02809	-0.00911
FAM222A-A	-0.043404793	0.000278466	0.07433	0.007616	0.000541	0.079933
FAM222B	0.036423333	-0.013157116	0.082336	-0.01864	-0.04828	-0.20594
FAM223B	-0.00137737	-0.119435044	0.076009	-0.08919	0.203146	-0.04516
FAM224A	-0.03824942	-0.025536829	-0.04565	-0.0113	0.044859	-0.05295
FAM227B	-0.072457765	-0.075247279	-0.04547	-0.02237	0.062592	0.039652
FAM228A	-0.034097753	-0.007630029	-0.00883	-0.02115	0.090239	0.126439
FAM228B	0.018892679	0.021975895	-0.06203	-0.0602	0.207731	0.138369
FAM229A	-0.010316507	-0.16402424	0.124603	-0.00214	0.009164	0.002555
FAM229B	-0.014092933	-0.028513648	0.260483	0.110604	-0.30795	-0.24562

FAM230B	-0.017614318	-0.011303803	0.179087	0.009509	-0.03991	-0.17929
FAM230C	-0.043997937	-0.03133466	-0.0767	0.006825	-0.00596	0.014339
FAM24A	-0.052670274	-0.08909977	0.028178	0.016203	-0.01702	-0.11905
FAM24B	0.010522122	-0.063510854	0.185604	0.00372	-0.02283	-0.13419
FAM26D	-0.0444288	-0.036398592	0.077684	0.002151	-0.00056	-0.03578
FAM26E	-0.035982431	-0.047529174	-0.06059	-0.0396	0.080312	0.025185
FAM26F	0.007190736	-0.062998791	-0.35946	-0.12185	0.290398	0.257269
FAM32A	0.096023794	0.027042559	-0.14644	0.00288	0.02661	0.191789
FAM35A	0.043071635	0.122724671	0.152691	0.02044	-0.04812	0.07106
FAM3A	0.017029433	-0.064121193	0.18419	0.008296	-0.05475	-0.05046
FAM3B	-0.02240606	-0.029884023	-0.10082	0.005522	-0.00137	0.152054
FAM3C	-0.034493668	-0.025617755	-0.05837	0.091536	-0.14742	-0.15498
FAM3D	-0.02924069	-0.120441759	0.056367	0.01039	-0.09932	-0.02817
FAM43A	0.005660054	0.118905855	-0.19619	-0.10714	0.29153	-0.05437
FAM43B	-0.025900976	-0.025388049	-0.15889	0.004125	-0.00647	0.062983
FAM45A	-0.052953971	-0.047782108	-0.03697	-0.00036	0.021729	0.055846
FAM46A	-0.023430512	-0.059969619	-0.1622	-0.03739	0.523638	0.221096
FAM46B	-0.028016641	-0.061902056	-0.04128	-0.01471	0.048173	0.00037
FAM46C	0.008460782	0.075532868	0.125104	-0.01134	0.002146	-0.41609
FAM46D	-0.048428684	-0.080765573	0.038087	0.012846	-0.03095	-0.01695
FAM47A	-0.053718163	0.002444759	0.04909	-0.00794	0.024283	-0.02258
FAM47B	-0.043872474	-0.048032108	0.001925	-0.00913	0.026304	0.132093
FAM47C	-0.043226106	-0.041523729	0.096687	-0.01153	0.05602	-0.10914
FAM47E	-0.047113202	-0.033746078	0.133712	-0.00352	0.0178	-0.15307
FAM49A	0.000166873	0.000710587	-0.50146	-0.09554	0.243682	0.415987
FAM49B	0.023497115	0.142742106	-0.07442	-0.05486	0.160417	-0.14579
FAM50A	0.052909033	0.060125793	-0.09316	0.067082	-0.20172	0.363081
FAM50B	0.008486002	0.035207132	-0.02742	-0.03702	0.054873	-0.08111
FAM53A	-0.025540376	-0.09482335	-0.07122	-0.01239	0.069079	0.020508
FAM53B	0.01650624	0.091710798	-0.13961	-0.02911	0.071559	-0.11468
FAM53B-AS	-0.049406857	-0.034407492	-0.00384	0.000961	0.013992	0.064539
FAM53C	0.043865754	-0.044461155	-0.22314	0.039973	-0.08731	-0.10972
FAM57A	-0.010435983	0.027773147	0.0728	0.053388	-0.19195	-0.09073
FAM58A	0.054613826	-0.043042282	-0.02414	0.020025	-0.0999	-0.17922
FAM60A	0.023857327	0.002815794	0.13827	0.052342	-0.15294	-0.2516
FAM63A	0.046125161	-0.068035788	-0.11959	-0.08842	0.239003	-0.12576
FAM63B	-0.006287901	0.114060063	0.014915	-0.01532	0.053076	-0.03371
FAM64A	-0.018290945	0.008087919	0.089652	0.13573	-0.39704	-0.01918
FAM65A	0.011981478	-0.138993791	-0.38205	-0.0988	0.23567	0.180754
FAM65B	0.022589291	-0.004277247	0.031272	-0.07454	0.202184	-0.14456
FAM65C	-0.026641354	-0.05274749	-0.07556	-0.02825	0.105075	0.029528
FAM66D	-0.029295222	-0.11103234	-0.02452	-0.00977	-0.00948	-0.04849
FAM69A	0.012320327	0.072643261	0.137481	0.009721	-0.01345	-0.45128
FAM69B	-0.002876042	-0.082078463	-0.12151	0.052135	-0.0365	0.077216
FAM69C	-0.021469977	-0.084738864	-0.13223	-0.0164	-0.05858	-0.05852
FAM71A	-0.025855468	-0.094103607	-0.09991	-0.02055	-0.02035	-0.07043
FAM71B	-0.036638555	0.04763023	-0.0143	0.073689	-0.0722	-0.0806
FAM71C	-0.032006814	0.009145922	0.068986	0.023127	-0.04967	-0.13592
FAM71D	-0.033026813	-0.031717667	0.086122	0.019716	-0.0381	-0.10415
FAM71E1	-0.03139426	-0.083143178	-0.07792	0.012488	-0.01355	0.079256
FAM71E2	-0.044549859	-0.068542949	0.075914	0.014455	-0.01123	0.099734

FAM71F1	-0.051853908	-0.023403563	-0.12256	0.018127	-0.01705	0.008756
FAM71F2	-0.028151541	-0.082229964	-0.05789	-0.01684	0.013509	-0.08264
FAM73A	-0.005588922	0.091723906	0.157171	-0.03137	0.089369	-0.00716
FAM73B	0.028272094	-0.027465291	0.217708	-0.04497	0.129076	-0.10855
FAM76A	-0.013067119	0.02029419	0.078847	-0.00376	0.037443	0.045404
FAM76B	-0.036803721	0.011528927	-0.05975	0.002006	-0.05754	-0.0424
FAM78B	-0.029199644	-0.020867189	-0.01609	0.075396	-0.2094	-0.05315
FAM81A	-0.027168697	-0.137808442	-0.04096	0.023175	-0.04435	-0.15935
FAM81B	-0.028314074	-0.05312985	0.063387	-0.15272	0.451188	0.024607
FAM83A	-0.028788076	-0.051132404	-0.01906	-0.00195	-0.04317	-0.07374
FAM83B	-0.05035129	-0.025367503	-0.03713	-0.00401	0.01373	-0.15099
FAM83C	-0.03962255	-0.082229288	-0.09462	0.023266	-0.04808	0.016954
FAM83C-AS	-0.027989734	-0.010137718	-0.05973	0.013989	-0.03023	-0.00696
FAM83D	-0.01325817	-0.039869685	0.074349	0.179124	-0.43916	-0.14259
FAM83E	-0.03118798	-0.069489348	-0.05249	0.012645	-0.00768	0.044691
FAM83F	-0.03163665	-0.063507367	-0.02387	-0.00149	0.028831	0.14191
FAM83G	-0.005870129	-0.02628864	0.007397	0.003698	-0.08395	-0.05238
FAM83H	-0.032747127	-0.041931967	-0.02761	-0.02588	0.077177	-0.00669
FAM84A	-0.044272137	-0.118902829	-0.00489	0.094034	0.018228	-0.15111
FAM84B	-0.008700124	0.009795389	0.38959	-0.01648	-0.00612	-0.33615
FAM86A	-0.009610852	-0.037196871	0.138754	0.077258	-0.25444	-0.15587
FAM86B3P	-0.041841994	-0.15161805	0.122134	0.060814	-0.06675	-0.05433
FAM86C1	-0.026765718	-0.036133944	0.014217	0.091188	-0.23899	0.021377
FAM89B	0.048896319	-0.021044133	-0.01062	-0.06469	0.154384	0.085309
FAM8A1	0.022647735	0.11035804	0.060792	-0.01091	0.044315	-0.08925
FAM90A1	-0.028384908	-0.033759723	-0.04716	-0.01397	0.029971	-0.01652
FAM91A1	0.028880112	0.002930256	-0.30725	0.027643	-0.09972	0.089773
FAM92A1	-0.028936575	-0.037904455	-0.10898	0.013198	0.038637	0.297767
FAM95A	-0.026671175	0.05782209	-0.00966	0.048395	-0.14259	0.095681
FAM96A	0.060381397	-0.054757037	-0.20939	0.041658	-0.10686	0.253635
FAM96B	0.065198435	0.083543769	0.064212	0.108107	-0.30061	0.172057
FAM98A	0.011239871	0.124959819	0.008498	0.046084	-0.13491	-0.08745
FAM98B	0.031995116	0.081979018	0.07543	0.069923	-0.17237	-0.01017
FAM98C	0.024164515	0.031142905	0.078929	0.006224	-0.02518	-0.06495
FAM99B	-0.027024097	-0.066520786	-0.02254	0.016026	-0.02083	0.040311
FAM9A	-0.057969819	-0.031538165	-0.02763	-0.01798	0.053336	-0.04251
FAM9B	-0.055514797	-0.064904221	-0.07458	-0.00249	0.012369	-0.01872
FAM9C	-0.050316548	-0.071451388	-0.08421	########	0.019872	0.176251
FAN1	-0.043737348	0.011677832	0.057155	-0.01081	0.00658	-0.15329
FANCA	0.0383047	0.042368893	-0.27257	0.059241	-0.20069	0.169713
FANCB	-0.021835152	0.044746305	-0.02686	0.081246	-0.25937	-0.05706
FANCC	-0.057977833	0.062030579	0.092301	0.010246	0.039428	-0.07448
FANCD2	-0.023143018	-0.075264113	-0.04152	0.102094	-0.29334	-0.21664
FANCD2OS	-0.029294657	-0.048996615	-0.00509	-0.00117	-0.05698	-0.06202
FANCE	-0.008211918	0.006488297	0.102742	0.030598	-0.17772	-0.08048
FANCF	0.014891915	0.069885777	0.158686	-0.02338	0.069584	-0.15391
FANCG	0.004062025	0.060038883	0.100205	0.094727	-0.3021	-0.22499
FANCI	-0.008287647	0.083880478	-0.00556	0.165414	-0.41558	-0.15824
FANCL	0.011844769	0.037448649	-0.09879	0.053033	-0.15372	-0.05267
FANCM	0.0068682	0.062738222	0.022022	0.123399	-0.23068	-0.10027
FANK1	-0.025015532	-0.02687999	0.033854	-0.00492	0.037388	-0.09875

FAP	-0.033460926	-0.048252392	-0.0806	0.01216	-0.02238	-0.06345
FAR1	0.049598382	0.08162714	-0.42111	-0.03312	0.135497	0.326835
FAR2	-0.004629467	-0.109132586	-0.20459	-0.0561	0.154166	0.440525
FARP1	-0.198910126	0.042567911	-0.06613	0.048212	0.054206	-0.03403
FARP2	-0.016289876	-0.087832933	-0.11138	-0.01777	0.054643	-0.2866
FARS2	0.0192988	0.000332254	0.128387	0.047944	-0.07001	0.00453
FARSA	0.011420033	0.055726718	0.027732	0.08044	-0.26585	0.103309
FARSB	-0.010383146	0.004306109	0.109408	0.017308	-0.04792	-0.0458
FAS	0.01627337	-0.014195038	-0.01745	0.049228	-0.1238	0.045084
FAS-AS1	-0.038215185	0.011083607	-0.03527	0.025625	-0.04407	0.100197
FASLG	-0.00736981	0.196441724	0.038586	0.004535	-0.04364	-0.10455
FASN	-0.009615784	-0.052917705	0.075475	0.080504	-0.23355	-0.1691
FASTK	0.063588066	-0.046512297	0.253802	-0.01624	-0.10571	-0.16445
FASTKD1	0.027711988	0.008757783	0.127035	0.055778	-0.14636	0.101458
FASTKD2	-0.028108386	0.013447329	0.133056	0.007761	0.056864	-0.10897
FASTKD3	0.029676303	0.053127876	0.105607	0.054086	-0.14927	0.165883
FASTKD5	0.051487514	-0.026581024	0.044516	0.017741	-0.07299	-0.12337
FAT1	-0.022284743	-0.013163006	-0.04627	0.000692	0.006898	0.024815
FAT2	-0.03980731	-0.064500964	0.039713	-0.00067	0.023032	-0.00618
FAT3	-0.056530969	-0.054245068	0.035776	-0.02551	0.008316	-0.01974
FAT4	-0.018638349	0.281042906	-0.05361	0.008455	0.00492	-0.11395
FATE1	-0.029828232	-0.04276132	-0.11995	0.028377	-0.09549	-0.02184
FAU	0.090290234	0.069365043	0.066337	0.111853	-0.16393	0.017701
FAXC	-0.074500436	-0.024444639	-0.01459	-0.0087	0.012973	-0.09321
FAXDC2	-0.013392596	-0.038175845	-0.02992	-0.2663	0.738359	0.112029
FBF1	-0.024216596	-0.037381712	0.155142	0.038335	-0.13394	-0.19894
FBL	0.035559544	0.077469987	0.262679	0.042366	-0.09615	-0.03847
FBLIM1	-0.006602612	-0.066159034	-0.02906	0.019956	-0.02742	0.001277
FBLL1	-0.025820751	-0.013264682	-0.11222	0.024361	-0.10479	-0.02759
FBLN1	-0.019032555	-0.069236278	-0.01063	0.032742	-0.05997	-0.01219
FBLN2	-0.012803256	-0.077433974	0.019049	0.004659	-0.00494	-0.14732
FBLN5	-0.002603975	-0.027728139	0.460126	-0.02542	0.088052	-0.26442
FBLN7	-0.00973943	-0.045550846	-0.02795	-0.02079	0.051711	-0.11831
FBN1	0.018175189	-0.011391777	0.038999	0.018754	0.031334	-0.12456
FBN2	-0.031635419	0.048357912	-0.22825	-0.03964	0.029321	0.354276
FBN3	-0.034910125	-0.0384678	-0.06179	0.019961	-0.05895	-0.08926
FBP1	0.008647892	-0.051855781	-0.22915	-0.09442	0.271928	0.414317
FBP2	-0.037533961	-0.064824692	-0.0253	-0.00264	0.009362	0.139349
FBRS	0.011859842	-0.131701443	0.042839	0.031828	-0.12992	-0.11262
FBRSL1	-0.000263223	-0.136227473	-0.06404	-0.0378	-0.0416	-0.09558
FBXL12	0.022182885	-0.050657955	0.155411	0.032253	-0.10333	########
FBXL13	-0.021902716	-0.054177515	-0.16686	-0.00727	0.021296	-0.17025
FBXL14	-0.043558323	-0.0538268	0.12743	0.001524	0.063758	0.10357
FBXL15	0.021949752	0.016013699	0.044626	-0.03927	0.137736	0.215955
FBXL16	-0.004201248	-0.140196831	0.338814	-0.07357	0.207714	-0.01576
FBXL17	-0.047878593	0.101417348	0.045769	-0.00778	0.107976	-0.14435
FBXL18	-0.003319688	0.00497083	-0.09675	0.052726	-0.20616	0.084569
FBXL19	-0.001526899	-0.08055411	0.038707	0.034907	-0.1259	0.144551
FBXL19-A!	-0.029721873	-0.040797197	-0.02242	-0.00164	0.019928	0.184498
FBXL2	-0.031271385	-0.066373606	0.124198	0.02239	-0.04914	-0.05787
FBXL20	-0.003751959	0.025098491	-0.13636	0.044487	-0.01206	0.036817

FBXL21	-0.009182521	-0.057873189	-0.06369	0.075573	0.023777	-0.06146
FBXL22	-0.045644096	-0.05885373	0.125454	-0.01983	0.052881	-0.00728
FBXL3	0.050865538	0.052988196	-0.04939	0.00243	0.026446	-0.29398
FBXL4	-0.013500702	0.032157675	0.034876	-0.00684	0.065874	0.090854
FBXL5	0.044608543	-0.028619665	-0.32464	-0.05443	0.1762	0.433851
FBXL6	-0.023491067	-0.000467775	-0.13348	0.084897	-0.22783	0.054577
FBXL7	-0.018306645	-0.009191345	-0.0063	0.159267	-0.38927	-0.02
FBXL8	-0.013762835	0.020245016	0.00513	0.020146	-0.03272	-0.00047
FBXO10	-0.041996658	-0.128728462	-0.00852	0.018181	-0.02399	0.050696
FBXO11	0.067678239	0.052668484	-0.12907	-0.04704	0.051772	-0.16226
FBXO15	-0.025101523	-0.102891986	0.106579	-0.02237	0.04897	-0.18619
FBXO16	-0.022672619	-0.083128458	-0.00841	-0.02205	0.08413	-0.14345
FBXO17	-0.027487842	-0.010462787	-0.03372	0.016523	-0.03259	-0.06721
FBXO18	0.02205787	-0.087120457	-0.03315	-0.08408	0.195411	-0.13398
FBXO2	-0.026385574	0.032114159	0.16938	0.017343	-0.04304	-0.06228
FBXO21	0.023826724	0.083730945	0.214228	0.018554	-0.0288	-0.08046
FBXO22	0.01769961	0.001935491	-0.06861	0.135026	-0.30859	0.18621
FBXO24	-0.035528761	-0.035839375	-0.01066	0.028932	-0.04126	0.125603
FBXO25	0.029220661	0.056329248	0.081044	-0.13486	0.333963	-0.04113
FBXO27	-0.032451273	-0.028152926	-0.10191	0.00295	-0.00095	-0.06136
FBXO28	0.088269054	0.047907718	-0.04709	0.053623	-0.16715	0.037956
FBXO3	0.027054662	0.102289722	0.165744	-0.00657	0.10701	-0.11576
FBXO30	0.003179818	0.037350909	-0.12959	-0.00827	0.014166	0.054781
FBXO31	0.012957786	0.060057862	0.239679	0.015284	-0.08022	-0.30867
FBXO32	-0.086194841	0.06681886	0.412488	-0.04561	0.062405	-0.18267
FBXO33	0.040969711	0.034962433	0.040566	0.022454	-0.06116	-0.0926
FBXO34	0.055098188	0.209998108	-0.027	0.026924	-0.06404	-0.04224
FBXO36	-0.051006982	0.020291567	0.087755	0.05379	-0.13746	-0.10793
FBXO38	0.057605147	0.031010643	-0.10072	0.033342	-0.06317	-0.18271
FBXO39	-0.056050733	-0.009799835	-0.05176	0.014632	-0.00088	0.159957
FBXO4	-0.0333897	0.0651019	0.126971	-0.0101	0.006086	-0.10645
FBXO40	-0.045064239	-0.038472544	-0.03464	-0.0026	0.026404	-0.08972
FBXO42	0.084152828	0.018359641	0.043046	-0.01434	-0.0068	-0.0028
FBXO43	-0.019866815	-0.033652182	0.01291	0.023283	-0.11757	-0.02317
FBXO44	-0.017590329	-0.107771751	0.002859	-0.01528	-0.05153	-0.17493
FBXO45	0.003089563	0.097045939	-0.14109	0.090327	-0.23173	0.086384
FBXO46	0.027914927	0.06062407	0.368934	-0.00969	-0.05997	-0.34618
FBXO5	0.005768059	0.116343142	0.055972	0.128687	-0.36649	-0.20429
FBXO6	0.017656702	0.177520673	-0.212	0.03013	-0.0951	0.30699
FBXO7	0.035639895	0.183690428	0.123276	0.066871	-0.09817	-0.19597
FBXO8	0.007775053	0.058844498	-0.00062	0.014617	-0.03672	-0.04901
FBXO9	0.000362349	0.03459639	0.106106	-0.04654	0.300462	-0.00158
FBXW11	0.01459951	0.048418391	-0.08977	0.022702	-0.08932	-0.10927
FBXW12	0.051927498	0.0393724	0.02346	0.02561	-0.02003	-0.17657
FBXW2	-0.094609585	0.090638305	0.119932	-0.02508	-0.01954	-0.00229
FBXW4	0.033851883	0.029701038	0.323142	0.005319	-0.01887	-0.27939
FBXW4P1	-0.002642402	0.088215621	0.098118	-0.01826	0.082781	-0.09093
FBXW5	0.035803007	0.067817427	0.080566	-0.03539	0.076293	-0.00912
FBXW7	0.019680738	0.09996792	-0.03944	-0.00098	0.000954	0.057769
FBXW8	-0.027300843	0.100848317	0.07896	0.008993	0.007165	-0.13057
FBXW9	-0.010546148	-0.071890644	-0.07651	0.01854	-0.08055	0.069922

FCAMR	-0.061257822	-0.041131475	-0.05715	-0.03902	0.113042	0.004656
FCAR	-0.011588161	-0.077039212	-0.2748	-0.04193	0.139063	0.461071
FCER1A	0.003612034	-0.090640152	-0.22235	-0.08625	0.25774	0.188862
FCER1G	0.019591845	0.126218283	-0.43055	-0.08646	0.272732	0.406755
FCER2	-0.017760316	-0.074884389	-0.14697	-0.04576	0.110937	-0.12379
FCF1	0.022975971	-0.019465343	0.113734	-0.05188	-0.01973	-0.05394
FCGBP	0.001193899	-0.052284345	0.625799	-0.04375	0.094345	-0.20842
FCGR1A	-0.042921049	-0.033382126	-0.11702	-0.02803	0.098449	0.387811
FCGR1B	-0.000638598	-0.088593817	-0.30812	-0.08832	0.254919	0.60607
FCGR2A	0.003381551	-0.046939367	-0.43096	-0.09345	0.261514	0.388254
FCGR2B	-0.000527656	-0.087217754	-0.28651	-0.07998	0.210934	-0.00168
FCGR2C	0.007004778	-0.061449113	-0.42277	-0.07217	0.192469	0.273695
FCGR3B	0.010578421	0.13975068	-0.21892	-0.05646	0.149431	0.121036
FCGRT	0.017042251	-0.187182157	-0.29851	-0.08382	0.239965	0.396717
FCHO1	0.010848434	-0.143092729	-0.09669	-0.02634	0.031209	-0.00527
FCHO2	-0.008555959	0.040697985	-0.33657	-0.06308	0.189106	0.216561
FCHSD1	0.0012207	0.017368383	0.105398	-0.01735	0.063332	-0.15871
FCHSD2	0.017528863	-0.117673563	-0.34826	-0.03051	0.085786	0.000109
FCN1	0.003761633	-0.111396451	-0.25797	-0.10507	0.315033	0.562504
FCN2	-0.028556663	-0.059484204	-0.05871	-0.03082	0.04274	0.191278
FCN3	-0.042013171	-0.060833789	0.044631	0.019415	-0.03861	0.085812
FCRL1	-0.005161209	-0.064090513	-0.08894	-0.06465	0.178643	-0.13133
FCRL2	0.002669416	-0.069460849	-0.13054	-0.02855	0.107856	-0.1495
FCRL3	-0.000237027	0.184166096	0.141433	-0.08149	0.226222	-0.23872
FCRL4	-0.020890186	-0.016132039	0.092574	0.054465	-0.11697	-0.05961
FCRL5	0.00781305	-0.06707742	-0.18846	-0.05278	0.142489	-0.13088
FCRLA	-0.005931174	-0.061365376	-0.15129	-0.08229	0.238708	-0.21211
FCRLB	-0.009440147	0.201839515	0.091597	0.009423	-0.00617	-0.2048
FDCSP	-0.060216737	-0.067957672	0.024645	0.004446	0.016747	-0.03995
FDFT1	0.018821067	0.096340408	-0.01659	0.09551	-0.23879	-0.27392
FDPS	0.056435723	0.135850437	-0.01708	0.128795	-0.36251	-0.03566
FDPSP2	-0.012508422	-0.044568209	0.092885	0.000772	0.017568	-0.10054
FDX1	0.026023981	0.056100988	0.129497	0.038196	-0.10798	0.129184
FDX1L	-0.00771676	-0.046731727	0.034369	0.045404	-0.17993	-0.07567
FDXACB1	-0.005453443	-0.026881397	0.141863	0.077896	-0.17532	-0.07268
FDXR	-0.007105134	0.01370432	-0.02784	0.144173	-0.40716	0.095819
FECH	0.014252093	0.050169707	-0.05672	0.012776	-0.00469	-0.02575
FEM1A	0.022528354	0.01380245	-0.06863	0.019265	-0.12687	-0.02973
FEM1B	0.019058586	0.013738888	-0.09616	0.000408	-0.04531	0.373113
FEM1C	0.015729287	0.051953381	-0.16429	0.026472	-0.09048	0.115968
FEN1	0.015578336	0.141492557	-0.00535	0.170593	-0.50932	-0.22681
FENDRR	-0.046963045	-0.126735938	-0.06116	0.020088	-0.06877	0.021065
FER	0.049285816	0.013200452	-0.04177	0.046005	-0.11024	0.158833
FER1L4	-0.051005626	-0.071451847	0.007647	0.017387	-0.07061	0.143899
FER1L5	-0.034621379	-0.063052626	0.069947	0.017443	-0.03333	-0.01051
FER1L6-AS	-0.044067577	-0.056267578	-0.13186	-0.01071	0.066715	-0.05964
FERD3L	-0.043239497	-0.057185028	0.010464	0.008872	-0.01551	0.031285
FERMT1	-0.046246376	-0.07808541	0.056963	0.009508	-0.02767	-0.05626
FERMT2	-0.027792662	0.10714273	0.009785	-0.00554	0.030956	-0.06649
FERMT3	0.073193531	0.046066924	-0.21661	-0.02386	0.091214	0.378016
FES	0.005537805	0.014126612	-0.41266	-0.04129	0.121984	0.329726

FETUB	-0.051902094	-0.025577224	-0.04261	-0.02494	0.069542	0.072621
FEV	-0.029901565	-0.041039498	-0.01823	0.010471	-0.04056	-0.02175
FEZ1	-0.005212353	0.280625321	0.038273	-0.02814	0.055009	-0.11873
FEZ2	0.043924862	0.038443154	-0.39202	-0.06145	0.18605	0.329107
FEZF2	-0.0550105	-0.029500653	-0.01238	-0.04548	0.143212	0.115399
FFAR1	-0.061493886	-0.049538717	-0.07965	0.009168	-0.01149	-0.06341
FFAR2	-0.005928918	-0.079984249	-0.23365	-0.01532	0.035743	0.015229
FFAR4	-0.091220341	-0.016686699	-0.05662	-0.00957	0.055666	0.277764
FGA	-0.032263236	0.012417327	-0.0714	0.007449	0.022833	0.072213
FGB	-0.060777485	-0.049518091	-0.06734	0.010209	-0.01034	-0.00399
FGD1	-0.028973366	-0.024310276	-0.00011	-0.02682	0.051409	0.075233
FGD2	0.015937067	-0.102392092	-0.26286	-0.07527	0.307218	0.273254
FGD3	0.022927667	0.026397227	0.050658	-0.03625	0.116764	-0.06754
FGD4	0.049340942	-0.144532048	-0.26561	-0.10774	0.133667	0.528587
FGD5	-0.0373973	-0.067124498	-0.05594	-0.00032	-0.05546	-0.02026
FGD5-AS1	0.058123482	0.059576569	-0.1189	-0.00914	-0.02244	-0.07798
FGD6	-0.017239039	-0.05427142	-0.22968	-0.10395	0.209731	0.707843
FGF1	-0.04814027	-0.058135469	0.180509	0.009099	-0.00302	0.024567
FGF10	-0.044774127	-0.05128129	-0.04095	0.003165	0.013215	0.022137
FGF10-AS1	-0.035121711	-0.036812068	-0.08978	0.014973	-0.02152	-0.0368
FGF11	-0.031717389	-0.090140034	-0.05715	0.00942	-0.04325	0.102296
FGF12	-0.084007211	-0.032780811	0.001013	-0.02147	0.013965	0.081703
FGF13	-0.007947458	-0.042709424	-0.06527	0.057883	-0.15069	-0.07929
FGF13-AS1	-0.04004719	-0.06540283	0.072448	0.007188	-0.00112	-0.07454
FGF14	-0.084614172	-0.006095258	-0.12825	-0.03285	0.037221	0.019297
FGF14-AS2	-0.044085394	-0.01650243	0.134815	-0.00121	0.030456	-0.06848
FGF14-IT1	-0.025824499	-0.0645754	-0.07773	0.018863	-0.04089	0.005608
FGF16	-0.031259266	0.01745592	0.046539	-0.00138	0.02212	-0.01891
FGF17	-0.025448434	-0.121398968	-0.03184	0.029101	-0.09203	-0.02313
FGF18	-0.085023905	-0.064861865	0.025315	0.000474	-0.06339	0.064877
FGF19	-0.04351939	-0.052880307	0.014891	-0.01588	0.054533	-0.03851
FGF2	-0.028315754	-0.05709708	-0.00638	-0.0289	0.011049	-0.05742
FGF20	-0.033197855	0.019274317	-0.062	-0.02376	0.095955	-0.12359
FGF21	-0.03499157	-0.048893624	0.035634	0.007664	-0.02478	0.013041
FGF22	-0.05597688	-0.048070549	0.143055	0.002394	-0.03152	0.094094
FGF23	-0.03348754	-0.009588749	-0.00177	-0.00314	0.021211	0.034248
FGF3	-0.031434821	-0.058092268	0.011734	0.019702	-0.02425	0.108051
FGF4	-0.049420581	-0.098194134	-0.01382	0.051313	-0.13165	0.074787
FGF5	-0.014459633	-0.060237732	0.01242	0.012602	-0.0483	-0.01664
FGF6	-0.027327191	-0.025740643	0.022264	0.000953	-0.06061	0.03034
FGF7	-0.022525221	-0.200779889	0.01248	0.059156	0.103309	-0.04886
FGF8	-0.026443129	-0.024460328	-0.06618	0.009373	-0.00246	-0.01562
FGF9	-0.01304824	-0.108864436	0.267805	0.002112	0.015618	-0.10944
FGFBP1	-0.030759535	0.008468613	0.082122	0.009267	-0.05638	-0.15929
FGFBP2	0.005821936	0.279115485	0.372911	-0.07296	0.189373	-0.19132
FGFBP3	-0.027983394	0.108851014	0.12309	-0.02977	0.113458	-0.07552
FGFR1	-0.050221035	-0.036402522	0.120401	-0.0079	-0.09723	-0.09026
FGFR1OP	0.059726305	-0.014573186	-0.22298	-0.00465	-0.29072	-0.14434
FGFR1OP2	0.027264702	0.243802496	-0.2317	0.039781	0.062696	0.036401
FGFR2	-0.088293603	-0.146487278	-0.03075	0.002091	0.060155	-0.06904
FGFR3	-0.053224352	-0.095238292	-0.01943	0.012849	-0.00784	0.066578

FGFR4	-0.080807524	-0.110361796	-0.01673	0.024141	-0.13231	0.060799
FGFRL1	-0.026120843	-0.084010981	-0.0383	-0.03205	0.073204	0.048491
FGG	-0.033469236	-0.066724256	-0.08446	0.036718	-0.08385	-0.01412
FGGY	-0.023214351	-0.040379064	0.04796	0.07876	-0.25603	-0.08339
FGL1	-0.023351711	-0.100881524	0.041079	0.000224	-0.12246	-0.12525
FGL2	0.016571047	-0.018767707	-0.45875	-0.10031	0.287302	0.402042
FGR	0.026667102	0.113633015	-0.35607	-0.06619	0.186539	0.366166
FH	0.046878039	0.013579414	-0.02279	0.102495	-0.22939	0.108474
FHAD1	-0.033424077	-0.058588713	0.036451	-0.03283	0.101687	-0.08171
FHDC1	-0.020978718	-0.036532431	-0.14578	-0.00011	-0.06399	-0.0859
FHIT	-0.005321394	-0.082093205	0.21737	0.00071	0.004281	-0.16001
FHL1	-0.030739031	-0.032671701	0.119716	-0.15556	0.450432	-0.20753
FHL2	-0.014632183	-0.04935279	0.138977	0.02925	-0.07188	-0.0775
FHL3	-0.001029703	0.035717658	-0.13981	-0.02748	0.0111	0.083948
FHL5	-0.064675738	-0.119588875	0.066044	-0.03089	0.055376	-0.04817
FHOD1	0.041380301	0.096444619	-0.23848	0.039398	-0.13139	0.227598
FHOD3	-0.036342939	-0.033286826	-0.03889	0.004467	0.010382	0.034022
FIBCD1	-0.033886952	-0.097628301	-0.01836	-0.01728	0.014006	-0.00953
FIBIN	-0.033755853	-0.060431245	0.016171	0.01318	-0.02385	-0.11911
FIBP	0.040276493	0.050902789	-0.03572	0.057624	-0.17238	0.231693
FICD	0.005787539	0.075026787	-0.03229	0.029698	-0.11671	-0.12469
FIG4	0.03211381	0.013574356	-0.33671	-0.03882	0.110892	0.277459
FIGLA	-0.034381132	-0.03649041	-0.04696	0.014745	-0.04003	-0.03696
FIGN	-0.045983706	-0.065379132	0.111973	-0.03089	-0.00377	-0.11436
FIGNL1	-0.003706454	0.047310292	0.103071	0.114153	-0.35227	-0.19997
FILIP1	-0.050518874	-0.044409309	-0.05389	0.080625	-0.10777	-0.01924
FILIP1L	0.011122996	-0.028056756	-0.058	-0.00548	7.79E-05	0.046061
FIP1L1	0.01957094	0.086075389	0.039623	0.079322	-0.20712	-0.17234
FIRRE	-0.034030903	-0.051688107	0.020643	0.011042	-0.01243	-0.17408
FIS1	0.059012831	0.0647677	0.134031	0.066452	-0.17353	0.14934
FITM1	-0.004435927	-0.009014136	0.184153	-0.01077	0.03962	-0.10402
FITM2	-0.040069655	-0.055514233	0.015249	0.042213	-0.22858	0.003072
FIZ1	-0.003528979	-0.05713915	0.092143	-0.01895	-0.02958	-0.09689
FJX1	-0.030765296	-0.062162353	-0.06685	0.056721	-0.15239	0.16751
FKBP10	-0.033241908	-0.014895075	0.048833	-0.00443	-0.01266	-0.16031
FKBP11	0.002852048	0.172724543	0.471568	0.036923	-0.13224	-0.36709
FKBP14	-0.016485868	0.037440089	-0.08107	0.002808	0.036307	-0.0808
FKBP15	-0.070202906	-0.080380663	-0.21643	-0.09182	0.193487	0.712655
FKBP1B	0.003645999	-0.0183334	-0.06719	-0.09722	0.259888	0.192084
FKBP2	0.039632924	0.049414297	0.012858	0.033144	-0.11645	0.200862
FKBP3	0.040483724	0.037987286	0.138154	0.06178	-0.16967	-0.04054
FKBP4	-0.015675564	-0.005163845	0.084431	0.06213	-0.17573	0.05652
FKBP5	0.031181489	-0.029494274	0.235071	0.01245	-0.01335	0.054678
FKBP6	-0.026863896	-0.075688401	0.014954	0.011987	-0.03878	0.111929
FKBP7	-0.051262372	-0.046040236	-0.00273	0.086217	-0.12702	-0.10417
FKBP8	0.002769998	-0.095015737	0.04432	0.026042	-0.09667	0.049682
FKBP9	-0.016766295	-0.082372855	-0.22544	-0.04775	0.128382	0.414629
FKBPL	-0.013692558	-0.042039094	-0.10232	0.067917	-0.27652	0.00833
FKRP	-0.000976601	-0.0135636	-0.00309	0.035354	-0.08862	0.067836
FKSG29	-0.031877434	-0.029785048	-0.01021	0.005867	-0.01229	-0.02715
FKSG49	0.0290021	0.016581184	0.082848	0.001301	-0.01974	-0.19396

FKTN	0.001919917	0.041161146	0.114479	0.048174	-0.10023	0.063479
FLAD1	0.014397656	0.035095145	0.043544	0.059581	-0.16943	0.011824
FLCN	0.002073192	0.005713681	-0.1926	0.066541	-0.15057	0.213258
FLG	-0.059721224	-0.025326289	-0.06371	-0.0031	-0.00443	0.002874
FLG-AS1	-0.046531005	-0.067341955	0.023416	0.011814	-0.03138	-0.07479
FLG2	-0.060110849	0.042559565	0.035344	-0.01866	0.054678	0.087602
FLI1	0.011039817	0.055770646	-0.04111	-0.03746	0.089703	-0.0007
FLII	0.021514526	-0.060615374	-0.2406	0.008395	0.029201	0.266741
FLJ10038	-0.008671169	0.011881987	0.064939	-0.03678	0.103143	-0.16307
FLJ11292	-0.006103834	0.045470061	0.100661	0.05837	-0.0466	-0.14838
FLJ11710	-0.029187748	-0.080496144	0.108738	-0.00834	-0.06532	-0.10537
FLJ12120	-0.004067252	0.200742446	0.013578	-0.01054	0.032327	-0.1531
FLJ12825	-0.036065855	-0.038259631	-0.07178	0.006747	-0.00949	-0.04923
FLJ13224	-0.023561466	-0.011694146	0.017248	0.00479	0.00607	0.020475
FLJ13744	-0.042511909	-0.070833661	0.058909	0.012129	-0.00355	0.161626
FLJ13773	-0.016683192	-0.014278392	0.106155	-0.0023	0.034384	0.040182
FLJ16734	-0.017430324	0.016878204	0.169852	0.014264	-0.02645	-0.10574
FLJ16779	-0.032169111	-0.095549699	0.140169	-0.00569	0.03834	0.021351
FLJ20021	-0.003715203	-0.069957691	-0.05839	0.009551	-0.10861	0.010593
FLJ20518	-0.054574152	-0.054861647	-0.00434	0.065214	-0.10471	5.23E-05
FLJ20712	-0.034687799	-0.057207385	-0.01438	0.004558	-0.01303	0.04219
FLJ21369	-0.038444912	-0.058075129	0.086238	-0.00013	-0.00614	-0.03714
FLJ21408	-0.047836141	0.004081913	0.077135	0.013455	-0.0713	-0.12525
FLJ22184	-0.023693004	-0.051299689	-0.04192	0.038516	-0.11664	-0.06588
FLJ22763	-0.044430778	-0.06516137	0.163975	-0.00307	0.015302	0.017142
FLJ25758	-0.022679667	-0.063352559	-0.08382	0.017654	-0.07898	0.157352
FLJ25917	-0.019353698	-0.015125531	-0.03313	-0.00099	-0.00527	0.010294
FLJ26850	-0.037720123	0.003830898	-0.02649	0.007538	-0.00848	0.051649
FLJ27354	-0.019661865	-0.060349656	0.271089	-0.0229	0.095448	0.006423
FLJ30064	-0.024262343	-0.072236104	-0.04621	-0.00983	-0.05058	-0.02579
FLJ30403	-0.035479963	-0.064537897	-0.02828	0.015987	-0.02712	-0.08162
FLJ30679	-0.037255726	-0.089753523	-0.00857	0.012501	-0.03288	-0.07922
FLJ31104	-0.037274252	-0.079263302	0.07798	-0.00616	0.029012	-0.08675
FLJ31306	0.003027798	0.039382272	0.066114	-0.04281	0.130828	-0.21554
FLJ31356	-0.044027547	-0.008622613	0.004778	0.020708	-0.05365	0.028136
FLJ31713	-0.033530909	-0.048695668	-0.03326	0.023278	-0.04913	-0.08912
FLJ31715	-0.030253035	-0.026663398	0.104165	-0.0037	0.033925	0.049798
FLJ31945	-0.041216638	-0.059434678	-0.00572	-0.01008	0.056797	0.176835
FLJ32154	-0.037636248	-0.041803875	0.043584	0.010673	0.001132	0.056064
FLJ32255	0.018036657	0.095027415	0.301267	-0.01942	0.066208	-0.27314
FLJ32790	-0.042060644	-0.028636654	0.035727	0.029758	-0.06571	0.057848
FLJ32955	-0.051524425	-0.061954018	0.060453	0.023137	-0.05042	-0.04365
FLJ33360	-0.039640216	-0.028061685	0.013195	0.026222	-0.05477	-0.17658
FLJ33534	-0.027934196	-0.012031015	0.088161	0.011587	-0.04208	-0.16963
FLJ33544	-0.039345842	0.002696875	-0.10434	0.019818	-0.0244	0.088948
FLJ34503	-0.042058595	0.004401194	-0.05454	0.018759	-0.04096	-0.02597
FLJ34521	-0.037181543	0.008118261	-0.11148	-0.0004	0.025489	0.107811
FLJ35700	-0.038552565	-0.04726429	0.292804	0.013204	-0.01574	-0.06899
FLJ35816	-0.058023104	-0.055137137	-0.03607	-0.01313	0.047061	0.004262
FLJ35934	-0.025251636	0.037707721	0.136642	-0.02174	0.091656	-0.06747
FLJ36840	-0.021744482	-0.049494151	0.016312	0.017925	-0.06921	-0.13205

FLJ36848	-0.01080887	-0.006059809	0.035204	0.016804	-0.06123	0.10912
FLJ37035	-0.069916043	-0.04222089	0.02437	-0.0163	-0.03746	0.037776
FLJ37201	-0.024495462	-0.032973163	0.04612	0.006784	-0.03063	-0.13034
FLJ37453	-0.022434304	-0.027244571	0.053727	-0.0711	0.216386	0.107125
FLJ37786	-0.02048484	-0.022708753	-0.00237	-0.01332	0.002742	-0.03372
FLJ38379	-0.030218882	-0.128494743	0.079386	-0.03112	0.094475	-0.37251
FLJ38576	-0.062218537	-0.055853009	-0.01676	-0.01892	0.095493	-0.05203
FLJ38668	-0.023802117	-0.057566018	-0.04106	0.01098	-0.02994	0.047304
FLJ38717	0.00927519	-0.087306916	0.036824	-0.0393	0.11394	-0.20558
FLJ38773	-0.030937432	-0.044367949	-0.01443	-0.01194	0.050575	-0.01812
FLJ39080	-0.039518412	-0.057916931	0.005771	0.011787	-0.01835	-0.01921
FLJ40288	-0.027891036	-0.063860733	0.017037	-0.00742	-0.06965	-0.10293
FLJ41170	-0.041188897	-0.07715831	0.099546	0.020006	-0.03447	0.034863
FLJ41455	-0.014392754	-0.066827923	-0.13948	-0.00544	0.043107	0.001678
FLJ42627	0.002176027	-0.040439987	-0.05295	-0.00042	-0.04195	-0.00022
FLJ44087	-0.031513358	-0.041936885	-0.08933	0.012829	-0.01388	-0.02069
FLJ45482	-0.009426172	0.009725889	0.048225	0.05602	-0.16366	-0.17042
FLJ45513	-0.005725843	0.050219387	0.023544	-0.05252	0.134938	0.030631
FLJ45825	-0.038617657	-0.037337425	0.090891	0.007102	-0.00945	0.033132
FLJ46026	-0.027720576	-0.071211251	0.141799	-0.00768	-0.02381	-0.02459
FLJ46875	-0.004910267	-0.04447519	0.021009	-0.01578	0.00754	0.046565
FLJ90680	-0.035326823	-0.071161816	0.06507	0.009768	-0.00926	0.011626
FLNA	0.026652807	0.150740191	0.128073	-0.01071	0.024371	0.1594
FLNB	0.012248982	-0.135596044	0.068227	-0.00357	-0.01226	-0.31883
FLNC	-0.018777795	-0.079720969	-0.02966	-0.01178	0.015578	-0.04976
FLOT1	0.023943559	0.017292056	-0.1918	0.086737	-0.228	-0.04505
FLOT2	0.018860222	-0.043356406	-0.07431	0.005925	-0.00779	-0.11068
FLRT1	-0.031060974	-0.00796482	-0.09805	0.033729	-0.09387	0.112525
FLRT3	-0.02256861	-0.018338981	0.058855	0.1581	-0.28433	-0.04426
FLT1	0.017743148	-0.028329627	0.00764	0.023812	0.004385	-0.03276
FLT3	0.001104678	-0.127635808	-0.18517	-0.05778	0.177123	0.080075
FLT3LG	0.005630619	-0.031154411	0.444217	-0.02414	0.077753	-0.15995
FLT4	-0.007836624	-0.15721374	0.218552	0.011738	-0.03546	-0.13301
FLVCR1	0.008144342	-0.060002821	-0.16574	0.070628	-0.17921	-0.1788
FLVCR1-AS	0.005518224	-0.02647941	0.047127	0.015103	-0.05983	-0.03256
FLVCR2	-0.020735166	-0.061758974	-0.19714	-0.07896	0.121481	0.48993
FLYWCH1	-0.019294219	-0.070626862	0.017975	-0.04612	-0.04433	-0.1221
FLYWCH2	0.005552137	-0.066227362	0.039944	0.082931	-0.29746	-0.00824
FMN1	-0.001877041	-0.103603075	-0.00246	-0.0089	0.112934	0.116561
FMN2	-0.045885757	-0.005681095	-0.0072	0.046094	-0.07137	-0.03102
FMNL1	0.027662212	0.026338192	-0.13519	-0.04038	0.127854	0.118684
FMNL2	0.010359499	-0.080996889	-0.16867	0.021858	0.05474	0.165805
FMNL3	-8.87E-06	0.045716372	0.026651	0.00436	-0.01896	-0.25334
FMO1	-0.025936922	-0.107949303	0.016347	0.016376	-0.04132	-0.19421
FMO2	-0.034009676	-0.006882991	0.100902	-0.00058	-0.02492	-0.07339
FMO3	-0.054380342	-0.045129308	0.033581	0.023719	-0.05478	0.00444
FMO4	-0.015278428	0.060132613	0.066711	-0.02295	0.029008	0.064
FMO5	-0.064314253	-0.010316972	-0.14048	-0.15763	0.371364	0.351366
FMO6P	-0.026405599	-0.010917802	-0.04611	0.007476	-0.01073	0.0184
FMO9P	-0.056358184	-0.035914065	-0.03327	-0.02885	0.070294	0.004434
FMOD	-0.024171448	-0.046222101	0.059749	-0.00047	-0.06218	-0.0362

FMR1	0.052875855	0.148529889	-0.01544	0.006481	-0.00458	-0.14874
FMR1NB	-0.121421863	-0.068972481	-0.01531	-0.00512	0.04545	0.0795
FN1	0.030341389	-0.027138455	-0.05061	0.105989	-0.21911	0.076395
FN3K	-0.036445219	-0.056279846	0.010361	0.016946	-0.01378	-0.03336
FN3KRP	0.037841154	0.079468935	0.080596	0.023623	-0.0795	-0.08586
FNBP1	0.053351628	0.040359651	0.053602	-0.00575	-0.00786	-0.32623
FNBP1L	-0.018742638	-0.123679313	-0.08399	-0.05239	0.112421	-0.19554
FNBP4	-0.027547278	0.013381847	0.152679	-0.03704	0.055583	-0.13688
FNDC1	-0.036657647	-0.099667896	0.025151	-0.02043	0.072207	-0.09926
FNDC3A	-0.036005822	-0.006207945	-0.02069	-0.06053	-0.03661	0.124467
FNDC3B	-0.020092312	0.053226732	-0.13594	0.011489	-0.01779	-0.03365
FNDC4	-0.013770847	-0.064064606	0.025262	-0.01125	-0.04837	-0.08958
FNDC5	-0.055721252	-0.029917099	0.02047	-0.03158	-0.08868	-0.02018
FNDC7	-0.025503969	-0.026963142	0.028041	0.010636	-0.0506	-0.07256
FNDC8	-0.035819751	-0.015416075	-0.01269	-0.0285	0.112619	0.021497
FNDC9	-0.031374333	-0.008557714	-0.03201	0.009627	-0.01392	-0.00038
FNIP1	-0.023426813	-0.065671478	-0.23147	0.009352	-0.00375	0.095424
FNIP2	0.009386812	0.083098281	0.015383	-0.02406	0.095665	0.258931
FNTA	0.060768805	0.119390606	0.191266	0.006803	-0.01702	-0.18875
FOCAD	0.004342831	0.010303963	0.064763	0.065899	-0.17152	-0.05355
FOLH1	-0.027412473	-0.061812962	0.160181	0.027861	-0.07711	-0.11303
FOLH1B	-0.05133587	-0.034501112	-0.08503	0.027934	-0.06123	0.0096
FOLR1	-0.029378942	-0.067647377	0.005225	0.026226	-0.0676	0.011056
FOLR2	0.000441756	-0.130824132	-0.17124	-0.02606	0.04326	0.424976
FOLR3	-0.001959756	-0.082031821	-0.25054	-0.09472	0.271764	0.599276
FOPNL	0.027929674	0.118913633	0.031077	0.049788	-0.13184	0.055987
FOS	0.007853002	-0.123348566	-0.19051	-0.05441	0.145629	0.304162
FOSB	0.00110918	-0.094982488	-0.04167	0.003976	-0.01485	0.060737
FOSL1	-0.011145189	-0.014876287	-0.00596	0.036535	-0.11525	0.027275
FOSL2	-0.001876579	0.066283919	-0.16616	0.0068	0.012279	0.160402
FOXA1	-0.066361178	-0.043291653	0.040199	-0.02471	0.077963	0.018069
FOXA2	-0.004004783	-0.053655832	0.178741	0.03068	-0.01698	0.027864
FOXA3	-0.040629617	-0.018642997	-0.03902	0.026403	-0.05131	0.037031
FOXB1	-0.039875203	-0.060889174	0.048255	0.017108	-0.02284	0.099567
FOXC1	-0.0256903	0.032109755	-0.00231	-0.0057	0.010741	0.046948
FOXC2	-0.046551649	-0.075939307	-0.01382	-0.0211	0.098296	0.086998
FOXD1	-0.019707353	0.002048105	-0.03576	0.066018	-0.15061	0.073621
FOXD2	-0.032097828	0.004793553	0.015402	0.049441	-0.12023	0.050476
FOXD2-AS	-0.03377876	0.001649421	-0.01142	0.161527	-0.43764	0.052084
FOXD3	-0.052684657	-0.073018716	-0.06502	0.020642	-0.05135	0.24851
FOXD3-AS	-0.028526789	-0.011218762	-0.01131	0.011155	-0.00652	0.019873
FOXE1	-0.063932999	-0.027735926	-0.03148	0.013452	-0.02306	0.07605
FOXE3	-0.038550336	-0.071171356	-0.05887	-0.02342	0.087139	0.049687
FOXF1	-0.019034642	-0.020844708	-0.0331	0.000274	-0.01722	-0.05218
FOXF2	-0.039312108	0.008720444	0.054176	0.014977	-0.01601	0.024095
FOXG1	-0.045856867	-0.065752655	-0.00313	0.002617	0.013243	0.024376
FOXH1	-0.039425123	-0.028936737	0.141991	-0.0083	0.023685	-0.10141
FOXI1	-0.034779913	-0.121200706	-0.0672	-0.02727	0.110219	0.135785
FOXJ1	-0.027630055	-0.110576815	0.097902	0.023545	-0.07955	-0.18911
FOXJ2	0.030909342	-0.032239405	0.016009	-0.024	0.050881	-0.08486
FOXJ3	0.05074083	0.102604671	0.262538	0.056467	-0.12409	-0.41221

FOXK1	-0.042236125	-0.14139315	0.089397	0.004698	-0.04221	-0.07845
FOXK2	-0.00088379	-0.012317985	0.08536	0.119385	-0.08125	-0.07036
FOXL1	-0.037153088	-0.050119826	-0.10755	0.019577	-0.02891	0.213145
FOXL2	-0.034701713	-0.016055498	-0.18436	0.013703	-0.02712	0.044381
FOXM1	-0.012973603	-0.004856369	-0.00049	0.119776	-0.33325	-0.0363
FOXN1	-0.048015594	-0.09431559	-0.02224	0.022875	-0.03365	0.090589
FOXN2	0.036603016	0.042555818	-0.28965	-0.01825	0.044229	0.033385
FOXN3	0.005297093	-0.039632571	0.076329	-0.03396	0.031155	-0.07927
FOXN3-AS1	-0.021455569	-0.010623157	-0.05628	0.009043	-0.0585	0.154553
FOXN3-AS2	-0.030194102	-0.038369975	-0.07283	0.023681	-0.05381	0.029895
FOXN4	-0.028747771	-0.052011853	-0.02937	-0.03821	0.044147	-0.05675
FOXO1	-0.014015849	-0.049050408	0.241136	-0.02148	0.08742	-0.17222
FOXO3	0.043161699	0.080275756	-0.12463	-0.04222	0.127454	0.075579
FOXO4	-0.005187068	-0.030138096	-0.04582	-0.03801	0.065788	0.10148
FOXO6	-0.027464761	-0.053547394	-0.16416	-0.00995	-0.00178	-0.01511
FOXP1	8.25E-05	-0.072518915	0.223445	-0.11148	0.247982	-0.34636
FOXP1-IT1	0.007333465	-0.185812768	-0.11238	-0.03453	0.110482	0.079956
FOXP2	-0.081353605	-0.054814789	0.011484	0.184689	-0.20996	-0.05051
FOXP3	-0.057657193	-0.112534377	0.026928	0.076157	-0.08177	-0.08068
FOXP4	-0.012442726	-0.075229228	-0.20154	0.040824	-0.13323	0.265415
FOXQ1	-0.039712886	0.007958967	-0.00895	-0.00346	-0.05209	-0.09465
FOXR1	-0.055073458	-0.074997052	-0.01209	-0.00154	0.026557	0.080082
FOXR2	-0.057898958	-0.067832661	-0.09639	-0.0369	0.111304	0.068352
FOXRED1	0.011813598	-0.019446114	0.067472	0.062799	-0.20955	0.001758
FOXRED2	0.028558878	0.01053602	-0.06773	0.020212	-0.46474	0.081772
FOXS1	-0.037282725	-0.042561117	-0.07898	0.013363	-0.01354	-0.02312
FPGS	0.012714395	-0.015667895	0.126341	0.031997	-0.1821	-0.23634
FPGT	-0.002537665	0.045119065	-0.12705	-0.02437	0.073337	0.352455
FPR1	0.00405937	-0.095196808	-0.34771	-0.09117	0.252491	0.414492
FPR2	0.001555348	-0.052096544	-0.36245	-0.05287	0.164662	0.391637
FPR3	-0.020723474	-0.040346615	-0.10615	-0.04591	0.101878	0.170817
FRA10AC1	-0.011098031	-0.011092127	0.155603	-0.002	-0.00798	-0.19967
FRAS1	-0.011544683	-0.042169121	-0.08833	0.038077	0.026299	0.046024
FRAT1	0.014568957	-0.029937197	-0.19984	-0.07013	0.209698	0.22917
FRAT2	0.02216634	-0.021462092	-0.29754	-0.06733	0.196071	0.247687
FREM1	-0.04386395	-0.090162485	0.016303	-0.01205	0.05696	-0.0425
FREM2	-0.041531873	-0.022566097	0.036547	0.126228	-0.12011	-0.0744
FREM3	-0.043439438	-0.040071359	0.11	-0.01578	0.037735	-0.0099
FRG1B	-0.013463955	-0.002541249	0.065554	-0.02206	0.007537	-0.10827
FRK	-0.032831474	-0.02547159	0.017828	0.003042	-0.01565	-0.10287
FRMD1	-0.023806627	-0.038413421	0.036898	-0.0117	-0.04515	-0.08454
FRMD3	0.007592393	0.035805057	-0.18902	-0.2895	0.469797	0.554706
FRMD4A	-0.029050937	-0.0249321	0.007262	0.018818	0.017085	-0.09434
FRMD4B	-0.03181149	0.044014474	0.035776	-0.03956	0.023103	0.087327
FRMD5	-0.038781828	-0.019868896	0.029664	0.040307	-0.15263	-0.1529
FRMD6	-0.011266464	0.146476384	-0.06386	0.069571	-0.17954	-0.07328
FRMD6-AS1	-0.031489344	0.042457021	0.119766	0.040951	-0.09837	-0.09033
FRMD7	-0.03943961	-0.038626417	-0.05583	-0.03345	0.10935	0.062162
FRMD8	-0.011475152	-0.043788551	0.038203	-0.01536	-0.06455	-0.12863
FRMD8P1	-0.02792046	-0.038161059	-0.08901	0.007741	-0.07468	-0.012
FRMPD1	-0.03183419	-0.03966851	-0.00911	-0.00163	0.032613	0.045748

FRMPD3	-0.025522676	0.073237074	0.053187	0.006621	-0.03281	-0.12271
FRMPD4	-0.047209448	-0.042524591	-0.03729	0.076562	-0.19015	0.010477
FRRS1	-0.036621694	-0.077031078	0.025667	-0.01661	0.003066	-0.09707
FRRS1L	-0.022202444	0.04901967	-0.05815	0.042927	-0.01694	-0.00353
FRS2	-0.060226775	0.013312243	-0.16868	-0.02231	0.123814	0.032743
FRS3	-0.018525095	-0.049393524	-0.16775	0.036236	-0.10049	0.091042
FRY	-0.017926889	-0.053854176	-0.2473	-0.05349	0.148486	0.170145
FRY-AS1	-0.041825906	-0.042084208	0.018212	-0.01715	0.060467	0.061542
FRYL	-0.003566813	0.156889228	0.188958	-0.00442	-0.00257	-0.295
FRZB	-0.006740256	-0.046170228	-0.06299	0.024136	0.042309	-0.0109
FSCB	-0.051588087	0.001226845	0.036347	-0.02168	0.082378	-0.06684
FSCN1	-0.013460291	-0.041493677	-0.06483	0.00147	-0.03461	0.086394
FSCN2	-0.032735451	-0.060660362	-0.13781	0.009833	-0.00757	0.126102
FSCN3	-0.029435647	-0.034137688	0.001233	-0.00544	-0.03845	-0.00803
FSD1	-0.011690927	0.12308697	0.036867	0.101196	-0.31403	-0.16752
FSD1L	-0.026295746	-0.074496211	-0.10578	-0.024	-0.04313	0.153998
FSD2	-0.03491267	-0.011924321	0.059441	0.011589	-0.02858	-0.08765
FSHB	-0.033802893	-0.035955985	0.010166	0.012866	-0.00559	0.120009
FSHR	-0.034685258	-0.067608889	0.053751	0.011739	-0.00475	-0.12003
FSIP1	-0.033066414	0.226949222	0.019308	0.017003	-0.04923	-0.02835
FSIP2	-0.068252405	-0.040005692	-0.01185	-0.02234	0.060029	-0.06753
FST	0.026570313	0.026098839	-0.03823	0.110326	-0.07367	-0.10169
FSTL1	-0.023326522	-0.04503944	-0.01335	-0.18333	0.504635	-0.07317
FSTL3	-0.0164495	-0.072572343	-0.01805	-0.01321	-0.01398	0.316627
FSTL4	-0.013121853	-0.077527656	-0.00128	0.011516	0.07152	0.088174
FSTL5	-0.026216294	-0.026671246	-0.02942	0.129502	-0.09644	0.011423
FTCD	0.001798898	-0.168437718	-0.03554	0.03494	-0.10312	-0.12181
FTCDNL1	-0.019344563	-0.125151556	-0.07133	-0.01622	0.073687	0.213544
FTH1	0.038433964	-0.120124745	-0.45683	0.013485	0.00061	0.412653
FTH1P5	0.052217794	-0.112226542	-0.44357	0.023172	-0.02372	0.33359
FTHL17	-0.050126905	0.055622408	-0.06097	-0.00686	0.027058	0.152348
FTL	0.044127632	-0.093169807	-0.29286	0.062249	-0.05262	0.428333
FTO	0.027172792	0.0236074	0.165851	0.011285	-0.0235	0.028718
FTSJ1	0.024513459	0.09674116	0.236535	0.035816	-0.1205	-0.01038
FTSJ2	0.029417834	-0.016893811	-0.11149	0.039869	-0.11525	0.256937
FTSJ3	0.038710914	0.065880843	0.102064	0.057836	-0.19278	-0.00853
FTX	0.006301123	-0.023362616	-0.0585	-0.01088	0.037919	0.089692
FUBP1	0.040031746	0.059868987	0.287863	-0.0066	-0.02961	-0.224
FUBP3	0.016379359	0.074838464	0.092099	0.022432	-0.03636	0.065762
FUCA1	0.015965595	0.003288023	-0.27659	-0.0381	0.12696	0.387719
FUCA2	0.01257558	0.010982237	-0.25128	-0.10087	0.274369	0.520852
FUK	0.023210027	-0.001606873	0.127832	-0.00984	0.024404	0.036282
FUNDC1	0.017181118	0.016349063	-0.03235	0.002454	0.026412	-0.0471
FUNDC2	0.020367637	0.044423012	0.191917	0.004058	-0.06411	0.060797
FUOM	0.010312209	-0.087880107	-0.21237	-0.02392	0.068856	0.433606
FURIN	0.03610196	0.108556006	-0.18676	0.026683	-0.10378	-0.09694
FUS	-0.018901329	-0.006158107	0.254899	0.025168	-0.03715	-0.3214
FUT1	-0.025358494	-0.010189414	-0.10494	0.014906	-0.05283	-0.04088
FUT10	0.01097757	0.060066336	0.005602	0.108194	-0.0262	-0.0641
FUT11	0.016669007	0.346046209	0.193714	-0.02128	0.047346	-0.10106
FUT2	-0.042504365	-0.066657566	0.087058	-0.0256	0.025799	-0.12071

FUT3	-0.04688346	-0.135221297	0.017035	-0.01556	0.001592	-0.03849
FUT4	0.014360383	-0.023568537	-0.17151	-0.09061	0.258125	0.525861
FUT5	-0.024237594	-0.086194265	0.042801	-0.00586	-0.08404	-0.11551
FUT6	-0.04171503	-0.035273132	0.007371	-0.00726	-0.02372	-0.12221
FUT7	-0.00762626	-0.04072384	-0.06928	-0.02951	0.065027	-0.06876
FUT8	0.00322707	-0.019531567	0.152544	0.113794	-0.33921	-0.26726
FUT8-AS1	-0.011308314	-0.077072844	0.124729	-0.0322	0.072872	-0.21338
FUT9	-0.10105952	-0.063890602	-0.00126	-0.03489	0.024655	0.098323
FUZ	-0.006382624	-0.150241773	0.027777	0.000932	-0.0726	-0.15993
FW339973	-0.082892062	-0.081171914	-0.08321	-0.02728	0.106439	0.209032
FW340027	-0.03167319	0.002284816	0.03775	0.064724	-0.16945	-0.07854
FXN	-0.003186664	0.032068316	0.209145	0.052976	-0.11983	0.117189
FXR1	0.06598102	0.066430044	0.148057	0.10134	-0.2738	-0.22044
FXR2	0.030604865	0.025783959	0.080705	0.011256	-0.10113	-0.1084
FXYD1	-0.029313204	-0.152561937	0.027361	-0.01091	0.007171	-0.08378
FXYD2	-0.02029182	-0.127468612	0.293251	0.031045	-0.09052	-0.06286
FXYD3	-0.026819988	-0.072386274	-0.01162	0.002394	-0.09239	-0.01329
FXYD4	-0.039144008	-0.085245555	-0.03451	-0.00099	0.027933	0.022911
FXYD5	0.040778974	0.01307055	0.180671	0.001061	0.007761	0.185213
FXYD6	-0.00180661	-0.069277159	-0.24851	-0.07234	0.200196	0.623853
FXYD7	-0.00888772	-0.019367818	0.384318	-0.0481	0.168404	-0.19811
FYB	-0.003273282	-0.044713905	0.113961	-0.11357	0.181707	0.271495
FYCO1	0.019290667	0.075629275	0.233325	-0.00636	-0.03188	0.013064
FYN	0.024559167	0.177192337	0.269601	-0.0252	0.077932	0.026236
FYTTD1	0.044442624	0.183075113	0.148198	0.016398	-0.03481	-0.06192
FZD1	-0.001948892	-0.073029924	-0.19503	-0.07265	0.308315	0.380205
FZD10	-0.040758254	-0.023342101	-0.0892	0.014113	-0.04062	-0.09765
FZD10-AS1	-0.028128577	-0.060372905	0.041242	0.007373	-0.04657	-0.0395
FZD2	-0.009323899	-0.111793172	-0.23893	-0.064	0.194062	0.439872
FZD3	-0.003972454	-0.083574079	0.090942	-0.02245	0.100349	-0.34513
FZD4	-0.042862419	-0.017575185	-0.01997	0.007776	-0.03072	-0.02631
FZD5	-0.014140818	-0.021225418	-0.10938	0.03215	-0.08058	0.047404
FZD6	-0.010391878	0.071510459	0.038249	-0.03644	0.100847	-0.21702
FZD7	-0.01871228	0.003745989	-0.0746	-0.01729	0.052315	-0.02384
FZD9	-0.033319028	-0.051688273	-0.05494	0.004714	-0.00727	-0.04783
FZR1	-0.038086405	-0.047130204	-0.04662	0.031686	-0.10899	0.004336
G0S2	-0.000294951	-0.044966108	-0.17787	-0.01514	0.020733	-0.00956
G2E3	0.022249171	0.078345005	0.056716	0.0953	-0.19881	0.053213
G3BP1	-0.05344573	-0.00349396	0.046275	-0.03632	-0.12152	-0.0595
G3BP2	0.066886839	0.06421246	0.196512	0.041853	-0.13933	-0.16589
G6PC	-0.07794981	-0.053747354	-0.02583	0.029075	-0.05842	0.045606
G6PC2	-0.033722897	-0.02209176	0.076581	-0.00925	-0.01671	-0.1831
G6PC3	0.020821578	-0.077198016	-0.11708	0.07349	-0.22652	0.319953
G6PD	0.019009611	0.066606776	-0.2459	0.017601	-0.05255	0.239652
GAA	0.01439006	-0.121801972	-0.27704	-0.06346	0.165531	0.456715
GAB1	-0.006900341	-0.050872925	-0.35857	-0.14011	0.23118	0.134193
GAB2	0.023760412	-0.137680287	-0.36936	-0.03166	0.078955	0.314311
GAB3	0.016994787	0.122169375	-0.06489	-0.02909	0.083729	0.045624
GAB4	-0.036778827	-0.052160856	0.01821	0.007781	-0.04537	-0.05585
GABARAP	0.036018472	-0.075972466	-0.25087	-0.01443	0.030326	0.256981
GABARAPL1	0.021270652	0.088631574	0.058438	0.012295	-0.03505	-0.07579

GABARAPL2	0.087267089	-0.006897804	-0.09233	0.025957	-0.04233	-0.17285
GABARAPL3	-0.03350609	-0.018917719	0.043355	-0.00145	0.023424	-0.07254
GABBR1	-0.000502327	-0.255788545	-0.05596	-0.06787	0.201901	0.285027
GABBR2	-0.037042711	-0.045856391	0.045916	0.034506	-0.03975	0.064701
GABPA	-0.000617899	0.069579301	-0.03929	0.037136	-0.12949	-0.08163
GABPB1	0.01244091	0.062708654	-0.07465	0.048497	-0.17237	-0.12469
GABPB1-AS	0.006557132	-0.074138464	0.182344	0.030974	-0.04541	-0.13272
GABPB2	0.019146177	0.04674184	0.309245	-0.01586	0.008339	-0.20706
GABRA1	-0.049816584	-0.066625196	0.095395	-0.00497	0.010252	-0.17763
GABRA2	-0.130251659	-0.073887711	-0.1144	0.010363	0.071152	0.102529
GABRA3	-0.040690384	0.012385418	-0.06062	-0.00527	0.033764	-0.00885
GABRA4	-0.026971408	-0.052692405	0.051455	-0.00244	-0.00342	-0.17141
GABRA5	-0.059646378	-0.102566364	0.004342	-0.01743	0.053809	-0.01259
GABRA6	-0.05612265	-0.060012901	-0.07387	-0.0074	0.013456	0.09361
GABRB1	-0.042094067	-0.047247654	0.166951	-0.00229	0.007933	-0.17551
GABRB2	-0.047168472	-0.077068643	-0.01852	-0.02897	0.087584	-0.11665
GABRB3	-0.033147255	-0.016924944	-0.03383	-0.0018	0.043943	-0.13618
GABRD	-0.046984045	-0.033045764	-0.17153	0.007062	-0.02545	-0.01589
GABRG1	-0.049604974	-0.048155561	-0.02402	-0.00084	-0.02331	-0.10258
GABRG2	-0.042180173	-0.052388225	-0.01712	0.00503	-0.0162	-0.08194
GABRG3	-0.079863931	-0.079005815	0.036869	-0.02247	0.05523	-0.0616
GABRP	-0.042874623	-0.089606784	0.087087	0.050122	-0.12206	-0.03546
GABRQ	-0.052627646	-0.05089158	-0.02114	0.008349	0.004195	-0.07043
GABRR1	-0.056014374	0.015803637	0.001749	-0.0375	0.110476	-0.03199
GABRR2	-0.025601114	-0.124749102	-0.02541	-0.0022	-0.08409	-0.05515
GABRR3	-0.051294465	-0.042088643	-0.09285	-0.01349	0.034549	0.013281
GAD1	-0.000279305	-0.081973722	-0.07853	0.073881	-0.00629	-0.00247
GAD2	-0.032846488	0.047190594	-0.09494	-0.04454	-0.01185	0.142211
GADD45A	0.024230438	-0.050251003	-0.00937	0.050747	-0.1463	-0.03572
GADD45B	0.025163933	-0.006216961	0.000372	-0.01097	0.01887	0.06607
GADD45G	-0.005543521	-0.065500173	-0.07006	-0.00847	-0.03476	0.099554
GADD45GII	-0.03613201	0.019522198	-0.02223	0.003503	-0.1589	0.034436
GADL1	-0.034807646	-0.003201485	-0.01308	0.006873	0.005522	-0.10555
GAFA1	-0.037142374	-0.051428216	0.099097	-0.01029	0.047995	0.013267
GAFA2	-0.025500701	-0.047032796	-0.0099	-0.01712	-0.04532	-0.12727
GAFA3	-0.042151517	-0.01148558	-0.03445	-0.01835	0.061044	0.041849
GAGE1	-0.020995198	-0.024174031	-0.00882	-0.0088	-0.02551	-0.06789
GAGE3	-0.032513075	-0.041445063	0.065229	0.0067	0.001139	0.048388
GAK	0.089307177	0.091385702	-0.08939	-0.01736	0.0524	0.059287
GAL	-0.035180289	-0.011401263	-0.0791	-0.00246	-0.0061	-0.01379
GAL3ST1	-0.034030156	-0.078847543	-0.00107	0.002959	0.009236	0.148245
GAL3ST2	-0.021964283	-0.051366227	0.091141	-0.00393	-0.02262	-0.15172
GAL3ST3	-0.038289787	-0.07544611	-0.02103	0.001862	-0.02962	-0.11827
GAL3ST4	-0.00845038	-0.145892015	0.2638	-0.03148	0.07978	-0.21361
GALC	0.017471464	0.129338094	-0.0781	-0.02412	0.063308	0.154253
GALE	0.018471721	0.005401549	-0.09758	-0.0067	-0.17573	0.08066
GALK1	-0.019391801	-0.032156828	-0.13632	0.014416	-0.02217	0.356096
GALK2	0.019049339	0.086341493	0.067694	-0.06519	0.078629	0.005244
GALM	0.018890418	0.015916246	0.242224	0.076532	-0.25905	-0.22754
GALNS	0.022057007	-0.031219135	-0.07998	-0.09826	0.258078	0.118484
GALNT1	0.064679657	0.129902006	-0.24933	0.087722	-0.2253	0.123214

GALNT10	-0.016319008	0.227623179	-0.02301	0.063221	-0.09191	-0.15733
GALNT11	0.035006491	0.124246278	0.298298	-0.10895	0.274123	-0.02077
GALNT12	0.004527303	0.075660025	0.366408	-0.0888	0.25837	-0.35994
GALNT13	-0.074502491	-0.07113413	0.056619	-0.03337	-0.01497	0.076653
GALNT14	-0.025115256	-0.02683339	-0.06435	-0.02363	0.05029	-0.10369
GALNT15	-0.027364154	-0.067808749	0.06697	0.035537	-0.1112	-0.00127
GALNT16	-0.042863294	-0.019041851	-0.17877	0.011773	-0.06504	0.051676
GALNT18	-0.049690464	-0.020215679	0.005316	-0.01518	0.046848	0.04083
GALNT2	-0.00733694	-0.089698189	0.049036	0.012725	0.00743	0.043119
GALNT3	0.00285059	0.150890809	-0.2832	0.004372	-0.00029	-0.09064
GALNT5	-0.07025309	-0.048200788	0.133094	-0.07019	-0.03437	-0.02411
GALNT6	0.012744928	0.037701261	-0.18761	-0.07304	0.169366	0.225594
GALNT7	0.028678919	-0.035697901	-0.39405	-0.02607	0.076466	0.278839
GALNT8	-0.027410015	-0.045492692	-0.04266	-0.00095	-0.01607	0.014319
GALNT9	-0.019727569	-0.088383776	-0.08661	-0.00401	-0.07788	-0.04939
GALNTL5	-0.074964075	-0.010460709	-0.05081	-0.01398	0.01819	0.045901
GALNTL6	-0.041359702	-0.110792618	-0.0461	0.013762	-0.02624	-0.04407
GALP	-0.04935255	-0.079535691	-0.00103	0.009736	-0.01135	0.046897
GALR1	-0.025928176	-0.024915631	0.053894	-0.0134	0.049554	-0.16509
GALR2	-0.035026848	-0.045895965	-0.08815	0.023844	-0.0408	0.003243
GALR3	-0.015829392	-0.028860862	0.021472	-0.01842	-0.06491	-0.00792
GALT	-0.020817491	0.013993155	0.217458	-0.08711	0.192768	-0.14994
GAMT	0.049839718	-0.132996338	0.116729	0.043559	-0.15531	-0.20947
GAN	-0.019202175	0.007271585	0.04959	0.012243	-0.09232	-0.06618
GANAB	0.033696565	0.069092027	0.160319	0.038056	-0.10871	0.008482
GANC	-0.004798656	-0.09429836	-0.16456	-0.0756	0.157066	0.096649
GAP43	-0.055410428	-0.022866488	-0.17456	0.015344	-0.06358	0.156527
GAPDH	0.069840729	0.076787918	-0.11638	0.119733	-0.25377	0.490603
GAPDHS	-0.042484119	0.002722752	-0.122	0.090176	-0.24765	0.120448
GAPT	-0.003731128	-0.187728235	-0.47757	-0.10579	0.293857	0.188705
GAPVD1	0.040861179	0.071305057	-0.14159	0.04112	-0.15536	0.193654
GAR1	0.037052858	0.081364314	0.110463	0.06642	-0.22168	-0.03486
GAREM	-0.053604213	-0.066708991	0.152501	-0.04178	0.129403	-0.21165
GAREML	-0.027314037	-0.006099221	-0.04175	0.01281	-0.09389	-0.13502
GARNL3	-0.051856142	-0.035892466	0.025819	-0.00214	0.041296	-0.09715
GARS	0.050592724	0.045604021	0.06016	0.070523	-0.21437	-0.02656
GART	-0.055097152	-0.038439559	0.058816	0.044579	-0.21519	-0.02074
GAS1	-0.020590632	0.124549688	-0.06149	-0.00204	-0.00529	-0.05554
GAS2	-0.053915556	-0.020122826	0.047923	-0.01692	0.038158	-0.16756
GAS2L1	-0.041775995	-0.106528274	-0.19697	-0.1226	0.410426	0.560385
GAS2L2	-0.026010692	-0.075265536	-0.07109	-0.02329	-0.01457	0.070486
GAS2L3	-0.010765953	-0.069502368	-0.11102	-0.01146	0.038475	0.222903
GAS5	-0.014124359	0.019210727	0.075509	0.025407	-0.04892	0.0338
GAS5-AS1	-0.026671702	0.072841546	-0.01617	0.004188	0.007042	0.124357
GAS6	0.007307873	-0.123938841	-0.1286	-0.02612	0.057741	-0.1171
GAS6-AS1	-0.016211371	-0.144856138	-0.1406	-0.03873	0.133378	0.060821
GAS7	0.006922422	0.07259029	-0.36179	-0.09552	0.283625	0.574576
GAS8	-0.014580412	-0.059039216	0.03507	-0.04491	0.00221	-0.16485
GAST	-0.029985814	-0.049896673	-0.01802	-0.00137	-0.04883	-0.02201
GATA1	-0.015164748	-0.077923817	-0.05786	-0.04136	0.036372	-0.10436
GATA2	0.010601531	-0.060129242	0.044558	-0.10389	0.11302	-0.17057

GATA3	-0.032399325	0.156580832	0.460432	-0.03041	-0.04007	-0.27484
GATA3-AS:	-0.026303655	-0.017759582	-0.01766	-0.0032	0.002504	0.041646
GATA4	-0.07367317	-0.028694386	0.05048	0.031812	-0.09765	0.021246
GATA5	-0.057712322	-0.063402746	-0.13534	-0.00328	0.040811	0.102994
GATA6	-0.028635451	-0.012751376	0.06316	-0.0015	-0.04849	-0.03913
GATA6-AS:	-0.028396649	-0.023750264	0.05246	0.00981	-0.0276	-0.01841
GATAD1	0.020714172	0.065328593	-0.01231	0.007259	0.00985	0.16879
GATAD2A	0.018739854	0.157275012	0.359103	-0.0074	-0.00019	-0.26889
GATAD2B	0.026044181	0.030272607	0.279275	-0.00363	0.025184	-0.37694
GATC	0.029766166	0.063851576	0.097077	0.020859	-0.07942	0.116738
GATM	-0.003354883	-0.03863611	-0.12798	0.041218	0.080208	-0.16522
GATS	-0.014877299	0.108560507	0.021865	-0.01143	0.002319	-0.2731
GATSL2	0.010678765	0.074517675	0.062943	-0.01033	-0.05906	-0.203
GATSL3	-0.003402891	-0.015557216	0.13589	-0.04127	0.133058	0.041562
GBA2	0.022825276	0.047594329	-0.29021	-0.03329	0.013097	0.055613
GBA3	-0.034674143	-0.058153501	-0.03833	0.012261	-0.03075	-0.02337
GBAP1	0.030251349	0.058774933	-0.15824	0.033838	-0.1083	0.329051
GBAS	0.040976599	-0.021842388	-0.19431	0.027305	-0.05977	0.46179
GBE1	0.022940652	-0.000508009	-0.25029	-0.10618	0.266548	0.170559
GBF1	0.02063733	-0.019362176	0.097038	0.028002	-0.06573	-0.08428
GBGT1	-0.003812834	-0.155290546	-0.29556	-0.08728	0.229253	0.573968
GBP1	0.017673612	0.072348471	0.115	-0.01545	0.062779	0.157764
GBP1P1	-0.030721769	0.024637905	0.023781	-0.01002	0.02868	0.141933
GBP2	0.018820396	0.109182495	0.113208	0.006885	-0.01342	0.124978
GBP3	0.017676755	0.124994022	0.19883	-0.01744	0.056675	0.149222
GBP4	0.011160277	0.163508931	0.03759	0.004916	-0.02196	0.04855
GBP5	0.017366908	0.173889286	0.19303	0.019767	-0.06561	-0.09348
GBP6	-0.044848287	-0.032080446	-0.15834	0.018114	-0.05457	-0.15837
GBX1	-0.029935082	-0.076705893	0.063706	0.006696	-0.07459	-0.02506
GBX2	-0.028022443	-0.05298986	-0.15823	-0.00848	0.053455	0.03522
GC	-0.021337886	-0.014571532	-0.01562	0.142584	-0.09931	-0.00222
GCA	0.014587079	-0.200179047	-0.39322	-0.05068	0.156476	0.3252
GCAT	-0.018411967	-0.088518707	0.062388	0.166287	-0.49585	-0.11111
GCC1	-0.060207964	0.025926512	-0.02794	-0.0286	-0.05578	-0.01016
GCC2	0.024404018	0.018222126	0.322862	-0.02605	0.063692	-0.56371
GCDH	0.026395115	0.044542876	0.189683	0.039108	-0.12196	0.035655
GCFC2	-0.000762344	-0.023660656	0.146345	-0.02036	0.063074	-0.08811
GCG	-0.043968289	-0.039997427	-0.04322	0.041947	-0.11961	-0.09987
GCGR	-0.032200264	-0.039963687	-0.00367	0.012682	-0.07157	-0.04943
GCH1	0.030798599	0.148075225	0.056288	0.021235	-0.05545	0.38929
GCHFR	0.013777122	0.123750476	0.098829	0.0392	-0.06746	0.14858
GCK	-0.029778938	-0.043142091	-0.01301	-0.00754	0.02371	0.06775
GCKR	-0.018763339	-0.054851137	-0.00753	0.008902	-0.0117	0.03162
GCLC	0.03268242	0.125448073	0.047066	0.010231	-0.03929	0.001488
GCLM	0.03776388	0.165608674	0.114965	0.036348	-0.10606	-0.15533
GCM1	-0.030346605	-0.058152944	-0.04301	0.01231	-0.0148	-0.14353
GCM2	-0.083256195	-0.013628189	-0.06204	-0.01157	0.00802	0.026204
GCN1L1	0.029072589	0.023137008	0.203422	0.055889	-0.16211	-0.17061
GCNT1	-0.001411303	0.00217123	-0.19346	-0.0054	-0.03275	-0.00176
GCNT2	-0.011296335	-0.169756265	-0.21252	-0.05143	0.156307	0.389638
GCNT3	-0.034195006	-0.039909541	0.020082	0.007003	-0.03869	-0.09291

GCNT4	-0.050197388	-0.052759557	0.054448	0.015354	-0.01363	-0.03737
GCNT7	-0.015620913	-0.069298883	-0.16048	########	-0.04934	-0.11985
GCSAM	-0.009371907	-0.093904829	0.168181	-0.02884	0.09645	-0.36823
GCSAML	-0.007021369	-0.092628516	0.00131	-0.04382	0.169446	-0.07636
GCSAML-AS	-0.019503288	-0.038764653	-0.11464	-0.01369	0.047056	-0.10144
GDA	-0.025218552	-0.034053766	0.007288	0.00262	-0.01924	-0.08528
GDAP1	-0.009807347	0.030738586	-0.00324	0.074656	-0.20592	0.207905
GDAP1L1	-0.028996526	-0.05316777	-0.13497	0.001353	-0.00244	0.190506
GDAP2	0.003359484	-0.054361001	-0.27209	-0.02621	0.049497	0.301201
GDE1	0.038207961	0.038427105	-0.25459	-0.00107	0.017062	0.441235
GDF10	-0.072659354	-0.114260241	0.080434	-0.01724	0.039649	-0.02316
GDF11	-0.029405809	0.12659792	0.200794	0.058455	-0.16044	-0.31653
GDF15	-0.054173015	-0.026467148	-0.16665	-0.06926	0.088471	0.32043
GDF2	-0.01373926	-0.065453748	-0.0218	0.001971	-0.09319	-0.08702
GDF3	-0.036567708	0.001764457	0.151953	0.01037	-0.02617	-0.01847
GDF5	-0.018065039	-0.058708123	0.02398	-0.01986	-0.05982	-0.09558
GDF9	-0.023889237	-0.026480675	0.037308	-0.0118	-0.01654	-0.08594
GDI1	0.075093654	-0.034508596	0.021936	0.057796	-0.11958	-0.15174
GDI2	0.075782212	0.010015294	-0.23718	0.034128	-0.0497	0.044899
GDNF	-0.04607683	-0.021436384	-0.0336	0.023593	-0.06935	-0.00019
GDPD1	0.005336297	0.033429338	0.150719	0.095797	-0.01907	0.012235
GDPD2	-0.030430289	-0.093359291	-0.00376	-0.00821	-0.06418	-0.07991
GDPD3	-0.008246951	-0.080837971	-0.02539	-0.04378	0.077818	-0.03633
GDPD5	0.025314078	-0.073353804	0.127357	-0.04885	0.120743	-0.0051
GDPGP1	-0.031296448	0.087404882	0.154931	-0.00885	0.046209	-0.04896
GEM	-0.008005529	-0.000374255	-0.05074	0.069623	-0.18371	-0.03034
GEMIN2	0.002719644	0.052972617	0.079383	0.092649	-0.29869	0.079574
GEMIN4	0.00821612	-0.007686582	0.134808	0.05898	-0.19721	-0.03876
GEMIN5	0.006378606	0.053943525	0.046526	0.043335	-0.15222	-0.06907
GEMIN6	0.01044366	0.019180342	0.070282	0.047112	-0.16626	0.212401
GEMIN7	0.032514179	0.099571393	-0.07941	0.051141	-0.12086	0.067001
GEMIN8	-0.01837166	-0.027212748	0.082876	0.042814	-0.07287	-0.07593
GEN1	-0.008535774	0.015267025	-0.06389	0.077834	-0.19431	-0.11159
GET4	0.037794444	-0.018711978	0.195294	0.001049	-0.06772	-0.17411
GFAP	-0.050305201	-0.041149617	-0.01076	-0.00956	0.059671	0.018078
GFER	0.006559003	-0.096438517	-0.03639	0.097035	-0.32728	0.30295
GFI1	0.009147776	0.256018	0.277477	0.034022	-0.1318	-0.26733
GFI1B	-0.014086605	-0.056698609	-0.09642	-0.16282	0.424101	-0.07064
GFM1	0.024115747	0.072980318	0.077693	0.08698	-0.18714	0.09994
GFM2	-0.011557925	0.089127043	-0.04295	0.060874	-0.1721	-0.05821
GFOD1	0.00081522	0.185838701	-0.14715	-0.00294	-0.01279	-0.07317
GFOD2	-0.009625075	-0.03538654	-0.09119	0.038377	-0.20315	-0.0074
GFPT1	0.009134083	0.06389681	0.193512	0.074608	-0.19006	-0.14696
GFPT2	-0.012998569	-0.041647679	0.032841	0.002324	-0.01133	-0.04385
GFRA1	-0.031087218	-0.046259904	-0.0271	-0.00226	0.025255	0.014535
GFRA2	-0.030478048	-0.087039051	-0.16413	-0.02387	0.078854	0.260576
GFRA3	-0.041100753	-0.080430796	0.039049	-0.00944	-0.02221	-0.02754
GFRA4	-0.059003495	-0.054594607	-0.06713	0.021377	-0.04475	0.066211
GGA1	0.012455955	-0.10049968	0.279864	0.009752	-0.12623	0.03422
GGA2	0.022921784	-0.063367285	-0.09156	-0.00333	0.039149	-0.13297
GGA3	0.02700657	-0.10035745	-0.12794	-0.02171	0.038426	0.051338

GGACT	-0.015423577	0.085148302	0.012732	-0.03528	0.126076	0.052573
GGCT	0.041629121	0.064110168	0.047559	0.076168	-0.22416	0.103137
GGCX	0.045313774	0.021410875	-0.20182	-0.01909	-0.04131	0.323424
GGH	0.00198334	-0.017776588	-0.05059	0.119716	-0.32538	-0.01944
GGN	-0.046371743	-0.063902641	-0.00216	0.004526	0.002703	0.094693
GGNBP2	0.00920991	-0.020960706	0.132105	-0.03289	0.11361	-0.17522
GGPS1	0.030773568	0.045393161	0.04175	-0.0055	0.044558	0.032334
GGT1	-0.018128592	-0.009573302	0.030063	-0.00918	-0.08015	-0.14095
GGT5	-0.033141263	-0.062080252	-0.12345	0.003912	-0.04015	-0.01331
GGT6	-0.038332432	-0.081769751	0.002348	0.018525	-0.05238	-0.02792
GGT7	-0.041170499	-0.078401498	0.177647	-0.09175	0.222238	0.101655
GGTA1P	0.009302209	-0.114903477	-0.13335	-0.1311	0.371102	0.373728
GGTLC1	-0.012936425	-0.114165424	-0.11567	0.059747	-0.22654	0.15529
GGTLC2	-0.023566732	-0.153946851	0.022137	-0.01609	-0.04324	-0.11246
GH1	-0.037609814	-0.031451178	4.62E-05	0.008411	0.014343	0.007208
GH2	-0.066516007	-0.106356073	0.10042	0.027667	-0.07861	0.024417
GHDC	0.007994957	-0.056042796	-0.01807	-0.03376	0.071182	0.267364
GHET1	-0.025071059	0.030722872	0.185247	0.079941	-0.21226	-0.11043
GHITM	0.160349159	0.14524524	-0.14965	0.106237	-0.21557	0.0346
GHR	-0.04959129	-0.046542618	0.011626	-0.02109	0.041399	-0.01438
GHRH	-0.026535091	-0.054420908	-0.09517	0.010558	-0.01936	0.017596
GHRHR	-0.030121156	0.017626323	0.005282	0.031499	-0.0978	0.124105
GHRL	-0.018331094	-0.156046108	-0.23251	-0.01795	0.019108	0.000383
GHRLOS	-0.008405668	-0.007205244	-0.00921	-0.00172	0.02399	-0.0819
GHSR	-0.039657601	-0.00863908	0.004547	0.006763	-0.00057	-0.02633
GID4	-0.003479995	0.032635251	0.060317	0.00515	-0.00499	0.03113
GID8	0.096698482	0.059620418	0.142487	0.066939	-0.22359	-0.23294
GIF	-0.046945818	0.01872901	0.00207	-0.00909	0.062968	0.179984
GIGYF1	-0.002877139	-0.020184826	-0.00053	0.029216	-0.0267	-0.08524
GIGYF2	-0.003487159	0.012266507	-0.16759	0.020516	-0.18342	-0.0369
GIMAP1	0.016157073	0.075345252	0.261686	-0.0513	0.213655	0.230855
GIMAP2	0.018838774	0.094097125	0.269453	-0.02486	0.069432	0.196736
GIMAP4	0.015531872	0.104478393	0.184016	-0.0586	0.159117	0.217691
GIMAP6	0.013587148	0.145971006	0.291952	-0.03477	0.097054	0.221539
GIMAP7	0.014400518	0.135884373	0.275975	-0.06936	0.198129	0.231057
GIMAP8	0.007519751	0.044837711	0.07597	-0.10243	0.294023	0.416748
GIN1	0.010993698	0.062264474	0.06173	-0.00583	0.022035	-0.03208
GINM1	0.05223222	-0.021966033	-0.34698	0.028355	-0.0763	0.29763
GINS1	-0.009857998	0.027796465	-0.00125	0.131028	-0.31272	-0.11092
GINS2	0.028172967	0.030743984	0.016796	-0.02767	-0.37718	-0.10516
GINS3	-0.007296485	0.054030258	-0.10308	0.130842	-0.35711	-0.00979
GINS4	-0.031649921	0.009270582	-0.01515	0.149946	-0.31333	-0.03925
GIP	-0.037392833	-0.039746825	0.164227	0.003703	0.012744	-0.03881
GIPC1	0.015184341	-0.001356185	0.296012	0.001411	-0.07215	-0.28672
GIPC2	-0.035128435	-0.093388603	0.057256	-0.02285	0.03415	-0.06535
GIPC3	-0.040656375	-0.113695255	0.088402	-0.01299	-0.03133	-0.1292
GIPR	-0.023863628	-0.029352583	0.017785	-0.00157	-0.04666	-0.15842
GIT1	0.047728623	-0.039537423	0.031976	0.008123	-0.08532	-0.15421
GIT2	0.021836459	-0.021176112	-0.14791	-0.04683	0.142036	0.168595
GJA1	-0.021748146	-0.008295119	0.001702	0.112448	-0.12264	-0.02506
GJA10	-0.028113463	-0.013932801	-0.12705	0.034725	-0.08809	0.098319

GJA3	-0.052276399	-0.09176235	0.17026	-0.01102	0.040453	0.045646
GJA4	-0.052205323	-0.082213774	0.059661	-0.00034	0.006934	-0.06716
GJA5	-0.051923582	-0.020243592	-0.06569	0.011936	-0.0066	-0.00175
GJA8	-0.030879632	-0.11343642	0.05822	0.015546	-0.07582	-0.055
GJB1	-0.019093219	-0.065928419	-0.03651	0.012758	-0.11806	-0.01081
GJB2	-0.011950103	-0.016771715	-0.01554	-0.00538	-0.01057	0.011661
GJB3	-0.013458579	-0.11247358	-0.1754	0.010587	-0.0843	-0.01017
GJB4	-0.026106162	-0.038282942	-0.05349	-0.02008	0.007029	-0.09305
GJB5	-0.040245615	-0.052105388	-0.02221	0.034717	-0.08078	0.082491
GJB6	-0.019536365	-0.068935465	0.14414	-0.02475	0.007141	-0.10005
GJC1	-0.036943344	-0.04418528	-0.00993	0.035938	-0.00811	-0.0245
GJC2	-0.022436236	-0.062549584	0.077305	0.002661	-0.09335	-0.06749
GJD2	-0.025568552	-0.073559344	-0.00415	0.017153	-0.09497	-0.11768
GJD3	-0.041578068	-0.044991858	-0.0959	-0.0013	0.002492	0.161395
GJD4	-0.031022306	-0.064884848	-0.0459	0.034074	-0.07178	-0.0038
GK	-0.002084619	-0.064792772	-0.21793	0.015011	-0.03128	0.122493
GK2	-0.031405802	-0.032718519	-0.03575	0.020962	-0.04442	-0.01509
GK3P	-0.009949947	-0.031821069	-0.18342	0.00835	-0.04169	0.099351
GK5	-0.026462864	0.327659015	0.002497	0.035206	-0.0193	-0.07716
GKAP1	-0.011231022	0.005170568	0.160992	0.048199	0.034452	-0.25836
GKN1	-0.047313771	-0.022791515	-0.02526	0.021395	-0.01991	0.069172
GKN2	-0.025211519	-0.0374914	0.062936	-0.0103	0.009859	-0.02801
GLA	0.037596648	-0.02725365	-0.29392	-0.00093	0.004936	0.415763
GLB1L	-0.00778811	-0.108145898	-0.13217	-0.02796	0.084259	0.572523
GLB1L2	-0.01484544	-0.080390927	0.121785	0.004828	-0.07043	-0.07081
GLB1L3	-0.041679553	-0.083271401	0.056721	-0.00239	-0.05265	-0.14314
GLCCI1	-0.047159409	0.152218501	0.145854	-0.01578	-0.03054	-0.38815
GLCE	-0.008079751	-0.076787399	-0.13845	-0.01787	0.073545	0.412789
GLDC	-0.011766079	-0.032274906	-0.03375	-0.023	0.030477	-0.0226
GLDN	-0.020910145	-0.08207918	-0.02253	-0.00248	-0.03532	-0.1477
GLE1	-0.004910568	0.03665861	-0.08726	0.061629	-0.23396	-0.10857
GLG1	0.04468967	0.092032014	0.208849	-0.02401	0.085597	-0.08253
GLI1	-0.031663367	-0.088879134	-0.00403	0.012172	-0.06336	-0.10491
GLI2	-0.036859167	-0.033265128	-0.10264	0.02094	-0.03568	0.181981
GLI3	-0.023034874	0.128744134	-0.08479	0.020791	-0.06103	-0.12479
GLI4	-0.035595236	-0.06476385	-0.11652	0.001291	-0.00854	0.029291
GLIDR	0.009426166	-0.010311087	-0.01795	-0.00159	-0.0552	-0.08707
GLIPR1	0.024984212	-0.012980461	-0.25625	-0.07795	0.297434	0.358494
GLIPR1L1	-0.034341871	-0.046770146	0.062723	0.025112	-0.05585	-0.09018
GLIPR2	0.024163531	0.096286991	-0.2145	-0.03841	0.118971	0.360826
GLIS1	-0.028044729	-0.053889454	0.088881	0.019294	-0.02892	0.06523
GLIS2	-0.029331696	-0.04557747	-0.04351	0.101552	-0.30497	-0.01177
GLIS3	-0.027406639	-0.069922245	0.169481	-0.02897	0.05711	-0.06192
GLIS3-AS	-0.031849989	-0.044082671	-0.14357	0.00226	0.000864	-0.00922
GLMN	0.021136603	0.113373443	0.068087	0.098134	-0.31212	-0.30979
GLO1	0.039763252	0.044816956	0.033819	0.075666	-0.21863	-0.16859
GLOD4	0.027205338	0.063389286	0.224368	0.031535	-0.09172	-0.0493
GLOD5	-0.097608326	-0.051016464	-0.07397	0.006356	0.047673	-0.02129
GLP1R	0.00352121	-0.084137654	-0.04267	0.007162	0.071107	-0.04773
GLP2R	-0.039846974	-0.016679184	0.146268	0.012714	-0.01225	0.100254
GLRA1	-0.041180078	-0.064646788	-0.13181	-0.00823	0.050127	0.137893

GLRA2	-0.032702791	-0.060810795	-0.05518	0.04813	-0.16082	-0.0443
GLRA3	-0.060953153	-0.069377296	-0.00234	-0.0135	0.057843	0.044197
GLRB	-0.130283981	-0.101390348	-0.10452	0.051114	0.027633	-0.02375
GLRX	0.040483462	0.046706844	-0.05734	-0.02407	0.072473	0.231565
GLRX2	0.028363965	0.108267476	-0.09956	0.080005	-0.24275	0.281849
GLRX3	0.022592127	0.020822217	-0.01984	0.035689	-0.12248	0.017706
GLRX5	0.082858103	0.100296285	0.058888	0.104383	-0.27536	-0.14685
GLS	0.035643852	0.114833424	0.234865	0.028859	-0.09373	-0.38702
GLS2	-0.036586584	-0.093131916	0.362283	0.004287	0.045327	-0.09025
GLT1D1	0.013516776	-0.095507023	-0.29702	-0.08326	0.233985	0.412917
GLT8D1	0.040871885	0.110708961	0.050783	0.012061	-0.02847	-0.01337
GLT8D2	-0.067542188	-0.073700583	-0.13239	0.015991	-0.08449	0.078485
GLTP	0.049633924	0.175638547	-0.07534	-0.02293	0.062922	0.417263
GLTPD2	-0.029794579	-0.025981407	-0.03851	0.001162	0.026902	0.130543
GLTSCR1	0.020794749	-0.057704665	0.155754	-0.02425	-0.00224	-0.18498
GLTSCR1L	0.028601248	0.100432872	0.130544	-0.02098	0.047476	-0.18076
GLTSCR2	-0.011419417	-0.087864615	0.005592	0.002115	-0.04779	0.025048
GLUD1	0.040429771	0.058022733	-0.0227	0.049114	-0.13973	0.21114
GLUD2	0.003409888	0.044899716	-0.00768	0.023503	-0.07861	0.122166
GLUL	0.015532901	-0.01807591	-0.3341	-0.01105	0.043094	0.204639
GLYAT	-0.05480478	-0.029578808	0.04553	-0.01088	-0.09227	-0.12871
GLYATL1	-0.039215316	-0.103247397	0.008664	-0.03354	0.103071	-0.14887
GLYATL2	-0.044734154	0.029142905	-0.1199	-0.0096	0.05213	-0.01984
GLYCTK	-0.009046255	-0.029058599	0.012118	-0.03984	-0.03129	0.153296
GLYCTK-AS	-0.038201988	-0.056908586	0.087143	0.01957	-0.03165	-0.0968
GLYR1	0.028321639	0.017306612	0.084125	-0.07181	0.17774	-0.00507
GM2A	0.018995711	-0.096509264	-0.33666	-0.08643	0.136638	0.428441
GMCL1	0.027132252	0.124512482	0.048069	0.006591	-0.02917	-0.32269
GMDS	-0.009389744	-0.000637728	0.040236	0.039897	-0.06947	0.09763
GMEB1	0.023792279	0.159165358	-0.0712	0.015203	-0.02533	-0.01232
GMEB2	0.05361111	-0.036035421	0.104577	-0.01902	0.008424	-0.25216
GMFB	0.043131125	0.075022945	-0.32098	0.054081	-0.15429	0.168864
GMFG	0.040807732	-0.002893688	-0.03313	0.020368	-0.04814	0.047343
GMIP	0.025520414	-0.044963671	-0.14061	-0.07857	0.180124	0.041393
GML	-0.024888652	-0.052670994	0.227502	0.054712	-0.16877	-0.22225
GMNC	-0.032686059	-0.002148808	0.040162	-0.00031	0.015755	-0.0549
GMNN	0.022176185	0.097715988	-0.05891	0.123665	-0.33367	-0.00397
GMPPA	0.015805081	-0.012687598	-0.13311	0.031351	-0.11601	0.288047
GMPR	-0.010981222	-0.082882336	-0.13195	-0.12834	0.36282	0.202
GMPR2	0.035411166	-0.018994315	-0.05237	-0.00941	0.038263	0.091105
GMPS	-0.020084472	0.089417407	0.184978	0.007785	-0.05233	-0.16382
GNA11	-0.008293813	-0.179790053	-0.00069	-0.06582	0.195499	0.25864
GNA12	0.020503911	-0.074760855	-0.13801	-0.00799	-0.00548	-0.01899
GNA13	0.040968468	0.159142375	-0.09968	-0.00602	0.01204	-0.08159
GNA14	-0.043811788	-0.094398067	0.117275	0.020778	-0.03329	-0.11787
GNA14-AS	-0.035687395	-0.010675639	-0.10228	-0.00475	0.040629	0.023631
GNA15	0.011241714	-0.110973695	-0.26181	-0.05704	0.124456	0.2941
GNAI1	-0.017260523	-0.021479492	0.005162	-0.0589	0.174815	0.003194
GNAI2	0.040937548	0.00903658	-0.18734	-0.01737	0.06697	0.190331
GNAI3	0.057873787	0.068237546	-0.34982	0.042636	-0.10758	0.159681
GNAL	-0.022045215	0.246728858	-0.05085	0.145754	-0.19071	-0.0646

GNAO1	-0.026151234	-0.050346884	0.064006	-0.03738	0.084792	-0.03879
GNAQ	-0.006256987	-0.123296353	-0.18905	-0.10045	0.273562	0.23203
GNAS	0.037052828	-0.096487108	0.095084	-0.03249	0.014449	-0.36408
GNAS-AS1	-0.046778085	0.0374332	0.040976	0.046912	-0.11302	-0.02412
GNAT1	-0.046555709	-0.052720993	-0.00693	0.012222	-0.00448	0.017179
GNAT2	-0.031035393	-0.026581678	-0.09031	-0.02869	0.084233	-0.03821
GNAT3	-0.052195119	-0.029256923	-0.0633	-0.00363	-0.00125	-0.04719
GNAZ	-0.013568438	-0.000345254	-0.08231	-0.19095	0.492948	-0.12058
GNB1	0.144756144	0.189486647	-0.25897	0.059332	-0.08136	-0.08346
GNB1L	-0.034508519	-0.075177217	-0.0396	-0.02749	-0.06521	-0.0357
GNB2	0.034265281	-0.03030836	-0.27051	0.038929	-0.08043	0.158935
GNB3	-0.017924543	-0.077449539	-0.02948	7.12E-05	-0.10092	-0.15571
GNB4	0.05632159	-0.143756116	-0.56931	-0.07087	0.198673	0.190535
GNB5	-0.015245592	-0.003851345	0.186172	0.009138	0.064939	-0.29871
GNE	0.022939326	0.018010853	0.079777	0.066629	-0.16858	-0.00454
GNG10	0.047417146	-0.057727844	-0.3044	0.037741	-0.09135	0.222823
GNG11	-0.010804916	-0.031823916	-0.0816	-0.21438	0.609822	0.045052
GNG12	-0.04555808	-0.061102581	-0.01455	-0.02622	0.009039	0.056537
GNG13	-0.041839924	0.003021989	0.017426	-0.01052	0.054696	-0.03572
GNG2	-0.005430675	0.176782328	0.013544	0.019802	-0.03829	-0.05492
GNG3	-0.028842862	-0.061625853	-0.1368	0.0002	-0.02713	-0.24109
GNG4	-0.018996204	-0.019431903	-0.02644	0.011662	-0.03134	-0.01606
GNG5	0.080529324	0.022159328	-0.32129	0.047027	-0.09112	0.382485
GNG7	-0.007495532	-0.162027559	-0.11847	-0.07912	0.243463	-0.2042
GNG8	-0.018968727	-0.095313931	-0.0208	-0.11974	0.358687	0.053491
GNGT1	-0.065314418	-0.020695436	-0.07454	-0.02045	0.052037	0.002847
GNGT2	0.010553344	0.226269377	-0.10536	0.032952	-0.13154	0.134398
GNL1	-0.08782037	-0.02730138	-0.07638	0.017336	-0.1414	-0.03591
GNL2	0.028280858	0.042889372	-0.06119	0.056178	-0.13898	0.245498
GNL3	-0.005119215	-0.05311736	0.159191	-0.00663	0.061871	-0.11176
GNL3L	0.011358731	0.067297839	-0.0712	0.064569	-0.16978	0.193434
GNLY	0.015677487	0.263068235	0.360792	-0.02224	0.059968	-0.22186
GNMT	-0.008494968	-0.101783584	-0.1807	-0.00767	0.04469	0.10158
GNN	-0.050731867	-0.08711882	0.051493	-0.00558	0.041019	-0.15383
GNPAT	0.052182358	0.052148482	-0.06355	0.054353	-0.14516	0.085392
GNPDA1	0.024349803	-0.002671764	0.115487	-0.06449	0.182126	0.253736
GNPDA2	0.01125833	0.078702291	0.174074	-0.02392	0.072586	-0.22725
GNPNAT1	0.002704395	0.063324747	0.227611	0.045582	-0.17066	-0.23894
GNPTAB	-0.007101795	0.217544124	0.003987	-0.00199	-0.07412	-0.0514
GNPTG	0.059724419	-0.043830897	-0.14172	-0.08167	0.207054	0.380336
GNRH1	-0.016274447	0.146654052	0.034206	0.004891	-0.01308	-0.1715
GNRH2	-0.026968694	-0.029547314	-0.09725	0.027674	-0.09876	-0.02635
GNRHR	-0.064233736	-0.081150107	0.052007	0.015653	0.068607	0.038473
GNRHR2	-0.030817099	-0.007885957	0.14293	-0.003	0.00382	-0.11106
GNS	0.026659654	0.068209538	-0.2963	-0.04313	0.129848	0.504873
GOLGA1	0.014442901	0.012495628	0.016514	-0.03064	0.079579	-0.07144
GOLGA2	0.105653303	-0.028838138	-0.08353	0.081377	-0.04622	-0.04288
GOLGA2P5	-0.007639873	-0.029721078	0.213178	0.014222	0.060595	-0.31859
GOLGA3	0.022094859	0.020044048	0.094008	-0.01342	-0.03749	-0.1835
GOLGA4	0.03966253	0.075481913	0.135877	0.015942	-0.04958	-0.16998
GOLGA5	0.035828601	0.027974554	-0.19579	0.026649	-0.0811	0.01225

GOLGA6A	-0.026187088	-0.0243145	0.142479	0.002369	0.002486	-0.12734
GOLGA6L2	-0.038239082	-0.037616166	0.124567	-0.00228	0.025599	-0.00325
GOLGA6L6	-0.053922027	-0.040856653	0.021862	0.003762	-0.00383	-0.02083
GOLGA7	0.076853182	0.219769745	0.066403	0.000974	0.02661	-0.04946
GOLGA7B	-0.015761523	-0.003557546	0.244683	-0.01526	-0.02457	-0.2062
GOLGA8A	0.016766952	0.069719022	0.349967	-0.0164	0.03867	-0.56383
GOLGA8N	0.059297257	0.298428002	-0.00342	0.02745	-0.05109	-0.25229
GOLGB1	0.048158373	-0.030536497	-0.12854	-0.00945	0.039046	-0.10965
GOLIM4	-0.012715179	0.093843381	-0.3086	0.040128	-0.11862	0.234186
GOLM1	-0.003747962	0.162834589	-0.12664	-0.08405	0.244944	0.483878
GOLPH3	0.126749936	0.2678046	-0.11517	0.058241	-0.12532	-0.04387
GOLPH3L	0.01998035	0.056648584	0.073629	-0.01195	0.013511	-0.28227
GOLT1A	-0.045599845	-0.088988392	0.13678	0.0041	0.024364	-0.02523
GOLT1B	0.042592032	0.070062341	0.084846	0.028022	-0.09783	0.035284
GON4L	0.050924678	0.103644294	0.245476	-0.01287	-0.00226	-0.10302
GOPC	0.027742837	0.173596114	0.206927	0.012509	-0.08495	-0.17633
GORAB	0.015604511	0.034204971	0.076029	0.037935	-0.06126	0.173417
GORASP1	-0.000892202	-0.155810405	-0.28846	-0.07233	0.198709	0.311764
GORASP2	0.025149458	0.085456789	0.137795	0.03459	-0.0614	0.046275
GOSR1	0.026079173	0.081017827	-0.02748	0.060907	-0.1496	0.026612
GOSR2	-0.01954959	0.043239629	-0.0346	0.00674	-0.03976	0.004224
GOT1	0.023229843	0.05110181	0.038291	0.094689	-0.27862	-0.18414
GOT1L1	-0.029622596	-0.097491947	-0.06851	0.006489	0.019368	-0.04347
GOT2	0.041805423	-0.011777148	0.163138	0.105126	-0.30474	-0.18663
GP1BA	-0.007354383	-0.043701502	-0.04065	-0.15329	0.378721	0.060135
GP2	-0.055536915	-0.115697115	-0.02039	0.010186	-0.00016	-0.02205
GP5	-0.019191472	-0.089560929	0.450337	-0.14574	0.433429	-0.18754
GP6	-0.024718961	-0.038485975	-0.01425	-0.21161	0.612179	0.090478
GP9	-0.012386634	-0.051938189	-0.00508	-0.19027	0.458396	-0.04392
GPA33	-0.011633924	0.013089092	0.197926	-0.11968	0.280961	-0.19644
GPAA1	0.039926571	0.072348175	-0.00183	0.077017	-0.27939	-0.0427
GPALPP1	0.007469708	0.043140623	0.232605	0.057307	-0.03441	-0.05739
GPAM	-0.002221029	0.129224199	0.119162	0.039381	-0.08181	-0.20901
GPANK1	0.035489797	0.047660086	0.029394	0.038813	-0.16379	0.038701
GPAT2	-0.004513436	0.023413414	0.079766	-0.01657	0.022549	0.007033
GPATCH1	0.029866282	0.06009838	-0.00271	0.008698	-0.00985	0.00922
GPATCH11	0.008475441	0.025788486	-0.05009	0.015941	-0.00926	-0.10316
GPATCH2	0.008511656	0.035369996	-0.00631	0.072087	-0.16776	0.021969
GPATCH2L	0.033785015	0.063292031	-0.05785	-0.13691	-0.04924	0.036212
GPATCH3	0.013483765	0.006117574	-0.06335	-0.02064	0.028104	0.22406
GPATCH4	-0.036688555	-0.126649545	-0.0134	0.005324	0.018967	-0.05745
GPATCH8	0.052035744	0.251070872	0.225794	-0.00901	0.003282	-0.30139
GPBAR1	-0.000960466	-0.074335212	-0.26562	-0.08673	0.264872	0.672921
GPBP1	0.06617765	0.145843132	0.059813	0.032264	-0.07334	-0.39252
GPBP1L1	0.087648978	0.028324323	-0.03136	0.008326	-0.05667	-0.18935
GPC1	-0.034138824	-0.083489941	-0.10991	0.045802	-0.19923	-0.03761
GPC2	-0.014458114	-0.087489116	0.174477	-0.00646	0.025027	-0.14943
GPC3	-0.038283029	-0.061487293	0.068885	0.054138	-0.13826	-0.07881
GPC4	-0.039342067	-0.060380448	-0.08361	0.008995	-0.01927	0.058254
GPC5	-0.048998403	-0.053701506	0.013979	-0.02398	0.072421	-0.02459
GPC6	-0.03312438	-0.065501406	-0.01515	0.079931	-0.2473	-0.03755

GPCPD1	0.034477083	0.026793208	-0.28712	-0.08759	0.269212	0.223923
GPD1	-0.028821464	-0.064551472	-0.15527	0.013122	0.069444	0.067584
GPD1L	0.024186316	0.044185152	0.110902	0.001491	-0.02614	-0.14409
GPD2	-0.026389021	0.003687892	-0.12178	-0.10489	0.239017	0.245069
GPER1	-0.02618429	-0.091401687	-0.16123	-0.03529	0.118396	0.315304
GPHA2	-0.031166677	-0.071484228	-0.05636	-0.02275	0.035849	-0.0679
GPHB5	-0.037155651	-0.016171641	-0.10665	0.006604	-0.02008	0.041043
GPHN	0.055952226	-0.034239673	0.133624	-0.11967	0.086368	-0.01667
GPI	0.025116708	0.002733989	0.12461	0.094498	-0.24924	0.071033
GPIHBP1	-0.034747347	-0.041877525	-0.06417	-0.00536	-0.01422	-0.08737
GPKOW	0.047814641	0.177722575	0.022152	0.026647	-0.11807	-0.01977
GPLD1	-0.101999079	-0.105069543	-0.03973	0.029099	0.032934	-0.19702
GPM6A	-0.040052286	-0.089910163	-0.13256	-0.04508	0.008649	-0.26639
GPM6B	-0.018032377	-0.053577528	-0.07236	-0.01539	0.044998	-0.18295
GPN1	0.048405093	0.124793868	0.134754	0.060973	-0.17635	-0.01137
GPN2	0.016188871	0.006697394	0.041349	0.042749	-0.11539	0.200512
GPN3	0.030516656	0.010756388	-0.17417	0.08066	-0.21473	0.172975
GPNMB	-0.007971601	-0.053205692	-0.04536	0.07111	-0.20202	0.221425
GPR1	-0.049979723	-0.014531102	-0.0176	-0.0251	0.110403	-0.00185
GPR1-AS	-0.052709868	-0.075331593	-0.09955	-0.01843	0.066893	0.266239
GPR101	-0.036548856	-0.045997364	-0.07876	0.014352	-0.03634	-0.00875
GPR107	-0.006714002	-0.022281861	-0.19324	0.019983	-0.11885	0.238817
GPR110	-0.051475598	-0.01063028	0.022575	0.001379	0.037975	0.001945
GPR111	-0.046983378	-0.055840136	-0.03655	0.019445	-0.0464	-0.08731
GPR112	-0.039261173	-0.036941487	0.035376	0.000977	0.033355	-0.07342
GPR113	-0.045480963	-0.08254893	0.167204	-0.00527	-0.0282	-0.11179
GPR114	0.000310635	0.128134079	-0.05554	0.015685	-0.08164	-0.35386
GPR115	-0.085219541	-0.030185813	0.004697	0.018157	-0.08092	-0.01555
GPR116	-0.038524698	-0.056287398	0.055613	0.009497	-0.0174	-0.04051
GPR12	-0.041668144	-0.05475768	-0.13086	0.002451	0.011471	0.131455
GPR123	-0.029841005	-0.002213531	-0.01034	-0.00033	0.024033	0.034559
GPR124	-0.018140495	-0.001297929	-0.12904	-0.04435	0.14553	0.565244
GPR125	-0.022142096	0.073027893	0.016922	0.02898	-0.08977	-0.14521
GPR126	0.007809511	-0.072479712	0.069511	-0.01768	0.059737	-0.17714
GPR128	-0.026900741	-0.064692744	-0.02729	-0.00892	0.006594	-0.03431
GPR132	-0.000939286	-0.053246365	0.096664	0.031172	-0.09896	0.041247
GPR133	-0.010426545	-0.11404893	-0.21595	-0.04248	0.141028	0.699559
GPR135	-0.023084434	-0.093516342	0.070222	0.013004	0.060742	0.122176
GPR137	-0.008330248	-0.001765723	-0.01471	0.027946	-0.12802	-0.01389
GPR137B	0.011647096	0.014662267	0.037202	-0.069	0.1638	0.176022
GPR137C	-0.028864175	-0.052211496	-0.00338	0.000104	-0.04283	-0.02784
GPR143	-0.030455547	0.00907073	0.078398	0.017035	-0.03295	-0.0942
GPR144	-0.016743567	-0.129457736	0.082316	0.016885	-0.08986	-0.00725
GPR146	-0.008290976	-0.03614311	0.027831	-0.01023	0.045061	-0.07569
GPR15	-0.009612006	-0.003840938	0.059357	0.044554	-0.13339	-0.17304
GPR150	-0.025411383	-0.017586007	-0.03272	0.042075	-0.09714	0.059109
GPR153	0.014966825	0.051593872	0.029387	-0.02188	-0.01291	-0.19736
GPR155	0.05194041	0.01781972	0.195907	-0.03337	0.079775	0.14831
GPR156	-0.032764434	-0.090547344	-0.01271	0.007648	-0.09795	0.035085
GPR157	-0.008197495	-0.072246908	0.062871	-0.03558	0.06043	-0.0708
GPR158	-0.023783655	-0.030305805	-0.0295	0.068994	-0.18494	-0.06207

GPR160	0.012011715	-0.196568459	-0.1917	-0.09301	0.248439	-0.05153
GPR161	-0.069001717	-0.058168486	-0.06032	0.024579	0.033786	-0.02253
GPR162	-0.021956543	-0.126682443	-0.20078	-0.04023	0.103769	0.380606
GPR17	-0.052797443	-0.072791756	0.097881	0.003955	0.023003	-0.02983
GPR171	0.005567488	0.031881852	0.45546	0.031704	-0.11012	-0.42602
GPR173	-0.063221132	-0.069469287	-0.03473	0.036032	-0.11769	0.200361
GPR174	-0.008143212	0.108208616	0.204397	-0.03402	0.101485	-0.28156
GPR176	-0.032972668	-0.043505683	0.000495	-0.005	-0.05777	-0.10401
GPR18	0.008086689	0.077602784	0.226041	-0.01125	0.029294	-0.41362
GPR180	-0.009549209	0.017410158	0.118009	0.073389	-0.11585	-0.14346
GPR182	-0.033557626	-0.054075471	-0.00129	0.011387	-0.07641	-0.02896
GPR183	0.020048721	-0.095654001	0.134128	-0.04741	0.128853	-0.39423
GPR19	-0.007885088	0.015024911	0.073497	0.092066	-0.27156	-0.11688
GPR20	-0.028037881	-0.033370421	-0.09032	-0.01059	0.047785	0.271129
GPR21	-0.030613432	-0.018854662	-0.01979	-0.01423	-0.03973	0.019748
GPR22	-0.176200464	-0.045639868	0.047549	0.035907	0.017991	0.067871
GPR25	-0.030983567	0.054867306	-0.05761	-0.092	0.291429	0.033658
GPR26	-0.020965787	-0.103870118	-0.02457	0.037492	-0.06463	-0.11146
GPR27	-0.000539521	-0.11199749	-0.17945	-0.06334	0.154217	0.296332
GPR3	-0.034442875	-0.05150164	-0.10401	0.056758	-0.16389	-0.13176
GPR31	-0.030763365	-0.037349349	0.003337	-0.00772	0.023934	-0.03634
GPR32	-0.046080761	-0.065863279	-0.08451	0.015061	-0.01047	0.019131
GPR34	0.001389985	-0.038961047	-0.23209	-0.09201	0.250442	0.436256
GPR35	-0.00192609	-0.035431198	-0.16359	-0.02675	0.020837	0.302354
GPR37	-0.082750296	-0.116111115	-0.05038	0.009912	0.035236	0.003898
GPR37L1	-0.038610267	-0.014565976	-0.01331	0.02136	-0.05645	-0.01428
GPR39	-0.069302681	-0.062718004	-0.11949	-0.03925	-0.04402	-0.06517
GPR4	-0.040161792	-0.078739394	-0.09178	0.038647	-0.1141	0.002146
GPR45	-0.037633084	-0.059153695	-0.00834	0.027812	-0.06439	-0.03746
GPR50	-0.043950017	-0.058182155	-0.08958	0.002564	0.025763	-0.04301
GPR52	-0.025585492	-0.040812414	0.043301	0.013153	-0.06476	-0.12568
GPR55	-0.009874739	-0.037877594	0.091166	-0.01111	0.043557	-0.05812
GPR56	0.002929913	0.310862391	0.366015	-0.0864	0.211482	-0.23837
GPR6	-0.052156232	-0.008615216	0.020166	0.019107	-0.04675	-0.12629
GPR61	-0.035133813	-0.046191917	0.072282	-0.00313	0.036553	-0.14385
GPR62	-0.031904212	-0.031393215	-0.06331	-0.00626	-0.05177	-0.09132
GPR63	-0.017957436	-0.036687137	-0.02054	0.010554	-0.03784	-0.12122
GPR64	-0.029256423	0.017667271	0.019639	0.067594	-0.18574	-0.03938
GPR65	0.042005365	0.240221804	0.029889	-0.00814	0.046534	-0.0537
GPR68	0.001130391	0.202234574	0.102773	0.061916	-0.22197	-0.14342
GPR75	-0.029513099	-0.075234369	0.043107	0.007462	-0.00313	0.035006
GPR78	-0.027146883	-0.05872943	-0.03022	0.037294	-0.12595	-0.04819
GPR82	-0.008202724	0.114868365	-0.15882	-0.04224	0.096416	-0.0496
GPR83	-0.036540384	0.019051249	-0.01075	0.012907	-0.01459	-0.07043
GPR84	-0.006773362	-0.0875528	-0.1202	-0.02324	0.071918	0.440748
GPR85	-0.024989269	-0.048274878	-0.02046	-0.01764	0.018178	0.014661
GPR87	-0.032742124	-0.051790065	-0.03429	-0.03365	-0.01983	-0.04942
GPR88	-0.09494917	-0.029883224	-0.05112	-0.02776	0.080906	0.059498
GPR97	-0.005502132	0.000196656	-0.19684	-0.00339	-0.00704	-0.17141
GPR98	-0.08532736	-0.007353596	-0.06257	-0.00937	0.074947	-0.01056
GPRASP1	-0.001809458	-0.017744626	0.431848	-0.105	0.298477	-0.40953

GPRC5A	0.039467368	-0.001947585	-0.03433	0.114034	0.014637	-0.00962
GPRC5B	-0.058636156	-0.078092859	-0.081	-0.01928	0.102887	0.165957
GPRC5C	-0.026460658	-0.110710628	-0.10698	0.026603	-0.08062	0.04767
GPRC5D	-0.028356275	-0.037348459	-0.15469	-0.05977	0.135227	-0.00988
GPRC6A	-0.024828133	-0.041004446	0.073107	0.010262	-0.06964	-0.14563
GPRIN1	-0.034232406	-0.036015907	0.010462	0.072712	-0.20221	0.039827
GPRIN2	-0.026673154	-0.055753598	0.06754	-0.01469	-0.03552	-0.12822
GPRIN3	0.020208785	0.115011747	0.119934	0.062969	-0.14399	-0.06966
GPS1	0.033347765	0.024079206	0.03088	0.055951	-0.15953	0.141455
GPS2	0.062834189	0.054579792	0.153227	0.015464	-0.05127	-0.19448
GPSM1	-0.015181229	-0.035825534	0.020553	-0.03307	0.002711	-0.07306
GPSM2	0.012937443	-0.03903204	-0.12643	0.037706	-0.32038	-0.01379
GPSM3	0.039791465	-0.067037971	-0.01516	-0.03536	0.092757	-0.0235
GPT	-0.024001811	-0.046457835	-0.01784	-0.00443	-0.08176	-0.07936
GPT2	-0.009132401	-0.027505964	-0.09097	-0.01152	-0.00161	0.028208
GPX1	0.02689596	-0.170772195	-0.26092	-0.04953	0.131257	0.509995
GPX2	-0.032362162	-0.047677115	0.023198	-0.02032	0.048091	-0.00333
GPX3	-0.019605938	-0.036247237	-0.05483	-0.02626	0.041393	-0.01675
GPX4	0.06412485	-0.076566628	0.085248	0.020244	-0.02692	0.294759
GPX5	-0.059397655	-0.051503989	0.093928	0.03655	-0.06978	0.01685
GPX7	0.007597552	0.048805644	0.241881	-0.03026	0.093716	-0.3379
GPX8	-0.053410466	-0.023277899	-0.03909	0.003057	-0.00691	-0.07632
GRAMD1A	0.014365231	-0.189875137	0.201955	-0.03644	0.080815	-0.12176
GRAMD1B	0.012294972	-0.110401646	-0.19822	0.075111	-0.24667	0.054267
GRAMD1C	-0.010679714	-0.030141791	-0.13524	-0.02139	0.007074	-0.21864
GRAMD2	-0.030756967	0.04079129	0.113268	0.010274	-0.02745	-0.16698
GRAMD3	-0.004815837	0.078109942	0.164654	-0.04776	0.13026	-0.32612
GRAMD4	0.003676013	0.050903384	-0.24076	-0.04781	0.108233	0.209457
GRAP	-0.007725126	-0.13453607	0.250512	-0.04089	0.121927	-0.19801
GRAP2	0.010013553	0.065072814	0.35442	-0.0974	0.230052	-0.19349
GRAPL	-0.021337251	-0.060610058	0.000626	-0.0135	0.052936	-0.16968
GRASP	0.012190893	-0.111803697	0.022615	-0.01203	-0.01265	-0.13192
GRASPOS	-0.040574792	0.006237255	-0.10052	-0.00976	0.066317	-0.06149
GRB10	0.008148504	-0.065897206	-0.08847	-0.03474	-0.01119	0.085757
GRB14	-0.024232922	-0.087020815	-0.04721	-0.15544	0.462804	-0.09848
GRB2	0.06454817	-0.039908207	-0.36235	-0.00203	0.032663	0.280864
GRB7	-0.028900165	-0.045007823	0.134435	0.014903	-0.07155	-0.04989
GREB1	-0.061742025	-0.027231736	-0.07204	0.182004	0.025533	-0.0676
GREB1L	-0.015166763	-0.067193047	-0.01939	0.015742	0.009688	0.026587
GREM1	-0.047078061	-0.050273564	-0.03878	-0.00729	0.028391	0.0093
GREM2	-0.020549899	0.001145866	-0.02344	0.115416	-0.28011	-0.07101
GRHL1	-0.016679802	-0.034858179	-0.06483	-0.11091	0.284233	-0.08285
GRHL2	-0.042911189	0.000709103	0.049281	-0.00732	0.039327	-0.03439
GRHL3	-0.03590191	-0.036681674	-0.0828	0.024866	-0.0571	0.137167
GRHPR	0.058970651	-0.045337027	-0.08735	0.058116	-0.14499	0.174223
GRIA1	-0.061712483	-0.091043969	-0.0823	0.006949	-0.01065	-0.00877
GRIA2	-0.024232117	-0.089247477	0.038167	0.021613	0.09123	-0.08905
GRIA3	-0.101732653	-0.104659873	-0.06165	-0.11314	0.14279	0.005131
GRIA4	-0.056909731	-0.115302477	-0.05403	0.052136	-0.05062	-0.06229
GRID1	-0.013306633	0.07055357	0.015786	-0.0222	-0.25653	0.011883
GRID1-AS	-0.046955204	-0.096446326	0.04206	-0.01068	0.059539	-0.12956

GRID2	-0.043489288	-0.034221337	-0.1033	0.003342	0.028371	0.339171
GRIK1	-0.051750474	-0.052782576	-0.1117	0.006293	0.003223	0.061506
GRIK1-AS1	-0.031836414	0.018505467	-0.16497	0.012604	-0.00873	-0.01042
GRIK1-AS2	-0.033097095	-0.04590912	0.039996	-0.0037	0.039131	-0.00214
GRIK2	-0.039385299	-0.120856334	-0.00795	-0.00946	-0.09793	-0.02193
GRIK3	-0.062114046	-0.094708996	-0.06906	-0.01192	0.031975	0.057757
GRIK4	-0.029331232	0.127354062	-0.08711	0.016063	-0.01871	-0.01446
GRIK5	-0.041869795	-0.035132957	0.038044	-0.0336	-0.01603	-0.04344
GRIN1	0.003425471	-0.094713032	0.055322	0.032237	-0.05677	0.016965
GRIN2A	-0.063510256	-0.05184133	0.070653	0.013384	-0.10277	-0.07058
GRIN2B	-0.088451962	-0.089586647	0.076082	-0.05776	0.031193	0.077849
GRIN2C	-0.039342707	-0.080070315	-0.00768	-0.01474	0.009449	-0.07206
GRIN2D	-0.036870244	-0.049928161	-0.00443	-0.01891	0.080235	0.169906
GRIN3A	-0.046561855	-0.056467298	-0.05699	-0.00182	0.021487	0.207201
GRIN3B	-0.025564066	-0.066135352	0.049513	0.011546	-0.0142	0.044325
GRINA	0.006856646	-0.128770451	-0.19513	-0.04668	0.098305	0.393931
GRIP1	-0.013305049	-0.050350829	-0.01659	-0.02981	0.10229	-0.05196
GRIP2	-0.016864827	-0.024335501	0.051998	-0.01546	-0.0485	-0.02608
GRIPAP1	0.059672954	-0.028589106	-0.24014	0.041934	-0.15744	0.105355
GRK1	-0.029047056	-0.063215927	-0.0269	0.024198	-0.05252	0.06421
GRK4	-0.038400314	-0.011852611	0.057761	-0.0346	0.038249	0.082693
GRK5	0.044486333	0.066602224	0.030343	-0.08669	0.078566	-0.08548
GRK6	0.040862697	-0.029532541	0.051205	-0.03154	0.108459	-0.06096
GRK7	-0.045560853	-0.000638139	0.157846	-0.01398	0.057041	-0.01815
GRM1	0.028613751	-0.08718589	-0.12266	0.075914	-0.05243	0.044324
GRM2	-0.039190127	-0.025037599	0.059255	-0.00671	-0.00862	-0.106
GRM3	-0.024044707	-0.010122409	-0.02199	0.005371	-0.01511	-0.11023
GRM4	-0.016779013	-0.09813588	0.065627	0.037468	-0.17921	-0.00901
GRM5	-0.048807496	0.000607034	-0.03798	-0.03132	0.043593	0.103154
GRM6	-0.02745098	-0.004345902	0.002078	0.015247	-0.0607	-0.14234
GRM8	-0.089423691	-0.018499695	0.085606	-0.06553	-0.0685	-0.06326
GRN	0.013406669	-0.14636961	-0.40534	-0.05163	0.161314	0.513136
GRP	-0.045934999	-0.070428506	0.075282	0.016458	-0.03174	-0.14119
GRPEL1	0.030847247	-0.027858758	0.020512	0.058961	-0.17588	0.109536
GRPEL2	0.000786396	0.008350271	0.249021	0.073372	-0.20609	-0.1715
GRPR	-0.025133073	-0.076631524	-0.00775	-0.02479	0.002758	0.023516
GRSF1	-0.006215603	0.078619516	0.064823	0.027705	-0.06029	0.147467
GRTP1	-0.070574058	-0.0756595	-0.03953	-0.02541	0.110522	0.136201
GRTP1-AS1	-0.118818685	-0.083426613	-0.02217	-0.02201	0.166542	0.045399
GRWD1	0.011220731	-0.042522074	0.035812	0.033216	-0.16055	-0.1332
GS1-111G1	0.041645072	-0.035019901	0.342338	0.029183	-0.06769	-0.08019
GS1-124K5	-0.020320744	-0.01506195	0.049726	-0.03732	0.083887	-0.09252
GS1-124K5	-0.034881583	-0.023877583	0.006055	-0.01404	0.050957	0.159654
GS1-164F2	-0.002116683	-0.076160492	0.0338	0.077892	-0.22683	0.115898
GS1-166A2	-0.028599784	0.055962272	0.040989	-0.0182	0.077178	-0.07501
GS1-24F4.	-0.056625767	-0.007212556	-0.04566	-0.02141	0.047236	0.041469
GS1-259H1	-0.015839677	-0.022285347	0.124058	0.05487	-0.14049	-0.21167
GS1-279B7	-0.02966259	-0.03782218	0.002408	-0.02457	0.052535	-0.01702
GS1-304P7	-0.049101843	-0.002949015	-0.06714	0.029426	-0.05377	0.002673
GS1-600G8	-0.058568412	-0.119000329	-0.04378	0.024135	-0.03936	-0.00294
GSAP	0.011796005	0.146649486	-0.05858	-0.09082	0.245089	0.134859

GSC	-0.030280031	0.007152089	-0.0235	0.008564	-0.00338	0.071035
GSC2	-0.04727361	-0.017807962	-0.13466	-0.01533	0.016906	0.05656
GSDMB	-0.084960744	-0.077164321	0.180523	0.03973	0.073052	-0.34536
GSDMC	-0.030182036	-0.022826801	-0.0738	-0.00841	-0.02105	-0.03356
GSDMD	0.035392052	-0.022211952	-0.00138	-0.01991	0.013805	0.049997
GSE1	0.023387797	0.163440955	0.052699	-0.00543	0.026454	0.008013
GSG1	-0.058568533	-0.056035448	0.006914	0.013882	0.002381	-0.00743
GSG1L	-0.0223877	-0.090096176	-0.02146	-0.00089	-0.02663	0.008905
GSG2	-0.019430934	-0.011376014	-0.04465	0.19987	-0.56455	-0.01602
GSK3A	0.009986401	-0.062652533	-0.0187	-0.01222	-0.08056	-0.02416
GSK3B	0.020739563	0.055037751	0.116938	-0.01455	0.06547	-0.00652
GSKIP	0.056313781	0.089780805	-0.10765	0.030657	-0.08955	-0.13555
GSN	0.055919796	-0.034653471	-0.17982	-0.01956	0.019676	-0.11829
GSN-AS1	-0.038592593	-0.019560957	0.103331	0.05088	-0.12161	0.041388
GSPT1	0.023228676	-0.010514819	0.318019	-0.01153	-0.07412	-0.1617
GSPT2	0.011136103	0.063807853	0.08535	-0.079	0.228611	-0.21128
GSR	0.03101901	0.102547775	-0.20524	0.058441	-0.18964	0.240192
GSS	0.043506019	0.006762572	0.123629	0.091036	-0.29917	0.155043
GSTA1	-0.029707463	0.072697014	0.085216	-0.0342	0.09684	-0.09555
GSTA3	-0.039958608	0.044767983	-0.05535	-0.0147	0.067934	-0.03614
GSTA4	-0.010963552	0.03815765	0.079403	0.008377	-0.03611	-0.19896
GSTCD	-0.024380639	-0.004263152	0.061054	0.122801	-0.24312	-0.1182
GSTK1	0.045879759	-0.032284709	0.159656	0.001269	-0.01522	0.155316
GSTM1	0.008476311	-0.006150127	0.001855	-0.01214	-0.01874	-0.15602
GSTM2	0.010425002	0.016329463	0.053428	-0.02142	0.010668	-0.18591
GSTM3	-0.010534648	-0.066637851	0.344494	-0.042	0.096123	-0.2008
GSTM4	-0.011652737	-0.013210492	-0.07096	-0.00836	-0.01633	0.013999
GSTM5	-0.024801306	-0.027344453	-0.00283	0.003938	-0.05211	-0.00197
GSTO1	0.057720771	0.027925499	-0.11273	0.028287	-0.07005	0.588074
GSTO2	-0.03538257	-0.017369069	0.050188	-0.00145	0.007221	-0.10093
GSTP1	0.034121178	0.056606599	-0.15601	0.046645	-0.13338	0.524824
GSTT1	-0.007868162	0.001182167	-0.06314	-0.02906	0.099292	0.148699
GSTT2	-0.035304728	-0.068722645	-0.11723	0.004925	0.002605	0.013279
GSTTP1	-0.02375405	-0.060461267	-0.00376	-0.02022	-0.05487	-0.12689
GSTZ1	0.006729308	-0.071396246	-0.17616	0.05005	-0.12249	0.369489
GSX1	-0.048856423	-0.069510922	0.053174	0.017412	-0.03746	-0.05596
GSX2	-0.037115612	-0.079249865	-0.01756	-0.00931	0.031815	0.160651
GTDC1	-0.037551558	0.162241067	0.199935	-0.07969	0.123659	-0.20402
GTF2A1	0.036255785	0.080248123	-0.1018	0.028063	-0.10608	-0.11931
GTF2A1L	-0.058752844	-0.078914135	0.177674	0.030735	-0.08383	-0.10824
GTF2A2	-0.052858625	0.101319865	0.109764	0.098001	0.105633	-0.02013
GTF2B	0.099257669	0.07514011	0.006816	0.054619	-0.10113	-0.07188
GTF2E1	0.032384946	0.147092655	-0.01118	0.064339	-0.2189	-0.04515
GTF2E2	0.046212508	0.056712834	0.003931	0.058812	-0.17876	-0.01723
GTF2F1	0.010604165	0.02197438	0.073062	0.035219	-0.15434	0.338366
GTF2F2	0.007100866	-0.022215799	-0.12662	0.085937	-0.19734	0.255669
GTF2H1	0.025335884	0.035955509	-0.09974	0.001264	-0.11235	-0.02044
GTF2H2B	0.003172714	0.061945615	0.047246	0.034309	-0.07648	-0.06545
GTF2H3	0.036761383	0.064152792	0.164114	0.060959	-0.1565	-0.12047
GTF2H4	0.002626264	-0.007697484	0.094465	0.023629	-0.04322	0.064779
GTF2H5	-0.031804005	0.058913252	0.239538	-0.06425	0.1825	-0.14189

GTF2I	-0.00569895	0.000807356	-0.01084	-0.01428	0.016303	-0.07112
GTF2IRD1	-0.022843941	-0.067994863	0.028786	-0.00548	-0.04678	-0.10861
GTF2IRD2	0.014963049	0.033329365	0.05411	0.01236	-0.02272	-0.04811
GTF2IRD2I	-0.025436604	-0.014872707	0.142573	-0.05729	0.172621	-0.21402
GTF3A	0.050191474	0.086374301	0.247473	0.051994	-0.10444	-0.19457
GTF3C1	0.034276639	0.262262744	0.163054	0.012629	-0.06963	-0.19358
GTF3C2	0.065514522	0.019463773	0.086201	0.040378	-0.05051	0.085014
GTF3C2-AS	-0.04905585	-0.060345448	-0.09077	0.0107	-0.01396	-0.09659
GTF3C3	-0.001440215	0.095048931	0.083998	0.070949	-0.15441	0.072361
GTF3C4	0.010030488	0.147007852	-0.0124	0.072244	-0.20325	0.074098
GTF3C5	0.032776066	0.023877865	-0.02022	0.082715	-0.26496	0.000535
GTF3C6	0.04670797	0.108320787	0.069606	0.044957	-0.1268	0.193141
GTPBP1	-0.035905355	-0.000764436	-0.01596	0.001136	-0.05068	-0.04228
GTPBP10	-0.049241542	-0.000395163	-0.06143	-0.01836	0.058686	0.052428
GTPBP2	-0.01134311	-0.075487232	0.0673	-0.06483	-0.04806	-0.07737
GTPBP3	0.002239022	-0.038187462	0.093595	0.063845	-0.22868	-0.17354
GTPBP4	0.028130397	0.066686361	0.118807	0.058156	-0.13635	-0.02266
GTPBP6	0.031014469	0.019109316	0.123716	-0.00801	0.026347	0.086821
GTPBP8	0.02644725	0.094806046	0.133784	0.09078	-0.29343	-0.12106
GTSE1	-0.021976148	0.05734066	0.011236	0.125545	-0.3987	-0.05979
GTSE1-AS	-0.040670282	0.024414192	0.143461	0.035259	-0.0679	0.007585
GTSF1	0.002548304	0.003550128	0.145875	0.020996	-0.05194	-0.08913
GTSF1L	-0.034503139	-0.070559403	0.043461	-0.03558	0.061036	-0.19943
GUCA1A	-0.051241594	-0.097811929	-0.01169	0.019407	-0.05827	-0.12335
GUCA1B	-0.037753354	-0.069850199	0.141393	0.024647	-0.05526	-0.1576
GUCA1C	-0.061335852	0.011858972	0.000657	0.002114	-0.00037	0.030808
GUCA2A	-0.028280126	-0.075635819	0.105629	0.016277	-0.07204	0.003427
GUCA2B	-0.044503021	-0.02158591	-0.01208	-0.00898	0.060246	0.101744
GUCD1	0.043824283	-0.031086265	-0.32084	0.027351	-0.06626	-0.10166
GUCY1A2	-0.094903001	-0.056204333	0.008524	-0.01646	-0.00411	0.019076
GUCY1A3	-0.017612388	0.066490752	-0.12186	-0.15079	0.406849	-0.14592
GUCY1B2	-0.035565167	-0.014999202	-0.05859	-0.00615	0.037083	0.015094
GUCY1B3	-0.006304646	0.089216631	-0.12612	-0.19458	0.556249	0.015437
GUCY2C	-0.033176793	-0.110742042	-0.20929	-0.01883	0.085812	0.107211
GUCY2D	-0.010719751	-0.093267063	-0.15343	0.000622	0.021953	-0.06111
GUCY2F	-0.038659139	-0.066490893	-0.06163	0.007594	0.010297	0.045066
GUF1	0.005176709	0.031331947	0.071471	0.082693	-0.18303	0.107858
GUK1	0.044995558	0.054170413	0.103318	0.103622	-0.24857	-0.02982
GULP1	-0.045993821	-0.0479974	-0.01344	0.23258	-0.13567	-0.05212
GUSB	0.045238326	0.03416058	-0.17982	0.049891	-0.13392	0.337875
GUSBP1	-0.030252217	-0.086691884	0.072689	0.03993	-0.10377	-0.0459
GUSBP11	-0.019628487	-0.067237172	0.057257	-0.01335	-0.10596	-0.02016
GUSBP2	-0.017705978	0.058178177	0.195296	0.011032	-0.00174	-0.24239
GUSBP5	-0.01805428	-0.013711969	0.112843	-0.01299	0.024459	-0.04856
GVINP1	0.00091508	0.12898249	0.154635	-0.0198	0.041212	-0.24345
GVQW1	-0.028831504	0.007480736	-0.03824	0.019196	-0.06831	-0.04596
GXYLT1	0.006463789	0.107864424	-0.00554	0.014669	-0.01872	0.04546
GXYLT2	-0.039429812	-0.11395141	-0.03044	0.007775	0.002788	-0.1272
GYG1	0.053429208	0.135706385	-0.06517	0.082636	-0.22535	0.281988
GYG2	-0.085781485	-0.155680354	0.06323	-0.03674	-0.10453	-0.03194
GYLTL1B	-0.008871316	-0.117153042	0.017663	-0.00759	-0.00674	-0.26218

GYPA	0.027089716	-0.038991237	-0.03919	0.091386	0.061152	-0.16665
GYPB	-0.024673111	-0.062388815	0.037384	-0.0097	-0.01728	-0.09595
GYPC	0.026151337	0.062068217	0.342104	0.017591	-0.05815	-0.19445
GYPE	-0.090016184	-0.055828525	-0.00691	-0.03906	0.182049	-0.0757
GYS1	0.015098211	-0.005066013	0.034692	0.053446	-0.15901	0.05254
GYS2	-0.074438756	-0.038639115	0.131049	-0.05555	0.12806	0.009461
GZF1	0.009187153	0.032189907	-0.14785	0.062817	-0.13938	0.111624
GZMA	0.01276675	0.263056847	0.4644	0.012888	-0.04098	-0.26979
GZMB	0.011871578	0.195565084	0.193823	0.032101	-0.08779	-0.27415
GZMH	0.009023996	0.230229707	0.47358	-0.00481	0.021794	-0.24084
GZMK	0.005017175	0.087574732	0.578339	0.004252	0.001839	-0.26894
GZMM	0.007649635	0.270962482	0.448362	0.004936	-0.03101	-0.29472
H1F0	-0.002251696	-0.142690746	-0.15245	-0.04817	0.123314	0.316564
H1FNT	-0.051153174	-0.042181212	-0.20031	0.006079	-0.05601	0.067961
H1FOO	-0.027481589	-0.013925612	-0.07776	0.009857	-0.02641	-0.12664
H1FX	0.017920382	-0.051453216	0.109607	0.003973	-0.01462	0.040266
H2AFJ	0.022451341	-0.006519364	-0.03583	-0.11425	0.325914	0.214213
H2AFV	0.019335081	0.073809775	0.183489	0.118614	-0.08873	-0.23119
H2AFX	-0.023693545	-0.056810438	0.090096	0.112217	-0.28318	-0.06523
H2AFY	0.037837403	-0.127245125	-0.20324	-0.06987	0.048827	0.493547
H2AFY2	-0.010276221	-0.144975774	0.278315	-0.02229	-0.00562	-0.10005
H2AFZ	0.070743411	0.083562248	-0.02293	0.149286	-0.3446	-0.09242
H2BFM	-0.036687332	0.000133376	########	0.07788	-0.19293	0.087531
H2BFS	0.029302912	-0.03916723	-0.36605	-0.0407	0.112653	-0.11419
H2BFXP	-0.01180624	0.024257783	0.19971	0.071925	-0.18089	-0.2442
H3F3A	0.025295974	-0.056560558	-0.14696	-0.02555	0.04272	0.071053
H6PD	0.030043336	-0.04055742	0.041877	0.024595	-0.13635	0.192674
HAAO	-0.015483543	-0.136985455	-0.03843	-0.02927	-0.00652	0.161769
HAB1	-0.005790591	-0.112370483	-0.043	-0.01135	-0.06565	-0.14002
HABP2	-0.033300604	-0.046948132	-0.12638	0.022012	-0.06342	0.005152
HABP4	-0.003138969	-0.05361219	0.360288	0.017812	-0.05004	-0.23706
HACE1	-0.008356364	0.042318291	0.079692	0.007726	-0.09292	-0.10353
HACL1	0.035086537	0.064454201	0.116341	0.040619	-0.11932	-0.06543
HADH	0.008605961	0.024981265	0.120609	0.057489	-0.14949	-0.15127
HADHA	0.029922852	-0.061976388	0.013089	-0.01482	-0.07899	0.416652
HADHB	0.094986485	-0.044172183	-0.21977	0.011961	########	0.3453
HAGH	0.046821063	-0.028188716	0.055503	0.043628	-0.15298	-0.04383
HAGHL	-0.001793109	-0.067752949	0.145908	0.018376	-0.14055	-0.15957
HAGLR	-0.035799261	-0.062402334	-0.08517	-0.06359	-0.00947	0.091923
HAGLROS	-0.034989022	-0.057340399	-0.03366	0.011428	0.001378	0.104612
HAL	0.008449441	-0.069455251	-0.30214	-0.08341	0.174615	0.47496
HAMP	-0.020399454	-0.071408637	-0.13861	0.025366	-0.08518	0.009789
HAND1	-0.026494658	-0.055079864	-0.01748	0.03765	-0.12113	-0.02294
HAND2	-0.020730831	-0.013455657	0.035376	0.014044	-0.00777	0.009618
HAND2-AS1	-0.072127735	-0.080730998	-0.01846	0.014432	-0.03141	0.047789
HAO1	-0.044184708	-0.012969321	-0.02822	0.016691	-0.03054	-0.01785
HAO2	-0.052432039	-0.021884134	-0.07768	0.018288	-0.02538	0.019498
HAP1	-0.025687317	-0.041339105	-0.02591	-0.02694	0.008596	-0.08326
HAPLN1	-0.106140544	-0.076848359	0.116071	0.026125	-0.03883	-0.06143
HAPLN2	-0.028035185	-0.05279664	-0.08416	0.020462	-0.03515	0.115306
HAPLN3	0.003635218	-0.101237819	0.230259	0.020606	-0.14994	-0.17027

HAPLN4	-0.031721316	0.008232443	-0.07703	0.007958	-0.03866	-0.01624
HAR1A	-0.012776271	0.05577302	0.40231	-0.06582	0.209655	-0.22461
HARBI1	-0.011811901	0.017058227	0.025734	0.032592	-0.05494	0.080009
HARS2	0.068962184	0.094549101	0.012697	0.023078	-0.08072	-0.3066
HAS1	-0.019388595	-0.024224781	0.006955	0.005926	-0.017	-0.02139
HAS2	-0.067003034	-0.069482328	0.119958	-0.01171	0.048385	-0.12512
HAS3	-0.041878574	-0.044292618	0.038074	0.015853	-0.02017	0.097508
HAT1	0.061325082	0.184664904	-0.10539	0.108547	-0.28267	0.103712
HAUS1	0.019896692	0.035027693	0.082066	0.035092	-0.09048	0.139236
HAUS2	0.008037325	0.058812608	0.012264	0.09091	-0.27251	0.191444
HAUS3	0.032689333	0.055319901	0.281418	0.032893	-0.10744	-0.34286
HAUS5	-0.004635759	-0.06792717	0.075196	0.067921	-0.27894	-0.15128
HAUS6	0.00011405	0.141736674	0.184923	0.085413	-0.19599	-0.15178
HAUS7	0.014879518	0.003151472	-0.10019	0.053671	-0.12458	0.374708
HAUS8	-0.00030292	-0.036541734	-0.06392	0.101501	-0.2551	0.098698
HAVCR1	-0.033164033	0.060770595	0.101982	-0.01899	0.072471	-0.09527
HAVCR1P1	-0.025346952	-0.048925722	0.003662	-0.00062	-0.07946	0.029342
HAVCR2	0.006675728	0.229884017	-0.19759	0.026059	-0.10442	0.222877
HAX1	0.044289927	-0.032323249	0.220471	0.084262	-0.22504	-0.18307
HBB	0.007093633	0.026343194	-0.10627	-0.12584	0.330657	0.002958
HBBP1	-0.033525699	-0.068659454	-0.12428	0.006535	0.002138	-0.04992
HBD	-0.003674261	-0.027605847	-0.06961	-0.01505	0.040815	-0.11905
HBE1	-0.042363141	-0.091238728	-0.08384	-0.02096	0.061237	-0.01879
HBEGF	-0.00726282	-0.030244055	-0.04985	-0.01184	-0.04471	0.103166
HBM	-0.022686169	-0.071425799	-0.11903	-0.03242	0.110931	0.087915
HBP1	-0.020170503	-0.023681609	0.062444	-0.03062	0.142387	0.068661
HBQ1	-0.017387093	0.114180767	-0.10516	-0.14693	0.414752	-0.01322
HBS1L	0.003844537	0.018047439	0.1659	0.1047	-0.21865	-0.08335
HBZ	-0.033500713	-0.017984356	0.002076	-0.00404	0.038667	0.02404
HCAR1	-0.020689677	-0.029281032	-0.0344	9.12E-05	-0.02039	-0.0679
HCAR3	0.003549981	-0.060272944	-0.25601	-0.04347	0.114878	0.13687
HCCAT5	-0.03110812	-0.015635824	-0.08033	-0.01345	-0.02101	-0.05958
HCCS	0.025795053	-0.021597441	-0.27144	0.068613	-0.21234	0.302848
HCFC1	-0.012396423	-0.115123832	-0.02046	0.03544	-0.17441	0.005991
HCFC1R1	0.037210125	-0.056085452	-0.06204	0.033583	-0.11697	0.27458
HCFC2	0.011885976	0.112260257	-0.02115	-0.01497	0.06461	0.137908
HCG11	-0.015222717	0.038236576	-0.00711	-0.03352	0.121499	0.344433
HCG18	-0.051371615	0.143626713	0.225379	-0.01346	-0.05081	-0.13316
HCG22	-0.029417645	-0.072812828	-0.01509	-0.01299	-0.04498	-0.09757
HCG26	0.015079361	0.058117946	0.103743	-0.02082	0.005437	-0.16758
HCG27	-0.007577161	-0.10869025	-0.13217	-0.03349	0.072618	-0.05482
HCG4	-0.036105364	-0.046110639	0.17119	-0.01329	0.044468	-0.00754
HCG4B	-0.031272485	-0.04963623	-0.00231	0.016283	-0.03977	-0.1347
HCG9	-0.038072171	-0.040730593	-0.03179	0.026496	-0.10783	-0.12307
HCK	0.01005322	-0.171819321	-0.44414	-0.0972	0.285766	0.385416
HCLS1	0.054027533	0.038751118	-0.17933	0.020231	-0.04058	-0.01411
HCN1	-0.069356061	-0.066172966	-0.0084	-0.00366	0.01383	-0.07582
HCN2	-0.037148794	-0.068921702	-0.11344	-0.01747	0.004638	0.005764
HCN3	-0.027289177	-0.056840234	########	0.001235	-0.06734	-0.08525
HCN4	-0.047124849	-0.091152185	-0.01699	0.037601	-0.09249	-0.07016
HCP5	0.037084542	0.190559105	0.105563	0.03255	-0.1063	-0.25826

HCP5B	-0.033978836	-0.021142657	0.059707	-0.0043	-0.0192	-0.17772
HCRP1	0.007218472	-0.003516235	-0.01554	0.010599	-0.05552	-0.10489
HCRT	-0.031085491	-0.055206983	0.009494	-0.00337	0.020803	0.05206
HCRTR1	-0.042773515	-0.06774476	0.026561	0.001188	0.013401	-0.00491
HCRTR2	-0.032342674	-0.055331218	-0.04183	-0.01449	0.055584	-0.03028
HCST	0.02849692	0.170394906	0.347905	0.00418	-0.01978	0.241188
HDAC1	0.067342786	0.105053501	0.126021	0.043191	-0.10613	-0.31844
HDAC10	-0.01995954	-0.085480098	-0.0066	-0.00388	-0.08749	-0.10908
HDAC11	-0.029372765	-0.104594101	0.11032	-0.06871	0.093788	0.081735
HDAC2	0.02557481	0.007813523	-0.03165	0.071882	-0.22278	4.39E-05
HDAC3	0.049105703	-0.03321003	-0.02785	0.051492	-0.14233	-0.04424
HDAC4	-0.003494829	0.039871363	-0.11525	0.005788	0.034836	0.295093
HDAC5	0.001212463	-0.145096707	-0.04666	-0.0616	0.098367	-0.03414
HDAC6	-0.008967736	-0.095274717	0.109995	-0.02088	-0.02192	-0.12521
HDAC7	0.002336422	0.027208512	0.16265	-0.01759	0.004044	-0.12138
HDAC8	-0.018999016	-0.013524937	-0.05694	0.070576	-0.16066	0.276916
HDAC9	-0.000882251	0.001139624	-0.23527	0.05214	-0.06159	0.062301
HDC	-0.005443596	-0.048866997	-0.05205	-0.02916	0.072867	-0.1318
HDDC2	0.016465218	0.192617877	0.159347	0.040166	-0.09389	0.020973
HDDC3	0.022116086	-0.018506524	0.143732	0.032766	-0.13437	-0.01393
HDGF	0.09864204	0.023051492	-0.13961	0.09064	-0.21057	0.333937
HDGFL1	-0.005748134	-0.051810289	0.033721	-0.01978	-0.05673	-0.08185
HDGFRP2	0.025626576	-0.044703169	-0.08227	0.039877	-0.15295	-0.03581
HDGFRP3	-0.022567533	0.154896767	-0.04359	0.190018	-0.43992	-0.06759
HDHD1	0.022491806	-0.046954644	-0.14016	0.039376	-0.11871	0.485421
HDHD2	0.027281949	0.056989743	0.109063	0.000691	-0.00302	0.088189
HDHD3	0.003501463	-0.075881996	0.18083	0.020991	-0.03429	-0.15915
HDLBP	0.033724125	-0.048833502	0.028951	0.008785	-0.01091	0.137587
HDX	-0.03438331	-0.038821786	-0.12921	-0.08224	0.218643	0.298574
HEATR1	0.017788675	0.022656383	0.119747	0.052807	-0.13739	-0.08203
HEATR2	0.01477823	-0.04345517	0.114977	0.078343	-0.21092	0.014752
HEATR3	0.014137355	0.022992255	-0.03247	-0.00275	0.025263	0.436471
HEATR4	-0.037544742	0.010992004	0.03208	-0.01842	0.078565	0.011706
HEATR5A	0.006034032	-0.0178835	-0.23937	-0.0229	0.068223	0.257378
HEATR5B	0.025652158	0.015273614	0.00392	-0.01062	0.042493	-0.24321
HEATR6	0.035370283	-0.006419109	-0.05792	-0.03269	-0.00134	-0.004
HEATR9	-0.009712618	0.17353012	0.1363	0.014248	-0.02174	-0.08367
HEBP1	0.018267529	-0.0584499	-0.16508	-0.12539	0.332911	0.433109
HEBP2	0.029428572	-0.124767588	-0.18397	-0.0178	0.020348	0.280756
HECA	0.016636696	-0.100301312	0.144139	-0.09687	0.277038	-0.1317
HECTD1	0.012177148	0.03672851	0.03856	0.008459	-0.01097	0.140712
HECTD2	-0.024944519	-0.03079256	0.012317	-0.03957	0.010679	-0.04058
HECTD3	0.029168883	-0.009918251	-0.00799	-0.04687	0.105329	0.073158
HECTD4	-0.064902021	0.068447662	0.019698	-0.03434	-0.06079	0.004502
HECW1	-0.032426312	-0.05257215	0.096311	3.05E-05	-0.00457	-0.06302
HECW1-IT	-0.103641813	-0.113607793	-0.00536	-0.0124	0.02203	0.139231
HECW2	-0.062425099	-0.072298998	-0.02083	0.029371	-0.16226	-0.10122
HEG1	0.011941063	0.238555211	0.109632	0.010179	-0.06461	-0.04536
HEIH	0.044467108	-0.035593509	0.144985	-0.03834	0.07893	0.195514
HELB	-0.059619696	0.01819111	0.211876	-0.00213	-0.05994	-0.17643
HELLS	0.010054411	-0.013291606	0.112293	0.16598	-0.27479	-0.18512

HELQ	-0.062518107	0.051913015	0.15203	-0.02519	-0.01384	0.06146
HELZ	0.009948744	0.244864416	0.027097	0.01193	0.034067	0.101188
HELZ2	0.002883328	-0.03310287	-0.03982	-0.10332	0.075056	0.001762
HEMGN	-0.015653454	-0.056670611	0.286178	-0.17422	0.505989	-0.11787
HEMK1	0.014639642	-0.020360677	0.284924	0.023403	-0.09714	-0.2502
HENMT1	0.02638815	0.22698908	0.280868	0.040255	-0.12188	-0.14079
HEPACAM2	-0.028297107	-0.096814045	-0.02618	-0.0091	0.002129	0.035969
HEPH	-0.058867395	-0.048800302	0.095259	0.08672	-0.16534	-0.00332
HERC1	-0.008542633	0.022563814	0.102615	-0.00267	0.034859	-0.11808
HERC2	0.018406639	0.046513842	0.096276	-0.00398	0.02388	-0.19894
HERC2P7	-0.026380516	-0.027406755	0.006032	0.005872	-0.0456	-0.0516
HERC4	-0.035686712	0.016309584	0.057049	0.00794	0.018949	-0.05149
HERC5	0.019796693	0.035237939	-0.09224	-0.00508	0.033105	-0.00595
HERC6	0.002171972	0.039973096	0.132592	-0.0418	0.114025	-0.02109
HERPUD1	0.044377876	-0.071958102	-0.1692	-0.00858	-0.0058	-0.0361
HERPUD2	0.023187171	0.145036426	0.196515	-0.04742	0.160786	-0.21939
HES1	-0.040689858	-0.057834457	-0.13265	-0.03046	0.053384	0.08005
HES2	-0.029704933	-0.040715721	0.001103	-0.03971	0.062356	0.086542
HES4	-0.0102104	-0.055163542	-0.12826	-0.03877	0.087066	0.109156
HES5	-0.038228362	-0.062027463	-0.02322	0.008914	0.008394	0.166383
HES6	0.001566117	0.180339623	-0.06294	-0.009	-0.0293	-0.07621
HES7	-0.024993762	-0.054899788	0.117231	0.013728	-0.03231	0.182582
HESX1	-0.021385594	-0.07090026	-0.05117	0.011776	-0.02072	0.022075
HEXA	0.000297625	0.014469707	0.085388	-0.04106	0.138289	-0.07326
HEXA-AS1	-0.056544211	-0.005689343	0.005223	-0.02061	0.085109	-0.0272
HEXB	0.051202373	0.004573809	-0.30815	-0.02505	0.07337	0.456302
HEXDC	0.004798746	-0.020325355	0.11446	-0.02752	0.040308	-0.15721
HEXIM1	0.041470887	0.166375519	-0.03948	-0.01704	0.043891	-0.11964
HEXIM2	-0.005941522	0.050932608	-0.05679	-0.04716	0.152256	0.238388
HEY1	-0.014588515	-0.04236941	-0.1293	0.025285	-0.12267	-0.14074
HEY2	-0.025032469	0.058733332	-0.01943	-0.02529	0.037926	-0.12704
HEYL	-0.03377807	-0.112758633	-0.05766	-0.00652	-0.04936	-0.03984
HFE	-0.03261846	-0.096753282	-0.05442	0.040657	0.081909	0.320384
HFE2	-0.042836962	-0.051971047	-0.00361	0.002715	0.015764	0.045793
HFM1	-0.054880274	-0.063352877	-0.02438	0.005012	0.003428	-0.06793
HGC6.3	-0.029487629	-0.097821879	-0.02022	0.00057	0.011383	0.072779
HGD	-0.035207255	-0.069503748	0.005322	-0.17038	0.823342	-0.02149
HGF	-0.052407604	-0.108475762	-0.20598	-0.08785	0.231903	0.703655
HGFAC	-0.038580417	-0.02611689	-0.02187	0.012826	0.008023	0.183138
HGH1	0.00270918	-0.027090963	0.100987	0.034601	-0.19003	-0.14766
HGS	0.017270741	-0.003258442	-0.08893	-0.01278	0.034715	0.053536
HGSNAT	0.007637235	-0.09040534	-0.1605	-0.01468	0.123055	0.257991
HHAT	-0.004877093	-0.125361178	0.039242	-0.01076	0.052541	-0.06437
HHATL	-0.031890839	-0.032485414	-0.0274	0.019882	-0.0364	-0.01089
HHEX	-0.001418967	-0.08442471	-0.49893	-0.12349	0.337943	0.243903
HHIP	-0.040703659	-0.002680701	-0.10221	0.039268	-0.04936	-0.03028
HHIP-AS1	-0.013207739	-0.10258094	0.008466	-0.00514	0.001224	-0.20783
HHIPL1	-0.030421956	-0.041986694	0.01852	0.024897	-0.071	-0.04718
HHIPL2	-0.029663037	-0.038248457	0.046921	-0.0287	0.069828	-0.0709
HHLA1	-0.026228514	-0.067227802	-0.03765	-0.01815	-0.02782	-0.0037
HHLA2	-0.039875337	-0.060294167	0.032072	0.140568	-0.25283	0.033368

HHLA3	-0.038623565	-0.054563753	0.218656	0.144294	-0.14658	-0.15932
HIAT1	0.06914497	0.099380445	-0.05046	0.04268	-0.09751	0.006043
HIATL1	0.031803203	-0.0078669	-0.27063	0.01648	-0.0377	0.454202
HIATL2	0.019926903	-0.118832569	0.027685	-0.00453	-0.07126	0.132629
HIBADH	-0.023909208	0.05520686	0.14488	0.124592	-0.11009	0.068112
HIBCH	0.009804819	0.053549215	0.072824	0.031276	-0.12816	-0.17862
HIC1	0.014165459	0.055323652	-0.08342	0.061356	-0.26311	0.133511
HIC2	-0.024280867	-0.030616856	0.187637	-0.02459	0.061732	0.110256
HID1	-0.04098148	-0.106116481	0.019751	0.00925	-0.06487	-0.05561
HIF1A	0.04471535	-0.040225967	-0.2942	0.059966	-0.12769	0.014922
HIF1AN	0.008839147	0.049977248	-0.07617	0.02467	-0.19559	0.044806
HIF3A	-0.052660954	-0.089174043	-0.00376	-0.02592	-0.00556	0.064054
HIGD1A	0.070198286	0.063071219	-0.06798	0.081015	-0.20295	-0.10895
HIGD1B	-0.034564678	-0.058926098	-0.07493	0.034787	-0.11774	0.197394
HIGD2A	0.039485427	-0.054526406	0.151344	0.013731	-0.04703	0.276373
HILPDA	-0.009731888	-0.02836558	-0.06258	0.086757	-0.28693	-0.01379
HILS1	-0.031667945	-0.064141404	-0.04751	0.00949	-0.00051	0.000519
HINFP	0.019385816	-0.002630656	0.077625	-0.01905	-0.03044	-0.06218
HINT1	0.048989223	0.016757769	0.235559	0.078512	-0.16248	-0.12633
HINT2	0.026109813	0.033312145	0.05348	0.054375	-0.16124	0.004748
HINT3	-0.027960445	0.01983477	-0.22551	0.067534	0.075444	0.324818
HIP1	-0.008487608	0.000301038	-0.32845	-0.03612	0.055631	0.035603
HIP1R	-0.030181023	-0.022357099	0.109579	-0.04722	0.031775	-0.30592
HIPK1	0.0102749	0.122289544	0.058891	-0.08229	0.205043	-0.03203
HIPK1-AS1	-0.086388631	-0.044336933	0.135191	-0.02472	0.0655	0.069541
HIPK2	-0.004078743	0.289483765	-0.10002	0.01446	-0.11354	0.111931
HIPK3	-0.020495958	-0.035298399	-0.1489	-0.08369	0.152596	0.22001
HIPK4	-0.003348701	-0.086495828	-0.0449	-0.01648	-0.07662	-0.06166
HIRA	0.009471904	0.210352809	0.052803	-0.01707	0.022754	-0.24507
HIRIP3	0.010465558	0.048323997	0.158623	0.076371	-0.23678	-0.23708
HIST1H1A	-0.036722659	-0.04710472	-0.01375	-0.00386	0.043033	-0.13359
HIST1H1B	-0.024878677	-0.020992585	-0.04356	0.049901	-0.1266	-0.09748
HIST1H1C	0.012488714	-0.057699937	-0.08014	0.01923	-0.08175	-0.22832
HIST1H1D	0.00017263	0.023453634	0.157997	0.038142	-0.08702	-0.22911
HIST1H1E	-0.003898348	-0.002167353	-0.01366	0.030268	-0.05961	0.063545
HIST1H1T	-0.041910245	-0.052099848	0.015712	-0.02128	0.04657	-0.0525
HIST1H2AI	-0.031307859	0.009907987	-0.1193	0.005319	-0.01693	0.002263
HIST1H2A(	0.018220902	-0.058352654	-0.26186	-0.08858	0.236534	-0.12968
HIST1H2AI	-0.009435936	-0.021371383	-0.06923	-0.1432	0.396551	-0.04792
HIST1H2A\	0.008683017	-0.050127125	-0.13036	-0.01229	-0.01458	-0.04128
HIST1H2AI	-0.04740054	-0.029019135	-0.03764	-0.03072	0.116534	-0.07865
HIST1H2AI	-0.024032131	-0.036043592	-0.08489	0.033209	-0.09152	0.054413
HIST1H2AM	-0.018005715	-0.006663515	-0.0162	-0.02778	0.087322	-0.0483
HIST1H2BA	-0.058635263	-0.037482916	-0.12245	-0.0329	0.081855	-0.05042
HIST1H2BI	-0.024763619	-0.036210713	-0.10096	-0.00131	0.032175	-0.05777
HIST1H2B(	-0.005027514	-0.062630733	-0.1616	-0.09473	0.271548	-0.10813
HIST1H2BI	0.013886019	-0.027824416	-0.24473	-0.06041	0.137974	-0.23604
HIST1H2BI	0.008711789	-0.048939393	-0.26043	-0.05937	0.151749	-0.17061
HIST1H2BI	-0.005490202	-0.039662956	-0.31022	-0.07774	0.204434	-0.13731
HIST1H2B\	-0.02749539	-0.025036964	-0.07778	-0.15984	0.485285	0.143687
HIST1H2BI	0.047825649	-0.015180504	-0.38279	-0.03554	0.115269	-0.1223

HIST1H2BI	-0.032904157	-0.054762578	0.005688	-0.01102	0.050422	0.094409
HIST1H2BM	-0.037314155	-0.027585796	-0.13652	0.030426	-0.07678	0.10136
HIST1H2BN	-0.03132763	-0.031596417	0.001948	-0.02658	0.092104	0.043417
HIST1H2BO	-0.025454382	-0.04521133	0.001311	-0.14105	0.413999	-0.06297
HIST1H3A	-0.019157667	0.065309966	-0.02481	0.001853	0.009165	-0.02582
HIST1H3B	-0.028664792	-0.028476731	-0.10418	0.024931	-0.04613	0.10326
HIST1H3C	-0.026391743	-0.008777989	-0.20876	0.054584	-0.13614	0.176456
HIST1H3E	-0.023104374	-0.044750512	-0.06576	0.004555	-0.01365	-0.05825
HIST1H3F	-0.038602583	0.051380492	-0.04552	0.03221	-0.09844	0.136034
HIST1H3G	-0.017264084	-0.054257094	-0.08493	-0.06597	0.135151	-0.02771
HIST1H3I	-0.022564214	0.034439369	-0.00161	0.03778	-0.09467	-0.00896
HIST1H4A	-0.031161528	-0.052821053	0.040628	0.027484	-0.05552	-0.00211
HIST1H4D	-0.030306542	-0.09381226	-0.02366	0.052172	-0.14105	-0.03612
HIST1H4E	-0.016227997	-0.028909479	-0.00111	0.005425	-0.05148	0.001728
HIST1H4G	-0.033908025	-0.036916697	-0.02693	0.014187	-0.02768	0.043752
HIST1H4H	-0.013752113	-0.001262731	-0.10316	-0.08943	0.246843	-0.12369
HIST1H4I	-0.026093518	-0.076434147	-0.03762	-0.01342	0.010547	0.089255
HIST1H4J	0.004065623	0.053831985	0.253742	0.047635	-0.17236	-0.27382
HIST1H4L	-0.032408584	-0.050340331	-0.09471	0.035369	-0.07337	-0.01287
HIST2H2BI	0.006692769	-0.089785327	-0.18739	-0.1159	0.322258	-0.02653
HIST3H2A	0.012206391	0.052799847	0.162102	0.074973	-0.21958	-0.13061
HIST3H3	-0.030616008	-0.061967843	-0.12423	-0.06301	0.194641	0.090839
HIVEP1	0.003371573	-0.062079961	-0.10408	0.028759	-0.07438	-0.03506
HIVEP2	0.009613889	-0.02658966	0.270342	-0.0173	0.025273	-0.38115
HIVEP3	0.026713287	0.05043989	-0.00775	-0.02505	0.032294	0.222304
HJURP	-0.014890138	-0.022720101	-0.00996	0.156397	-0.38606	-0.03952
HK1	0.050534577	0.001256629	0.045889	0.021315	-0.05344	0.462171
HK2	0.009528746	-0.187406597	-0.24476	0.010926	-0.0355	0.357094
HK3	0.003644051	-0.100496021	-0.26726	-0.08845	0.23377	0.539843
HKDC1	0.006777431	-0.091688539	0.284688	0.002955	0.095281	-0.18169
HKR1	-0.037689927	-0.010843407	-0.09117	0.009608	0.003932	-0.0233
HLA-A	0.08324016	0.244935169	-0.22297	0.120132	-0.04227	-0.06831
HLA-B	0.04653534	0.184119321	-0.15543	0.072988	-0.05328	-0.06909
HLA-C	0.084465082	0.205735023	-0.24055	0.097005	-0.02206	-0.11956
HLA-DMA	0.023302269	-0.152800275	-0.25685	0.008851	-0.01833	0.28556
HLA-DMB	0.023468707	-0.158187518	-0.34519	-0.00816	0.023037	0.299292
HLA-DOA	-0.004821449	-0.090733526	-0.02259	0.028088	-0.17532	-0.0518
HLA-DOB	0.000138075	-0.071166132	-0.14342	-0.01711	0.038916	-0.23222
HLA-DPA1	0.022226782	-0.095353412	-0.18094	0.018486	-0.03336	0.282819
HLA-DPB1	0.016800851	-0.099936038	-0.1332	0.004276	-0.00033	0.199604
HLA-DPB2	-0.002314239	-0.074120012	-0.1483	0.042668	-0.09187	-0.15064
HLA-DQA1	0.011423474	-0.10506906	-0.1387	-0.02639	0.067356	0.021425
HLA-DQB1	0.007907424	-0.120412629	-0.22684	0.032749	0.013116	0.165567
HLA-DQB2	-0.02534565	-0.114887966	-0.06265	0.029072	-0.12439	-0.07981
HLA-DRA	0.019415887	-0.223097774	-0.33079	0.009992	-0.02217	0.308134
HLA-DRB4	-0.00224155	-0.101631674	-0.22747	-0.03008	0.094514	0.237509
HLA-DRB6	0.014935067	-0.208981266	-0.27429	0.00657	-0.01857	0.278724
HLA-E	0.056155111	0.121728305	-0.08041	-0.00181	0.189322	-0.27231
HLA-F	0.0599767	0.153538589	0.029432	0.029692	-0.06243	-0.20011
HLA-F-AS1	0.007665365	0.042205022	0.275715	0.007365	-0.01722	-0.07992
HLA-G	0.063979638	0.155435709	-0.01572	0.040336	-0.0698	-0.19518

HLA-J	0.068621357	0.143621734	0.022647	0.042717	-0.06696	-0.21888
HLCS	-0.015908208	0.127405828	0.173853	0.075459	-0.19443	-0.30427
HLF	-0.000743568	0.008949693	0.01173	0.045777	-0.19305	-0.01111
HLTF	0.01215668	0.093063588	0.171598	0.025345	-0.05752	-0.30218
HLX	0.002971599	-0.108870158	-0.2375	-0.03172	0.08357	0.209805
HM13	-0.074122307	-0.008132673	-0.07827	-0.07674	0.061352	0.166456
HMBOX1	0.032998187	-0.036147386	0.077558	-0.03199	0.082932	-0.10719
HMBS	0.017748729	-0.115682975	0.034121	0.081197	-0.25275	0.127514
HMCES	0.020186245	0.041732223	0.326802	0.009573	-0.04714	-0.47243
HMCN1	-0.079210841	-0.061549691	0.002793	0.03392	-0.00054	0.014204
HMCN2	-0.077154834	-0.032606208	0.173823	-0.01117	-0.08978	-0.14848
HMG20A	0.025610337	0.102653858	0.201637	0.018652	-0.05137	-0.15931
HMG20B	0.003000627	0.014002547	0.031515	-0.00384	0.006575	0.143747
HMGA1	0.016353427	-0.14442581	0.146574	0.086447	-0.26622	-0.104
HMGA2	-0.038799446	-0.090019038	-0.05038	0.066203	0.016087	-0.00405
HMGB1	0.131827686	0.19186422	0.073148	0.096035	-0.14302	-0.34212
HMGB2	0.030972865	0.001929443	-0.01895	0.062967	-0.1636	0.145449
HMGB3	-0.01232327	-0.03904779	-0.04936	0.091675	-0.35411	0.055977
HMGB3P1	-0.014875835	-0.018797919	0.042427	0.10373	-0.37684	-0.0009
HMGB4	-0.033588758	-0.049588256	0.006334	-0.00333	0.038195	0.101539
HMGCL	0.038762283	0.012974662	-0.04951	0.016938	-0.08815	0.075448
HMGCLL1	-0.09826271	-0.095769429	0.013974	-0.04932	0.085768	-0.05708
HMGCR	0.024523544	0.097288715	-0.32395	0.037766	-0.09159	0.245516
HMGCS1	0.002611952	0.03917107	-0.08017	0.044367	-0.15561	-0.01331
HMGCS2	-0.039243481	0.037265555	0.002076	-0.00423	0.018053	-0.04831
HMGN1	0.070563667	0.071409979	0.166154	0.124484	-0.27114	-0.61754
HMGN2	0.078305422	0.043212665	-0.09013	0.154513	-0.30738	-0.24548
HMGN2P46	-0.033330253	-0.010213168	-0.06316	-0.01858	-0.01343	-0.01016
HMGN3	0.027295493	0.079528466	0.120345	-0.05502	0.171408	0.108613
HMGN3-AS1	-0.055665851	0.034966451	-0.09633	-0.04464	0.146554	-0.02432
HMGN4	0.013121504	0.210335481	0.194311	0.058042	-0.02272	-0.43788
HMGN5	-0.018808397	0.037497014	0.047613	0.058063	-0.11842	-0.0083
HMGXB3	0.034355262	-0.017542922	0.091816	0.035299	-0.17688	-0.16824
HMGXB4	0.046783331	0.046529709	-0.07705	0.07721	-0.24997	0.23097
HMHA1	0.024697117	-0.032274785	0.07667	-0.0403	0.126518	-0.03247
HMHB1	-0.013761056	-0.083115897	-0.07535	0.012148	-0.01058	-0.15623
HMMR	-0.012343288	0.027200461	0.016793	0.178436	-0.34344	-0.06316
HMMR-AS1	-0.022677753	-0.022202769	-0.0061	0.060718	-0.16463	-0.04618
HMOX1	0.009762894	-0.068630706	-0.30072	-0.09349	0.234753	0.53713
HMOX2	0.0237833	0.151226883	0.282054	0.023935	-0.13349	-0.394
HMP19	-0.040459478	0.010672709	-0.10072	0.01722	-0.0159	0.138224
HMX1	-0.010056651	-0.12678751	0.087873	0.007774	-0.09041	-0.08198
HMX2	-0.044662415	-0.024976162	-0.07728	-0.01974	0.084262	0.020308
HN1	0.044808109	0.041544756	-0.20693	0.066672	-0.17252	0.140378
HN1L	0.005445514	-0.014032717	0.15196	0.03521	-0.17136	-0.27901
HNF1A	-0.036839155	-0.037416528	-0.02049	0.019331	-0.10353	-0.10247
HNF1B	-0.015902615	-0.06300317	0.098796	-0.0166	-0.04522	-0.09112
HNF4A	-0.038905057	-0.075773506	0.02794	0.058939	-0.05161	0.085208
HNF4A-AS1	-0.059443182	-0.045455514	-0.01465	-0.02763	0.062403	0.019894
HNF4G	-0.069180001	-0.066273632	0.013486	0.022581	-0.06736	-0.0085
HNMT	0.024831189	-0.05019414	-0.32295	-0.05795	0.225593	0.683712

HNRNPA0	0.066380385	0.039714456	0.087076	0.061082	-0.19	-0.17915
HNRNPA1	0.020098063	-0.034932725	0.208179	-0.02332	0.07331	-0.09405
HNRNPA2B1	0.100499356	0.139872651	-0.06589	0.035896	-0.03525	-0.30622
HNRNPA3	0.089876456	0.240164896	0.136178	0.039034	-0.12452	-0.19068
HNRNPA3P1	-0.042822545	0.039116975	-0.02851	0.034508	-0.09171	0.01334
HNRNPAB	0.045230064	0.128393126	-0.03175	0.099092	-0.26701	0.118347
HNRNPC	0.017539604	0.08515777	0.074864	0.070081	-0.18232	-0.17484
HNRNPD	0.043855076	-0.05021941	0.082469	0.038353	-0.12286	-0.33935
HNRNPDL	0.042857555	0.077412225	0.244723	0.020503	-0.07109	-0.12191
HNRNPF	0.061413292	0.136135623	0.087899	0.053552	-0.15538	-0.11986
HNRNPH1	0.007379964	0.007087603	0.153465	0.045622	-0.14706	-0.15502
HNRNPH3	0.076767081	-0.006204625	0.016474	0.037604	-0.12487	-0.24611
HNRNPK	0.128238776	0.15915722	-0.10254	0.11129	-0.24865	-0.33925
HNRNPL	0.071324014	0.047944193	-0.07022	0.086231	-0.23947	-0.06408
HNRNPLL	-0.021951791	-0.085266677	0.095456	0.130962	-0.17063	0.004732
HNRNPM	-0.010882897	-0.110082429	0.052867	0.009755	-0.07953	-0.06036
HNRNPR	0.067952549	0.154597181	0.155778	0.068906	-0.19277	-0.07696
HNRNPU	0.00104813	0.099425103	0.012123	0.032372	-0.0832	-0.14731
HNRNPU-AS	0.021649522	0.012748806	0.031822	-0.00103	0.035641	-0.07549
HNRNPUL1	0.064099902	0.032645654	0.0601	0.035294	-0.08831	-0.06322
HNRNPUL2	0.074874633	0.138578362	0.070274	0.056914	-0.19692	-0.07833
HOGA1	-0.030315199	-0.026984539	0.047652	0.021361	-0.05365	-0.02067
HOMER1	-0.002669149	0.025010171	0.051499	-0.00638	0.004759	-0.13294
HOMER2	-0.01926136	-0.036150197	-0.06488	-0.04764	0.109696	-0.07261
HOMER3	-0.029137141	-0.098558689	-0.19694	-0.10217	0.306547	0.65302
HOMEZ	-0.028839121	0.018356848	0.06384	0.015835	-0.02393	0.084763
HOOK1	-0.008976734	-0.007570701	0.368394	-0.06805	0.193772	-0.4403
HOOK2	-0.004856707	-0.062539976	0.070969	0.051652	-0.16731	0.146823
HOOK3	-0.028381491	0.037235822	-0.1963	-0.03853	0.004045	0.288911
HOPX	-0.03821099	0.029601133	-0.00263	-0.02513	-0.0666	-0.01504
HORMAD1	-0.045665616	-0.095496735	-0.22019	-0.0406	0.122796	0.25041
HORMAD2	-0.030897892	-0.02827373	-0.13972	0.006678	-0.00717	-0.05129
HOTAIR	-0.065926547	-0.04649767	-0.11241	-0.03757	0.071884	0.009657
HOTAIRM1	0.010414975	0.046347864	-0.18501	-0.02585	0.076318	0.238406
HOTS	-0.041191264	-0.021710109	0.011191	-0.01183	0.077936	0.05514
HOTTIP	-0.060311464	0.032818046	0.07268	0.018699	-0.00751	-0.08288
HOXA-AS2	-0.034943846	-0.01300404	-0.05954	-0.01079	0.065316	0.214005
HOXA-AS3	-0.035625626	-0.027735658	0.000109	0.01173	-0.00594	0.101556
HOXA1	-0.009351927	0.044205532	-0.03746	0.069187	-0.18063	0.178057
HOXA10	-0.014038046	0.138670764	-0.10152	-0.00936	-0.03216	-0.10686
HOXA11	-0.044460551	-0.019854749	-0.09227	0.017792	-0.06164	-0.03
HOXA11-AS	-0.039273757	-0.039878829	-0.14226	0.022303	-0.0431	0.031151
HOXA13	-0.080504043	-0.036990044	-0.07369	-0.00407	-0.06536	-0.07812
HOXA2	-0.029878131	0.000732653	-0.03177	-0.00494	0.018169	-0.1201
HOXA3	-0.021644158	0.08452926	-0.0808	0.011413	-0.06673	-0.09915
HOXA4	-0.029546889	-0.024232934	-0.0174	0.017971	-0.06688	-0.06417
HOXA5	-0.010394451	0.042492979	-0.02355	0.038319	-0.12286	-0.07013
HOXA6	-0.028130034	0.017719146	-0.06956	0.032217	-0.06931	0.053132
HOXA7	-0.019653699	-0.043765342	-0.05351	0.000883	-0.01277	-0.0294
HOXB-AS1	0.000502191	0.133244737	0.109134	0.006615	########	-0.22521
HOXB-AS3	-0.029603321	-0.036632878	-0.02215	-0.00225	0.051185	0.085236

HOXB1	-0.044556916	-0.070397614	-0.05978	0.024525	-0.04102	0.037918
HOXB13	-0.045888849	-0.114458037	0.048288	0.017878	-0.00833	0.099197
HOXB2	0.014652123	0.141525612	0.120384	0.014646	-0.05401	-0.24885
HOXB3	-0.001781046	0.192276848	0.078356	0.043552	-0.12565	-0.2871
HOXB4	0.007139311	0.048230844	0.002511	0.040748	-0.1985	-0.13466
HOXB5	-0.034578078	-0.046343596	-0.04194	0.0103	-0.10689	-0.07823
HOXB6	-0.019710568	-0.028282307	-0.0008	0.044537	-0.14042	0.004932
HOXB7	-0.028848262	0.042260647	-0.02266	0.194252	-0.43322	-0.00316
HOXB8	-0.055440084	-0.038738946	-0.05543	0.009668	-0.03188	0.008906
HOXB9	-0.036598854	-0.015500423	0.03098	0.075526	-0.26764	-0.08801
HOXC-AS3	-0.033179252	0.036954939	-0.07045	-0.01846	0.062454	-0.07213
HOXC10	-0.028444223	0.032225131	-0.0214	0.028621	-0.08311	-0.09403
HOXC11	-0.02750754	-0.026998486	0.030374	-0.01811	-0.03997	-0.0647
HOXC12	-0.031571028	-0.050351918	-0.04409	0.018763	-0.02662	0.078487
HOXC13	-0.031542087	-0.088751777	-0.00306	0.004525	0.00934	-0.01605
HOXC4	-0.010107793	0.074448677	0.059322	0.087274	-0.29924	-0.17792
HOXC5	-0.031257854	-0.058732919	-0.05266	-0.00318	-0.05324	-0.00331
HOXC6	-0.016652217	0.019687857	0.041638	0.067622	-0.21256	-0.06559
HOXC8	-0.027008627	-0.000213403	-0.11739	0.005091	-0.02366	0.102553
HOXC9	-0.036540172	-0.037121671	-0.11216	-0.00656	0.042253	0.100401
HOXD-AS2	-0.037013399	-0.085734727	0.003604	0.017716	-0.05126	-0.01754
HOXD1	-0.053699615	0.001811413	0.008563	-0.00389	-0.00124	-0.02385
HOXD10	0.014787635	-0.068922232	0.03896	0.073767	0.005809	0.034363
HOXD11	-0.041099842	-0.044224468	0.006902	0.021472	-0.03744	-0.03211
HOXD12	-0.046213707	0.012153513	-0.08166	-0.00361	0.015752	0.138878
HOXD13	-0.030966202	0.016933997	-0.1211	0.012628	0.00388	0.181329
HOXD4	-0.030501185	-0.046485356	-0.0114	0.010926	-0.00503	0.021837
HOXD8	-0.032654882	0.013116269	0.0216	0.025233	-0.05707	0.208168
HOXD9	-0.043345998	-0.06779021	-0.10273	0.009805	-0.02602	-0.01273
HP	0.002753321	-0.114289741	-0.00867	-0.02828	0.100767	0.609939
HP08942	-0.030684682	-0.037992553	-0.17509	0.000492	0.011641	0.0484
HP09025	-0.037271599	-0.00903766	0.02071	0.002979	0.009564	-0.10978
HP1BP3	0.09270516	0.054096714	0.13402	0.006601	-0.02572	-0.38717
HPCA	-0.031639158	-0.133018277	0.04205	0.024074	-0.08309	-0.0141
HPCAL1	0.023777317	-0.019159394	-0.01367	-0.04686	0.119852	0.161617
HPCAL4	-0.022990688	-0.196708685	0.332225	-0.03682	0.102042	-0.21329
HPD	-0.022595858	-0.057796428	-0.05832	0.013647	-0.02461	0.058054
HPDL	-0.014636729	-0.008456214	0.004225	0.091358	-0.26828	-0.05744
HPGD	-0.014851692	0.011487183	0.201965	0.093008	-0.23626	-0.17576
HPGDS	-0.003944659	0.015154109	-0.00408	-0.01303	0.042835	-0.07365
HPN	-0.036273491	0.004780707	-0.00322	0.007136	-0.00452	-0.04392
HPR	-0.043909222	-0.01316165	-0.0455	0.009031	0.005722	0.053007
HPRT1	0.002057657	0.016507989	0.078562	0.092209	-0.21939	0.143012
HPS1	0.077302832	-0.048726535	-0.14043	-0.08056	0.141977	0.07868
HPS3	-0.002727305	0.018541211	0.0573	0.004905	-0.03418	-0.04358
HPS4	0.088003767	-0.013849229	0.155664	0.060761	-0.08233	-0.15244
HPS5	0.030605913	0.010344921	-0.19355	0.021268	-0.04367	0.06235
HPS6	0.018685056	0.025453986	-0.08778	-0.0082	-0.02274	0.192948
HPSE	0.002229265	-0.064438031	-0.2222	-0.09889	0.257356	0.589895
HPSE2	-0.042076351	-0.06957188	-0.1153	-0.03186	0.121786	0.082398
HPX	-0.018966613	-0.044049645	0.004427	-0.01791	-0.04935	-0.09604

HPYR1	-0.050116798	-0.058211284	0.130724	-0.0039	0.019025	-0.14741
HR	-0.046196442	-0.033790074	-0.06738	-0.07753	-0.10888	0.063133
HRAS	0.01837279	0.02426122	0.081697	0.095091	-0.2688	0.04742
HRASLS	-0.025603979	-0.038428818	-0.02906	-0.25971	0.754238	0.015203
HRASLS2	-0.006538201	-0.053674503	-0.03659	0.063799	-0.06598	-0.15604
HRASLS5	-0.029148909	-0.002915969	-0.08571	-0.00219	-0.02869	-0.09035
HRC	-0.032448924	-0.079640835	0.00436	0.019142	-0.04002	-0.00511
HRCT1	-0.023028713	-0.08894816	0.028651	0.026096	-0.05915	-0.00853
HRG	-0.046728747	-0.057610382	0.055985	0.021901	-0.04657	-0.07163
HRH1	-0.009648814	-0.043515411	0.027229	0.008769	-0.03781	-0.00272
HRH2	-0.01952696	-0.150222167	-0.12817	-0.00643	-0.01661	0.037445
HRH3	-0.022922912	-0.06706515	-0.07887	0.008498	-0.0857	-0.04648
HRH4	-0.016737889	-0.056309805	-0.06297	-0.01739	0.042167	-0.12934
HRK	-0.007551436	-0.066076704	-0.05278	-0.01848	0.060766	-0.12925
HRSP12	-0.002160506	-0.014994077	0.162223	0.065289	-0.15548	0.130559
HS1BP3	-0.036041183	-0.066094639	-0.11727	-0.00195	-0.00249	0.304792
HS2ST1	0.031352281	0.044023899	-0.08406	0.007836	-0.06779	0.196832
HS3ST1	-0.031040539	-0.146515359	-0.14549	0.002909	0.053752	-0.07628
HS3ST2	-0.008066061	-0.039696332	-0.03254	-0.01631	-0.01618	-0.08592
HS3ST3A1	-0.058592897	-0.020542408	0.101025	0.012672	-0.03572	-0.12126
HS3ST3B1	0.0500802	0.081157963	0.071386	0.080675	0.003601	-0.16553
HS3ST4	-0.030051997	-0.010010192	0.052664	0.010789	-0.03172	0.011956
HS3ST5	-0.028465249	-0.067836075	0.080025	0.011211	-0.0385	-0.04252
HS3ST6	-0.035943355	-0.042512193	-0.12379	0.002965	0.022041	0.17105
HS6ST1	-0.017494481	-0.008145117	-0.12419	-0.03506	0.064886	0.000536
HS6ST2	-0.061101878	-0.017168746	-0.01086	0.010978	0.018918	-0.00864
HS6ST3	-0.037502876	-0.075730058	-0.00187	0.019746	0.007167	0.172792
HSBP1	0.05398132	-0.017683413	-0.52686	0.004965	0.010071	0.515584
HSBP1L1	0.004614152	-0.026110943	0.324758	-0.04693	0.161403	0.017059
HSCB	0.027252589	0.058482752	0.035364	0.038601	-0.09794	0.142938
HSD11B1	-0.006370982	-0.025514287	-0.04898	0.037773	-0.12039	0.021981
HSD11B1L	-0.027163573	-0.120972273	0.035883	0.035169	-0.12572	0.007492
HSD11B2	-0.020762881	-0.043141547	-0.02673	0.005665	-0.00254	-0.00737
HSD17B1	-0.002789356	0.012558775	-0.0664	0.007835	-0.04112	0.015403
HSD17B10	0.0458601	0.043356807	0.056879	0.083824	-0.24948	0.260051
HSD17B11	0.033134442	-0.028422624	-0.10927	-0.08068	0.231786	0.170396
HSD17B12	-0.029057805	-0.04090942	-0.042	-0.0218	-0.00339	-0.02183
HSD17B14	-0.00012675	-0.175380307	0.02483	0.17553	-0.33808	0.007117
HSD17B2	-0.052937282	-0.006365651	0.013702	0.054227	-0.14074	-0.01516
HSD17B3	-0.024431042	-0.000933327	0.071031	-0.15653	0.435198	-0.08037
HSD17B4	0.015414943	-0.043202415	-0.27378	-0.02128	0.046494	0.145369
HSD17B6	-0.022526468	-7.61E-05	0.089892	0.083079	-0.19778	-0.05701
HSD17B7	0.058609162	0.061534194	0.088549	0.065294	-0.19853	-0.27333
HSD17B7P2	-0.019670271	0.062330617	0.065348	0.001418	0.016208	-0.0348
HSD17B8	0.01302046	-0.04324577	0.526906	0.046205	-0.16105	-0.26717
HSD3B1	-0.054445193	-0.082314607	-0.04285	-0.00298	0.018427	-0.00843
HSD3B2	-0.057376901	-0.076090786	-0.07989	-0.01145	0.055746	-0.01555
HSD3B7	-0.025505571	-0.073682611	-0.02849	0.057878	-0.23563	-0.07519
HSDL1	-0.000188753	0.045362425	0.069216	-0.01727	0.04837	0.052705
HSDL2	-0.004041478	-0.046546268	-0.1694	0.037284	-0.10562	-0.05139
HSF1	-0.00589691	-0.12239115	0.125602	0.064108	-0.19472	0.148826

HSF2	-0.003931175	0.02077563	0.42228	0.084731	-0.22067	-0.30065
HSF2BP	-0.030494689	-0.038109111	0.070628	0.056627	-0.16793	-0.10008
HSF4	-0.005344931	-0.102719125	0.041089	-0.01168	-0.08846	0.094729
HSF5	-0.014113356	0.015572397	0.258322	-0.05864	0.193379	-0.22674
HSH2D	0.007061355	0.170276979	-0.3362	########	-0.016	-0.08203
HSP90AA1	0.088013788	0.199358993	0.144705	0.132603	-0.28208	-0.14764
HSP90AB1	0.008626488	0.054338349	0.227175	0.005981	-0.126	-0.07455
HSP90B1	0.041936528	0.107125696	0.137571	0.057624	-0.1633	0.059529
HSPA12A	-0.040063627	-0.056289308	-0.03287	0.003892	0.006657	-0.02509
HSPA12B	-0.04426421	-0.053954777	-0.09639	0.016846	-0.03686	0.108016
HSPA13	0.023636215	0.062991882	0.013959	0.05855	-0.18025	0.008813
HSPA14	0.001193218	0.010715377	0.063292	0.038464	-0.11662	-0.10265
HSPA1L	-1.51E-05	-0.009958074	0.086889	-0.03456	0.055318	0.079613
HSPA2	-0.014589302	0.009486764	-0.0072	0.006478	-0.04744	-0.01247
HSPA4	0.026501608	-0.010134787	-0.14896	0.063524	-0.20546	0.162398
HSPA4L	-0.055532993	-0.057387528	0.011115	-0.00745	0.026116	0.052999
HSPA5	0.04838205	0.116908634	-0.08408	0.071441	-0.18996	0.077109
HSPA6	0.007781286	0.016125979	-0.39061	-0.0637	0.172442	0.287214
HSPA9	0.028360271	0.078670508	0.143981	0.101971	-0.28993	0.020129
HSPB1	0.007804582	-0.071693671	0.221405	-0.09465	0.212777	-0.124
HSPB11	0.030934255	0.076534964	0.119368	0.079113	-0.1938	-0.02288
HSPB2	-0.032143355	-0.055740815	-0.12921	0.015544	-0.0594	-0.01145
HSPB3	-0.032073903	-0.044715876	-0.12524	-0.00353	0.03192	0.012315
HSPB6	-0.032313467	0.002407751	0.044845	0.036202	-0.08351	0.019136
HSPB7	-0.042845604	-0.0857463	-0.02722	-0.00659	0.056117	0.128763
HSPB8	-0.044222805	-0.030135144	-0.00485	0.026733	-0.07551	0.091009
HSPB9	-0.030911025	-0.008987429	0.021584	0.002894	0.01222	-0.02521
HSPBAP1	0.028343471	-0.038298268	-0.29137	-0.10676	0.313229	0.26177
HSPBP1	0.015960384	-0.052369796	0.055425	0.045416	-0.20559	-0.106
HSPC081	-0.024401466	-0.045660965	-0.04092	0.015132	-0.05117	-0.03111
HSPD1	-0.038902272	-0.109257819	-0.05353	0.159116	-0.063	-0.10216
HSPE1	0.031868809	0.053885562	0.237287	0.070465	-0.19564	-0.04037
HSPG2	-0.026043857	-0.134976326	0.000993	0.019083	-0.10321	-0.05741
HSPH1	0.013450073	0.068832216	0.164929	0.058772	-0.1636	-0.07051
HTATIP2	0.019786333	-0.004834104	-0.02798	-0.04408	0.101088	0.179099
HTATSF1	0.034703422	0.126595873	0.214009	0.071147	-0.19702	-0.0481
HTATSF1P2	-0.006978964	0.040293133	-0.16375	0.023244	-0.05689	0.397138
HTN1	-0.046380776	-0.092962658	0.012099	0.001893	0.016259	-0.06693
HTN3	-0.044760664	-0.015056055	-0.06699	0.017116	-0.04482	-0.0181
HTR1A	-0.03496637	-0.044451612	-0.0588	0.011142	-0.01659	-0.00891
HTR1B	-0.03207192	-0.080205162	-0.0855	0.006722	-0.01831	-0.03868
HTR1D	-0.033857315	-0.005100155	0.098095	0.007577	-0.00329	0.072266
HTR1E	-0.031286379	-0.00935783	0.029862	0.00686	-0.0297	-0.12592
HTR1F	-0.031648892	-0.023048521	0.035608	0.027698	-0.07976	-0.1073
HTR2A	-0.033782968	-0.011825887	-0.0399	0.000317	0.036851	-0.00023
HTR2B	-0.0171879	0.003913472	-0.01931	-0.0374	0.109334	-0.00358
HTR2C	-0.076448642	0.001812555	-0.07292	-0.00713	0.019416	-0.06993
HTR3A	-0.025185525	-0.085811098	-0.15353	-0.02595	0.081251	-0.22043
HTR3B	-0.029590386	0.012706848	-0.11487	0.017344	-0.03995	0.020944
HTR3C	-0.033505968	-0.047694382	-0.00264	0.010914	-0.03769	-0.00776
HTR4	-0.082577609	-0.031596048	0.136118	-0.03298	-0.02893	-0.0809

HTR5A	-0.028861451	-0.047718118	0.013878	-0.01119	0.030327	-0.01443
HTR6	-0.054884709	-0.097175664	0.044731	0.020994	-0.04907	-0.04514
HTR7	-0.081722404	-0.059446259	-0.06049	-0.09046	0.045967	-0.00093
HTR7P1	-0.024803477	-0.117870188	-0.05544	-0.03112	0.111803	0.32538
HTRA1	-0.023207165	-0.080134565	-0.07352	-0.04121	0.112941	0.410756
HTRA2	0.03334786	0.061882284	0.066517	0.090136	-0.28595	-0.49227
HTRA3	-0.030250735	-0.079043057	0.121219	0.010312	-0.08624	0.033942
HTRA4	-0.009799715	0.022098765	-0.00804	-0.00111	0.01936	-0.06099
HTT	0.026489156	0.0401896	0.02719	-0.01695	0.030446	0.042892
HUNK	-0.04628317	-0.019580829	-0.08929	0.014616	-0.05378	-0.11298
HUS1	0.032659363	0.102054487	-0.01016	0.023924	-0.05069	0.092222
HUS1B	-0.028575059	-0.003922225	0.002128	0.017563	-0.03223	-0.04836
HUWE1	-0.027244652	-0.026554788	0.178724	0.02481	-0.10755	-0.26091
HVCN1	0.025665882	-0.097054057	-0.37448	-0.09163	0.238467	0.112046
HYAL1	-0.03134532	-0.050334908	-0.10138	0.025562	-0.07012	-0.0843
HYAL2	-0.003730598	-0.087226675	-0.10843	0.000565	-0.06871	0.134827
HYAL3	-0.016222321	-0.099678717	0.001136	0.026352	-0.0887	0.007732
HYAL4	-0.027373179	0.00419095	-0.04594	0.074519	-0.18089	0.016316
HYALP1	-0.065566943	-0.070198679	-0.03581	0.000217	-0.01881	-0.12408
HYDIN	-0.03587215	-0.070559187	0.13053	0.011023	-0.00604	0.004019
HYDIN2	-0.048268367	-0.068549408	-0.19622	0.012515	-0.02621	0.06843
HYI	0.010193391	-0.05520149	-0.07995	-0.01951	0.025029	-0.18577
HYKK	-0.031449169	-0.131058584	0.210868	-0.06434	0.214475	-0.0803
HYLS1	0.019527621	0.092141638	-0.03396	0.031497	-0.07582	-0.06812
HYMAI	-0.014453055	-0.115304557	-0.13377	-0.01092	-0.00105	0.00385
HYOU1	0.029164134	0.133906945	0.058223	0.061109	-0.17219	-0.08067
HYPK	-0.003741532	-0.017279174	0.078446	0.028159	-0.10681	-0.04467
HYPM	-0.026732717	-0.034602745	-0.09663	0.019428	-0.03281	0.05009
IAH1	0.017358157	0.118907317	-0.02422	0.000962	-0.05275	0.09463
IAPP	-0.050944442	-0.065553086	0.050398	-0.00851	0.054851	0.008561
IARS	0.026295258	0.083339276	0.167889	0.049951	-0.13013	-0.12379
IARS2	0.039106558	0.050099478	0.104358	0.051908	-0.1262	0.115703
IBA57	-0.033690005	-0.034736539	-0.03131	0.035425	-0.17314	-0.02393
IBA57-AS1	-0.032101453	-0.018048154	-0.07843	0.010162	-0.01262	0.028192
IBSP	-0.057659128	-0.083001521	0.015606	-0.00298	0.016881	-0.04974
IBTK	0.014889462	0.050753283	0.064087	0.013117	-0.03578	0.135448
ICA1	0.004795071	0.025411631	-0.18164	-0.06474	-0.25074	0.154201
ICA1L	-0.033180907	0.052227953	-0.03901	0.009785	-0.01622	0.074302
ICAM1	0.00500273	-0.01454045	-0.29015	0.064132	-0.19105	0.210303
ICAM2	0.030034549	0.143971595	0.206536	-0.03494	0.106022	0.060114
ICAM3	0.026806403	0.016782536	-0.06025	-0.0412	0.112131	-0.06754
ICAM4	-0.004942226	-0.096997752	-0.18438	-0.02532	0.096938	0.444482
ICAM5	-0.026163207	-0.108659223	-0.06567	0.031819	-0.06706	0.080142
ICE1	0.037023937	0.117693384	0.163673	0.018074	-0.03336	-0.10992
ICE2	0.009053536	-0.009681683	0.228634	-0.02244	-0.04003	-0.10841
ICK	-0.039505192	0.078498655	0.030039	-0.01587	0.089359	0.003233
ICMT	0.028602293	0.098545317	0.041299	0.083075	-0.22287	0.088826
ICOS	-0.006903717	-0.096207458	0.336549	0.056312	-0.20439	-0.25236
ICOSLG	-0.023616538	-0.15875195	-0.03069	-0.03446	-0.00493	-0.04426
ICT1	0.024841761	0.019000707	0.099804	0.04467	-0.11095	0.203461
ID1	-0.005490196	-0.101832991	0.017078	0.007407	0.01006	0.234652

ID2	0.025535477	0.169860534	0.214404	-0.0006	0.012392	0.13512
ID2B	-0.018076459	0.024050208	0.059173	-0.00308	0.024221	0.047475
ID3	-8.04E-05	-0.122852995	0.076759	-0.06954	0.183533	-0.22838
ID4	-0.092185067	-0.061700305	0.079117	-0.00572	0.044355	-0.0385
IDE	0.025204002	-0.069055473	-0.04271	0.064663	-0.21029	0.197413
IDH1	0.021372466	0.009380514	-0.31133	-0.00988	0.027943	0.413496
IDH1-AS1	-0.023540261	-0.024110349	-0.1147	0.068037	-0.16634	0.206818
IDH2	0.020298846	0.061226963	0.044958	0.004967	-0.0182	0.058595
IDH3A	0.027340368	-0.052067298	-0.10162	0.074145	-0.11811	0.077848
IDH3B	0.059418202	0.052426483	0.023302	0.061212	-0.19642	0.259195
IDH3G	0.050708871	-0.025934547	-0.0983	-0.00452	-0.01991	0.350621
IDI1	0.066280725	0.180601013	-0.13911	0.065674	-0.12953	-0.32125
IDI2	-0.033777453	-0.020706085	-0.05011	-0.00766	0.005079	-0.04342
IDI2-AS1	-0.027466076	-0.008826788	0.014911	0.022861	-0.06832	-0.13728
IDNK	0.021652686	0.085267568	0.182717	0.003648	-0.01849	-0.21456
IDO1	0.000877099	-0.068979734	-0.13217	0.003956	-0.01028	-0.14794
IDO2	-0.008764089	-0.068048156	-0.11293	-0.00558	0.018881	-0.10638
IDS	0.014226679	0.10317113	-0.01429	0.012379	-0.07821	-0.19502
IDUA	0.04890743	0.022853747	-0.0056	0.129191	0.043044	0.166803
IER2	0.051692929	0.112090265	-0.12087	-0.00013	0.009814	-0.06645
IER3	0.01218546	-0.016312413	-0.14441	0.004603	-0.01315	0.249681
IER3IP1	0.035681515	0.072434774	0.065321	0.048584	-0.15764	0.109776
IER5	0.041762288	0.000438238	-0.34443	0.039754	-0.09186	0.3246
IER5L	-0.01981116	-0.105786969	-0.08211	-0.00875	0.040847	0.468741
IFFO1	0.038852663	0.041410467	-0.05396	-0.09205	0.234697	0.31031
IFFO2	0.013812895	0.114387766	0.270965	-0.01719	0.054762	-0.18794
IFI16	0.041199763	0.154146202	-0.08217	0.039773	-0.08365	0.034252
IFI27	-0.010001856	-0.014105333	-0.02085	5.44E-05	0.004211	0.037983
IFI27L1	0.004641098	-0.016310362	-0.06055	0.077776	-0.30589	-0.12232
IFI27L2	0.03889977	0.000133748	0.037028	0.072463	-0.24211	0.291594
IFI35	0.023224266	0.070364488	-0.16665	0.016156	-0.05573	0.235982
IFI44	0.009520207	0.014935255	-0.05757	-0.06365	0.184255	0.23491
IFI44L	0.009304208	-0.028423141	-0.14304	-0.09437	0.2699	0.199147
IFI6	0.017636273	0.02744285	-0.07214	-0.00126	0.001573	0.313219
IFIH1	-0.014336785	-0.027186661	-0.08329	0.004148	0.036242	0.214889
IFIT1	0.007311312	-0.007068614	-0.20017	-0.06772	0.196122	0.21358
IFIT2	0.013539894	0.022745429	-0.3082	-0.04936	0.148276	0.094671
IFIT3	0.013545561	0.044226524	-0.33203	-0.04389	0.137387	0.152831
IFIT5	0.019237163	0.047034887	-0.10025	-0.0569	0.133923	-0.01766
IFITM1	0.027592637	0.17775186	0.259774	-0.0098	0.029257	-0.4087
IFITM10	-0.028714485	-0.09008677	0.027948	0.001323	-0.04976	0.052009
IFITM2	0.02973298	0.136679107	-0.03476	-0.021	0.070058	-0.05551
IFITM3	0.034126063	0.118834697	-0.07895	-0.03431	0.109012	0.063097
IFNA1	-0.069113575	-0.041880877	0.066327	-0.03195	0.062736	0.017167
IFNA10	-0.029816009	-0.057011797	0.061086	-0.00724	0.02903	-0.09723
IFNA14	-0.025385446	-0.041013068	-0.00835	3.70E-05	-0.00118	-0.10705
IFNA16	-0.029982285	-0.04292661	-0.05093	-0.00805	0.041544	-0.06438
IFNA17	-0.029566014	-0.016646088	0.114554	-0.00791	-0.02698	-0.0816
IFNA2	-0.044005353	0.000516091	-0.0709	0.006613	-0.03957	-0.01817
IFNA21	-0.032603959	-0.052671389	-0.04356	-0.01341	0.042144	0.008302
IFNA4	-0.029402068	-0.01478341	0.0858	0.004464	-0.00476	-0.07611

IFNA5	-0.055925519	-0.07043337	0.02354	0.011925	-0.02623	0.038228
IFNA7	-0.026868427	-0.004944934	-0.06514	-0.00435	0.012345	-0.05005
IFNA8	-0.096223607	-0.056380676	-0.13744	-0.03517	0.085061	0.13519
IFNAR1	0.010433486	-0.024781264	-0.35809	-0.03632	0.108804	0.165322
IFNAR2	0.079610619	0.070994172	-0.20071	-0.08235	0.176425	-0.11904
IFNB1	-0.035824629	-0.113684095	-0.15876	0.006079	0.0042	0.187837
IFNE	-0.030111478	-0.014913798	0.01213	0.004979	-0.00768	-0.17477
IFNG	-0.006887049	0.032620001	0.102574	0.074902	-0.21986	-0.1078
IFNGR1	0.033320609	0.052279163	-0.46425	-0.02538	0.101121	0.265393
IFNGR2	0.020755757	-0.199335622	-0.38083	-0.06918	0.190236	0.382936
IFNK	-0.053615477	-0.05750373	0.001259	-0.02211	0.042702	0.051465
IFNL1	-0.021834714	0.037662527	0.194501	0.025277	-0.05262	-0.14433
IFNL2	-0.026045799	-0.02994626	0.013965	0.004218	-0.00705	-0.10562
IFNLR1	-0.004801509	-0.105443772	0.080837	-0.05174	0.085292	-0.27213
IFNW1	-0.036753245	0.012575052	-0.03645	0.026167	-0.04844	0.088928
IFRD1	0.01841111	-0.012770239	-0.17258	0.028469	-0.0574	-0.10925
IFRD2	0.014770148	-0.018191348	0.170582	0.038936	-0.13196	-0.09294
IFT122	-0.040183479	-0.03337883	0.022853	0.006492	-0.1182	-0.14731
IFT140	-0.127734226	-0.041937618	0.179467	-0.02264	0.026764	-0.03281
IFT172	-0.007909525	0.117290077	0.057812	-0.00697	0.028075	0.01837
IFT20	0.063595151	-0.016529048	-0.09301	-0.02765	0.077404	0.238016
IFT22	-0.004024555	-0.001303909	0.162419	0.031385	-0.09597	-0.21228
IFT27	0.00480977	0.067809226	0.02138	-0.0787	0.03384	-0.01969
IFT43	0.000880758	-0.004336011	0.150711	0.010761	-0.00427	-0.06288
IFT46	-0.002290789	-0.008377999	0.13703	0.078031	-0.26181	-0.14029
IFT52	0.017654143	0.0660031	-0.08666	0.057324	-0.17149	0.040415
IFT57	-0.006355314	-0.047263228	-0.01499	-0.00133	0.007951	-0.0713
IFT74	-0.010092777	-0.00319052	0.053997	-0.05454	0.170812	0.039779
IFT80	0.005792918	0.039572654	0.245166	0.001044	5.42E-05	-0.23155
IFT81	-0.029042428	-0.096921088	-0.01232	0.060261	-0.18601	-0.06178
IFT88	0.015915536	0.009184851	0.141792	-0.04771	0.120712	-0.04497
IGBP1	0.065765896	-0.017623745	0.189659	-0.004	0.014079	0.065381
IGDCC3	-0.03187991	-0.035045338	0.096835	-0.01028	-0.05658	-0.07463
IGDCC4	-0.025193466	0.010937506	-0.00631	0.004358	-0.01382	-0.08533
IGF1	-0.045017425	-0.041320743	-0.01252	0.271733	-0.12552	-0.06131
IGF1R	-0.003588453	0.046776424	-0.00916	-0.08589	0.257427	-0.17002
IGF2-AS	-0.022815266	0.00486104	-0.04998	-0.00745	-0.00172	0.005938
IGF2BP1	-0.010678294	-0.004420744	0.052957	0.086573	0.023331	0.010585
IGF2BP2	-0.002698372	-0.00761176	-0.21548	-0.12288	0.34696	0.416627
IGF2BP3	-0.026158888	-0.039577029	-0.0614	-0.1601	0.419292	0.09246
IGF2R	0.024644697	0.151239243	-0.12457	0.000766	0.001012	0.075177
IGFALS	-0.024033577	-0.102150025	0.010027	0.007498	-0.06002	-0.00848
IGFBP1	-0.08859136	-0.043807283	-0.10509	-0.01826	0.035591	0.094661
IGFBP2	-0.029719582	-0.003397957	-0.03714	-0.03958	0.14266	0.096806
IGFBP3	-0.004986638	-0.072582236	0.165603	0.063851	-0.17495	-0.24525
IGFBP4	-0.012853456	0.005601249	-0.01571	-0.03424	0.058649	-0.17618
IGFBP5	-0.040400578	-0.045317505	-0.09495	0.118728	-0.1059	0.029266
IGFBP6	-0.019899335	-0.096284833	0.248298	0.00735	-0.01201	-0.05004
IGFBP7	-0.001557054	0.231738511	-0.28039	-0.04638	0.141868	0.363919
IGFBP7-A!	-0.066214576	-0.002578289	0.012908	-0.05504	0.143387	0.020115
IGFBPL1	-0.040939672	-0.111774126	0.047979	0.013666	-0.0185	-0.01526

IGFL1	-0.037440718	-0.028533201	-0.13556	0.038575	-0.10005	0.028903
IGFL2	-0.030552881	-0.033942194	-0.03958	0.03343	-0.08756	-0.03661
IGFLR1	0.04157409	-0.058063427	-0.1766	0.000731	-0.01924	0.061651
IGFN1	-0.0298403	-0.064008361	-0.10481	0.024051	-0.05178	-0.03117
IGH	-0.019936938	-0.085466746	-0.08279	-0.00416	-0.07129	-0.07286
IGHD	0.006804794	-0.09156903	-0.14509	-0.08425	0.249915	-0.14678
IGHG1	-0.040153396	-0.00451311	0.116331	-0.0181	0.068043	-0.13583
IGHM	0.059137823	-0.134503996	-0.04512	-0.04823	0.201811	-0.13073
IGHMBP2	-0.009632249	0.029169882	-0.00295	0.000851	-0.09662	-0.05226
IGHV1-69	-0.026688329	-0.025310729	-0.05843	-0.0043	0.005396	-0.07254
IGHV3-54	-0.039365723	-0.070803682	-0.12321	-0.03554	0.129801	-0.11766
IGHV5-78	-0.008923042	-0.034581374	-0.14236	-0.01155	0.063001	-0.23872
IGIP	0.001747493	0.031962016	0.189342	-0.01525	0.040407	-0.13892
IGJ	0.002463834	-0.124770069	0.027503	-0.08465	0.239611	-0.19024
Igk	-0.02802619	-0.006992525	-0.01137	0.018762	-0.06076	-0.06945
IGK	-0.013324137	-0.081674877	-0.04436	-0.14151	0.385213	-0.0661
IGKC	-0.042283498	-0.080309304	0.026495	-0.05686	0.156991	-0.16417
IGLC1	0.009701153	-0.064121467	-0.04463	0.004987	0.161926	-0.03933
IGLJ3	-0.024907152	-0.077814609	-0.10058	-0.09682	0.177593	-0.15681
IGLL1	-0.025119654	-0.033547738	-0.00129	0.040522	-0.06489	-0.06189
IGLL3P	0.006104086	-0.110296344	0.014979	-0.10516	0.274487	-0.19832
IGLL5	-0.000632228	-0.06078608	-0.05943	-0.08906	0.23334	-0.21183
IGLON5	-0.028569491	-0.044633966	-0.05435	0.014142	-0.05555	-0.2215
IGLV1-44	-0.026265266	-0.066107921	-0.084	-0.08354	0.195075	-0.15451
IGLV6-57	-0.024246182	-0.095145558	-0.09936	-0.03121	0.094472	-0.09561
IGSF1	-0.054561049	-0.06123551	-0.0291	0.037269	-0.08671	-0.06714
IGSF10	-0.024114648	-0.054178308	-0.02516	-0.01211	0.036309	-0.03649
IGSF11	-0.024058341	-0.028443679	0.021435	-0.00844	0.033714	-0.14011
IGSF11-AS	-0.069245652	-0.005232201	-0.02907	0.011221	-0.02981	0.002214
IGSF21	-0.03674477	-0.102987199	-0.1975	0.02103	-0.03667	0.182466
IGSF22	-0.017167703	-0.062799335	0.259329	-0.02383	0.00088	-0.35255
IGSF3	-0.032151481	-0.131120878	-0.1011	-0.01693	-0.02749	0.001709
IGSF5	-0.035971516	-0.066927139	0.02796	-0.00105	0.034657	0.080417
IGSF6	0.006828421	-0.123984282	-0.40053	-0.08474	0.239687	0.514793
IGSF8	-0.003071381	-0.043918359	0.325362	-0.00287	0.030254	-0.16277
IGSF9	-0.037866159	-0.095303194	-0.14989	-0.01054	0.036739	-0.11961
IGSF9B	-0.033052317	-0.065100055	0.085116	0.018726	-0.07757	-0.17896
IHH	-0.036082279	-0.158876686	-0.03034	0.002001	-0.08228	0.017944
IK	0.116911862	0.147457449	0.11607	0.060478	-0.09597	-0.42762
IKBIP	0.007159803	0.025061172	-0.2496	0.032366	-0.0629	0.104784
IKBKAP	0.015126723	0.126635308	0.247167	0.015069	-0.0319	-0.11341
IKBKB	0.015662649	0.01403976	-0.0775	-0.06276	0.116374	-0.01142
IKBKE	0.0035502	-0.058466489	0.285983	-0.00256	-0.02656	0.22091
IKBKG	0.050785446	0.039901396	-0.14183	-0.00799	-0.01273	0.001489
IKZF1	-0.064134673	0.064771549	0.11924	-0.06063	0.094085	-0.21591
IKZF2	0.009179956	0.035133343	0.012584	-0.07237	0.035803	-0.17118
IKZF3	0.001276821	0.136782201	0.290969	-0.02403	0.07665	-0.36423
IKZF4	-0.008460517	-0.024793864	-0.02498	0.017064	-0.00351	0.173619
IKZF5	0.016219892	5.15E-08	0.089301	-0.00502	0.000799	-0.1964
IL10	-0.019080992	-0.030299431	-0.02571	0.012186	-0.09208	0.022212
IL10RA	0.029278848	0.083238572	0.168444	-0.0267	0.059979	0.158336

IL10RB	0.036485157		-0.025182596	-0.31614	-0.0163	0.066959	0.27519
IL10RB-AS	0.004793677		-0.085888314	-0.35845	-0.03382	0.106009	0.619545
IL11	-0.026949459		-0.025481264	-0.03736	0.019288	-0.1135	-0.02847
IL11RA	0.003927198		-0.037834534	0.306373	-0.0592	0.171655	-0.17422
IL12A	-0.020810733		0.011707479	0.069284	0.058584	-0.14467	-0.05998
IL12A-AS1	-0.03259417		-0.026013377	0.027253	-0.00914	0.053948	-0.03455
IL12B	-0.027664875		-0.020577738	0.004083	-0.0276	0.019396	-0.06638
IL12RB1	0.012517439		0.050916125	-0.0328	0.029652	-0.13006	0.006846
IL12RB2	-0.005290826		0.276069754	-0.0842	0.086823	-0.25545	-0.09796
IL13	-0.015158062		-0.032616712	-0.02355	-0.00227	-0.06091	-0.02865
IL13RA1	-0.003531402		-0.18284287	-0.46412	-0.10725	0.283523	0.393776
IL13RA2	-0.043394767		-0.032106587	-0.01492	-0.0192	0.057016	-0.04513
IL15	0.001480631		0.01376255	-0.17093	0.011872	-0.01399	0.499769
IL15RA	0.008344963		0.000438908	-0.02285	0.035363	-0.13527	0.21832
IL16	0.011741606		0.030102848	0.075666	-0.0742	0.196903	-0.22923
IL17A	-0.029434525		-0.041122286	0.004213	-0.01798	0.023784	-0.0225
IL17B	-0.021976314		-0.023487472	-0.10736	0.025229	-0.0475	-0.01145
IL17C	-0.01245253		-0.065035749	-0.06696	-0.00901	-0.06186	0.000663
IL17D	-0.030651149		-0.013068014	-0.09767	0.029348	-0.05715	0.020922
IL17F	-0.009575347		-0.007705452	-0.05368	0.00146	0.001968	-0.00932
IL17RA	0.042442544		-0.074934292	-0.14315	-0.06248	0.194973	0.367071
IL17RB	0.016658181		-0.007612251	0.002821	0.005477	-0.10807	-0.04652
IL17RC	-0.051379331		-0.099023869	-0.08752	-0.05925	0.050217	0.258937
IL17RD	-0.055061333		-0.054709145	-0.08901	0.054614	-0.13406	-0.02816
IL17RE	-0.034969062		-0.063941605	0.035997	0.022715	-0.02203	0.134253
IL18	-0.001314826		0.00434692	-0.18487	-0.07447	0.233884	0.320533
IL18BP	-0.012588679		-0.062043619	0.077841	-0.09454	0.022657	-0.02845
IL18R1	0.004732338		0.161059059	-0.00984	0.01619	-0.0581	-0.46979
IL18RAP	0.013288921		0.262266318	0.046867	-0.00413	0.002283	-0.31906
IL19	-0.015252146		-0.026600598	0.023864	0.008519	-0.0344	-0.00584
IL1A	-0.017887331		-0.013846085	-0.05603	-0.03542	0.055857	-0.0486
IL1B	0.01077691		-0.06730816	-0.22661	-0.02602	0.081235	0.227705
IL1F10	-0.02925205		-0.085075818	0.014785	0.032022	-0.09202	0.113162
IL1R1	-0.007012496		-0.034665607	-0.16886	0.006756	-0.01799	0.000383
IL1R2	0.002199345		-0.080649192	-0.24602	-0.00808	0.028416	0.043763
IL1RAP	-0.005210045		-0.042573167	-0.1604	0.036371	-0.11588	-0.01219
IL1RAPL1	-0.04780907		-0.084356141	-0.01136	0.038833	-0.08136	0.189362
IL1RAPL2	-0.028469698		-0.041238371	-0.04847	0.019107	-0.06291	-0.0605
IL1RL1	-0.013959433		-0.057066273	-0.05437	-0.00645	-0.03004	-0.06627
IL1RL2	-0.028736982		-0.027316979	-0.08009	0.017368	-0.04338	0.030103
IL1RN	-0.009522605		-0.048503843	-0.28446	-0.03907	0.119289	0.390477
IL2	-0.032383101		-0.052555487	-0.02333	-0.03835	-0.01535	-0.04915
IL20	-0.030555915		-0.024043651	-0.03983	0.031748	-0.07973	-0.09393
IL20RA	-0.041611412		-0.07036634	-0.07237	-0.00527	0.028396	0.065797
IL20RB	-0.030835149		0.007157114	-0.04459	0.033229	-0.07201	0.218514
IL21	-0.017943369		-0.025302609	-0.02734	0.005355	-0.05549	-0.00189
IL21R	-0.014364269		0.098055721	0.129165	0.037022	-0.0778	-0.17704
IL21R-AS1	-0.021899193		-0.071338975	-0.05868	-0.00137	-0.09831	-0.0974
IL22	-0.026545688		-0.038019507	-0.03922	0.008606	-0.06148	-0.02158
IL22RA1	-0.039532614		-0.119414625	-0.13661	0.025209	-0.07587	0.034171
IL22RA2	-0.047015917		-0.004166601	-0.02567	0.002456	0.004292	-0.00996

IL23A	-0.002842632	-0.084859729	0.121927	-0.01515	0.011187	-0.06329
IL23R	-0.019189496	-0.02455455	0.082684	0.152868	-0.25727	-0.04917
IL24	-0.00786318	-0.007373105	0.019638	-0.0271	0.069263	-0.09997
IL25	-0.031166964	-0.016258338	-0.11075	0.010659	-0.05985	0.036831
IL26	-0.034796709	-0.060762835	-0.03655	0.0313	-0.09762	-0.03656
IL27	-0.023362306	-0.05112142	-0.08019	0.004341	-0.05799	0.089948
IL27RA	0.045273615	0.127212947	0.053938	-0.0093	0.090141	0.174012
IL2RA	-0.004649557	0.00311819	-0.05881	0.099591	-0.28228	-0.12667
IL2RB	0.019669451	0.34346806	0.329161	0.018105	-0.05124	-0.33873
IL2RG	0.034607679	0.164139726	0.270845	0.047294	-0.11608	-0.55946
IL3	-0.029048579	-0.034491123	-0.04036	-0.03805	-0.00629	-0.01961
IL31RA	-0.056834673	-0.089497945	-0.246	-0.03112	0.14301	0.381257
IL32	0.01088527	0.095091613	0.529681	0.028933	-0.10129	-0.31464
IL33	-0.070199242	-0.047237283	-0.14728	-0.03791	0.087215	-0.00802
IL34	-0.00146341	-0.081346541	0.045159	-0.00932	-0.05801	-0.07667
IL36A	-0.031978761	-0.042033367	-0.0214	0.02667	-0.07323	-0.01243
IL36B	-0.062102718	-0.049467153	0.127575	0.011269	0.004373	-0.07506
IL36G	-0.014999369	0.000985561	-0.01239	0.005675	-0.00066	-0.01918
IL36RN	-0.040536599	-0.045683666	0.047123	0.014278	-0.01237	0.073177
IL37	-0.042681514	-0.071945272	-0.01351	-0.02547	-0.02758	-0.04686
IL3RA	-0.002109549	-0.101135088	-0.13373	-0.01894	0.021889	-0.10904
IL4	-0.026977545	-0.044049527	-0.01762	-0.00875	-0.07647	-0.03395
IL4I1	-0.014415433	-0.049687455	-0.03105	0.015704	-0.06897	0.110481
IL4R	0.037239008	-0.090087717	-0.13777	-0.01927	0.057932	-0.21327
IL5	-0.028336686	-0.044529763	-0.02559	-0.01366	-0.07097	-0.02765
IL5RA	-0.017121473	-0.045177129	-0.07174	-0.01885	0.040315	-0.156
IL6	-0.009322361	-0.026157811	-0.04561	-0.01165	-0.01239	-0.03897
IL6R	0.005136377	-0.226125795	-0.07468	-0.1228	0.278713	0.281124
IL6ST	-0.016101877	-0.095286668	0.138907	-0.07549	0.159421	0.157959
IL7	-0.014140032	-0.08089658	-0.10402	-0.06267	0.174186	-0.12656
IL7R	0.011336363	0.002913314	0.426841	-0.04957	0.142486	-0.22151
IL9	-0.024899671	-0.032484726	-0.03556	0.076679	-0.10671	-0.0267
IL9R	-0.013064384	-0.00236269	-0.07698	0.066641	-0.20668	-0.05116
ILDR1	-0.02483125	-0.068167438	-0.16437	0.069698	-0.19533	0.068973
ILDR2	-0.02988867	-0.008146616	-0.10166	-0.00668	-0.0036	-0.02663
ILF2	0.032290466	0.066323259	0.119437	0.079532	-0.22615	-0.00245
ILF3	0.009801615	0.045509898	0.227611	-0.02164	-0.13956	-0.02988
ILF3-AS1	0.006043945	-0.042643087	0.359344	-0.02373	0.081852	0.002767
ILK	0.048695677	0.070243837	-0.17946	0.008551	-0.00022	0.133523
ILKAP	0.037942659	0.186258764	0.146081	0.066679	-0.19975	-0.09094
ILVBL	0.012105281	-0.031637882	0.086049	0.066393	-0.19283	0.062341
IMMP1L	0.007178955	0.126737486	0.285245	0.051717	-0.14154	-0.38121
IMMP2L	-0.011180501	-0.128765214	0.201893	-0.03592	0.093396	-0.0606
IMMT	0.043360053	0.084375168	0.093613	0.063069	-0.16152	0.171014
IMP3	0.031479955	0.083341816	0.285483	0.006727	-0.02429	-0.06832
IMP4	0.027969621	0.027788832	0.082208	0.053737	-0.14126	0.107205
IMPA1	0.058910581	0.175003738	0.023314	0.121457	-0.32047	-0.39563
IMPA2	0.000779643	-0.132119726	-0.31864	-0.06433	0.16912	0.319395
IMPACT	0.002039183	-0.059305937	-0.08478	-0.03221	0.113971	0.25889
IMPAD1	0.0344242	0.150711834	-0.16575	0.05483	-0.13957	0.239217
IMPDH1	0.028430683	-0.067256184	-0.21718	-0.02619	0.056384	0.30398

IMPDH2	0.02375371	-0.01104429	0.195755	0.069786	-0.18838	-0.10489
IMPG1	-0.033780248	-0.033562999	0.013235	0.218611	-0.1462	-0.01481
IMPG2	-0.071908021	-0.044236939	-0.05518	-0.0373	0.119006	-0.03744
INA	-0.036160787	-0.088088868	0.100681	-0.00722	0.010938	0.046953
INADL	0.019080274	-0.066301454	0.292429	0.004076	0.085796	-0.37454
INCENP	-0.026409834	0.053034645	0.000358	0.115141	-0.34295	0.018099
INE1	-0.024320959	-0.027214232	-0.0874	-0.01139	-0.06658	-0.01787
INF2	-0.015726179	-0.146603666	0.144189	-0.07716	0.240416	0.001123
ING1	-0.00657284	-0.05671637	-0.1585	-0.01212	-0.02566	0.12528
ING2	0.011636572	-0.078333572	-0.05089	0.03458	-0.10482	0.151716
ING3	0.017088467	-0.0032302	-0.19306	-0.02533	0.042557	0.148216
ING4	0.025749955	-0.017746296	0.044545	-0.09997	0.211093	-0.08727
ING5	0.022321998	-0.148516652	0.122353	-0.00231	0.003847	-0.14067
INGX	-0.02419701	0.033134529	-0.01686	-0.0078	0.039696	0.016581
INHA	-0.028790592	-0.120740574	-0.00378	-0.00231	0.035923	0.030018
INHBA	-0.037462565	-0.048730924	-0.00796	0.056659	-0.12551	-0.07526
INHBA-AS1	-0.067947415	-0.090964202	0.056844	-0.00905	0.026292	-0.02666
INHBB	-0.026182212	-0.02376533	0.049517	0.002245	-0.02227	-0.01276
INHBC	-0.026119599	-0.007057995	-0.00617	-0.00309	-0.03285	-0.00153
INHBE	-0.022847629	-0.037839509	-0.02612	0.09806	-0.17786	0.002021
INIP	0.016974291	0.010554797	-0.1204	0.066551	-0.12651	0.195586
INMT	-0.025235858	-0.000451197	0.002411	0.020465	-0.04174	-0.05618
INO80	0.057718287	0.064036387	4.64E-05	0.014492	-0.04679	0.023932
INO80B	0.00370202	-0.048868587	0.081626	-0.01533	-0.05727	0.021541
INO80C	-0.003540456	-0.039293499	-0.05823	0.006807	-0.0778	-0.09511
INO80D	0.059608018	-0.061149394	0.091684	-0.08131	-0.04843	-0.02237
INO80E	0.022163279	-0.042549665	0.073367	0.019401	-0.0522	-0.01078
INPP1	0.013781657	0.060755074	-0.12034	-0.0037	-0.0173	0.112765
INPP4A	0.022750814	0.151895833	0.16171	0.04287	-0.00694	-0.07885
INPP4B	0.006442106	-0.072822961	0.440189	-0.03517	-0.0292	-0.26753
INPP5A	-0.002573667	-0.054707487	-0.16287	-0.08588	0.2358	0.072971
INPP5B	-0.003690975	0.040715764	-0.01139	0.025782	-0.0911	-0.22559
INPP5D	-0.025538616	0.073653175	-0.09359	-0.0324	0.071452	-0.06127
INPP5E	0.026401381	-0.0110469	0.231941	0.009216	-0.02962	-0.18449
INPP5F	-0.037731595	0.005634913	-0.11596	0.011285	-0.04805	0.072361
INPP5J	-0.022222357	-0.075032744	0.049543	0.000655	-0.05441	-0.05254
INPP5K	0.03646358	0.056428162	0.037033	-0.03114	0.03348	-0.12187
INPPL1	0.016552979	-0.042319346	-0.14623	0.004852	-0.02904	0.406118
INS	-0.034569839	-0.036263669	0.009054	0.012341	-0.00506	0.09931
INSC	-0.018627627	-0.014556756	-0.01554	0.060455	-0.13736	-0.10634
INSIG1	0.012166565	0.064217591	-0.02262	0.038614	-0.10785	-0.09222
INSIG2	0.020106297	0.07250665	-0.0183	-0.01574	0.040957	-0.08831
INSL3	0.003436221	-0.0939717	0.135475	0.002723	0.001966	-0.11795
INSL4	-0.043494574	-0.017343317	0.07113	0.020655	-0.01912	0.017691
INSL5	-0.039036251	-0.01110292	-0.08537	-0.00528	0.037187	-0.03244
INSL6	-0.032062076	-0.028796553	0.019119	0.024285	-0.04356	0.023377
INSM1	-0.017277196	-0.044048835	-0.065	-0.0175	0.007627	-0.03929
INSM2	-0.028608515	-0.033244898	0.05598	-0.02205	0.051362	-0.03537
INSR	-0.019430612	-0.160759433	-0.26075	-0.04263	0.108577	0.507654
INSRR	-0.034445023	-0.059172571	-0.13065	0.023713	-0.04671	-0.06087
INTS1	0.008700805	-0.095699	0.026369	0.01601	-0.0619	-0.06544

INTS10	-0.008723742	-0.029079867	0.035759	0.038272	-0.11469	-0.14698
INTS12	0.044291405	0.004063541	-0.11531	0.044546	-0.14309	-0.10348
INTS2	0.008445068	0.064241796	0.140621	0.086477	-0.19689	-0.04406
INTS3	0.025904067	-0.081180689	-0.04531	0.019974	-0.04998	-0.16021
INTS4	-0.043460096	-0.003995885	-0.0194	0.030874	-0.19565	-0.0295
INTS4L1	-0.044156602	-0.026376381	0.104868	0.0435	-0.09961	0.038807
INTS5	0.019151146	0.080574862	0.215767	0.065434	-0.24507	-0.10947
INTS6	0.00885765	0.147270195	0.030281	0.011919	-0.02985	-0.24117
INTS6-AS1	-0.009158483	-0.054222676	0.244895	-0.05219	0.171953	-0.05915
INTS7	0.004580716	0.08144475	-0.09251	0.057805	-0.11103	-0.06126
INTS8	0.060697067	-0.038512514	-0.07823	0.005424	-0.00845	-0.05329
INTS9	0.02970693	0.025742168	0.005444	0.006774	-0.07575	0.0506
INTU	-0.036570424	-0.058601648	-0.12272	-0.01617	0.047609	0.012573
INVS	-0.035307223	-0.004225436	0.041384	0.017137	-0.05449	-0.05543
IP6K1	0.030522116	0.011895831	-0.03915	0.022853	-0.09539	0.001697
IP6K2	0.039195922	0.054958644	0.099208	-0.0306	0.014774	-0.32924
IP6K3	-0.033820937	-0.028185859	0.134774	0.045649	-0.11627	0.055271
IPCEF1	0.011642599	0.058313608	0.247942	-0.05096	0.123729	-0.00944
IPMK	-0.014353919	-0.081986058	-0.24444	-0.04099	0.087861	0.179609
IPO11	-0.026411032	-0.046191263	0.148896	0.001748	-0.00044	-0.11138
IPO13	0.020300841	-0.004600319	0.007366	0.027081	-0.18254	-0.03061
IPO4	0.01559156	-0.045974739	0.067734	0.063942	-0.16137	-0.13835
IPO5	-0.004608483	0.009689405	0.072495	0.042497	-0.18501	0.072595
IPO5P1	-0.038447314	-0.011620161	0.103066	-0.02207	0.014137	-0.17363
IPO7	0.030436873	0.058472298	0.129074	0.030463	-0.09016	0.216426
IPO8	0.044951317	0.063542899	-0.02102	0.009079	-0.07654	-0.05397
IPO9	-0.003105222	0.026580128	0.280423	0.058695	-0.13469	-0.06105
IPO9-AS1	-0.022124144	-0.033080899	-0.11651	-0.02615	0.042107	0.112081
IPP	-0.002286138	0.164151743	0.096399	0.072922	-0.06749	-0.31767
IPPK	-0.015368357	-0.02361764	-0.01545	0.04017	-0.22883	-0.14945
IQCA1	-0.031941635	0.005059621	-0.00416	-0.00808	0.080097	0.056877
IQCB1	0.021482296	-0.010163833	-0.00836	0.050746	-0.14935	-0.1245
IQCC	-0.018160275	-0.089998829	0.08428	0.027783	-0.10839	-0.02861
IQCD	-0.025036185	-0.092714686	-0.16057	0.050463	-0.14223	0.13533
IQCE	-0.001212236	-0.05217834	-0.21094	-0.05458	0.168258	0.247667
IQCF1	-0.033671276	-0.060206378	0.037556	0.003237	-0.03894	-0.04473
IQCF2	-0.029252814	-0.078718176	-0.05827	-0.01314	0.062809	-0.0263
IQCF3	-0.037089921	-0.009530593	-0.07386	0.025599	-0.04098	0.118055
IQCF4	-0.075615964	-0.035285339	-0.14438	-0.01526	0.033862	0.00509
IQCF5	-0.03830192	-0.050513054	-0.12103	-0.02568	0.09068	0.006526
IQCF5-AS1	-0.034926833	-0.003715937	-0.07559	0.014935	-0.01836	-0.04496
IQCF6	-0.029847841	-0.050165617	-0.08312	0.043584	-0.10051	0.081005
IQCG	-0.020529496	0.012407897	0.059606	-0.02351	0.032006	0.021215
IQCH	-0.096807599	-0.006230271	0.0405	0.011871	-0.05622	-0.06102
IQCH-AS1	-0.001407314	0.019589464	0.219368	-0.01552	0.055109	0.006925
IQCJ-SCHI	-0.031283084	-0.083863264	-0.04727	0.026574	-0.06704	-0.01883
IQCK	-0.031385315	-0.121990057	0.115424	-0.01107	0.079261	-0.11001
IQGAP1	0.067689746	0.207958652	-0.33728	0.031902	-0.04323	0.141544
IQGAP2	0.024269548	0.156320685	-0.04287	0.023366	-0.03901	0.111047
IQGAP3	-0.025883705	-0.016268079	-0.0326	-0.0024	-0.1424	-0.00276
IQSEC1	0.035075364	-0.026075951	-0.22589	-0.04022	0.110493	0.027719

IQSEC2	-0.025135567	-0.073262209	-0.17041	-0.00716	0.023947	0.258667
IQSEC3	-0.013884724	-0.076308145	0.131339	0.056958	-0.04387	0.024223
IQUB	-0.021743576	-0.024274032	-0.00528	0.034615	-0.1062	-0.07005
IRAK1	0.045857724	-0.02355599	-0.15714	0.027383	-0.10471	0.171102
IRAK1BP1	-0.014037416	0.025604333	0.214487	-0.00702	0.043103	-0.17444
IRAK2	-0.010086462	0.007214836	-0.04524	0.019233	-0.02044	-0.04899
IRAK3	-0.026618096	-0.118999553	-0.27878	-0.08377	0.256611	0.728821
IRAK4	0.029864539	0.046520468	-0.06512	0.002917	0.013078	-0.08125
IREB2	-0.03874687	0.034635984	-0.04985	0.042177	0.055504	0.051583
IRF1	0.037231086	0.097767793	-0.08396	-0.0356	0.102752	0.101608
IRF2	0.022546918	0.01896545	-0.13736	0.000559	-0.00989	-0.00199
IRF2BP1	-0.005118837	-0.084205593	0.115901	0.029319	-0.128	-0.0894
IRF2BP2	0.067096289	-0.07268145	0.032379	-0.0295	0.04527	0.059793
IRF2BPL	0.04899379	0.150810095	-0.14277	0.029376	-0.06209	-0.17921
IRF3	0.029867809	0.029304987	0.164224	0.004422	-0.02334	-0.13229
IRF4	-0.01103453	-0.040818904	-0.04073	0.033053	-0.10519	-0.024
IRF5	0.02133194	-0.063972785	-0.32905	-0.01212	0.075145	0.450758
IRF6	-0.077551529	-0.008058736	-0.01605	-0.04015	0.017436	-0.09724
IRF7	0.023530922	-0.034201862	-0.30777	-0.05712	0.155825	0.034829
IRF8	0.019108389	0.002707685	-0.3262	-0.06168	0.172654	0.059985
IRF9	0.046733053	0.067409762	-0.14429	-0.03879	0.122127	-0.13732
IRG1	-0.01225185	-0.028650392	-0.01949	-0.00661	-0.00023	-0.00299
IRGC	-0.03401688	-0.063529762	-0.07452	0.020616	-0.02168	0.049064
IRGM	-0.04550331	-0.00088463	0.17098	-0.05812	0.154766	-0.06559
IRGQ	0.009736609	-0.013383122	-0.21219	0.070604	-0.17955	0.172827
IRS1	0.010221914	-0.12714854	0.143003	-0.04164	0.058038	0.010733
IRS2	0.021787004	-0.062717403	-0.15489	-0.06035	0.163153	0.277971
IRS4	-0.034804068	-0.069315157	0.017711	0.023786	-0.05114	0.08325
IRX1	-0.049610868	-0.03293646	-0.09257	-0.02172	0.041762	0.092521
IRX2	-0.052584169	-0.04063851	-0.0014	0.030698	-0.08796	-0.04115
IRX3	-0.030137853	-0.009162688	-0.02949	-0.08025	0.183947	-0.03456
IRX4	-0.040583805	-0.011172241	-0.11998	0.004425	0.010993	0.067853
IRX5	-0.034412939	-0.036011897	-0.03158	0.064139	-0.16388	-0.03182
ISCA1	0.041890448	0.105893603	0.186681	0.003797	-0.03825	-0.00039
ISCA2	0.037704275	-0.039539599	0.098478	0.025326	-0.09094	0.032617
ISCU	0.090105834	-0.008564923	0.180974	0.042796	-0.05566	-0.41201
ISG15	0.02908262	0.047019904	-0.02635	0.040909	-0.09214	0.09152
ISG20	0.023696066	0.060891799	0.236146	-0.0249	0.07168	-0.44666
ISG20L2	0.046375763	0.171710752	0.085399	0.070549	-0.15158	-0.09974
ISL1	-0.021337781	0.00713381	-0.05404	-0.00598	0.011675	-0.08059
ISL2	-0.016845676	-0.062212409	-0.12867	0.077356	-0.22083	0.112977
ISLR	-0.03321129	-0.061348265	0.088741	0.021841	-0.05154	-0.01346
ISLR2	-0.029257697	0.016759701	-0.0379	0.030015	-0.06681	0.026239
ISM1	-0.027821858	-0.054331072	0.040225	-0.03405	0.059624	-0.04486
ISM1-AS1	-0.044530999	-0.078015391	-0.11095	-0.01097	0.063025	0.0493
ISM2	-0.034513609	-0.015761461	-0.0017	-0.00287	-0.01052	-0.00215
ISOC1	0.027226955	0.148895193	0.057103	0.068095	-0.2397	-0.19768
ISOC2	0.000443875	-0.061955614	0.003705	0.120041	-0.34332	0.049134
ISPD-AS1	-0.089211833	-0.129404073	-0.02279	-0.01096	0.027419	-0.08592
IST1	0.06148351	0.086194344	-0.08979	0.004959	0.050283	-0.29834
ISX	-0.043790189	0.006537467	-0.03433	-0.00345	0.039684	-0.01049

ISY1	0.055016517	0.114853889	0.001627	0.005233	-0.098	-0.01244
ISYNA1	-0.012912191	0.00993316	0.101996	0.004811	0.104556	-0.09618
ITCH	0.033460493	0.117461923	-0.13161	0.018432	0.014399	-0.09148
ITFG1	0.01492861	0.057923953	-0.09948	-0.01347	0.079985	0.115116
ITFG2	0.01855148	-0.067485864	0.118488	-0.01694	0.014687	-0.16155
ITFG3	0.012346254	-0.078071507	-0.05558	0.020815	-0.09536	0.141701
ITGA1	-0.047784135	-0.074911183	-0.03781	0.227698	-0.08342	-0.06538
ITGA10	-0.028365516	0.004897892	0.08075	-0.02203	0.007436	-0.27009
ITGA11	-0.035298232	-0.030846561	0.007176	0.010064	-0.01188	-0.01105
ITGA2	-0.017148675	-0.009996584	-0.02074	0.0632	-0.15844	-0.02323
ITGA2B	-0.01448063	-0.041171828	-0.04907	-0.21206	0.74692	0.064095
ITGA3	-0.018852042	-0.065810813	0.061412	0.082202	-0.23814	-0.0309
ITGA4	0.012831971	0.013969308	0.0059	-0.03718	0.087501	0.124383
ITGA5	0.029801206	0.04488998	-0.15866	-0.06708	0.183175	0.177206
ITGA6	-0.000998601	0.069571081	0.402606	-0.07713	0.205621	-0.34722
ITGA7	-0.027326771	-0.043854533	-0.09352	0.041072	-0.12185	0.033212
ITGA8	-0.060831546	-0.047002574	0.048914	-0.00798	0.008813	-0.06663
ITGA9	-0.029901804	-0.076052478	0.033029	0.031204	-0.06592	0.056809
ITGA9-AS:	-0.043104318	-0.033911516	0.096808	-0.03435	0.12647	0.000728
ITGAD	-0.030530945	0.032198074	-0.07775	0.021046	-0.10822	-0.11785
ITGAE	0.053763565	0.031838568	-0.05653	0.018253	-0.0663	0.114286
ITGAL	0.025443631	0.14321456	0.115373	-0.00319	0.009944	0.042115
ITGAM	0.023712174	0.148824185	-0.21799	-0.03992	0.094221	0.324113
ITGAV	0.01482482	0.060700395	-0.06858	-0.09125	0.228461	0.228706
ITGAX	0.007210007	0.045358318	-0.34307	-0.02976	0.079664	0.200002
ITGB1	-0.022105929	0.077675083	0.018663	-0.04927	0.082929	-0.01812
ITGB1BP1	0.020052239	0.047353024	0.119772	0.017469	-0.04275	-0.00846
ITGB1BP2	-0.024901937	-0.02834126	0.071535	0.071214	-0.18448	-0.01493
ITGB2	0.017530926	0.098474059	-0.13195	-0.03907	0.155204	0.256871
ITGB2-AS:	0.014548127	-0.079412216	0.049108	-0.08348	0.203122	0.090062
ITGB3	-0.002061444	-0.038760857	-0.08048	-0.24746	0.638632	0.090701
ITGB3BP	0.016399468	0.051992764	0.183761	0.026793	-0.06612	-0.05709
ITGB4	-0.064529083	-0.032882892	-0.12099	-0.01139	-0.00854	0.03427
ITGB5	-0.022575649	-0.034621325	-0.01288	-0.11103	0.31976	0.124582
ITGB7	0.022810276	0.109768171	0.195288	0.008285	-0.04345	-0.41532
ITGB8	-0.012874452	-0.026968878	0.010486	-0.02096	-0.00498	-0.08014
ITGBL1	-0.0905131	-0.029053914	0.03876	0.033306	0.046283	0.036187
ITIH1	-0.027609689	-0.126346839	-0.09421	0.002323	0.024099	0.259829
ITIH2	-0.025367598	-0.022588422	0.130758	0.022021	-0.05965	-0.05158
ITIH3	-0.037229259	-0.051795363	-0.06882	0.004522	0.010413	0.041566
ITIH4	-0.006455241	-0.100447786	0.061143	-0.08845	0.293439	0.292838
ITIH5	-0.057551602	-0.057959074	0.053567	-0.0006	0.018639	-0.03256
ITIH6	-0.040176701	-0.095794908	-0.07053	0.001871	0.017106	0.007197
ITK	0.009901142	0.144837817	0.517065	-0.01777	0.042159	-0.35087
ITLN1	-0.028733603	-0.021897377	-0.17793	-0.05147	0.174383	0.221758
ITM2A	0.008768769	0.096463295	0.401483	-0.01251	0.035938	-0.49565
ITM2B	0.04831761	0.007455577	-0.08019	-0.02511	0.093612	0.042701
ITM2C	0.011654741	-0.144977008	-0.0278	-0.01183	0.014725	-0.32747
ITPA	0.056251497	0.062057533	0.108716	0.064638	-0.18478	0.124001
ITPK1	-0.019860475	-0.127390057	-0.02306	0.002091	-0.09021	0.003723
ITPK1-AS:	-0.034953871	-0.025369232	-0.12993	-0.00457	0.001855	-0.02333

ITPKA	-0.029083495	-0.042595894	-0.15759	-0.00145	0.008414	-0.03774
ITPKB	-0.044138326	-0.090937385	0.208795	-0.04923	0.145294	-0.15728
ITPKB-IT	-0.017897131	-0.106279749	0.027563	-0.0419	0.032438	-0.06774
ITPKC	-0.013367745	-0.019758421	-0.06311	0.015826	-0.07377	0.124313
ITPR1	0.009543331	-0.023656034	-0.06242	0.037683	-0.09183	-0.11742
ITPR1-AS	-0.012012201	-0.014824882	-0.00212	-0.01443	0.044458	0.065449
ITPR2	0.024709605	0.029959555	-0.13729	-0.03519	0.062284	0.007382
ITPR3	0.004571239	0.06134296	0.295016	0.056241	-0.09834	-0.28559
ITPRIP	0.018144637	0.035782885	-0.23185	-0.02283	0.067579	0.076545
ITPRIPL1	-0.012628169	0.212698423	0.132662	0.11603	-0.30477	-0.12149
ITPRIPL2	-0.014134672	-0.155254354	-0.14965	-0.07254	0.079729	0.511056
ITSN1	-0.10660089	-0.128769013	-0.04757	-0.08107	0.15728	0.216486
ITSN2	0.042986806	-0.007928537	-0.23446	-0.04495	0.129984	-0.18319
IVD	0.019260264	-0.004957962	0.059252	0.016898	-0.05641	0.065326
IVL	-0.032318019	-0.042758016	0.122308	-0.00534	0.025515	-0.01296
IVNS1ABP	0.042012225	0.202893843	-0.14861	0.049105	-0.12023	-0.04921
IWS1	0.043805507	0.023456739	-0.19158	0.03226	-0.11496	-0.00534
IYD	-0.127348249	-0.026399834	-0.06968	-0.03233	0.099766	0.03429
IZUMO1	-0.032564936	-0.027116555	-0.16815	0.009178	-0.02322	0.113288
IZUMO2	-0.022446463	-0.03118226	0.025846	0.02213	-0.05346	-0.0598
IZUMO4	-0.022678938	-0.162955033	0.104902	0.021528	-0.0337	0.043806
JADE1	0.004568032	0.10678955	0.073322	0.001939	5.52E-05	-0.32298
JADE2	-0.050765359	0.042094957	0.090368	0.03289	0.097699	-0.22541
JADE3	-0.012868369	-0.085048866	-0.17642	-0.03645	0.076394	-0.08744
JAG1	0.049323004	0.059833629	-0.11161	-0.00442	-0.01819	0.1642
JAG2	-0.010686845	0.043780285	-0.05631	0.033385	-0.14059	-0.02594
JAGN1	0.0479019	0.047821139	0.046526	0.076824	-0.22837	0.199582
JAK1	0.039979773	0.235313105	0.072134	-0.03284	0.096239	-0.39323
JAK2	-0.005934181	-0.038524288	-0.30416	-0.04254	0.152425	0.319275
JAK3	-0.002513157	-0.033115439	0.062824	0.09147	-0.13844	-0.1352
JAKMIP1	0.002009723	0.088549119	0.347787	0.074753	-0.23567	-0.20297
JAKMIP2	-0.008202561	0.28072627	0.189523	-0.02254	0.08214	-0.28465
JAKMIP2-A	-0.031608856	-0.042590508	-0.05273	0.009797	-0.01951	0.05061
JAKMIP3	-0.023812311	-0.050182149	-0.09171	0.016873	-0.0368	0.058001
JAM2	-0.038647929	-0.081359828	-0.08504	0.047873	-0.13678	-0.10163
JAM3	-0.020082943	-0.054432347	-0.07797	-0.14201	0.352125	-0.10289
JARID2	0.026313668	0.080292598	-0.19557	-0.0025	0.034799	0.058243
JARID2-AS	-0.021759094	-0.034186736	-0.05315	-0.0329	0.096624	0.028476
JAZF1	0.018031688	0.150035394	-0.3442	-0.08587	0.239235	0.251259
JAZF1-AS	-0.034955232	-0.012731543	-0.21853	-0.05108	0.151896	0.444956
JDP2	-0.009599959	-0.145808912	-0.31461	-0.06707	0.166976	0.385729
JHDM1D-AS	-0.023663684	-0.096791989	-0.01907	-0.03456	0.032977	-0.02253
JKAMP	0.032845764	0.021806383	-0.23088	0.01449	-0.00758	0.187036
JMJD1C	-0.0059283	-0.109759278	-0.14702	-0.06151	0.214218	-0.03894
JMJD1C-AS	-0.028642631	0.017121233	-0.08221	0.007256	-0.01473	0.023023
JMJD4	0.011428236	-0.057516771	0.047387	0.105518	-0.23722	0.000849
JMJD6	0.016845301	-0.02167514	-0.04284	0.02979	-0.07663	-0.06689
JMJD8	0.018794718	-0.066529025	0.032633	-0.0673	0.125595	0.042859
JMY	0.012663141	0.077180903	0.259448	0.005907	0.028331	-0.08647
JOSD1	0.057735747	0.156659943	0.179454	0.069225	-0.15724	-0.23947
JOSD2	0.009421595	-0.111734555	-0.0808	0.06423	-0.26084	0.291252

JPH1	-0.066528987	-0.053609094	0.050258	0.016804	-0.02035	-0.03407
JPH2	-0.014234206	0.005304154	-0.01367	-0.08733	-0.05832	-0.07454
JPH3	-0.032321633	-0.074252109	-0.09606	0.021106	0.012644	0.037353
JPX	0.002314317	0.039769543	0.015423	0.01779	-0.01825	0.029371
JRK	0.015033206	-0.003305022	0.154787	0.077271	-0.23172	-0.23249
JRKL	0.009905548	0.041256802	-0.05919	-0.00742	0.03966	-0.00838
JSRP1	-0.01461968	-0.060174287	-0.07948	0.002061	-0.02896	0.016002
JTB	0.118175209	0.135303608	0.062872	0.086107	-0.19268	-0.18908
JUN	-0.004050842	-0.126093092	0.034231	0.054683	-0.17285	0.148242
JUNB	0.023601193	-0.070720571	-0.09598	0.011449	-0.02186	0.175041
JUND	-0.005895523	-0.108100728	0.018742	0.035865	-0.06184	0.021308
JUP	-0.005749725	-0.135781326	-0.26257	-0.09907	0.260165	0.306889
KAAG1	-0.035744164	-0.044151687	0.015513	0.029707	-0.06095	-0.07489
KAL1	-0.037105682	-0.097044022	0.02301	-0.00521	0.036462	0.146122
KALRN	-0.080361357	-0.067106926	0.14455	-0.03794	0.014663	-0.08757
KANK1	0.059629138	-0.091627637	0.122626	0.018497	0.104895	-0.20768
KANK2	-0.05392754	-0.01545865	-0.01187	0.031977	-0.10241	0.147853
KANK3	-0.012958131	0.159099025	0.007049	0.011417	-0.00349	-0.13005
KANK4	-0.029249928	-0.070364647	0.154953	0.028277	-0.0736	-0.00969
KANSL1	0.041373518	0.114493622	0.133105	0.016957	-0.02853	-0.36825
KANSL1-AS	0.003837986	0.040538707	0.165932	-0.03984	0.114451	-0.09517
KANSL1L	-0.027523005	-0.057740966	-0.12569	-0.02732	0.245097	0.232377
KANSL2	0.050204713	0.021093058	0.117774	0.034524	-0.12266	-0.21663
KANSL3	-0.060014508	-0.071611768	0.023525	-0.02364	-0.0586	-0.06151
KARS	0.071378609	0.063780062	0.165546	0.076761	-0.1843	0.023302
KAT2A	0.025000241	-0.094667821	0.186172	0.012486	-0.04837	-0.23751
KAT2B	0.034407611	0.160569401	0.044308	0.041989	-0.09676	-0.29982
KAT5	0.023954536	0.003248129	-0.00899	-0.02325	-0.04078	-0.05818
KAT6A	0.007841426	0.10149676	0.084975	-0.0417	0.047361	-0.33602
KAT6B	0.008580485	0.156400598	0.259182	-0.04045	0.096912	-0.27435
KAT7	0.05280677	0.098482196	0.072758	0.031448	-0.12709	-0.23169
KAT8	0.026516908	-0.144347071	-0.08094	-0.00515	0.018173	0.112113
KATNA1	0.057798598	0.108664703	0.020793	0.074811	-0.24253	-0.08201
KATNAL1	-0.001713642	0.056162002	0.030358	0.04506	-0.12347	-0.18606
KATNAL2	-0.051441728	-0.002086075	-0.0691	0.007579	0.002645	0.065122
KATNB1	-0.01604184	0.051260018	-0.00048	0.056041	-0.04541	0.078345
KATNBL1	-0.001918862	0.00497543	-0.14805	0.048124	-0.06705	-0.16968
KAZALD1	-0.083891439	-0.044732768	-0.07318	0.077526	-0.20685	0.014927
KAZN	-0.044508	-0.03171839	-0.15867	0.009128	-0.05527	-0.04009
KB-1000E4	-0.067904006	-0.023235733	-0.01403	0.006624	-0.04109	0.015933
KB-1410C5	-0.045543776	0.00742362	-0.06445	0.017341	-0.01429	0.091227
KB-1568E2	-0.032502526	0.00208842	0.179932	0.003181	-0.00315	-0.02918
KB-1836B5	-0.040397859	-0.049228328	-0.15001	0.032218	-0.06979	-0.03234
KB-431C1.	-0.001575146	-0.167099841	-0.40538	-0.04495	0.120058	0.144184
KB-431C1.	-0.025637429	-0.068388453	-0.05136	-0.01793	0.067227	-0.10743
KBTBD11	-0.003184298	-0.156468819	-0.09904	-0.0646	0.174082	0.470892
KBTBD12	-0.041165477	-0.042703343	0.034812	-0.00954	0.05128	0.009536
KBTBD2	0.021755122	-0.001108289	-0.09456	0.001081	-0.00668	-0.01498
KBTBD3	0.0044851	0.082940206	0.127034	0.006437	0.012358	-0.00452
KBTBD4	-0.000545106	0.084592005	0.176753	0.038651	-0.10293	-0.37848
KBTBD6	-0.008372913	0.131377186	0.130592	0.031315	-0.05587	-0.11109

KBTBD7	-0.001915544	0.072682438	-0.10564	-0.02808	0.019927	0.073905
KBTBD8	0.004878054	0.016915937	-0.16401	0.02839	-0.09056	-0.13732
KCMF1	-0.049664946	0.131703055	0.092587	0.030297	0.03023	-0.26635
KCNA1	-0.03537082	0.018217823	-0.00411	0.016196	-0.02677	0.005972
KCNA10	-0.03123337	-0.101714207	-0.06618	-0.00813	0.02248	0.086444
KCNA2	-0.022859985	-0.025459559	0.056286	0.036959	-0.10606	-0.002
KCNA3	0.006492177	-0.028922644	0.408649	0.010737	-0.01326	-0.30291
KCNA4	-0.042151848	9.10E-05	0.129374	0.017832	-0.04356	0.038801
KCNA5	-0.009773929	-0.077007318	-0.05861	-0.00478	0.006744	-0.18123
KCNA6	-0.029777621	-0.037735541	-0.03406	0.02058	-0.06951	-0.08961
KCNA7	-0.0483694	-0.136580796	-0.11612	-0.00915	0.061436	0.03845
KCNAB1	-0.052102978	-0.15478192	-0.03946	0.038659	-0.03063	-0.08026
KCNAB2	0.023931663	0.115558569	0.091497	0.025817	-0.08463	0.111569
KCNAB3	-0.027710626	-0.057543854	-0.07126	-0.01799	0.045811	-0.04087
KCNB1	-0.060372577	-0.065271232	0.015782	-0.01145	-0.01064	-0.08092
KCNB2	-0.052654355	-0.058597853	-0.08284	-0.00181	0.021979	-0.00433
KCNC1	-0.05857134	0.00261118	0.049202	0.007279	0.006003	-0.13838
KCNC2	-0.04031108	-0.086044187	-0.13662	-0.00452	0.027143	0.118568
KCNC3	-0.043380901	-0.075860625	-0.08194	0.009369	-0.04858	-0.02525
KCNC4	-0.007331148	-0.1007627	-0.04321	0.072375	-0.14132	0.07037
KCND1	-0.022942484	-0.051297522	-0.13543	0.043452	-0.13044	0.039689
KCND2	-0.038123882	-0.04556188	0.008031	0.132139	-0.07486	-0.06512
KCND3	0.008612327	-0.082296905	-0.06189	-0.03331	0.210918	-0.01895
KCNE1	-0.029902423	-0.103448429	-0.20065	-0.04608	0.173663	0.505915
KCNE1L	-0.024009032	0.011340781	-0.12265	0.006382	-0.00674	0.123215
KCNE2	-0.036256291	-0.059358111	-0.01246	0.060378	-0.17627	0.027986
KCNE3	0.018154544	-0.117446568	-0.28337	-0.01733	0.219813	0.544277
KCNE4	-0.010098154	-0.062640446	-0.00976	0.105693	-0.16599	0.0289
KCNF1	-0.019260327	-0.09473663	-0.10831	0.015267	-0.08953	0.024194
KCNG1	-0.025298982	-0.106075993	-0.12114	-0.04082	0.098821	-0.07069
KCNG2	-0.02354649	0.03508034	0.000519	0.032778	-0.06052	-0.04673
KCNG3	-0.037052977	-0.097081344	0.09581	-0.0057	0.038456	-0.0356
KCNG4	-0.033461694	-0.085193128	-0.03744	-0.01408	0.045522	0.018505
KCNH1	-0.035744064	-0.033540606	-0.0566	0.021448	-0.04585	0.029767
KCNH2	-0.020671773	-0.136148187	-0.01674	-0.0341	0.060784	-0.19376
KCNH3	-0.020805666	-0.13193884	0.0431	-0.04545	0.032606	0.033685
KCNH4	-0.033729667	-0.007465361	-0.09229	0.007084	-0.03459	0.115548
KCNH5	0.05414646	-0.043482273	-0.0418	0.112556	0.0614	-0.10292
KCNH6	-0.036454011	-0.014202263	-0.11866	0.007123	0.017691	0.147328
KCNH7	-0.024146889	-0.035768695	0.012801	-0.02299	0.022609	-0.06235
KCNH8	-0.009129218	-0.087929989	-0.12709	0.000389	0.00134	-0.21699
KCNIP1	-0.036671692	-0.063949664	0.019048	-0.00619	0.037554	-0.02807
KCNIP2	-0.041783377	-0.120504196	-0.13336	-0.01207	0.071164	0.085873
KCNIP2-AS	-0.015075614	-0.074064241	-0.00254	-0.01318	-0.05723	-0.06636
KCNIP3	-0.03589807	-0.105549146	-0.01443	0.047912	-0.15736	-0.0595
KCNIP4	-0.048717682	-0.028560696	0.080581	0.044816	-0.10349	-0.02632
KCNJ1	-0.05652514	-0.037388406	-0.03843	0.019869	-0.02561	-0.04274
KCNJ10	-0.050979932	-0.076427874	-0.12394	-0.01509	0.079366	0.113301
KCNJ11	-0.035612577	-0.026045122	-0.01006	0.047386	-0.10659	0.011937
KCNJ13	-0.066595959	-0.042557806	-0.03943	-0.01628	0.040997	0.024603
KCNJ14	-0.03434812	-0.046201165	-0.09966	0.048206	-0.13742	0.071553

KCNJ15	-0.021935163	-0.097658395	-0.23832	-0.05889	0.111299	0.263921
KCNJ16	-0.023547179	-0.029447635	0.004294	0.189593	-0.21909	-0.02103
KCNJ2	-0.003964919	-0.059942826	-0.20513	-0.05388	0.132565	0.21995
KCNJ2-AS1	-0.037053083	-0.037969699	-0.09454	-0.01711	0.053498	-0.03851
KCNJ3	-0.037324579	-0.012125258	0.024696	0.089066	-0.19218	0.01486
KCNJ4	-0.037847369	-0.045463767	-0.13468	-0.01846	0.060449	0.00642
KCNJ5	0.025404642	-0.08252622	-0.00109	-0.03601	-0.07482	-0.0218
KCNJ6	-0.083964791	-0.039207581	0.037136	-0.0419	0.107351	-0.00783
KCNJ8	-0.0565714	-0.061592349	0.012146	0.002996	0.020527	-0.10392
KCNJ9	-0.040316285	-0.070624523	-0.02971	0.005446	0.005303	0.002478
KCNK1	-0.025796038	-0.061995498	-0.06732	0.048206	-0.13424	-0.11096
KCNK10	-0.02547305	-0.050217304	-0.07779	-0.01792	-0.00591	-0.19885
KCNK12	-0.029376218	0.066964013	0.024279	0.053199	-0.13309	0.10885
KCNK13	-0.037984844	-0.068359326	-0.02149	-0.01931	0.08308	0.112442
KCNK15	-0.05172552	0.003960446	-0.04988	0.012018	-0.01651	0.090063
KCNK16	-0.018500471	-0.093399484	-0.00059	-0.01674	-0.0333	-0.10355
KCNK17	-0.006136261	-0.069155821	-0.03844	-0.02029	0.062609	-0.16042
KCNK2	-0.034006764	-0.062217791	-0.09437	0.032414	-0.07138	-0.09016
KCNK3	-0.030235675	-0.066266681	0.126751	-0.01495	0.034086	-0.02605
KCNK4	-0.03001807	-0.035200403	-0.14154	0.028594	-0.04655	0.074526
KCNK5	-0.010629755	-0.022924483	-0.00341	0.03503	-0.13217	-0.00058
KCNK6	0.006891521	-0.119572659	-0.13819	-0.07236	0.197315	0.282988
KCNK7	-0.005272067	-0.106282547	-0.01045	0.046891	-0.0952	-0.00223
KCNK9	-0.031037054	0.003442419	-0.09872	0.068466	-0.19699	-0.04262
KCNMA1	-0.087827368	-0.031493187	0.10899	0.00941	-0.04401	-0.05489
KCNMB1	-0.012505824	-0.105318223	-0.21049	-0.09499	0.267021	0.546465
KCNMB2	-0.058683956	-0.047457699	0.004154	-0.00056	0.001403	0.04192
KCNMB3	-0.023339875	-0.062868282	-0.03557	-0.03179	0.066318	0.008738
KCNMB4	-0.043931694	-0.07421305	0.058433	0.02962	-0.03862	-0.03043
KCNN1	-0.022614946	-0.035840331	0.013148	0.012958	-0.1304	-0.09516
KCNN2	-0.029945467	-0.048907722	-0.07524	-0.02065	0.025372	-0.01708
KCNN3	-0.037384053	-0.074363671	-0.03888	0.040808	-0.08753	0.136138
KCNN4	-0.004928393	-0.17630425	0.099515	0.001654	-0.02364	0.203807
KCNQ1	0.008636947	-0.262600831	-0.09353	-0.0642	0.129846	0.338987
KCNQ1-AS1	-0.046565527	-0.08445355	0.034297	0.023759	-0.03535	0.074345
KCNQ1DN	-0.028089404	-0.093022472	-0.03237	0.030343	-0.11699	-0.01397
KCNQ2	-0.031403184	-0.07206118	0.078584	0.014021	0.003239	-0.03838
KCNQ3	-0.014091385	-0.092143562	0.047402	-0.00032	-0.0414	-0.00101
KCNQ4	-0.042002933	-0.073258624	0.018198	-0.02253	0.067657	0.001719
KCNQ5	-0.016806837	0.102512685	0.167922	0.065755	-0.1466	-0.21888
KCNRG	-0.018701341	-0.031106403	-0.03574	-0.02611	0.091495	-0.03711
KCNS1	-0.020383299	-0.066142772	0.033445	-0.00907	-0.05004	-0.04258
KCNS2	-0.066566599	-0.003128547	0.037605	-0.01584	-0.08381	-0.14256
KCNS3	-0.042269368	-0.053620905	-0.03111	0.03968	-0.08862	0.084333
KCNT1	-0.046634217	-0.058327387	-0.03833	-0.01977	0.018002	0.06989
KCNT2	-0.040895445	-0.047095205	0.054832	-0.01499	0.008945	-0.04598
KCNU1	-0.037742737	-0.069113113	0.059454	0.005554	0.009801	-0.08564
KCNV1	-0.05175249	-0.010226427	-0.17198	0.023486	-0.08408	0.018038
KCNV2	-0.051319937	-0.084211247	-0.02	-0.01007	0.029717	-0.07755
KCP	-0.096403478	-0.068434052	-0.04798	0.057074	-0.16604	0.108448
KCTD1	-0.032281522	0.001961486	0.004369	0.038786	-0.13652	-0.05231

KCTD10	0.039779011	0.37343309	-0.09311	0.01509	-0.08142	-0.13156
KCTD11	-0.005416306	0.113718878	-0.07988	-0.03453	0.067588	0.222836
KCTD12	0.009559649	-0.181250168	-0.29351	-0.10826	0.323794	0.479177
KCTD13	0.014862902	-0.056782648	0.041117	0.012289	-0.02122	-0.0887
KCTD15	-0.06142627	-0.172317748	-0.15507	-0.08963	0.149259	0.530009
KCTD16	-0.063160121	-0.086615882	-0.02369	0.008121	-0.02914	-0.07169
KCTD17	-0.013913833	-0.054645734	0.02958	0.00324	-0.12754	-0.04886
KCTD18	0.028416706	0.008344391	-0.01376	-0.05195	0.042012	-0.1552
KCTD19	-0.014858845	-0.073610675	-0.03925	0.019212	-0.04032	-0.1128
KCTD2	0.036371663	-0.037379245	-0.03857	0.002613	-0.0801	-0.13128
KCTD20	0.029484689	0.009138079	-0.25635	-0.02089	0.000835	0.416688
KCTD21	0.002232351	-0.015712397	-0.17798	-0.01695	0.068678	0.369651
KCTD3	-0.011050507	-0.079137391	-0.09929	0.028148	-0.02831	0.179925
KCTD4	-0.079949609	-0.097897292	-0.03665	-0.00655	0.042935	0.011356
KCTD5	0.016677637	-0.05958652	-0.03995	-0.00198	-0.07859	-0.01136
KCTD6	0.007138581	0.010367368	-0.02102	-0.00632	0.006814	0.230138
KCTD9	0.032118663	0.19483004	0.074567	0.093671	-0.28131	-0.22406
KDELC1	-0.007110405	0.05582445	0.052189	0.139209	-0.37424	-0.11034
KDELC2	0.010323188	0.142883363	0.165444	0.000728	0.009995	-0.30833
KDELR1	0.041243623	-0.027259471	-0.04495	0.056191	-0.13343	0.413179
KDELR2	0.023592694	0.033707736	-0.23063	0.043214	-0.17061	0.174207
KDELR3	0.005518489	-0.068306976	0.054298	0.046593	-0.07561	-0.10795
KDF1	-0.026733395	-0.112563008	0.257941	-0.02409	0.097047	-0.27881
KDM1A	0.031343334	-0.05441084	0.086895	0.082262	-0.27939	-0.15773
KDM1B	0.001898996	-0.104378741	-0.31501	-0.05919	0.191766	0.460204
KDM2A	0.120874095	-0.018220116	0.058386	-0.01976	-0.01626	-0.2691
KDM2B	0.035090271	0.042446166	0.032207	0.008311	-0.01806	0.026121
KDM3A	0.026510577	0.096131044	0.180994	-0.00538	0.026102	-0.41811
KDM3B	0.075633057	-0.008976081	-0.29793	0.036077	-0.08026	-0.05831
KDM4A	0.032263897	0.080382841	-0.00999	-0.0115	-0.06925	-0.04713
KDM4B	0.018007115	-0.082368647	-0.15661	-0.01973	-0.02215	-0.07197
KDM4C	0.003629939	-0.005448177	0.093983	0.015601	0.065353	-0.19691
KDM4D	-0.043141157	-0.062426039	0.102599	0.017741	-0.07921	-0.06261
KDM5A	0.012133061	0.131307073	0.000402	-0.0554	0.13201	-0.11818
KDM5B	0.005700038	0.071345213	-0.11476	-0.06184	0.146678	-0.07453
KDM5C	0.00622896	-0.126692536	0.124148	0.009826	-0.05255	-0.05352
KDM5D	0.009478247	-0.024512272	0.11835	-0.0791	0.242656	-0.07686
KDM6A	-0.003417871	0.069410652	-0.16314	0.012916	-0.01085	0.001784
KDM6B	-0.017180791	-0.111862197	-0.06924	-0.0364	0.040228	0.07489
KDM7A	0.027696097	-0.029617463	-0.21916	-0.03562	0.103338	0.188672
KDM8	-0.032474707	-0.026877429	0.06216	0.002877	-0.07332	-0.14122
KDR	-0.05308164	-0.018855869	-0.13763	-0.01209	0.058892	0.005552
KDSR	-0.003831406	-0.045103505	0.146249	-0.01801	0.073537	0.065924
KEAP1	0.028068656	0.121470387	-0.02174	0.063929	-0.21019	0.191464
KEL	-0.028731599	-0.080595432	0.00169	0.03375	-0.13046	-0.02467
KERA	-0.063668262	-0.143589601	-0.06707	-0.00169	0.012503	0.037109
KGFLP2	-0.02099075	-0.012671395	0.060586	-0.03711	0.078506	-0.08374
KHDC1	-0.022477534	0.027765759	0.089761	0.05903	-0.20187	-0.16092
KHDC1L	-0.03646124	0.001592462	0.006218	0.052315	-0.1236	-0.092
KHDC3L	-0.030580934	-0.044804933	-0.05003	0.011239	-0.03172	-0.07019
KHDRBS1	0.142612511	0.147137096	0.100715	0.090337	-0.20824	-0.29307

KHDRBS2	-0.020116033	-0.05276685	-0.00719	0.004476	-0.03664	-0.1751
KHDRBS3	-0.043298426	-0.100934808	-0.11616	0.033183	-0.10978	-0.05507
KHK	-0.025670602	-0.06468414	0.087189	0.061575	-0.21617	0.100612
KHNYN	0.058022158	0.00287084	-0.1142	0.03782	-0.11895	-0.13793
KHSRP	0.021360406	-0.065622796	-0.01043	0.037632	-0.19641	0.01607
KIAA0020	0.008477961	0.059857747	0.094487	0.058038	-0.16844	-0.09058
KIAA0040	0.01657516	-0.045823389	-0.121	-0.02464	0.047167	-0.10234
KIAA0087	-0.04407485	-0.046107818	-0.04086	0.007089	-0.01327	-0.05906
KIAA0100	0.042833954	-0.036998392	-0.15404	0.012911	-0.05487	0.284116
KIAA0101	0.007506991	0.075556229	0.207613	0.120875	-0.35059	-0.24654
KIAA0125	-0.004628683	-0.10692107	-0.12343	-0.06342	0.165989	-0.22142
KIAA0141	0.007733171	-0.012059163	-0.21076	-0.01085	-0.01132	0.425539
KIAA0196	0.043775689	0.046036806	-0.05482	0.009818	-0.01624	0.244175
KIAA0226	-0.011548711	-0.016647346	-0.01552	0.0032	-0.07464	-0.07984
KIAA0226l	0.003848599	-0.144583717	-0.33166	-0.06772	0.180535	-0.14134
KIAA0232	0.022367713	0.111529048	-0.13887	-0.00317	0.029527	0.192915
KIAA0247	0.0489382	0.089351091	-0.30831	0.014915	-0.02361	-0.02419
KIAA0319	-0.027791572	-0.033875039	-0.11632	0.008416	-0.02013	-0.04917
KIAA0319l	0.03143018	-0.068243528	-0.16608	-0.0253	0.05287	0.366829
KIAA0355	0.019990125	-0.029255192	0.198533	-0.01296	0.044855	-0.29753
KIAA0368	0.02454679	0.074440205	-0.00465	0.038819	-0.13512	0.146497
KIAA0391	-0.017064989	-0.006730645	0.052655	########	0.020237	-0.05649
KIAA0408	-0.042224815	-0.071346455	0.209442	-0.02679	0.069452	-0.1169
KIAA0430	0.019965419	0.01609606	-0.06151	-0.05686	0.159721	-0.18803
KIAA0485	-0.029761105	-0.077610822	0.134666	-0.01028	0.024662	0.114767
KIAA0509	-0.033574403	-0.080670234	0.06009	0.015292	-0.0295	0.041022
KIAA0513	0.010110112	-0.079470121	-0.22722	-0.0825	0.242438	0.361117
KIAA0556	0.023385579	-0.084001648	-0.12335	-0.01866	0.059752	0.040426
KIAA0586	0.007712403	0.01043513	0.201584	0.059685	-0.14905	-0.16901
KIAA0753	0.02256261	-0.065689606	-0.0621	0.00633	-0.07062	-0.05753
KIAA0754	-0.017330643	0.076464214	0.055461	-0.01385	0.046299	-0.05839
KIAA0825	-0.1150752	-0.032920994	-0.01834	0.030672	0.038318	-0.02466
KIAA0895	-0.035590977	-0.077123596	0.071442	-0.03216	0.054786	-0.11196
KIAA0895l	-0.008409306	-0.059063297	-0.08677	0.056397	-0.15285	0.186767
KIAA0907	0.021053137	0.052020693	0.020196	0.037682	-0.08589	-0.19759
KIAA0922	0.026834607	0.038874775	-0.07652	-0.07966	0.190748	0.081849
KIAA0930	0.085316	-0.091871152	-0.26818	-0.08767	0.201302	0.387142
KIAA1024	-0.024115289	-0.033588603	-0.08975	0.000913	-0.00641	-0.12433
KIAA1033	0.063463752	-0.069855556	-0.43301	-0.01913	0.08203	0.251998
KIAA1045	-0.066064779	-0.114866516	-0.06196	-0.00369	0.01871	-0.12822
KIAA1107	-0.029410944	0.034470529	0.154111	-0.00612	0.03528	-0.10731
KIAA1109	-0.04783662	-0.023223709	0.127086	-0.04121	-0.05437	-0.13001
KIAA1143	0.071832221	0.090692389	0.057086	0.085229	-0.24502	-0.0081
KIAA1147	0.014864764	0.072200302	0.265524	0.005855	0.02337	-0.30614
KIAA1161	-0.034987245	-0.030846651	0.050409	0.041156	-0.1305	-0.0755
KIAA1191	0.04117426	0.057587691	0.181022	0.007724	-0.02505	-0.05443
KIAA1210	-0.030929565	-0.073128857	0.012216	0.033311	-0.08489	0.018298
KIAA1211	0.023699969	-0.004582605	-0.04028	-0.02755	0.078528	-0.0191
KIAA1211l	0.069394187	-0.080679688	-0.07227	0.032602	0.07154	0.008579
KIAA1217	-0.007587218	0.007520342	0.11319	0.163369	-0.2821	-0.05116
KIAA1244	-0.098774641	-0.091019143	-0.04878	-0.03327	0.047484	0.028407

KIAA1257	-0.032376256	-0.055303245	-0.11914	-0.01487	0.048186	-0.09944
KIAA1279	0.012399879	0.041264457	0.10249	0.020602	-0.04814	-0.0749
KIAA1324	0.004565932	-0.013788554	0.026609	0.014083	0.036718	-0.198
KIAA1324I	-0.009359827	0.059133525	0.125318	-0.03445	0.110241	-0.21559
KIAA1328	-0.033028237	-0.044035791	0.088702	0.02242	-0.01517	0.004844
KIAA1377	0.003450182	0.223777648	0.104239	-0.01578	0.119651	-0.32601
KIAA1407	-0.008138509	-0.065880097	-0.02547	-0.06288	0.174537	-0.22795
KIAA1429	-0.018277401	0.049937965	0.033228	0.054886	0.014855	-0.05469
KIAA1430	-0.034722158	0.027332629	0.12947	0.008441	-0.04846	-0.1993
KIAA1432	0.005012218	-0.095366119	-0.27302	-0.05201	0.212075	0.440581
KIAA1456	-0.035395565	-0.061234487	-0.01712	0.014344	-0.01175	0.198176
KIAA1462	-0.07137306	0.025033113	-0.07267	-0.06692	0.09271	0.009879
KIAA1467	-0.03593257	-0.115545973	0.026574	-0.06922	-0.00062	0.035944
KIAA1468	0.0503072	0.001321585	-0.14516	-0.06371	0.177824	-0.03599
KIAA1522	-0.016856264	-0.086416449	-0.07534	-0.02746	-0.02335	0.021367
KIAA1524	-0.019913099	-0.037819068	0.007628	0.119046	-0.23728	-0.07267
KIAA1549	-0.028288981	-0.079238999	0.056232	0.004982	-0.06769	0.015778
KIAA1549I	-0.06767285	-0.054720967	0.142141	0.07824	0.014821	-0.14323
KIAA1551	0.02841842	0.102630497	0.145779	-0.02382	0.077663	-0.22756
KIAA1586	-0.008364459	0.004353875	0.071131	-0.00544	0.046319	0.033309
KIAA1598	0.007379417	-0.113917188	-0.30205	-0.0659	0.209799	0.517347
KIAA1614	-0.026531882	-0.060276624	0.051669	0.012431	-0.14415	-0.18762
KIAA1614-	-0.027387466	-0.034848554	-0.08642	0.02842	-0.10285	-0.01456
KIAA1644	-0.052209394	-0.072197099	-0.12645	-0.00112	-0.00966	0.021913
KIAA1652	-0.010596327	-0.197890262	0.127369	-0.02035	0.021131	0.247186
KIAA1654	-0.034722914	-0.048980257	0.028156	0.01467	-0.04621	-0.02371
KIAA1656	-0.036074085	-0.129891797	-0.04583	0.032084	-0.10493	-0.0164
KIAA1661	-0.039129787	-0.004472599	-0.11074	0.014442	-0.0177	0.066725
KIAA1671	-0.002208243	-0.005327205	-0.0555	0.023149	-0.02641	-0.01539
KIAA1683	-0.025546411	-0.136322371	-0.17813	-0.01039	-0.01154	-0.08957
KIAA1715	0.003803183	0.07148486	0.010947	0.016858	0.021045	0.025486
KIAA1731	0.028314479	-0.010753581	-0.03581	0.0421	-0.10047	-0.00721
KIAA1751	-0.033750799	-0.023201053	0.10196	-0.01117	-0.02389	-0.10071
KIAA1755	-0.041926409	-0.037976584	-0.1227	-0.0023	0.011424	0.085766
KIAA1804	-0.01473611	-0.094687982	-0.07005	0.043807	-0.13629	0.075634
KIAA1841	-0.010402521	0.010171592	-0.05708	-0.0102	0.027063	0.075285
KIAA1875	-0.028924497	-0.053692132	-0.02316	-0.01814	0.083667	0.089868
KIAA1919	0.032472033	0.033870252	-0.03358	0.013683	-0.02943	0.084923
KIAA1958	-0.005637754	-0.069377449	-0.10397	0.026979	-0.04914	0.082738
KIAA2013	0.036335725	0.065614412	-0.19525	0.007167	-0.04002	-0.02596
KIAA2018	-0.020798208	0.032806688	-0.05254	-0.02311	0.013613	-0.10453
KIAA2022	0.00442788	-0.036900675	0.115283	0.081616	0.025751	-0.10918
KIAA2026	-0.015187249	0.183192248	0.12762	-0.03429	0.048385	-0.18544
KIDINS22(	-0.011121105	0.028921896	-0.09979	0.000588	-0.00158	0.042304
KIF11	-0.00703537	0.035658708	0.0365	0.107539	-0.19511	-0.04535
KIF12	-0.027838487	-0.10534831	0.004682	0.007577	-0.06577	-0.02706
KIF13A	-0.022920159	-0.025025467	-0.34547	-0.06213	0.165558	0.48961
KIF13B	0.035727779	0.065195339	-0.10637	0.004008	-0.00566	0.253067
KIF14	-0.01481306	0.009708543	0.019902	0.171652	-0.32481	-0.03645
KIF15	-0.009927178	0.023434904	0.045664	0.104637	-0.21816	-0.03716
KIF16B	-0.006972813	-0.044486554	-0.01406	0.025535	-0.04552	0.044271

KIF17	-0.026370959	-0.025548484	0.053232	0.028785	-0.08167	-0.23779
KIF18A	-0.023740824	0.028188426	-0.03325	0.199434	-0.32656	-0.14188
KIF18B	-0.01435659	0.007523475	0.036133	0.192215	-0.41491	-0.05187
KIF19	-0.047900816	0.030948596	0.09456	-0.00465	-0.03035	-0.05449
KIF1A	-0.043651411	-0.043267801	-0.06761	-0.01209	0.021919	-0.08528
KIF1B	0.018578059	-0.00452186	-0.21214	0.043959	-0.07813	0.22669
KIF1C	-0.006167266	-0.082305912	-0.08563	0.084833	-0.27872	0.194005
KIF20A	-0.015139534	-0.022403595	0.055352	0.149633	-0.29355	-0.03322
KIF20B	-0.001075371	-0.038056744	-0.00922	0.088119	-0.31246	-0.29237
KIF21A	-0.006502395	0.192492506	0.334257	0.039963	-0.11887	-0.24511
KIF21B	0.018188004	0.127479838	0.195438	0.025087	-0.09154	-0.34713
KIF22	0.004069281	-0.014576428	0.044134	0.109568	-0.2955	0.161919
KIF23	-0.018519817	-0.019434139	-0.04203	0.124068	-0.2307	0.148415
KIF24	-0.056193075	-0.061934328	0.161323	0.020841	-0.06347	0.100199
KIF25	-0.034224097	-0.094384152	0.023547	-0.00783	-0.05021	0.025086
KIF25-AS1	-0.01338061	0.022821673	0.03403	0.006854	0.028802	-0.00493
KIF26A	-0.044276023	-0.162080595	0.106595	-0.00686	0.057739	-0.0175
KIF26B	-0.016567188	-0.144787294	-0.0905	-0.04438	-0.03451	0.018921
KIF27	-0.029777037	-0.023598282	-0.08372	-0.02224	0.062813	-0.03699
KIF2A	0.02366934	0.104554992	0.390188	0.015357	-0.04231	-0.31256
KIF2B	-0.047379719	-0.034796895	-0.10581	0.035288	-0.07361	0.119809
KIF2C	-0.011693379	-0.011988071	-0.01752	0.18054	-0.4236	-0.03525
KIF3A	0.000156042	0.115910475	0.307507	0.128364	-0.34269	-0.24675
KIF3B	0.022292589	-0.016697222	-0.02585	0.009526	-0.05107	0.013762
KIF3C	-0.013680432	0.012675749	0.032472	-0.06196	0.065337	-0.0635
KIF4A	-0.014091325	-0.005638451	0.006528	0.161505	-0.35626	-0.04374
KIF5A	-0.008501115	-0.044017391	0.245701	-0.06164	-0.1281	-0.03807
KIF5B	0.090661179	0.064757603	-0.25917	0.077819	-0.18256	-0.02527
KIF5C	-0.007570698	-0.093216429	0.250536	-0.01789	0.051213	-0.16024
KIF6	-0.066111814	-0.023970284	0.070652	-0.0869	0.019123	-0.03861
KIF7	-0.024028172	0.011777308	0.039572	-0.00619	0.003088	0.036059
KIF9	-0.025932578	-0.019257815	-0.08608	0.06894	-0.21424	0.129477
KIF9-AS1	-0.033063373	-0.089689171	-0.18181	5.52E-05	-0.02508	0.012462
KIFAP3	0.012870468	0.153190893	0.125108	0.046024	-0.14277	-0.26162
KIFC1	-0.001602666	-0.022929803	0.031398	0.112402	-0.40125	-0.10366
KIFC2	0.013689796	-0.114213947	0.110297	-0.05411	0.153163	-0.07043
KIFC3	-0.021954444	0.099926573	-0.10278	-0.03257	0.076023	0.009328
KIN	-0.013196621	0.049301758	-0.02084	-0.04183	0.133701	0.037571
KIR2DL1	0.001794176	0.304574078	-0.03651	-0.01764	0.01451	-0.14327
KIR2DL2	-0.000139934	0.3030488	-0.0408	-0.01474	0.005131	-0.13851
KIR2DL3	-0.001862406	0.300449038	-0.04513	-0.01179	-0.00529	-0.13667
KIR2DL4	-0.013214078	0.290552331	-0.07924	0.038008	-0.1135	-0.13254
KIR2DL5A	-0.000111873	0.344865096	-0.04805	-0.02078	0.006801	-0.15364
KIR2DS1	-0.00820369	0.298017415	-0.05773	-0.02008	0.022177	-0.11286
KIR2DS2	-0.001685776	0.350391271	0.019173	-0.01488	0.023772	-0.13714
KIR2DS3	-0.003942621	0.356070908	-0.07141	-0.01776	0.036105	-0.10836
KIR2DS4	-0.003803088	0.321103379	-0.02283	-0.00448	-0.00701	-0.12853
KIR2DS5	0.004210112	0.35045644	-0.02774	-0.0196	-0.00531	-0.19264
KIR3DL1	0.000431334	0.325281737	-0.00093	-0.00614	-0.00505	-0.1323
KIR3DL3	-0.001494	0.336562673	0.025843	-0.02226	0.018123	-0.16317
KIR3DS1	-0.003102779	0.332922008	0.017522	0.003022	-0.00515	-0.1189

KIR3DX1	-0.032202392	0.108281392	0.051859	0.073753	-0.20481	-0.01415
KIRREL	-0.044769195	0.003877577	-0.09771	0.059053	-0.08083	-0.06973
KIRREL2	-0.02847387	-0.024725272	-0.01976	0.069401	-0.16013	0.092137
KIRREL3	-0.005251399	-0.069538178	-0.04882	-0.00109	0.018557	-0.14095
KIRREL3-/	-0.042975767	-0.047792538	-0.01897	0.005971	0.023228	0.060072
KIRREL3-/	-0.036163882	-0.107079986	0.052421	0.022403	-0.04679	-0.15279
KISS1	-0.041381967	-0.033716536	-0.04798	0.002327	0.024578	0.04185
KISS1R	-0.014705097	-0.072188476	0.01859	-0.00625	0.006758	-0.17864
KIT	-0.005919166	0.057749689	-0.07871	0.003662	-0.02321	-0.22293
KITLG	-0.06196471	-0.114217344	-0.01333	-0.00511	0.089685	-0.01392
KIZ	0.016437752	0.006221678	0.095884	-0.00068	0.001374	-0.14431
KL	-0.129672213	-0.03370661	-0.01826	-0.03294	0.105846	0.351005
KLB	-0.045209016	-0.017764981	0.13568	0.001502	0.016182	-0.02728
KLC1	0.019665963	-0.047107338	-0.10291	0.075083	-0.21951	0.012707
KLC2	-0.012089163	-0.021297984	0.153359	0.165625	-0.49872	-0.28123
KLC3	-0.031096653	-0.04528639	0.042231	0.003451	-0.04943	-0.06172
KLC4	-0.003660438	-0.05633376	0.16428	-0.00503	-0.03551	-0.19188
KLF1	-0.021601346	-0.057639968	-0.03896	-0.03437	0.12201	0.148974
KLF10	0.022418142	0.005248681	-0.16364	-0.02257	0.073884	0.440903
KLF11	0.008328193	0.011229546	-0.21426	-0.11406	0.319194	0.454406
KLF12	0.018739812	0.197911703	0.290528	-0.02885	0.120364	-0.35284
KLF13	0.010070203	0.050532461	0.152558	-0.03312	0.093545	0.085133
KLF14	-0.044476689	-0.030120095	-0.05331	0.001946	0.003933	-0.01509
KLF15	-0.037283242	-0.046846766	-0.03326	-0.01102	-0.03528	-0.0992
KLF16	0.003065781	-0.145373564	-0.02669	-0.00501	-0.06599	-0.02221
KLF17	-0.037708226	-0.004521716	0.014692	0.00544	-0.00144	-0.01002
KLF2	0.014138089	0.038571066	0.130148	-0.07396	0.188248	-0.08809
KLF3	-0.003817831	-0.019567052	-0.02916	-0.07503	0.336342	0.047622
KLF3-AS1	-0.017596536	-0.001713408	0.28316	-0.06213	0.19078	-0.23455
KLF4	0.00441297	-0.094488256	-0.28173	-0.11099	0.329085	0.520915
KLF5	-0.011482267	-0.120191608	0.051916	-0.01788	0.052568	0.020223
KLF6	0.060013876	-0.037449728	-0.00737	-0.03782	0.129472	-0.0513
KLF7	-0.039595409	-0.105320268	-0.03764	-0.13325	0.335857	0.013018
KLF8	-0.012365611	0.021830193	-0.13974	-0.03202	0.103848	-0.2199
KLF9	0.006394028	0.028632	0.045711	-0.02605	-0.00865	0.095331
KLHDC1	-0.036573415	-0.030465612	0.151505	-0.03387	0.161576	-0.17878
KLHDC10	0.01777398	0.144904704	-0.07851	0.021725	-0.09577	0.092187
KLHDC2	0.035794455	0.032960108	0.184777	0.00069	-0.00472	-0.25048
KLHDC3	0.03591171	-0.024492553	0.001743	0.009316	-0.10917	0.150948
KLHDC4	-0.03156037	0.216022276	0.185196	0.024003	-0.02901	-0.28677
KLHDC7A	-0.041111996	-0.063494074	0.167626	0.001423	0.005371	-0.16673
KLHDC7B	-0.003068216	-0.008903987	0.02458	-0.07537	0.1954	0.143412
KLHDC8A	-0.036477392	-0.042797685	0.015297	-0.01468	0.000149	-0.05759
KLHDC8B	-0.006906655	-0.120543717	-0.11969	-0.07157	0.136723	0.250856
KLHDC9	-0.024384052	-0.090915962	0.092661	-0.02812	0.079105	-0.16061
KLHL1	-0.037263975	-0.055056718	-0.01588	0.121635	-0.10309	0.031813
KLHL10	-0.050091495	-0.047072535	-0.10881	0.003951	0.019539	0.079561
KLHL11	-0.020362827	-0.022088268	0.014825	0.013978	-0.04152	-0.0257
KLHL12	0.026479414	0.015975547	-0.17512	0.097764	-0.26664	0.085271
KLHL13	-0.010455208	-0.064788596	-0.04697	0.039533	-0.07962	-0.16318
KLHL14	-0.032641529	-0.090124663	-0.16266	-0.00724	0.228815	-0.12544

KLHL15	0.01735806	0.033511985	0.016336	0.05221	-0.13691	-0.03982
KLHL17	-0.02632626	-0.041219357	0.104354	0.020526	-0.04667	0.116057
KLHL18	0.01586652	-0.076282325	-0.22455	0.012193	-0.0372	0.411394
KLHL2	0.038253676	-0.019973739	-0.35285	0.048895	-0.11109	0.21804
KLHL20	-0.006996559	0.046971375	0.17416	0.032802	-0.0384	-0.08362
KLHL21	0.027847058	0.001889279	0.004422	-0.02632	0.051023	-0.07673
KLHL22	-0.010839042	-0.058605356	0.119822	-0.06417	0.175692	-0.18931
KLHL23	-0.016595963	0.055318814	-0.0268	0.154345	-0.43824	-0.07443
KLHL24	0.0536506	0.066326816	-0.00154	-0.03336	0.109554	0.024615
KLHL25	-0.021492764	-0.021004969	-0.04445	0.011456	-0.1242	-0.13271
KLHL26	-0.006333639	0.029261395	0.065834	0.052002	-0.08152	0.063467
KLHL28	0.047077543	0.143587886	0.072805	-0.00481	0.027059	-0.16572
KLHL29	-0.029286833	-0.091836662	0.035633	-0.00936	-0.02582	-0.09635
KLHL3	-0.006755441	-0.001522817	0.306193	-0.02915	0.103731	-0.46372
KLHL30-AS	-0.028393041	-0.02125139	0.056782	0.003894	-0.04646	-0.09313
KLHL31	-0.05609009	-0.064902917	-0.03776	0.015261	0.001118	-0.00191
KLHL32	-0.092198629	-0.081542554	0.090318	0.006124	0.034363	-0.17695
KLHL34	-0.045992286	-0.060452437	0.269717	-0.04129	0.131193	-0.25425
KLHL35	-0.018637941	-0.054427659	-0.01387	-0.05849	0.065727	0.027473
KLHL36	4.69E-05	-0.04036373	0.339349	-0.07886	0.172533	-0.21112
KLHL4	-0.023883738	-0.025802706	0.005444	0.07622	-0.15997	0.04567
KLHL40	-0.030916034	0.012490168	0.000266	-0.00076	0.020292	-0.03707
KLHL41	-0.092276083	-0.074525235	0.094449	-0.03103	0.106148	-0.0817
KLHL42	-0.020554709	0.025745453	0.098636	0.040105	-0.01658	0.001532
KLHL5	0.002160376	-0.087832655	-0.31334	-0.03115	0.122839	0.291057
KLHL6	0.03204355	-0.120838049	-0.08055	-0.0232	0.060727	-0.00205
KLHL7	0.085348158	0.070863732	-0.05893	0.019876	-0.28408	0.297116
KLHL8	0.028130651	0.019404439	-0.29953	0.0073	0.011557	0.470355
KLHL9	0.008922977	0.098843662	0.084965	-0.08911	0.211687	0.085893
KLK1	-0.010188512	-0.110284022	-0.04537	-0.01343	-0.05903	0.003804
KLK10	-0.040885184	-0.050066755	-0.00683	-0.00037	0.007508	0.080475
KLK11	-0.031103583	-0.085143769	-0.00529	-0.00644	0.021172	0.024401
KLK12	-0.036823605	-0.061736959	0.010401	-0.00785	-0.08107	-0.04961
KLK13	-0.062486438	-0.051678879	-0.00249	0.008896	-0.04534	0.011367
KLK14	-0.028050579	-0.052492848	-0.01585	-0.01192	-0.0206	-0.0353
KLK15	-0.052755816	-0.045862781	-0.12595	-0.02345	0.034797	0.066349
KLK2	-0.072161342	-0.096852014	-0.14917	0.020686	-0.05085	0.062177
KLK3	-0.034220042	-0.102000075	-0.13078	0.047437	-0.17008	-0.05793
KLK4	-0.048944173	-0.101409709	-0.10543	0.015742	0.008597	-0.04651
KLK5	-0.044922915	-0.045060097	0.100894	0.014894	-0.00546	0.110005
KLK6	-0.032509439	-0.107663923	-0.05778	0.012074	-0.05182	-0.05198
KLK7	-0.041342399	-0.040641513	-0.09481	0.038767	-0.11192	-0.11296
KLK8	-0.047953524	-0.049681625	0.018799	0.015852	-0.02558	-0.0229
KLKB1	-0.019022069	-0.022426209	0.123466	0.007002	-0.02363	-0.14003
KLLN	-0.026591806	-0.02977475	0.014773	0.020311	-0.07056	0.023148
KLRAP1	-0.012817029	-0.034809353	0.375544	-0.03169	0.110611	-0.17518
KLRB1	0.011236823	0.217434955	0.358965	-0.05498	0.142101	-0.23591
KLRC3	-0.002400599	0.29720224	0.39652	-0.09351	0.258466	-0.2192
KLRC4	-0.003388719	0.197006272	0.640522	-0.05304	0.153989	-0.24718
KLRD1	0.008881284	0.288903306	0.437796	-0.02256	0.075083	-0.24123
KLRF1	0.003595728	0.324536317	0.301091	-0.07827	0.226235	-0.21382

KLRG1	0.003019373	0.13684212	0.523324	-0.0456	0.101204	-0.3061
KLRG2	-0.038024656	-0.060024852	0.007715	0.008453	-0.05314	-0.08175
KMO	0.002580994	-0.114638865	-0.28104	-0.09112	0.234192	0.159437
KMT2A	-0.039866682	0.053726131	0.303956	-0.03672	-0.02827	-0.2049
KMT2B	0.00340878	-0.051797903	-0.0577	0.026991	-0.07104	-0.15767
KMT2C	0.043453902	0.073781045	-0.10534	-0.03279	0.113975	-0.01641
KMT2D	-0.007744655	-0.061071426	-0.03895	0.095404	-0.16357	-0.03305
KMT2E	0.052221032	0.13127799	0.116273	-0.03238	0.12253	-0.23627
KMT2E-AS	-0.010194481	-0.070035152	0.001562	0.003847	0.008068	0.024497
KNCN	-0.031982396	0.029648196	-0.04454	-0.00775	0.016631	-0.09136
KNDC1	-0.026246863	-0.083444951	-0.17727	0.020564	0.008549	0.128637
KNG1	-0.036561277	-0.101221338	0.084365	0.031267	-0.05991	-0.0516
KNOP1	0.013616994	-0.007232709	0.233941	0.034733	-0.13617	-0.05662
KNSTRN	0.008482058	0.005744531	-0.21571	0.122955	-0.34213	0.386966
KNTC1	-0.000839407	0.031596452	0.050217	0.091516	-0.25078	-0.23499
KPNA1	0.007638754	0.076636735	-0.155	-0.01663	-0.04214	0.030221
KPNA2	0.054525997	0.035284666	-0.16296	0.138072	-0.38653	0.111062
KPNA3	0.052641737	0.191737669	0.051779	0.072451	-0.19276	0.017637
KPNA4	0.005057163	0.077650349	-0.08637	0.002245	0.020449	-0.20261
KPNA5	-0.011273471	0.035394727	0.303688	-0.04292	0.113453	-0.2834
KPNA6	0.039034847	0.09800072	0.067614	0.0408	-0.10592	0.024013
KPNB1	0.076059953	0.113056607	0.053417	0.073463	-0.17424	-0.25748
KPTN	0.009147518	-0.133995123	-0.08209	0.016724	-0.05595	0.080582
KRAS	0.004630089	-0.074800847	-0.01851	-0.00461	-0.04333	0.010415
KRBA1	-0.007141988	-0.079938893	0.051783	-0.0293	-0.02643	-0.09325
KRBA2	-0.022455599	0.016720231	0.04665	-0.03051	0.026396	-0.06056
KRBOX1	-0.027787556	-0.078785492	-0.08132	-0.00659	-0.02951	-0.11611
KRBOX1-AS	-0.036269382	-0.012799366	0.086373	0.000596	0.023721	-0.06054
KRBOX4	0.02007285	0.053447431	0.094087	-0.01838	0.075209	-0.09086
KRCC1	0.00713763	0.039672063	-0.08631	0.01396	0.010541	-0.08126
KREMEN1	0.028936595	0.016352142	-0.32213	0.080727	-0.11552	-0.00529
KREMEN2	-0.014593316	-0.083664269	-0.00825	-0.0129	-0.05033	0.020658
KRI1	0.029307649	0.0326235	0.242213	0.007717	-0.03937	-0.22296
KRIT1	-0.0157592	0.097289586	0.096804	0.018477	-0.02062	0.041058
KRMP1	-0.04202666	-0.095436971	0.054831	0.000354	0.02447	0.122622
KRR1	0.018935595	0.077138057	0.243002	-0.01799	0.014542	-0.31026
KRT1	-0.014759053	-0.034280592	0.220748	0.005069	-0.0192	-0.1305
KRT10	0.023587465	0.066360484	0.255705	0.019227	-0.08277	-0.07205
KRT12	-0.048066738	-0.044024626	0.056916	-0.00266	0.024775	0.000137
KRT13	-0.040353516	-0.073887256	-0.13368	0.019744	-0.05451	0.039391
KRT14	-0.030622416	-0.068976972	-0.07504	0.000243	0.023091	0.105972
KRT15	-0.033157981	-0.029680508	-0.08423	0.038296	-0.08363	0.023253
KRT16	-0.025314253	-0.044706781	-0.08561	0.045783	-0.1065	-0.00926
KRT17P5	-0.031769188	-0.061227872	-0.01448	0.016418	-0.08287	-0.06062
KRT18	-0.013537466	-0.100415451	0.259578	-0.03222	0.070766	-0.23973
KRT19	-0.045411406	0.013550319	-0.07565	0.023282	-0.04558	0.009189
KRT19P2	-0.031513768	-0.005406569	0.035995	0.029079	-0.0583	0.007787
KRT2	-0.025356635	0.056451427	0.250724	-0.0187	0.054881	-0.20727
KRT20	-0.054290883	-0.017444538	0.002497	-0.03939	0.124306	-0.05367
KRT222	-0.09457704	-0.078437608	0.123007	-0.02322	0.050558	0.024342
KRT23	-0.00493644	-0.06425203	-0.19221	-0.01291	0.040784	0.004081

KRT24	-0.032120697	-0.006136557	-0.01932	0.00962	-0.01366	-0.03441
KRT25	-0.042489288	-0.056617812	-0.07814	-0.02756	0.102617	-0.04021
KRT27	-0.044818425	-0.047651978	-0.06812	0.013889	-0.01114	0.025176
KRT3	-0.02571156	-0.045175907	-0.08055	0.048603	-0.10947	0.106364
KRT31	-0.040303129	-0.041751824	-0.177	-0.02482	0.108562	0.055372
KRT32	-0.036270153	-0.096730462	0.052843	-0.02813	0.015365	0.052912
KRT33A	-0.034425649	-0.071218673	-0.09257	0.012931	-0.01685	-0.00676
KRT33B	-0.031611526	-0.061930874	0.0252	0.028202	-0.09608	0.028978
KRT35	-0.039413574	0.015206288	0.027583	0.011167	-0.00996	-0.06433
KRT36	-0.03930358	-0.007253698	-0.10052	-0.00289	0.045396	0.236894
KRT37	-0.039481342	-0.058052632	-0.00871	0.043632	-0.10296	0.154338
KRT38	-0.03062855	-0.021980389	0.00124	-0.00081	0.025477	-0.09645
KRT4	-0.047518253	0.0269207	0.039333	0.033146	-0.07931	0.083402
KRT40	-0.171744516	-0.115196028	0.00089	0.012239	0.050604	0.012904
KRT5	-0.006635836	-0.089606647	-0.08436	0.005505	-0.01065	-0.1257
KRT6A	-0.050510561	-0.080623906	-0.01047	-0.0225	0.044786	-0.0555
KRT6B	-0.029063594	-0.039386324	0.165429	0.012334	-0.09228	-0.0971
KRT7	-0.03620925	-0.065798679	-0.08562	0.109894	-0.20295	0.136644
KRT71	-0.031143957	-0.013907346	-0.01547	-0.00074	0.02105	-0.00512
KRT72	-0.002968402	0.132823592	0.324354	-0.03378	0.102501	-0.25819
KRT73	-0.01816549	0.084794571	0.314537	-0.04466	0.118017	-0.23828
KRT74	-0.040530702	-0.003946212	0.086087	0.010814	-0.05697	-0.14571
KRT75	-0.030960004	-0.049352969	0.036094	0.030386	-0.09405	-0.02654
KRT76	-0.030221166	-0.031356068	0.092584	-0.00841	-0.00246	-0.17267
KRT77	-0.03155543	-0.022861475	-0.07523	-0.00148	0.017027	-0.09248
KRT78	-0.054332237	-0.008878025	-0.14713	0.009586	-0.0295	0.088871
KRT79	-0.036699402	-0.052764506	-0.02529	0.005763	0.012113	-0.11817
KRT8	-0.032646817	-0.11101336	0.131187	0.029753	-0.10731	-0.11917
KRT80	-0.027036051	-0.03729475	0.102178	0.002307	-0.01555	-0.03228
KRT81	-0.011671848	-0.00164218	-0.18563	0.077742	-0.20725	0.024296
KRT82	-0.03020901	-0.062093453	-0.02613	0.002313	-0.028	0.039937
KRT83	-0.038368953	-0.050643269	0.020934	0.000656	-0.01704	-0.06673
KRT84	-0.037033488	-0.043518341	-0.00871	-0.01826	0.077706	-0.15294
KRT85	-0.032515567	-0.020529003	0.069339	0.002405	-0.05828	-0.00326
KRT8P12	-0.03289614	-0.073979805	0.052667	0.012714	-0.10812	-0.02452
KRT9	-0.03210875	-0.037131207	-0.05516	-0.00117	-0.07782	-0.03285
KRTAP1-1	-0.039907933	-0.042216071	0.075385	0.005743	0.014358	-0.05883
KRTAP1-3	-0.038154299	-0.067725768	-0.06213	0.024253	-0.10382	-0.02188
KRTAP1-5	-0.027356344	-0.069422804	0.083663	-0.0152	0.043547	-0.11423
KRTAP10-[illegible]	-0.016450474	-0.023117491	0.022218	-0.01214	-0.05202	-0.06928
KRTAP11-[illegible]	-0.030380771	-0.019895562	-0.027	0.001457	-0.06591	-0.13202
KRTAP13-[illegible]	-0.028027233	-0.034855014	-0.02765	-0.01622	-0.0321	-0.09532
KRTAP17-[illegible]	-0.030735488	-0.021202547	-0.02028	-0.01661	0.029068	0.021253
KRTAP19-[illegible]	-0.03649372	-0.080172647	0.099057	0.009757	-0.02023	-0.10058
KRTAP19-[illegible]	-0.048066689	-0.00753287	-0.16072	0.038894	-0.08081	0.089008
KRTAP2-1	-0.053302993	-0.017808282	-0.00644	0.026088	-0.06399	0.172852
KRTAP3-1	-0.023555107	-0.043833719	-0.06413	-0.00601	-0.04581	-0.07282
KRTAP3-2	-0.0523492	-0.096099532	0.011941	0.029469	-0.07124	0.031522
KRTAP3-3	-0.089719393	-0.077795265	-0.04287	-0.03269	0.070214	0.169338
KRTAP4-1	-0.031524327	-0.023645961	-0.00136	0.004728	-0.00491	-0.071
KRTAP4-1[illegible]	-0.044843432	-0.046014545	-0.07315	-0.00605	0.049899	-0.11503

KRTAP4-1:	-0.030027636	-0.068847698	0.043882	0.022922	-0.05546	0.059034
KRTAP4-2	-0.029421654	-0.030077992	0.08305	0.00826	-0.02556	-0.09055
KRTAP4-3	-0.043739769	-0.092496091	-0.03071	-0.00705	0.034232	-0.0435
KRTAP4-4	-0.050243525	0.045772921	0.006239	-0.00656	0.030657	0.044303
KRTAP4-5	-0.027755111	-0.081988681	0.009882	-0.0067	-0.02299	-0.08092
KRTAP4-7	-0.063951129	-0.024897064	0.086939	0.004031	-0.02187	-0.13517
KRTAP4-8	-0.029167345	-0.057585294	0.031433	0.028448	-0.06578	0.093167
KRTAP4-9	-0.031405642	0.005158111	0.01514	0.005949	-0.00258	0.06801
KRTAP5-2	-0.035141623	-0.055087066	-0.0155	0.014208	-0.0532	-0.0148
KRTAP5-8	0.001902327	-0.057598781	0.05059	-0.00378	-0.09827	-0.17819
KRTAP5-9	-0.030630644	-0.047846565	-0.02003	-0.01583	0.036755	0.018653
KRTAP5-AS	-0.043233435	-0.048154502	-0.03165	-0.01775	0.035204	-0.10147
KRTAP7-1	-0.032835947	-0.043722614	-0.01069	0.029035	-0.07529	0.042309
KRTAP8-1	-0.030674216	-0.074700041	-0.14239	-0.01265	0.029265	0.008323
KRTAP9-3	-0.060507556	-0.074062002	0.020175	-0.0251	0.086367	-0.01273
KRTAP9-4	-0.026992577	-0.024066303	0.014513	0.013607	-0.03723	-0.03886
KRTAP9-8	-0.032365125	-0.069755509	0.196152	0.024583	-0.04042	-0.03653
KRTAP9-9	-0.092014761	-0.038706948	-0.06146	-0.00906	0.03432	0.039693
KRTCAP2	0.0386706	0.055920322	0.192425	0.084398	-0.23727	-0.04501
KRTCAP3	-0.021492502	-0.062715417	0.2842	-0.00519	0.038842	-0.10798
KRTDAP	-0.045956907	-0.000277206	-0.04912	0.010853	-0.01354	0.065415
KSR1	-0.042977892	-0.084354357	-0.0954	-0.0135	-0.07266	-0.01634
KSR2	-0.042702826	-0.083837841	-0.10428	0.014261	-0.01582	-0.12266
KTN1	0.053870804	0.086848473	0.124516	0.062245	-0.12455	-0.05994
KXD1	-0.023350814	-0.081429418	0.033291	-0.05518	0.034621	-0.09258
KY	-0.056453931	-0.0725784	-0.03805	-0.02622	-0.02691	-0.05615
KYNU	0.050929035	-0.116353768	-0.3974	-0.05343	0.083656	0.377696
L1CAM	-0.032397431	-0.06989164	-0.0694	-0.00143	-0.02084	-0.06064
L1TD1	-0.032461712	-0.055104831	-0.029	-0.06906	0.217482	0.192654
L2HGDH	-0.019205264	0.124148644	0.018058	0.143819	-0.30714	-0.08417
L3HYPDH	0.003352958	0.008596509	-0.0172	-0.10135	0.283589	-0.03513
L3MBTL1	-0.087054044	-0.118777194	0.100817	-0.00639	0.084315	-0.12081
L3MBTL2	0.034936355	0.051397823	0.218847	0.02485	-0.11342	-0.30309
L3MBTL3	0.010600811	-0.082673609	0.082115	-0.07865	0.23112	-0.09868
L3MBTL4	-0.019818083	0.115486	-0.08672	-0.0117	0.035753	0.013116
LA16c-358	-0.006346452	0.001326437	-0.04509	-0.02582	0.085436	0.141236
LA16c-38(	-0.020849942	-0.11020713	0.028982	-0.01093	-0.05621	-0.07027
LA16c-38(	-0.018359804	-0.107361237	-0.01137	0.01031	-0.09084	0.044499
LA16c-38:	-0.022061262	-0.085656443	-0.0328	0.000975	-0.05799	-0.00052
LA16c-39:	-0.04014109	-0.044038561	-0.03487	0.019422	-0.03292	0.089166
LA16c-39:	-0.031531914	-0.10258607	0.02983	0.031902	-0.06099	0.057063
LA16c-83I	-0.02168175	-0.022160698	0.055592	0.005389	-0.05289	-0.07821
LACC1	-0.026998107	0.004519805	-0.21392	-0.09168	0.212616	0.287307
LACE1	-0.029226744	-0.0237604	0.011006	-0.00283	-0.00521	-0.05265
LACRT	-0.035783817	-0.089877475	-0.10176	0.029961	-0.05641	0.032874
LACTB	-0.0251367	-0.015465515	-0.23575	-0.07467	0.115924	0.458935
LACTB2	0.013927284	0.017147627	-0.02917	-0.11052	0.309528	0.303587
LAD1	-0.01303358	-0.032399844	-0.04258	-0.00642	-0.01962	-0.00121
LAG3	-0.004297273	-0.040257102	0.314873	0.086543	-0.28112	-0.08928
LAGE3	0.028408769	0.033897133	0.143009	0.07134	-0.19436	0.047638
LAIR1	0.021806723	0.09195562	-0.09249	-0.07442	0.187405	0.058547

LAIR2	0.009646806	0.219225651	0.228599	-0.01497	0.02503	-0.16142
LALBA	-0.036302807	-0.04106209	-0.04514	0.022866	-0.06773	-0.08596
LAMA1	-0.05451543	-0.02589971	-0.02563	0.017222	-0.03046	0.052556
LAMA2	-0.003751007	0.099930916	-0.05142	0.032936	-0.02431	-0.07163
LAMA3	-0.081480864	-0.053637696	-0.016	0.007684	-0.09783	-0.01244
LAMA4	0.011970664	-0.096175286	-0.00973	0.056445	0.027754	0.034259
LAMA5	-0.014928755	-0.135792707	-0.16181	-0.0074	0.019909	-0.1841
LAMA5-AS:	-0.020134715	-0.078801473	-0.13723	0.000218	-0.06203	-0.05205
LAMB1	-0.083690973	-0.004095611	-0.07539	-0.01658	0.058904	0.035097
LAMB2	-0.025256539	-0.078743653	-0.19709	-0.02719	0.094394	0.433587
LAMB2P1	-0.010512821	-0.076543725	0.256649	0.001913	-0.04289	-0.12617
LAMB3	-0.016166522	-0.024599451	-0.0403	0.044985	-0.14504	0.006399
LAMB4	-0.039664159	-0.067998369	-0.1202	0.02577	0.044445	0.043975
LAMC1	-0.007612436	-0.102653634	-0.12837	-0.02691	0.069637	-0.06289
LAMC2	-0.028215088	-0.030595171	-0.04404	-0.00331	0.023793	-0.02507
LAMC3	-0.040320153	-0.116446598	0.085638	0.016211	-0.05617	-0.06929
LAMP1	0.096230546	0.114194377	-0.07158	-0.00935	-0.00359	0.192337
LAMP2	0.034556869	-0.050694089	-0.40176	-0.02621	0.064527	0.375511
LAMP3	-0.005073568	-0.051493694	0.032602	-0.02516	0.032248	-0.13509
LAMP5	-0.002345713	-0.068233718	-0.12982	-0.05593	0.149023	-0.12424
LAMTOR1	0.070774352	-0.052409114	-0.29937	-0.03003	0.112475	0.458922
LAMTOR2	0.032632821	-0.023984042	-0.18783	0.044461	-0.12137	0.422072
LAMTOR3	0.044972506	0.047470666	-0.24117	-0.00291	0.05591	0.316257
LAMTOR4	0.04979213	-0.062267622	-0.15403	0.048182	-0.09634	0.080711
LAMTOR5	0.102430945	0.005113123	-0.30417	0.086934	-0.19626	0.044334
LAMTOR5-/	-0.023634015	-0.014702946	-0.00895	0.069651	-0.17648	0.065991
LANCL1	0.016832022	0.052819565	0.210014	0.006148	-0.03237	-0.30643
LANCL2	0.080405997	0.050295752	0.076841	0.02182	-0.05702	0.086972
LANCL3	-0.033192836	0.215510958	-0.04057	-0.04378	0.131977	0.003186
LAP3	0.051829248	0.016535896	-0.35466	0.031246	-0.07374	0.452839
LAPTM4A	0.088779246	-0.023206487	-0.46248	-0.01523	0.086873	0.404969
LAPTM4B	0.012927601	-0.019349099	0.109806	-0.08208	0.100842	-0.11188
LAPTM5	0.032640984	-0.033255076	-0.14056	-0.06354	0.21552	0.052629
LARGE	-0.031215601	-0.022185174	-0.07632	-0.02485	0.020639	-0.17272
LARGE-AS:	-0.085012867	-0.07207781	-0.04163	-0.02415	0.045185	0.041307
LARP1	0.020286469	-0.04835805	0.197675	0.055311	-0.16361	0.021335
LARP1B	-0.024147823	-0.089417907	-0.03324	0.030006	-0.09089	-0.06618
LARP4	-0.005954763	0.016720151	-0.01061	0.049972	-0.18579	-0.00267
LARP4B	-0.040319747	-0.037564766	-0.17857	-0.03092	-0.02843	0.183777
LARP6	0.020623689	0.00156967	0.042313	0.038387	0.052912	0.044475
LARP7	-0.014297057	0.033126942	-0.04089	0.02021	-0.00903	-0.02173
LARS	-0.004225059	0.036047651	0.149935	0.051089	-0.13237	-0.04772
LARS2	0.021646773	0.054411683	0.072679	0.085198	-0.21938	0.015498
LAS1L	0.018137264	-0.022525415	0.097824	0.050575	-0.14605	-0.06301
LASP1	0.051010905	0.17651627	-0.1773	0.021122	-0.05918	-0.04173
LAT	0.017654136	0.120395244	0.526677	-0.02449	0.045686	-0.29678
LAT2	0.016845676	0.111879391	-0.48541	-0.06964	0.179938	0.27843
LATS1	-0.040099841	-0.035841301	0.049889	-0.04306	0.087681	0.009189
LATS2	0.013693486	0.148421537	-0.21455	-0.05998	0.289931	0.249296
LAX1	0.010847131	0.18858231	0.335344	-0.06198	0.137923	-0.37004
LAYN	-0.07442345	-0.033847856	0.034183	0.025209	-0.05187	0.014373

LBH	0.013382404	0.114863611	0.385639	-0.03728	0.086392	-0.47824
LBP	-0.037659548	-0.02400271	0.008496	0.009417	-0.03343	-0.0297
LBR	0.069089805	0.14820665	-0.09709	0.093104	-0.22518	-0.19166
LBX1	-0.032517111	-0.026429667	-0.01983	0.056142	-0.13938	0.04366
LBX1-AS1	-0.040890321	0.0046262	-0.03209	0.014153	-0.00319	0.123212
LBX2-AS1	-0.019073432	0.041898145	-0.22103	-0.02946	0.075046	0.412354
LCA5	-0.055522554	-0.102257986	-0.08339	-0.00471	-0.00499	-0.07058
LCA5L	-0.027558995	-0.026708284	0.029685	0.008018	-0.04221	-0.2289
LCAT	-0.026836626	-0.068703225	-0.06738	-0.00214	0.040489	0.278151
LCE1B	-0.024879392	-0.027682285	0.038112	0.057842	-0.16152	-0.1299
LCE1E	-0.016095958	-0.033250362	0.025644	0.002198	-0.09572	-0.1467
LCE2B	-0.036352887	-0.058677854	-0.0477	0.017889	-0.02379	0.123779
LCE3D	-0.028462358	-0.0170581	0.041567	-0.01058	-0.00866	0.013438
LCK	0.010404886	0.142353698	0.47909	-0.02765	0.070955	-0.34502
LCLAT1	0.016824547	0.018672974	0.250175	0.018317	-0.13457	-0.39397
LCMT1	0.049804281	0.069147355	0.244923	0.032514	-0.12558	0.047443
LCMT2	0.003143276	0.053405444	0.083323	0.055886	-0.17167	-0.19664
LCN1	-0.024382336	-0.089794549	-0.06549	-0.00081	0.029505	-0.05671
LCN10	-0.01245915	-0.074161228	-0.03309	-0.0534	0.114435	-0.16513
LCN12	-0.024124891	-0.039005877	-0.10619	0.010945	0.003772	-0.09327
LCN15	-0.031069968	-0.09476885	-0.03007	0.030581	-0.10316	-0.04561
LCN2	-0.003483636	-0.080906773	0.419109	-0.11978	0.335173	-0.04654
LCN6	-0.036557843	-0.12226629	-0.06009	-0.02341	0.064427	0.035292
LCN8	-0.040484795	-0.079274529	-0.12499	-0.00936	0.048893	-0.07663
LCNL1	-0.017133942	-0.035683607	0.024683	-0.00728	0.004288	-0.1551
LCOR	0.036481997	0.081050486	-0.10296	-0.01576	0.05598	-0.17775
LCORL	-0.003694854	0.036292596	-0.17611	0.005082	0.006564	0.347858
LCP1	0.055232172	0.146593389	-0.19321	0.039109	-0.08435	0.018891
LCP2	0.012949719	0.044531561	0.148173	-0.01493	0.053185	0.013575
LCT	-0.023940621	-0.077968898	0.0401	0.028278	-0.11214	-0.09224
LCTL	-0.110330288	-0.067382775	-0.01677	-0.01771	0.061679	0.01793
LDB1	0.029115803	0.029248235	0.103173	-0.00631	-0.03664	-0.06259
LDB2	-0.017870735	0.299480655	-0.08903	0.015134	-0.00731	-0.16794
LDB3	-0.023830782	-0.093241947	0.071072	0.030356	-0.03633	0.093832
LDHA	0.05756143	0.113218094	-0.01455	0.143283	-0.33387	0.122365
LDHAL6A	-0.030520418	-0.035173954	-0.00222	-0.00334	-0.00048	-0.12022
LDHAL6B	-0.048608764	-0.003599344	-0.04161	0.004256	-0.00122	0.03557
LDHB	0.037738824	0.022933344	0.333322	0.04457	-0.08399	-0.13122
LDHC	-0.021191453	0.042029966	0.00342	0.073886	-0.21238	-0.00631
LDHD	-0.037044754	-0.032338121	-0.0733	-0.01311	0.068487	0.315982
LDLR	0.027515627	0.161536675	-0.03259	0.114342	-0.19163	0.194588
LDLRAD3	-0.011964283	-0.082651566	-0.19165	-0.09034	0.230774	0.476867
LDLRAD4	-0.036762609	-0.049095993	-0.10774	-0.08231	0.183855	0.191775
LDLRAD4-/	-0.0267796	-0.03533469	0.200047	-0.01055	0.026548	-0.0782
LDLRAP1	0.007898966	-0.027358087	0.520021	-0.07518	0.21416	-0.17134
LDOC1	0.005418108	0.037431904	0.201734	-0.06229	0.153636	-0.42572
LDOC1L	0.024129451	0.215161169	0.41808	-0.00992	0.004932	-0.05183
LEAP2	0.002571903	0.032555197	0.163798	0.027366	-0.10237	-0.00468
LECT1	-0.039266939	0.005088634	-0.05382	0.007442	0.004408	0.031218
LECT2	-0.031545309	-0.026457142	-0.05696	0.012489	-0.012	0.067306
LEF1	-0.045032686	-0.145085274	0.604061	-0.11811	0.146335	-0.14554

LEF1-AS1	-0.008149905	-0.136120852	0.513397	-0.06202	0.197352	-0.23712
LEFTY1	-0.032623723	-0.039163688	0.01757	-0.14632	0.424094	-0.10297
LEKR1	-0.037411672	-0.081737962	0.035625	-0.01439	0.062373	-0.13907
LELP1	-0.031953533	-0.02031021	-0.12374	-0.0023	0.027073	-0.02877
LEMD1	-0.023297332	-0.052623311	0.026564	0.033514	-0.08297	-0.06993
LEMD1-AS1	-0.099790886	-0.098049636	-0.00683	-0.01584	0.083359	0.062859
LEMD2	-0.008313405	0.007944916	0.13724	0.036606	-0.06522	0.107541
LEMD3	0.068943849	0.153108767	0.091691	0.003687	-0.005	-0.42963
LENEP	-0.031794068	0.001831683	0.057128	-0.04431	0.080281	-0.04565
LENG1	-0.00332923	-0.028228633	-0.01724	-0.01151	-0.02171	0.0333
LENG8	0.029330098	-0.084405106	0.14302	-0.02092	0.075091	-0.1255
LENG9	-0.028760298	-0.098593622	-0.04561	-0.01265	0.063647	0.07108
LEO1	0.022775907	0.15126638	0.114584	0.053428	-0.13252	0.02864
LEP	-0.018567507	-0.037831178	0.083794	-0.00319	0.012104	0.009559
LEPR	-0.025143613	-0.056664988	-0.01932	0.031711	-0.09077	0.028811
LEPRE1	0.033331278	-0.049628152	-0.05983	0.004476	-0.04791	0.221398
LEPREL1	-0.009612704	-0.092731143	-0.11138	-0.02434	0.065896	-0.15488
LEPREL2	-0.025022391	-0.02119049	-0.10579	0.007575	-0.02095	-0.01144
LEPREL4	-0.010525697	0.192500327	0.168112	-0.01996	0.088255	-0.21191
LEPROT	0.031875846	-0.022834484	-0.25301	-0.03228	0.124842	0.492025
LEPROTL1	0.038313313	0.018781047	0.404913	-0.00769	0.037937	-0.1012
LETM1	-0.02875033	-0.041648129	-0.05788	0.030352	-0.15683	-0.04203
LETM2	-0.024757447	-0.021816632	-0.08849	0.085453	-0.2287	0.141173
LETMD1	0.034692922	-0.0601246	0.310067	0.004016	-0.03064	-0.1126
LFNG	0.012810791	0.063656602	-0.13976	-0.03597	0.081026	0.156448
LGALS1	0.018973551	0.068117775	-0.05683	0.06433	-0.16324	0.459646
LGALS12	-0.007521736	-0.092770981	-0.18339	-0.09213	0.243517	0.20926
LGALS13	-0.037445249	-0.039826495	-0.03004	0.027389	-0.05529	-0.02379
LGALS14	-0.039033166	-0.035624462	0.041881	0.040298	-0.09003	-0.09444
LGALS2	0.002611393	-0.091000548	-0.29066	-0.07037	0.213025	0.607325
LGALS3	0.008399374	-0.045302826	-0.16223	-0.03408	0.087987	0.633899
LGALS3BP	0.006451471	-0.128003572	0.212811	-0.03113	0.06422	0.028997
LGALS4	-0.027480287	0.053965672	0.296521	-0.01077	0.053257	-0.0244
LGALS8	0.052066214	0.026310319	-0.00683	-0.03362	0.060227	0.332143
LGALS8-AS	-0.034109169	-0.03332679	-0.08242	-0.0117	0.004673	0.065926
LGALS9	0.027254182	-0.008082858	-0.45653	-0.12823	0.336637	0.391571
LGALSL	-0.008056913	-0.068477937	-0.1412	-0.11179	0.299625	-0.07312
LGI1	-0.068890407	-0.074450425	-0.00669	-0.02106	0.037827	-0.03472
LGI2	-0.015215454	-0.080160221	0.005771	-0.01992	-0.05593	-0.07008
LGI3	-0.026257504	-0.043825374	-0.01396	0.006809	-0.11797	-0.11422
LGI4	-0.025712731	-0.097598053	-0.02268	0.000325	-0.06283	-0.04098
LGMN	0.008256544	-0.106248574	-0.05773	-0.02764	0.086991	-0.21029
LGR4	-0.045467331	-0.042012121	0.012693	0.009109	-0.02848	-0.00679
LGR5	-0.057240901	-0.059298059	0.004702	-0.00574	0.018971	-0.05953
LGR6	-0.004755265	0.328609425	0.180676	-0.06234	0.18864	-0.22448
LGSN	-0.052784699	-0.02212479	0.087803	-0.00457	-0.0035	-0.15394
LHCGR	-0.072605967	-0.018717164	-0.0181	-0.02049	0.04019	0.018046
LHFP	-0.02017589	-0.081000138	-0.01511	-0.02238	0.034115	-0.08975
LHFPL1	-0.045644676	0.004335234	-0.0182	-0.02857	0.105116	-0.05147
LHFPL2	0.001882944	-0.102792741	-0.2103	-0.00715	0.012464	0.121712
LHFPL3	-0.034200612	-0.009224664	0.151822	0.037555	-0.09703	-0.04459

LHFPL3-AS	-0.043805364	-0.049928588	-0.02925	-0.00615	0.115452	0.058748
LHFPL3-AS	-0.024113192	-0.043159855	-0.0334	0.003632	-0.06774	-0.06055
LHFPL4	-0.029036018	-0.014885335	0.116962	-0.01499	-0.03003	-0.11359
LHFPL5	-0.038787922	-0.090793771	-0.02552	0.007464	0.001644	0.174611
LHPP	-0.003084538	-0.099864613	-0.05487	-0.07175	0.191642	0.096708
LHX1	-0.035441676	-0.008950689	-0.01704	0.010898	-0.02151	0.060298
LHX2	-0.047715691	-0.03595195	-0.04118	-0.00454	0.039887	0.021024
LHX3	-0.027903438	-0.019983959	0.113226	0.002596	-0.0244	-0.08944
LHX4	-0.069330773	-0.117475592	-0.08855	-0.00115	-0.08283	-0.03784
LHX4-AS1	-0.005636301	0.005023773	0.029009	0.000266	0.02499	-0.01616
LHX5	-0.029070425	-0.074279097	0.053491	0.008099	-0.00772	0.10519
LHX6	-0.044551827	-0.064098975	-0.04518	-0.00263	-0.00514	0.132461
LHX8	-0.085970843	-0.063906464	0.03626	-0.02448	0.070269	-0.08414
LHX9	-0.055657125	-0.038459809	-0.15258	0.030749	-0.01754	-0.04673
LIAS	0.012758928	0.035026774	0.176361	0.020384	-0.03511	0.057159
LIF	-0.009138149	0.008235176	-0.04277	0.054012	-0.189	0.003415
LIFR	-0.018060805	0.023748989	-0.04124	-0.06233	-0.01458	-0.11432
LIG1	0.009177569	0.026840179	0.31017	0.080427	-0.26913	-0.33667
LIG3	0.018089325	0.098634519	0.013878	0.039824	-0.19626	-0.03194
LIG4	0.007558323	0.043143037	-0.18966	0.027908	-0.05829	0.035933
LILRA1	-0.000529628	-0.112767241	-0.36312	-0.11483	0.32082	0.572804
LILRA2	0.013285566	-0.116575904	-0.3787	-0.08198	0.207345	0.494767
LILRA3	0.001514939	-0.086575079	-0.33319	-0.01447	0.037663	0.640041
LILRA4	0.002251839	-0.106582661	-0.15111	-0.05431	0.139826	-0.2108
LILRA5	-0.006443756	-0.11316957	-0.35214	-0.05934	0.148981	0.61415
LILRA6	0.007120973	-0.09306012	-0.37484	-0.11521	0.325626	0.464532
LILRB1	0.020447892	-0.118703225	-0.34192	-0.09874	0.274786	0.504039
LILRB2	0.010725271	-0.13297225	-0.41715	-0.09896	0.273904	0.523512
LILRB3	0.025351762	-0.138695993	-0.3304	-0.08164	0.224323	0.436787
LILRB4	-0.004872129	-0.112119625	-0.28769	-0.10553	0.311204	0.429579
LILRB5	-0.010759727	-0.062893489	-0.10457	-0.01843	0.076368	0.111726
LILRP2	-0.017096569	0.030898815	-0.10798	0.071446	-0.1952	0.074031
LIM2	-0.009781168	0.23531799	-0.04403	0.033258	-0.10973	-0.07234
LIMA1	0.007031157	0.09573329	0.26793	0.015317	-0.04611	-0.27241
LIMCH1	-0.014084639	-0.044114485	-0.01197	0.09431	-0.14941	-0.06371
LIMD1	0.016798629	0.097906136	-0.06463	0.032631	-0.01431	-0.09877
LIMD1-AS1	-0.022476524	-0.010378213	0.08001	-0.01707	-0.06412	-0.16061
LIMD2	0.024791544	-0.082380422	-0.02545	-0.02595	0.049578	0.036357
LIME1	0.009567654	-0.006546034	0.349069	-0.0431	0.12954	-0.30491
LIMK1	-0.042630104	-0.022978526	-0.05313	-0.00878	-0.05983	-0.11393
LIMK2	0.026291272	-0.019337419	-0.18775	0.012094	-0.03973	-0.02959
LIMS1	-0.019209374	-0.008853923	0.014057	-0.02195	0.063076	-0.04147
LIMS2	-0.009277672	-0.103068263	-0.00969	-0.04499	0.029701	-0.16579
LIN28A	-0.043394883	0.020539823	0.120114	-0.00604	0.033639	-0.07913
LIN28B	-0.027397735	-0.048627585	0.145341	-0.00573	0.027132	-0.10136
LIN37	0.004979792	0.002258314	-0.12487	0.000979	-0.04732	0.045523
LIN52	0.019564915	0.085634204	0.170828	0.075686	-0.21058	-0.12886
LIN54	-0.020655826	0.107746489	0.007649	0.055119	-0.1403	-0.05041
LIN7A	-0.021740091	-0.060862737	-0.24225	-0.02068	0.06689	0.274617
LIN7B	-0.006794584	-0.013546869	0.15899	-0.01361	0.07784	0.088876
LIN7C	0.041921301	0.054179306	0.026179	0.025636	-0.05034	0.187389

LIN9	-0.046051661	-0.021986287	0.000287	-0.02283	-0.12627	0.089634
LINC-PIN	0.009438545	-0.033673601	0.065121	0.007325	-0.0311	-0.36126
LINC0002	-0.024174995	-0.038363071	0.023337	-0.01377	0.003632	-0.08443
LINC0002	-0.038762514	-0.036354937	-0.02423	0.01852	-0.03098	0.075507
LINC0003	-0.043509277	-0.109889994	0.177244	-0.00275	-0.01351	-0.12976
LINC0005	-0.059446089	-0.057825115	-0.04985	-0.0308	0.105279	0.011041
LINC0005	-0.035467151	-0.039047754	0.049122	-0.00779	0.034077	0.031765
LINC0008	-0.02180041	-0.075106042	0.31695	-0.01954	0.071877	-0.16442
LINC0009	-0.023650131	0.087580671	0.149041	0.016424	-0.08673	-0.13591
LINC0009	0.005545987	0.010776316	0.231905	-0.0258	0.08165	-0.08835
LINC0010	-0.049082831	-0.014955033	-0.13398	-0.002	0.040699	-0.00606
LINC0011	-0.032973818	-0.040659393	-0.02892	-0.00891	0.032922	-0.09254
LINC0011	-0.05317808	-0.090081685	0.001573	-0.03461	0.119575	0.114272
LINC0011	-0.017580148	0.023457893	-0.01682	0.014783	-0.02898	0.083756
LINC0011	0.016210652	0.021051454	0.234373	0.047655	-0.11679	0.197153
LINC0015	-0.019515625	-0.050416726	-0.0549	0.033722	-0.13565	-0.07978
LINC0016	-0.075295201	-0.03954559	-0.05183	0.022516	-0.04744	-0.02417
LINC0016	-0.040872135	-0.020651549	0.003348	0.02789	-0.04469	0.139632
LINC0016	-0.036344303	-0.01159498	0.077709	-0.01733	0.022176	-0.04433
LINC0016	-0.034007356	-0.072761963	0.054625	-0.0118	0.060403	0.008666
LINC0016	-0.038285683	-0.04796755	-0.04611	0.002247	0.01711	-0.13923
LINC0017	-0.010552292	-0.175436332	-0.2059	-0.03709	0.086224	0.012026
LINC0017	-0.023674928	-0.034786062	0.104296	0.002032	-0.06563	-0.01665
LINC0017	-0.018814858	-0.056423323	0.069988	0.08249	-0.23241	0.026838
LINC0018	-0.044969898	-0.04832424	-0.12714	0.0101	-0.00813	0.051796
LINC0018	-0.022191338	-0.062736892	-0.20311	-0.06503	0.19521	0.239081
LINC0020	-0.022029418	-0.02935444	0.087617	-0.00747	0.00342	-0.13171
LINC0020	-0.020343002	-0.095191466	0.046544	-0.05079	0.072599	-0.18235
LINC0020	-0.027622661	0.057504473	0.082494	0.005823	-0.02238	-0.05628
LINC0021	-0.055688752	-0.022694152	-0.09246	-0.00765	0.044609	0.156002
LINC0021	-0.032151263	-0.014107059	-0.01786	0.005786	-0.00282	-0.01787
LINC0022	-0.054540529	0.01961447	0.000792	0.01657	-0.04251	0.035712
LINC0022	-0.052337387	-0.05620125	-0.02999	-0.02448	0.061325	0.006747
LINC0023	-0.090947214	-0.097916542	0.056455	-0.01606	0.091777	0.017567
LINC0024	-0.063256589	-0.010250237	-0.0145	-0.02642	0.0765	-0.03771
LINC0024	-0.036357532	-0.05384783	0.002774	0.009054	-0.00382	0.121881
LINC0024	-0.03157057	-0.071961935	0.021304	0.011218	-0.03073	-0.04582
LINC0026	-0.002993615	0.160460203	-0.00917	0.012595	-0.01417	-0.12429
LINC0026	-0.019212628	-0.036046572	-0.02423	0.092219	-0.27577	-0.06666
LINC0026	-0.025499703	-0.00687055	0.087169	0.069624	-0.19821	-0.13328
LINC0026	-0.025111717	-0.019202254	-0.01436	-0.05601	0.152853	-0.01857
LINC0026	-0.028827699	-0.06531315	-0.11196	0.015642	-0.07449	0.058408
LINC0027	-0.023164891	-0.018323782	-0.09159	-0.00671	0.032592	-0.07109
LINC0027	-0.042324759	-0.078588138	0.057985	-0.00547	0.027966	0.033343
LINC0027	-0.02147133	-0.062931127	1.06E-05	########	-0.04016	-0.11355
LINC0028	-0.040249467	0.037267936	-0.09283	0.003251	0.011291	-0.05925
LINC0028	-0.009691938	-0.111555951	0.304948	-0.0381	0.12725	-0.27134
LINC0028	-0.038923509	-0.034485885	0.075186	0.050388	-0.12918	-0.14791
LINC0029	-0.056429068	0.012084443	-0.10567	-0.01385	0.033851	-0.06147
LINC0029	-0.037033139	0.00436056	-0.02455	0.009824	-0.01622	-0.02227
LINC0029	0.01717369	0.067523982	-0.07665	0.017006	-0.06482	0.151891

LINC0029[illegible]	-0.035001246	0.098163232	-0.09774	0.014004	-0.03842	-0.05456
LINC0029[illegible]	-0.043253544	0.116978887	-0.04989	0.042046	-0.09155	-0.07671
LINC0030[illegible]	0.007000021	-0.061538513	0.006034	0.02421	0.069216	0.000178
LINC0030[illegible]	-0.052522954	-0.047171053	-0.16294	0.017264	-0.01625	0.008973
LINC0030[illegible]	-0.0174986	-0.088284764	-0.19929	-0.00877	0.053443	0.21688
LINC0030[illegible]	-0.050181576	-0.010263391	-0.0062	0.014789	-0.0223	-0.009
LINC0030[illegible]	-0.073941563	-0.103826619	0.040049	-0.00833	0.015445	0.092858
LINC0030[illegible]	-0.048272809	-0.049262235	-0.06889	0.016538	-0.0281	-0.00025
LINC0030[illegible]	-0.0633538	-0.105312367	0.061054	-0.00867	0.011873	-0.01585
LINC0031[illegible]	-0.040714357	-0.001658852	-0.05937	0.031508	-0.06036	0.101639
LINC0031[illegible]	-0.048941868	-0.050149898	-0.01722	-0.01098	0.061297	0.146371
LINC0031[illegible]	-0.031081652	-0.038387311	0.030144	0.00493	-0.04739	-0.07562
LINC0031[illegible]	-0.055397803	-0.052524573	-0.07278	0.035495	-0.07665	0.130084
LINC0031[illegible]	-0.073371322	-0.035690825	-0.15352	-0.00246	0.001224	-0.12495
LINC0031[illegible]	-0.026739469	-0.023547029	0.020751	0.000623	-0.10334	-0.13229
LINC0031[illegible]	-0.032806652	-0.019405418	0.05355	-0.01082	0.054526	-0.19605
LINC0031[illegible]	-0.03394904	-0.077566601	-0.05902	-0.02546	0.065486	0.161902
LINC0032[illegible]	-0.060535314	-0.104286474	0.038102	-0.00483	0.004676	-0.00415
LINC0032[illegible]	-0.078141225	-0.042431766	0.060859	-0.01576	0.039753	-0.02597
LINC0032[illegible]	0.015227164	0.007071844	-0.01024	-0.08161	0.208629	0.236884
LINC0032[illegible]	-0.051280849	0.026513267	0.00859	-0.02348	0.077206	-0.06107
LINC0032[illegible]	-0.028356431	-0.036316705	-0.03916	0.031703	-0.06336	-0.00766
LINC0032[illegible]	-0.036281531	-0.016715812	0.039009	1.59E-05	0.006931	-0.07638
LINC0033[illegible]	-0.032397291	-0.04515706	-0.00601	0.001323	-0.01408	-0.05088
LINC0033[illegible]	0.013814655	-0.117737113	-0.01531	0.032674	-0.09381	0.015798
LINC0033[illegible]	-0.032718791	-0.018991826	0.049624	0.125651	-0.34122	-0.07801
LINC0033[illegible]	0.013430882	0.039048609	0.141797	-0.00329	-0.02388	-0.01949
LINC0034[illegible]	0.019294062	0.01303891	0.335406	0.03202	0.005589	-0.33202
LINC0034[illegible]	-0.060464764	0.066735436	-0.03221	-0.02674	0.07359	-0.0173
LINC0034[illegible]	-0.056540788	-0.089956019	-0.01045	-0.03599	0.117538	-0.04986
LINC0034[illegible]	-0.056793333	-0.032556551	0.018224	0.00134	-0.00906	-0.07257
LINC0035[illegible]	-0.047927843	-0.051150906	-0.00806	-0.00881	0.046676	-0.10084
LINC0035[illegible]	-0.037886081	-0.009919513	-0.01592	0.036069	-0.09005	-0.03521
LINC0035[illegible]	-0.039873203	-0.072008051	-0.02944	-0.00511	0.017832	-0.14994
LINC0038[illegible]	-0.048774059	-0.055044974	-0.00135	0.031483	-0.06658	-0.06665
LINC0040[illegible]	-0.13171602	-0.107237394	0.081898	-0.01761	0.088074	0.036397
LINC0040[illegible]	-0.042927642	-0.07689131	-0.02927	0.138677	-0.17103	-0.1123
LINC0041[illegible]	-0.030519823	-0.046962978	-0.09879	0.03172	-0.06904	-0.0223
LINC0042[illegible]	-0.032389216	-0.048895744	-0.02242	0.032933	-0.1045	-0.04353
LINC0042[illegible]	-0.035984424	-0.08573936	-0.08715	0.01388	-0.02208	-0.07606
LINC0042[illegible]	-0.038586558	-0.062584363	-0.00499	-0.0155	-0.05631	-0.20438
LINC0042[illegible]	-0.037188651	-0.008953578	0.066383	-0.01933	0.076209	0.081883
LINC0044[illegible]	-0.057520555	-0.01313347	0.116626	0.037236	-0.09959	-0.10554
LINC0044[illegible]	-0.033359752	-0.036840277	-0.05038	0.024967	-0.06258	0.002899
LINC0045[illegible]	-0.034568472	-0.047936799	-0.08096	0.002675	-0.03807	-0.09213
LINC0045[illegible]	-0.032504913	-0.006545862	0.033032	0.029082	-0.0795	0.050644
LINC0045[illegible]	-0.089905757	-0.064387947	0.065152	-0.01857	0.002657	-0.00853
LINC0046[illegible]	-0.053194092	-0.090895149	-0.03418	0.040308	-0.09898	0.005641
LINC0046[illegible]	-0.049637032	-0.033813605	0.019775	0.014911	-0.0003	-0.01445
LINC0046[illegible]	-0.026354089	-0.043097846	0.003358	0.022246	-0.04043	0.082935
LINC0046[illegible]	-0.042141772	-0.067952684	-0.05166	0.014457	-0.01875	-0.11633

LINC0046'	-0.010263656	-0.005175747	-0.11112	0.059321	-0.15862	0.124739
LINC0047(	-0.038995926	-0.081295082	0.122953	0.025793	0.057083	-0.03238
LINC0047]	-0.055752327	-0.044489977	0.056166	0.005575	-0.01748	0.001576
LINC0047:	-0.031425897	-0.049016862	-0.02464	-0.01606	0.015376	-0.06916
LINC0047:	-0.040938327	-0.035630712	-0.03303	-0.0183	0.076174	-0.02053
LINC0047⁄	-0.058570411	-0.02818285	-0.09143	0.012694	-0.04275	-0.14052
LINC0047!	0.016553611	-0.113507915	0.055027	0.015589	-0.13728	-0.0102
LINC0047(	-0.009067262	0.061167754	0.063948	-0.01863	0.072081	-0.06989
LINC0047'	-0.032660102	-0.070781147	-0.03393	0.001243	0.003726	-0.07874
LINC0047{	0.043811614	-0.038326438	0.019244	-0.0703	-0.25352	-0.03178
LINC0047!	-0.032450341	-0.033706937	-0.03692	-0.00121	-0.00375	0.059021
LINC0048:	-0.016793382	-0.088509156	0.000583	-0.0075	-0.05037	-0.04139
LINC0048:	-0.047788068	-0.016375561	-0.02944	-0.04129	0.131923	-0.0572
LINC0048!	-0.099627918	-0.099854124	0.031171	0.007522	0.062706	-0.01805
LINC0048(	-0.043410549	-0.003094906	-0.04066	0.048564	-0.12081	-0.05984
LINC0048'	-0.038801518	-0.033060539	-0.03812	-0.02862	0.06746	-0.07879
LINC0048{	-0.047490896	-0.095469708	0.126552	-0.01959	0.080907	-0.15483
LINC0049]	-0.112611409	-0.050863319	0.021298	-0.06705	0.059161	0.005593
LINC0049:	0.063742976	-0.003526943	0.131911	0.027143	-0.07105	0.142277
LINC0049⁄	-0.006319911	-0.095446282	-0.2037	-0.01557	0.049176	-0.17519
LINC0050'	-0.112123267	-0.042566243	-0.04697	-0.01669	0.031296	0.093853
LINC0051⁄	-0.030638132	-0.01751138	-0.00572	-0.00834	0.050721	0.006945
LINC0051!	-0.018258307	-0.028745164	-0.00741	0.002524	-0.05168	-0.04595
LINC0051{	-0.043842368	-0.036904466	-0.02837	-0.01216	-0.01601	-0.07859
LINC0052(	0.011480976	-0.035099658	0.00407	0.067363	-0.21308	-0.08691
LINC0052]	-0.032463448	-0.008974859	-0.11797	0.017182	0.00521	0.050821
LINC0052:	-0.013653505	-0.065212445	-0.03596	-0.00839	-0.09015	-0.1401
LINC0052!	-0.021894927	-0.036447559	0.036029	-0.00961	0.014402	-0.14453
LINC0052(	0.003526167	0.06474684	0.248897	-0.07168	0.201996	-0.18
LINC0052'	-0.041665576	-0.054090508	0.065234	-0.01258	0.020524	0.022219
LINC0052{	0.003979263	0.014724384	-0.08215	-0.00213	0.031064	-0.00306
LINC0052!	-0.052135712	-0.048351749	-0.08227	0.014499	-0.05175	0.137991
LINC0053!	-0.046328506	-0.016703453	-0.06118	0.029192	-0.05496	-0.01487
LINC0053(	-0.048530895	-0.051297006	-0.0508	-0.02174	0.084875	0.090756
LINC0053'	-0.013177139	-0.049325672	0.093411	-0.02456	-0.0238	-0.05004
LINC0054(	-0.010091309	-0.030250267	0.040971	-0.00845	0.015118	-0.08191
LINC0054⁄	-0.037437991	-0.070908291	0.017671	0.017096	-0.02492	-0.14808
LINC0054!	-0.029563021	-0.050864054	0.054322	-0.01161	0.036427	-0.0775
LINC0055(	-0.071320967	-0.08240907	0.053035	-0.02101	0.037885	0.009792
LINC0055]	-0.093924339	-0.100080936	0.059252	0.002916	0.063503	0.008702
LINC0055!	-0.037190462	-0.056466097	-0.01424	0.026168	-0.04901	0.045013
LINC0055(	-0.024450991	-0.042183569	-0.00221	0.001976	0.003033	-0.07619
LINC0055'	-0.033265889	-0.020759359	0.001617	0.01298	-0.02758	-0.16676
LINC0055{	-0.036953147	-0.078250022	0.005575	-0.01913	0.036841	-0.1141
LINC0055!	-0.052789956	-0.042517593	-0.04455	-0.03228	0.114573	0.084417
LINC0056(	-0.050903912	-0.102177066	-0.01074	-0.01746	0.072267	-0.00977
LINC0056]	-0.053310169	-0.059946496	0.022124	-0.02425	0.064785	0.070424
LINC0056:	-0.029735413	-0.009883227	0.063969	-0.00947	0.020623	-0.0583
LINC0056⁄	-0.096266059	-0.070978425	-0.03174	-0.02041	0.063537	-0.0267
LINC0056!	-0.01115029	0.040664186	0.324267	-0.03563	0.110835	-0.18147
LINC0056(	-0.045543954	-0.009904348	-0.01586	0.001699	0.004045	0.002183

LINC00567	-0.047765257	0.022959143	0.059388	-0.00238	0.025584	-0.02049
LINC00572	-0.044565283	-0.012894481	0.012495	0.018422	-0.01631	0.111697
LINC00574	-0.027496128	-0.017970352	0.044655	0.001758	-0.0225	-0.02471
LINC00577	-0.034678996	-0.058474495	0.044214	0.007444	-0.0379	0.083439
LINC00582	-0.037929739	-0.045337535	0.032725	0.013312	-0.05243	-0.02362
LINC00587	-0.039484025	-0.011078683	-0.02558	0.006224	-0.0068	-0.0811
LINC00588	-0.029780944	-0.022579874	0.015631	0.063038	-0.12482	-0.13494
LINC00589	-0.028068968	-0.044800134	0.106883	0.037646	-0.11512	-0.01283
LINC00592	-0.029313023	-0.034450103	0.053073	0.036746	-0.09558	0.00826
LINC00593	-0.052317234	-0.069434455	-0.0498	0.012445	-0.00535	-0.05895
LINC00595	-0.033938736	0.006545974	0.051393	0.015927	-0.03848	-0.02973
LINC00597	-0.010383152	0.111428837	-0.06965	-0.00517	0.007207	-0.0067
LINC00598	-0.037596061	-0.066973946	-0.00727	0.027809	-0.10085	-0.12044
LINC00605	-0.023392135	-0.030510391	0.023905	-0.01715	0.031361	-0.05826
LINC00606	-0.080132247	-0.061466283	-0.02125	0.009544	-0.0032	0.081528
LINC00607	-0.02968428	-0.00655078	-0.06227	0.009269	-0.01591	-0.01703
LINC00608	-0.050766307	-0.055136404	-0.12485	-0.00629	0.035361	0.09914
LINC00610	-0.031251682	-0.02993691	0.070667	0.006761	-0.01159	0.175678
LINC00612	-0.020965232	-0.055151234	0.024714	0.000604	-0.01764	-0.13805
LINC00615	-0.068238866	-0.062240502	0.074572	0.011294	-0.0235	0.001428
LINC00616	-0.060407157	-0.051071399	-0.12016	-0.00498	0.021556	0.037779
LINC00619	-0.066925162	-0.022522818	0.021686	-0.02613	0.061606	0.012688
LINC00620	-0.037716712	-0.039648909	-0.04941	0.003031	0.023382	0.034966
LINC00621	0.044394105	0.036869566	0.020538	0.017321	-0.06082	0.037656
LINC00622	-0.019644627	-0.099358625	-0.14196	-0.01634	0.03683	0.453672
LINC00623	0.048879344	0.188774827	0.264299	-0.00497	0.035521	-0.18505
LINC00624	-0.056213071	-0.01245538	-0.02621	0.012281	-0.03874	0.071189
LINC00626	-0.042143011	-0.115234123	0.112483	0.031849	-0.06194	-0.00049
LINC00628	-0.041931152	-0.088195801	-0.11182	0.018202	-0.03936	-0.0055
LINC00629	-0.027258807	-0.019747829	-0.05033	-0.00084	0.014755	-0.14075
LINC00630	-0.073399411	-0.002264982	0.002518	0.040998	-0.1116	0.045903
LINC00632	-0.059599694	-0.086581069	-0.09419	-0.00607	0.031965	0.018154
LINC00635	-0.049012391	-0.066593569	0.063358	-0.00425	0.02032	0.014619
LINC00636	-0.051052078	-0.035308884	-0.10721	0.001351	0.035857	-0.02335
LINC00638	-0.028788977	-0.055730757	0.041298	0.010441	-0.12241	-0.06038
LINC00639	-0.029811481	-0.072984448	-0.13218	0.003565	-0.00235	0.024767
LINC00641	-0.028771908	0.014091747	0.03532	-0.02557	0.086944	0.083573
LINC00642	-0.063613825	-0.062181524	-0.00609	-0.05766	0.098396	-0.05331
LINC00643	-0.035452184	-0.074370919	0.016225	-0.01011	0.01449	-0.05775
LINC00644	-0.06947925	0.017094218	-0.06083	-0.04756	0.12867	0.017661
LINC00645	-0.052460039	-0.065179248	0.051815	0.00063	0.002523	-0.11366
LINC00648	-0.02220316	-0.070844483	0.03548	0.004278	-0.02901	-0.09431
LINC00652	-0.032919129	-0.042741093	-0.06664	-0.00328	-0.04507	-0.05478
LINC00654	-0.026326896	-0.017339995	0.048149	0.001544	0.003763	-0.06134
LINC00656	-0.033459902	-0.082438345	-0.05173	-0.0006	-0.04047	0.012768
LINC00657	0.049708004	0.160724038	0.019274	-0.00972	0.082226	0.156986
LINC00658	-0.034801319	-0.149677796	0.072416	-0.00853	0.056971	-0.05709
LINC00659	-0.036615342	-0.041177892	-0.03843	0.034947	-0.07909	0.101853
LINC00661	-0.048461242	-0.087680912	-0.15011	0.002468	0.014923	-0.13407
LINC00662	-0.012290231	0.018191881	0.105809	-0.07446	0.038157	0.053444
LINC00663	-0.042869051	-0.026801689	0.344812	0.019519	0.062635	-0.31598

LINC0066[illegible]	-0.059687488	0.01427718	-0.06356	-0.03785	-0.01455	-0.16073
LINC0066[illegible]	-0.062392967	-0.001738928	-0.0845	-0.02167	0.051472	-0.17299
LINC0066[illegible]	0.019280029	-0.014960934	0.095378	-0.10216	0.274996	-0.06794
LINC0066[illegible]	-0.020118957	-0.029897742	-0.019	-0.01211	-0.03306	-0.04608
LINC0066[illegible]	-0.055510467	-0.107969738	-0.05126	-0.01682	0.038337	0.254282
LINC0067[illegible]	-0.062080826	-0.048622159	0.051329	-0.00036	0.006937	-0.0465
LINC0067[illegible]	-0.000197326	0.080780151	0.165268	-0.04831	0.131914	-0.19891
LINC0067[illegible]	-0.041640637	-0.077885599	-0.00394	0.014414	-0.0137	0.016296
LINC0067[illegible]	0.003037986	0.142536914	-0.06169	-0.0902	0.247422	0.132109
LINC0067[illegible]	-0.04750637	-0.010220533	-0.01466	0.010374	-0.05223	-0.09279
LINC0068[illegible]	-0.046023129	-0.031828176	-0.02062	0.007738	-0.01658	0.002848
LINC0068[illegible]	-0.009132438	0.020765848	0.218843	-0.0149	0.025386	-0.21543
LINC0068[illegible]	-0.024769121	-0.05093088	-0.02366	-0.00652	-0.00022	0.015063
LINC0068[illegible]	-0.036810466	-0.04708555	-0.09944	-0.03361	-0.04449	0.018707
LINC0069[illegible]	-0.047361179	-0.047391581	-0.14498	0.005969	-0.00314	-0.01338
LINC0069[illegible]	-0.045357222	-0.031590647	0.077633	0.007594	-0.00344	-0.08005
LINC0069[illegible]	-0.062137256	-0.083783502	0.032983	-0.00923	0.025216	-0.05227
LINC0069[illegible]	-0.016581016	0.033267726	-0.0103	0.115605	-0.30384	0.009379
LINC0070[illegible]	-0.023528954	-0.018570912	0.002869	-0.01547	0.000846	-0.12484
LINC0070[illegible]	-0.073159624	-0.018485456	-0.04777	-0.01314	0.020823	-0.00201
LINC0070[illegible]	-0.029191707	-0.088237234	-0.01444	0.001046	0.013459	-0.06066
LINC0070[illegible]	-0.02821001	0.02006738	-0.03211	0.008925	-0.07211	-0.05414
LINC0070[illegible]	-0.040399462	-0.003673913	0.024418	-0.01191	0.060563	-0.00195
LINC0071[illegible]	-0.066038205	0.014833119	-0.08137	-0.00726	0.014793	0.10924
LINC0082[illegible]	-0.037332573	-0.01531889	0.169916	0.006533	-0.03349	-0.02706
LINC0083[illegible]	-0.038654356	-0.101685933	-0.04716	-0.00966	0.057426	0.11456
LINC0083[illegible]	-0.047052343	-0.028079087	-0.12664	-0.0256	0.068278	-0.01041
LINC0083[illegible]	-0.047017821	-0.001265938	0.125914	0.032537	-0.06627	0.010937
LINC0083[illegible]	-0.029850632	-0.037598285	-0.00308	-0.02082	0.056515	0.131551
LINC0084[illegible]	-0.028098717	-0.047327963	-0.10477	-0.00873	0.041588	0.045841
LINC0084[illegible]	-0.032167615	-0.019033343	0.025846	0.010572	-0.00333	-0.16977
LINC0084[illegible]	-0.076651104	-0.045533919	-0.03468	-0.00911	0.017076	0.007512
LINC0084[illegible]	-0.034869746	-0.032519934	0.031355	-0.01565	0.06314	0.023866
LINC0084[illegible]	0.015671882	0.034251593	0.072064	0.029956	-0.05434	0.100474
LINC0085[illegible]	-0.018268603	-0.052394124	0.023297	-0.03034	-0.01005	-0.13034
LINC0085[illegible]	-0.008414425	0.029504964	0.171921	-0.00923	0.065895	-0.07689
LINC0085[illegible]	-0.040035438	-0.031415388	-0.04354	-0.11955	0.370394	0.081841
LINC0085[illegible]	-0.029983645	-0.08131919	-0.05486	-0.01956	0.054709	-0.08737
LINC0085[illegible]	-0.04304473	-0.058072644	0.056254	-0.01481	0.079649	0.009555
LINC0085[illegible]	-0.04653449	-0.037510617	0.018167	0.028199	-0.05074	-0.04544
LINC0086[illegible]	-0.03944695	-0.074882267	-0.05361	0.024008	-0.05056	-0.0892
LINC0086[illegible]	-0.005049693	-0.104013066	-0.09415	-0.01159	0.035487	-0.20107
LINC0086[illegible]	-0.039964891	-0.067587548	0.004299	0.021605	-0.03906	0.114362
LINC0086[illegible]	-0.034259267	0.00041202	-0.0166	0.010073	-0.06451	-0.04309
LINC0086[illegible]	-0.017190352	0.088493491	0.066525	-0.00879	0.017112	-0.07954
LINC0087[illegible]	-0.044235517	-0.017049211	0.079069	-0.01117	0.056413	-0.06171
LINC0087[illegible]	-0.00338845	-0.117200672	-0.28589	-0.05072	0.142936	0.332564
LINC0087[illegible]	-0.028439521	-0.024691096	0.00668	0.012951	-0.02337	-0.10452
LINC0088[illegible]	-0.031294518	-0.062224722	0.035728	0.051745	-0.13527	-0.0776
LINC0088[illegible]	-0.02934551	-0.047367598	-0.21188	0.049886	-0.12411	-0.05252
LINC0088[illegible]	-0.037615949	-0.048726633	0.089887	0.027974	-0.05276	-0.09671

LINC0088:	-0.028953038	0.033044004	0.067295	0.08882	-0.21984	-0.15944
LINC0088	-0.038099888	-0.100382765	0.06985	0.025211	-0.08029	0.016143
LINC0088(	-0.010190879	-0.008777648	0.03734	0.000538	-0.002	0.034566
LINC0088'	-0.041764014	-0.072687491	0.159388	0.011219	-0.01413	-0.0767
LINC0088:	0.016322262	0.131715036	-0.01243	0.079627	-0.18754	0.057975
LINC0088!	-0.058399738	-0.08235194	-0.0677	-0.0351	0.099781	-0.01201
LINC0089:	-0.0212382	-0.020928413	0.014157	0.079011	-0.18671	-0.04491
LINC0089:	-0.014238099	0.007206183	0.007266	-0.02373	0.075633	-0.0852
LINC0089	-0.03092502	-0.019482308	0.046644	-0.06289	0.150583	-0.02348
LINC0089!	-0.045837573	-0.049621043	0.146967	-0.01448	0.04579	-0.03254
LINC0089(	-0.028153353	-0.030972161	0.00759	-0.00588	0.020843	0.048748
LINC0089!	-0.028617129	-0.01959215	-0.06917	-0.05106	0.152574	0.13072
LINC0090(	-0.03936251	-0.080878797	-0.09486	-0.0189	0.075722	0.099229
LINC0090:	-0.056736941	-0.038766857	0.027615	-0.03404	0.083066	-0.09333
LINC0090!	-0.036096189	-0.047637881	-0.10343	-0.01317	0.039881	0.014239
LINC0090(	-0.038540464	0.024955701	-0.12645	0.012895	-0.01548	-0.12407
LINC0090'	-0.012480787	-0.046097057	-0.00542	0.023289	0.0006	-0.19125
LINC0090:	-0.031470415	-0.025064066	-0.05443	0.05422	-0.15105	-0.02365
LINC0090!	0.022111107	0.07475108	0.134337	-0.02123	0.048228	-0.31514
LINC0091(	-0.036934664	-0.036666594	-0.04566	-0.03242	0.111066	-0.10672
LINC0091:	-0.025291751	-0.047158692	0.017572	0.056116	-0.1525	-0.03681
LINC0091'	-0.039434806	-0.102832415	-0.06689	0.006533	0.016853	-0.01228
LINC0091!	-0.037072699	-0.047799065	0.020438	0.008239	-0.00327	-0.0115
LINC0092(	-0.011539667	-0.030270204	0.382969	0.001626	0.024566	-0.14912
LINC0092:	-0.034102158	-0.005626293	-0.11067	0.010325	-0.01594	-0.01946
LINC0092:	-0.04233124	-0.041791635	0.065063	0.008393	-0.00245	-0.14679
LINC0092	-0.039837492	0.006666902	-0.04106	0.020663	-0.03168	-0.06919
LINC0092!	-0.020763085	0.033604256	0.056435	-0.00026	0.026257	-0.23817
LINC0092(	-0.002936352	-0.076566933	-0.09373	-0.05655	0.144676	-0.05357
LINC0092'	-0.043176011	-0.096028352	-0.02926	0.021399	-0.0393	-0.12306
LINC0092:	-0.035376311	-0.01894684	-0.02578	-0.00268	0.005565	-0.09038
LINC0092!	-0.030392724	-0.009225016	-0.01076	0.045228	-0.11158	-0.07554
LINC0093(	-0.029436393	-0.025827184	-0.07076	-0.01552	-0.01946	-0.13689
LINC0093:	-0.047057573	-0.056869094	0.080992	0.007042	0.005007	-0.16684
LINC0093	-0.031235428	-0.04244514	0.027942	0.012758	-0.02753	-0.1754
LINC0093!	-0.054714518	-0.0415551	-0.14171	-0.03417	0.124709	-0.00743
LINC0093(	0.013113609	-0.09513445	-0.16525	-0.09697	0.242437	0.256085
LINC0093'	-0.031442233	-0.079253494	0.038785	-0.02946	0.021406	0.059944
LINC0093:	-0.055802144	-0.048881739	0.062585	-0.0442	0.050802	-0.0018
LINC0093!	-0.040303508	-0.028196485	-0.03603	-0.04009	0.143479	-0.01424
LINC0094:	-0.044746974	-0.022614389	0.022316	0.015261	-0.03936	-0.01815
LINC0094:	-0.025952999	-0.061179047	0.015432	0.005675	-0.00602	0.12847
LINC0094	-0.022108661	0.042536068	0.325508	0.037857	-0.08941	-0.19817
LINC0094:	-0.049448368	-0.024043836	-0.02767	-0.00781	0.035424	0.050916
LINC0095(	-0.024318457	-0.074314317	-0.07779	0.001559	0.014204	-0.14923
LINC0095:	-0.035879273	-0.03126143	0.118814	0.018092	-0.05235	-0.10492
LINC0095	-0.006525298	0.02783528	0.244803	-0.05972	0.187529	-0.05678
LINC0095!	-0.054728485	-0.042269611	0.051884	-0.00777	0.018747	-0.00822
LINC0095'	4.93E-05	-0.096802817	0.083152	-0.05852	0.104482	-0.0327
LINC0095:	-0.021620036	-0.009671373	-0.06261	0.048776	-0.13734	0.016835
LINC0095!	0.007367666	0.040993335	0.281651	-0.01986	0.078253	-0.07531

LINC0096(	-0.031959149	-0.018531258	-0.11516	-0.01829	0.061918	0.012463
LINC0096:	-0.026974829	0.042950284	-0.16686	-0.00925	-0.02152	-0.00902
LINC0096:	-0.01429703	-0.104014723	-0.19371	-0.01542	0.034028	0.504545
LINC0096·	-0.053290348	-0.009190288	0.034476	-0.00241	0.02037	-0.13903
LINC0096!	-0.019314398	0.004794134	0.228176	-0.02067	0.044762	-0.17195
LINC0096'	-0.02723251	0.003801183	-0.15657	0.02553	-0.08633	-0.04616
LINC0096{	-0.016809258	-0.086922342	-0.19839	-0.09334	0.273199	0.687861
LINC0096!	-0.006957442	-0.005571099	0.102073	0.006514	-0.01346	-0.04508
LINC0097(	-0.041933771	-0.052602986	0.197927	0.000239	0.019788	-0.09518
LINC0097:	-0.027759231	-0.070494323	-0.00125	0.090097	-0.09853	-0.04042
LINC0098:	-0.050069917	-0.090490615	-0.15494	0.038102	-0.09414	0.249461
LINC0099:	-0.026214442	0.003282241	-0.0802	0.002831	-0.01338	-0.07123
LINC0099(	-0.0015268	0.028438141	-0.17019	0.005976	-0.00849	-0.18493
LINC0099'	-0.03262583	-0.067028612	-0.1117	0.000381	0.043809	-0.1218
LINC0099{	0.018740054	-0.061169578	0.030839	0.037538	-0.12518	0.114259
LINC0100(	0.021584402	-0.118790958	-0.15923	-0.01964	0.057253	0.13631
LINC0100:	0.024073475	0.086216429	-0.09984	-0.05386	0.170706	0.319534
LINC0100·	0.004521318	-0.029447107	-0.0687	0.085199	-0.05101	0.127656
LINC0100'	-0.029856232	-0.003242795	0.005927	0.001654	-0.04425	-0.06409
LINC0101(	-0.018194818	-0.015004902	-0.06018	0.04678	-0.15051	-0.01645
LINC0101:	-0.05192222	-0.063813973	0.024133	-0.02646	0.096118	-0.02816
LINC0101:	-0.029835299	-0.079796828	-0.19796	-0.02291	0.07672	-0.12387
LINC0101!	-0.033209957	-0.077685375	0.027547	-0.00315	0.023045	0.005594
LINC0101(	-0.037321274	-0.096289319	-0.05738	0.004182	-0.03842	0.017085
LINC0101{	-0.025219535	-0.036206837	0.001993	0.007157	-0.03544	-0.08158
LINC0101!	-0.03722961	-0.036681333	0.099137	0.010726	-0.01149	-0.07449
LINC0102(	-0.074084202	-0.039961018	-0.00839	0.027038	-0.05546	-0.06209
LINC0102:	-0.042164318	-0.060504471	-0.04896	0.228515	-0.15931	-0.1436
LINC0102:	-0.025281661	-0.055374374	-0.13792	-0.04916	0.160059	0.18531
LINC0102!	-0.042526161	-0.02528653	-0.00311	-0.02067	0.0871	0.023631
LINC0104!	-0.031452462	-0.058217061	-0.13008	-0.00464	0.031686	0.142395
LINC0105(	-0.040584273	-0.06810752	-0.08779	0.006657	-0.06228	-0.02385
LINC0105'	-0.038049115	-0.056433689	0.053068	0.015659	-0.01917	0.009599
LINC0106!	-0.041248357	-0.027753684	-0.00178	0.018156	-0.02744	-0.05334
LINC0108:	-0.025968174	-0.048566445	-0.07542	-0.01016	0.030566	0.04932
LINC0108!	-0.03229721	-0.037144234	-0.06714	0.065793	-0.12835	-0.01867
LINC0108'	-0.079729629	-0.061522456	0.045283	-0.05151	0.130089	-0.04478
LINC0108{	-0.014102045	-0.005150087	-0.06383	-0.33651	0.292049	0.087848
LINC0108!	0.017174975	-0.193518481	0.190146	-0.09523	0.232273	-0.2411
LINC0109:	-0.045138105	-0.079290967	-0.01438	0.010148	-0.00369	-0.09187
LINC0109:	-0.034521647	-0.055395404	-0.12663	0.056847	-0.15442	-0.06779
LINC0109·	-0.011644146	-0.081274495	-0.24359	-0.06761	0.211978	0.562951
LINC0109!	-0.051693521	-0.067764465	-0.05436	0.003288	-0.00232	0.056993
LINC0109(	-0.043288036	-0.040846967	0.00923	-0.00969	0.052471	0.057319
LINC0109'	-0.054575513	-0.013619357	-0.13694	-0.00337	0.052109	0.192852
LINC0109{	-0.030415605	0.001694794	-0.05349	0.081161	-0.1341	0.01126
LINC0110:	-0.035775764	-0.09750709	0.110754	0.011814	-0.03487	6.25E-05
LINC0110:	-0.052220404	-0.076240285	-0.0538	-0.0029	0.021349	0.030968
LINC0110:	-0.046699427	-0.031135071	0.09788	0.019013	-0.02941	0.028188
LINC0110·	-0.035315664	-0.046372064	0.034582	0.015025	-0.0198	-0.10518
LINC0110!	-0.098138346	-0.017731411	0.079036	-0.0383	-0.1151	-0.06477

LINC0110	-0.034538024	-0.005074889	0.057944	-0.01104	0.034733	-0.10323
LINC0111	-0.022766687	-0.006286366	0.029302	-0.01377	0.014065	-0.10392
LINC0111	-0.04334948	-0.031146343	0.012857	0.031095	-0.06378	-0.02563
LINC0111	-0.043678948	-0.010729643	-0.12236	-0.0083	0.04792	-0.08184
LINC0111	-0.045635512	-0.132246645	-0.04615	0.000227	0.019929	0.161975
LINC0111	-0.034829902	-0.05305099	0.005921	-0.0229	0.059655	0.044255
LINC0111	-0.034132066	-0.074170798	0.025146	-0.01081	0.032902	0.019503
LINC0112	-0.0514114	-0.014660651	-0.0413	0.048914	-0.11782	-0.03935
LINC0112	-0.024483826	-0.057393751	0.005518	0.020264	-0.15046	0.000492
LINC0112	-0.054795007	-0.046442648	-0.02572	-0.01026	0.034298	-0.22465
LINC0112	-0.015125122	-0.061591805	-0.00502	-0.01645	0.037576	-0.0562
LINC0112	-0.023755105	-0.042705055	0.006586	-0.01806	0.06806	-0.03561
LINC0112	-0.010826942	-0.092563614	-0.2202	-0.07419	0.207121	0.531222
LINC0112	-0.043530091	0.067812905	0.20946	-0.01497	-0.00527	-0.14807
LINC0113	-0.067078772	-0.085233137	-0.13816	0.01675	-0.03404	-0.05719
LINC0113	-0.006048683	-0.068423279	-0.00419	-0.0232	-0.06365	-0.12282
LINC0113	-0.031481573	-0.030617286	0.045343	-0.00688	-0.0109	-0.01691
LINC0113	-0.008162653	0.104233493	0.270372	0.006877	-0.01792	-0.07423
LINC0113	-0.023111797	-0.067860487	########	-0.00383	0.00428	-0.14067
LINC0113	-0.057428509	-0.117560235	0.04923	0.002396	0.051883	0.076352
LINC0114	-0.04016643	-0.080278236	-0.08747	0.028209	-0.06771	-0.11026
LINC0114	-0.031939616	0.005465761	-0.07662	-0.00593	0.030763	0.027931
LINC0114	-0.027593422	-0.005324958	-0.01072	0.009154	-0.02309	0.001282
LINC0114	-0.028374517	-0.046867655	-0.03807	-0.0112	-0.02979	-0.04604
LINC0114	-0.064684988	0.11061888	-0.02749	-0.00206	-0.01775	-0.01467
LINC0114	-0.031217472	-0.079904934	0.004424	-0.01813	0.066209	-0.11106
LINC0115	-0.040114224	-0.067084354	0.123982	0.025972	-0.04774	-0.09008
LINC0115	-0.026136115	-0.039388506	0.094187	-0.01827	0.030687	-0.07666
LINC0115	-0.032360836	-0.052610503	-0.04324	-0.02786	0.074609	-0.00553
LINC0116	-0.040155063	-0.062930581	0.080386	0.013826	-0.02277	-0.03905
LINC0116	-0.037933506	0.005376761	-0.13696	0.008144	-0.00054	0.016889
LINC0118	-0.045073545	-0.057619191	0.003422	0.004582	0.021421	-0.10391
LINC0118	-0.03436799	-0.001745799	-0.08287	0.038887	-0.0951	-0.04724
LINC0118	-0.037855256	-0.047624609	-0.03307	0.017636	-0.02609	0.040755
LINC0118	-0.044312671	0.037601296	-0.07136	0.022611	-0.05227	-0.0739
LINC0119	-0.039436853	-0.023912276	-0.10076	0.0125	-0.02943	-0.04082
LINC0119	-0.027188913	-0.039425711	-0.02413	0.006742	-0.05574	-0.1377
LINC0119	-0.046485229	0.04062163	-0.09549	-0.0158	0.072024	-0.05033
LINC0120	-0.034908896	-0.014991212	-0.06374	0.003375	0.005185	0.027317
LINC0120	-0.048737026	-0.08571992	0.05439	0.048886	-0.14786	-0.10046
LINC0120	-0.035649378	-0.063994891	-0.14084	-0.02897	0.08428	0.024676
LINC0120	-0.016751554	7.45E-07	-0.03639	0.016643	-0.08561	-0.00605
LINC0120	-0.040086296	-0.06048384	-0.05604	0.032333	-0.07272	0.04805
LINC0121	-0.051317392	-0.050948923	0.007346	-0.06075	0.058193	-0.10491
LINC0121	-0.049304664	-0.046827341	-0.03319	-0.00547	0.015631	-0.08271
LINC0121	-0.043579867	0.004790277	-0.02623	0.021488	-0.03101	-0.03926
LINC0121	-0.035063377	-0.004743643	0.05829	0.006961	0.002484	0.143806
LINC0121	0.00195424	-0.043661875	0.261606	-0.01367	0.056963	-0.3397
LINC0122	-0.036864538	-0.044763889	-0.20118	0.006434	-0.00049	0.269984
LINC0122	-0.026889217	-0.053510379	-0.01129	0.011095	-0.03443	-0.05774
LINC0122	-0.025095708	-0.055741652	-0.03253	-0.00025	-0.04634	-0.10368

LINC0122(	-0.025203723	-0.049964865	-0.06088	0.009903	-0.03019	-0.12885
LINC0122'	-0.038912387	-0.004489707	-0.06462	0.07578	-0.20039	0.061346
LINC0123:	-0.024687912	-0.027734935	-0.07447	-0.00752	-0.0504	0.055752
LINC0123:	-0.047100676	0.002652576	0.072521	-0.03328	0.106707	-0.03899
LINC0123⁄	-0.034654442	-0.037653096	0.004775	0.167124	-0.20561	0.031922
LINC0124:	-0.036987058	-0.045303073	-0.0463	-0.0056	0.024465	-0.07437
LINC0124:	-0.068772941	-0.094776559	0.068042	-0.01702	0.078763	-0.19079
LINC0124:	-0.039229384	-0.046942532	-0.12595	0.044276	-0.11412	-0.02885
LINC0125:	-0.023067225	0.03170802	-0.00416	0.012895	-0.01931	-0.24395
LINC0125⁄	-0.049812346	-0.064405322	-0.14329	0.035797	-0.09789	0.059999
LINC0125!	-0.036649968	-0.067770445	-0.14627	-0.00329	0.019533	0.0376
LINC0126(	-0.020708616	-0.049447317	0.435841	0.025628	-0.10122	-0.21774
LINC0126'	-0.043146145	-0.04141249	-0.07855	0.006675	0.011891	-0.04037
LINC0126{	-0.023655187	-0.032772604	0.027813	0.076309	-0.19622	-0.02942
LINC0127(	-0.020840996	-0.047926376	-0.15455	-0.01384	0.042318	-0.07291
LINC0127:	-0.035173013	-0.030414438	-0.01764	0.006803	-0.00454	0.01588
LINC0127:	-0.01772484	-0.058141656	-0.24277	-0.06999	0.153226	0.249409
LINC0127{	-0.006533961	0.164208713	0.177176	-0.08632	0.263937	-0.09883
LINC0127!	-0.023170736	-0.045968512	0.048033	0.054154	-0.14246	0.012684
LINC0128:	-0.028830001	-0.019621752	0.058627	-0.0111	0.05205	-0.01005
LINC0128:	-0.038159853	-0.072437026	-0.08255	-0.01325	0.065778	0.157531
LINC0128!	-0.053413238	-0.042833699	0.046905	0.01229	-0.00744	-0.0882
LINC0129:	-0.035319733	-0.038267017	-0.05296	0.003782	0.001946	0.069404
LINC0130(	-0.062271836	-0.060346687	0.140359	0.014727	-0.01631	-0.06487
LINC0130⁄	-0.041944023	-0.066819664	-0.06244	0.026048	-0.06286	0.04646
LINC0130!	-0.043700424	0.052561682	0.016668	-0.00407	0.019553	-0.14371
LINC0131(	-0.034716665	-0.056680887	0.025592	-0.01093	-0.03203	-0.07457
LINC0131:	-0.031331253	-0.096498887	-0.07737	-0.00408	0.038304	0.092566
LINC0131⁄	0.002997518	-0.051288636	-0.0049	0.070261	-0.03524	0.086736
LINC0131!	-0.014086175	-0.026190834	-0.02666	0.009489	-0.01688	0.005992
LINC0133(	-0.099429211	-0.121764162	-0.00229	-0.02362	0.102472	0.020726
LINC0133:	-0.0266874	-0.073560165	0.015746	0.023613	-0.0568	-0.15317
LINC0133!	-0.025349881	-0.091344866	-0.07287	-0.01074	0.03563	0.082644
LINC0134:	-0.025315596	-0.098567292	0.004047	0.033081	-0.09781	-0.12135
LINC0134:	-0.031722297	-0.073801437	-0.07373	0.002569	-0.01421	-0.02687
LINC0134:	-0.036771081	-0.07315681	-0.04239	-0.02041	0.069644	0.062328
LINC0134(	-0.045346686	-0.100738663	0.011202	-0.00553	0.040988	-0.03363
LINC0134{	-0.013414343	-0.054245564	0.015717	0.008244	-0.08408	0.029008
LINC0135(	-0.033739699	-0.032687459	-0.09394	-0.00198	0.024669	-0.03618
LINC0135:	-0.03950718	-0.061936511	-0.11904	-0.00153	0.01788	0.008376
LINC0135:	-0.055276415	-0.100486422	0.07921	-0.01229	0.048471	0.211921
LINC0135⁄	-0.049614346	0.048487256	-0.03017	-0.03569	0.056042	-0.05173
LINC0135!	-0.019123441	-0.048565274	0.013416	0.023271	-0.0728	-0.17767
LINC0135(	-0.035106706	-0.093738868	-0.03349	-0.01376	0.017546	-0.00696
LINC0135!	-0.024883182	-0.012493855	-0.04988	-0.00867	-0.02286	-0.1556
LINC0136(	-0.06715608	-0.057331024	-0.04053	-0.01809	-0.04548	0.001583
LINC0136:	-0.065346511	-0.044202337	-0.03991	-0.01556	0.044752	-0.07758
LINC0136:	-0.042407438	-0.042306883	0.016363	-0.00392	0.031356	-0.03969
LINC0136⁄	-0.048583197	-0.054138282	0.067758	-0.00787	0.054868	0.155092
LINC0136!	-0.035032998	-0.018105646	0.121104	-0.00715	0.042503	-0.03332
LINC0136(	-0.047667186	-0.017609443	0.025078	0.021431	-0.0881	-0.02836

LINC0139:	-0.037204788	-0.063631185	0.003673	-0.0174	0.051756	-0.03045
LINC0139!	-0.046836496	-0.023347971	-0.04429	0.050275	-0.12549	0.119561
LINC0139(	-0.042361248	-0.070323853	-0.00698	0.018162	-0.02365	0.173808
LINC0140!	-0.037563737	-0.036833914	-0.14812	0.012491	-0.02214	0.063185
LINC0141(	-0.008885637	-0.195279182	-0.33136	-0.0733	0.172445	0.331793
LINC0141!	-0.075843614	-0.081800983	-0.06586	-0.02021	0.069712	0.06522
LINC0142(	0.012897332	-0.023748936	0.14944	-0.0096	0.043569	-0.04044
LINC0142	-0.033280357	-0.039417589	0.131132	0.066627	-0.17316	-0.05808
LINC0142!	-0.030901911	-0.031208246	-0.06768	0.022315	-0.06225	0.019177
LINC0142(	-0.037704615	-0.04442045	0.184636	-0.0174	0.058381	-0.16059
LINC0142(	-0.038487043	-0.015598638	0.039407	0.051521	-0.13185	-0.09478
LINC0143	-0.033622285	0.01307436	0.016873	0.000578	0.027665	-0.09174
LINC0143:	-0.041628846	-0.021927994	0.003963	-0.00296	0.029291	-0.06957
LINC0144:	-0.033003393	0.002639434	-0.00778	-0.00179	0.024118	0.059787
LINC0144(	-0.022429911	-0.074476904	0.020615	0.000658	-0.01336	-0.0329
LINC0144!	-0.040831019	-0.068228299	0.04903	0.041023	-0.09935	0.000476
LINGO1	-0.031509767	-0.113340931	0.095564	0.020066	-0.03927	0.051451
LINGO2	-0.009874611	0.418538177	-0.0716	-0.03686	0.122067	-0.15193
LINGO3	0.004265663	-0.043465077	0.064265	-0.02833	-0.05001	-0.04758
LINS	0.005891876	0.041307846	0.414085	-0.04023	0.096591	-0.23433
LIPA	0.030969799	0.021052881	-0.03847	-0.03691	0.09781	0.314806
LIPC	-0.017318621	0.081037276	0.106461	-0.09295	0.271241	-0.05104
LIPE	-0.025814551	-0.056689666	-0.00738	0.058779	-0.14195	-0.06996
LIPE-AS1	-0.036161734	-0.004558612	0.031739	0.00815	-0.00507	-0.05077
LIPF	-0.043220567	-0.030687787	-0.06233	0.001674	0.017012	0.005181
LIPG	-0.039748469	-0.076603834	-0.0692	-0.00595	0.015405	-0.0324
LIPH	-0.046592622	-0.09002644	-0.03737	-0.0019	0.032989	-0.01271
LIPI	-0.077917041	-0.085594651	0.040865	-0.00635	0.024456	0.011598
LIPJ	-0.0525175	-0.090522883	-0.00479	-0.03346	0.12996	0.068109
LIPT1	0.01544239	0.06270754	0.155922	-0.01188	0.075754	0.027774
LITAF	0.044594492	0.169023198	0.122586	-0.01826	0.045792	-0.40967
LIX1	-0.044700423	-0.062948753	-0.02484	-0.01255	0.00373	-0.05742
LIX1L	0.023752141	0.097346208	0.317056	0.034599	-0.06859	-0.25819
LKAAEAR1	-0.013376425	-0.08919472	-0.05095	0.01186	-0.08254	0.053874
LL0XNC01-	-0.023544568	0.027721836	0.020112	0.024397	-0.081	-0.03993
LL0XNC01-	-0.020434254	-0.100183233	0.205608	-0.01593	0.05271	-0.00951
LL22NC03-	-0.016646708	0.017740876	0.085746	-0.01185	-0.01505	-0.16611
LLGL1	-0.018757929	-0.047607582	0.068401	-0.01021	-0.08711	-0.08908
LLGL2	-0.003907958	0.252028036	0.150038	-0.05012	0.154225	-0.31481
LLNLR-24(	-0.026497757	-0.081521868	-0.16543	-0.00448	0.023325	-0.11411
LLPH	0.011048107	0.012557012	-0.09267	-0.03159	0.157617	0.215412
LMAN1	-0.031070853	0.081893366	0.148075	0.05583	-0.25151	-0.18817
LMAN1L	-0.032243519	-0.058230724	0.003178	-0.03974	0.030979	-0.03355
LMAN2	0.034594167	0.078636461	-0.15245	0.082335	-0.21696	0.05124
LMAN2L	0.020271913	-0.033522311	-0.00745	0.001408	-0.07015	-0.05548
LMBR1	-0.013639647	-0.014512187	0.002028	0.015319	-0.21848	0.043768
LMBR1L	0.046480866	-0.084700027	0.129907	-0.04633	0.09793	-0.16649
LMBRD1	0.033484265	-0.017727846	-0.25242	-0.01269	0.068439	-0.09084
LMBRD2	-0.017593003	0.012039676	0.022258	0.01957	-0.05353	0.032361
LMCD1	-0.091680247	0.033736652	-0.03396	0.045768	-0.0516	-0.00343
LMF1	0.035201007	-0.138466975	0.209297	-0.08593	0.128742	-0.13301

LMF2	0.060980596	-0.01734244	0.15319	-0.0054	-0.02339	-0.30274
LMLN	0.010024157	0.012139423	0.390525	-0.07611	0.103655	-0.0482
LMNA	0.025649419	-0.028503043	-0.03292	-0.01162	-0.04357	-0.01199
LMNB1	0.015061413	0.010475173	-0.17232	0.095025	-0.27744	0.064629
LMNB2	0.002343513	-0.009340502	-0.00103	0.101713	-0.32716	-0.04186
LMNTD1	-0.030902398	-0.002959914	-0.0992	-0.00531	-0.04324	-0.11823
LMNTD2	-0.023772248	-0.127502816	-0.10503	-0.01279	0.055038	0.075733
LMO1	-0.029595075	-0.054066773	-0.13929	0.002988	0.014141	0.050478
LMO2	0.005668138	-0.128131692	-0.39341	-0.12532	0.361119	0.430589
LMO3	-0.077516082	-0.074868095	-0.07444	-0.03605	0.042108	0.011118
LMO4	-0.0420783	0.140463651	-0.14518	0.062789	0.018619	0.063216
LMO7	-0.088541607	-0.129674807	0.219716	-0.01439	0.076829	-0.20693
LMO7DN	-0.038642357	-0.011890351	0.048487	0.007368	-0.0016	-0.08067
LMOD1	-0.047612212	-0.106902544	0.012589	0.021072	-0.08193	0.007397
LMOD2	-0.039612613	-0.049750272	0.018203	0.003738	0.0134	0.049784
LMOD3	-0.0167162	-0.087782785	-0.07644	-0.09821	0.142999	0.051123
LMTK2	-0.031985099	-0.013453111	-0.0883	0.004735	-0.00685	-0.0154
LMTK3	-0.031576056	-0.028417647	0.008173	-0.02071	0.089984	0.021885
LMX1A	-0.035896476	-0.043984247	-0.01379	3.34E-05	0.024841	-0.05731
LMX1B	-0.030407379	-0.051356422	-0.06299	0.029947	-0.09066	-0.02231
LNP1	-0.023420239	-0.033562737	0.094504	0.047496	-0.18192	-0.1264
LNPEP	0.000190408	0.15480896	-0.00515	-0.02961	0.05401	-0.0621
LNX1	-0.022784161	0.083428139	0.035041	0.01565	-0.02869	-0.08327
LNX2	0.017206965	0.062562308	0.033747	0.031898	-0.06895	-0.20212
LOC100049	0.008891664	0.020921724	-0.13464	-0.06997	0.207787	0.244204
LOC100126	-0.057692674	-0.005837832	-0.09479	-0.03924	0.041976	0.102769
LOC100127	-0.047613367	-0.041862372	-0.07272	-0.02698	0.086035	0.061116
LOC100127	-0.052661734	-0.012049055	-0.02709	0.001657	-0.00529	-0.01497
LOC100127	-0.001820343	0.051382933	0.269203	-0.0416	0.156266	0.01243
LOC100127	-0.056957977	0.002372842	0.032928	-0.00061	0.013023	0.074436
LOC100128	-0.055479181	-0.043341374	0.109233	0.026284	-0.04246	-0.0208
LOC100128	-0.004710466	0.031579655	0.248929	-0.04469	0.153019	-0.31272
LOC100128	-0.042112521	-0.056166116	-0.11382	0.008358	0.002507	-0.01736
LOC100128	-0.054500293	-0.037776329	0.107736	0.022203	-0.03466	-0.0885
LOC100128	-0.074943687	-0.05889028	-0.05987	-0.01947	0.02261	-0.016
LOC100128	-0.045701734	-0.005190853	0.078216	0.000303	0.021349	0.047727
LOC100128	-0.029545317	-0.026413973	-0.03908	0.011557	-0.04423	-0.02758
LOC100128	-0.035265361	-0.067487797	-0.03638	0.004283	0.018507	-0.0262
LOC100128	-0.062626641	0.057040015	-0.10304	0.030608	-0.06195	0.070946
LOC100128	-0.02189928	0.018876584	0.014997	-0.06024	0.176092	-0.02868
LOC100128	-0.02206401	-0.051505853	-0.12412	-0.00257	-0.02096	-0.04504
LOC100128	-0.000257104	-0.086669813	0.088073	-0.02214	-0.05781	-0.11683
LOC100128	-0.045428151	-0.03475055	-0.11448	0.005429	0.010513	0.011723
LOC100128	-0.031173453	-0.091701971	0.075163	0.002316	-0.01626	0.009037
LOC100128	-0.013022345	-0.021411606	-0.03933	0.004781	-0.09548	-0.09283
LOC100128	0.000699771	-0.039875729	-0.03644	-0.00691	-0.00232	-0.06656
LOC100128	-0.026292513	-0.026222941	0.006988	0.012474	-0.02997	-0.09957
LOC100128	-0.050807338	-0.045279703	0.036167	-0.01595	0.024863	-0.0593
LOC100128	-0.031004027	0.007157763	0.093029	0.042287	-0.10702	0.011727
LOC100128	-0.041441687	0.041242637	-0.01043	0.006222	0.002061	-0.07356
LOC100129	0.004346879	-0.04458986	-0.40542	-0.08224	0.198239	0.124555

LOC10012!	-0.040822524	-0.087862594	0.081952	-0.00684	0.057187	-0.11808
LOC10012!	-0.028537622	-0.067378442	0.082211	-0.00385	-0.01138	-0.03906
LOC10012!	-0.029218132	-0.018288886	-0.00205	-0.00741	0.049375	0.117192
LOC10012!	-0.026387822	-0.029853093	0.018892	-0.00115	-0.07214	-0.0724
LOC10012!	-0.032490412	-0.039436532	-0.11708	0.019533	-0.02904	-0.07909
LOC10012!	-0.019066272	-0.017882359	0.052683	-0.01815	0.03785	0.023639
LOC10012!	-0.018292592	0.082906628	0.190551	-0.0422	0.107191	-0.07064
LOC10012!	-0.005997444	-0.067119001	-0.0045	-0.01148	-0.06923	-0.12168
LOC10012!	-0.046196792	-0.040930257	-0.1027	-0.0053	0.011504	-0.10054
LOC10012!	-0.018356596	-0.020938425	-0.05324	-0.03392	0.101729	-0.11849
LOC10012!	-0.034624522	-0.016429872	0.076024	0.050585	-0.12648	-0.05327
LOC10012!	-0.049891608	-0.024074229	-0.11614	0.006768	-0.02741	0.064075
LOC10012!	-0.020976974	-0.059265111	-0.06031	0.028117	-0.06166	0.33482
LOC10012!	-0.033517332	-0.036630956	-0.11529	-0.01105	0.050751	0.079714
LOC10012!	-0.027510899	-0.068461427	0.047601	0.010268	-0.01756	0.065933
LOC10012!	0.016351281	-0.021451443	-0.20405	-0.04667	0.174516	0.375003
LOC10012!	-0.026224761	0.030128571	-0.00842	0.005179	-0.01352	-0.05173
LOC10012!	-0.098846002	-0.062164282	-0.0142	-0.01174	0.03983	0.098693
LOC10012!	-0.032542401	-0.108408736	0.160915	0.025183	-0.12124	-0.13924
LOC10012!	-0.020183675	-0.0007341	0.095285	-0.01422	0.044571	-0.09949
LOC10012!	-0.015454449	0.100926541	0.09605	0.012659	-0.01403	-0.07001
LOC10012!	-0.02730044	-0.039720319	-0.0175	0.104164	-0.28986	-0.1167
LOC10012!	-0.016580428	-0.095345637	0.192891	-0.03575	0.107792	-0.16302
LOC10013(	-0.026664085	-0.049608581	-0.00411	-0.01462	-0.05008	-0.05802
LOC10013(	-0.048575537	0.005098086	-0.0497	0.016266	-0.04666	-0.04148
LOC10013(	-0.031300211	-0.069605108	0.049582	-0.04471	0.15679	0.140949
LOC10013(	-0.029907487	-0.02816796	0.157918	0.021854	-0.04831	-0.14361
LOC10013(	-0.025268021	-0.059947632	-0.02699	0.040703	-0.10381	0.002461
LOC10013(	-0.029597011	-0.067128745	0.061951	-0.00415	0.031446	-0.00994
LOC10013(	-0.027755926	-0.078779465	0.004337	0.011064	-0.0261	0.296361
LOC10013(	-0.03162787	-0.044747047	-0.02998	-0.00655	0.03975	0.158651
LOC10013(	-0.021619705	-0.048160303	-0.05437	0.003027	0.004182	-0.13623
LOC10013(	-0.027614821	-0.09553503	-0.05421	0.000779	-0.06441	-0.07821
LOC10013(	-0.036105235	-0.053466351	-0.11076	-0.00256	-0.02348	-0.00466
LOC10013(	-0.028219937	0.043158052	0.073652	0.01388	-0.05234	-0.03769
LOC10013(	-0.01910529	-0.005018136	0.032833	-0.02766	0.087036	-0.10598
LOC10013(	-0.044374071	-0.113964791	-0.08909	0.003734	0.019676	0.050855
LOC10013(	-0.018555969	-0.03204042	0.045524	-0.00253	-0.07451	-0.03547
LOC10013(	-0.015675383	-0.065723356	-0.09957	-0.04248	0.133248	-0.11119
LOC10013(	-0.009188292	-0.022153751	-0.02108	-0.01109	0.048242	-0.15225
LOC10013(	-0.00707138	-0.060767884	-0.0189	-0.01352	-0.05654	0.095122
LOC10013(	-0.041510755	0.01070675	-0.00276	-0.00851	0.053344	0.060064
LOC10013(	-0.028702529	0.045943231	-0.04051	0.000187	0.002977	-0.13729
LOC10013(	-0.042443122	-0.091743518	0.08035	0.022498	-0.03387	-0.0171
LOC10013(	-0.027881109	-0.003163766	0.131961	-0.00691	0.046528	0.151028
LOC10013(	-0.041157736	-0.060546239	0.046526	-0.01197	0.021988	0.018181
LOC10013(	-0.022541906	0.027059399	0.026989	0.060765	-0.16161	0.064924
LOC10013(	-0.040560123	-0.03713833	-0.05932	0.02396	-0.04499	0.077236
LOC10013(	-0.00683773	0.02313564	0.113636	-0.01627	0.070549	-0.12264
LOC10013(	-0.038204862	-0.031406906	-0.10904	0.028147	-0.05625	-0.0996
LOC10013(	-0.005092448	0.256644866	0.172494	-0.01008	0.038701	-0.23562

LOC10013(	-0.016305933		-0.052860099	-0.05027	-0.24035	0.679845	0.101533
LOC10013(	-0.042302565		-0.005419675	-0.14556	-0.02406	0.059857	0.03768
LOC10013(	-0.056941418		-0.063510073	-0.03417	-0.02037	0.042754	0.07254
LOC10013(	-0.017858091		-0.100428378	0.187144	0.005711	0.001264	-0.22328
LOC10013(	-0.035781569		0.000321157	-0.07564	-0.01295	0.021624	-0.04129
LOC10013:	-0.023138766		-0.040894216	-0.00183	-0.07086	0.159491	-0.14138
LOC10013:	-0.031792942		-0.032731964	-0.17484	0.006801	-0.00733	0.051847
LOC10013:	-0.027833608		-0.042614077	-0.17338	4.65E-05	0.015331	0.096385
LOC10013:	-0.04069215		-0.043569692	0.057066	0.003046	0.012213	0.075244
LOC10013:	-0.015341329		0.018888401	-0.08968	0.065908	-0.19801	0.191672
LOC10013:	-0.02844499		-0.088261385	0.008339	-0.008	-0.03348	-0.11993
LOC10013:	-0.027052951		-0.043765656	0.144057	0.031431	-0.08688	-0.08872
LOC10013:	-0.047772858		-0.054554745	-0.02041	-0.02264	0.089854	0.135668
LOC10013:	-0.073912588		-0.076664666	-0.03676	-0.03805	0.087974	0.103182
LOC10013:	-0.02714178		-0.086091681	0.24691	-0.00952	0.029288	-0.10064
LOC10013:	-0.034385414		-0.067220326	-0.01797	0.02019	-0.04422	0.06404
LOC10013:	0.001373975		-0.069544338	0.198739	-0.01221	0.011051	-0.12187
LOC10013:	0.026736628		-0.097739261	0.252699	-0.01927	0.050529	-0.31923
LOC10013:	-0.024883927		-0.04181638	0.137224	0.000735	-0.05193	-0.07101
LOC10013:	-0.038100541		-0.071370424	0.008175	0.021391	-0.03834	-0.02883
LOC10013:	-0.019679586		-0.123963022	0.418802	-0.06844	0.218219	-0.0786
LOC10013:	-0.043313972		-0.024478383	0.060163	0.013152	-0.02874	-0.0118
LOC10013:	-0.044437884		-0.073357713	0.006848	-0.01206	0.060585	-0.01387
LOC10013:	-0.039922955		-0.011269443	0.026155	0.021014	-0.0282	-0.06954
LOC10013:	-0.0385652		0.012483247	-0.0225	0.045043	-0.10723	-0.08176
LOC10013:	-0.029231684		-0.012288001	-0.03883	-0.02051	0.014645	-0.05723
LOC10013:	-0.025309842		-0.06982587	-0.06264	0.003623	-0.03401	-0.05545
LOC10013:	-0.018492932		-0.013202721	0.274074	-0.02472	0.075176	-0.21933
LOC10013:	-0.034607257		-0.051253688	0.070859	-0.00831	0.042081	0.158414
LOC10013:	-0.042188606		-0.016679271	0.091409	0.017559	-0.02122	0.080031
LOC10013:	-0.021295556		-0.059661324	0.162152	-0.01473	-0.01799	-0.1537
LOC10013:	-0.042493803		-0.024346417	-0.01811	0.006434	0.0066	0.11334
LOC10013:	-0.040690142		-0.090423153	0.084409	0.01118	-0.01341	0.049987
LOC10013:	0.013679324		-0.005457393	0.012991	-0.00761	0.048068	-0.05374
LOC10013:	-0.028688632		-0.0333192	0.034356	0.001994	-0.0148	-0.15049
LOC10013:	-0.019459121		-0.081041443	0.050042	-0.00954	0.031478	-0.082
LOC10013:	-0.038590686		-0.04084754	0.002768	-0.00668	0.044117	0.049938
LOC10013:	-0.018156933		0.049146455	-0.04534	-0.00234	0.028755	-0.1752
LOC10013:	-0.03658209		-0.035902929	-0.06814	-0.00103	0.021773	0.334309
LOC10013:	-0.016007762		-0.033108063	0.080276	0.092984	-0.26455	-0.06399
LOC10013:	-0.02645733		0.023730192	0.040343	0.010836	-0.02005	-0.09287
LOC10013:	0.027628522		-0.039639368	0.039194	0.00432	-0.07249	-0.19938
LOC10013:	-0.002104474		-0.007526732	0.172631	0.026777	-0.09986	-0.03969
LOC10013:	-0.034535202		0.007554746	0.062394	-0.00084	0.010281	-0.05623
LOC10013:	-0.004779134		-0.017521103	0.212918	0.019196	-0.05273	-0.13807
LOC10013:	-0.021907501		-0.086618997	-0.10724	-0.02768	-0.02177	-0.04027
LOC10013:	-0.027095919		-0.094594535	0.080649	0.017883	-0.08379	-0.01873
LOC10013:	-0.050386643		-0.069265033	0.044819	-0.00248	0.031928	0.02842
LOC10013:	-0.037487609		-0.014968145	-0.06198	0.016157	-0.02114	0.025535
LOC10013	-0.030699923		-0.069779613	0.05861	-0.00783	-0.00201	-0.13652
LOC10013	-0.022742769		-0.03620931	0.036854	-0.00566	0.000145	-0.11195

LOC10013	0.030868803	0.072673192	0.149141	-0.04215	0.110109	-0.05439
LOC10013	-0.005427012	-0.046030587	0.046275	-0.05491	0.104178	0.093709
LOC10013	-0.002988556	0.005973628	0.047939	-0.09114	0.278374	-0.11278
LOC10014	-0.026284524	-0.003665216	0.135242	0.029242	-0.05448	-0.03752
LOC10019	0.011273177	0.029395797	0.119307	0.017136	-0.04398	-0.1175
LOC10024	-0.027343677	-0.011166503	-0.07092	0.000879	-0.00164	-0.07527
LOC10024	-0.027420516	-0.039298525	0.109818	0.003796	-0.00623	-0.03878
LOC10026	-0.050416954	-0.033835237	-0.0244	0.008586	-0.03734	0.021339
LOC10027	-0.001597879	0.038175137	0.260293	-0.02543	0.090115	-0.24503
LOC10027	-0.033912413	-0.0427546	-0.06699	-0.01455	0.054055	-0.01492
LOC10027	-0.016065677	0.01132585	0.074725	0.02808	-0.06586	-0.02879
LOC10027	0.01730831	0.009546426	0.178831	0.026377	-0.06991	-0.18975
LOC10027	-0.075794018	-0.064725109	0.038539	0.003353	0.009009	-0.00767
LOC10028	-0.043516267	-0.073851997	-0.00227	0.010961	0.001474	-0.04135
LOC10028	-0.016437834	-0.002343249	-0.20762	-0.01404	0.064857	0.46029
LOC10028	-0.042987992	-0.021855327	0.111398	0.072456	-0.19524	-0.09928
LOC10028	-0.02021329	-0.09994105	-0.09128	-0.00131	-0.04649	0.046655
LOC10028	-0.03327328	-0.048861211	0.098641	0.023877	-0.03846	0.038227
LOC10028	-0.031104053	-0.089019542	-0.01477	0.000259	########	-0.1049
LOC10028	-0.066116947	-0.089173342	-0.03913	0.003139	0.020217	-0.00247
LOC10028	-0.055620742	0.028914594	0.073981	-0.02373	0.036793	-0.0581
LOC10028	-0.015656534	-0.066447043	0.024058	0.028694	-0.08537	-0.07877
LOC10028	-0.020695742	-0.070459589	-0.1212	0.001183	-0.03399	0.099521
LOC10028	-0.015153524	0.02097685	0.022435	-0.00182	0.027608	0.022025
LOC10028	-0.030790277	-0.092261525	-0.17297	-0.01939	0.031589	-0.04947
LOC10028	-0.035789199	-0.034837009	-0.06505	0.015046	-0.00166	0.12248
LOC10028	-0.035140894	-0.038992022	-0.09346	0.006459	0.002465	0.013017
LOC10028	-0.011230597	0.108644771	0.156892	-0.09566	0.282694	-0.07186
LOC10028	-0.031557002	-0.077674219	-0.13456	0.003523	0.023196	0.047121
LOC10028	-0.005980883	-0.006823098	0.229361	0.068573	-0.17654	-0.05426
LOC10028	-0.026893228	-0.067801667	-0.12848	-0.01661	-0.00131	0.027182
LOC10028	-0.045748807	-0.06075237	-0.0655	0.03795	-0.08163	-0.09465
LOC10028	-0.08090599	-0.115829355	-0.0023	-0.02445	0.060036	-0.07196
LOC10028	-0.027708018	-0.088524629	-0.04053	-0.01396	0.026025	-0.01357
LOC10028	-0.047640379	-0.041376505	0.096393	-0.00208	0.039851	-0.10035
LOC10028	-0.031774759	0.021560111	0.054506	0.023834	-0.04846	0.123161
LOC10028	0.010099755	0.077581133	-0.09011	0.006078	0.024088	0.144446
LOC10028	-0.05835783	0.008880815	-0.03197	-0.01987	0.0571	-0.06784
LOC10028	-0.029865603	0.022037463	-0.03059	-0.00626	0.00054	-0.11675
LOC10028	-0.030661424	-0.019408595	-0.05516	0.013714	-0.01784	0.021476
LOC10028	-0.017413706	-0.059598866	-0.07023	-0.01139	0.050806	-0.01461
LOC10028	-0.024685733	0.001338331	-0.03932	0.022443	-0.06364	-0.05714
LOC10028	-0.110147661	-0.063619896	-0.08561	0.002489	0.008985	0.123256
LOC10028	0.015421895	-0.088590857	0.178209	-0.01255	0.041805	-0.17886
LOC10028	-0.036898587	-0.098864726	0.057482	-0.00936	0.036881	-0.12063
LOC10028	-0.014152203	-0.088009496	0.18452	0.004419	0.005852	-0.13493
LOC10028	-0.019836804	-0.043365511	-0.15342	-0.01573	0.001686	-0.05308
LOC10028	-0.014900656	0.034263985	0.019166	0.131568	-0.37793	-0.02865
LOC10028	-0.03526076	0.024707222	0.019342	0.028464	-0.06833	-0.00454
LOC10028	-0.001969776	-0.048620931	0.279103	0.045339	-0.09588	-0.02844
LOC10028	-0.011252432	-0.000151249	0.057549	-0.0219	0.083289	-0.07448

LOC10028!	0.006071823	-0.114314157	-0.00826	-0.01921	-0.03974	-0.11823
LOC10028!	0.032931672	-0.000214382	0.086902	-0.02913	0.032137	-0.27925
LOC10028!	-0.021963295	-0.054491139	0.03945	0.04845	-0.10762	0.014249
LOC10028!	-0.026859966	-0.046241743	0.128836	-0.03656	0.125914	0.233633
LOC10028!	-0.029141827	-0.047974068	-0.07476	0.007427	-0.01696	0.148367
LOC10029	-0.017167563	0.025335973	0.130368	0.04546	-0.10863	-0.14128
LOC10029	-0.031609462	-0.058994987	0.029937	-0.02706	0.102679	0.351305
LOC10029	-0.032901193	-0.08899715	-0.04495	-0.11673	0.351327	-0.06405
LOC10029	-0.029376226	-0.006702401	-0.01078	-0.00467	0.020241	-0.18956
LOC10029	-0.055842115	-0.001165887	-0.01662	-0.00191	0.020504	0.080581
LOC10031(	0.013594749	-0.069609936	-0.05985	0.004901	-0.02394	0.192708
LOC10037!	-0.056827408	-0.008558503	0.028877	-0.00572	-0.00112	-0.03671
LOC10042	-0.038782204	0.06028127	-0.00477	-0.00307	0.022759	-0.06689
LOC10042	-0.048012261	-0.097533553	-0.03175	0.007918	0.004091	-0.07068
LOC10042	-0.03043607	-0.053732719	-0.01445	-0.00205	-0.07014	-0.10535
LOC10049!	-0.028597425	-0.023907067	-0.07311	0.041285	-0.13305	-0.00296
LOC10049!	-0.02143897	0.053657317	0.054465	-0.01513	0.041193	-0.00693
LOC10050!	-0.019081154	-0.014405413	-0.0303	-0.00983	-0.00128	0.003137
LOC10050!	-0.007651479	-0.071276725	-0.04096	0.021326	-0.14814	-0.05
LOC10050!	-0.053694727	-0.053939395	0.067138	0.002674	-0.0295	-0.11619
LOC10050!	0.000374482	0.264724943	0.155989	0.003648	0.005149	-0.20538
LOC10050!	-0.02957477	0.000415125	0.003847	-0.00749	-0.01485	-0.05796
LOC10050!	-0.054533125	-0.071604241	-0.03605	0.016531	-0.02798	0.025145
LOC10050!	-0.03510838	-0.036828104	-0.00938	-0.00194	0.030279	-0.07716
LOC10050!	0.005851586	-0.022189748	-0.01799	-0.04747	0.138815	0.018825
LOC10050!	-0.006923507	-0.148701794	-0.20884	-0.04031	0.132926	0.154171
LOC10050!	-0.035382627	-0.084877374	-0.11444	0.008277	-0.0284	-0.00861
LOC10050!	-0.037563017	-0.089947279	0.056492	-0.04229	0.144886	-0.05618
LOC10050!	-0.030847994	-0.046989133	0.020537	0.065788	-0.19106	-0.13577
LOC10050!	-0.010865936	-0.029856472	-0.15864	-0.02737	0.077386	0.2784
LOC10050!	-0.037686795	-0.025689103	0.051798	-0.00827	0.053669	-0.11235
LOC10050!	-0.040651281	-0.015817421	-0.0374	0.002618	0.015405	-0.09661
LOC10050!	-0.027805191	0.00027786	0.035616	0.007404	-0.00269	-0.02284
LOC10050!	-0.031847928	-0.086323426	-0.06892	0.026945	-0.05364	0.065407
LOC10050!	-0.024358023	-0.056302246	-0.02375	-0.01491	0.053047	-0.22573
LOC10050!	-0.029482206	-0.034370001	0.009595	-0.02745	0.013218	-0.08474
LOC10050!	-0.015942748	-0.041839715	0.09547	0.011401	-0.12477	-0.01914
LOC10050!	0.004875372	0.076184375	0.044013	-0.07622	0.181764	-0.22216
LOC10050!	-0.032545077	-0.039466366	0.008912	0.015707	-0.03432	-0.08791
LOC10050!	-0.045101723	-0.078452812	-0.00771	0.005435	-0.01084	-0.19166
LOC10050!	-0.034251666	-0.029327504	0.102393	-0.01375	0.030623	-0.12199
LOC10050!	-0.029080349	-0.043420392	-0.11742	0.007529	-0.06017	0.023996
LOC10050!	-0.04514708	-0.07350388	0.003378	0.023086	0.019446	-0.22676
LOC10050!	-0.053655717	0.012409066	0.054572	-0.0057	0.010218	0.000481
LOC10050!	-0.051636091	-0.023721516	-0.05662	-0.016	0.043638	-0.0067
LOC10050!	-0.047079978	-0.025092427	-0.16582	-0.01033	0.045752	-0.0815
LOC10050!	-0.063666016	-0.048697891	-0.02902	-0.00101	0.003823	-0.04569
LOC10050!	0.024940236	0.06020478	-0.31868	-0.04246	0.128287	0.251304
LOC10050!	-0.021926576	-0.056609261	0.079089	-0.00508	-0.0097	-0.02896
LOC10050!	-0.037098467	-0.061792111	-0.03841	0.02496	-0.05181	-0.05103
LOC10050!	-0.043364173	0.016579248	-0.02621	-0.00269	0.030049	0.029761

LOC10050!	-0.031864247	0.001376116	0.022992	0.043718	-0.1138	-0.03424
LOC10050!	-0.047772905	-0.045111787	0.080036	-0.01934	0.073634	-0.09349
LOC10050!	-0.036909539	-0.064029502	0.046875	0.007073	-0.03465	-0.07955
LOC10050!	-0.025703431	-0.034749757	0.30392	-0.09502	0.292794	-0.26181
LOC10050!	-0.088191467	-0.013591562	-0.00524	-0.01691	0.046986	-0.01751
LOC10050!	-0.027793729	0.011289326	-0.00277	0.029281	-0.07453	-0.07439
LOC10050!	-0.04285565	-0.002324077	0.033718	-0.01551	0.067934	-0.02933
LOC10050!	0.000208284	-0.063102725	0.097872	-0.01001	0.011235	-0.22197
LOC10050!	-0.019847711	-0.026163297	-0.07835	0.029444	-0.12369	-0.02046
LOC10050!	-0.042274261	-0.026905511	-0.18822	0.013422	-0.00755	0.015733
LOC10050!	-0.041043217	-0.129208129	-0.0197	0.008143	0.001947	0.072108
LOC10050!	-0.040231852	-0.161242693	-0.12318	-0.00036	0.014853	0.100213
LOC10050!	-0.04238976	-0.022469906	-0.06534	0.02692	-0.04536	-0.03432
LOC10050!	-0.055330733	-0.042834047	-0.06777	########	0.046329	-0.01329
LOC10050(	-0.011711587	-0.003427547	0.030677	0.000103	0.039745	0.064448
LOC10050(	-0.06501169	-0.097718496	-0.07876	-0.01869	0.055564	0.003962
LOC10050(	-0.033376146	0.026072286	-0.15503	0.012833	-0.00613	0.031785
LOC10050(	-0.015931338	0.046114856	0.189517	0.029167	-0.07964	-0.11244
LOC10050(	-0.050030562	-0.049713481	0.053004	-0.0178	0.04817	0.115966
LOC10050(	-0.045715352	-0.052731784	-0.11294	-0.02749	0.078233	0.038298
LOC10050(	-0.039682599	-0.008075298	0.086624	0.041237	-0.12172	-0.02744
LOC10050(	-0.011822097	-0.008709428	-0.01601	0.049114	-0.15573	-0.14098
LOC10050(	0.024034596	-0.023798243	0.256974	0.051053	-0.16206	0.060882
LOC10050(	-0.044754083	0.006790987	0.045037	0.044555	-0.09735	-0.05257
LOC10050(	-0.028052783	-0.084608542	-0.13166	0.003551	0.005598	0.009143
LOC10050(	-0.026676583	-0.100338942	-0.08424	-0.06432	0.196259	0.223824
LOC10050(	-0.000192868	-0.08481384	-0.28606	-0.04404	0.134844	0.789531
LOC10050(	-0.02597683	-0.038878452	0.026329	0.058177	-0.13882	0.004743
LOC10050(	-0.040575517	0.006645015	-0.07302	0.018793	-0.01837	0.03544
LOC10050(	-0.093462647	0.006323294	0.079349	-0.00197	0.006542	-0.0282
LOC10050(	-0.042138567	-0.037453057	-0.07737	0.006907	-0.02674	0.021021
LOC10050(	-0.074226633	-0.066733663	0.057782	-0.03318	0.090069	-0.0513
LOC10050(	-0.022435201	-0.081924423	0.077032	-0.01148	-0.07232	-0.16078
LOC10050(	-0.051391815	-0.093834119	0.03225	-0.00435	0.027319	-0.04017
LOC10050(	-0.037556134	-0.027240599	0.003539	0.020539	-0.03916	0.007823
LOC10050(	-0.048732304	-0.022893671	-0.02749	0.017536	-0.03985	0.030929
LOC10050(	-0.046739627	-0.092918171	-0.07469	0.002241	0.006778	0.065378
LOC10050(	-0.037215164	-0.081584703	-0.03262	0.012311	-0.00481	-0.03554
LOC10050(	-0.034945876	-0.042041541	-0.10614	0.000873	0.006482	0.066657
LOC10050(	-0.044077073	-0.023110729	0.182653	0.015001	-0.05502	0.025451
LOC10050(	-0.030407895	0.058546906	0.138207	0.0258	-0.03609	-0.05716
LOC10050(	-0.039887377	-0.08402741	-0.06212	-0.01029	0.049794	0.258179
LOC10050(	-0.042651767	-0.025196512	0.028347	0.017805	-0.02268	0.03587
LOC10050(	-0.015454915	-0.05532412	-0.14188	-0.0756	0.207845	0.644445
LOC10050(	-0.007659942	0.054357354	0.105877	0.024634	-0.04598	-0.17796
LOC10050(	-0.03764006	0.04119465	-0.00562	-0.0029	0.032448	-0.04749
LOC10050(	-0.025849094	-0.015106393	0.067066	0.034558	-0.09403	0.038429
LOC10050(	-0.021717732	-0.07725258	-0.03937	0.068479	-0.17328	0.137071
LOC10050(	-0.031860353	-0.049464507	0.041821	0.011733	-0.01995	-0.13682
LOC10050(	-0.028841572	-0.024949386	-0.07192	-0.0189	0.01948	-0.03575
LOC10050(	-0.036117232	-0.063217924	-0.08326	0.006362	0.005837	0.019846

LOC10050(	-0.023380874	0.066009292	0.141947	0.018526	-0.08136	-0.13135
LOC10050(	-0.020862082	-0.053952083	0.05572	0.068252	-0.09811	-0.1295
LOC10050(	-0.016830334	-0.086093087	-0.03253	-0.01699	0.065598	-0.03842
LOC10050(	-0.035632554	-0.04417165	-0.00415	0.046999	-0.11123	-0.05287
LOC10050(	-0.024236924	-0.035149412	-0.12046	0.0157	-0.08314	-0.04957
LOC10050(	-0.067472737	-0.05822998	0.002661	0.016654	-0.04031	-0.15503
LOC10050(	-0.031016999	-0.021111739	-0.04543	0.023802	-0.03255	0.078428
LOC10050(	-0.047278453	-0.091788856	-0.04825	0.003826	0.014314	0.042167
LOC10050(	-0.005856295	0.051018612	-0.09956	0.011513	-0.02434	0.072823
LOC10050(	-0.008064349	-0.058151848	0.179243	0.025615	-0.07268	-0.08488
LOC10050(	-0.023395661	-0.073019739	-0.1172	0.016649	-0.08097	0.095114
LOC10050(	-0.030407831	-0.053617592	0.000969	-0.00772	-0.0078	0.05152
LOC10050(	-0.01752824	-0.013226969	0.151987	0.023389	-0.04936	-0.16702
LOC10050(	-0.055857385	-0.092206658	0.103711	-0.02871	0.078289	-0.1872
LOC10050(	-0.034140803	-0.033930899	0.148673	0.011909	-0.03434	-0.0163
LOC10050(	-0.035937533	-0.060171063	0.037136	0.09596	-0.20832	-0.01888
LOC10050(	-0.026492087	-0.098828287	0.077725	-0.01457	9.52E-05	-0.00741
LOC10050(	-0.025503111	-0.091710715	-0.00602	0.008333	-0.0263	-0.11973
LOC10050(	-0.055921166	-0.003010589	-0.05135	-0.00922	0.043236	-0.06095
LOC10050(	-0.050847149	-0.000948193	-0.15839	-0.00121	-0.02186	-0.11462
LOC10050(	-0.041229819	-0.059083326	-0.05065	0.021464	-0.02777	-0.04423
LOC10050(	-0.018493352	-0.013099597	-0.05963	0.007826	-0.01165	-0.00977
LOC10050(	-0.015928371	-0.092460987	0.076156	-0.00095	-0.07897	-0.07318
LOC10050(	-0.019507134	-0.061023089	-0.16539	-0.05566	0.12814	0.304883
LOC10050(	-0.008041069	-0.024870024	0.099362	0.002558	0.033691	0.153266
LOC10050(	-0.018446942	-0.025252795	0.074641	-0.02118	0.048235	-0.30429
LOC10050(	-0.006402493	0.08692946	0.080356	-0.02925	0.110849	-0.10553
LOC10050(	-0.043559671	-0.084773655	0.053625	0.014605	-0.01954	0.020882
LOC10050(	-0.035825002	0.009094973	-0.10962	0.031679	-0.07767	0.005007
LOC10050(	-0.049025474	-0.085518968	-0.09178	-0.02497	0.058718	0.037057
LOC10050(	-0.035045608	-0.081736557	-0.05625	0.020182	-0.07519	-0.1044
LOC10050(	-0.029442389	-0.041765227	-0.02727	0.015744	-0.02396	0.020289
LOC10050(	-0.042717421	-0.00676698	-0.00213	0.00423	0.014013	-0.04474
LOC10050(	-0.009996325	-0.085646888	-0.11319	-0.04046	0.098347	0.107426
LOC10050(	-0.03358306	0.010112278	-0.05071	0.010214	-0.01017	-0.00168
LOC10050(	-0.023924225	-0.011450238	0.014734	0.065411	-0.19031	0.040856
LOC10050(	-0.067825991	-0.064610334	0.056915	-0.02356	0.04659	0.079267
LOC10050(	-0.025150912	-0.045780282	-0.05735	0.003984	-0.00267	0.061668
LOC10050(	-0.030844957	-0.011115114	-0.01231	-0.00153	-0.01041	-0.03382
LOC10050(	-0.019285294	0.022996671	0.067371	-0.01424	-0.05397	-0.13003
LOC10050(	-0.035342863	-0.099317225	0.05006	-0.01168	-0.03582	-0.08301
LOC10050(	-0.031403995	-0.056047295	0.006012	0.017203	-0.04101	-0.13231
LOC10050(	-0.035281906	-0.07491914	-0.00933	-0.01948	0.038851	-0.06432
LOC10050(	-0.014567124	0.037784843	0.121068	0.073125	-0.20028	-0.25485
LOC10050(	-0.03928238	-0.064809205	-0.05182	-0.00141	0.023537	-0.06682
LOC10050(	-0.027386722	-0.010735275	0.031109	-0.02203	-0.00023	-0.12092
LOC10050(	-0.048317367	-0.105816854	-0.06525	-0.0114	0.011534	-0.01621
LOC10050(	-0.036887677	-0.048005288	0.02221	-0.02299	0.089805	0.004854
LOC10050(	-0.0367647	-0.07354229	0.066692	0.004667	0.012353	-0.05163
LOC10050(	-0.001955513	-0.115691863	-0.17062	0.008531	-0.00079	-0.00134
LOC10050(	-0.033258303	-0.02814428	-0.16667	0.011636	-0.01917	-0.0996

LOC10050(	0.001708801	-0.106696178	0.420744	-0.02603	0.041716	-0.26961
LOC10050(	-0.040862518	-0.067108138	0.008122	-0.02839	0.037719	-0.02757
LOC10050'	-0.009291475	-0.172381147	-0.29033	-0.02787	0.040035	0.284071
LOC10050'	-0.035430236	-0.077086645	-0.07792	0.009499	-0.00707	-0.07542
LOC10050'	-0.049543382	0.015169065	0.079229	0.043913	-0.1065	-0.22999
LOC10050'	-0.006715476	-0.106666631	-0.04905	-0.08304	0.238659	0.324728
LOC10050'	-0.040021335	-0.024729173	0.023604	-0.02608	0.099562	-0.08768
LOC10050'	-0.033144724	0.023050246	0.098846	-0.0057	0.00596	-0.10758
LOC10050'	-0.02767294	-0.005083319	0.016549	0.011632	-0.01772	0.016537
LOC10050'	-0.028273511	-0.035467376	-0.00892	-0.0044	-0.04235	-0.06011
LOC10050'	-0.040546211	-0.013557423	0.068779	0.030596	-0.06055	-0.10188
LOC10050'	-0.034223178	-0.004108498	-0.06788	0.01731	-0.03642	-0.04668
LOC10050'	-0.081468512	-0.096496052	-0.04408	-0.026	0.044754	0.141535
LOC10050'	0.001197457	-0.042895921	0.074637	-0.08475	0.272866	-0.03961
LOC10050'	-0.075803964	-0.050510436	0.137168	-0.00994	0.046909	-0.12934
LOC10050'	-0.020877956	-0.084733388	0.331238	0.009094	-0.00143	-0.2982
LOC10050'	-0.027467909	-0.109060417	-0.01214	0.013339	-0.02954	0.013283
LOC10050'	-0.061298514	-0.102385676	0.091465	-0.01556	0.06712	-0.06963
LOC10050'	-0.019146748	-0.015170283	0.049826	0.019975	-0.14506	-0.00942
LOC10050'	-0.042698424	-0.028998316	0.10508	0.006297	0.016363	-0.10292
LOC10050'	-0.10228761	-0.140269407	0.148412	0.064136	-0.08667	-0.01885
LOC10050'	-0.035961769	-0.032255016	-0.02395	-0.04255	0.127783	-0.04097
LOC10050'	-0.035068054	-0.030838843	-0.07318	-0.00064	0.013863	-0.04606
LOC10050'	-0.016368699	-0.015588357	0.008708	0.078415	-0.1917	0.014626
LOC10050'	-0.048927256	-0.066802914	-0.12555	0.006727	0.009716	-0.04375
LOC10050'	-0.025760452	-0.011222091	-0.06591	0.01011	-0.0432	-0.06887
LOC10050'	-0.05689984	-0.048350157	0.157497	0.02522	-0.05576	-0.08147
LOC10050'	-0.039032006	-0.098448222	-0.03435	0.004799	-0.0424	-0.06798
LOC10050'	-0.025522852	-0.039921522	0.019364	0.008884	-0.06591	-0.03517
LOC10050'	-0.028208007	-0.060221268	0.01176	0.007681	-0.07624	0.011513
LOC10050'	-0.027548613	0.084622748	0.001365	0.012551	-0.01552	-0.11097
LOC10050'	-0.105703243	-0.043879269	0.099762	-0.03501	0.202295	-0.18245
LOC10050'	-0.048081388	0.005843404	0.015924	0.022005	-0.0403	-0.00314
LOC10050'	-0.028564654	-0.033833636	-0.04324	0.033153	-0.08181	0.068441
LOC10050'	-0.021939271	-0.051860196	0.041094	-0.00239	-0.01347	-0.06318
LOC10050'	-0.031430825	-0.028444564	0.001762	-0.01731	0.00669	-0.10133
LOC10050'	-0.048327905	-0.053824442	7.59E-05	-0.01764	0.071091	-0.00977
LOC10050'	-0.053953769	-0.062401697	-0.00088	0.002482	-0.02284	0.166296
LOC10050'	-0.032336337	-0.00540675	-0.04303	0.030974	-0.09273	-0.09801
LOC10050'	-0.037439296	-0.095421638	-0.05014	-0.00052	-0.02339	0.024262
LOC10050'	-0.014268206	-0.058181453	0.206499	0.009884	-0.09705	-0.12879
LOC10050'	-0.031447782	-0.071144497	0.007591	0.000611	0.014079	-0.14002
LOC10050'	-0.025344457	-0.051445356	0.087087	-0.02499	0.070253	0.271282
LOC10050'	-0.030008571	-0.036731966	0.117751	0.02965	-0.06861	-0.05511
LOC10050'	-0.049674369	-0.024175666	-0.00659	0.025143	-0.07016	-0.0602
LOC10050'	-0.027233925	-0.054009135	-0.18723	-0.06462	0.138448	0.316665
LOC10050'	-0.042770421	-0.037243907	-0.03667	-0.0064	0.034418	-0.01522
LOC10050'	-0.043147618	-0.071921885	-0.00951	0.016297	-0.05352	-0.07009
LOC10050'	0.032133418	0.045990547	-0.01286	-0.01836	0.029836	0.110431
LOC10050'	-0.078437747	-0.046015531	-0.04464	-0.04341	0.079982	0.023357
LOC10050'	-0.012510577	0.00692452	-0.21892	-0.03738	0.064973	0.253876

LOC10050'	-0.0166664		-0.049903534	0.097465	-0.01961	0.069675	-0.14453
LOC10050'	-0.033811664		-0.098572721	0.051886	0.013632	-0.06433	-0.10633
LOC10050'	-0.045995522		0.010088133	-0.06168	-0.00884	0.060425	-0.01957
LOC10050'	-0.033225327		0.004410627	-0.00681	0.109073	-0.30017	-0.03733
LOC10050'	-0.012034357		-0.034103454	-0.09062	0.009124	-0.02807	-0.10739
LOC10050'	-0.010588592		-0.078723126	-0.12305	-0.00242	0.012042	-0.15897
LOC10050'	-0.023246416		-0.03089854	-0.12706	0.011057	-0.02246	-0.06811
LOC10050'	-0.021837566		-0.036624787	-0.04226	-0.01563	0.067074	-0.11439
LOC10050'	0.001226128		0.029450703	-0.09186	-0.04854	0.14996	0.311771
LOC10050'	-0.082165679		0.006648287	0.032094	0.002644	-0.02621	-0.01858
LOC10050'	-0.017853874		-0.100758507	-0.12458	0.005491	-0.02091	-0.05391
LOC10050'	-0.019332959		-0.000926588	-0.18763	0.035545	-0.12191	0.245728
LOC10050!	-0.035277286		-0.053869629	-0.09944	0.002149	0.001869	-0.07806
LOC10050!	-0.038544101		0.000548208	-0.02792	0.036164	-0.08347	0.022026
LOC10050!	-0.044121099		-0.058612211	0.009458	-0.02614	0.08995	0.01476
LOC10063(	-0.021688817		-0.037341582	-0.01393	0.010359	-0.06694	0.097239
LOC10063(	-0.025988991		0.040415576	-0.01352	0.026334	-0.05566	0.019556
LOC10063:	-0.056669486		-0.078071172	-0.06146	-0.0027	0.037556	-0.05482
LOC10065!	0.005417554		-0.003503784	0.154216	-0.02723	0.031646	-0.21854
LOC10065!	-0.098424015		-0.065588115	-0.01229	-0.0995	-0.01608	-0.02178
LOC10065!	-0.040004847		-0.026888994	0.023354	0.003817	0.003425	-0.00923
LOC10065!	-0.021794519		-0.066009275	0.00836	-0.00609	-0.00764	-0.0267
LOC10065!	-0.041277716		-0.117276852	0.021827	-0.0185	0.020557	-0.00264
LOC10065:	-0.04175673		-0.032424222	-0.02334	0.012264	-0.0102	0.084631
LOC10065:	-0.023319131		-0.071096162	0.022287	-0.00857	0.02055	0.232483
LOC10099(	0.000380764		-0.039273177	0.267354	-0.04357	0.141151	-0.08695
LOC10099(	-0.083888374		-0.015378662	0.084977	-0.03197	0.092513	0.017996
LOC10099(	0.019513768		0.047488264	-0.04005	-0.02718	0.069987	-0.01171
LOC10099(	-0.029574175		-0.053781542	0.014424	0.011622	-0.02118	-0.00421
LOC10099(	0.002540273		-0.040091421	0.548406	-0.05161	0.111446	-0.3005
LOC10099(	-0.145329756		-0.033621528	-0.03614	0.001317	0.03766	0.12298
LOC10099(	-0.029586521		-0.057603991	-0.09838	-0.04879	0.081889	0.182687
LOC10099(	-0.029618091		-0.039322501	0.040388	0.010628	-0.02188	-0.0449
LOC10099(	-0.04691736		-0.097877813	0.25445	-0.03757	0.11302	-0.08745
LOC10099(	-0.067219716		-0.029933619	-0.08481	-0.03227	0.068624	0.102466
LOC10099(	0.012099862		0.070990935	-0.1954	-0.05509	0.152958	0.402215
LOC10099(	-0.026554991		-0.073999589	-0.07986	0.025223	-0.11871	-0.03027
LOC10099(	-0.009866463		-0.119965033	-0.18619	-0.03329	0.11901	0.216262
LOC10099(	-0.022317606		0.000376906	0.004254	0.071525	-0.17991	-0.08949
LOC10099(	-0.007592834		-0.046197567	0.012289	-0.01754	-0.04317	-0.14278
LOC10099(	-0.032535543		-0.040175196	-0.06151	0.040025	-0.09939	-0.08428
LOC10099(	-0.045432924		-0.053134169	-0.03521	-0.01894	0.075579	0.171512
LOC10099(	-0.01661968		-0.040699084	0.138221	-0.03316	0.070251	-0.15441
LOC10099(	-0.008599314		-0.12925581	0.254662	-0.05611	0.18507	-0.2733
LOC10099(	-0.061902058		-0.036459899	0.044808	0.006632	0.02252	-0.05628
LOC10099(	-0.055305068		-0.111363295	0.066726	0.010342	-0.01539	-0.08649
LOC10099(	-0.034177292		-0.052133115	-0.01749	0.009882	-0.01824	0.033956
LOC10099(	-0.041293001		-0.028265565	-0.11221	0.015842	-0.01876	0.174882
LOC10099(	-0.033725594		-0.021241967	-0.06805	0.029171	-0.05578	0.003288
LOC10099(	-0.026285467		-0.070548607	-0.04356	-0.00519	-0.07874	-0.10009
LOC10099(	-0.033179675		-0.073842783	-0.02497	-0.00273	-0.00442	-0.01785

LOC10099(	-0.016682237	-0.05062361	0.136709	0.010851	-0.06998	-0.17155
LOC10099(	0.040117694	0.028236533	0.313526	-0.0141	0.039816	-0.23078
LOC10099(	0.009379398	-0.007005472	0.204396	-0.05802	0.203857	-0.1215
LOC10099(	-0.029181165	-0.057301613	-0.13814	-0.01314	0.057406	-0.03453
LOC10099(	-0.037408023	0.014497052	0.010739	0.035593	-0.10842	0.105127
LOC10099(	-0.030792779	-0.042571717	0.162732	0.013548	-0.01336	-0.00486
LOC10105!	-0.039544437	0.009388967	-0.12675	0.001066	0.029122	0.097707
LOC10106(	-0.032888184	-0.070747841	-0.05572	0.012895	-0.00909	0.094869
LOC10106(	-0.053094541	0.043874278	-0.04679	0.004434	-0.01794	0.004266
LOC10106(	-0.015155875	0.043860258	0.1095	0.009075	-0.01048	-0.19694
LOC10106(	-0.040419693	-0.035808864	-0.03003	0.039178	-0.09613	0.010336
LOC10106(	-0.009969172	-0.169740747	0.466495	-0.04369	0.146632	-0.18242
LOC10106(	-0.049790022	-0.052895086	0.054103	0.048829	-0.12883	0.127139
LOC10106(	-0.018726523	-0.015788239	0.023629	-0.00459	-0.03741	-0.08813
LOC10106(	0.005857089	-0.001190904	0.103657	-0.00928	0.084815	0.096502
LOC10106(	-0.030123161	-0.059876732	0.105965	0.003066	0.004479	-0.05733
LOC10106(	-0.015578954	0.025989585	0.194694	-0.01883	0.068432	-0.23939
LOC10106(	-0.004940344	0.022538726	-0.06437	0.004352	0.021128	0.095421
LOC10155!	-0.043296273	0.00343971	0.068741	0.040986	-0.09035	0.00056
LOC10159:	-0.032658333	-0.015755799	0.119723	-0.0009	0.011686	-0.08253
LOC10192(	-0.048425098	-0.025083401	0.103874	-0.01077	0.044345	0.096149
LOC10192(	-0.026148009	-0.051527496	0.01915	0.021853	-0.0529	0.038656
LOC10192(	-0.022824744	0.044185272	0.103185	0.013546	-0.01245	-0.13674
LOC10192(	-0.036958128	-0.05180089	0.021306	0.025704	-0.06225	-0.07166
LOC10192(	-0.056685154	-0.070169807	-0.08829	-0.00508	0.020381	-0.09126
LOC10192(	-0.026169863	-0.041970311	0.024224	0.012487	-0.04218	-0.07012
LOC10192(	-0.035534235	-0.065051008	-0.0499	0.02554	-0.05492	0.021341
LOC10192(	-0.013595431	-0.018624865	-0.03473	-0.00978	-0.02948	-0.02719
LOC10192(	-0.089327041	-0.102852402	0.002654	-0.00738	0.0213	-0.02003
LOC10192(	-0.041640267	-0.034224111	-0.12398	-0.01599	0.061094	0.18299
LOC10192(	-0.031168243	0.001031582	-0.09117	0.050548	-0.13535	0.037231
LOC10192(	-0.029044838	-0.068811087	0.18478	-0.00144	0.017398	-0.10696
LOC10192(	-0.036868667	-0.042476739	0.040595	-0.00018	0.00808	-0.11252
LOC10192(	-0.049568816	-0.053859281	0.022168	-0.01588	0.060759	-0.04558
LOC10192(	-0.06273521	-0.056432718	0.097344	-0.04125	0.100477	-0.01482
LOC10192(	-0.041900903	0.004854746	0.030643	0.022186	-0.03369	-0.14574
LOC10192(	0.007262138	0.001201304	-0.02509	-0.01	0.007925	0.130984
LOC10192(	0.003238554	-0.064042097	0.159589	-0.02671	0.114274	-0.02897
LOC10192(	-0.024711817	-0.034806581	-0.01418	-0.00152	0.00885	-0.03006
LOC10192(	-0.026723829	-0.084146266	0.313348	-0.04304	0.096902	-0.32482
LOC10192(	-0.038780528	-0.036534872	0.036967	0.02188	-0.06373	-0.04197
LOC10192'	-0.041244683	-0.070328683	0.039601	0.002701	-0.01936	-0.05384
LOC10192'	-0.052875154	-0.000438616	-0.00795	-0.0171	0.061923	0.078948
LOC10192'	-0.037942609	-0.052316962	0.014025	0.023705	-0.043	-0.0634
LOC10192'	-0.028198259	-0.048699633	-0.05027	0.070989	-0.19791	0.026205
LOC10192'	-0.010643376	-0.03474922	0.075694	0.128105	-0.12843	0.138482
LOC10192'	-0.031440798	-0.05940004	-0.05652	0.017821	-0.04028	0.091949
LOC10192'	-0.033680627	-0.051008968	0.106058	0.0008	0.020989	0.012472
LOC10192'	-0.065438264	-0.05415057	0.095375	-0.00611	0.037046	-0.01879
LOC10192'	-0.02892312	-0.036275663	0.033662	-0.01097	0.051614	-0.06641
LOC10192'	-0.04029055	-0.059064818	0.085505	0.009775	-0.01249	-0.06311

LOC10192'	-0.027828148	-0.057488023	0.007586	0.000621	-0.0039	-0.17583
LOC10192'	-0.047606522	0.01776889	-0.08732	0.013059	-0.0233	-0.09309
LOC10192'	-0.050681606	-0.096223946	0.15754	0.009929	0.006745	-0.14337
LOC10192'	-0.0342133	-0.065918081	0.138317	0.016127	-0.01297	0.011444
LOC10192'	-0.09351235	-0.067600859	0.052411	-0.01873	0.050866	-0.0328
LOC10192'	-0.049442013	0.006063236	-0.00483	0.045287	-0.10693	-0.07351
LOC10192'	-0.008522302	-0.117658403	-0.24536	-0.03819	0.122531	0.633847
LOC10192'	-0.030589318	-0.110975877	0.05208	0.027697	-0.07997	-0.07578
LOC10192'	-0.03796312	-0.043508912	0.027756	0.001767	0.016468	-0.08993
LOC10192'	-0.036101888	-0.027318435	0.043404	0.008848	-0.01367	-0.06717
LOC10192'	-0.036304546	0.011404037	0.033811	0.024965	-0.05145	0.047264
LOC10192'	-0.058586274	-0.062103653	-0.07489	0.02813	-0.07873	0.062555
LOC10192'	-0.029470027	-0.025209066	-0.09679	0.022796	-0.07977	-0.06231
LOC10192'	-0.020090038	-0.068020134	0.126298	0.009834	-0.03356	-0.05551
LOC10192'	-0.033813809	-0.01266317	0.004749	0.018744	-0.03879	-0.03741
LOC10192'	-0.055881881	-0.064875592	0.036267	-0.00383	0.021038	-0.06385
LOC10192'	-0.013160409	-0.081595643	0.415926	-0.04256	0.197049	-0.38619
LOC10192'	-0.018199525	-0.048692144	0.009242	-0.00693	-0.07968	-0.08502
LOC10192'	-0.051416179	-0.0516572	-0.00813	0.005104	-0.0093	-0.01696
LOC10192'	-0.071956386	-0.009179903	-0.06248	-0.00886	0.02862	0.124442
LOC10192'	-0.038720347	-0.04814688	0.010976	0.046959	-0.1103	0.011917
LOC10192'	-0.046558755	0.035787032	0.015365	0.005879	0.015978	-0.04868
LOC10192'	-0.032495794	-0.048113666	-0.11093	0.023702	-0.08695	-0.12904
LOC10192'	-0.053194906	-0.046834861	0.014757	-0.01368	0.073883	-0.1046
LOC10192'	-0.053913498	-0.043997527	-0.08726	0.006946	-0.02784	-0.05951
LOC10192'	-0.016465738	-0.061460677	-0.02853	-0.02839	-0.04183	-0.09503
LOC10192'	-0.029358746	-0.110397069	-0.12019	0.025874	-0.06453	0.12005
LOC10192'	-0.013799508	-0.027275067	-0.01344	0.001713	-0.10881	-0.05382
LOC10192'	-0.030762302	-0.062500428	-0.0307	0.003163	-0.0239	-0.0933
LOC10192'	-0.031592466	-0.025971801	-0.01849	0.008812	-0.01552	-0.135
LOC10192'	-0.024945967	0.005296633	0.069943	0.015493	-0.03809	-0.05099
LOC10192'	0.014472015	-0.002582718	0.055917	0.092266	-0.15066	-0.16515
LOC10192'	-0.012308177	0.11718977	0.115184	-0.00899	-0.04069	-0.26583
LOC10192'	-0.068010563	-0.049734738	-0.01697	0.002397	-0.0153	-0.11893
LOC10192'	0.001010727	0.113178426	0.118478	0.018538	-0.05196	-0.04865
LOC10192'	-0.026805591	-0.038199877	-0.05876	0.021785	-0.04808	-0.04323
LOC10192'	-0.026322188	-0.020707718	-0.02492	0.010603	-0.04793	-0.05962
LOC10192'	-0.043343514	-0.057687334	-0.05025	-0.00797	0.039609	-0.08838
LOC10192'	-0.038363015	-0.034273216	0.036414	0.020461	-0.02835	-0.09871
LOC10192'	-0.08072256	0.017390049	0.04779	-0.05046	0.124568	-0.0277
LOC10192'	-0.042416997	-0.033000649	0.00748	0.010508	-0.01437	-0.02631
LOC10192'	-0.022101763	-0.111063185	0.060772	-0.02017	0.070476	0.003118
LOC10192'	-0.040928124	-0.051675958	-0.03205	-0.00916	0.054889	-0.00301
LOC10192'	-0.034673615	0.001432898	0.128045	0.015763	-0.01911	0.0553
LOC10192'	-0.029536931	-0.043181235	-0.08057	0.03762	-0.0904	0.16862
LOC10192'	-0.033284521	-0.040836946	-0.11647	0.005852	0.011542	0.041015
LOC10192'	-0.031276421	-0.091055124	-0.09301	0.016537	-0.02042	-0.04437
LOC10192'	-0.030024952	-0.021146791	0.007421	0.002	0.008591	0.002914
LOC10192'	-0.040924464	-0.081747168	0.093346	-0.01318	0.066911	0.001184
LOC10192'	-0.039830032	-0.044897948	0.089337	0.02149	-0.04556	-0.01504
LOC10192'	-0.035923446	-0.091363423	0.029036	-0.00391	0.004301	-0.03985

LOC10192'	-0.026118089	-0.057966218	0.014819	-0.00237	-0.01741	0.002968
LOC10192'	-0.048944318	-0.053240962	0.002687	0.002819	-0.00075	0.018789
LOC10192'	-0.027318761	-0.036073613	0.062031	0.012713	-0.02423	-0.02666
LOC10192'	-0.034023228	-0.090492785	0.00881	0.032113	-0.07454	0.037011
LOC10192'	-0.067718682	-0.028678273	0.041869	-0.03476	0.063835	-0.00764
LOC10192'	-0.021471923	-0.042705418	-0.03721	-0.01265	0.013392	-0.05403
LOC10192'	-0.070622405	-0.071424373	0.059859	-0.02544	0.069114	0.105331
LOC10192'	-0.046977191	-0.071095791	-0.0108	0.024291	-0.04013	0.03734
LOC10192'	-0.076549889	-0.076545948	0.11745	-0.01092	0.021714	0.03105
LOC10192'	-0.018976391	-0.007715662	-0.02627	0.087908	-0.24269	0.065494
LOC10192'	-0.041283586	-0.0016456	-0.03178	0.033808	-0.06754	-0.06427
LOC10192'	-0.042651047	-0.020154506	0.167982	0.001641	0.03703	-0.01708
LOC10192'	-0.043803385	-0.08757717	-0.00254	-0.00563	0.041666	-0.04237
LOC10192'	-0.052185616	0.044431089	0.013516	0.066303	-0.16791	-0.01005
LOC10192'	-0.035045872	-0.022071314	0.080503	0.022305	-0.03614	-0.15186
LOC10192'	-0.053657623	-0.007039982	-0.00375	-0.01581	0.037864	-0.06815
LOC10192'	-0.030097608	-0.092440355	0.037054	-0.00335	-0.00761	-0.08475
LOC10192'	-0.018012723	-0.065753517	-0.01026	-0.01665	-0.06185	-0.15554
LOC10192'	-0.04199038	-0.02741554	-0.02547	-0.00338	-0.00569	-0.08434
LOC10192'	-0.002282816	-0.032822448	0.140278	-0.01008	0.05975	0.125536
LOC10192'	-0.064466442	-0.069126393	-0.0013	-0.00812	0.022215	0.012658
LOC10192'	-0.031734274	-0.06777958	-0.01274	0.000995	-0.01349	-0.00524
LOC10192'	-0.029018123	-0.036360356	0.013495	0.010815	-0.02046	1.11E-05
LOC10192'	-0.029765728	0.095083831	0.05789	-0.05325	0.155324	-0.08798
LOC10192'	-0.055798735	-0.037813232	0.047809	-0.00438	-0.00558	-0.1031
LOC10192'	-0.025799508	-0.071740522	-0.03493	0.097978	-0.24005	-0.08635
LOC10192'	-0.034891031	-0.016810121	-0.03457	0.021703	-0.06226	-0.17491
LOC10192'	-0.035616587	-0.03632899	-0.20774	0.005686	0.006843	0.029175
LOC10192'	-0.044064138	-0.110529469	0.102235	-0.0032	0.03503	0.057609
LOC10192'	-0.024670594	0.037584577	0.01777	0.016652	-0.02148	-0.05297
LOC10192'	-0.022159845	-0.095994822	-0.0199	-0.02195	-0.01811	0.106848
LOC10192'	-0.018091082	-0.107310011	-0.16793	0.007065	-0.00277	-0.1183
LOC10192'	-0.051431535	-0.084963569	-0.0557	0.021404	-0.03865	0.037966
LOC10192'	-0.05850144	-0.088881636	0.03435	-0.00314	0.010223	-0.07425
LOC10192'	0.002386384	-0.049633715	0.120849	-0.01055	0.055006	-0.27965
LOC10192'	-0.028887224	-0.031628807	-0.04467	0.028138	-0.06867	-0.07753
LOC10192'	-0.027746428	-0.030108746	-0.01222	0.061248	-0.16021	0.015006
LOC10192'	-0.012200721	-0.047667769	0.055344	-0.00722	-0.00875	-0.1115
LOC10192'	-0.035391152	-0.043258209	-0.09563	0.005331	-0.00281	-0.09546
LOC10192'	-0.056724772	-0.158281555	0.021849	-0.00208	0.021592	-0.09406
LOC10192'	-0.035824299	0.015464165	-0.00724	0.022053	-0.0458	-0.01596
LOC10192'	-0.04134037	-0.004017444	-0.05392	0.011164	-0.00073	0.111311
LOC10192'	-0.036479724	-0.056104975	-0.04239	-0.00703	0.041497	0.015013
LOC10192'	-0.02394045	-0.094653078	-0.03728	0.017592	-0.10796	-0.04722
LOC10192'	-0.029239388	-0.026084525	0.02739	0.030129	-0.07678	-0.01427
LOC10192'	-0.058460062	-0.070217703	0.12024	0.016299	-0.06226	0.048591
LOC10192'	-0.024310333	-0.062547745	0.060466	0.001918	-0.06377	-0.13767
LOC10192'	-0.046672058	-0.043302205	0.037595	-0.00198	0.027505	0.047524
LOC10192'	-0.030673696	-0.020438522	-0.12484	0.004674	-0.00531	-0.04541
LOC10192'	-0.035434258	-0.08139889	-0.00744	-0.00234	0.025675	-0.03867
LOC10192'	-0.02810695	-0.09175546	-0.07314	-0.01592	-0.04598	-0.04554

LOC10192′	-0.027848872	0.003490763	0.083809	0.011585	-0.02706	-0.03804
LOC10192′	-0.030560832	-0.06086733	-0.01814	0.005083	-0.0417	-0.03553
LOC10192′	-0.042786632	-0.063217171	-0.00903	0.017247	-0.02389	0.131611
LOC10192′	-0.021496225	-0.050474001	-0.01472	0.014118	-0.01314	-0.03901
LOC10192′	-0.024992576	0.000773084	-0.02526	-0.00377	-0.00325	-0.07989
LOC10192′	-0.027982898	0.012978718	0.042238	0.005439	-0.01579	-0.06733
LOC10192′	-0.032800446	0.01675493	0.056837	0.01763	-0.03164	0.122968
LOC10192′	-0.039414679	-0.01445718	0.006765	0.039709	-0.0938	-0.02066
LOC10192′	-0.044767546	-0.086240683	-0.00834	-0.01261	0.060006	-0.053
LOC10192′	-0.030153465	-0.016066369	-0.07698	-0.024	0.077214	0.036329
LOC10192′	-0.023924109	0.0722381	-0.10669	0.001848	-0.02245	-0.00298
LOC10192′	-0.041446902	-0.005911707	-0.01509	0.017861	-0.02857	0.046351
LOC10192′	-0.044341619	-0.050171681	0.075754	0.008755	-0.00149	-0.13209
LOC10192′	-0.026956667	-0.07399408	0.074728	0.021166	-0.09605	-0.0637
LOC10192′	-0.004150209	-0.113077833	0.019754	-0.0217	0.049278	0.28942
LOC10192′	-0.063691163	0.028466008	0.02438	-0.03685	0.084849	-0.02002
LOC10192′	-0.016895599	0.099259278	0.195018	0.003687	0.004256	-0.08669
LOC10192′	-0.027778549	-0.087633702	0.125505	0.003501	-0.04077	-0.11062
LOC10192′	-0.036829706	-0.002445898	-0.07528	0.049146	-0.12207	0.126232
LOC10192′	-0.045908208	-0.031373303	0.018816	0.02826	-0.04851	-0.04548
LOC10192′	-0.020095779	-0.026886116	0.106472	0.004218	-0.02588	-0.08979
LOC10192′	-0.035177659	-0.05859045	0.042558	0.017862	-0.03394	-0.0496
LOC10192′	-0.035009848	-0.01868929	0.070975	-0.01403	0.015037	-0.15776
LOC10192′	-0.0280902	0.008820029	-0.00586	-0.00592	0.0226	0.06862
LOC10192′	-0.04527265	-0.027437974	0.097874	0.007726	0.00034	-0.07953
LOC10192′	-0.051913554	-0.090419697	-0.07836	-0.0114	0.024624	0.058033
LOC10192′	-0.035257099	-0.043321822	0.009445	0.017376	-0.02681	-0.10083
LOC10192′	-0.066823714	-0.003331844	0.016658	0.01085	-0.016	0.035817
LOC10192′	-0.023108203	-0.059531468	-0.06022	0.012945	-0.10213	0.014514
LOC10192′	-0.047048168	-0.067977394	-0.03488	-0.00962	0.052312	0.099393
LOC10192′	-0.041647372	-0.100306339	-0.1102	-0.06831	0.146475	0.234028
LOC10192′	-0.030675502	-0.03336212	0.010806	0.04501	-0.09976	0.071864
LOC10192′	-0.032169783	-0.032516544	0.094023	0.032469	-0.11276	-0.04797
LOC10192′	-0.018458588	-0.020825905	-0.08	-0.0082	-0.05108	0.096292
LOC10192′	-0.122752679	-0.072362836	0.00286	-0.00013	0.080591	-0.03282
LOC10192′	-0.023579765	-0.055008636	0.029908	-0.00899	-0.06948	-0.09649
LOC10192′	-0.048269021	-0.040912508	0.03925	-0.00477	0.029804	-0.05079
LOC10192′	-0.033800298	-0.009408497	0.079365	-0.04539	0.151132	-0.31763
LOC10192′	-0.038058858	-0.033458776	0.204801	0.019168	-0.06232	-0.07088
LOC10192′	-0.054973843	-0.073454139	0.023271	-0.03532	0.116948	-0.14178
LOC10192′	-0.091473379	-0.093423005	-0.01805	-0.02384	0.092146	0.055082
LOC10192′	-0.040307965	-0.03025705	-0.15248	0.015582	-0.02588	0.021236
LOC10192′	-0.033543772	-0.00593559	-0.03231	-0.01585	0.049086	-0.01613
LOC10192′	0.008491023	0.037703415	0.065424	-0.10208	0.303561	0.228781
LOC10192′	-0.079027837	-0.091746568	0.050217	-0.00974	0.011038	-0.12349
LOC10192′	-0.031781318	-0.03419687	0.008725	0.061913	-0.16687	0.0524
LOC10192′	-0.032716843	-0.102608917	-0.01029	-0.00214	-0.00086	-0.06811
LOC10192′	-0.025693282	-0.082857466	0.072342	-0.00284	0.0255	0.003357
LOC10192′	-0.024466576	-0.053337859	0.025941	-0.00296	0.036698	-0.05337
LOC10192′	-0.039027465	-0.106128238	0.01156	0.017483	-0.02478	0.036932
LOC10192′	-0.034441921	-0.020238767	-0.0251	0.009056	-0.03162	-0.0852

LOC10192'	-0.061371296	-0.019008902	-0.06755	0.009269	-0.03925	-0.01639
LOC10192'	-0.045080429	-0.039390717	0.031726	0.019789	-0.03766	-0.01797
LOC10192'	-0.03934711	-0.024868345	-0.04598	0.012678	-0.02346	0.000229
LOC10192'	-0.00982046	-0.025958697	0.031947	-0.02473	0.038037	-0.05057
LOC10192'	-0.032882885	-0.033494229	-0.01123	0.009159	-0.0202	0.003554
LOC10192'	-0.023686494	-0.020738631	0.02291	0.005615	-0.01263	-0.07358
LOC10192'	-0.068661081	-0.098646419	-0.01947	-0.02553	0.059438	0.100973
LOC10192'	-0.031524876	0.029386416	0.072758	0.012113	-0.01039	-0.1064
LOC10192'	-0.060572435	-0.052666095	0.048881	-0.02482	0.063354	0.108817
LOC10192'	-0.025721826	-0.060251357	0.022448	0.036468	-0.07973	-0.00374
LOC10192'	-0.004218366	-0.038116793	0.110926	0.059683	-0.14387	-0.13812
LOC10192'	-0.046981997	-0.010417139	-0.04684	-0.02322	0.091745	0.014107
LOC10192'	-0.027772116	0.0049081	0.028472	0.009746	-0.04948	-0.14467
LOC10192'	-0.039386258	-0.047879217	-0.09114	-0.01319	0.069567	-0.03135
LOC10192'	-0.070651107	-0.04902925	-0.00295	-0.02868	0.068994	0.021524
LOC10192'	-0.140418766	-0.134924227	0.048855	0.000188	0.066102	0.012169
LOC10192'	-0.038875406	0.007288902	0.135757	0.038407	-0.07644	0.055825
LOC10192'	-0.030338929	-0.020315754	0.02488	0.003803	-0.02667	0.039247
LOC10192'	-0.026932127	-0.007324851	0.008801	-0.00388	-0.02535	-0.03981
LOC10192'	-0.001212235	-0.115396674	-0.156	0.0757	-0.18655	0.071825
LOC10192'	-0.035064437	-0.024655251	-0.10413	0.04743	-0.11075	-0.12583
LOC10192'	-0.038705036	-0.081703286	0.001718	0.009111	-0.02348	0.006939
LOC10192'	-0.030855893	-0.039985733	0.019211	0.001857	0.016323	0.02198
LOC10192'	-0.038978419	-0.091726878	-0.02292	0.009593	-0.01707	0.004475
LOC10192'	-0.008276568	-0.004651533	0.091899	0.013141	-0.08354	-0.33361
LOC10192'	-0.037747161	-0.062833267	0.015261	0.003439	0.028685	0.146137
LOC10192'	-0.026464833	0.018541682	0.01276	0.019217	-0.03352	-0.06196
LOC10192'	-0.038886268	0.022940678	-0.14047	0.000945	0.021528	0.116617
LOC10192'	-0.024177169	-0.012864642	0.103371	-0.00388	-0.02847	-0.0056
LOC10192'	-0.041346741	-0.002158618	0.165537	1.91E-06	0.005362	0.035966
LOC10192'	-0.05633506	-0.069001331	-0.04667	-0.0226	0.052199	0.059175
LOC10192'	-0.002521236	-0.028899188	-0.16171	-0.00172	-0.024	0.13092
LOC10192'	-0.101965854	-0.082671629	0.175505	-0.01714	0.0769	-0.09443
LOC10192'	-0.021312985	-0.011141481	-0.00648	-0.00738	0.019537	-0.1328
LOC10192{	0.009510047	0.017248342	0.241914	0.065888	-0.18002	-0.32899
LOC10192{	-0.100642449	-0.063951276	-0.07325	-0.04113	0.157345	-0.02241
LOC10192{	-0.06205945	-0.066896554	-0.02834	-0.01266	0.020948	0.030541
LOC10192{	-0.037576897	0.007827272	-0.09642	0.016767	-0.019	-0.13966
LOC10192{	-0.072705233	-0.077410093	-0.05643	-0.02964	0.055655	0.168707
LOC10192{	-0.028720384	-0.099030316	-0.06038	0.027242	-0.0725	0.034098
LOC10192{	-0.045778111	-0.03966055	-0.05889	-0.02395	0.065329	-0.10379
LOC10192{	-0.042147974	-0.065115484	-0.07375	-0.0023	0.028125	-0.08891
LOC10192{	-0.048166837	-0.014110123	-0.06598	-0.03165	0.108942	0.100081
LOC10192{	-0.037877132	-0.047374855	0.11197	0.021536	-0.03046	0.02572
LOC10192{	-0.039016766	-0.031387831	0.128485	-0.01682	0.081209	-0.07585
LOC10192{	-0.04145265	-0.105573871	-0.02106	-0.01374	0.063297	0.199068
LOC10192{	-0.067476431	-0.036494931	0.008279	-0.00877	0.015089	-0.11527
LOC10192{	0.014811368	0.145341178	0.100992	-0.04902	0.115906	-0.21196
LOC10192{	-0.048477335	-0.048628506	-0.05299	-0.01283	0.002667	0.08977
LOC10192{	-0.04024283	-0.099650832	0.101467	0.008542	-0.07679	-0.0883
LOC10192{	-0.033394164	-0.016063825	0.009276	-0.00059	0.020987	-0.14512

LOC101928	-0.024787605	-0.098086081	-0.02835	0.040189	-0.09351	0.108731
LOC101928	-0.055993945	0.03929505	0.082868	0.00036	0.013497	0.071711
LOC101928	-0.029324656	-0.02696927	0.038739	0.01608	-0.0424	-0.18095
LOC101928	-0.030939185	0.069418211	0.073974	0.020181	-0.08074	-0.07866
LOC101928	-0.037278339	-0.107762059	0.14575	-0.01185	0.052361	-0.12618
LOC101928	-0.032539297	-0.002223298	-0.02858	0.023158	-0.05186	0.032093
LOC101928	-0.037349708	-0.022053027	0.020751	0.013716	-0.01911	-0.12013
LOC101928	-0.013601362	0.022987601	0.107509	0.005088	0.015551	0.063068
LOC101928	-0.009347687	-0.079824888	-0.11587	-0.00992	0.045279	-0.05262
LOC101928	-0.031229122	-0.039241738	0.058562	0.005159	0.00542	-0.05627
LOC101928	-0.029586505	-0.035890233	0.007017	-0.00814	-0.02935	-0.03636
LOC101928	-0.034712052	-0.081321395	0.000545	0.040023	-0.08485	-0.01025
LOC101928	-0.026351321	-0.016834142	0.032914	0.002738	0.010231	-0.05427
LOC101928	-0.013145094	-0.159896541	0.697754	-0.00796	0.050071	-0.15308
LOC101928	-0.024362924	0.037943694	0.014345	0.005544	-0.04593	-0.10224
LOC101928	-0.005735113	-0.088965231	0.268551	-0.0682	0.17073	-0.19187
LOC101928	-0.039582043	-0.036269963	0.027665	-0.01383	0.060793	0.05487
LOC101928	-0.050322039	-0.018883641	0.026361	0.011975	-0.01983	0.070586
LOC101928	-0.040681293	-0.077273091	0.013001	-0.00314	0.027939	-0.09999
LOC101928	-0.055012497	-0.030863925	-0.08595	-0.03343	0.093628	-0.03301
LOC101928	-0.00838032	-0.025909815	0.133208	0.103856	-0.28078	-0.0504
LOC101928	-0.033591525	-0.041531869	-0.06619	0.035751	-0.09666	-0.01282
LOC101928	-0.056330515	-0.035020887	-0.04061	0.023141	-0.0651	-0.06675
LOC101928	-0.036724725	-0.054788177	-0.04254	0.011876	-0.01365	-0.02804
LOC101928	-0.049460806	-0.005128345	0.097346	-0.0117	0.046159	-0.10218
LOC101928	-0.024337998	-0.025832103	0.010217	-0.00764	-0.07914	-0.1226
LOC101928	-0.036319239	0.0087167	0.087917	0.057324	-0.14021	-0.05729
LOC101928	-0.032452565	-0.037325859	-0.1122	0.015828	-0.02901	-0.1743
LOC101928	-0.044708518	-0.032332204	-0.06559	0.012081	0.005241	-0.04326
LOC101928	-0.126962624	-0.040470641	0.00305	-0.03767	0.099149	-0.00879
LOC101928	-0.026074527	-0.054271402	0.03457	0.011227	-0.10747	-0.16992
LOC101928	-0.026756969	-0.06504607	0.026573	0.002421	-0.01481	-0.10075
LOC101928	-0.008732858	0.054799956	-0.00032	0.016308	-0.08841	-0.06037
LOC101928	-0.044065011	-0.07763326	-0.10704	0.028127	-0.05525	0.03897
LOC101928	-0.03277659	-0.070994103	0.11206	0.032674	-0.10998	-0.0514
LOC101928	-0.044028993	-0.035083571	-0.0283	0.026962	-0.04665	-0.08179
LOC101928	-0.034919128	-0.059151003	-0.08958	0.02367	-0.06453	0.101234
LOC101928	-0.033295995	0.023639663	-0.06555	-0.00423	-0.00333	-0.06467
LOC101928	-0.043105578	-0.012221719	-0.057	0.005235	0.011455	0.026186
LOC101928	-0.02895022	-0.052927747	0.190112	-0.02087	0.055696	-0.06319
LOC101928	-0.079504377	-0.03862503	-0.07313	-0.0029	0.016446	0.007177
LOC101928	-0.033938076	-0.036710376	0.013214	-0.01597	0.069649	0.106269
LOC101928	-0.036487205	0.003585419	-0.09882	0.02992	-0.08166	-0.19009
LOC101928	-0.050169137	-0.048473305	-0.00139	-0.02712	0.077098	-0.06425
LOC101928	-0.040432643	-0.083249802	-0.09883	-0.01801	0.077208	0.04862
LOC101928	-0.016403405	-0.042717699	0.004777	-0.00162	-0.04554	-0.12451
LOC101928	-0.06909339	-0.067411322	0.089955	-0.00675	0.024146	0.001739
LOC101928	-0.037586692	-0.074993185	-0.09728	0.012696	-0.01409	0.168818
LOC101928	-0.028778096	-0.05822553	0.003358	-0.00313	-0.03164	-0.05022
LOC101928	-0.039434157	-0.070211771	0.028242	0.000115	0.024996	-0.11824
LOC101928	-0.060936921	-0.059884214	-0.04181	-0.02214	0.066344	-0.08262

LOC101928	-0.018652854	-0.084820068	-0.12683	-0.02224	0.090576	0.296004
LOC101928	-0.041192642	-0.061784998	0.009523	-0.02727	0.090868	-0.02073
LOC101928	-0.056363154	-0.064742265	-0.13843	0.010558	-0.00205	0.045839
LOC101928	-0.0380539	-0.060252063	0.070079	0.022171	-0.04166	-0.1378
LOC101928	-0.038523751	-0.082228403	0.026378	-0.00094	0.02199	-0.09669
LOC101928	-0.00457749	-0.093682193	-0.20396	-0.0715	0.154168	0.316224
LOC101928	-0.010271439	-0.01237564	0.239772	-0.03118	0.128594	-0.19828
LOC101928	-0.033508059	-0.001423644	-0.00969	-0.01078	-0.01525	-0.08694
LOC101928	0.004750666	-0.054972205	0.059535	-0.00737	0.008342	-0.05653
LOC101928	-0.087942258	-0.076102569	0.056312	-0.04004	0.079823	-0.0561
LOC101928	-0.083961381	-0.045416596	-0.11164	-0.01714	0.034216	0.007665
LOC101928	-0.043763121	-0.048693088	-0.11769	-0.01355	0.068198	0.237167
LOC101928	-0.027152846	-0.121949478	0.029516	0.009741	-0.02672	0.015675
LOC101928	-0.023209199	-0.05343656	0.048787	0.005638	-0.02175	-0.07588
LOC101928	-0.018910574	0.030550985	0.238169	0.029433	-0.0711	-0.27301
LOC101928	-0.028855127	-0.091272711	0.132591	-0.01131	0.041086	-0.09051
LOC101928	-0.02521535	-0.017744294	-0.00745	0.039948	-0.12129	-0.08481
LOC101928	-0.052816362	-0.097117544	0.070793	-0.036	0.127961	-0.06588
LOC101928	-0.053538887	-0.08815756	0.165142	-0.00555	0.039422	0.003735
LOC101928	-0.01243177	-0.047601581	0.035068	-0.22427	0.618887	-0.00574
LOC101928	-0.043470187	-0.013837041	0.061043	0.001475	0.015645	-0.11167
LOC101928	-0.028038638	-0.119076719	0.008878	0.064215	-0.15695	0.080552
LOC101928	0.001060572	-0.038735464	-0.3909	-0.09242	0.273847	0.597051
LOC101928	-0.021333634	0.021499018	-0.00463	0.023766	-0.10313	-0.09169
LOC101928	-0.028933885	-0.041659084	-0.02534	-0.00164	-0.04768	-0.07788
LOC101928	-0.033039528	-0.030422168	-0.01739	0.011376	-0.01384	-0.11075
LOC101928	-0.044426489	-0.035651597	0.025735	0.02533	-0.04884	0.046608
LOC101928	-0.049162451	-0.042044142	0.024455	0.030092	-0.05318	-0.0038
LOC101928	-0.049210883	-0.087455621	-0.11134	-0.00651	0.019753	0.084978
LOC101928	-0.036981567	-0.021576717	-0.1284	0.02118	-0.04943	0.033393
LOC101928	-0.025868849	0.0243514	0.00545	0.039	-0.10548	0.033973
LOC101928	0.007608592	-0.115572063	0.142584	0.005434	0.012254	-0.0533
LOC101928	-0.059046116	-0.0516305	-0.02708	-0.00238	0.002287	0.12077
LOC101928	-0.018851224	-0.012366254	0.036769	-0.005	-0.01055	-0.02242
LOC101928	-0.034107791	0.001091045	0.01669	-0.02439	0.023554	0.012873
LOC101928	-0.011680728	-0.009037823	-0.06839	-0.0089	-0.02176	0.115712
LOC101928	-0.056373338	-0.061599393	0.040553	0.004336	-0.01127	-0.04325
LOC101928	-0.090498081	-0.051543826	0.039639	-0.01904	0.024415	0.069031
LOC101928	-0.061248518	-0.100271857	-0.11303	-0.01269	0.043575	0.064363
LOC101928	-0.041500615	0.013694933	0.010991	0.031109	-0.07097	0.053268
LOC101928	-0.024739882	-0.05560167	-0.00054	-0.01392	-0.00998	-0.04108
LOC101928	-0.025484422	-0.024800174	0.009247	0.01613	-0.05005	-0.05936
LOC101928	-0.089537963	-0.041176591	0.033293	0.031287	-0.0603	-0.02331
LOC101928	-0.034794686	-0.016660858	-0.06572	-0.00589	0.050283	0.084878
LOC101928	-0.028101931	0.001817007	-0.10748	-0.00042	-0.05758	0.093463
LOC101928	-0.066071306	-0.054967126	0.155798	-0.00473	0.02471	-0.07307
LOC101928	-0.036908045	-0.041401318	-0.02668	0.011211	-0.01669	0.103795
LOC101928	-0.07344577	-0.084518323	-0.02263	-0.02022	0.034384	0.021165
LOC101928	-0.018467471	-0.082762097	0.257109	0.005363	-0.01089	-0.16722
LOC101928	-0.060021583	-0.040028951	-0.05126	-0.01709	0.001731	0.030939
LOC101928	-0.056015559	-0.018539958	-0.12919	0.023661	-0.05724	-0.01264

LOC101928	-0.026737468	-0.099880672	0.032041	0.006853	0.001023	-0.0893
LOC101928	-0.084828161	0.050593196	-0.08643	-0.00952	0.076375	-0.10484
LOC101928	-0.030879436	-0.035751228	-0.14306	0.020023	-0.05323	0.079209
LOC101928	-0.055368263	-0.081989133	-0.05407	0.000356	0.018752	0.004682
LOC101928	-0.035321039	-0.033941406	0.019517	0.013312	-0.01517	-0.13024
LOC101928	-0.032890406	-0.037976764	0.046181	0.013231	-0.01628	-0.1643
LOC101928	-0.039373226	-0.058998534	0.095319	-0.01886	0.070414	0.009393
LOC101928	-0.029137341	-0.041619864	-0.10112	0.01308	-0.03859	-0.12315
LOC101928	-0.067187935	-0.029035105	-0.05457	0.002287	-0.00627	0.001074
LOC101928	-0.034699019	-0.061005467	0.024634	0.007313	0.00163	-0.1281
LOC101928	-0.032357734	-0.043983926	-0.00949	0.003679	-0.02278	-0.06386
LOC101928	-0.035984269	-0.038367138	-0.22041	0.022823	-0.03712	0.055797
LOC101928	-0.045146166	-0.060062244	0.151667	0.014906	-0.01858	-0.09699
LOC101928	-0.089912946	-0.045216588	-0.07863	-0.03095	0.059882	-0.09888
LOC101928	-0.108371186	-0.102371125	-0.02869	0.006813	0.038846	-0.01439
LOC101928	-0.025381956	0.006687824	0.145803	0.006104	0.007978	-0.07177
LOC101928	-0.026151957	-0.024876991	0.021354	-0.00489	-0.04539	-0.06484
LOC101928	-0.031221274	-0.060124048	-0.04439	0.012765	-0.05319	0.101949
LOC101928	-0.059245537	-0.07191556	-0.04942	0.038517	0.021437	-0.01592
LOC101928	-0.039910608	-0.00015944	-0.01122	0.004236	0.001841	-0.14282
LOC101928	-0.026998994	-0.023490778	-0.05235	0.000167	0.005206	0.010655
LOC101928	-0.041971208	-0.029237326	-0.01602	0.049614	-0.10196	-0.04237
LOC101928	-0.033301492	-0.066153319	0.165784	-0.00479	0.02112	-0.06163
LOC101928	-0.034992834	-0.12621848	-0.04934	0.01664	-0.05332	0.056723
LOC101928	-0.044331472	-0.020955587	-0.011	-0.00392	0.007508	0.000296
LOC101928	-0.050190615	-0.001525836	0.02756	-0.00739	0.036867	-0.08287
LOC101928	-0.047598098	-0.003755421	-0.01702	0.023069	-0.07525	0.027261
LOC101928	-0.020651742	-0.048393683	0.036064	0.090646	-0.21747	-0.05729
LOC101928	-0.036991807	-0.054863433	-0.04007	0.001787	0.023634	-0.01555
LOC101928	-0.033714743	-0.084251098	-0.19907	0.021775	-0.03214	0.249823
LOC101928	-0.032255227	0.00118024	0.176219	0.01768	-0.04026	-0.13878
LOC101928	-0.014533661	0.00783805	0.034786	0.07281	-0.20289	-0.02652
LOC101928	-0.04279349	-0.075590656	0.072464	-0.00363	0.041231	-0.02677
LOC101928	-0.02640885	0.001457371	-0.04369	-0.00697	0.017604	-0.19709
LOC101928	0.018762275	0.000111597	0.064038	0.044587	-0.04588	-0.02648
LOC101928	-0.033015552	-0.071236738	0.014252	0.006817	0.003945	0.148313
LOC101928	-0.046163206	-0.048308264	-0.056	0.039228	-0.07832	0.097824
LOC101928	-0.038472171	-0.002943616	0.001621	0.003965	0.006795	0.000876
LOC101928	-0.074310055	-0.083650117	-0.06133	-0.04022	0.11006	0.049008
LOC101928	-0.042707017	-0.050832539	-0.06156	0.028995	-0.0527	-0.08914
LOC101928	-0.042792787	0.007229594	0.001645	-0.00152	0.023351	0.045396
LOC101928	-0.010566223	0.042834747	-0.14715	-0.01976	0.018418	0.118097
LOC101928	-0.02912213	0.130790005	0.178507	-0.02633	0.096863	-0.14424
LOC101928	-0.041177207	-0.056470669	-0.05216	0.003819	0.021921	0.038147
LOC101928	-0.031239635	-0.052326407	-0.06664	-0.00302	-0.06303	-0.05205
LOC101928	-0.040817357	-0.049247722	-0.12891	-0.00827	0.059204	0.068292
LOC101928	-0.07974503	-0.065521158	-0.10686	-0.04923	0.129057	0.022299
LOC101928	-0.032434397	0.019020272	-0.00035	-0.00264	0.021549	-0.15164
LOC101928	-0.030792628	-0.034824298	-0.06013	0.00149	0.016184	0.035882
LOC101928	-0.050935291	-0.018820939	-0.02176	0.003258	0.010394	0.022424
LOC101928	-0.039116479	-0.051247798	-0.05809	0.028501	-0.05883	0.087084

LOC101928	-0.014638785		-0.078884889	0.081196	-0.00537	0.032954	-0.27357
LOC101928	-0.065478944		-0.055116624	0.164402	-0.02682	0.0592	-0.21824
LOC101928	-0.038759818		-0.050307978	-0.08056	-0.05991	0.21046	0.111644
LOC101928	-0.043243375		-0.070899406	0.039704	0.006853	0.000616	-0.02124
LOC101928	-0.01974675		-0.015478252	0.015942	########	-0.02168	-0.1073
LOC101928	-0.059326946		-0.039004509	-0.00488	0.00943	-0.02277	-0.03311
LOC101928	-0.056027955		-0.064821153	-0.01896	-0.01664	0.031256	-0.05864
LOC101928	-0.033340248		-0.000764846	0.03507	-0.18293	0.551613	-0.04343
LOC101928	-0.033632928		-0.007325568	-0.03817	-0.01015	0.047796	-0.00334
LOC101928	-0.030392727		-0.047975161	-0.01597	0.003781	-0.05442	-0.15817
LOC101928	-0.029442547		-0.045362474	-0.15422	0.001177	0.020465	-0.06271
LOC101928	-0.03677668		0.126140001	-0.17913	0.004619	0.015586	-0.01732
LOC101928	-0.036398531		-0.028082038	0.027999	0.021737	-0.0493	-0.09933
LOC101928	-0.031858362		-0.077997133	-0.01433	-0.01078	0.028584	-0.1718
LOC101928	-0.037238042		0.030933989	0.006252	0.037755	-0.07848	-0.09887
LOC101928	-0.049844323		-0.110892417	-0.05879	-0.01089	0.051144	-0.04253
LOC101928	-0.031405254		-0.038587714	-0.08103	-0.01616	0.070587	0.049614
LOC101928	-0.029023518		-0.042464987	0.056284	0.010929	-0.08602	-0.05651
LOC101928	-0.06543228		-0.03323179	0.067176	-0.01467	0.022589	0.072813
LOC101928	-0.028383745		-0.01300395	0.027014	-0.01908	-0.04345	-0.05931
LOC101928	-0.033077383		-0.032683285	0.056304	0.003462	0.011701	-0.03525
LOC101928	-0.005102788		-0.041209138	-0.23332	-0.00079	0.050524	0.219563
LOC101928	-0.035599857		-0.053010656	0.03125	0.001576	-0.00643	-0.03398
LOC101928	-0.029783892		-0.006026159	-0.07191	-0.01645	0.034992	-0.06324
LOC101928	-0.049362593		-0.004615783	0.051372	0.023924	-0.03331	-0.05603
LOC101928	-0.045222926		-0.076243754	0.021324	0.03047	-0.06309	0.050842
LOC101928	-0.030871939		-0.103891041	0.087723	0.010344	-0.01656	0.014352
LOC101928	-0.038432814		0.013904003	-0.11124	0.033234	-0.08249	-0.02988
LOC101928	-0.0340466		-0.000354008	-0.01536	0.026269	-0.07456	-0.02212
LOC101928	-0.023656735		-0.025528804	0.077507	0.005051	-0.01719	-0.09566
LOC101928	-0.045408569		-0.024054476	-0.16468	-0.01774	0.07537	-0.05299
LOC101928	-0.004039214		-0.011710752	-0.00022	0.01435	-0.01139	0.008639
LOC101928	-0.055780642		-0.090396876	-0.16729	0.02719	-0.03988	0.080596
LOC101928	-0.048167725		-0.072290592	0.00587	-0.01859	0.023003	-0.04716
LOC101928	-0.013924159		-0.037437143	0.13966	-0.01229	0.046701	-0.10977
LOC101928	-0.046383154		-0.053094856	-0.09356	0.014969	-0.02032	-0.17135
LOC101928	-0.013361785		-0.107496835	-0.06928	-0.00557	-0.03686	-0.06931
LOC101928	-0.037393727		-0.073651884	-0.15139	0.007876	0.009824	-0.01762
LOC101928	-0.019279984		-0.033558653	-0.05968	0.002367	-0.01903	-0.19817
LOC101928	-0.032185263		0.039095239	-0.05493	0.001126	0.00606	-0.02612
LOC101928	-0.061525207		-0.017611884	-0.01715	0.008588	-0.03743	0.050548
LOC101929	-0.031986541		0.003232726	0.016466	0.032565	-0.10549	-0.09967
LOC101929	-0.035339779		-0.002774483	-0.07637	0.000971	0.021173	0.10849
LOC101929	-0.030181094		0.004926079	-0.07721	0.009977	-0.02389	0.000786
LOC101929	-0.048395197		-0.017827014	0.034974	0.022732	-0.03282	-0.04558
LOC101929	-0.021953845		-0.058779098	-0.01965	-0.01344	0.050267	-0.10151
LOC101929	-0.035834093		-0.062703054	-0.03081	0.004987	0.005612	0.027849
LOC101929	-0.043211744		0.025375221	0.151664	0.003229	0.022792	-0.03187
LOC101929	-0.038063937		-0.085311634	-0.01412	0.007103	0.005256	-0.08644
LOC101929	-0.060462868		-0.034690892	-0.0327	-0.02324	0.070274	-0.03274
LOC101929	-0.038033103		-0.075239047	0.023335	-0.00509	0.026465	0.021302

LOC10192!	-0.055153145	-0.054616771	0.041789	0.010386	-0.02628	-0.12614
LOC10192!	-0.032550508	-0.067351664	-0.00184	0.013353	-0.05687	-0.13656
LOC10192!	-0.041588635	-0.032757113	0.033268	-0.00177	0.048801	0.089912
LOC10192!	-0.031605425	-0.04988218	-0.15032	0.001993	-0.03629	-0.0311
LOC10192!	-0.043474181	-0.021955322	-0.09265	-0.02158	0.072862	-0.02075
LOC10192!	-0.008231982	0.021995855	0.263207	0.020251	-0.06257	-0.36834
LOC10192!	-0.028437619	-0.003590966	-0.0343	0.001803	0.007771	-0.07728
LOC10192!	-0.029961699	-0.063047417	0.091664	-0.01924	0.075381	0.029319
LOC10192!	-0.023708378	-0.014448691	-0.01032	0.011526	-0.07484	-0.13757
LOC10192!	-0.044831304	-0.043727911	-0.1118	-0.00252	0.03233	0.017676
LOC10192!	-0.037193849	-0.088079895	0.116457	0.01625	-0.04434	-0.10462
LOC10192!	-0.008670853	-0.010936055	0.152712	-0.0004	0.025008	-0.0524
LOC10192!	-0.046428313	-0.108676395	-0.05827	0.000163	0.014624	0.210293
LOC10192!	-0.037475816	-0.015022928	-0.05187	-0.03147	0.112891	-0.1251
LOC10192!	-0.028339763	-0.018563191	-0.02529	0.017705	-0.02905	0.012547
LOC10192!	-0.034333846	0.009946258	-0.13391	0.01646	-0.02986	-0.06537
LOC10192!	-0.044380076	-0.055581781	0.06054	0.014821	-0.11968	-0.09979
LOC10192!	-0.047102552	-0.02489984	0.09857	-0.00622	0.0542	-0.10093
LOC10192!	-0.052055079	-0.035190897	-0.05202	-0.00522	0.022627	-0.01624
LOC10192!	-0.039496058	0.001586281	-0.16182	0.000575	0.024087	0.028151
LOC10192!	-0.043839247	0.046258835	0.16766	0.001337	0.019665	-0.12442
LOC10192!	-0.055611115	-0.063570727	-0.1034	0.017305	-0.0782	-0.08421
LOC10192!	-0.034661043	-0.028019752	-0.16298	0.00574	-0.0162	0.102177
LOC10192!	0.016469696	-0.03144264	-0.0738	-0.0134	-0.00377	-0.13569
LOC10192!	-0.026570059	-0.051833582	-0.01069	-0.00162	-0.05192	-0.04903
LOC10192!	-0.030437447	-0.055574647	-0.13453	0.007437	0.004044	0.022064
LOC10192!	-0.033260324	-0.070336035	0.052066	0.006125	-0.00664	-0.07964
LOC10192!	-0.058900233	-0.046949897	0.062571	-0.00207	0.000344	0.006214
LOC10192!	-0.049340596	0.024955779	-0.01571	0.022675	-0.06211	0.10497
LOC10192!	-0.046794947	-0.036976689	-0.01062	0.009709	0.000519	0.096304
LOC10192!	-0.03455819	0.000486742	0.112451	0.022972	-0.05329	-0.1058
LOC10192!	-0.077713754	-0.007515597	0.100203	-0.02601	0.067893	0.025996
LOC10192!	-0.035380132	0.000314347	0.138023	0.032801	-0.06892	-0.02223
LOC10192!	0.024487055	-0.048187122	0.082958	-0.02616	0.038264	0.233568
LOC10192!	-0.046612232	-0.052186841	0.057284	0.012839	0.00153	-0.05648
LOC10192!	-0.053895918	-0.023963427	0.172435	-0.0431	0.119663	-0.08633
LOC10192!	-0.042602915	0.003471778	-0.16965	0.038706	-0.09302	-0.04818
LOC10192!	-0.047134633	-0.006676365	0.004438	0.009126	0.008967	-0.08425
LOC10192!	-0.028878791	-0.03175453	0.017243	0.008646	-0.01604	-0.04075
LOC10192!	-0.03431178	-0.021608588	-0.11177	0.046546	-0.11761	0.026066
LOC10192!	-0.020638438	-0.052325216	-0.12312	-0.06605	0.20362	-0.03085
LOC10192!	-0.025620703	-0.017321293	0.006546	0.020859	-0.07979	-0.05078
LOC10192!	0.002853307	-0.059349576	-0.06	-0.01572	-0.08841	-0.11188
LOC10192!	-0.030633414	-0.035749564	0.024582	0.029225	-0.07804	-0.0523
LOC10192!	-0.044163124	0.000408231	-0.26156	0.017118	-0.03176	0.034814
LOC10192!	-0.018656243	-0.068151111	-0.03408	0.019418	-0.04615	-0.02902
LOC10192!	-0.03570756	-0.071221029	-0.00363	0.009239	-0.00342	-0.05003
LOC10192!	-0.040796675	-0.087671353	-0.04713	-0.00143	0.033614	-0.00687
LOC10192!	-0.04633988	-0.058876444	0.108528	0.064063	-0.1499	-0.01744
LOC10192!	-0.03959599	-0.051513623	0.015184	-0.01043	0.044299	-0.04192
LOC10192!	-0.024992157	-0.024428496	-0.01036	-0.01385	-0.01689	-0.08462

LOC10192!	-0.033798419	-0.078434747	-0.03171	0.005474	-0.02931	-0.07806
LOC10192!	-0.059192813	-0.080867106	-0.03386	0.004362	-0.01089	-0.16845
LOC10192!	-0.046534831	0.024241391	0.133172	-0.03859	0.133768	0.017319
LOC10192!	-0.06606191	-0.027658792	0.049431	-0.01426	-0.00286	0.013926
LOC10192!	-0.030749027	-0.030190238	-0.11806	0.032599	-0.0926	0.001303
LOC10192!	-0.027499936	-0.06578683	-0.03582	0.014698	-0.05445	-0.05813
LOC10192!	-0.034641568	0.023039179	-0.12802	-0.00357	0.035772	-0.01176
LOC10192!	-0.028578705	-0.023358408	0.074791	0.012989	-0.08565	-0.10932
LOC10192!	-0.040504125	-0.068994569	0.061765	-0.00077	0.040092	0.028868
LOC10192!	-0.057799998	0.00024651	0.133813	0.024933	-0.04313	-0.0306
LOC10192!	-0.029102455	-0.037960017	0.0612	-0.00871	-0.04622	-0.03943
LOC10192!	-0.038410193	-0.050376039	-0.05784	0.0116	-0.01636	-0.07599
LOC10192!	-0.065717009	-0.030884719	0.035201	0.003186	-0.01605	-0.03134
LOC10192!	-0.026302927	-0.024641274	0.01905	-0.01626	-0.00705	-0.08821
LOC10192!	-0.057461527	-0.009520167	-0.02807	0.017882	-0.0438	0.050104
LOC10192!	-0.039943878	-0.054144406	0.027995	0.027703	-0.07791	-0.11737
LOC10192!	-0.034333756	-0.055616502	-0.05774	0.024778	-0.08436	0.028355
LOC10192!	-0.031379364	-0.034194029	-0.02365	0.018537	-0.05123	-0.01646
LOC10192!	-0.05544261	-0.093620488	-0.00939	-0.02513	0.093045	-0.10478
LOC10192!	-0.032775068	-0.066712432	0.03358	0.001083	-0.03051	-0.10323
LOC10192!	-0.030473607	-0.047458366	-0.03521	0.00307	-0.07151	-0.0315
LOC10192!	-0.030768141	-0.004992296	-0.05691	-0.00542	0.029935	0.028718
LOC10192!	-0.03951657	-0.012481622	-0.0033	0.049754	-0.13736	-0.12979
LOC10192!	-0.028614812	-0.018695387	-0.03328	0.001757	-0.059	-0.12811
LOC10192!	-0.036243709	-0.062217747	-0.00331	0.004006	-0.02862	-0.12875
LOC10192!	-0.029872311	-0.061762404	-0.089	0.019972	-0.04953	-0.02879
LOC10192!	-0.035508646	-0.031598789	0.097028	0.017547	-0.0348	-0.08838
LOC10192!	-0.029484986	-0.053310287	-0.00147	0.016314	-0.03377	0.001959
LOC10192!	-0.048920302	-0.06870514	0.065195	0.011091	-0.00176	-0.0821
LOC10192!	-0.058756337	-0.043447521	-0.00729	0.034769	-0.10553	-0.04504
LOC10192!	-0.060156396	-0.091558809	0.122377	-0.01562	0.044779	-0.03662
LOC10192!	-0.032720764	-0.037109592	-0.00628	0.026121	-0.05803	0.071084
LOC10192!	-0.028385518	-0.055547624	-0.01029	0.026894	-0.0648	-0.14394
LOC10192!	-0.048434811	-0.064483726	-0.06955	0.01759	-0.01437	0.092027
LOC10192!	-0.026292868	-0.007008841	-0.00085	-0.05863	0.184997	-0.17055
LOC10192!	-0.030639878	-0.080630207	0.043249	0.031021	-0.10437	-0.1411
LOC10192!	-0.026113849	-0.006628012	0.088773	0.051107	-0.1582	-0.06125
LOC10192!	-0.031620944	0.022283485	0.05485	-0.02209	0.081134	-0.00957
LOC10192!	-0.074124143	-0.061986256	-0.03077	-0.01604	0.065706	0.036478
LOC10192!	-0.031930176	-0.023879051	-0.06815	-0.01781	0.011562	-0.04784
LOC10192!	-0.033153291	-0.037762169	-0.13997	0.001387	0.007356	0.0246
LOC10192!	-0.072325072	-0.050724691	-0.0409	-0.0349	0.116458	-0.01103
LOC10192!	-0.043949315	-0.054686205	0.000455	0.020903	-0.03643	0.005008
LOC10192!	-0.149758538	-0.081153295	-0.05046	0.032116	0.081821	0.017461
LOC10192!	-0.030812563	-0.070853945	-0.08193	0.044292	-0.12374	-0.03037
LOC10192!	-0.059043561	-0.015688859	0.008196	-0.03315	0.075078	0.130064
LOC10192!	-0.038636708	-0.073088712	0.058695	-0.01322	0.043521	-0.09128
LOC10192!	-0.031872253	-0.023138157	0.027738	-0.0038	0.017548	-0.02422
LOC10192!	-0.03668942	-0.031476211	0.048177	0.031562	-0.07919	-0.08865
LOC10192!	-0.039104829	-0.025556679	-0.05128	-0.00397	0.02942	0.057914
LOC10192!	-0.029089536	-0.052648164	0.060314	0.035038	-0.09375	-0.09597

LOC101929	-0.037381193	-0.04623024	-0.09547	0.00227	0.00861	0.020286
LOC101929	-0.035203541	-0.030240897	-0.09053	0.031687	-0.06766	-0.00582
LOC101929	0.005351144	0.14176251	0.315546	0.023223	-0.10241	-0.15674
LOC101929	-0.024592344	-0.031793581	0.168651	-0.01902	-0.01048	-0.22454
LOC101929	-0.033709892	-0.074768461	-0.04209	0.02616	-0.07214	-0.0638
LOC101929	-0.028794986	-0.082592863	-0.06023	0.011641	-0.04048	-0.05397
LOC101929	-0.007438603	-0.002652876	0.071553	0.019481	-0.04557	-0.0972
LOC101929	-0.081930525	-0.052837187	-0.04145	-0.03689	0.07207	-0.14708
LOC101929	-0.035659612	-0.031344888	0.016312	-0.01273	0.070483	-0.13489
LOC101929	-0.040019923	-0.090569268	-0.12411	-0.00686	0.055411	0.178571
LOC101929	-0.014824859	-0.031436789	-0.05508	0.008934	-0.05442	0.011107
LOC101929	-0.038750373	-0.035213702	-0.04709	-0.00781	0.052257	-0.05223
LOC101929	-0.028142425	-0.026050775	0.093787	-0.01463	0.053857	0.024086
LOC101929	-0.029757988	-0.108478458	0.008258	-0.0204	0.037354	0.005496
LOC101929	-0.028599405	-0.033100456	0.05487	-0.03148	0.104432	0.022161
LOC101929	-0.011977214	-0.061024805	-0.06953	-0.01576	0.030778	-0.12413
LOC101929	-0.011932571	0.16233309	0.223712	0.004928	-0.01335	-0.26545
LOC101929	-0.022546562	-0.02141411	0.024177	-0.03859	0.138118	0.12129
LOC101929	-0.050271269	-0.01136471	-0.08702	0.035203	-0.06411	0.255127
LOC101929	-0.012251253	-0.032614267	0.343144	-0.06428	0.172391	-0.08194
LOC101929	-0.02757512	-0.028011542	-0.06935	-0.02336	0.01177	-0.01202
LOC101929	-0.039821917	0.010744487	-0.14092	-0.01125	0.049459	0.066708
LOC101929	-0.028788233	-0.04783399	-0.0131	0.001425	0.01921	-0.12225
LOC101929	-0.005776402	-0.097935486	0.39463	-0.04782	0.154599	-0.13468
LOC101929	-0.022702503	-0.057403968	0.038564	0.032549	-0.08236	0.039501
LOC101929	-0.043172819	-0.08970008	0.010413	-0.00583	0.028466	-0.05674
LOC101929	-0.037435298	-0.063749129	0.021433	-0.02851	0.102055	-0.01499
LOC101929	-0.02009672	-0.025501284	0.09584	-0.02236	0.055373	-0.12596
LOC101929	0.010434302	0.227441234	0.067545	-0.01471	0.060805	-0.01603
LOC101929	-0.01881298	-0.03743773	-0.01323	-0.02559	-0.04076	-0.0568
LOC101929	-0.038355557	-0.015167839	-0.0346	-0.01344	0.071442	0.026713
LOC101929	-0.038456616	-0.055037711	-0.02663	-0.00897	0.062732	-0.08707
LOC101929	-0.03435759	-0.05839188	-0.07741	0.02375	-0.04036	-0.05492
LOC101929	-0.00942532	0.035782172	0.021929	0.01206	-0.00918	-0.18605
LOC101930	-0.00277735	-0.107059583	-0.0055	-0.01306	-0.06008	-0.11416
LOC101930	-0.064809446	-0.041621281	-0.04664	-0.00556	0.006112	-0.10939
LOC101930	-0.015513357	-0.096105675	-0.01362	-0.03704	0.102485	0.107033
LOC101930	-0.011904235	0.007434158	0.109553	-0.00203	-0.03974	-0.13767
LOC101930	-0.018430997	-0.056026318	0.086586	-0.06264	0.183185	-0.11301
LOC101930	0.018282823	0.101300707	0.176225	-0.01795	0.073191	-0.0456
LOC101930	-0.029359168	-0.08768004	0.069729	-0.01047	-0.00947	-0.05084
LOC101930	-0.040216599	-0.022241391	-0.05858	0.003501	0.015703	0.039615
LOC101930	-0.023469774	0.026795834	0.082096	-0.01849	0.063359	-0.06756
LOC102467	-0.048074158	-0.083163602	-0.03783	0.007808	0.00149	0.049142
LOC102540	-0.0631553	0.024570543	-0.06931	0.011402	-0.05133	0.095652
LOC102540	-0.035261858	-0.073987421	0.046219	0.038047	-0.10174	-0.14361
LOC102540	-0.093779558	-0.054795752	0.021401	0.000926	-0.00768	-0.00917
LOC102600	0.002132601	0.004125871	0.277101	-0.00436	0.018822	-0.28356
LOC102659	-0.026951532	-0.000202865	-0.00475	0.073511	-0.1502	0.039238
LOC102723	-0.030920697	-0.063369254	-0.10189	-0.01025	0.046831	-0.03876
LOC102723	-0.023055063	-0.029766682	-0.01881	-0.02286	0.017773	-0.02272

LOC102723	-0.060331203	-0.051376073	-0.02992	-0.02123	0.057343	0.075571
LOC102723	-0.031829463	-0.015623596	0.005857	-0.06051	0.164906	-0.06004
LOC102723	-0.046348228	-0.04613572	0.060742	-0.01262	0.057419	-0.03123
LOC102723	-0.024354967	-0.04141426	-0.11022	-0.01128	0.001116	-0.01637
LOC102723	-0.020675624	-0.032684939	-0.0161	-0.00599	0.018813	-0.05329
LOC102723	-0.034401373	-0.111633934	-0.11638	-0.01329	0.040273	-0.07071
LOC102723	-0.030000495	-0.022844814	-0.06528	0.000194	-0.00686	-0.02977
LOC102723	-0.048412724	-0.018095313	0.018698	-0.00959	0.044697	0.075893
LOC102723	-0.033729293	0.027728762	-0.03646	0.016568	-0.03893	-0.07529
LOC102723	-0.049047891	-0.020611627	-0.04561	0.002622	0.008164	-0.02342
LOC102723	-0.014985584	0.035034941	-0.18985	0.030249	-0.11089	-0.13355
LOC102723	-0.040171788	-0.047877218	0.015932	-0.00854	0.044143	0.104428
LOC102723	-0.03959722	-0.061569907	-0.02186	-0.00122	0.021841	0.098459
LOC102723	-0.030449399	-0.012332203	0.051941	0.038133	-0.10244	-0.05147
LOC102723	-0.070956797	-0.006593872	0.095912	-0.03982	0.090778	0.044347
LOC102723	-0.046813441	-0.020663311	0.017046	0.010856	-0.00176	0.027984
LOC102723	-0.007000035	-0.003346984	-0.0169	-0.00553	-0.0212	0.049704
LOC102723	-0.031305672	-0.066400393	-0.03287	-0.02411	0.091209	0.063128
LOC102723	-0.031550743	0.029149356	-0.03872	0.013353	-0.02153	0.019815
LOC102723	-0.019077006	-0.113070914	-0.23986	-0.04451	0.138	0.399337
LOC102723	-0.029823042	-0.044446417	-0.03252	-0.02755	0.040498	0.099005
LOC102723	-0.024159935	0.015026658	-0.07393	0.076084	-0.1977	-0.06041
LOC102723	-0.02151152	0.013734621	0.04043	0.110188	-0.29827	-0.03884
LOC102723	-0.013108324	-0.112762315	-0.14418	-0.0617	0.188713	0.706794
LOC102723	-0.013540506	-0.090241563	0.017914	-0.00114	0.029223	-0.15882
LOC102723	-0.026697697	-0.037422911	-0.07721	-0.00015	-0.019	0.075582
LOC102723	-0.035768204	0.007115335	0.009056	-0.03095	0.105666	0.035666
LOC102724	-0.027625618	-0.0846496	0.139648	-0.00687	0.005627	0.015831
LOC102724	0.007907057	-0.069341445	0.032438	0.021447	-0.10815	-0.16755
LOC102724	-0.038708714	-0.052018478	-0.04526	0.011933	-0.00539	0.038208
LOC102724	-0.036523671	-0.07446553	-0.01358	-0.00533	0.045031	0.019091
LOC102724	-0.016943854	-0.057278782	0.116348	0.017738	-0.12677	-0.04659
LOC102724	-0.028916219	-0.018512093	-0.09072	0.002188	0.021989	0.190924
LOC102724	-0.017680325	-0.047990887	-0.10906	-0.00652	0.032278	-0.16416
LOC102724	-0.021655311	-0.05858942	-0.06202	0.014268	-0.07066	0.009255
LOC102724	-0.053209128	-0.004737418	-0.04961	-0.00745	0.035466	0.022038
LOC102724	-0.020995665	-0.026944473	0.114325	-0.01138	0.046771	-0.16777
LOC102724	-0.044801649	-0.080018913	0.013165	-0.01756	0.003788	-0.11059
LOC102724	-0.023443535	0.008940702	-0.02225	-0.02904	0.105068	0.129195
LOC102724	-0.030554566	-0.051056342	-0.11268	0.036263	-0.08549	0.13608
LOC102724	0.007457991	0.016900829	0.176241	-0.02399	0.072268	-0.24204
LOC102724	-0.034065708	-0.063405457	0.019821	-0.0227	0.05091	-0.01584
LOC102724	-0.067258453	-0.035339341	0.01293	-0.18842	0.520635	-0.0176
LOC102724	-0.042142659	-0.054265902	-0.05779	-0.0003	0.026528	-0.09658
LOC102724	-0.053384922	-0.05106444	-0.06528	-0.02128	0.107495	0.146568
LOC102724	-0.034349058	-0.059478435	-0.06275	0.017419	-0.03186	-0.00729
LOC102724	-0.054440963	-0.030681239	0.046134	-0.01107	0.037547	-0.03716
LOC102724	0.009957687	-0.019462508	0.013183	0.007848	-0.10469	-0.15178
LOC102724	-0.022046663	0.064950482	-0.06857	0.094739	-0.2336	0.05493
LOC102724	-0.005607161	0.026819976	0.344935	-0.0853	0.252242	-0.19447
LOC102724	-0.041364629	-0.0259928	-0.20807	-0.03095	0.122739	-0.04132

LOC10272⁴	-0.037925186	-0.055361989	-0.01821	0.045219	-0.14928	-0.08784
LOC10272⁴	-0.023727788	-0.008112624	0.004097	-0.01827	0.040578	-0.0689
LOC10272⁴	-0.015670051	0.004102003	0.073869	0.015426	-0.036	-0.05409
LOC10272⁴	-0.004283965	-0.005265171	0.080968	0.086285	-0.24812	-0.01367
LOC10272⁴	-0.029573979	-0.107798826	0.012073	0.002737	0.012203	-0.06825
LOC10272⁴	-0.00167527	-0.062293238	-0.02855	-0.07957	0.232181	0.338524
LOC10272⁴	-0.039551235	-0.032911047	-0.07702	0.0289	-0.06152	0.086534
LOC10272⁴	0.019944484	-0.019307118	-0.02014	-0.04334	0.055669	-0.16589
LOC10272⁴	-0.024765521	-0.069385174	-0.02286	0.030822	-0.0732	0.02181
LOC10272⁴	-0.02831373	-0.037926359	-0.06497	-0.00493	-0.0216	-0.07059
LOC10272⁴	-0.037915303	-0.065009352	0.024968	-0.00299	0.023101	-0.00923
LOC10272⁴	-0.033562103	-0.011777875	0.038454	-0.01421	0.044612	-0.05369
LOC10272⁴	-0.05150515	-0.098834966	-0.04494	0.001469	0.00266	0.082927
LOC10272⁴	-0.024526367	-0.035123674	-0.11201	0.012667	-0.0103	0.131144
LOC10272⁴	0.008439391	-0.112209592	-0.06121	-0.00462	-0.04361	0.146612
LOC10272⁴	-0.031356708	-0.039145305	0.032109	-0.00531	0.047925	0.215647
LOC10272⁵	-0.008897421	-0.030780613	0.124961	-0.04222	0.052698	-0.09921
LOC10272⁵	-0.013360222	-0.085260297	0.030013	-0.02034	-0.01186	-0.03144
LOC10272⁵	-0.059998768	-0.028491938	0.100023	0.011751	-0.03006	-0.042
LOC10272⁵	-0.042009254	-0.005527634	-0.04027	-0.006	0.050768	0.070869
LOC10272⁵	-0.04101389	-0.103777653	0.030551	0.002054	0.027186	0.038484
LOC10272⁵	-0.033932075	-0.089442764	-0.05825	0.036982	-0.10717	0.142968
LOC10272⁵	-0.022329405	-0.002037433	0.116696	-0.01377	0.019634	-0.11878
LOC10272⁵	-0.037017003	-0.061501347	-0.01803	0.027909	-0.04931	0.001302
LOC10272⁵	-0.033633696	-0.003312442	-0.00095	0.022676	-0.05047	-0.09172
LOC10272⁵	-0.024327346	-0.073780046	-0.12246	-0.00147	-0.05234	-0.02044
LOC10334⁴	-0.006537276	0.031962755	0.088469	0.005099	0.00088	-0.08617
LOC14293⁷	-0.013264367	-0.059503717	-0.07839	0.027049	-0.06155	0.166748
LOC14547⁴	-0.012861769	-0.045636172	-0.13047	0.016114	-0.0504	0.088557
LOC14567⁸	-0.022180754	-0.018952898	-0.05518	0.005202	-0.03189	-0.09006
LOC14569⁴	-0.02090583	-0.035621877	-0.10534	0.002957	0.004314	0.003686
LOC14583⁷	-0.074426372	-0.097914339	-0.00659	0.015555	-0.02023	0.132504
LOC14584⁵	-0.018755318	-0.036962327	-0.00048	0.000349	-0.02068	-0.07018
LOC14594⁵	-0.029246697	-0.040067661	-0.0501	0.009852	-0.06586	-0.09569
LOC14651³	-0.039376168	-0.059822777	-0.15604	-0.01203	0.046285	-0.05368
LOC14679⁵	-0.043447359	-0.074034324	-0.07213	0.02259	-0.04094	-0.06982
LOC14688⁰	-0.027803066	-0.100947684	-0.05621	-0.01422	0.054692	0.069849
LOC14700⁴	-0.039023032	-0.086144817	0.019166	0.018787	-0.0375	0.123082
LOC14779¹	-0.026279677	-0.057252952	-0.03737	-0.0105	0.007957	-0.04833
LOC14841³	0.038449612	0.044486016	-0.00159	0.020581	-0.12096	-0.15681
LOC14869⁰	-0.027501011	-0.074962997	-0.01348	0.001497	-0.00445	0.068409
LOC14870⁹	-0.043435702	-0.000825739	0.007496	0.020341	-0.03938	-0.03995
LOC14935¹	-0.096647991	-0.033033465	-0.04228	-0.00443	0.034176	-0.02555
LOC14937³	-0.027859428	-0.062421806	-0.01043	0.028209	-0.09303	-0.03987
LOC14970³	-0.034449032	-0.095481247	0.029755	0.006418	-0.01065	-0.01592
LOC15000⁵	-0.071776845	0.00706391	-0.06877	0.03702	-0.09379	0.014248
LOC15005¹	-0.024705528	-0.01420168	0.021914	0.082014	-0.23967	-0.01529
LOC15112¹	-0.03318011	-0.042340632	-0.02441	0.00098	0.024814	0.076137
LOC15117⁴	-0.020571089	0.086229924	-0.06275	-0.01651	0.037858	-0.03247
LOC15148⁴	-0.031483171	-0.024200988	-0.05812	0.050171	-0.13295	-0.04526
LOC15165⁷	-0.009639485	0.063361711	0.164617	-0.00903	-0.0045	-0.27894

LOC15176(	-0.048011996		-0.045888576	-0.07406	-0.00681	0.048128	-0.08353
LOC15222!	-0.026090625		-0.027222608	-0.03686	-0.0171	0.065057	0.124875
LOC15227‹	-0.069973091		-0.02716652	-0.2161	-0.0226	0.06896	0.029187
LOC15257{	-0.047976759		-0.068230262	-0.10434	-0.00782	0.046511	0.059839
LOC15258(	-0.051091239		-0.108340774	0.041836	0.003519	-0.01297	-0.08464
LOC15354(	0.003805044		-0.070470819	-0.10845	-0.03252	0.102017	0.327719
LOC15357'	-0.001414105		0.035635912	-0.13067	-0.02813	0.120269	0.316769
LOC15368:	-0.004454664		0.102477928	0.122882	-0.02343	0.08418	-0.11901
LOC15368‹	0.01611906		-0.024025497	0.095015	-0.00396	0.027246	0.026784
LOC15391(	-0.027058297		-0.060179013	-0.00918	-0.00499	-0.03443	-0.18668
LOC15476]	0.004070801		0.038227577	0.311411	0.058845	-0.19968	-0.37431
LOC15487:	-0.024471525		-0.052114248	0.043963	0.000716	0.002249	-0.13209
LOC15506(	-0.008935511		-0.167521032	0.204203	0.003731	-0.08404	-0.16462
LOC15727:	-0.05377946		-0.088014701	0.065637	0.023095	-0.06032	0.007851
LOC15756:	-0.017501392		-0.054214218	0.17275	-0.03883	0.034447	-0.22623
LOC15774(	-0.036348742		-0.039747022	-0.07902	0.010288	-0.02434	0.045133
LOC15786(	-0.028290912		-0.074796089	-0.05427	0.004734	0.008434	0.028239
LOC15793]	-0.040795583		-0.114026223	0.074871	0.000193	0.019903	0.030605
LOC15840:	-0.004168013		0.079919406	-0.13086	-0.07473	0.226371	0.147486
LOC15843‹	-0.058061281		-0.068240969	0.250861	-0.01196	0.0445	-0.06895
LOC15896(	0.040526205		-0.017597608	0.001814	0.015099	-0.06481	-0.01556
LOC1720	-0.074281537		-0.122293746	-0.09324	-0.04725	0.150808	0.061907
LOC20060!	-0.035405337		-0.051991556	0.248516	-0.00255	0.02582	-0.15389
LOC20077:	-0.002901761		-0.069474665	-0.10849	-0.07323	0.218476	0.367641
LOC20083(	-0.055581211		-0.035062405	0.019929	0.001675	0.004297	-0.10767
LOC20202!	0.017997475		-0.02284976	0.279983	-0.11578	0.298205	-0.47515
LOC20218]	0.003489027		0.001811702	-0.01147	-0.06803	0.207317	-0.10315
LOC21968{	-0.028653733		0.023997661	0.069286	-0.00305	-0.01481	-0.11364
LOC21969(	-0.032416459		-0.063527581	-0.02307	-0.00887	0.006907	-0.09834
LOC22007'	-0.040759404		-0.024415026	-0.08464	-0.00142	-0.03745	-0.0032
LOC22112:	-0.028407227		-0.031634388	-0.0769	0.010835	-0.0905	-0.04589
LOC22127:	-0.024010958		-0.021517874	-0.02738	-0.00783	0.039398	0.00435
LOC22181‹	-0.026228976		-0.051157454	0.075933	0.002907	0.003915	0.036831
LOC22194(	-0.023838443		-0.016302946	-0.00174	-0.00452	-0.01716	-0.05572
LOC25304‹	-0.028104881		-0.046645893	-0.00946	0.009249	-0.02764	-0.11349
LOC25357:	-0.076465645		-0.066714796	-0.01842	-0.03183	0.078829	0.034896
LOC25380!	-0.04673208		-0.006013313	0.024457	-0.02727	0.109576	0.0281
LOC25402{	-0.028436274		-0.059807617	-0.09195	-0.01236	0.034137	0.077881
LOC25405'	-0.02822081		-0.06140305	0.04499	0.002438	-0.03916	-0.06917
LOC25517'	-0.045901927		-0.106377134	-0.01287	0.028342	-0.06517	-0.10974
LOC25565‹	-0.031201812		-0.036782102	-0.01151	0.004131	-0.0656	-0.05666
LOC25715:	-0.0560599		0.036921276	0.041546	-0.00747	0.044139	-0.05827
LOC25739(	-0.023211175		0.008560954	0.210983	0.032702	-0.08018	-0.19239
LOC28066!	-0.023374905		-0.047523022	0.077689	-0.00653	0.02082	-0.17704
LOC28303{	-0.051371695		-0.107548362	0.038089	-0.01131	0.028669	0.003015
LOC28304!	-0.030527236		-0.0325041	-0.02006	-0.03147	0.022687	-0.05396
LOC28307(	0.021985274		-0.011909599	-0.16572	-0.04165	0.126771	-0.17779
LOC28307!	-0.079163321		-0.131498842	-0.05748	-0.04551	0.141038	0.070489
LOC28311:	-0.104670901		-0.051850694	0.038528	-0.02993	0.107858	0.057384
LOC28317'	-0.033983283		-0.021760812	-0.0542	-0.02057	-0.0079	-0.10425
LOC28319‹	-0.028065371		-0.071602979	0.005086	-0.05516	0.091146	-0.11814

LOC28321	-0.047726575	-0.061957824	-0.09906	-0.00218	0.027461	0.050912
LOC28333	-0.020774325	-0.064806742	0.00597	-0.00055	-0.07792	-0.1562
LOC28335	-0.06246039	-0.030810837	-0.02624	0.030677	-0.06426	-0.03053
LOC28335	0.009624374	0.115392432	0.074269	-0.01673	0.0841	-0.0216
LOC28338	-0.031260476	-0.045599306	-0.059	-0.00592	-0.00454	-0.0627
LOC28343	-0.034541781	-0.022942648	0.02724	0.035553	-0.09176	-0.08953
LOC28345	-0.036613249	-0.072935136	0.00968	-0.00775	0.037516	-0.05259
LOC28348	-0.034497958	-0.028189784	-0.08666	0.033571	-0.09198	0.011111
LOC28348	-0.073221961	-0.085192761	-0.1216	0.008914	-0.0099	-0.01414
LOC28348	-0.025626982	-0.022324836	-0.01882	0.171717	-0.32071	-0.01398
LOC28351	-0.057562078	-0.011889462	-0.0033	-0.00977	0.034685	0.086278
LOC28358	-0.021145864	-0.098774463	0.048109	-0.00055	-0.03254	-0.0725
LOC28358	-0.008360107	0.001460666	-0.02304	0.017334	-0.05093	-0.09669
LOC28366	-0.067202063	-0.079418985	0.022472	-0.0276	0.077503	-0.05467
LOC28367	-0.048018434	-0.024920695	-0.08756	0.045016	-0.14098	0.04027
LOC28368	-0.060122599	0.026461005	-0.1411	0.031441	-0.06303	0.03569
LOC28369	-0.042981699	-0.06394882	0.022977	0.014938	-0.02394	-0.06444
LOC28369	-0.039351636	-0.044526642	0.020665	-0.03995	0.101791	0.11463
LOC28373	-0.059964804	-0.040435647	0.172807	-0.00961	0.039302	-0.11117
LOC28373	-0.040882707	0.011217683	-0.10794	-0.0057	0.049829	0.121546
LOC28374	-0.047031446	-0.059091922	0.031521	0.02287	-0.05951	0.059207
LOC28378	-0.019187238	0.039325022	0.082091	-0.04667	0.067485	-0.08631
LOC28385	-0.027513406	-0.018208289	0.090957	0.015048	-0.02998	-0.12453
LOC28386	-0.017082219	0.072731386	0.119945	-0.03344	0.112182	0.065895
LOC28388	-0.033287454	-0.036875943	0.037032	-0.00382	-0.04688	-0.06168
LOC28391	-0.027505016	-0.092884138	0.016991	-0.0067	-0.06155	-0.15927
LOC28401	-0.021842358	0.011243699	0.047566	0.022264	-0.02864	0.172209
LOC28402	-0.003462762	-0.006276117	0.127188	-0.05477	0.028016	-0.11401
LOC28408	-0.041070067	-0.069670558	-0.01955	0.017046	-0.03382	-0.11375
LOC28421	-0.019794718	-0.051316534	-0.09071	0.003995	-0.02258	-0.09253
LOC28424	-0.037283026	-0.108060595	-0.04578	-0.00266	0.026886	0.002031
LOC28424	-0.026624917	-0.034271641	-0.03665	0.012576	-0.0241	0.170733
LOC28426	-0.045319892	0.00770237	-0.06359	0.012801	-0.01748	-0.02363
LOC28429	-0.031082678	-0.032470526	0.051311	0.011816	-0.02019	-0.0208
LOC28437	-0.027857427	0.011270539	0.046611	-0.0077	-0.00305	-0.31256
LOC28437	-0.027947893	-0.047882728	-0.06059	-0.00561	0.021163	-0.12236
LOC28439	-0.020265613	-0.015403552	-0.02027	-0.01315	-0.05633	-0.044
LOC28451	-0.007445151	0.003303664	0.059245	-0.00067	0.024841	-0.02366
LOC28456	-0.041559429	-0.059424527	0.016418	0.049926	-0.12369	-0.09882
LOC28457	-0.031325651	-0.05107689	-0.07571	0.017461	-0.04825	-0.05992
LOC28460	-0.029412024	-0.113039412	-0.07307	-0.00485	0.003608	-0.05619
LOC28463	-0.046285712	-0.059454123	-0.04276	-0.00911	0.050039	0.069156
LOC28464	-0.031289636	-0.064293407	-0.01424	0.00035	0.009927	-0.09274
LOC28466	-0.0699783	-0.07203839	-0.04849	-0.01389	0.031308	0.104147
LOC28466	-0.057030791	-0.064932571	-0.11919	0.013621	-0.01159	0.018562
LOC28478	-0.035749671	-0.091056376	0.01428	0.001378	0.02036	-0.03023
LOC28479	-0.032453508	-0.073044844	-0.04849	0.014853	-0.0618	-0.08162
LOC28482	-0.046180504	-0.012393092	-0.02672	0.003029	0.016866	-0.00557
LOC28483	0.001393799	-0.09353167	-0.16589	-0.10127	0.296969	0.66953
LOC28486	-0.032674708	-0.056775237	0.035198	0.023793	-0.10501	-0.07491
LOC28488	0.001690624	-0.012008539	0.031933	-0.00759	0.031946	-0.01718

LOC284898	-0.030864262	-0.017686056	0.010189	0.031916	-0.08806	0.024872
LOC284926	-0.009811937	0.025286124	-0.03196	-0.02463	0.08664	0.083262
LOC284930	-0.035819226	0.032773181	0.039746	0.012689	-0.01169	-0.10479
LOC284933	-0.030702047	-0.040064706	-0.00335	0.058358	-0.15612	-0.06461
LOC284950	-0.078730831	-0.082133326	-0.02833	-0.03448	0.108385	-0.02918
LOC285000	-0.053127227	-0.038386969	-0.08459	0.004281	0.002228	0.001228
LOC285043	-0.041503365	-0.035896387	-0.0985	-0.0112	0.052651	0.235562
LOC285095	-0.041145815	-0.012134702	-0.05407	0.010658	-0.00289	0.019716
LOC285147	-0.011117872	0.005225353	-0.04447	-0.05388	0.139592	0.107706
LOC285178	-0.008814078	0.116967845	0.140604	0.01396	-0.03637	-0.25871
LOC285181	-0.016013752	-0.09195792	-0.18826	-0.01868	0.079988	0.570643
LOC285191	-0.025381637	-0.083375474	-0.12539	0.001706	-0.04515	0.001721
LOC285300	-0.031502743	-0.078093138	0.069987	0.00658	0.00153	-0.02623
LOC285419	-0.06888806	-0.056330747	-0.047	0.007822	-0.03943	-0.04052
LOC285422	-0.042723032	-0.015817248	-0.04509	-0.00625	0.032737	0.038155
LOC285423	-0.047996119	-0.041231165	-0.01896	0.054802	-0.14312	-0.01675
LOC285500	-0.060538056	-0.013443046	0.003506	-0.04403	0.122969	-0.05096
LOC285556	-0.068778215	-0.09427484	0.033986	0.010155	-0.05494	0.05937
LOC285593	-0.056792378	-0.108656232	0.097401	0.021572	-0.058	-0.01092
LOC285627	-0.035192564	-0.049473683	-0.01481	0.01765	-0.02414	0.065263
LOC285638	-0.034671303	0.006164734	0.064007	-0.00036	-0.00496	-0.16506
LOC285692	-0.06587187	-0.03994272	-0.07338	-0.00044	0.035196	-0.00911
LOC285696	-0.028028029	-0.04766434	0.129231	0.032838	-0.07966	-0.13703
LOC285740	-0.065935726	-0.083196244	-0.07308	0.040409	-0.08404	-0.03738
LOC285766	-0.04147611	-0.041224746	0.01077	0.011559	-0.00224	-0.01362
LOC285768	-0.037142303	0.017949325	0.059599	0.014535	-0.02407	-0.11689
LOC285778	-0.030768998	-0.023199972	-0.05377	0.027395	-0.06687	0.02404
LOC285812	0.008513279	0.218807895	-0.15241	-0.0211	0.033474	0.000512
LOC285819	-0.029291348	-0.058105205	0.093993	0.017529	-0.03399	-0.05022
LOC285847	-0.059217883	0.001496981	0.152704	-0.01988	0.009856	-0.19909
LOC285857	-0.038191633	-0.03645053	-0.03425	-0.00838	0.044289	-0.08042
LOC285889	-0.055626909	-0.008660034	0.027239	0.00907	-0.01326	0.056797
LOC285957	0.004269542	0.09248441	0.077426	0.069017	-0.16317	0.107524
LOC286009	-0.023765901	0.00569113	0.072276	-0.00509	-0.04569	-0.1683
LOC286052	0.011256425	-0.063749134	-0.02916	-0.02489	0.059511	-0.02863
LOC286058	-0.022335247	-0.043620659	-0.17607	-0.00531	0.010187	0.008686
LOC286059	-0.04030671	-0.089452531	-0.05769	0.014072	-0.02895	-0.0189
LOC286068	-0.037028291	-0.007329635	0.105387	0.0018	0.001935	-0.05352
LOC286071	-0.02463856	0.072907024	-0.00796	-0.00018	0.023289	-0.24959
LOC286083	-0.03720244	-0.036949605	-0.16174	0.0245	-0.04482	-0.02178
LOC286087	-0.004870417	0.280430053	-0.05476	-0.05743	0.179373	-0.09849
LOC286114	-0.047019356	-0.083192952	-0.06739	0.000414	0.033236	-0.04623
LOC286121	-0.036307587	0.025600043	-0.05436	0.024369	-0.06115	-0.14642
LOC286161	-0.007185603	0.004133621	0.162516	-0.0308	0.118162	-0.11817
LOC286177	-0.04178727	-0.054934082	-0.09978	-0.00875	0.025184	-0.10474
LOC286178	-0.03542879	-0.023616456	0.010944	0.011422	-0.0086	-0.00101
LOC286189	-0.027866613	-0.085499344	0.041593	-0.00906	-0.00846	-0.13312
LOC286190	-0.09153129	-0.039486265	-0.03826	-0.02234	0.044387	0.071408
LOC286238	-0.036542562	-0.037265005	-0.09796	-0.00238	0.034563	-0.195
LOC286272	-0.006511565	0.02359506	0.183914	-0.02028	0.071927	-0.22709
LOC286359	-0.053372314	-0.118086158	-0.07405	-0.00099	-0.02056	0.096211

LOC28636'	-0.01091574	-0.143072746	-0.14771	-0.01622	0.060176	-0.20867
LOC28637(	-0.056909204	-0.015173439	0.040309	0.027496	-0.06524	0.116972
LOC28638:	-0.036845181	-0.049613328	0.015719	0.021413	-0.04391	-0.11108
LOC28643'	0.013548887	-0.016938728	0.070321	-0.04943	0.149086	-0.19857
LOC33862(	-0.008630129	0.036233064	0.274592	-0.03873	0.122123	-0.20753
LOC33866'	-0.044438216	-0.005659357	-0.06087	-0.01095	0.071932	-0.03597
LOC33869(	-0.047318971	-0.061457479	-0.04622	0.007537	0.000362	-0.05519
LOC33896:	-0.024748673	-0.031683976	-0.09335	0.001029	-0.07115	-0.02791
LOC33929(	-0.047980509	0.00721058	-0.05142	0.045944	-0.10083	0.056811
LOC33946(	-0.043106884	-0.048062218	0.065383	0.020807	-0.04242	-0.15053
LOC33953(	-0.032771595	-0.052232449	-0.11357	-0.00545	0.005409	0.020122
LOC33956(	-0.034204196	-0.085052586	-0.02659	0.021736	-0.04089	-0.04588
LOC33959:	-0.04627569	-0.054771644	0.026975	0.02203	-0.03956	0.0526
LOC33962:	-0.056829292	0.002572747	-0.0891	-0.00168	0.000476	-0.03414
LOC33966(	-0.023776132	-0.102414768	0.001273	-0.01264	-0.02867	-0.0204
LOC33968(	-0.032125708	-0.108564874	-0.07545	0.026638	-0.06196	-0.07202
LOC33980:	0.005263077	0.0587877	-0.05697	0.087045	-0.22395	0.063144
LOC33980'	-0.03486955	-0.025120418	0.03255	-0.01232	0.02127	0.009027
LOC33986:	-0.037038621	-0.022686249	-0.07182	-0.0235	0.04606	-0.11097
LOC33987(	-0.046413293	-0.123025859	-0.17087	0.012203	-0.01604	0.281258
LOC33997(	-0.04150106	-0.059630194	0.059475	0.028309	-0.05227	-0.06979
LOC33997(	-0.032234104	-0.03573108	0.027295	0.047599	-0.14066	-0.08365
LOC33998(	0.010710225	0.148451061	0.179627	-0.02665	0.03907	-0.13998
LOC34001'	-0.117466747	-0.077356513	0.037117	0.008934	0.049838	-0.05187
LOC34007(	-0.036983388	-0.064842816	-0.06316	0.004389	-0.01399	-0.06172
LOC34008(	0.014290236	0.022664598	0.082553	-0.02126	0.017589	-0.15278
LOC34009(	-0.048606879	-0.019457139	-0.06369	-0.0242	0.077209	-0.00822
LOC34010'	-0.044090988	-0.065113269	-0.08109	0.014736	-0.12465	-0.12965
LOC34017(	-0.04214479	-0.053652227	0.010691	0.012452	-0.01139	0.098269
LOC34018(	-0.047317098	-0.010228119	0.112756	-0.01741	0.028443	-0.08285
LOC34034(	-0.021648563	-0.015285175	0.023588	-0.01809	0.013852	-0.07728
LOC34035'	-0.026736867	-0.027789327	-0.0355	0.056245	-0.1441	-0.04123
LOC34058:	-0.037746305	8.31E-05	-0.05028	-0.0034	0.029084	0.051021
LOC34488'	-0.024911006	-0.046925018	-0.04032	-0.00135	-0.05572	-0.01211
LOC37444:	-0.021787909	0.203110937	0.154866	-0.0552	0.101959	-0.23867
LOC37489(	-0.036394964	-0.019658778	0.078845	0.044654	-0.11985	-0.03229
LOC37519(	-0.049267895	-0.087715136	-0.1033	-0.04085	0.131028	0.43352
LOC38845(	-0.035643773	-0.022418809	-0.0234	0.019638	-0.04081	-0.14664
LOC38869:	-0.007429919	0.1706169	0.222769	-0.0289	0.06268	-0.0904
LOC38878(	-0.021167328	-0.042160429	-0.04307	-0.0286	0.038075	0.022461
LOC38888:	-0.032183428	-0.038865716	-0.04967	0.012849	-0.0388	-0.06433
LOC38894:	-0.028341958	-0.027839752	-0.05094	0.003089	-0.00959	0.02192
LOC38919(	-0.024960039	-0.013728587	0.058861	-0.0063	-0.0139	-0.02805
LOC38924'	-0.02636202	-0.060313406	-0.03123	0.015139	-0.03567	-0.04176
LOC38933:	-0.036798253	0.015055544	-0.02355	0.004967	0.001498	-0.03608
LOC38964:	-0.027137515	-0.089333777	-0.11281	0.04124	-0.14514	-0.05248
LOC38976(	0.007161939	0.121019142	0.043153	0.006508	0.019147	-0.10676
LOC38983:	-0.035773863	-0.018719762	0.072222	-0.08075	0.24288	-0.08509
LOC38983(	0.022970521	0.071273437	0.146131	-0.00461	-0.09458	-0.10836
LOC38989(	-0.024127597	-0.005767027	-0.0234	-0.00511	0.012998	-0.09206
LOC38990(	-0.051024987	-0.013960574	0.251833	0.081703	-0.09219	-0.20591

LOC39070!	-0.022178099		-0.078222474	-0.05688	0.009616	-0.12411	-0.1581
LOC39949	0.067651661		0.048232197	0.304236	0.0097	4.62E-06	-0.37696
LOC39988	-0.035384567		-0.057092585	-0.09444	0.043652	-0.10509	0.131864
LOC39990(	-0.024569223		-0.100894228	-0.11027	-0.00402	0.022652	0.033439
LOC40004:	-0.021053339		-0.050775765	0.02643	-0.01161	0.014137	0.001875
LOC40054	-0.045122434		-0.004091004	-0.05665	-0.03236	0.110744	0.026405
LOC40054!	-0.026341403		-0.076476005	0.012159	0.013447	-0.01694	0.110908
LOC40056!	-0.039172293		-0.071975317	0.028274	0.017849	-0.03736	0.0012
LOC40059(	-0.130621381		-0.037934216	-0.02526	-0.0613	0.14514	-0.01905
LOC40062(	-0.053209597		-0.048851626	-0.01995	0.000597	-0.02643	0.023415
LOC40062:	-0.04289108		-0.027789507	-0.08018	0.024291	-0.04124	0.008756
LOC40065	-0.098402533		-0.091682915	0.071679	-0.01094	0.051311	0.051714
LOC40065!	-0.038611682		-0.080577646	0.04089	-0.01891	0.057602	-0.05018
LOC40068	-0.030923943		0.010975247	0.022859	0.028765	-0.0566	-0.09724
LOC40069:	-0.092515095		-0.043818377	-0.02137	-0.02494	0.054338	-0.01704
LOC40074!	-0.030929323		-0.0042909	0.023624	0.009539	-0.00932	0.086944
LOC40075(	-0.029680177		-0.017866747	0.067363	0.019309	-0.07723	-0.18399
LOC40076!	-0.050275305		-0.015585409	-0.05329	0.002663	0.011228	0.141699
LOC40079	-0.001421393		-0.10633864	-0.09697	0.032532	-0.00087	-0.00025
LOC40089	-0.045968543		-0.031542139	0.088777	-0.00955	0.041567	0.040365
LOC40094(	-0.053045929		-0.061306114	-0.05391	0.003147	0.001145	0.135792
LOC40095!	-0.036828687		-0.030444814	-0.07065	-0.03683	0.127933	-0.04096
LOC40096!	-0.031817264		-0.042038188	0.449993	0.006077	0.006779	-0.01953
LOC40105:	-0.039245913		-0.083460286	-0.03826	-0.02806	0.105212	0.346692
LOC40106!	-0.021252597		-0.034961572	0.265444	0.01929	-0.04519	-0.10125
LOC40109!	-0.07376933		-0.080458451	0.100034	-0.01455	0.01816	-0.02849
LOC40113	-0.090763438		-0.061592585	-0.05042	-0.04237	0.087286	-0.03924
LOC40117(	-0.041329291		-0.003190765	-0.11477	-0.01024	0.03789	0.033579
LOC40122(	-0.056116528		-0.053352403	0.323413	-0.03223	0.114088	-0.20068
LOC40131:	-0.045062326		-0.076734215	-0.11753	-0.03577	0.025056	-0.08494
LOC40132(	0.011488714		-0.034023623	-0.03149	-0.02652	0.00822	-0.02461
LOC40132	-0.032675812		0.029807571	-0.1055	0.027602	-0.06334	-0.06211
LOC40144:	-0.027594082		-0.015508379	-0.04318	-0.00622	0.033031	-0.0833
LOC40146:	-0.060144087		-0.080884505	-0.02604	0.012398	-0.01388	0.142942
LOC40191:	-0.022747964		-0.098155405	-0.01003	-0.01222	0.033373	-0.08173
LOC40216(	-0.03408705		-0.044112427	-0.03727	0.018307	-0.03247	0.059029
LOC40332:	-0.026126913		-0.037540138	0.164359	0.001477	-0.03311	-0.11126
LOC41430(	-0.054499622		-0.040560928	0.045485	-0.05125	0.122531	0.020467
LOC43991	0.009686153		-0.039151198	-0.21435	-0.06026	0.199093	0.245505
LOC43993:	-0.092678359		-0.029364231	0.119	0.006355	-0.00095	-0.11632
LOC43993!	-0.035190374		-0.014001849	0.020384	0.037023	-0.08363	-0.15753
LOC43995	-0.033507291		-0.050632296	-0.11588	-0.00255	0.012156	0.052627
LOC44002!	-0.032834067		-0.017605094	0.026042	-0.00499	-0.04247	-0.10423
LOC44011'	-0.032583819		-0.026612129	0.160014	0.016789	-0.03954	-0.01486
LOC44014!	-0.012448382		-0.088619874	-0.16915	0.020832	-0.0249	0.577266
LOC44017:	-0.03473664		-0.064797401	-0.13867	0.025776	-0.0568	-0.06276
LOC44033(	-0.027917124		-0.067099086	-0.02288	-0.0101	-0.05295	-0.11833
LOC44034(	-0.052887636		0.000584042	0.066403	-0.01625	0.03394	-0.04378
LOC44041(	-0.058647637		-0.039473908	0.034922	0.096125	-0.21311	-0.05338
LOC44043	0.050603094		-0.027561784	-0.09551	0.012429	-0.03233	-0.14649
LOC44057(	-0.033680065		-0.027005345	0.046337	0.006303	0.001591	0.01481

LOC44060	-0.027204318	-0.0231526	-0.08682	-0.014	-0.02792	-0.03033
LOC44070	-0.065090607	-0.066621489	0.082769	0.000116	-0.02272	0.005226
LOC44079	-0.038180535	-0.155406345	0.045027	########	-0.02034	0.053608
LOC44093	-0.027708723	-0.017036115	-0.01045	-0.0071	0.040931	0.01928
LOC44102	-0.047548081	-0.087659308	0.063743	0.009512	-0.00103	-0.03996
LOC44105	-0.03020073	-0.070478085	-0.1059	0.015418	-0.03155	-0.05565
LOC44108	-0.006370193	-0.099228704	-0.25946	-0.04337	0.113129	0.418651
LOC44108	-0.032954647	-0.034111158	-0.02156	0.019528	-0.03183	0.042497
LOC44112	-0.000999519	-0.066984717	-0.08254	-0.0055	-0.03598	0.016414
LOC44117	-0.04296395	0.001559675	0.039545	-0.01741	0.039513	-0.04251
LOC44117	-0.022800027	0.008033111	-0.04446	-0.0204	0.021656	-0.1043
LOC44120	-0.070313117	-0.005726844	0.002767	-0.03587	0.045222	0.096314
LOC44124	-0.036471303	-0.069602893	0.154092	-0.01468	0.076261	-0.07032
LOC44145	-0.021939197	-0.017672457	0.006249	-0.01245	-0.00991	-0.0397
LOC44146	0.021172721	0.003714561	0.057538	0.035556	-0.09451	-0.18528
LOC44152	-0.023107964	0.01954084	0.15955	0.024814	-0.06774	-0.2082
LOC44160	-0.038205206	-0.042544893	-0.06616	0.002455	-0.01072	-0.02251
LOC44166	-0.032490063	-0.046639886	0.045207	-0.0116	0.003884	-0.03695
LOC49415	-0.012202625	0.013434029	-0.01359	0.060219	-0.14584	0.121174
LOC49725	-0.07017387	-0.0364741	-0.09343	0.022851	-0.06445	-0.07843
LOC51145	-0.048488094	-0.007260787	0.077902	-0.01564	0.069073	0.01797
LOC54147	-0.023338752	-0.057313464	-0.15288	0.003929	-0.00828	-0.19525
LOC54944	-0.030949187	-0.075582593	-0.0685	0.020305	-0.03996	-0.06971
LOC55310	-0.030323611	0.003410963	0.055697	0.003535	-0.03749	-0.06598
LOC55417	-0.030947143	-0.037681837	-0.13389	0.015151	-0.05168	0.014694
LOC55420	-0.031424355	0.080086222	0.164197	-0.05028	0.154346	-0.10331
LOC55420	-0.043448037	-0.005820785	-0.02865	0.01932	-0.04328	-0.0671
LOC57399	-0.056991462	-0.047850576	0.085629	-0.00446	3.49E-05	-0.05404
LOC57453	-0.052616392	-0.035675824	-0.06699	0.003392	0.009731	-0.00261
LOC61326	-0.02716198	-0.062139355	-0.13487	-0.02481	0.094245	-0.15653
LOC64151	-0.053924467	0.047932586	0.031943	-0.0151	0.038459	-0.03356
LOC64223	-0.013118186	0.071775409	0.164091	-0.03351	0.082631	-0.13938
LOC64242	-0.093833347	-0.097870208	0.119801	0.016203	0.110223	-0.22695
LOC64253	-0.015205116	0.039875561	0.037876	-0.01613	-0.03222	-0.16435
LOC64262	-0.043281927	-0.025737158	-0.0092	0.005612	0.009802	0.075223
LOC64275	-0.050734715	-0.070334237	-0.0966	0.005657	-0.0017	0.119558
LOC64277	-0.026453435	-0.037121304	0.008464	0.019388	-0.04022	-0.03065
LOC64285	-0.019570666	-0.111586324	0.152913	-0.01356	0.0626	-0.00241
LOC64298	-0.029018124	-0.010910479	0.06329	0.005412	-0.01348	-0.11042
LOC64307	-0.010425319	-0.104413286	-0.24665	-0.0823	0.217687	0.28937
LOC64308	-0.064723991	-0.029421617	0.008225	-0.01708	0.019397	-0.02659
LOC64320	-0.047191113	-0.025046393	0.038579	0.036027	0.035739	-0.03777
LOC64335	-0.038195348	-0.080144453	-0.05558	0.007036	-0.00768	-0.04328
LOC64354	-0.037060545	-0.066468764	0.041596	-0.01033	0.056881	0.032966
LOC64365	-0.044104604	-0.072898502	-0.04923	0.000548	-0.01464	0.041978
LOC64371	-0.103141127	-0.056945284	0.07839	-0.10417	0.027614	0.067669
LOC64373	-0.007369265	0.105110414	0.333701	-0.027	0.077638	-0.3689
LOC64409	-0.022933367	0.057579756	0.012891	-0.00619	0.013951	0.109235
LOC64413	-0.061649415	-0.009111909	-0.06364	-0.03355	0.091669	0.131745
LOC64465	-0.007095495	-0.042632828	-0.06205	0.074801	-0.18096	0.03839
LOC64479	-0.02607352	-0.082411695	0.067529	0.000516	-0.0681	-0.13343

LOC64485:	-0.041680482	0.005737556	-0.06769	-0.01386	0.063138	-0.1848
LOC64518(	-0.069335951	-0.064114412	-0.04995	-0.0242	0.070109	0.121163
LOC64526:	-0.043082133	0.036237799	0.013387	0.018954	-0.03499	-0.12114
LOC64532:	-0.036971662	-0.040254344	0.016711	0.006423	-0.01222	0.094375
LOC64535!	-0.039978845	-0.031210333	0.052061	0.010492	-0.01373	-0.03442
LOC64548!	-0.083321779	-0.087303072	-0.0011	-0.02416	0.081336	-0.02626
LOC64551:	-0.017257526	-0.049243533	0.138605	0.033077	0.022286	-0.06892
LOC64598	-0.019898516	-0.040945947	-0.07479	0.013008	-0.01693	0.238428
LOC64601	0.010068991	-0.11058415	-0.11925	-0.0006	-0.00602	-0.01794
LOC64621	0.018924879	-0.043242841	-0.24274	-0.02507	0.009751	-0.01224
LOC64624:	-0.027152802	-0.054005921	-0.01588	0.004176	-0.01834	-0.0615
LOC64626(	-0.050563309	-0.072093505	-0.14783	0.007471	0.007977	0.167017
LOC64648:	-0.025123086	-0.037980198	0.076733	0.041739	-0.12394	-0.09105
LOC64648	-0.033227902	-0.035560628	0.029366	0.045615	-0.10943	-0.07879
LOC64652:	-0.036048373	-0.096160597	-0.00019	-0.00708	0.040999	-0.0533
LOC64658(	-0.028423651	-0.038817108	0.016126	-0.01055	0.035716	-0.0796
LOC64662(	-0.018907898	-0.077165334	-0.00682	0.005406	-0.00299	-0.02658
LOC64673(	-0.054518353	-0.061165044	0.008512	0.053873	-0.12933	-0.03029
LOC64676:	-0.031966906	0.036186006	-0.19867	-0.02681	0.05984	0.098493
LOC64677(	-0.036737314	0.206696424	-0.02119	-0.00871	0.052163	-0.12051
LOC64690:	-0.034389347	-0.040845414	-0.06618	-0.02011	0.054162	-0.03468
LOC64707(	0.016889978	-0.037198045	0.062849	-0.01485	-0.05522	-0.1598
LOC64732:	-0.026117699	-0.041369105	0.078504	0.025122	-0.06299	-0.1084
LOC64869:	-0.044689315	-0.062592919	0.012336	0.0062	0.007033	0.014608
LOC64898'	0.012686076	-0.068656748	0.025595	0.025932	-0.10424	0.068928
LOC65227(	-0.017559797	-0.068103275	-0.00999	-0.01877	-0.03478	-0.10807
LOC65299:	-0.026824739	0.060210911	0.046223	0.001636	0.010081	-0.06934
LOC65316(	-0.034303094	-0.040834381	-0.06898	-0.00427	-0.02527	-0.15554
LOC65358:	-0.035244963	-0.14083781	0.007662	-0.0114	0.06105	-0.01952
LOC65434:	-0.037451776	-0.040565918	0.017458	0.002691	-0.01673	-0.0729
LOC65478(	0.010051156	-0.048428461	0.114414	-0.0174	-0.03715	-0.18554
LOC65484:	-0.035714055	-0.03416957	-0.15878	0.054033	-0.12903	-0.01604
LOC69224'	-0.068829419	-0.144682828	0.01542	-0.05406	0.02744	-0.12329
LOC72782(	0.012078961	0.110897378	0.216834	-0.02993	0.066048	-0.21036
LOC72791(	-0.047049112	-0.041758535	-0.05068	0.012903	-0.03171	0.00932
LOC72794	-0.033704556	-0.126883894	-0.10244	0.02197	-0.04105	0.055208
LOC72802	-0.031110528	-0.032492321	0.006636	-0.0333	0.118941	0.079192
LOC72804(	-0.033672985	-0.077419382	-0.07029	0.013135	-0.01673	0.086638
LOC72806:	-0.017430744	0.001555333	-0.08459	0.006054	-0.02184	-0.09702
LOC72807:	-0.026945288	0.020764046	0.071012	-0.00091	-0.01346	-0.10259
LOC72808	-0.03656255	-0.096963538	-0.00709	-0.00036	0.028743	-0.06788
LOC72809!	-0.027082723	-0.064002217	0.018939	-0.00375	0.012252	-0.07987
LOC72811	-0.038303416	0.035011372	-0.0422	-0.00377	0.024378	-0.01228
LOC72817!	-0.031590159	-0.027885141	-0.01734	0.043609	-0.07936	-0.045
LOC72819(	-0.025832887	-0.005895002	-0.03113	0.001461	0.00419	-0.13359
LOC72835:	-0.04409844	-0.11534128	0.094682	-0.00635	0.007562	-0.06601
LOC72839:	0.016984113	-0.011101303	0.05333	-0.05088	0.145923	0.15662
LOC72844!	-0.035883139	-0.019033342	-0.03832	0.005953	0.002928	-0.02886
LOC72848!	-0.030604064	-0.006091597	0.04976	-0.01728	0.041857	-0.12172
LOC72861:	0.011565284	0.053892585	0.231213	0.049616	-0.13112	-0.27301
LOC72869(	-0.04779739	-0.084907488	-0.19282	0.007966	0.008187	0.214871

LOC72873(	-0.032272469	-0.024062521	0.091729	0.060269	-0.14576	-0.0104
LOC72874:	-0.024848122	-0.073833928	0.154582	-0.00226	-0.01782	-0.22365
LOC72880!	-0.036541669	-0.025584266	0.034244	-0.02067	0.084946	-0.05582
LOC72881!	-0.054406258	0.001070844	0.009547	-0.03182	0.125085	-0.00199
LOC72886!	-0.032997581	-0.043752629	-0.01867	0.012485	-0.02356	0.026724
LOC72908:	-0.020021532	-0.074334694	-0.25906	-0.04288	0.127482	0.371199
LOC72916	-0.024915086	-0.021102822	0.054874	-0.01397	0.048439	0.007172
LOC72917:	-0.024153241	-0.022385491	-0.01127	-0.00176	-0.06169	-0.09396
LOC72922	-0.028562977	-0.050290968	0.007728	-0.00647	0.039584	0.048994
LOC72929!	-0.044324117	-0.109668129	0.086745	-0.0219	0.071589	-0.1262
LOC72929(	-0.034127972	0.053994062	-0.12232	0.000153	0.022797	-0.01307
LOC72930'	-0.053951897	0.004335276	-0.01305	-0.01074	0.036472	-0.08459
LOC72950(	-0.028225141	-0.02080948	0.030524	-0.01232	-0.02319	-0.05449
LOC72965:	-0.058218988	-0.01293402	0.050711	0.008474	-0.01917	0.028033
LOC72965!	-0.040829736	-0.060232079	-0.04753	-0.00341	0.024095	-0.05102
LOC72968(	-0.01639613	0.063343213	0.026547	0.081022	-0.18122	-0.06064
LOC72968:	0.010682766	-0.028254901	0.167416	-0.05578	0.090119	-0.01095
LOC72973:	-0.020477735	-0.042293395	-0.0873	-0.00954	-0.01056	0.015897
LOC72986(	-0.056550607	-0.011761344	0.049969	-0.00714	0.053084	-0.01818
LOC72987(	-0.034954978	0.027078024	0.077547	0.023973	-0.05123	-0.03856
LOC72988'	-0.018772867	-0.052571787	0.107733	0.036373	-0.06791	0.162195
LOC72997(	-0.068196015	-0.081835591	0.151736	-0.05605	0.103536	-0.08047
LOC73009!	-0.029365105	-0.119757349	0.00895	0.0224	-0.09582	-0.00126
LOC73010!	-0.012738794	-0.040883596	-0.12284	-0.00341	0.021414	-0.14376
LOC73010:	-0.029023907	-0.093515654	-0.20398	-0.08848	0.272097	0.524748
LOC73013!	-0.018410588	-0.075536788	0.037769	-0.00699	-0.06303	-0.09432
LOC73020:	-0.017087346	-2.93E-05	0.061743	0.016955	-0.03607	0.012259
LOC73096!	-0.032261453	-0.03234146	0.049918	0.00219	0.006847	-0.03575
LOC73115'	-0.018564128	-0.02201324	0.089356	0.015493	-0.04221	-0.14293
LOC73142	-0.015111065	-0.037146395	-0.06721	0.022308	-0.08916	-0.04693
LOC78052!	-0.040785507	-0.018178709	-0.02604	0.04005	-0.08997	-0.03185
LOC79999	-0.032269044	-0.115340442	-0.02841	0.006278	-0.04005	-0.04738
LOC80154	-0.017387392	-0.029246379	0.206712	0.059008	-0.24727	-0.28267
LOC81691	-0.013673043	-0.019668936	-0.00843	0.109594	-0.32213	-0.09116
LOC90246	-0.030691742	-0.058723942	0.019915	-0.00097	0.007558	-0.05588
LOC91450	-0.028999204	-0.091954112	0.01505	-0.00536	-0.03849	0.04203
LOC91548	0.0269065	-0.021098111	0.125215	0.001768	-0.04605	-0.27268
LOC93432	-0.059296002	-0.048401989	0.055062	0.028682	-0.06798	-0.05408
LOC93444	-0.026744315	-0.061500188	-0.07773	0.006267	-0.08661	-0.14075
LOC93463	-0.093095348	0.004035965	-0.00158	-0.03212	0.073551	0.002151
LOC93622	0.023119103	0.147053301	0.426854	-0.01465	0.020357	-0.32128
LOH12CR1	0.00396319	0.049680829	0.165764	-0.0272	0.084521	-0.12922
LOH12CR2	-0.035022231	-0.00896209	0.048541	0.029178	-0.06149	-0.03605
LONP1	0.028425765	-0.0035517	0.148853	0.094781	-0.26972	0.048596
LONRF1	0.006001196	-0.152348837	-0.18143	-0.01829	0.039233	-0.08014
LONRF2	-0.020751777	-0.017083804	-0.01639	0.011571	-0.06261	-0.10402
LONRF3	-0.013655048	0.0870753	-0.00432	-0.00712	0.045352	0.082646
LOR	-0.014491198	-0.037260624	0.036836	0.019414	-0.05221	0.021896
LOX	-0.047319391	0.021005999	-0.04694	-0.03107	0.052821	-0.07521
LOXHD1	-0.030325321	-0.074110543	-0.01219	-0.01116	0.057384	0.109628
LOXL1	-0.014114292	-0.033661669	0.133252	0.0722	-0.20163	-0.06219

LOXL1-AS1	-0.011679661	-0.029346807	0.319877	0.036373	-0.02699	-0.16221
LOXL2	-0.070354556	-0.103832046	-0.05021	-0.02166	0.036633	-0.13147
LOXL3	-0.003028799	-0.123259952	-0.25022	-0.07673	0.202802	0.551082
LOXL4	-0.028570536	-0.03008869	-0.02205	-0.00915	-0.04814	-0.09869
LPA	-0.060289004	-0.054811026	-0.11275	0.007779	-0.0368	0.147268
LPAL2	-0.029919124	0.242863853	0.063113	-0.00724	0.04387	-0.13598
LPAR1	-0.011539908	-0.114298375	-0.14616	-0.01026	0.114991	0.527697
LPAR2	0.016705213	-0.090584607	0.008594	-0.02102	0.039857	-0.1324
LPAR3	-0.043413184	-0.059364281	0.008803	-0.01929	0.046788	-0.11651
LPAR4	-0.047030952	-0.065494649	-0.09453	-0.06561	0.210567	-0.06623
LPAR5	-0.000173333	0.119790215	0.101858	-0.04077	0.118701	-0.2716
LPAR6	0.009881111	0.08295177	0.094989	-0.04733	0.129142	0.354261
LPCAT1	0.041943278	0.342994042	0.00825	-0.00331	0.031411	-0.0865
LPCAT2	0.009232102	-0.103914255	-0.27963	-0.05651	0.159136	0.259122
LPCAT3	-0.006456297	-0.01104976	-0.18225	-0.00294	-0.05969	0.092259
LPCAT4	-0.043375064	-0.066635095	0.114458	0.019201	-0.02295	-0.12738
LPGAT1	0.004845641	0.074944197	-0.44952	0.042026	0.095143	0.076501
LPHN1	-0.023211754	0.126159378	0.203642	-0.03105	0.120927	-0.27255
LPHN3	-0.0337467	-0.00513193	-0.0264	0.074792	-0.15532	-0.06336
LPIN1	0.026166259	0.156903191	0.324215	0.029471	-0.04472	-0.36093
LPIN2	0.031369716	0.136477682	0.171976	-0.00134	########	-0.25491
LPIN3	-0.034150919	-0.101186045	0.014143	0.013958	-0.01327	0.097058
LPL	-0.002849297	-0.052657912	-0.10317	-0.04786	0.123132	0.293335
LPO	-0.029486819	-0.096792755	-0.16368	-0.00147	0.006388	0.129956
LPP	0.016753726	0.117067279	-0.23163	-0.07251	0.186816	0.14249
LPP-AS2	-0.04110782	0.085923793	-0.16019	-0.04631	0.147817	0.205458
LPPR1	-0.046191785	-0.05006874	0.012012	-0.01216	0.044097	-0.00939
LPPR2	-0.008727303	-0.082578259	-0.21209	-0.03057	0.051876	0.352763
LPPR3	-0.01530972	-0.05415199	-0.01075	0.034482	-0.11925	-0.01907
LPPR4	-0.055582877	-0.00535517	-0.07227	-0.02199	0.068059	0.133602
LPPR5	-0.084323932	-0.085838879	-0.05044	-0.02047	0.078272	-0.08875
LPXN	0.038153602	0.070947287	0.1707	0.051184	-0.11897	-0.13505
LQFBS-1	-0.023661668	-0.041527247	0.029497	-0.00805	0.011571	-0.12681
LRBA	0.017581047	0.091060783	0.29073	1.22E-05	0.004567	-0.39019
LRCH1	-0.003218075	0.126684735	-0.10339	0.023462	-0.04859	-0.01722
LRCH2	-0.07293999	-0.060443712	0.022365	-0.01375	0.013533	-0.08759
LRCH3	0.011639777	0.029620931	0.071458	-0.00698	-0.1166	-0.09789
LRCH4	0.018935969	-0.183500763	0.02471	-0.01528	-0.03359	0.023584
LRFN1	-0.004372924	0.006658625	-0.09407	-0.02635	0.056215	-0.0134
LRFN2	-0.037619895	0.001259156	-0.07946	0.007673	-0.00186	-0.03041
LRFN3	-0.012273118	-0.011828527	0.324956	0.022531	-0.05877	-0.1281
LRFN4	-0.007774177	-0.116590278	-0.09709	0.020724	-0.17918	-0.06039
LRFN5	-0.022510557	-0.046508705	0.03263	-0.0103	-0.01723	-0.08668
LRG1	-0.000684743	-0.079748657	-0.16505	0.060817	-0.1757	0.011011
LRGUK	-0.024786047	-0.058540348	-0.1427	0.005837	1.25E-05	-0.02193
LRIF1	0.014832959	0.145085111	0.023881	0.05694	-0.17911	-0.29597
LRIG1	-0.030193427	-0.059024537	-0.04149	0.029367	-0.10792	0.019216
LRIG2	0.034885508	0.041222491	0.11563	-0.04563	0.01899	0.104723
LRIG3	-0.074794802	-0.048825565	-0.00346	-0.00081	0.015099	-0.01223
LRIT1	-0.043937637	-0.081525691	0.038961	0.008602	0.009563	-0.0265
LRMP	0.029615948	-0.008024803	-0.28443	-0.03299	0.109976	-0.08058

LRP1	-0.025149686	-0.101036825	-0.19039	-0.05571	0.134363	0.674895
LRP10	0.022510758	0.012295237	-0.11653	-0.00098	-0.00183	-0.30523
LRP11	-0.009702021	-0.002935311	0.021531	0.107624	-0.20193	0.043375
LRP12	-0.007628265	0.03496754	-0.07411	0.02161	-0.11832	0.026139
LRP1B	-0.049277037	-0.048663764	-0.00313	0.022862	0.009337	-0.11333
LRP2	-0.043629352	-0.077733593	-0.01588	0.033339	-0.08153	-0.18865
LRP2BP	-0.031126401	0.022056446	0.098998	0.017104	-0.02893	-0.18367
LRP3	-0.022766086	-0.110218303	-0.09639	-0.03806	0.073892	0.470978
LRP4	-0.047756799	-0.071971963	-0.01185	0.013161	-0.02093	-0.00758
LRP5	-0.034314757	-0.099536089	0.026531	0.024828	-0.04547	0.054428
LRP5L	-0.004666123	-0.089457757	-0.00619	0.011484	-0.02016	0.128546
LRP6	-0.106074248	-0.123165428	0.177093	-0.12808	0.029048	-0.14412
LRP8	-0.023870101	0.042067134	-0.08671	0.112512	-0.28333	-0.09355
LRPAP1	-0.030928851	-0.068336201	0.027375	-0.04918	-0.00961	0.038918
LRPPRC	0.022006033	0.020706105	0.180052	0.067036	-0.05327	0.00155
LRR1	0.013133711	0.056774652	-0.06223	0.140736	-0.37718	0.153595
LRRC1	-0.023214714	0.014567566	0.023025	0.01417	-0.06754	-0.26935
LRRC10B	-0.029313941	-0.077901735	0.012367	0.012894	-0.02534	0.120058
LRRC14	0.007038237	-0.023061479	0.252821	0.018767	-0.10031	-0.25627
LRRC15	-0.05716004	-0.075897901	0.044491	0.015009	-0.01977	-0.07535
LRRC16A	0.027899961	-0.149901044	0.116099	-0.13995	0.259057	-0.14808
LRRC16B	-0.005366999	-0.049889922	-0.10718	-0.00619	-0.07004	-0.10353
LRRC17	-0.037378535	-0.061839703	0.013898	0.008129	-0.00391	-0.14404
LRRC18	-0.018609918	-0.01630853	0.103288	0.076104	-0.21749	-0.07882
LRRC19	-0.084526089	-0.028776182	-0.02999	-0.04373	0.108195	-0.04886
LRRC2	-0.035274919	-0.025085393	-0.04372	-0.0186	0.027485	0.040649
LRRC2-AS	-0.031403156	-0.044395317	0.006157	-0.00221	0.017078	-0.07252
LRRC20	-0.020025305	-0.082869324	0.104079	0.07569	-0.2668	-0.10098
LRRC23	-0.019548916	-0.048080477	-0.03694	-0.00125	0.036197	0.083269
LRRC25	0.00715274	-0.121444647	-0.32887	-0.10782	0.294803	0.43795
LRRC27	-0.003538431	0.063303917	-0.12719	0.026979	0.098774	0.02677
LRRC28	-0.027785755	0.075819963	-0.03713	-0.01911	0.081399	0.181253
LRRC29	-0.031192237	-0.077263102	-0.0446	-0.01777	0.052044	0.170257
LRRC3	-0.034094737	-0.038276818	0.020248	-0.00429	0.048883	-0.00306
LRRC31	-0.045642437	-0.075997997	-0.07086	0.001496	0.014326	-0.02308
LRRC32	-0.029701253	-0.064455046	0.029726	-0.00461	-0.03194	-0.005
LRRC34	-0.013551755	-0.068019247	-0.0644	0.00149	-0.00938	-0.11089
LRRC36	-0.007275252	-0.087125994	-0.09072	-0.01878	0.006356	-0.01833
LRRC37A2	0.029232314	0.017860352	0.140591	-0.03661	0.097526	-0.32013
LRRC37A3	-0.061388773	-0.043662097	-0.01538	0.100358	-0.02266	-0.1435
LRRC37A4I	0.011868172	-0.055834285	0.127962	-0.02355	0.028184	-0.30446
LRRC37A5I	-0.060498653	-0.004834836	-0.10286	-0.00686	0.029979	-0.00363
LRRC37BP	-0.023740802	-0.014991365	-0.07305	0.009545	-0.01465	-0.04808
LRRC38	-0.068841525	-0.066480488	0.064824	0.012041	-0.04766	0.030936
LRRC39	-0.027780055	0.02063582	0.023817	-0.01267	0.043171	-0.06175
LRRC3B	-0.061675058	-0.118728104	-0.0698	-0.01576	0.051944	-0.03325
LRRC4	-0.007987202	-0.086886475	-0.18576	-0.02371	0.021693	0.040286
LRRC40	0.032105099	0.047900031	0.088664	0.030101	-0.10399	0.023741
LRRC41	-0.011242327	0.033229777	-0.0546	0.074936	-0.22253	0.11305
LRRC42	0.011369436	-0.014935168	-0.07484	0.119455	-0.33278	0.114413
LRRC43	-0.022048971	-0.003653582	-0.00016	-0.02324	-0.04834	-0.06125

LRRC45	-0.003312875	0.011162861	0.004779	0.005115	-0.07318	-0.02615
LRRC46	-0.041813557	-0.053336783	-0.07619	0.031701	-0.07182	0.004382
LRRC47	0.078668054	0.123882918	0.051262	-0.00449	-0.00277	0.12719
LRRC48	-0.035703757	-0.051499004	0.008564	0.001771	0.021302	0.193032
LRRC49	-0.039259507	-0.009364799	0.082546	0.059186	-0.13343	-0.08089
LRRC4B	-0.035470571	0.004485318	0.017875	-0.01187	0.056301	0.089984
LRRC4C	-0.057756946	0.007756793	-0.13101	0.00454	0.00086	-0.0767
LRRC52	-0.029167481	-0.000337323	0.037985	0.01677	-0.07649	-0.1895
LRRC55	-0.045675144	-0.003555751	-0.05664	0.003779	0.016374	0.061626
LRRC56	-0.019414101	-0.114305945	0.036391	-0.01787	-0.00506	-0.09695
LRRC57	0.008360615	0.042622779	-0.18097	0.01502	-0.02528	0.273403
LRRC58	0.017146835	0.104383824	0.04679	0.048251	-0.11297	0.230442
LRRC59	-0.062265078	-0.025825082	-0.01531	-0.00256	-0.07327	-0.04207
LRRC6	-0.017003568	0.026674026	-0.15313	-0.04525	0.12508	0.121704
LRRC61	-0.000644738	-0.057682534	-0.02422	0.096611	-0.36204	-0.10827
LRRC63	-0.128943408	0.045286921	0.009799	0.0071	-0.01475	-0.07341
LRRC66	-0.047608423	-0.089827586	0.018699	0.016052	-0.0219	0.060099
LRRC69	-0.010855125	-0.023048489	0.14556	0.017526	-0.01536	-0.16147
LRRC7	-0.034937322	-0.079552135	0.208579	-0.01294	0.01848	-0.04133
LRRC71	-0.02737597	-0.009438967	-0.06776	-0.01024	0.016163	0.134071
LRRC72	-0.044858326	-0.045531222	-0.0106	0.019952	-0.037	0.073658
LRRC73	-0.031669543	-0.037126415	0.058173	-0.00218	0.031584	0.170496
LRRC74	-0.047449589	-0.091983975	-0.08685	-0.00733	-0.04998	0.051055
LRRC75A	-0.009195029	-0.003112278	0.137527	-0.06349	0.193291	0.11147
LRRC75B	-0.031655491	-0.073163738	-0.11789	0.027254	-0.10025	0.018314
LRRC8A	0.033087406	0.269030564	0.229041	-0.00212	-0.01067	-0.20874
LRRC8B	-0.072195742	-0.105357106	-0.04886	-0.05004	-0.01282	0.05868
LRRC8D	0.044298467	-0.043033567	0.021305	-0.04643	0.114906	0.241116
LRRC8E	-0.055426782	0.008158264	0.106453	0.010674	0.011983	0.125589
LRRCC1	-0.009432357	0.010059805	0.018826	0.060419	-0.16657	-0.08964
LRRD1	-0.058748378	-0.042224746	-0.0896	-0.01306	0.018109	-0.04443
LRRFIP1	0.005220261	-0.07223356	-0.23217	0.02091	0.006925	0.109481
LRRFIP2	-0.021865009	-0.066416465	-0.13214	0.001496	-0.06631	0.157612
LRRIQ1	-0.042862578	-0.06333935	0.173387	-0.01841	0.074218	-0.05692
LRRIQ3	-0.033936254	0.017759829	0.307963	-0.00567	0.030421	-0.22379
LRRK1	-0.001078642	-0.183033093	-0.30357	-0.0588	0.166254	0.163479
LRRK2	0.000836469	-0.169303068	-0.45787	-0.08607	0.24738	0.400712
LRRN1	-0.022770993	0.032308063	-0.03375	-0.02246	0.047328	-0.22354
LRRN2	-0.06532526	-0.073366676	0.153869	-0.01245	0.02639	-0.07519
LRRN3	-0.002542509	-0.089204983	0.509485	-0.03485	0.088046	-0.20757
LRRN4	-0.032995528	-0.016935337	-0.02671	0.00426	-0.00802	-0.03713
LRRN4CL	-0.03472861	-0.12006862	-0.04565	0.053188	-0.13821	0.056028
LRRTM1	-0.04708398	-0.112897239	-0.00953	-0.00373	0.039733	0.023782
LRRTM2	-0.021939661	-0.029280645	0.07654	0.016242	-0.03241	-0.01075
LRRTM4	-0.074692462	-0.012773672	0.002313	0.008436	-0.01153	-0.03245
LRSAM1	-0.017670556	-0.091007798	-0.01311	0.044808	-0.12774	0.077329
LRTM1	-0.063475043	-0.094906543	-0.01976	-0.02742	0.063219	-0.07776
LRTM2	-0.024675714	-0.127702726	-0.07317	-0.01779	-0.01695	-0.11876
LRTOMT	-0.049762211	-0.034188248	-0.00729	0.064565	-0.20237	-0.08969
LSAMP	-0.016094753	-0.022839985	-0.02706	0.126054	-0.14823	-0.01421
LSAMP-AS	-0.029757519	0.009342472	-0.0784	0.065654	-0.17144	-0.02351

LSG1	0.017657588	0.054041067	0.174236	0.014259	-0.09387	-0.035
LSM1	0.104604916	0.18102054	-0.04401	0.061	-0.16552	0.132415
LSM10	0.044665891	-0.033593834	-0.29666	0.022088	-0.07314	0.194484
LSM11	-0.034663194	0.072624525	0.007203	0.116313	-0.30824	0.155041
LSM12	0.029487498	0.003225351	-0.03766	0.079302	-0.23221	0.276467
LSM14A	0.173003191	0.297745116	0.134371	0.06834	-0.0712	-0.34972
LSM14B	-0.056818821	-0.013428601	0.058973	0.035402	-0.10346	0.092388
LSM2	0.021736306	0.056908357	0.175502	0.070299	-0.19053	-0.03106
LSM3	0.049651745	0.089898566	0.061602	0.106404	-0.26127	-0.04283
LSM4	-0.038685346	0.026040489	0.072183	0.08477	-0.20938	0.272905
LSM5	0.006883225	-0.018783164	0.120406	0.066187	-0.13894	0.100787
LSM6	0.044002434	0.014561639	0.107871	0.059855	-0.17449	0.319479
LSM7	0.026827162	0.020444607	0.111554	0.044303	-0.10151	0.085465
LSM8	-0.024506717	0.007771907	0.088576	-0.02607	0.025727	-0.16112
LSMEM1	-0.023514264	-0.050106965	-0.13712	-0.04606	0.093007	-0.16162
LSMEM2	-0.024334918	-0.051935724	-0.11391	-0.00279	0.003014	0.049471
LSP1	0.032720351	-0.015738956	-0.18615	0.014092	-0.02443	0.132956
LSR	-0.004478304	-0.092697677	0.425817	-0.04046	0.135812	-0.19111
LSS	-0.033904812	0.013267831	0.048189	-0.05778	0.060749	-0.08605
LST1	0.017273628	-0.144361854	-0.38551	-0.05445	0.131375	0.416259
LTA	-0.002920786	-0.013661983	0.089812	0.071458	-0.21759	-0.11971
LTB	0.01880535	-0.10270656	0.191498	9.43E-05	-0.01301	-0.43012
LTB4R	0.020296245	-0.041800405	-0.15612	0.008144	0.017801	0.272703
LTB4R2	-0.016982593	-0.063254811	-0.01268	0.011679	0.002877	0.058528
LTBP1	-0.023305881	-0.022527872	-0.00144	-0.22518	0.564835	0.029561
LTBP2	-0.023789825	0.049515326	-0.09272	-0.02462	0.022633	-0.08979
LTBP3	-0.077301809	-0.016918826	0.196127	-0.05636	0.309149	-0.22247
LTBP4	-0.01911254	-0.075262751	0.032715	2.26E-05	0.038923	0.004937
LTBR	-0.011033534	-0.159920151	-0.09597	0.018437	-0.0018	0.012198
LTC4S	-0.021345131	-0.129409105	-0.07905	-0.00631	-0.00152	0.004927
LTF	0.001612449	-0.070328702	0.438377	-0.05783	0.135222	-0.1718
LTK	-0.008136052	-0.115746235	0.118077	-0.01827	0.074559	-0.2465
LTN1	0.018039731	0.065996521	-0.12798	0.00298	0.001339	0.220403
LTV1	0.013783042	0.014501368	0.088923	0.016233	-0.03456	-0.02387
LUC7L	0.043166183	0.025726941	0.182165	0.016065	0.003829	-0.12382
LUC7L2	0.035418937	0.106811726	0.040113	0.06326	-0.19935	-0.02798
LUC7L3	0.045409191	0.067137443	0.255546	-0.00562	0.080043	-0.28838
LUM	-0.063989056	-0.105002346	-0.03607	-0.02654	0.032114	-0.07391
LURAP1	-0.027716625	-0.118887669	0.147545	-0.0024	-0.06854	-0.08536
LURAP1L	-0.02049888	-0.030234717	-0.01948	0.007271	-0.03114	-0.09825
LUZP1	0.02381587	0.124586083	0.099277	0.030653	-0.03104	0.050096
LUZP2	-0.02714385	-0.064320207	-0.00423	0.025788	-0.10838	0.060131
LUZP4	-0.03028321	-0.016423691	0.006693	0.001916	-0.03413	-0.07754
LXN	0.014002676	0.034338849	-0.12442	-0.0606	0.176286	-0.10557
LY6D	-0.026206133	-0.07082447	-0.07888	0.041493	-0.09509	-0.08311
LY6E	0.02178717	0.071514506	0.050598	0.023726	-0.08632	0.077019
LY6G5C	-0.0088633	0.050747002	0.120752	-0.10287	0.299125	0.175036
LY6G6C	-0.031686517	-0.020500229	-0.10172	-0.0078	-0.02826	-0.13072
LY6G6E	-0.036672338	-0.0524041	-0.00149	0.013413	-0.00194	0.097569
LY6H	-0.037017906	-0.079118454	-0.0096	-0.00925	0.0553	0.107534
LY6K	-0.037886455	-0.048254539	-0.07232	0.02705	-0.06129	0.074224

LY75	0.028902277	0.045470241	0.044681	-0.03668	0.121462	-0.16801
LY86	0.009163086	-0.129284196	-0.30483	-0.08406	0.231259	0.455934
LY86-AS1	-0.060170559	-0.033880839	-0.02002	-0.03527	0.061844	-0.00041
LY9	0.017920853	-0.014538912	0.417116	-0.05813	0.193988	-0.44365
LY96	0.01703744	-0.182780188	-0.32132	-0.05237	0.167525	0.398438
LYAR	0.021439394	0.12113175	0.302749	0.029111	-0.1078	0.021914
LYG1	-0.016666682	0.025828365	0.01518	-0.02493	0.061555	0.058371
LYG2	-0.033913468	0.028275554	-0.05906	0.005424	0.019527	0.06309
LYL1	0.017914093	-0.203431587	-0.22968	-0.11541	0.279031	0.224841
LYN	0.021595967	0.056417809	-0.71052	-0.0532	0.149571	0.197927
LYNX1	-0.026830304	-0.087268188	-0.07553	-0.01041	-0.01331	0.159191
LYPD1	-0.031342332	-0.058116805	-0.06851	0.019013	-0.04127	0.052548
LYPD3	-0.008481367	-0.140203984	0.194345	-0.01377	-0.02015	-0.1138
LYPD4	-0.053839347	-0.101174278	-0.02697	-0.01748	0.041569	-0.04247
LYPD5	-0.02970555	-0.042601798	-0.07583	0.009686	-0.00671	0.04553
LYPD6	-0.040246259	-0.04341237	0.06404	0.006016	0.025459	-0.10622
LYPD6B	-0.027928011	-0.125738026	-0.15228	-0.0043	0.014032	0.01009
LYPD8	-0.035098842	-0.023359601	-0.0362	0.009558	-0.03334	-0.04817
LYPLA1	0.078900801	0.1062747	-0.38843	0.08458	-0.21067	0.277597
LYPLA2	0.042383237	-0.017391181	0.081182	0.00897	-0.13865	-0.06627
LYPLAL1	0.013236285	-0.052593726	-0.13549	-0.02169	0.067349	0.116488
LYRM1	0.048810969	-0.029423468	0.026575	0.095048	-0.26689	-0.27605
LYRM2	0.033115297	0.1212076	0.179206	0.03823	-0.054	0.049826
LYRM4	0.015840242	-0.038074808	0.232759	0.035896	-0.08215	0.043513
LYRM5	0.032315076	-0.055611847	0.135261	-0.01366	0.041938	0.014632
LYRM7	0.036686	0.021497708	0.1803	-0.00821	-0.02833	-0.13715
LYRM9	-0.006053836	-0.065884756	0.247297	-0.06687	0.191622	-0.18969
LYSMD1	-0.012212531	-0.011678942	-0.04586	0.082348	-0.2575	-0.0665
LYSMD2	0.062998765	-0.020434898	-0.11554	-0.00732	0.048657	-0.14281
LYSMD3	0.036061989	0.15739025	-0.08644	0.004759	-0.01907	-0.1582
LYSMD4	-0.002795932	-0.010672979	0.07769	-0.01708	0.011561	-0.17615
LYST	0.008560964	0.006400411	-0.27086	-0.06371	0.113993	0.282638
LYVE1	-0.056642965	-0.055997828	-0.11117	0.008975	-0.03825	0.011739
LYZ	0.01467721	-0.149871321	-0.20865	-0.11255	0.272542	0.417852
LYZL1	-0.040437755	-0.025563127	0.005186	0.03457	-0.07669	-0.0203
LYZL4	-0.033943086	-0.08857793	0.009512	-0.0182	0.001959	-0.05184
LYZL6	-0.029173691	-0.062196105	0.013125	0.007656	-0.03049	0.017424
LZIC	0.031770052	0.033389796	-0.03041	0.040488	-0.18147	0.26208
LZTFL1	0.007545522	0.113064491	-0.11999	-0.13675	0.272603	-0.12886
LZTR1	0.024153865	-0.045368705	0.100439	0.031134	-0.12524	-0.00905
LZTS1	-0.074561327	-0.060725778	-0.07168	0.070917	-0.05556	0.022276
LZTS1-AS:	-0.032691636	-0.03437115	0.09349	-0.00257	0.021416	-0.02935
LZTS2	-0.031418756	-0.074026174	0.169026	-0.01235	0.12077	-0.1541
LZTS3	-0.018793239	-0.132078886	0.278802	-0.01172	0.034643	-0.06729
M1AP	-0.075391411	-0.133905303	-0.07784	-0.04484	0.098258	0.272911
M6PR	0.045390501	0.102823587	-0.03092	-0.01472	0.035185	0.19087
MAATS1	-0.100499877	-0.080646238	-0.03595	-0.04928	0.040518	-0.13936
MAB21L2	-0.054636226	-0.054723759	-0.0008	0.001628	3.99E-05	-0.04055
MAB21L3	-0.045028921	-0.025637921	-0.15065	-0.00068	0.01913	-0.01384
MACC1	-0.004758827	-0.040715257	-0.0359	0.067645	-0.20927	-0.10808
MACF1	0.004731963	0.219262579	0.208015	-0.01782	0.057806	-0.26647

MACROD1	-0.032214307	-0.082517728	0.09297	0.022682	-0.04195	-0.07143
MACROD2	-0.001871013	-0.041201246	-0.10842	0.010102	0.038118	-0.09549
MACROD2-/	-0.037323509	-0.05467804	0.079141	0.022767	-0.04975	-0.03829
MAD1L1	0.009878779	0.024435127	0.149914	0.020814	-0.04255	0.062314
MAD2L1	0.014647882	0.000345425	0.341955	0.153467	-0.30039	-0.31286
MAD2L1BP	0.040713541	0.019793358	-0.15122	0.044602	-0.18607	0.063216
MAD2L2	0.026952649	0.125997518	-0.03112	0.115219	-0.3259	0.195125
MADCAM1	-0.021626706	-0.06859102	-0.0207	-0.02674	-0.021	-0.12204
MADD	0.055401342	0.103872513	0.017931	-0.03714	0.068907	-0.1199
MAEL	-0.040452439	-0.025031016	-0.01613	-0.01679	0.07467	0.049196
MAF	-0.013644126	0.141897308	0.070638	-0.00462	0.004224	-0.13661
MAF1	0.058111514	0.046705649	-0.00912	0.011848	-0.04922	0.141227
MAFB	0.010005803	-0.0650206	-0.27785	-0.11336	0.30148	0.606502
MAFF	0.0142123	0.159447729	-0.01221	0.007065	-0.03227	-0.15096
MAFG	0.00999305	-0.088903015	-0.07588	0.031334	-0.12218	0.200577
MAFG-AS1	-0.022028759	-0.069406446	0.062444	0.017084	-0.15154	-0.11183
MAFK	0.012598718	-0.037537893	-0.04935	-0.0254	0.062705	-0.09779
MAG	-0.037967478	-0.023237852	-0.14399	0.028672	-0.04523	-0.0011
MAGEA1	-0.031365029	-0.033974197	-0.00622	0.009598	-0.01088	-0.01542
MAGEA10	-0.034289052	-0.070884624	0.023952	0.034493	-0.09498	-0.00603
MAGEA11	-0.05478923	-0.02436007	-0.01133	-0.01018	0.036986	0.045256
MAGEA12	-0.032064731	-0.001079931	0.003616	-0.00166	0.025433	-0.11588
MAGEA4	-0.048463494	-0.046687159	-0.06424	0.003789	-0.03039	0.01387
MAGEA6	-0.036210603	-0.060597975	0.01166	-0.00997	0.047596	0.007294
MAGEA8	-0.039751823	-0.075211623	-0.06538	0.015649	-0.0132	0.12628
MAGEB1	-0.043617753	-0.093584243	-0.16962	0.021845	-0.06363	0.082495
MAGEB18	-0.110224384	-0.059715778	-0.00745	-0.00963	0.06575	-0.05444
MAGEB2	-0.032544194	-0.029200019	-0.02087	0.011369	-0.01842	-0.02261
MAGEB3	-0.042231513	0.015962835	0.038101	0.009543	-0.0227	-0.08349
MAGEB4	-0.031747539	-0.042497934	-0.04231	0.006569	-0.03025	-0.01243
MAGEB6	-0.099850913	-0.171740397	0.03654	0.039975	0.058055	0.043706
MAGEC1	-0.042418535	0.028444006	-0.06623	0.031275	-0.06768	0.028822
MAGEC2	-0.052249992	-0.081187443	-0.0294	0.023962	-0.05849	-0.07893
MAGEC3	-0.054448657	-0.058523761	0.006865	0.016477	-0.04077	0.008625
MAGED1	0.028254294	0.027907699	0.222439	-0.06682	0.141102	-0.42008
MAGED2	0.024459876	0.035100096	0.290003	0.01112	-0.03265	-0.17665
MAGEE1	-0.002035372	0.144119914	0.364763	-0.03865	0.086366	-0.42073
MAGEE2	-0.054973704	-0.114307418	-0.15422	0.006096	-0.02294	0.138924
MAGEF1	0.005410442	0.019234242	0.079133	0.033442	-0.07816	-0.08292
MAGEH1	0.017627	0.045851321	0.217317	-0.06203	0.177914	-0.20867
MAGEL2	-0.033037867	-0.025996658	-0.07953	-0.0248	0.08577	0.012643
MAGI1	-0.02135741	0.015657735	-0.01765	-0.00343	-0.04818	0.013935
MAGI1-IT:	-0.03216888	-0.031601534	-0.06847	0.029442	-0.08412	-0.04874
MAGI2	-0.047330967	0.06970098	-0.08542	0.002479	0.062184	-0.18288
MAGI2-AS:	-0.044208673	-0.021690934	-0.01006	-0.01294	0.059582	0.194494
MAGI2-AS:	-0.047286651	-0.052131526	0.286372	-0.18173	0.548145	-0.00844
MAGI2-IT:	-0.057396172	-0.077068195	0.170062	-0.01986	0.051128	0.007477
MAGI3	-0.083110838	-0.100920283	0.043215	0.008922	0.074072	-0.0015
MAGIX	-0.106380051	-0.080820058	-0.07003	0.033293	-0.00116	0.006654
MAGOH2	-0.024102819	-0.035606051	0.003909	0.068445	-0.21526	0.152386
MAGOHB	-0.003430399	0.043241195	0.185338	0.066272	-0.18608	-0.04779

MAGT1	0.026772838	0.136196762	0.039949	0.025369	-0.0244	0.282709
MAK	-0.013306392	-0.073975668	-0.22049	0.000394	0.001963	0.100203
MAK16	0.019841356	0.057068311	0.135709	0.038803	-0.11277	-0.09586
MAL	-0.001048718	-0.09865661	0.45135	-0.04618	0.111452	-0.22266
MAL2	-0.070743258	-0.087608632	0.046397	-0.03536	0.081726	-0.01117
MALAT1	0.047949479	0.052644761	0.109087	-0.01791	0.114274	-0.2189
MALL	-0.034379644	-0.061701941	0.014927	-0.02022	0.052226	-0.04196
MALRD1	-0.024016273	-0.050296746	-0.01769	0.07022	-0.18384	-0.0294
MALSU1	0.041928894	0.000759336	0.182257	0.053471	-0.16451	0.050043
MALT1	0.025613118	-0.03977956	0.073946	-0.02253	0.061877	-0.07874
MAMDC2	-0.077068401	-0.080585541	0.111531	-0.05344	0.127958	0.013545
MAMDC2-AS	-0.055790455	-0.105745618	-0.01208	0.008835	-0.0139	0.04823
MAMDC4	-0.013141326	-0.055244321	0.001074	0.01874	-0.0593	-0.07303
MAML1	0.076403671	0.122680142	-0.05602	0.016079	-0.03133	-0.13387
MAML2	0.011025379	-0.066498684	0.309488	-0.01667	0.047729	-0.10205
MAML3	0.066297457	-0.06536652	-0.16361	-0.17056	0.082439	0.212576
MAMLD1	-0.00901998	-0.017652488	0.045467	0.053419	-0.17529	-0.01586
MAMSTR	-0.020647636	-0.026247995	0.013173	0.017341	-0.10077	-0.03572
MAN1A1	0.023279206	0.18991277	-0.18189	0.015292	-0.0552	-0.10228
MAN1A2	-0.044538764	0.081300691	0.048046	-0.01363	-0.04399	0.094091
MAN1B1	0.023933621	-0.013568749	0.033101	0.006966	-0.04889	-0.02872
MAN1B1-AS	0.033916172	-0.044415813	0.131217	-0.01419	-0.02415	-0.16016
MAN1C1	0.008339628	-0.065385428	0.27647	-0.05637	0.158742	0.151204
MAN2A1	0.007813941	-0.013815985	0.084979	-0.03565	0.122022	0.042769
MAN2A2	0.05168594	-0.051846681	-0.14697	-0.03132	0.110904	0.080346
MAN2B1	0.034360889	-0.18406928	-0.24811	-0.03562	0.101195	0.433174
MAN2B2	0.031336599	0.025310221	-0.00358	-0.01418	0.035029	0.036627
MAN2C1	0.018938479	-0.019606324	0.114062	0.02262	-0.04281	-0.00971
MANBA	0.028052473	-0.095144214	-0.39632	-0.07389	0.209635	0.306742
MANBAL	0.027270462	-0.115136752	-0.18221	0.027496	-0.0917	0.171274
MANEA	-0.013723373	0.106660623	-0.06167	0.00351	-0.02485	0.151845
MANEAL	-0.015618377	0.073280372	0.145623	0.129939	-0.40833	-0.1989
MANF	0.034325889	0.182290851	0.030758	0.099742	-0.27396	-0.0889
MANSC1	-0.006077017	-0.067507705	-0.20148	-0.01968	0.065396	0.055448
MAOA	-0.019415088	-0.029484784	0.002588	0.07015	-0.14954	-0.0292
MAOB	-0.052441387	-0.018838716	-0.1375	0.009555	-0.04781	-0.02858
MAP10	-0.045878931	-0.047272716	-0.04239	-0.02871	0.083883	0.008674
MAP1A	-0.015205074	-0.125107158	-0.13361	0.004614	-0.03341	-0.01136
MAP1B	-0.023356711	0.010447061	0.084636	-0.04421	0.18006	-0.03477
MAP1LC3A	0.022131412	-0.045189539	-0.01767	-0.00687	-0.04787	-0.16562
MAP1LC3B	0.088136893	0.134145686	-0.22548	0.012231	0.007611	-0.07708
MAP1LC3B2	-0.031391085	-0.017944805	-0.07608	0.01988	-0.03076	0.039992
MAP1LC3C	-0.030205025	-0.031206603	-0.05018	0.007496	-0.02921	-0.09409
MAP1S	0.022736925	-0.043282506	-0.2228	0.035042	-0.11066	0.185382
MAP2	-0.040778838	-0.064664301	-0.14746	0.001516	0.014393	-0.26519
MAP2K1	0.089634099	0.036721342	-0.13752	0.030654	-0.07417	0.31769
MAP2K2	0.002775358	0.067701519	0.04766	0.036848	-0.11125	0.021343
MAP2K4	0.029562254	0.048850111	-0.23361	0.043359	-0.10603	0.000425
MAP2K5	0.009206408	-0.038036657	0.067253	0.005808	0.052446	0.070979
MAP2K6	0.005785187	-0.024226781	0.053067	-0.04152	0.143879	0.016513
MAP2K7	0.050760271	-0.000606205	-0.00584	0.055279	0.048819	-0.01631

MAP3K1	0.021085079	-0.069472701	-0.27918	-0.08996	0.268417	0.227348
MAP3K10	-0.003744062	-0.09179296	-0.00958	-0.01142	-0.08262	-0.13951
MAP3K11	0.022397948	-0.138779167	-0.22588	-0.02983	0.080798	0.261189
MAP3K12	-0.064107118	0.031674669	0.164366	-0.00337	0.036867	0.165639
MAP3K13	0.007304764	-0.143623859	-0.04234	0.017815	-0.00295	0.025799
MAP3K14	0.01546024	-0.093423046	-0.07806	0.003094	-0.0185	-0.03173
MAP3K14-/	-0.032030804	-0.027068683	0.104438	-0.00585	0.024503	-0.08527
MAP3K15	-0.036116021	0.002082769	0.04197	-0.01144	0.055096	-0.02594
MAP3K19	-0.030945556	-0.081302808	0.009411	0.00852	-0.08129	-0.02313
MAP3K2	0.010246849	0.013753265	-0.20277	-0.01498	0.11437	0.092253
MAP3K3	0.015439893	-0.025796003	-0.26704	-0.02004	0.114722	0.374819
MAP3K4	0.032471546	0.060993344	0.165621	0.022366	-0.04156	-0.04067
MAP3K5	0.044488973	0.046707662	-0.38076	-0.02487	0.089845	0.212434
MAP3K6	-0.004083447	-0.059270958	-0.09636	-0.08722	0.055054	0.145897
MAP3K7	-0.01215328	0.125129596	-0.06206	-0.0003	-0.0654	-0.11609
MAP3K7CL	-0.003378219	-0.039924761	-0.14489	-0.16623	0.496099	0.45434
MAP3K8	0.023469196	0.171958227	-0.34645	-0.02273	0.06253	-0.11485
MAP3K9	-0.019524646	-0.022404619	0.117003	-0.01184	0.02806	-0.28728
MAP4	0.010473171	0.004493295	0.062577	0.033202	-0.18788	-0.16407
MAP4K1	0.02084014	-0.036819086	0.13652	0.027577	-0.06828	-0.17158
MAP4K2	0.005666291	-0.11928323	0.085302	-0.04902	0.146502	-0.05922
MAP4K3	0.005451218	-0.004848209	-0.02351	-0.02961	0.112128	0.192274
MAP4K4	0.003844758	-0.088684886	-0.08996	0.049385	0.000367	0.048291
MAP4K5	0.00744741	0.10566126	0.026756	0.025836	-0.06532	-0.17481
MAP6	-0.06753781	-0.072037936	0.045991	-0.0338	0.017001	-0.07165
MAP6D1	0.032282876	-0.138621464	0.016304	0.072093	-0.03191	0.157619
MAP7	0.008459664	-0.089750935	-0.15983	-0.04385	0.119461	0.083976
MAP7D1	0.038054338	0.057101786	0.017767	0.019133	-0.03813	0.069842
MAP7D2	-0.022563072	0.000123559	-0.0685	0.040431	-0.10986	-0.04563
MAP7D3	-0.008136704	0.010523066	-0.05612	0.008451	-0.08633	0.047301
MAP9	0.020025008	0.060884573	0.23162	-0.06312	0.09301	-0.4275
MAPK1	0.034886115	0.213054698	-0.32279	-0.01028	0.03308	0.158584
MAPK10	-0.054764178	-0.025913545	-0.11919	-0.02107	-0.00588	-0.01408
MAPK11	-0.017406887	-0.107793527	-0.03248	0.101076	-0.23255	0.063469
MAPK12	-0.06038061	-0.069484296	-0.08214	0.140559	-0.08193	0.004507
MAPK13	0.004446338	0.050053068	0.105069	-0.01084	-0.03793	-0.27373
MAPK14	0.013906115	0.019708273	-0.21651	-0.02488	0.06913	0.238475
MAPK15	-0.038531631	-0.055735688	-0.18329	-0.00347	-0.01575	0.156304
MAPK1IP1l	0.05563195	0.088774162	0.000302	0.049997	-0.1401	-0.13
MAPK3	0.037202191	-0.066387876	-0.02263	0.011086	-0.06536	-0.02001
MAPK4	-0.073563646	0.013600598	0.038344	-0.00725	-0.01266	-0.12937
MAPK6	0.019095981	-0.002839573	0.079055	0.078702	-0.21193	0.058987
MAPK7	0.011522549	0.111656915	-0.11588	-0.01753	0.054937	0.3115
MAPK8	0.022501492	0.059276518	-0.04301	0.017745	-0.27159	0.005905
MAPK8IP1	-0.032082267	-0.044404752	-0.05403	0.002072	0.023776	-0.06122
MAPK8IP2	-0.02986566	-0.060092728	-0.05391	0.037634	-0.11484	-0.01579
MAPK8IP3	-0.046137697	-0.060761577	0.021007	-0.06752	-0.0312	-0.04791
MAPK9	-0.005546086	0.008972246	0.05556	0.033787	-0.10743	-0.12354
MAPKAP1	-0.050659854	-0.079591697	0.055798	-0.03729	-0.00394	0.098898
MAPKAPK2	-0.002663327	-0.040539303	0.006242	-0.02895	-0.06425	-0.15617
MAPKAPK3	0.038562843	-0.055409813	-0.32204	0.027296	-0.0618	0.428522

MAPKAPK5	0.020136545	0.012317601	0.080312	0.030906	-0.0659	0.16524
MAPKAPK5-	0.004499474	-0.059535427	0.337933	-0.06603	0.093375	0.199222
MAPKBP1	0.006103058	0.065414841	0.117931	0.04173	-0.10116	0.022961
MAPRE1	0.079783533	0.176982044	-0.12801	0.090684	-0.22427	-0.0527
MAPRE2	0.013153138	0.169578599	0.212314	-0.05998	0.177343	-0.25146
MAPRE3	-0.034524159	-0.142930639	-0.01905	-0.05622	0.15947	-0.02671
MAPT	-0.037547534	-0.051352476	0.218533	0.022723	-0.10911	-0.09982
MAPT-AS1	0.008916836	-0.030291553	-0.0478	-0.0197	-0.0232	-0.0967
MARC1	-0.000914777	-0.120357048	-0.19645	-0.08248	0.189605	0.477889
MARC2	0.004693733	-0.086653966	-0.16621	0.073787	0.092664	-0.08979
1-Mar	-0.007472957	-0.175733924	-0.38877	-0.10855	0.268652	0.403753
10-Mar	-0.050525279	-0.039151651	0.027498	-0.00109	0.042469	-0.13581
11-Mar	-0.033329907	-0.075075129	-0.07281	0.007621	-0.00963	-0.09364
2-Mar	0.015457945	-0.018899032	-0.19855	-0.08463	0.256677	0.211591
3-Mar	-0.014629025	-0.02180718	-0.08609	-0.02537	0.041006	-0.23216
4-Mar	-0.033431251	-0.023801629	-0.06308	0.028272	-0.0645	-0.00133
5-Mar	0.038334303	0.037365068	-0.13235	0.040949	-0.10001	0.104255
6-Mar	-0.032418474	0.047402699	0.098904	-0.05279	0.191185	-0.22879
7-Mar	0.00167566	-0.019558867	-0.09615	-0.00241	0.032258	-0.03059
8-Mar	-0.013470902	0.004832105	-0.11634	-0.08292	0.168945	-0.10539
9-Mar	0.003601389	-0.064803269	0.155615	0.029637	-0.06046	-0.36055
MARCKS	0.028193128	-0.152596889	-0.34431	-0.11069	0.274002	0.359965
MARCKSL1	0.027504511	-0.083062764	-0.19603	-0.10808	0.269171	-0.11409
MARCO	-0.007613402	-0.07716672	-0.22527	-0.05729	0.17122	0.630659
MARK1	-0.064232354	-0.018592428	-0.0663	0.003768	-0.03152	-0.10313
MARK2	-0.005972199	-0.015018781	0.003552	-0.1087	0.254302	0.020038
MARK3	0.036185744	0.01448173	-0.2351	0.02267	-0.08198	0.103002
MARK4	0.03748089	-0.088063108	0.046636	-0.01488	-0.03462	0.153405
MARS2	-0.016497927	0.020281358	0.145314	0.05731	-0.22829	-0.17074
MARVELD1	0.001885893	-0.14461951	-0.10923	-0.09584	0.261341	0.50055
MARVELD2	-0.020782531	-0.030804622	0.029631	0.000478	-0.00395	-0.10588
MARVELD3	0.001235021	-0.043785276	-0.01113	0.059011	-0.03872	0.118861
MAS1	-0.025870581	0.007894452	-0.00292	0.026332	-0.07798	-0.06066
MAS1L	-0.034835927	-0.022087355	0.083794	0.041371	-0.10437	-0.16423
MASP1	-0.020102504	-0.006762966	-0.0162	0.019062	-0.05753	-0.11836
MASP2	-0.043209759	-0.088212312	-0.00492	-0.00385	0.052268	-0.02277
MAST1	-0.028762465	0.015487525	0.042169	0.018429	-0.03186	0.032108
MAST2	-0.022201529	-0.001103121	-0.00227	0.096203	-0.28923	0.04232
MAST3	0.031907385	0.066317156	-0.12853	-0.04821	0.140682	-0.05208
MAST4	0.03550783	-0.004317805	0.25988	0.114642	-0.17573	-0.38769
MASTL	0.001591546	0.004388113	-0.00923	0.10674	-0.28041	0.209392
MAT1A	-0.026882759	-0.048960321	-0.0021	-0.00709	-0.03624	-0.01512
MAT2A	0.028458847	0.050364323	-0.01188	0.038352	-0.12467	0.088675
MAT2B	0.038936386	0.158635409	0.038306	0.007026	-0.01823	-0.3023
MATK	0.008122916	0.318397703	0.179671	-0.03227	0.069044	-0.2336
MATN1	-0.042738158	-0.058426166	-0.07337	0.009027	-0.04344	-0.00049
MATN1-AS:	-0.010402177	-0.056177711	0.351004	-0.05139	0.166642	-0.18033
MATN3	-0.104297495	-0.047534592	-0.10154	-0.01906	0.071506	0.068748
MATN4	-0.026525609	-0.043898544	-0.02013	0.054807	-0.16128	-0.01798
MATR3	-0.003653594	-0.073224405	0.142239	0.078655	-0.22855	-0.04667
MAU2	-0.070199899	-0.02026023	0.049094	-0.05429	0.084047	-0.01705

MAVS	0.006846815	-0.019092478	-0.0094	0.013073	-0.04413	0.249565
MAX	0.009312044	-0.031522396	0.071577	-0.07788	0.182987	-0.16656
MAZ	0.021920568	-0.032531263	0.183928	0.07132	-0.20143	-0.01317
MB	-0.012436527	-0.000878804	-0.02703	0.031797	-0.09884	0.000393
MB21D1	0.041262111	0.083320778	-0.09676	0.004148	-0.06918	0.10507
MB21D2	0.006172885	-0.061034227	0.070416	0.10385	-0.352	-0.05764
MBD1	0.007981465	-0.055921335	-0.02094	-0.0191	-0.02988	0.031886
MBD2	-0.036523095	-0.060464815	-0.1616	-0.01938	0.056354	0.099904
MBD3	-0.015473484	-0.063241811	0.09265	0.004358	-0.19377	-0.0365
MBD3L1	-0.059776578	-0.064067093	0.044297	0.017985	-0.05732	0.052599
MBD3L2	-0.031313689	-0.059692472	-0.02302	0.036733	-0.09144	-0.05102
MBD4	0.045489417	0.0103096	-0.32162	0.014235	-0.02445	-0.10294
MBD5	-0.025184321	0.048702317	0.152261	-0.09397	-0.02283	-0.15009
MBD6	-0.070812709	-0.119885236	-0.09598	-0.0437	-0.03171	-0.01857
MBIP	0.002339738	0.021498774	0.100755	0.040076	-0.08231	0.033822
MBL2	-0.029575907	-0.071261188	0.098923	0.000331	-0.0277	-0.07144
MBLAC1	-0.011686987	-0.064619821	0.059606	0.022457	-0.14466	-0.07635
MBLAC2	-0.021591126	0.058587613	0.116507	0.026389	-0.02808	-0.10538
MBNL1	0.065592365	0.070055193	0.126064	-0.06352	0.038975	-0.28323
MBNL1-AS1	-0.011652788	-0.011132081	0.053317	-0.16138	0.443903	0.071543
MBNL2	-0.027589673	0.043624764	0.061555	-0.0047	0.114498	0.021568
MBNL3	-0.041090234	0.067039092	0.057372	-0.01117	0.130889	-0.00048
MBOAT1	0.009954624	-0.189084443	-0.20243	-0.01319	0.031702	0.314122
MBOAT2	-0.011135328	-0.069189758	-0.17894	-0.02243	0.024444	-0.01031
MBOAT7	0.01120533	-0.081588505	-0.24474	-0.01324	0.019742	0.107537
MBP	-0.029488112	0.071754975	-0.02571	-0.03055	0.05215	-0.1879
MBTD1	-0.015388271	0.034204057	0.023726	-0.10655	0.229989	0.102893
MBTPS1	0.034620579	0.003441806	0.241879	-0.0383	0.065983	0.073655
MBTPS2	-0.02413582	-0.012693127	-0.03648	0.10711	-0.307	0.066378
MC1R	-0.020275722	-0.035812304	0.049263	0.050366	-0.18333	-0.23619
MC2R	-0.066490108	-0.073350143	0.060968	-0.03185	0.011779	0.011991
MC3R	-0.03643292	-0.050737147	-0.02691	0.005209	0.005122	-0.05472
MC4R	-0.036912311	0.004719444	-0.11291	0.032772	-0.07215	0.036236
MC5R	-0.025415476	-0.021888744	0.065893	0.085951	-0.25088	-0.10163
MCAM	-0.027821013	-0.045580224	0.04977	0.04467	-0.13466	-0.11959
MCAT	4.13E-05	-0.042124297	-0.09648	0.054563	-0.15721	0.160457
MCC	-0.0104978	-0.065924751	0.147914	-0.00596	0.028084	-0.19
MCCC1	0.03108878	-0.073026499	-0.00188	0.022867	-0.06451	-0.027
MCCC2	-0.007723494	-0.010423569	-0.018	0.103594	-0.33458	-0.03946
MCEE	0.017636847	-0.014302935	0.046901	0.010096	0.008252	0.183424
MCEMP1	0.007339848	-0.076334313	-0.2201	-0.07755	0.221723	0.648595
MCF2	-0.060653463	0.267814064	0.102003	-0.04076	0.056425	-0.11952
MCF2L	-0.029107323	-0.105781879	0.302641	-0.05382	0.058184	-0.30057
MCF2L-AS1	-0.018766786	-0.088538929	0.289537	-0.05012	0.172122	-0.15212
MCF2L2	-0.08000387	-0.09050403	-0.03405	-0.00324	0.058004	0.04649
MCFD2	0.019758633	-0.057969747	-0.16277	0.034221	-0.09688	0.293915
MCHR1	-0.037693216	0.010336737	-0.01031	0.01131	-0.12501	0.089145
MCHR2	-0.039646577	-0.016449403	-0.04906	0.006488	-0.04587	0.124646
MCHR2-AS1	-0.095986836	-0.150380259	-0.09503	0.022348	0.00411	0.017259
MCL1	0.053334189	-0.049317343	-0.18554	-0.04161	0.065085	0.143488
MCM10	0.010623868	0.031806487	0.004349	0.081165	-0.35084	-0.13174

MCM2	0.006137789	0.078609139	0.035651	0.126577	-0.39118	-0.27954
MCM3	0.023661735	0.117825438	0.173789	0.083481	-0.25615	-0.29622
MCM3AP	0.001245429	-0.000485729	-0.13628	0.019818	-0.07683	-0.08351
MCM3AP-AS	-0.012053577	0.146124887	0.144347	0.030543	0.025632	-0.29995
MCM4	-0.034350816	0.007422718	-0.09734	0.281014	-0.50412	0.044804
MCM5	0.018784509	0.01717519	-0.17203	0.077559	-0.21783	0.01835
MCM6	0.012251452	0.102007449	0.229783	0.110018	-0.29249	-0.31152
MCM7	0.026366488	0.084325155	0.112874	0.099526	-0.27958	-0.23441
MCM8	0.00244071	0.148028594	0.07863	0.13112	-0.28265	-0.25156
MCM9	-0.004471622	0.011857181	0.010666	0.041854	-0.13993	-0.09386
MCMBP	0.030443793	0.06331016	-0.22215	-0.00163	0.003991	0.317727
MCMDC2	-0.063307061	-0.036323944	-0.03525	-0.02049	0.047372	-0.05283
MCOLN1	0.005904942	-0.073399664	-0.28054	-0.02277	0.028674	0.339823
MCOLN2	0.004961456	-0.101486235	0.108312	0.032031	-0.1337	-0.32392
MCOLN3	-0.055602647	-0.126435278	0.034403	0.003002	-0.01212	-0.08605
MCPH1	0.058861505	0.076093502	0.10551	0.002351	-0.0755	-0.06647
MCRS1	0.044264504	0.10956704	-0.03366	0.01401	-0.08487	0.123664
MCTP1	0.00860451	-0.10405519	-0.27885	-0.10918	0.229811	0.565326
MCTP2	0.031695685	0.275626996	-0.12175	-0.00843	-0.17545	-0.24125
MCTS1	0.000885934	-0.047037528	0.174012	-0.02869	0.076671	-0.13789
MCU	0.015733555	-0.041778022	-0.22264	-0.01431	-0.02168	0.103654
MCUR1	0.024963287	0.016759112	0.014901	0.004837	0.000199	0.016986
MDC1	0.013033061	0.154479963	0.182149	0.083219	-0.22422	-0.44046
MDFI	-0.033141934	-0.030597087	-0.0877	-0.00361	-0.01989	-0.04945
MDFIC	-0.012133548	-0.030743797	0.080684	########	-0.07772	-0.05069
MDGA1	-0.025422023	-0.065552578	-0.04601	0.018978	-0.08716	0.018217
MDGA2	-0.13209723	-0.058377505	0.011058	-0.01038	0.095131	-0.12229
MDH1	0.057971971	0.082824101	0.183248	0.054701	-0.15373	0.032273
MDH1B	-0.040341491	-0.053850055	-0.02436	0.041713	-0.11259	-0.03973
MDH2	0.044140319	0.036316038	0.071915	0.070934	-0.20785	0.202598
MDK	-0.032876184	-0.06610932	-0.00826	0.009941	-0.03373	-0.01468
MDM1	0.007110128	0.067043061	0.036457	-0.03457	0.116792	0.045449
MDM2	-0.042349128	-0.038220618	-0.09435	0.237984	-0.39034	-0.0154
MDM4	-0.032788974	-0.027171409	-0.03071	0.005626	-0.06478	-0.1703
MDN1	-0.031945804	-0.06660985	0.181874	0.036098	-0.02776	-0.21728
MDS2	-0.014429117	-0.14444928	0.345367	-0.0399	0.117658	-0.33587
ME1	0.034396724	0.076478074	0.085585	-0.04596	-0.00307	0.15157
ME2	0.058173713	0.038421772	-0.19365	0.040764	-0.07846	0.073868
ME3	-0.024937443	0.018692918	0.151007	0.003912	0.021729	-0.05727
MEA1	0.068792541	0.093439562	0.057708	0.079062	-0.20547	-0.0576
MEAF6	-0.007357898	0.061287674	-0.01694	-0.00717	-0.01736	0.022932
MECOM	-0.044731192	-0.046147359	-0.05338	-0.00496	-0.0219	-0.02113
MECP2	0.045779151	0.106116563	0.117735	-0.02349	-0.02229	-0.21697
MECR	-0.006439323	-0.038746394	0.110769	0.107308	-0.32672	0.049898
MED1	0.047497693	0.155108104	0.178045	0.015295	-0.1267	-0.14377
MED10	0.048761023	0.07738428	0.335189	0.033762	-0.15212	-0.25539
MED11	0.044387464	0.04112837	0.023695	0.029613	-0.10808	-0.18002
MED12	0.039626145	0.056778244	-0.03733	0.00788	-0.04384	-0.15332
MED12L	-0.024179329	-0.058700601	-0.01798	-0.00327	-0.01105	-0.09285
MED13	0.009011486	0.089226693	-0.08703	0.033648	-0.06494	-0.14461
MED13L	-0.021166686	-0.03058367	-0.28956	-0.04496	0.082747	-0.01335

MED14	-0.002350943	-0.016867402	-0.06311	0.054968	-0.05687	0.014907
MED14OS	-0.02249812	-0.055701248	-0.19977	-0.01208	0.04941	0.331742
MED15	0.03354254	0.065706867	0.253366	-0.01424	0.022238	-0.09024
MED15P9	-0.039550456	0.004927847	-0.07767	0.035311	-0.07848	0.003701
MED16	0.048223819	-0.044734622	0.084477	0.060725	-0.05712	-0.05535
MED17	-0.08330126	0.076607056	0.197884	-0.04701	0.082518	-0.09615
MED19	0.043831366	0.072422289	0.090322	0.006727	-0.06641	0.0961
MED20	0.007275258	0.073294894	-0.26909	0.044301	-0.16302	0.200088
MED22	0.004161571	-0.004056149	-0.07101	-0.0009	0.002342	0.070062
MED23	0.013617132	0.067118264	0.08794	0.021932	-0.0198	-0.2199
MED24	-0.027628685	-0.022013019	-0.03783	0.025402	-0.05884	0.004913
MED25	-0.00101699	-0.106740974	-0.13685	-0.02355	0.018822	-0.13433
MED26	0.02220689	-0.040984162	-0.06748	0.029031	-0.07062	-0.07208
MED27	0.022270866	0.008181932	0.109269	0.107628	-0.31013	0.002615
MED28	-0.029226022	0.003446747	-0.02782	-0.06287	-0.00141	-0.05636
MED29	0.024450506	0.076360885	0.055601	-0.013	0.064424	0.13642
MED30	0.018211301	0.078891451	0.009032	0.078505	-0.21961	-0.09116
MED31	-0.002152274	-0.06675272	0.155961	-0.02514	0.097248	-0.06942
MED4	0.031729445	0.091137421	-0.01657	0.047554	-0.12761	0.082088
MED6	0.035616405	0.067475893	0.070646	0.068288	-0.15256	-0.04428
MED7	0.056242307	0.121202476	0.033398	0.053737	-0.10044	0.051813
MED8	0.094292681	0.059283061	-0.24088	0.0538	-0.13696	0.290557
MED9	-0.004637174	-0.004815911	0.079293	0.045589	-0.17346	-0.0785
MEDAG	-0.037226173	-0.017426811	-0.02966	0.012958	-0.08102	-0.14942
MEF2A	0.024482341	-0.130630328	-0.34736	-0.05425	0.152294	0.443929
MEF2BNB	0.021219719	-0.070284488	-0.30527	0.012176	-0.06335	0.223269
MEF2C	0.017289551	-0.086717935	-0.41889	-0.07743	0.24029	0.174315
MEF2C-AS1	-0.034840374	-0.053909953	0.016701	-0.00646	0.034887	-0.06252
MEF2D	-0.008434544	-0.0592001	-0.08078	0.064749	-0.12459	0.01904
MEFV	-0.011133118	-0.05638531	-0.09904	-0.01555	-0.00661	-0.03436
MEG3	-0.074932489	-0.097846359	-0.1173	-0.04281	-0.06235	0.203691
MEG9	-0.04892417	0.01129322	-0.12681	-0.01067	0.06623	0.085558
MEGF10	-0.081061007	-0.125907059	0.083658	-0.07565	-0.04827	-0.01475
MEGF11	-0.036396977	0.028710659	-0.06333	-0.01203	0.062704	0.057203
MEGF6	-0.010576965	-0.2269481	0.150977	-0.05443	0.125341	-0.2655
MEGF8	-0.015176203	-0.034527565	-0.00723	0.022439	-0.10449	0.053182
MEGF9	0.013408021	-0.007153744	-0.32354	-0.06343	0.194437	0.436554
MEI1	0.028070154	0.08984158	0.289931	0.026489	-0.0299	-0.19445
MEIG1	-0.029885959	0.02509245	0.053931	0.097128	-0.26307	0.021838
MEIS1	-0.013632975	0.027959069	-0.09801	-0.23832	0.673763	-0.01128
MEIS2	-0.016416436	-0.03362765	-0.08138	0.10419	-0.16874	-0.09513
MEIS3	-0.026621246	-0.062180257	0.036879	-0.00948	-0.08462	-0.10485
MEIS3P1	-0.009697085	-0.089995263	0.083435	-0.13154	0.366085	0.113303
MELK	-0.003652667	0.017119765	-0.00172	0.139105	-0.33674	-0.06379
MEN1	0.017902262	0.046428905	0.199833	-0.02627	0.08837	-0.05449
MEOX1	-0.020775663	-0.048061907	0.11504	-0.11022	0.335858	-0.13107
MEOX2	-0.128235911	-0.074645562	-0.06838	-0.04089	0.120102	0.053434
MEP1A	-0.03601945	-0.104599534	0.118669	0.001281	0.015542	-0.03479
MEP1B	-0.031362571	0.020892512	0.01288	0.010113	-0.01012	-0.08908
MEPCE	0.068309201	0.00773745	-0.08027	0.025099	-0.06199	-0.19502
MEPE	-0.032092156	-0.055463813	0.003839	0.007583	-0.03035	-0.01905

MERTK	0.009733221	-0.045824488	-0.15313	-0.05245	0.375201	0.63584
MESDC1	0.032805813	0.10400647	-0.07965	0.060486	-0.19156	-0.03087
MESDC2	0.070364271	0.121846156	0.042464	0.075982	-0.19705	-0.03851
MESP1	-0.062799975	0.019910987	0.052429	0.042405	-0.18944	0.009523
MESP2	-0.022445046	-0.050727421	0.000119	-0.02497	-0.04956	-0.10723
MEST	-0.00638297	-0.116442435	0.356421	-0.12426	0.351157	-0.07807
MET	-0.045654973	-0.058023227	0.010907	-0.01325	-0.07148	-0.0563
METAP1	0.052527189	0.123217877	0.119063	0.062807	-0.19452	-0.01756
METAP1D	-0.007775064	-0.019291969	0.229377	0.02819	-0.03902	-0.16078
METAP2	0.027235077	0.0224366	0.100546	0.045429	-0.07883	-0.02066
METRN	0.004788858	0.009969938	0.059271	-0.03654	0.00748	0.008798
METRNL	0.015103205	0.14936742	-0.20766	0.002355	-0.00053	0.210143
METTL1	-0.004960582	-0.060225106	0.068831	0.024112	-0.11341	-0.03834
METTL10	-0.014751455	-0.111822529	0.251407	0.125836	-0.0978	-0.15162
METTL13	0.029775506	0.06636644	0.011955	0.032703	-0.12341	-0.00456
METTL14	-0.010436306	0.077705186	0.105502	0.017153	-0.058	-0.1825
METTL15	-0.068587792	0.037329827	-0.01131	0.053009	-0.09471	-0.05602
METTL17	0.038195495	-0.002521594	0.229653	0.025688	-0.06593	-0.147
METTL18	0.010915974	0.112577315	0.272537	0.004431	-0.05652	-0.29774
METTL20	-0.056746995	-0.032132189	0.04431	-0.06323	0.077929	-0.03465
METTL21A	0.008911186	-0.045721066	-0.09817	0.024838	0.042356	0.199778
METTL21B	-0.001242238	-0.057098898	-0.01434	0.010646	-0.04049	0.204711
METTL21C	-0.044734867	-0.029653603	-0.03141	-0.02334	0.070685	-0.00125
METTL21EI	-0.024231523	-0.032469718	0.031904	-0.00383	0.014791	-0.11975
METTL22	0.017197817	-0.111525818	-0.19375	-0.01545	-0.01529	0.319316
METTL23	0.048087254	0.073116823	0.351195	0.009977	-0.03545	-0.1306
METTL25	0.019114255	-0.02585712	-0.02529	-0.03873	0.098143	0.02816
METTL2B	-0.011814301	0.06919349	0.206647	0.020311	-0.03762	-0.04683
METTL3	0.037378712	0.023319542	0.157154	-0.02711	0.097749	-0.18377
METTL4	-7.27E-05	0.01029211	0.173504	0.02632	-0.08141	-0.2547
METTL5	0.019597249	0.038581939	0.210272	0.051724	-0.12854	-0.00399
METTL6	-0.042936255	-0.013458152	0.035116	0.009384	-0.10746	-0.12453
METTL7A	0.015348134	0.019045978	-0.30151	-0.00715	0.092698	0.127974
METTL7B	-0.028562704	0.001653552	-0.17709	0.017788	-0.06582	0.116416
METTL8	0.01606935	0.018059737	0.033992	0.047876	-0.0283	-0.18441
METTL9	0.01872014	-0.080418063	-0.25043	-0.0578	0.185159	0.607378
MEX3A	-0.016536186	-0.020957051	0.060384	0.029899	-0.1152	-0.05661
MEX3B	-0.031770106	-0.021130999	0.026272	0.03388	-0.08054	-0.00425
MEX3C	-0.084954947	0.121605529	-0.07578	-0.05756	0.01443	-0.14133
MEX3D	-0.007307928	0.150681271	-0.0454	0.066701	-0.05782	0.028202
MFAP1	0.048854688	0.174086674	-0.10578	0.079148	-0.21518	-0.17933
MFAP2	-0.034535411	-0.05044672	-0.07382	0.041979	-0.12912	0.05744
MFAP3	-0.034250321	-0.018118394	-0.07074	-0.00544	-0.10502	-0.06309
MFAP4	-0.035530586	-0.066365291	-0.05972	0.015625	-0.03619	0.180863
MFAP5	-0.033059502	-0.116139705	-0.03546	0.020103	-0.0011	-0.05567
MFF	0.022422812	-0.018970062	0.023574	0.003654	-0.03028	0.057879
MFGE8	-0.015802032	-0.131947609	0.140928	0.007209	-0.10419	-0.12647
MFHAS1	0.020404684	0.06331387	0.2701	0.038306	-0.14188	-0.15909
MFI2	-0.056139118	-0.029697899	-0.04088	0.007488	-0.09195	-0.09831
MFI2-AS1	-0.037726898	-0.011534778	0.052784	0.029901	-0.08919	-0.14462
MFN1	-0.002144728	0.010873825	-0.19346	0.05842	-0.1127	-0.2621

MFN2	-0.018112529	0.001847676	-0.13491	-0.01435	0.002441	0.130677
MFNG	0.002670779	-7.61E-05	0.176567	-0.0341	0.034206	0.019176
MFSD1	0.03918315	-0.063858942	-0.30259	-0.12914	0.406307	0.31023
MFSD10	0.028055285	-0.007289715	0.151885	-0.014	-0.02662	-0.01573
MFSD11	0.052992217	-0.013075883	0.109835	-0.02349	-0.00247	-0.04089
MFSD12	0.02282875	-0.207343712	0.08498	-0.02538	0.04167	0.120821
MFSD2A	-0.004435353	-0.048675796	-0.07333	-0.00541	-0.00632	0.040193
MFSD3	-0.019699221	-0.089967667	-0.03007	0.071088	-0.18041	0.058066
MFSD4	-0.057253606	-0.058957264	0.135289	0.070584	-0.10583	-0.15523
MFSD5	0.042837491	0.063845073	-0.31088	0.013263	-0.06303	0.160352
MFSD6	0.01319772	0.109959538	0.203159	0.046187	-0.10969	0.139511
MFSD6L	-0.032871989	-0.04059974	-0.01957	-0.0211	-0.01613	-0.0194
MFSD7	-0.035612988	-0.1091203	-0.13967	-0.04415	0.070133	0.32847
MFSD8	0.035717432	0.042018549	0.106012	-0.0768	0.107419	-0.05469
MFSD9	-0.003934627	0.071289984	0.016776	0.012295	-0.0088	-0.17282
MGA	0.002141995	0.065535137	0.121386	-0.01774	0.060637	-0.02906
MGAM	-0.002590287	0.126908804	-0.21841	-0.032	0.08496	0.007631
MGARP	-0.029642916	0.018811626	0.005812	-0.02071	0.081391	0.0119
MGAT1	0.066741972	0.035274112	-0.31257	-0.00576	0.040863	0.441362
MGAT2	0.085311738	0.137362042	-0.00368	0.041889	-0.1181	0.071901
MGAT3	-0.04447761	-0.138259001	-0.0873	-0.01635	-0.04392	-0.04015
MGAT4A	-0.001653941	0.103623275	0.300759	-0.02798	0.152299	-0.21763
MGAT4B	0.027441578	-0.017338003	-0.04316	-0.01969	0.039889	0.170178
MGAT4C	-0.072004078	-0.058616659	-0.05078	-0.02067	0.049	-0.07525
MGAT5	0.027874183	0.019835384	0.014042	-0.00164	0.011804	-0.36461
MGAT5B	-0.039767043	-0.087919818	-0.02134	0.038834	-0.12482	-0.10367
MGC10814	-0.023890509	-0.076593606	-0.00149	0.010894	-0.03318	0.011784
MGC12488	0.002192825	0.029810674	0.00898	0.008943	0.013226	0.048927
MGC12916	-0.013362022	0.08274176	-0.02095	0.03751	-0.08995	-0.13894
MGC15705	-0.041478453	-0.059497052	0.176214	0.011546	-0.00994	0.074798
MGC15885	-0.032040023	-0.049841735	0.012612	0.028386	-0.08178	-0.1452
MGC16025	-0.047059176	-0.016777766	-0.04808	0.021373	-0.05689	0.07506
MGC16142	-0.029873009	0.085757047	-0.04141	-0.0443	0.151651	0.000933
MGC16275	-0.011737323	-0.121798024	-0.02599	-0.08866	0.265634	0.382588
MGC20647	-0.039538567	-0.047465663	-0.00243	0.02213	-0.05322	-0.00819
MGC24103	-0.007420882	0.399397163	-0.0903	-0.0031	0.024079	-0.16298
MGC27382	-0.079648014	-0.051136909	0.092484	-0.02321	0.058426	0.031816
MGC2889	-0.020940311	-0.006254931	0.028906	0.013272	-0.04259	-0.06623
MGC32805	-0.03961581	-0.09246496	0.095608	0.003318	-0.05846	-0.09596
MGC34796	-0.022918947	-0.102762555	-0.05731	0.004221	-0.09974	-0.0042
MGC34800	-0.036052131	-0.035466986	0.092813	0.005872	-0.00031	0.018792
MGC39545	-0.084959093	-0.061458108	0.021601	-0.04749	0.124883	0.049298
MGC39584	-0.033901739	-0.059487445	0.063342	-0.02262	0.080828	-0.15408
MGC40069	-0.018645478	-0.145926598	0.490641	-0.1159	0.350428	-0.31695
MGC4294	-0.02209401	-0.024769113	-0.0114	0.00798	-0.03323	-0.00127
MGC45800	-0.030390788	-0.048634201	-0.01379	0.086879	-0.23155	0.041858
MGC45922	-0.017432922	-0.109327372	-0.04416	0.024671	-0.1406	-0.11817
MGC4859	-0.050093284	-0.057509751	-0.09855	0.009727	-0.02689	-0.00707
MGC50722	-0.03222637	-0.085846323	0.013933	0.022977	-0.04861	0.05809
MGC57346	-0.028504559	0.017814989	0.211964	0.085136	-0.21967	-0.16485
MGC70870	-0.000953288	0.081956129	-0.16132	-0.06315	0.180034	0.052815

MGEA5	0.063354806	0.097873551	0.03622	-0.05136	-0.05593	-0.42821
MGLL	0.010524207	-0.087869146	-0.15398	-0.08727	0.24141	0.155368
MGME1	0.023017833	-0.001294422	-0.29496	0.034287	-0.08689	0.01354
MGMT	0.013808101	0.040795231	0.090325	-0.06544	0.189021	0.166541
MGP	-0.0583992	-0.136200816	0.349233	-0.02954	0.081564	-0.15198
MGRN1	0.056018213	-0.049670892	-0.04082	0.042451	-0.14913	0.128346
MGST1	-0.046190618	-0.139711965	-0.15268	-0.13034	0.281832	0.759554
MGST2	0.019467179	-0.018660137	-0.34564	-0.03524	0.087361	0.412379
MGST3	0.031054547	-0.013513392	0.089609	0.030082	-0.06987	-0.11938
MIA	-0.025122795	-0.146943989	0.048579	0.002102	-0.07545	-0.07886
MIA2	-0.073195821	-0.054490607	0.028547	-0.02962	0.081855	0.000729
MIA3	-0.007738542	-0.015074243	-0.06234	0.005585	0.08294	-0.15259
MIAT	0.00044595	0.099787912	0.326952	0.006644	0.039816	-0.36171
MIATNB	-0.002959074	-0.066837753	0.176903	-0.0628	0.07158	-0.28234
MIB1	0.024164846	0.118844432	-0.08809	0.046498	-0.16777	0.131348
MIB2	0.08834882	-0.067724462	0.121294	-0.02876	0.035092	0.043182
MICA	0.010875681	0.006109231	0.045772	-0.03545	0.077483	0.079395
MICAL1	0.052502574	-0.156645917	-0.26848	-0.02053	0.070465	-0.0647
MICAL2	-0.000620529	-0.004591738	-0.18398	0.013369	-0.02096	0.42154
MICAL3	-0.008411005	-0.005755911	0.016062	-0.043	0.142792	-0.27515
MICALCL	-0.015201238	-0.074050568	-0.14713	-0.06815	0.206659	0.49823
MICALL1	0.030062555	0.095296797	0.027281	0.048675	-0.15591	-0.13061
MICALL2	-0.022037798	-0.055126875	-0.08819	-0.01438	0.04225	0.229663
MICB	0.048046722	0.122342553	-0.10711	0.047003	-0.13911	0.27307
MICU1	0.028620962	-0.111602251	-0.30947	-0.00803	0.008528	0.395491
MICU2	0.102944089	0.196627133	0.060605	0.056403	-0.1302	-0.22099
MICU3	-0.005551862	0.013175087	0.206253	-0.02209	0.085814	-0.29577
MID1	-0.010423992	-0.04503153	-0.01046	0.030997	-0.12305	-0.0089
MID1IP1	0.019021692	-0.213617258	-0.0744	0.009674	-0.06922	0.204743
MID2	-0.016774031	-0.098605276	0.410791	-0.06158	0.114435	-0.31608
MIDN	-0.030920838	-0.018645109	-0.05868	-0.02543	0.085114	0.075092
MIEF1	0.00619561	0.031903204	0.173965	0.023373	-0.23531	0.113168
MIEF2	-0.02261171	0.013797335	0.047471	-0.00211	-0.04087	-0.08262
MIEN1	0.082601551	0.236207616	-0.14482	0.045273	-0.11655	0.196184
MIER1	0.006994447	0.018332501	-0.02914	-0.00525	-0.02874	-0.01573
MIER2	-0.002113591	-0.110394103	-0.11088	-0.01566	0.103249	0.325771
MIER3	-0.000903417	0.101310154	0.069846	0.079575	-0.13335	-0.16881
MIF	0.018012508	0.056838453	0.264007	0.076338	-0.21264	-0.04416
MIF4GD	0.027762211	0.031884121	-0.00762	0.024185	-0.05468	0.099471
MIIP	0.033685242	-0.12142854	-0.09823	0.055301	-0.20147	0.142984
MILR1	-0.002132182	-0.108606905	-0.37972	-0.06484	0.20219	0.505342
MINA	0.002474973	-0.051066484	0.08307	0.090441	-0.2492	0.085837
MINK1	-0.00897898	-0.120502397	0.011462	-0.04012	-0.04052	0.050333
MINOS1	0.054720385	0.081397721	0.181235	0.032585	-0.08492	0.044995
MINOS1P1	-0.008177762	0.010452173	0.043597	0.018156	-0.03588	-0.13669
MINPP1	0.008027084	0.088768073	-0.0259	0.015605	-0.02451	0.1283
MIOS	0.031286443	0.030296448	-0.005	0.003642	-0.01112	0.070897
MIOX	-0.036062394	-0.0490529	0.009626	0.007174	-0.00994	0.028815
MIP	-0.049279945	-0.011937215	0.060388	-0.00635	0.048217	0.109606
MIPEP	-0.004290054	0.020105142	0.095256	0.056818	-0.14432	-0.07869
MIPEPP3	-0.02198351	0.000195295	0.027938	0.008283	-0.02184	-0.09892

MIPOL1	-0.100836464	-0.091977949	-0.01637	-0.01245	0.044973	-0.06712
mir-223	0.004784941	-0.139889827	-0.34509	-0.08455	0.237683	0.323331
MIR100HG	-0.101604137	-0.041585208	0.001932	-0.03716	0.099443	0.053007
MIR10A	-0.018465733	0.020654269	0.007942	0.006608	-0.00177	0.004961
MIR124-2I	-0.026848184	0.026154872	0.031759	0.011268	-0.06812	-0.00749
MIR1244-3	0.082593191	0.131064576	0.09432	0.069176	-0.15689	-0.10345
MIR133A1I	-0.055371681	0.013267693	-0.01989	0.008516	-0.00088	0.04827
MIR142	0.032760667	0.012221618	-0.13229	-0.0202	0.032551	-0.12696
MIR143HG	-0.052039094	-0.04412259	-0.01409	-0.00232	0.034722	-0.03565
MIR146A	-0.027499665	-0.044109769	0.064951	0.0035	-0.01313	0.025836
MIR17HG	-0.033449959	-0.054256299	0.089477	-0.00535	-0.00762	-0.11049
MIR194-2	-0.028939886	-0.076019836	0.094011	-0.00258	-0.04565	-0.01861
MIR205	-0.052157087	-0.023115769	-0.13922	-0.03217	0.12346	0.134596
MIR205HG	-0.036838521	0.041801959	-0.07099	0.018336	-0.02908	0.042253
MIR210HG	-0.021818903	-0.005315695	0.001303	0.064008	-0.19764	-0.08427
MIR302B	0.0100833	0.030178991	-0.0324	0.018591	0.007098	0.172835
MIR31HG	-0.031292747	-0.093765843	0.057089	-0.00737	-0.004	-0.14305
MIR34A	-0.015833054	0.003523257	-0.04204	0.016312	-0.02948	0.060578
MIR3663H(	-0.065555934	-0.055508183	0.053306	-0.02614	0.06374	-0.06161
MIR3682	-0.002986886	0.107291221	0.027726	-0.00142	0.003187	-0.1692
MIR3916	-0.005669047	0.092106737	0.017973	-0.00158	0.048181	-0.07836
MIR4296	-0.032907071	0.115950943	-0.19893	-0.02429	0.091917	0.231177
MIR4313	-0.037776716	-0.012898833	0.09016	0.028514	-0.06984	0.140661
MIR4435-1	-0.014434367	0.120184708	-0.00103	0.010642	-0.01206	-0.02715
MIR4500H(	-0.017737321	-0.009424747	-0.00563	0.093083	-0.16304	0.016082
MIR4755	-0.023206501	-0.067702572	-0.03257	-0.01392	-0.01641	-0.12201
MIR5188	-0.027601308	-0.039021195	-0.08397	0.011873	-0.03124	0.102105
MIR622	-0.032974212	-0.073717591	-0.04022	0.051233	-0.13107	0.052028
MIR646HG	-0.009610284	0.11997878	-0.16812	-0.02763	0.084154	0.127895
MIR663AH(	-0.065420545	-0.097421094	0.216092	-0.00845	0.00894	-0.05374
MIR670HG	-0.033108289	-0.026551272	-0.13734	0.012538	-0.06104	0.010655
MIR7-3HG	-0.044261235	-0.072015671	0.015435	0.011836	0.000334	0.080371
MIRLET7BI	-0.029546918	-0.023944268	0.017055	-0.03292	0.074999	-0.10362
MIRLET7D	0.006408229	-0.034677284	0.060682	-0.00139	-0.03317	0.098998
MIRLET7DI	-0.033831231	-0.016855555	0.126127	-0.0177	0.067498	-0.01324
MIS12	0.026732921	0.156724875	0.225977	0.046742	-0.13318	-0.1877
MIS18A	-0.031041025	0.094815459	0.015186	0.109096	-0.31195	-0.01842
MIS18BP1	0.009705432	-0.040626578	-0.34791	-0.00625	0.037086	0.348526
MISP	-0.009666167	-0.060239543	0.016918	-0.01779	-0.0518	-0.11182
MITD1	0.030277367	0.034948004	0.088321	0.035306	-0.11684	-0.02216
MITF	-0.000865319	-0.083379494	-0.22213	-0.11211	0.33616	0.552941
MIXL1	-0.030931624	-0.072540148	-0.07579	0.002535	0.002248	-0.10796
MKI67	-0.015014349	-0.00240295	0.053797	0.190885	-0.39632	-0.09636
MKKS	0.032456252	-0.001651892	-0.07769	0.006769	-0.02255	0.299732
MKL1	-0.100870986	-0.140126707	-0.03144	-0.0191	-0.08956	0.07169
MKL2	-0.050252329	-0.031812816	0.0263	0.031226	-0.07542	-0.10106
MKLN1	0.006623266	0.151536356	-0.14124	0.038542	-0.10798	0.04379
MKNK1	0.018266435	-0.023735574	-0.14109	0.004235	-0.00909	0.064516
MKNK1-AS1	-0.031693701	-0.048323681	-0.00537	0.005723	-0.02253	0.00401
MKNK2	0.047590292	-0.000619977	-0.0907	-0.00625	0.019896	-0.16078
MKRN1	0.0676383	-0.013820854	-0.1733	-0.03439	0.122895	0.082582

MKRN2	-0.002500486	0.054212813	0.050622	0.001664	-0.07396	-0.14099
MKRN3	-0.028551862	-0.029674124	-0.01946	-0.0071	0.053124	0.010252
MKRN7P	-0.031272652	-0.093477419	-0.12154	0.000933	-0.00218	-0.05691
MKS1	-0.023351869	-0.056145479	0.219602	0.002904	-0.08343	-0.14779
MKX	-0.057930776	-0.071141624	0.008705	-0.01832	-0.0274	-0.05382
MLANA	-0.075105151	0.028295953	-0.05013	0.000205	0.011245	0.006666
MLC1	-0.004945258	0.401462889	-0.1018	-0.07377	0.218769	0.038805
MLEC	0.052000169	0.136549593	-0.17159	0.054562	-0.15782	0.085213
MLF1	-0.01827431	0.078545527	0.233193	-0.02044	0.072526	-0.20197
MLF2	0.036197731	-0.067763527	-0.21706	0.022048	-0.05576	0.007766
MLH1	0.043480699	0.068665989	0.065185	0.075972	-0.20588	0.032739
MLH3	0.011008702	0.078907732	-0.02464	-0.20854	0.556726	0.008021
MLIP	-0.042151732	-0.037952157	0.028839	0.031566	-0.0681	-0.08076
MLK7-AS1	-0.023192579	-0.022464199	-0.00189	0.089248	-0.18882	0.060501
MLKL	0.028421961	0.002483731	-0.15892	-0.03862	0.10573	0.355995
MLLT1	0.030063746	-0.02152141	-0.10262	0.036613	-0.07888	0.068484
MLLT10	0.014781862	-0.015734205	0.07357	0.012152	0.028775	0.15696
MLLT11	0.010135795	0.138821015	0.359455	-0.0197	0.030866	-0.32625
MLLT3	-0.029031627	0.050971656	0.32506	0.104187	-0.07305	-0.25093
MLLT4	-0.01713393	-0.044012943	-0.05934	0.067338	-0.08511	-0.04396
MLLT4-AS	-0.0272592	-0.019341485	0.116166	0.027121	-0.05262	-0.04521
MLLT6	0.026379211	0.138091823	0.280338	-0.00371	-0.02364	-0.37812
MLN	-0.034548014	-0.09386963	-0.15574	-0.00844	0.019315	0.038582
MLNR	-0.047481828	0.006417627	-0.01115	-0.02518	0.081126	0.171194
MLPH	-0.023763612	-0.033754397	0.029293	-0.0188	-0.04517	-0.08938
MLST8	0.012251262	-0.036330925	0.006806	0.060936	-0.16354	0.248679
MLX	0.041973427	-0.01212545	-0.2299	0.024653	-0.05646	0.356408
MLXIP	-2.01E-06	-0.089736682	0.169876	-0.06983	0.250201	0.243238
MLXIPL	-0.023127591	-0.048453975	0.00738	-0.01304	-0.0458	0.011612
MLYCD	-0.004775373	0.080757657	-0.12636	-0.08202	0.19671	0.186266
MMAA	-0.024034764	0.063949925	0.081022	-0.01048	0.041439	-0.03214
MMAB	0.028073576	0.09080009	0.313436	4.75E-05	-0.15844	-0.24593
MMACHC	-0.020791172	0.030852436	0.122137	0.121303	-0.35049	0.009206
MMADHC	0.126313181	0.142914853	-0.00628	0.171645	-0.40218	-0.44946
MMD	0.015334755	0.036917582	0.096882	-0.09103	0.21673	-0.18196
MMD2	-0.045619271	-0.042329816	0.003621	0.011511	-0.05522	-0.10689
MME	-0.001396735	-0.044328648	-0.19402	-0.00724	0.013552	-0.16445
MMEL1	-0.006173569	-0.052688121	0.002213	0.004817	0.008803	0.427433
MMGT1	0.066351684	0.036315733	-0.01091	0.024876	-0.05552	0.222227
MMP1	-0.010169522	-0.000497679	-0.00024	-0.03154	0.117633	-0.01122
MMP10	-0.019528825	-0.029700293	-0.03478	-0.01321	0.008321	-0.03225
MMP11	0.013489506	-0.078694817	0.009229	0.025464	-0.0399	-0.19448
MMP12	-0.011593477	-0.018440847	0.017411	0.118156	-0.24506	-0.01115
MMP13	-0.037809263	-0.073887858	-0.06426	-0.01146	0.04865	-0.04355
MMP14	-0.025459305	-0.089388306	-0.03005	-0.0153	-0.04048	0.012679
MMP15	-0.037591193	-0.091121618	-0.07548	0.004242	-0.00983	-0.129
MMP16	-0.12184116	-0.105341876	-0.05163	-0.06911	-0.00235	-0.03489
MMP17	-0.026368135	-0.093421922	-0.10812	0.007854	-0.03708	0.222692
MMP19	-0.016020116	-0.040763637	-0.00674	-0.01077	-0.02188	0.074241
MMP2	0.00438175	-0.172545225	0.066271	0.038896	-0.0173	-0.06007
MMP20	-0.05123828	-0.035150316	-0.06533	-0.0006	-0.00384	-0.0363

MMP21	-0.053659278	-0.094786523	-0.05679	-0.00386	-0.00135	-0.1171
MMP24	-0.045350348	-0.055308599	-0.00796	-0.03547	0.07028	-0.07843
MMP24-AS1	0.023265724	-0.039358452	0.002076	-0.02152	-0.0441	0.130165
MMP25	0.004507389	-0.059395184	-0.16405	0.014378	-0.07335	-0.08499
MMP26	-0.024867815	-0.045881588	0.057471	0.048094	-0.14	-0.10105
MMP27	-0.036305008	-0.053943548	0.037461	0.001236	0.015722	-0.04359
MMP28	-0.065992524	-0.122277673	0.482426	-0.0672	0.12262	-0.09278
MMP3	-0.068160836	-0.028125304	0.067203	-0.01204	0.011026	0.053328
MMP7	-0.016776335	-0.037529725	0.003458	-0.00696	-0.01453	0.008477
MMP8	-0.011579134	-0.058299857	0.485669	-0.02166	0.088221	-0.12806
MMP9	0.010102297	-0.054800952	-0.05381	0.018964	-0.056	-0.03913
MMRN1	-0.01988618	0.019199569	-0.05604	-0.20133	0.574027	-0.09285
MMRN2	-0.000463552	0.019438841	-0.10438	0.017695	0.064022	0.045269
MMS19	0.043021029	-0.008374352	0.226629	0.010167	-0.03345	-0.10218
MN1	-0.016825254	0.014297455	-0.07185	-0.02632	0.043201	0.006653
MNAT1	0.003541588	0.073357302	0.082184	0.129856	-0.38909	-0.28317
MND1	-0.017655127	0.00430449	0.01818	0.186197	-0.33954	-0.04431
MNDA	0.010403361	-0.157356109	-0.35034	-0.1102	0.30198	0.402466
MNS1	-0.047265077	0.018699711	-0.02204	-0.02675	0.079491	-0.02919
MNT	0.026106652	-0.08812888	-0.11475	-0.03008	0.066935	0.154877
MNX1	-0.02530737	-0.065911584	0.032786	0.008803	-0.00691	0.037659
MNX1-AS1	-0.047392709	-0.060561526	-0.10093	0.015831	-0.00974	-0.00826
MOAP1	0.052134982	0.144115279	0.315655	0.009226	-0.02525	-0.26749
MOB1A	0.071732945	0.182208699	-0.31154	0.016585	-0.05297	0.124279
MOB1B	0.022724893	0.016636003	-0.18201	-0.04288	0.132104	0.315022
MOB2	0.027572292	0.065149872	0.010611	-0.06016	0.12101	-0.00231
MOB3A	0.029117378	0.060032714	-0.18102	-0.01578	0.030571	0.115732
MOB3B	-0.021071843	-0.09152106	-0.15502	-0.02913	0.057609	0.05787
MOB3C	0.004127197	0.00449118	-0.06408	-0.07493	0.127166	0.03399
MOB4	0.061176513	0.065978686	0.019	0.011022	-0.03016	-0.25916
MOBP	-0.08295173	-0.081024802	0.061602	-0.01206	0.03784	0.023589
MOCOS	-0.033699277	-0.043136066	-0.03083	0.041977	-0.10251	0.004435
MOCS1	-0.020306732	-0.048521739	0.019131	0.085073	-0.02054	-0.00545
MOCS2	0.014909098	0.071310516	0.170728	0.043748	-0.0858	-0.06409
MOCS3	-0.019899503	0.001932748	0.04274	0.017351	-0.06047	0.04841
MOG	-0.051290798	-0.003950143	0.059648	0.082299	0.003193	-0.22581
MOGAT1	-0.027887418	-0.091210081	-0.00911	0.009907	-0.03951	0.034696
MOGAT2	-0.044367978	-0.007745928	-0.01606	-0.00284	-0.00774	-0.11857
MOGAT3	-0.046535345	-0.048251577	-0.03693	0.017154	-0.02991	0.021397
MOGS	0.025891115	0.076556033	-0.03945	0.01867	-0.06847	0.11364
MOK	-0.019251068	0.045744066	-0.03347	0.060877	-0.1495	0.01287
MON1A	0.002438535	0.059784666	-0.0041	0.06755	-0.20749	0.05709
MON1B	0.074573103	0.127715066	-0.10129	-0.00303	-0.02983	-0.02427
MON2	-0.0195078	0.050586109	0.058648	-0.00177	0.056416	-0.05661
MOP-1	-0.03912293	-0.082586903	-0.04575	-0.02026	0.080855	0.062607
MORC1	-0.042510821	-0.078654614	0.013716	-0.03046	0.049224	-0.02438
MORC2	0.005989327	-0.024830112	0.298631	0.024301	0.007533	-0.20078
MORC2-AS1	0.012225591	-0.079675199	0.102669	-0.09644	0.178239	-0.1162
MORC3	0.050016858	0.103924635	-0.25249	-0.04429	0.11353	-0.10846
MORC4	-0.001603663	-0.008195616	0.147203	-0.042	0.127231	-0.44951
MORF4L1	0.100091517	0.170060935	0.036955	0.097246	-0.19719	-0.13728

MORF4L2	0.021748975	-0.038605816	0.033731	0.006828	-0.03227	-0.08844
MORF4L2-/	-0.029096149	-0.100976773	0.112184	-0.02414	0.082223	-0.17612
MORN1	-0.045699152	-0.045539791	0.010063	0.012077	-0.09693	-0.11717
MORN2	-0.00274554	-0.051093046	-0.12202	0.031527	-0.05524	0.657388
MORN3	-0.006956398	-0.015277167	0.07639	-0.06638	0.138527	-0.17476
MORN4	-0.030584819	-0.077934875	0.064556	0.042442	-0.19669	-0.08969
MORN5	-0.031836177	-0.031798818	-0.02768	0.027891	-0.05971	-0.02211
MOS	-0.035543837	-0.09372845	-0.0258	0.015322	-0.01907	0.171885
MOSPD1	0.019505505	0.040750633	0.056441	0.075597	-0.20451	0.064317
MOSPD2	0.018319362	-0.001489068	-0.3232	-0.01748	0.055383	0.335482
MOSPD3	0.020283921	-0.038689449	0.098825	0.01852	-0.12134	0.107587
MOV10	0.008413331	0.06805357	0.222401	0.031551	-0.07585	0.005626
MOV10L1	-0.050089317	-0.102821072	0.014245	0.0003	-0.01836	0.021057
MOXD1	-0.004537064	-0.071675403	-0.05441	0.007925	0.043616	-0.25417
MPC1	0.027005695	0.017106458	-0.01144	0.001323	-0.00013	0.24461
MPC2	0.037376581	0.050355453	0.012006	0.043445	-0.08264	-0.05632
MPDU1	0.003759761	-0.023229724	-0.10913	0.048875	-0.14512	0.394553
MPDZ	-0.026617927	-0.051608865	-0.02968	-0.0105	-0.00053	-0.07159
MPEG1	0.01073296	-0.17217865	-0.48177	-0.11006	0.316124	0.375788
MPG	0.031377444	0.001022883	-0.07045	0.031062	-0.11263	0.11586
MPHOSPH1(	0.033158776	0.072070034	0.174424	0.011178	-0.01896	-0.00364
MPHOSPH6	0.015193966	0.002365402	0.082146	0.063266	-0.16053	0.182345
MPHOSPH8	0.031549239	0.061119543	0.182779	-0.02682	0.064465	-0.31294
MPHOSPH9	-0.016435695	0.112536335	0.291052	0.01515	-0.0783	-0.13326
MPI	-0.011582946	-0.038711623	0.319142	0.013039	-0.06076	-0.14814
MPL	-0.092349666	-0.031031458	-0.07768	-0.02555	0.192359	0.019204
MPLKIP	0.042332325	0.004443077	-0.0193	0.033634	-0.1315	0.169353
MPND	0.015578183	0.077324677	-0.09739	0.005539	-0.00799	0.251608
MPO	-0.010396786	-0.114500962	-0.07736	-0.0367	0.114195	0.217517
MPP1	0.02424976	-0.100702134	-0.30106	-0.04968	0.132733	0.320727
MPP2	-0.032952264	-0.050995448	0.086129	0.060397	-0.19386	-0.07536
MPP3	-0.02891192	-0.0755458	-0.04222	-0.0013	-0.03354	-0.09902
MPP4	-0.071364477	-0.1166827	-0.03125	-0.02016	0.062385	-0.06118
MPP5	-0.001019865	-0.017269182	0.132006	0.027386	-0.03417	-0.06401
MPP6	-0.015021035	-0.046707658	0.06829	0.066189	-0.20085	-0.15287
MPP7	0.058407987	0.006863537	0.141801	-0.13762	0.287647	0.334294
MPPE1	0.029640804	-0.015111984	-0.12032	-0.02531	0.065216	-0.09082
MPPED1	-0.038946781	-0.104104837	0.013835	0.027206	-0.1182	-0.01541
MPPED2	-0.03785008	0.014308706	0.09038	-0.04649	0.143348	-0.17779
MPRIP	-0.046646485	-0.090717629	0.195835	-0.04182	-0.10841	-0.26099
MPST	0.016145217	0.057128912	-0.1294	0.042505	-0.14964	0.188859
MPV17	0.024411216	0.025004332	0.058786	0.018477	-0.05019	0.28362
MPV17L	0.013430174	-0.013960127	0.007732	0.008791	-0.04887	-0.14047
MPV17L2	0.007181985	-0.039344321	-0.08372	0.102269	-0.2965	0.309326
MPZ	-0.018481534	-0.071841888	-0.01445	0.065343	-0.22672	0.010147
MPZL1	0.003736513	-0.062365574	-0.22768	0.010528	-0.02091	-0.00292
MPZL2	-0.005462465	-0.099755374	-0.19854	-0.11405	0.182876	0.364192
MPZL3	-0.011223107	-0.046165082	0.182141	-0.00691	0.073289	-0.2317
MR1	0.045003545	0.091034535	-0.16115	0.024359	-0.1163	0.300673
MRAP	-0.022203517	-0.053879106	-0.03954	0.021908	-0.00634	0.036241
MRAP2	-0.01915042	-0.028856638	-0.02034	0.075153	-0.18847	-0.08152

MRAS	-0.015235778	-0.11676916	-0.20374	-0.02399	0.045819	0.528256
MRC1	0.002426997	-0.084796912	-0.14647	-0.05499	0.149974	0.061067
MRC2	0.006912367	-0.102795022	0.0731	-0.03937	-0.00862	-0.20155
MRE11A	-0.045488334	0.057614307	0.018093	-0.03585	0.016006	0.006134
MREG	-0.000884403	0.002320721	0.063013	0.029592	-0.13319	-0.17474
MRFAP1	0.158403756	0.229723444	-0.16064	0.099359	-0.24829	0.076095
MRFAP1L1	0.046423565	0.219447369	0.29842	0.021007	-0.05458	-0.42671
MRGBP	0.01379758	0.106098565	-0.00483	0.109686	-0.28792	0.136311
MRGPRF	-0.031461296	-0.051042889	0.062103	-0.00165	0.036224	0.024655
MRGPRG-AS	-0.026782693	-0.043352839	0.014487	0.000162	-0.09906	-0.0738
MRGPRX1	-0.049528997	-0.046442827	-0.10035	0.015961	-0.02011	0.133321
MRGPRX2	-0.03281144	-0.043861155	-0.01568	0.042403	-0.12332	-0.01054
MRGPRX3	-0.051277852	-0.074268798	-0.05793	0.00791	-0.00514	-0.01691
MRGPRX4	-0.04328747	-0.044648704	0.037815	0.047307	-0.12233	-0.01265
MRI1	0.032572672	-0.045668506	0.264841	-0.03113	0.044177	-0.17321
MRM1	-0.015597226	-0.048328632	0.036798	0.025904	-0.14801	-0.08572
MRO	0.006804259	-0.038222331	0.001049	0.095997	-0.07092	0.039781
MROH1	-0.017512055	-0.028910148	-0.11888	-0.00585	0.011701	0.418337
MROH2A	-0.054027857	-0.047195304	-0.05016	-0.00625	0.024594	0.043222
MROH2B	0.034629912	-0.053271794	-0.05856	0.009986	-0.05741	0.088746
MROH6	0.01312243	-0.134707365	-0.00338	-0.01409	-0.04605	0.006927
MROH7	-0.038248122	-0.06617336	-0.05994	-0.01084	0.057837	-0.08278
MROH8	-0.035847341	-0.058952477	-0.05474	0.004382	-0.0002	-0.05909
MROH9	-0.029183838	-0.062756903	-0.02495	-0.00359	0.028785	0.004733
MRPL1	0.027661388	0.089894246	0.25991	0.034731	-0.08816	-0.07057
MRPL10	0.041245149	0.198471786	0.264941	0.031919	-0.08665	0.015828
MRPL11	0.033337649	0.005262891	0.156347	0.120556	-0.33196	0.17459
MRPL12	0.007523881	0.001493654	0.040799	0.092542	-0.28811	0.169039
MRPL13	0.027144765	0.071656067	0.041026	0.087592	-0.24248	0.247386
MRPL14	0.041747657	0.06511917	0.097097	0.068901	-0.20161	0.209809
MRPL15	0.026036825	0.03795512	-0.00709	0.058776	-0.14048	0.290173
MRPL16	0.040219471	0.063815199	0.112105	0.011442	-0.03119	0.195555
MRPL17	0.028613482	-0.00550546	0.05214	0.0974	-0.28856	0.035205
MRPL18	0.059195876	0.0621308	0.099229	0.066252	-0.18666	-0.0248
MRPL19	-0.007341914	0.02160534	0.078497	0.055224	-0.11323	0.013157
MRPL2	0.009872808	-0.053260848	0.141909	0.09164	-0.24703	0.190938
MRPL20	-0.075508725	-0.067668842	-0.08707	-0.06113	0.060615	-0.05384
MRPL21	0.030327194	-0.011232608	0.107355	0.0745	-0.21183	0.217427
MRPL22	0.031309903	0.067416899	0.057805	0.098374	-0.28282	0.087569
MRPL23	0.012109571	-0.071668543	-0.02415	0.079839	-0.21649	0.325348
MRPL24	0.038086456	0.0282077	0.229403	0.096026	-0.27445	-0.0292
MRPL27	0.03266071	0.085210407	-0.04892	0.080017	-0.22328	0.266315
MRPL28	0.046884891	-0.004940427	-0.10044	0.097922	-0.31252	0.153693
MRPL3	0.040793257	0.02605168	0.168575	0.063885	-0.18674	0.102774
MRPL30	0.06977757	0.078532021	0.043744	0.046531	-0.06092	-0.04895
MRPL32	0.039963164	0.041008079	0.188828	0.03908	-0.11008	-0.05261
MRPL33	0.074375641	0.049012604	0.05701	0.067323	-0.18511	0.287732
MRPL34	0.025725688	0.02835394	0.181247	0.043243	-0.10514	0.167509
MRPL35	0.020474972	0.080121043	-0.06675	0.077986	-0.21527	0.058613
MRPL36	0.034895097	0.064686226	0.138928	0.069678	-0.20459	0.027669
MRPL37	0.025912979	0.00254693	0.086632	0.089647	-0.24481	0.197703

MRPL38	0.006267214	-0.014890769	0.174922	0.048719	-0.12691	0.026226
MRPL39	0.013078912	0.048266299	0.155411	0.063346	-0.18584	-0.14842
MRPL4	0.038884039	-0.037724073	0.012444	0.058022	-0.17029	0.014516
MRPL40	0.031374516	0.014585164	0.092933	0.049973	-0.12094	0.154155
MRPL41	0.043778273	-0.052445186	-0.06978	0.027299	-0.02642	0.001743
MRPL42	0.042491214	0.096303224	-0.05389	0.087835	-0.17897	0.037914
MRPL43	-0.004098551	0.018711651	0.121266	0.015528	-0.04442	0.220481
MRPL44	0.013056936	0.018458484	-0.13957	0.053126	-0.17213	0.191521
MRPL45	0.030702957	-0.035450585	0.092994	0.031092	-0.07478	0.106646
MRPL45P2	-0.061368041	-0.053966126	0.004563	-0.02811	0.051507	0.027319
MRPL46	0.025045509	0.048417558	0.213567	0.058529	-0.17712	-0.02944
MRPL47	0.035979152	0.087756121	0.091714	0.118143	-0.33627	0.08462
MRPL48	0.026692042	0.033695977	0.175086	0.044442	-0.11841	0.059544
MRPL49	0.077090987	0.080965205	0.220258	0.075633	-0.20064	-0.47056
MRPL50	-0.004235038	0.012881542	0.146748	0.041572	-0.12597	0.064664
MRPL51	0.063235994	0.099555714	0.115264	0.052899	-0.1638	0.0077
MRPL52	0.027522875	0.004134205	0.011373	0.090183	-0.24081	0.234357
MRPL54	0.040676676	0.036451539	0.127862	0.083071	-0.24882	0.236349
MRPL55	0.012378646	-0.07280097	0.099236	0.064051	-0.18488	0.091113
MRPL57	0.060658995	0.073919585	0.140647	0.050178	-0.0634	-0.02622
MRPL9	0.033387134	0.073855343	0.244098	0.064667	-0.17574	-0.095
MRPS10	0.023256431	0.100860068	0.157974	0.049009	-0.19153	-0.04148
MRPS11	0.021782778	0.12386116	0.075988	0.079108	-0.20948	0.145809
MRPS12	-0.0094752	0.033877891	0.03024	0.06056	-0.18761	0.001566
MRPS14	0.029563439	0.102849079	0.129291	0.029772	-0.06358	0.03567
MRPS15	0.023000902	-0.030613313	0.084127	0.078	-0.22735	0.234803
MRPS16	0.028798709	0.002649878	0.068139	0.073863	-0.2215	0.18566
MRPS17	0.017166651	0.072116867	0.089351	0.074731	-0.20492	0.121517
MRPS18A	0.033478493	0.03194082	-0.00885	0.124303	-0.38511	0.12988
MRPS18B	0.035811914	0.058763934	0.206715	0.046082	-0.1294	-0.00012
MRPS18C	-0.074820222	0.005213262	-0.04242	-0.06566	0.048211	0.015736
MRPS2	0.010060192	0.028270209	0.133039	0.069427	-0.17219	0.152042
MRPS21	0.041192379	-0.011749629	0.223981	0.051931	-0.14123	0.064841
MRPS22	0.057568121	0.054573518	0.014995	0.065922	-0.20411	0.131135
MRPS23	0.018530473	0.05231399	0.064981	0.061571	-0.15492	0.140463
MRPS25	0.00428289	-0.060620802	0.153223	0.016006	-0.02365	0.078598
MRPS26	0.018518375	0.018731595	0.163957	0.080861	-0.22101	0.005829
MRPS27	0.016336125	-0.020277358	0.194015	0.047915	-0.13473	-0.01541
MRPS28	0.025186714	0.045278793	0.091374	0.065535	-0.17508	0.230319
MRPS30	0.012237392	0.083634835	0.178092	0.02256	-0.03001	-0.15536
MRPS31	0.016546407	0.122965784	0.254668	0.013633	-0.05095	-0.08042
MRPS33	0.030039245	-0.007903999	0.26274	0.060229	-0.15787	0.021042
MRPS34	0.035931923	-0.014660528	0.058585	0.057745	-0.1912	0.126384
MRPS35	0.031698993	0.019678441	0.079979	0.03211	-0.06611	0.179498
MRPS36	0.055150144	0.05916005	0.004307	0.1008	-0.29025	0.224415
MRPS5	-0.004518358	-0.012802485	0.165482	0.064773	-0.16416	0.235118
MRPS6	0.039265596	0.165970674	0.161052	0.068542	-0.18261	-0.07413
MRPS7	0.030082867	0.023801536	0.109058	0.038041	-0.10509	0.208352
MRPS9	0.032978633	0.098697919	0.150673	0.046691	-0.11834	-0.04458
MRRF	0.025222253	-0.064764922	-0.01274	0.064557	-0.18043	0.141904
MRS2	0.018774443	0.061315445	-0.02098	-0.05251	0.118962	0.116936

MRTO4	0.007607479	-0.012187077	0.088104	0.080107	-0.2366	0.026804
MRVI1	-0.030153636	-0.06488649	-0.11694	-0.13855	0.342339	0.243198
MRVI1-AS:	-0.019640644	-0.094232779	-0.0899	-0.01894	-0.03511	-0.12697
MS4A1	0.00061452	-0.094258514	0.021707	-0.09981	0.254465	-0.02257
MS4A12	-0.049319618	0.037642939	-0.09747	0.031304	-0.07554	0.07081
MS4A14	0.002819931	-0.104300637	-0.28158	-0.08433	0.25462	0.725007
MS4A15	-0.048400961	-0.034149623	0.069657	-0.00867	0.051267	-0.03024
MS4A2	-0.017528495	-0.067199335	-0.01043	-0.02148	0.075456	-0.14054
MS4A3	-0.000302154	-0.121513992	0.218238	-0.06677	0.226679	-0.18204
MS4A4A	-0.003588497	-0.084004804	-0.30475	-0.01262	0.055274	0.468326
MS4A5	-0.0307862	-0.05991319	-0.01174	0.038781	-0.10495	0.048861
MS4A6A	0.009255024	-0.120587216	-0.33385	-0.09567	0.259419	0.516234
MS4A6E	-0.02007109	-0.007126157	-0.09909	0.085682	-0.18656	0.118175
MS4A7	0.009243214	-0.088991431	-0.35491	-0.12273	0.248165	0.616631
MS4A8	-0.018363059	-0.018027999	-0.03633	0.089859	-0.25542	-0.0399
MSANTD1	-0.034836832	-0.018513326	-0.10682	-0.01772	0.077421	0.092386
MSANTD2	0.02051622	0.094796983	0.165824	0.004525	0.024332	-0.03704
MSANTD3	0.010188276	0.052249772	0.150788	-0.02528	0.050678	-0.22141
MSANTD4	-0.018895146	0.04392872	-0.00048	0.031382	-0.08785	0.091549
MSC	-0.012223871	-0.036168591	0.077713	0.093807	-0.26996	-0.04839
MSH2	0.007852434	0.09610055	0.237436	0.042678	-0.11592	-0.34571
MSH3	0.026363302	0.009001614	0.032872	0.021402	-0.04151	-0.00886
MSH4	-0.041608682	-0.040545364	0.000517	0.012902	-0.03919	-0.13471
MSH6	0.005342816	0.08523279	0.084736	0.077491	-0.19447	-0.12944
MSI1	-0.030402858	0.004094713	-0.0352	0.010784	-0.00676	-0.07211
MSI2	-0.065790859	-0.080456581	0.232769	0.04448	-0.14384	-0.27559
MSL1	0.070869871	0.021434237	0.038054	-0.0057	0.081819	-0.31301
MSL2	0.04523598	0.135796192	0.13762	0.000836	-0.00725	-0.40452
MSL3	-0.048064119	-0.165662972	0.033013	-0.03821	0.127562	-0.09818
MSLN	-0.015551459	-0.031270993	-0.05769	-0.0027	0.026086	0.152028
MSMB	-0.047459013	0.001853254	-0.04985	-0.01844	0.035247	-0.0513
MSMO1	0.014942796	0.133347701	0.121464	0.050215	-0.1435	-0.18296
MSN	0.036071028	0.176194858	-0.06708	-0.15021	0.429355	0.076078
MSR1	0.010435785	-0.147982927	-0.14555	-0.0554	0.212275	0.21226
MSRA	0.014819152	-0.116800496	-0.19396	-0.04838	0.122917	0.124396
MSRB1	0.02219303	-0.064195126	-0.35867	-0.01334	0.049481	0.325987
MSRB2	-0.00652669	-0.083285042	-0.066	-0.09349	0.250534	0.378313
MSRB3	-0.026927987	-0.10627815	-0.08821	-0.11997	0.305245	-0.19797
MSS51	-0.00893013	0.018945232	-0.05311	-0.00823	0.04926	0.04845
MST1	0.000697698	-0.047013689	0.160438	-0.03302	-0.01084	-0.26234
MST1L	-0.030638141	-0.006347488	0.103889	-0.0042	0.031073	-0.17534
MST1R	-0.040338034	-0.055433609	-0.00726	0.017386	-0.04081	-0.07438
MST4	0.032894597	0.248824622	0.12716	0.004927	-0.01077	-0.37102
MSTN	-0.178442358	-0.1259354	0.045895	0.020449	0.096266	-0.0861
MSTO1	0.006865347	-0.014958862	0.231602	0.055799	-0.18609	-0.2799
MSX1	-0.042150838	0.016006019	0.053349	0.010767	-0.0273	0.022854
MSX2	-0.101982839	0.018283416	0.177406	-0.10655	0.106568	-0.11801
MT1E	0.02012985	-0.040423299	0.269589	0.019391	-0.1049	0.12204
MT1F	0.023072226	-0.005504836	0.473024	-0.0106	-0.02155	0.061791
MT1G	0.018284038	0.005971034	0.284151	0.024745	-0.10567	0.057081
MT1H	0.02948293	0.021550396	0.321387	0.022999	-0.09591	0.115644

MT1HL1	0.034686854	0.015038857	0.320198	0.027447	-0.09215	0.13176
MT1M	-0.048005457	0.011616972	-0.03084	0.009772	-0.01766	0.028736
MT1X	0.023290144	0.013205297	0.383372	0.030812	-0.10359	0.230751
MT2A	0.037415763	0.073270438	0.268979	0.027921	-0.08568	0.180896
MT3	-0.028680011	-0.099682733	-0.00478	0.038567	-0.15181	-0.00215
MT4	-0.026984357	-0.015734945	0.04391	0.013607	-0.12479	-0.07516
MTA1	0.004196884	-0.031423277	0.108365	0.056116	-0.12769	0.083464
MTA2	-0.011140247	0.079273729	0.031014	0.059694	-0.13241	0.062167
MTA3	-0.01779838	-0.073694988	0.307527	-0.02981	0.067612	-0.24119
MTAP	0.009519499	-0.064569647	0.053363	-0.00301	0.075221	0.117997
MTBP	-0.04372677	0.047514272	0.052411	0.071373	-0.2927	-0.12993
MTCH1	0.114853892	0.008235092	-0.07341	0.075587	-0.14696	-0.06045
MTCH2	0.034448066	0.074356213	-0.04292	0.06356	-0.16667	0.302534
MTCL1	-0.010716039	-0.070861185	-0.12334	0.003584	0.008283	-0.19765
MTCP1	-0.016496622	0.044992602	0.14906	0.05442	-0.13053	-0.06098
MTDH	0.041408015	0.077431082	0.082044	0.013079	-0.05091	0.161674
MTERF1	0.016655514	0.063522907	0.028794	0.033359	-0.06699	-0.2069
MTERF2	0.003309665	0.128271946	0.117161	0.012426	0.004857	-0.42056
MTERF3	0.02521224	0.075704401	0.137302	0.057693	-0.14156	0.023792
MTERF4	0.001306554	-0.057530402	0.374415	0.005243	0.052115	-0.18358
MTF1	0.025334713	-0.05230856	-0.21763	-0.03639	0.114755	0.262254
MTF2	0.037497537	0.033684693	0.037276	0.027381	-0.14193	-0.18537
MTFMT	0.003013328	-0.022961892	-0.00752	0.081875	-0.19881	-0.11608
MTFP1	0.019905712	0.018410904	0.334176	0.121929	-0.37005	-0.17511
MTFR1	-0.004673337	0.041367416	-0.12014	0.088588	-0.21954	0.145583
MTFR1L	0.013806824	-0.073319756	-0.08977	-0.04016	0.088066	0.093717
MTFR2	-0.010359614	0.007197052	-0.02791	0.149744	-0.4078	-0.01463
MTG1	0.012176978	-0.007143895	-0.00796	0.022368	-0.13575	-0.11792
MTG2	0.032302942	-0.049031486	-0.00172	0.028552	-0.16212	0.040887
MTHFD1	0.026118822	0.009451496	0.083789	0.082638	-0.22978	0.01566
MTHFD1L	-0.030773591	-0.027691456	-0.0241	0.015255	-0.02431	-0.02984
MTHFD2	0.017230975	0.057206479	-0.18545	0.0853	-0.26689	0.205736
MTHFD2L	0.000522006	0.005168191	-0.05013	0.03917	0.008665	-0.024
MTHFR	-0.131269214	-0.058787474	-0.00337	-0.11695	0.1538	0.15625
MTHFSD	-0.001586016	-0.065853904	0.079737	0.037371	-0.11689	0.001604
MTIF2	0.03405204	0.027065504	0.090961	0.04008	-0.09802	0.205529
MTIF3	0.017964034	0.01342592	-0.02953	-0.01778	0.030094	0.167746
MTL5	-0.049678741	-0.104894058	-0.07884	-0.02697	0.083875	0.044372
MTM1	0.004528016	-0.15877223	-0.20824	0.018539	-0.06473	0.13661
MTMR1	0.005392642	-0.041661519	0.08302	-0.0264	-0.0167	-0.51545
MTMR10	0.02294175	-0.083648901	-0.37286	-0.04047	0.128024	0.047202
MTMR11	-0.001532032	-0.08665409	-0.30934	-0.0641	0.185957	0.704715
MTMR12	0.027559858	0.189608087	-0.12275	-0.01425	0.071703	-0.12619
MTMR14	0.033843462	-0.126316809	-0.23541	-0.0231	0.060411	0.187139
MTMR2	-0.0060805	0.08210866	0.079583	0.060862	-0.19701	-0.1369
MTMR3	-0.002926624	-0.033572856	-0.17766	-0.04901	0.103576	0.200962
MTMR4	0.023022254	-0.003787966	-0.03988	0.049993	-0.15799	-0.15087
MTMR6	0.030898761	0.066651095	-0.11915	0.034493	-0.0474	0.222117
MTMR7	-0.067643656	-0.084097822	0.112625	-0.00399	-0.00332	0.062634
MTMR9	0.023706369	0.071261347	0.016371	-0.0163	0.046483	-0.11084
MTMR9LP	-0.036563213	0.025076553	0.042713	0.001792	-0.02508	-0.0883

MTNR1A	-0.046528688	-0.052965594	-0.05505	0.021241	-0.02328	-0.00351
MTNR1B	-0.037535712	-0.095045065	0.126906	0.022765	-0.06247	-0.03184
MTO1	0.024031178	0.058631821	0.189172	0.022781	-0.00486	0.065284
MTOR	-0.011156905	-0.035423495	-0.05699	0.079956	-0.18708	0.117073
MTPAP	0.021262949	0.008718815	-0.03643	0.028151	-0.08661	0.142945
MTPN	-0.005720093	0.017121176	-0.14116	-0.09216	0.251345	0.110672
MTR	0.019163197	0.041216754	0.256862	0.025439	-0.04951	-0.01764
MTRF1	-0.058127236	0.010824279	0.182264	0.043028	-0.01802	-0.11214
MTRF1L	0.023836751	0.100330508	-0.15718	0.023144	-0.07323	0.12454
MTRR	0.0266805	0.019256872	0.162419	0.060654	-0.15587	-0.04828
MTSS1	-0.014055684	0.015269555	0.01787	-0.08929	0.288109	0.090354
MTSS1L	0.01853084	-0.07692109	0.084994	-0.04006	0.008025	-0.02472
MTTP	-0.047078597	-0.049419703	-0.02037	0.018835	-0.07649	0.064649
MTURN	-0.003091975	-0.048322419	-0.1804	-0.17634	0.495363	-0.09338
MTUS1	-0.016887619	-0.073988662	0.387639	0.040062	-0.07071	-0.20922
MTUS2	-0.041674543	-0.028792936	0.142449	0.018698	-0.05119	0.063129
MTUS2-AS1	-0.033390491	-0.050920657	0.12954	0.019262	-0.02618	-0.19365
MTX1	0.041931418	-0.024970208	-0.22095	0.067119	-0.15092	0.30912
MTX2	0.020329404	-0.005187136	0.127867	0.087691	-0.23623	-0.03613
MTX3	0.005346958	-0.024606952	0.376913	0.062939	-0.17723	-0.35878
MUC1	-0.027526641	-0.011374046	-0.00274	0.14929	-0.32629	-0.02454
MUC12	-0.039392496	-0.07311742	-0.01193	0.01332	-0.03354	-0.05582
MUC13	-0.036918018	-0.050452732	0.022642	0.012139	-0.03578	0.043728
MUC15	-0.068745973	-0.10371843	0.007201	0.022086	-0.06347	0.050214
MUC16	-0.034841892	-0.0396958	0.012078	0.050442	-0.12734	-0.07356
MUC17	-0.046714806	0.005335952	-0.11444	0.024951	-0.12569	-0.05055
MUC19	-0.042236409	-0.033950014	0.172615	0.030371	-0.05632	-0.11602
MUC2	-0.033735468	-0.070476288	-0.09023	0.017753	-0.06965	0.031223
MUC20	-0.016012687	-0.032268445	0.110426	0.025487	-0.02862	-0.06138
MUC3	-0.040188338	-0.062539342	-0.11987	0.017901	-0.06504	-0.07006
MUC3B	-0.032836953	-0.070993674	0.020456	-0.00235	-0.05949	-0.08484
MUC4	-0.092953204	0.001428083	-0.06571	0.096704	-0.09942	-0.00765
MUC5AC	-0.026697134	-0.077369314	-0.08462	0.023209	-0.02594	-0.06818
MUC5B	-0.042724462	-0.054087044	-0.04915	0.000429	0.031964	0.041577
MUC6	-0.040675182	-0.056251962	-0.05262	0.001025	0.005964	0.128854
MUC7	-0.066401508	-0.069096388	-0.09025	0.000414	0.025801	0.03896
MUC8	-0.001746395	-0.088868294	0.028667	-0.0128	-0.02917	-0.08652
MUCL1	-0.023266902	0.006046129	-0.0111	0.004983	-0.08049	-0.06933
MUL1	0.040889637	0.004843125	-0.02954	0.021728	-0.1286	-0.11203
MUM1	-0.00282822	0.05286975	0.090475	0.0384	-0.08035	-0.05489
MUM1L1	-0.070681794	-0.063765767	-0.00883	-0.00425	0.006063	-0.02298
MURC	-0.051523965	0.010217124	-0.04906	-0.02062	0.063228	-0.08073
MUS81	0.045946323	0.067520476	0.196944	0.005674	-0.0505	-0.17441
MUSK	-0.065612186	-0.035409774	-0.00675	0.00368	0.004437	0.133141
MUT	0.017524026	0.012323929	-0.01747	0.031793	-0.0748	0.002912
MUTYH	0.030110889	-0.024654794	0.291456	0.043317	-0.15887	-0.09016
MVB12A	0.008522497	0.00179484	-0.06294	0.0358	-0.07491	0.342976
MVB12B	-0.052386381	-0.148047854	0.171439	0.100969	-0.19542	0.116833
MVD	-0.015645349	0.100915986	0.008252	0.018776	-0.09761	-0.06664
MVK	-0.040053678	0.04869708	-0.09483	0.012099	-0.16486	-0.08839
MVP	0.045458958	-0.02486804	-0.10727	-0.01445	0.050679	0.182452

MX1	0.023610901	-0.071359685	-0.19079	-0.09141	0.248857	0.038689
MX2	0.028168025	-0.089809661	-0.16676	-0.08876	0.262718	0.126355
MXD1	0.019690772	0.016519344	-0.30415	-0.00909	0.033152	0.130431
MXD3	0.02154549	-0.080757243	-0.0042	0.030674	-0.18676	-0.0051
MXI1	0.042374165	-0.102366012	-0.05295	-0.08729	0.229198	0.055808
MXRA5	-0.026824374	-0.023696686	-0.08452	0.024948	-0.0663	0.10399
MXRA7	-0.00405384	0.16621753	0.10195	-0.02086	0.055578	-0.19879
MXRA8	-0.020831899	-0.135239394	0.345332	-0.03442	0.036184	-0.15266
MYADM	0.020347627	-0.090767579	-0.17609	-0.0655	0.177786	0.23383
MYADML2	-0.029376582	-0.114711184	-0.07059	0.013013	-0.02746	-0.04278
MYB	-0.000280008	-0.112441554	-0.01105	0.074929	-0.2224	-0.07898
MYBBP1A	-0.00035397	-0.04947528	0.069255	0.049241	-0.16704	-0.10526
MYBL1	-0.003100977	0.257283139	0.401584	-0.00485	0.01772	-0.24514
MYBL2	-0.007079921	-0.096892717	-0.02474	0.081846	-0.30122	-0.1798
MYBPC1	-0.034841063	0.017601604	0.040828	0.011794	-0.01772	-0.01683
MYBPC2	-0.025870536	-0.027619693	-0.03464	0.064351	-0.15388	-0.00087
MYBPC3	-0.005205099	-0.095539771	0.028832	-0.02923	-0.02742	-0.02794
MYBPH	-0.030363738	-0.036742371	-0.06273	0.002778	-0.0046	-0.02792
MYC	0.007464713	-0.080162715	0.341401	-0.0045	-0.00456	-0.13895
MYCBP2	0.063890176	0.0487713	0.147181	-0.00469	0.047333	-0.16192
MYCBPAP	-0.047776876	-0.056915698	-0.02503	0.026937	-0.03911	0.078241
MYCL	-0.000190842	-0.122912579	-0.26112	-0.06769	0.19966	0.343871
MYCN	-0.050343245	-0.04971933	-0.0488	0.079795	-0.10278	-0.00044
MYCNOS	-0.047089153	-0.024592701	0.093185	-0.00087	0.023652	-0.03337
MYCT1	-0.025137445	-0.071503296	-0.04375	-0.02778	0.078193	-0.13771
MYD88	0.054234761	-0.046587117	-0.33754	-0.02047	0.088685	0.233278
MYEF2	0.017517682	-0.069701209	0.144275	-0.04639	0.046089	-0.45442
MYEOV	-0.029012979	-0.089860035	-0.07915	-0.0412	0.047377	-0.08068
MYEOV2	0.049330456	0.079095208	0.119749	0.021387	-0.05507	0.231205
MYF5	-0.025883528	-0.044031994	0.001969	-0.00164	0.008892	-0.04489
MYF6	-0.032876949	0.006712628	-0.03939	0.010843	-0.0268	0.081454
MYH1	-0.06242973	-0.024399777	0.069462	-0.00389	-0.01755	-0.0136
MYH10	-0.016648996	-0.044565225	0.055482	0.07162	-0.19561	-0.05407
MYH11	-0.067128037	-0.120101997	-0.00502	-0.01665	0.004279	0.241519
MYH13	-0.030461466	-0.040055462	-0.1414	0.045563	-0.09845	0.01871
MYH14	-0.036681661	-0.072867497	-0.04455	-0.00269	-0.05401	-0.04785
MYH15	-0.044908665	-0.055164244	-0.03591	0.009537	-0.00141	-0.0711
MYH16	-0.047129982	0.006967835	-0.11064	-0.00132	0.032905	0.064402
MYH2	-0.040185177	-0.029732633	-0.09785	0.004462	0.012762	-0.10845
MYH3	-0.016100866	-0.024051764	0.067307	-0.05898	0.183255	-0.0886
MYH4	-0.055074997	-0.044234722	0.003484	0.040952	-0.10552	-0.11967
MYH6	-0.020589912	0.012540126	-0.05048	0.120945	-0.32957	0.022073
MYH7	-0.031686778	-0.040919407	0.121449	0.05327	-0.12638	-0.11111
MYH7B	-0.044505289	-0.070237079	-0.13277	0.014126	-0.00898	-0.08314
MYH8	-0.073446336	-0.021786036	0.040893	0.002272	0.026251	0.003
MYH9	0.053367644	0.054252674	0.044222	-0.00106	0.023884	-0.09905
MYL1	-0.035176137	-0.078876948	0.06942	0.017062	-0.02297	-0.0412
MYL10	-0.021819029	-0.114500547	-0.01124	-0.00911	-0.03978	0.022286
MYL12A	-0.002284963	0.078273133	0.222676	-0.02216	0.096404	-0.25015
MYL12B	0.095582928	0.121908096	-0.0698	0.071484	-0.14941	-0.20628
MYL2	-0.048151457	-0.055982591	0.033846	0.010671	0.008513	0.061843

MYL3	-0.027384279	-0.045349254	-0.15815	0.011167	-0.1012	-0.00581
MYL4	-0.024014994	-0.062649573	-0.07644	-0.00024	-0.09404	-0.03316
MYL5	0.00526139	-0.031432509	-0.03061	0.053505	-0.09556	0.140976
MYL6	0.049856709	0.000400627	-0.21029	0.031422	-0.06527	-0.01176
MYL6B	0.012632459	-0.042837266	-0.0494	0.105681	-0.27106	-0.03494
MYL7	-0.03078956	-0.043277147	0.032773	0.003011	0.005822	0.048401
MYL9	-0.022795813	-0.032262601	-0.04682	-0.23525	0.623544	-0.01738
MYLIP	0.02622466	0.10419327	0.106907	-0.07524	0.227514	-0.25676
MYLK	0.062768204	0.020339375	-0.13834	-0.34306	0.152827	0.089631
MYLK-AS1	-0.012137531	-0.038294083	0.037837	0.010199	-0.06176	-0.15541
MYLK2	-0.005868057	-0.035436161	-0.01964	-0.00762	-0.08744	-0.07478
MYLK3	-0.068064896	0.040157487	-0.0815	-0.03865	-0.01714	0.015269
MYLK4	0.027871454	-0.063670357	0.024117	0.090381	0.05353	-0.09426
MYLPF	-0.011743976	-0.037206066	-0.07534	0.020567	-0.07635	0.069996
MYNN	-0.039679559	-0.029743653	0.040275	-0.05856	0.009665	-0.06205
MYO10	-0.037727077	-0.066591583	-0.03961	0.017346	-0.14594	0.327534
MYO15A	-0.035377549	-0.050828167	-0.01623	-0.0138	0.039257	-0.06862
MYO15B	-0.022706201	-0.118622252	-0.04809	-0.11331	0.277421	0.093272
MYO16	-0.030145515	-0.037157547	0.044111	0.101071	-0.2634	-0.06617
MYO18B	-0.021616642	-0.032817108	0.076705	0.0357	-0.08298	-0.05792
MYO19	0.023575781	-0.022876821	0.097937	0.033378	-0.10242	-0.13352
MYO1A	-0.027536729	-0.048947206	-0.07241	-0.03277	0.082632	0.140537
MYO1B	-0.011931895	-0.053090638	-0.02869	0.036819	-0.09051	-0.07969
MYO1C	0.013860437	-0.107822129	0.040073	-0.02168	-0.02396	-0.16819
MYO1D	-0.036107079	-0.019474956	0.042697	0.068825	-0.15954	-0.10923
MYO1E	-0.002060626	-0.025901215	-0.19655	-0.01284	0.027722	-0.00133
MYO1F	0.02262662	0.083027416	-0.15173	-0.01532	0.045951	0.211484
MYO1G	0.025352043	0.102855487	0.092438	0.007947	-0.03006	0.157155
MYO1H	-0.047372797	-0.015350887	-0.0333	0.020159	-0.02864	-0.077
MYO3A	-0.016951843	-0.044503137	0.050406	-0.0093	0.074597	0.037998
MYO3B	-0.039195911	-0.013688946	0.035783	0.239012	-0.21639	-0.06258
MYO5A	0.048633476	0.009980111	-0.31583	-0.02202	0.019841	0.327087
MYO5B	-0.016290654	-0.029170712	-0.0388	0.072138	-0.1878	-0.03967
MYO5C	-0.00738629	-0.037886157	-0.03552	0.036309	-0.08651	-0.1005
MYO6	0.024506179	0.148554397	0.146112	0.0847	-0.15195	-0.19525
MYO7A	-0.004915343	-0.060285485	-0.23713	-0.02624	0.121872	0.58227
MYO7B	-0.051234582	-0.077810048	-0.00383	-0.02367	0.049658	0.097738
MYO9A	-0.007494425	0.000959994	0.134063	-0.0403	0.060148	-0.2655
MYO9B	0.019429624	-0.059272474	-0.16172	-0.02301	0.035974	0.152177
MYOC	-0.029815965	-0.052265455	0.053137	0.020154	-0.07797	-0.01881
MYOCD	-0.029450133	0.00766126	-0.01743	0.007393	-0.03002	-0.08413
MYOD1	-0.027370373	-0.093195312	0.02154	0.003365	-0.02337	0.093051
MYOF	-0.00874594	-0.112261506	-0.23851	-0.04992	0.214388	0.504009
MYOG	-0.025707302	-0.054816833	0.076671	-0.0026	-0.02645	-0.01484
MYOM1	-0.019525292	0.014822096	0.028986	-0.03713	0.105585	-0.10095
MYOM2	0.006827196	0.322705197	-0.07388	-0.1049	0.278307	-0.12974
MYOM3	-0.046887402	-0.089334844	-0.07857	0.027516	-0.08756	-0.00469
MYOT	-0.029078873	-0.013213253	-0.02052	-0.01386	0.029293	-0.08702
MYOZ1	-0.030772395	-0.044465431	-0.01879	0.01552	-0.07201	0.144275
MYOZ2	-0.083427979	-0.0949107	-0.07315	0.016028	-0.04558	-0.10898
MYOZ3	-0.028069481	-0.076268491	0.104266	-0.00436	-0.05377	-0.11009

MYPN	-0.036322789	-0.049853285	-0.01566	0.022799	-0.05589	-0.0134
MYPOP	0.004754858	-0.04278616	-0.01452	0.036347	-0.16527	0.119763
MYRF	-0.012968024	-0.104071921	-0.17177	0.027548	-0.01448	-0.01258
MYRFL	-0.02638118	-0.015232503	-0.00821	0.025618	-0.05876	0.060857
MYRIP	-0.059108946	0.002208476	0.002879	0.000356	0.043093	-0.1056
MYSM1	-0.015927424	0.099758537	0.019493	0.018656	-0.04351	-0.09528
MYT1	-0.040096803	-0.074534928	-0.10845	-0.00943	0.008267	-0.08608
MYT1L	-0.094755837	-0.059944349	0.078962	-0.06403	0.083347	-0.04136
MYZAP	-0.017610143	-0.04402377	-0.10403	-0.10581	0.23788	-0.15123
MZB1	-0.00100496	-0.090067258	-0.09689	0.032062	-0.0885	-0.19325
MZF1	0.015836929	-0.093356587	0.23142	-0.04224	0.035261	-0.12771
MZT1	0.007397678	0.053509141	0.058278	0.17414	-0.48024	-0.03484
MZT2B	0.047297505	0.013168589	0.131942	-0.00561	0.000427	-0.22146
N4BP1	0.051914066	0.023455152	-0.02258	0.030713	-0.05265	-0.18686
N4BP2	0.006411956	0.005418461	0.04765	0.050127	-0.14438	-0.10466
N4BP2L1	0.049934963	0.015938564	0.063247	-0.03781	0.101044	-0.00356
N4BP2L2	0.056057897	-0.032751842	0.028438	-0.03977	0.0829	-0.24118
N4BP2L2-[illegible]	-0.013302394	-0.043606232	0.014534	-0.0146	0.060018	-0.00469
N4BP3	-0.02029899	-0.031758362	-0.02208	0.051616	-0.12219	-0.11802
N6AMT1	0.085841412	-0.011275117	0.101004	0.105337	-0.29818	-0.12784
N6AMT2	0.014046231	0.041211332	0.212637	0.059073	-0.18813	-0.07874
NAA10	0.040656901	0.006627358	0.101598	0.076111	-0.2579	0.012802
NAA11	-0.019697646	-0.014890973	0.00544	-0.00065	-0.00894	-0.10822
NAA15	-0.043371517	0.032134235	0.037826	0.062569	-0.18869	0.010882
NAA16	0.015508214	0.03909137	0.475536	-0.01122	0.065525	-0.04402
NAA20	0.034150584	0.054366609	0.082164	0.058074	-0.15026	0.226177
NAA25	-0.022412014	-0.020100965	0.096754	0.062238	-0.0867	0.030134
NAA30	-0.021827771	0.040324209	0.102357	-0.00327	0.059267	-0.06738
NAA35	0.026181365	0.096058397	0.190013	0.068902	-0.18255	-0.00352
NAA38	0.032677458	0.0120707	-0.089	0.044615	-0.12948	0.296167
NAA40	0.019658971	0.120009181	0.12527	0.038499	-0.12665	-0.33566
NAA50	0.028442603	0.087965175	-0.08209	0.037965	-0.11913	-0.06888
NAA60	0.054350297	-0.018896118	-0.04781	0.004334	-0.00446	0.144668
NAAA	0.024663932	-0.102664657	-0.14729	-0.07089	0.18967	0.476316
NAALAD2	-0.093774248	-0.11358179	-0.06516	0.107718	-0.0281	-0.07699
NAALADL1	0.001707135	0.161559592	0.011685	-0.05436	0.146821	0.039718
NAALADL2	-0.095711794	-0.012024763	0.044999	-0.02605	0.064962	-0.00909
NAALADL2-	-0.07599625	-0.135870271	0.024523	-0.01388	0.051674	0.020944
NAB1	-0.019221215	-0.016456384	-0.14652	-0.00097	-0.06625	0.061682
NAB2	-0.010257947	-0.05347863	-0.00468	-0.00235	-0.05058	0.038833
NABP1	0.021814154	0.043286344	-0.18227	0.010729	-0.0422	0.172127
NABP2	0.011344118	0.042773436	0.008508	0.113774	-0.31577	-0.01616
NACA	0.062120237	0.061186018	0.027965	0.080966	-0.07415	0.144648
NACA2	-0.032885854	-0.06033896	0.008245	0.035019	-0.08561	0.075954
NACAD	-0.034225368	-0.009909331	-0.01719	0.054946	-0.14043	0.029552
NACAP1	0.150420096	0.126619768	-0.0133	0.09541	-0.18111	-0.19405
NACC1	0.006994284	-0.061746534	0.014783	0.03522	-0.18682	-0.02756
NACC2	0.00759862	0.02372906	-0.16738	-0.08489	0.229763	0.533901
NADK	0.008684575	-0.068040778	-0.30717	-0.06091	0.12888	0.194877
NADK2	-0.003040243	0.09454803	0.014909	0.046679	-0.09971	-0.00982
NADK2-AS[illegible]	-0.038884853	-0.100422636	0.026059	0.001076	0.019439	0.000177

NADSYN1	0.028812409	0.01140077	-0.23647	0.015056	-0.05566	0.133717
NAE1	0.031446312	0.127722363	0.361524	0.058248	-0.15744	-0.40291
NAF1	0.004401623	0.023034858	0.03057	0.077966	-0.1849	0.07397
NAG18	-0.05151192	-0.01139078	0.112467	-0.06923	0.216357	-0.05001
NAGA	0.006675107	-0.073388897	-0.29526	-0.05304	0.123988	0.516797
NAGK	0.037615549	-0.097940988	-0.48004	-0.08028	0.231953	0.492348
NAGLU	-0.009924811	0.041000368	-0.06333	0.069933	-0.16253	0.110892
NAGPA	0.023387686	-0.032718168	-0.03641	-0.06459	0.204341	0.354559
NAGS	-0.020476088	0.045746726	-0.15237	-0.07978	0.217213	0.320742
NAIF1	-0.001663379	-0.015604801	0.056964	0.020174	-0.01945	0.006779
NAIP	0.003399608	-0.093255608	-0.22002	-0.07402	0.251776	0.419419
NALCN	-0.088571937	-0.081266665	0.045112	-0.03565	0.094937	-0.05253
NALCN-AS1	-0.025548997	-0.073803432	0.076741	0.015714	-0.05738	-0.01102
NAMPT	0.008419021	-0.09086754	-0.26702	0.010502	-0.054	0.145545
NANOG	-0.02489142	-0.072523197	0.033365	0.017887	-0.05901	-0.2183
NANOS1	-0.015821209	-0.015455923	-0.02304	0.007196	-0.03151	-0.05482
NANOS2	-0.036281008	-0.058040965	-0.07925	0.051524	-0.1287	0.051275
NANOS3	-0.022527202	-0.036005583	-0.00124	0.018121	-0.04795	0.134001
NANP	0.001280074	0.139230191	-0.16988	0.06437	-0.19759	0.13632
NANS	0.023512394	-0.085205197	-0.25193	0.067472	-0.18306	0.497933
NAP1L1	0.057763025	-0.062726033	0.169818	0.030923	-0.06171	-0.08092
NAP1L2	-0.003858207	0.130694468	0.335017	-0.05734	0.179042	-0.31458
NAP1L3	-0.005858495	0.083084516	0.436436	-0.12029	0.345169	-0.3575
NAP1L4	0.011460328	0.041276129	0.418498	0.015482	-0.06668	-0.37922
NAP1L5	0.006536836	0.203476167	0.196607	0.040532	-0.06719	-0.23378
NAPA	0.025329065	0.020844611	-0.16629	0.019336	-0.03373	0.246933
NAPA-AS1	-0.038323215	-0.132304007	-0.0555	0.040555	-0.1295	-0.06087
NAPB	-0.001879797	0.045614096	0.123517	-0.04393	0.111606	-0.06019
NAPEPLD	-0.004065891	0.044215187	0.106324	-0.05588	0.028622	-0.13404
NAPG	0.020653955	0.06529049	-0.27636	0.078973	-0.21497	0.153788
NAPRT	0.017749072	-0.141374661	-0.14205	-0.088	0.222341	0.387549
NAPSA	-0.008586318	-0.101611246	-0.08992	0.076471	-0.18778	0.010238
NAPSB	0.000655916	-0.142615983	-0.31468	-0.0371	0.127527	0.235103
NARF	0.023915628	0.051948965	-0.15651	0.042659	-0.10904	-0.12219
NARFL	0.002879785	-0.077746267	-0.03501	-0.00455	-0.10205	-0.04343
NARS	0.063412836	0.062044803	-0.06148	0.031572	-0.07747	0.32212
NARS2	0.019307891	-0.023656591	0.150799	0.061319	-0.17375	-0.01265
NASP	0.027597025	0.076611614	0.055498	0.068026	-0.2371	-0.25463
NAT1	0.029541978	0.104696405	-0.32457	0.039927	-0.12094	0.114808
NAT10	0.030414066	0.004148438	0.156078	0.05782	-0.17847	-0.16418
NAT14	-0.007024337	-0.052770404	-0.00708	0.10665	-0.30392	-0.04326
NAT16	-0.032128232	-0.088442327	-0.03336	0.012612	-0.07105	0.077479
NAT2	-0.025961444	-0.021008562	0.05423	-0.01288	0.040996	0.005206
NAT6	0.006136665	-0.058906897	0.111174	0.007826	-0.05691	-0.11625
NAT8B	-0.017850824	-0.025925013	-0.06787	-0.2442	0.692464	0.051986
NAT8L	-0.013466486	-0.074969667	-0.04792	-0.01207	-0.02274	-0.12143
NAT9	0.015982919	-0.155686732	0.040194	-0.01686	0.013023	0.153212
NATD1	0.006626488	0.001456472	-0.19615	-0.08001	0.200985	0.160239
NAV1	-0.031678506	-0.098281983	-0.01379	-0.03552	0.059604	-0.01086
NAV2	-0.088241803	-0.040208283	0.014118	0.086596	-0.0948	-0.10457
NAV2-AS2	-0.040401626	-0.019031096	-0.0299	0.023511	-0.04097	0.067078

NAV2-AS4	-0.024066173	-0.057199707	-0.07945	-0.01024	-0.02707	-0.00571
NAV2-AS5	-0.035020704	-0.066785856	0.019238	0.003782	0.011509	-0.17111
NAV2-IT1	-0.032508713	-0.103609991	-0.00293	0.021713	-0.0438	-0.09087
NAV3	-0.021556226	-0.14463199	-0.06674	0.016637	-0.01419	0.074477
NBAS	-0.015087161	0.002362	0.059223	-0.01988	0.063716	0.081576
NBEA	-0.015859983	-0.137497272	0.215894	-0.06652	0.208806	-0.28249
NBEAL1	-0.020167598	0.044118087	0.002574	0.003549	0.000133	0.292509
NBEAL2	0.014199486	0.13582994	-0.11599	-0.0146	0.049467	0.163419
NBEAP1	-0.055244159	-0.05273709	0.011452	0.00492	-0.03056	-0.11904
NBL1	-0.007110173	-0.071714384	0.169319	0.004652	-0.09395	-0.09976
NBN	0.054838044	0.054405496	-0.31442	0.07596	-0.11366	0.133395
NBPF1	0.05860594	-0.027579225	0.009136	-0.00728	0.065873	-0.19379
NBPF20	-0.017183839	-0.077906837	0.02931	0.001868	-0.06849	-0.04172
NBPF3	-0.005438416	0.054192947	0.199325	-0.00574	0.058501	-0.09018
NBPF4	-0.020463142	-0.048254202	0.018921	0.00536	-0.01486	-0.14604
NBPF8	-0.038181449	-0.067159419	0.092859	-0.02255	0.096476	-0.06776
NBR1	0.012836608	0.083818162	-0.03698	-0.02709	0.06602	0.000449
NBR2	0.001329426	-0.056845009	-0.07136	-0.02897	0.044749	-0.0119
NCALD	-0.000423335	0.233218952	0.307172	0.044141	-0.13193	-0.29009
NCAM1	-0.042667732	0.436386729	-0.13965	-0.09697	-0.00254	-0.05584
NCAM2	-0.044422301	-0.074521397	-0.03103	0.035618	-0.08016	-0.10261
NCAN	-0.028635627	-0.003959868	-0.06218	0.026746	-0.07578	0.019249
NCAPD2	0.016388852	0.053602489	0.155032	0.052557	-0.13325	-0.06337
NCAPD3	0.018730216	0.068615765	0.200275	0.122389	-0.34416	-0.11918
NCAPG	-0.011774956	0.015119104	-0.00314	0.159986	-0.29656	-0.05148
NCAPG2	-0.000391867	0.025282664	0.107811	0.099795	-0.27938	-0.12235
NCAPH	-0.007811883	0.00693991	0.05586	0.18547	-0.49403	-0.06701
NCAPH2	-0.030512266	-0.130389526	0.064576	0.076998	-0.29281	-0.17751
NCBP1	0.001323833	0.078187435	-0.02573	0.070918	-0.16866	0.145928
NCBP2	0.02227918	0.022993845	0.13294	0.051788	-0.15655	0.04327
NCBP2-AS	0.040699984	0.027582165	0.165523	0.023337	-0.06862	0.001023
NCDN	-0.026261469	-0.119992545	0.083193	0.04366	-0.19906	-0.04228
NCEH1	-0.003361904	0.031058775	-0.15942	0.031886	-0.07714	0.502828
NCF1	0.008484221	-0.218359397	-0.32614	-0.10156	0.274677	0.25585
NCF2	0.014022254	-0.171489851	-0.39627	-0.06268	0.176222	0.38682
NCF4	0.011813888	-0.183584941	-0.36647	0.020328	-0.05008	0.193512
NCK1	0.018926211	0.204523269	0.137871	-0.00163	0.022996	0.039633
NCK1-AS1	0.004889038	0.067161941	0.042189	0.028278	-0.05265	0.16972
NCK2	0.035112809	-0.066264887	0.3582	-0.03775	0.089542	-0.52382
NCKAP1	-0.022385419	0.203618088	0.054617	-0.08271	0.238586	-0.10291
NCKAP1L	0.033601565	0.053954048	-0.13734	-0.01761	0.105499	0.140747
NCKAP5	-0.02979518	-0.025143725	-0.02528	0.036863	-0.0036	-0.02728
NCKAP5L	-0.020117382	-0.073824475	-0.08156	-0.04298	0.113106	0.417034
NCKIPSD	-0.01547543	-0.084827388	-0.00759	0.001549	-0.14277	-0.03068
NCL	0.027645735	0.103040818	0.189088	0.065508	-0.16437	-0.17018
NCLN	-0.101653063	-0.056483803	0.044925	-0.09524	0.030596	-0.20113
NCOA1	0.053457748	0.144555029	-0.18835	-0.01834	0.112732	-0.08377
NCOA2	-0.011331495	0.034910815	-0.12487	-0.01334	-0.04523	-0.0361
NCOA3	0.040417312	-0.037368953	-0.09235	-0.08008	0.135225	-0.14211
NCOA4	0.054288526	-0.039025239	-0.38879	-0.03905	0.135775	0.201619
NCOA5	0.001547576	0.042170952	0.031225	0.038407	-0.15883	-0.19735

NCOA6	0.042193416	0.094239043	-0.15703	0.029934	-0.04328	-0.11083
NCOA7	0.00289144	0.134466628	-0.05361	0.030816	-0.05151	-0.16754
NCOR1	-0.019609567	0.048488415	-0.1257	-0.0348	-0.04737	-0.05768
NCOR2	-0.063817732	-0.090300028	0.01337	-0.02746	-0.03554	0.030758
NCR1	-0.013947288	0.409078398	-0.03836	0.020542	-0.06612	-0.11399
NCR2	-0.020445062	-0.035339302	-0.02612	0.0248	-0.12508	-0.11939
NCR3	-0.00077673	0.221822599	0.268857	0.024108	-0.11607	-0.32626
NCS1	-0.031138254	-0.02840188	-0.06941	-0.02059	-0.0766	-0.00563
NCSTN	0.022148821	-0.076726079	-0.34862	-0.01421	0.051206	0.197183
ND2	0.084331455	0.110738782	-0.05643	0.10024	-0.07279	0.039403
ND4	0.107691825	0.208292498	-0.20709	0.180308	-0.10102	-0.10284
ND6	0.038403417	0.005882096	0.034842	-0.04204	0.115012	-0.18573
NDC1	0.014028345	0.037170196	0.082407	0.098023	-0.26314	0.0023
NDC80	0.001645411	0.073861117	0.166979	0.081365	-0.20525	-0.19846
NDE1	-0.043028897	-0.052955005	-0.05087	-0.03978	0.046617	-0.01586
NDEL1	0.031256447	0.050562512	-0.14016	0.023364	-0.03579	-0.0824
NDFIP1	-0.026957506	-0.070600243	0.383947	-0.05333	0.112287	0.076114
NDFIP2	-0.016652026	0.067189142	-0.00425	0.116753	-0.29571	-0.14386
NDN	0.003878472	-0.038908893	-0.09453	-0.01188	0.031752	0.03814
NDNF	-0.033309519	-0.009546059	-0.00289	0.031096	-0.08533	-0.13338
NDNL2	0.011876266	0.042798936	0.352385	-0.00163	0.002351	-0.21075
NDOR1	-0.025384356	-0.066987563	-0.05424	-0.00865	-0.05402	0.015008
NDP	-0.036172388	-0.043901794	-0.05187	-0.01609	0.066873	-0.00119
NDRG1	0.050723111	-0.039844624	-0.03708	-0.04106	0.120239	-0.01496
NDRG2	0.030787861	-0.096578615	0.055002	-0.13544	0.498989	-0.11724
NDRG3	-0.010259144	0.025376636	0.046867	-0.05116	-0.03112	-0.10857
NDRG4	-0.025414071	-0.06544073	0.086371	0.042704	-0.10712	-0.06283
NDST1	-0.007399386	-0.102046845	-0.1461	-0.05051	0.098345	0.287678
NDST2	0.019718291	-0.079047809	-0.08769	-0.01444	0.046578	0.25587
NDST3	-0.050800652	-0.028705899	0.037107	0.002711	0.020938	-0.03657
NDST4	-0.04744537	-0.06480691	-0.00493	0.025532	-0.05786	-0.03551
NDUFA1	0.094692194	0.065111409	0.056051	0.160969	-0.36653	0.074043
NDUFA10	0.028311295	-0.022647294	-0.01189	-0.08283	-0.14887	0.103374
NDUFA11	0.021195394	0.026481166	0.095851	0.083916	-0.23183	0.227083
NDUFA12	0.045106266	0.12587784	0.112725	0.056278	-0.15342	0.133851
NDUFA13	0.058596422	0.058369893	0.057241	0.119846	-0.31878	0.223289
NDUFA2	-0.018552746	0.069004391	0.184603	-0.00018	-0.00193	-0.13059
NDUFA3	0.054632988	0.06580823	0.225412	0.082119	-0.2214	0.108759
NDUFA4	0.049931646	0.030461734	0.160335	0.071086	-0.18739	0.08804
NDUFA4L2	-0.024455247	-0.030165406	-0.07781	0.027754	-0.07111	0.142726
NDUFA5	0.021349581	0.073071713	0.224158	-0.00573	0.023837	-0.02753
NDUFA6	0.067949321	0.140242433	0.073912	0.012954	-0.04142	0.168818
NDUFA7	0.031015839	-0.024428883	0.04457	0.098498	-0.26143	0.200021
NDUFA8	0.029241219	0.072231906	0.022806	0.062873	-0.16204	0.226786
NDUFA9	0.040536682	0.064774206	0.08835	0.051344	-0.14442	0.094356
NDUFAB1	0.04666829	0.060669118	0.106177	0.068465	-0.18544	0.187941
NDUFAF1	0.027271695	0.066705409	-0.17627	0.04774	-0.17157	0.141739
NDUFAF2	0.024609233	-0.009573409	0.219439	0.053001	-0.13925	0.231756
NDUFAF3	0.032116731	0.025437515	0.142904	0.014963	-0.04508	0.149936
Ndufaf4	-0.032431781	-0.023498433	0.004668	0.012799	-0.01888	0.071894
NDUFAF4	0.014673117	0.072449572	0.230216	0.049731	-0.1545	-0.27982

NDUFAF5	0.008647956	0.043751562	-0.009	0.103215	-0.24797	0.067593
NDUFAF7	0.030898686	0.029122652	-0.0208	0.0365	-0.04011	0.09637
NDUFB1	0.067815124	0.08518938	0.034062	0.076831	-0.14364	0.246024
NDUFB10	0.04833178	-0.00946797	0.079444	0.059184	-0.17383	0.21028
NDUFB11	0.047662748	0.018288902	0.197383	0.060625	-0.17156	0.107504
NDUFB2	0.040888048	0.130356185	0.120735	0.042997	-0.11128	0.216493
NDUFB2-AS	-0.006869135	0.085573608	0.160953	-0.01413	0.036724	-0.21745
NDUFB3	0.08006187	0.040663739	-0.04919	0.109117	-0.26722	0.112756
NDUFB4	0.03817682	0.029827724	0.164763	0.067416	-0.14241	0.048144
NDUFB5	0.054811257	-0.023897142	0.041533	0.043478	-0.1193	0.250977
NDUFB6	0.050805575	0.056157208	0.057375	0.102455	-0.26018	-0.08885
NDUFB7	0.0131402	0.104862269	0.053992	0.070683	-0.1689	0.22154
NDUFB8	0.076861028	0.078537386	0.214888	0.103961	-0.28419	0.050286
NDUFB9	0.055594702	0.059176923	0.217352	0.064438	-0.17671	0.207928
NDUFC1	0.035139237	-0.016237289	0.213044	0.04105	-0.11026	0.035038
NDUFS1	-0.022342821	0.085349295	0.007483	0.005843	-0.00879	0.142917
NDUFS2	0.080797032	0.055241878	-0.05715	0.088825	-0.22805	0.135868
NDUFS3	0.048289934	0.063077889	0.001511	0.056519	-0.15247	0.241962
NDUFS4	0.016647464	0.008368922	0.110091	0.036925	-0.07982	0.181758
NDUFS6	0.068501394	0.109662848	-0.00138	0.140435	-0.36631	0.22033
NDUFS7	0.014745704	-0.009608088	-0.03869	0.056459	-0.14692	0.364812
NDUFV1	0.031614501	0.079859494	0.16683	0.093538	-0.26368	0.007899
NDUFV2	0.059483348	-0.062816167	0.040903	0.104163	-0.3024	-0.03864
NDUFV2-AS	-0.005770459	-0.107126443	0.250875	-0.0418	0.146938	-0.20054
NDUFV3	0.018617815	-0.046413092	-0.14482	0.042584	-0.09927	0.497192
NEB	-0.039743003	0.102159232	-0.01194	-0.01259	0.063536	-0.15925
NEBL	-0.03525879	-0.076005414	-0.10658	0.047981	-0.11121	0.296384
NEBL-AS1	-0.033024419	-0.012475273	-0.13407	0.018346	-0.02116	0.164752
NECAB1	-0.056181018	-0.101449579	-0.07094	0.052898	-0.05703	0.006574
NECAB2	-0.014653455	-0.085339479	-0.09909	-0.00717	-0.06813	-0.0804
NECAB3	-0.011632911	-0.070180113	0.050103	0.036703	-0.19858	0.001933
NECAP1	0.042657482	-0.030038676	-0.03514	-0.01841	0.042192	-0.04784
NECAP2	0.032154615	-0.053806507	0.153793	-0.01072	0.026286	0.200724
NEDD1	0.04528456	0.177241775	0.248662	0.042498	-0.16196	-0.33598
NEDD4	-0.006887767	0.080106577	0.052748	0.004101	0.027644	0.052113
NEDD4L	-0.006331754	-0.018207668	0.008837	-0.01197	0.047196	0.126388
NEDD8	0.096428573	0.086318137	0.025842	0.090317	-0.19581	-0.08198
NEDD9	0.046078867	0.007903439	-0.20884	-0.03063	0.019438	-0.06399
NEFH	-0.01101653	-0.055410691	0.021015	0.11356	-0.29363	0.240529
NEFL	0.005086497	-0.098544555	0.161991	-0.02006	0.192522	-0.16638
NEFM	-0.05508462	0.003605364	0.124251	0.023881	-0.04171	-0.07842
NEGR1	-0.005239037	-0.069024471	-0.09865	-0.01334	0.108386	-0.0228
NEGR1-IT	-0.021151327	-0.05451441	-0.03728	0.002449	-0.01901	-0.01481
NEIL2	-0.004397338	-0.012654212	0.133506	0.043043	-0.1303	-0.29124
NEIL3	-0.013478143	-0.002969282	0.023681	0.092534	-0.22075	-0.07703
NEK1	-0.009618749	0.094548703	0.180248	0.030143	-0.0758	-0.10272
NEK10	-0.0389001	-0.052389608	-0.00461	0.033069	-0.06808	0.031179
NEK11	-0.026124226	-0.081528433	0.038937	0.020626	0.052952	-0.15085
NEK2	-0.017649933	0.011706274	0.016686	0.150987	-0.29592	-0.06673
NEK3	0.00075401	-0.021815843	-0.32703	-0.09522	0.286532	0.383519
NEK4	0.009607031	0.019373255	-0.16646	0.036866	-0.08729	0.451469

NEK5	-0.059560182	-0.0108742	0.136905	-0.0059	0.021778	-0.16035
NEK6	-0.020412783	-0.056817027	-0.27833	-0.06185	0.101228	0.121977
NEK7	0.078870129	0.163161067	0.025157	0.049306	-0.10769	-0.08738
NEK8	0.011619132	-0.083825774	0.046115	-0.02919	0.04031	-0.20384
NEK9	-0.010270104	-0.089408274	0.093745	0.000495	-0.06364	-0.06592
NELFB	0.040769981	0.084817567	0.00538	0.029314	-0.08929	0.097695
NELFCD	0.03829866	0.045611559	0.198108	0.039754	-0.09952	-0.05627
NELL1	-0.041752844	-0.037571863	0.033566	0.038948	-0.11303	-0.05659
NELL2	0.002882462	-0.077684085	0.606291	-0.06044	0.16428	-0.21176
NEMF	0.078899665	0.078847902	0.058124	0.020542	-0.06885	-0.22253
NENF	-0.010679457	-0.117077055	0.018976	-0.01191	-0.06072	-0.06384
NEO1	0.02024211	0.159959673	0.147228	0.005733	0.020233	-0.19492
NES	-0.036809909	-0.074503723	-0.06742	0.026068	-0.06611	-0.02138
NET1	0.003078319	-0.038091764	-0.0184	0.047227	-0.1458	-0.26336
NETO1	-0.006367929	-0.095433065	-0.09975	0.012178	0.078743	-0.17565
NETO2	-0.00460369	-0.05838712	-0.13798	-0.04588	0.096021	0.355769
NEU1	0.024537324	0.04799028	-0.39348	0.016116	-0.03765	0.445149
NEU2	-0.033218754	-0.098982181	-0.14033	0.024171	-0.06561	0.09413
NEU3	-0.038057738	-0.035126332	-0.06348	-0.00573	-0.07237	0.027915
NEU4	-0.004145661	-0.06623589	-0.022	-0.00376	-0.10354	-0.13608
NEURL1	-0.029261842	-0.138266794	-0.11035	-0.05489	0.086231	0.159522
NEURL1B	-0.020406832	0.008181541	0.005651	-0.02436	0.020499	-0.14293
NEURL2	-0.033711308	-0.129983265	0.165673	1.88E-05	0.036986	0.001931
NEURL3	-0.007694202	-0.01453766	-0.03377	-0.00503	-0.03247	-0.01786
NEURL4	0.005505598	-0.061423744	0.033151	0.015319	-0.05683	0.001906
NEUROD1	-0.041075173	-0.019696934	0.099141	0.00933	-0.01292	-0.09971
NEUROD2	-0.042342201	-0.048218771	-0.02393	0.03753	-0.131	0.029283
NEUROD4	-0.032509226	-0.044941626	0.016953	-0.02654	0.089062	0.033757
NEUROD6	-0.034563928	-0.035433696	-0.0369	0.018107	-0.06126	-0.12458
NEUROG1	-0.035536679	-0.097140166	-0.14456	0.00593	-0.00247	0.209479
NEUROG2	-0.043472078	-0.028214343	-0.08044	-0.00179	0.006308	0.058418
NEUROG3	-0.033654535	-0.04308606	-0.06052	0.02935	-0.05037	0.063858
NEXN	-0.005205366	-0.020709036	-0.15667	-0.16962	0.479088	0.432167
NEXN-AS1	-0.106242769	-0.067695197	-0.07191	-0.11709	0.270486	-0.02006
NF1	-0.027722795	-0.059952062	0.054267	0.008551	-0.01405	-0.10366
NF2	-0.056724278	0.075245816	0.014482	0.088371	-0.21003	0.096827
NFAM1	0.003799721	-0.113687461	-0.27561	-0.06654	0.165434	0.480633
NFASC	-0.038033909	-0.101372077	-0.01192	-0.00375	-0.06182	-0.03499
NFAT5	0.001922231	0.083606205	-0.04784	0.010902	-0.07779	-0.09795
NFATC1	0.046138509	-0.022503308	0.108953	-0.06356	0.197385	-0.00405
NFATC2	0.003958464	0.190710895	0.266887	0.011258	-0.03051	-0.60292
NFATC2IP	0.051079681	0.117131674	0.167727	-0.04857	0.028174	-0.06747
NFATC3	0.017977006	0.205493369	0.295853	-0.01614	0.041547	-0.35981
NFATC4	-0.022249108	-0.098051054	-0.05376	0.000796	-0.01682	0.064309
NFE2	-0.002755286	-0.110003593	-0.32585	-0.09385	0.264696	0.449888
NFE2L1	0.039810523	0.085114473	-0.11119	0.043627	-0.18083	0.139946
NFE2L2	0.005628454	-0.012229085	-0.16673	-0.01746	0.010579	0.174758
NFE2L3	-0.007100945	-0.042603698	-0.09693	0.096785	-0.27803	0.242855
NFE4	-0.012661051	-0.062053689	-0.15701	-0.01141	0.016884	-0.12452
NFIA	0.025410293	-0.071687996	0.329655	-0.09982	0.306146	0.234465
NFIA-AS2	-0.043653908	0.001408787	-0.02514	-0.00161	-0.0982	0.140041

NFIB	-0.061850411	-0.052496648	-0.02095	-0.05343	0.16048	-0.1251
NFIC	0.021488891	-0.128856887	-0.03472	-0.04943	0.205029	0.434134
NFIL3	0.018510837	0.085432775	-0.26648	0.042663	-0.11737	0.11252
NFIX	-0.03586958	-0.097316139	-0.10508	0.030501	-0.07326	0.175157
NFKB1	0.030669893	-0.020452432	0.050123	0.011423	-0.02933	-0.1007
NFKB2	-0.003721504	-0.033531168	-0.07077	0.070988	-0.20476	0.112681
NFKBIA	0.019669773	-0.056347694	-0.17416	0.024949	-0.08763	0.137002
NFKBIB	0.013070811	0.054074447	-0.05493	0.086047	-0.24376	-0.10416
NFKBID	-0.021030562	-0.040057305	-0.09466	-0.03346	0.002554	0.013188
NFKBIE	0.018116568	-0.096560134	-0.22296	0.020167	-0.09919	0.274639
NFKBIL1	-0.005040501	-0.130255422	-0.15066	0.075234	-0.26615	0.193432
NFKBIZ	0.030891321	-0.134996432	-0.06444	-0.02426	0.081222	0.17906
NFRKB	-0.017987471	-0.048813696	0.147007	-0.00621	-0.0554	-0.09081
NFS1	0.005019075	0.037392532	0.091759	0.071475	-0.18768	0.083445
NFU1	0.030652645	0.047670031	0.173797	0.032729	-0.08865	-0.07822
NFX1	-0.003500371	-0.036845454	0.075416	-0.01896	-0.02118	-0.24401
NFXL1	0.030093553	-0.023271194	-0.11748	0.024485	-0.06386	0.177272
NFYA	-0.008347672	-0.028175163	-0.09819	-0.0052	-0.0328	-0.16242
NFYB	0.008633552	0.103044878	0.047307	0.034616	-0.07195	-0.03867
NFYC	0.018134999	-0.061201436	-0.33434	0.047172	-0.12208	0.239349
NFYC-AS1	-0.007735105	-0.007387691	0.131565	0.013679	-0.0483	-0.02795
NGB	-0.035645166	-0.039858532	-0.03979	0.008394	-0.07479	-0.08928
NGDN	0.0215358	0.055612984	0.175525	0.018524	-0.04671	-0.1118
NGEF	-0.062658134	-0.05224184	-0.04204	-0.00621	0.049555	0.012103
NGF	-0.019944953	-0.080880323	0.031298	0.027006	-0.06932	0.004212
NGFR	-0.032850727	-0.014900928	-0.02637	-0.00385	0.033703	0.013621
NGFRAP1	0.011149092	-0.103604448	0.397335	-0.15053	0.395412	-0.21847
NGLY1	0.038218835	0.039199044	-0.02799	0.015354	-0.03757	-0.43186
NGRN	0.049907387	0.051590666	0.282993	0.007816	-0.18734	-0.23936
NHEG1	-0.037644513	-0.017112208	0.05072	0.000357	-0.01344	-0.01706
NHLH1	-0.014983959	-0.083754157	-0.05812	-0.01238	-0.04448	0.044442
NHLH2	-0.061436328	-0.044254157	-0.03701	0.032996	-0.04572	0.055734
NHLRC2	-0.001050687	0.038016627	-0.0494	0.104782	-0.04561	0.049584
NHLRC3	0.019191063	0.02914163	0.032077	0.040636	0.082894	0.124285
NHLRC4	-0.003946236	-0.066711478	0.025404	-0.01933	-0.06276	-0.1384
NHP2	0.03594278	0.004528926	0.184453	0.054783	-0.15589	0.044384
NHP2L1	0.033355367	0.012350888	0.191501	0.005677	-0.02683	-0.13947
NHS	-0.011841564	-0.086784607	-0.20877	-0.02025	0.063174	0.49574
NHSL1	0.021190371	-0.152072402	-0.14992	-0.07751	0.119096	0.388835
NHSL2	-0.025529237	-0.040903274	-0.13743	-0.01401	0.0315	-0.03089
NICN1	0.020068058	0.008943021	0.056155	0.011511	-0.0528	########
NID1	-0.053601585	-0.077378844	-0.15181	-0.1139	0.213963	0.710321
NID2	-0.030716822	-0.011855482	0.030343	-0.04229	0.131429	-0.03984
NIF3L1	0.024499867	0.028419072	0.106159	0.038843	-0.10373	0.020997
NIFK	0.043656034	0.068811124	0.13598	0.062767	-0.082	-0.02187
NIFK-AS1	0.001590988	0.125730407	0.21711	0.038749	-0.12348	-0.15567
NIM1K	-0.026344961	-0.015138338	0.102191	0.061555	-0.16929	-0.04604
NIN	-0.000765334	-0.109877168	-0.16586	-0.0424	0.058981	-0.05138
NINJ1	0.017180842	-0.043977162	-0.13533	-0.00049	0.000879	0.291027
NINJ2	0.014442009	-0.033816404	-0.22361	-0.01727	0.036715	0.403675
NINL	-0.008913793	0.115759979	0.221173	-0.0314	0.068372	-0.18491

NIP7	0.024508021	0.102988131	0.148002	0.060386	-0.19409	-0.11483
NIPA1	-0.003103914	0.010921935	0.023616	-0.00984	-0.00965	-0.16653
NIPA2	0.043195988	0.050590609	-0.16403	0.032439	-0.09874	-0.08967
NIPAL2	0.088402783	0.086972102	-0.39626	-0.03433	0.131498	0.212967
NIPAL3	0.045192048	0.034333125	0.305969	-0.01856	0.119816	-0.28529
NIPAL4	-0.020932756	-0.047234943	-0.01991	0.005663	-0.05818	-0.09031
NIPBL	0.0462726	0.088499696	-0.00194	-0.11669	0.103607	-0.16853
NIPSNAP1	0.025253923	0.014769252	0.313458	0.085356	-0.2326	-0.25306
NIPSNAP3/	0.017680835	-0.025389356	-0.17916	0.000796	0.016211	0.386159
NIPSNAP3I	-0.015897749	-0.081314616	-0.10179	-0.05203	0.168902	-0.19703
NISCH	0.065997513	0.022116564	0.063957	0.004783	0.023832	0.006703
NIT1	0.0189925	-0.099074763	-0.09114	-0.03935	0.099411	0.33055
NIT2	0.025676738	0.051162351	0.139054	0.087135	-0.22966	0.025481
NKAIN1	-0.032612902	-0.029890911	0.057068	0.001858	-0.02409	-0.07567
NKAIN2	-0.056080756	-0.071964632	0.070903	-0.02786	0.058772	-0.09673
NKAIN3	-0.035142296	-0.101487898	0.048769	0.026618	-0.05299	-0.0291
NKAIN4	-0.082107671	-0.088545747	-0.18233	-0.0054	-0.05634	0.177977
NKAP	0.043104439	0.005537242	-0.19673	0.060605	-0.15693	0.214387
NKAPL	-0.050534564	-0.060454999	-0.16685	-0.01203	0.069635	0.215253
NKAPP1	0.001943292	0.034898993	0.149926	-0.01466	0.069553	-0.21627
NKD1	-0.086893691	-0.0145031	-0.10577	0.012685	-0.06453	0.008617
NKD2	-0.023069076	-0.111944198	-0.06931	0.0037	-0.04149	0.095977
NKG7	0.014990656	0.280655812	0.425559	0.021201	-0.05757	-0.1591
NKIRAS1	-0.006008547	0.032617227	-0.00731	0.049489	-0.09642	0.081137
NKIRAS2	0.034198869	-0.082233955	-0.28579	-0.01287	0.025801	0.466813
NKPD1	-0.021921619	-0.05702989	0.07718	0.014147	-0.05995	-0.13879
NKRF	0.033503355	-0.075041661	0.122309	0.043187	-0.18399	-0.28092
NKTR	-0.037078883	0.022889313	0.18179	-0.04213	0.03336	-0.10661
NKX1-1	-0.019694248	-0.063979934	-0.05525	0.017315	-0.0765	0.119298
NKX2-1	-0.044311227	-0.055214891	0.005627	0.014547	-0.02797	0.028587
NKX2-1-AS	-0.03549025	-0.066924493	-0.01088	-0.02253	0.091176	-0.0288
NKX2-2	-0.049355345	0.017652829	0.133408	-0.01441	0.050401	-0.13196
NKX2-3	-0.060414523	-0.052320421	-0.0293	-0.0181	0.05163	0.131886
NKX2-5	-0.034143619	-0.021869314	0.077118	0.009527	-0.04224	0.026234
NKX2-8	-0.030679008	-0.058128198	-0.08279	-0.00347	0.006055	0.121314
NKX3-1	-0.019742193	-0.095966515	-0.00319	0.018515	0.013589	-0.11153
NKX3-2	-0.032015541	-0.022586925	0.009534	0.007658	-0.00788	-0.03099
NKX6-1	-0.02120187	-0.020103745	-0.1464	0.012941	-0.00757	0.094074
NKX6-2	-0.037271106	-0.082184728	-0.016	0.025794	-0.0591	0.033164
NKX6-3	-0.02758372	-0.0739958	-0.08207	-0.00924	-0.0613	-0.08624
NLE1	-0.0113717	-0.057255945	0.067537	0.062694	-0.2049	-0.04833
NLGN1	-0.027358608	-0.025558451	0.009988	0.127811	-0.09503	-0.03368
NLGN2	0.001106392	-0.07547411	0.045666	0.023356	-0.0866	-0.10141
NLGN3	-0.031619707	-0.084720444	0.054973	0.012818	-0.04278	-0.10473
NLGN4X	-0.041689069	-0.067316768	-0.03177	-0.00737	0.011359	-0.18794
NLGN4Y	-0.076169394	-0.071658198	-0.06727	-0.00662	0.019083	-0.07727
NLK	0.009755502	0.086783237	-0.06587	-0.03717	0.01738	0.022771
NLN	-0.025673014	-0.112357371	-0.11616	0.018541	-0.07413	0.606018
NLRC3	0.007916443	0.158336644	0.325558	0.001068	-0.00701	-0.37926
NLRC4	-0.004893007	-0.088357391	-0.33208	-0.13202	0.375537	0.639556
NLRC5	0.042498481	0.043588096	0.375031	-0.05243	0.151364	-0.31316

NLRP1	-0.035316914	-0.012813199	0.064829	-0.10234	0.19124	0.07498
NLRP10	-0.037560683	-0.011510499	-0.06521	-0.01196	0.060306	0.033501
NLRP11	-0.02850289	-0.039449018	0.023201	0.049528	-0.13924	-0.07685
NLRP12	-0.005036979	-0.132364084	-0.3056	-0.09247	0.239646	0.50847
NLRP13	-0.034128706	-0.05299562	-0.00592	-0.00721	-0.00207	-0.04858
NLRP14	-0.030007208	-0.070202629	-0.09027	-0.02633	0.060107	-0.06516
NLRP2	-0.013074595	0.218116589	-0.02558	-0.01404	0.010802	-0.2207
NLRP3	-0.006771851	-0.096803271	-0.1913	-0.10414	0.305079	0.616308
NLRP4	-0.029661234	-0.050044424	-0.10584	-0.00054	0.020032	-0.12925
NLRP5	-0.037618004	-0.019914537	0.043483	0.025762	-0.04087	-0.0499
NLRP6	-0.025234395	-0.054309349	-0.07027	0.003303	0.011384	0.019157
NLRP7	-0.006341403	-0.052845519	-0.09961	-0.00526	0.03319	-0.12926
NLRP8	-0.036906881	-0.044470261	-0.01354	-0.00403	-0.01588	0.104899
NLRP9	-0.031349432	-0.037245135	0.003523	0.012908	-0.04341	-0.148
NLRX1	0.007112879	0.006200426	-0.15109	0.025458	-0.10812	0.206763
NMB	0.003004812	0.041574365	0.009845	0.008366	-0.06884	-0.15018
NMBR	-0.027058536	-0.037147379	-0.01028	-0.00279	0.02999	-0.15717
NMD3	0.039197887	0.017395086	0.087551	0.087315	-0.25057	-0.08073
NME1	0.0186452	-0.006553491	0.105306	0.106762	-0.30897	0.023799
NME3	0.026719897	-0.009523543	0.103729	-0.05777	0.165153	0.229073
NME4	0.01109013	-0.146015121	0.021117	-0.06712	0.203983	-0.10926
NME5	-0.041855616	-0.072100205	-0.05043	0.012977	-0.01181	-0.11617
NME6	0.022834446	0.079569902	-0.05088	-0.02009	0.03481	0.085195
NME7	0.011039154	0.020848143	0.121396	0.005541	-0.04519	-0.11231
NME8	0.001102758	0.144613783	-0.24293	-0.046	0.155554	0.165641
NME9	-0.029451259	-0.023510394	0.046424	0.004718	-0.01097	0.01618
NMI	0.06585358	0.038411078	-0.25632	0.054399	-0.12931	0.15361
NMNAT1	-0.012923539	0.019879887	-0.18371	0.012116	-0.02355	0.45179
NMNAT2	-0.051838982	-0.036371317	-0.02174	0.035863	-0.01669	-0.04997
NMNAT3	-0.018679835	-0.143103027	0.060463	-0.06933	0.157811	0.013647
NMRAL1	0.020233615	0.045802061	0.109796	0.045704	-0.11847	0.11282
NMRK1	0.009894751	0.060269035	0.189652	0.015063	-0.03498	-0.19144
NMRK2	-0.033530886	-0.002899896	-0.06218	0.009682	-0.01774	-0.01413
NMT1	0.011531612	0.066575459	0.070459	0.000585	-0.01237	0.052561
NMT2	0.002434931	0.121314019	0.316401	-0.02183	0.07609	-0.27999
NMU	-0.033242317	-0.015350148	0.174201	0.029397	-0.06315	-0.13768
NMUR1	-0.012464239	0.271061708	-0.08806	-0.0156	0.065297	-0.13453
NMUR2	-0.025973902	-0.041475699	0.0086	0.011104	-0.10796	-0.05382
NNAT	-0.032298684	-0.108670779	-0.00353	4.43E-05	-0.0473	0.064732
NNMT	-0.029222813	-0.062496772	0.060629	-0.00641	0.04561	0.001377
NNT	0.003513119	0.034335904	0.063967	-0.01996	0.04965	0.014333
NNT-AS1	0.002567947	0.061757103	0.124793	0.012033	-0.0017	-0.06621
NOA1	0.042071508	-0.070217894	0.137023	0.035242	-0.09676	0.093118
NOB1	0.029091301	0.01708352	0.197392	0.059821	-0.17083	0.008512
NOBOX	-0.029279276	-0.04790887	-0.02349	-0.01476	-0.01097	-0.05148
NOC2L	-0.001009739	-0.003047499	0.064073	0.040008	-0.11067	0.156809
NOC3L	0.013667221	0.017550048	0.026366	0.037852	-0.1334	-0.17054
NOC4L	0.008298197	-0.064737063	-0.00697	0.052803	-0.19577	0.033315
NOD1	0.007783681	0.00425425	-0.0133	-0.05497	0.082611	-0.16356
NOD2	-0.000114365	-0.095997711	-0.35507	-0.08014	0.222812	0.521236
NODAL	-0.034723455	-0.069944653	-0.08042	0.009316	-0.0692	-0.06854

NOG	-0.004499922	-0.130521957	0.486605	-0.10854	0.319813	-0.21572
NOL10	0.007739522	0.048453528	0.030066	0.072909	-0.22392	-0.00034
NOL11	0.032233425	0.073561262	0.245122	0.045073	-0.13021	-0.19857
NOL12	0.020355202	-0.062703965	-0.10603	-0.00652	0.021156	0.376254
NOL3	0.010743167	-0.062862707	-0.10867	0.050129	-0.12483	0.274722
NOL4	-0.026020518	-0.035431758	0.007226	0.078009	-0.14017	-0.05169
NOL4L	-0.023642108	0.040950696	0.054244	-0.04533	0.037684	-0.0047
NOL6	-0.016215951	-0.026463738	0.083436	0.045161	-0.12817	-0.10211
NOL7	0.05202826	-0.013675798	0.164985	0.052924	-0.1499	-0.02511
NOL8	0.036317895	0.065823963	0.185221	0.013905	-0.03211	-0.11116
NOL9	-0.006079069	-0.052692907	0.213433	-0.03672	-0.07341	-0.21266
NOLC1	-0.001766931	0.036745234	0.075194	0.031887	-0.14744	-0.09899
NOM1	0.007215835	0.035765604	0.025413	0.039799	-0.10769	0.10437
NOMO3	0.017226273	0.016399155	-0.00908	0.002149	-0.06537	-0.1695
NONO	0.053622899	0.101948814	0.165836	0.061284	-0.18281	-0.13444
NOP10	0.068474594	0.048018839	-0.26872	0.059585	-0.13725	0.152463
NOP14	0.025824334	0.00682827	0.199632	0.035921	-0.11197	-0.04959
NOP14-AS:	-0.00578778	0.043543832	0.102748	0.013668	0.055989	-0.07167
NOP16	-0.006045563	-0.066694806	0.027291	0.057888	-0.13884	0.109831
NOP2	0.031854545	0.000799647	0.150012	0.03407	-0.10352	-0.13184
NOP58	0.035483502	0.044553763	0.252187	0.056099	-0.14978	-0.07547
NOP9	0.003929286	-0.040279118	0.030308	0.047586	-0.1807	0.128713
NOS1	-0.069109896	-0.040497565	0.047981	0.020678	-0.08843	-0.11804
NOS1AP	-0.044288832	-0.042055534	0.012793	0.000149	-0.05437	-0.0927
NOS2	-0.035634187	-0.097230735	-0.13644	0.008985	-0.04061	0.00663
NOS3	-0.034776577	-0.047573264	0.005657	0.000857	0.024354	0.039746
NOSIP	0.028173593	-0.038755876	0.493623	-0.01744	0.037491	-0.26435
NOSTRIN	-0.041665842	-0.015087397	-0.00538	-0.02594	0.10092	0.076476
NOTCH1	0.014354287	0.110525155	-0.24088	0.015545	-0.05344	-0.07268
NOTCH2	0.041898426	-0.064826387	-0.47754	0.008438	-0.01476	0.288238
NOTCH2NL	0.054044036	-0.049461559	-0.18286	-0.0021	0.033818	-0.04826
NOTCH3	-0.039218985	-0.08993056	-0.08556	0.00387	-0.04907	-0.10863
NOTCH4	-0.011243152	-0.103609921	-0.11375	-0.02114	0.046031	-0.01565
NOTUM	-0.009018876	-0.095252999	-0.03581	-0.01186	-0.08128	-0.09791
NOV	-0.016436919	-0.075076213	-0.16284	-0.01475	0.003933	-0.11542
NOVA1	-0.060060547	0.066434568	0.007509	-0.02556	0.034313	-0.0839
NOVA2	-0.035230445	-0.087071053	-0.05038	-0.00088	-0.06913	-0.00603
NOX1	-0.036142504	-0.067185892	-0.11237	0.082509	-0.09323	0.040826
NOX3	-0.054726502	-0.067791289	0.010394	0.035047	-0.10234	-0.0576
NOX4	-0.030728342	-0.076526735	0.052066	0.013509	-0.0662	-0.03431
NOX5	-0.040321124	-0.048391623	-0.07841	0.024718	-0.12658	-0.04153
NOXA1	-0.012259107	-0.104576088	0.019232	-0.02073	0.00731	0.096653
NOXO1	-0.025925267	-0.088567429	0.016523	0.017639	-0.05756	0.064867
NOXRED1	-0.030197668	-0.035834694	0.006862	0.000563	0.021204	-0.00444
NPAP1	-0.033658506	-0.100951318	-0.0441	0.011404	-0.02182	0.079024
NPAS1	-0.028764097	-0.074530581	-0.02376	0.06185	-0.15705	0.070283
NPAS2	-0.044838887	-0.092021097	0.19887	-0.00554	-0.01684	-0.05128
NPAS3	0.033911451	-0.096162513	0.035433	0.101774	0.052135	-0.01083
NPAS4	-0.050142472	-0.07462021	0.001799	0.028563	-0.07489	0.174457
NPAT	2.75E-05	0.092763867	0.375348	-0.03729	0.063815	-0.26396
NPB	0.000510254	-0.064254372	-0.07212	-0.01849	-0.0401	-0.01403

NPBWR1	-0.030018186	-0.066006944	-0.119	0.025636	-0.04885	0.230377
NPBWR2	-0.050455057	-0.014272321	-0.03961	-0.00522	0.02531	0.099634
NPC1	0.000206619	0.18284533	0.044636	-0.0055	0.061137	-0.14902
NPC1L1	-0.064947999	-0.175752678	0.094264	0.015003	-0.11259	0.008602
NPC2	0.042550037	-0.115491313	-0.39305	-0.02286	0.075274	0.449126
NPCDR1	-0.017820798	-0.086212507	0.071336	0.017987	-0.04244	-0.08553
NPDC1	-0.005625789	-0.138373624	0.275843	0.08351	-0.26977	-0.11266
NPEPL1	0.015306394	-0.055830996	0.022369	-0.08632	0.212489	0.167083
NPEPPS	0.031619269	0.045561886	-0.29263	0.066194	-0.11289	-0.05781
NPFF	-0.012570286	-0.011954676	-0.11173	-0.04094	0.083152	0.10881
NPFFR1	-0.010483703	-0.036531783	-0.00473	0.003943	-0.06193	-0.10385
NPFFR2	-0.041855568	-0.007380346	0.061901	0.016873	-0.02814	-0.01752
NPHP1	-0.026332304	-0.083604119	0.221347	0.007827	-0.20925	-0.16074
NPHP3	0.016325542	-0.007949562	0.096318	-0.0923	0.294077	-0.06744
NPHP3-AS	-0.027621376	-0.039602181	-0.00117	0.062148	-0.16995	-0.08597
NPHP4	-0.012089561	-0.088653455	-0.09134	0.044585	-0.13623	0.189353
NPHS1	-0.037809768	-0.023849114	-0.12715	0.030714	-0.05647	0.140527
NPHS2	-0.028163162	-0.027466434	-0.01405	0.026748	-0.09566	-0.08179
NPIPA1	0.060914426	0.089031754	0.312197	0.01591	-0.01175	-0.34359
NPIPB15	0.013992982	-0.050276988	0.074992	-0.07062	0.140405	-0.06315
NPL	-0.003842353	-0.066587576	-0.28085	-0.04786	0.144359	0.506057
NPLOC4	0.067148098	-0.011084471	-0.14868	0.059525	-0.17456	0.301129
NPM1	0.043830546	0.075374634	0.285726	0.078943	-0.16971	-0.23454
NPM2	-0.009497475	-0.048591927	-0.00572	-0.01194	-0.08956	-0.06621
NPM3	-0.005198194	-0.039935392	0.197096	0.072507	-0.22167	-0.13131
NPNT	-0.036143666	-0.047065227	0.02766	-0.01785	0.002084	-0.05498
NPPA	-0.019932622	-0.092238418	0.087297	-0.01236	-0.04705	-0.05298
NPPB	-0.031833061	-0.029294785	-0.11505	0.007664	-0.02275	0.008698
NPPC	-0.034091238	-0.093782099	-0.1138	-0.01705	0.075413	0.073559
NPR1	-0.026831044	-0.108129041	-0.01692	-0.00967	-0.07607	-0.06923
NPR2	-0.02236864	-0.044671825	0.26372	0.00552	0.000387	-0.34835
NPR3	-0.065973993	-0.02207273	0.196798	0.012396	-0.07212	-0.05494
NPRL2	0.019303358	0.174444765	0.250493	0.008737	-0.0382	-0.27666
NPRL3	-0.016826082	0.078641651	0.004675	0.104562	-0.22172	-0.0362
NPSA	-0.051254193	-0.041903573	-0.07196	-0.00977	0.036234	-0.01778
NPSR1-AS	-0.046024703	-0.055073026	-0.02605	0.000102	0.024219	0.069558
NPTN	0.064376477	0.050006542	-0.32126	-0.01098	0.029234	0.358012
NPTN-IT1	-0.004952232	-0.052428237	-0.13221	0.003456	-0.0116	0.090131
NPTX1	-0.017086061	0.008680205	0.024903	-0.02011	0.010981	-0.03832
NPTX2	-0.021298777	0.018817364	-0.00032	-0.00885	0.041149	-0.03517
NPTXR	-0.028623965	-0.018723487	0.090607	-0.00026	-0.04722	-0.08264
NPVF	-0.054542417	0.038987897	-0.08048	-0.02267	0.061972	-0.00652
NPW	-0.035059469	-0.045761386	0.077616	-0.02114	0.0664	0.008558
NPY	-0.029815429	-0.06211643	-0.0736	0.008342	-0.00328	0.012597
NPY1R	-0.06311751	-0.084804589	0.046182	-0.04054	0.128089	-0.10402
NPY2R	-0.022653645	-0.025425985	-0.07798	0.037596	0.007632	0.065757
NPY5R	-0.044969085	-0.053098916	-0.01265	0.009724	0.001097	0.012431
NPY6R	-0.049177085	-0.051990379	-0.01068	0.00796	-0.02283	0.054519
NQO1	-0.000597513	0.081191247	-0.02492	0.083381	-0.33999	-0.00266
NQO2	0.017291242	-0.016287104	-0.19443	-0.00517	0.019366	0.365196
NR0B1	-0.060327731	-0.041526044	-0.17518	-0.01405	0.058864	0.015033

NR0B2	-0.023495599	-0.069620913	-0.03564	-0.00975	-0.07177	-0.12832
NR1D2	0.017174805	0.052123361	0.208899	-0.0118	0.069542	-0.05394
NR1H2	0.008037516	-0.08762362	-0.17537	0.010135	-0.02422	0.199736
NR1H3	-0.005929523	0.149770708	-0.04995	0.014433	-0.01616	0.314705
NR1H4	-0.059326732	-0.100114157	0.087703	-0.0389	0.008939	-0.14576
NR1I2	-0.046364683	-0.081411827	0.052095	0.009504	-0.04226	-0.07375
NR1I3	-0.049895193	-0.111891	-0.0171	0.002675	0.028722	0.107477
NR2C1	-0.01784854	0.029596024	0.138096	0.008299	0.06253	-0.1253
NR2C2	0.022847635	0.129564787	0.081107	0.025883	-0.05696	-0.24867
NR2C2AP	0.015035443	-0.010635385	0.264201	0.03522	-0.16024	-0.24804
NR2E1	-0.040450305	-0.061364849	0.010502	-0.0026	0.02934	0.072395
NR2E3	-0.043654247	-0.01763968	-0.08852	########	0.025223	0.017803
NR2F1	-0.048705885	-0.061893461	-0.04956	0.012329	-0.02923	0.016758
NR2F1-AS	-0.142631944	-0.094483965	-0.02686	-0.05326	0.031687	-0.15523
NR2F2	-0.062362796	-0.034990429	0.147032	0.007715	0.00894	-0.04028
NR2F2-AS	-0.02068783	-0.036036474	0.12622	0.009392	-0.03448	-0.11107
NR2F6	-0.028410132	-0.128509113	-0.0359	0.092164	-0.2144	0.135815
NR3C1	0.047706957	0.233955213	-0.25376	0.048887	-0.07994	-0.10327
NR3C2	-0.004409033	-0.110967704	0.506762	-0.04196	0.134517	-0.28549
NR4A1	-0.018460734	-0.08651614	-0.03575	-0.006	-0.06016	-0.05038
NR4A2	0.005459298	0.016380634	-0.01127	0.003339	-0.01791	0.016927
NR4A3	-0.010657422	-0.000112288	-0.05033	0.004352	-0.06366	-0.03717
NR5A1	-0.025795232	-0.024521884	-0.06024	0.009468	0.00389	-0.01242
NR5A2	-0.062459938	-0.046964721	-0.006	0.169223	-0.18464	-0.01466
NR6A1	-0.033327627	-0.067615467	-0.06875	0.062265	-0.07686	0.026723
NRAP	0.006107215	-0.071602836	-0.17879	0.042235	-0.04975	0.037584
NRARP	-0.002216135	-0.026499323	0.046234	0.008606	-0.06705	-0.05417
NRAS	0.045945141	0.09874398	-0.13058	0.080599	-0.24631	0.165451
NRBF2	0.030112784	0.001475468	-0.32228	0.003811	0.001707	0.119387
NRBP1	0.035366029	0.058173275	-0.09023	0.006671	-0.04699	-0.00769
NRCAM	-0.014386792	-0.100848736	0.553673	0.039282	-0.06923	-0.13314
NRD1	0.036266762	-0.014845647	-0.22758	0.010278	0.007308	-0.05817
NRDE2	-0.020068315	0.000229397	-0.07755	0.008254	0.023101	0.084009
NREP	0.003940478	-0.028467271	-0.11587	-0.07424	0.240058	0.0109
NRF1	-0.032414554	-0.020874205	0.042532	0.070337	-0.02285	-0.19099
NRG1	-0.022596295	-0.113492199	-0.17127	-0.11717	0.26606	0.759318
NRG1-IT1	-0.040953211	-0.060589752	-0.04693	0.023939	-0.04422	-0.00113
NRG2	-0.045716655	0.037580647	-0.00191	-0.02207	0.046305	0.003372
NRG3	-0.050923984	-0.042085782	-0.11529	-0.034	0.110782	0.130497
NRG4	-0.064763188	-0.041533673	-0.01397	-0.02842	0.078123	0.127682
NRGN	0.000989454	-0.059526027	-0.1211	-0.18638	0.513639	0.368341
NRIP1	0.013258634	0.033177611	0.021878	-0.12603	0.35459	0.14285
NRIP2	-0.028485393	-0.043608952	0.054646	0.011476	-0.02546	0.114727
NRIP3	-0.002792336	-0.024304934	0.006297	-0.0003	-0.02763	-0.05738
NRK	-0.074095517	-0.109746892	0.000283	0.001501	0.000992	-0.20156
NRL	-0.039621846	-0.043526759	-0.01968	0.026327	-0.07127	-0.06506
NRM	0.02576957	-0.096941471	-0.27947	0.022681	-0.10007	0.296104
NRN1	-0.02978313	-0.032018835	0.121988	0.00195	0.019087	0.071002
NRP1	0.000939149	-0.037551372	-0.07891	0.014256	0.032181	-0.00981
NRP2	-0.021182037	-0.02556823	-0.05474	-0.00192	-0.01364	0.032026
NRROS	0.015277961	0.022343865	-0.2164	-0.00214	-0.00179	0.24872

NRSN1	-0.02863518	-0.036806053	0.041464	0.007894	-0.01458	0.047824
NRSN2	-0.026612026	-0.042183781	-0.06906	0.029032	-0.10761	-0.0977
NRTN	-0.032403658	-0.051044718	0.060454	-0.02904	0.068487	-0.03859
NRXN1	-0.025617225	-0.046372595	0.025215	0.062801	-0.16542	0.028877
NRXN2	-0.02811241	0.149101696	-0.1219	0.044291	-0.092	0.07744
NRXN3	-0.043656464	-0.033446259	0.051091	0.105611	-0.22737	0.053256
NS3BP	-0.031124014	0.009379599	-0.01288	-0.00897	-0.04025	0.055256
NSA2	0.046318304	0.03483662	0.250754	0.018054	-0.02719	0.0251
NSAP11	-0.040655074	-0.072287488	-0.07073	0.011235	-0.00758	0.022273
NSD1	-0.083196044	-0.083456748	-0.09009	-0.01059	-0.13076	0.060981
NSDHL	0.001801894	0.073063209	-0.16585	0.061389	-0.16372	0.275798
NSF	0.002027039	-0.010500175	0.046935	-0.02377	0.001398	-0.14611
NSFL1C	0.015090359	-0.111364132	-0.31959	0.003879	-0.02996	0.136483
NSG1	-0.019698761	0.021301424	0.51764	-0.09018	0.207115	-0.21745
NSL1	0.006301158	-0.080737934	0.004922	0.035824	-0.07902	0.029393
NSMAF	-0.002518096	-0.065952079	-0.04079	-0.00521	0.037123	-0.16024
NSMCE1	0.039252515	-0.009332988	0.235865	0.028929	-0.08299	-0.06383
NSMCE2	0.042620876	0.043759115	0.209161	0.064536	-0.20159	0.066638
NSMCE4A	0.012684824	0.044147536	0.157776	-0.00804	0.06044	-0.05612
NSMF	-0.023673575	-0.114184528	-0.0209	0.006263	-0.11186	-0.05682
NSRP1	0.045125569	0.044430549	0.001873	0.048658	-0.11465	0.015158
NSUN2	0.050303808	0.072116265	0.075997	0.076453	-0.2266	-0.10138
NSUN3	-0.046265528	0.049288296	-0.05835	0.008679	-0.06399	0.090464
NSUN4	0.033319382	-0.052597508	-0.07271	0.065368	-0.10957	0.09668
NSUN5	0.051600184	-0.020754897	0.261501	0.024142	-0.08152	-0.37496
NSUN5P1	0.035095981	-0.012905246	0.293198	-0.00104	-0.00496	-0.38782
NSUN6	0.043080178	0.079587246	0.380169	-0.06335	0.100358	-0.31631
NSUN7	-0.027847994	-0.059241071	-0.15934	0.00608	-0.02275	-0.0991
NT5C	0.009326099	0.067939373	-0.03979	0.030725	-0.12152	-0.08251
NT5C1A	-0.034571511	-0.062023546	-0.10933	0.004487	-0.03895	0.040227
NT5C2	0.06633162	0.055487916	-0.35488	-0.02945	0.091667	0.080387
NT5C3A	0.05553818	0.03902768	-0.0378	-0.06034	0.17386	-0.23218
NT5C3B	0.016967238	-0.102562167	0.189073	0.014058	-0.03774	-0.00919
NT5DC1	0.012169443	-0.020552931	0.077251	0.006394	-0.01668	-0.04584
NT5DC2	-0.042795057	-0.049359111	-0.01308	0.024549	-0.17457	0.068726
NT5DC3	-0.021722895	-0.05909271	-0.03342	0.00905	-0.01941	-0.12932
NT5DC4	-0.003157821	-0.023540183	-0.06572	0.180682	-0.12957	0.051174
NT5E	-0.001731179	-0.095556558	0.34453	-0.05409	0.189354	-0.24118
NT5M	-0.017835658	-0.053527984	0.024389	-0.06521	0.178217	0.03163
NTAN1	0.02256141	-0.01320808	0.003539	-0.00079	0.003727	-0.01744
NTF3	-0.028083535	-0.081392769	0.050247	0.015962	-0.08831	-0.02336
NTHL1	-0.00374342	0.03617571	0.010743	0.086843	-0.20973	0.109669
NTM	-0.03339153	-0.045531035	-0.00716	0.012516	-0.08904	-0.04504
NTMT1	0.016588877	-0.083888509	-0.04829	0.071634	-0.21314	0.309619
NTN1	-0.052412841	-0.021035832	-0.03651	-0.00764	0.059108	0.119999
NTN3	-0.030591273	-0.06431347	-0.08824	0.025736	-0.03915	0.086462
NTN4	-0.037732241	-0.039534607	0.2499	-0.06153	0.141347	-0.12091
NTN5	-0.045880789	-0.084709007	0.203683	0.024862	-0.0578	-0.04277
NTNG1	-0.046768097	-0.076698393	0.046262	0.02466	-0.05036	-0.16009
NTNG2	-0.015423954	-0.008870798	-0.07798	-0.01828	0.01007	0.057853
NTPCR	-0.007061036	-0.019297657	-0.00369	0.113641	-0.17683	0.118025

NTRK1	-0.016221537	0.000897961	-0.03354	0.01457	-0.0364	-0.00348
NTRK2	-0.014316749	-0.019888378	0.031672	0.021134	-0.1304	-0.03516
NTRK3	-0.005967389	-0.030601594	0.041434	0.01943	-0.04808	-0.04798
NTRK3-AS:	-0.034131488	-0.140893648	-0.04114	0.019663	-0.07365	-0.1441
NTS	-0.031079123	-0.040880782	0.024117	0.05514	-0.12948	0.060398
NTSR1	-0.024146637	-0.063424817	-0.29556	-0.00678	0.012977	0.462724
NTSR2	-0.03756319	-0.029069384	0.045241	-0.01984	0.083913	-0.07133
NUAK1	-0.007497479	0.126408403	0.140116	0.033956	-0.07971	-0.11726
NUB1	-0.006078271	0.024348441	0.035337	-0.03335	0.118432	-0.16007
NUBP1	0.034697843	-0.015308288	-0.21606	0.006318	-0.0389	0.487125
NUBP2	0.035996697	-0.023691066	0.213205	0.047348	-0.17479	-0.12765
NUBPL	-0.005511788	-0.000409212	0.17228	0.019345	-0.0362	-0.12565
NUCB1	0.021682623	-0.007212143	-0.08353	0.024476	-0.08066	0.253809
NUCB2	0.013168069	0.032720169	0.423084	0.033255	-0.08428	-0.19023
NUCKS1	0.02455964	0.143304168	0.263352	0.091412	-0.26435	-0.24352
NUDC	0.043793375	0.069954191	0.086006	0.111421	-0.26838	-0.02242
NUDCD1	0.001289006	0.015996044	0.035401	0.068035	-0.20772	-0.03818
NUDCD2	0.005610967	-0.066439961	0.085325	0.03109	-0.11507	0.028434
NUDCD3	-0.032780417	-0.127658589	0.070476	0.012106	-0.03655	0.113575
NUDT1	0.019633816	-0.005804799	-0.01961	0.045994	-0.13209	0.162918
NUDT10	-0.050602679	-0.04370177	0.008162	-0.05158	0.099703	-0.07692
NUDT11	-0.015197403	0.164483439	0.027004	-0.00266	-0.01392	-0.22274
NUDT12	-0.039613398	-0.114263438	-0.03352	-0.04896	0.134954	-0.04446
NUDT13	-0.012475363	-0.020694245	-0.00579	-0.06854	0.209985	0.154496
NUDT14	-0.017710687	-0.027468544	-0.14041	0.028819	-0.10826	0.299082
NUDT15	0.011535539	-0.020589211	-0.06135	0.130464	-0.34634	0.259351
NUDT16	-0.000481429	-0.076626604	-0.2466	-0.08724	0.237937	0.595751
NUDT16L1	0.032902692	-0.056034432	0.156764	0.025044	-0.09487	-0.1558
NUDT16P1	-0.015215885	-0.106803649	-0.30082	-0.07914	0.225773	0.51136
NUDT17	-0.004695833	-0.103333798	-0.07442	-0.01288	0.019998	-0.17409
NUDT18	0.009224276	-0.017062291	-0.03649	0.033982	-0.11303	0.10045
NUDT19	0.028946445	0.057733208	0.12341	0.044398	-0.11714	0.307475
NUDT2	0.022637287	-0.030461307	0.144664	0.048873	-0.16089	0.089772
NUDT21	0.115339895	0.13227762	0.074168	0.041109	-0.36763	0.089844
NUDT22	0.022034587	-0.053848202	-0.00416	-0.00595	0.010535	0.137659
NUDT3	0.028444545	-0.038986426	-0.1626	-0.03489	0.137907	0.18842
NUDT4	-0.026290193	-0.061886148	-0.09552	0.016893	0.031226	-0.031
NUDT5	0.043631304	0.037509502	0.129034	0.043207	-0.11275	-0.37209
NUDT6	-0.006283596	0.028106351	0.022852	0.125524	-0.33871	0.080871
NUDT7	-0.007336863	-0.032757823	0.114534	-0.0473	0.139835	0.172362
NUDT9	0.035498709	0.047039308	0.139646	0.031793	-0.07694	0.033431
NUDT9P1	-0.028650047	-0.01666716	0.189225	0.012391	-0.01338	-0.16609
NUF2	-0.009472302	0.07798712	0.042573	0.145204	-0.27327	-0.05311
NUFIP1	0.011567607	0.013862986	0.060958	0.075679	-0.19318	-0.00297
NUFIP2	0.050495856	0.021285693	-0.03349	0.052093	-0.15718	-0.06051
NUMA1	-0.035678484	0.067773234	0.11603	-0.03648	0.108227	-0.10321
NUMB	-0.008596771	-0.103107781	-0.35303	-0.01983	0.057032	0.228081
NUMBL	0.050774817	0.086928808	-0.00839	0.039028	-0.07787	-0.25174
NUP107	0.038571839	0.115712984	0.118552	0.081197	-0.22994	-0.22492
NUP133	0.019953413	0.131758753	-0.04062	0.105022	-0.27273	0.135304
NUP153	0.031780881	0.152668223	0.002002	0.085189	-0.21408	-0.25474

NUP155	0.011410523	0.043600968	0.065654	0.128571	-0.32666	-0.00946
NUP160	0.004890237	-0.034931405	-0.02256	0.023834	-0.0344	-0.03145
NUP188	-0.044628081	-0.126373133	0.112601	0.037549	-0.10788	-0.08208
NUP205	0.015844117	0.057021809	0.211309	0.079207	-0.22317	-0.23096
NUP210	0.01264382	0.007913709	0.23401	0.043322	-0.03243	-0.21472
NUP210L	-0.043970149	-0.014034269	0.042941	-0.01711	0.074959	0.048481
NUP210P1	-0.037120937	-0.006791441	-0.1714	0.002249	0.000185	0.071004
NUP214	-0.081335542	0.040049611	-0.15927	-0.0643	-0.09371	0.343163
NUP35	0.016419053	0.068438252	0.093712	0.082078	-0.22881	-0.05606
NUP37	0.019271072	0.03352588	0.134927	0.092021	-0.25839	0.067754
NUP43	0.016058244	0.074715953	0.081037	0.038018	-0.08087	0.06335
NUP50	0.020483884	-0.005627977	-0.1853	0.025255	-0.09012	0.11034
NUP50-AS1	-0.014432186	-0.025108168	-0.15329	-0.00414	-0.04198	0.362132
NUP54	-0.033944876	-0.011249054	0.103993	0.018619	-0.07792	0.002977
NUP62	0.012058938	0.018262912	-0.09187	0.023176	-0.06641	0.296079
NUP62CL	-0.02324717	-0.055827128	0.044134	0.039762	-0.09025	-0.08058
NUP85	0.045040085	0.050132	0.022958	0.063076	-0.18295	0.105217
NUP88	0.018121695	0.031724387	0.167223	0.014572	-0.0388	-0.18603
NUP93	0.022404301	-0.032732927	-0.11032	0.079575	-0.20205	0.064917
NUP98	0.074325591	-0.016960753	-0.05221	0.037765	-0.10655	-0.06059
NUPL1	0.06542367	0.062762919	-0.10583	0.097304	-0.21983	0.153628
NUPL2	0.017119443	0.034804451	-0.15051	0.019174	-0.07371	0.120308
NUPR1	-0.019898854	-0.078721008	-0.04959	0.005739	-0.0344	-0.07165
NUS1	0.036853279	0.033234093	0.027937	0.023509	-0.08055	0.026492
NUS1P3	-0.013856664	0.028456767	0.000501	0.031722	-0.06539	0.019753
NUSAP1	0.007008584	-0.002279154	-0.05956	0.142201	-0.38996	-0.03444
NUTM1	-0.024322564	-0.076576132	-0.0196	-0.00587	-0.07346	-0.10333
NUTM2A-AS	-0.020032992	-0.028283226	-0.09744	0.016832	-0.04793	0.019825
NUTM2B	-0.022788329	-0.097497528	-0.00884	-0.02578	-0.0034	-0.00644
NVL	0.029259214	0.03134677	0.189689	0.034274	-0.10583	-0.10332
NWD2	-0.063255502	-0.015206091	-0.08045	-0.00707	0.013275	0.116457
NXF1	0.090373376	0.001649245	-0.08909	-0.03907	0.155382	0.053125
NXF3	-0.031816533	-0.031818933	-0.04237	-0.13788	0.395802	-0.00595
NXF5	-0.027959858	-0.06421905	-0.1172	-0.03675	0.114418	0.000372
NXN	-0.026122689	-0.031621347	0.012484	-0.00684	0.042227	-0.07687
NXNL1	-0.009185893	-0.042698196	0.040317	-0.00845	-0.06955	-0.10673
NXNL2	-0.047862209	-0.022077487	0.069712	-0.00238	0.007236	0.000285
NXPE1	-0.050615573	-0.089431411	0.055526	0.008524	0.00028	-0.1783
NXPE3	0.026791615	-0.010147803	0.122032	-0.02211	0.113616	0.222839
NXPE4	-0.029398799	-0.033653597	0.022976	0.022425	-0.05945	-0.07714
NXPH1	-0.081555707	-0.049802502	-0.07516	0.000128	-0.00511	-0.06131
NXPH2	-0.011750666	-0.012809601	-0.05029	0.051806	-0.07778	-0.03038
NXPH3	-0.017478112	-0.076754517	-0.03871	-0.01706	-0.06016	-0.10992
NXPH4	-0.025802248	-0.033951151	-0.01606	-0.01777	0.036948	0.0419
NXT1	0.033636724	-0.007640853	0.130872	0.073166	-0.21506	-0.18262
NXT2	0.014429864	0.145540215	-0.0218	0.047415	-0.14619	0.048736
NYAP1	-0.04392673	-0.03887456	0.024282	-0.00446	0.027428	-0.09646
NYAP2	-0.033513723	-0.061558793	0.070209	0.011905	-0.0135	-0.18552
NYNRIN	-0.033632262	-0.053035673	-0.0002	-0.00778	0.044696	-0.04434
NYX	-0.026007551	-0.077023622	0.046739	0.039839	-0.15761	-0.02389
OAF	-0.01432505	-0.12579266	-0.01807	-0.06549	0.121837	0.233278

OARD1	0.028330566	0.025392546	0.150626	0.020369	-0.06631	-0.19246
OAS1	0.013803419	-0.023396204	-0.27865	-0.06443	0.185634	0.386869
OAS2	-0.00509913	0.012608228	0.024901	-0.12239	0.166958	0.0796
OAS3	0.00980477	-0.023791756	-0.02838	-0.05379	0.133294	0.242454
OASL	0.014824836	0.063421413	-0.01862	0.035002	-0.10806	0.019418
OAT	0.051726866	0.076124327	-0.02701	-0.06737	0.168087	0.14042
OAZ1	0.061167259	-0.011519883	-0.35321	0.053156	-0.05501	0.326699
OAZ2	0.032631656	-0.093451904	-0.35185	-0.00333	0.021295	0.282279
OAZ3	-0.030366403	-0.028959578	0.028803	0.046459	-0.11163	0.144238
OBFC1	0.011326221	0.04828366	0.296701	-0.02366	0.070527	-0.18134
OBP2A	-0.035282532	-0.104487092	-0.05288	-0.01297	-0.09813	-0.03032
OBP2B	-0.033055472	-0.089983712	-0.01404	0.033294	-0.08088	-0.02323
OBSCN	-0.013754452	-0.20212717	0.289919	-0.03715	0.146371	-0.24541
OBSL1	4.12E-05	-0.066961207	-0.13879	0.022888	-0.0019	-0.20309
OCA2	-0.032306242	0.031417049	0.027529	0.009758	-0.07522	-0.09203
OCEL1	0.019898382	-0.001847356	-0.03491	-0.01398	0.01413	0.2459
OCIAD1	0.102100253	0.109643287	0.167523	0.046917	-0.06887	-0.25222
OCIAD2	0.014863406	0.022687104	0.336669	-0.02291	0.030662	-0.53685
OCLM	-0.0335638	-0.066974337	0.016532	0.027497	-0.10081	-0.17887
OCLN	-0.001068701	-0.011560371	0.072347	-0.046	0.125975	-0.03929
OCM2	-0.021785897	-0.097755296	0.183066	-0.00675	0.04321	0.001028
OCR1	-0.04004352	-0.038750308	-0.13031	-0.00254	0.031749	-0.04268
OCRL	-0.021873888	-0.189978326	-0.16964	-0.08252	0.196411	0.346051
ODAM	-0.046361248	-0.039517884	0.114843	0.018167	-0.02495	-0.10005
ODC1	0.030686878	-0.030872632	0.080975	0.022412	-0.06395	-0.20842
ODF1	-0.026089597	-0.028953777	-0.03254	0.000123	-0.0268	0.015716
ODF2	0.013908116	0.048825784	0.050914	0.074105	-0.2247	0.014791
ODF2L	-0.007435683	0.063413864	0.170053	-0.04517	0.114581	-0.26397
ODF3	-0.032675513	-0.085167078	-0.08389	0.000718	-0.00878	0.113103
ODF3B	0.001783038	-0.112947756	-0.22456	-0.03166	0.059884	0.329419
ODF3L1	-0.039648746	-0.047547553	-0.03535	0.015692	-0.02328	0.040308
ODF3L2	-0.022102723	-0.050208889	0.046284	-0.00155	-0.07233	-0.02217
ODF4	-0.044531517	-0.022456285	0.114691	0.016797	-0.1209	-0.09779
OFCC1	-0.099904494	0.011592021	0.00423	-0.00416	-0.15894	-0.06246
OFD1	0.016941248	0.075881321	0.174121	-0.00084	0.021552	-0.53813
OGDH	-0.029289737	-0.074825046	-0.06872	-0.03202	-0.01573	0.145775
OGDHL	-0.031056889	-0.047381292	-0.03199	0.0205	-0.06366	-0.08736
OGFOD1	0.01375941	0.034253034	0.199186	0.032613	-0.11073	-0.23933
OGFOD2	-0.005911928	-0.03155832	-0.07563	0.014888	-0.09228	0.250926
OGFOD3	0.074061187	0.094580183	-0.04541	0.100188	-0.06816	0.084714
OGFR	0.030798623	-0.059814644	-0.08239	-0.01633	-0.00305	0.224419
OGFRL1	0.027889902	-0.054398525	-0.28708	-0.06367	0.209973	0.326929
OGFRP1	-0.017804103	-0.054979028	0.018736	-0.01306	-0.08116	-0.1196
OGG1	0.005888079	0.06169262	-0.12155	0.02513	-0.08999	0.223001
OGN	-0.069442247	-0.079097018	0.195795	-0.01603	0.044845	-0.06827
OGT	0.041427315	0.077013798	0.127357	-0.00881	-0.01399	-0.29284
OIP5	-0.010298145	-0.004419863	0.024952	0.132725	-0.29091	-0.08696
OIP5-AS1	0.018271551	0.024250342	0.089313	0.037441	-0.14261	-0.09108
OIT3	-0.051957091	-0.101389062	0.04673	0.024978	-0.06403	-0.10465
OK/SW-CL.	-0.030685517	-0.0040984	0.056172	0.012648	-0.02405	-0.06459
OK/SW-CL.	-0.01374652	-0.041035	0.078597	0.006891	-0.01526	-0.0219

OLA1	0.023930359	0.047201452	0.142942	0.038812	-0.08051	0.166778
OLAH	-0.040336172	-0.027186146	0.00063	0.236556	-0.23914	-0.0379
OLFM1	-0.047171634	-0.137188778	-0.1203	-0.07506	0.270789	0.61282
OLFM2	-0.031724837	-0.031176885	0.124734	-0.00857	-0.01142	-0.07029
OLFM3	-0.046488704	-0.093092542	0.023292	-0.0183	0.021277	-0.04379
OLFM4	-0.007626145	-0.035658303	0.369051	0.03699	-0.06294	-0.10784
OLFML1	-0.042079672	-0.006117474	-0.04819	-0.01214	0.042375	0.026158
OLFML2A	-0.018602474	-0.072554633	-0.02032	-0.02004	0.039098	0.020826
OLFML2B	-0.0305196	-0.061644833	-0.21837	-0.01917	0.071959	0.299942
OLFML3	-0.031050032	-0.020725818	0.026706	-0.00387	0.006791	0.014154
OLIG1	0.002362202	-0.078076294	-0.34594	-0.07937	0.249119	0.520558
OLIG2	-0.018203228	-0.076134412	-0.09513	-0.0059	0.013235	-0.07518
OLIG3	-0.057560084	-0.00643369	-0.01428	0.000503	-0.00262	0.100166
OLR1	-0.017296065	-0.026761318	0.097204	0.000939	0.00021	-0.06346
OMA1	0.015502836	0.009053658	0.046922	0.013327	-0.03626	-0.05187
OMD	-0.069521623	-0.036733921	0.115955	0.024577	-0.05903	-0.07846
OMP	-0.032301062	-0.040688217	-0.13358	0.027135	-0.04813	0.217889
ONECUT1	-0.040498954	-0.051733257	-0.02397	0.030706	-0.07595	-0.06874
ONECUT2	-0.049676417	-0.039211719	0.004534	0.012572	-0.06282	0.04438
ONECUT3	-0.030724667	-0.102769129	-0.11188	0.027689	-0.08736	0.002843
OOEP	-0.030023908	-0.046013812	-0.0178	0.012966	-0.04953	-0.09912
OOSP2	-0.058355137	-0.069029293	0.031203	-0.01389	0.022286	-0.04396
OPA1	-0.003988794	0.030174906	0.009695	0.084754	-0.19857	0.001778
OPA3	-0.022269272	0.067733938	0.102845	0.013298	-0.05637	0.058243
OPALIN	-0.02666689	-0.05251713	-0.03372	-0.00539	0.019904	-0.0394
OPCML	-0.030765797	-0.050202646	-0.03001	0.017069	-0.04525	-0.17141
OPHN1	0.039262756	0.007084407	0.018293	0.037503	-0.06884	-0.20046
OPLAH	-0.019278631	-0.114853812	-0.13143	-0.00284	0.040167	0.514488
OPN1SW	-0.038419814	-0.016107153	0.072215	0.012587	-0.00725	-0.00223
OPN3	0.015740199	-0.10771598	-0.18123	-0.02932	0.086876	0.129463
OPN4	-0.035365805	-3.17E-05	-0.0974	-0.0188	0.002636	-0.05183
OPN5	-0.041700838	-0.027266805	0.069495	0.012761	-0.03334	-0.0916
OPRD1	-0.029043987	-0.076385453	0.100709	0.012085	-0.02826	0.039885
OPRK1	-0.069398276	-0.09433251	-0.00322	0.01645	-0.02495	0.055313
OPRL1	-0.033711128	-0.137407538	-0.29212	-0.02639	0.079871	0.279661
OPRM1	-0.06292052	-0.017857563	-0.00857	-0.04375	-0.05785	0.043166
OPTC	-0.024721979	0.004594274	-0.04182	0.067561	-0.17137	0.057126
OPTN	0.014409194	0.192952695	0.292703	0.009339	-0.04203	-0.32806
OR10A3	-0.052551025	-0.029650715	-0.17781	-0.00032	0.026126	0.060995
OR10A4	-0.035853981	0.013047709	0.039192	0.003591	0.000819	-0.08523
OR10A5	-0.025522399	-0.047062648	-0.0525	0.01862	-0.0571	-0.03461
OR10C1	-0.038765322	-0.008728076	-0.09496	0.022324	-0.04036	0.020994
OR10D1P	-0.042125815	-0.04997079	-0.06094	0.020022	-0.04429	0.005739
OR10D3	-0.038900429	-0.051330559	0.024469	0.011571	-0.05098	-0.1085
OR10H1	-0.031081761	-0.061055669	-0.01166	-0.00722	-0.02142	-0.00323
OR10H2	-0.041918418	-0.070576711	0.137768	0.018907	-0.04468	0.032213
OR10H3	-0.029858228	-0.040522731	0.030573	0.007577	-0.01763	-0.08885
OR10J1	-0.035946874	-0.056899272	0.069066	-0.00043	-0.01751	-0.05656
OR11A1	-0.045417694	-0.083406565	0.037441	0.000892	0.019546	0.030238
OR12D2	-0.034499524	-0.103269493	0.048841	-0.00829	-0.00301	0.005807
OR12D3	-0.025115013	-0.036684541	0.05826	-0.01292	0.022144	-0.10605

OR13C4	-0.04866504	-0.087889136	-0.00572	-0.01054	-0.01559	-0.1279
OR14J1	-0.036605376	-0.126569702	-0.02398	0.015189	-0.02446	0.119771
OR1A1	-0.026975536	-0.048474372	0.026603	0.015899	-0.04433	0.001146
OR1A2	-0.048359933	-0.062480979	0.068236	-0.00336	0.028137	-0.04611
OR1C1	-0.048470389	-0.060658527	-0.07404	0.030148	-0.0648	0.057896
OR1D2	-0.030982146	-0.000226922	0.010273	0.02758	-0.07078	-0.0269
OR1D5	-0.03723023	-0.035711212	0.083459	0.038329	-0.08024	0.055684
OR1E1	-0.049144081	-0.098346421	-0.03244	-0.00069	0.015757	0.045575
OR1F1	-0.029074384	-0.056013248	-0.02592	0.00281	-0.08458	-0.11931
OR1F2P	-0.037285509	-0.045480119	-0.10933	0.019376	-0.06235	-0.03784
OR1G1	-0.026118995	0.007663938	0.106599	0.018733	-0.06631	-0.06781
OR1I1	-0.019782003	-0.057881582	-0.01535	-0.00563	-0.08222	-0.01566
OR1J2	-0.038512448	-0.057651534	-0.06005	-0.00778	-0.03654	-0.03387
OR1J4	-0.055956752	-0.09643111	0.023724	0.019636	-0.03642	0.022944
OR1Q1	-0.042769448	-0.022576089	-0.04797	0.003143	-0.02725	-0.15656
OR2A4	-0.029113128	0.003189229	-0.00623	-0.00472	0.017008	-0.08758
OR2B2	-0.040909259	-0.055328817	0.150824	0.009543	-0.00632	-0.02988
OR2B3	-0.032323041	-0.049316594	0.08403	0.016689	-0.03395	-0.09599
OR2B6	-0.041689809	-0.041752012	-0.08894	0.011321	-0.00378	0.028239
OR2C1	-0.025194248	-0.080853377	0.067155	-0.00596	-0.02521	-0.06903
OR2C3	-0.062872206	-0.013154506	-0.01946	-0.01693	0.028038	-0.08229
OR2F1	-0.044365363	0.047389239	-0.0979	0.041573	-0.09465	0.046784
OR2F2	-0.063030432	-0.086564363	0.016085	-0.0241	0.078916	0.045832
OR2H1	0.005253042	-0.046530589	-0.0769	0.09787	-0.05599	-0.12447
OR2H2	-0.036281293	-0.061692763	-0.0554	-0.00062	0.029074	0.085706
OR2J2	-0.066791951	-0.021679095	-0.05023	-0.02825	0.099877	0.021232
OR2J3	-0.057797983	-0.053562116	0.059534	0.031813	-0.08531	0.124902
OR2K2	-0.051786304	-0.114000234	-0.03399	-0.01344	0.07407	-0.05405
OR2L13	-0.058844862	-0.046874428	-0.10301	-0.02563	0.061968	-0.07288
OR2L1P	-0.047015699	-0.055136896	-0.06572	0.0216	-0.04449	0.01495
OR2L2	-0.050270502	0.039072962	-0.14775	-0.0176	-0.02918	0.02772
OR2M4	-0.053943791	-0.069447748	-0.06692	-0.00276	0.027823	0.084685
OR2S2	-0.032306038	-0.064251837	-0.03596	0.028191	-0.07408	0.076525
OR2W1	-0.047430201	-0.038421664	-0.00983	-0.03385	0.098026	0.05107
OR3A1	-0.038106334	0.002474867	-0.08195	0.040893	-0.08645	0.059746
OR3A2	-0.044765857	-0.036917652	-0.01469	0.026826	-0.05208	-0.0283
OR3A3	-0.023770843	-0.056722564	-0.04121	0.024512	-0.07366	-0.10763
OR4C1P	-0.038514979	0.001889476	0.003719	0.033125	-0.07569	-0.0688
OR4D1	-0.035475376	-0.081512976	-0.10518	0.008296	-0.033	-0.04467
OR4D2	-0.034518824	0.01906559	0.069659	0.015967	-0.04209	0.003849
OR51B2	-0.024492674	-0.072830661	0.003353	-0.02241	0.023796	-0.0118
OR51B4	-0.02610228	-0.0369317	0.032539	0.020964	-0.06205	-0.04038
OR51B5	-0.036115992	-0.025382631	0.039716	0.00616	-0.01031	-0.05252
OR51B6	-0.034740466	-0.053766421	-0.01057	0.031145	-0.08469	-0.09001
OR51E1	-0.028696067	-0.057113732	0.030778	0.00767	-0.02517	-0.00924
OR51E2	-0.029480807	-0.026383011	0.039577	-0.00603	-0.07425	-0.03301
OR51I1	-0.031466778	-0.026066709	0.010455	-0.0073	-0.04265	-0.1771
OR51I2	-0.02770032	0.026630465	0.034632	0.001299	-0.01274	0.002244
OR51M1	-0.030071136	-0.055531724	0.049517	0.002222	0.000797	-0.08519
OR52A1	-0.032524623	-0.026017506	0.03616	-0.00561	0.020103	-0.07463
OR52D1	-0.030328894	-0.045012038	-0.03177	-0.00179	0.004091	-0.03195

OR52K3P	-0.005861525	-0.107948406	-0.28124	-0.06582	0.183948	0.556141
OR5AK4P	-0.066055766	-0.121819669	-0.10304	0.005721	-0.00239	0.066298
OR5E1P	-0.033817076	-0.002296627	-0.03122	0.006833	-0.00044	0.014573
OR5H1	-0.120602236	-0.128876917	0.058278	-0.00106	0.031966	0.021003
OR5I1	-0.029038274	-0.017088221	0.064679	0.023267	-0.06698	0.025278
OR5J2	-0.076369413	-0.04683327	-0.11532	-0.03272	0.09727	0.10084
OR5K1	-0.038526099	-0.096992655	0.05059	-0.00764	0.03837	-0.12764
OR5L2	-0.038218608	-0.034921944	-0.01585	0.030627	-0.0942	-0.07636
OR5P2	-0.02599484	-0.020651932	0.101165	0.02258	-0.05214	-0.11872
OR5P3	-0.029751493	-0.007375551	-0.02901	0.036214	-0.1019	-0.10405
OR5V1	-0.035495432	-0.03902353	0.002747	0.01161	-0.01742	-0.00644
OR6A2	-0.032227145	-0.005616468	-0.0417	0.01953	-0.02731	0.037145
OR6B1	-0.035915803	-0.046325468	0.001934	-0.00854	0.053517	0.096721
OR6W1P	-0.033782198	-0.031731636	0.001086	0.028813	-0.06211	0.085161
OR7A10	-0.046910894	-0.028123705	-0.05156	-0.02858	0.098261	0.067839
OR7A17	-0.034631302	0.00505567	-0.03468	0.015059	-0.02268	-0.07135
OR7A5	-0.024523167	-0.021023482	-0.06803	-0.00793	0.002158	0.016765
OR7C1	-0.040484017	-0.09105377	-0.04451	0.014335	-0.01914	0.058331
OR7C2	-0.026995086	-0.032654697	0.055392	0.004996	-0.07899	-0.04891
OR7D2	-0.016282182	-0.033273406	0.033248	-0.0228	0.021284	-0.06139
OR7E104P	0.041058931	-0.012224523	0.023961	0.02364	-0.06599	-0.20629
OR7E12P	-0.012987011	-0.04221356	-0.02127	0.002839	-0.09501	0.007951
OR7E14P	-0.040021787	-0.015083247	0.04245	-0.00857	0.049288	0.092351
OR7E156P	-0.018976019	-0.038265154	-0.04108	-0.0236	-0.00784	0.015855
OR7E19P	-0.030076143	-0.012343907	-0.03658	0.011033	-0.02555	-0.05974
OR7E24	-0.037199797	-0.050417863	0.055159	0.031258	-0.07519	-0.14334
OR7E2P	-0.068865904	-0.01624639	0.034008	-0.0655	0.171634	0.014999
OR7E37P	0.020418814	-0.05181968	-0.05159	0.05444	-0.18504	0.110472
OR7E47P	-0.016931558	-0.061965322	-0.06949	0.001599	-0.05403	-0.00842
OR8B2	-0.034125518	-0.046227483	0.017325	0.007092	-0.0145	-0.1403
OR8B8	-0.031728804	-0.057377534	-0.01923	0.007586	-0.03351	-0.01953
OR8D1	-0.030935401	-0.045040757	0.043893	-0.01526	0.033459	-0.04548
OR8D2	-0.033536664	-0.059910529	0.012568	0.00801	-0.01639	-0.05406
OR8G1	-0.020802568	-0.067074743	0.03952	-0.00788	0.00538	-0.14137
OR8G2	-0.070138812	-0.023534915	-0.096	-0.01162	0.074149	0.010975
OR9A1P	-0.073304137	-0.008324869	-0.07713	-0.02841	0.068523	0.087364
ORAI1	0.020020686	0.099936597	0.110822	-0.01772	0.005975	-0.04526
ORAI2	0.013277318	-0.005338615	-0.27787	-0.00734	0.063576	-0.07422
ORAI3	0.025975373	-0.012393871	-0.25247	-0.05301	0.136845	0.133418
ORAOV1	-0.027974425	-0.030603781	0.05155	-0.009	0.017452	0.003433
ORC1	-0.01625611	0.020143616	0.007007	0.188852	-0.41613	-0.0266
ORC2	0.018687686	0.05707452	0.121405	0.036315	-0.1063	-0.30821
ORC3	0.022238664	-0.002087065	0.112702	0.041208	-0.09422	-0.07192
ORC4	0.021924115	0.068331867	-0.01184	0.008875	0.004742	-0.06379
ORC5	-0.005731423	0.077353025	0.131616	0.065675	-0.15338	0.008032
ORC6	0.019044421	-0.010985127	-0.00941	0.137776	-0.41727	-0.15594
ORF1	-0.057962498	-0.016135958	-0.07562	-0.00112	0.02397	0.087718
ORM1	-0.01194505	-0.062729361	-0.10291	-0.00264	0.005383	0.188037
ORMDL1	-0.035014392	-0.070694752	0.150563	-0.1452	0.18896	0.245921
ORMDL2	0.046449726	0.1071671	-0.03106	0.055012	-0.16036	0.040627
ORMDL3	0.010131654	-0.00096067	0.305353	-0.04824	0.103089	-0.33604

OS9	0.065992229	-0.035695137	-0.32494	0.007087	0.018977	0.178218
OSBP	0.030878613	0.059552283	0.032674	0.03447	-0.12892	-0.1621
OSBP2	0.025795976	-0.051685178	0.070475	-0.06057	0.105039	-0.00936
OSBPL10	-0.079938171	-0.107777378	-0.05424	0.032863	0.087236	-0.14619
OSBPL11	0.033795971	-0.022790536	-0.47792	-0.02421	0.100511	0.393922
OSBPL1A	-0.003886466	-0.15881627	-0.17125	-0.03162	0.163822	0.40401
OSBPL2	0.028144912	0.076654216	-0.20328	-0.01354	0.037797	-0.16434
OSBPL3	0.013110058	0.144647452	0.147637	0.033984	-0.09945	-0.13632
OSBPL5	0.006898836	0.300633266	-0.03042	-0.03803	0.100112	0.024113
OSBPL6	-0.04517406	-0.025899975	-0.06147	0.108432	-0.21186	-0.04415
OSBPL7	-0.080398673	-0.023033642	0.007239	-0.05515	0.102076	-0.03504
OSBPL8	0.052417098	0.02265936	-0.19571	0.004655	0.013451	0.106493
OSBPL9	0.067019825	0.008886731	-0.12752	0.051875	-0.11593	-0.23263
OSCAR	-0.004380742	-0.127617508	-0.28929	-0.09592	0.261718	0.592144
OSCP1	-0.029600032	-0.063629786	-0.00324	0.050448	-0.13809	-0.05803
OSER1	0.042918149	-0.034552832	-0.33403	0.001434	-0.00354	-0.08158
OSER1-AS1	-0.013581561	0.042335286	0.228774	-0.02701	0.095636	-0.37696
OSGEP	0.02156077	-0.07230455	-0.10002	0.047858	-0.16205	0.018723
OSGEPL1	0.009453857	0.064704104	0.22385	0.014553	-0.04018	-0.22293
OSGIN1	-0.011620766	-0.103391693	0.010681	0.021109	-0.0987	0.030954
OSGIN2	-0.019614866	-0.019097112	-0.1457	0.018067	-0.08203	0.010585
OSM	-0.000325979	-0.035291576	-0.02136	0.093435	-0.27298	-0.0691
OSMR	-0.026173318	-0.007010926	-0.04318	-0.04411	-0.14955	-0.0662
OSR1	-0.041891961	-0.026606518	0.013706	0.001208	0.011663	-0.0088
OSR2	-0.031806147	-0.019493158	-0.09385	0.062975	-0.16254	-0.02664
OST4	0.062815136	0.006715702	0.22961	-0.03019	0.098494	-0.03417
OSTC	0.043954236	0.090955965	0.147559	0.034469	-0.09897	0.15587
OSTCP1	-0.049104523	-0.000339938	-0.10267	0.011873	-0.01869	0.055337
OSTF1	0.044310982	0.146927896	-0.21137	0.057703	-0.13516	0.068489
OSTM1	0.025471804	0.028679972	-0.08596	-0.0306	0.074604	0.214357
OSTM1-AS1	-0.044036082	-0.030192273	-0.03516	-0.0082	0.031531	0.026572
OTC	-0.037648668	-0.061719565	-0.05462	0.031576	-0.07184	-0.02815
OTOF	-0.050961655	-0.029720051	0.009566	0.023951	-0.05524	-0.04818
OTOGL	-0.039904502	-0.043976241	0.062234	0.015117	-0.04182	-0.02011
OTOP2	-0.036734092	-0.063041854	-0.09344	0.014081	-0.05812	-0.01324
OTOR	-0.031870321	-0.093827305	-0.09327	-0.01378	0.039825	0.060452
OTOS	-0.034063073	-0.082621798	-0.04169	0.006062	0.007464	0.087754
OTP	-0.053128471	-0.03671789	-0.12151	0.018049	-0.03773	-0.02141
OTUB2	-0.007269573	-0.001545599	0.037679	0.022037	-0.37497	0.114166
OTUD1	0.011936857	-0.080233439	-0.05299	-0.10362	0.240313	0.089734
OTUD3	-0.018024104	-0.006315628	0.215222	-0.00848	-0.00623	-0.20802
OTUD4	-0.014888824	0.022181427	0.06262	-0.00075	-0.03319	-0.00504
OTUD5	0.010860679	-0.010212574	0.089846	0.015163	-0.17916	-0.26963
OTUD6B	0.022568198	0.021418041	-0.10938	0.041177	-0.1385	0.066094
OTUD6B-AS	0.018452793	0.12404215	0.217948	0.007026	-0.01689	-0.21823
OTUD7A	-0.007297324	0.069608458	0.199544	0.025455	0.030404	-0.11398
OTUD7B	-0.020163028	0.109199236	0.106604	0.074143	-0.13902	-0.15379
OTULIN	-0.001084124	0.029502924	-0.10786	0.089813	-0.19463	0.016509
OTX1	-0.033554255	-0.030622105	0.045451	0.029133	-0.05269	0.073922
OTX2	-0.064841921	-0.070697368	########	-0.02827	0.042564	-0.07144
OTX2-AS1	-0.022499289	-0.051634803	-0.02801	-0.00309	-0.00069	-0.07517

OVCH1-AS:	-0.027471952	-0.054712735	-0.0827	-0.02419	0.051261	0.107849
OVGP1	-0.00766199	-0.118664617	0.195758	-0.01136	0.049413	-0.20403
OVOL1	-0.031910406	-0.02784193	-0.02353	-0.01352	-0.03567	-0.08613
OVOL1-AS:	-0.033484637	-0.01010803	-0.0068	0.008859	-0.07166	-0.00216
OVOL2	-0.047233964	-0.048389265	0.065209	0.023126	-0.04526	-0.09753
OVOL3	-0.034472756	-0.060021187	-0.11687	0.036519	-0.09568	-0.02004
OXA1L	0.05509644	-0.03835756	0.077845	0.025058	-0.0549	0.172641
OXCT1	0.021722566	0.058382649	0.300881	0.051302	-0.16233	-0.5203
OXCT2	-0.028574519	-0.037408093	-0.02348	-0.00371	-0.02865	-0.02204
OXER1	0.022305646	-0.061414995	0.001206	-0.00441	-0.04501	-0.15872
OXGR1	-0.028176165	-0.059234943	-0.01266	0.001048	0.006513	-0.15281
OXLD1	0.029881058	-0.006241733	0.267312	0.047754	-0.14941	0.01039
OXNAD1	0.012117808	0.013381066	0.586751	9.06E-05	-0.00464	-0.26648
OXR1	-0.023536746	-0.042067133	-0.11764	-0.04654	0.030363	0.209238
OXSM	0.024575605	0.026046419	0.144514	0.052545	-0.16064	-0.18966
OXSR1	0.087605624	0.111848411	-0.13929	0.098363	-0.23103	-0.12961
OXT	-0.023240652	-0.098197283	-0.09741	0.00011	-0.00987	-0.04956
OXTR	-0.011484135	-0.012657787	0.003945	-0.04527	0.137283	-0.03071
P2RX1	0.002411197	-0.164049157	-0.31626	-0.11155	0.291489	0.242584
P2RX2	-0.027716681	-0.078476702	-0.02361	0.016699	-0.03245	-0.09021
P2RX3	-0.030482005	0.000341372	-0.11699	0.007994	-0.00436	0.125976
P2RX4	0.021129411	0.027570502	0.023271	-0.00625	0.011782	0.371012
P2RX5	0.008757953	-0.070096202	0.056611	-0.0359	0.074902	-0.20806
P2RX6	-0.04297278	-0.107257415	-0.02914	0.00688	-0.04556	-0.09637
P2RX7	0.0079899	0.035740918	-0.12053	-0.0721	0.218383	0.525755
P2RY1	-0.019338839	-0.017395708	-0.09812	-0.01611	0.056796	0.061127
P2RY10	0.002485875	-0.048981988	0.117185	-0.01106	0.014869	-0.50243
P2RY12	-0.010859566	-0.05842316	-0.15588	-0.19028	0.593857	0.39496
P2RY13	0.006737442	-0.107262778	-0.38515	-0.0863	0.260912	0.45022
P2RY14	0.002785762	-0.115750644	-0.21307	-0.05419	0.119785	-0.34411
P2RY2	-0.008977503	-0.11061109	-0.16105	-0.06705	0.17395	0.307342
P2RY4	-0.034844713	0.023602784	-0.09039	0.019587	-0.03532	-0.0288
P2RY6	-0.009256446	-0.084775693	-0.15784	-0.02537	0.058381	-0.08148
P2RY8	0.024474503	0.079359902	0.196299	-0.05064	0.121729	-0.11445
P4HA1	0.012329148	-0.05208628	-0.11038	0.060014	-0.16433	0.06072
P4HA3	-0.038090106	-0.052010174	0.0594	-0.00115	-0.04712	-0.09023
P4HB	0.007989981	0.153383322	-0.09888	0.055296	-0.14891	0.316136
P4HTM	0.019669673	-0.019650545	0.324599	0.043678	-0.12581	-0.02095
PA2G4	0.027012548	0.069570395	0.278335	0.082692	-0.23192	-0.01787
PAAF1	0.02135295	0.008542021	0.102434	0.055859	-0.16588	0.014323
PABPC1	0.047720501	-0.009825196	-0.03626	0.070469	0.010906	0.063106
PABPC1L	0.00516196	-0.036793262	0.027102	-0.07061	0.099196	-0.21192
PABPC3	0.103749845	0.024785398	0.012778	0.063399	-0.07252	0.155542
PABPC4L	-0.046178687	-0.033746558	-0.05712	0.018583	-0.04015	-0.06861
PABPC5	-0.165417163	-0.106742363	0.038017	0.012823	0.135897	0.026507
PABPN1	0.040621729	0.01250802	-0.12506	-0.09155	0.25079	-0.04465
PACRG	-0.05088824	-0.080960749	-0.03412	0.025704	-0.04338	-0.02344
PACRG-AS:	-0.043292808	-0.011876687	-0.0968	0.005268	0.007621	0.016724
PACRGL	-0.026930499	-0.075568324	0.020679	0.038554	-0.07982	0.114756
PACS1	0.012824218	-0.076574558	0.062806	-0.07757	0.192578	-0.18761
PACS2	0.010255393	-0.106203767	-0.031	-0.0524	0.021376	0.098649

PACSIN1	-0.006073113	-0.019453374	-0.03289	-0.02922	0.095342	-0.20228
PACSIN2	0.014886277	-0.074745321	-0.12853	-0.03754	0.05832	-0.02869
PACSIN3	-0.02794036	-0.050967784	0.017727	0.010974	-0.02402	-0.05906
PADI1	-0.006653052	-0.06865649	0.01905	-0.01759	-0.02637	-0.10323
PADI2	-0.02984046	-0.079065209	-0.14528	-0.01181	0.018802	0.050716
PADI3	-0.033155147	-0.058501611	-0.09999	0.014036	-0.05235	0.09128
PADI4	0.010557751	-0.131466909	-0.24119	-0.07589	0.247227	0.516287
PAEP	-0.034000722	-0.026219682	0.011219	0.020731	-0.02898	0.100897
PAF1	0.050582055	0.000645529	-0.08994	0.05129	-0.18687	-0.07909
PAFAH1B1	0.103838007	0.133421047	-0.03406	-0.00911	-0.13581	-0.13704
PAFAH1B2	-0.017085336	-0.064393909	-0.168	0.033972	-0.14161	0.198102
PAFAH1B3	0.006899011	-0.11245256	0.121929	0.068921	-0.22189	0.027008
PAFAH2	0.008772802	0.183367554	0.029048	-0.02664	0.088165	-0.17204
PAG1	-0.009888049	-0.053207747	0.210381	0.00146	0.042261	-0.12339
PAGE1	-0.037702656	-0.020628818	-0.07665	0.012992	-0.00624	-0.13694
PAGE4	-0.038604671	0.041063476	-0.17833	-0.00011	0.028827	0.040882
PAGE5	-0.028813128	-0.083338354	0.059502	0.012093	-0.03277	-0.00686
PAGR1	0.017908092	-0.005325498	0.03901	0.032057	-0.07696	0.020897
PAICS	0.024731236	-0.009409624	0.121205	0.103036	-0.22113	-0.12788
PAIP1	0.009357089	-0.030553642	0.03203	0.038318	-0.18015	0.125475
PAIP2	0.109415627	0.142175424	-0.03235	0.03482	-0.04977	-0.18246
PAIP2B	-0.012370538	-0.048687541	0.160702	-0.06207	0.159033	-0.32169
PAK1	-0.012248814	-0.140339992	-0.26704	-0.09113	0.268182	0.404062
PAK1IP1	0.025378789	0.047999302	-0.01351	0.046702	-0.11616	0.094259
PAK2	0.051261809	0.006357211	-0.34047	-0.01942	-0.04015	0.140306
PAK3	-0.052944095	-0.087819704	0.05081	-0.00698	-0.01164	-0.09269
PAK4	0.002606267	-0.071368133	-0.04583	0.027262	-0.15015	0.002955
PAK6	-0.020371451	0.309495894	-0.00016	-0.01733	0.052053	-0.19646
PAK7	-0.149370312	-0.111712068	-0.0182	0.012805	0.033992	-0.00939
PALB2	0.007531479	0.046133133	0.08669	0.053496	-0.22211	-0.06844
PALD1	-0.011736636	-0.077462011	-0.10513	-0.00256	-0.03192	-0.21468
PALLD	0.012738208	0.172342504	-0.04189	-0.04803	0.124819	0.214575
PALM	-0.021736433	-0.060999862	-0.03122	-0.02267	-0.0494	-0.11016
PALM2	-0.024003995	0.0082717	0.038359	0.10177	-0.16965	-0.0428
PALM3	-0.030072166	-0.041368514	-0.10781	-0.01757	-0.00263	-0.0617
PALMD	-0.097417946	-0.074765885	-0.04432	0.013203	0.097912	-0.02312
PAM	0.032725612	0.204030168	0.108835	0.011009	0.042817	0.031824
PAM16	0.01363288	-0.084395698	-0.08293	0.095902	-0.29168	0.150215
PAMR1	-0.023216458	-0.062586132	0.016553	0.003457	-0.05005	0.080861
PAN2	0.015655743	-0.030861637	0.142723	-0.01231	0.05204	-0.03947
PAN3	0.096304375	0.04963169	-0.20581	-0.00115	0.057002	-0.32251
PAN3-AS1	-0.004432446	-0.056961099	-0.08647	-0.04917	0.167899	-0.00479
PANK1	-0.01469763	0.018997197	-0.02014	0.101475	-0.27081	-0.00426
PANK2	-0.038598224	-0.010171044	0.00608	0.004508	0.027543	0.18392
PANK3	0.024130216	0.107211763	-0.01237	0.054061	-0.16112	0.052586
PANK4	0.030855778	0.049383849	0.23884	-0.00853	-0.00449	0.128628
PANX1	-0.025952002	0.11045355	-0.03285	-0.03905	0.047018	0.199746
PANX2	-0.047042728	-0.134244402	-0.03894	-0.04944	-0.03187	-0.09594
PANX3	-0.062958884	-0.057122957	-0.00775	0.000439	0.005597	-0.04259
PAOX	-0.039223217	-0.067731423	-0.03359	-0.02514	0.121032	0.020165
PAPD4	0.001847691	0.101508759	-0.10659	-0.01056	0.016823	0.002543

PAPD5	0.037130288	0.179829511	0.068623	0.016903	-0.03781	-0.09732
PAPD7	0.038986675	-0.018131345	0.167323	0.051454	-0.135	-0.18345
PAPLN	-0.011642923	-0.095292546	-0.13257	-0.01354	0.04581	-0.04922
PAPOLA	0.067396924	-0.014929169	-0.12209	0.039503	-0.0694	0.028137
PAPOLB	-0.063948137	-0.01425115	-0.01359	-0.00133	0.006112	########
PAPOLG	0.043627264	0.120411362	0.117139	0.05275	0.00944	-0.16225
PAPPA	-0.03211975	-0.076033414	0.022235	0.0051	-0.04686	0.013917
PAPPA2	-0.046170555	-0.014980363	-0.11365	0.023863	-0.06397	0.008529
PAPSS1	0.040010947	-0.047468474	-0.2628	-0.01797	0.063092	0.124878
PAPSS2	-0.000762674	-0.132129849	-0.17971	-0.08947	0.287249	0.469796
PAQR3	-0.011123845	0.058221735	0.067578	0.10482	-0.26388	-0.03216
PAQR4	-0.002687664	-0.055922831	-0.08995	0.080501	-0.30628	0.163423
PAQR5	-0.082382353	-0.02774325	-0.00116	0.012057	-0.01171	-0.04456
PAQR6	-0.005667692	-0.089726317	-0.10868	0.014246	-0.10133	0.009218
PAQR7	-0.010411531	-0.10891423	-0.14319	-0.03776	0.09708	0.300729
PAQR8	0.01531904	-0.01925177	0.202104	-0.01414	0.032945	0.009544
PAQR9	-0.049654418	-0.026186047	-0.00328	-0.02484	0.066684	-0.07203
PARD3	-0.030397846	-0.052024334	0.032806	-0.04028	0.137573	-0.10142
PARD3-AS1	-0.057317024	-0.066906458	0.062797	-0.01625	0.032164	0.108222
PARD3B	-0.054063946	-0.048075518	0.031586	0.024322	-0.08098	-0.06992
PARD6A	0.001971841	-0.062691752	0.014605	0.041992	-0.23872	-0.13466
PARD6B	-0.013988664	-0.030317568	0.059926	-0.0017	-0.06943	-0.11015
PARD6G	0.030236692	-0.107457728	-0.06783	-0.15761	-0.01344	0.112274
PARD6G-AS	-0.032547696	-0.02377825	0.057272	-0.01672	-0.01595	-0.08694
PARG	0.028172527	0.020813841	-0.00243	0.017768	-0.13066	-0.03322
PARK2	-0.028557872	-0.050903519	0.048808	0.076029	-0.18663	-0.0444
PARK7	0.065665366	0.114108591	0.039797	0.113208	-0.24667	0.075259
PARL	0.032444251	-0.04248264	-0.04684	0.025081	-0.06778	0.341722
PARM1	-0.014953189	-0.077840886	-0.15782	-0.04169	0.085475	-0.23652
PARN	0.031792128	0.051992528	0.077166	0.052995	-0.16025	-0.2087
PARP1	0.02888969	0.019659227	0.179067	0.062836	-0.19878	-0.32338
PARP10	-0.045094339	-0.101145626	0.053521	-0.03275	-0.03454	-0.12769
PARP11	-0.06026487	0.042810507	0.077881	0.033625	0.072378	-0.07379
PARP12	0.033406485	0.029767238	-0.00662	-0.00671	0.020637	-0.0796
PARP14	0.010364832	0.039429092	-0.18663	0.005903	-0.01624	0.06357
PARP15	-7.95E-05	0.164322488	0.131198	-0.04364	0.138913	-0.31749
PARP16	0.017050049	0.021560988	0.188078	-0.02376	-0.02365	-0.39528
PARP2	0.034737386	0.079420471	0.129943	0.11877	-0.1978	-0.11921
PARP3	-0.011250455	-0.117195894	0.087188	0.033514	-0.09693	0.050391
PARP4	0.065742138	0.10952859	-0.12263	0.005664	0.014149	-0.09212
PARP6	0.042350062	-0.038492109	0.16491	-0.03802	0.084062	-0.08095
PARP8	0.023810844	0.125268681	0.191955	-0.05677	0.171387	-0.09342
PARP9	0.023592993	0.050135263	-0.13585	-0.01383	0.037097	0.075569
PARPBP	-0.026864786	0.009584229	-0.02993	0.180456	-0.28695	-0.05775
PARS2	-0.013975198	0.029677798	-0.01771	0.062978	-0.18126	0.034769
PART1	-0.072797587	-0.04157916	0.14687	0.02015	-0.0315	-0.16996
PARVA	-0.007843853	0.007444052	0.055284	0.058594	0.027343	-0.17951
PARVB	0.002912503	-0.121325823	-0.18509	-0.02927	0.057326	0.193025
PARVG	0.0105648	-0.110910604	-0.16859	-0.00923	0.031497	0.17781
PASD1	-0.019733146	-0.013676769	-0.07042	0.070694	-0.19042	0.001486
PASK	0.007824155	-0.081347343	0.494438	-0.01585	0.071922	-0.28282

PATE1	-0.04747633	-0.037444583	-0.03759	0.005421	-0.00675	-0.0891
PATE2	-0.036384416	-0.025501258	-0.05218	-0.00199	0.024632	-0.024
PATL1	-0.016848705	-0.039367166	-0.15016	0.028404	-0.22409	-0.00403
PATL2	0.010666272	0.107575752	0.30808	-0.05629	0.097341	-0.38062
PATZ1	0.013783266	-0.086698062	0.215589	0.020281	0.003615	-0.15224
PAWR	-0.037200604	-0.062793663	-0.01773	-0.04397	0.188586	-0.22887
PAX1	-0.000847879	0.072236764	-0.13696	-0.00122	0.047656	0.102
PAX2	-0.03288051	-0.050576148	-0.0677	0.009319	-0.01894	-0.04167
PAX3	-0.067545244	-0.084357993	0.05961	0.065829	-0.01146	0.160736
PAX4	-0.079421821	-0.070853272	-0.10426	0.012058	-0.04644	0.004453
PAX5	-0.007935496	-0.098011812	-0.06092	-0.06968	0.169274	-0.05318
PAX6	-0.085286568	-0.087389329	0.100462	-0.01904	0.040805	-0.07385
PAX7	-0.044304422	-0.079759017	0.164625	0.025253	-0.05707	0.121244
PAX8	-0.066510557	-0.071928716	-0.06902	-0.01852	-0.0585	0.098391
PAX8-AS1	0.009575954	-0.010175246	0.013382	-0.04801	0.111028	0.039751
PAX9	-0.042643402	-0.071340073	0.03769	0.114941	-0.13278	-0.04125
PAXBP1	0.018408437	-0.001294993	0.063725	0.018275	-0.02312	-0.00097
PAXBP1-AS	-0.025555581	-0.095944514	0.049225	-0.05108	0.152723	0.275934
PAXIP1	0.026268136	0.042947056	0.001785	0.108403	-0.32348	-0.11165
PAXIP1-AS	0.010687867	0.014200147	0.213418	0.034343	-0.09291	-0.20355
PAXIP1OS	-0.000852038	0.076635561	0.115024	-0.04106	0.153063	0.043683
PBDC1	0.020384705	0.120518184	0.16955	-0.01292	0.046356	0.023972
PBK	-0.013255792	-0.008066969	0.016349	0.130145	-0.17653	-0.03888
PBLD	-0.017382643	-0.051636243	-0.19769	-0.05034	0.165152	0.438563
PBOV1	-0.056532099	-0.08832287	0.212718	0.009786	-0.0169	-0.03306
PBRM1	0.004427815	0.08064043	-0.03083	0.015028	-0.04792	0.069655
PBX1	-0.060625925	-0.003092778	-0.02264	-0.16741	0.633375	-0.028
PBX2	-0.006347778	-0.121496423	-0.16042	-0.033	0.013367	-0.03671
PBX3	0.033224567	-0.059314921	-0.11977	-0.03441	0.103515	0.003129
PBX4	0.00286672	-0.060331267	0.360699	0.01537	-0.07526	-0.20981
PBXIP1	0.01695372	-0.042820898	0.145705	-0.04038	0.093037	-0.26143
PC	-0.02671114	-0.068515315	-0.00172	-0.01785	0.061947	0.261485
PCA3	-0.060752215	-0.042538416	-0.01636	-0.00378	0.04536	-0.05909
PCAT18	-0.022969666	0.012450497	-0.02275	0.065278	-0.18165	-0.0554
PCAT19	-0.026461023	-0.059638868	0.009022	0.05491	-0.21221	-0.12895
PCAT4	-0.031747883	-0.047501684	0.048594	0.002911	-0.04796	-0.08544
PCAT6	-0.029090931	-0.087485969	-0.01464	0.014412	-0.02345	0.100499
PCBD1	0.006059879	-0.02604016	-0.05053	0.085234	-0.25001	0.315842
PCBD2	0.020846709	-0.069541242	-0.03789	0.033971	-0.15233	0.255931
PCBP1	0.088826946	0.136533201	-0.11815	0.024639	-0.04069	-0.16172
PCBP1-AS	-0.052887606	-0.019276055	-0.13644	0.002803	-0.05861	-0.13079
PCBP2	0.076827927	0.002101408	-0.05537	-0.01202	0.032503	0.15346
PCBP3	-0.021750774	-0.050610053	-0.04693	0.056313	-0.20933	-0.02907
PCBP4	-0.00806695	-0.001692903	0.175091	-0.01011	-0.04322	-0.29983
PCCA	-0.028759838	-0.00245727	0.020178	0.028457	-0.09197	0.237279
PCCB	0.016936447	0.094227911	0.07612	0.072588	-0.19237	0.022621
PCDH1	-0.023771593	-0.003058522	-0.03979	0.008844	-0.08731	-0.04764
PCDH10	-0.104417506	-0.11135972	0.048428	0.024816	-0.00444	-0.04651
PCDH11X	-0.04243515	-0.071962299	-0.0319	-0.01982	0.060372	0.062079
PCDH12	-0.022628535	-0.078859364	-0.02118	-0.01396	-0.0523	0.050264
PCDH15	-0.099062568	-0.077449899	-0.02249	-0.0137	0.036871	0.043367

PCDH17	-0.036664393	-0.051984482	-0.00548	-0.05446	0.158275	-0.05117
PCDH18	-0.027561021	-0.048394753	0.049313	0.066219	-0.16765	0.078133
PCDH19	-0.045795285	0.004482962	-0.01619	-0.00571	0.053135	0.041885
PCDH7	-0.071934349	-0.104400594	-0.02618	0.021722	0.02564	-0.04593
PCDH8	-0.07991792	-0.056952264	0.015385	-0.02912	0.094801	-0.09384
PCDH9	-0.039305693	-0.08334876	-0.11835	-0.04503	0.004811	-0.09975
PCDHA10	-0.03034545	-0.047169141	-0.04002	0.016964	-0.04512	0.074005
PCDHA2	-0.042714307	-0.054273237	-0.17043	0.018267	-0.02311	-0.07744
PCDHA5	-0.038658355	-0.029930713	-0.02961	0.035854	-0.1017	-0.10627
PCDHA9	-0.032794924	0.052959889	0.08306	-0.01267	0.057058	-0.0346
PCDHAC1	-0.044443319	-0.064835893	0.011139	0.041886	-0.09961	-0.14216
PCDHAC2	-0.040957591	-0.017115527	-0.03891	0.022453	-0.03365	-0.02315
PCDHB1	-0.0371464	-0.032153958	-0.05081	0.017715	-0.03481	0.002514
PCDHB11	-0.029232693	0.000250246	-0.00591	0.01601	-0.04659	-0.02283
PCDHB12	-0.037746367	-0.029765001	-0.05921	0.035509	-0.09484	-0.10398
PCDHB13	-0.079075883	-0.093669621	-0.00437	-0.02672	0.014375	-0.07927
PCDHB14	-0.038049829	-0.017577981	-0.00823	-0.01041	-0.00378	-0.06899
PCDHB15	-0.051396441	-0.060205242	-0.02737	-0.01037	0.033115	-0.13622
PCDHB16	-0.056924486	-0.055392704	0.015913	-0.01444	0.029982	-0.15491
PCDHB17	-0.056329545	-0.074401233	0.054485	-0.01683	0.026759	0.156802
PCDHB18	-0.05058599	-0.042265424	-0.06493	0.004225	-0.06961	-0.02777
PCDHB2	-0.064322589	-0.001011411	0.024468	-0.01925	0.037745	-0.12089
PCDHB3	-0.119963016	-0.088605619	0.012884	0.024081	-0.02475	-0.1118
PCDHB4	-0.073362383	-0.007334314	-0.0619	-0.00119	0.017327	0.125485
PCDHB5	-0.056260496	-0.017314485	0.024996	0.014897	-0.05744	-0.02112
PCDHB6	-0.074523417	-0.03238108	-0.12901	0.014424	-0.06519	-0.05997
PCDHB7	-0.044381033	-0.085510285	-0.01007	-0.00526	0.038319	-0.00171
PCDHB8	-0.047982045	-0.023318305	0.077649	-0.00583	0.048093	0.087047
PCDHB9	-0.034762603	-0.005128385	-0.0119	0.031862	-0.08812	-0.11771
PCDHGA1	-0.034331538	-0.034411434	-0.03079	0.040538	-0.0966	-0.08277
PCDHGA10	-0.026115511	0.094838401	-0.03374	-0.00669	0.030073	-0.09183
PCDHGA3	-0.048900914	-0.053001328	-0.08246	0.021223	-0.03281	-0.01504
PCDHGA4	-0.053503322	0.03816194	0.018937	-0.03652	0.105368	-0.08282
PCDHGA8	-0.036240861	-0.033868972	0.039798	-0.02191	0.079806	-0.13713
PCDHGA9	-0.030169164	0.021575679	-0.03072	0.002927	-0.03923	0.048968
PCDHGB5	-0.02796788	-0.091024564	-0.02301	-0.0238	0.031159	0.077177
PCDHGB6	-0.038277809	-0.011830795	-0.04078	-0.01185	0.061387	0.042681
PCDHGB7	-0.094365571	-0.073213812	0.062841	0.05965	-0.01491	0.00443
PCDHGB8P	-0.041454963	-0.068101025	0.01425	0.029949	-0.06971	-0.01372
PCDHGC3	-0.046313334	-0.034760638	-0.04425	-0.00061	0.035513	-0.13551
PCDHGC4	-0.055040013	-0.047510601	-0.13773	0.013622	-0.01285	-0.04236
PCDHGC5	-0.058971595	-0.051299282	-0.08588	-0.02418	0.064963	0.111404
PCDP1	-0.01148874	-0.083781994	-0.00343	0.0318	-0.00209	-0.08895
PCED1A	0.008085074	0.009957326	0.211747	-0.02666	0.08903	-0.04229
PCED1B	0.012484864	0.017063213	0.476913	-0.06013	0.149373	-0.47934
PCED1B-AS	-0.000887928	0.138105612	0.431775	-0.03475	0.096913	-0.37613
PCF11	0.062222326	-0.057803279	0.167462	-0.03868	0.089193	-0.35868
PCGEM1	-0.056152831	-0.069629704	0.060859	0.000576	0.002686	-0.03148
PCGF1	0.024083581	-0.035528848	-0.1009	0.003015	-0.07535	0.273415
PCGF2	-0.018437959	-0.099485658	0.013564	0.016339	-0.05943	-0.06883
PCGF3	-0.068108156	0.061711711	-0.21394	0.033242	-0.17831	0.29147

PCGF5	0.12026551	0.039706383	-0.11044	-0.04394	0.173906	-0.01495
PCGF6	0.002435078	0.087147239	0.105661	0.062471	-0.20043	-0.17095
PCID2	0.02007702	0.095842058	0.222137	0.023106	-0.06601	-0.14924
PCIF1	-0.021899035	-0.079653617	0.017812	-0.02039	-0.07958	0.009948
PCK1	-0.075381444	-0.05510222	-0.05872	0.016426	-0.07003	-0.02594
PCK2	0.021697666	-0.057371338	-0.13526	0.038215	-0.11825	0.253035
PCLO	-0.009898924	-0.033494459	-0.01101	0.107438	-0.14422	-0.01649
PCM1	0.007672588	-0.00316298	0.205104	0.003226	-0.08328	-0.33223
PCMT1	0.020719298	0.072908287	-0.16193	0.020968	-0.10267	0.367399
PCMTD1	-0.031019825	0.096432097	-0.08127	-0.10963	0.127228	-0.09019
PCMTD2	0.01836735	0.055543781	0.167199	-0.02544	0.092556	-0.17299
PCNA	0.035836453	0.17337811	0.145666	0.122881	-0.34978	-0.11636
PCNP	0.026387706	0.097407879	0.216929	0.036956	-0.09761	-0.31342
PCNT	0.018870247	0.111949245	0.291682	0.019979	-0.04094	-0.19497
PCNX	0.029672538	0.012420249	-0.03184	0.038036	-0.08892	-0.0459
PCNXL2	0.014248961	-0.045768469	0.310168	0.12473	-0.07313	-0.06479
PCNXL3	0.007974286	0.073297	-0.0317	0.057518	-0.14873	-0.01753
PCNXL4	-0.015567058	0.119334892	-0.02005	0.022489	-0.06355	-0.05224
PCOLCE	-0.006378124	0.028946159	0.054182	0.077705	-0.22215	-0.07868
PCOLCE-AS	-0.01990721	-0.071171394	0.030022	-0.02174	-0.03981	-0.11687
PCOLCE2	-0.052734971	-0.077958958	0.000601	-0.02762	0.105576	0.008545
PCP2	-0.022754665	-0.017178097	0.074783	-0.15619	0.427405	-0.05521
PCP4	-0.036560562	0.027325776	-0.13916	0.033695	-0.07024	0.069138
PCP4L1	-0.039724094	-0.021304415	0.03711	0.00353	0.021434	0.039508
PCSK1	-0.055009124	-0.04616082	-0.00851	-0.03774	0.127885	-0.04906
PCSK1N	-0.014184081	-0.086334115	0.257493	0.064635	-0.16708	-0.06908
PCSK2	-0.055437382	-0.117134872	0.159998	0.047624	-0.12574	0.038241
PCSK4	-0.017154298	-0.122464423	-0.08608	-0.01904	-0.03571	-0.05278
PCSK5	0.001019974	0.153160932	0.082808	-0.09244	0.318051	0.348677
PCSK6	-0.082925693	-0.03761347	0.13852	-0.10519	0.151131	-0.05654
PCSK7	0.031909191	0.122897545	0.312389	-0.02658	0.071272	-0.3412
PCSK9	-0.023596677	-0.052792646	-0.03886	0.009085	-0.04972	-0.04692
PCTP	0.015072307	0.055590746	-0.32843	-0.08545	0.224134	0.295546
PCYOX1	0.011028592	0.066987984	0.105839	-0.02466	0.099424	0.046731
PCYOX1L	0.019846297	-0.065891325	0.179952	0.003447	-0.01386	0.10481
PCYT1A	0.035153486	0.067581856	-0.16215	-0.00067	-0.02256	0.018047
PCYT1B	-0.04420523	-0.042091389	0.039894	-0.19454	0.45704	0.057108
PCYT2	-0.016150007	-0.003303429	-0.00705	0.038613	-0.15654	-0.16109
PDAP1	0.003300187	0.040529638	0.10028	0.032203	-0.11086	0.195874
PDC	-0.066577926	-0.014970293	-0.00634	-0.00845	-0.00115	0.055717
PDCD1	-0.016655526	-0.079200849	0.076666	-0.00036	-0.06752	-0.07307
PDCD10	0.10618328	0.206704292	-0.01081	0.091473	-0.21076	-0.24372
PDCD11	0.018955233	-0.01814451	0.042683	0.032759	-0.15344	-0.14046
PDCD1LG2	-0.032412488	-0.041964582	-0.0131	0.0592	-0.16485	-0.02832
PDCD2	-0.010134707	0.072692006	0.139985	-0.00613	-0.00056	-0.13867
PDCD2L	0.006667223	0.012694148	0.119765	0.061131	-0.18232	-0.231
PDCD4	-0.004552773	-0.014794342	0.104903	-0.0254	0.066589	-0.12955
PDCD4-AS	0.003282126	-0.147823201	0.186643	-0.06709	0.20754	-0.29665
PDCD5	0.00996634	0.005008134	0.160266	0.038111	-0.10546	0.1148
PDCD6	0.015757322	0.002237233	0.084944	0.063718	-0.12912	-0.31658
PDCD6IP	0.066197993	0.020744233	-0.20891	-0.01705	0.054223	0.169512

PDCD7	0.030266335	0.077519833	0.079868	0.034453	-0.11056	-0.0804
PDCL	0.032971708	0.103988259	-0.0257	0.066854	-0.24776	-0.06398
PDCL2	-0.087254379	-0.05550087	-0.01058	-0.04626	0.084251	-0.00051
PDDC1	0.03200865	-0.027905798	0.095747	0.003423	-0.0272	0.008207
PDE10A	-0.041893415	-0.016926399	-0.09168	0.161805	-0.05129	-0.05824
PDE11A	-0.064147432	-0.043645646	0.022097	0.032971	-0.07392	-0.05684
PDE12	-0.005497529	0.039960352	0.022641	0.044849	-0.11246	0.139939
PDE1A	-0.070612195	-0.148145046	0.134546	0.017229	-0.06798	-0.1544
PDE1B	-0.014889658	-0.024922425	0.03927	-0.00315	-0.04933	0.004728
PDE1C	-0.050584456	-0.056556408	0.074871	0.002579	0.016797	-0.06592
PDE2A	-0.018177126	-0.014195204	-0.0287	-0.00099	0.02946	0.003017
PDE3A	-0.079895078	-0.082490419	0.017625	-0.05932	0.177301	-0.00713
PDE3B	0.013902534	-0.066867463	0.4512	-0.05593	0.003516	-0.1837
PDE4A	-0.028937552	0.02704983	-0.11491	0.060495	-0.09679	0.025666
PDE4B	0.01500882	-0.126669028	0.002065	0.015855	-0.03547	-0.3445
PDE4D	-0.015157633	0.071155634	0.081236	0.04521	-0.14296	-0.14952
PDE4DIP	-0.069300953	-0.103599747	-0.03049	0.079627	-0.20595	0.030326
PDE5A	-0.061440959	-0.056672098	-0.01778	-0.11785	0.564295	0.0066
PDE6A	-0.036095386	-0.059603266	-0.13468	0.024813	-0.05018	0.020387
PDE6B	-0.032562178	0.04934558	0.181643	-0.02362	0.073528	-0.32834
PDE6C	-0.103480276	-0.042252734	-0.0184	-0.04846	0.104966	-0.02966
PDE6D	0.044507255	0.054840705	0.012145	0.029358	-0.13209	-0.02869
PDE6G	-0.007579002	0.048072846	-0.05034	-0.01027	-0.0755	-0.03868
PDE6H	-0.041425542	-0.064320262	-0.0766	-0.0341	0.108281	0.337786
PDE7A	0.009839493	-0.013426723	0.24362	-0.04973	0.03015	-0.39897
PDE7B	-0.021953	0.010039999	-0.02227	########	0.0022	-0.02929
PDE8A	0.010703235	-0.001224656	-0.07858	-0.04308	0.138108	0.087987
PDE8B	-0.076863755	0.000160787	-0.09528	-0.08321	0.226368	0.232504
PDE9A	-0.021948248	-0.12096058	0.315598	-0.03275	0.110396	-0.18857
PDGFA	-0.013458161	0.030573387	-0.0393	-0.09537	0.232634	-0.02222
PDGFB	-0.057680343	-0.085528687	0.022674	-0.01391	0.023593	-0.07986
PDGFC	-0.002524373	-0.116528309	-0.27986	-0.07663	0.237056	0.553386
PDGFD	-0.006505075	0.342470136	0.153858	-0.06385	0.171597	-0.23774
PDGFRA	-0.067435746	-0.058877924	0.053795	0.048029	-0.0849	-0.04507
PDGFRB	-0.008100043	0.22701338	-0.05652	0.010203	-0.05375	-0.01259
PDGFRL	-0.016857782	-0.038922197	-0.06655	-0.00593	0.002561	0.001715
PDHA1	0.047020845	0.074481202	0.093335	0.07504	-0.18707	0.078643
PDHA2	-0.029784009	-0.017522874	-0.04682	0.007945	-0.02507	-0.05567
PDHB	0.06404136	0.133775844	0.022938	0.049014	-0.13487	0.105225
PDHX	0.03052394	0.066265591	0.055952	0.061221	-0.17152	0.206979
PDIA2	-0.039172669	-0.122883291	-0.00856	0.002032	-0.01159	0.079857
PDIA3	0.022820788	0.164197924	-0.04154	0.03544	-0.09929	0.057914
PDIA4	0.035228931	0.112233534	-0.05572	0.042966	-0.13925	-0.05905
PDIA6	0.047673916	0.157734007	-0.07664	0.072237	-0.19899	0.087291
PDIK1L	0.00927093	0.017491908	0.028284	0.037529	-0.06138	0.066347
PDILT	-0.037219585	-0.081686579	-0.01767	-0.01215	0.066536	0.062575
PDK1	-0.025674413	-0.161548	0.285131	-0.032	0.03797	-0.18465
PDK2	-0.008039264	-0.026343008	0.079682	0.004267	-0.05182	0.022128
PDK3	0.034507596	0.127055335	-0.11655	-0.01703	-0.00227	0.007087
PDK4	-0.012321696	-0.0741545	-0.32758	-0.02138	0.131191	0.447665
PDLIM1	0.008579884	0.0189385	-0.11273	-0.03432	0.083912	-0.25178

PDLIM2	0.014128786	-0.029652189	-0.10828	-0.01485	0.047184	0.064238
PDLIM3	0.002042673	-0.109074552	0.013993	0.07416	-0.03764	-0.11633
PDLIM4	-0.01622048	-0.049676493	-0.12396	0.005859	-0.02193	-0.0337
PDLIM5	-0.037054692	-0.001341817	-0.11603	-0.06563	0.159481	0.316363
PDLIM7	-0.033860784	-0.090106323	-0.17897	-0.01204	0.026992	0.233118
PDP1	0.026640529	-0.025426711	-0.11899	-0.06453	0.191417	0.317609
PDP2	-0.005090133	0.129963946	0.034572	0.106432	-0.32937	-0.16029
PDPK1	-0.018678105	0.038893502	-0.14736	-0.00709	0.049171	0.109654
PDPN	-0.068472192	-0.023074096	-0.1007	-0.03317	0.060973	0.00069
PDPR	0.063112634	0.120287095	0.048301	-0.0068	0.053647	-0.05615
PDRG1	0.039755338	0.022007959	0.086959	0.081187	-0.27109	-0.22655
PDS5A	-0.005843637	0.052712301	0.15032	0.051473	-0.13594	-0.09982
PDS5B	-0.0217344	0.085143262	-0.05774	0.010643	-0.06944	-0.02863
PDSS1	-0.000942985	-0.01535735	0.007674	0.007294	0.001644	0.24514
PDSS2	0.018874081	-0.005901976	0.050636	0.005746	-0.00486	0.181931
PDX1	-0.071684063	0.019503765	-0.01951	-0.03442	-0.01308	0.07941
PDXDC1	-0.005306222	0.005005546	0.126394	0.030933	-0.02816	-0.04338
PDXK	0.022386039	0.02605916	-0.23538	0.029946	-0.09388	0.453717
PDYN	-0.031020322	-0.036058762	-0.06693	0.027771	-0.05982	0.030981
PDZD11	0.026309062	0.100005974	0.127229	0.053854	-0.14156	0.0749
PDZD2	-0.026489585	0.056889817	0.121707	-0.03274	-0.06566	-0.11144
PDZD3	-0.030556101	-0.021923594	-0.01487	-0.00835	-0.06704	-0.08732
PDZD4	-0.008199514	0.285375726	0.063526	-0.04589	0.116946	-0.19637
PDZD7	-0.03663221	-0.053568014	-0.15776	0.017283	-0.04825	0.035114
PDZD8	0.004196069	0.070482356	-0.10005	0.045727	-0.10008	-0.028
PDZD9	-0.042264554	-0.058314775	-0.0317	0.019972	-0.05077	0.0595
PDZK1	-0.018275919	0.166897267	-0.00635	0.047412	-0.12747	-0.07877
PDZK1IP1	-0.013282474	-0.044673995	-0.09107	-0.25066	0.704758	0.091293
PDZRN3	-0.027019323	-0.069569282	-0.05259	-0.00022	0.007691	-0.12507
PDZRN3-AS	-0.036096322	-0.025877892	-0.07129	0.019825	-0.0396	0.01631
PDZRN4	-0.055523441	-0.056554313	0.102302	-0.01018	0.016807	0.092661
PEA15	0.021081017	-0.020842038	-0.26022	-0.0158	0.045499	0.378934
PEAK1	0.006088276	0.134343302	-0.09959	0.004309	-0.07349	-0.00991
PEAR1	-0.022371021	-0.045825328	0.003132	-0.22648	0.627568	0.045263
PEBP1	0.06339814	-0.037214908	0.306986	0.017471	-0.06183	-0.20874
PEBP4	-0.031163838	-0.111238434	-0.03986	-0.01596	0.069363	0.02648
PECAM1	0.011011694	-0.11714583	-0.13799	-0.09178	0.303263	0.276938
PECR	0.027581889	0.05999435	0.203059	0.011557	-0.04602	-0.05205
PEF1	0.066401153	0.128480816	-0.14497	0.027477	-0.09195	-0.03137
PEG10	-0.026524385	-0.075695315	-0.09267	-0.01351	0.040227	-0.07432
PEG3	-0.023577146	-0.076335282	-0.07907	-0.00973	0.043442	-0.1702
PEG3-AS1	-0.03344669	-0.055752611	0.137247	0.000336	0.017488	-0.03743
PELI1	0.013522945	-0.14792652	-0.26944	-0.02007	0.048834	0.220311
PELI2	0.00489283	0.017661725	-0.40007	-0.07885	0.253738	0.370023
PELI3	0.000872458	-0.132962963	-0.13926	-0.04947	0.126755	0.547305
PELO	-0.04890766	-0.024403465	-0.18118	0.001414	-0.06319	-0.00618
PELP1	0.020069671	-0.102602274	0.133223	0.052946	-0.17892	-0.14348
PEMT	0.016770009	-0.020783317	0.013318	0.056602	-0.13311	0.353733
PENK	-0.027046569	-0.049036398	-0.0567	0.003032	-0.0273	0.017296
PEPD	0.026889638	0.016684662	-0.03205	-0.05752	0.19469	0.46935
PER2	0.004656324	0.025351669	-0.08257	0.001989	0.009028	0.154255

PER3	-0.012055599	0.185302956	0.084616	-0.07527	0.028267	0.047015
PER4	-0.044655214	-0.069777874	0.053351	0.012528	-0.0286	-0.03542
PERM1	-0.021924459	-0.096373165	-0.00029	-0.02037	-0.00774	-0.09223
PERP	0.007569363	0.033190708	0.181535	-0.05493	0.074419	-0.09791
PES1	0.004402545	-0.065456751	-0.09526	0.032685	-0.11945	0.36085
PET100	0.104553637	0.032118908	-0.07783	0.117242	-0.26514	0.236467
PET112	0.020347523	-0.056542246	-0.05111	0.005704	-0.01442	0.159644
PET117	0.010908648	0.014583715	0.132671	0.031255	-0.0727	0.160048
PEX1	0.002684643	0.044028748	0.260274	0.017429	-0.01042	-0.24313
PEX10	-0.005330991	-0.119150855	0.034755	0.032828	-0.07976	0.145218
PEX11A	-0.018543056	0.241855599	0.031723	-0.04454	0.126156	-0.11531
PEX11B	0.038590934	0.095927429	0.043849	0.025148	-0.09219	-0.05491
PEX11G	-0.009635987	-0.094591921	-0.07663	0.010324	-0.11028	0.069686
PEX12	0.001175863	0.043881431	0.146387	0.04941	-0.17715	-0.1526
PEX13	-0.042936947	0.056877962	0.074244	0.061434	-0.05731	-0.0884
PEX14	0.019796947	0.053819615	0.140502	0.07377	-0.16108	0.074872
PEX16	0.022436663	-0.059290806	0.14845	0.019387	-0.12634	-0.02078
PEX19	0.037824879	0.020440371	0.029963	0.080011	-0.25706	-0.06381
PEX2	0.019269286	0.105608736	-0.10543	0.056348	-0.1544	0.143568
PEX26	-0.02742251	0.04674342	-0.04027	0.053912	-0.1407	0.102009
PEX3	0.018452699	0.050990535	0.163535	0.053601	-0.08345	-0.30229
PEX5	0.00428709	0.002101365	0.069027	-0.03673	0.078471	-0.09224
PEX5L	-0.033745191	-0.051415873	-0.02022	0.152717	-0.16559	-0.01396
PEX6	-0.002174784	-0.058609483	0.13755	0.024135	-0.06731	0.012661
PEX7	-0.006549084	-0.052741116	0.068273	0.074226	-0.22053	0.057666
PF4	0.005835293	-0.02139917	-0.04898	-0.16577	0.499234	0.121839
PF4V1	-0.011029404	-0.017194338	-0.02577	-0.21475	0.712836	0.079736
PFAS	0.010587247	0.002706404	0.1013	0.062156	-0.1664	0.002678
PFDN1	0.041286245	0.028959337	0.096925	0.037137	-0.12577	0.080073
PFDN2	0.053453442	0.018582883	-0.06191	0.109419	-0.30512	0.140829
PFDN4	-0.013591431	-0.0330692	-0.03176	0.10078	-0.23588	0.00077
PFDN5	0.065199637	0.011517003	0.170319	0.012229	-0.01086	0.181264
PFKFB1	-0.039166145	-0.052255187	0.092571	0.001882	0.010382	-0.10523
PFKFB2	0.003070451	-0.080875662	-0.24646	-0.03952	0.115178	-0.0893
PFKFB3	0.030537217	-0.045066268	-0.23634	0.046419	-0.11263	0.237106
PFKFB4	-0.005275793	-0.082837877	-0.23905	-0.01649	0.014501	0.251796
PFKL	0.02801558	-0.097297928	-0.09449	0.007447	-0.04874	0.17411
PFKM	0.005943505	-0.095766595	0.006001	-0.00143	-0.0399	0.007938
PFKP	0.014953491	0.023923346	0.172431	0.064035	-0.19597	-0.24349
PFN1	0.03477435	0.153935432	0.05042	0.090784	-0.16189	-0.06012
PFN2	-0.00366689	-0.0163617	0.259248	-0.03089	0.071202	-0.21819
PFN4	-0.037358543	0.004395391	-0.03552	0.018799	-0.05658	-0.04846
PGAM1	0.049208411	0.05378645	-0.16374	0.09288	-0.19925	0.270129
PGAM2	-0.030976775	-0.033205402	-0.00193	0.03303	-0.06361	0.123216
PGAM5	-0.028085922	-0.03961032	-0.12379	0.075593	-0.21985	-0.00891
PGAP1	-0.009707662	-0.011071278	0.134412	0.160508	-0.24603	-0.05034
PGAP2	0.006894777	-0.115303067	0.251248	0.04905	-0.20092	-0.27222
PGAP3	0.012199025	0.133478417	0.153242	-0.06602	0.15791	-0.09645
PGBD1	-0.018809366	-0.014761756	0.001406	0.043481	-0.12284	-0.14007
PGBD2	-0.005419014	0.01673786	-0.03135	0.027805	-0.0507	0.053141
PGBD4	-0.012930951	0.076984143	0.193741	0.0477	-0.10694	-0.22096

PGBD5	-0.025355728	-0.032717918	-0.01078	-0.01525	0.059294	0.157086
PGC	-0.046555388	-0.039759003	0.049672	-0.01518	0.026056	-0.03593
PGD	0.003039574	-0.107354753	-0.10261	-0.03865	-0.10565	0.15555
PGF	0.051026673	0.014340703	0.020815	0.050819	-0.09079	-0.22563
PGGT1B	0.050181299	-0.011515913	-0.30852	-0.0717	0.187422	0.196588
PGK1	0.075703095	-0.033583053	-0.22083	0.104059	-0.23927	0.295718
PGK2	-0.035561662	-0.030496165	-0.09374	0.029558	-0.05646	0.12731
PGLS	-0.00642051	-0.03438872	-0.22332	0.006034	0.056737	0.294024
PGLYRP1	-0.011811303	-0.094051452	-0.04143	-0.01236	-0.01722	-0.15115
PGLYRP2	-0.026608707	-0.054692005	0.033548	-0.00396	0.038378	0.076435
PGLYRP3	-0.043463432	-0.065802069	-0.11558	-0.01859	0.083414	0.08898
PGLYRP4	-0.023876314	-0.047399935	0.010151	0.059903	-0.20558	-0.04339
PGM1	0.048628136	0.166050167	-0.11046	0.047828	-0.11844	-0.00156
PGM2	0.017908013	0.018706813	-0.37561	0.0183	-0.06343	0.173009
PGM2L1	0.004576589	0.004550818	0.144088	-0.04188	0.156212	-0.20966
PGM3	-0.000983826	0.001780838	0.003865	0.062519	-0.10847	-0.02207
PGM5	-0.041794268	-0.067920227	-0.15902	-0.05216	0.112654	0.409593
PGM5-AS1	-0.024030349	-0.015255259	-0.08571	-0.0011	-0.03107	0.065388
PGP	0.011938514	-0.019683512	-0.04971	0.042777	-0.14894	0.225344
PGPEP1	0.001184856	0.056679266	0.111889	0.026729	-0.12768	0.116268
PGR	-0.06890466	-0.03378773	0.01256	-0.00244	0.024637	0.022081
PGRMC1	0.04401432	0.013143302	-0.12152	-0.08823	0.199648	0.320254
PGRMC2	0.030020221	0.083024141	0.255667	-0.00325	0.013624	-0.14489
PGS1	0.019436806	-0.059661569	-0.11545	-0.04466	0.085984	-0.02079
PHACTR1	-0.036098729	-0.161350352	-0.232	-0.06474	0.023794	-0.17575
PHACTR2	-0.014272576	0.048400375	0.182096	-0.02352	0.088096	0.241108
PHACTR3	-0.050880181	-0.092117005	-0.01195	-0.06903	0.231552	0.069055
PHACTR4	0.030178914	0.059098468	0.098057	-0.00164	0.035872	0.060045
PHAX	0.021227193	0.147457453	0.15755	0.013081	-0.09449	-0.0234
PHB	0.027690323	0.032743305	0.127748	0.072263	-0.20232	-0.03612
PHB2	0.063098127	0.019411264	0.178167	0.031866	-0.08175	-0.02925
PHC1	0.015706271	-0.017173067	0.211914	-0.05594	0.114225	-0.34647
PHC2	0.021174346	-0.055055951	-0.19076	-0.01594	0.058802	0.194959
PHC3	0.002770101	0.074198918	0.086318	0.054475	-0.11733	-0.10859
PHEX	-0.006790905	-0.070509464	-0.10537	-0.01031	0.045668	-0.14961
PHEX-AS1	-0.031830206	-0.033855308	-0.05056	0.011219	-0.01603	-0.02364
PHF1	0.024409246	-0.030073984	0.221935	-0.01951	0.052377	-0.45553
PHF10	0.028947804	-0.004703156	0.339152	0.027219	-0.07358	-0.2984
PHF11	0.041572087	0.067410917	0.104506	-0.02554	0.097761	-0.13815
PHF12	0.002457045	-0.063373844	-0.00363	-0.00594	0.034239	-0.15427
PHF13	0.020421345	0.072140194	0.018709	0.071231	-0.23322	-0.28239
PHF14	0.011875397	-0.004210984	0.101109	0.035609	-0.06698	0.037465
PHF19	0.002417227	-0.001707519	0.02661	0.072463	-0.17484	0.306482
PHF2	0.026103789	0.00058569	-0.03928	-0.00036	0.007395	-0.01759
PHF20	-0.008232896	-0.011870466	-0.08613	0.019653	-0.03761	0.046876
PHF20L1	0.06511533	-0.005212497	-0.27876	0.036739	-0.06609	0.160485
PHF21A	0.000579716	-0.01068506	-0.19743	-0.0144	-0.01947	0.12934
PHF21B	-0.048560562	-0.073297031	-0.06381	0.080722	-0.18248	-0.01279
PHF23	0.029438332	0.091528201	-0.10647	0.014238	-0.05858	-0.03646
PHF2P1	-0.043609702	7.37E-05	-0.04786	0.0149	-0.04673	0.047888
PHF3	0.010259078	0.027109536	0.038548	-0.01614	0.016066	-0.11006

PHF5A	0.044461312	0.049172361	-0.05508	0.064867	-0.21131	0.0598
PHF6	0.011616219	0.053537732	0.120396	0.016058	-0.05489	-0.09686
PHF7	-0.022030597	-0.185173952	-0.06639	0.063692	-0.00924	0.092622
PHF8	-0.033577276	-0.091022899	0.015441	0.00987	-0.02804	-0.05372
PHGDH	-0.004465077	-0.03153829	0.138173	-0.01782	-0.03179	-0.11971
PHGR1	-0.038225398	-0.012098491	-0.05447	0.023103	-0.03102	0.006877
PHIP	0.005891016	0.036152496	-0.17476	0.041819	-0.1155	0.086413
PHKA1	-0.034705593	-0.03975534	-0.03884	0.015518	-0.02115	-0.00762
PHKA2	-0.025573988	-0.146263959	-0.17322	-0.08596	0.126302	0.21926
PHKB	0.019190902	-0.024688892	-0.15623	-0.03212	0.130707	0.222761
PHKG1	-0.033635721	-0.095086997	-0.08674	-0.03122	0.043455	0.028925
PHKG2	-0.048350689	-0.040812595	-0.23305	0.00509	-0.00546	0.162475
PHLDA1	0.004403006	0.028639378	0.00285	0.074265	-0.31921	-0.01614
PHLDA2	0.010240108	0.076211158	0.031971	0.04986	0.045328	0.159011
PHLDA3	-0.021982142	-0.036062889	-0.04794	0.036566	-0.19975	-0.08111
PHLDB1	-0.026877671	-0.016197731	0.078858	-0.00296	-0.04293	-0.1015
PHLDB2	-0.031746826	0.287496333	0.002206	-0.00068	0.019155	-0.13926
PHLDB3	-0.00502349	-0.020221911	0.193835	-0.03703	0.024028	0.016547
PHLPP1	-0.021585328	-0.028517704	-0.07718	0.041906	-0.11135	-0.13304
PHLPP2	-0.002282701	0.003339085	-0.01328	0.031384	-0.09675	-0.23384
PHOSPHO1	-0.029240959	-0.091101647	-0.10246	-0.00765	0.02287	-0.02173
PHOSPHO2	0.001004825	0.042818136	0.151706	0.098249	-0.25631	-0.26156
PHOX2A	-0.029651012	-0.067322364	0.045008	0.02086	-0.04395	0.032646
PHOX2B	-0.020943935	-0.006858493	0.027752	-0.00943	0.001828	-0.12577
PHPT1	0.026944914	0.088286338	0.035216	0.08843	-0.23856	0.250594
PHRF1	0.002538126	0.047193614	0.09287	0.013692	-0.07	0.031461
PHTF1	0.003188654	0.10204606	-0.17288	0.024535	-0.12492	-0.06428
PHTF2	-0.022561184	-0.033600874	-0.04584	0.042708	-0.16192	-0.04747
PHYH	0.019835826	-0.038693364	0.143257	0.027516	-0.05427	0.246748
PHYHD1	-0.017199042	-0.024341987	-0.04428	-0.03293	0.102448	-0.15406
PHYHIP	-0.03858885	-0.054944328	0.058777	0.004923	-0.01523	-0.02967
PHYHIPL	-0.027290513	0.002352031	-0.00909	0.019122	-0.07123	0.041273
PHYKPL	0.095514366	-0.121221841	0.080603	-0.13147	0.094818	-0.10479
PI15	-0.040475843	-0.06556072	0.074401	0.024678	-0.05362	-0.03058
PI16	-0.014968383	-0.091558437	0.023662	-0.0256	-0.01737	-0.08324
PI3	-0.004557627	-0.04017424	-0.17888	0.000252	0.005739	-0.10337
PI4K2A	0.006610112	0.045473129	0.021589	0.005442	-0.08903	-0.00941
PI4KA	0.038714911	0.002094213	0.015698	-0.03148	0.094628	0.07378
PI4KB	0.029689422	-0.05629135	-0.06892	-0.0216	-0.06035	-0.10181
PIANP	-0.046824113	-0.111022343	0.048626	-0.02908	0.077959	-0.02558
PIAS1	0.036719617	0.052735274	-0.08591	-0.03349	0.168011	-0.00202
PIAS2	-0.000934089	0.12029644	0.082799	-0.04973	0.031448	-0.05045
PIAS3	0.016682206	0.067529679	0.017211	0.05749	-0.13627	0.047114
PIAS4	-0.004116568	0.076777668	-0.05002	0.024892	-0.07644	0.134526
PIBF1	0.007800745	0.006869585	0.206182	0.022619	-0.04477	-0.14716
PICALM	-0.003460119	0.042393755	-0.24906	-0.04497	0.107706	0.257221
PICK1	-0.001627953	-0.087487469	-0.03912	-0.03725	-0.00339	0.00164
PID1	0.038743517	-0.054805101	-0.3015	-0.06356	0.245713	0.560076
PIDD1	0.007950291	-0.040714389	0.140501	0.080728	-0.23708	-0.07946
PIEZO1	-0.064912149	-0.012161931	0.00829	-0.0141	-0.02051	0.094788
PIEZO2	-0.067568621	-0.066633076	0.029097	0.033527	-0.18383	0.064788

PIF1	-0.022274568	0.082145951	-0.05821	0.116011	-0.3389	-0.04923
PIF0	-0.032622759	-0.033883334	0.064714	0.00974	-0.01236	-0.11386
PIGA	-0.00041907	-0.077957293	0.028896	0.089842	-0.22782	0.09349
PIGB	0.019967459	0.022334999	-0.12372	0.033554	-0.05967	-0.04147
PIGC	0.003797098	0.060312945	0.161616	0.009723	-0.09216	-0.10687
PIGF	0.026102327	0.01484443	-0.02066	0.021662	-0.06118	0.168895
PIGG	-0.017905615	-0.051350849	0.196988	-0.01808	0.038489	-0.28052
PIGH	0.014416284	0.030712456	0.075544	-0.00313	0.070255	-0.05578
PIGK	0.02625293	0.080353724	0.150978	-0.00177	0.020481	-0.18224
PIGL	0.025848917	-0.005063465	0.317777	-0.0318	-0.00946	-0.10162
PIGM	-0.007108564	0.085861779	-0.00886	-0.03737	0.112742	0.256449
PIGN	0.010413237	0.00151347	-0.0676	0.013746	-0.06053	0.003751
PIGO	-0.089417169	0.03205547	0.025757	0.034443	-0.10208	-0.10588
PIGP	0.026774949	0.003223228	0.221378	0.033585	-0.07302	0.081324
PIGQ	-0.027689478	-0.102632695	-0.06633	0.012043	-0.12571	0.012953
PIGR	-0.030515012	-0.069743944	-0.01233	-0.0048	-0.01145	-0.06261
PIGS	0.048383835	0.045876015	-0.18793	-0.00342	-0.02811	0.093533
PIGT	0.041672483	0.048069271	-0.09179	0.036883	-0.14763	-0.0258
PIGU	0.008565565	0.038958199	0.150576	0.055828	-0.17033	-0.12381
PIGV	0.011114015	0.120258751	-0.00494	-0.0038	-0.04764	-0.06119
PIGW	0.009406225	0.045763844	0.048542	0.050414	-0.12804	0.010721
PIGX	0.013929463	0.035870359	-0.23732	0.056685	-0.14936	0.060533
PIGZ	-0.012408587	-0.037367983	-0.12416	-0.00665	0.032658	0.091318
PIH1D1	0.038894186	0.012283157	0.117649	0.049032	-0.13551	0.063302
PIH1D2	-0.057412661	-0.05252003	0.143752	0.035907	-0.126	-0.01794
PIH1D3	-0.036922203	-0.018447793	-0.08231	-0.00051	0.015407	0.000937
PIH2	-0.09570152	-0.101479644	0.015163	-0.03023	0.121415	0.044376
PIK3AP1	0.020549803	0.110628132	-0.48396	-0.05773	0.155985	0.185055
PIK3C2A	0.058643769	0.120277498	-0.02522	0.051811	0.040784	-0.01913
PIK3C2B	0.012960477	-0.000517882	0.168063	0.003306	-0.05754	-0.3586
PIK3C2G	-0.019207642	-0.031976918	-0.05448	0.0957	-0.22491	0.051547
PIK3C3	0.017236012	0.102447591	0.212822	-0.15659	0.17369	-0.06388
PIK3CA	-0.005391329	0.019667729	0.079043	-0.01147	0.054914	-0.13105
PIK3CB	0.010364059	-0.132978781	-0.1632	-0.00331	0.040489	0.324037
PIK3CD	0.036444387	0.027983527	0.008464	-0.00452	0.002105	-0.21674
PIK3CG	0.009163915	0.114743787	-0.1878	-0.0344	0.062319	-0.1182
PIK3IP1	0.023227152	-0.029616059	0.396506	-0.06843	0.196293	-0.20131
PIK3R1	0.037214211	0.172399018	0.255177	0.016906	-0.00336	-0.18899
PIK3R2	-0.050851046	-0.091970039	0.031956	-0.07615	-0.06602	-0.01772
PIK3R3	-0.013292397	0.165478229	0.049407	0.082599	-0.22403	-0.10395
PIK3R4	-0.053691374	-0.039139915	0.068457	-0.02371	0.049438	-0.14782
PIK3R5	0.015989104	0.069105529	0.046547	-0.07334	0.163789	-0.07656
PIK3R6	-0.010781761	-0.099858949	-0.17663	0.0104	-0.05252	0.112276
PIKFYVE	-0.052769219	-0.103805777	-0.04863	-0.00476	0.001636	-0.13889
PILRA	0.00949701	-0.094419596	-0.36716	-0.07605	0.226355	0.577032
PIM1	0.021747636	0.077680478	0.184946	0.032538	-0.1026	-0.25063
PIM2	0.01758366	-0.003084911	0.188251	0.015321	-0.07614	-0.59399
PIM3	0.03372252	-0.076753888	-0.13005	0.043144	-0.09967	0.058207
PIN1	0.02558826	0.06196785	0.019057	0.062648	-0.17386	0.161606
PIN1P1	-0.021895947	-0.055305801	-0.06843	0.061519	-0.16032	0.098331
PIN4	0.02728715	0.063963998	0.06809	0.010349	-0.02952	0.075913

PINK1	0.027598911	-0.010886095	-0.15186	-0.0342	0.093285	0.104631
PINK1-AS	-0.004627208	0.059823449	0.115054	0.003334	0.033788	-0.00241
PINLYP	-0.009787969	-0.036464533	0.006508	0.10169	-0.26907	0.022076
PINX1	0.001010791	-0.037913106	0.056098	0.02719	-0.16139	-0.06459
PIP	-0.031250798	-0.090419039	-0.05833	0.01701	-0.07728	0.109127
PIP4K2A	0.035514714	0.201321057	0.182462	-0.03025	0.112715	-0.2235
PIP4K2B	-0.003859656	-0.071516452	-0.06119	0.027358	-0.11955	0.013489
PIP4K2C	0.043088675	0.165001406	0.03425	0.004996	-0.0304	-0.36139
PIP5K1A	-0.031189048	-0.051563809	0.062967	0.03857	-0.25908	-0.02431
PIP5K1B	-0.011431405	-0.106873095	-0.22936	-0.06229	0.152677	-0.00203
PIP5K1C	0.038347152	0.04081305	-0.08361	-0.01407	-0.00998	0.177766
PIP5KL1	-0.028236925	-0.045583848	0.049556	0.021329	-0.06591	0.092305
PIPOX	-0.013331305	-0.027749455	0.011426	0.002499	0.002483	-0.04274
PIR	-0.00678514	0.00369754	-0.03178	0.028973	-0.11297	-0.00624
PIRT	-0.030996357	-0.04627369	0.002423	0.011877	-0.02879	-0.05574
PITHD1	-0.015595768	-0.003516661	0.000709	0.034876	-0.15526	-0.08891
PITPNA	0.089688728	0.073754344	-0.43413	0.057912	0.007795	0.210143
PITPNA-AS	0.01148601	-0.012610578	0.433512	0.019711	-0.05778	-0.28275
PITPNB	0.073780284	0.127938439	0.045126	0.089087	-0.22087	-0.27767
PITPNC1	0.037949916	0.17173741	0.220752	-0.07938	0.12057	-0.08901
PITPNM1	0.018426188	-0.100334249	-0.01313	-0.02398	0.009072	0.191393
PITPNM2	-0.059139682	0.19804865	0.216861	-0.12402	0.433028	-0.17568
PITPNM3	-0.062413235	0.005409027	-0.02858	0.00508	0.020729	0.055966
PITRM1	0.017525881	0.056016102	0.172036	0.006964	-0.03764	-0.00369
PITRM1-AS	-0.019479143	-0.003561583	0.113789	0.000423	0.002702	-0.06104
PITX1	-0.042527306	-0.019674433	0.001503	0.00733	0.001585	0.058359
PITX2	-0.121532485	-0.058103366	-0.06227	-0.00728	0.068938	-0.01587
PITX3	-0.024298227	-0.082567562	0.081923	0.00209	-0.02725	0.026553
PIWIL1	-0.047613133	-0.07592447	0.023612	-0.00779	0.024359	-0.04559
PIWIL2	-0.032795275	-0.079030525	0.107432	0.05221	-0.08073	-0.00028
PIWIL4	-0.008805559	-0.020260984	-0.0735	-0.05583	0.182591	0.450511
PJA1	0.023443153	0.123398567	0.377534	0.028494	-0.08177	-0.26036
PJA2	0.043136676	0.09882282	-0.13047	0.014179	-0.01488	-0.06297
PKD1L1	-0.114291361	-0.097588941	0.003143	0.001258	0.053349	-0.0189
PKD1L2	-0.046486526	-0.053759968	0.043125	0.030154	-0.03848	-0.17772
PKD1P1	0.01045599	0.020238144	0.216145	0.040556	-0.09241	-0.13464
PKD2	0.012776131	-0.026831053	0.005844	0.020622	-0.06382	0.121326
PKD2L1	-0.022512229	-0.103812095	0.018882	-0.01013	-0.05675	-0.0765
PKD2L2	-0.062159782	-0.009335231	-0.09016	-0.03536	0.108795	0.062115
PKDCC	-0.033235451	-0.079825582	0.117327	-0.00837	-0.02017	-0.06838
PKDREJ	-0.033980025	0.023485229	-0.00328	0.024858	-0.06169	-0.05874
PKHD1	-0.033526195	-0.157041588	-0.05733	0.150428	-0.07642	-0.06808
PKHD1L1	-0.011544033	-0.05607403	-0.17344	-0.07951	0.246757	-0.15758
PKI55	-0.010503562	0.040770637	0.215775	0.001263	0.017218	-0.15088
PKIA	-0.006120986	-0.09671466	0.213842	-0.04737	0.11343	-0.24119
PKIB	-0.015327788	-0.035309691	-0.04473	0.031171	-0.07406	-0.03256
PKIG	-0.001539022	-0.118856544	-0.05783	-0.05278	0.146069	-0.1563
PKLR	-0.046126048	-0.065501079	-0.14194	-0.00581	-0.04187	0.019717
PKM	0.012395444	-0.086279624	-0.03431	0.036418	-0.11605	0.311325
PKMYT1	-0.005069659	-0.026937934	0.050697	0.042459	-0.24653	-0.0917
PKN1	0.018169711	0.056688884	0.061324	0.012944	-0.02689	0.195479

PKN2	0.003109294	-0.043424884	-0.25134	-0.02601	0.119438	0.076088
PKN3	-0.01671058	0.014704585	0.011094	0.01062	-0.04748	-0.1134
PKNOX1	0.008685595	0.06644558	0.106662	0.00718	-0.07276	-0.09425
PKNOX2	-0.030355786	-0.048855944	-0.02326	0.029902	-0.09689	-0.0577
PKP1	-0.038519669	-0.060214134	-0.02946	0.02916	-0.0957	-0.02423
PKP2	-0.020031237	-0.069321042	-0.07217	-0.03447	0.149886	0.028565
PKP3	-0.033484327	-0.097274697	-0.06565	-0.01091	-0.07094	0.006202
PKP4	-0.012724333	-0.044088803	0.02811	0.103289	-0.28557	0.043564
PLA1A	-0.021083334	-0.029705006	-0.03264	0.028684	-0.07639	-0.0073
PLA2G12A	-0.005507933	0.057156627	0.238586	-0.1365	0.338062	-0.10669
PLA2G12B	-0.047900151	-0.097985358	-0.0231	0.021148	-0.05475	-0.02433
PLA2G15	0.009839673	-0.108378532	-0.16157	-0.00818	0.046696	0.489522
PLA2G16	0.013090296	0.173745611	0.090965	0.000343	0.012777	0.160971
PLA2G1B	-0.045742832	-0.016637724	-0.0318	-0.0184	0.068406	0.040255
PLA2G2A	-0.037747083	-0.04529936	-0.00599	-0.0092	-0.01528	-0.13324
PLA2G2D	-0.027525648	-0.051136972	-0.02523	-0.00205	-0.044	-0.19579
PLA2G2E	-0.030225041	-0.002494562	-0.09363	0.045872	-0.09318	0.070352
PLA2G2F	-0.029621133	-0.028010297	-0.07472	0.014051	-0.01438	0.132708
PLA2G3	-0.028600005	-0.06761414	-0.09568	0.013231	-0.03416	0.045726
PLA2G4A	-0.01373411	-0.086804411	-0.16876	-0.02906	0.063764	0.295181
PLA2G4C	-0.021179053	-0.104815925	-0.19835	-0.0388	0.132764	0.151871
PLA2G4D	-0.040280246	-0.003704697	-0.03758	########	0.017969	-0.06575
PLA2G4F	-0.021260259	-0.105193217	0.028526	-0.01205	-0.02601	-0.04081
PLA2G5	0.041166462	-0.01670586	-0.11241	0.00641	-0.01376	-0.01715
PLA2G6	0.029083674	0.045768728	0.186783	0.031541	-0.04163	-0.32801
PLA2G7	0.006926439	-0.064553201	-0.21375	-0.08078	0.240087	0.736089
PLA2R1	-0.08724789	-0.001601346	-0.04299	-0.04603	0.066278	0.041779
PLAA	-0.029831129	0.041737856	-0.03171	0.032815	-0.12631	0.062198
PLAC1	-0.023112527	-0.044952955	-0.044	0.071417	-0.22237	-0.06821
PLAC4	-0.024469683	-0.099966056	0.028589	0.102797	-0.07467	0.087811
PLAC8	0.022552155	0.126229397	0.084652	-0.07837	0.227679	-0.04106
PLAC8L1	-0.030086952	-0.000752351	0.058507	0.009283	-0.0227	-0.13478
PLAC9	-0.053459992	-0.082078881	-0.11043	-0.0006	-0.0163	0.074758
PLAG1	-0.008848249	-0.088276279	0.338589	-0.04762	0.158216	-0.29408
PLAGL1	0.008220446	-0.113694574	-0.36263	-0.08695	0.252733	0.289239
PLAGL2	0.005158606	-0.024864051	-0.15629	0.003295	-0.02543	0.347829
PLAT	-0.023706807	-0.029227599	-0.02166	-0.02656	-0.00259	-0.03509
PLAU	-0.010646159	-0.005932505	-0.0603	0.001475	-0.05912	-0.06779
PLAUR	0.010429122	-0.102341309	-0.32563	-0.05773	0.13991	0.352833
PLB1	-0.027280997	-0.095169663	-0.2295	-0.04945	0.066006	0.198004
PLBD1	0.008707736	-0.122236154	-0.25265	-0.09802	0.304783	0.504296
PLBD2	0.021057196	-0.140369006	-0.10178	0.011559	-0.0623	0.493307
PLCB1	0.017716424	0.094932048	0.003646	-0.00609	0.097521	0.204525
PLCB2	0.014756639	-0.03151859	-0.10137	-0.03952	0.102209	0.080639
PLCB3	-0.005283551	-0.141371133	-0.14344	-0.02643	-0.01547	0.043063
PLCB4	-0.014107137	-0.063789882	-0.06673	0.008451	-0.00789	-0.1567
PLCD1	0.020313823	-0.044073296	0.167252	-0.02544	0.020897	-0.2836
PLCD3	-0.010289099	-0.049939798	-0.03423	0.018614	-0.13382	-0.05965
PLCD4	-0.027359232	-0.001922484	0.067188	-0.06365	0.186605	0.039354
PLCE1	-0.05505373	-0.110371589	0.013869	0.021127	-0.07158	0.02229
PLCG1	0.009034164	0.005712843	0.501192	-0.01536	-0.00643	-0.32365

PLCG1-AS	-0.024245663	-0.014231632	0.440742	-0.01739	0.062412	-0.12072
PLCG2	-0.015740102	0.095014265	-0.43874	-0.00075	0.05861	-0.02604
PLCH1	-0.040807105	-0.001408028	-0.00102	-0.03598	0.113065	-0.16221
PLCH1-AS	-0.028779853	-0.036522768	0.068877	0.008546	-0.01566	-0.07106
PLCH2	-0.005856733	0.104203084	0.019856	0.014934	-0.03426	-0.18503
PLCL1	-0.011357999	-0.091573216	0.099114	-0.07016	0.199108	0.009177
PLCL2	0.013042311	0.119618788	0.124098	-0.05422	0.168874	-0.17312
PLCXD1	0.011901598	-0.048166004	0.15186	0.026779	-0.07189	-0.0058
PLCXD2	-0.001456961	0.211027558	0.301667	0.00991	-0.03529	-0.48871
PLCXD3	-0.020515212	-0.016648511	-0.02257	0.134707	-0.19569	-0.02701
PLCZ1	-0.046552804	-0.030471809	-0.08868	-0.01254	0.052198	0.10606
PLD1	0.008423097	0.013687638	-0.18488	-0.05089	0.187231	0.35919
PLD2	0.001669319	-0.147395064	-0.16158	-0.0132	-0.04863	0.208676
PLD3	0.005834663	-0.053964963	-0.0864	-0.02386	0.070901	0.456134
PLD4	-0.007407964	-0.117619914	-0.17532	-0.05631	0.168427	-0.11725
PLD5	-0.033433332	-0.033282815	-0.056	0.118205	-0.10153	-0.01497
PLD6	-0.028970664	-0.041942039	0.065981	0.013557	-0.00871	-0.00273
PLEC	0.009523955	-0.124400494	-0.04765	-0.06196	0.171167	0.195665
PLEK	0.034022709	0.133289815	-0.19465	-0.05087	0.129653	-0.00749
PLEK2	-0.015003168	-0.032121567	0.010829	2.23E-05	-0.09436	-0.08672
PLEKHA1	0.012493771	0.168846587	0.316303	-0.10253	0.284299	-0.25583
PLEKHA2	0.012131392	0.060074462	-0.22609	-0.02741	0.050115	-0.03491
PLEKHA3	0.004029919	0.124768177	0.181273	0.031584	-0.11663	-0.2947
PLEKHA4	-0.028076457	-0.067216629	0.07124	0.021104	-0.0378	0.072123
PLEKHA5	0.003805385	0.018799394	0.137394	-0.00365	0.025472	-0.12806
PLEKHA6	-0.0419043	-0.040446512	-0.04607	-0.00105	0.00332	-0.1354
PLEKHA7	-0.015636238	-0.076171373	0.047056	-0.03902	0.116646	-0.12216
PLEKHA8	-0.040261094	-0.036272594	-0.11262	0.075644	-0.1564	0.080979
PLEKHA8P	0.010167037	0.094337463	0.036756	-0.05748	0.154218	-0.02765
PLEKHB1	-0.038323889	-0.051099283	0.285883	0.033624	0.164659	-0.2201
PLEKHB2	0.047296781	0.095003652	-0.23128	-0.01397	0.029774	0.474074
PLEKHF1	0.013229344	0.279809596	0.214086	0.010689	-0.06743	-0.27277
PLEKHF2	0.026825422	-0.089876198	-0.20258	-0.07095	0.181476	-0.11648
PLEKHG1	-0.010125153	-0.062230498	-0.03966	-0.00845	0.026609	-0.14923
PLEKHG2	-0.006100754	-0.021288131	0.012097	-0.01553	-0.02406	-0.09895
PLEKHG3	-0.031731691	0.132488399	-0.06171	-0.07964	0.106463	-0.2026
PLEKHG4	-0.002911456	-0.045100724	0.411633	0.017899	-0.06222	-0.26513
PLEKHG4B	-0.020402284	-0.092424714	-0.19084	0.000655	0.019348	-0.1167
PLEKHG5	-0.024940667	-0.112857001	0.050816	-0.00017	-0.07713	0.05312
PLEKHG6	-0.035921907	-0.077048277	0.089098	0.045519	-0.11174	-0.01057
PLEKHH1	-0.022700442	-0.022021247	0.015708	-0.01593	0.065527	-0.03366
PLEKHH2	-0.059375845	-0.067835057	0.269218	0.03063	0.082816	-0.25893
PLEKHH3	-0.040337663	-0.051590473	-0.01565	-0.00289	-0.0903	-0.03314
PLEKHM1	0.033079438	0.049742861	-0.1607	-0.03247	0.095089	0.161611
PLEKHM2	0.023349854	-0.138799775	-0.03648	0.001592	-0.02782	0.338894
PLEKHM3	0.008877783	0.020830879	-0.01561	-0.01251	0.064731	-0.03852
PLEKHN1	-0.00711079	-0.083769537	-0.00386	0.001696	-0.1079	-0.1452
PLEKHO1	0.031115827	-0.101673777	-0.24497	-0.03035	0.110668	0.361806
PLEKHO2	0.037697393	0.078651312	-0.32593	-0.01606	0.051568	0.235436
PLEKHS1	-0.086330991	-0.023799436	0.009816	-0.03014	0.05506	-0.03322
PLG	-0.078623361	0.181586154	0.067832	0.063958	-0.07902	-0.07207

PLGLB2	-0.019731501	0.021887893	0.023071	0.010482	-0.02088	-0.17059
PLGRKT	0.017037948	0.047289329	0.220901	0.05201	-0.19066	-0.2082
PLIN1	-0.025480936	0.027630255	-0.01462	-0.02088	-0.01805	-0.0603
PLIN2	0.018778486	-0.027816027	0.062178	0.008336	-0.02777	0.235791
PLIN3	0.038680908	-0.116671611	-0.2822	-0.00487	0.023366	0.401237
PLIN4	-0.026735382	-0.07350367	-0.11396	-0.00537	-0.03513	-0.06292
PLIN5	-0.005964936	-0.052586611	-0.11892	0.013457	-0.05002	-0.17693
PLK1	-0.015783065	-0.040843605	-0.00497	0.178235	-0.46551	-0.00764
PLK2	-0.014688114	-0.054985553	-0.05761	-0.02052	0.04583	-0.1354
PLK3	0.019190148	-0.114715267	0.043366	0.024463	-0.0513	-0.03697
PLK4	-0.03328058	0.034101493	-0.02817	0.233154	-0.28148	-0.07459
PLK5	-0.034429882	-0.06856477	-0.07556	-0.01368	-0.01371	0.041375
PLLP	-0.014589569	-0.074574686	0.141291	-0.02078	0.072238	0.008081
PLN	-0.051228555	0.013704838	0.012661	-0.00884	0.0201	0.128843
PLOD1	0.017633165	0.097766661	-0.2202	-0.00788	0.016067	0.401996
PLOD2	-0.02981433	-0.057862283	-0.04132	-0.0468	0.216724	-0.0119
PLOD3	0.036596912	-0.008077909	-0.07138	-0.00162	-0.00052	0.335876
PLP1	-0.035873965	0.030631456	0.000315	0.009477	-0.01261	0.062383
PLP2	0.047477923	-0.247020168	-0.09801	0.017329	-0.0256	0.263405
PLRG1	0.030777916	0.064514589	0.099559	0.074186	-0.17763	-0.06292
PLS1	-0.015722673	0.144408481	0.198519	0.105569	-0.29931	-0.2142
PLS3	-0.011731314	-0.072433176	-0.05529	0.018029	-0.04297	-0.1331
PLSCR1	0.01513368	0.028890503	-0.35902	-0.02176	0.066094	0.451871
PLSCR2	-0.072093549	-0.052422933	-0.00012	-0.02965	0.097405	0.090029
PLSCR4	-0.023475987	-0.030229857	0.023229	-0.02106	0.014192	0.009771
PLTP	-0.009774625	-0.017360032	-0.13467	0.023528	-0.08887	0.004632
PLVAP	-0.002290761	-0.058580516	-0.07883	-0.00889	0.001537	-0.09927
PLXDC1	0.002811885	0.058371651	0.431567	-0.03727	0.137436	-0.31634
PLXDC2	-0.001605269	-0.034742238	-0.32166	-0.04997	0.154494	0.566895
PLXNA1	0.018350919	-0.041804774	0.080414	0.075544	-0.15006	-0.12072
PLXNA2	-0.0398706	0.005061001	-0.04846	-0.02289	0.024649	0.106243
PLXNA3	-0.056693322	-0.092258782	0.076694	0.00337	-0.01716	-0.01468
PLXNA4	-0.030181775	0.059515583	-0.11516	-0.02251	0.009819	-0.12948
PLXNB1	-0.032996013	-0.034067084	-0.0901	-0.0087	-0.03935	0.040379
PLXNB2	0.004017163	-0.152125249	-0.23681	-0.08051	0.234336	0.52977
PLXNB3	-0.037990072	-0.025285964	-0.01567	0.036504	-0.07898	-0.01828
PLXNC1	-0.026577408	-0.120662433	-0.30006	-0.07853	0.159635	0.323349
PLXND1	-0.015143622	0.066847365	-0.15873	-0.07268	0.257431	0.551943
PM20D1	-0.032798631	0.002291454	0.248803	0.004048	-0.00032	-0.14205
PM20D2	0.051087176	0.039958798	0.246513	0.083084	-0.11562	-0.33793
PMAIP1	0.017358713	0.032970203	-0.20763	0.058519	-0.15852	-0.03009
PMCH	-0.009537336	-0.016820957	0.001083	0.095089	-0.20877	-0.02376
PMCHL1	-0.056793607	-0.100120074	0.03374	0.020228	-0.00666	-0.11905
PMCHL2	-0.047752744	-0.01850001	0.159936	-0.02055	0.06625	-0.14101
PMEL	-0.026944373	-0.008671598	0.056205	0.019945	-0.08962	-0.04975
PMEPA1	-0.0108032	-0.058974078	-0.11367	0.006882	0.043392	-0.17276
PMF1	0.038207109	-0.035957922	-0.19281	0.089313	-0.26701	0.243138
PMFBP1	-0.027722802	-0.086553979	0.032419	-0.00467	-0.00516	0.011948
PML	0.016812022	-0.008397105	-0.01773	0.051055	-0.07372	0.031646
PMM1	0.008913431	-0.058524306	0.104415	0.043244	-0.13761	0.022708
PMM2	0.018939452	0.002204556	-0.00081	0.018254	-0.04485	0.163894

PMP2	-0.101169127	-0.145359076	-0.13678	0.001766	0.012402	0.016823
PMP22	-0.073670773	-0.058019396	-0.1764	0.102803	0.009332	-0.13336
PMPCA	0.037448038	0.02116747	0.136029	0.048943	-0.15468	0.085644
PMPCB	0.053464298	0.002204327	0.20265	0.073885	-0.205	-0.15111
PMS1	-0.015567304	0.03189424	0.003102	0.046543	-0.01456	0.223812
PMS2L2	-0.009223905	-0.011917907	0.170083	0.047158	-0.11504	-0.24615
PMS2P1	0.036475921	-0.022087308	0.242563	0.063746	-0.26421	-0.40417
PMS2P3	0.031798037	0.00627769	0.280733	0.064905	-0.20045	-0.3249
PMS2P4	-0.027681922	0.112309819	0.012368	0.011467	-0.02725	-0.13005
PMS2P5	-0.000475417	0.038002552	0.201233	0.01473	-0.03283	-0.16198
PMS2P8	-0.001305381	-0.021111153	0.152965	0.048989	-0.17942	-0.3423
PMVK	0.022840241	-0.029028017	0.056995	0.091997	-0.26872	0.164388
PNCK	-0.029676648	-0.043599322	-0.01919	-0.02062	-0.02967	0.044472
PNISR	0.046775534	-0.004727788	0.233429	-0.06101	0.136193	-0.48945
PNKD	0.004668756	-0.073963594	-0.23852	-0.11708	0.198704	0.675664
PNKP	0.02046957	0.045352212	-0.13285	0.061922	-0.18316	0.213934
PNLDC1	-0.044056611	-0.042331824	0.067319	-0.00443	0.044309	-0.06526
PNLIP	-0.041521763	-0.112041356	0.004166	0.011465	-0.01611	0.078982
PNLIPRP1	-0.03943309	-0.033166555	0.164208	0.011405	-0.03325	-0.06879
PNLIPRP2	-0.061540858	0.001675228	0.061492	-0.00012	-0.00211	0.029197
PNLIPRP3	-0.033688154	-0.05668353	-0.00422	0.059446	-0.15476	0.125535
PNMA1	0.02173934	0.044853472	0.347852	0.008438	-0.04793	-0.3923
PNMA2	-0.043779903	-0.046693796	-0.05464	0.020671	-0.03636	-0.04244
PNMA3	-0.011784365	-0.079768376	0.110813	-0.00558	-0.03691	-0.1056
PNMA6A	-0.018183346	-0.073017008	-0.06832	-0.01114	0.009201	-0.12622
PNMAL1	-0.017912286	-0.04341772	0.175876	0.002457	-0.00759	-0.05633
PNMAL2	-0.03133988	-0.10087279	-0.02291	-0.01271	-0.03422	-0.08591
PNMT	-0.023870511	-0.062030956	0.055652	0.011761	-0.13043	-0.12886
PNN	0.062861504	0.073060627	0.108043	0.037947	-0.08733	-0.32745
PNO1	0.048098276	0.012233318	0.015267	0.099225	-0.13434	-0.08404
PNOC	-0.00418909	-0.097050804	-0.16035	-0.02287	0.064365	-0.04226
PNP	0.027580453	0.077742408	0.115975	0.028101	-0.07051	0.112625
PNPLA1	-0.039886224	-0.08057711	-0.14741	-0.01725	0.07212	0.389764
PNPLA2	0.051048177	-0.108792293	0.047298	0.010084	-0.01447	-0.00773
PNPLA3	-0.04555577	-0.024172492	0.060946	0.008636	-0.00304	-0.01939
PNPLA4	-0.007279998	-0.068763213	0.048295	-0.09117	0.237141	0.052112
PNPLA5	-0.035235006	0.011126732	-0.0638	0.017603	-0.02264	0.143324
PNPLA6	0.021730982	-0.056281847	-0.24843	0.005107	-0.01094	0.273035
PNPLA7	-0.035653363	-0.06297104	0.089582	-0.00783	0.045927	-0.22322
PNPLA8	0.066668334	0.022334144	-0.33842	-0.01002	0.033162	0.269458
PNPO	0.022358796	-0.056371896	0.074953	0.052702	-0.1608	0.266901
PNPT1	0.01964796	0.022986601	0.095826	0.039027	-0.11866	-0.09177
PNRC1	0.060495641	0.040358062	-0.02455	-0.04339	0.154175	-0.28355
PNRC2	0.066555706	0.176000437	0.149893	0.003056	-0.00389	-0.16011
POC1A	-0.015266811	-0.03061604	-0.10423	0.105439	-0.36142	0.037516
POC1B	0.029446046	0.150195516	-0.09984	0.043949	-0.16615	0.038992
POC5	0.015658886	0.05649591	0.0212	0.026788	-0.09343	-0.22541
PODN	-0.038783708	0.048291929	-0.00195	0.007947	-0.02591	-0.03765
PODNL1	0.011846384	-0.088513679	0.045161	-0.02173	-0.02459	-0.12487
PODXL	-0.013956199	0.033865394	0.062625	0.03827	-0.10715	-0.02621
PODXL2	-0.024716739	-0.045252757	-0.04928	0.011531	-0.00808	-0.02111

POF1B	-0.041098788	-0.033157486	-0.04093	0.155036	-0.09856	-0.01195
POFUT1	-0.005834081	-0.042427967	-0.03875	0.026796	-0.09617	0.070535
POFUT2	-0.033813417	-0.118717948	-0.0257	-0.02005	-0.02797	-0.09653
POGK	0.029794575	-0.03892988	-0.01981	0.030274	-0.08048	0.294688
POGLUT1	0.024348168	0.058086098	0.196173	-0.00564	0.003369	-0.00684
POGZ	0.044209993	0.073551337	-0.01343	0.043954	-0.09968	-0.30407
POLA1	0.00898885	0.061628983	0.031205	0.094153	-0.24472	-0.11225
POLA2	-0.031683357	-0.03187101	0.007546	0.073208	-0.20017	-0.17087
POLB	0.021694953	0.010464891	-0.15251	-0.00798	0.021112	-0.06776
POLD1	0.005330375	-0.043058584	0.028551	0.107653	-0.29419	0.037854
POLD2	0.010606421	-0.043523123	0.119945	0.065503	-0.20495	-0.11065
POLD3	0.011706931	-0.037403095	-0.21608	0.039001	-0.16906	0.312734
POLD4	0.029707965	-0.109142774	-0.05725	-0.02828	0.039527	0.106719
POLDIP2	0.049922656	-0.055819009	-0.0399	0.138092	-0.44047	-0.02389
POLDIP3	-0.01343636	-0.053934879	-0.05363	-0.01282	-0.03045	0.062739
POLE	0.004967809	0.007322132	-0.01786	0.064249	-0.22312	-0.10847
POLE2	-0.009802068	0.098762789	0.047235	0.156083	-0.37957	-0.12859
POLE3	0.038706282	0.067859151	0.094029	0.032478	-0.09033	-0.01648
POLE4	0.024189575	-0.037894382	-0.03464	0.036571	-0.07861	0.516822
POLG	0.024144169	0.109536767	0.068853	0.013506	-0.05871	-0.13312
POLG2	0.02331048	-0.007317482	0.258758	-0.00155	-0.06175	-0.26767
POLH	-0.026441465	0.03628109	0.04343	0.10662	-0.1814	-0.14759
POLI	0.001043249	-0.026850846	-0.0165	-0.02226	0.081155	0.102959
POLK	0.038542166	-0.017373241	-0.079	-0.01037	0.088633	0.256439
POLL	0.010729136	-0.108889482	0.089026	-0.04579	-0.00283	0.029782
POLM	-0.000291963	-0.055676045	0.056958	0.008001	-0.01809	-0.13713
POLN	-0.036235068	-0.075416972	0.018589	-0.00292	-0.05075	-0.0865
POLQ	-0.012685561	0.010155185	0.036007	0.115219	-0.34689	-0.04323
POLR1A	-0.013823504	0.045388912	0.039854	0.061873	-0.21809	-0.03998
POLR1B	0.024491067	0.079314255	0.111504	0.051228	-0.12579	-0.07547
POLR1C	0.010378684	-0.007380789	0.103215	0.038745	-0.11259	-0.02617
POLR1D	0.02364646	-0.034219558	-0.00047	0.009257	-0.06445	0.340945
POLR1E	-0.048167035	0.00369629	0.087786	0.045919	-0.19851	-0.15493
POLR2A	-0.045602999	-0.009794007	0.05646	-0.02676	0.026463	-0.0707
POLR2B	0.12984983	0.275178986	0.075205	0.081978	-0.15791	-0.41668
POLR2C	0.101652019	0.026914549	0.118668	0.084801	-0.17033	-0.28507
POLR2D	0.010189637	0.014475717	0.053554	0.071491	-0.21628	0.006546
POLR2E	0.035601142	0.022801218	-0.10498	0.037729	-0.09756	0.372613
POLR2F	0.030512186	0.010910179	0.036512	0.079817	-0.23064	0.059453
POLR2G	0.088177943	0.196370306	0.049228	0.07061	-0.17206	-0.02664
POLR2H	0.032335021	0.046124352	0.226599	0.084681	-0.23992	-0.08285
POLR2I	0.036919717	-0.000487121	0.203591	0.093586	-0.27375	0.002975
POLR2J	0.083407455	0.004367037	0.053054	0.069894	-0.1602	0.185111
POLR2J2	0.006229022	-0.134064189	0.067057	-0.02143	-0.0193	-0.05809
POLR2J4	-0.039640491	-0.008532081	0.170797	0.019386	-0.10106	-0.14268
POLR2K	0.036356981	0.117908708	0.119737	0.059392	-0.17569	0.064763
POLR2L	0.028519932	0.075014922	0.045944	0.070811	-0.19528	0.291393
POLR3A	0.041992606	0.091088719	0.125656	0.072835	-0.10238	0.055814
POLR3B	-0.040486389	-0.057957712	-0.05804	-0.03023	0.091479	0.184621
POLR3C	0.013259495	-0.00815473	-0.01989	0.032777	-0.10216	0.090377
POLR3D	0.001000335	-0.003745435	0.073786	0.070281	-0.26452	-0.12416

POLR3E	-0.034291602	-0.058784414	-0.01598	0.010139	-0.04257	-0.04582
POLR3F	0.01593806	0.037117665	-0.00725	0.058329	-0.08889	0.140375
POLR3G	0.041531267	0.031000816	-0.03723	0.05801	-0.3977	-0.11416
POLR3GL	0.027192693	0.080892696	0.180065	0.004356	-0.01747	-0.14059
POLR3H	0.061637568	-0.021431451	0.034935	-0.01554	-0.17726	-0.00588
POLR3K	0.011746593	0.073351683	-0.01875	0.113258	-0.32311	-0.11165
POLRMT	-0.018263733	-0.043084795	0.090326	0.065058	-0.11021	0.149428
POM121	-0.007548713	-0.075800232	0.145077	-0.06112	0.129845	-0.14052
POM121C	0.046450641	0.124417279	0.149414	0.055096	-0.16869	-0.30512
POM121L1(	-0.029634068	-0.081813946	-0.02305	0.00751	-0.0036	0.031149
POM121L1:	-0.001925263	-0.064021322	0.01218	-0.00643	-0.06518	-0.1016
POM121L2	-0.033987039	-0.001330104	0.047951	-0.02484	0.043742	-0.062
POM121L8I	-0.018547904	-0.034148358	0.122853	-0.00318	-0.00215	-0.14272
POM121L9I	-0.026906316	-0.050440086	0.14202	0.01798	-0.0534	-0.20022
POMC	-0.013532627	-0.005006843	-0.08234	0.013098	-0.01841	0.068436
POMGNT1	-0.021719549	-0.020050263	0.054312	0.061545	-0.19475	0.080052
POMGNT2	-0.00215267	0.109133841	0.020077	-0.00283	0.017405	0.094112
POMK	-0.024104277	-0.029746012	0.018797	-0.00405	-0.04234	-0.06373
POMP	0.047214456	0.100977241	-0.26748	0.133834	-0.35308	0.205254
POMT1	0.007933018	-0.015584517	0.165632	0.023727	-0.04535	-0.09795
POMT2	-0.013692289	-0.12919086	0.04736	-0.01748	-0.05223	-0.11156
POMZP3	0.001543333	-0.027670028	0.035346	0.023227	-0.02087	0.245412
PON1	-0.043562937	-0.032485302	-0.11904	0.035435	-0.08059	-0.00943
PON2	-0.010706805	0.04230589	-0.10706	0.028363	0.051445	0.087488
PON3	-0.039534937	-0.087193219	0.086755	0.023497	-0.04515	0.037795
POP1	-0.012359078	0.08335354	0.022363	0.050967	-0.19349	-0.14272
POP4	0.046343989	0.178772181	0.136729	0.060599	-0.22673	0.098354
POP5	0.024631184	0.056559909	0.210129	0.015232	-0.02031	0.0736
POP7	0.031173746	0.138909457	-0.06409	0.098625	-0.28836	0.281909
POPDC2	-0.019977785	-0.026011988	0.079223	-0.01849	0.060774	-0.16218
POPDC3	-0.019093089	-0.024784175	0.009646	0.09791	-0.22096	0.004984
POR	0.01022837	-0.042230485	-0.26455	0.004447	-0.01452	0.308805
PORCN	0.007546968	0.082841398	0.115819	0.083221	-0.27236	-0.00295
POSTN	-0.106481187	-0.061746555	0.123176	-0.00499	-0.02311	-0.04099
POT1	0.011968651	0.024159945	0.095407	0.033477	-0.1087	-0.1035
POTEKP	-0.001830226	-0.01571333	-0.10146	-0.00498	-0.05368	0.105106
POTEM	-0.018072348	-0.018219178	0.146788	-0.00181	-0.05342	-0.14884
POU1F1	-0.098136663	-0.09123657	-0.0587	-0.00627	0.029846	-0.02853
POU2F1	0.031122974	-0.033120227	-0.0869	0.031841	-0.12217	-0.13708
POU2F2	0.018518425	-0.106491801	-0.17049	-0.09721	0.198624	0.328309
POU2F3	-0.02795893	-0.027225365	-0.01164	0.17632	-0.14015	-0.02719
POU3F1	-0.03301133	-0.076980813	0.008379	-0.02233	0.083802	0.126799
POU3F2	-0.035933036	-0.011419995	-0.03017	0.021127	-0.04078	-0.0735
POU3F3	-0.036424925	0.022298959	-0.07213	0.031955	-0.00355	0.010787
POU3F4	-0.032345773	-0.088183461	-0.18715	0.026312	-0.04993	0.079482
POU4F1	-0.017905437	-0.062149479	-0.10723	0.015728	-0.02396	-0.19151
POU4F2	-0.022304238	-0.003082566	-0.02329	-0.01273	-0.00991	-0.06149
POU4F3	-0.034883407	-0.077751701	-0.05829	0.054614	-0.14781	0.093291
POU5F1B	-0.005067708	-0.047666584	0.12324	-0.00627	-0.10754	-0.23397
POU5F1P3	-0.032526547	-0.049853757	0.198784	0.018507	-0.0432	-0.13176
POU5F1P4	-0.037518512	-0.009436804	0.230246	0.012604	-0.04312	-0.12323

POU5F2	-0.028263597	-0.047288503	0.010461	-0.00838	0.026918	-0.01855
POU6F1	0.014713074	-0.130132637	0.225421	-0.12538	0.090597	-0.0837
POU6F2	-0.048795034	0.013139124	0.052875	0.002332	0.017572	0.016588
POU6F2-AS	-0.095856162	-0.115253714	0.013757	-0.0238	0.088841	0.052527
PP12613	-0.037805353	-0.035447137	-0.12714	-0.00163	0.024018	-0.13597
PP12708	-0.034569451	0.099626817	0.086931	-0.0191	0.037309	-0.09651
PP12719	-0.012157264	0.015960665	-0.05567	-0.0074	0.027598	-0.06437
PP13	-0.015016338	-0.108452316	0.220895	0.008197	-0.05227	-0.10238
PP13439	-0.02588475	0.016012763	-0.04448	0.002142	0.012505	0.137652
PP14571	-0.036264403	-0.050497062	-0.05393	0.006836	-0.00573	0.028192
PP2672	-0.041033138	-0.085517977	0.113168	-0.0152	0.070817	-0.00053
PP2D1	-0.054844441	-0.082672114	0.046303	-0.00567	0.011859	0.049869
PP7080	-0.002695867	-0.031807195	0.347158	0.039738	-0.08466	-0.25311
PPA1	0.031554785	-0.002486673	0.247456	0.037833	-0.09167	0.093769
PPA2	0.0064664	-0.038398838	0.079877	0.041821	-0.07832	-0.09294
PPAP2A	0.008162631	-0.072789115	0.183059	0.088634	-0.26697	-0.30493
PPAP2B	-0.008831255	-0.050118484	-0.05876	-0.07143	0.134606	-0.01107
PPAP2C	-0.029713524	-0.057212091	-0.03363	0.006287	0.002246	0.143998
PPAPDC1A	-0.036920633	-0.075536931	-0.0207	0.023828	-0.05148	-0.01542
PPAPDC1B	0.002639453	-0.046757369	-0.05457	-0.01052	0.019071	-0.17347
PPAPDC2	0.010744129	0.048286286	0.201638	0.001786	0.01671	-0.11477
PPAPDC3	-0.030712783	-0.074076233	-0.14002	-0.05193	0.108708	0.12767
PPARA	0.016156488	0.058964618	-0.03435	-0.03502	0.084712	-0.02537
PPARD	-0.016492584	-0.140307094	0.059674	0.015626	-0.06486	0.076216
PPARG	-0.008808541	-0.022957638	0.015856	-0.01563	0.010788	0.013173
PPARGC1A	-0.046485242	-0.113479153	-0.04166	0.018693	-0.03362	0.259208
PPARGC1B	-0.016101745	-0.143279307	0.00098	-0.02317	0.015336	0.387843
PPAT	0.000970456	0.056709978	0.110167	0.074914	-0.24064	-0.26523
PPBP	0.000826398	0.013248075	-0.10159	-0.13712	0.419503	0.160423
PPBPP2	-0.027262634	-0.025236628	0.029577	0.060886	-0.13634	-0.02015
PPCDC	0.012238414	-0.040911708	-0.26893	0.031784	-0.09605	0.149482
PPCS	0.089357709	0.033404262	-0.05503	-0.01322	0.029843	0.120514
PPDPF	0.043707921	-0.051871527	0.151316	0.046317	-0.103	-0.04563
PPEF1	-0.031072701	-0.049473866	-0.03364	-0.02376	0.083866	-0.00638
PPEF2	-0.080061087	-0.115734625	0.147338	-0.03776	0.081286	0.103452
PPFIA1	0.011939452	-0.037965972	-0.06989	0.008562	-0.04791	0.189162
PPFIA2	-0.04370442	-0.096331248	0.132234	-0.0047	0.041494	-0.0225
PPFIA3	-0.037200482	0.00799803	0.128494	0.086927	-0.21854	-0.01282
PPFIA4	-0.015077262	-0.080331786	-0.12807	-0.00777	0.01872	0.268977
PPFIBP1	0.025023469	-0.01967832	-0.00705	0.009855	-0.04687	-0.05315
PPFIBP2	0.007372195	-0.091206483	-0.00983	-0.08083	0.257849	0.350684
PPHLN1	0.052053699	0.043248341	0.170963	0.009121	-0.04774	-0.09097
PPIA	0.048447368	0.104493725	0.006838	0.112264	-0.21269	0.022077
PPIB	0.047670973	0.160991731	0.048638	0.084515	-0.20421	0.014489
PPIC	-0.041809468	-0.020457336	-0.04604	0.001931	-0.00132	-0.00662
PPID	0.041102401	0.130171263	0.080534	0.106434	-0.32031	-0.2254
PPIE	0.018691781	0.126263274	0.210298	0.075799	-0.18093	-0.03752
PPIEL	-0.025724049	-0.076194765	0.093518	-0.03222	0.019655	-0.0585
PPIF	0.022535222	-0.023374463	-0.23929	0.079222	-0.21328	0.195391
PPIG	0.067247654	0.041279841	0.094778	0.029017	-0.07641	-0.1389
PPIH	0.035946724	0.05886411	0.209566	0.070793	-0.20683	-0.13169

PPIL1	0.014919117	0.074394405	0.181564	0.074949	-0.21235	-0.10538
PPIL2	0.08177855	0.037619096	0.08883	0.034472	-0.02599	-0.23503
PPIL3	0.02589373	0.029363983	0.149274	0.020996	-0.04994	-0.01354
PPIL4	0.014882556	-0.040214752	-0.10366	0.051301	-0.1431	0.115934
PPIL6	-0.001824058	-0.095905661	-0.14658	0.113937	0.029466	0.035074
PPIP5K1	-0.01584784	-0.07817992	0.149548	0.108479	-0.30364	-0.1685
PPIP5K2	0.038449608	0.032917735	-0.27639	0.012546	-0.0211	0.182139
PPL	-0.034250745	-0.011792861	0.101777	-0.00145	0.022284	-0.07896
PPM1A	-0.014258336	0.09956853	-0.1471	-0.04298	0.10803	-0.16954
PPM1B	0.081682146	0.163344467	-0.12244	0.042752	-0.1012	-0.13736
PPM1D	0.018394682	0.074695554	-0.3266	0.037493	-0.0942	0.18358
PPM1E	-0.039064269	-0.006861654	-0.03458	-0.0074	0.016949	-0.12368
PPM1F	0.026682353	-0.107654927	-0.21071	-0.05426	0.107583	0.203122
PPM1G	0.041629629	0.064554221	-0.03169	0.092554	-0.26056	-0.13383
PPM1H	-0.023156673	-0.135444835	-0.20677	-0.01647	0.062639	0.237768
PPM1J	-0.007501675	-0.10433888	-0.11327	-0.01231	0.037998	-0.21012
PPM1K	0.014917394	0.011495568	0.188075	-0.00867	-0.013	-0.38126
PPM1L	0.015279986	0.295725328	-0.16303	-0.06771	0.157457	0.270079
PPM1M	0.036688552	-0.038864326	0.004327	-0.01131	0.03676	0.166396
PPM1N	-0.024495698	-0.056018532	0.23042	0.00723	-0.01485	0.033998
PPME1	0.037185735	-0.036695846	0.015534	0.078582	-0.29175	-0.14512
PPOX	-0.075502158	-0.077633951	-0.07076	0.063925	0.071772	0.139996
PPP1CA	0.059899864	0.160363738	-0.03011	0.048303	-0.0989	-0.12293
PPP1CB	0.019508972	0.017704651	-0.18113	0.036096	-0.03045	0.0529
PPP1CC	0.107919459	0.049703503	-0.02625	0.085409	-0.1717	-0.08457
PPP1R10	0.029234323	0.033181265	-0.06023	0.022805	-0.09924	-0.02208
PPP1R11	0.010204957	-0.00654074	-0.06585	-0.04066	0.042785	0.011361
PPP1R12A	0.140800036	0.15651097	-0.11323	-0.00865	0.015725	-0.26615
PPP1R12B	-0.019488212	0.032126635	-0.09568	0.001036	0.000285	-0.06805
PPP1R12C	-0.028337723	-0.11564708	-0.14602	0.004643	-0.07541	-0.08544
PPP1R13B	-0.001115485	0.147409277	0.256401	0.020694	-0.01466	-0.28814
PPP1R13L	-0.02634913	-0.077448954	0.106818	0.058225	-0.18818	-0.06529
PPP1R14A	-0.008954182	-0.079874352	-0.1547	-0.0824	0.239798	-0.15091
PPP1R14B	0.025762177	-0.090719477	-0.14347	0.064748	-0.17563	-0.13823
PPP1R14C	-0.014684111	-0.04026374	0.010587	-0.00474	-0.0022	-0.09274
PPP1R14D	-0.029494723	-0.029619038	-0.01534	-0.01651	0.021625	0.010315
PPP1R15A	0.01472793	-0.121277244	-0.13354	-0.01028	0.022842	0.128762
PPP1R15B	0.046499286	0.029247573	-0.11248	0.050676	-0.1207	0.116965
PPP1R16A	-0.006094211	-0.036257755	-0.00587	0.026327	-0.05644	0.050028
PPP1R16B	0.015926223	0.12119384	0.120181	0.041472	-0.11984	-0.558
PPP1R17	-0.021879885	-0.072178426	-0.05474	-0.03336	0.095382	0.184256
PPP1R18	0.063836924	0.14361077	-0.12288	0.008456	0.000709	-0.11656
PPP1R1A	-0.052055413	-0.103252034	-0.13232	0.024147	-0.02367	0.134943
PPP1R1B	-0.031830736	-0.00947093	-0.06033	0.002265	-0.02609	-0.0594
PPP1R1C	-0.05356026	-0.05074855	0.042702	0.017986	-0.02351	0.1867
PPP1R2	0.02500069	-0.071757988	0.281007	-0.06839	0.155383	-0.33977
PPP1R21	0.023573143	-0.018008867	-0.07664	-0.02233	0.047072	-0.04831
PPP1R26	-0.011018316	-0.023380329	-0.21528	-0.07523	0.157941	0.278995
PPP1R26-/	-0.048817984	0.002496196	-0.06433	0.021402	-0.04264	-0.00335
PPP1R27	-0.017559471	-0.031863067	-0.01618	-0.02658	-0.03917	-0.16545
PPP1R2P9	-0.025948276	-0.137713675	0.133188	0.005573	0.007249	-0.11056

PPP1R32	-0.032445967	-0.085119296	-0.00419	0.003237	0.014605	0.052594
PPP1R35	0.042814466	0.051239371	0.270781	0.04176	-0.15862	-0.40259
PPP1R36	-0.026974926	0.033873981	-0.05584	0.002818	-0.02236	-0.03629
PPP1R37	-0.039957575	0.005892443	-0.05486	0.034572	-0.04165	0.059736
PPP1R3A	-0.08934274	-0.109992019	0.073595	-0.01622	0.097238	-0.08997
PPP1R3B	-0.013757204	-0.022411658	-0.18965	0.00831	-0.04963	-0.12482
PPP1R3C	-0.034877662	-0.031039195	0.032282	0.002662	-0.00844	-0.17563
PPP1R3D	0.000333156	0.050161458	-0.238	-0.05975	0.164612	0.268701
PPP1R3E	-0.025541137	-0.035553654	0.246794	-0.02171	0.035629	-0.24367
PPP1R3F	0.018113173	-0.053784202	0.06605	-0.0293	-0.08726	-0.03498
PPP1R7	0.024255203	0.05669047	0.03007	0.019323	-0.0467	0.145912
PPP1R8	0.075983668	0.257277735	0.151058	0.048367	-0.14216	-0.143
PPP1R9A	-0.053869648	0.388032345	-0.11295	-0.00653	-0.01027	-0.15095
PPP1R9B	0.001071278	-0.071254973	-0.19969	-0.00374	0.015108	0.212725
PPP2CA	0.031597947	0.04800651	-0.09757	0.001197	-0.00337	0.088046
PPP2CB	0.033492535	0.065037122	-0.03088	0.008291	-0.01825	0.261307
PPP2R1A	0.043275133	0.068056819	0.036341	0.037882	-0.14896	-0.05303
PPP2R1B	-0.040861668	0.023080829	0.019918	0.069126	-0.23073	-0.04984
PPP2R2A	0.02104162	0.026912638	0.018923	0.002312	-0.0172	-0.20243
PPP2R2B	-0.009448275	0.32088893	0.315481	0.067571	-0.17859	-0.28101
PPP2R2C	-0.041350005	-0.061016726	-0.12835	0.076399	-0.10582	-0.081
PPP2R2D	0.007672422	0.004891945	0.068286	-0.04749	0.076699	-0.2017
PPP2R3A	-0.004540245	0.013848108	-0.03607	0.038697	0.024807	0.069385
PPP2R3C	0.015368426	0.065391189	-0.16579	-0.00191	0.026671	-0.02472
PPP2R4	0.016519183	-0.090621668	0.028003	0.000933	-0.20202	-0.01093
PPP2R5A	0.046073753	0.150895885	-0.17398	-0.00545	0.065636	0.006945
PPP2R5B	0.007268053	-0.029843386	-0.13148	-0.01866	-0.00626	0.146372
PPP2R5C	0.020748508	0.110627291	0.224652	-0.02701	0.006393	-0.19719
PPP2R5D	0.033611529	-0.019618765	0.063615	0.106837	-0.35993	-0.14727
PPP2R5E	0.054985813	0.177461952	0.182135	0.032383	-0.0782	-0.14081
PPP3CA	0.071203585	0.191760513	-0.39773	0.001064	-0.03583	-0.07849
PPP3CB	0.038873518	0.059066902	0.084893	0.047655	-0.12245	-0.16144
PPP3CB-AS	0.007351738	0.010986744	0.091374	-0.06089	0.191447	0.05291
PPP3CC	0.002092678	0.109617239	0.251891	-0.00592	0.006326	-0.39418
PPP3R1	0.018016633	0.056115827	-0.25605	0.022	-0.06735	-0.04174
PPP3R2	-0.08177404	-0.054674859	-0.04235	-0.0032	0.017094	0.055275
PPP4C	0.045709848	0.033126999	-0.19541	0.057231	-0.11824	0.155261
PPP4R1	0.044820385	0.045894507	-0.38728	0.028013	-0.05581	0.065722
PPP4R1L	-0.029647072	-0.116171685	-0.00521	0.002727	0.103833	0.077733
PPP4R2	0.033090038	0.1639871	0.065614	0.032878	-0.0917	-0.17831
PPP4R4	0.021447293	-0.053495507	-0.13626	0.088596	-0.05423	-0.02056
PPP5C	-0.024156861	0.049956287	0.082123	0.042564	-0.17957	0.008251
PPP5D1	-0.043150042	-0.007074673	0.022506	-0.00643	0.041555	0.070517
PPP6C	0.073083375	0.068896445	0.054933	-0.00411	0.033077	-0.09517
PPP6R1	0.061742956	0.030819119	0.070019	0.017773	-0.07799	-0.18412
PPP6R2	0.002910725	-0.013528467	0.080989	-0.05993	0.081205	-0.04439
PPP6R3	0.014000786	-0.008536848	0.046333	0.049857	-0.11627	-0.26277
PPRC1	0.012640954	0.000170995	0.121602	0.042427	-0.10232	-0.04051
PPT1	0.046295871	-0.147770716	-0.2265	0.009206	-0.00799	0.421111
PPTC7	0.065748498	0.07869232	-0.18876	0.031688	-0.0189	0.055836
PPWD1	0.014712453	0.062077906	0.174239	-0.01208	0.040126	-0.21051

PPY	-0.028560154	-0.06387815	-0.07271	0.045393	-0.10858	-0.12149
PPY2	-0.029009056	-0.061581775	-0.06428	-0.01605	0.06686	0.001204
PQBP1	0.039584834	0.024264846	0.098734	0.068617	-0.17633	0.162522
PQLC1	0.018719735	-0.174728517	-0.26459	-0.03512	0.086872	0.201719
PQLC2	0.003733464	-0.104377632	-0.13748	0.045284	-0.05187	0.212339
PQLC3	0.021235386	0.044826296	0.004617	-0.06773	0.181868	0.297968
PRAC1	-0.047482464	-0.016106365	-0.09867	-0.00324	0.011129	0.00331
PRADC1	0.019806911	0.009786466	-0.03337	0.069787	-0.232	0.138475
PRAF2	0.008571635	0.003898294	0.170839	-0.08031	0.141623	-0.23687
PRAM1	0.003326861	-0.098616008	-0.25209	-0.07715	0.228192	0.552373
PRAME	-0.033802169	-0.128929399	0.075738	0.038569	-0.08039	-0.19763
PRAMEF10	-0.0317879	-0.031252526	0.023508	0.025669	-0.10258	-0.02909
PRAMEF11	-0.027923579	-0.025305037	0.08327	0.031004	-0.09983	-0.08033
PRAMEF12	-0.032821753	-0.074858552	-0.0272	0.018247	-0.06283	-0.07509
PRAP1	-0.034014857	-0.136682456	-0.07697	0.010395	-0.03246	0.056348
PRB1	-0.027143879	-0.091482096	-0.01895	-0.04899	-0.04341	-0.10595
PRB3	-0.026475441	-0.103426258	0.002257	-0.01163	-0.01037	-0.04147
PRB4	-0.020255694	-0.060661904	0.091872	0.015382	-0.05666	-0.12571
PRC1	-0.006404497	0.002765527	-0.03071	0.134631	-0.34811	-0.10908
PRCC	0.008552762	0.012566314	-0.14133	0.068374	-0.15203	0.031604
PRCD	-0.026402772	-0.114230083	0.201547	-0.01175	-0.01891	-0.23412
PRCP	-0.030597934	-0.090219037	-0.14411	-0.03519	0.067595	0.172394
PRDM1	0.003436076	0.132812296	0.095927	0.008178	-0.09238	-0.25779
PRDM10	0.006649927	0.140870458	-0.15053	0.01174	-0.02219	-0.06704
PRDM11	-0.096434825	0.193565499	0.133607	0.03924	0.180265	-0.1204
PRDM12	-0.022417889	-0.089726776	-0.09405	-0.01164	-0.04544	-0.09534
PRDM13	-0.025515311	-0.04165711	-0.0028	0.072705	-0.19159	0.044972
PRDM14	-0.023075245	-0.077032906	-0.15895	0.001453	-0.04438	-0.05869
PRDM15	-0.010987166	0.007246431	0.015331	0.004478	-0.0134	0.032965
PRDM16	-0.052272161	-0.033155126	-0.00856	0.062384	-0.16039	0.01986
PRDM2	0.026714361	0.186645933	0.061398	-0.06881	0.16806	-0.21846
PRDM4	0.055498532	0.055555832	-0.00339	0.023523	-0.09619	-0.34944
PRDM5	-0.04115136	-0.074803294	-0.13041	-0.01889	0.021694	-0.07116
PRDM6	-0.040984695	-0.055669925	0.057599	0.003651	-0.07795	-0.07894
PRDM7	-0.050416646	-0.031817079	-0.00735	0.026616	-0.05254	-0.07122
PRDM8	-0.034122258	-0.079376973	-0.06625	0.089392	-0.21025	0.028195
PRDM9	-0.046014569	-0.071482875	0.003518	0.023976	-0.03428	-0.03363
PRDX1	0.031086391	-0.01596597	0.072649	0.062802	-0.18315	0.23586
PRDX2	0.03761887	0.104199454	0.269781	-0.21423	0.048737	-0.07487
PRDX3	0.046703818	-0.001753021	-0.23869	0.071621	-0.19514	0.227274
PRDX4	0.020829409	0.057664348	0.035145	0.061868	-0.15398	0.256223
PRDX5	0.04200612	0.120848623	-0.22088	0.132644	-0.33781	0.164862
PRDX6	0.085890531	0.021802844	0.207712	0.066923	-0.15634	0.064085
PREB	0.047559065	0.097338905	-0.11814	0.047084	-0.16477	0.122425
PRELID1	0.047362596	0.029438811	-0.28377	0.076954	-0.19871	0.326912
PRELID2	-0.030443579	-0.09139481	-0.056	-0.03826	0.05086	0.020948
PRELP	-0.03030246	-0.019580327	-0.15961	0.035547	-0.09775	0.00353
PREP	0.016904973	0.105666256	0.050083	0.02244	-0.12139	0.185727
PREPL	0.008423829	0.117730294	0.126075	0.051437	-0.1464	-0.13827
PREX1	0.04952071	0.145260051	-0.17961	0.009019	0.004976	0.012528
PREX2	-0.083353011	0.004516617	0.072249	0.050941	0.030349	0.007323

PRF1	0.0174973	0.31726163	0.397032	0.013879	-0.04587	-0.3047
PRG2	-0.01439673	-0.069346848	-0.16131	0.043553	-0.14166	0.029048
PRG3	-0.035772677	-0.057964961	-0.00932	0.031109	-0.09171	0.023275
PRG4	-0.057539747	-0.06950674	0.052765	-0.04637	0.12978	0.017651
PRICKLE1	0.031167256	-0.085702961	-0.20707	0.003148	0.03646	-0.07868
PRICKLE2	-0.021852573	0.055581734	-0.03625	0.051145	-0.15388	-0.05837
PRICKLE2-	-0.023991939	-0.038741056	-0.01882	0.002672	-0.00868	-0.06383
PRICKLE3	-0.028581823	-0.08847898	-0.17833	-0.02338	0.081418	0.227497
PRIM1	0.011295789	0.035521596	0.219968	0.060631	-0.16285	-0.22571
PRIM2	-0.00774727	0.063954666	0.068515	0.138061	-0.37191	-0.03982
PRIMA1	-0.034617137	-0.043047365	0.04664	0.007218	-0.01471	-0.06134
PRIMPOL	0.006331905	0.023140573	0.001153	0.002721	-0.01106	-0.10753
PRKAA1	0.037196901	0.078095464	0.104735	-0.02219	0.027451	0.012994
PRKAA2	-0.07347028	-0.044588792	0.124899	-0.06049	0.041082	-0.06762
PRKAB1	0.014785459	0.029560265	-0.12216	-0.02451	-0.00478	-0.16871
PRKAB2	-0.006547379	0.079278808	0.081193	-0.01649	0.039363	0.074096
PRKACA	0.005556847	-0.076261076	-0.24648	-0.02905	0.096248	0.534202
PRKACB	0.008145277	0.132920176	0.291542	-0.02932	0.059994	-0.30381
PRKACG	-0.039697437	-0.040148543	-0.10706	-0.00832	0.011067	-0.07251
PRKAG1	0.064055484	0.122907588	-0.06712	0.081023	-0.24451	0.010027
PRKAG2	-0.003444901	0.02519308	-0.09062	-0.13448	0.319015	0.117617
PRKAG2-AS	-0.016341876	-0.115741371	0.05535	-0.08988	0.303213	0.357098
PRKAG3	-0.029720524	-0.048574525	0.047627	0.01341	-0.06888	-0.15421
PRKAR1A	0.030533326	0.137150052	-0.35795	0.02612	-0.02917	0.042137
PRKAR1B	-0.023447136	-0.125508839	0.153358	0.009408	-0.10345	-0.1963
PRKAR2A	0.081603758	0.169608602	-0.0009	0.029293	-0.01617	0.119782
PRKAR2B	0.007467107	-0.044405572	-0.13723	-0.1647	0.465127	0.209782
PRKCA	-0.065895325	0.017522813	0.026761	0.003517	-0.10029	0.012536
PRKCB	0.023493425	0.010670933	-0.07415	-0.0576	0.157686	-0.08537
PRKCD	0.016090306	-0.100573669	-0.46916	-0.07527	0.214387	0.402552
PRKCDBP	-0.01346465	-0.05832316	-0.11234	-0.00937	0.048678	0.291124
PRKCE	-0.017804184	-0.004381013	-0.04438	0.05077	-0.13006	0.039762
PRKCG	-0.038380584	-0.094848073	-0.02772	0.026358	-0.10774	-0.09367
PRKCH	0.013769143	0.241044623	0.423218	-0.01185	0.00925	-0.34521
PRKCI	0.023496613	0.01771427	0.102558	-0.0333	0.063941	0.104973
PRKCQ	0.009401038	0.165496886	0.454632	-0.02113	0.033372	-0.31091
PRKCQ-AS	-0.002087231	0.094701168	0.382555	0.021995	0.016878	-0.30091
PRKCSH	0.04528901	-0.069060816	0.006118	0.042686	-0.12548	0.03167
PRKCZ	-0.00544584	-0.019738389	0.064078	-0.09496	0.231762	-0.27003
PRKD1	-0.048728928	-0.079682423	-0.12938	-0.0201	0.061516	-0.09211
PRKD2	0.001790157	0.083350128	0.081722	-0.02891	-0.00544	-0.27214
PRKD3	0.052367214	0.104279231	-0.09512	0.022956	0.073154	0.063861
PRKDC	0.027513017	0.051721154	0.046938	0.102667	-0.26909	-0.25202
PRKG1	0.001890522	-0.022363231	0.064818	-0.00224	0.078206	0.000225
PRKG1-AS	-0.021059758	-0.080201023	0.003451	-0.0025	0.004946	-0.0372
PRKG2	-0.024680573	-0.008146795	-0.00446	0.005953	-0.01984	-0.15074
PRKRA	0.02343487	-0.041418223	0.13119	0.028421	-0.04807	-0.0413
PRKRIR	0.072862578	0.173651849	0.267987	0.02121	-0.04332	-0.2521
PRKX	0.027033007	0.173383506	-0.012	0.000745	########	0.121348
PRKXP1	0.015190256	-0.007539261	0.311269	-0.03514	0.095351	-0.22199
PRKY	0.003213779	0.08635512	0.20476	0.001257	0.036866	-0.10511

PRL	-0.034847034	-0.075398163	0.21765	0.000892	0.018108	0.083545
PRLH	-0.018622531	-0.002367602	-0.03701	0.029894	-0.07868	0.073961
PRLHR	-0.046344935	-0.094353888	-0.04941	-0.00832	0.048447	-0.01325
PRLR	-0.087036937	-0.038865495	-0.0681	0.044515	0.051893	0.471196
PRM1	-0.032909177	-0.016320102	0.00262	0.018489	-0.06087	-0.06859
PRM2	-0.033879228	-0.067376876	-0.05502	0.05333	-0.12191	0.028669
PRM3	-0.03288244	0.003785286	-0.07098	0.023402	-0.05332	-0.12019
PRMT1	0.014345236	0.015670932	0.095617	0.084808	-0.21685	-0.07694
PRMT2	0.017950808	0.060339324	0.26418	-0.01269	0.064398	-0.15978
PRMT3	0.009267159	-0.003914618	0.03786	0.069337	-0.17853	0.140352
PRMT5	-0.015043972	-0.051260735	-0.07692	0.048952	-0.2298	0.074457
PRMT5-AS1	-0.037100528	-0.0012539	-0.00992	0.012411	-0.00989	-0.09255
PRMT6	0.007902508	0.084284702	0.045376	-0.02517	0.091163	0.060844
PRMT7	0.003839357	0.003326867	0.03927	0.068509	-0.16767	-0.04029
PRMT8	-0.047779021	-0.07174256	-0.03109	0.008071	-0.01067	-0.01512
PRMT9	0.022306103	0.071287851	0.100782	0.06958	-0.16355	-0.06437
PRND	-0.055595649	-0.112378519	0.012066	-0.04683	0.162949	-0.15264
PRNP	0.035862852	0.126926426	0.112173	0.011954	-0.03671	0.283531
PRNT	-0.028206046	-0.021164337	-0.00873	0.026659	-0.06291	-0.20669
PRO0471	-0.025255738	0.011697347	0.078706	-0.01667	0.053487	-0.06346
PRO1082	-0.028617753	-0.011039951	0.11536	-0.00874	0.040807	-0.03749
PRO1483	-0.045729748	-0.096631958	0.007334	0.003157	-0.00453	-0.13477
PRO1596	-0.033382916	-0.01240453	-0.05042	8.15E-05	0.015693	0.033954
PRO1804	-0.039059948	0.031811631	0.135137	-0.01182	0.024741	-0.10661
PRO2012	-0.026900411	-0.068333794	-0.00666	-0.00102	0.017656	-0.11801
PRO2214	-0.040814746	0.013396897	0.038335	6.15E-05	-0.05208	-0.06554
PRO2852	0.011237303	-0.000205586	-0.08704	-0.00021	-0.00064	-0.02178
PRO2949	-0.074154972	-0.069896015	-0.02003	0.029063	-0.06892	0.013085
PRO2958	-0.03514831	-0.012868894	0.107115	0.001017	0.014048	-0.12231
PRO2964	-0.045833486	0.005433494	0.044192	-0.01792	0.074494	0.087125
PROC	-0.008133018	-0.087418152	-0.05546	-0.02541	0.079669	-0.10154
PROCA1	-0.016145238	-0.152210656	-0.03328	-0.07281	0.14648	0.01542
PROCR	-0.011155706	0.166669576	0.130967	-0.03193	0.113315	-0.0248
PRODH2	-0.029372689	-0.097867391	-0.03067	0.009595	-0.10236	-0.01872
PROK1	-0.036084928	-0.038615668	-0.0308	-0.02299	0.055398	-0.09303
PROK2	0.000188063	-0.112359264	-0.10558	-0.02461	0.073842	0.280409
PROKR2	-0.03202963	-0.037522134	-0.0548	0.017249	-0.0237	0.028062
PROL1	-0.029560679	-0.038815479	-0.00253	0.00136	0.017083	-0.18726
PROM1	-0.031489501	0.005400037	0.210134	-0.00944	0.031127	-0.11765
PROM2	-0.021105615	-0.093957183	-0.01813	0.016306	-0.07664	-0.05458
PROP1	-0.025331563	-0.047588949	-0.04107	-0.00305	-0.01271	0.023223
PRORSD1P	-0.002855595	0.161213454	0.093824	-0.03773	0.127589	-0.16124
PRORY	-0.025178514	-0.137963579	0.080316	0.003712	-0.04147	-0.08528
PROS1	-0.012675374	-0.038262234	-0.06581	-0.22818	0.650509	0.17194
PROSC	0.009040691	0.044444596	-0.01616	-0.0013	-0.01473	0.240293
PROSER1	0.027341143	0.083100083	0.045709	0.061677	-0.13634	0.081754
PROSER2	-0.015915124	-0.090061532	-0.11335	-0.14545	0.404603	0.153678
PROSER2-A	-0.058677982	-0.088419434	-0.10351	-0.02435	0.110561	0.0401
PROSER3	-0.002155307	0.006549476	0.216019	0.049218	-0.13947	0.044996
PROX1	-0.041646238	-0.038271323	-0.00798	-0.01827	-0.02115	-0.13351
PROX1-AS1	-0.05460485	-0.028228308	-0.10512	-0.02629	0.077421	0.071548

PROX2	-0.034885859	0.096868452	-0.05725	-0.02321	0.081711	-0.07248
PROZ	-0.033182441	-0.087765468	0.013327	-0.00901	-0.01319	-0.11753
PRPF18	0.021862302	0.114554819	-0.01694	0.005652	-0.03939	0.055788
PRPF19	0.046154181	-0.033386737	0.145238	0.052297	-0.16087	0.059706
PRPF3	0.048799694	0.034131495	0.141926	0.046024	-0.13787	-0.12277
PRPF31	-0.004037865	0.058850905	0.116819	0.010881	-0.06323	0.099235
PRPF38A	0.105252387	0.186022583	0.128218	0.048224	-0.13085	-0.04283
PRPF38B	0.060334757	0.166435024	0.224927	-0.00348	0.003571	-0.40758
PRPF39	0.015770195	0.070998661	0.181753	0.046239	-0.11569	-0.16
PRPF4	0.04031673	0.086623983	-0.083	0.060929	-0.18603	0.05475
PRPF40A	0.041989904	0.096815973	-0.03747	0.070874	-0.14089	0.077568
PRPF40B	-0.025676699	-0.06491014	0.060268	0.013404	-0.08472	-0.18177
PRPF4B	0.07283507	0.120092764	0.153773	-0.02766	0.035764	-0.0861
PRPF6	0.0474372	0.050861267	0.222864	0.048967	-0.16568	-0.06987
PRPF8	0.073255811	0.070379813	0.238445	0.071608	-0.17929	-0.29348
PRPH	-0.01666549	-0.033665627	-0.01596	0.006937	-0.09028	-0.06565
PRPH2	-0.024969685	-0.019002703	-0.01741	-0.00173	-0.02732	-0.03571
PRPS1	0.02464794	0.072724117	0.300202	0.046755	-0.15004	-0.35852
PRPS1L1	-0.045889843	-0.028840766	-0.02293	0.01624	-0.02441	-0.01934
PRPS2	0.033152943	0.039663994	0.0877	0.045829	-0.12707	-0.03213
PRPSAP1	0.017414887	0.056289659	0.0815	0.018578	-0.07778	-0.06743
PRPSAP2	0.039825975	0.072240758	0.217537	0.031109	-0.08556	-0.17513
PRR11	0.083080162	0.048629815	0.021157	0.111264	-0.04296	-0.25107
PRR12	0.004954824	0.010648748	0.165123	0.012572	-0.03392	-0.05004
PRR13	0.119586524	0.068800229	-0.16467	0.057542	-0.08226	-0.00644
PRR14	0.058423246	0.027968882	-0.06907	0.002078	-0.01209	-0.20904
PRR14L	-0.012500681	0.015194172	-0.10856	0.000989	-0.03747	0.104203
PRR15	-0.033640141	-0.035328867	0.054944	########	0.027094	0.086823
PRR15L	-0.032617216	-0.095646508	-0.01956	0.001182	-0.01537	0.04845
PRR16	-0.119919005	-0.089272163	-0.09614	-0.11328	0.3631	0.266506
PRR18	-0.032815148	-0.073890164	-0.0151	0.01133	-0.05336	-0.08217
PRR19	-0.023729294	-0.070044867	-0.06947	0.054767	-0.14302	0.012989
PRR22	-0.033792027	-0.041221113	-0.00829	0.017882	-0.05119	0.04254
PRR24	0.010927464	-0.138115267	-0.15208	-0.0201	-0.00548	0.096119
PRR26	-0.054917674	-0.066301728	0.070282	-0.01812	0.046778	-0.00685
PRR29	-0.008953621	-0.058931564	0.035987	-0.01882	-0.08238	-0.23485
PRR3	-0.009921281	0.001007842	0.081396	0.079553	-0.27449	-0.08276
PRR30	-0.030061812	-0.028746782	-0.02087	0.000758	-0.05004	-0.05787
PRR32	-0.044246662	-0.017972418	0.119535	-0.00692	0.050501	0.041285
PRR34	-0.02097277	-0.052043292	0.083434	-0.03551	-0.00973	-0.11741
PRR34-AS	0.024321775	0.023629417	-0.15555	-0.10755	0.281698	0.193241
PRR35	-0.020252411	-0.056952161	-0.01226	-0.01728	-0.05072	-0.03675
PRR5	0.003089734	0.197944074	0.418829	-0.03485	0.09468	-0.23948
PRR5L	-0.025258552	0.293718079	0.061312	-0.00216	0.116023	-0.16282
PRR7	-0.011503027	0.001542073	0.140691	-0.00943	0.067753	-0.17267
PRR7-AS1	-0.029351109	-0.036814197	0.005629	0.014029	-0.04662	-0.05544
PRR9	-0.039206713	0.002800139	-0.06414	0.021087	-0.02884	-0.12913
PRRC1	0.04567738	0.104411967	0.166493	0.014797	-0.05792	-0.02454
PRRC2A	0.026447386	-0.078816107	0.046718	0.04978	-0.21814	-0.11951
PRRC2B	0.022661192	0.084918655	0.262263	0.030374	-0.11735	-0.007
PRRC2C	0.069016761	0.10189774	0.212335	0.075845	-0.16589	-0.23825

PRRG1	-0.02054489	-0.043723306	0.012771	-0.01109	0.015759	0.072
PRRG2	-0.031584547	-0.109638459	0.02131	0.030728	-0.05003	0.158575
PRRG3	-0.052362773	-0.046999821	-0.0956	0.024681	-0.0872	0.08193
PRRG4	-0.003960164	-0.084751497	-0.22363	-0.07973	0.193097	0.394452
PRRT2	-0.02467414	-0.037297974	0.212243	-0.00738	0.052599	-0.11599
PRRT3	-0.010054877	0.046708966	0.141873	0.032427	-0.09127	-0.09467
PRRT3-AS	-0.029275582	0.034257256	0.070304	0.010607	-0.01501	-0.02545
PRRX1	0.045828621	0.068587508	-0.04605	0.055555	0.063524	-0.1444
PRRX2	-0.008684245	-0.062060927	0.057434	-0.00473	-0.07378	-0.06013
PRRX2-AS	-0.029787754	-0.088154775	0.020551	0.001814	-0.06185	-0.11378
PRSS12	-0.06452479	-0.018356166	0.021386	-0.00595	0.026617	0.030817
PRSS16	-0.03588972	-0.055634801	0.060052	-0.02462	0.095831	-0.1446
PRSS2	-0.034247657	0.065083806	-0.07104	0.006434	-0.02241	-0.10906
PRSS21	-0.015006263	-0.067421779	-0.02255	-0.03636	0.050621	0.065004
PRSS22	-0.027459042	-0.037925639	0.045393	-0.0098	0.001113	0.017098
PRSS23	-0.025624636	0.325631173	0.112363	-0.06325	0.193887	-0.12993
PRSS27	-0.035096566	-0.027097202	0.039087	0.03602	-0.06881	0.019858
PRSS3	-0.031217086	-0.066854568	-0.04041	-0.00635	-0.03523	-0.01713
PRSS30P	-0.002201791	0.258808938	0.06545	0.050107	-0.19037	-0.08336
PRSS33	-0.00531476	-0.047390126	-0.09436	-0.00239	-0.01122	-0.15714
PRSS35	-0.022810437	-0.013161148	0.193743	-0.04238	0.044098	-0.06849
PRSS36	-0.028004072	-0.024106282	-0.07374	-0.03055	-0.01007	-0.01759
PRSS37	-0.032935494	-0.04296726	0.028309	-0.00281	0.031783	0.068497
PRSS3P2	-0.020167423	-0.090979932	0.065685	-0.03351	-0.00884	-0.07622
PRSS3P3	-0.03317581	-0.117389257	0.029573	-0.01385	0.015402	-0.03243
PRSS50	-0.026874267	-0.08468824	-0.04114	0.008261	-0.00993	0.082581
PRSS53	-0.015669196	-0.055148692	0.184837	-0.02872	-0.00828	-0.11089
PRSS54	-0.032302279	-0.047192322	0.157827	0.022408	-0.04762	-0.14903
PRSS55	-0.040015276	-0.041162758	0.115521	-0.00309	0.030436	-0.03562
PRSS58	-0.031995446	0.042333354	0.123843	0.022542	-0.04362	-0.0638
PRSS8	-0.036705863	-0.029675775	-0.08877	0.007881	0.003301	-0.04027
PRTFDC1	-0.015350054	0.001880848	0.119036	-0.15054	0.419023	-0.12417
PRTG	-0.098534401	-0.034019222	0.079359	-0.05697	-0.00086	-0.06355
PRTN3	-0.024296243	-0.064801495	-0.07325	-0.0165	0.053475	-0.12152
PRUNE	-0.01391964	0.053482675	0.085723	-0.06223	0.125867	-0.19773
PRUNE2	-0.041122349	-0.063322052	-0.00998	0.054635	-0.17824	0.065809
PRX	-0.034849292	-0.130818788	0.05941	-0.0191	-0.02073	-0.04263
PSAP	0.029175179	-0.065237601	-0.48813	-0.06803	0.217564	0.559249
PSAPL1	-0.01877053	-0.029758898	-0.04312	-0.00601	-0.05726	-0.14685
PSAT1	-0.001979103	0.035333987	0.105761	0.09308	-0.28411	-0.12042
PSCA	-0.030522979	-0.101924597	########	0.014153	-0.02693	-0.11291
PSD	-0.027953168	-0.077733438	-0.01134	0.012079	-0.03075	0.036262
PSD2	-0.033806737	0.04604692	-0.06822	-0.02311	0.009293	-0.05563
PSD3	-0.008233309	-0.048538941	-0.04446	-0.01044	-0.03236	-0.0469
PSD4	0.020220275	0.073827017	0.075987	-0.00637	-0.01833	-0.24339
PSEN1	-0.047153986	0.00011722	-0.17562	-0.10871	0.224812	0.128983
PSEN2	0.02040545	0.103723542	-0.22837	-0.06246	0.107676	0.216839
PSENEN	0.027197442	0.006740254	-0.27212	0.066575	-0.2025	0.241645
PSG1	-0.035480505	-0.104116188	-0.02887	-0.00658	-0.00063	-0.03448
PSG11	-0.043128585	-0.007330812	0.021631	-0.03172	0.114672	-0.10985
PSG2	-0.029484626	-0.070976332	0.02496	0.010036	-0.02167	-0.05079

PSG3	-0.04204749	-0.068216131	-0.03795	-0.00742	0.044357	0.083416
PSG4	-0.031464804	-0.065315858	0.064124	0.004475	-0.04661	-0.04303
PSG5	-0.036795019	-0.056010433	0.047692	-0.01323	-0.00722	-0.10763
PSG6	-0.027629877	-0.08449114	0.021327	0.069219	0.015927	-0.09316
PSG7	-0.030687551	-0.065462836	0.043951	0.008825	-0.05984	-0.05567
PSG9	-0.058950998	-0.074703183	0.029971	-0.01158	-0.06895	-0.03848
PSIP1	0.015410239	-0.002038075	0.208068	0.023355	-0.05476	-0.30218
PSKH1	0.000422246	-0.050360369	-0.04061	0.003977	-0.07986	0.153386
PSKH2	-0.032029873	-0.06672569	-0.0938	-0.01203	0.006143	0.074107
PSMA1	0.063788467	0.117325447	0.01754	0.097697	-0.23594	0.200027
PSMA2	0.061154562	0.083822784	-0.00629	0.061114	-0.17804	0.100461
PSMA3	0.001061764	0.055777705	0.008597	-0.04104	0.177171	0.001352
PSMA4	0.091854404	0.177125812	-0.19625	0.091722	-0.23346	0.363415
PSMA5	0.039106466	0.172349648	0.177394	0.05247	-0.12723	-0.10709
PSMA7	0.065829213	0.093126579	-0.01639	0.093209	-0.24738	0.234993
PSMA8	-0.018028392	0.04567739	0.092021	0.036804	-0.09604	-0.11091
PSMB1	0.108631632	0.159155088	0.060096	0.146077	-0.34134	0.111811
PSMB10	0.056652048	0.136194273	-0.08668	0.047693	-0.11808	0.227296
PSMB2	-0.010298996	0.115228088	-0.05036	0.128699	-0.31028	0.079013
PSMB3	0.061530933	0.053705319	-0.21549	0.149833	-0.37709	0.178569
PSMB4	0.099405528	0.121852078	0.013058	0.126273	-0.29993	-0.08447
PSMB5	0.041693638	-0.016879079	0.02471	0.099459	-0.29289	0.106965
PSMB6	0.069882223	0.100847142	-0.06286	0.122184	-0.33708	0.250963
PSMB7	0.038463929	0.031137975	0.040597	0.058203	-0.15698	0.180264
PSMB8	0.087400693	0.126935489	0.027913	0.099186	-0.23955	-0.00449
PSMB8-AS1	0.036378101	0.092318904	-0.01335	-0.02346	0.078066	-0.2734
PSMB9	0.058051145	0.11742476	-0.05113	0.048742	-0.10003	-0.08289
PSMC1	0.110011875	0.13910331	-0.11821	0.144083	-0.35258	0.028973
PSMC2	0.036594228	0.050769595	0.174182	0.015114	-0.0275	0.01767
PSMC3	0.027096984	0.103837466	0.050375	0.118376	-0.33025	0.151722
PSMC3IP	0.01110791	0.036142274	-0.14514	0.101101	-0.34651	-0.11168
PSMC4	0.05773236	0.119165416	-0.06645	0.099577	-0.2814	0.07353
PSMC5	0.06135046	0.116175481	0.300217	0.070136	-0.19586	-0.06381
PSMC6	0.070457743	0.136164858	-0.0627	0.105451	-0.27882	-0.0144
PSMD1	0.046380432	0.085959993	-0.07653	0.091709	-0.25764	0.139537
PSMD10	0.056245176	0.139605261	0.052753	0.064024	-0.1907	-0.10585
PSMD11	0.04548477	0.096134408	0.136945	0.069636	-0.19809	-0.08195
PSMD12	0.034658529	0.057977947	-0.02743	0.094363	-0.22897	0.271154
PSMD13	0.040281106	0.077359483	-0.10059	0.081787	-0.23667	0.02035
PSMD14	0.037805879	0.099902095	0.073984	0.089457	-0.26538	-0.01497
PSMD2	0.088531089	0.082366727	-0.10269	0.130274	-0.33273	0.074235
PSMD3	0.030168752	-0.000204838	0.011121	0.088496	-0.2315	0.085195
PSMD4	0.09962071	0.060731532	-0.20431	0.134608	-0.29047	0.109308
PSMD5	0.023243284	0.029765463	0.073679	0.042829	-0.14311	-0.02738
PSMD5-AS1	-0.034588628	0.046414362	0.103611	-0.03853	0.059175	-0.18778
PSMD6	0.059920107	0.118925173	0.067238	0.051533	-0.13839	0.037661
PSMD6-AS2	0.024809922	-0.020985434	0.073656	-0.03837	0.123	-0.1873
PSMD7	0.07289346	0.111519163	0.013221	0.093617	-0.26418	0.137926
PSMD8	0.071734214	0.115723843	-0.05513	0.10562	-0.30056	0.293648
PSMD9	0.043820024	-0.004971802	-0.02831	0.062897	-0.15736	0.125371
PSME1	0.092730411	0.195122357	-0.01186	0.089599	-0.19959	-0.12732

PSME3	0.021227736	0.025455914	-0.11744	0.040387	-0.17008	0.129502
PSME4	0.02127291	0.156495367	0.019404	-0.00034	-0.02017	-0.34782
PSMF1	0.038192122	-0.069035481	-0.08104	0.055009	-0.11785	0.322629
PSMG1	0.019495346	0.022945481	0.073517	0.098407	-0.25897	0.148079
PSMG2	0.070247213	0.05769682	0.103327	0.040529	-0.11001	0.217402
PSMG3	0.041984443	0.026993602	0.118024	0.047764	-0.14356	0.166638
PSMG3-AS	-0.009609534	-0.039957059	0.223013	-0.01157	0.032386	-0.29579
PSMG4	-0.057058547	-0.164041351	0.038195	-0.03796	0.001214	-0.08595
PSORS1C1	-0.039159618	-0.017730791	-0.12895	0.00119	0.024266	0.053872
PSORS1C2	-0.035755839	-0.025287208	-0.10152	0.034059	-0.11679	-0.03327
PSORS1C3	-0.042920836	-0.029497582	-0.024	-0.00303	0.044394	0.013375
PSPC1	0.042165806	0.068266272	0.065266	-0.09742	0.130378	-0.17243
PSPH	-0.026881773	-0.05048655	-0.0387	0.137252	-0.20467	0.045678
PSPN	-0.030844515	-0.113201556	-0.13532	0.025312	-0.0859	0.018359
PSRC1	-0.0095123	-0.04574802	-0.23151	0.046179	-0.15119	0.545353
PSTK	-0.011415086	0.057399033	0.047159	0.043846	-0.06074	0.025496
PSTPIP1	0.023093947	0.065889137	-0.06456	-0.01011	0.029899	0.272976
PSTPIP2	0.013617114	0.033102866	-0.166	-0.0746	0.197605	0.328332
PTAFR	0.000288243	-0.116546642	-0.35879	-0.04908	0.107467	0.405386
PTAR1	-0.002177272	0.034825347	0.097915	-0.00733	-0.00549	-0.03191
PTBP1	-0.014767544	-0.090754348	-0.03491	0.003545	-0.05266	0.04778
PTBP2	0.006406517	0.045105589	0.069196	0.042678	-0.10651	-0.14401
PTBP3	0.016507772	0.024663698	-0.169	0.01716	-0.09172	-0.02684
PTCD2	-0.00313569	0.015799879	0.189988	0.025369	-0.05844	-0.15332
PTCD3	0.001239744	0.000624917	0.218526	-0.02882	0.115446	-0.03704
PTCH1	0.004468747	0.230585959	0.202815	0.02396	0.021117	-0.18708
PTCH2	-0.030377022	-0.080065592	0.012979	-0.00672	0.009194	0.066162
PTCHD1	-0.062523032	-0.058092377	-0.10813	0.00714	-0.00576	0.04711
PTCHD2	-0.030398287	-0.063982723	0.04741	-0.0071	-0.05766	-0.07268
PTCHD4	-0.023763965	-0.052650874	0.01488	0.008422	-0.03821	-0.1469
PTCRA	0.009075316	-0.09106729	-0.0157	-0.08327	0.109796	-0.16825
PTCSC1	-0.020867381	-0.090664026	0.036723	0.025462	-0.12233	-0.12948
PTDSS1	0.056451489	0.007391597	0.111443	0.041955	-0.11771	0.104484
PTDSS2	0.007244422	-0.119326074	0.020094	0.017638	-0.14434	0.019591
PTEN	0.064758795	-0.075444257	-0.33792	-0.02632	0.072337	0.186054
PTER	0.020354934	0.088206504	0.085225	-0.03869	0.118169	0.092946
PTGDR	0.021821934	0.412778479	0.205728	-0.10433	0.183606	-0.22139
PTGDR2	-0.005576378	-0.06736185	-0.03156	-0.02023	0.050463	-0.11005
PTGDS	0.007313248	0.202125621	-0.08648	-0.04895	0.109901	-0.25827
PTGER1	-0.018607767	-0.075642059	-0.01046	-0.00795	-0.05471	-0.20855
PTGER2	0.012207731	0.165888565	0.26869	-0.04476	0.100701	0.069619
PTGER3	-0.025214178	-0.124108156	-0.00358	0.055929	0.038007	-0.03561
PTGER4	0.019694171	0.063338612	0.016837	0.011656	-0.03305	0.053589
PTGER4P2-	-0.030933002	-0.058195205	-0.05216	0.000868	-0.02218	-0.15838
PTGES	-0.023744782	-0.043964094	0.002966	-0.00346	-0.07488	-0.04962
PTGES2	0.024631481	-0.054109807	0.017485	0.043552	-0.18083	-0.07897
PTGES3	0.07374034	0.091930304	0.106466	0.130599	-0.25289	-0.10994
PTGES3L	-0.033973443	-0.079998463	-0.0293	-0.04516	0.155482	-0.06945
PTGFR	-0.078490761	-0.151514933	0.021639	-0.05745	0.165531	0.169016
PTGFRN	-0.015491756	-0.032305651	-0.1223	-0.01321	0.022428	0.179201
PTGIR	-0.002300814	-0.072744017	-0.02694	-0.06733	0.120634	0.117262

PTGIS	-0.008029392	0.017129862	-0.04475	0.018482	-0.16019	0.016029
PTGR1	2.46E-06	-0.008218852	-0.05574	0.030731	0.013976	-0.00354
PTGR2	-0.03384546	-0.01157496	0.121953	0.032859	-0.07132	0.05722
PTGS1	0.004368004	-0.108994654	-0.28666	-0.17497	0.435528	0.266634
PTGS2	-0.011107542	-0.063041575	-0.19756	-0.05766	0.11675	0.057403
PTH	-0.0824226	-0.092628132	-0.01747	-0.0162	0.03116	0.046524
PTH1R	-0.029678053	-0.054371812	0.080194	0.011671	-0.0436	-0.10621
PTH2	-0.01639414	-0.045866938	0.140338	-0.01625	-0.0752	-0.18753
PTH2R	-0.02183876	0.067792295	-0.02242	0.018831	-0.03313	-0.00942
PTHLH	-0.104912619	-0.072617018	-0.02139	-0.00796	0.086474	-0.04537
PTK2	-0.039434453	-0.06209516	-0.21121	-0.01129	0.101017	0.047796
PTK2B	0.017066012	-0.007845228	-0.14788	-0.06429	0.13835	0.045642
PTK6	-0.048797968	-0.145994073	-0.10269	-0.04036	0.091956	0.270374
PTK7	-0.021636201	-0.067369198	0.127049	-0.0061	0.037898	-0.27157
PTMA	0.078416945	0.14214628	0.074554	0.059623	-0.12106	-0.04418
PTMS	-0.018609885	-0.089733139	-0.1153	0.069324	-0.20282	0.056269
PTN	-0.041530169	-0.108253623	0.039556	0.041293	-0.11755	-0.06849
PTOV1	0.027234393	-0.103363718	-0.02013	0.055395	-0.15876	-0.06966
PTOV1-AS1	-0.052499688	-0.055640553	0.010052	0.014834	-0.01335	0.096951
PTP4A1	0.008784221	-0.050129619	-0.07434	0.068626	-0.16274	0.047786
PTP4A2	0.096679671	0.202083517	0.116607	0.022304	-0.05772	-0.0555
PTP4A3	0.019816079	-0.126839011	-0.00058	-0.02045	0.099954	0.020679
PTPDC1	-0.019773698	-0.024561548	0.188178	0.05222	-0.14853	-0.14625
PTPLA	-0.021815911	-0.015355262	-0.09507	-0.03388	0.053028	-0.039
PTPLAD1	0.023696036	0.147942108	0.266698	0.073297	-0.16018	-0.16245
PTPLAD2	0.01379275	-0.008169546	-0.06981	-0.06434	0.183168	0.228939
PTPLB	0.02430621	0.062842824	0.044113	0.031851	-0.083	0.054583
PTPMT1	0.017407677	-0.024308696	0.000728	0.044488	-0.08444	0.148445
PTPN1	-0.066213205	-0.010055287	0.041524	0.015708	-0.0275	-0.08831
PTPN11	-0.011984902	0.069709907	0.015333	0.067116	-0.19938	-0.02433
PTPN12	0.002617104	0.236996408	-0.27631	-0.11364	0.329384	0.203017
PTPN13	-0.036940656	-0.033599439	0.050353	-0.04989	0.16572	0.023952
PTPN14	-0.033635049	0.013587872	0.033883	0.040356	-0.07192	-0.07455
PTPN18	0.059717613	0.094084386	-0.19537	-0.06634	0.200675	0.211598
PTPN2	-0.03111745	0.161712192	-0.08379	-0.04643	-0.09099	-0.04666
PTPN20B	-0.049666829	-0.078626554	0.094541	-0.06064	0.174321	-0.06475
PTPN21	-0.020075791	-0.073422108	-0.12411	0.040885	0.002813	0.060335
PTPN22	0.006181791	0.16149572	0.095116	0.021691	-0.07348	-0.13477
PTPN23	-0.004661951	-0.125974649	0.031486	0.028802	-0.07418	0.063246
PTPN3	-0.01709874	-0.017106716	0.006217	0.127987	-0.31414	-0.02477
PTPN4	-0.006078592	0.267941059	0.27886	-0.03369	0.142684	-0.25568
PTPN5	0.001101497	-0.028513067	-0.02309	0.084468	-0.01705	-0.01119
PTPN6	0.039853042	-0.03079749	-0.18086	-0.09576	0.258919	0.002081
PTPN7	0.008562941	0.08690168	0.169676	0.062636	-0.21331	-0.35408
PTPN9	-0.04707865	0.072824884	0.053818	0.042705	-0.04154	0.089053
PTPRA	0.015681842	0.173825727	0.255278	-0.0171	0.028845	-0.19704
PTPRB	-0.079744581	0.23529178	-0.01852	0.107609	-0.01445	-0.04774
PTPRC	0.048233413	0.16814027	0.041706	0.001603	0.046135	-0.23349
PTPRCAP	0.01658158	0.131479675	0.319555	0.024632	-0.07893	-0.48183
PTPRD	-0.047767426	-0.043408745	0.018445	0.037097	-0.05047	-0.14867
PTPRE	-0.017104668	-0.070303717	-0.20203	-0.06928	0.162642	0.026922

PTPRF	-0.016020549	-0.035627447	0.074119	-0.02388	0.091518	-0.00013
PTPRG	-0.043700574	0.026583038	-0.02139	0.103737	-0.14166	-0.02876
PTPRG-AS:	-0.071702542	-0.03997173	0.101401	-0.02643	0.082915	0.024464
PTPRH	-0.033523251	-0.043710524	-0.01922	0.021342	-0.06481	-0.05906
PTPRJ	-0.053920723	-0.005538904	-0.11479	0.034919	0.067389	-0.08963
PTPRK	-0.014871693	-0.109583686	0.170548	-0.03599	0.091595	-0.22724
PTPRM	-0.002362905	0.00900998	0.157402	0.033265	-0.05886	-0.19125
PTPRN	-0.038422663	-0.020079463	0.038224	0.002557	0.005631	0.073467
PTPRN2	-0.009641282	0.043467588	-0.1277	-0.0041	-0.04519	0.011222
PTPRO	-0.005072876	-0.145588463	-0.35254	-0.08522	0.230034	0.143498
PTPRR	-0.021456007	0.02921234	-0.03866	0.152396	-0.15846	-0.06723
PTPRS	-0.000828017	-0.073013876	-0.08266	0.034092	-0.00887	-0.13925
PTPRT	-0.030341264	-0.025732946	0.081514	0.004463	-0.05161	-0.02616
PTPRU	-0.023954163	-0.075227139	-0.02252	0.000472	-0.09987	-0.07916
PTPRZ1	-0.097785402	-0.014763368	-0.01331	-0.02836	0.090234	-0.03759
PTRF	-0.035536093	-0.057147602	0.019389	-0.01478	-0.0285	-0.09471
PTRH1	-0.013736266	0.011924964	0.042361	0.038413	-0.12527	0.079868
PTRH2	0.026309973	-0.023311168	-0.11172	0.040115	-0.12171	0.359246
PTRHD1	0.031609132	0.008529833	0.052677	0.00856	-0.00349	0.248388
PTS	0.034494562	0.038490507	-0.07659	0.092226	-0.27867	-0.12642
PTTG1	0.018429356	-0.029013106	0.203574	0.13363	-0.39701	-0.31398
PTTG1IP	0.064888487	0.021113403	-0.28422	-0.01939	0.073537	0.240101
PTTG2	-0.022296481	-0.009970618	-0.15166	0.025352	-0.06391	-0.08952
PTTG3P	-0.021114498	-0.033777599	0.06758	0.219807	-0.48099	-0.13404
PTX3	-0.006258463	-0.095440153	-0.0678	-0.07371	0.205795	0.530272
PUF60	0.077386783	0.111546586	0.120838	0.11954	-0.32211	-0.01676
PUM1	0.053421219	0.064168432	0.146932	-0.04786	0.054359	-0.05624
PUM2	0.086753109	0.222648214	0.056866	0.05418	-0.0964	-0.44608
PURA	0.012372695	0.127577718	0.11063	0.020521	-0.01809	-0.02429
PURB	-0.030835659	-0.05099938	-0.07482	-0.00398	0.035955	0.092488
PURG	-0.056746138	-0.044743601	0.02859	0.0071	-0.03999	0.014594
PUS1	0.015384319	-0.05734534	0.129071	0.027834	-0.08424	0.003531
PUS10	-0.019281399	0.13248627	-0.12193	0.067706	-0.16003	-0.00665
PUS3	0.029684019	0.054316825	0.200085	0.022626	-0.0479	0.133629
PUS7	0.004658926	0.029820717	0.047652	0.053923	-0.14711	0.062064
PUS7L	0.000867459	0.125782699	-0.00159	0.079608	-0.22273	-0.06287
PUSL1	0.006771976	-0.080621856	-0.16183	0.089672	-0.23476	0.299917
PVALB	-0.02374017	-0.008975827	0.007212	-0.24663	0.705572	0.03152
PVR	-0.032572587	-0.070109014	-0.07216	-0.03536	-0.05465	0.073919
PVRIG	0.009418143	0.200531464	0.375925	-0.01161	-0.00208	-0.39249
PVRL1	-0.04516475	-0.102424428	-0.08962	0.010521	-0.04349	-0.0419
PVRL2	-0.029181982	-0.051221893	-0.1162	-0.01352	-0.00765	0.081258
PVRL3	-0.01873633	-0.024672169	0.090931	0.123809	-0.26263	-0.11534
PVRL3-AS:	-0.026554466	-0.09781511	0.068855	0.015253	-0.04269	-0.10358
PVRL4	-0.025612862	-0.054343133	0.005794	0.030312	-0.15485	-0.08094
PWAR5	-0.014999902	0.02603779	0.311039	-0.01754	0.057441	-0.29196
PWAR6	0.000317067	0.024435979	0.226827	-0.02973	0.107815	-0.3217
PWP1	0.037706718	0.091895984	0.141982	0.046767	-0.14117	-0.18349
PWP2	0.005526109	-0.059989576	0.131208	0.001373	-0.03532	0.046139
PWRN2	-0.072393867	-0.00783103	-0.02339	-0.02964	0.10894	0.078276
PWWP2A	0.038821945	0.123666836	-0.09659	-0.0472	0.037371	0.112832

PWWP2B	0.016900136	-0.057029779	-0.07199	-0.01179	-0.02843	0.085597
PXDC1	-0.01165175	-0.077468801	-0.07998	-0.06224	0.086192	-0.03492
PXDN	-0.025772354	-0.022968829	0.069531	0.014628	-0.05261	-0.18629
PXDNL	-0.056729608	-0.029455458	0.022898	0.034974	-0.0224	-0.01008
PXK	0.020812439	0.031433457	-0.40629	-0.00969	0.02646	0.113254
PXMP2	0.004398019	-0.016961753	-0.05241	0.113247	-0.36477	-0.11743
PXMP4	-0.090378479	0.129454573	0.25985	-0.05182	0.16125	-0.03123
PXN	0.011572602	0.118500303	0.046471	-0.0248	0.066356	0.074313
PXN-AS1	-0.015541135	-0.026913907	0.159444	0.093638	-0.2467	-0.23767
PXT1	-0.038557925	-0.042586228	0.107594	0.043273	-0.10656	-0.09672
PXYLP1	0.007030548	0.094408082	0.253027	0.048128	-0.11178	0.061556
PYCARD	0.021766757	-0.031876675	-0.22788	-0.05613	0.153693	0.282108
PYCARDOS	-0.004112484	-0.0250048	-0.0294	-0.03784	0.10848	-0.10108
PYCR1	-0.007556344	-0.048013427	-0.00584	0.083031	-0.30143	-0.06314
PYCRL	-0.018769159	0.00886468	-0.05601	0.072547	-0.19802	-0.17943
PYDC1	-0.030117293	-0.018600758	-0.12961	-0.0019	0.035006	0.219827
PYGB	0.003798609	-0.050046223	0.104358	-0.01566	-0.00575	0.014622
PYGL	0.004039569	-0.115185752	-0.29253	-0.0745	0.211492	0.381354
PYGM	-0.014110193	-0.017706043	0.121045	-0.05459	0.147018	-0.20668
PYGO1	-0.083456285	-0.104606778	-0.05451	-0.05636	-0.03142	-0.05013
PYGO2	0.000276297	-0.120604762	0.018677	0.016587	-0.12152	-0.10974
PYHIN1	0.003447034	0.306889416	0.317203	0.004188	-0.00921	-0.31251
PYROXD1	0.038119505	0.093535789	0.198738	-0.04097	0.106059	-0.02741
PYROXD2	-0.00837902	0.073380434	0.094236	-0.04507	0.089581	-0.27171
PYY	-0.045071739	-0.056585637	-0.13534	0.020507	-0.06491	0.130128
PYY2	-0.029756881	-0.077837079	-0.05296	0.004068	-0.02602	0.116056
PZP	-0.021659841	-0.065085421	-0.07581	-0.0015	-0.0107	-0.04677
QDPR	0.011486979	-0.045666868	0.110157	-0.03921	0.086073	0.074989
QKI	0.028302522	0.073507072	-0.45305	-0.03178	0.180153	0.319871
QPCT	0.006993494	-0.087218379	-0.31468	-0.08266	0.230816	0.497775
QPCTL	-0.02709995	-0.053339306	-0.07156	0.031677	-0.10643	-0.02874
QPRT	0.00029088	0.031490531	-0.1082	0.009347	-0.04189	0.109058
QRFPR	-0.040582449	-0.076511552	-0.03827	0.015536	-0.04011	-0.11167
QRICH1	0.061314161	0.038423559	0.1831	0.024586	-0.11071	-0.32391
QRICH2	-0.026684964	-0.011239238	0.011842	0.039782	-0.09487	0.042582
QRSL1	-0.021424001	-0.013306595	-0.07577	0.027208	0.071119	-0.20069
QSER1	0.010604452	0.029690481	-0.18487	-0.06958	0.06243	0.394118
QSOX1	0.020145389	-0.004171171	-0.12469	0.012594	-0.03535	0.209962
QSOX2	0.006132494	-0.04253013	0.006032	-0.00034	-0.04366	-0.18968
QTRT1	0.000896616	-0.11015797	0.273164	0.02076	-0.09366	-0.10162
QTRTD1	-0.017769915	-0.048313814	-0.06589	-0.02493	0.068483	0.102789
R3HCC1	0.034750433	0.084416527	0.089886	0.066633	-0.16955	0.095277
R3HCC1L	0.002545995	0.094965971	-0.15481	0.005526	-0.00458	0.014996
R3HDM1	0.021178139	0.043852708	0.0809	0.055741	-0.13909	0.000531
R3HDM2	0.037210493	0.011468404	-0.006	-0.02034	0.056211	-0.00981
R3HDM4	0.039268432	-0.067433315	-0.03403	-0.01214	0.046717	-0.24329
R3HDML	-0.032983067	-0.056958632	-0.09045	-0.02709	0.076969	-0.03807
RAB10	0.052220211	0.044477332	-0.31651	0.033282	-0.07688	0.402129
RAB11A	0.033511122	-0.04697418	-0.45467	-0.03466	0.101191	0.198373
RAB11B	-0.020426943	-0.11852793	-0.07913	-0.01276	0.016065	0.112696
RAB11B-AS	-0.007110855	0.009529754	-0.05691	-0.023	0.099004	0.287694

RAB11FIP1	0.015120885	0.067848537	-0.4521	0.003905	-0.05762	0.158914
RAB11FIP2	0.04181248	-0.028233096	-0.05572	-0.06945	0.203129	0.049175
RAB11FIP3	0.051993244	0.121691958	0.211018	0.031506	-0.05095	-0.22465
RAB11FIP4	-0.007391225	0.015631396	0.034573	-0.05969	0.130239	-0.19677
RAB11FIP5	-0.001230907	0.010752383	0.188684	-0.04104	0.064272	-0.18573
RAB12	0.017643746	-0.015216331	-0.13284	-0.04824	0.107352	0.264136
RAB13	0.015106755	-0.184771527	-0.08596	-0.09037	0.227729	0.403234
RAB14	-0.026772207	0.11059419	-0.22256	0.026222	-0.00027	0.053607
RAB15	0.010666835	-0.051829868	0.048903	-0.01701	0.013521	-0.3133
RAB17	-0.033907333	-0.047025481	0.065071	0.011604	-0.02658	-0.09163
RAB18	0.037346896	0.014296792	-0.06249	-0.03074	0.081075	-0.04715
RAB1A	0.047326976	0.027103702	-0.31606	0.020638	-0.04659	0.274878
RAB1B	0.055150445	0.06684539	-0.09597	0.046854	-0.1165	0.059112
RAB20	-0.002451054	-0.09860877	-0.1635	-0.05963	0.149303	0.352997
RAB21	0.001994929	0.040509245	-0.13265	-0.01462	0.018025	0.019543
RAB22A	0.063339102	0.186044555	0.018346	0.0122	-0.04897	-0.28649
RAB23	0.039498001	0.007650223	-0.0518	0.048143	-0.33476	-0.05619
RAB24	0.030680126	-0.032240053	-0.30686	-0.03098	0.09832	0.314347
RAB25	-0.03559379	-0.038980776	0.1112	-0.04554	0.171872	-0.05494
RAB26	-0.039499895	-0.064751577	0.002073	0.005997	-0.04124	-0.13688
RAB27A	0.038968455	0.138640404	-0.00643	0.014467	-0.06197	0.307765
RAB27B	-0.01641268	0.252207208	-0.13846	-0.14457	0.464598	-0.01402
RAB28	-0.005606389	0.072283955	-0.08474	0.065839	-0.17696	0.033325
RAB29	-0.002342058	0.18630377	0.254275	-0.04488	0.006137	-0.29766
RAB2A	0.114968717	0.073528126	-0.30505	0.058419	0.035743	0.081818
RAB2B	0.04379113	0.117296262	0.140553	0.040644	-0.14982	-0.42685
RAB30	-0.031498336	-0.10579977	0.094544	-0.06121	0.180415	-0.36684
RAB30-AS1	0.006398521	-0.000902695	0.019612	0.00709	-0.03166	-0.01915
RAB31	0.0081179	-0.155483889	-0.43784	-0.1049	0.387348	0.371597
RAB32	0.008207942	-0.132119814	-0.27506	-0.12127	0.322006	0.529495
RAB33A	0.004678953	0.073574867	0.294289	0.052865	-0.16877	-0.29285
RAB33B	0.015421948	0.003445953	-0.22597	0.007139	0.027973	0.071987
RAB35	0.025255376	0.072941782	-0.08867	0.005518	-0.07026	-0.06038
RAB36	-0.027438361	0.011118368	-0.08408	-0.02843	0.01681	-0.18407
RAB37	0.010455098	0.080149331	0.091401	-0.05853	0.146161	-0.08484
RAB38	-0.007421815	0.09285195	-0.11409	0.00943	-0.06077	-0.07867
RAB39A	-0.023522524	-0.050592453	-0.06614	-0.03535	0.072289	0.084131
RAB39B	-0.001890047	0.135214594	0.244052	0.068791	-0.15321	-0.39285
RAB3A	-0.027153214	-0.113490597	-0.01405	0.02866	-0.07207	0.150022
RAB3B	-0.073985827	-0.037196267	0.064402	0.00987	-0.1128	-0.02986
RAB3C	-0.067352152	-0.136441803	-0.12478	0.028681	0.05154	-0.01357
RAB3D	0.001792986	-0.092693611	-0.29493	-0.06793	0.174286	0.455149
RAB3GAP1	0.005254673	-0.145082221	-0.00451	-0.00373	0.007825	0.108153
RAB3GAP2	-0.024425821	0.021271703	-0.03501	0.008275	-0.01431	-0.06497
RAB3IL1	0.006572764	-0.035020766	-0.03656	0.000974	-0.09849	-0.1021
RAB3IP	-0.026085966	-0.056713529	0.259916	-0.02874	0.03662	-0.08293
RAB40A	-0.044411439	-0.08384492	-0.0203	0.005981	0.017628	-0.03562
RAB40AL	-0.037582487	-0.003781749	-0.08714	0.041875	-0.09952	-0.08016
RAB40B	0.001508872	0.066921922	0.084415	-0.04337	0.176808	-0.13713
RAB40C	0.038806009	0.013993194	-0.1418	0.014337	-0.0012	0.118566
RAB42	-0.003936773	-0.043230135	0.034553	-0.0113	-0.06858	-0.13319

RAB43	-0.010062444	-0.085729647	-0.09068	-0.01149	-0.02922	0.073798
RAB4B	-0.015840198	-0.024108569	0.062812	-0.03419	0.014843	-0.0924
RAB5A	0.015421846	0.023827092	-0.13713	0.038375	-0.13109	0.036587
RAB5B	0.024353728	0.013087225	-0.13086	-0.03212	0.063554	-0.09752
RAB5C	0.046631505	-0.019129756	-0.18598	-0.03765	0.090894	0.094447
RAB6A	-0.02700581	0.055964406	-0.04877	-0.00509	0.039004	0.06996
RAB6B	-0.03279422	-0.088204946	0.025501	-0.07174	0.14317	0.075601
RAB7A	0.003530441	-0.060868757	-0.03355	-0.02685	0.002236	0.102771
RAB7B	-0.013001369	-0.075050083	-0.1208	-0.00901	0.023718	-0.09743
RAB8A	0.086851959	0.16368645	-0.49758	0.0327	-0.06211	0.150295
RAB8B	0.073805013	0.118631976	-0.13493	-0.03939	-0.03993	0.131313
RAB9A	0.026684603	0.146715767	0.024859	-0.00466	-0.01189	0.159533
RAB9B	-0.024577528	0.035273259	0.069719	0.049139	-0.12316	-0.17905
RAB9BP1	-0.07136079	-0.004488331	0.004678	-0.04302	0.087986	0.09493
RABAC1	0.043648901	0.067560516	0.101101	0.0641	-0.14958	0.062052
RABEP1	0.000666404	0.106462101	-0.02672	-0.00867	-0.08033	-0.19437
RABEP2	0.074179387	0.034279036	-0.06021	-0.01119	0.069709	-0.09404
RABEPK	0.008236768	-0.014103709	0.22559	0.057606	-0.20621	-0.11275
RABGAP1	0.03903246	0.019605287	-0.00669	-0.00316	0.028334	0.027759
RABGAP1L	-0.07600157	0.034288334	-0.15774	-0.02947	0.281705	0.172381
RABGEF1	0.009255981	-0.074842555	-0.04053	-0.01018	-0.02769	0.166101
RABGGTA	0.023708657	0.049331114	-0.14268	0.048069	-0.10708	0.347177
RABGGTB	0.04084215	0.005467081	0.07995	0.044853	-0.12694	0.045462
RABIF	0.043125925	0.18480148	-0.21648	0.029152	-0.10628	0.203252
RABL3	0.013071125	-0.032908402	0.214648	0.073374	-0.1239	-0.17766
RABL6	0.07377351	-0.014848872	0.015736	0.114981	-0.22102	0.193332
RAC1	0.071029883	0.098561064	-0.35952	0.042312	-0.02417	0.361775
RAC2	0.034013752	0.134306925	0.042424	0.029482	-0.04411	-0.23015
RAC3	-0.028043658	0.024019539	-0.06394	0.028858	-0.04746	0.077044
RACGAP1	0.014820669	0.091594565	0.075851	0.112069	-0.30677	0.032831
RACGAP1P	-0.055278704	-0.032947646	0.020027	0.003901	-0.01458	-0.02122
RAD1	0.034530136	0.070335922	0.085929	0.066817	-0.27125	-0.08772
RAD17	0.031447262	0.059036995	0.156889	0.048611	-0.07142	-0.32992
RAD18	0.000372539	0.052727799	-0.0387	0.13544	-0.40629	-0.04174
RAD21	0.063626938	0.127464772	0.083137	0.095549	-0.23532	-0.35598
RAD21L1	-0.086202156	-0.01260836	-0.06803	-0.03986	0.123107	-0.02984
RAD23A	0.035806309	0.022257756	0.121507	0.061262	-0.16355	0.127328
RAD23B	0.063047655	0.16852418	-0.22995	0.071455	-0.20184	0.213601
RAD50	-0.008926815	0.01020912	0.150731	0.012484	-0.12757	-0.0496
RAD51	-0.013457592	0.049290324	-0.00171	0.149408	-0.41452	-0.03049
RAD51-AS	-0.008579562	0.090295509	0.176944	-0.06647	0.17586	-0.19449
RAD51AP1	0.000994624	0.030280772	-0.09639	0.098925	-0.25905	-0.08107
RAD51B	0.009984924	-0.067567968	0.060402	0.162199	-0.16584	-0.07595
RAD51C	0.012385851	0.047920574	0.168632	0.101548	-0.27664	-0.05668
RAD51D	-0.013896346	0.130964396	0.094193	0.063834	-0.20668	-0.22743
RAD52	-0.007145439	-0.026793552	0.119173	0.009019	0.005832	-0.03748
RAD54B	-0.008384201	0.006376063	0.113938	0.095766	-0.26376	-0.14664
RAD54L	-0.010355001	0.001643609	-0.01201	0.140066	-0.43682	-0.03383
RAD54L2	0.022139655	0.01514679	0.107523	0.030708	-0.18977	-0.24024
RAD9A	0.014199136	0.075600696	0.042293	-0.02503	0.028389	-0.13556
RAD9B	-0.05816688	-0.020505779	-0.02455	0.027191	-0.09923	0.103724

RADIL	-0.032409337	-0.02737191	-0.21205	-0.01634	0.007883	-0.01096
RAE1	0.042644646	0.040957552	-0.02158	0.082507	-0.26988	-0.00098
RAET1E	-0.034892126	0.014713297	-0.03913	0.000196	0.021828	-0.03599
RAF1	0.046534376	-0.017254877	-0.14592	-0.00337	0.02395	-0.11385
RAG1	-0.042140952	-0.038672831	-0.02069	0.008077	-0.02522	-0.09632
RAG2	-0.075122066	-0.01548694	-0.09193	-0.02123	0.043738	0.099395
RAI1	-0.01868525	-0.066483311	-0.07991	0.017069	-0.11819	-0.08275
RAI14	-0.020913515	0.09768968	-0.04094	0.040523	-0.08632	0.001557
RAI2	-0.01895564	-0.049941608	0.119339	-0.02499	0.058901	-0.05893
RALA	0.043391052	0.050291158	0.140523	0.038116	-0.11856	0.035569
RALB	0.022318388	-0.029633617	-0.34926	0.017123	-0.0476	0.166887
RALBP1	-0.002344035	0.124458516	-0.11099	-0.01127	-0.0501	-0.03649
RALGAPA1	0.032387808	-0.008841991	0.071184	0.077732	0.026297	-0.0768
RALGAPA2	-0.000812199	-0.096863541	-0.05676	-0.04583	0.144467	-0.00678
RALGAPB	0.050775621	0.007983605	-0.13727	0.020494	-0.19505	-0.09448
RALGDS	0.036799923	0.225138105	0.318984	-0.03	0.055729	-0.33679
RALGPS1	0.01921064	0.047831139	-0.05534	0.067395	0.02491	-0.18306
RALGPS2	-0.016213854	-0.055555654	-0.17176	-0.07085	0.210976	-0.24613
RALY	0.068347251	0.11461202	-0.03474	0.072527	-0.20125	-0.08914
RALY-AS1	-0.015924259	-0.044506236	-0.15614	0.038373	-0.08924	0.349381
RALYL	-0.017987507	-0.027437254	0.050185	0.079984	-0.19964	-0.0362
RAMP1	0.009061253	0.133838371	-0.078	-0.0172	-0.00635	-0.13315
RAMP2	-0.031025748	-0.090162453	-0.12903	0.003894	-0.02207	-0.0857
RAMP2-AS1	-0.035996322	-0.026948546	0.056701	0.01089	3.04E-05	-0.10126
RAMP3	-0.025397361	-0.075719893	0.055461	-0.03202	0.056073	-0.01878
RAN	0.030193631	0.05726861	0.138797	0.059486	-0.1517	0.051927
RANBP1	0.017727539	-0.050109621	0.002152	0.051971	-0.12375	-0.05949
RANBP10	0.003952518	0.031156053	0.04202	0.024781	0.034575	-0.03115
RANBP17	-0.049802381	0.162767706	0.034341	0.02021	-0.04817	-0.03267
RANBP2	0.032228083	0.034307399	0.166065	-0.00337	-0.09665	-0.22628
RANBP3	0.017806545	-0.063429284	0.088338	0.027341	-0.10059	0.028218
RANBP3L	-0.028587727	-0.043290478	-0.05486	0.07749	-0.13999	-0.02948
RANBP6	0.033133657	0.070930439	0.217031	0.022864	-0.04646	0.0129
RANBP9	0.022646922	0.092130372	0.129932	0.056947	-0.16047	-0.18095
RANGAP1	-0.005766889	-0.008356827	0.025056	0.060128	-0.29101	0.009605
RANGRF	0.021202499	0.08074455	0.237597	0.048949	-0.16477	-0.14799
RAP1A	0.004990672	0.010440987	0.06258	-0.03209	0.105803	-0.04746
RAP1B	0.084063039	0.369226523	0.11455	0.027092	0.003894	-0.09236
RAP1GAP	-0.036612825	-0.112126	-0.0148	-0.00187	-0.07349	-0.06835
RAP1GAP2	0.016815075	0.114192069	-0.03847	-0.09768	0.271883	0.02754
RAP1GDS1	0.037798045	0.11918034	-0.0458	0.010209	-0.03679	-0.0717
RAP2A	-0.067684198	-0.029268564	0.026374	0.001868	-0.09178	-0.03407
RAP2B	0.015336495	0.195179864	-0.07057	-0.06414	0.194581	0.3627
RAP2C	0.072203021	0.150498229	-0.19006	0.052512	-0.11902	-0.0602
RAP2C-AS1	-0.019171038	0.011615464	0.003147	-0.00263	0.02815	0.218875
RAPGEF1	-0.017373773	0.109849591	-0.07861	0.008448	-0.02137	-0.15543
RAPGEF2	-0.019010226	-0.072948088	-0.19027	0.032294	0.001212	0.111911
RAPGEF3	-0.039766016	-0.071649947	0.022309	0.026376	-0.09346	-0.03101
RAPGEF4	-0.117498248	-0.037700657	-0.00561	-0.00144	0.0528	-0.12047
RAPGEF4-A	-0.033170005	-0.064144816	-0.00785	0.012614	-0.00959	-0.0598
RAPGEF5	-0.054403872	0.004127581	0.0064	0.06659	-0.09419	-0.05232

RAPGEF6	-0.013341509	0.036158951	0.33694	-0.04628	0.110991	-0.38092
RAPGEFL1	-0.02299824	-0.114336337	-0.03268	0.024438	-0.05794	-0.02406
RAPH1	-0.010119452	-0.044190494	-0.0621	-0.01758	0.031084	0.236061
RAPSN	-0.039842299	-0.030398273	-0.09185	0.031398	-0.05966	0.278009
RARA	0.038796408	-0.072425695	-0.14124	-0.05564	0.15716	0.05179
RARA-AS1	-0.002623453	-0.116150435	-0.19668	-0.05791	0.143174	0.272463
RARB	-0.047848688	-0.010118971	-0.12974	0.023102	0.026566	0.016152
RARG	-0.022545776	-0.052832897	0.033041	0.011953	-0.06032	-0.08747
RARRES1	0.017656409	-0.019570534	0.019641	-0.01817	0.016477	0.00334
RARRES2	-0.028486441	0.032093194	0.070147	0.021874	-0.06823	-0.05454
RARRES3	0.019280994	0.176815771	0.359911	-0.01323	0.028618	-0.02669
RARS	0.047136593	0.020749139	0.122192	0.072997	-0.22262	0.074391
RARS2	-0.024047717	-0.012530433	0.148396	0.009836	-0.01198	-0.00854
RASA1	0.034334212	0.039626752	0.039304	0.034269	-0.09135	0.008683
RASA2	0.039650985	0.133784499	0.201539	0.002138	0.026018	-0.29807
RASA3	0.001548639	0.021856564	0.078107	-0.12987	0.350324	-0.0456
RASAL1	-0.023459765	-0.046040616	0.025673	0.000732	-0.02593	-0.03736
RASAL2	-0.00959351	-0.158161805	-0.05886	-0.08494	0.044583	0.152307
RASAL3	0.034099156	0.124312126	0.347579	-0.00467	-0.01629	-0.37318
RASD1	-0.000514385	-0.071687808	-0.03613	0.006426	-0.04025	-0.16073
RASD2	-0.029314734	0.033593486	-0.05644	-0.0035	0.025228	0.031032
RASEF	-0.019782229	-0.020781528	-0.02374	0.146978	-0.24277	-0.0617
RASGEF1A	0.000294657	0.164793342	0.186313	-0.04916	0.142495	-0.25546
RASGEF1B	-0.017284913	-0.073439752	-0.14677	-0.00987	0.02562	0.118583
RASGEF1C	-0.034421697	-0.056908665	-0.12666	0.005394	0.003894	-0.05079
RASGRF1	-0.058467828	-0.126386381	-0.12426	-0.01451	0.067427	0.073712
RASGRF2	-0.007474795	-0.015152023	0.463911	-0.05025	0.102618	-0.22923
RASGRP1	0.013595521	0.152638021	0.402136	-0.01209	0.023005	-0.43901
RASGRP2	0.004012711	-0.04824588	0.114719	-0.08097	0.192651	-0.05473
RASGRP3	-0.006521641	-0.072505365	-0.10688	-0.04991	0.118161	-0.15292
RASGRP4	-0.022873132	-0.100427534	-0.25327	-0.08806	0.218826	0.221864
RASIP1	-0.03473594	-0.035950217	-0.06019	0.007078	-0.00604	0.116056
RASL10A	-0.023229523	-0.053798924	-0.06702	0.015962	-0.04912	0.086369
RASL10B	-0.026605494	-0.100616525	-0.01649	-0.01823	-0.03013	0.000692
RASL11A	-0.023784307	-0.043910121	-0.04491	0.019302	-0.03077	0.25196
RASL11B	-0.038436476	-0.082842029	0.008852	-0.00823	-0.02171	-0.03549
RASL12	-0.028839481	-0.035340905	0.030195	-0.04093	0.124459	-0.05515
RASSF1	0.047409033	0.21632209	0.121889	0.005003	-0.03173	-0.12173
RASSF1-AS	-0.015632159	-0.119153397	-0.15176	0.010323	-0.00713	0.184793
RASSF10	-0.062542692	0.028209057	0.068119	-0.02954	0.080845	-0.03325
RASSF2	0.033253469	-0.193731108	-0.36548	-0.0615	0.172269	0.233671
RASSF3	0.047205753	0.023116218	-0.09977	-0.05527	0.171471	0.131844
RASSF4	0.028625197	0.140017788	-0.24436	-0.05029	0.207457	0.524546
RASSF5	0.033520634	0.130196693	0.081952	-0.00918	0.014529	-0.32411
RASSF6	0.016109471	-0.025315906	-0.08155	-0.04751	0.016957	-0.18091
RASSF7	0.039172368	-0.082781071	0.175846	-0.01732	-0.01789	-0.33395
RASSF8	-0.017400814	0.019589021	-0.12549	0.022595	-0.08649	0.057964
RASSF8-AS	-0.03241959	-0.035130463	0.00233	0.003853	-0.00825	-0.11716
RASSF9	-0.117683609	-0.055803363	-0.04873	0.010633	0.057098	-0.0274
RAVER1	-0.000409837	-0.07085089	-0.04787	0.057939	-0.22801	-0.03051
RAVER2	-0.023617053	-0.080245437	-0.07913	-0.03121	0.084724	-0.00262

RAX	-0.028030938	-0.042140336	0.125068	-0.02197	0.056953	0.009375
RAX2	0.00281234	-0.077548774	0.000548	-0.02624	-0.04515	-0.15521
RB1	0.026131093	-0.032140414	-0.27359	0.035238	-0.09556	0.22714
RB1CC1	0.055300971	0.102370598	-0.12739	0.010891	-0.00929	-0.11111
RBAK	-0.031309722	0.15219567	0.0738	-0.06247	0.081978	-0.02253
RBAKDN	-0.023291016	-0.096234792	0.072421	-0.00053	-0.06075	-0.03008
RBBP4	0.014009184	0.038421232	0.196072	-0.01409	-0.0293	-0.12741
RBBP5	0.017948371	0.07322573	-0.05665	0.030236	-0.0826	-0.15006
RBBP6	0.043279978	0.012099469	0.17457	-0.06168	0.027414	-0.33031
RBBP7	0.057736877	0.147063186	0.170244	0.075388	-0.21332	-0.3156
RBBP8	0.020881791	-0.026454612	-0.34331	0.074028	-0.21619	0.312687
RBBP8NL	-0.024407884	-0.100812214	-0.00107	0.02068	-0.05712	0.061321
RBBP9	-0.045546867	0.006315759	-0.10556	0.170608	-0.20366	0.093482
RBCK1	0.062389132	0.000488823	-0.14251	-0.01505	0.024579	0.037895
RBFA	0.003994113	-0.016716681	0.182539	0.046803	-0.14697	-0.07685
RBFADN	-0.051235157	-0.081688667	0.038654	0.037982	-0.11872	-0.00139
RBFOX1	-0.039333525	0.000211225	-0.0082	0.001093	0.02465	-0.10584
RBFOX2	-0.034645815	0.055901335	0.029524	-0.01973	0.11139	-0.00285
RBFOX3	-0.038139809	0.141021467	-0.03712	0.011143	-0.00443	0.063478
RBKS	-0.00263288	-0.043873328	0.098981	0.040752	-0.18304	-0.04076
RBL1	-0.00347671	0.064522021	0.110216	0.05622	-0.18606	-0.13977
RBL2	0.032309671	0.112146163	0.337124	-0.03382	0.089523	-0.31017
RBM10	0.061763463	0.042526955	0.034324	0.069238	-0.27869	-0.13068
RBM11	-0.029219043	-0.129126712	0.459446	-0.06888	0.198496	-0.31392
RBM12	0.037578438	0.079928527	0.095385	0.039059	-0.09558	-0.1713
RBM12B	0.009122558	0.072033798	0.072894	0.030164	-0.07087	-0.0247
RBM12B-AS	-0.035619462	-0.06751534	-0.04127	0.010087	-0.00651	0.111753
RBM14	0.004754191	-0.034533732	0.078179	0.01419	0.001769	-0.18794
RBM15	0.018321619	0.135859817	-0.04051	0.112322	-0.24221	-0.29327
RBM15B	0.037386536	-0.024011997	0.112809	-0.00247	-0.08599	-0.05147
RBM17	0.033807389	0.024599778	0.174966	0.003932	-0.06823	0.048157
RBM18	-0.001037127	0.068254775	-0.07954	-0.05367	0.165585	0.02078
RBM19	0.005434433	-0.027600633	0.025941	0.025075	-0.09888	0.039086
RBM20	-0.043052768	-0.075287384	0.211393	0.00359	-0.01237	0.023563
RBM22	0.007962094	0.177214684	-0.10737	0.092045	-0.19302	-0.26734
RBM23	0.071015524	0.118865328	-0.34211	0.029909	-0.05123	0.057788
RBM24	-0.055507371	-0.052520737	0.11992	-0.05786	0.106712	-0.02504
RBM25	-0.001641522	0.082160611	0.113783	-0.00837	-0.0797	-0.15994
RBM26	0.024502872	0.009498879	0.120882	0.071253	-0.11514	-0.20605
RBM26-AS1	-0.020664021	-0.056929337	0.21088	-0.00609	0.028872	-0.23676
RBM27	0.058894192	0.074616507	0.192289	0.019524	-0.08065	-0.20505
RBM28	0.026879507	0.099132893	0.000833	0.081911	-0.23418	0.056827
RBM3	0.046060469	0.073743242	0.108995	0.006095	0.001095	0.134942
RBM33	-0.069043759	0.011632638	-0.10633	0.036088	-0.09857	0.02182
RBM34	-0.013999975	0.020435274	0.051815	0.008673	-0.01996	-0.01115
RBM38	0.026487737	0.101688113	0.17088	-0.00756	0.003243	-0.22916
RBM39	0.049289196	0.125846155	0.094819	-0.01993	0.07459	-0.35654
RBM4	0.024949331	-0.022578011	0.259913	-0.02127	0.083062	-0.11495
RBM41	0.011001138	0.028817201	0.04419	-0.01481	0.081971	0.092924
RBM42	0.040956727	-0.000438449	-0.06536	0.077857	-0.24055	0.238566
RBM43	0.003140394	0.062251425	0.235833	-0.03544	0.114055	0.050717

RBM45	0.016913592	-0.000661234	0.043244	0.042439	-0.08446	0.004779
RBM46	-0.108928639	-0.091356422	-0.01607	-0.04249	0.128223	-0.01961
RBM47	0.075324673	-0.10115629	-0.48354	0.066124	0.130296	0.141167
RBM48	0.00383419	0.097830977	0.165517	-0.01314	0.074612	-0.37749
RBM4B	0.001736025	0.059119633	-0.20191	-0.02468	-0.00939	-0.15701
RBM5	0.036520016	0.1160223	0.004404	-0.03714	0.13483	-0.1567
RBM6	0.024772016	0.00374947	-0.06831	-0.06022	0.186138	0.080223
RBM7	0.048022499	0.102823607	-0.07683	0.080871	-0.22838	-0.06738
RBM8A	0.052968376	0.17987841	0.083836	0.044976	-0.11176	-0.13285
RBMS1	0.064085596	0.088453187	0.070101	-0.02329	0.092496	0.035338
RBMS2	-0.031828893	0.010914507	-0.14388	0.003485	0.038995	0.436021
RBMS3	-0.06757758	-0.101247391	-0.00034	-0.01578	0.019558	-0.19497
RBMS3-AS:	-0.060398978	-0.045320244	0.015868	-0.01692	0.02914	0.093068
RBMX2	0.039120663	0.020362102	0.125899	0.011233	-0.0275	0.155252
RBMXL1	0.031236152	0.03554714	0.1146	0.033074	-0.09113	-0.13807
RBMXL2	-0.056888312	-0.028878617	-0.03316	0.029342	-0.05828	0.054353
RBMY3AP	-0.040501069	-0.073269445	-0.01255	-0.00779	0.055678	0.060717
RBP1	-0.048031614	-0.054148157	-0.04313	0.016188	-0.05803	-0.0727
RBP2	-0.036506462	-0.106154599	-0.0746	0.019787	-0.0459	-0.10823
RBP3	-0.035281941	-0.100086957	0.145018	0.029431	-0.06846	-0.1538
RBP4	-0.051138084	-0.113535302	-0.07358	-0.00283	0.046209	0.106413
RBP5	-0.03253272	-0.042592734	0.015229	0.014051	-0.05007	-0.06472
RBP7	0.000587963	-0.079526084	-0.31798	-0.07587	0.215507	0.693946
RBPJ	0.039075083	-0.035978922	-0.28145	0.049358	-0.11819	0.242421
RBPJL	-0.030381911	-0.038483544	0.048913	-0.02091	0.059212	0.005481
RBPMS	-0.008637048	-0.015855453	-0.03158	0.156696	-0.27136	-0.02211
RBPMS-AS:	-0.034244964	-0.040340901	0.076841	-0.02047	0.039827	-0.08072
RBPMS2	-0.011813667	0.164934436	-0.04206	-0.10236	0.278379	-0.04561
RBSG3	-0.054885794	-0.048835657	-0.03312	-0.01272	0.064255	0.025454
RBX1	0.069145606	0.061484229	0.053907	0.055072	-0.16139	0.174307
RC3H1	-0.004919965	0.021812043	-0.12281	-0.0195	-0.02771	-0.02163
RC3H2	-0.045420412	-0.077976096	-0.05835	-0.03791	-0.0021	0.088084
RCAN1	-0.004349843	0.028088788	0.020106	-0.06614	-0.04478	0.048448
RCAN2	-0.01670526	-0.014944179	0.069208	0.207007	-0.34522	-0.05321
RCAN3	0.006589951	-0.185600427	0.419578	-0.03385	0.109182	-0.20897
RCBTB1	-0.091895438	-0.05628685	0.018465	-0.05754	0.005111	-0.07559
RCBTB2	0.01700016	0.092076214	-0.08427	-0.01185	0.03407	0.416166
RCC1	0.002603605	-0.066406034	0.15631	0.072055	-0.26648	0.051529
RCC2	0.054112894	0.066686949	-0.02714	0.088834	-0.24485	0.126529
RCCD1	-0.007151693	-0.057743468	0.11741	0.094344	-0.32522	-0.17702
RCE1	-0.002209668	-0.099753517	0.045104	-0.00028	-0.11368	-0.1206
RCHY1	0.018208296	0.230750354	-0.03122	0.042702	-0.02057	0.085471
RCL1	0.006948911	-0.022329344	0.048053	0.049398	-0.15072	-0.20017
RCN1	0.012935971	0.063511514	-0.03677	0.069182	-0.17713	0.287719
RCN2	0.031049154	0.1673225	0.240071	0.041201	-0.12188	-0.40598
RCN3	-0.016595882	-0.133655219	-0.2138	-0.02495	0.002641	0.162102
RCOR1	0.022958052	-0.080941341	-0.39801	-0.0205	0.049556	0.374084
RCOR2	-0.01296691	-0.094454003	-0.01804	-0.00754	-0.08467	-0.08137
RCOR3	0.022521122	-0.002485884	-0.08737	-0.02215	0.073686	-0.12892
RCSD1	0.02949714	-0.006771253	-0.06127	-0.02005	0.056201	0.018727
RCVRN	-0.027494314	-0.016008187	-0.14633	0.020533	-0.03777	0.188012

RD3	-0.033911182	-0.095519218	-0.10376	0.002956	0.00227	-0.02448
RDH10	-0.005842636	0.030584215	0.003163	0.069985	-0.27675	0.005572
RDH11	0.013188126	0.021212503	0.152184	0.02981	-0.10457	0.058541
RDH12	-0.029123189	0.03055951	-0.01287	0.01378	-0.07122	-0.06733
RDH13	-0.07309552	0.021338511	0.135291	0.016863	-0.05146	-0.14883
RDH16	-0.028391005	-0.075466441	-0.00109	0.015257	-0.08524	-0.09429
RDH5	-0.001889968	0.017023475	-0.07455	-0.00099	0.016842	-0.01862
RDH8	-0.028127651	-0.065001791	-0.11589	0.017681	-0.07595	0.066371
RDM1	-0.016454995	0.007691164	0.103727	0.142665	-0.41537	-0.06761
RDX	-0.001187564	0.060960253	-0.06596	0.06993	-0.19845	0.16689
REC114	-0.043046537	-0.060367701	0.028896	0.018141	-0.03084	-0.06696
REC8	0.01080083	-0.093798675	0.008491	-0.02438	0.041627	-0.12475
RECK	-0.016378193	-0.001863132	0.100848	-0.01655	-0.01019	0.130534
RECQL	0.045627524	0.174020644	0.121653	0.012912	-0.05246	-0.04455
RECQL4	-0.008560056	-0.050912037	-0.06691	0.085933	-0.34314	-0.04296
RECQL5	-0.035040535	-0.052088214	-0.10373	0.029058	-0.07252	-0.17061
REEP1	-0.034516531	-0.056128566	0.098724	-0.14354	0.408594	-0.01329
REEP2	-0.01538896	-0.09144127	0.029162	0.001488	-0.1153	-0.15253
REEP3	0.01614378	-0.020107846	-0.08781	-0.05259	0.134114	0.30068
REEP4	0.011478277	0.026886974	-0.14705	0.044929	-0.17649	0.337695
REEP5	0.089016858	0.06675727	-0.26862	0.026149	-0.04677	0.39562
REEP6	-0.020737624	0.018173334	0.196445	-0.01351	0.062537	-0.06373
REG1A	-0.036021932	-0.04469439	-0.01422	0.009943	-0.03394	-0.01872
REG1B	-0.037782426	-0.015216826	0.023047	0.001173	0.026188	0.035511
REG1P	-0.023823239	-0.027822698	0.03534	0.022782	-0.05898	0.12575
REG3A	-0.051817386	-0.036436318	0.002256	0.025327	-0.03791	0.080261
REG3G	-0.028249762	-0.058527334	-0.09943	0.020903	-0.03955	0.056237
REG4	-0.102284191	0.007270451	0.03026	-0.03539	-0.10717	0.022017
REL	0.010897863	0.062320302	-0.19038	-0.03212	0.099465	0.082423
RELA	0.045646147	0.035893228	0.095787	0.001645	-0.03381	-0.20763
RELB	0.007394469	-0.051718007	-0.22351	0.030226	-0.12332	0.110434
RELL1	0.016832394	-0.044834215	-0.12379	0.011911	-0.01991	0.292014
RELL2	0.001473403	-0.204295078	0.288046	-0.03349	0.031714	-0.16656
RELN	-0.018932212	-0.022170636	0.042308	0.079922	-0.21876	-0.04018
RELT	0.020377393	-0.150894938	-0.26325	-0.01863	0.025556	0.113032
REM1	-0.025224402	-0.055118368	0.058782	-0.00651	-0.06653	-0.07805
REM2	-0.009229541	-0.074485781	-0.02623	-0.01286	0.025315	-0.3008
REN	-0.043514982	-0.039918267	-0.02332	-0.00148	0.027238	0.037139
RENBP	-0.007393497	-0.077692265	-0.08227	-0.07994	0.200818	0.37363
REPIN1	0.025207667	-0.078992831	-0.04319	0.012787	0.007441	-0.11237
REPS1	-0.016481647	-0.005545779	0.116929	-0.04238	0.10745	-0.04772
REPS2	-0.009924781	-0.123391145	-0.30008	0.007797	0.005803	0.096074
RER1	0.053506319	0.07879824	-0.15192	0.044913	-0.12316	0.097203
RERE	-0.063008743	-0.091942781	-0.05716	-0.07289	-0.02232	-0.03246
RERG	-0.110399751	-0.098384015	0.012807	-0.01982	0.058748	-0.09586
RERGL	-0.042349618	-0.026931863	-0.07745	0.05343	-0.15434	0.024413
REST	0.035187872	0.083209573	0.139673	0.01647	-0.10371	-0.1872
RET	-0.014869486	-0.068124454	-0.05388	0.03789	-0.1228	-0.00836
RETN	0.000852882	-0.125485714	0.072557	-0.02377	0.08794	0.682749
RETNLB	-0.05201401	-0.044349289	0.010253	-0.00804	0.00683	-0.00184
RETSAT	0.024207431	0.045780042	0.043734	0.03697	-0.12309	0.092296

REV1	0.023453345	-0.003171512	0.188829	-0.03348	0.116899	0.022792
REV3L	0.017372657	-0.038773665	0.073517	0.019309	-0.043	-0.07329
REXO1	-0.020548037	-0.109400911	-0.03959	0.005968	-0.0726	-0.0188
REXO1L1P	-0.031927415	-0.016169485	0.043931	0.01125	-0.00912	0.094717
REXO2	-0.045134868	0.021679369	0.255382	0.016829	-0.15965	-0.15455
REXO4	0.028243912	-0.024291596	0.213647	0.039911	-0.18884	-0.13876
RFC1	0.031514696	0.131693828	0.117851	0.049316	-0.12649	-0.1182
RFC2	0.005358476	0.076417926	-0.2341	0.105817	-0.29871	0.172646
RFC3	-0.043546524	-0.028435013	0.012941	0.220825	-0.18101	0.014676
RFC4	0.015777495	0.073672275	0.195215	0.085763	-0.22309	-0.23009
RFC5	-0.040206942	0.152703712	0.024951	0.133832	-0.29225	-0.13227
RFESD	-0.017510357	0.043519717	0.32233	-0.02571	0.082447	-0.309
RFFL	-0.007019932	0.007366142	-0.01239	0.029989	-0.06586	-0.13769
RFK	0.029540977	0.026349491	-0.10622	0.025148	-0.07832	0.360377
RFNG	0.014657835	-0.072066997	0.026348	0.001654	-0.01758	0.164278
RFPL1	-0.096905876	-0.083451993	-0.02133	-0.00685	0.021271	0.07408
RFPL1S	-0.019632783	-0.012841715	0.026285	0.08558	-0.18482	0.189991
RFPL2	-0.018341114	-0.055733753	0.110362	-0.02229	0.04	-0.01961
RFPL3	-0.02111733	-0.05167061	0.065277	-0.01875	0.019572	-0.03318
RFPL3S	-0.030075914	-0.065705094	0.205231	-0.05681	0.14999	0.018605
RFT1	-0.004771672	-0.071140877	-0.15811	0.075453	-0.2512	0.126642
RFTN1	0.026558038	0.181558628	0.089187	0.03222	-0.08262	0.008398
RFTN2	-0.076850938	-0.02579831	-0.00069	-0.01658	0.054052	-0.07995
RFWD2	0.047253672	-0.039292785	-0.32402	0.019666	-0.0281	0.164363
RFWD3	0.000626323	0.068218266	0.00266	0.112277	-0.3193	-0.06237
RFX1	0.02165302	-0.087453203	-0.01869	-0.0558	0.139899	0.219487
RFX2	-0.003957129	-0.104716162	-0.0383	0.007555	0.013615	0.152913
RFX3	0.036205101	0.057809161	0.079343	-0.01134	0.081661	-0.12131
RFX4	-0.061891766	-0.104814601	0.194092	-0.02985	0.098706	-0.03718
RFX5	0.041842126	-0.006575953	-0.08673	0.086234	-0.25079	-0.10149
RFX6	-0.039296022	-0.059865807	-0.07302	-0.00591	0.033966	-0.0527
RFX7	0.018038883	0.104563035	0.177251	0.055008	-0.11735	-0.04397
RFXANK	0.036557424	-0.0131242	0.02696	0.038661	-0.13277	0.033832
RFXAP	-0.000463173	0.090447608	0.052661	0.09544	-0.23131	-0.1108
RGAG1	-0.029156039	-0.014086821	0.03924	0.014982	-0.03556	-0.01412
RGAG4	-0.022143954	-0.123617409	-0.12299	-0.02263	0.05	0.241572
RGCC	0.005022228	-0.089986003	0.352275	-0.04423	0.113885	-0.1489
RGL1	0.004928963	-0.060808189	-0.0062	0.009303	-0.05575	0.115192
RGL2	0.036489124	-0.065121305	-0.2137	-0.0476	0.139687	-0.10247
RGL3	-0.018542413	-0.085532529	-0.08565	0.015113	0.007971	0.043976
RGL4	-6.40E-05	0.007269488	0.222263	-0.01074	0.003736	-0.28647
RGMA	-0.040390559	-0.07415701	-0.13374	0.013042	-0.03262	0.134248
RGMB	0.005620996	-0.144726143	0.213675	-0.06608	0.179493	-0.3167
RGMB-AS1	-0.035667885	-0.032862418	0.028543	-0.00011	0.023007	-0.04358
RGN	-0.033024466	-0.061641519	-0.04323	-0.02576	0.093076	0.011189
RGP1	0.027846122	0.032902498	0.199505	-0.01689	-0.01793	-0.23525
RGPD4-AS1	-0.064949026	-0.037067648	0.077122	-0.00021	0.00296	-0.06808
RGR	-0.046589805	-0.104204995	0.015346	-0.00976	0.027591	-0.05827
RGS1	0.004326126	-0.043000368	0.11761	0.100724	-0.30005	-0.24491
RGS10	0.019882296	-0.107955336	0.293579	-0.13662	0.282768	0.060193
RGS11	-0.042176873	-0.09838621	-0.087	-0.02482	0.029917	0.117224

RGS12	-0.030847708	-0.076417652	-0.05997	-0.01926	0.146033	0.103562
RGS13	-0.037908913	-0.09799747	-0.08599	0.017365	-0.02818	-0.00225
RGS14	0.001796149	-0.06632026	0.007574	-0.05494	0.111739	0.061046
RGS16	0.006933524	-0.034471025	-0.03396	0.056071	-0.21205	-0.0227
RGS17	-0.019200428	0.180657971	0.08907	-0.03385	0.099614	-0.17666
RGS18	0.00978719	-0.078641949	-0.35946	-0.07282	0.209013	0.281412
RGS19	0.04957181	-0.011568502	-0.31369	-0.04933	0.149948	0.19257
RGS2	0.02452905	-0.068951292	-0.3486	-0.08993	0.22703	0.318129
RGS20	-0.047181741	-0.064865259	0.032127	0.036652	-0.12039	-0.07639
RGS22	-0.035174866	-0.072941016	0.017232	0.034894	-0.07838	-0.00648
RGS3	0.003447082	0.234024805	0.026631	-0.01425	0.022189	0.056163
RGS4	-0.028575574	-0.041710152	-0.03525	0.004842	-0.1705	-0.00445
RGS5	-0.074678549	-0.067714873	0.176818	0.056205	-0.09469	-0.17488
RGS6	-0.029043604	-0.077610417	-0.05487	-0.00492	0.107652	0.107248
RGS7	-0.014064622	-0.080350342	-0.11264	-0.01407	0.054434	-0.18282
RGS7BP	-0.061395256	-0.0254529	-0.09369	-0.0121	0.024402	0.061348
RGS8	-0.033771126	-0.02167805	-0.02549	0.043996	-0.11411	0.059573
RGS9	-0.005929937	0.181321478	0.082662	0.019775	-0.05816	-0.09295
RGS9BP	-0.036933881	-0.021467879	-0.05352	0.009411	-0.00823	0.105101
RGSL1	-0.033448402	-0.036505143	-0.04722	-0.00234	-0.02358	-0.11318
RHAG	0.00699372	-0.044932526	0.019762	0.06584	-0.02932	-0.14654
RHBDD1	0.025448406	-0.111227481	0.021612	0.002689	-0.0093	0.00749
RHBDD2	0.019734277	-0.024125216	0.062131	0.045591	-0.16566	-0.06624
RHBDD3	-0.008539917	-0.065896525	0.02133	0.055518	-0.26751	-0.0796
RHBDF1	-0.049713391	-0.094312271	-0.13743	0.011078	-0.04456	-0.02766
RHBDF2	0.029499579	0.087640752	-0.19683	-0.0564	0.154652	0.214522
RHBDL1	-0.042533041	-0.09976654	-0.08651	0.051173	-0.1203	-0.045
RHBDL2	-0.040285275	-0.064271955	-0.14644	0.02085	-0.0369	-0.01854
RHBDL3	-0.050676225	-0.037560043	-0.04919	-0.00895	-0.03463	0.006436
RHBG	-0.014200637	-0.107496759	0.036443	-0.00109	-0.10268	-0.10583
RHCG	-0.036637678	-0.114937008	-0.14279	0.004885	-0.03513	0.043774
RHD	-0.012489646	-0.010443587	0.023749	-0.01182	0.034333	-0.01587
RHEB	0.023432905	-0.004739958	0.010118	-0.03609	-0.08805	0.077551
RHEBL1	-0.008066747	0.161592021	0.090816	0.139568	-0.39313	-0.2282
RHNO1	0.011778527	-0.032222343	0.164625	0.051484	-0.176	-0.04717
RHO	-0.0384855	-0.088339047	-0.03057	-0.01726	-0.0145	0.028413
RHOA	0.05181793	0.093926168	-0.06474	0.014525	-0.01924	0.028452
RHOB	-0.04128464	-0.051169933	-0.10993	-0.05193	0.027405	0.207857
RHOBTB1	-0.018034255	-0.041681061	-0.11287	-0.19828	0.565751	0.153308
RHOBTB2	-0.008655627	0.036533857	-0.18709	-0.01847	0.037181	-0.08186
RHOBTB3	-0.00168298	0.302856598	-0.17033	-0.04907	0.094231	-0.23684
RHOC	0.014383538	0.28162604	-0.02756	0.034452	-0.09929	0.145157
RHOD	0.019084111	-0.087124232	0.071547	0.008053	-0.05038	-0.13824
RHOF	0.006900286	0.131329718	0.227043	0.005104	-0.07648	-0.27756
RHOG	0.042148466	-0.027717502	-0.23606	-0.01485	0.05974	0.303262
RHOH	0.006209247	-0.014089088	0.346655	-0.02778	0.045147	-0.52947
RHOJ	-0.07752338	-0.076127684	-0.06645	-0.07619	0.032586	-0.04114
RHOQ	0.031412964	0.017954296	-0.50093	-0.083	0.214302	0.338331
RHOT1	0.049213934	0.027668166	-0.21071	0.014492	0.025879	0.319739
RHOT2	0.047377022	-0.041122437	0.152269	-0.02242	-0.01649	-0.28503
RHOU	0.004906901	-0.135419595	-0.04306	-0.02808	0.092095	0.497532

RHOV	-0.020860657	-0.057148649	-0.05341	-0.02092	-0.00278	-0.07574
RHOXF1	-0.031800651	0.025204957	-0.08031	-0.00059	-0.00461	-0.09497
RHPN1	-0.005821063	-0.196725823	0.048777	-0.01598	-0.03924	-0.17746
RHPN1-AS	-0.027239544	-0.027977318	0.093262	0.010922	-0.091	-0.12127
RHPN2	-0.028751171	-0.082192949	0.03051	-0.02166	0.029544	-0.04657
RIBC1	-0.044117729	-0.104708709	-0.05526	0.00788	-0.01426	0.091015
RIBC2	-0.023559949	-0.002425812	-0.04247	-0.00639	0.027773	0.050108
RIC3	-0.027425755	-0.158683554	0.156678	-0.07244	0.213604	-0.19043
RIC8B	0.002913903	0.066674038	0.235794	-0.08731	0.220246	-0.07389
RICTOR	0.025572652	0.057263696	0.013071	0.011822	-0.01806	-0.35081
RIIAD1	-0.036233829	-0.050894174	-0.12708	0.024813	-0.07725	-0.04982
RILP	0.016818852	-0.137334444	-0.10933	-0.03642	0.035271	0.125013
RILPL1	-0.004241429	-0.004926918	0.129498	-0.0673	0.179696	-0.13867
RILPL2	0.043641493	-0.113365643	-0.35886	-0.02848	0.093862	0.349068
RIMBP2	-0.040028155	-0.039373038	-0.07235	0.024764	-0.02909	-0.10558
RIMKLA	-0.052404559	-0.000993026	-0.04347	0.158744	-0.14044	0.0058
RIMKLB	-0.033642764	-0.074715794	0.09157	-0.05271	0.168971	-0.07448
RIMS1	-0.012903996	-0.004424953	-0.04276	0.056971	-0.01415	-0.17203
RIMS2	-0.029473456	-0.015940476	0.095918	0.014615	-0.03795	-0.21414
RIMS3	-0.007454341	-0.066534301	0.002015	0.063929	-0.18005	-0.24027
RIMS4	-0.032377108	-0.066467323	-0.11903	-0.01544	0.031827	-0.0285
RIN1	-0.003408777	-0.113465852	-0.04932	-0.01662	-0.00588	0.296185
RIN2	0.000813393	-0.090113163	-0.25534	-0.07603	0.227357	0.737149
RIN3	-0.021317271	0.051587671	-0.10435	-0.08211	0.171322	0.141224
RING1	0.08799935	-0.030619682	0.302693	0.033566	-0.1003	-0.1465
RINT1	0.00953768	0.037347085	0.123127	0.062998	-0.12101	-0.13108
RIOK1	0.025250218	0.065314244	0.118318	0.055145	-0.1348	-0.00085
RIOK2	0.011956677	0.031089033	0.03471	0.044595	-0.09162	0.115524
RIOK3	0.012320595	0.136176219	-0.28175	-0.08635	0.151153	0.142592
RIPK1	0.028686011	0.169043574	-0.0146	-0.01525	0.005116	-0.11737
RIPK2	0.01825131	-0.032817655	-0.19545	-0.00976	0.045525	0.188372
RIPK3	0.012732294	0.018823589	-0.15493	-0.1023	0.25661	0.048576
RIPK4	-0.0439231	-0.168754189	-0.05712	-0.01063	-0.01087	0.089195
RIPPLY1	-0.05298741	-0.02604763	-0.0026	-0.01067	0.070269	-0.07858
RIPPLY2	-0.039356332	-0.07232913	-0.11887	0.035154	-0.06332	-0.03907
RIPPLY3	-0.026689192	-0.028066738	0.013246	0.015836	-0.05208	-0.04738
RIT1	-0.00498944	0.066791323	-0.22976	-0.01349	0.102093	0.277783
RIT2	-0.024113489	-0.044311355	-0.0013	0.091391	-0.14747	-0.00573
RITA1	0.018168986	0.052859565	0.038121	0.052113	-0.1269	0.118984
RLBP1	-0.031284371	-0.073663397	-0.03408	-0.01355	0.031129	0.037759
RLF	0.034163486	0.035263876	-0.07751	0.06512	-0.17224	-0.08689
RLIM	0.029930997	0.022841927	-0.17035	0.041312	-0.08409	0.147179
RLN1	-0.015577001	0.009597651	0.252071	-0.01758	0.027006	-0.20151
RLN2	-0.012050347	0.011660315	0.297297	-0.02756	0.078177	-0.34687
RLN3	-0.038927201	-0.06989854	-0.0313	0.006315	0.022101	0.019632
RLTPR	-0.01645684	-0.158014625	0.270893	0.020495	-0.07755	-0.29511
RMDN1	0.01691588	0.015464621	-0.02334	0.014762	-0.05183	0.01161
RMDN2	-0.065229183	0.047820591	-0.09277	-0.07074	0.1081	0.099322
RMDN3	0.039872173	0.222097332	0.040653	0.040757	-0.14678	-0.28248
RMI1	0.014190131	0.002119617	-0.28191	0.049215	-0.13375	0.129672
RMI2	-0.011308129	0.017631324	0.018265	0.080182	-0.28843	-0.18431

RMND1	0.004127256	-0.001198725	0.023297	0.047839	-0.12378	0.203525
RMND5A	0.040739514	0.152087762	-0.0829	-0.05039	-0.01289	-0.00435
RMND5B	0.003699467	-0.184830407	-0.04713	0.058651	-0.21476	0.124543
RMST	-0.047261861	-0.050139248	-0.01337	0.010702	0.023261	-0.09402
RN7SKP15(	-0.029711362	-0.044660287	0.106769	-0.00517	0.03193	-0.19851
RNA45S5	0.009299213	0.006872893	-0.07555	0.048654	-0.12828	-0.0381
RNASE1	-0.036353584	-0.018867358	-0.13451	-0.03282	0.039733	0.237751
RNASE11	-0.040029687	-0.043621529	0.099705	-0.01567	0.061662	-0.02572
RNASE2	0.003132579	-0.131173224	-0.14996	-0.05117	0.152311	0.483491
RNASE3	-0.003974264	-0.127928207	0.059132	-0.01468	0.020871	0.033272
RNASE4	-0.014206673	-0.082602123	-0.2449	-0.06837	0.203992	0.682443
RNASE6	0.012162968	-0.129881596	-0.3871	-0.07808	0.216889	0.289115
RNASE7	-0.02552653	-0.135862585	0.006693	-0.01958	0.128337	0.145195
RNASEH1	0.054130874	0.133243451	0.234347	0.011223	-0.11793	-0.03336
RNASEH1-/	-0.002996199	0.004627729	0.129301	0.07535	-0.1813	-0.06121
RNASEH2A	0.012446751	0.074296817	-0.01341	0.11709	-0.31346	0.081197
RNASEH2B	0.010987788	-0.056034391	0.082558	-0.07523	-0.02207	-0.07625
RNASEH2C	0.041767843	0.055220952	0.115984	0.048863	-0.15639	0.181634
RNASEL	0.015983781	-0.033736074	-0.20562	-0.04166	0.093375	0.088289
RNASET2	-0.010273705	-0.166426172	-0.13999	-0.10589	0.234548	-0.06936
RND1	-0.018567186	-0.094375619	0.034688	0.066861	-0.2119	-0.02939
RND2	-0.061102321	-0.05712715	-0.06908	0.012995	-0.00418	0.040393
RND3	-0.029209088	-0.023473489	-0.02799	0.034258	-0.09606	-0.06262
RNF10	0.060499439	-0.033732913	-0.13676	0.01741	-0.03715	-0.16197
RNF103	0.021561565	-0.054756694	0.036935	-0.0182	0.048623	-0.20391
RNF11	0.043014744	0.096025344	0.003167	-0.06868	0.195683	0.018908
RNF111	0.060094741	-0.000179417	-0.28128	0.011639	-0.00191	0.168665
RNF112	-0.034064709	0.065397445	-0.0358	-0.04558	0.12564	-0.01611
RNF113A	0.046644133	0.107659615	0.218501	0.000951	-0.0266	-0.18051
RNF114	-0.007331817	-0.049499528	-0.1748	0.035072	-0.02184	0.222796
RNF115	0.045483281	0.181766754	0.076976	0.066768	-0.18924	-0.00021
RNF11B	-0.062053244	-0.053782836	0.061073	-0.00578	0.004154	0.076563
RNF121	-0.005230496	-0.012538691	-0.08193	0.023028	-0.11777	-0.062
RNF122	-0.010200038	-0.034588158	-0.08125	-0.02418	-0.00942	-0.04102
RNF123	0.021114377	-0.051704106	-0.06952	0.01425	-0.09349	0.151636
RNF125	0.019784305	0.144622924	0.28528	-0.02391	0.070242	0.008291
RNF126	0.015914703	0.107156381	0.229642	0.004424	-0.03064	-0.10414
RNF126P1	-0.006530408	-0.015042755	0.141786	0.015598	-0.05461	0.017265
RNF128	-0.070726345	-0.074560699	-0.01417	-0.01971	0.070427	0.036832
RNF13	0.043401407	-0.01638338	-0.29961	-0.09286	0.266829	0.195933
RNF130	-0.013752148	-0.122266845	-0.28171	0.000131	0.003183	0.40091
RNF133	-0.029362802	-0.096636666	-0.04073	-0.0004	0.004617	-0.07118
RNF135	0.023039738	0.003341872	-0.42645	-0.08609	0.241662	0.402353
RNF138	0.039063491	-0.030696048	0.059526	0.034867	-0.07395	0.049918
RNF139	0.106791324	0.105237026	0.049755	0.038522	-0.06998	-0.13529
RNF139-A!	-0.00939403	0.037954429	0.083872	0.006763	0.005346	-0.22087
RNF14	0.020898096	0.147967384	-0.06846	0.035614	-0.0545	0.026399
RNF141	-0.009395462	-0.197307551	-0.29517	-0.07049	0.108232	0.137011
RNF144A	-0.002708531	0.063484408	0.257521	0.005649	-0.02026	-0.28096
RNF144A-/	-0.033550082	-0.043943761	0.109285	-0.02012	0.049633	-0.04871
RNF144B	0.019156437	-0.159186331	-0.39741	-0.0325	0.082932	0.356377

RNF145	0.006887017	-0.081603919	-0.06068	0.006819	-0.06922	-0.05428
RNF146	0.034246883	0.090404228	-0.36384	-0.02477	0.08946	0.345213
RNF148	-0.033725966	-0.053915723	-0.04804	0.033528	-0.08407	-0.07684
RNF149	0.032113573	0.038455224	-0.20181	-0.01312	0.041469	0.151336
RNF150	-0.029062468	-0.052786836	-0.19379	0.011169	0.052294	0.259627
RNF151	-0.023792957	-0.07982816	-0.03442	0.005157	-0.00381	0.090057
RNF152	-0.064175447	-0.059338635	-0.06281	-0.03445	0.088264	0.058292
RNF157	-0.008035619	-0.028169164	0.448944	0.035053	-0.13993	-0.21887
RNF157-AS	-0.022238897	-0.038946586	0.501071	-0.10591	0.323259	-0.26392
RNF165	-0.006878726	0.308644979	-0.0248	-0.02914	0.090335	-0.1779
RNF166	0.018745709	0.074644477	0.03068	-0.04586	0.13423	0.131268
RNF167	0.051966383	0.056641372	0.187336	0.017979	-0.07116	-0.25928
RNF168	0.022067482	0.218550611	-0.01748	0.045525	-0.12075	-0.01013
RNF169	0.06503591	0.142770824	-0.23742	0.011604	-0.02571	0.121075
RNF17	-0.054723078	-0.057515831	-0.07451	0.034175	0.05949	0.038026
RNF170	-0.049283716	0.057678444	-0.04752	0.067096	-0.00655	0.100571
RNF175	-0.017999776	-0.114893842	-0.15281	-0.04621	0.145196	0.067389
RNF180	-0.100865737	-0.044068653	-0.0129	0.061068	0.052669	0.037525
RNF181	0.093549689	0.064909998	-0.2186	0.099509	-0.24923	0.426659
RNF182	-0.019480591	-0.063298407	0.009828	0.002642	0.002319	-0.088
RNF183	-0.027401543	-0.024206117	0.072651	-0.01083	-0.02403	-0.12879
RNF185	0.058631307	0.064919788	-0.19915	0.002341	-0.05157	0.095859
RNF185-AS	-0.027178405	-0.02466951	0.063935	-0.00628	-0.01851	-0.01112
RNF186	-0.038476638	-0.056607384	0.092457	-0.0298	0.081325	-0.01067
RNF187	-0.03867742	0.033293366	-0.00106	0.047657	-0.20111	0.010321
RNF19A	0.017783869	0.075022133	-0.00062	0.008357	-0.03524	-0.15834
RNF19B	0.021690516	-0.030961883	-0.29534	0.035299	-0.08681	0.207929
RNF2	0.004615681	0.012639158	-0.06316	-0.00151	0.03918	0.125765
RNF20	0.054403772	0.112061898	-0.08862	0.027593	-0.05762	-0.01939
RNF207	2.80E-05	-0.156838227	-0.09996	0.04379	-0.15456	0.055458
RNF208	-0.033576111	-0.01016322	-0.01245	-0.03532	0.089634	0.073926
RNF212	-0.030214593	-0.062804093	-0.06965	-0.01297	-0.04428	-0.01158
RNF212B	-0.024663842	-0.0295527	-0.00457	-0.16338	0.457521	-0.0468
RNF213	0.00819995	0.08767481	0.153696	0.074591	-0.13196	-0.05202
RNF214	0.005227914	-0.058714163	0.156416	-0.01878	0.025034	-0.09216
RNF215	-0.036801648	-0.03338806	-0.03898	-0.00033	-0.0026	-0.01298
RNF216	0.006649065	-0.068256353	0.20427	-0.03024	0.090964	-0.08217
RNF217	0.020391455	0.018304381	-0.24087	0.014496	0.104631	0.480956
RNF219	0.008600047	0.134351189	0.052276	0.062726	-0.21271	-0.18167
RNF220	0.077056521	0.020090713	0.157836	0.007068	-0.05195	-0.07637
RNF24	0.008361753	-0.066700078	-0.18556	0.002085	0.015922	0.131865
RNF25	0.001047859	0.032048646	-0.035	0.072899	-0.16039	0.035476
RNF26	0.003095653	-0.068445216	0.13039	0.093687	-0.31737	0.02955
RNF31	-0.008071593	-0.068166229	-0.08246	0.022371	-0.13763	-0.10339
RNF32	-0.073302942	-0.056354181	-0.0352	-0.04446	-0.01905	0.004394
RNF34	-0.038696847	0.035845605	0.15776	0.024215	-0.05051	-0.19807
RNF38	0.037829688	0.084780495	-0.0256	-0.04661	0.127139	-0.16746
RNF39	-0.02380916	-0.012306594	-0.01767	-0.01322	-0.00592	-0.02083
RNF4	0.10019836	0.22919068	0.10537	0.045704	-0.10187	-0.43686
RNF40	-0.029361347	-0.02746773	-0.12062	0.057307	-0.11338	0.309063
RNF41	-0.029700731	-0.043701995	-0.3398	-0.02883	0.012806	0.213548

RNF43	-0.026205585	-0.02977714	0.020737	0.014854	-0.02478	-0.17172
RNF44	0.049209245	0.089957614	0.12278	-0.02936	0.086668	-0.1278
RNF5	-0.031513322	-0.044763983	0.007755	0.026062	-0.10961	-0.04999
RNF6	-0.02447851	0.064599686	-0.14975	-0.03325	0.105299	0.13012
RNF7	0.063556581	0.078117039	0.059413	0.042509	-0.09728	0.435747
RNF8	-0.004333404	0.132164391	0.109939	0.006215	-0.05592	-0.03624
RNFT1	0.013778228	0.080188002	-0.29747	-0.05179	0.159437	0.263663
RNFT2	-0.074169237	-0.11066174	0.155182	0.105226	-0.13689	-0.17307
RNGTT	0.01608846	0.03653048	0.0022	0.030583	-0.07998	-0.25963
RNH1	0.025712341	-0.048839136	-0.07339	-0.00833	0.023296	0.540786
RNLS	-0.030407802	0.003627011	-0.05839	-0.06017	0.168348	0.064882
RNMT	0.015060104	0.010846614	0.074857	0.043563	-0.09808	0.038478
RNMTL1	0.033843615	0.00486383	0.040863	0.047615	-0.13679	0.095906
RNPC3	0.008509805	0.028265444	-0.08996	-0.01693	0.063239	-0.02054
RNPEP	0.037354183	-0.030562417	-0.28699	-0.01668	0.066915	0.349582
RNPEPL1	0.034761686	0.030250604	-0.07058	-0.01912	0.016771	0.092265
RNPS1	0.082948264	0.102391721	0.105237	0.082714	-0.22515	-0.27366
RNU2-22P	-0.041813962	-0.009958766	0.091206	0.0233	-0.07346	-0.06212
ROBO1	-0.012858274	-0.133635594	0.306283	0.037717	-0.06871	-0.1083
ROBO2	-0.048865206	-0.102961162	-0.03895	0.022861	0.111055	0.068586
ROBO3	0.004204576	-0.006118377	0.230272	-0.03597	0.11511	-0.2718
ROBO4	-0.041965941	-0.082443174	0.02663	0.008455	-0.04801	-0.14492
ROCK1	0.017048544	-0.053192135	0.034185	0.020098	-0.10675	-0.06527
ROCK2	-0.001212816	0.065476743	-0.13756	-0.0152	0.021209	0.198233
ROGDI	0.007602412	-0.126626646	-0.16212	-0.04673	0.154978	0.476631
ROM1	-0.02131937	-0.058950724	-0.07804	0.057934	-0.15959	-0.01739
ROMO1	0.039853428	0.012143785	0.14092	0.125167	-0.35037	0.163963
ROPN1	-0.010485597	-0.074873528	0.007553	0.049232	-0.08758	-0.09462
ROPN1B	-0.034593323	-0.03967027	0.009452	0.030751	-0.06884	-0.02031
ROPN1L	-0.020875267	-0.008526417	-0.13029	-0.00846	0.009957	-0.17865
ROR1	-0.017088399	-0.083135054	0.13293	0.004453	0.042454	-0.16932
ROR1-AS1	-0.055942474	-0.064061305	-0.0877	-0.02841	0.084837	-0.02055
ROR2	-0.019811744	-0.002565991	0.02867	0.042887	-0.11999	0.017719
RORA	0.043421674	0.210024012	0.377164	0.04636	0.015444	-0.39374
RORB	-0.011048239	0.092394366	-0.05546	0.08024	-0.14241	-0.06565
RORC	-0.009865762	-0.057998618	0.184193	-0.02024	0.025417	-0.09124
ROS1	-0.065724098	-0.108500071	0.057411	0.037073	-0.06464	0.004655
RP1	-0.04256254	-0.042955981	-0.09628	0.069226	-0.11362	0.004182
RP1-100J	-0.031535758	-0.037953394	-0.07512	0.031089	-0.06611	0.200797
RP1-101G	-0.030132892	-0.026209272	-0.13298	0.054766	-0.11639	0.081525
RP1-102H	-0.03346271	0.000147002	0.131881	-0.03752	0.090088	-0.08124
RP1-118J	-0.020511893	-0.10243814	-0.00719	-0.02125	-0.00671	0.006781
RP1-130G	0.000448387	-0.082659178	0.140082	0.026696	-0.05595	0.114765
RP1-135L	-0.033825391	-0.032991239	-0.10795	0.016376	-0.02734	0.022379
RP1-140C	-0.034471722	0.133821581	0.10742	0.015392	-0.0304	-0.11113
RP1-142L	-0.02889699	-0.052428646	0.04813	-0.00141	0.014721	-0.14581
RP1-142L	-0.024491489	-0.020352101	0.023469	-0.0174	-0.00572	-0.08067
RP1-149A	-0.034417942	-0.034406313	0.052223	0.008595	-0.01461	-0.05476
RP1-149C	-0.047879268	-0.086350028	-0.00904	-0.02362	0.052859	-0.09061
RP1-151F	-0.008520764	0.050696361	0.194908	0.032179	-0.08692	-0.10088
RP1-155D	-0.034635614	-0.110953826	-0.07397	0.007643	-0.00505	0.164441

RP1-155D2	-0.02331514	-0.014996002	0.011534	0.01663	-0.0763	-0.06879
RP1-170O1	-0.027880658	0.003055135	0.064934	0.036514	-0.08277	0.127628
RP1-170O1	-0.021769642	0.034692674	-0.13977	0.001064	0.003029	-0.18459
RP1-178F1	-0.029414319	0.125904432	0.210448	0.015913	-0.02424	-0.27419
RP1-179N1	-0.026450702	-0.112852059	0.164388	0.000702	0.009359	-0.15807
RP1-181J2	-0.037915882	0.006528425	0.091915	0.027216	-0.05893	-0.01832
RP1-187B2	-0.029440589	0.01321563	0.062588	-0.00571	0.003111	-0.19474
RP1-190J2	-0.03159723	-0.036043867	0.138474	0.008427	-0.00865	-0.01846
RP1-192P9	-0.053339573	-0.101519974	-0.05629	-0.0162	0.08197	0.085225
RP1-193H1	-0.002887007	-0.029898587	0.01631	-0.0393	0.135256	-0.02833
RP1-199J3	-0.035155721	-0.088682723	0.030606	0.035932	-0.05768	-0.00649
RP1-202O8	-0.028415552	-0.025852021	0.070559	-0.00436	0.041884	-0.08163
RP1-20C7.	-0.017481755	-0.066349188	-0.01265	-0.01082	-0.05206	-0.11104
RP1-212P9	-0.036410226	-0.047947954	0.006228	-0.00115	-0.03481	0.019175
RP1-217P2	-0.054140569	0.021193898	-0.13579	0.008588	-0.00163	-0.06299
RP1-228H1	-0.021564794	0.035166069	0.125874	-0.0388	0.103373	-0.10577
RP1-263J7	-0.080925007	-0.117334932	0.072029	-0.05755	0.132331	0.056385
RP1-265C2	-0.034620899	-0.032195127	0.033297	0.01493	-0.03345	-0.0496
RP1-272E8	-0.047084841	-0.050411143	0.151366	-0.00258	0.026085	-0.15882
RP1-286D6	-0.047315149	-0.071931031	-0.02013	0.018172	-0.01777	0.052346
RP1-28C20	-0.060303331	0.01614884	-0.06222	-0.02716	0.048227	-0.01121
RP1-28O17	-0.043666905	-0.039438584	-0.06194	########	0.02936	-0.03043
RP1-305G2	-0.037679257	-0.046815824	0.003845	0.000212	0.016103	0.132857
RP1-30M3.	0.001786692	0.042823139	-0.00182	-0.04797	0.163989	0.054506
RP1-31B8.	-0.042101129	-0.05494491	0.021719	0.006602	0.004384	0.168451
RP1-35C21	-0.05637264	-0.003208416	-0.08796	0.006632	-0.02371	0.040639
RP1-39G22	0.009579789	-0.008915855	0.1851	0.000395	0.019009	0.006112
RP1-39J2.	-0.077328836	-0.027437968	0.017705	-0.01466	0.011401	0.02483
RP1-58B11	-0.020446236	-0.018920127	0.004052	0.010176	-0.02296	0.036907
RP1-68D18	-0.02497244	-0.025093696	-0.03913	0.003965	0.010807	0.029645
RP1-6P5.2	-0.038385674	-0.050856504	0.216286	-0.03214	0.082132	-0.12737
RP1-74M1.	-0.042568489	-0.077399867	0.084508	0.000986	0.009191	-0.06967
RP1-78O14	-0.090254631	-0.070456145	0.00248	-0.02012	0.050369	0.112936
RP1-86D1.	-0.022444448	-0.044559703	0.123765	0.000214	-0.00709	-0.03953
RP1-8B22.	-0.03365986	-0.005323726	-0.19744	0.02722	-0.04849	0.294379
RP1-93H18	0.004003246	-0.076091625	-0.30138	-0.11391	0.319443	0.257069
RP11-1007	-0.048845322	0.00020033	0.112247	0.002428	-0.02871	0.109025
RP11-1007	-0.03225985	-0.074126053	0.002794	0.00884	-0.05434	0.06011
RP11-1008	-0.043750836	-0.039401577	-0.08124	0.021873	-0.05283	0.000585
RP11-1012	-0.026371007	-0.05022483	0.057919	0.005687	-0.02934	-0.0757
RP11-1017	-0.064174509	-0.089792374	0.026751	-0.03842	0.071973	0.120404
RP11-1017	-0.029237079	-0.001789362	-0.00856	0.0271	-0.07455	-0.0529
RP11-1024	-0.017189095	-0.106057288	0.093089	-0.04214	0.123751	-0.02563
RP11-102O	-0.045689387	-0.14000816	0.025001	-0.01101	0.063688	0.078008
RP11-102I	-0.039941216	-0.063951253	0.074388	-0.01293	0.042773	-0.04662
RP11-102M	-0.026448053	-0.044329867	0.023206	0.006398	-0.02201	-0.04022
RP11-1069	-0.028835339	-0.014761353	-0.04306	-0.01793	-0.03058	-0.13981
RP11-1072	-0.057952383	-0.000489817	########	-0.00616	-0.0156	0.077574
RP11-1078	-0.022393888	-0.048572536	-0.05028	0.003294	-0.03158	-0.08156
RP11-108I	-0.029180843	-0.066074625	-0.00311	0.03731	-0.07721	0.077862
RP11-108I	-0.051610345	-0.100815805	-0.0131	-0.01432	0.064781	-0.17747

RP11-108I	-0.02952797	-0.075473593	-0.01591	0.005369	-0.01751	-0.00615
RP11-108I	-0.031009948	0.012397563	-0.03923	0.093291	-0.25026	0.067607
RP11-108I	-0.040221843	-0.081022	0.048698	0.014881	-0.04737	0.004239
RP11-108I	-0.027075668	-0.015684353	0.010577	-0.01591	0.000762	-0.09801
RP11-109	0.004747451	-0.007461918	0.015762	-0.01801	0.023151	0.035992
RP11-109I	-0.034477343	-0.045656056	0.086075	0.004434	0.010215	-0.00138
RP11-109I	-0.037336598	-0.079070985	-0.03052	0.014922	-0.0237	0.157427
RP11-109I	-0.038360943	-0.096560701	0.025134	0.022263	-0.04234	-0.04785
RP11-109I	-0.025003128	-0.07993432	0.000365	-0.02152	-0.03888	-0.16846
RP11-109(	-0.024963236	0.013884601	0.023526	0.001516	0.0182	0.102363
RP11-109N	-0.040999167	-0.077171638	-0.07049	0.009953	-0.0081	0.047032
RP11-10K	-0.047143636	-0.060794641	-0.07975	-0.0241	0.051478	0.095433
RP11-10N	-0.040753622	-0.06415117	-0.02491	0.006762	-0.08002	-0.00042
RP11-110	-0.028156864	-0.0315706	0.043505	0.015945	-0.02677	0.000395
RP11-110	-0.054297011	0.019095871	-0.13552	-0.0104	0.066344	0.108782
RP11-110	-0.037808656	-0.061038316	-0.09437	-0.04574	0.151887	0.067394
RP11-110	-0.065552925	-0.021771967	0.061643	-0.01503	0.036179	-0.02357
RP11-111	-0.023747765	-0.014935002	-0.05693	-0.02227	0.089872	0.019824
RP11-111	-0.057010914	0.004193621	0.119964	-0.04986	0.122301	-0.09083
RP11-111I	-0.028732275	-0.045542058	-0.04788	0.000931	0.008087	-0.04649
RP11-111N	-0.005827858	-0.001351176	0.342736	0.046367	-0.11104	-0.09053
RP11-111N	-0.012153211	0.012764045	0.213483	0.025528	-0.05539	-0.23881
RP11-112	-0.011055296	-0.021590219	0.166387	0.001349	0.007197	-0.33641
RP11-112I	-0.080734002	-0.060086378	-0.0881	-0.04755	0.110231	0.041242
RP11-113	-0.046878708	-0.01838452	-0.01567	-0.01443	0.02561	-0.02438
RP11-114I	-0.030193836	-0.035517388	0.005839	0.01645	-0.06737	-0.01928
RP11-115	-0.039289907	0.031487943	-0.00175	0.031809	-0.09131	0.073338
RP11-115	-0.021919538	-0.031316945	0.010674	0.008466	-0.00943	-0.07237
RP11-115	-0.033632281	-0.014917494	-0.05044	0.051036	-0.1267	0.089507
RP11-116I	-0.043600739	-0.034582395	0.022202	0.008557	-0.01904	-0.09698
RP11-116(	-0.095504605	-0.122907157	0.075246	-0.02433	0.072671	0.057066
RP11-118(	-0.040634984	-0.007860958	0.009786	0.035226	-0.07394	0.049678
RP11-118(	-0.036574927	-0.030474841	-0.07062	0.027066	-0.05809	-0.05386
RP11-119	-0.005117159	-0.053633255	0.21791	-0.03313	0.109606	-0.26625
RP11-119I	-0.013568077	0.099645607	0.172184	-0.0227	0.073648	-0.16121
RP11-120I	-0.037743484	-0.015147804	-0.14076	0.046255	-0.13537	0.060293
RP11-121	-0.053297114	-0.076546553	-0.03907	-0.03754	0.093108	0.050834
RP11-121(	0.001893865	-0.008306866	0.15622	-0.00998	-0.01579	-0.18231
RP11-123I	-0.038479657	-0.039764367	-0.05999	0.020363	-0.03433	0.039727
RP11-124I	-0.009102266	0.049345932	0.049523	0.036288	-0.09207	-0.08713
RP11-126(	-0.047096312	-0.047073874	-0.12448	0.008414	-0.00406	0.055503
RP11-127	-0.029117095	-0.124497244	-0.05961	-0.00509	0.038512	0.331363
RP11-127	-0.028113592	-0.050701553	0.066728	-0.02201	0.032048	-0.10408
RP11-127	-0.02391799	0.049460755	-0.01923	0.01871	-0.02941	-0.01831
RP11-127I	-0.007940678	-0.010823808	0.311547	-0.062	0.189073	-0.18195
RP11-12M	-0.035729903	-0.074811493	0.043952	-0.00169	0.016708	-0.03424
RP11-131I	-0.070670262	-0.016296422	0.002101	-0.01652	0.033672	0.052562
RP11-134(	-0.027977157	-0.020315076	0.038268	0.068963	-0.19481	0.017023
RP11-134I	-0.013396433	-0.001409408	-0.13152	-0.00342	-0.03191	-0.16668
RP11-135	-0.030106773	-0.086436903	-0.14219	-0.01127	0.051909	0.134412
RP11-138	0.013033965	-0.002566184	0.07253	0.026304	-0.06796	-0.3663

RP11-138I	-0.0232388	-0.059193069	-0.06668	0.015356	-0.04768	-0.02362
RP11-138I	-0.030132996	-0.083389839	-0.06579	-0.0204	0.075172	-0.12874
RP11-13K	-0.035134393	-0.013158252	-0.13064	0.014384	-0.01999	-0.00586
RP11-140I	-0.009504009	0.012546894	0.247857	-0.04623	0.130951	-0.30311
RP11-141M	-0.034968342	-0.041337912	-0.03229	0.004573	0.005129	0.073789
RP11-143I	-0.054596078	-0.067474515	-0.10135	-0.01166	0.021254	-0.05307
RP11-143I	-0.04146192	-0.029892696	0.104354	0.031619	-0.05984	0.055889
RP11-151I	-0.035617373	-0.04011716	0.034981	0.024819	-0.04954	-0.08818
RP11-152I	-0.033700516	-0.056728544	0.050577	-0.09836	0.311961	-0.16719
RP11-153I	-0.06914807	0.035295985	0.035571	-0.02485	0.077391	0.029073
RP11-155(	-0.029038067	-0.022616993	0.031304	0.000578	-0.03928	-0.12248
RP11-157I	-0.042483798	-0.03152743	0.044805	0.019402	-0.03234	-0.02062
RP11-157I	-0.048198086	-0.07241193	0.060448	-0.01035	0.049342	0.014364
RP11-157I	-0.036964686	-0.050348525	-0.13783	0.008548	0.006253	0.109178
RP11-158(	-0.005105907	-0.000788818	0.368082	0.005067	-0.01337	-0.34379
RP11-15P	-0.053955533	-0.005408457	0.004795	0.042118	-0.09669	0.051794
RP11-161I	-0.031388297	-0.090401781	-0.05776	0.050422	-0.13389	-0.01164
RP11-161I	-0.074210176	-0.078766237	0.083405	-0.01912	0.036903	0.139584
RP11-164I	-0.009605598	-0.04468551	0.032323	0.015242	-0.02718	-0.17395
RP11-164I	-0.016845119	-0.071773028	-0.09179	-0.00392	0.014854	-0.12021
RP11-166I	-0.039826546	-0.029428333	0.116811	-0.01011	0.061944	-0.02283
RP11-167I	-0.036814964	-0.052190456	-0.03461	0.004887	0.004228	-0.0502
RP11-16N	-0.053710865	0.110337485	0.003998	-0.03484	0.108681	0.01903
RP11-16P(	-0.030024104	-0.048524991	0.282591	-0.02481	0.083674	-0.09773
RP11-171I	-0.122504463	-0.067367266	-0.05286	-0.02467	0.099888	-0.0501
RP11-171I	-0.027530744	-0.071066731	0.045499	-0.00015	0.011239	-0.07303
RP11-171N	-0.123180038	-0.097622422	-0.22519	0.008511	0.107437	0.044419
RP11-173I	-0.019897135	-0.073347276	-0.00475	-0.01904	0.008399	-0.04082
RP11-173N	0.004663558	0.010699432	-0.2677	-0.04461	0.131143	0.416936
RP11-174(	-0.006329624	-0.048230584	0.066937	0.001035	0.006596	-0.24178
RP11-177N	-0.038165597	0.004411262	-0.06713	0.00083	0.029623	0.052715
RP11-179I	-0.031000459	-0.017602523	0.084645	0.012114	-0.0367	-0.03486
RP11-17A	-0.033942596	-0.076496528	-0.02089	-0.01998	0.066627	0.170865
RP11-180N	-0.047257152	-0.061499146	-0.04632	-0.02076	0.066532	-0.05325
RP11-182	-0.053059502	-0.005408788	-0.12432	-0.01509	0.055101	-0.01034
RP11-182I	0.00414038	-0.028214892	-0.18099	-0.03393	0.064008	-0.02012
RP11-184I	-0.039997581	-0.01477425	0.088082	0.009073	-0.0298	-0.13042
RP11-186I	-0.046509352	-0.045395739	-0.09218	-0.00492	0.047168	-0.0083
RP11-18F	-0.024015214	-0.031171366	-0.07059	-0.00505	0.008237	-0.12948
RP11-18I	-0.033138188	0.022096299	0.006562	-0.00046	-0.03869	-0.00017
RP11-190	-0.019805116	0.001617538	0.235504	-0.0162	0.07021	-0.1797
RP11-194I	-0.023815591	-0.174963905	-0.29966	0.010629	-0.01485	0.350796
RP11-195N	-0.029312997	-0.040846866	-0.02522	-0.01196	-0.00712	-0.02268
RP11-196(	-0.021536719	-0.011670573	0.010633	-0.01024	0.016822	0.060958
RP11-196(	0.001139841	-0.012542243	-0.10788	-0.01665	0.071961	0.006981
RP11-199I	-0.010288438	-0.015873571	0.160543	0.039554	-0.16881	-0.09865
RP11-1C8.	-0.028594758	-0.019297352	0.131641	0.014308	-0.03664	-0.18992
RP11-1E4.	-0.030980065	-0.014222616	0.060295	0.000291	0.010108	-0.06343
RP11-1L1	-0.029291137	-0.074462022	-0.05244	0.001302	-0.00141	-0.10556
RP11-201	-0.08204789	-0.066863414	-0.05037	-0.01052	0.0563	0.015274
RP11-203I	-0.039024993	-0.070070016	-0.03161	0.00377	0.008787	0.026979

RP11-203I	-0.036055713	-0.031638634	-0.0464	-0.00245	0.030731	0.014449
RP11-209/	0.04049764	0.046280537	0.272068	0.020154	-0.04298	-0.0386
RP11-209I	-0.044462221	-0.13123178	0.026928	-0.01158	0.013278	0.04143
RP11-210I	-0.056113477	-0.028044724	-0.03376	-0.01662	0.042058	-0.09243
RP11-210M	-0.069952954	0.061820777	0.06589	-0.03358	0.0698	-0.03554
RP11-212I	-0.001657601	0.048929112	0.10811	0.004219	0.023193	0.091876
RP11-214I	-0.018529525	0.05404205	0.065273	0.009921	-0.06474	-0.23015
RP11-214I	-0.064817161	0.142186876	-0.02082	-0.02356	0.051195	-0.04831
RP11-214M	-0.055362853	-0.10520924	0.134754	-0.01881	0.061372	0.0349
RP11-214M	-0.033976261	-0.085439741	0.12538	0.011068	-0.00458	-0.05488
RP11-215I	-0.025577774	-0.02084894	0.122616	-0.00274	0.010563	-0.17128
RP11-215I	-0.028428941	-0.032884669	-0.05982	-0.01325	0.040318	-0.12506
RP11-216I	-0.004592008	-0.040609979	-0.03677	-0.00056	0.018856	-0.12909
RP11-217I	-0.051573608	-0.06440686	-0.00512	0.02594	-0.0357	-0.05387
RP11-217I	-0.033468859	-0.092037145	0.099819	0.01529	-0.02154	0.022915
RP11-218(	-0.042739986	-0.072714133	-0.17774	-0.04306	0.14117	0.088887
RP11-218I	-0.030594673	-0.08948807	0.154854	-0.01641	0.047268	-0.15721
RP11-218.	-0.042119728	-0.068437222	0.03184	0.01734	-0.0216	-0.02112
RP11-219I	-0.049064495	-0.035911878	-0.0364	0.015912	-0.02891	-0.0243
RP11-21G.	-0.027427572	-0.025091713	0.026636	0.012309	-0.01932	-0.02705
RP11-21L2	-0.059490983	-0.081559987	0.020904	-0.02087	0.057991	-0.05241
RP11-220.	-0.027292576	0.054321203	-0.05255	0.034742	-0.09856	-0.03779
RP11-222I	-0.028423876	0.022494536	0.056768	0.042145	-0.10235	-0.09507
RP11-225I	-0.02719575	0.013311733	0.00782	-0.00304	-0.01128	0.035558
RP11-226I	-0.006769619	0.047514273	0.057265	-0.04531	0.144948	0.231806
RP11-227I	-0.050127598	-0.008883484	-0.09505	-0.00119	0.016626	0.021285
RP11-231(	-0.02835309	-0.058598458	-0.12045	0.011071	-0.01863	-0.00955
RP11-231I	-0.047049323	-0.010513551	-0.05921	-0.00756	0.043043	-0.00458
RP11-235I	-0.009865826	-0.011874179	0.042384	-0.00534	0.012612	-0.04278
RP11-245.	-0.04721634	0.009685247	0.0369	-0.02716	0.074764	0.073879
RP11-245I	-0.036504033	-0.095660865	0.187883	-0.02529	0.100519	0.09037
RP11-247I	-0.001159373	0.119675427	0.086066	-0.03015	0.114262	0.045106
RP11-248.	-0.020383864	-0.013648179	-0.00051	-0.01892	0.055534	-0.08604
RP11-248.	0.012463306	-0.10392989	-0.06557	-0.01668	0.081247	0.016014
RP11-24D.	-0.036353804	0.028197726	0.082864	-0.00648	0.051358	-0.01972
RP11-24P.	-0.038489224	-0.049735563	0.026067	0.017213	-0.03242	0.077882
RP11-250I	-0.036661301	-0.015598521	0.051206	0.02564	-0.05637	-0.07027
RP11-250I	-0.025713367	0.020044288	0.064244	0.035327	-0.10814	-0.05144
RP11-251(	-0.033289624	-0.004209355	0.057312	0.021415	-0.0708	0.166359
RP11-252I	-0.029110382	-0.024542377	-0.00117	0.070926	-0.18993	0.009952
RP11-254I	-0.049026771	-0.074473287	0.084136	-0.0104	0.063277	-0.07319
RP11-255/	-0.05343487	-0.058239969	0.029172	0.001838	-0.01777	-0.04197
RP11-255(	-0.026294048	-0.036474775	0.065903	0.013863	-0.06161	-0.06858
RP11-255(	-0.018735795	0.105993213	0.232896	0.037437	-0.10271	-0.05962
RP11-255I	-0.056513922	-0.046626176	0.00546	-0.00478	0.005629	0.060001
RP11-257.	-0.060589435	-0.074382869	0.044834	-0.02509	0.052041	0.04998
RP11-258(	-0.002544404	-0.017604512	0.122476	-0.00205	0.035222	-0.12737
RP11-259(	-0.031494368	0.02905177	-0.05816	-0.03037	0.110276	0.136616
RP11-25I.	-0.028762019	-0.068807889	-0.06206	-0.0092	-0.03432	0.082813
RP11-263I	-0.03131234	-0.037536311	-0.05631	0.016323	-0.02221	0.156236
RP11-263I	-0.046844407	-0.045522325	-0.13425	0.017732	-0.03243	0.015096

RP11-266/	-0.037397355		-0.066846514	0.129039	-0.08768	0.280442	-0.11558
RP11-266I	-0.040585788		-0.025248297	-0.07867	-0.0019	0.030791	0.019372
RP11-271(	0.018104878		0.06609682	0.03796	-0.02672	0.096181	-0.09537
RP11-272I	-0.024764038		-0.109000692	-0.06986	-0.0092	0.010263	-0.04631
RP11-274I	-0.032101948		-0.037701644	-0.02513	-0.00419	0.03408	0.022822
RP11-274I	-0.054220521		-0.041334948	-0.00858	-0.04746	0.145764	0.014207
RP11-278.	-0.067078168		-0.091527008	0.026276	-0.01758	0.030455	-0.16552
RP11-279(	-0.04747331		0.014466693	-0.07215	0.0207	-0.02299	0.007221
RP11-27I	-0.021781896		0.023050648	0.219252	0.000657	-0.01045	-0.18594
RP11-284I	-0.05932014		-0.002496349	0.022198	-0.05516	0.141128	0.039979
RP11-285/	-0.059861635		-0.034161878	0.000476	0.022261	-0.0392	0.048382
RP11-285I	-0.042148673		-0.022116398	-0.04684	-0.00631	0.041502	0.037543
RP11-285I	0.012258623		-0.034674351	0.278871	-0.06113	0.189263	-0.2359
RP11-285(	-0.036929722		-0.016007148	0.042971	-0.00773	0.055815	0.083755
RP11-285.	-0.007197958		0.015463507	0.023817	0.011813	-0.0196	-0.08669
RP11-286I	-0.047150119		-0.041727756	-0.01681	-0.00143	########	-0.02112
RP11-288I	-0.007413013		0.080403216	-0.196	-0.01353	0.043905	0.00766
RP11-288I	-0.028400603		-0.014866278	-0.10981	0.004686	-0.02492	-0.00127
RP11-28F	-0.004908548		-0.034290267	0.171368	0.008496	-0.05584	-0.21801
RP11-292I	-0.064857859		-0.073422636	-0.0286	0.005303	-0.03426	-0.06427
RP11-295(	-4.09E-05		-0.078210075	-0.33137	0.021799	-0.05809	0.433037
RP11-295N	-0.039016641		-0.093461791	-0.0711	0.00819	-0.00136	-0.00059
RP11-295I	-0.043412433		0.010010189	0.009034	-0.0429	0.109549	-0.07499
RP11-297I	-0.022130992		-0.054119308	0.043624	-0.00035	-0.07942	-0.09849
RP11-298I	-0.054780432		-0.083080483	-0.02206	-0.00066	0.003408	-0.0007
RP11-298I	-0.037639923		-0.076877441	-0.01336	-0.00759	0.03943	-0.01455
RP11-29H	-0.012099596		0.104099007	0.195147	-0.0072	0.0555	-0.12916
RP11-29P	-0.053171881		-0.096881275	-0.01273	-0.00093	0.002874	0.024571
RP11-2E1	-0.03303327		-0.082717431	0.202923	0.034153	-0.07793	-0.16309
RP11-300/	-0.028436086		-0.029292878	-0.00358	0.025113	-0.05829	-0.03042
RP11-301(	-0.016756664		-0.007587872	0.06829	0.026589	-0.04592	-0.02561
RP11-303I	-0.089674783		-0.04266229	-0.00988	-0.05469	0.134786	0.05724
RP11-304(	-0.032684686		-0.018423235	-0.05869	-0.00704	0.028932	-0.03464
RP11-304I	-0.029515551		-0.036039689	-0.12323	0.003394	0.0092	0.080932
RP11-305I	-0.011643371		0.032422792	-0.01737	0.030986	-0.06763	-0.00567
RP11-305I	-0.001864506		-0.047249769	0.069297	0.031083	-0.02588	-0.01907
RP11-305I	-0.006720198		0.118142077	0.154456	0.11469	-0.34839	-0.12505
RP11-305(	-0.084813275		0.045355337	-0.02298	-0.02668	0.050205	0.027802
RP11-305(	-0.013202975		-0.067498966	-0.04778	-0.0085	0.044453	-0.27865
RP11-307(	-0.051586451		-0.040645308	0.078153	-0.00972	0.024319	0.049927
RP11-307I	-0.039740871		-0.044169027	-0.03212	0.021469	-0.04007	0.099687
RP11-308I	-0.043515695		-0.062381929	0.005782	-0.01932	0.079158	0.171674
RP11-309(	-0.018861314		0.02600734	-0.08533	0.063876	-0.20812	0.053364
RP11-310.	-0.031381589		-0.028638043	-0.05714	0.0063	-0.00594	0.190124
RP11-313(	-0.060978906		-0.071341338	-0.01777	-0.01083	0.017651	0.053383
RP11-314I	-0.017539788		-0.030077311	0.051248	0.018711	-0.02826	-0.14787
RP11-315I	-0.041744727		-0.103996699	-0.03636	0.023343	-0.04043	0.066314
RP11-318/	-0.022014268		0.018071345	0.093173	-0.01252	0.025505	-0.02988
RP11-319I	-0.029663667		-0.083101007	0.165978	-0.01209	-0.03294	-0.09485
RP11-319(	-0.027992979		-0.059954684	0.149428	0.043025	-0.12614	-0.1207
RP11-31K	-0.068437453		-0.047477358	-0.02075	-0.02443	0.029198	-0.07144

RP11-320I	-0.062792535	-0.069714213	-0.02859	-0.01558	0.044668	-0.02114
RP11-320N	-0.041517019	-0.053448394	-0.03409	0.01411	-0.01201	0.069999
RP11-324J	-0.018386272	-0.027815525	0.008154	-0.00606	-0.00151	-0.02365
RP11-324I	-0.040970439	-0.041775263	0.013404	0.018937	-0.02583	0.050052
RP11-326I	-0.016944451	-0.031125057	-0.01808	0.007875	-0.00433	0.076175
RP11-326I	-0.021876005	0.003011678	-0.10605	0.01566	-0.02322	0.053715
RP11-327J	-0.032053561	-0.083448797	0.019328	-0.00611	0.028066	0.035162
RP11-329I	-0.033332005	-0.031821863	-0.01068	0.001112	0.016106	-0.00666
RP11-329I	-0.049986551	-0.003549831	0.004813	0.019112	-0.0288	0.042065
RP11-330N	-0.037995422	-0.017058123	0.037198	0.016058	-0.03997	-0.03683
RP11-330(	-0.02556007	-0.053167148	-0.10974	-0.00354	0.028518	0.272149
RP11-332I	-0.039620768	-0.114904562	-0.06582	-0.01443	0.029115	0.042837
RP11-333I	-0.042032692	-0.093484175	0.00166	-0.00167	0.037804	0.025249
RP11-333(	-0.022010236	-0.002934458	-0.01497	-0.00209	-0.02939	-0.09071
RP11-334(	0.009122395	-0.024311453	0.184092	-0.03637	0.112622	0.26132
RP11-334J	-0.010718723	-0.065971329	0.111268	-0.01789	-0.06584	-0.20733
RP11-338I	-0.034293424	-0.017242941	0.115809	-0.00505	0.030575	0.142469
RP11-338N	-0.025174411	-0.029931153	-0.00498	-0.01052	-0.03637	-0.04791
RP11-339A	-0.042841401	0.009513858	0.142694	-0.00156	0.016832	-0.07887
RP11-339I	-0.019411596	-0.06090407	0.145944	0.048643	-0.16329	-0.01198
RP11-3304	-0.008107248	0.016452779	0.130444	0.001693	0.004157	0.20851
RP11-340A	-0.094722317	-0.100724098	0.052171	-0.03577	0.13944	0.057622
RP11-341N	-0.041779421	-0.113498061	-0.07442	-0.02268	0.077279	0.155593
RP11-342I	-0.041848481	-0.042307564	-0.08825	0.002182	0.01905	0.01661
RP11-343I	-0.023116643	-0.016682007	0.030136	0.007183	0.000252	-0.04078
RP11-347I	-0.041593745	-0.020224495	-0.05276	0.01377	-0.019	-0.02659
RP11-348I	-0.04855808	-0.082502683	0.110121	-0.00818	0.022324	-0.10232
RP11-348N	-0.016076028	-0.078583831	0.057668	0.003934	0.000471	-0.07846
RP11-348I	-0.017303533	0.2210583	-0.11257	-0.02845	0.104821	-0.02158
RP11-349I	-0.01751705	-0.032525255	-0.00959	-0.02132	-0.02714	-0.06794
RP11-350I	0.011897756	0.06025645	0.096173	0.032286	-0.05847	0.0667
RP11-352I	-0.052152675	-0.030127802	-0.01234	-0.0403	0.102146	-0.02549
RP11-352(	-0.039601095	-0.115008467	0.002373	0.017376	-0.03866	-0.0359
RP11-353N	-0.005617772	-0.06660347	-0.02114	0.002721	-0.13053	-0.04702
RP11-353N	-0.048999855	-0.046670838	0.175052	0.011668	-0.03259	-0.03353
RP11-354I	-0.030508121	-0.045064637	-0.00613	0.040559	-0.11261	0.026207
RP11-355I	-0.005793347	0.062698511	0.089724	-0.09711	0.294879	-0.03521
RP11-355I	-0.090957642	-0.07067761	0.021842	-0.00312	0.036594	-0.06422
RP11-356I	-0.024001939	-0.046831999	-0.08338	-0.00552	0.024239	-0.09504
RP11-357(	-0.033067828	-0.103906609	-0.00976	0.049337	-0.11891	0.009416
RP11-357(	-0.05674338	-0.035366374	0.059704	0.007916	-0.03672	0.134579
RP11-359I	-0.035989156	-0.076806387	0.004251	0.028555	-0.05902	0.027022
RP11-359I	-0.054955315	-0.056333119	0.105554	-0.01453	0.057108	-0.02425
RP11-360A	-0.035007727	-0.035771964	-0.09524	-0.02165	0.07578	-0.13645
RP11-360I	-0.035216853	-0.04515672	0.096726	-0.01112	0.048338	0.063946
RP11-362I	-0.032990662	-0.018851577	0.08592	-0.02792	0.05255	-0.0941
RP11-362I	-0.03175131	-0.041500835	-0.01814	-0.03466	0.102427	-0.0434
RP11-363I	-0.027333229	-0.043073469	0.206821	-0.09005	0.278902	-0.14153
RP11-365I	-0.038902802	-0.021796957	0.099495	-0.03754	0.10089	-0.01773
RP11-366I	-0.061520738	-0.082059549	-0.09768	-0.05697	0.156288	0.276245
RP11-369(	-0.040969526	-0.015499296	-0.04905	-0.01053	0.04496	-0.06292

RP11-36B(	-0.03520195	-0.00930288	0.057377	0.030963	-0.08829	-0.07169
RP11-373I	-0.003409705	0.065397471	-0.06467	-0.03786	0.099146	-0.00798
RP11-375I	-0.036176536	-0.05483638	0.048318	0.036556	-0.08112	-0.01985
RP11-378/	0.015147619	0.11114627	-0.02933	-0.05861	0.181517	0.019872
RP11-378.	-0.026033344	-0.034118331	0.075818	-0.02897	0.094638	-0.10663
RP11-379I	-0.021921833	0.049258336	0.005203	-0.00816	0.041717	0.008742
RP11-379I	-0.01811345	0.050553892	0.107167	-0.01701	0.076774	0.216587
RP11-37C'	-0.027960386	0.034460839	0.040597	0.006685	-0.01043	-0.05765
RP11-381I	-0.026444274	-0.053983099	0.11071	0.005681	-0.01143	-0.16603
RP11-382I	-0.019262852	-0.086958171	-0.13286	-0.01977	0.070587	0.19837
RP11-384I	-0.035503794	-0.019740302	0.004221	0.010233	0.002028	0.001273
RP11-384I	-0.031486731	-0.04746307	-0.00044	0.005969	-0.00065	-0.09823
RP11-385I	-0.020253341	-0.040470981	0.077075	0.00794	-0.02398	-0.1489
RP11-388M	-0.025682187	-0.047607741	-0.07674	0.002646	0.011074	-0.1043
RP11-388M	-0.030641534	-0.049078663	0.093802	-0.00946	0.006944	-0.01152
RP11-389(	-0.003817379	-0.085144225	-0.14864	-0.06165	0.168573	0.123163
RP11-389(	-0.018992173	-0.088883603	0.053914	0.006381	0.008032	-0.07282
RP11-389(	-0.0287607	-0.061140573	-0.02112	0.015987	-0.03624	-0.13582
RP11-38C	-0.02890726	-0.043057819	-0.05029	7.82E-05	-0.03897	2.75E-05
RP11-38C	-0.03634473	-0.065236069	-0.06012	0.019797	-0.02583	0.000721
RP11-38L	-0.039920182	-0.014505041	0.036045	0.005452	0.018168	0.006542
RP11-38P	-0.007342259	-0.045614488	-0.11812	-0.0175	0.065769	0.066945
RP11-391M	0.004263176	-0.010241586	0.179325	0.035533	-0.06631	-0.12212
RP11-394	-0.028727773	-0.056677532	0.033719	-0.02177	0.049612	0.091577
RP11-395I	0.02380016	0.109092836	0.061876	0.006614	-0.01574	-0.07495
RP11-395	-0.007718483	-0.018852957	0.117213	-0.01011	0.053945	-0.24417
RP11-395M	-0.034754292	-0.056748064	0.038159	0.014925	-0.02506	-0.09055
RP11-396I	-0.027543894	-0.009531593	-0.02128	-0.04509	0.151648	0.217934
RP11-397/	-0.025663593	-0.049875411	-0.00315	0.002161	-0.02338	-0.15073
RP11-399I	-0.037004182	-0.051265677	-0.05639	-0.01056	0.046263	0.030416
RP11-399(	-0.020333571	-0.021712132	-0.00487	0.051565	-0.16504	-0.00582
RP11-39H	-0.035585083	-0.049011609	-0.05601	0.028906	-0.08572	-0.0063
RP11-3L8.	-0.061969668	-0.009123678	-0.04633	0.028278	-0.0923	-0.10892
RP11-400M	-0.035128802	-0.094245557	-0.01491	0.028092	-0.07134	0.028206
RP11-400M	-0.049151952	-0.037924275	-0.02284	-0.01876	0.068724	0.001291
RP11-401I	-0.016129412	-0.009528145	0.063931	-0.02039	0.045871	-0.12032
RP11-402I	-0.018248043	-0.062438571	0.045412	0.013293	-0.02764	0.039386
RP11-402(	-0.042667666	-0.117267307	0.050336	0.017646	-0.02467	-0.03531
RP11-403M	-0.046565239	-0.083829769	-0.01257	0.009972	-0.00365	0.161241
RP11-403I	0.012576298	-0.018058897	-0.04955	-0.06465	0.107569	0.099064
RP11-403I	0.079740176	0.076416541	0.043406	0.030576	-0.03627	-0.23767
RP11-408	-0.037857172	-0.073030553	-0.07605	0.050706	-0.12609	0.161364
RP11-410(	-0.03670261	0.005358529	-0.18261	-0.01436	0.006002	0.042548
RP11-410I	-0.047541659	-0.083056061	-0.04694	0.015069	0.003774	0.029436
RP11-410I	-0.042734063	-0.053919329	0.02744	-0.00258	0.014954	-0.02687
RP11-410I	0.009575444	-0.127603959	0.121707	0.033196	-0.07271	0.041643
RP11-413I	-0.046783275	-0.071223261	-0.0729	0.003364	0.0263	-0.06952
RP11-414I	-0.036753779	-0.12745863	0.014249	0.048406	-0.12854	0.095016
RP11-416	-0.029003459	-0.077065368	0.122888	0.035309	-0.1028	-0.16119
RP11-416M	-0.031717539	-0.116501222	0.052459	0.001619	0.006793	0.010107
RP11-420I	-0.020987542	-0.044571935	0.058578	-0.09884	0.292734	-0.1265

RP11-421I	-0.021158214	0.084587321	0.097098	0.000101	0.017812	-0.06927
RP11-421I	-0.032758866	-0.03137195	-0.00611	0.016819	-0.03322	0.120683
RP11-422I	-0.017580256	0.046678598	0.18294	0.018435	-0.04225	-0.0193
RP11-425I	-0.01336982	-0.016382142	0.027134	0.027069	-0.03453	-0.00301
RP11-426(	-0.001703959	0.034071644	-0.20351	-0.0371	0.10623	0.266386
RP11-429I	-0.031410018	-0.099569104	0.131349	-0.00363	-0.00706	-0.04854
RP11-432、	-0.026436213	-0.021343987	-0.19014	0.025356	-0.08067	0.010308
RP11-432N	-0.013860236	-0.076043532	-0.04199	-0.00913	-0.08051	-0.1645
RP11-433/	-0.032483148	-0.096758192	0.013814	0.008434	-0.04767	0.08539
RP11-435(	-0.023276683	-0.039061366	########	-0.00652	-0.01487	-0.04962
RP11-436I	-0.027847772	0.002510801	-0.16422	0.035098	-0.09859	0.017279
RP11-439I	-0.037382869	-0.00238567	-0.10388	0.017062	-0.02797	-0.02847
RP11-440I	-0.017878301	-0.007697218	0.068955	0.009302	-0.06888	-0.07837
RP11-440I	-0.039948886	-0.055127624	-0.09108	-0.0057	-0.01803	-0.10771
RP11-442(	-0.031380333	-0.006041442	-0.05462	0.011455	-0.02791	-0.03983
RP11-443I	-0.012068319	-0.104991676	-0.32995	-0.01268	0.024157	0.293011
RP11-443(	-0.021608631	-0.0035892	0.053094	-0.00828	-0.0646	-0.04377
RP11-445I	-0.031520611	0.027102624	-0.06416	0.058023	-0.14187	0.059476
RP11-445I	-0.038878489	-0.000916405	-0.01442	0.012516	-0.01206	0.056397
RP11-448/	-0.037402351	0.016048336	########	0.000803	0.003592	0.1206
RP11-44F	-0.017345096	-0.066287499	-0.0029	0.04149	-0.14295	-0.13842
RP11-44F:	-0.030047098	0.00723721	0.039287	0.00499	-0.02117	-0.21806
RP11-44N	-0.039321055	-0.065564778	0.029172	-0.00747	0.045106	0.029276
RP11-452I	-0.01456174	0.052941211	0.173837	0.036567	-0.10202	-0.20931
RP11-454I	-0.029040514	0.01402981	-0.1047	0.019377	-0.02109	-0.04547
RP11-454I	-0.043179109	-0.011695155	0.066585	0.038428	-0.08694	-0.0381
RP11-456I	-0.0287877	-0.030715729	-0.02422	0.006408	-0.0283	-0.03727
RP11-456(	-0.029273367	-0.049734983	-0.01824	-0.00713	-0.01555	-0.15022
RP11-456I	-0.015909079	-0.034467616	-0.09577	0.006685	-0.01074	-0.00103
RP11-457	-0.032097055	-0.069832475	-0.08943	-0.01563	0.041476	0.089233
RP11-457I	-0.064634086	-0.124475017	-0.05361	0.001529	-0.01728	0.130497
RP11-45M:	-0.0344306	-0.056452117	0.027671	0.006375	-0.01147	0.045922
RP11-45M:	-0.026156557	-0.057439316	-0.04496	0.021359	-0.11415	0.059848
RP11-461/	-0.022502147	-0.048205489	0.09547	0.006024	-0.0071	-0.06686
RP11-461I	-0.0633008	-0.007163694	-0.05342	-0.00394	0.009583	0.103038
RP11-464I	-0.020190401	-0.045431608	0.153795	0.040151	-0.11035	-0.01692
RP11-465I	-0.022124276	-0.105314928	0.084645	-0.00812	-0.0438	-0.06934
RP11-465	-0.043941004	-0.006491981	0.026382	0.01089	-0.00725	-0.05693
RP11-466/	-0.039318035	0.035302967	0.084802	0.030426	-0.06675	-0.02834
RP11-466I	-0.051690235	-0.077193516	-0.03536	0.012159	-0.02267	0.062951
RP11-467I	-0.041441598	-0.043331064	0.01942	-0.02643	0.074076	0.081631
RP11-468I	0.014196615	0.006806575	-0.018	-0.07396	0.224218	0.112506
RP11-469N	0.013300675	0.066041295	0.020664	0.021723	-0.05798	-0.02187
RP11-470N	-0.029049491	-0.010726181	0.024285	0.015918	-0.0442	-0.13181
RP11-471I	-0.014927495	0.085093944	0.171204	-0.03074	0.083969	-0.1783
RP11-471(	-0.023910586	-0.02915247	-0.03226	0.065634	-0.15378	-0.00617
RP11-471N	-0.062991032	-0.001877267	0.014762	0.006566	-0.03364	-0.06708
RP11-472I	-0.035318102	-0.044193325	-0.09656	0.017446	-0.04394	-0.21139
RP11-473	0.028932483	0.041518631	-0.11584	0.012108	-0.06013	0.05756
RP11-474I	-0.058641188	-0.022719034	0.041176	0.003462	-0.02044	0.092639
RP11-474I	-0.01359266	-0.091769366	0.004848	-0.0106	-0.07465	-0.05874

RP11-475/	-0.139822618	-0.099659287	-0.01668	0.018491	0.101207	0.021371
RP11-476I	-0.022156102	-0.021310061	-0.13336	-0.02197	0.085267	0.041166
RP11-477I	-0.012977119	0.006089985	0.133181	-0.02643	0.095233	-0.22822
RP11-480/	-0.010104245	-0.060188492	0.009295	0.032426	-0.16498	-0.26831
RP11-480I	-0.03373908	0.042793986	0.091078	0.015481	-0.03764	-0.1099
RP11-480	-0.026259881	-0.085933052	-0.04296	-0.00768	-0.05583	-0.04673
RP11-483(	-0.029391313	-0.049118784	-0.03912	-0.01691	0.037448	-0.07896
RP11-483	-0.016544973	-0.081617858	0.017125	-0.04336	0.102854	0.246568
RP11-486/	-0.056282178	-0.039379283	-0.07018	0.022491	-0.06914	-0.03011
RP11-486(	-0.022492846	-0.047825421	0.25573	0.011674	-0.02473	-0.29741
RP11-488I	0.035238459	0.147103833	0.035453	-0.00817	0.027043	-0.11045
RP11-489I	-0.001540308	-0.03725672	0.205882	-0.02269	0.064253	-0.14161
RP11-489(	-0.103898321	-0.102612924	-0.0088	-0.01584	0.044792	0.122449
RP11-48B	-0.007394984	0.052940715	-0.01772	0.000214	0.021437	-0.19642
RP11-490(	-0.048945815	-0.011985052	0.018852	-0.04426	0.165109	0.179725
RP11-493I	-0.027562074	-0.06720485	0.061722	-0.00167	0.010119	-0.1208
RP11-495I	-0.040055808	-0.07168729	0.007374	-0.00447	0.051537	0.114735
RP11-495I	-0.041426078	0.001972756	-0.06372	-0.011	0.048713	-0.11524
RP11-495I	-0.028155482	-0.027247944	-0.02989	0.005538	-0.02606	-0.06196
RP11-495I	-0.038899738	-0.040098099	-0.04754	0.007879	0.003842	-0.04425
RP11-496	-0.021270229	-0.013886452	0.056977	-0.00027	-0.00606	-0.08827
RP11-498(	-0.00833396	0.089868238	-0.01508	0.108734	-0.27735	0.072333
RP11-498(	-0.025748477	-0.027951584	0.048565	-0.01064	0.017622	-0.02987
RP11-498I	-0.021642819	-0.026236438	0.107602	0.026124	-0.04815	0.149826
RP11-499I	-0.01928148	0.006800284	0.03318	0.042407	-0.14145	-0.10961
RP11-49K	-0.033481507	-0.020858427	0.091776	0.034128	-0.08012	0.037611
RP11-4M2	-0.025732141	-0.094238566	0.254826	0.000514	0.021223	-0.17367
RP11-502I	-0.048335111	-0.009149126	-0.06944	0.002645	0.015856	0.070115
RP11-503(	-0.033653803	-0.106936687	-0.07568	0.006273	-0.00334	0.255942
RP11-504/	-0.071885061	-0.046446004	0.011844	-0.01532	0.012479	-0.07754
RP11-505I	-0.051815714	-0.0252716	-0.16266	-0.00366	0.034699	0.104353
RP11-506I	-0.034050009	0.034090561	-0.06021	-0.01044	0.052111	-0.12433
RP11-506I	-0.069360197	0.012139067	-0.13346	-0.00813	0.013444	0.008987
RP11-506(	-0.05979653	-0.026674699	0.05039	0.015284	-0.0339	0.051823
RP11-508I	-0.034607775	-0.047951774	0.069388	0.002806	-0.00688	0.017457
RP11-50B	0.000380012	-0.009119582	0.137999	-0.00657	0.012706	-0.08138
RP11-50E	-0.009703388	0.071672613	-0.13977	-0.00525	0.05435	0.159727
RP11-510(	-0.039824426	-0.033695974	0.020611	0.056966	-0.13594	-0.11989
RP11-510(	-0.046103822	0.013020362	-0.00873	-0.01866	0.048424	-0.14672
RP11-513I	-0.030913881	-0.070818411	0.084533	-0.00984	0.030852	-0.06224
RP11-513I	-0.046181816	-0.033001584	-0.026	-0.00012	0.030642	0.045157
RP11-515(	-0.030077516	-0.024753224	0.019299	-0.02683	0.083422	-0.05895
RP11-517I	-0.030591361	0.053716323	0.000192	-0.00579	0.044647	0.09628
RP11-517(	-0.022915685	-0.098758907	-0.00538	-0.00207	-0.00142	0.05524
RP11-521I	-0.026441758	-0.017412444	0.011758	-0.00383	-0.0597	-0.07955
RP11-521	-0.029371309	-0.067621735	-0.00997	0.021919	-0.04044	0.071092
RP11-521(	-0.04569432	0.01774704	-0.00945	0.018784	-0.02831	0.029249
RP11-524(	-0.042216113	-0.039910923	0.066996	-0.0095	0.046962	-0.15264
RP11-524I	-0.045425329	-0.058903799	0.010323	########	0.030429	-0.00256
RP11-52A	-0.032072267	-0.067379802	0.070627	-0.00616	0.026133	-0.01182
RP11-532I	-0.02315166	-0.107589353	-0.13838	-0.01197	0.037077	-0.04915

RP11-533I	-0.026078078	-0.013351974	0.076134	0.016091	-0.04436	0.049915
RP11-533I	-0.028101127	-0.068643081	0.088219	0.003567	-0.01399	-0.10157
RP11-534I	-0.013292433	-0.051805454	0.150158	0.007554	0.003579	0.077968
RP11-536(	-0.02145885	-0.124566656	0.104483	0.018205	-0.03523	-0.00342
RP11-539(	-0.057720987	-0.033399754	0.092589	-0.0192	0.051386	-0.04006
RP11-539I	-0.033669362	-0.002327006	-0.13042	-0.02304	0.053098	-0.09686
RP11-539I	0.001065277	-0.040997154	0.307742	-0.02159	0.082491	-0.03911
RP11-53A1	-0.044417857	-0.029300426	-0.0083	0.008319	-0.00649	-0.10522
RP11-53B2	-0.027761065	-0.050388442	0.048746	-0.02664	0.057387	-0.04765
RP11-53O1	-0.002424028	0.086582631	0.069448	0.002954	-0.00086	-0.15391
RP11-540(	-0.007200698	-0.061739404	0.033071	-0.01655	-0.08146	-0.18956
RP11-543(	-0.02767569	-0.042069724	0.043945	0.003357	-0.05887	-0.18474
RP11-544I	-0.046691518	-0.008259474	-0.00148	0.006588	-0.00066	-0.10168
RP11-546(	-0.039614372	0.044974357	-0.06187	0.031701	-0.0776	-0.11324
RP11-548I	-0.007417761	-0.03118429	-0.1857	0.002226	-0.04309	0.049757
RP11-548M	-0.027567881	-0.062433898	-0.02402	0.001153	-0.02677	-0.02756
RP11-549J	-0.022729163	0.039553777	-0.00548	-0.01981	0.059052	-0.19186
RP11-54K1	0.014942054	-0.047643216	0.083065	0.076014	-0.28423	-0.1288
RP11-550I	-0.04778296	-0.02044203	-0.02097	-0.02793	0.106961	0.106266
RP11-552I	-0.039536221	-0.079494684	-0.15718	0.014378	-0.017	0.251697
RP11-552M	-0.027986096	-0.052379867	0.148341	-0.017	0.07074	-0.10095
RP11-554I	-0.022987715	-0.006872354	-0.10447	0.002564	-0.00596	0.076927
RP11-554J	0.009397367	-0.050408606	0.2305	-0.03376	0.125826	-0.0463
RP11-559M	-0.020497001	-0.023276316	-0.05651	0.015732	-0.08797	-0.01567
RP11-55K1	-0.034675163	0.064613058	-0.08206	0.002643	0.018618	-0.05801
RP11-560A	-0.031405865	-0.034052597	-0.00236	0.016772	-0.04245	0.001639
RP11-560(	-0.033770182	-0.065519296	-0.00629	########	0.017051	0.06195
RP11-567(	-0.026202053	-0.076234838	-0.08112	-0.02357	0.070366	-0.0802
RP11-567I	-0.024587375	-0.013906972	-0.09456	0.042952	-0.10944	0.082211
RP11-568I	6.42E-05	-0.090705579	-0.14631	-0.05526	0.156746	0.193451
RP11-573I	-0.041278766	-0.060798496	-0.09634	0.001818	0.01615	-0.05813
RP11-574I	-0.02373468	-0.031070502	-0.02652	0.076253	-0.15484	-0.01743
RP11-579(	-0.06054698	-0.032058565	-0.04126	-0.03422	0.060768	-0.0891
RP11-585I	-0.005019535	0.031675937	0.285442	0.008126	0.009152	-0.4012
RP11-586I	-0.061610961	-0.051187484	-0.0727	-0.02555	0.058575	-0.00204
RP11-587I	-0.039761154	-0.0477062	-0.05572	0.046086	-0.10913	-0.17345
RP11-589I	0.013212884	-0.056141518	0.090603	-0.06028	0.111525	-0.13926
RP11-58O3	-0.083826695	-0.051723627	-0.14348	0.015862	0.005017	0.083251
RP11-59H7	-0.04268095	-0.053688556	-0.05496	0.012324	-0.05198	0.00079
RP11-5C23	0.012914729	0.068400392	0.09147	-0.03497	0.136614	-0.00972
RP11-5C23	-0.007641765	0.039107442	0.118692	-0.05405	0.168122	-0.03234
RP11-5N11	-0.035925997	-0.077510602	0.03354	0.034773	-0.10413	-0.02634
RP11-60A2	-0.020115931	-0.058668687	0.017068	0.014567	-0.07009	-0.09054
RP11-613M	-0.011815579	-0.03542438	0.307499	0.042327	-0.0688	-0.1773
RP11-619I	-0.036418355	-0.010763128	0.013061	0.010042	0.001391	-0.12015
RP11-619I	-0.0375107	-0.028537065	-0.03294	0.031132	-0.0752	0.065933
RP11-61L1	-0.037302813	-0.038932514	0.071862	0.019731	-0.02994	-0.00421
RP11-61L1	-0.012393983	0.080828233	-0.12458	0.015792	-0.05584	0.008776
RP11-629I	-0.031125985	0.028052176	0.066615	0.026862	-0.10019	-0.09194
RP11-629(	-0.009575998	-0.009837529	0.098246	0.064777	-0.14782	0.201143
RP11-631I	-0.0232895	-0.009972576	-0.09197	-0.00161	0.016857	0.052645

RP11-634I	-0.031081876	-0.040682057	-0.09596	0.005223	-0.01542	-0.02233
RP11-635I	-0.024026525	-0.108450773	0.117241	-0.04438	0.149976	-0.00866
RP11-63A	-0.041444255	-0.020477063	-0.08681	0.069169	-0.17281	-0.08979
RP11-63A	-0.038441227	-0.043042546	0.050943	0.001435	0.016929	-0.07543
RP11-63D	-0.035273307	-0.0549437	0.003842	0.013891	-0.01346	-0.10294
RP11-63K(	-0.029757821	-0.134666785	-0.0767	0.00864	-0.10349	-0.07139
RP11-642I	-0.037136934	-0.01725025	0.172266	0.008474	0.003961	-0.13135
RP11-644I	-0.017103467	-0.009374813	0.003135	0.020046	-0.14152	0.062032
RP11-646I	-0.040540126	-0.02039146	0.079409	0.00749	-0.00034	-0.08077
RP11-646	-0.025593941	-0.040957117	0.063746	0.005624	-0.031	-0.15387
RP11-649	-0.021862451	-0.046371682	0.031603	0.00991	0.004272	0.05124
RP11-650I	-0.027228576	-0.001795563	-0.02799	-0.00429	0.02698	-0.00221
RP11-654	-0.028312462	-0.026642964	0.071364	0.003207	0.015669	0.078313
RP11-65D	-0.031756884	-0.033784222	-0.19035	-0.01067	0.062368	0.035084
RP11-65J	-0.046125988	-0.028967855	-0.01111	0.012903	-0.00058	0.001958
RP11-65L	-0.016452762	-0.086750617	0.109464	-0.00941	-0.08954	-0.06994
RP11-661	-0.03371699	-0.057311389	-0.18361	0.027007	-0.03591	0.179711
RP11-663I	-0.031309694	-0.071302346	-0.11182	-0.02293	0.081585	-0.08193
RP11-664I	-0.005928973	-0.072464664	0.352262	-0.04731	0.12075	-0.21763
RP11-665(	-0.050503531	0.013687587	-0.0776	0.017502	-0.06673	0.045412
RP11-669(	-0.035205286	-0.051610213	-0.05864	0.001617	0.013584	0.057858
RP11-669	-0.044393877	0.004891564	-0.04458	-0.02604	0.097582	-0.08077
RP11-669I	-0.036725727	-0.059441052	-0.12905	0.010993	-0.00447	-0.04307
RP11-66N	-0.033371656	-0.03583077	-0.08109	0.007749	0.01304	0.034523
RP11-66N	-0.002446923	0.004348753	0.061939	0.00911	0.022487	-0.01425
RP11-672I	-0.022569576	0.097383387	0.088171	-0.10749	0.323914	-0.11407
RP11-673I	-0.043369437	-0.032682037	-0.04746	0.008619	0.011874	0.097101
RP11-674I	-0.110208133	-0.121143561	0.097536	-0.01155	0.064896	-0.05481
RP11-676	-0.027895493	-0.016793285	0.021809	-0.02057	0.067651	0.001419
RP11-676	-0.042684666	-0.014328255	-0.00045	0.017065	-0.01825	-0.03842
RP11-677(	-0.027410939	-0.019021257	-0.05033	-0.006	0.01096	-0.14594
RP11-678(	-0.037173358	-0.073558243	-0.11242	0.009372	-0.00766	-0.02755
RP11-67L	-0.03777742	-0.009270994	-0.09851	0.042153	-0.09829	-0.08753
RP11-680(	-0.034364379	-0.00070736	-0.03929	0.013002	-0.01631	-0.02474
RP11-680I	-0.026640992	-0.010895253	0.048686	-0.00885	0.033818	-0.19973
RP11-680I	-0.03919095	-0.056200319	-0.04665	0.011648	0.001439	-0.18084
RP11-680(	-0.021870836	0.046042431	0.259512	0.024802	-0.05696	-0.17626
RP11-686I	0.015495664	0.145137466	-0.11192	-0.01477	0.061447	0.353712
RP11-687I	-0.02641064	0.032921564	0.243191	-0.01432	0.03517	-0.13052
RP11-68I	-0.013044703	-0.030596695	-0.16892	-0.02915	0.076152	0.031059
RP11-690	-0.000180748	0.064504841	0.163224	-0.01642	0.072075	-0.16379
RP11-692I	-0.0363899	-0.025357009	0.105604	-0.00981	0.055402	-0.02199
RP11-69I	-0.030303607	0.00542832	0.169437	-0.00482	0.032577	-0.13948
RP11-6F2.	-0.040400973	-0.026970275	0.096833	-0.00959	0.067167	-0.12558
RP11-6I2.	-0.018589898	-0.06885573	-0.31032	0.005533	-0.00632	0.286072
RP11-700I	-0.027562561	-0.061101459	0.019305	-0.02606	-0.01889	-0.06751
RP11-700	-0.010826965	-0.030805031	0.102051	0.005762	0.008414	-0.02636
RP11-701	-0.034007139	-0.08229352	-0.00718	-0.00384	0.018092	0.092189
RP11-708	-0.022923395	0.032486756	0.127214	0.037827	-0.08929	-0.10067
RP11-710(	-0.076917496	-0.054717937	0.027186	-0.02757	0.068086	0.093606
RP11-714I	-0.043914362	-0.054069786	-0.14595	0.042811	-0.09326	0.152671

RP11-715.	-0.035182756	-0.079664756	0.025045	0.009792	-0.09607	-0.0554
RP11-722I	-0.012000925	0.046709148	0.061724	0.020019	-0.03547	-0.1099
RP11-727/	-0.01301275	0.059682342	0.126292	-0.00626	0.024977	-0.1337
RP11-727I	-0.026534417	-0.005784561	0.058613	-0.02278	0.075849	-0.06679
RP11-728I	-0.011248186	-0.083539532	-0.12028	-0.03829	0.124044	0.499292
RP11-731.	-0.113439506	-0.07286426	0.077109	-0.02724	0.008965	-0.06426
RP11-732/	-0.0096238	0.105286238	0.085691	0.004881	0.000745	-0.13424
RP11-736I	-0.03587594	-0.086866148	-0.16313	0.010685	-0.01462	0.078777
RP11-73K9	-0.038691682	-0.062671304	0.011283	0.009166	-0.03568	0.138478
RP11-73M!	-0.020054593	0.069972037	-0.03081	0.017883	-0.02118	0.051911
RP11-73M!	0.032873169	-0.014055261	-0.01867	0.032137	-0.11495	-0.14062
RP11-740(	-0.043881565	-0.009477421	0.059308	-0.00076	0.021331	0.012818
RP11-742I	-0.059580126	-0.107827936	0.042258	0.008036	-0.01909	0.045238
RP11-744I	-0.072788221	-0.085083629	-0.02842	-0.01777	0.056845	0.027564
RP11-747I	-0.002338325	0.059616356	0.275462	-0.02521	0.071251	-0.18656
RP11-748I	-0.035271698	-0.087600449	-0.08306	0.036947	-0.08805	0.055997
RP11-749I	-0.039453468	-0.020401267	-0.08412	-0.01672	0.060405	0.037333
RP11-753/	-0.044148828	-0.028992751	0.021611	0.016681	-0.0464	0.043645
RP11-753/	-0.027954514	0.004442565	-0.02897	-0.00142	-0.01017	-0.14607
RP11-753I	-0.040434428	-0.039716512	-0.10221	0.01948	-0.03386	-0.12021
RP11-757I	-0.051246191	-0.032770494	-0.05152	-0.02664	0.086596	-0.03221
RP11-75C9	-0.081678329	-0.090633617	0.041371	-0.02858	0.063595	-0.0738
RP11-764I	-0.05243048	-0.04117152	-0.04503	0.032745	-0.07657	0.010979
RP11-769(	-0.009099335	0.243566813	0.058221	0.031419	-0.0977	-0.18117
RP11-774(	-0.020044281	0.01283323	-0.08229	-0.00831	0.044899	-0.04059
RP11-77H9	-0.026100191	-0.145762165	0.010085	-0.01385	0.059261	-0.2271
RP11-783I	-0.022685059	-0.101157891	0.009033	0.028858	-0.07731	0.038592
RP11-787I	-0.024166024	-0.014281153	0.019141	0.086821	-0.21515	0.003278
RP11-791M	-0.037357697	-0.011825968	-0.04159	-0.00336	0.029696	0.07284
RP11-792/	-0.012679052	0.029443984	-0.04001	-0.02032	0.087019	0.187497
RP11-793I	-0.013526351	-0.010799411	-0.06394	0.004132	-0.01897	0.029607
RP11-796I	0.001693361	-0.112838433	0.124568	-0.03905	0.039012	-0.17322
RP11-798M	-0.053748627	0.000401854	0.005033	0.000444	-0.01913	0.136286
RP11-799I	-0.028070122	-0.009508265	0.289373	-0.00566	0.024899	-0.09762
RP11-79P5	-0.031069518	-0.07824263	-0.09023	0.002208	0.009612	0.078988
RP11-79P5	-0.023914538	-0.016640212	0.30793	0.024899	-0.05585	-0.02819
RP11-7F1'	-0.082164293	-0.02630403	0.006029	-0.0174	0.042914	-0.02633
RP11-803I	-0.01190009	0.054985925	-0.13087	0.07168	-0.19699	0.046928
RP11-804I	-0.001833736	-0.012037807	-0.16344	-0.00957	0.053777	0.170392
RP11-805I	-0.025775103	-0.0669273	-0.03131	-0.02067	-0.04037	-0.04742
RP11-806I	-0.03812948	-0.004667368	-0.0636	0.027945	-0.05808	-0.08633
RP11-809I	-0.064633953	-0.092576703	0.003063	-0.01148	0.020739	-0.04894
RP11-819(	-0.011694181	0.071018673	0.136493	-0.0204	0.065671	-0.15991
RP11-82L!	-0.014877225	-0.047863122	0.064338	-0.0211	-0.05475	-0.12859
RP11-83N9	-0.027767194	-0.024422827	-0.12438	0.096047	-0.30421	0.005965
RP11-843I	-0.01497193	-0.044028501	-0.00585	-0.01847	-0.02375	-0.1278
RP11-845(	-0.023149292	0.001603682	0.008747	0.01527	-0.06714	0.084902
RP11-846I	0.009105673	0.049922969	0.201602	-0.01418	0.04207	-0.24065
RP11-846I	-0.009311231	0.012253044	0.134485	-0.01254	0.04787	-0.16625
RP11-847I	-0.029008791	-0.051145441	0.070783	0.003967	0.002458	0.039925
RP11-84D!	-0.028475388	-0.090985375	0.129713	-0.00593	-0.03258	0.043949

RP11-85A	-0.040260203	-0.065653524	-0.01952	0.009829	-0.08208	0.020637
RP11-862	-0.032651142	-0.081072216	-0.0848	-0.01019	0.055989	-0.12844
RP11-873	-0.03274929	0.029364395	-0.01384	0.011721	-0.01955	0.074645
RP11-874	-0.018810454	-0.03143698	-0.00244	-0.24056	0.703416	0.03742
RP11-876	-0.019796067	-0.054446877	-0.07468	0.05068	-0.20118	0.047835
RP11-884	-0.00351709	0.129818948	0.074727	0.026927	-0.04449	-0.10312
RP11-893	-0.045302892	-0.092142181	0.066656	-0.00061	-0.02749	-0.06568
RP11-90C	-0.030871767	0.036210932	0.13198	-0.02983	0.104173	-0.11964
RP11-90P	-0.030078641	-0.035745982	0.041685	0.026943	-0.15929	-0.18111
RP11-91I	-0.03962678	0.032197201	-0.10679	0.006474	0.009128	0.011969
RP11-930	-0.027794523	-0.080671956	-0.01338	0.009054	-0.01345	0.060476
RP11-932	-0.012849633	-0.050528837	0.008828	0.072753	-0.23897	-0.05514
RP11-932	-0.041094119	-0.096375135	-0.18691	0.03566	-0.09703	-0.01422
RP11-945	-0.049154928	-0.10643255	-0.14112	0.003959	0.007142	0.016231
RP11-953	-0.027311543	-0.009258279	-0.00803	0.022431	-0.08033	-0.27451
RP11-95D	-0.025735438	0.016993212	0.154158	0.003476	0.002792	0.000808
RP11-95H	-0.027412455	-0.024345184	0.10421	0.002822	0.009705	-0.09258
RP11-950	-0.0438832	0.005306133	0.006591	-0.03291	0.100471	-0.12347
RP11-960	-0.03524746	-0.05533068	-0.08094	-0.00191	0.02623	-0.08529
RP11-96D	-0.020694847	0.026815624	0.098048	0.016229	-0.03784	-0.0082
RP11-96H	-0.119724884	-0.107580703	0.079261	-0.04145	0.114867	-0.07635
RP11-96K	-0.019736958	-0.029021778	0.05591	0.00649	-0.05239	-0.09772
RP11-97C	0.008338393	0.010896726	0.043218	0.006154	-0.0122	-0.1259
RP11-981	-0.041151132	-0.102101641	0.05807	-0.04688	0.12519	0.0168
RP11-998	-0.027612313	-0.044019324	0.045473	-0.00203	0.035552	0.029676
RP11-999	-0.061544409	-0.054950749	-0.00773	-0.02681	0.081867	-0.06159
RP13-103	-0.024364536	-0.073892226	-0.0188	-0.01546	0.017198	0.041162
RP13-122	-0.042628561	0.029913267	0.037408	-0.01146	0.075333	0.074504
RP13-20L	-0.036249122	-0.038852897	0.185053	0.035693	-0.08832	-0.11541
RP13-238	-0.056312746	-0.022023916	-0.05121	-0.00987	0.038023	-0.06336
RP13-270	0.00444694	-0.018016396	0.106178	-0.04743	0.15675	0.061004
RP13-30A	-0.035610748	-0.085527113	-0.14891	0.018995	-0.03548	0.098761
RP13-379	-0.022544658	-0.018676777	-0.04258	0.004151	-0.03078	-0.09436
RP13-39P	-0.03294191	0.197985818	-0.00075	-0.04958	0.16436	-0.01453
RP13-436	-0.037577628	-0.036268815	-0.2207	0.034315	-0.11418	-0.06983
RP13-638	-0.047729185	-0.022639964	-0.05976	0.005278	0.017801	0.001672
RP1L1	-0.030245646	-0.022629578	0.0153	0.027225	-0.07624	-0.17532
RP2	0.001231434	-0.034674153	-0.45872	-0.0263	0.080083	0.35594
RP3-327A	-0.006684706	0.08825578	0.16841	-0.01385	0.071857	-0.07436
RP3-329A	-0.021478565	-0.08896932	0.037845	-0.00954	-0.06157	-0.10098
RP3-333B	-0.031067505	-0.019395769	-0.00223	0.0045	-0.02128	-0.03727
RP3-334F	0.021132234	-0.077461911	0.30562	0.025319	-0.12416	-0.29379
RP3-334F	-0.031308352	-0.038466531	0.068898	0.026076	-0.04602	-0.07931
RP3-336K	-0.042933271	-0.056228769	0.034571	-0.00658	0.024689	-0.0397
RP3-337H	-0.02678247	-0.045799656	-0.0882	0.024304	-0.06322	-0.01614
RP3-359N	-0.045133478	-0.102682513	0.002121	0.020915	-0.024	0.084385
RP3-368A	-0.002860844	-0.017162668	-0.07116	-0.00516	0.031947	0.033619
RP3-384D	-0.026679629	-0.06189702	0.077698	0.060591	-0.17844	-0.01046
RP3-388M	-0.027394302	0.024257094	-0.00824	0.011193	-0.02737	-0.10185
RP3-400B	-0.060751652	-0.026816976	-0.15216	-0.02082	0.044461	0.147778
RP3-406A	-0.038230508	-0.01160789	0.011403	-0.00721	0.034387	-0.08556

RP3-406C:	-0.045081275	0.000770066	-0.02529	0.016302	-0.02039	0.101942
RP3-406P:	0.01389418	-0.012897654	-0.03347	0.046328	-0.17541	0.065681
RP3-412A!	-0.030962082	-0.070122625	-0.11579	0.027754	-0.0863	0.21069
RP3-428L:	-0.008576015	0.057725875	0.056584	0.077131	-0.18951	-0.06036
RP3-452M:	-0.066088886	-0.061408605	-0.04958	0.005122	-0.02517	0.172449
RP3-476K!	-0.027986225	-0.046550591	-0.09326	0.002615	-0.01088	0.064322
RP3-486D:	0.055833726	0.059213214	0.269346	0.045001	-0.09919	-0.09629
RP3-496C:	-0.070043308	-0.023005928	-0.00673	-0.01635	0.052913	0.012121
RP3-497J:	-0.042897985	-0.101116633	-0.02277	-0.0357	0.133699	0.136734
RP3-507I:	0.041798751	0.0051345	0.255525	0.039764	-0.09414	0.046652
RP3-507I:	-0.100429609	-0.063269902	-0.0484	-0.04473	0.094545	0.125069
RP3-508D:	-0.035141547	-0.033985632	0.043198	-0.00109	0.019444	-0.05545
RP3-508I:	-0.004311999	-0.096006155	-0.15859	-0.00389	-0.02727	-0.10388
RP3-522P:	-0.023559352	-0.055977722	0.236855	0.003706	0.010026	-0.09952
RP3-522P:	0.004839638	0.00509877	-0.06794	0.032332	-0.17629	-0.07732
RP3-525N:	-0.009027268	-0.096454405	-0.17942	-0.05505	0.175695	0.643566
RP4-539M(	-0.039057528	-0.080838744	0.014306	-0.00685	0.046537	-0.12582
RP4-539M(	-0.028266608	-0.040294808	-0.02286	0.004176	0.005484	0.003323
RP4-545K:	-0.020169248	-0.028750825	-0.00221	-0.01379	0.021827	-0.07323
RP4-555D:	-0.047252614	0.019833201	-0.05375	0.015062	-0.01932	0.085971
RP4-560B!	-0.032144056	0.003483008	-0.07285	0.033071	-0.06809	0.219099
RP4-561L:	-0.037681028	0.021627909	0.004619	-0.02192	0.063773	-0.08247
RP4-575N(	-0.010749902	0.02757059	0.31064	-0.02274	0.07053	-0.30755
RP4-581F:	-0.011246825	0.082313712	0.004755	0.063034	-0.16217	0.073628
RP4-584D:	-0.040671452	-0.038567006	0.088814	0.009158	-0.03401	0.082375
RP4-593C:	-0.064249726	-0.041077311	0.09657	-0.03042	0.092727	-0.01292
RP4-593H:	-0.031159406	-0.051856136	0.003168	0.020118	-0.04207	-0.06157
RP4-594L!	-0.041699447	-0.025209309	-0.03356	0.045172	-0.09743	0.014087
RP4-595K:	0.038924864	0.011997222	0.24653	0.028918	-0.06683	0.022389
RP4-612B:	-0.013426999	0.115295459	-0.01077	-0.04891	0.154014	-0.06692
RP4-612B:	-0.03419712	-0.079602895	0.151131	0.020657	-0.02928	0.000557
RP4-613A:	-0.030492548	-0.007564223	0.043224	-0.00904	0.025953	-0.16626
RP4-614O	-0.015528685	-0.012658982	0.010536	0.002337	3.34E-05	-0.03247
RP4-621B:	-0.086579804	-0.030700659	-0.0328	-0.01694	0.086989	-0.13973
RP4-633H:	-0.045123979	-0.062525916	-0.04976	-0.00522	0.022272	0.160864
RP4-635E:	0.028044634	-0.074734347	0.154791	-0.06629	0.218884	-0.20356
RP4-647C:	-0.016798735	-0.167149452	-0.19139	-0.03489	0.063211	0.225153
RP4-657D:	-0.009925563	-0.02084428	-0.00475	0.045456	-0.11264	0.064494
RP4-665N	-0.034083534	-0.064755583	-0.02191	-0.01918	0.023062	-0.1579
RP4-675G!	-0.058292593	-0.043382053	0.074767	0.037291	-0.07938	-0.00311
RP4-676L:	-0.024727014	-0.018633592	0.003865	-0.01399	0.006683	0.022371
RP4-680D!	-0.019129466	-0.102896713	0.380277	-0.04092	0.136166	-0.17802
RP4-710M:	0.020590729	-0.106981977	0.235935	0.015193	-0.12987	-0.39167
RP4-714D!	-0.016641552	0.038585922	0.103821	-0.01228	0.047897	-0.17712
RP4-730D	-0.027634908	-0.04762573	0.118153	-0.00188	0.009213	0.001749
RP4-740C	-0.017517308	-0.052605524	0.00599	0.001336	0.006887	-0.20167
RP4-742J:	-0.049004972	-0.045195268	-0.06635	-0.01642	0.067141	0.071103
RP4-758J:	-0.003058103	-0.004273821	-0.03944	-0.00481	0.073309	0.117855
RP4-773N:	-0.009739996	0.048636098	0.12035	-0.03144	0.116685	-0.11189
RP4-781K!	-0.019942016	-0.09647408	0.014467	-0.00227	-0.06782	0.078244
RP4-781L:	0.032001707	0.136179472	0.102168	-0.02252	0.071259	-0.15055

RP4-794H	-0.023839247	-0.010673093	-0.03694	-0.01174	0.00231	0.103396
RP4-798A	-0.025505336	0.044098287	0.072077	0.022721	-0.08559	-0.27505
RP4-813F	-0.015228354	0.079598487	0.198736	-0.00896	0.036834	-0.2032
RP5-1007I	-0.030526359	-0.082929066	0.086077	-0.02864	0.046221	-0.02477
RP5-1027(	-0.048535789	-0.018183255	-0.02486	-0.00828	0.045208	-0.02616
RP5-1031I	-0.026192592	-0.099348118	-0.05863	0.012128	-0.02755	-0.03143
RP5-1039I	-0.03432506	-0.031681937	-0.08651	0.011903	-0.08548	-0.06087
RP5-1039I	-0.025746455	-0.020947785	0.067982	0.018722	-0.03883	-0.04718
RP5-1061I	-0.02637568	-0.061884099	-0.03542	-0.00166	0.010383	0.039496
RP5-1065	-0.022568286	-0.060317186	0.091498	-0.00424	-0.00398	-0.03831
RP5-1068I	-0.017000839	0.080102203	0.157453	0.007925	0.008539	-0.09559
RP5-1074I	-0.003273929	0.034008091	0.032122	0.038473	-0.09354	-0.05366
RP5-1085I	0.014935657	-0.045471123	0.064398	0.013706	-0.05172	-0.29981
RP5-1092/	0.001801308	0.080494893	0.127425	-0.0294	0.107821	-0.23358
RP5-1098I	-0.02565733	-0.081988554	-0.05811	0.022235	-0.04101	-0.03341
RP5-1102I	-0.039458345	-0.057697649	0.110176	-0.00955	0.050478	0.006378
RP5-1103(	-0.059710806	-0.007190497	-0.07621	-0.00256	-0.00064	0.022268
RP5-1118I	-0.018755162	-0.071066429	0.20948	-0.01111	0.058049	-0.13856
RP5-1119/	-0.028530282	-0.069791825	-0.22512	0.001659	0.005069	0.063992
RP5-1136(	-0.003853284	0.023532894	0.004915	0.063712	-0.16522	0.115869
RP5-1154I	-0.029230817	-0.032815532	-0.04279	-0.00998	0.03287	-0.04372
RP5-1170I	-0.03662	-0.042243195	-0.05471	0.039148	-0.1035	-0.14386
RP5-1180I	-0.024184653	0.116336223	0.304999	-0.04461	0.120346	-0.17396
RP5-1184I	-0.034470049	-0.077001981	-0.15688	0.003629	-0.03101	-0.05699
RP5-1189I	-0.061376439	-0.010339386	0.024777	-0.01353	-0.00365	0.032545
RP5-855D	-0.030886986	0.017729316	-0.07691	0.001184	0.000752	-0.08217
RP5-856G	-0.036458753	-0.045239928	-0.02963	0.026415	-0.05031	-0.12688
RP5-882O	0.047482373	0.05158604	0.069949	0.077497	-0.07278	0.106086
RP5-890E	-0.03058554	0.007709127	0.055591	0.006179	-0.02755	-0.09764
RP5-892K	-0.040502336	-0.073708258	-0.0445	0.01541	-0.02407	0.050071
RP5-894A	-0.012569984	-0.000217508	0.03848	0.003701	-0.02004	-0.0048
RP5-894D	-0.031902595	0.012663292	-0.03234	0.050351	-0.13903	0.033868
RP5-930J	0.042864926	0.010611066	0.036614	-0.01502	0.004433	-0.04977
RP5-935K	0.017346777	0.061367047	-0.0051	0.067655	-0.17249	0.140702
RP5-944M	-0.033020855	-0.029717813	0.007973	0.025766	-0.05581	-0.11479
RP5-963E	-0.030927575	-0.054304713	0.01218	0.014721	-0.08223	0.082149
RP5-968J	-0.02278769	-0.106257155	0.047313	0.008416	-0.03429	0.079632
RP5-991G	-0.025098647	-0.019681119	-0.03099	0.073117	-0.21851	-0.07172
RP5-991O	-0.04572854	-0.042803226	0.048811	0.003993	0.015358	0.060521
RP6-149D	-0.09437766	0.001884467	0.031326	-0.04501	0.122962	0.022371
RP6-201G	-0.030120896	-0.050117004	-0.07152	0.015251	-0.02629	-0.0218
RP6-24A2	-0.038351241	-0.045793357	-0.02908	0.005625	0.009771	-0.11257
RP6-91H8.	-0.048118492	-0.059776482	-0.03264	-0.02337	0.096835	0.126234
RP6-91H8.	-0.03290202	-0.055513943	-0.00328	0.024005	-0.07343	0.044781
RP6-99M1.	-0.027204884	-0.004654841	-0.07547	0.013695	-0.11982	-0.04172
RP9	0.014515179	-0.01078726	-0.03398	0.008168	-0.0392	-0.11278
RP9P	0.022014619	-0.068963245	-0.08223	0.001782	-0.07313	-0.18883
RPA1	0.022634676	0.091182959	0.148785	0.009208	-0.02178	-0.20532
RPA2	0.048446167	0.192842263	0.173307	0.043452	-0.13119	-0.4157
RPA3	0.027327781	0.037990749	0.204794	0.095635	-0.27515	-0.18678
RPA30S	-0.000165499	0.004861198	-0.00676	0.002656	0.019842	-0.01125

RPA4	-0.022905671	-0.110222916	-0.14289	-0.00021	0.027188	0.388441
RPAIN	0.015964467	-0.075548704	0.033924	-0.04654	-0.14377	-0.02141
RPAP1	0.019749665	-0.018265267	0.254448	0.045987	-0.15306	-0.18526
RPAP2	0.013923541	0.101119219	0.199628	0.050872	-0.13141	-0.18254
RPAP3	0.008942393	0.002885456	-0.2051	0.037879	-0.12351	-0.07755
RPARP-AS:	-0.004325921	0.039043762	0.232772	-0.00302	0.039718	-0.29738
RPE	0.02036273	0.110670803	-0.13611	0.053721	-0.13543	0.203146
RPE65	-0.08642243	-0.06954595	-0.02498	-0.01418	0.056776	0.054071
RPF1	-0.070867269	-0.033190845	0.160336	-0.03777	0.032727	0.007297
RPF2	0.024983992	0.036932149	0.149822	0.063308	-0.17705	0.060172
RPGR	0.004174323	-0.068892915	-0.21196	0.02208	-0.02243	0.033178
RPGRIP1	-0.011221133	-0.106793156	-0.21788	-0.05207	0.129027	0.329448
RPGRIP1L	-0.046821433	-0.001794635	0.189364	0.047588	-0.10117	-0.10739
RPH3A	-0.003494739	-0.063407664	-0.19155	-0.10605	0.305208	0.666486
RPH3AL	-0.026137764	-0.012125912	-0.12641	0.040347	0.103678	-0.00977
RPIA	0.048514888	0.055075192	0.186886	0.025017	-0.08519	-0.06835
RPL10A	0.028374329	0.062497475	0.176317	0.027774	-0.03087	0.060895
RPL10L	-0.007884577	-0.074541508	0.295981	0.001979	-0.00127	-0.02506
RPL11	0.055325378	0.04828069	0.144186	0.050876	-0.10577	0.083554
RPL12	0.053648402	0.048316039	0.152769	0.058172	-0.0815	0.057426
RPL13AP1'	-0.010677431	-0.051436167	0.099628	-0.02552	-0.04449	-0.20661
RPL14	0.045116043	0.05712222	0.234754	0.049002	-0.10473	0.005814
RPL15	0.03593524	0.011150797	0.222784	0.009117	-0.01721	0.079598
RPL18	0.039249206	0.034980207	0.208533	0.025101	-0.07807	0.061864
RPL19	0.043642838	0.053554763	0.113929	0.049392	-0.02317	0.065159
RPL22	0.041865039	0.050633672	0.154607	0.033063	-0.06295	0.116954
RPL22L1	0.034395874	0.01004152	0.111486	0.065102	-0.18177	0.073841
RPL23A	0.022289654	0.06711101	0.15633	0.052886	-0.0509	0.035611
RPL23AP3:	-0.009960571	-0.060089894	0.166379	0.025612	-0.03844	-0.06204
RPL23AP5:	-0.032609498	-0.05807131	-0.07053	-0.02436	0.034366	0.024882
RPL24	0.047710075	0.073107652	0.148472	0.056857	-0.10627	0.080209
RPL26L1	0.051635348	-0.018976141	0.028119	0.128549	-0.36248	0.316714
RPL27	0.046442339	0.082010112	0.080515	0.07033	-0.07238	0.085532
RPL27A	0.03680335	-0.013922566	0.051026	-0.02008	0.048556	-0.12778
RPL29	0.046441255	0.056550641	0.178531	0.045635	-0.09754	0.102013
RPL3	0.04076662	0.097171359	0.186404	0.039321	-0.05024	-0.11541
RPL30	0.035742353	0.052742183	0.142436	0.061601	-0.01537	0.026274
RPL31	0.020201395	-0.047329914	0.264072	-0.02937	0.057164	0.094202
RPL32	0.037233954	0.042960802	0.145618	0.054678	-0.05186	0.060571
RPL32P3	-0.075498361	0.001185703	-0.03815	0.01064	0.069296	-0.04237
RPL34	0.055585341	0.06005934	0.117189	0.069124	-0.037	0.002802
RPL34-AS:	-0.04526914	-0.091894416	-0.09839	-0.01344	0.037468	-0.01937
RPL35	0.04091917	0.073098189	0.181672	0.050299	-0.10925	0.032042
RPL35A	0.013602278	-0.086674955	0.234206	-0.0384	-0.02414	-0.16652
RPL36	0.03311622	0.009481573	0.277591	0.044979	-0.09873	-0.00441
RPL36A	-0.008061863	-0.048371033	-0.02784	-0.08767	0.252163	-0.03949
RPL36AL	0.094646516	0.12850968	0.250637	0.085932	-0.18902	-0.12555
RPL37	0.037188883	0.070755569	0.05657	0.086857	-0.07532	0.084632
RPL37A	0.059091261	0.017385651	0.121386	-0.00974	0.027344	-0.06315
RPL38	0.049180064	0.030894571	0.101766	-0.01601	-0.0002	-0.16425
RPL39	0.040300208	0.053624427	0.057081	0.090663	-0.07137	0.067078

RPL39L	-0.01222032	0.112403472	0.034057	0.175681	-0.38537	-0.0592
RPL3L	-0.035382164	-0.002948231	-0.10593	0.026716	-0.04666	0.162601
RPL41	0.035331367	0.122016607	0.106408	0.143119	-0.1241	-0.02985
RPL6	0.048605905	0.082187332	0.125084	0.043283	-0.04962	0.088369
RPL7	0.05508258	0.096579237	0.095559	0.071281	-0.08571	0.056243
RPL7AL2	0.013048924	-0.036292269	0.218798	-0.00145	-0.06434	-0.15365
RPL7L1	0.056087733	0.09732584	0.138329	0.058266	-0.15416	-0.01808
RPL8	0.052861576	0.046228585	0.134115	0.05949	-0.10738	0.098377
RPL9	0.045687071	0.065191936	0.110514	0.059801	-0.04125	0.038625
RPLP0	0.029664592	0.044188295	0.141847	0.049462	-0.07586	0.083235
RPLP1	0.027350594	0.055529367	0.066623	0.064054	-0.06266	0.148982
RPLP2	0.007914209	-0.031715283	0.019428	0.020638	-0.05144	0.013244
RPN1	0.055733573	0.08698457	-0.2126	0.100982	-0.26807	0.134217
RPN2	0.062146062	0.102609594	0.041196	0.059377	-0.12768	0.048302
RPP14	0.0434592	0.00589504	0.098464	0.049056	-0.0976	0.080802
RPP25	-0.004358391	-0.093792836	-0.05332	-0.03938	0.098423	0.078216
RPP25L	0.05234243	0.008686331	0.08685	0.057902	-0.17585	0.066951
RPP30	-0.012479122	0.005732308	0.135683	-0.00533	0.075593	-0.10161
RPP38	0.031127777	0.040243343	0.100753	0.004195	-0.02499	0.10408
RPP40	0.002846878	-0.025804164	0.053613	0.03718	-0.13583	0.091121
RPPH1	-0.013358221	-0.078973449	-0.11468	-0.00251	-0.02186	0.023189
RPRD1A	0.019776523	-0.027323266	-0.09264	0.025005	-0.1013	0.123946
RPRD1B	-0.000321711	0.008095219	-0.12284	0.047144	-0.18333	0.026348
RPRD2	0.015247747	0.060420954	0.165127	0.017742	-0.02686	-0.06709
RPRM	-0.023970513	-0.004752491	0.029784	-0.01594	-0.00077	-0.05087
RPRML	-0.027413787	-0.023829507	-0.09326	0.0147	-0.01301	-0.01778
RPS10	0.058885665	0.072466146	0.126998	0.069298	-0.08407	0.011164
RPS10L	0.039874391	-0.005050133	0.30697	0.021834	-0.05205	-0.03845
RPS10P7	-0.02544059	-0.103065524	0.200565	0.048944	-0.11004	-0.05128
RPS11	0.037497415	-0.025825535	-0.02708	-0.04043	0.108159	-0.18713
RPS12	0.02363198	0.05692471	0.181541	0.035032	-0.0432	0.061553
RPS14	-0.03067807	-0.066658492	0.170937	-0.02138	0.036835	-0.07985
RPS15	0.033810847	0.039393752	0.133584	0.048722	-0.06463	0.070383
RPS15A	-0.003158722	-0.088225418	0.238909	-0.05289	0.078803	-0.03524
RPS16	0.04448547	0.063990981	0.169748	0.042878	-0.05706	0.082994
RPS16P5	0.0078706	-0.097701354	-0.10266	0.004007	-0.02505	-0.29778
RPS17	0.044416088	0.067902844	0.131605	0.052912	-0.07392	0.059447
RPS18	0.014415496	0.048302978	0.174597	0.042491	-0.04967	0.085728
RPS19	0.035371392	0.083532606	0.165833	0.060194	-0.10345	0.054155
RPS19BP1	0.067719459	0.081793844	0.066741	0.043906	-0.13814	0.088783
RPS21	0.041717723	0.007043595	0.208237	0.059843	-0.12962	-0.01299
RPS23	0.019116428	0.008163104	0.226322	-0.00285	0.068449	0.067339
RPS24	0.022831352	0.041217101	0.070717	0.055048	-0.08269	0.083664
RPS25	0.064611311	0.070155465	0.188681	0.069622	-0.11333	-0.0318
RPS27	0.021093566	0.006715246	0.020842	0.051831	-0.02624	0.026376
RPS27A	0.037969154	-0.002371348	0.116167	-0.02286	0.050554	-0.09952
RPS27L	0.020908846	0.036292311	-0.01164	0.079778	-0.22691	0.230256
RPS28	0.015142035	-0.109315362	0.312036	-0.03018	0.039093	-0.16429
RPS29	0.033426026	0.059493269	0.168634	0.048812	-0.01917	0.019492
RPS3	0.054973858	0.116674397	0.186849	0.070467	-0.09148	-0.06821
RPS4X	0.03526941	0.070411364	0.120749	0.042057	-0.05	0.074484

RPS4Y1	0.014412874	0.012948757	0.158641	-0.08191	0.266791	0.002069
RPS5	0.041375376	0.054777257	0.203369	0.05042	-0.10203	-0.02468
RPS6	0.042966411	0.07485648	0.155955	0.059462	-0.06264	0.010438
RPS6KA1	0.043550944	0.077218734	-0.25629	0.004737	-0.00151	0.03883
RPS6KA2	-0.046399343	-0.095470979	-0.15879	-0.04148	0.169169	-0.09371
RPS6KA2-/	-0.026840716	-0.045105253	-0.20009	-0.01863	0.048876	0.047115
RPS6KA3	0.065344851	0.062379144	0.14414	0.001997	0.013102	-0.25382
RPS6KA4	0.011228167	-0.112024437	-0.13505	-0.00842	0.015112	0.198135
RPS6KA5	0.009641796	0.006693848	-0.08058	0.021446	-0.08973	-0.10812
RPS6KA6	-0.060939661	-0.10376953	-0.02743	0.000779	0.002258	-0.09694
RPS6KB1	0.037468421	0.065361162	0.052415	0.005976	-0.12147	0.012801
RPS6KB2	0.028847804	-0.002078848	-0.0157	0.011069	-0.10976	0.067313
RPS6KC1	0.025745402	0.062423219	-0.13987	-0.00797	0.034011	0.369516
RPS6KL1	-0.01924009	-0.022589243	0.082308	0.012695	-0.02278	-0.17313
RPS7	0.057285976	0.080903442	0.147189	0.050535	-0.07876	0.023585
RPS8	-0.030134097	-0.093170101	0.04456	0.028977	-0.05524	0.075496
RPS9	0.059603632	0.008475961	0.12157	0.078041	-0.1253	0.10091
RPTN	-0.053539348	-0.039730375	0.027121	-0.01883	0.067023	0.032623
RPTOR	-0.00348216	-0.064134542	0.110884	-0.00288	-0.04368	-0.0795
RPUSD1	0.003117757	-0.110536897	0.106415	0.019749	-0.14744	-0.0303
RPUSD2	0.003494164	0.055028785	0.234434	0.057438	-0.1582	-0.3278
RPUSD3	0.015804133	0.133493306	0.151267	-0.03728	-0.03852	-0.10916
RPUSD4	0.035482882	-0.010275608	0.200725	0.052238	-0.15433	-0.02647
RQCD1	0.006532141	0.023432709	0.063707	0.100929	-0.28455	-0.07949
RRAD	-0.051169976	-0.047040594	-0.08684	0.010697	-0.03703	0.02783
RRAGA	0.082584022	0.143568111	-0.1409	0.043133	-0.10525	-0.11065
RRAGB	-0.016371343	-0.00885851	0.033449	-0.01147	0.062139	0.036212
RRAGC	0.031499183	0.006137383	-0.19888	0.005817	-0.00794	-0.02476
RRAGD	0.009266789	-0.193474399	-0.20821	-0.08972	0.245495	0.325416
RRAS	-0.001541303	-0.063721741	-0.09943	-0.03723	0.118532	0.60493
RRAS2	0.008499411	0.199592376	0.152335	0.062548	-0.2089	-0.35083
RRBP1	-0.023699459	0.033229306	-0.18226	0.038162	-0.02213	-0.02787
RREB1	-0.015767936	-0.115919641	-0.04202	-0.07464	0.350016	0.131285
RRH	-0.036549192	-0.026034948	-0.04898	0.00181	0.022104	-0.0313
RRM1	0.016484649	0.068757277	0.227063	0.120093	-0.33842	-0.19348
RRM2	-0.00051315	0.128520594	0.083526	0.086533	-0.2534	-0.16415
RRM2B	0.023024613	-0.02844352	-0.38368	0.092515	-0.23524	-0.00041
RRN3	0.020840229	0.081293596	0.181171	0.029419	-0.12354	-0.23488
RRN3P1	-0.001934056	0.012684522	0.386953	-0.06566	0.178976	-0.2233
RRN3P2	0.006550596	0.033774263	-0.15329	-0.01184	0.04712	0.185098
RRN3P3	-0.027221476	0.012118031	-0.05302	-0.00138	0.014763	0.097084
RRNAD1	0.012959517	-0.053189116	-0.04913	0.072226	-0.19276	0.181995
RRP1	-0.001442431	-0.05534774	-0.02696	0.03075	-0.18765	-0.14987
RRP12	-0.02624175	-0.083971268	-0.00084	-0.02206	0.003184	-0.06
RRP15	0.002887493	0.029523423	0.165099	0.031142	-0.09438	-0.15139
RRP1B	0.025317715	0.051693797	0.359751	0.050469	-0.13886	-0.14418
RRP36	0.041430042	0.126507616	0.117956	0.089158	-0.24951	-0.04057
RRP7A	0.008676346	-0.048069413	-0.0388	0.065422	-0.16919	0.185972
RRP8	-0.052831397	-0.058553268	0.053727	-0.01699	0.059293	-0.0486
RRP9	-0.004494869	-0.031413918	-0.01722	0.056565	-0.20452	-0.01707
RRS1	0.030875643	-0.006459761	0.241371	0.038385	-0.10824	0.064431

RRS1-AS1	-0.035716867	-0.054956276	0.040867	-0.00666	0.020705	-0.11888
RS1	-0.047621885	-0.03094582	-0.05335	-0.00539	0.038884	0.009287
RSAD1	0.031780182	0.053038129	0.266107	0.007007	-0.00979	-0.10078
RSAD2	0.007306941	0.043667479	-0.0918	-0.0351	0.085778	0.064678
RSBN1	0.030854982	0.068531099	0.293879	-0.02989	0.073465	-0.36605
RSBN1L	0.029085279	0.136806724	-0.03935	-0.00977	-0.00711	-0.24264
RSF1	0.012194855	0.147610011	0.01939	0.053382	-0.0868	-0.21897
RSG1	-0.021027426	-0.020193111	-0.00337	0.055114	-0.12283	-0.0474
RSL1D1	0.038691165	0.033848885	0.150034	0.027981	0.020828	0.219268
RSL24D1	0.059280515	0.052538079	0.237572	0.050234	-0.15018	0.003609
RSPH1	-0.014117639	-0.088741646	-0.0296	-0.01344	0.056155	-0.14592
RSPH3	0.00199542	-0.065202455	-0.2716	-0.03487	0.114037	0.338653
RSPH4A	-0.067712287	-0.021487236	-0.0274	-0.06335	0.130779	0.030805
RSPH6A	-0.030160512	-0.131357556	-0.11952	-0.01132	-0.02241	0.031569
RSPH9	-0.038533742	-0.054616797	-0.15619	-0.07497	0.254772	0.049816
RSPO1	-0.02923874	-0.072682013	-0.10557	0.020133	-0.06061	-0.04075
RSPO2	-0.087402852	-0.038140967	-0.10801	-0.04342	0.105616	0.234241
RSPO3	-0.036289446	-0.050574034	-0.05824	0.01598	-0.02569	0.058476
RSPO4	-0.032634197	-0.000458971	-0.00274	-0.01706	0.057966	0.008384
RSPRY1	0.065588339	0.052966317	-0.10828	0.047281	-0.07923	0.138833
RSRC1	0.017984632	0.034413086	-0.07383	0.107918	-0.23526	0.182719
RSRC2	0.087151598	0.16422783	0.060317	0.051484	-0.09953	-0.27242
RSU1	-0.004978369	0.048962133	0.096441	-0.00498	0.037079	-0.02639
RSU1P2	-0.077116959	-0.072326018	-0.04926	-0.00449	0.026604	-0.04042
RTBDN	-0.02719289	-0.055265672	-0.07554	0.008258	-0.05881	-0.06236
RTCA	0.025081826	0.115577544	0.039434	0.080623	-0.21621	0.21335
RTCB	0.038149027	0.032698547	0.03577	0.043726	-0.12472	0.129516
RTDR1	-0.048505949	-0.037952995	0.010268	0.005236	-0.02833	-0.14122
RTEL1	-0.00833305	-0.035957559	0.089874	0.01221	-0.14795	-0.05078
RTF1	0.037938302	0.096557835	-0.18095	0.01829	-0.11527	-0.11936
RTFDC1	0.074917285	0.003595134	-0.31528	0.097693	-0.21448	-0.00093
RTKN	-0.029539697	0.047148726	-0.12839	0.017536	-0.01589	-0.10613
RTKN2	-0.022565478	-0.039615223	0.039848	0.02827	-0.07106	-0.08397
RTN1	0.003202316	-0.104165685	-0.27719	-0.08706	0.256703	0.589775
RTN2	-0.026577901	-0.079863923	-0.18283	-0.09821	0.212468	0.061619
RTN3	0.035829779	-0.034014801	-0.35026	0.00904	-0.0025	0.402142
RTN4	0.092474718	0.111634691	-0.30176	-0.00078	0.03968	0.296273
RTN4IP1	0.001401226	-0.020149662	-0.02087	0.062821	-0.16198	0.003474
RTN4R	-0.022048881	0.025853916	0.211137	-0.00941	0.049189	-0.06934
RTN4RL1	-0.032693918	-0.048883258	-0.02059	0.001075	0.02048	0.01316
RTN4RL2	-0.041995941	-0.048222398	-0.01944	0.001581	0.022454	0.093308
RTP1	-0.040904554	-0.055353191	0.030329	0.067795	-0.17397	0.052117
RTP3	-0.046650237	-0.085008927	0.039983	0.012863	-0.03393	-0.04783
RTP4	0.012994494	0.142362008	0.043965	-0.01571	0.054611	0.222286
RTP5	-0.038028975	-0.013113271	-0.17036	0.020231	-0.08029	-0.01481
RTTN	0.005026135	0.014831928	0.04048	0.04775	-0.1312	-0.1434
RUFY1	0.014071581	-0.039253098	-0.22024	-0.10563	0.222428	0.043252
RUFY2	-0.014967483	0.072626843	0.025061	-0.0316	0.088327	-0.02731
RUFY3	-0.016390525	-0.042199148	-0.09643	-0.10522	0.293712	0.194605
RUNDC1	0.012757221	-0.036666478	-0.27088	-0.02083	0.069026	0.10329
RUNDC3A	-0.031681657	-0.072537533	-0.08762	-0.00257	-0.03815	-0.13541

RUNDC3B	-0.018547225	0.025735206	0.107571	0.077766	-0.11338	-0.02921
RUNX1-IT	0.00851154	0.044637424	0.053579	-0.06287	0.164996	-0.18416
RUNX1T1	-0.08175762	0.047097349	-0.06182	0.002871	-0.0442	-0.08033
RUNX2	-0.066403655	-0.133459801	0.51176	-0.03389	0.030964	-0.20499
RUNX3	0.032596351	0.266427797	0.327378	0.003865	-0.0009	-0.31415
RUSC1	0.023569693	0.008211989	0.021805	-0.01028	-0.03108	0.112201
RUSC1-AS	0.000355931	0.09939185	0.018749	0.016593	-0.03795	0.009454
RUSC2	-0.014908941	-0.082933955	-0.05324	-0.04028	0.037118	0.265883
RUVBL1	0.016863075	-0.022487545	0.241893	0.060352	-0.19874	-0.12988
RUVBL2	0.030898029	0.041317503	0.021563	0.114174	-0.29767	0.098455
RWDD1	0.032983999	0.113722006	0.160272	0.023997	-0.05819	0.083858
RWDD2A	-0.008983747	-0.088256609	0.096943	0.007499	-0.00214	-0.05313
RWDD2B	0.015867013	0.02108322	0.08002	0.066832	-0.2285	-0.13891
RWDD3	0.021142867	0.037147698	0.139361	0.003546	0.006442	0.029063
RWDD4	0.042841471	0.085994031	0.076983	0.048368	-0.16411	-0.00791
RXFP1	-0.026940623	-0.030174459	-0.0334	0.192491	-0.16371	-0.0243
RXFP2	-0.051660884	-0.041330445	-0.14191	-0.0122	0.050018	0.209558
RXFP3	-0.044341874	-0.085932268	-0.03822	0.006119	0.017836	0.002416
RXRA	0.008339121	-0.034966593	-0.30971	-0.08666	0.241454	0.474376
RXRB	-0.074096091	0.085763563	0.072949	-0.05233	0.014703	-0.19203
RXRG	-0.038229551	-0.046611476	-0.06198	0.007307	0.007683	-0.11068
RYBP	0.04627338	0.043262546	-0.30668	0.022961	-0.02426	0.015675
RYK	-0.031267974	0.022751884	0.172513	-0.02653	0.007004	-0.10379
RYR1	-0.013314011	-0.042764728	-0.02969	0.007054	-0.01579	0.1376
RYR2	-0.033513021	-0.010891773	-0.02299	0.139082	-0.26528	-0.01712
RYR3	-0.029237702	-0.016477021	-0.04844	-0.01125	0.037684	-0.12083
S100A1	-0.026842615	-0.053037677	0.065675	0.018952	-0.0884	-0.0084
S100A10	0.025890088	-0.018753909	0.102939	-0.02746	0.096309	0.437303
S100A11	0.026030164	-0.01879307	-0.24165	0.015994	-0.03207	0.364334
S100A12	0.011831028	-0.114268066	-0.20536	-0.08701	0.250898	0.542086
S100A13	-0.009366005	-0.016050978	-0.07098	0.031773	-0.15334	0.12779
S100A14	-0.030375444	-0.054265519	0.059933	0.019984	-0.06182	0.022544
S100A16	-0.010031322	-0.046919093	0.049899	-0.01957	-0.0527	-0.08843
S100A2	-0.022122202	-0.033228883	-0.09759	0.043768	-0.14094	0.082327
S100A3	-0.024836743	-0.048900411	-0.05887	0.030606	-0.08592	0.031155
S100A4	0.028341507	0.061207842	0.011058	0.034429	-0.07632	0.344192
S100A5	-0.033636323	-0.070854384	-0.13899	0.057615	-0.16763	0.130493
S100A6	0.022476374	-0.056788679	-0.09282	0.005837	0.01982	0.520946
S100A7	-0.036419228	-0.011346447	-0.03523	0.076837	-0.19333	-0.02389
S100A7A	-0.033926132	-0.073139122	0.006719	0.002915	-0.02596	-0.14423
S100A8	0.009672743	-0.143212461	-0.05516	-0.1338	0.325552	0.328827
S100A9	0.012201983	-0.152608399	-0.20259	-0.09172	0.275878	0.479401
S100B	-0.010456276	0.040568232	0.374248	-0.02298	0.092587	-0.1497
S100G	-0.037277008	0.01148842	-0.07268	0.012935	-0.02041	-0.10347
S100P	0.006887754	-0.102650607	-0.15403	-0.01306	0.027995	0.181052
S100PBP	0.014931229	0.100042143	0.030044	0.043984	-0.14905	-0.23806
S100Z	-0.00405807	-0.121540546	-0.33562	-0.04669	0.141842	0.737155
S1PR1	0.009182438	0.103352012	0.360005	-0.04528	0.101294	-0.46317
S1PR2	-0.015147566	-0.017459399	0.058449	0.013837	-0.07805	-0.07089
S1PR3	-0.006714169	-0.060960275	-0.20864	-0.11793	0.345233	0.7644
S1PR4	0.031079288	-0.005848313	0.053926	-0.0086	0.021924	-0.15771

S1PR5	-0.030248601	0.377081318	0.069394	-0.08203	0.208525	-0.15456
SAA3P	-0.035027882	-0.05039988	-0.02671	0.006999	0.013512	0.137999
SAAL1	0.014607519	0.000542567	0.066722	0.110989	-0.36774	-0.13309
SAC3D1	0.022645157	0.002529405	-0.06293	0.052173	-0.12708	0.15778
SACM1L	0.071535164	0.300372341	0.048786	0.041676	-0.08072	-0.20532
SACS	0.009826702	0.072095696	0.243697	0.050588	-0.13589	-0.28821
SACS-AS1	-0.068737238	-0.041875039	-0.07447	0.008073	-0.01848	0.009437
SAE1	-0.030431301	0.061164695	0.226722	0.063857	-0.17482	-0.13041
SAFB	-0.00700944	-0.006118148	0.008116	0.043601	-0.18442	-0.0302
SAFB2	0.044457467	-0.085597216	0.110053	0.022296	-0.08446	-0.22664
SAG	-0.023382431	-0.106454705	-0.13863	-0.02034	0.04035	0.062663
SAGE1	-0.066250722	-0.046492769	-0.07186	-0.04426	0.12876	0.065198
SALL1	-0.079903335	-0.014308567	-0.11095	-0.02629	0.043237	-0.10166
SALL2	-0.005393777	-0.007288223	0.328092	-0.0533	0.101293	-0.20718
SALL3	-0.040065801	-0.042409557	-0.08634	0.021705	0.002525	-0.01878
SALL4	-0.025177848	-0.010458727	0.007276	-0.00473	-0.03837	-0.08049
SAMD1	-0.004078429	-0.080245175	0.097794	0.044815	-0.21272	0.07174
SAMD10	-0.024591922	-0.057110092	0.146994	0.061492	-0.25154	-0.15414
SAMD11	-0.026969282	-0.031247606	-0.03483	-0.01752	-0.04882	-0.07882
SAMD12	-0.035601794	-0.177046274	0.151258	-0.13828	0.422455	0.004775
SAMD13	-0.02312727	-0.065573837	-0.10162	-0.02779	0.075802	-0.09804
SAMD14	-0.043417565	-0.09250968	0.0915	-0.03234	0.02485	0.085256
SAMD15	-0.042505021	-0.035606269	-0.13316	-0.00512	0.019875	-0.10333
SAMD3	0.0032202	0.251636277	0.380677	-0.03187	0.080183	-0.25443
SAMD4A	-0.002292629	-0.046494611	-0.08709	0.007712	-0.01872	0.412246
SAMD4B	-0.038992087	-0.068158066	0.060197	-0.0704	-0.1103	-0.13626
SAMD5	-0.041960275	-0.013564545	0.092049	0.063379	0.065771	-0.15193
SAMD8	0.00285497	-0.031026402	-0.23836	0.004122	-0.00445	0.086167
SAMD9	0.016881335	0.127472972	-0.08484	-0.01127	0.035751	-0.11798
SAMD9L	0.002132173	0.089849278	-0.10469	-0.03272	0.045866	-0.04731
SAMHD1	0.022647462	0.042283295	0.071968	-0.02535	0.081917	0.285616
SAMM50	0.003011979	0.049906856	0.059515	0.07553	-0.1593	0.083052
SAMSN1	0.024691449	0.013228268	-0.04745	0.002953	-0.04129	0.092922
SAP130	0.030911038	0.016173465	-0.10315	0.051865	-0.14621	-0.09082
SAP18	0.05238606	0.100970513	0.114988	-0.05993	0.130424	-0.19303
SAP30	-0.014815669	-0.082340061	-0.1277	0.094677	-0.22872	0.508713
SAP30BP	0.047283713	0.045606157	-0.14283	-0.01078	-0.03042	-0.07387
SAP30L	-0.005265054	-0.008685138	-0.04007	0.001366	0.102108	-0.09581
SAPCD2	-0.001140586	-0.055299028	0.025266	0.045233	-0.21628	-0.0079
SAR1A	0.036543985	0.119055583	0.001543	0.049217	-0.16726	0.136068
SAR1B	0.081987296	0.165822148	-0.12953	-0.02656	-0.1619	0.231362
SARAF	0.067752359	-0.013618166	0.177765	-0.03998	0.193788	-0.40262
SARDH	-0.038753106	-0.107238207	0.003936	-0.01788	0.03878	0.076864
SARM1	0.039403083	0.097071434	0.211871	0.034692	-0.03215	-0.10769
SARNP	0.051516881	0.058872094	-0.05087	0.079552	-0.25478	0.022679
SARS	0.032590332	0.096190593	0.08009	0.087079	-0.23614	0.043973
SARS2	0.005470567	0.016481712	0.025387	0.050719	-0.12604	0.062264
SART1	0.024070275	-0.042017685	-0.13343	0.067834	-0.20726	0.062334
SART3	0.074949826	0.125155802	0.132598	0.060869	-0.12647	-0.27637
SASH1	-0.008967475	-0.057652986	-0.08836	-0.02762	0.050823	0.483845
SASH3	0.042146078	0.165638626	-0.06613	0.008215	-0.00609	-0.04277

SASS6	-0.004090805	0.067288526	0.004221	0.093077	-0.25402	-0.06465
SAT1	0.038288777	-0.10821396	-0.38184	-0.05012	0.186399	0.328387
SAT2	0.031229422	-0.014260556	-0.00338	-0.04588	0.122848	0.447499
SATB1	-0.018180116	0.031161227	0.420372	0.074154	-0.03003	-0.33534
SATB2	-0.037048026	-0.025896754	0.083448	-0.01905	0.039415	0.011333
SATB2-AS1	-0.058143553	-0.074430709	-0.03263	0.023651	-0.04701	-0.01314
SAV1	-0.010743565	-0.079769505	-0.08433	-0.03028	0.086678	-0.11257
SAYSD1	-0.002616502	-0.003641509	0.211811	-0.01834	0.030779	-0.121
SBF1	-0.018492611	0.136502077	0.134017	0.018582	-0.07382	-0.10816
SBF2	-0.048122669	-0.071661115	-0.15424	-0.05134	0.152696	0.356892
SBF2-AS1	-0.029535772	-0.023099421	0.007226	0.023291	-0.06245	-0.17874
SBK1	0.003111884	0.254604672	0.372356	-0.04698	0.135604	-0.27212
SBNO1	0.033979399	0.099917031	-0.13535	0.084054	-0.22695	-0.01902
SBNO2	-0.011508646	-0.121966669	-0.12898	-0.00332	-0.03758	0.090018
SBSN	-0.036871742	-0.124958114	0.08649	-0.00844	0.054093	0.099816
SBSPON	-0.022467029	-0.068429253	-0.00789	0.052865	-0.03059	-0.08731
SC5D	0.009350283	0.010605946	0.110168	0.013401	-0.02974	-0.0829
SCAF1	-0.02704624	-0.084528499	-0.07504	0.074993	-0.20793	0.177842
SCAF11	0.022238331	0.072735592	-0.12399	0.027365	0.083256	-0.30276
SCAF4	-0.021952949	0.008033096	-0.05305	-0.0623	0.08846	-0.04545
SCAF8	0.079772113	0.1630067	0.133924	0.038911	-0.09933	-0.41988
SCAI	0.03058094	0.064561033	0.057939	0.04047	-0.05432	-0.13543
SCAMP1	-0.01822088	0.086149346	-0.1472	-0.02552	0.059336	0.189819
SCAMP1-AS	0.028032788	0.04627178	0.222113	-0.00714	0.02354	-0.05412
SCAMP2	0.080095725	0.016536101	0.061753	-0.02126	0.030699	0.130365
SCAMP3	0.052745143	0.044412659	0.192956	0.109086	-0.32482	-0.10003
SCAMP4	-0.018352273	-0.119846257	-0.03067	0.019836	-0.04605	0.062252
SCAMP5	-0.009520634	-0.08955639	-0.04468	-0.00619	-0.04283	-0.21003
SCAMPER	-0.034092944	-0.047480298	0.010671	0.032377	-0.07699	-0.00166
SCAND1	0.050603372	-0.013237549	0.03277	0.057455	-0.12714	0.190077
SCAND2P	-0.091993107	-0.014991542	0.059761	-0.14072	0.154303	-0.04939
SCAP	0.056813775	-0.029614924	0.01063	0.033251	-0.09583	-0.18063
SCAPER	-0.023662204	0.082484395	0.172034	-0.02645	0.02789	-0.03457
SCARA3	-0.035881153	-0.046896641	-0.08627	0.005145	0.016343	-0.03911
SCARA5	-0.035987735	-0.070196183	-0.098	-0.02452	-0.03843	-0.09032
SCARB1	-0.048120638	-0.099264407	-0.09166	0.000182	-0.0624	0.019406
SCARB2	-0.024560911	-0.061483715	-0.10201	-0.05684	0.144436	0.350413
SCARF1	-0.017921854	-0.088738376	-0.20681	-0.04541	0.088821	0.185386
SCARF2	0.005310939	-0.081736095	-0.08831	0.013058	-0.0107	0.096414
SCARNA15	0.01484414	-0.075111637	0.23458	0.021974	-0.07412	-0.09465
SCARNA17	0.01250524	0.230495122	0.156334	-0.03622	0.08423	-0.18195
SCARNA2	-0.000301323	-0.005482991	0.095737	-0.00284	0.044305	-0.13637
SCART1	-0.019855011	-0.00819968	0.11907	-0.03527	0.08353	-0.07418
SCCPDH	0.012510638	0.011343695	0.102207	0.085298	-0.2322	0.242122
SCD	0.012014593	-0.003771196	0.0677	0.100894	-0.48821	-0.10636
SCD5	0.01764109	0.122260941	0.102217	0.071144	0.031703	-0.09915
SCEL	-0.065054585	-0.112161751	0.047182	-0.02894	-0.02337	-0.08956
SCFD1	0.027772584	0.037726451	-0.17809	0.02662	-0.057	0.156011
SCFD2	0.019322912	0.026533174	0.127874	-0.02962	0.099547	-0.06012
SCG2	-0.041515913	-0.056329951	-0.06955	0.017874	-0.0387	-0.00868
SCG3	-0.090864245	-0.078597579	-0.03485	-0.04954	0.130847	0.072725

SCG5	-0.027900721	-0.037235484	0.006343	0.032262	-0.07773	0.013397
SCGB1A1	-0.046016522	-0.05282128	-0.05569	0.014488	-0.00932	0.046378
SCGB1D1	-0.039718479	-0.050549932	-0.03046	0.018986	-0.032	-0.05376
SCGB1D2	-0.03911847	-0.01358209	-0.1074	-0.00155	0.024219	0.106997
SCGB2A1	-0.053226503	0.020368754	-0.10742	-0.00566	0.016307	0.106003
SCGB2A2	-0.075541844	-0.068632598	0.006841	0.009434	-0.04212	-0.03102
SCGB2B2	-0.030030652	-0.017478402	0.00855	0.035887	-0.09607	0.069555
SCGB3A1	-0.011529763	-0.197901564	0.139645	-0.01671	0.037819	0.026256
SCGB3A2	-0.031028812	-0.122995246	-0.09691	-0.0074	0.04602	0.338812
SCGN	-0.030968212	-0.045236552	-0.01457	0.015513	-0.06363	0.075955
SCIMP	-0.036679816	-0.065138571	-0.22558	-0.01979	0.050093	0.173625
SCIN	0.006575872	-0.047806366	-0.05659	-0.01598	-0.05073	0.008878
SCLY	0.003825672	-0.010120209	0.037827	0.03788	-0.17099	-0.0615
SCMH1	-0.000620121	-0.171337605	0.066195	-0.02264	-0.00491	-0.25351
SCML1	-0.003180754	-0.164670524	0.256558	-0.04968	0.172189	-0.01411
SCML2	0.012122167	-0.168266426	0.156309	-0.0127	-0.02274	-0.23137
SCML4	-0.003586451	0.09792724	0.441447	-0.03312	0.096802	-0.35496
SCN10A	-0.026207625	0.016334381	0.065355	-0.00206	0.010332	-0.04453
SCN11A	-0.050322295	0.011258234	0.084977	-0.01899	0.085136	-0.15433
SCN1A	-0.052753805	-0.081271694	0.035331	0.011251	-0.02332	-0.14606
SCN1B	-0.014751837	-0.087034743	0.004723	-0.07603	0.16962	-0.01897
SCN2A	-0.058000614	-0.080659129	-0.02842	0.063555	-0.11363	-0.15082
SCN2B	-0.045128675	-0.01758974	-0.01041	0.004171	-0.03928	0.147
SCN3A	-0.010268486	-0.058413662	-0.08198	0.062368	-0.06312	-0.19521
SCN3B	-0.035826926	-0.031882693	0.071293	0.012012	-0.02642	-0.07236
SCN4A	-0.031200864	-0.008597619	-0.00875	-0.0123	0.037004	-0.06376
SCN4B	-0.029194753	-0.096940879	-0.08563	-0.00282	0.021749	-0.01026
SCN5A	-0.023361289	-0.081717512	-0.06365	-0.01237	0.032353	0.05716
SCN7A	-0.064203855	-0.0861042	-0.0274	-0.02159	0.067619	-0.06221
SCN8A	-0.036870866	-0.109483637	-0.13544	0.017132	0.062421	0.228656
SCN9A	-0.008771248	-0.063117861	-0.08954	-0.01522	0.059273	-0.14627
SCNN1A	-0.005049094	-0.10983259	0.002853	0.009003	-0.08376	-0.08029
SCNN1B	-0.031515364	-0.021014601	0.038059	0.011029	-0.00389	0.142502
SCNN1D	-0.030859963	-0.048777994	0.116724	0.005866	-0.00173	0.025013
SCNN1G	-0.071386291	-0.030596875	-0.12125	-0.01132	0.034758	0.114248
SCO1	0.021340943	0.047541441	-0.01483	0.081301	-0.21876	0.196518
SCO2	0.025980844	-0.076425076	-0.29319	-0.0172	0.06199	0.507025
SCOC	0.010068317	0.055748892	0.145443	-0.07103	0.205969	-0.08418
SCOC-AS1	-0.042952248	-0.109093614	0.066832	-0.0136	0.072509	-0.11428
SCP2	0.03823819	0.120364377	0.067728	-0.0043	0.025809	0.133165
SCP2D1	-0.037206228	-0.06050061	0.050903	0.025351	-0.06026	0.00893
SCPEP1	0.010152768	-0.111884276	-0.34726	-0.03383	0.086905	0.441084
SCRG1	-0.059033011	-0.059983269	-0.03952	-0.01105	0.048387	0.284752
SCRIB	0.035303867	0.094460981	-0.00363	0.071465	-0.1839	0.088523
SCRN1	0.018836011	0.045879979	-0.19042	-0.10244	0.283001	0.008572
SCRN2	0.005730157	0.012241571	0.075026	-0.00594	-0.00088	0.007667
SCRN3	-0.039164363	0.04226433	-0.02657	-0.02995	0.176315	0.083348
SCRT1	-0.042506958	-0.010906626	-0.00052	-0.01079	0.055673	0.018214
SCT	-0.004198922	-0.053859724	-0.05907	0.009105	-0.00825	-0.06766
SCTR	-0.03326167	0.009520112	0.026747	0.023148	-0.0509	-0.0654
SCUBE1	-0.019765043	-0.04287178	-0.02482	0.002184	0.002575	0.047739

SCUBE2	-0.039727029	-0.078647895	-0.15599	-0.00462	0.034848	0.087747
SCUBE3	-0.026065407	-0.016413751	0.114558	0.020168	-0.05531	-0.24031
SCYL1	0.006048052	-0.096651259	-0.13325	0.023646	-0.07405	0.069571
SCYL2	0.053012812	0.146098985	-0.19224	-0.00266	0.015416	-0.0229
SCYL3	0.029133442	0.014941795	-0.00062	0.031278	-0.09621	-0.29724
SDAD1	0.040903353	0.064539652	0.183526	0.052292	-0.03996	-0.0103
SDC1	-0.043298402	-0.107270101	-0.08181	-0.00151	-0.01719	-0.06929
SDC2	-0.006268961	-0.044266645	-0.07034	0.044031	-0.07954	0.027489
SDC3	-0.044955368	-0.129959318	-0.0815	-0.00571	0.017346	0.22191
SDC4	-0.003368561	-0.018230051	-0.04477	0.017234	-0.06241	0.061289
SDCBP	0.040669946	-0.069137336	-0.3456	-0.04503	0.176933	0.305179
SDCBP2-AS	-0.028589986	-0.048443465	0.123205	-0.00421	0.016659	0.089268
SDCCAG3	-0.014519909	0.026363848	0.145614	-0.00482	0.035182	0.001036
SDCCAG8	0.010544671	-0.113998915	-0.09383	-0.03028	0.052632	0.406053
SDE2	0.024825615	0.044047661	-0.12297	0.057882	-0.17669	-0.10713
SDF2	0.058175242	0.137457688	-0.21869	0.027664	-0.10036	0.04311
SDF2L1	0.024472782	0.160159867	0.050713	0.052688	-0.16135	0.061869
SDF4	0.067069264	0.153743984	-0.04077	0.032139	-0.10593	0.084458
SDHA	0.092562308	0.082735994	0.184639	0.089772	-0.22762	-0.22965
SDHAF1	0.037974443	0.060712511	0.104808	0.006371	-0.0653	0.039808
SDHAF2	0.056964156	0.131659671	0.193879	-0.01082	-0.01117	-0.02077
SDHB	0.036198189	-0.031463395	-0.20585	0.048063	-0.09994	0.421065
SDHC	-0.018945812	0.023522999	0.150136	-0.00266	-0.01215	0.22713
SDHD	0.039743249	0.068044411	0.012012	0.000861	0.011596	0.096015
SDK1	-0.036804545	-0.078024967	0.005638	0.003839	0.012795	-0.15061
SDK2	-0.078250198	-0.15683302	0.179074	-0.07794	0.097613	-0.21368
SDPR	-0.004468694	-0.016828595	-0.04234	-0.22256	0.658758	0.068369
SDR16C5	-0.024739418	-0.041036244	-0.02574	-0.01893	0.028834	-0.11113
SDR39U1	0.031050234	0.045494392	0.235002	-0.014	0.060307	-0.01282
SDR42E1	-0.031163077	0.125071304	0.194458	-0.03996	0.122863	-0.34979
SDR9C7	-0.048217883	-0.056717052	0.104313	-0.0043	0.046027	0.159204
SDS	-0.024579652	-0.034972013	0.027793	0.008857	-0.07241	-0.10718
SDSL	-0.012608055	-0.04794416	-0.2125	0.034705	-0.0859	0.2083
SEC11A	0.082945155	0.13344394	-0.1218	0.048158	-0.09094	0.437191
SEC11C	0.027722023	0.117781157	0.108123	0.082794	-0.22772	-0.11995
SEC13	0.08943633	0.059376627	-0.23107	0.103667	-0.24396	0.118537
SEC14L1	0.022158625	-0.000646004	-0.38115	-0.01921	0.053003	0.228463
SEC14L1P1	-0.006295141	0.083026779	0.172059	0.001406	0.028276	-0.18062
SEC14L2	-0.036149991	-0.148181859	0.095107	0.051756	-0.03551	0.002934
SEC14L3	-0.028452238	-0.038746509	0.013715	0.019006	-0.05325	-0.01203
SEC14L4	-0.045282272	-0.085586652	0.031276	0.016279	-0.0155	-0.03568
SEC14L5	-0.026423463	0.025423589	-0.02664	-0.17267	0.458533	0.037937
SEC16A	0.092702279	0.142774833	-0.10905	0.031572	-0.06936	-0.04995
SEC16B	-0.046791709	-0.037008729	-0.05667	-0.0046	-0.09173	0.291668
SEC1P	-0.022215397	-0.039683828	0.074496	0.020155	-0.04139	-0.00629
SEC22A	-0.005147503	0.086873742	-0.02315	0.014531	-0.01272	0.102479
SEC22B	0.060936722	0.127535863	-0.06928	0.021358	-0.04234	0.044138
SEC22C	0.011633872	0.057626229	0.114828	0.05688	-0.12638	-0.06007
SEC23A	0.03168419	0.07299454	-0.08551	0.024348	-0.06522	0.112301
SEC23B	0.051399636	0.117179312	-0.24048	0.114874	-0.15612	0.182688
SEC23IP	0.00209536	0.081726137	-0.10185	0.011355	-0.03895	0.132992

SEC24A	0.01859251	0.081462587	-0.07332	0.050191	-0.11777	0.15522
SEC24B	0.074660528	0.114151726	-0.09412	0.017982	-0.03401	-0.08118
SEC24B-AS	-0.023144328	-0.048483524	0.146187	0.012553	-0.01806	-0.07954
SEC24C	0.073414903	0.192758603	-0.00127	0.047009	-0.11683	-0.13415
SEC24D	-0.010618517	-0.008590711	-0.08733	0.00095	0.006361	-0.05363
SEC31A	0.046869837	0.071570779	0.054441	0.037835	-0.07877	0.116333
SEC31B	0.011402704	-0.092320803	0.310323	0.023097	-0.05821	-0.07005
SEC61A1	0.036749174	0.055871675	-0.03245	0.015459	-0.0636	0.160132
SEC61A2	-0.016693445	-0.03925974	-0.04925	0.049794	-0.07053	-0.34422
SEC61B	0.029513182	0.048964472	-0.01068	0.035416	-0.0668	0.056419
SEC61G	0.04111701	0.076503305	0.113385	0.072323	-0.21606	0.080069
SEC62	0.034004642	0.03121109	0.058448	-0.01067	0.012753	-0.18899
SEC63	-0.012258442	-0.000114035	0.011441	0.003754	0.180213	0.032888
SECISBP2	0.074304717	0.012166574	0.146665	-0.05053	0.146008	-0.32899
SECISBP2L	0.028383559	0.102982597	-0.11317	-0.01193	0.01556	-0.05504
SECTM1	0.001094402	-0.080231466	-0.21308	-0.09326	0.237172	0.365794
SEH1L	0.015813867	0.023827895	-0.00577	0.068749	-0.17443	0.162543
SEL1L	0.013062925	0.028062445	-0.21186	0.018523	-0.08327	0.161198
SEL1L2	-0.043807594	0.022126531	-0.1048	0.020025	-0.0287	0.061928
SEL1L3	-0.003717677	-0.062285071	0.029596	0.001178	-0.02508	-0.35355
SELE	-0.061902188	-0.093373764	0.064374	-0.02898	0.079126	0.01226
SELENBP1	-0.021372563	-0.070260037	-0.16356	-0.0172	0.062946	0.050877
SELK	0.09590937	0.192154104	0.040626	0.109904	-0.25307	-0.29079
SELL	0.035782696	-0.066998216	-0.02302	-0.08912	0.283033	-0.13004
SELM	-0.002718534	-0.036201505	0.417303	-0.05797	0.182539	-0.2435
SELO	0.02322676	-0.036413601	-0.18953	-0.01989	0.080955	0.337758
SELP	-0.014398195	-0.042712105	-0.01547	-0.24494	0.62934	0.018878
SELPLG	0.030681917	0.076731337	0.013609	-0.02795	0.079052	-0.05395
SELT	0.060335614	0.220519162	-0.20207	0.019664	-0.03074	0.044909
SEMA3A	-0.046234799	-0.06038717	-0.02669	0.162042	-0.11535	0.033852
SEMA3B	-0.026061633	-0.021292813	-0.06143	-0.00056	0.015012	-0.08139
SEMA3C	-0.00516626	-0.133792231	-0.30087	-0.05732	0.181046	0.44955
SEMA3D	-0.052757726	-0.084573702	-0.01265	0.018939	-0.03858	-0.04236
SEMA3E	-0.041319996	-0.042655626	-0.01783	-0.01148	0.007184	-0.07843
SEMA3F	-0.015849136	-0.090199655	0.050225	0.005973	-0.08533	-0.04317
SEMA3G	-0.025170124	-0.036076377	0.110262	-0.00235	0.015091	-0.13169
SEMA4A	0.006999336	-0.162314598	-0.28617	-0.0692	0.165585	0.355094
SEMA4B	0.007773886	-0.079261651	-0.27266	-0.0761	0.200243	0.127306
SEMA4C	0.013562799	0.133049648	0.194097	-0.04312	0.066572	-0.19841
SEMA4D	0.025838455	0.029064325	0.159586	-0.05261	0.153985	-0.11449
SEMA4F	-0.001047255	0.113320589	0.288595	-0.01397	0.108138	-0.35031
SEMA4G	-0.039958121	-0.039981098	0.004219	0.022254	-0.04581	0.087103
SEMA5A	-0.023952379	-0.120498849	-0.04572	-0.03738	0.0004	-0.12545
SEMA5B	-0.001663063	-0.035159769	0.07693	-0.00909	-0.08089	-0.15644
SEMA6A	-0.043652481	-0.055651729	-0.00098	0.21442	-0.05063	-0.05989
SEMA6B	-0.017609663	-0.094124229	0.021911	-0.00852	-0.05608	-0.05757
SEMA6C	-0.006304135	-0.051445531	-0.0662	-0.02051	-0.03984	-0.00576
SEMA6D	0.053240535	-0.04952726	-0.03375	0.123237	0.07004	-0.16142
SEMA7A	-0.005595095	-0.04112932	-0.03136	0.004725	-0.05768	-0.12438
SEMG1	-0.021744048	-0.064171942	-0.17249	-0.00256	0.013725	-0.11982
SEMG2	-0.049589121	0.007102409	-0.04897	-0.02193	0.060789	0.020831

SENCR	0.000579805	-0.041313824	-0.15439	-0.08824	0.173869	0.204994
SENP1	0.002968423	0.130122921	0.042088	0.131282	-0.37809	-0.16038
SENP2	-0.002308224	-0.020178862	-0.11375	0.089826	-0.22427	-0.15657
SENP3	0.007620191	0.021970644	0.060248	0.02444	-0.09567	0.089143
SENP5	-0.006447087	0.076263437	-0.14643	0.069009	-0.21593	-0.02731
SENP6	0.019341775	-0.054737624	-0.10765	-0.01822	0.125806	0.063909
SENP7	0.001858759	0.037433922	0.033121	-0.03585	0.109123	-0.11448
SENP8	-0.032921926	-0.013455514	-0.11316	0.067089	-0.17896	0.113872
15-Sep	0.106591636	0.104651295	-0.01341	0.043744	-0.05777	-0.16668
SEPHS1	0.007410135	0.020393432	0.050865	0.123156	-0.13786	-0.01458
SEPHS2	0.070318594	0.141302718	-0.09701	0.061765	-0.15238	-0.14407
SEPN1	-0.010289445	-0.102228801	-0.07778	-0.10665	0.279081	0.426575
SEPP1	-0.01633126	0.007664112	0.063483	-0.02059	0.04912	-0.0922
SEPSECS	0.002770233	0.107963964	0.015715	0.143845	-0.05345	-0.04001
SEPSECS-A	-0.010800195	0.084361875	0.037826	-0.00357	0.034031	-0.1059
SEPT1	0.007708922	0.069130033	0.313552	0.023644	-0.04982	-0.36204
SEPT10	-0.012485967	-0.072355482	-0.1856	-0.09915	0.294593	0.677203
SEPT11	-0.008524708	0.053939089	-0.00964	-0.02247	0.060345	-0.13702
SEPT12	-0.031969483	-0.057671934	-0.09524	0.025982	-0.10526	0.010461
SEPT2	0.006082452	0.005949463	-0.07811	0.049769	-0.08397	-0.0246
SEPT3	-0.022258842	-0.086369773	-0.06482	-0.01083	0.000891	-0.13307
SEPT4	0.007260796	-0.047991699	-0.01153	0.0605	0.015833	-0.04836
SEPT5-GP1	-0.017543044	-0.118036405	0.016615	-0.02614	0.015561	-0.01994
SEPT6	-0.003567028	0.073477987	0.31677	-0.0015	-0.06551	-0.4364
SEPT7	-0.001651383	0.175971985	0.106926	0.072648	0.003694	-0.19206
SEPT7P2	-0.010965629	0.037513633	0.118619	0.00203	0.026751	-0.09873
SEPT7P9	-0.022709893	0.03230762	0.134548	0.005784	-0.00075	-0.05321
SEPT8	-0.000702696	0.036206152	0.191552	0.143267	-0.47556	-0.02135
SEPT9	0.013682336	-0.002910397	0.165278	-0.04551	0.140548	-0.00196
SEPW1	0.036864063	0.095732307	0.313886	0.019116	-0.04138	0.059996
SERAC1	-0.011887701	0.007711292	-0.10508	0.000536	-0.0157	0.288717
SERBP1	0.035760128	0.16124161	0.075592	0.051962	-0.18517	0.03969
SERGEF	0.001443436	0.004100463	0.180499	0.007746	0.00889	-0.03734
SERHL2	-0.030981525	-0.112654042	-0.11793	0.017983	-0.06981	-0.18933
SERINC1	0.078340505	0.135689559	-0.08463	-0.00682	0.068503	-0.12112
SERINC2	0.002042623	-0.10309918	-0.13224	0.034865	-0.0565	0.123153
SERINC3	0.073732822	0.155526045	-0.02689	0.024879	-0.01991	-0.10999
SERINC5	0.021686994	-0.138105009	0.2305	-0.07949	0.189391	0.275984
SERP1	0.078621636	0.041942419	-0.19746	0.027459	-0.04877	0.287779
SERP2	-0.084568487	0.09367256	0.129942	-0.02398	0.031219	-0.09998
SERPINA1	-0.001387322	-0.107685158	-0.38412	-0.06933	0.196667	0.498216
SERPINA10	-0.030416156	-0.082390267	-0.08465	0.018756	-0.03864	0.022795
SERPINA12	-0.039238021	-0.042637147	0.086217	0.002798	0.016418	-0.08636
SERPINA2	-0.042956064	-0.059252131	0.026164	-0.00522	0.031884	0.12513
SERPINA3	-0.032272717	-0.010121447	0.085326	0.012793	-0.02136	-0.08416
SERPINA4	-0.026910211	-0.04463494	0.072359	0.014346	-0.05144	-0.07378
SERPINA5	-0.031163199	-0.008127186	-0.10608	0.000465	-0.03142	-0.02159
SERPINA6	-0.037383814	-0.035896018	0.073289	0.007118	0.002994	-0.02039
SERPINA7	-0.037592291	-0.035752914	-0.06461	0.040156	-0.08842	-0.10523
SERPINA9	-0.017850992	-0.053292785	0.012806	-0.00384	-0.08665	-0.11659
SERPINB1	0.039153591	0.04000679	-0.2981	-0.0168	0.058689	0.383858

SERPINB1(	-0.027911991	-0.059878842	-0.00562	-0.02913	0.104117	0.285053
SERPINB1:	-0.052836814	-0.042491584	-0.00402	0.020847	-0.04417	0.031105
SERPINB1:	-0.036803981	-0.044044416	-0.04564	0.008034	-0.00372	-0.00433
SERPINB1:	-0.021886972	-0.142742327	-0.05712	0.069149	-0.02116	0.019342
SERPINB2	-0.005242514	-0.032509357	-0.11973	-0.0424	0.107713	0.29977
SERPINB3	-0.041575502	-0.02938725	0.006262	0.076463	-0.0988	-0.04904
SERPINB4	-0.042468677	-0.104624259	0.001191	0.031283	-0.06181	0.084073
SERPINB5	-0.054986362	-0.023895162	0.108708	0.011532	-0.0346	-0.0851
SERPINB6	0.04085347	-0.012356517	0.155498	-0.06891	0.046467	0.054187
SERPINB7	-0.018430314	-0.017132171	0.009911	0.002748	0.004988	0.04721
SERPINB8	0.003163261	-0.09986565	-0.22673	-0.04971	0.101972	0.391861
SERPINB9	0.024767464	0.028183543	0.123787	-0.07861	0.150874	0.024887
SERPINB9I	-0.002447345	-0.056996938	-0.03385	-0.01683	0.029707	-0.07539
SERPINC1	-0.050669679	-0.005897827	-0.0563	0.018915	-0.02922	0.03281
SERPIND1	-0.043446197	-0.041979207	-0.04554	0.004948	0.007662	0.027318
SERPINE1	-0.012522566	-0.01280415	0.025623	0.018041	0.014211	0.030824
SERPINE2	-0.008889523	0.085678912	0.045489	0.000605	-0.02289	-0.14055
SERPINF1	-0.000280072	-0.160478834	-0.05418	-0.06168	0.181621	-0.09277
SERPINF2	-0.014856545	-0.140236702	-0.15008	0.008303	-0.00241	0.00035
SERPING1	-0.006892175	-0.068765533	-0.26979	-0.07194	0.201792	0.164915
SERPINH1	-0.007190292	0.049166714	0.022015	-0.01365	-0.02222	-0.04445
SERPINI1	0.003690266	0.127961712	0.116442	-0.02427	0.08675	-0.38426
SERPINI2	-0.020651221	-0.114066801	-0.12606	-0.01495	0.063333	0.192567
SERTAD1	0.027052297	0.023999812	-0.11081	0.013288	-0.05066	0.055889
SERTAD2	0.058682024	-0.017536516	-0.22048	0.038331	-0.09075	-0.04653
SERTAD3	0.014754756	-0.081504031	-0.34369	-0.02642	0.075864	0.256704
SERTAD4	-0.055795449	-0.003737852	-0.00965	0.050428	-0.002	0.034832
SERTAD4-/	-0.039056113	-0.056526683	-0.10081	0.023892	-0.04223	0.148312
SERTM1	-0.057777521	-0.076967589	-0.07629	-0.00519	0.020599	-0.01116
SESN1	0.016447742	0.071107013	0.168478	0.017507	-0.04306	-0.33979
SESN2	0.019857971	0.097805864	0.009118	0.070922	-0.2179	-0.08869
SESN3	0.025449371	-0.077962724	0.03374	-0.11206	0.168265	0.025287
SESTD1	-0.0053761	-0.081709222	-0.2448	-0.05385	0.200949	0.427424
SET	0.094126943	0.171608538	0.083454	0.033871	-0.06093	-0.24123
SETBP1	0.004945098	0.084295313	-0.14555	0.005722	-0.01156	-0.1421
SETD1A	-0.002973773	-0.048139848	0.042989	0.00601	-0.03128	-0.02651
SETD1B	0.08651675	-0.026877569	0.09706	0.00716	0.043698	-0.33979
SETD2	-0.002491371	0.040123206	-0.0183	0.016937	0.002586	-0.14314
SETD3	0.035512848	0.162934869	-0.18508	0.031753	-0.08093	0.192527
SETD4	0.020864498	0.113247516	0.201117	-0.13124	0.007303	-0.06375
SETD5	0.03236255	0.012018607	0.136247	0.05898	-0.19346	-0.29623
SETD6	0.013723589	0.048990223	0.202546	-0.00754	0.054163	-0.08542
SETD7	0.006540956	-0.183506302	-0.23108	-0.04508	0.118382	0.408434
SETD8	-0.022799028	0.013225489	-0.13536	0.031567	-0.17216	-0.10953
SETD9	-0.007320291	-0.056830051	-0.08305	0.034776	-0.07826	0.394599
SETDB1	0.020749899	-0.096769979	-0.14458	-0.00995	-0.02385	0.288742
SETDB2	-0.005869904	-0.037108011	0.05704	-0.01504	0.084803	-0.09653
SETMAR	0.080417925	-0.116150826	-0.02566	0.128717	0.011594	-0.03483
SETX	0.115077834	0.118192493	-0.20806	-0.04352	0.088817	-0.07177
SEZ6	-0.034920579	-0.082242283	-0.00047	0.019223	-0.06461	0.02263
SEZ6L	-0.000140219	-0.069784153	-0.08043	-0.02995	0.094164	0.163711

SEZ6L2	-0.026758499	-0.080358534	0.028393	0.018277	-0.08092	-0.00978
SF1	0.022866986	-0.063476268	0.169321	-0.06225	0.166988	-0.25063
SF3A1	0.149529744	0.016510948	0.047728	0.012982	-0.05646	-0.25389
SF3A2	0.02269788	-0.074439404	0.106219	0.06132	-0.15808	0.094993
SF3A3	0.042131304	0.009058764	0.078939	0.036501	-0.11313	-0.10127
SF3B1	0.090505009	0.08382594	0.093289	0.032222	-0.02824	-0.20107
SF3B2	0.03462508	0.047580034	-0.04757	0.027936	-0.12682	-0.20671
SF3B3	0.023180878	0.040516909	0.156729	0.10128	-0.3139	-0.07938
SF3B4	0.053646236	0.003377724	-0.12904	0.077792	-0.21906	0.20039
SF3B5	0.074916728	0.038229536	0.100865	0.065766	-0.1752	0.231837
SF3B6	0.05488226	0.150680933	0.149928	0.064026	-0.11603	-0.00463
SFI1	0.028758586	0.003383959	0.425562	-0.01656	0.048593	-0.33726
SFMBT1	-0.009543573	0.013718308	0.22796	0.015228	-0.0895	-0.29801
SFMBT2	0.014480494	0.066957649	0.158704	0.00821	-0.03559	-0.11302
SFN	-0.013813035	-0.072123916	-0.10286	0.003365	-0.03292	0.00937
SFPQ	-0.021935173	0.027862616	0.032417	0.03466	-0.15546	-0.03835
SFR1	0.01273263	0.060972864	0.013068	0.001278	0.009657	0.194901
SFRP1	-0.009228329	-0.157591445	-0.03293	0.024829	0.007861	-0.08022
SFRP2	-0.061916841	-0.116523647	0.07297	0.004013	0.029682	0.084641
SFRP4	-0.068518868	-0.069225976	0.005653	0.026276	-0.05225	0.030595
SFRP5	-0.03017013	-0.118150951	0.344837	0.004809	0.000495	-0.07007
SFSWAP	0.030336529	-0.001302193	0.181665	-0.02507	0.071461	-0.00766
SFT2D1	-0.003651968	-0.027679074	-0.12572	-0.0498	-0.08555	0.012001
SFT2D2	0.052446723	0.065268841	-0.15292	0.021176	-0.07839	0.016787
SFT2D3	0.004378068	-0.030019995	0.146824	-0.0049	-0.05346	-0.15062
SFTA1P	-0.036631325	-0.055370691	-0.12664	0.024839	-0.04366	0.080518
SFTA2	-0.036933299	0.07222529	-0.00636	-0.00657	0.033391	-0.02679
SFTA3	-0.072016489	-0.055048887	-0.02519	-0.03159	0.05541	-0.02299
SFTPB	-0.000464646	-0.060400809	0.09332	-0.01381	-0.0026	-0.00598
SFTPC	-0.100988573	-0.055715862	-0.06229	-0.00479	-0.05902	-0.00374
SFTPD	-0.021307555	-0.066175314	-0.15022	-0.03196	0.115674	0.447781
SFXN1	-0.004980934	0.025937372	0.452827	0.079182	-0.13486	-0.29077
SFXN2	-0.008564535	-0.019371401	0.270457	0.024349	-0.13953	-0.41473
SFXN3	0.0178614	0.086614443	0.032104	-0.05828	0.121333	0.317064
SFXN4	-0.012578654	-0.103373082	0.116478	0.06932	-0.1234	0.040693
SFXN5	-0.03161426	-0.030863441	-0.0782	-0.00179	-0.0138	0.239611
SGCA	-0.024581607	-0.031117588	-0.02413	0.026071	-0.04973	-0.12721
SGCB	-0.019671817	-0.081613384	0.186951	0.092124	-0.25663	-0.00616
SGCD	0.004961851	0.045731923	0.203515	0.069854	0.069285	-0.15905
SGCE	-0.013879733	-0.048867004	-0.004	0.012184	-0.02759	-0.11305
SGCG	-0.045557281	-0.073006045	-0.02192	0.032275	-0.06612	-0.0119
SGCZ	-0.047235381	0.003456386	0.008265	0.015148	-0.02221	-0.10492
SGIP1	-0.067277131	-0.100322592	0.008242	-0.00348	0.010956	-0.02625
SGK1	0.013401951	-0.114142878	-0.22751	-0.0112	0.030915	0.265845
SGK2	-0.055925185	-0.087442661	0.011384	-0.03983	0.043102	-0.19496
SGK223	0.017017644	-0.031129257	0.259865	-0.10706	0.283955	-0.28122
SGK494	0.001965521	-0.090828349	-0.09612	0.025488	-0.06281	0.044486
SGMS1	0.012905605	-0.017722328	-0.15631	0.011101	-0.01901	0.334967
SGMS2	0.014331125	-0.028958893	-0.30818	-0.05473	0.14155	0.513154
SGOL1	-0.039176784	-0.053789225	-0.03566	0.179243	-0.41022	-0.14494
SGOL2	0.002511814	0.098866688	-0.02934	0.121048	-0.30945	0.023801

SGPL1	0.03364906	0.062619043	-0.09148	-0.01644	0.022512	0.163011
SGPP1	0.016000326	0.123143423	-0.02799	0.004108	-0.03994	-0.24628
SGPP2	-0.017544212	-0.025756763	0.027358	-0.00998	0.03109	-0.03459
SGSH	0.018670076	-0.024619812	0.07449	-0.0331	0.104564	0.318592
SGSM1	-0.030639277	0.038128647	0.020674	0.037393	0.014418	-0.1252
SGSM2	0.010835131	-0.13178768	0.227496	-0.04319	0.16749	-0.13276
SGSM3	0.033398944	-0.129845894	0.116874	-0.01989	0.089775	-0.12935
SGTA	0.01411973	-0.042016409	-0.11611	0.049742	-0.20392	0.017947
SGTB	0.011381347	-0.120066237	0.184506	-0.04391	0.12419	0.267741
SH2B1	0.01910904	-0.077467898	0.286326	-0.06902	0.122214	-0.22067
SH2B2	0.02962127	-0.098816921	-0.06527	-0.10591	0.255273	-0.06309
SH2B3	0.033202291	0.00957008	-0.33108	-0.10181	0.274015	0.295248
SH2D1A	0.012567536	0.175107316	0.514918	0.04781	-0.14022	-0.37818
SH2D1B	-0.001339953	0.429285415	-0.13928	-0.03869	0.117153	-0.05275
SH2D2A	0.006482855	0.283717442	0.23639	0.056367	-0.20029	-0.266
SH2D3A	-0.006161834	-0.044981784	0.345242	-0.0343	0.116589	-0.30349
SH2D3C	0.007709732	0.001231912	-0.14249	-0.0784	0.173951	0.056316
SH2D4A	-0.020199746	-0.058319052	-0.07723	0.042308	-0.17353	-0.00632
SH2D4B	-0.027608899	-0.028058516	-0.04317	0.012182	-0.02369	-0.04165
SH2D5	-0.027866448	-0.047054774	-0.12518	-0.00232	-0.01008	0.010869
SH3BGR	-0.018762102	-0.083431163	0.09099	0.043351	-0.14897	-0.24323
SH3BGRL	0.059568129	-0.004271583	-0.15674	-0.06536	0.197898	0.303033
SH3BGRL2	-0.008924306	-0.086587602	0.355243	-0.2299	0.629618	-0.08346
SH3BGRL3	0.057074467	0.079423516	-0.00383	-0.01032	0.077584	0.346967
SH3BP1	0.014905907	-0.064185084	0.069649	-0.01463	-0.0329	0.066345
SH3BP2	0.025523664	0.034683136	-0.25555	-0.06954	0.055453	0.232376
SH3BP4	-0.00923413	-0.106046457	-0.17358	-0.03953	0.12723	-0.00353
SH3BP5	0.044641062	0.181685555	-0.04685	-0.05983	0.152166	-0.29462
SH3BP5-AS	0.009272332	-0.095777442	0.179355	-0.06867	0.21751	-0.11244
SH3BP5L	0.005536592	-0.030880054	-0.20818	-0.03389	0.032153	0.052096
SH3D19	-0.028038864	-0.082962134	-0.06447	0.011532	-0.02792	-0.13518
SH3D21	-0.011676455	-0.102445222	-0.07309	0.014226	-0.08678	-0.21292
SH3GL1	0.042887696	-0.089052839	0.039695	0.006266	-0.07618	-0.09895
SH3GL1P1	-0.075506798	-0.046877219	-0.05662	-0.03443	0.090316	0.059085
SH3GL1P2	-0.037426783	-0.019664039	0.026557	0.047027	0.003392	0.02405
SH3GL2	-0.038420343	-0.035105097	-0.01682	-0.01055	0.021059	-0.07252
SH3GL3	-0.047765875	-0.010598639	0.118394	-0.03244	-0.06764	-0.08288
SH3GLB1	0.073837344	0.139763339	-0.3463	0.025749	-0.01704	0.118129
SH3GLB2	-0.014227648	-0.125591995	0.156191	0.010854	-0.06367	-0.0697
SH3KBP1	0.079806711	0.143972686	-0.02281	-0.00821	0.047857	-0.09133
SH3PXD2A	-0.016232249	-0.03956208	0.064199	0.025876	-0.04235	-0.12043
SH3PXD2A-	-0.02613942	-0.014922506	-0.13003	0.019738	-0.0504	-0.05485
SH3PXD2B	-0.038396549	-0.042096559	-0.04906	-0.00819	-0.0021	0.235017
SH3RF1	-0.01974781	-0.093817101	-0.25557	-0.05975	0.177432	0.441446
SH3RF2	-0.037195078	-0.038927279	-0.05132	0.104085	-0.16066	-0.01132
SH3RF3	-0.009541214	-0.169884213	0.222208	-0.02253	0.081586	-0.0156
SH3RF3-AS	-0.060303761	-0.076874952	0.120818	-0.03601	0.061353	0.000445
SH3TC1	-0.005840193	-0.096514845	-0.26942	-0.06361	0.194153	0.38827
SH3TC2	-0.045214888	-0.056868533	0.120584	0.020941	-0.02754	-0.09126
SH3YL1	0.008698159	0.014474205	0.408645	-0.06588	0.151977	-0.39719
SHANK1	-0.038103708	-0.02088652	-0.02801	-0.00962	0.051145	-0.0299

SHANK2	-0.060397025	-0.039429769	0.160604	0.005121	-0.01577	-0.12651
SHANK2-AS	-0.038002279	-0.055951915	-0.03906	0.008543	-0.00524	-0.02425
SHANK3	-0.048297062	-0.07341808	-0.04759	-0.01317	0.069839	-0.09624
SHARPIN	0.031653614	-0.035801614	0.096205	-0.00398	-0.06926	0.002037
SHB	-0.025582672	-0.017177289	-0.13148	0.016337	-0.01195	0.047165
SHBG	-0.025279656	-0.072007059	-0.11193	0.005102	-0.05438	-0.00563
SHC1	0.05845967	0.061756693	-0.12718	-0.03693	0.08188	0.124357
SHC2	-0.027937061	-0.076180182	0.129972	-0.01263	0.022613	-0.07058
SHC3	-0.057133317	-0.086960237	-0.06732	0.084072	-0.03708	-0.00409
SHC4	-0.024946919	-0.02682134	0.000337	0.044927	-0.1331	-0.03061
SHCBP1	-0.007322795	-0.00736445	-0.03642	0.072371	-0.20383	0.059912
SHCBP1L	-0.044383004	-0.089730695	-0.03399	0.024552	-0.03102	0.116489
SHD	-0.009335356	-0.084731367	-0.08482	0.012125	-0.00862	-0.09468
SHE	-0.031269289	-0.071053462	-0.07784	-0.03772	0.112051	-0.10478
SHF	-0.024062552	-0.092641425	0.037586	0.011551	-0.03673	0.045558
SHFM1	0.048212948	0.04973666	0.132388	0.0873	-0.25628	0.131214
SHH	-0.044747444	-0.059237844	-0.00162	-0.0069	0.026891	0.007843
SHISA2	-0.015152819	-0.055934648	0.148222	0.10318	-0.13794	-0.05884
SHISA3	-0.096286533	-0.077772077	0.050375	-0.06809	0.12762	0.018752
SHISA4	-0.02291447	0.007106449	0.005166	0.021447	-0.05275	0.084947
SHISA5	0.05470367	0.087794402	0.083632	0.018312	-0.02114	-0.10613
SHISA6	-0.058661309	-0.01052065	-0.08765	0.016186	-0.04832	0.113676
SHISA7	-0.03038316	-0.021657753	0.008663	-0.00945	-0.00964	-0.0405
SHISA8	-0.014108982	-0.03407316	-0.10496	-0.02332	0.082698	-0.02662
SHISA9	-0.045842058	-0.046113893	0.012406	0.025559	-0.04773	-0.16887
SHKBP1	0.031160458	-0.076236382	-0.34898	0.001231	0.009769	0.299922
SHMT1	-0.056905588	-0.000794346	-0.06472	0.069089	-0.16379	0.093022
SHMT2	0.013727649	0.018892755	-0.07721	0.062456	-0.20255	0.02187
SHOC2	0.082877074	0.057944808	-0.40549	-0.02084	0.082262	-0.15958
SHOX	-0.028083862	-0.018537474	0.020007	0.014457	-0.04498	-0.02421
SHOX2	-0.066532692	-0.052036418	-0.06083	-0.05986	0.047736	0.024602
SHPK	-0.015127278	-0.009331735	-0.01062	0.025682	-0.13058	-0.11203
SHPRH	0.017093455	0.066228766	0.018397	-0.00374	0.018442	0.076586
SHQ1	0.023104701	0.023261742	0.123777	0.019285	-0.11401	-0.12072
SHROOM1	-0.037297649	-0.06978938	-0.09825	-0.02156	0.008532	0.316303
SHROOM2	-0.034180416	0.010848676	0.012084	0.046501	-0.11394	-0.09828
SHROOM3	-0.109588586	-0.032172944	0.093259	-0.01281	0.016939	0.1023
SHROOM4	-0.042146198	-0.123506738	0.071591	-0.0006	-0.00464	-0.04164
SI	-0.079612198	-0.076792647	-0.01698	-0.02075	0.104993	0.010876
SIAE	0.018686736	-0.060920728	0.052108	-0.07466	0.090589	0.06711
SIAH1	-0.012731605	-0.036821097	-0.16476	0.029896	-0.1292	-0.11536
SIAH2	0.022499353	-0.033298378	-0.12584	0.009518	-0.0474	0.031969
SIAH3	-0.065534024	-0.049189277	-0.07085	-0.01938	0.063032	0.015074
SIDT1	0.009381611	0.09612372	0.181472	-0.0213	0.065724	-0.38341
SIDT2	0.031760048	-0.085946657	-0.32803	-0.0726	0.191877	0.40896
SIGIRR	0.019748188	0.12977141	0.250492	-0.04532	0.113473	-0.01908
SIGLEC1	0.001108691	-0.088358337	-0.14155	-0.07612	0.190876	0.327661
SIGLEC10	0.003683668	-0.143376275	-0.38033	-0.0748	0.194183	0.209024
SIGLEC11	-0.035255796	-0.110233842	-0.14927	-0.03124	0.117134	0.431334
SIGLEC15	0.007157935	-0.06182871	-0.08379	-0.07325	0.167814	0.450103
SIGLEC16	-0.012943424	-0.099987584	-0.16543	-0.06804	0.212051	0.69034

SIGLEC17P	0.004670224	0.224736319	0.192576	0.045771	-0.15076	-0.12745
SIGLEC5	0.00304914	-0.11828053	-0.34034	-0.04899	0.14006	0.352873
SIGLEC6	-0.027502001	-0.097358155	0.009716	-0.0136	-0.03319	-0.06725
SIGLEC7	0.027631311	0.168804836	-0.35572	-0.09229	0.183069	0.410683
SIGLEC8	0.075150077	-0.07073405	-0.1807	0.122105	0.020786	-0.11333
SIGLEC9	-0.001128728	-0.023227242	-0.29844	-0.08816	0.247673	0.586061
SIGLECL1	-0.044247965	-0.025021199	-0.07498	0.051605	-0.14214	0.071488
SIGMAR1	0.015116084	-0.026456167	0.11456	0.080121	-0.25622	0.014531
SIK1	0.003842811	-0.050913529	-0.00911	-0.00951	0.020033	-0.10679
SIK2	0.027983263	0.011304405	-0.1232	-0.01523	-0.02576	0.193479
SIK3	0.024068066	-0.013295695	-0.12529	-0.00301	0.153238	0.179856
SIKE1	0.001004445	0.086684169	0.11724	0.066487	-0.1223	0.060671
SIL1	0.018054114	-0.03864312	-0.18602	0.019785	-0.12762	0.29617
SIM1	-0.044315613	-0.044665527	0.070741	0.014282	-0.01837	-0.01682
SIM2	0.017363859	-0.045183909	-0.02057	0.083829	0.037764	-0.04913
SIMC1	0.013944884	0.064183379	-0.06799	-0.08552	0.244909	0.007045
SIN3A	0.021071221	0.037920221	0.095599	0.002354	-0.04077	-0.34291
SIN3B	-0.003044345	-0.119324434	-0.06075	-0.05086	0.16828	0.415324
SIPA1	0.030299116	0.103340686	-0.05224	-0.03448	0.095824	-0.03001
SIPA1L1	-0.073135924	-0.132604326	-0.1534	-0.06116	-0.02921	0.036502
SIPA1L2	0.00145681	-0.07276539	-0.15968	-0.03231	0.097946	0.11394
SIPA1L3	-0.012193949	-0.089885325	-0.07297	-0.00129	-0.01893	-0.00118
SIRPA	0.009265553	-0.072374289	-0.338	-0.08251	0.254599	0.388759
SIRPB1	-0.015103828	-0.106480628	-0.35867	-0.08178	0.211401	0.501639
SIRPB2	-0.000990181	-0.106796914	-0.39031	-0.05455	0.16841	0.642297
SIRPD	-0.006902089	-0.093520858	-0.33356	-0.04273	0.082219	0.538368
SIRPG	-0.007259545	-0.099657894	0.510948	-0.05421	0.138356	-0.21676
SIRT1	0.033630654	0.026675906	-0.05241	-0.02113	0.059497	-0.07368
SIRT2	0.029884485	0.188021881	0.03299	-0.01171	0.028524	-0.13043
SIRT3	-0.035023139	-0.058774549	0.115488	-0.02727	-0.06454	-0.05975
SIRT4	-0.016711073	0.088008284	0.094207	0.045921	-0.12034	-0.05939
SIRT5	0.030490586	0.006611694	0.064908	-0.04105	0.031981	-0.07124
SIRT6	0.002813966	-0.070655968	-0.07733	0.026625	-0.18945	-0.05216
SIRT7	0.064644901	0.040124381	-0.13602	-0.04679	0.113376	0.145141
SIT1	0.00683831	-0.17650632	0.290519	0.010592	-0.07609	-0.29226
SIVA1	0.026135895	-0.089700941	0.07631	0.111485	-0.35271	0.159469
SIX1	-0.023416607	-0.033058076	-0.09656	0.09981	-0.01507	0.093476
SIX2	-0.044766179	-0.104018406	-0.07048	-0.00523	0.024919	0.13153
SIX3	-0.087077559	-0.042331972	-0.05555	-0.03422	-0.0089	0.216389
SIX3-AS1	-0.032229104	-0.048773425	0.007568	0.009657	-0.00473	-0.12778
SIX4	-0.037753194	-0.043544555	0.046725	-0.01008	-0.00301	-0.05635
SIX5	-0.037617144	0.018074668	0.012279	0.043231	-0.11316	0.051155
SIX6	-0.041223868	-0.081262213	-0.14035	########	0.028834	0.040302
SKA1	-0.024749523	0.016494022	0.021329	0.179277	-0.2928	-0.03823
SKA2	0.016091059	0.0713471	0.066101	0.058601	-0.14208	0.049597
SKA3	-0.011173853	-0.011964671	-0.02359	0.117419	-0.35532	0.065163
SKAP1	0.010779059	0.177877474	0.423682	0.00834	-0.04302	-0.38607
SKAP2	0.014220213	0.015374681	-0.46466	-0.04855	0.157959	0.223002
SKI	0.015759741	0.019428567	0.063066	-0.01308	0.036017	-0.0275
SKIDA1	-0.09232077	-0.110878225	0.063749	-0.00053	-0.03018	0.098262
SKIL	-0.060322878	0.071379006	-0.16614	-0.0058	-0.04323	0.022665

SKIV2L	0.053661954	0.009228343	-0.06129	0.043649	-0.15069	-0.2363
SKIV2L2	0.037895695	0.118782423	0.067872	0.072194	-0.11823	-0.05355
SKP1	0.086002977	0.08788087	0.1731	0.073708	-0.15862	-0.19728
SKP2	-0.017862619	0.007562136	0.046923	0.067123	-0.15782	-0.03022
SLA	0.034163529	0.106304228	-0.03083	-0.01077	0.038636	0.027652
SLA2	0.010266351	0.176493622	0.260738	-0.04855	0.096059	-0.34205
SLAIN1	0.00153785	0.036535605	0.309935	0.008593	0.022751	-0.38235
SLAIN2	0.004659486	0.076922832	0.049451	0.003841	0.00224	-0.0471
SLAMF1	0.007668415	-0.073209885	0.18889	-0.06137	0.02669	-0.28646
SLAMF6	0.007984428	0.148687389	0.286622	-0.08053	0.148067	-0.45044
SLAMF7	0.015183321	0.199568465	0.147766	-0.00579	-0.00844	-0.1045
SLAMF8	-7.88E-06	-0.014661775	-0.07151	-0.00933	0.031669	0.140549
SLAMF9	-0.038894559	-0.020271099	-0.05476	0.002071	-0.04455	-0.04146
SLBP	0.072570439	0.140781497	-0.10075	0.105276	-0.24518	0.072429
SLC10A1	-0.02413967	-0.052450732	0.192962	-0.00095	-0.02748	-0.18419
SLC10A2	-0.042941423	0.038283231	-0.00651	-0.0207	0.078112	0.086462
SLC10A3	0.028974872	0.10384939	0.051917	-0.04545	0.092638	-0.15827
SLC10A4	-0.133097991	0.011057358	0.008785	0.001573	0.054682	-0.0328
SLC10A7	-0.027849018	-0.021245131	0.046409	0.013604	-0.02859	-0.03929
SLC11A1	0.036469099	-0.056441805	-0.32552	-0.02201	0.135051	0.333938
SLC11A2	0.027960402	0.022682989	0.114326	0.015527	0.000712	0.175904
SLC12A1	-0.088878992	-0.062560361	-0.04262	-0.02533	0.066994	-0.08522
SLC12A2	0.008338033	0.075288913	0.056025	0.112526	-0.28241	-0.22555
SLC12A3	-0.001771639	-0.031612207	-0.12062	0.014039	0.045897	-0.11142
SLC12A4	-0.028339643	-0.112888257	-0.02037	0.005139	-0.04741	-0.04047
SLC12A5	-0.034733579	-0.094424018	0.152004	0.013849	-0.02587	0.069373
SLC12A6	0.003835196	-0.065678699	-0.24478	-0.04728	0.135732	0.046508
SLC12A7	0.03587802	-0.020955302	0.066202	-0.0725	0.192491	0.217808
SLC12A8	-0.00838852	-0.007786104	-0.02334	0.026364	-0.07264	0.021722
SLC12A9	-0.043224984	-0.178337089	-0.05538	-0.13314	0.261451	0.231153
SLC13A1	-0.102752614	-0.094106986	-0.01017	-0.01166	0.081724	-0.14923
SLC13A2	-0.039565696	-0.109797171	-0.03721	0.005545	0.004681	-0.1553
SLC13A3	-0.061298252	-0.085049441	-0.04843	0.030786	-0.07469	0.043172
SLC13A4	-0.029965785	-0.054528103	0.026512	-0.00196	-0.04624	-0.19565
SLC13A5	-0.034994427	-0.013263188	-0.04797	0.011839	-0.02194	0.017621
SLC14A1	-0.011611385	0.158963481	0.196121	-0.00305	0.063616	-0.21624
SLC15A1	-0.023511991	-0.007075767	-0.0057	-0.00464	0.00431	-0.0611
SLC15A2	-0.023244181	-0.19373575	-0.26507	-0.01177	0.160462	0.093103
SLC15A3	0.010811148	-0.126508388	-0.44319	-0.08791	0.244739	0.494678
SLC15A4	0.036818361	0.162654451	-0.2881	0.008652	0.033278	-0.16535
SLC16A1	0.010113161	-0.041181169	0.010206	0.11224	-0.33261	0.086494
SLC16A1-/	0.013402894	-0.131535933	0.072282	0.163486	-0.16637	-0.11505
SLC16A10	-0.000401913	-0.095455903	0.443962	-0.03053	0.155115	-0.19792
SLC16A11	-0.029186529	-0.02908627	0.106225	-0.01123	-0.07082	-0.16041
SLC16A14	-0.014473009	-0.038143229	-0.02925	-0.01821	0.034493	-0.15179
SLC16A2	-0.032051168	-0.069493976	0.015114	0.00828	-0.02424	0.049526
SLC16A4	-0.048697785	-0.045450642	-0.05945	-0.00746	0.021304	-0.03347
SLC16A5	-0.007416502	-0.133751417	-0.2284	-0.09889	0.321839	0.464125
SLC16A6	0.012173356	0.117756919	-0.12036	-0.01067	0.033876	0.406975
SLC16A7	0.034243008	0.107694798	0.156813	0.005294	0.09905	0.037035
SLC16A8	-0.034407381	-0.030999268	-0.0262	-0.00887	-0.0201	-0.01335

SLC16A9	-0.032516186	-0.062602056	0.13847	0.014046	-0.01729	-0.07609
SLC17A1	-0.094749268	-0.082346923	-0.05598	-0.02133	0.028916	-0.03355
SLC17A2	-0.027052375	-0.007821578	-0.00766	-0.00071	0.014071	-0.00707
SLC17A3	-0.023453146	0.029120949	0.084373	0.021394	-0.04699	-0.0803
SLC17A4	-0.059091763	-0.015258486	-0.04508	-0.00869	0.034537	-0.03633
SLC17A5	0.017958731	0.073920026	-0.07367	-0.05655	0.122224	0.274762
SLC17A6	-0.036327726	-0.101599617	0.06878	-0.00473	0.036408	-0.11898
SLC17A7	-0.026458076	-0.04404964	0.049223	0.007522	-0.09796	-0.05599
SLC17A8	-0.092942309	-0.092070808	0.044635	-0.03559	0.065382	0.019654
SLC17A9	-0.028656035	-0.115944605	0.076583	-0.02282	-0.02564	-0.13939
SLC18A1	-0.038612705	-0.029215071	-0.08448	-0.00431	0.040599	0.148078
SLC18A2	-0.011585998	-0.110888514	0.023973	-0.04886	0.145533	-0.21163
SLC18A3	-0.033679316	-0.078918082	0.034864	0.037839	-0.08612	0.005098
SLC18B1	0.010325806	-0.131430571	0.044094	-0.06948	0.20465	0.124858
SLC19A1	-0.040806646	-0.051554489	-0.08285	0.017285	-0.01952	0.132868
SLC19A2	-0.004242599	0.038852669	-0.0135	0.065144	-0.16956	0.039715
SLC19A3	-0.056487752	-0.092693945	-0.02162	-0.01645	0.076384	-0.04796
SLC1A1	-0.037272203	0.016840168	-0.07682	-0.03972	0.05323	0.046714
SLC1A2	-0.041065486	-0.051736173	-0.00959	-0.01946	-0.00992	-0.0228
SLC1A3	-0.018818349	-0.055647172	-0.03855	-0.01592	0.029556	0.163389
SLC1A4	0.020793158	0.047352851	-0.14289	0.100116	-0.24409	0.059857
SLC1A5	0.008027654	0.004112379	-0.15179	0.056302	-0.19653	0.066554
SLC1A6	-0.038503925	-0.065032092	0.072473	-0.04467	0.061644	-0.0568
SLC1A7	-0.014115837	0.332555608	-0.0137	-0.05054	0.183257	-0.16298
SLC20A1	0.043917441	0.180630295	0.026616	0.033931	-0.06329	0.001362
SLC20A2	-0.013713804	0.103076019	0.018435	-0.00128	0.022441	-0.03948
SLC22A1	-0.013372079	0.018371834	-0.06646	0.005766	-0.0159	-0.07444
SLC22A11	-0.031821966	-0.025436884	-0.01202	0.037622	-0.09095	0.140717
SLC22A12	-0.025588621	-0.081339994	-0.11139	0.018475	-0.07316	0.086027
SLC22A13	-0.034138843	-0.057458439	0.104815	-0.00935	0.038579	-0.08939
SLC22A14	-0.012809033	-0.056660554	0.050711	-0.00759	-0.07018	-0.02376
SLC22A15	-0.004210825	0.032879673	-0.28902	-0.11491	0.321925	0.498831
SLC22A16	-0.023074495	-0.111873841	-0.13688	-0.07505	0.221738	0.657639
SLC22A17	-0.034858414	-0.11903648	0.269043	-0.01751	0.063473	-0.09949
SLC22A18	0.010656644	-0.042870614	-0.18282	-0.01794	0.042867	0.324431
SLC22A18/	-0.008417254	-0.07412459	-0.10027	-0.06057	0.066438	0.262728
SLC22A2	-0.033824633	0.009181451	0.041895	0.020145	-0.04634	0.01951
SLC22A23	-0.064950902	-0.109749231	0.024145	-0.05658	0.017771	-0.06846
SLC22A24	-0.029847743	-0.026813787	-0.01844	-0.00549	0.024755	-0.17719
SLC22A25	-0.03434585	-0.071616645	0.022808	-0.02356	-0.0377	-0.10414
SLC22A3	0.002371907	-0.050539701	0.04911	0.00119	0.018313	-0.22365
SLC22A31	-0.004052631	-0.109680958	-0.01725	-0.02028	-0.04541	-0.14542
SLC22A4	-0.00454684	-0.09800893	-0.28253	-0.06089	0.164564	0.419434
SLC22A5	-0.015254093	-0.051322751	0.085519	-0.0106	0.05981	0.233504
SLC22A6	-0.042227929	-0.097875418	0.023018	0.001169	0.020655	0.10824
SLC22A7	0.026712879	-0.114323271	-0.05832	0.061171	-0.1194	-0.03442
SLC22A8	-0.040826049	-0.033773226	0.116077	0.023395	-0.07451	-0.13341
SLC22A9	-0.031694413	-0.067167818	0.020598	0.008315	-0.04881	-0.16545
SLC23A1	-0.013150843	-0.04747532	-0.08095	0.02133	-0.03433	-0.16285
SLC23A2	-0.010491763	-0.034076569	-0.11969	0.021661	-0.06949	0.051329
SLC23A3	-0.04196546	-0.062617168	-0.00937	-0.0272	0.021118	-0.07067

SLC24A1	0.017478374	0.122915515	0.043736	0.125047	-0.01093	0.017483
SLC24A2	-0.04786925	-0.087888296	0.069793	0.02241	-0.05626	-0.14899
SLC24A3	-0.016292448	-0.042788175	-0.09393	-0.19867	0.546252	-0.06763
SLC24A4	0.000372728	-0.114347521	-0.25919	-0.08162	0.296714	0.664346
SLC25A1	0.009927676	-0.026867615	-0.00353	0.025459	-0.14585	0.0987
SLC25A10	-0.016145596	-0.05767829	0.018852	0.09876	-0.32495	0.128955
SLC25A11	0.022399707	0.049772176	-0.13279	0.064505	-0.20603	0.273992
SLC25A12	0.017369278	0.096295753	0.043286	0.067982	-0.16808	-0.00254
SLC25A13	-0.012239688	-0.003523447	-0.17354	0.015073	0.107089	0.466379
SLC25A14	0.016388709	-0.001632471	-0.08962	0.008818	-0.07148	-0.06642
SLC25A15	-0.002384238	0.078503214	0.294782	0.081821	-0.2196	-0.2061
SLC25A16	-0.011683788	0.068696569	-0.02844	-0.00163	0.021282	0.098287
SLC25A17	-0.004868125	-0.008058881	0.049683	0.052521	-0.23043	-0.18693
SLC25A18	-0.032613634	-0.03206924	0.017526	0.000296	0.021379	-0.01871
SLC25A19	0.021360754	-0.023119416	-0.09666	0.048085	-0.17263	1.32E-05
SLC25A2	-0.023694933	-0.029264021	0.066101	0.006569	-0.02394	-0.16926
SLC25A20	0.025126547	0.129184634	-0.06399	0.035719	-0.11768	0.007825
SLC25A21	-0.02788276	-0.041446582	-0.00915	0.146315	-0.07133	-0.00689
SLC25A21-	-0.062222183	-0.025846625	-0.07835	-0.07831	0.2061	0.090543
SLC25A22	-0.010373343	-0.007534035	0.052403	0.035877	-0.20868	-0.11914
SLC25A23	-0.016102713	-0.00188847	0.373311	-0.00582	0.019074	-0.32146
SLC25A24	0.014709874	-0.035845835	-0.14779	-0.03955	0.10484	0.245121
SLC25A25	0.015983356	-0.036501198	0.15687	-0.01745	-0.02002	0.06423
SLC25A26	0.033446167	0.023285571	0.26717	0.055541	-0.17579	-0.09734
SLC25A27	-0.01135236	-0.130031334	0.06678	0.066058	-0.01262	-0.08004
SLC25A28	0.023366527	-0.077001938	-0.07618	-0.05081	0.140742	-0.10089
SLC25A29	-0.010832448	-0.109261879	-0.12514	-0.023	0.064469	0.121865
SLC25A3	0.056787326	0.088263543	0.021397	0.085335	-0.18062	0.184344
SLC25A30	-0.043264584	-0.050057043	0.03143	0.048559	-0.12605	0.027615
SLC25A31	-0.115474458	-0.056314685	-0.05078	-0.01174	0.081796	0.025803
SLC25A32	0.021771603	0.050559524	0.115402	0.065773	-0.20379	-0.18775
SLC25A33	0.020406068	0.004003319	0.097433	0.068667	-0.19443	-0.04284
SLC25A34	-0.031432662	-0.173249147	0.054404	-0.01058	-0.00588	0.002507
SLC25A35	0.001995125	-0.051089573	0.076102	-0.01539	-0.02823	-0.03267
SLC25A36	0.047360626	0.07009425	0.116367	0.043316	-0.02582	-0.04207
SLC25A37	-0.000565966	-0.120099832	-0.14861	-0.02955	0.09136	0.092097
SLC25A38	0.027731109	0.087457808	0.373059	0.029776	-0.11045	-0.49033
SLC25A39	0.018752409	0.023560985	0.161937	0.072963	-0.22091	-0.0393
SLC25A3P1	-0.036538276	-0.031662383	0.150733	0.027504	-0.07099	-0.11686
SLC25A4	-0.00346578	0.035549126	0.232009	0.039585	-0.13646	-0.27489
SLC25A40	0.017268877	-0.015580276	-0.17476	0.017048	-0.06962	0.342834
SLC25A41	-0.023342998	-0.056034342	-0.01975	0.017023	-0.01669	0.00975
SLC25A42	-0.037480571	0.027402334	0.000137	-0.02078	0.065967	-0.02719
SLC25A43	0.003702124	0.052255708	-0.12472	-0.09079	0.265496	0.224497
SLC25A44	0.040481877	0.044145544	-0.28347	0.054176	-0.13347	0.101516
SLC25A45	0.0049421	-0.002502948	0.221087	-0.02437	0.077742	-0.23078
SLC25A46	0.036396215	0.064018135	0.084439	0.048446	-0.12232	0.033121
SLC25A47	-0.02502272	-0.048539187	-0.13695	0.011164	-0.03051	-0.07174
SLC25A48	-0.030464324	-0.050417565	0.016636	0.016537	-0.08004	-0.0437
SLC25A5	0.06758176	0.074427039	-0.05461	0.09226	-0.21019	0.236629
SLC25A5-/	-0.037747227	-0.034983623	0.185836	0.03746	-0.05868	-0.02276

SLC25A51	-0.01440844	-0.029970713	0.100233	0.024339	-0.17089	-0.05252
SLC25A53	-0.018705615	0.226012937	0.221038	0.046804	-0.12096	-0.26824
SLC25A6	0.031665615	0.004237988	0.11896	0.008992	-0.01804	0.262879
SLC26A1	0.028505268	-0.003733267	-0.08373	0.059274	-0.09935	-0.04426
SLC26A10	-0.034641597	-0.021199113	-0.07377	-0.00463	0.004456	-0.03119
SLC26A11	0.025147223	0.038645675	0.344958	-0.01269	0.035279	0.093029
SLC26A2	-0.006020344	0.126643688	0.112159	-0.021	-0.05005	0.088564
SLC26A3	-0.082809205	-0.085938539	0.082534	-0.00324	0.01024	-0.12765
SLC26A4	-0.027426005	-0.021909993	0.002249	0.248193	-0.30209	-0.02489
SLC26A4-/	-0.0301324	-0.019451129	-0.09051	0.021054	-0.08252	-0.06987
SLC26A6	-0.003674458	-0.063979106	-0.03727	0.008687	-0.01902	-0.08762
SLC26A7	-0.061474587	-0.099180514	-0.00503	-0.09415	0.039233	0.061545
SLC26A8	-0.015119361	-0.044947777	-0.1725	-0.00498	0.004851	-0.00233
SLC26A9	-0.031253793	-0.04560744	-0.12519	0.033327	-0.07942	-0.05393
SLC27A1	0.010089628	-0.028316952	-0.03921	-0.07007	0.20841	0.475127
SLC27A2	-0.009840247	0.008732977	-0.00319	0.153148	-0.31453	-0.04852
SLC27A3	0.017547072	0.11154149	-0.18199	-0.02584	0.079099	0.386422
SLC27A4	-0.012025525	0.007884864	-0.1254	0.065467	-0.26625	0.019438
SLC27A5	-0.012094413	-0.079347118	0.311765	0.063749	-0.25841	-0.25368
SLC27A6	-0.043965804	-0.048502632	-0.12275	-0.02491	0.104609	-0.08474
SLC28A1	-0.038144637	-0.047534736	-0.05703	0.037565	-0.11548	-0.09494
SLC28A2	-0.042964534	-0.061749599	0.148688	0.025176	-0.03779	-0.03814
SLC28A3	-0.047514563	-0.050631608	-0.06139	-0.00387	0.023288	0.316711
SLC29A1	-0.009843249	-0.041276612	-0.09315	0.036083	-0.14039	-0.02425
SLC29A2	0.004349907	-0.143460734	-0.02365	0.039393	-0.01051	-0.04677
SLC29A3	0.019551572	-0.077499371	-0.12471	-0.02998	0.066672	0.181902
SLC29A4	-0.029212541	-0.055517551	0.021066	-0.01279	-0.0386	-0.04532
SLC2A1	-0.004065014	-0.011708723	-0.05508	0.038693	-0.14677	-0.28358
SLC2A1-AS	-0.028397875	-0.10104507	-0.03611	0.000648	-0.01954	-0.07242
SLC2A10	-0.029904821	0.125431354	0.085855	-0.0123	0.047069	-0.1242
SLC2A11	0.007296709	-0.064077432	0.193172	0.028574	-0.00058	-0.27249
SLC2A12	-0.041153153	-0.005245642	-0.00014	-0.00709	0.003844	-0.05628
SLC2A13	-0.049132802	0.003343865	0.041927	-0.03773	-0.03704	0.19452
SLC2A2	-0.049450454	-0.025790234	0.029268	-0.00493	-0.00029	-0.03295
SLC2A3	0.043817839	0.070964925	-0.0509	0.039886	-0.08546	0.036281
SLC2A4	-0.042069624	-0.075673416	0.01424	0.013022	0.000285	0.008365
SLC2A4RG	0.017128748	-0.021886112	0.341836	0.001239	0.081292	-0.13283
SLC2A5	0.032268623	-0.138309734	-0.02525	-0.01605	0.103526	-0.09437
SLC2A6	0.012955089	-0.128590195	-0.25732	-0.08099	0.227961	0.392866
SLC2A8	0.000103676	-0.024932545	-0.10191	0.013189	-0.05516	-0.08035
SLC2A9	-0.016996132	-0.112142739	-0.24943	-0.0463	0.079817	0.312542
SLC30A1	0.000176046	-0.068490936	-0.22526	-0.03097	0.084366	0.427496
SLC30A10	-0.037019661	0.031778048	-0.02698	0.011974	-0.02489	0.00897
SLC30A2	-0.031651618	-0.067165159	0.085536	0.007389	-0.05925	-0.0688
SLC30A3	-0.000941421	-0.036094424	-0.00473	-0.00741	-0.08123	-0.12449
SLC30A4	-0.054122997	0.061983188	-0.01515	-0.03752	0.013229	-0.12162
SLC30A5	0.069638553	-0.035398618	-0.04084	0.107028	-0.13734	-0.11163
SLC30A6	0.044410644	0.062159499	-0.02958	0.049665	-0.11911	-0.05982
SLC30A7	0.022867697	0.086250324	0.042935	-0.02415	0.04073	0.194126
SLC30A8	-0.0465993	-0.086379466	0.045011	0.018502	-0.02224	0.00833
SLC30A9	0.014593504	-0.014391209	-0.02796	0.103489	-0.28544	0.170859

SLC31A1	0.038982336	0.016294413	-0.38402	-0.02919	0.022823	0.536475
SLC31A2	0.016498246	-0.09353344	-0.33452	-0.05909	0.171226	0.549183
SLC32A1	-0.019283808	-0.088564529	-0.06086	0.00362	0.005443	-0.13212
SLC33A1	0.005823674	0.060323988	-0.01202	0.066004	-0.14598	0.052731
SLC34A1	-0.046651345	-0.022575333	-0.1394	0.000191	0.015023	-0.02254
SLC34A2	-0.04712073	-0.017502875	-0.07183	-0.01422	0.067797	-0.00725
SLC34A3	0.012666353	-0.046440148	-0.00655	-0.00478	-0.02029	-0.07983
SLC35A1	0.034272064	0.015945808	-0.11437	-0.05048	0.143175	0.417192
SLC35A2	-0.027117584	-0.053383784	-0.21409	-0.01442	0.031852	0.324221
SLC35A3	0.031597351	0.069286971	0.058495	0.021579	-0.00204	0.077435
SLC35A4	0.061395769	0.077943637	0.119483	0.059993	-0.2068	-0.02599
SLC35A5	0.033559143	0.017309018	-0.33045	-0.00412	0.009651	0.277502
SLC35B1	0.045650668	0.110798984	-0.12661	0.064818	-0.18804	0.365833
SLC35B3	0.018154072	0.051583008	-0.02349	-0.00417	0.020256	0.154571
SLC35B4	0.01884092	0.025178193	0.013256	0.028052	0.006953	-0.01469
SLC35C1	0.010399089	-0.082869332	-0.04785	-0.00929	-0.09234	-0.00081
SLC35C2	0.018970652	-0.067209764	0.116649	-0.01342	-0.04966	-0.22417
SLC35D1	0.010849828	0.122753291	0.122306	0.068938	-0.1769	-0.2817
SLC35D2	0.003059215	-0.019691072	0.087412	-0.05094	0.191394	0.138339
SLC35D3	-0.050640198	0.016428628	0.011452	-0.13527	0.391146	0.092253
SLC35E1	0.060120833	-0.02358028	-0.19836	-0.02141	-0.05469	0.128658
SLC35E3	0.016732837	-0.012394949	-0.16814	0.008463	-0.05914	-0.0751
SLC35F1	-0.024220885	-0.075171788	0.048296	-0.01284	0.004157	-0.0101
SLC35F2	-0.004952922	-0.050196554	0.100947	0.079074	-0.24441	-0.22611
SLC35F3	-0.013324396	-0.062080203	-0.07796	0.049025	-0.12016	-0.04698
SLC35F4	-0.043764305	0.031208392	-0.02835	-0.01456	0.058868	0.05144
SLC35F5	-0.050949413	0.017444426	-0.1327	0.006774	-0.08235	0.039716
SLC35F6	0.001489437	-0.11093499	0.029827	-0.00122	-0.10053	0.005379
SLC35G1	-0.015201473	-0.065670579	0.159425	-0.02769	0.102325	-0.04583
SLC35G2	-0.021921184	0.007966106	0.052884	0.0207	-0.0575	-0.08337
SLC35G3	-0.025187367	-0.05970671	0.284298	-0.02091	0.073637	-0.11415
SLC36A1	0.00506192	-0.050802647	-0.19821	-0.00984	-0.02832	0.250336
SLC36A4	0.008682926	-0.081328728	-0.15786	-0.00656	0.037074	0.586003
SLC37A1	-0.001381099	-0.086075009	-0.11457	-0.0651	0.178843	-0.16756
SLC37A2	-0.001891351	-0.052619154	-0.16621	-0.14425	0.396801	0.487122
SLC37A3	0.01186333	0.018824975	0.12463	0.05954	-0.16191	-0.01958
SLC37A4	-0.003457636	-0.038214156	0.154409	0.092162	-0.3188	-0.13566
SLC38A1	0.010864152	0.101832385	0.314457	0.022871	-0.06463	-0.70364
SLC38A10	-0.062305419	-0.048795868	-0.10758	-0.04452	0.002274	0.054068
SLC38A11	-0.01048521	-0.085391052	-0.06685	0.002616	-0.00395	-0.13011
SLC38A2	0.053101606	0.157780899	-0.07064	0.046162	-0.1062	-0.27244
SLC38A3	-0.034344726	-0.078776376	-0.05504	0.002741	-0.01143	-0.05933
SLC38A4	-0.068875551	-0.065197938	0.057594	0.007336	-0.03239	-0.01721
SLC38A5	0.000809193	0.089160434	0.121704	0.104997	-0.33281	-0.24376
SLC38A6	0.012325978	-0.077028565	-0.03418	0.047646	-0.11643	0.385376
SLC38A7	-0.005967635	-0.035620368	-0.0183	-0.01933	0.054025	0.514653
SLC38A9	0.00844366	0.030742354	-0.048	0.015242	-0.03386	0.094194
SLC39A1	0.01219441	-0.068384411	-0.22553	0.052595	-0.2193	0.254532
SLC39A10	0.023276651	0.070954456	0.106816	0.022473	-0.04991	-0.16727
SLC39A11	0.014893824	0.012417643	-0.1424	-0.03811	0.082913	0.24248
SLC39A12	-0.022765308	-0.043651943	0.00857	0.041242	-0.10621	-0.03799

SLC39A13	0.009990104	-0.01180767	0.253545	-0.01423	-0.01193	-0.16811
SLC39A14	-0.019566525	-0.035924392	-0.0241	0.119214	-0.27268	0.015834
SLC39A2	-0.030914938	-0.043538444	-0.08559	0.002035	-0.02696	0.104309
SLC39A3	-0.01382793	-0.053817629	-0.04909	0.016304	-0.10047	0.030481
SLC39A4	0.002641481	-0.0338204	-0.0815	-0.03517	0.119603	0.320547
SLC39A5	-0.024074636	-0.062293615	-0.03466	-0.01914	-0.0421	-0.08064
SLC39A6	0.021722078	0.026732156	-0.06281	0.035652	-0.19034	-0.07924
SLC39A7	0.008707834	0.081618084	-0.13374	0.108215	-0.34948	0.115475
SLC39A8	-0.026909443	-0.034919457	0.036659	-0.10346	-0.08136	0.026478
SLC39A9	-0.050377303	-0.0301491	-0.05102	0.045413	-0.14304	-0.082
SLC3A1	-0.06253853	-0.033245798	0.048942	-0.03874	0.120319	-0.01958
SLC3A2	0.026030006	0.01955733	0.030472	0.058097	-0.17804	0.090233
SLC40A1	0.005417548	-0.160572208	-0.19608	-0.1115	0.308502	0.238955
SLC41A1	0.007167024	0.153184827	0.311324	0.063358	-0.1698	-0.2737
SLC41A2	-0.013803815	-0.056531648	-0.10395	0.077705	-0.1621	-0.14742
SLC41A3	0.02642037	-0.024107963	0.172871	0.01325	-0.06861	-0.1148
SLC43A1	0.007776655	0.143218345	0.235474	-0.02928	0.037163	0.05018
SLC43A2	0.002009631	-0.135081805	-0.3328	-0.07556	0.193916	0.399735
SLC43A3	0.004815875	-0.058904641	-0.14067	0.032211	-0.10198	0.315474
SLC44A1	-0.020824115	-0.05401455	-0.20123	-0.05734	0.179309	0.53442
SLC44A2	0.0217345	0.05606066	-0.04123	-0.05213	0.147863	-0.24241
SLC44A3	-0.015717592	-0.008795702	0.051558	-0.00629	0.021136	-0.0332
SLC44A4	-0.046242856	-0.06862745	-0.0392	-0.00562	0.05652	0.038726
SLC44A5	-0.049051853	0.030532362	-0.0101	0.001374	0.004744	-0.08981
SLC45A1	-0.039446844	-0.031295181	-0.04425	0.014627	-0.00576	-0.0201
SLC45A2	-0.054595287	-0.081448704	0.066777	0.002248	-0.01735	-0.1205
SLC45A3	-0.033217141	-0.110968837	-0.09814	-0.0435	0.089382	-0.13075
SLC45A4	0.008275196	-0.027298343	-0.17961	-0.0229	0.060855	-0.04138
SLC46A1	-0.016312212	-0.077045553	0.037321	-0.00223	0.025549	0.158983
SLC46A2	-0.002480039	-0.078134863	-0.26981	-0.09771	0.291237	0.777388
SLC46A3	0.018678591	0.007441526	0.046154	-0.06622	0.201936	-0.01208
SLC47A1	-0.003942469	-0.068952185	-0.09982	-0.03741	0.116823	0.003481
SLC47A2	-0.031301125	-0.076247124	-0.13168	0.004208	-0.00057	-0.10677
SLC48A1	-0.057446206	-0.170817708	-0.01075	0.160444	-0.18207	0.098742
SLC4A1	-0.035404902	-0.052036743	-0.00055	-0.04806	0.068001	-0.08813
SLC4A10	-0.074829928	-0.114758399	0.081273	-0.00344	-0.01213	-0.08686
SLC4A11	-0.039340685	-0.080508897	0.063733	0.015965	-0.0441	0.009669
SLC4A1AP	0.073847448	0.163582336	-0.08103	0.025522	-0.12855	0.160439
SLC4A2	0.006628036	-0.09365609	-0.14131	0.052603	-0.20497	0.311458
SLC4A3	-0.006543091	-0.070065665	-0.12978	-0.0073	0.029171	-0.16717
SLC4A4	0.059314533	0.252944781	0.164363	0.051375	0.066576	-0.25494
SLC4A5	-0.032675951	-0.052753501	0.09586	0.001558	-0.05764	-0.02653
SLC4A7	0.023631992	0.033769738	0.156486	0.080574	-0.20791	-0.17576
SLC4A8	-0.066374865	0.040114116	-0.07368	0.074215	-0.18409	0.088715
SLC4A9	-0.049993487	-0.105051613	-0.03032	-0.00352	0.032834	0.037984
SLC50A1	0.030778504	-0.056400192	0.009803	0.08407	-0.27965	-0.06526
SLC51A	-0.042171087	-0.053432916	0.115137	-0.00506	0.039005	-0.02159
SLC51B	-0.037824794	0.047809022	-0.03096	0.013037	-0.01924	-0.0464
SLC52A1	-0.027509735	-0.013459252	-0.07313	0.016962	-0.03754	-0.07361
SLC52A2	0.018582927	0.038135706	-0.08635	0.056812	-0.19101	0.070028
SLC52A3	-0.036804421	-0.051352463	0.035046	0.030553	-0.15138	-0.02265

SLC5A1	-0.037341162	-0.054343373	-0.03552	0.012953	-0.04329	-0.09825
SLC5A10	-0.034777755	-0.052066802	-0.08631	0.00898	-0.05191	-0.02409
SLC5A11	-0.024404633	-0.036300799	0.027905	0.014037	-0.03963	0.07273
SLC5A12	-0.13910363	-0.167744634	-0.02768	0.022352	0.050702	0.013623
SLC5A2	-0.027739594	-0.055080666	0.030905	-0.01916	-0.03906	-0.08215
SLC5A3	0.004579797	0.111513057	0.042849	0.046734	-0.15614	-0.06347
SLC5A4	-0.036150725	-0.052829444	-0.01288	0.00932	-0.00467	0.051272
SLC5A5	-0.017819321	-0.092233418	0.039395	-0.02743	-0.03504	-0.08826
SLC5A6	0.025561807	-0.065217397	0.090971	0.060413	-0.20371	-0.11252
SLC5A7	-0.042131507	-0.040479906	0.041625	-0.01213	0.046651	0.017613
SLC5A8	-0.025835974	-0.109651763	0.113186	0.052585	-0.14392	-0.09074
SLC5A9	-0.036836767	-0.048715625	-0.17168	-0.00565	0.018497	0.074188
SLC6A1	-0.048428458	-0.061741822	0.038908	0.012619	-0.05925	-0.00299
SLC6A11	-0.043433621	-0.057662778	0.02504	-0.00648	0.053433	0.033922
SLC6A12	-0.010369674	-0.035293665	-0.11491	-0.04562	0.116647	0.249421
SLC6A13	-0.035971261	-0.057753461	-0.01573	-0.00747	0.017855	0.000823
SLC6A14	-0.082505484	-0.104789154	0.027465	-0.0248	0.069575	-0.13002
SLC6A15	-0.100459099	-0.08457611	0.133963	0.024846	0.033795	0.028876
SLC6A16	-0.012447711	0.035998964	0.047083	-0.01809	0.03443	-0.29796
SLC6A17	-0.018354786	-0.075620478	0.01449	-0.01846	-0.07307	-0.12594
SLC6A18	-0.031706352	-0.051060565	0.007118	0.01753	-0.02698	0.13771
SLC6A19	-0.026315183	-0.015654948	0.057527	-0.01256	-0.00448	0.069642
SLC6A2	-0.052468078	-0.038674966	-0.03466	0.058816	-0.10601	-0.00146
SLC6A20	-0.055566057	0.037806677	0.015262	0.003336	0.025458	0.037182
SLC6A3	-0.020255673	-0.075654172	0.076129	-0.0095	-0.03545	-0.02695
SLC6A4	-0.061055223	-0.014147937	0.013953	-0.17082	0.38517	-0.06968
SLC6A5	-0.049655751	-0.045064344	-0.00903	0.029474	-0.07792	-0.10098
SLC6A6	-0.066053406	-0.121529039	-0.14576	0.022465	-0.06874	-0.03228
SLC6A7	-0.032406993	-0.062281143	-0.00201	-0.00329	-0.0332	-0.05461
SLC6A8	-0.034982794	-0.023539412	-0.07332	-0.04485	0.032854	0.081653
SLC6A9	-0.035445843	-0.014098243	-0.03798	-0.00775	0.051956	0.038391
SLC7A1	-0.011463306	-0.031088837	-0.0346	0.046188	-0.179	-0.03798
SLC7A10	-0.035170541	0.010629354	0.027959	0.024094	-0.04799	-0.02289
SLC7A11	-0.014095965	-0.063441251	-0.07965	0.052755	-0.22936	-0.11983
SLC7A11-A	-0.032791272	-0.066057739	-0.00124	0.024416	-0.05965	0.004067
SLC7A13	-0.023483262	-0.056628071	0.025376	-0.01437	-0.00555	-0.05931
SLC7A14	-0.050820003	-0.049769487	0.040419	0.038866	-0.09165	-0.24108
SLC7A2	-0.037605861	-0.07596864	-0.10124	-0.00231	-0.066	0.083152
SLC7A3	-0.030527063	-0.101743217	0.240379	0.025296	-0.04933	-0.06156
SLC7A4	-0.040975535	0.003366715	0.077138	0.036467	-0.08482	-0.03455
SLC7A5	0.012791785	-0.014567581	0.004863	0.072482	-0.21723	-0.18949
SLC7A6	0.010050483	-0.045488134	0.235756	-0.01992	0.024248	-0.26892
SLC7A6OS	0.047607701	0.080547525	0.056739	-0.02837	0.049718	-0.09163
SLC7A7	0.014400702	-0.120616619	-0.38581	-0.0928	0.251618	0.620749
SLC7A8	-0.096360759	-0.111010573	0.101143	-0.06438	0.075936	-0.11429
SLC7A9	-0.023792771	0.030684259	0.052009	-0.01148	0.008045	-0.11521
SLC8A1	0.033383361	-0.047541784	-0.38063	-0.00285	0.167818	0.406703
SLC8A1-AS	-0.016489752	-0.049655977	-0.06238	0.152005	-0.20636	-0.04842
SLC8A2	-0.029861529	-0.050213238	-0.05332	0.001764	-0.03687	-0.04648
SLC8A3	-0.034882494	-0.043630154	0.045324	-0.07407	0.186911	-0.04474
SLC8B1	-0.003146458	-0.164917714	-0.02426	-0.08404	0.172691	0.024618

SLC9A1	-0.002270535	-0.087935311	-0.12849	0.038784	-0.14649	-0.12324
SLC9A2	-0.043061731	-0.059174083	-0.03033	0.001005	0.035852	0.131612
SLC9A3	-0.030674158	-0.095599493	-0.00736	0.012635	-0.00986	0.079494
SLC9A3R1	0.031306139	0.171361985	0.149905	-0.00224	0.006911	-0.19996
SLC9A3R2	-0.03053067	-0.019369466	-0.07618	-0.01634	0.063529	0.081843
SLC9A5	-0.042526305	-0.045870291	-0.00613	0.041536	-0.07484	-0.11181
SLC9A6	0.0349629	0.066363458	0.125934	-0.00143	-0.00348	0.070247
SLC9A7	-0.007134961	-0.11999943	-0.19796	0.020911	-0.05859	-0.17944
SLC9A8	0.009485391	-0.03780211	-0.01677	0.010792	-0.05586	-0.09281
SLC9A9	0.011342592	-0.013258877	-0.05248	-0.035	0.106076	0.277274
SLC9B2	-0.006612518	0.010348465	0.184672	0.067501	-0.21946	-0.34678
SLC9C2	-0.059082693	-0.058327487	-0.07379	0.017576	-0.04834	-0.04563
SLCO1A2	0.004762565	-0.040621906	-0.05053	0.074928	-0.07961	-0.02007
SLCO1B1	-0.040502508	-0.053031842	-0.00125	0.004408	0.004882	0.000694
SLCO1B3	-0.05488386	0.014050609	0.134011	0.003637	0.027619	-0.04216
SLCO1C1	-0.035040079	-0.103109895	0.012344	-0.0107	0.057556	-0.0138
SLCO2A1	-0.038678379	0.065663655	-0.07501	-0.01738	0.064762	0.061207
SLCO2B1	-0.052158378	-0.010571256	-0.02856	0.041234	-0.28278	-0.09578
SLCO3A1	0.009381343	0.030106741	-0.14724	-0.05816	0.174647	0.263358
SLCO4A1	-0.012919717	-0.023354773	-0.02081	0.091475	-0.30149	-0.02382
SLCO4A1-/	-0.032363159	-0.057251346	0.029551	0.000183	-0.05603	-0.08233
SLCO4C1	-0.010669691	0.298690917	-0.10095	-0.0763	0.210052	-0.0869
SLCO5A1	-0.025479865	-0.060717498	-0.06385	-0.02335	0.012577	-0.05791
SLCO6A1	-0.036148844	-0.092049423	0.013678	0.023054	-0.04381	-0.01368
SLFN11	0.020986006	-0.014357087	-0.08491	0.015461	-0.01759	0.375194
SLFN12	0.008255788	0.095809318	-0.1065	-0.02731	0.100026	0.392678
SLFN13	-0.003240987	0.286820588	0.021458	0.030532	-0.08157	-0.21928
SLFN5	0.007189335	0.146254131	0.306218	-0.02073	0.058137	-0.12082
SLFNL1	-0.027868938	-0.019366285	0.037267	0.019941	-0.11368	-0.04016
SLFNL1-AS	-0.029126023	-0.041743513	0.009775	-0.00388	0.015048	0.07587
SLIRP	0.0411875	0.001572045	0.020949	0.097166	-0.28313	0.263633
SLIT1	-0.022534443	-0.113810551	-0.15715	0.01105	-0.00815	0.031512
SLIT2	-0.045353278	-0.091842185	0.00543	0.071511	-0.13992	-0.03161
SLIT2-IT	-0.033890799	-0.07280033	0.003042	-0.00423	0.021689	0.028555
SLIT3	-0.067295126	-0.150136195	-0.03498	0.026509	-0.06796	0.069659
SLITRK1	-0.05345048	-0.038541429	-0.03489	-0.00915	0.036204	-0.02588
SLITRK2	-0.037465533	-0.030256029	0.005901	0.020786	-0.03297	0.070702
SLITRK3	-0.044110326	-0.020509587	-0.04267	0.007079	0.011227	0.038599
SLITRK4	-0.016873332	-0.091967606	-0.27303	-0.07918	0.237378	0.716698
SLITRK5	-0.013042227	-0.054825312	-0.06849	-0.00589	0.041853	-0.14939
SLITRK6	-0.061021819	-0.00101032	-0.06128	-0.00062	0.019683	0.089225
SLK	0.040072788	0.151702334	-0.06894	-0.03972	0.109244	-0.07361
SLMAP	-0.018703636	0.084397513	-0.05054	-0.00241	-0.00641	-0.04839
SLMO1	-0.021671791	-0.053617273	0.004113	0.016411	-0.02834	0.01147
SLMO2	0.123946189	0.137322334	0.055503	-0.00901	-0.17129	-0.17453
SLN	-0.03618464	-0.08526817	-0.10724	-0.00373	0.002712	0.045323
SLPI	-0.003200267	-0.03602778	-0.01553	0.048154	-0.13712	-0.0709
SLTM	0.102071966	0.194666235	0.060419	-0.0084	0.057706	-0.23235
SLU7	0.073465019	0.123020532	-0.23882	0.020512	-0.03417	-0.05422
SLURP1	-0.033596141	-0.050502145	-0.084	0.033251	-0.1049	-0.03796
SLX4	-0.018280035	0.036131375	-0.07171	-0.00364	-0.11244	-0.03633

SLX4IP	0.012810387	-0.003866793	-0.03777	0.028596	-0.06556	0.030901
SMA4	0.021740917	0.009688321	0.09424	-0.06088	0.164862	-0.01092
SMAD1	-0.007925272	-0.048011797	-0.1838	-0.09298	0.265606	0.541003
SMAD1-AS1	-0.049338549	-0.039249402	0.070558	-0.00487	0.017411	0.060138
SMAD2	0.028829251	0.063326087	-0.00637	-0.0219	-0.00253	0.022555
SMAD3	0.0092888	-0.024236342	-0.1084	0.078406	-0.26449	-0.11816
SMAD4	-0.004060671	0.028244244	-0.0201	-0.01507	0.051031	-0.12716
SMAD5	0.017194082	0.163844662	-0.07477	0.034292	-0.02971	0.12313
SMAD5-AS1	-0.03756524	-0.019130404	-0.03511	0.023642	-0.04253	-0.04058
SMAD6	-0.063300753	-0.06019275	-0.04701	0.031469	-0.03098	0.161119
SMAD7	0.01223291	0.21118876	0.156113	0.006333	-0.02212	0.073022
SMAD9	-0.032270972	-0.046330114	-0.0006	0.023794	-0.03681	0.022833
SMAGP	0.002043293	0.107037376	0.217047	-0.08001	0.224274	-0.26014
SMAP1	0.067207283	0.146531259	0.008204	0.024229	-0.05618	0.168378
SMAP2	0.039184665	-0.08889625	-0.13064	0.020476	-0.04185	-0.03759
SMARCA1	-0.030386562	-0.068466923	0.038924	-0.00869	0.053657	-0.02733
SMARCA2	-0.004237809	0.076330851	0.111578	-0.04858	0.081551	-0.1294
SMARCA4	0.056874446	-0.021222513	0.179154	0.09752	-0.13962	0.154541
SMARCA5	0.046351136	0.13699285	-0.01237	0.082624	-0.24635	-0.22746
SMARCAD1	0.030967905	0.08443475	0.193196	0.029652	-0.13128	-0.22655
SMARCAL1	0.017519843	0.018619576	0.01715	0.011936	-0.02951	0.004011
SMARCB1	0.012605367	-0.101947485	-0.13072	0.030826	-0.09779	-0.02879
SMARCC1	0.037760882	0.105893127	0.173887	0.047212	-0.16628	-0.33796
SMARCC2	0.028959693	0.057639402	0.125586	0.05605	-0.08166	-0.25275
SMARCD1	0.007091246	-0.044827234	0.168851	0.078848	-0.27928	-0.2622
SMARCD2	0.058681495	-0.015214378	-0.20368	0.032346	-0.13133	0.122681
SMARCD3	-0.007533652	-0.13448433	-0.0846	-0.05384	0.169289	0.658253
SMARCE1	0.003425556	0.0523158	0.048342	-0.01777	-0.05466	-0.1469
SMC1A	-0.023550574	0.014021639	-0.00744	0.003902	-0.04945	0.237425
SMC1B	-0.054378147	-0.057997172	0.02896	-0.01778	0.092254	-0.08218
SMC2	0.001309704	0.099072582	0.009484	0.113252	-0.29964	-0.03849
SMC3	0.035275171	0.100921958	0.074964	0.050608	-0.12124	-0.07134
SMC4	0.017699692	0.108111807	-0.01819	0.126488	-0.36156	-0.31584
SMC5	0.029691593	0.034684246	0.051851	0.018801	-0.02784	0.078839
SMC5-AS1	-0.033933986	0.009500284	-0.07861	0.018641	-0.03646	-0.12778
SMC6	0.012758642	-0.01564192	-0.1023	0.034604	-0.09872	-0.22378
SMCHD1	0.0228824	-0.01444633	-0.0524	-0.01713	0.052099	-0.16169
SMCO3	-0.050388525	-0.052811808	-0.1301	-0.00377	0.028608	0.137043
SMCO4	0.014974236	-0.038242172	-0.33807	-0.02102	0.055817	0.535727
SMCP	-0.030286448	-0.122528889	0.029939	-0.00136	-0.02537	0.011186
SMCR5	-0.029422636	-0.003418718	0.105377	-0.01459	0.061865	-0.13251
SMCR8	0.060747837	-0.050432357	-0.06752	0.004364	0.118155	0.052365
SMDT1	0.024952497	0.002837332	0.181764	0.00451	0.000802	0.111857
SMEK1	0.033466679	0.105745156	0.04859	0.034854	-0.10449	-0.21337
SMEK2	-0.009396518	0.07468294	0.092486	0.051108	-0.06606	-0.14124
SMEK3P	-0.093291089	-0.067020547	-0.08725	-0.05925	0.121197	0.019987
SMG5	0.036836645	-0.034119612	-0.11008	0.036156	-0.17331	-0.01718
SMG6	-0.023938573	-0.034057754	0.030544	0.043443	-0.13708	-0.12246
SMG7	-0.02901568	-0.067421995	-0.01879	0.050941	-0.19405	-0.02327
SMG7-AS1	-0.056889206	-0.028045569	0.082307	-0.01704	0.086805	-0.06351
SMG8	0.035823082	0.056002785	-0.19017	0.043543	-0.14541	0.240319

SMG9	-0.010314637	-0.076093891	-0.04118	0.009835	-0.11661	0.097659
SMIM1	-0.026768737	-0.123904009	-0.00303	-0.01881	0.035617	0.075493
SMIM10	-0.05240363	-0.012900657	-0.05998	-0.01493	0.058266	0.110074
SMIM11	0.031044213	0.031453682	0.161205	-0.02638	-0.01575	-0.1452
SMIM12	0.045649131	0.07189057	-0.00176	0.074595	-0.18784	0.157617
SMIM13	-0.020798715	0.02631767	-0.08522	0.06896	-0.24087	-0.02574
SMIM14	0.023949589	0.013883261	-0.16211	-0.08492	0.262019	0.126572
SMIM15	0.034751746	0.103030841	0.03351	0.049572	-0.12096	0.211721
SMIM17	-0.046685155	0.05222833	-0.02552	-0.02026	0.056581	-0.03387
SMIM19	0.037265499	0.094041374	0.13833	0.037419	-0.11351	-0.00166
SMIM2	-0.033764128	-0.060098534	-0.08965	0.022484	-0.05507	-0.0968
SMIM2-AS	-0.042446647	-0.022985909	-0.01629	0.041564	-0.10704	-0.01444
SMIM20	0.02545475	-0.014782564	0.186396	0.008046	-0.00895	0.08965
SMIM21	-0.056082425	-0.038794417	-0.05396	-0.0033	0.015382	-0.1053
SMIM22	-0.036638597	-0.060542189	-0.03064	0.009179	-0.0096	-0.08087
SMIM24	-0.026108173	-0.013101814	-0.06725	0.023391	-0.09388	-0.14692
SMIM3	0.015782709	-0.031895873	-0.19778	-0.09614	0.242928	0.315467
SMIM4	0.008604807	0.02912782	-0.10013	0.020148	0.015008	0.129366
SMIM5	-0.00478902	-0.131072299	-0.10797	-0.09943	0.285834	-0.11619
SMIM6	-0.014785356	-0.060385931	-0.06097	-0.00243	0.037072	-0.09116
SMIM7	-0.018376927	-0.03034958	-0.04576	-0.07747	0.031401	0.249037
SMIM8	-0.023063253	0.094338213	0.053448	0.01977	-0.08524	-0.09423
SMKR1	-0.00496196	0.26115876	0.21002	0.034438	-0.09117	-0.23285
SMLR1	-0.072859445	-0.05011133	-0.1366	-0.01625	0.057899	0.16579
SMNDC1	0.102297731	0.115625147	-0.20242	0.091343	-0.23238	0.074966
SMO	-0.023737723	-0.036949595	-0.01472	-0.0198	-0.04504	-0.06123
SMOC1	-0.021245499	-0.088872265	-0.04015	-0.00578	-0.03633	-0.22286
SMOC2	-0.035275398	-0.03675908	0.004238	0.0044	-0.01747	-0.03716
SMOX	-0.02608044	-0.055397819	-0.04298	-0.05082	0.089188	0.014597
SMPD1	-0.092398721	-0.018746557	0.243754	-0.0759	0.014448	-0.01572
SMPD2	0.005947596	-0.056821958	0.0098	0.009501	-0.11407	-0.07556
SMPD3	-0.005423652	-0.068352245	-0.14666	-0.0085	0.015268	-0.23028
SMPDL3A	-0.006909009	-0.046903044	-0.22242	-0.08999	0.261574	0.615071
SMPDL3B	-0.029166834	-0.054258304	-0.04969	0.001614	-0.08243	-0.01115
SMPX	-0.045252182	0.025618661	-0.10693	0.033323	-0.08046	-0.06033
SMR3A	-0.05590347	-0.086484166	0.078459	-0.0089	0.032741	0.037693
SMR3B	-0.044594335	0.000126366	-0.01487	0.038935	-0.07385	-0.05412
SMS	0.034912391	-0.027909618	-0.13035	0.0743	-0.20997	0.210437
SMTN	0.009841006	-0.053664197	0.028005	0.106134	-0.42186	0.125734
SMTNL1	-0.032025472	-0.058012158	-0.10437	-0.0049	-0.00827	-0.00983
SMTNL2	-0.034948925	-0.063767677	0.034711	-0.0091	0.043763	-0.01559
SMU1	0.0699042	0.026166979	-0.11475	-0.00035	-0.15368	0.024086
SMUG1	-0.007674009	0.016134011	-0.14832	0.021767	0.010241	0.285232
SMURF1	-0.027282138	-0.010409526	-0.01954	0.053388	-0.14088	0.004165
SMURF2	0.016558211	0.112218362	-0.00647	-0.03735	0.136981	-0.17317
SMYD1	-0.054990467	-0.119594954	0.018044	-0.0104	0.019497	-0.01096
SMYD2	-0.113558979	-0.078238347	0.233154	-0.00593	0.092594	-0.15301
SMYD3	0.008979037	0.095619426	0.188307	0.057002	-0.12559	-0.16828
SMYD4	0.022497176	0.061259849	0.234249	0.012979	-0.09593	-0.06601
SMYD5	-0.008825772	-0.069973675	0.193739	0.048889	-0.172	-0.1639
SNAI1	-0.026986219	-0.086800028	-0.10293	0.010155	-0.02644	-0.06276

SNAI2	-0.030857028	-0.070826754	-0.04	-0.02619	0.057531	0.021819
SNAI3	-0.010253631	0.055810509	-0.04345	-0.08466	0.228524	0.042514
SNAI3-AS1	-0.032431519	0.090066365	-0.16886	0.01635	-0.0294	0.067402
SNAP23	-0.004075011	-0.022712438	-0.31665	-0.04853	0.111655	0.079616
SNAP25	-0.068822968	-0.070987553	0.021491	0.125727	-0.07057	-0.0334
SNAP29	0.047840459	0.069527777	-0.17522	-0.06504	0.121995	0.133998
SNAP91	-0.021609554	-0.1201583	0.019788	0.002898	-0.00271	-0.06503
SNAPC1	0.009406859	-0.027950091	-0.08648	0.047447	-0.15044	0.061661
SNAPC2	0.003224637	-0.083917051	0.015581	0.012714	-0.08672	0.163132
SNAPC3	-0.000210627	0.038089468	-0.04105	0.002919	0.028441	0.194941
SNAPC4	0.00975497	-0.045811978	-0.06193	0.011471	-0.07231	0.022774
SNAPC5	0.042718458	0.116872573	0.258767	0.057229	-0.18191	-0.05716
SNAPIN	0.057036427	0.113135725	-0.06338	0.032636	-0.10998	0.319078
SNCA	0.000944017	-0.043531645	-0.29564	-0.20735	0.31364	0.408045
SNCAIP	-0.028774551	-0.012476782	-0.09781	0.035303	-0.03243	0.124813
SNCB	-0.030765735	-0.109518251	-0.06779	0.002414	-0.02356	-0.03309
SNCG	-0.03674732	-0.10552144	-0.09213	0.018742	-0.08198	0.035042
SND1	0.040804809	0.000404469	-0.01506	0.03563	-0.10591	0.172665
SND1-IT1	0.00480107	-0.022144088	0.044674	0.01634	-0.03985	-0.06642
SNED1	0.010001452	-0.045876126	0.129	0.036791	0.056447	0.034881
SNF8	0.074712632	0.045717026	-0.12722	0.109344	-0.30867	0.340368
SNHG10	-0.040624436	-0.020304129	-0.0846	2.49E-05	0.034746	0.096199
SNHG17	0.023950189	-0.030036065	0.208218	0.023247	-0.08126	-0.01185
SNHG18	-0.029178534	-0.108820906	0.131432	-0.0143	0.057079	-0.0674
SNHG19	0.011555363	-0.04887162	0.31304	0.035162	-0.08928	-0.09092
SNHG22	-0.01392759	0.174844902	0.128417	0.025664	-0.0575	-0.18837
SNHG4	-0.018651897	-0.040593202	0.104433	0.080144	-0.19528	-0.03162
SNIP1	0.009422918	0.128308463	0.020844	0.008611	-0.02744	-0.08561
SNN	0.011929668	-0.265750292	-0.19105	-0.13623	0.383396	0.279327
SNORA21	-0.006650463	-0.012897085	-0.01377	0.027785	-0.08427	-0.00813
SNORA37	-0.031230034	-0.066478935	-0.02205	0.003464	0.007845	0.171093
SNORA43	-0.026771862	-0.091612302	-0.02917	0.009107	-0.08795	-0.07342
SNORA65	-0.026919625	0.028280296	-0.0548	0.020211	-0.05542	-0.07662
SNORA68	-0.020868573	-0.022426723	0.021977	-0.00764	0.001367	0.055796
SNORA71A	-0.037360577	-0.06938701	0.066821	0.015621	-0.01984	0.157055
SNORA71B	-0.022172488	-0.093544506	0.167624	0.007022	-0.01951	-0.05463
SNORA72	-0.018654505	-0.064764974	0.137808	-0.04371	0.129837	-0.02772
SNORA74A	-0.043562845	0.015608422	-0.04967	-0.01637	0.000626	-0.07875
SNORA78	-0.015312068	-0.03863922	0.086889	-0.03022	-0.01684	-0.10538
SNORD114-	-0.058671116	-0.01181431	-0.03995	-0.02791	0.060899	-0.06763
SNORD8	-0.031471862	-0.029456722	0.026441	0.002487	-0.00463	-0.00157
SNORD89	0.019865132	-0.054314934	-0.19169	-0.03191	0.097392	0.169905
SNPH	-0.005590928	-0.088802013	0.401796	-0.04708	0.103977	-0.20464
SNRK	-0.003111119	0.019156291	0.103524	0.009776	0.028541	-0.07188
SNRK-AS1	-0.016548466	-0.005112785	0.10192	-0.0163	0.070948	0.0114
SNRNP200	0.033153275	0.036833257	0.339617	-0.02171	0.051583	-0.4667
SNRNP25	0.029434397	-0.105231485	-0.16621	0.065312	-0.1872	-0.09669
SNRNP27	0.072299773	0.120236107	0.09099	0.079385	-0.24034	-0.05983
SNRNP35	0.052022095	0.025727663	-0.06364	0.004667	-0.08191	-0.06007
SNRNP40	0.031661964	0.074786119	0.116547	0.095069	-0.29985	-0.07398
SNRNP48	0.026378977	0.065022447	-0.10962	0.083917	-0.20786	0.006926

SNRNP70	0.03658095	-0.081489374	0.214381	-0.03888	0.113397	-0.2255
SNRPA	0.032518565	-0.055300654	0.154082	0.065178	-0.1653	0.113277
SNRPA1	0.022142805	-1.31E-05	0.236405	0.083704	-0.11757	-0.11132
SNRPB	0.058350508	0.122800156	0.014713	0.123965	-0.32831	0.031464
SNRPB2	0.040677377	0.118396516	0.12181	0.043261	-0.10464	0.02905
SNRPC	0.068567931	0.052511541	0.150694	0.123485	-0.35457	0.024581
SNRPD1	0.025946804	0.029582156	0.10277	0.08886	-0.26927	0.045263
SNRPD2	0.033932376	0.06899191	0.235602	0.059338	-0.13641	-0.02822
SNRPD3	0.077380577	0.109729993	0.034743	0.101572	-0.27224	0.052877
SNRPE	0.040942631	0.009153572	0.07021	0.05466	-0.15695	0.054056
SNRPF	0.029429107	0.021862541	0.201845	0.069199	-0.18533	0.067661
SNRPG	0.06413056	0.085574016	0.074874	0.120316	-0.29741	0.041093
SNRPN	-0.039549212	-0.048581495	0.056212	0.018984	-0.0352	-0.0744
SNTA1	-0.006748063	-0.095230983	0.076825	0.017757	-0.09337	-0.24884
SNTB1	-0.036796881	0.060301779	-0.04384	0.023685	0.01558	0.267296
SNTB2	-0.033838645	0.282047448	0.105992	0.037574	-0.07763	-0.06693
SNTG1	-0.076272942	-0.053831904	0.070587	0.001714	-0.0381	-0.16054
SNTG2	-0.057956487	-0.143989059	0.269417	-0.01965	0.075299	-0.11919
SNTN	-0.062872215	-0.071789009	0.140795	-0.00721	0.035009	-0.02078
SNUPN	0.032217851	0.0186636	0.114626	0.01055	-0.04566	0.086542
SNW1	0.117470414	0.15953326	-0.14943	0.084876	-0.21614	-0.13184
SNX1	0.044625843	0.003194742	-0.19427	-0.07727	0.207717	0.248174
SNX10	0.029886462	-0.014609099	-0.4245	0.027572	-0.04408	0.348359
SNX11	0.045516629	0.012843527	-0.24505	-0.02227	0.035186	0.165488
SNX12	0.025153605	0.057110093	-0.04252	0.031999	-0.12696	0.192104
SNX13	-0.026321965	-0.046460397	-0.18144	-0.04446	0.038262	0.173143
SNX14	0.038447859	0.094494191	-0.04914	0.016805	-0.04306	0.151916
SNX15	0.020619117	-0.043986687	0.015448	4.02E-05	-0.04543	-0.05329
SNX16	-0.007213889	0.002561554	-0.15925	0.01884	-0.03447	0.208591
SNX17	0.062862113	0.037327222	-0.06489	0.020197	-0.04603	0.241129
SNX18	0.029716487	0.076237546	-0.35045	-0.01006	0.025255	0.202948
SNX19	0.009212979	0.058955589	-0.13689	-0.08738	0.260171	0.094596
SNX2	0.03718035	-0.112160179	-0.27785	-0.04942	0.157887	0.269942
SNX20	0.008240153	-0.027556394	-0.06574	-0.08497	0.17438	-0.08365
SNX21	-0.029883607	-0.095057727	-0.09711	-0.05112	0.172659	0.452701
SNX22	-0.012471276	-0.126841493	-0.11171	-0.0241	0.011426	-0.13112
SNX24	0.003324011	0.036468823	-0.02977	-0.10773	0.11013	0.322651
SNX25	-0.007149327	-0.032345926	-0.01782	0.063164	-0.21657	-0.21681
SNX27	0.03124212	-0.060178021	-0.23995	-0.03948	0.104423	0.433274
SNX29	-0.012497994	-0.096821201	-0.15242	-0.01188	0.044673	-0.13146
SNX29P2	-0.01574142	0.049131342	-0.03812	-0.03538	0.115811	0.055529
SNX3	0.065010331	-0.043581962	-0.2768	-0.01075	0.049604	0.009788
SNX30	0.010536027	-0.051756919	-0.15071	-0.01369	0.073457	0.355094
SNX31	-0.070618861	-0.062852444	0.003703	-0.02073	0.033163	-0.00916
SNX32	-0.045235497	-0.049031808	0.039235	0.000725	0.018316	0.069475
SNX33	-0.019833822	-0.036537871	-0.07784	-0.02102	-0.02095	0.225262
SNX4	0.033769125	0.046356717	-0.02345	0.058463	-0.17269	0.215198
SNX5	0.014603332	-0.005901817	-0.15374	0.052702	-0.15703	0.127155
SNX6	0.12103708	0.176364866	0.038939	0.026584	-0.03185	-0.24017
SNX7	-0.021341616	-0.073830146	-0.0118	0.152222	-0.20307	-0.00689
SNX8	-0.018476043	-0.091140479	-0.11799	-0.00683	-0.02755	-0.08822

SNX9	0.014800186	-0.083753932	0.094707	-0.06979	0.179839	-0.08996
SOAT1	0.030920487	0.091538919	-0.10779	0.017772	-0.08076	0.454395
SOAT2	-0.025262641	-0.156860926	0.216823	-0.01563	0.000631	-0.11486
SOBP	-0.051115847	-0.115042881	0.006428	0.000504	-0.17911	0.017021
SOCS1	0.003050851	0.01656975	-0.01182	0.120376	-0.46723	-0.01222
SOCS2	-0.005466778	0.138215836	0.087714	0.059635	-0.12362	-0.18565
SOCS2-AS	-0.021541852	0.138577245	0.069696	-0.00182	0.024735	-0.11498
SOCS3	0.002258641	-0.132614433	-0.00409	0.018145	-0.07717	0.007379
SOCS4	0.005196648	0.089314385	0.01059	0.073363	-0.22625	-0.06399
SOCS5	0.011153389	0.069204769	0.003667	-0.00568	0.024675	-0.03045
SOCS6	0.021227491	-0.015666611	-0.17398	-0.02159	0.095061	0.270687
SOCS7	0.00390025	0.051399152	0.094898	0.065849	-0.10005	0.083065
SOD1	0.039006141	0.057397888	0.26566	0.076555	-0.19236	-0.07236
SOD2	0.022452912	-0.113889143	-0.28616	-0.05143	0.154953	0.347976
SOD3	-0.01956349	-0.09418028	0.023169	0.029317	-0.13832	-0.03037
SOGA1	0.003841176	-0.079607734	-0.11049	-0.01073	0.102762	0.081576
SOHLH1	-0.032794741	-0.033290284	-0.02436	0.004666	-0.07559	0.004162
SOHLH2	-0.0257252	-0.004400221	0.046628	0.021023	-0.05208	-0.03309
SON	0.079567901	0.247343992	0.073808	0.117132	-0.11805	-0.39297
SORBS1	-0.032376821	-0.036434273	-0.07972	0.049054	-0.06661	0.072905
SORBS2	-0.061519833	0.190988047	-0.03222	-0.03817	0.091742	-0.0344
SORBS3	-0.009180971	-0.168608703	0.20036	-0.07517	0.135583	-0.09363
SORCS1	-0.029828157	-0.036627581	0.059501	0.064879	-0.16618	0.070917
SORCS2	-0.023431321	0.061079582	-0.00774	-0.01001	0.040508	0.027324
SORCS3	-0.051282578	-0.080874488	-0.01219	-0.04854	0.148456	-0.10944
SORCS3-AS	-0.032115729	-0.065623341	0.02598	0.002284	-0.01511	0.00511
SORD	0.044839502	-0.109706401	-0.2591	0.019809	-0.03322	0.140732
SORL1	0.012372104	0.067286995	0.016086	-0.09416	0.2669	-0.04833
SORT1	-0.001907958	-0.111156397	-0.29762	-0.05588	0.134304	0.548763
SOS1	0.021366939	0.075652995	0.017763	0.031477	-0.14559	-0.17671
SOS2	-0.022453453	0.064083521	-0.17525	-0.01664	0.056678	0.111367
SOST	-0.047676715	-0.007265047	-0.10842	0.021148	-0.05402	-0.01176
SOSTDC1	-0.040425863	-0.140477858	-0.08574	-0.01317	0.053423	-0.08839
SOWAHA	-0.020453318	-0.026216372	-0.03329	0.036964	-0.10055	-0.0528
SOWAHC	-0.009458809	-0.052174354	-0.1006	-0.10407	0.305133	0.586388
SOX1	-0.066027303	-0.051431209	-0.11942	-0.05267	0.020836	0.100309
SOX10	-0.031078179	-0.078646227	-0.03209	-0.00613	-0.04707	-0.0373
SOX11	-0.009105894	-0.049014656	-0.04465	0.043139	-0.01463	-0.12737
SOX12	-0.018746178	-0.146912039	0.058429	-0.0346	0.126859	-0.29124
SOX13	-0.024537275	0.171970507	0.210242	-0.03654	0.08966	-0.1604
SOX14	-0.033005281	-0.075037517	-0.07535	-0.04307	0.146027	0.044753
SOX15	-0.021921506	-0.098746602	-0.11434	0.01283	-0.11526	0.080299
SOX17	-0.067289875	-0.019955488	-0.02874	-0.00947	0.051637	0.042333
SOX18	-0.055722962	-0.069464328	-0.07363	0.023649	-0.08212	0.038679
SOX2	-0.015282508	-0.048202966	-0.02139	0.102288	-0.0322	-0.0028
SOX2-OT	-0.042538256	-0.037511732	-0.04308	0.222987	-0.11259	-0.00556
SOX21	-0.026599426	-0.022683522	-0.04388	0.024318	-0.04554	0.065333
SOX21-AS	-0.051895192	-0.055736981	0.081913	-0.00404	0.021254	-0.01944
SOX3	-0.04838286	-0.056645883	-0.03411	0.031639	-0.05391	0.162328
SOX30	-0.057925094	-0.048437263	-0.10637	-0.03786	0.088344	0.012584
SOX4	-0.011158468	0.002137214	-0.16388	-0.06669	0.128776	0.350736

SOX5	-0.016446693	-0.060965192	-0.03576	-0.01106	-0.08705	-0.07966
SOX6	-0.034393998	-0.033576139	-0.05442	0.004722	-0.00837	-0.12704
SOX7	-0.038527347	-0.082606923	0.020472	-0.00311	0.011844	-0.15672
SOX8	-0.008364257	-0.097982172	0.311849	-0.05141	0.165059	-0.0987
SOX9	-0.028930093	-0.020288296	-0.02527	-0.01994	0.04437	-0.04993
SOX9-AS1	-0.047288843	-0.076563521	-0.05794	0.002966	0.000623	-0.08001
SP1	-0.011757313	-0.029618832	-0.21607	-0.06896	0.071033	0.116512
SP100	0.018028242	0.076503414	-0.03224	-0.03871	0.077985	-0.07429
SP110	0.04106204	0.01752312	-0.07594	-0.03731	0.027545	-0.1903
SP140L	-0.014725428	-0.015318154	0.052265	0.005965	-0.00959	-0.07635
SP2	-0.037951824	-0.022217106	-0.02712	-0.03185	0.004623	-0.04968
SP3	0.012455465	-0.027229764	-0.11799	-0.01014	0.052856	-0.07982
SP3P	-0.044940691	-0.033878933	-0.06547	-0.0166	0.07205	0.07103
SP4	0.01040986	0.154808091	0.242303	0.014498	-0.05143	-0.48613
SP5	-0.031757082	-0.083471129	0.002913	0.032184	-0.0677	-0.0215
SP6	-0.031197067	-0.049229502	-0.0134	0.009682	-0.04085	-0.17356
SP7	-0.033571209	-0.014710122	0.049625	0.021655	-0.04934	0.031241
SP8	-0.050521971	-0.076009149	-0.09964	-0.03371	0.040613	-0.02863
SPA17	-0.014388599	-0.042546886	-0.03131	0.090674	-0.25454	0.444634
SPACA1	-0.045384515	-0.035547945	-0.00486	0.010156	-0.01033	0.109487
SPACA3	-0.026484747	-0.093420326	0.021706	0.049486	-0.11019	-0.06218
SPACA4	-0.031179191	-0.094776711	0.022693	-0.01368	-0.01039	-0.0327
SPACA6P	-0.024017884	-0.095584746	-0.08146	-0.005	0.00353	0.04245
SPACA7	-0.053740574	-0.070650206	0.012925	0.023513	-0.0285	-0.04022
SPAG1	-0.003797419	0.079305911	-0.11732	0.059873	-0.16912	0.003416
SPAG11A	-0.032688667	-0.087829104	-0.08589	-0.01188	-0.00988	-0.07597
SPAG16	-0.047833116	-0.049021089	0.021873	-0.00949	-0.02659	0.081447
SPAG17	-0.046166706	-0.080496584	-0.14061	0.007769	-0.00589	0.253637
SPAG4	-0.016340465	-0.026029798	-0.0794	0.030358	-0.09666	-0.00269
SPAG5	0.002630928	-0.025796037	-0.03087	0.155043	-0.47502	-0.05521
SPAG5-AS1	-0.029869653	-0.073649433	0.05747	-0.03915	0.105214	-0.07355
SPAG6	-0.032087944	-0.020488786	-0.03879	0.050797	-0.12846	-0.02592
SPAG7	0.094014655	0.030206674	0.130399	0.036239	-0.09782	0.1845
SPAG8	-0.024871704	-0.071263147	-0.02118	-0.00142	0.022767	-0.04849
SPAG9	0.008997163	0.067396051	-0.14812	0.015134	-0.05698	-0.00773
SPAM1	-0.027142715	0.062946853	-0.00865	0.033815	-0.1263	-0.07998
SPANXA2-(	-0.048296923	-0.016987455	-0.02325	0.007161	-0.01085	0.036501
SPANXB1	-0.041556046	-0.021470662	0.014785	-0.00536	0.024625	0.02029
SPARC	-0.004648639	-0.018405896	-0.0974	-0.21951	0.615815	0.19563
SPARCL1	-0.023190076	-0.024226982	0.002752	0.110328	-0.26211	-0.0028
SPAST	0.015848978	0.114608038	-0.29639	-0.0099	0.041949	0.177662
SPATA1	-0.040393077	-0.000105327	-0.10561	-0.04977	0.149876	0.249922
SPATA12	-0.035709481	-0.043885233	0.02986	0.014843	-0.0267	-0.02114
SPATA13	0.00590475	0.088882886	0.208611	-0.01344	0.053928	-0.12468
SPATA16	-0.032227091	-0.049684524	0.017556	-0.00071	-0.01144	-0.11682
SPATA17	-0.057092468	-0.097395084	0.033224	0.001946	0.008576	-0.04533
SPATA18	-0.074937943	-0.049507917	-0.10337	-0.00673	-0.00525	0.023923
SPATA19	-0.022762096	-0.019609278	0.006411	0.065973	-0.17675	-0.01383
SPATA2	-0.002636563	-0.034459953	-0.05144	0.040749	-0.15171	0.035737
SPATA20	0.019335858	-0.052787167	0.038675	0.0154	-0.02704	0.185831
SPATA21	-0.023333203	-0.1015034	-0.08348	-0.02632	0.001163	-0.05467

SPATA22	-0.045340692	0.022487481	-0.01569	-0.02775	0.021378	0.031092
SPATA24	0.026742842	-0.012923043	0.010196	0.100811	-0.25696	-0.06556
SPATA25	-0.025268432	-0.056320019	-0.18412	0.030067	-0.06474	-0.03132
SPATA2L	0.028124996	0.031737067	-0.1726	0.003404	-0.03304	0.025141
SPATA3	-0.030179689	-0.068710962	-0.04289	0.036342	-0.09536	-0.09162
SPATA3-AS	-0.035196656	-0.043397761	0.031116	0.026093	-0.08063	-0.06039
SPATA31C2	-0.03848864	-0.02767668	-0.0814	-0.01766	0.060231	0.02485
SPATA31E1	-0.047830645	-0.090063018	-0.06094	0.028801	-0.04334	0.051809
SPATA32	-0.005925474	-0.065379288	-0.00189	-0.02	-0.00963	-0.11662
SPATA33	-0.012320117	0.038316931	-0.00423	0.031307	-0.0482	0.150853
SPATA4	-0.039768959	-0.000623646	0.004142	-0.0065	0.025306	-0.08345
SPATA41	-0.032216359	0.00030317	0.065073	-0.01402	0.052789	-0.08582
SPATA42	-0.051208567	-0.092881884	-0.07767	0.019053	-0.04344	0.093203
SPATA45	-0.038845834	-0.05793064	-0.12843	0.009326	-0.01141	-0.03451
SPATA5	0.002564304	0.045920661	0.116107	0.143492	-0.33641	-0.07475
SPATA5L1	0.006556784	-0.053909041	-0.09417	0.022286	-0.13571	0.027641
SPATA6	-0.004411115	-0.139456317	-0.24949	-0.06616	0.244163	0.478495
SPATA6L	-0.057481472	0.018951891	-0.08768	-0.02962	0.088262	-0.03937
SPATA7	-0.016196824	-0.017615634	0.076223	0.049821	-0.11939	0.138771
SPATA8	-0.029425572	-0.081290711	-0.06264	-0.02788	0.026537	-0.00796
SPATA9	-0.041097677	0.133975571	-0.10179	-0.03842	0.115436	-0.10589
SPATC1	-0.026462531	-0.06312171	-0.01274	0.020935	-0.03089	0.010719
SPATC1L	-0.008180423	-0.055827926	0.015122	0.014836	-0.02653	0.133974
SPATS1	-0.032805013	-0.044505879	-0.02261	-0.00334	0.023882	0.002838
SPATS2	0.00283899	0.070417096	-0.01613	0.078138	-0.16203	-0.20091
SPATS2L	-0.023384863	-0.04503828	-0.06415	0.026016	-0.11951	-0.02434
SPC24	-0.023728969	-0.034605237	0.083452	0.148239	-0.43178	-0.06133
SPC25	-0.014254935	0.00253259	0.043132	0.167279	-0.34879	-0.01948
SPCS1	0.071325205	-0.006297742	0.101153	0.056553	-0.13307	-0.10024
SPCS2	0.080905617	0.211118001	0.023604	0.058145	-0.16544	-0.15565
SPCS3	0.053626819	0.102304375	-0.08928	0.077801	-0.22927	0.274151
SPDEF	0.00567458	-0.060681301	0.002419	-0.03831	-0.03629	-0.1549
SPDL1	0.002521931	0.128913951	0.099726	0.100583	-0.27533	-0.24301
SPDYA	-0.04030875	-0.019921463	0.045474	0.013275	-0.01748	-0.07549
SPDYE2	0.009311853	-0.023191456	0.101731	0.037416	-0.09778	-0.05128
SPECC1	-0.016713148	0.009572436	-0.31189	-0.083	0.149966	0.367439
SPECC1L	0.038100408	0.06638647	0.101372	0.057982	-0.20123	-0.21413
SPEF1	-0.019162821	-0.032574337	-0.12447	0.008789	-0.01259	-0.01495
SPEF2	0.014789462	0.065554678	0.30989	0.024286	0.028811	-0.0906
SPEM1	-0.03308407	-0.068195516	-0.03327	0.019774	-0.04735	0.008254
SPEN	0.042598119	0.085280001	-0.12984	-0.00868	0.021675	-0.04364
SPERT	-0.040136355	-0.077125492	-0.11375	0.052467	-0.10964	0.200949
SPESP1	-0.02658332	-0.01849666	-0.15912	-0.02791	0.100715	0.277839
SPG11	0.007979996	-0.054868909	-0.14943	-0.04252	0.093321	0.145947
SPG20	-0.037447805	0.041742068	0.040933	-0.02562	0.128237	-0.14097
SPG20OS	-0.032408855	-0.036419439	-0.05734	0.007102	-0.01255	-0.00236
SPG21	0.095550455	-0.069728607	-0.26138	0.024115	-0.03601	0.246999
SPG7	-0.035743936	-0.054579725	-0.02167	-0.00269	0.018425	0.008762
SPHK1	-0.002575502	-0.045502044	-0.05592	-0.03143	0.059684	0.109999
SPHK2	-0.012053727	0.016799241	-0.07163	0.052741	-0.18292	0.260529
SPHKAP	-0.028628303	-0.056376478	-0.02378	0.006294	-0.00362	-0.0849

SPI1	-0.008332773	-0.148756368	-0.27277	-0.1381	0.364601	0.432287
SPIB	-0.001244501	-0.100232631	-0.16617	-0.04717	0.135466	-0.223
SPIC	-0.037908481	0.00216801	0.045985	-0.01731	0.009622	-0.11391
SPICE1	-0.013815838	-0.021810141	0.015448	0.052835	-0.12888	-0.01568
SPIDR	-0.025807588	-0.073240504	-0.18419	-0.11543	0.136118	0.570847
SPIN1	0.032647656	0.168998896	0.082772	0.028514	-0.0736	0.004627
SPIN2A	-0.030039537	-0.043157143	-0.00591	0.028922	-0.06881	0.072471
SPIN3	-0.031316849	-0.032096342	0.151409	-0.07818	0.146323	-0.06657
SPIN4	0.004469594	0.214312588	-0.02939	0.072167	-0.19151	0.057455
SPINK1	-0.010477424	0.006402057	0.009307	-0.00492	0.013751	0.001198
SPINK13	-0.041058168	-0.078773401	-0.09464	0.009455	0.001096	-0.00488
SPINK2	-0.006249185	-0.018133756	0.479506	-0.02342	0.070927	-0.34397
SPINK4	-0.035532964	-0.087062138	0.080203	0.000156	0.003443	-0.0339
SPINK5	-0.025021078	-0.00511006	0.058777	0.07246	-0.20106	-0.05555
SPINK6	-0.028902005	-0.05760857	0.046677	0.008379	-0.01059	0.078938
SPINK7	-0.049478145	-0.070730456	0.068749	0.029909	-0.05466	0.016805
SPINT1	-0.019594142	-0.174830018	-0.20774	-0.05874	0.099418	0.293544
SPINT2	0.01378454	-0.303954333	-0.04383	-0.09479	0.242907	0.128151
SPINT3	-0.036402269	-0.015154211	0.13516	0.023367	-0.03918	-0.08566
SPIRE1	-0.014148227	0.06391673	-0.0835	-0.04602	0.128	0.249061
SPIRE2	-0.048533418	0.021906367	0.077552	0.011857	-0.00196	-0.09545
SPN	-0.027127373	-0.102283196	-0.00545	-0.03768	0.056182	0.000685
SPNS1	0.024240039	-0.055483501	-0.09392	-0.02961	0.079642	0.555766
SPNS2	-0.010926984	-0.074191202	-0.02449	0.020476	-0.11546	-0.10099
SPNS3	-0.002048886	-0.1094628	0.003242	0.004393	-0.0299	-0.14874
SPO11	-0.0696356	-0.092208473	0.067017	-0.03649	0.046712	-0.05547
SPOCD1	-0.035762355	-0.038966264	-0.08626	-0.16712	0.425743	0.008282
SPOCK1	-0.015040765	-0.046570638	-0.15957	0.150458	-0.34022	0.454656
SPOCK2	0.011836205	0.056445838	0.370739	-0.07048	0.16641	-0.30743
SPOCK3	-0.058131526	-0.123415709	0.054435	-0.02881	0.005571	0.018374
SPON1	-0.077139636	-0.091102009	0.043765	0.078691	0.033407	-0.06597
SPOP	0.070806528	0.095287202	-0.02379	0.027108	-0.07957	-0.16072
SPOPL	0.030215562	0.004770948	-0.50479	-0.01535	0.055564	0.263302
SPP1	-0.002235485	-0.013955949	-0.06134	0.059383	-0.16667	-0.03716
SPP2	-0.028100454	-0.091157002	-0.0369	-0.00917	0.001664	-0.13383
SPPL2A	0.032088824	0.167281345	-0.11704	0.054972	-0.11416	0.020253
SPPL2B	-0.034064817	-0.110135343	0.065981	-0.05119	0.013633	-0.01637
SPPL2C	-0.030401589	-0.058559524	-0.00985	0.019632	-0.02869	0.016989
SPPL3	0.049197201	-0.143412497	-0.05313	0.057852	0.033516	-0.09814
SPR	-0.007825137	-0.087315054	0.014025	-0.03253	-0.02785	0.033479
SPRED1	-0.026337985	-0.058399517	-0.1346	0.044479	0.064945	0.46613
SPRED2	-0.024112059	-0.007191207	0.007841	0.103984	-0.23546	0.131779
SPRED3	0.008523498	-0.100123502	0.009955	-0.02503	-0.05034	-0.0452
SPRN	-0.021391211	-0.066825539	-0.03618	0.006091	-0.08245	-0.08991
SPRNP1	-0.045219498	-0.049637129	0.027201	0.013766	-0.02139	0.128542
SPRR1A	-0.035874007	-0.052686346	0.067419	0.009867	-0.05332	-0.08325
SPRR1B	-0.039721936	-0.044959558	-0.19882	-0.00146	0.033268	-0.02181
SPRR2B	-0.026233165	-0.062902321	0.025123	-0.0149	0.00821	-0.12166
SPRR2C	-0.042587325	-0.063937798	-0.07914	0.006918	-0.00937	-0.03819
SPRR2G	-0.029916349	-0.043858126	-0.04696	0.001315	-0.08039	-0.08091
SPRR3	-0.037539558	0.010428872	0.111811	0.023463	-0.06952	-0.14478

SPRR4	-0.005566167	-0.022962237	0.063995	-0.01851	-0.04618	-0.15686
SPRTN	-0.011236296	0.047320488	-0.14276	0.085062	-0.1941	0.070947
SPRY1	-0.010798705	-0.030429694	0.06275	-0.00632	-0.01386	-0.08528
SPRY2	-0.006976011	0.081970495	-0.13974	0.007533	-0.03673	-0.11109
SPRY3	-0.030222518	-0.072012091	-0.13013	-0.02645	0.093309	-0.00562
SPRY4	-0.022186758	-0.015426493	-0.04317	0.00951	-0.06669	-0.02346
SPRY4-IT1	-0.021614984	-0.042186554	0.042457	0.013943	-0.05555	-0.02346
SPRYD3	0.02202281	-0.044252399	0.140098	-0.03002	-0.00595	-0.10747
SPRYD4	-0.01239338	-0.016568281	-0.16655	0.044566	-0.15746	0.126803
SPRYD7	-0.022047422	0.054265011	0.003912	0.115991	-0.2957	-0.08317
SPSB1	-0.012824688	-0.00665598	-0.01599	0.027132	-0.05912	0.015216
SPSB2	0.011101671	0.011653515	0.001303	-0.04224	0.119483	0.041512
SPSB3	0.048794302	0.067905734	0.04141	-0.08344	0.239487	-0.10776
SPSB4	-0.030274776	-0.055970295	-0.01028	0.00082	-0.03988	-0.1487
SPTA1	-0.028605493	-0.047993018	-0.01844	0.068504	-0.17657	-0.00444
SPTAN1	-0.044154305	-0.006631399	0.291456	-0.05536	0.036451	-0.37298
SPTB	-0.033070595	-0.000556335	-0.03586	-0.02562	0.001126	-0.11214
SPTBN1	0.008722619	0.034871291	0.106208	-0.04371	0.060701	-0.23025
SPTBN2	-0.031918077	-0.050722942	0.052396	-0.00902	0.006102	-0.02898
SPTBN4	-0.019283323	-0.179639867	0.016689	0.014498	-0.06967	-0.05787
SPTBN5	-0.023525711	0.003693671	0.022089	-0.02683	0.039984	-0.05908
SPTLC1	0.011248201	0.02305734	-0.19779	0.044371	-0.15343	0.109653
SPTLC2	0.023986387	0.061189498	-0.37341	-0.04672	0.094338	0.445162
SPTLC3	-0.035935294	0.161066596	-0.03369	0.04887	0.043938	-0.006
SPTSSA	0.015712774	0.04370247	-0.20893	-0.00283	0.030504	0.685822
SPTSSB	-0.03449664	0.070260868	0.129273	-0.02936	0.097534	0.024653
SPTY2D1	0.025330424	0.148247355	-0.05928	0.041026	-0.13201	-0.07192
SPTY2D1-A	-0.051754091	-0.005539696	-0.20011	0.03176	-0.06315	0.176744
SPX	-0.030692178	-0.036050032	-0.02184	-0.22405	0.780729	0.090499
SPZ1	-0.057381835	-0.05683004	-0.02522	-0.01476	0.026077	0.005589
SQLE	0.004661082	0.052143226	-0.06424	0.067234	-0.15326	-0.0713
SQRDL	0.050995936	-0.003761442	-0.17538	0.049124	-0.11025	0.360583
SQSTM1	-0.007161409	-0.054614326	-0.03516	-0.0277	-0.06057	0.043132
SRA1	0.044598875	0.004756502	-0.3004	0.10565	-0.26591	0.395048
SRBD1	0.011964805	0.005273154	-0.12822	0.028186	-0.09732	0.040989
SRC	-0.011757582	-0.199395955	-0.03207	0.003468	0.026698	0.145204
SRCAP	-0.051070171	0.004215052	0.096711	0.00296	-0.13765	-0.08769
SRCIN1	-0.028725584	-0.054547772	-0.0179	-0.01566	0.042351	-0.10107
SRCRB4D	-0.021443584	-0.091575257	-0.00597	-0.00484	-0.03633	-0.0064
SRD5A1	-0.01405187	-0.047327427	-0.21531	0.01044	-0.05703	0.468146
SRD5A2	-0.056733284	-0.042697512	0.021149	0.007925	0.008367	-0.08438
SRD5A3	-0.010919855	0.010046228	-0.03596	0.043528	-0.12473	0.163595
SRD5A3-AS	-0.041378136	-0.052451551	-0.07346	0.007049	-0.0112	0.033885
SREBF1	-0.007254605	-0.123417893	0.250234	-0.00427	-0.01515	0.044884
SREBF2	-0.014629218	0.025211426	-0.01228	0.009591	-0.15872	-0.12663
SREK1	0.020676039	-0.004444011	-0.04494	-0.02142	0.129532	0.189395
SREK1IP1	0.015500936	0.082700446	0.080136	-0.03115	0.128137	0.066694
SRF	0.013031308	-0.050945115	-0.1547	0.062339	-0.21777	-0.04373
SRFBP1	-0.003996359	0.005715876	-0.04212	0.025327	-0.0588	0.082992
SRGAP1	-0.119293399	-0.037296419	0.021723	0.044209	0.015491	0.138307
SRGAP2	-0.001546638	-0.084525559	-0.16325	-0.01668	0.028049	0.255264

SRGAP2C	0.028017069	-0.080104458	-0.12419	0.004114	-0.01451	0.279476
SRGAP3	-0.022955627	-0.059270992	-0.08253	0.0141	-0.01673	-0.06523
SRGN	0.00913218	0.127245876	-0.13238	0.024509	-0.00692	0.232658
SRI	0.036706799	0.048955551	0.05888	0.108185	-0.27845	-0.08638
SRL	-0.017814437	-0.101993073	0.006155	-0.00036	-0.08231	-0.04466
SRM	0.019310848	-0.034273195	0.096592	0.080585	-0.2047	0.024089
SRMS	-0.036792059	-0.018782317	-0.02975	0.002622	0.005472	0.056173
SRP14	0.121642624	0.107534687	-0.01025	0.122796	-0.2735	-0.18031
SRP19	0.102518714	0.220111133	0.092853	0.114524	-0.2735	-0.14866
SRP54	0.063574828	0.128059697	-0.13988	0.097552	-0.25519	-0.11238
SRP68	0.053392524	0.059381477	0.149329	0.050744	-0.15554	0.042349
SRP72	0.035394981	0.04206321	0.027241	0.041217	-0.1288	0.177174
SRP9	0.103688576	0.187810512	0.150209	0.104087	-0.20347	-0.0796
SRPK1	0.020447251	0.022295969	0.015201	0.071717	-0.22858	-0.32469
SRPK2	0.015370356	0.127969363	-0.11698	0.002266	-0.02579	-0.20285
SRPK3	-0.027464945	-0.111139847	-0.11893	0.01707	-0.09656	0.025033
SRPR	0.071324019	0.063184174	0.050571	0.025252	-0.07726	0.051846
SRPRB	-0.002197244	0.059621387	0.153549	0.068122	-0.2404	0.065429
SRPX	-0.008204927	-0.048269991	-0.07494	-0.00477	0.01809	-0.14558
SRPX2	-0.06775901	-0.046517049	0.020871	0.015056	-0.02252	0.102839
SRR	0.012402813	0.045736959	0.050509	0.138876	-0.24958	0.025246
SRRD	0.02354683	0.079048816	0.086719	0.070336	-0.18992	-0.01509
SRRM1	0.08265565	0.084107263	0.174583	0.055737	-0.11839	-0.24473
SRRM2	0.046177155	-0.017890877	0.005013	-0.01372	0.009296	-0.18157
SRRM2-AS	-0.026237713	-0.049271078	0.09211	-0.00547	-0.01236	-0.05285
SRRM3	-0.040206283	-0.017554287	0.002954	7.85E-05	0.036152	-0.02344
SRRM4	-0.016490206	-0.038038161	0.016686	0.072233	-0.11979	0.073456
SRRM5	-0.036164306	-0.012511706	-0.09213	0.01605	-0.03482	0.052442
SRRT	-0.039085123	-0.126147158	0.030956	0.02263	-0.05308	-0.01968
SRSF1	0.050939104	-0.051760072	0.157757	0.091143	-0.1981	-0.30746
SRSF10	0.034076511	0.087816656	0.212803	0.041911	-0.09588	-0.12385
SRSF11	0.028088867	0.04323649	0.32902	-0.04613	0.138258	-0.18145
SRSF12	-0.056963703	-0.050480445	0.002253	-0.00376	0.036704	-0.1535
SRSF3	0.05714865	0.027788069	0.188039	0.014652	-0.04264	0.016045
SRSF4	0.001762874	0.013674585	0.092169	0.011351	-0.02959	-0.11399
SRSF5	0.072750634	0.077884702	0.205032	0.033399	0.073953	-0.39074
SRSF6	0.04061183	0.005061685	0.019429	0.002035	0.004994	-0.00199
SRSF7	0.034523727	0.041131633	0.239003	0.002982	-0.01043	-0.07967
SRSF8	0.022260515	0.063213264	0.311883	-0.00455	0.024302	-0.23258
SRXN1	0.026310186	0.057024538	-0.14549	0.041891	-0.11456	0.146885
SRY	-0.043353212	-0.040317445	-0.11739	0.024338	-0.05472	0.142566
SS18	-0.016744258	0.095664092	-0.0469	0.053368	-0.11984	0.070189
SS18L1	0.037352411	0.067254618	-0.04865	0.036458	-0.07297	0.1473
SS18L2	0.08171503	0.10276611	0.287456	0.079047	-0.21551	-0.22599
SSB	0.03692073	0.047517978	0.166329	0.041294	-0.10097	0.107823
SSBP1	-0.011790409	0.031606668	0.189071	-0.01048	0.040073	-0.10089
SSBP2	-0.012608437	-0.093113468	-0.02281	-0.09209	0.237019	0.027181
SSBP3	0.014642073	0.148717011	0.264622	-0.03132	0.056275	0.012287
SSBP3-AS	0.010547405	0.130466673	0.085887	-0.02223	0.069495	-0.07114
SSBP4	0.030903284	0.099840719	0.232953	0.018263	-0.02742	0.085614
SSC5D	-0.016402684	-0.094859005	0.042939	0.003422	-0.08053	-0.0589

SSFA2	-0.008805758	-0.052336269	-0.12768	-0.06125	0.120155	0.170365
SSH1	-0.014622637	0.157234721	-0.16933	0.070666	-0.14045	0.090343
SSH2	-0.010648476	-0.043325908	-0.20532	-0.01045	0.022893	0.128248
SSH3	-0.000402196	-0.027495215	-0.07985	-0.04401	0.040525	-0.06246
SSMEM1	-0.076510434	-0.030360437	0.012919	-0.00633	0.033129	-0.01837
SSNA1	0.048370843	0.043827215	-0.13371	0.064275	-0.25047	0.101598
SSPN	-0.015800772	-0.068954612	-0.19619	-0.06513	0.181971	-0.23157
SSPO	-0.031020601	-0.089816478	0.015761	0.029424	-0.09916	-0.01562
SSR1	0.030384579	0.031712694	-0.13202	0.039255	-0.15785	0.141784
SSR2	0.093385781	0.124858246	0.288802	0.088466	-0.17331	-0.1653
SSR3	0.016288902	0.004107938	-0.11463	0.025445	-0.0688	0.387375
SSR4	0.035708399	0.09324302	0.196985	0.046608	-0.11829	0.053738
SSR4P1	-0.034440995	-0.115137061	0.132761	0.02732	-0.09288	0.046001
SSRP1	0.034690266	0.032930916	0.172987	0.090069	-0.24576	-0.09402
SSSCA1	0.017647005	0.074727466	0.052931	0.076796	-0.22149	0.118186
SSSCA1-AS	-0.024225533	-0.005348864	0.011103	-0.01903	0.063467	-0.05899
SST	-0.035619796	-0.123262796	0.080009	0.023616	-0.03254	0.056427
SSTR1	-0.045397078	-0.061028999	-0.06519	0.104254	-0.09358	0.000798
SSTR2	-0.027231609	-0.022630252	0.118169	0.02545	-0.03768	-0.05101
SSTR3	-0.022146145	-0.101569781	0.055351	-0.0358	-0.01703	-0.10811
SSTR4	-0.035152081	-0.034888328	-0.02359	0.032317	-0.05267	0.100663
SSTR5	-0.032769807	-0.067858373	0.004261	0.039294	-0.07381	0.09013
SSTR5-AS	-0.02975096	-0.052800469	0.016446	-0.01199	0.027349	-0.12964
SSU72	0.030042674	-0.147147234	0.057783	0.122643	-0.22647	-0.04221
SSUH2	-0.032410454	-0.063037838	0.001563	-0.00866	0.021118	-0.03464
SSX1	-0.051845948	-0.063930433	-0.04087	0.080278	-0.24518	0.041639
SSX2B	-0.03541922	-0.057652369	0.121857	0.018743	-0.03549	-0.03872
SSX2IP	-0.013589946	0.23540791	-0.05031	-0.08007	0.135845	-0.15557
SSX3	-0.037821765	-0.037178363	0.122086	0.039288	-0.10883	-0.05142
ST13	0.051400771	0.07495696	0.208148	0.055598	-0.13899	-0.08519
ST14	-0.031447778	-0.138724382	-0.18592	-0.11293	0.218028	0.18495
ST18	-0.066027654	-0.074151827	-0.07167	0.041691	-0.08504	-0.12472
ST20	0.003138283	-0.042343746	-0.18224	-0.01472	0.011846	-0.03759
ST3GAL1	0.015832425	0.036754386	0.294485	0.032631	-0.06414	-0.18813
ST3GAL2	0.021567708	-0.063448009	-0.09526	0.012874	-0.06018	-0.22195
ST3GAL3	-0.048450461	-0.060071954	0.169011	-0.00334	0.001951	0.034054
ST3GAL4	-0.016824973	0.034848746	-0.14592	0.020611	-0.04763	-0.1071
ST3GAL4-/	-0.00022197	0.096995001	-0.15108	0.033242	-0.08917	-0.18038
ST3GAL5	0.03103858	0.055956868	0.086341	-0.03753	0.09144	0.167534
ST3GAL6	-0.016061768	-0.129452823	-0.32451	-0.07884	0.196967	0.410362
ST5	-0.022067309	0.061398617	-0.0041	0.04771	-0.12319	-0.15501
ST6GAL1	-0.030363497	-0.036863013	0.03601	-0.00266	0.329687	-0.19247
ST6GAL2	-0.082268191	-0.095838415	0.025012	-0.02131	0.063939	0.000916
ST6GALNA(	-0.011451478	-0.023063965	-0.03763	-0.02731	0.097742	-0.045
ST6GALNA(	-0.006725589	-0.095098661	-0.2169	-0.03775	0.079422	0.160045
ST6GALNA(	-0.019336471	-0.101834429	-0.02371	-0.1215	0.363326	0.437117
ST6GALNA(	0.021415607	-0.048431612	0.059198	0.009485	0.030191	-0.08484
ST6GALNA(	-0.038223812	0.014821743	-0.14433	0.015647	0.009598	0.194016
ST6GALNA(	0.014242696	0.19402687	0.173787	0.011016	-0.05118	-0.19992
ST7-AS1	-0.036967674	-0.070950899	-0.0388	-0.09036	0.287192	0.09492
ST7-AS2	-0.06531429	-0.032231796	-0.03979	-0.01246	0.031514	-0.07417

ST7-OT4	-0.052728296	-0.089955532	0.124608	0.009332	-0.03307	-0.07506
ST7L	-0.012546789	-0.060348909	0.107708	0.030715	0.023414	-0.05078
ST8SIA1	-0.023656936	-0.080542239	0.225844	0.001823	0.004296	-0.17161
ST8SIA2	-0.057154127	-0.092365727	-0.00753	0.013124	-0.00056	0.078096
ST8SIA3	-0.026432155	-0.051749447	0.038476	0.072507	-0.13081	0.085051
ST8SIA4	0.003322151	0.067243265	-0.29773	0.02215	-0.04877	0.209151
ST8SIA5	-0.073304941	-0.066628299	-0.06012	0.026207	-0.05099	-0.00795
ST8SIA6-/	-0.040982806	-0.088640518	-0.1334	-0.00097	0.034925	0.072598
STAB1	-0.004729574	-0.099091687	-0.22098	-0.09104	0.267126	0.717281
STAB2	-0.038891738	-0.074082317	-0.07157	-0.00518	0.010993	-0.00547
STAC	-0.023938606	-0.012794341	0.042945	-0.02441	-0.02908	-0.02143
STAC2	-0.010273509	-0.040939375	0.027012	-0.01897	-0.0546	-0.10987
STAC3	-0.012435452	-0.167304718	-0.05611	-0.05021	0.139312	0.311249
STAG1	-0.00368118	0.097104971	-0.16268	0.118287	-0.20236	-0.30822
STAG2	0.08814962	0.165332981	-0.14514	-0.01578	0.000647	-0.14987
STAG3	-0.002651519	-0.063606961	-0.06017	0.081872	-0.26099	-0.21288
STAG3L3	0.010181891	-0.018749407	0.209545	0.018086	-0.01553	-0.20666
STAG3L4	0.011211365	0.009905411	-0.0348	0.019791	-0.02888	0.090704
STAM	0.032393081	0.119705372	-0.11322	-0.0118	0.013761	0.070435
STAM-AS1	-0.056601294	-0.003559863	0.103098	-0.00357	-0.01316	-0.02297
STAM2	0.018610374	-0.011261713	-0.33334	0.015162	-0.04709	0.091977
STAMBP	-0.019867425	-0.082956502	0.088538	1.27E-05	0.048929	-0.05265
STAMBPL1	0.00728963	0.032661329	0.19081	0.041605	-0.12161	-0.39742
STAP1	-0.002328039	0.056028291	-0.15502	-0.02504	0.054656	-0.28912
STAP2	-0.0268784	-0.046390898	-0.04811	0.030216	-0.11489	-0.27284
STAR	-0.031829661	-0.024628621	-0.04513	-0.02903	0.110706	-0.05021
STARD10	-0.000502546	-0.11106875	-0.07628	0.064559	-0.05988	-0.03236
STARD13	-0.033739667	-0.086594419	-0.11488	0.018855	-0.04609	0.020525
STARD13-/	-0.030181567	-0.031958989	0.054794	-0.00054	-0.03445	-0.00521
STARD3	0.014891078	-0.011476485	-0.00051	-0.02834	0.013987	-0.10806
STARD3NL	0.061921714	0.346572933	-0.17564	0.071429	-0.18279	0.173582
STARD4	0.007050124	0.157383284	0.022573	0.040028	-0.12705	-0.11382
STARD5	-0.007117225	-0.12824739	0.110338	0.011061	-0.01138	-0.20428
STARD6	-0.054000777	-0.043304936	0.122711	0.040497	-0.11959	0.053882
STARD7	0.053098766	0.085917503	-0.00932	0.039103	-0.09253	0.107181
STARD7-AS	-0.018191555	-0.074583421	-0.08611	0.033317	-0.09169	0.115243
STARD8	-0.00746566	-0.083547815	-0.17672	-0.04347	0.076947	0.234442
STARD9	-0.006367817	0.155806901	0.10484	0.015242	-0.03814	-0.14826
STAT1	0.035740702	-0.021642336	-0.02329	0.011491	-0.05639	0.118849
STAT2	-0.048121044	-0.107771097	-0.13077	-0.06521	-0.02895	0.120703
STAT3	0.043374393	0.076747635	-0.1486	0.029608	-0.05903	-0.01136
STAT4	0.010330888	0.18267697	0.427643	-0.00507	-0.01053	-0.41363
STAT5A	0.030804839	-0.028027474	0.007137	0.012859	-0.03831	0.024791
STAT5B	0.037286198	-0.024791174	0.046514	-0.01621	0.044506	-0.19659
STAT6	0.039846349	-0.025544962	-0.2126	-0.065	0.186348	0.062968
STATH	-0.039183491	-0.027260087	-0.00991	0.021709	-0.05479	0.021463
STAU1	0.128417811	0.119436192	-0.14657	0.134542	-0.30355	0.001743
STAU2	0.01193922	0.102130647	0.009881	-0.01708	-0.15826	-0.08752
STAU2-AS	-0.027983748	0.004870637	0.052174	0.002092	-0.00995	-0.02003
STBD1	-0.033276666	-0.045971398	0.075295	0.025307	-0.0419	0.018181
STC1	-0.011554951	-0.020553424	-0.0041	0.125271	-0.16001	-0.00639

STC2	-0.02664402	-0.015740706	-0.04053	0.004112	-0.0238	-0.01301
STEAP1	-0.015025989	-0.030124806	-0.05151	0.135589	-0.21316	-0.07522
STEAP1B	-0.016456242	-0.033961018	-0.1818	0.016943	-0.03065	-0.12676
STEAP2	-0.016419266	-0.015311663	0.00088	0.057369	-0.17011	-0.03561
STEAP3	-0.021410049	0.00101087	-0.29152	-0.04324	0.138622	0.505544
STEAP4	-0.00523969	-0.057244639	-0.26901	-0.04586	0.138117	0.330842
STGC3	-0.031058477	-0.036770037	-0.0052	0.016545	-0.03014	0.057601
STH	-0.033064435	-0.001434282	-0.06011	0.034017	-0.11692	-0.0876
STIL	-0.003186092	0.013087376	-0.00186	0.170514	-0.42489	-0.06663
STIM1	0.006141725	0.089760662	0.101548	-0.02587	0.032669	-0.22095
STIM2	0.002540802	0.118951827	0.158583	0.022922	0.022184	-0.26492
STIP1	0.017655228	0.103427664	0.049514	0.100831	-0.30216	-0.05178
STK10	0.043918486	0.158664336	0.015187	-0.00714	0.045152	-0.05368
STK11	0.084409711	-0.029630735	0.029705	0.006029	-0.07618	0.219769
STK11IP	0.024481499	-0.059382661	-0.24475	-0.04001	0.102613	0.198101
STK16	-0.00259852	-0.079327623	0.09292	0.024747	-0.09431	0.040245
STK17A	0.002143981	0.152470984	0.286762	-0.00632	-0.00429	-0.50186
STK17B	0.022076217	-0.095598982	-0.2046	-0.01055	0.023476	0.127294
STK19	0.033748609	-0.059924747	0.045715	-0.01024	0.020113	-0.05379
STK24	-0.034437246	-0.04428661	-0.07912	0.018419	-0.02709	-0.08587
STK24-AS1	-0.043946198	-0.028464984	-0.00853	0.028461	-0.08368	0.025177
STK25	0.015979765	0.029281849	0.06299	-0.03147	0.073942	0.20586
STK3	-0.001738137	-0.036725813	-0.35031	-0.02312	0.051815	0.408008
STK31	-0.032765482	0.032843413	0.014905	0.002902	0.005953	-0.12478
STK32A	-0.056489237	-0.028726126	0.000904	-0.00287	-0.06	0.143674
STK32B	-0.02607064	-0.06284559	-0.02936	-0.0185	0.015541	0.099415
STK32C	-0.012937592	-0.03365381	-0.09272	-0.08009	0.17429	0.282321
STK33	-0.029486394	-0.091054372	0.140744	0.124741	-0.31939	-0.07706
STK35	-0.02348483	-0.031175789	0.006969	0.024676	-0.16704	-0.18176
STK36	-0.011871482	-0.014871064	0.045546	0.007574	-0.00401	0.225682
STK38	-0.062319932	-0.004702641	0.027479	-0.06909	0.212539	-0.10201
STK38L	0.016876365	-0.032158612	-0.2611	-0.0729	0.19949	0.1506
STK39	0.016454038	0.200543243	0.23168	0.047394	-0.1185	-0.0023
STK4	-0.035862737	-0.015040463	0.0531	0.012562	0.110722	-0.31817
STK4-AS1	-0.036028215	-0.067228215	0.055092	0.044234	-0.18431	-0.11601
STK40	0.021986061	-0.020974753	-0.3084	-0.01618	0.01969	0.083824
STKLD1	-0.046844151	0.013726809	0.020582	0.022097	-0.02433	0.060835
STL	-0.080677999	-0.027104755	0.040188	-0.01801	0.053479	0.013061
STMN1	0.000826309	0.015416317	0.147816	0.083211	-0.24274	-0.34198
STMN2	-0.059207955	-0.03226069	0.007896	0.030792	-0.06463	0.040758
STMN3	0.001010779	-0.134374671	0.370539	-0.05978	0.17396	-0.24706
STMN4	-0.036316501	-0.076174114	-0.00475	0.011008	-0.09413	-0.03222
STOM	0.040348471	0.296499473	0.289792	0.023598	-0.03428	-0.01704
STOML1	-0.030363439	-0.072230207	0.040051	-0.02481	-0.10265	-0.08678
STOML2	0.029911287	0.037132718	0.146699	0.088094	-0.25463	-0.02376
STOML3	-0.045616743	-0.089113725	0.028578	0.012881	-0.01471	-0.09994
STON1	-0.020824332	-0.026867289	0.008957	0.09397	-0.22005	-0.10169
STON2	-0.029007381	-0.051093374	-0.02566	-0.05419	0.091981	0.148895
STOX1	-0.028566509	-0.062280914	-0.00888	0.008533	-0.00942	-0.20412
STOX2	-0.023607254	-0.027819473	-0.0003	0.076044	-0.1895	0.047433
STPG1	-0.020615138	-0.111817676	0.149023	-0.03887	0.081483	-0.22035

STPG2	-0.027147138	-0.0507496	0.054664	-0.01126	0.02091	-0.10312
STRA13	0.045401416	-0.007578209	0.140211	0.082136	-0.26101	0.068497
STRA6	-0.037163305	-0.069650076	-0.11936	0.025613	-0.04182	0.071995
STRADA	0.037027056	-0.048362579	-0.07231	0.030583	-0.13942	-0.21224
STRADB	0.018829106	-0.046036761	-0.00906	-0.02465	0.066303	-0.06209
STRAP	0.042323383	0.099785494	0.068863	0.031726	-0.0642	-0.16273
STRBP	-0.015240024	-0.075147302	0.020789	-0.00099	0.041521	-0.30737
STRIP1	0.050293948	0.147468406	0.106887	-0.02564	0.021667	-0.20468
STRIP2	-0.023723221	-0.026859646	-0.02232	0.079184	-0.19293	-0.04054
STRN	-0.005041648	0.135611841	0.062297	-0.06499	0.056563	-0.15996
STRN3	0.002886133	0.030242399	-0.14856	0.041787	-0.14315	0.062181
STRN4	-0.001948415	-0.070959953	-0.24646	-0.01536	0.040743	0.374019
STS	-0.022004886	0.037859193	-0.28945	-0.02414	0.154235	0.597323
STT3A	0.027276528	0.078865811	-0.06986	0.085259	-0.25328	-0.01357
STT3B	0.068169985	0.083959614	0.107247	0.089336	-0.22258	-0.02999
STUB1	0.068641394	0.101375401	0.147556	0.045614	-0.12092	-0.13173
STX10	0.030402159	-0.068903325	-0.13866	-0.01389	0.044955	0.332681
STX11	0.008540999	0.004404666	-0.34866	-0.02748	0.067136	0.470254
STX12	0.045291134	-0.014593388	-0.37626	-0.01233	0.055546	0.498287
STX16	0.038843954	-0.032568894	0.253132	-0.0218	0.034825	-0.21243
STX17	-0.00706349	-0.017709671	0.176112	0.07021	-0.10203	-0.09674
STX18	0.044343272	-0.016859938	-0.07727	0.033204	-0.11556	0.083192
STX18-AS1	-0.021801251	-0.007891547	0.093391	0.006256	-0.011	-0.13064
STX19	-0.055825027	0.026536208	0.065929	-0.00911	0.035874	-0.01217
STX1A	-0.012506727	0.040299093	0.022126	0.014347	-0.09307	-0.11169
STX1B	-0.032127372	-0.049633899	-0.08293	-0.00657	-0.03276	0.026483
STX2	0.011629715	-0.052104437	0.264511	-0.02156	0.024224	0.019395
STX3	0.004391549	-0.099518778	-0.24912	0.005515	-0.02795	0.037803
STX4	0.051298808	0.057521791	-0.16285	-0.01778	-0.00124	0.122914
STX5	0.008927171	0.018842313	-0.15233	0.018194	-0.05462	0.104892
STX6	0.00356862	-0.015139615	-0.1742	0.021234	-0.11201	0.259847
STX7	0.011997434	-0.040531591	-0.51331	-0.06696	0.171811	0.120586
STX8	0.017942354	0.103294416	0.044942	0.041087	-0.11311	0.080202
STXBP1	-0.015172585	-0.065836977	0.307448	0.149174	-0.44739	-0.10683
STXBP2	0.018521215	-0.080202812	-0.33298	-0.04857	0.093099	0.426251
STXBP3	0.032708002	0.033834052	-0.14874	-0.00393	0.048731	0.188125
STXBP4	-0.052926403	-0.060819805	0.10676	0.112822	-0.13853	-0.07816
STXBP5	0.020329879	-0.007045144	-0.01992	-0.03759	0.05442	-0.2301
STXBP5-AS	0.017157965	-0.023436717	-0.02802	-0.00302	0.226999	0.038463
STXBP5L	-0.07971344	-0.104226292	0.022591	-0.03697	-0.06089	-0.0595
STXBP6	-0.015957218	-0.025916496	-0.01146	0.112342	-0.2511	-0.05922
STYK1	-0.011291693	0.081732029	0.101297	0.062736	-0.16134	-0.03572
STYX	-0.015748515	0.107806926	0.154921	-0.00144	-0.0027	-0.09586
STYXL1	0.03029313	0.00503834	-0.14367	0.037565	-0.18298	0.272767
SUB1	0.07104902	0.082373406	0.064273	0.134693	-0.26614	-0.19805
SUCLA2	0.029461203	0.081492024	-0.02161	0.071008	-0.20314	0.318021
SUCLG1	0.038251643	0.089788612	0.078415	0.025116	-0.07099	0.105251
SUCLG2	0.011768368	0.03275902	0.169316	0.021194	-0.05171	0.106843
SUCLG2-AS	-0.034437629	0.056955532	0.253974	0.049202	-0.10048	-0.24
SUCNR1	0.004486452	-0.036053799	0.030548	-0.12321	0.320803	-0.00703
SUCO	0.048936129	0.093581916	-0.03116	0.021309	-0.05874	-0.14763

SUDS3	0.021208626	0.035813308	-0.20523	0.043894	-0.1222	-0.10679
SUFU	-0.045685652	-0.022935914	-0.11076	-0.00331	0.000659	0.015507
SUGCT	-0.021021349	-0.091208056	0.034603	0.036164	-0.08498	-0.07888
SUGP1	0.045874847	-0.034453853	0.023719	0.048416	-0.20275	-0.0595
SUGP2	0.052091711	0.116774671	0.101864	0.022612	-0.00703	-0.13386
SUGT1	0.013093699	0.077898314	-0.0282	0.067939	-0.19031	0.098362
SUGT1P1	-0.056073918	-0.071841552	-0.00401	-0.02338	0.032933	-0.01342
SUGT1P3	-0.085534045	-0.073817428	0.145079	0.015728	0.049374	-0.14077
SULF1	-0.01806526	-0.017342649	-0.0162	0.129939	-0.22249	-0.00558
SULF2	0.003335426	-0.143862148	-0.3518	-0.09035	0.264179	0.38835
SULT1A1	0.027711146	-0.081439714	-0.1913	-0.06053	0.199675	0.555786
SULT1A2	0.021625729	-0.091292617	-0.21373	-0.06496	0.181503	0.6446
SULT1B1	-4.77E-05	0.017628648	-0.09432	-0.05776	0.167885	0.237264
SULT1C2	-0.059190527	-0.025903385	-0.01277	-0.03301	0.032144	-0.11475
SULT1C4	-0.053222142	-0.119209027	0.027931	-0.0064	0.026373	-0.00325
SULT1E1	-0.079683018	-0.053391259	-0.15012	-0.01564	0.06082	0.078412
SULT2A1	-0.070973902	-0.064824267	0.00453	0.02365	-0.07193	-0.04724
SULT2B1	-0.024354189	-0.08357421	-0.01285	0.011439	-0.00551	0.220706
SULT4A1	-0.030873644	-0.046697135	-0.10312	0.001758	-0.06367	-0.01928
SUMF1	0.045095418	0.00563397	-0.22955	-0.09853	0.257752	0.526321
SUMF2	0.033791126	0.026024466	0.171965	0.040083	-0.11304	-0.00449
SUMO1	0.120332275	0.2275515	0.006309	0.11408	-0.30092	-0.10537
SUMO3	0.048468938	0.015291054	0.027139	0.032832	-0.09822	0.246768
SUMO4	0.082056335	0.030575839	0.066121	0.072614	-0.19563	-0.14237
SUN1	-0.026185529	-0.098327905	0.008126	0.027714	-0.01155	0.155124
SUN2	0.03791914	0.155061375	0.236356	-0.03149	0.083987	-0.13133
SUN3	-0.046198828	-0.068764352	0.084154	-0.03076	0.073332	-0.05845
SUN5	-0.044605486	-0.019986487	0.050637	0.003395	-0.06216	-0.04667
SUOX	-0.009151864	-0.006305758	-0.27066	-0.10427	0.259762	0.345948
SUPT16H	0.028059077	0.090835652	0.1438	0.066091	-0.20944	-0.24144
SUPT20H	0.038610931	0.004458182	0.023127	-0.03719	0.111383	-0.32901
SUPT3H	0.005575441	-0.020065542	0.514135	0.024747	-0.05276	-0.37072
SUPT4H1	0.057372323	0.009439049	-0.3529	-0.02096	0.073209	0.278104
SUPT5H	0.063455486	-0.062851869	0.181942	0.044538	-0.16312	-0.26316
SUPT6H	0.040049326	0.027680306	-0.09723	0.072868	-0.18083	-0.0334
SUPT7L	-0.007249965	0.084250943	0.072586	-0.04311	0.140921	-0.01778
SUPV3L1	0.015669306	0.088313571	0.151538	0.120247	-0.34278	-0.15224
SURF1	0.055441343	-0.039030203	0.128346	0.004316	0.028984	0.044794
SURF2	0.014977006	-0.048824322	0.159425	0.049033	-0.11861	0.197638
SURF4	0.050881782	0.061589168	0.162611	0.062229	-0.27384	0.050381
SURF6	0.012652058	-0.022650827	0.013672	0.02799	-0.07602	0.019182
SUSD1	0.023699284	0.091597841	-0.19206	-0.06936	0.193894	0.24163
SUSD2	-0.04681136	-0.091647394	0.006855	-0.0052	-0.02922	-0.17307
SUSD3	-0.002841828	-0.200501744	0.210102	-0.08145	0.219749	-0.0965
SUSD4	-0.025733724	-0.067053289	0.231064	-0.01598	0.041309	-0.16245
SUSD5	-0.074265495	-0.055163272	-0.06099	-0.04813	0.124727	0.068028
SUV39H1	0.011901331	0.080012848	0.153682	0.121102	-0.38625	-0.08571
SUV39H2	-0.011242416	0.035145699	0.067179	0.110201	-0.30417	-0.14318
SUV420H1	0.088953666	0.102703115	-0.24942	-0.05021	0.032504	-0.12986
SUV420H2	-0.008776193	-0.088430032	0.058342	0.013361	-0.11127	-0.08491
SUZ12	-0.020643546	-0.051232375	-0.09563	0.03809	-0.14685	0.071899

SV2A	-0.008760857	-0.024894921	0.170562	0.038945	-0.12398	-0.10956
SV2B	-0.004879673	-0.081933097	-0.11547	0.054664	0.024248	-0.0851
SV2C	-0.039626492	-0.053065714	0.059887	-0.00533	0.033273	-0.04545
SVEP1	-0.031460202	-0.053625335	0.013698	0.013949	-0.02862	-0.11139
SVIL	-0.007977228	-0.017919969	-0.10235	-0.01598	0.05473	-0.0267
SVIP	0.001675462	-0.028135723	0.311532	-0.0549	0.165704	-0.46116
SVOP	-0.048470478	-0.048319568	-0.08106	0.008016	0.006638	-0.17236
SVOPL	-0.021754665	-0.088747532	0.085242	0.003344	-0.00643	-0.22192
SWAP70	-0.048698797	-0.027548035	-0.2944	-0.0693	0.240893	0.056905
SWI5	0.02277107	-0.020777008	0.089439	0.027534	-0.04529	0.053107
SWSAP1	0.002647908	-0.039208638	-0.06267	-0.04887	0.155473	0.494458
SWT1	-0.001067579	0.008534259	-0.00334	0.028813	-0.07598	-0.05404
SYAP1	0.047104193	0.116497705	-0.10187	0.086853	-0.2349	-0.04677
SYBU	-0.015909091	-0.028398188	-0.08947	0.095287	-0.1076	-0.0986
SYCE1	-0.046551785	-0.045524722	-0.01042	-0.00846	0.025958	0.086549
SYCE1L	-0.020348625	0.005300444	0.263691	-0.01393	0.067368	-0.12988
SYCE2	-0.024978066	-0.059995449	0.043756	0.155409	-0.43727	-0.03517
SYCE3	-0.038808236	-0.035622029	0.038059	########	0.038553	0.12415
SYCN	-0.02710739	-0.088927802	0.020247	0.006436	-0.00801	0.079128
SYCP1	-0.03072411	-0.083484028	0.007064	0.001807	-0.01868	-0.07225
SYCP2	-0.047159298	0.028209145	0.120515	-0.0368	0.141168	-0.03397
SYCP2L	-0.010674186	-0.094494999	-0.12584	-0.04872	0.141639	-0.17169
SYCP3	-0.056005739	-0.017622643	-0.17865	-0.02892	0.100834	0.047114
SYDE1	-0.0094212	-0.118123513	0.033052	0.003872	-0.03741	-0.08776
SYDE2	-0.042410829	-0.286166334	0.158637	-0.02351	0.106411	-0.08754
SYF2	0.06642341	0.031817874	0.005899	-0.06055	0.215248	-0.01553
SYK	0.013361001	0.019971578	-0.638	-0.13367	0.322247	0.215121
SYMPK	-0.003332561	-0.146195142	-0.03946	0.038647	-0.10445	0.143621
SYN1	-0.024258329	-0.083875606	-0.05403	-0.0091	-0.02826	-0.06428
SYN2	-0.040706155	-0.030768048	-0.07125	0.02382	0.063999	-0.04084
SYN3	-0.033797134	-0.131387652	-0.058	0.023734	-0.04163	-0.04199
SYNC	-0.009920332	0.015372029	0.0014	-0.01126	0.057967	0.253535
SYNCRIP	0.038949791	0.046530372	0.087988	0.056441	-0.18386	0.131213
SYNDIG1	-0.02595099	-0.020507267	-0.02169	-0.00053	0.009455	-0.02356
SYNDIG1L	-0.029838373	-0.065914843	0.020334	-0.00267	0.00462	0.023759
SYNE1	-0.024447958	0.167473815	0.056625	-0.02591	0.022294	-0.11278
SYNE2	-0.025343448	0.161002592	0.197807	-0.05386	0.029057	-0.44973
SYNE3	-0.022463675	-0.115619088	0.038627	0.022501	-0.07443	0.077811
SYNE4	-0.025330439	0.042850928	0.084958	0.004483	-0.01035	-0.0751
SYNGAP1	-0.026796038	-0.097470421	0.251062	-0.03748	0.074077	-0.14017
SYNGR1	0.020935203	0.05983942	-0.05822	-0.04912	0.011864	0.04612
SYNGR2	0.01672289	-0.12180963	-0.2811	0.011527	-0.01461	0.264608
SYNGR3	-0.005861349	0.099611542	-0.01589	0.006581	-0.03049	-0.07608
SYNGR4	-0.036891279	-0.015347548	-0.05636	0.050987	-0.13087	-0.00838
SYNJ1	-0.013704111	0.005213886	-0.12359	-0.03444	0.106887	0.191025
SYNJ2	0.037439429	0.104760799	0.127768	-0.05619	0.084642	-0.13951
SYNM	-0.010032113	-0.02840192	0.17691	-0.07958	0.175897	-0.08934
SYNPO	-0.069914766	-0.139706184	-0.02448	-0.01114	-0.03871	-0.10506
SYNPO2	-0.007360744	-0.011109714	0.0049	0.025774	-0.12182	-0.10168
SYNPO2L	-0.060305938	-0.029845831	-0.07342	-0.0104	-0.06338	-0.06183
SYNPR	-0.043241558	-0.056868742	0.062671	0.054638	-0.1466	0.015292

SYNPR-AS:	-0.027883714	-0.03401742	0.05557	0.024435	-0.06017	-0.08413
SYNRG	0.023425911	0.095128614	0.280762	-0.01878	0.011785	-0.3126
SYP	-0.026439414	-0.060259894	-0.05759	0.008245	-0.06614	-0.08287
SYPL1	0.026884079	-0.135023749	0.052288	0.003719	-0.01814	-0.00328
SYPL2	-0.025925376	-0.015535584	-0.08803	-0.00216	-0.02971	-0.0521
SYS1	0.034916435	-0.020426145	0.066787	-0.0012	-0.03653	-0.23346
SYT1	-0.072331445	-0.049820336	-0.10122	-0.01852	0.053005	0.067444
SYT11	-0.005456027	0.213215104	0.117768	0.107018	-0.15612	0.084595
SYT12	-0.048066324	-0.105728681	0.078519	0.042858	-0.03577	-0.0508
SYT13	-0.026245299	-0.035954955	-0.00907	-0.0108	0.015832	-0.0964
SYT14	-0.066549166	-0.021610252	-0.00423	-0.014	0.038689	-0.0608
SYT15	-0.000123537	-0.073036885	0.022209	0.007208	-0.07876	-0.06196
SYT16	-0.072067795	-0.134244149	0.074583	-0.00635	0.061529	-0.1132
SYT17	-0.013115086	-0.088009944	-0.16137	-0.04395	0.104655	0.070123
SYT2	-0.018382271	-0.04948171	-0.00174	-0.00936	-0.06755	-0.10118
SYT3	-0.039867044	-0.037309177	0.04111	0.025272	-0.03409	-0.05474
SYT4	-0.016691282	-0.020156847	0.01211	0.097365	-0.29013	-0.07125
SYT5	-0.038995187	-0.08307994	-0.09151	0.027137	-0.04975	0.049718
SYT6	-0.038117768	-0.02065609	-0.05906	0.015589	-0.04537	-0.04906
SYT7	-0.042540598	-0.065034894	0.072244	0.064757	-0.09355	-0.07287
SYT8	-0.021401733	-0.098031791	-0.0437	0.033745	-0.08669	-0.04288
SYT9	-0.050823655	-0.067977762	0.084886	0.007387	-0.04058	-0.13913
SYTL1	0.01591572	0.085612555	0.131552	-0.04477	0.111157	-0.15559
SYTL2	0.020359333	0.251304484	0.33548	-0.02805	0.109891	-0.26132
SYTL3	-0.026413475	0.23145526	0.041031	0.059345	-0.05429	0.03772
SYTL4	-0.069136434	-0.038299126	-0.06225	-0.01235	0.632455	-0.0136
SYTL5	-0.052901592	-0.044293282	0.024553	0.06149	-0.15476	-0.01666
SZRD1	0.027271547	0.052246617	0.047467	0.008384	-0.14457	0.041309
SZT2	-0.027424817	-0.04811223	0.015245	0.010656	-0.00495	0.045045
T	-0.021830937	-0.038012739	0.003258	-0.01751	-0.05106	-0.13889
T-18	-0.041323642	-0.053406753	-0.01347	0.013044	-0.00715	-0.05396
TAAR1	-0.078564518	-0.081097228	0.010488	-0.02233	0.049346	0.080957
TAAR2	-0.029700922	-0.015892529	-0.02059	0.005913	-0.01337	-0.09514
TAAR3	-0.034548701	-0.022651134	0.051779	0.00928	-0.01204	-0.04478
TAAR5	-0.029899135	-0.094959611	-0.05494	0.001104	-0.02254	-0.06437
TAAR8	-0.040581491	0.019052464	-0.00735	0.028051	-0.06117	-0.02033
TAAR9	-0.024563426	-0.04932863	0.012406	0.022083	-0.06247	-0.02575
TAB1	-0.017543568	-0.050012647	0.076998	-0.02537	0.08296	0.152058
TAB2	0.047614654	-0.008358892	0.084305	0.01529	-0.04988	-0.28492
TAB3	-0.070027836	-0.084347229	-0.07298	0.024928	-0.03512	-0.02179
TAC1	-0.033149866	-0.028328725	-0.03974	-0.01675	0.020158	-0.04141
TAC3	-0.026604785	-0.072533445	-0.02543	-0.01954	-0.01169	-0.0635
TACC1	0.031066228	0.034281233	-0.21636	0.026597	-0.02877	0.062745
TACC2	-0.099016694	-0.065077221	-0.02822	0.025958	0.034492	0.079006
TACC3	0.015714322	-0.140910294	0.01726	0.01491	-0.03424	0.193934
TACO1	0.006994342	-0.020318372	0.060436	0.066074	-0.23503	-0.00754
TACR1	-0.030263588	-0.107030337	0.008185	0.021661	0.038631	0.029635
TACR2	-0.027090064	-0.037973559	-0.01996	0.003071	-0.03193	0.053986
TACR3	-0.023762521	-0.012947648	0.008458	0.097558	-0.24556	-0.0051
TACSTD2	-0.034385836	-0.023508402	0.028466	-0.0363	0.043209	-0.123
TADA1	0.002546254	0.016758174	0.105482	0.048128	-0.12835	-0.01171

TADA2A	-0.013467641	0.041335441	0.049094	0.07171	-0.20222	0.054641
TADA2B	0.052546596	-0.045920813	-0.03033	-0.05711	0.139067	-0.11055
TADA3	0.015694672	0.071905964	-0.12671	0.018989	-0.05764	0.024479
TAF1	0.005402968	0.121680703	0.03441	-0.02216	0.067103	-0.04285
TAF10	-0.017924986	-0.039754332	0.123309	0.001649	-0.01096	-0.09889
TAF11	0.030085221	0.068135232	0.134054	0.025568	-0.08152	0.179012
TAF12	0.027082405	0.04894331	-0.09735	0.034978	-0.11641	0.129468
TAF13	0.018027004	0.142070932	-0.12993	0.032078	-0.07223	0.254992
TAF15	0.03108698	0.088785798	0.113628	-0.00508	-0.00584	-0.13533
TAF1A	-0.006239085	0.050209864	0.005413	0.063085	-0.16342	-0.15698
TAF1A-AS1	-0.020575146	0.014206655	0.306373	0.018623	-0.03389	-0.23436
TAF1B	0.006346808	0.129936864	0.12342	0.059588	-0.14807	0.149078
TAF1C	0.019825219	0.005142386	0.197395	-0.03344	0.104726	0.083313
TAF1D	0.01258959	0.013531205	0.03283	0.008048	-0.02486	-0.25578
TAF1L	-0.020005602	0.064627812	0.104922	0.025827	-0.05611	0.039202
TAF2	0.061404958	0.130249369	0.015571	0.098275	-0.32993	-0.20161
TAF3	0.019419885	0.040300955	0.284853	-0.00819	0.01364	-0.07454
TAF4	0.0269251	0.053272153	-0.00898	0.015812	-0.03612	0.006139
TAF4B	-0.003725218	-0.150736712	0.226208	0.0083	-0.02537	-0.21015
TAF5	0.020903356	0.114682173	-0.08513	0.073467	-0.18308	0.140218
TAF5L	0.01489664	0.023304616	-0.04775	0.055621	-0.1726	1.55E-05
TAF6	0.034094269	0.001429435	0.207093	0.056827	-0.18922	-0.06375
TAF6L	0.025133654	-0.133702198	-0.04142	0.03697	-0.11747	0.026929
TAF7	0.067788925	0.088448324	0.083459	0.045857	-0.08863	-0.6439
TAF7L	-0.075959136	-0.067839168	-0.02482	0.021579	-0.02545	0.094448
TAF8	-0.019642295	0.068333993	0.082605	0.008373	-0.04074	-0.01931
TAF9B	0.047713166	-0.058214023	0.005721	-0.05433	-0.11538	-0.09317
TAGAP	-0.011442622	-0.038130167	0.026768	-0.14687	0.199403	-0.14689
TAGLN	0.010370274	-0.132133845	-0.09017	-0.12056	0.29928	0.133292
TAGLN2	0.044181342	-0.078972922	-0.22513	0.017913	-0.02953	-0.19058
TAGLN3	-0.018259598	-0.011362116	-0.09406	0.020876	-0.08729	0.018679
TAL1	-0.004502343	-0.017454151	-0.09774	-0.14496	0.453964	0.076064
TAL2	-0.034569303	-0.086578493	0.057531	0.017366	-0.05683	-0.17635
TALDO1	0.029949623	-0.099927186	-0.33688	0.015883	-0.01624	0.335161
TAMM41	0.014825604	0.002678033	0.04756	-0.06409	0.027016	-0.11353
TANC1	-0.048093575	0.097723493	0.009215	-0.04038	0.01321	0.001891
TANC2	-0.045846863	0.18306289	0.131274	0.11069	-0.07827	-0.18197
TANGO2	0.038427996	-0.022268929	-0.23029	-0.0279	0.071319	0.154969
TANGO6	-0.009546216	-0.05360512	0.118196	0.006329	-0.1281	0.0509
TANK	0.024167765	-0.013214657	-0.323	0.037248	-0.13086	0.22822
TAOK1	0.05352182	0.077056478	0.055887	-0.01912	0.036151	-0.00808
TAOK2	-0.064422613	0.067659701	-0.08696	0.01738	-0.0779	0.037297
TAOK3	0.043740731	0.119428783	-0.13663	-0.02875	0.105764	-0.02699
TAP1	0.056535697	0.091830497	-0.00681	0.056893	-0.13871	-0.29029
TAP2	0.00595213	0.021576139	0.024249	0.00965	-0.09219	-0.20287
TAPBP	0.015477505	-0.008438366	0.075687	0.019293	-0.12057	-0.09137
TAPBPL	0.031919187	0.071331469	-0.1504	-0.03507	0.094758	0.047545
TAPT1	-0.030171283	-0.061272351	-0.06663	-0.01055	0.017524	-0.13836
TAPT1-AS1	-0.005101586	-0.035915677	0.091787	-0.00302	0.010836	-0.18617
TARBP1	0.018877053	-0.070583077	0.165842	0.020506	-0.04382	-0.25145
TARBP2	0.029309851	-0.005601398	0.13015	0.070958	-0.23992	0.037753

TARDBP	0.04697554	0.083068673	0.117114	0.040684	-0.09478	-0.213
TARP	-0.018405003	-0.029129327	0.390352	-0.0069	0.011019	-0.04277
TARS	0.016096993	0.116862569	0.020839	0.075508	-0.21496	-0.01383
TARSL2	-0.0008036	0.048042399	0.255793	-0.02012	0.072902	-0.26477
TAS1R1	-0.050774894	-0.080138911	0.055893	0.031865	-0.07453	0.061523
TAS1R2	-0.039095741	-0.068076731	0.051905	0.006117	0.004102	-0.0343
TAS2R1	-0.03057482	-0.069566512	0.010304	-0.01919	0.026797	0.045044
TAS2R10	-0.034878008	0.010171234	0.107768	-0.01493	0.057034	-0.18962
TAS2R13	-0.033766242	-0.060619864	-0.0719	0.013088	-0.01764	0.039731
TAS2R14	0.028126657	0.064992708	0.121245	-0.03685	0.046488	-0.09909
TAS2R16	-0.041702761	-0.089711204	0.096629	0.002348	0.021187	-0.07114
TAS2R19	-0.052320582	-0.032936713	0.003549	-0.00284	0.015473	0.043985
TAS2R3	-0.034974828	-0.073410569	-0.15812	0.014807	-0.02117	0.106348
TAS2R38	-0.02950391	-0.029412218	0.00083	0.009979	-0.05372	-0.02797
TAS2R39	-0.037439769	0.036592949	0.076836	0.003661	0.015139	-0.10788
TAS2R4	-0.021200904	-0.024822041	0.046946	-0.00068	-0.0072	-0.07905
TAS2R40	-0.067792776	-0.012157259	0.012927	-0.03102	0.098574	0.012985
TAS2R41	-0.035699863	-0.005593856	-0.0844	0.000959	-0.00131	-0.1261
TAS2R45	-0.042742362	0.029791851	-0.08722	0.006941	-0.00023	-0.09197
TAS2R5	-0.03263261	-0.047389971	-0.05423	0.039251	-0.09491	0.000404
TAS2R50	-0.04863713	0.029952157	-0.04041	-0.0073	0.043733	0.007953
TAS2R7	-0.038678215	-0.021259596	-0.01185	-0.00052	0.014449	-0.05973
TAS2R8	-0.031967141	-0.048066507	0.010997	0.014986	-0.03237	-0.08594
TAS2R9	-0.044727626	0.022458427	-0.03887	-0.00329	0.034127	0.00578
TASP1	-0.002239819	0.015633165	0.15677	0.005233	-0.0413	-0.31841
TAT	-0.073452105	0.017588195	0.080627	-0.04734	-0.01281	0.077293
TATDN1	0.027489998	-0.017264918	0.223698	0.040554	-0.09747	0.116974
TATDN2	0.044716115	0.111263471	0.299944	0.005778	-0.01894	-0.33337
TATDN3	0.010129071	-0.089811348	-0.25585	0.011352	-0.01588	0.090113
TAX1BP1	0.050375415	0.139318724	-0.18063	0.068948	-0.06986	-0.17087
TAZ	0.034185494	-0.101366716	0.124877	-0.03812	0.046893	-0.1357
TBATA	-0.040300386	-0.043571058	0.016277	-0.00298	0.031032	0.038803
TBC1D1	-0.05805171	-0.076945699	-0.03885	0.029677	0.167994	0.023585
TBC1D10A	0.013511697	-0.026969859	0.214752	-0.03053	0.078574	-0.14964
TBC1D10B	0.040147953	0.028307424	-0.21913	0.042166	-0.16173	0.217931
TBC1D10C	0.025088686	0.066929841	0.172954	-0.019	0.051061	-0.32677
TBC1D12	-0.021552743	-0.090307305	-0.24737	-0.11948	0.296399	0.395015
TBC1D13	0.021469528	-0.010729414	0.022319	-0.00018	-0.02757	-0.12345
TBC1D14	0.052086871	-0.026218379	-0.14748	-0.00335	0.024772	-0.01254
TBC1D15	0.028007843	0.006241537	0.110254	-0.02974	0.077817	-0.1657
TBC1D16	0.001770973	-0.07425399	-0.06111	0.003198	0.017799	0.049235
TBC1D17	-0.00388152	0.02071473	0.031632	-0.01217	0.037262	0.003039
TBC1D19	-0.009948755	0.144079277	0.079803	0.040582	-0.1153	-0.26819
TBC1D2	0.008509005	-0.083449226	-0.21933	-0.07044	0.218209	0.724741
TBC1D20	0.02498825	0.12409625	-0.0383	-0.03572	0.052644	-0.10099
TBC1D21	-0.038472465	-0.015206013	-0.24687	0.048742	-0.10386	0.221133
TBC1D22A	0.023151451	-0.060933821	-0.1163	-0.02057	0.061635	0.179175
TBC1D22B	-0.034653254	0.015914203	0.029584	0.050834	-0.07134	-0.12469
TBC1D23	0.04061836	0.072750788	-0.25831	-0.00849	0.025606	0.054739
TBC1D24	0.002784415	0.062324196	0.136049	0.036135	-0.10508	0.062599
TBC1D25	0.01698009	0.005535285	0.13478	-0.00856	-0.09036	-0.26314

TBC1D27	-0.021241387	-0.116166421	-0.1027	-0.02361	0.059929	-0.0754
TBC1D28	-0.035795347	-0.039198077	0.019172	0.007182	-0.00331	0.016402
TBC1D29	-0.035002475	-0.055518067	-0.02324	-0.00178	-0.01739	-0.08076
TBC1D2B	0.021950624	-0.097054296	0.143907	0.004833	-0.03664	0.085262
TBC1D30	-0.019122834	0.095965827	-0.20409	-0.02179	0.066815	0.177065
TBC1D31	0.002975632	0.27358343	0.177573	0.067334	-0.18264	-0.12378
TBC1D32	-0.012677988	-0.022607899	0.094429	-0.02508	0.073123	-0.26924
TBC1D4	0.003160146	-0.131633528	0.270337	-0.05361	0.146509	-0.12925
TBC1D5	0.028172534	0.004251581	-0.0754	-0.0088	0.019783	0.02339
TBC1D7	-0.000291289	0.022103165	-0.15211	0.094281	-0.27114	0.145564
TBC1D8	0.008961532	-0.138863655	-0.30985	-0.06849	0.207395	0.486403
TBC1D8B	-0.035727132	-0.078042267	0.015891	-0.01633	-0.02386	-0.12045
TBC1D9	-0.001328908	-0.16994797	-0.35403	-0.10526	0.295802	0.315482
TBC1D9B	0.041963814	-0.080469396	-0.00898	-0.03263	0.067641	0.002352
TBCA	0.070046262	0.04410277	0.227949	0.07289	-0.18813	0.060517
TBCB	0.066898849	0.143535752	0.013465	0.029817	-0.06136	0.076524
TBCC	0.056959989	0.041145666	0.198619	-0.05078	0.118896	-0.32399
TBCCD1	0.013622903	0.145133788	0.040233	0.042828	-0.12773	0.066808
TBCD	-0.002788166	0.004634448	0.065298	-0.00019	-0.03705	-0.02193
TBCE	0.017688689	0.050227464	0.00038	0.076753	-0.18346	0.151891
TBCEL	-0.066425091	0.028499528	-0.03443	0.025395	-0.02706	-0.061
TBCK	0.016957625	-0.003853744	0.125255	0.00427	0.00834	-0.00848
TBK1	0.0491541	0.185954906	-0.23886	0.064393	-0.15078	0.035373
TBKBP1	-0.008602283	-0.037598526	0.064131	-0.02317	-0.00633	-0.10809
TBL1X	0.016184076	0.032175395	-0.2098	-0.00321	0.044712	0.143652
TBL1XR1	-0.009645472	0.020121349	-0.00273	0.020712	-0.11731	-0.20984
TBL1Y	-0.026360399	-0.031800883	-0.10021	-0.0166	0.064234	0.100903
TBL2	0.028007039	0.046328897	-0.12118	0.079546	-0.21012	0.144565
TBL3	0.000717165	-0.011757624	-0.14531	0.054669	-0.16074	0.168904
TBP	0.041007933	0.028796341	0.089308	0.047509	-0.17809	-0.25841
TBPL1	0.067936848	0.204663249	0.130046	0.064045	-0.18594	-0.39183
TBR1	-0.055608097	-0.046833631	0.048281	0.010915	-0.02103	-0.05196
TBRG1	-0.022261402	0.048936862	0.135877	-0.03007	0.10612	0.231667
TBRG4	-0.020007789	-0.039414399	0.339617	-0.03047	0.069461	-0.03198
TBX1	-0.004578326	0.04521417	-0.05174	0.031024	-0.10572	0.003003
TBX10	-0.051134068	-0.029993556	-0.134	0.019824	-0.06203	0.013687
TBX15	-0.034465248	-0.023609019	-0.15216	-0.00839	0.042415	0.08997
TBX18	-0.098008545	-0.083925071	-0.05238	-0.02704	0.033383	-0.01002
TBX19	-0.002685047	-0.009860844	-0.10608	0.00536	-0.03519	0.023095
TBX2	-0.052682208	-0.064621954	0.064786	0.020817	-0.02038	0.057228
TBX20	-0.050597832	-0.036173879	-0.04174	0.012022	-0.02861	-0.12232
TBX21	0.006142396	0.296264383	0.233583	-0.01828	0.017665	-0.21902
TBX22	-0.151450901	-0.070003785	0.026239	-0.00039	0.056813	-0.17916
TBX3	-0.013826753	-0.112314536	-0.0176	-0.00075	-0.11208	-0.03281
TBX4	-0.035831744	-0.113736759	-0.05522	0.024135	-0.05974	0.060572
TBX5	-0.065030436	-0.050998174	-0.04976	-0.00525	-0.02399	0.061985
TBX5-AS1	-0.049291151	-0.005085811	-0.07874	0.005197	0.050405	0.07023
TBX6	-0.038508014	-0.021691484	0.111804	0.024265	-0.05783	-0.00713
TBXA2R	-0.047388482	-0.116505744	0.267053	-0.09501	0.197589	-0.15263
TBXAS1	0.003209163	-0.000643382	-0.43422	-0.0467	0.125982	0.418238
TC2N	0.023613219	0.076702069	0.386439	-0.03679	0.093251	-0.28762

TCAIM	-0.030171818	0.017170442	-0.16036	0.016844	-0.09271	0.42022
TCAM1P	-0.026564585	-0.007536133	-0.02478	0.013666	-0.03671	-0.05062
TCAP	-0.014555583	-0.106525923	0.035927	0.006126	-0.06383	-0.08417
TCEA1	-0.011601551	-0.079174592	0.043392	-0.01272	-0.05571	-0.21011
TCEA2	0.034282513	-0.051948577	0.020701	0.034536	-0.20981	0.253756
TCEA3	-0.011968705	-0.134951277	0.338408	-0.0492	0.143042	-0.22228
TCEAL1	0.004321012	0.064730592	0.16149	-0.08782	0.255054	-0.03183
TCEAL2	-0.037422268	-0.15841459	0.360738	-0.02639	0.087775	-0.1212
TCEAL3	0.01946343	0.040733315	0.24819	-0.16266	0.447536	0.012167
TCEAL4	0.015537227	-0.069190431	0.093768	-0.12253	0.362084	0.247391
TCEAL7	-0.075086394	-0.095204594	0.110148	-0.04533	0.120241	-0.06267
TCEAL8	0.009924374	0.079351289	0.12361	-0.07111	0.209215	0.004149
TCEANC	-0.088979205	0.009200636	0.123605	-0.05097	0.004199	-0.03856
TCEANC2	-0.032436457	-0.002223725	-0.03586	-0.01461	-0.04184	0.008828
TCEB1	0.04781104	0.064956738	-0.05721	0.096726	-0.26833	0.190781
TCEB2	0.065409857	0.014765701	-0.01445	0.090494	-0.27311	0.0801
TCEB3	0.025408217	-0.028801823	-0.12583	0.034531	-0.09548	0.210072
TCEB3-AS	0.012443597	-0.016219616	0.235808	0.020288	-0.05014	-0.10394
TCEB3B	-0.053114496	-0.10200336	-0.00481	0.010215	-0.00827	0.010197
TCERG1	-0.016318065	-0.022489655	0.063178	0.032621	-0.04612	0.060105
TCERG1L	-0.038994264	-0.063543655	0.028356	-0.00709	0.042447	0.024166
TCF12	0.017954468	0.033149865	0.075604	-0.07111	0.141177	0.09572
TCF15	-0.015768793	-0.067359885	-0.03502	0.022694	-0.0741	-0.03384
TCF19	0.002452377	0.071660658	0.051667	0.107451	-0.32137	-0.1939
TCF20	0.026537096	0.003605167	0.06417	0.016938	-0.07336	-0.2063
TCF21	-0.0608983	-0.099390382	-0.03789	0.012321	-0.02111	-0.02491
TCF24	-0.182566238	-0.115402833	-0.04951	-0.00298	0.096865	0.04695
TCF25	0.08643088	0.206627256	-0.14273	0.012626	0.062342	0.004752
TCF3	-0.054423798	-0.07298425	0.027862	-0.00345	-0.10621	-0.13983
TCF4	0.007276727	-0.043491921	-0.29226	-0.02023	0.055693	-0.09204
TCF7	0.014189834	-0.046256669	0.532676	-0.08969	0.246219	-0.30861
TCF7L1	-0.026435729	-0.082656335	0.103348	-0.01246	0.043162	-0.15879
TCF7L2	-0.011161401	-0.020951597	-0.15713	-0.1069	0.354771	0.400069
TCFL5	0.012606063	0.021345274	0.000832	0.007153	-0.00587	0.016424
TCHH	-0.014253546	0.003230011	0.009269	-0.00611	0.019639	-0.01236
TCHP	0.025622865	0.121502277	0.011858	0.014367	-0.05229	0.143983
TCIRG1	0.041130141	0.023363995	-0.38124	-0.029	0.102162	0.271973
TCL1A	0.00138825	-0.103838835	-0.0928	-0.06668	0.178315	-0.15323
TCL1B	-0.017981453	-0.070287957	-0.16025	-0.01536	-0.02148	-0.25194
TCL6	-0.031442039	-0.131718867	-0.1455	-0.01723	0.002374	-0.17485
TCN1	-0.00662852	-0.060920045	0.246926	-0.05577	0.151784	-0.18971
TCN2	-0.014521544	-0.131850284	-0.18778	-0.04446	0.118168	0.636602
TCOF1	5.40E-05	0.019141449	0.17588	0.104252	-0.18255	-0.07893
TCP10	-0.030153888	-0.005809942	-0.02451	0.016393	-0.00947	-0.04062
TCP10L	-0.050278314	-0.015959066	0.020325	0.011647	0.005389	-0.03669
TCP11	-0.03551438	-0.043671827	-0.02137	0.052192	-0.13105	-0.066
TCP11L1	0.00142356	0.004367779	-0.13151	0.049923	-0.141	0.161895
TCP11L2	-0.011580124	-0.041346291	-0.00995	-0.07306	0.090941	-0.11225
TCRBV12S	-0.016663108	-0.149912732	0.531851	-0.08297	0.247518	-0.13749
TCRBV15S	-0.010883977	-0.136024498	0.576736	-0.08309	0.239685	-0.22052
TCTA	0.033440026	0.055804152	0.090212	-0.03397	0.054492	-0.04289

TCTE1	-0.030924978	-0.087962416	-0.04172	0.01088	-0.00635	-0.00883
TCTE3	-0.070916066	-0.067822837	-0.05812	-0.01082	-0.003	-0.08985
TCTEX1D1	-0.055815806	-0.092401264	-0.0079	-0.02222	0.084495	0.097089
TCTEX1D2	0.015418944	0.047552492	0.141183	0.072781	-0.2029	-0.14347
TCTN1	-0.015212319	-0.147646709	-0.05904	-0.00701	-0.05353	-0.26294
TCTN2	-0.003594921	-0.058081668	0.025681	0.006592	-0.06726	-0.16036
TCTN3	0.024631178	-0.003000722	0.076941	0.027402	-0.09963	-0.15448
TDG	0.054704938	0.142829881	-0.00297	0.054729	-0.15241	0.023369
TDH	-0.040359909	0.013847151	-0.07811	0.010087	-0.00016	-0.0921
TDO2	-0.026752656	-0.036563249	0.015517	0.126086	-0.12072	-0.01285
TDP1	0.02970376	0.013569037	0.245697	-0.01143	-0.14342	-0.24871
TDP2	0.055121283	0.102260862	-0.00423	0.023827	-0.04839	-0.30976
TDRD1	-0.031475519	-0.020570165	0.006266	0.002092	0.005151	-0.07329
TDRD10	-0.029652405	-0.052767249	-0.0453	-0.00687	-0.03319	-0.11486
TDRD12	-0.026239402	0.000942326	0.146801	0.017646	-0.06262	-0.13917
TDRD3	0.008915637	0.027296772	0.113176	0.015683	-0.02703	-0.04269
TDRD5	-0.039794605	-0.015594964	-0.06819	0.004229	0.005902	-0.0021
TDRD6	-0.047568982	-0.097038596	0.006922	-0.02539	0.100349	0.218405
TDRD7	0.01930133	0.033980528	-0.15826	-0.02179	0.077303	0.042304
TDRD9	-0.003964423	-0.06620817	-0.17443	-0.0287	0.087304	0.656575
TDRG1	-0.010896586	-0.018709179	0.006288	0.04434	-0.15982	0.015985
TDRKH	-0.039389257	0.074182049	0.136513	0.091068	-0.2435	-0.11803
TDRP	0.008688695	0.004435657	-0.03234	-0.19642	0.34601	-0.03809
TEAD1	-0.014213065	-0.042362532	-0.00922	-0.03666	0.12344	0.055555
TEAD2	-0.031586434	-0.102371672	-0.13804	-0.016	0.017707	-0.04494
TEAD3	-0.038263542	0.025179015	0.013855	0.013026	-0.00642	-0.04983
TEAD4	-0.037550044	-0.025789803	-0.05828	0.007103	-0.05863	0.015363
TEC	-0.024517872	-0.079298395	-0.06145	-0.00749	-0.0054	-0.01278
TECPR1	0.009731037	-0.103177359	0.210294	-0.07193	0.11714	-0.02954
TECPR2	0.00771937	-0.032902253	-0.19894	-0.0359	0.078052	0.065195
TECR	0.010028865	0.00348847	0.372274	0.02306	-0.05021	-0.16418
TECRL	-0.086092362	-0.109094912	-0.01323	0.14708	-0.01339	-0.01618
TECTA	-0.046765729	-0.07119613	0.065269	0.005046	0.010708	-0.06705
TECTB	-0.028028292	-0.0165323	0.018483	0.014045	-0.09841	-0.07671
TEDDM1	-0.034341257	-0.054382074	0.092343	0.01176	-0.01688	-0.13271
TEF	-0.037284627	-0.025529299	-0.12579	-0.02134	0.084495	0.055434
TEFM	0.02645031	0.077972723	0.016977	0.014837	-0.03986	-0.023
TEK	-0.041095945	-0.06639645	-0.02987	0.019332	-0.10883	0.011013
TEKT1	-0.03849564	-0.071596912	-0.00345	0.00745	0.000521	0.040245
TEKT2	-0.024973536	-0.003609754	-0.08968	0.045413	-0.09061	0.026967
TEKT3	-0.042213623	-0.001655895	0.051289	-0.00769	0.040047	0.055818
TEKT5	-0.044475568	-0.111064103	-0.10431	0.014779	-0.01204	0.166288
TELO2	0.013509429	-0.12996925	-0.0652	-0.02318	0.003163	0.114411
TENC1	-0.031002229	0.024604666	0.052684	-0.01172	-0.01395	-0.19286
TENM1	-0.020161512	0.126673442	0.181425	0.034859	-0.0517	-0.13258
TENM2	-0.017950493	-0.018181219	0.026618	0.086559	-0.2289	-0.03323
TENM3	-0.032224086	-0.096764957	-0.08987	-0.06661	-0.30109	0.102043
TENM4	-0.021803087	-0.118619377	-0.15808	-0.00807	0.028254	-0.07481
TEP1	0.01982636	0.006497313	-0.18024	-0.04485	0.1514	0.20457
TEPP	-0.022975322	-0.097628814	0.325668	-0.01488	0.071554	-0.04188
TERF1	0.036649455	0.230972871	0.159692	0.014228	-0.0614	-0.21264

TERF2	0.002529163	-0.016520446	-0.06212	0.061138	-0.10886	-0.05511
TERF2IP	0.108951628	0.244769025	0.243738	0.085454	-0.17984	-0.62549
TERT	-0.036493034	-0.024689208	-0.16967	0.052176	-0.15522	-0.05764
TES	0.030929865	0.196914822	0.124313	-0.00036	-0.0069	-0.05303
TESC	-0.000715243	0.009595998	-0.19101	0.030971	-0.09698	0.50384
TESK1	0.019912197	0.074659011	-0.03366	0.007219	-0.05641	-0.03639
TESK2	0.003365065	-0.096483227	-0.07595	-0.03944	0.063315	-0.11129
TESPA1	0.002904371	0.017532668	0.457246	-0.06492	0.15452	-0.38429
TET1	-0.018102488	0.087842301	0.151327	-0.02266	0.077321	-0.26419
TET2	0.004548886	-0.006422726	-0.34023	-0.06029	0.127532	0.272169
TET3	-0.014700153	-0.051637084	-0.20058	-0.01174	-0.01521	0.286687
TEX10	0.01683305	0.049306716	0.179872	0.056741	-0.16202	-0.22145
TEX101	-0.025891934	-0.110229789	0.016273	-0.01209	0.033418	0.045602
TEX11	-0.024639465	-0.027717213	0.028125	0.095025	-0.26348	-0.05956
TEX12	-0.051760083	-0.075306936	-0.08104	0.015208	-0.042	0.110692
TEX13A	-0.048294201	-0.068477341	-0.07863	0.015632	-0.00276	0.036451
TEX13B	-0.03794054	0.008220252	-0.05434	-0.00428	-0.01179	-0.00729
TEX14	-0.026123775	-0.016006323	-0.03015	0.038122	-0.09512	-0.01033
TEX15	-0.0471189	-0.046939614	0.041749	-0.00712	0.000407	-0.16468
TEX19	-0.01685837	-0.013332776	-0.00621	0.188404	-0.28409	-0.02482
TEX2	0.020176088	-0.050243722	-0.06374	-0.00506	-0.01285	-0.07153
TEX22	-0.033808053	-0.063440844	-0.0182	0.006909	-0.0667	-0.03787
TEX26	-0.044432652	0.019683542	-0.1887	-0.0062	0.040429	0.019452
TEX26-AS	-0.034607941	-0.035474433	-0.17933	-0.01171	0.051467	0.023496
TEX261	0.032672371	-0.030980357	0.02567	0.017783	-0.12213	-0.01813
TEX264	0.016280007	0.020676104	0.064261	0.01671	-0.04808	0.161213
TEX28	-0.031083788	-0.014108642	-0.08389	0.02252	-0.0444	-0.04304
TEX29	-0.033492341	-0.097144786	-0.05546	-0.00187	0.030181	0.070769
TEX30	0.010937817	0.059169964	0.081214	0.1148	-0.36158	-0.25586
TEX33	-0.063265109	-0.004899451	-0.09834	-0.00928	0.000659	0.089897
TEX35	-0.030907482	-0.074234275	-0.00765	0.005912	-0.01271	0.021116
TEX36	-0.04260243	-0.101309296	0.029708	0.005185	0.006934	0.03478
TEX36-AS	-0.062876263	-0.002411171	0.011314	0.021805	-0.0741	0.099474
TEX37	-0.034320116	-0.01203551	-0.11913	0.018911	-0.07686	0.033569
TEX38	-0.038011188	-0.045874425	0.070072	-0.00426	0.040643	0.021525
TEX40	-0.019968216	-0.08256325	-0.05336	0.019064	-0.09662	-0.01315
TEX41	-0.025335621	-0.067644966	-0.01561	-0.01206	0.026533	-0.01789
TEX43	-0.045872431	0.021964961	0.084689	0.012596	-0.03299	-0.06965
TEX9	-0.083682069	-0.018840693	-0.09612	-0.10659	0.241408	-0.17805
TF	-0.033027402	0.033188831	-0.00011	0.031033	-0.06601	-0.07056
TFAM	0.023446182	0.009122681	0.068691	0.048843	-0.14337	0.162538
TFAP2A	-0.053434619	-0.056086893	-0.01639	0.049774	-0.16792	########
TFAP2A-AS	-0.029594311	-0.046410858	0.053721	0.101807	-0.35106	0.043294
TFAP2B	-0.032122455	-0.053644723	-0.04348	-0.12088	0.006654	0.120825
TFAP2C	-0.04753613	-0.04321989	-0.09693	0.005678	0.003701	-0.0012
TFAP2D	-0.034905686	-0.079182735	0.070919	0.007311	0.005013	0.116208
TFAP2E	-0.014436824	0.052321189	0.099158	-0.0236	-0.01073	-0.04592
TFAP4	-0.013969651	-0.065177471	-0.02433	-0.00109	-0.08532	-0.09424
TFB1M	0.010405143	-0.04199123	0.159031	0.065467	-0.1496	-0.14108
TFB2M	-0.024144663	-0.085160975	0.050375	0.009669	-0.00952	-0.13761
TFCP2	0.034926105	0.036125991	-0.18574	-0.03654	0.023001	0.264345

TFCP2L1	-0.035090283	-0.008880293	-0.02899	-0.01184	0.008522	0.023827
TFDP1	-0.037867931	-0.046959136	-0.07036	0.121907	-0.40876	0.100212
TFDP2	0.024207413	0.276445679	0.126438	-0.01505	0.016048	-0.31974
TFDP3	-0.031372742	-0.083649379	0.039911	0.05387	-0.15118	0.054363
TFE3	0.009550861	-0.075091513	-0.3285	0.029873	-0.06206	0.340922
TFEB	0.021182897	0.002134327	-0.42138	-0.0706	0.170256	0.220634
TFEC	-0.000973197	-0.107973323	-0.29314	-0.06271	0.188258	0.619557
TFF1	-0.030129317	-0.010169485	-0.13991	0.011434	-0.02381	-0.02336
TFF2	-0.03143137	-0.057412151	-0.12625	0.019093	-0.02742	0.045058
TFF3	-0.023785836	-0.076262528	-0.07801	0.000885	-0.05989	-0.09924
TFG	-0.031316574	-0.069331919	0.054754	0.036384	-0.12314	0.0752
TFIP11	0.034232174	0.156850828	-0.09501	0.026165	-0.06827	-0.08418
TFPI	-0.036678366	-0.067171547	-0.02699	-0.07641	0.071449	-0.05259
TFPI2	-0.02451675	-0.028633314	-0.02218	-0.01887	-0.00435	-0.01011
TFPT	0.017226676	-0.012080506	0.142406	0.060366	-0.19557	0.080344
TFR2	-0.088358245	-0.016360256	0.052093	-0.07633	0.005099	-0.14405
TFRC	-0.077114406	-0.11588825	-0.06529	0.077552	-0.10645	-0.10595
TG	-0.025975159	-0.070419036	-0.02243	0.045373	-0.13962	0.041054
TGDS	0.00501117	0.120894041	0.182141	0.050931	-0.12496	-0.2099
TGFA	-0.03436905	0.015513102	-0.09208	-0.08651	-0.00337	-0.06582
TGFB1	0.006746167	0.074227359	-0.04486	-0.02732	0.092401	0.118352
TGFB1I1	-0.017831594	-0.029057845	0.074609	-0.25511	0.728845	0.05931
TGFB2	0.013725176	-0.06212563	0.049402	-0.01868	0.051519	-0.12515
TGFB3	-0.035954854	-0.070466241	0.080336	-0.02656	0.064015	-0.14732
TGFBI	0.013059353	-0.119404863	-0.27759	-0.08412	0.253505	0.470626
TGFBR1	0.020960314	0.163503577	0.047409	0.029417	-0.07064	-0.26359
TGFBR2	0.026723856	-0.016345333	0.06799	-0.05491	0.151891	-0.25944
TGFBR3	0.008611993	0.284777333	0.405729	-0.01295	0.025423	-0.34893
TGFBRAP1	0.049631428	0.026006913	0.099075	0.01796	-0.07184	-0.0288
TGIF1	-0.078870999	-0.056114521	-0.08257	-0.00498	0.013319	-0.00824
TGIF2	0.013106734	-0.050262754	0.031602	0.028783	-0.11017	-0.09249
TGIF2LY	-0.034918677	-0.036690406	-0.08517	0.00257	0.010537	0.004966
TGM1	-0.030139687	-0.043678229	-0.04839	0.003315	-0.00033	0.126264
TGM2	-0.012572516	0.007799338	-0.17936	0.009723	0.037217	0.145142
TGM3	-0.025256288	-0.014821548	-0.0363	-0.0073	0.006011	-0.10033
TGM4	-0.043309198	0.04720519	0.035348	0.064535	-0.20002	-0.04007
TGM5	-0.016712121	-0.06117984	-0.00763	0.025069	-0.11131	0.068598
TGM6	-0.034039588	-0.089038321	-0.06589	0.000729	-0.02682	0.00125
TGM7	-0.045931424	0.021592005	-0.00427	0.025035	-0.03998	0.024851
TGOLN2	0.038405601	0.086398396	-0.02024	-0.06248	0.148043	0.239878
TGS1	0.019707483	0.044207091	0.115858	0.062694	-0.18576	-0.10592
TH	-0.034192784	-0.088222699	-0.10483	0.000351	0.021301	-0.01368
THADA	-0.015392799	-0.048379953	0.051806	-0.04564	0.02364	-0.08733
THAP1	0.019439298	0.065773801	-0.01399	-0.00967	0.017971	-0.01379
THAP10	-0.032439663	-0.050918259	-0.05709	0.005817	-0.00167	0.190026
THAP11	0.052890261	0.045014289	0.308051	0.00495	-0.03572	-0.15034
THAP2	0.010795035	-0.040938324	-0.00994	0.06393	-0.00903	0.153342
THAP3	-0.010954931	-0.105727645	0.017478	0.029921	-0.12018	-0.05994
THAP4	0.042907551	-0.018221256	0.191965	0.066055	-0.22626	-0.13109
THAP5	0.009507895	0.063700867	-0.08556	0.017751	-0.06471	-0.17467
THAP6	0.010578592	0.019071213	0.0158	0.027994	-0.03988	-0.00243

THAP7	0.023754763	-0.070378643	0.231708	0.050473	-0.1189	0.090018
THAP7-AS:	-0.037204223	-0.051066829	0.225688	0.007151	-0.01292	-0.11152
THAP8	-0.011988104	0.019230205	-0.05966	-0.01427	0.022829	0.172139
THAP9	-0.020775248	0.010908639	-0.04623	-0.00345	0.032628	0.124846
THAP9-AS:	0.042604638	0.048764756	-0.07298	0.048914	-0.12397	-0.22471
THBD	0.014857578	-0.086004471	-0.258	-0.05621	0.105239	0.072733
THBS1	0.010408101	-0.036103618	-0.14005	-0.04574	0.164042	0.334743
THBS2	-0.034883237	0.002615959	0.050012	-0.00059	0.026553	0.090955
THBS3	-0.002330685	-0.08092943	-0.03543	-0.00309	0.010833	0.116099
THBS4	-0.019964273	-0.100098233	-0.0752	0.005416	-0.0626	-0.04294
THEG	-0.025833919	-0.026483191	-0.06249	0.010155	-0.04931	-0.00571
THEG5	-0.027572122	-0.087901105	0.065928	0.026083	-0.07002	-0.08968
THEM4	0.014120091	-0.000625709	0.382738	0.019555	0.007694	-0.32387
THEM5	-0.049405941	-0.036735409	-0.02319	0.026602	-0.06736	0.019397
THEM6	-0.003220266	0.012286515	0.15505	0.045973	-0.13345	-0.05765
THEMIS	-0.008019572	-0.1141932	0.643319	0.001756	-0.01778	-0.25884
THEMIS2	0.023223652	0.031903894	-0.3024	-0.09732	0.276395	0.261229
THG1L	0.021284021	-0.096872763	-0.00934	0.044486	-0.15527	0.362074
THNSL1	-0.019573728	-0.005195032	0.316433	0.016459	-0.03469	-0.46016
THNSL2	-0.041632933	-0.135916812	-0.0847	-0.01602	0.040869	-0.0467
THOC1	0.044980339	0.048838107	0.165365	0.059069	-0.18139	-0.20487
THOC2	0.043652678	0.120950619	0.146103	0.050683	-0.11095	-0.08136
THOC5	0.013335314	-0.012519843	-0.23184	0.060487	-0.17902	0.03241
THOC6	0.006005551	-0.040452177	-0.02027	0.095213	-0.26103	0.227977
THOC7	0.067313169	0.058824192	0.115317	0.092684	-0.26049	0.161318
THOP1	-0.00316723	-0.053472573	0.031227	0.108861	-0.30952	-0.01388
THPO	-0.050628657	-0.060137723	0.033789	0.170356	-0.02437	0.056609
THRA	-0.048027643	-0.015789873	0.20026	0.013999	0.111453	########
THRAP3	0.041907455	0.054069994	-0.08659	0.036336	-0.12667	-0.14174
THRB	-0.056193148	-0.115095587	-0.06529	-0.01303	-0.01538	-0.01039
THRSP	-0.032963322	-0.054094763	-0.07118	0.017887	-0.05343	0.113703
THSD4	0.014954865	-0.024607804	0.00853	0.09882	0.00016	-0.16184
THSD7A	-0.063638784	-0.048958055	0.215637	-0.01015	-0.00581	-0.08229
THSD7B	-0.046445258	-0.042616601	-0.02783	0.007903	0.003124	0.000173
THTPA	0.013595566	-0.005625004	-0.11755	-0.01402	0.00601	0.282482
THUMPD1	0.06084491	0.155077101	0.304723	0.007928	-0.02557	-0.31651
THUMPD2	0.015706593	-0.016469824	0.032443	0.051217	-0.10963	-0.01684
THUMPD3	0.023092602	0.058361489	0.218544	0.055054	-0.2046	0.118028
THUMPD3-/	-0.000702275	0.022504187	0.05576	0.024281	-0.03408	-0.1851
THY1	-0.010798552	-0.068252405	0.066387	-0.02932	-0.1322	-0.10307
THYN1	0.019774641	-0.002744156	0.190009	0.035684	-0.08992	-0.03922
TIA1	0.024075402	0.008442048	0.063231	-0.00744	0.127556	-0.13522
TIAF1	-0.028861969	-0.034759561	-0.06803	0.005838	-0.0049	-0.08001
TIAL1	0.029726605	0.070720735	0.240502	-0.00028	0.02066	-0.14312
TIAM1	0.005753883	-0.009577908	-0.02158	-0.02765	0.076687	0.391507
TIAM2	0.09428321	0.113105535	-0.02809	0.018627	-0.07042	0.149385
TICAM1	0.009928088	-0.023725949	-0.17688	-0.00204	-0.02468	0.278619
TICRR	-0.025771462	-0.043155988	-0.09253	0.125335	-0.41573	-0.02921
TIE1	-0.030633174	0.135504913	-0.05277	0.128425	-0.31977	-0.06954
TIFA	-0.03020297	-0.012738012	-0.10677	0.048312	-0.12469	-0.20502
TIFAB	-0.006668714	-0.059218649	-0.08153	-0.0231	0.04807	-0.03312

TIGD1	0.001206286	-0.07195773	0.24503	0.008616	0.029119	-0.11308
TIGD2	0.013988836	0.119019339	-0.03566	0.054175	-0.10655	0.026538
TIGD3	-0.014113813	-0.102487633	-0.23759	-0.0166	0.005569	-0.04726
TIGD4	-0.062745744	-0.021974876	0.103762	0.060336	-0.14887	-0.02658
TIGD5	0.013081804	-0.028565265	0.052846	-0.02358	-0.0047	-0.0064
TIGD6	-0.066155031	-0.01641334	0.015542	-0.02206	0.038453	0.162804
TIGD7	-0.010041051	0.119344777	0.066098	-0.07069	0.199949	-0.29994
TIGIT	0.001517266	0.195003443	0.380138	-0.05154	0.121053	-0.30442
TIMD4	-0.027042634	0.065992026	0.122427	0.044414	-0.11261	-0.16587
TIMELESS	-8.32E-05	0.078059889	-0.06362	0.088883	-0.25055	-0.07509
TIMM10	0.033979614	-0.01540312	0.062525	0.087441	-0.24829	0.107971
TIMM10B	0.036783961	0.125569366	0.187683	-0.00381	-0.0298	-0.09148
TIMM13	0.01215715	-0.0233692	0.035507	0.032879	-0.0824	0.3049
TIMM17A	0.020815493	0.049789936	-0.0283	0.095463	-0.25217	0.181958
TIMM17B	0.010044151	-0.117921819	-0.15583	0.038997	-0.1485	0.172965
TIMM21	0.025393833	0.070272048	0.113864	0.070231	-0.17387	0.067059
TIMM22	-0.001967094	0.026788839	0.289647	0.050663	-0.15429	-0.03144
TIMM23	-0.029978273	0.010227187	-0.05846	-0.01626	0.055824	0.005266
TIMM23B	0.0099755	-0.001481333	0.142022	0.025972	-0.09841	-0.11873
TIMM44	0.012381851	-0.000632615	0.037377	0.075392	-0.23703	0.058974
TIMM50	0.009961583	0.025333622	0.05779	0.088577	-0.24537	0.165695
TIMM8A	-0.01229687	-0.021405442	0.126733	0.034187	-0.09102	-0.11222
TIMM8B	0.052312377	0.082651553	-0.11262	0.069735	-0.19624	0.243779
TIMM9	0.025351227	-0.041090616	0.184745	0.034261	-0.08123	0.088007
TIMMDC1	0.047859441	0.005632506	-0.01261	0.023126	-0.07421	0.086676
TIMP1	0.022844794	0.049798338	-0.04991	-0.00408	0.039097	0.439886
TIMP2	0.008544966	-0.162255361	-0.36431	-0.09028	0.262315	0.419801
TIMP3	-0.038061841	-0.064940342	-0.02264	-0.00477	-0.06898	0.015138
TIMP4	-0.032609838	-0.050928139	-0.02173	0.004675	0.004771	-0.04141
TINAG	-0.039603065	-0.008987453	-0.03946	0.000131	0.04458	-0.04048
TINAGL1	-0.017528496	-0.044868455	0.014082	-0.01056	-0.06858	-0.08985
TINCR	-0.013422956	-0.144449823	-0.05616	-0.03374	-0.04455	-0.01225
TINF2	0.100987339	0.270372397	-0.20669	-0.00737	0.03369	-0.22239
TIPARP	0.042186832	0.139713622	0.050132	0.031021	-0.06968	0.045876
TIPARP-AS	-0.028074797	0.033906643	0.092655	0.000958	0.014332	-0.00595
TIPIN	-6.21E-05	0.050263561	0.059951	0.120763	-0.37185	0.008192
TIPRL	0.030442757	0.112850157	-0.06494	0.119638	-0.33257	0.050382
TIRAP	-0.019802588	0.052459115	-0.0822	-0.01208	0.153794	0.312233
TJAP1	0.048237991	-0.046347817	0.152349	0.010963	-0.0667	-0.1806
TJP1	-0.043200317	-0.081762058	0.060769	0.033572	-0.08093	-0.01558
TJP2	-0.012838687	-0.001164353	-0.05198	-0.0668	0.028816	0.123435
TJP3	-0.027370617	-0.108758683	0.147095	-0.00488	0.011011	-0.0932
TK1	-0.009897698	0.01816765	0.015252	0.161205	-0.46183	-0.07309
TK2	0.01314419	-0.007079629	-0.19794	-0.00579	0.053052	0.472632
TKT	0.027600851	-0.125538932	-0.3089	-0.00959	0.043553	0.495402
TKTL1	-0.002051363	0.258140664	0.177076	-0.05459	0.141611	-0.34038
TKTL2	-0.042025267	-0.048514818	-0.19207	0.010403	-0.00674	0.002553
TLCD1	-0.010001294	-0.022069932	-0.04929	0.031459	-0.09649	-0.04881
TLCD2	-0.014781867	-0.013681286	0.004671	-0.00244	0.027762	-0.03821
TLDC1	0.037286027	0.016220858	0.09802	0.081147	-0.0499	-0.04637
TLDC2	-0.023805723	-0.035597255	-0.10024	0.044821	-0.10852	0.08036

TLE1	-0.013456241	0.253525955	-0.12349	-0.09839	0.092136	-0.28741
TLE2	-0.009715999	-0.037787033	0.231127	0.016291	-0.04386	-0.15006
TLE3	0.024124386	-0.12887041	-0.2828	0.021403	-0.02846	0.13097
TLE4	-0.012288706	-0.001622753	-0.03624	-0.04251	0.116045	0.112205
TLE6	-0.038880186	-0.065461459	-0.05267	0.104815	-0.21458	0.008262
TLK1	-0.003967761	0.058158998	-0.05657	-0.05026	0.182087	-0.08226
TLK2	0.013482739	-0.009162143	-0.02043	0.011787	-0.06175	-0.2747
TLL1	-0.043593074	-0.064537292	0.020219	0.002594	-0.05527	-0.00624
TLL2	-0.119622307	-0.045355668	-0.009	-0.10213	-0.07803	0.097311
TLN1	-0.063426366	-0.118667758	-0.19867	-0.10198	0.149625	0.22513
TLN2	-0.026549807	-0.074445828	0.121904	0.135047	-0.26468	-0.12972
TLR1	0.012397428	-0.00206645	-0.43187	-0.05666	0.206779	0.313484
TLR10	-0.005808849	-0.093465292	-0.21922	-0.07042	0.198811	-0.25277
TLR2	0.010219526	-0.106221299	-0.3456	-0.09598	0.258274	0.520268
TLR3	-0.016562613	0.127112417	0.035703	0.029366	-0.01675	-0.16005
TLR4	0.007580714	-0.097135465	-0.38764	-0.09201	0.286534	0.529097
TLR5	0.004516623	-0.096812949	-0.26212	-0.10498	0.288665	0.543057
TLR6	-0.012443967	-0.089217305	-0.33946	-0.09058	0.249512	0.245896
TLR7	-0.001164099	-0.130006423	-0.35025	-0.0958	0.282718	0.371968
TLR8	0.002587782	-0.098308953	-0.36338	-0.09768	0.280944	0.543311
TLR8-AS1	-0.044820135	-0.007907114	-0.03396	0.020995	-0.02554	0.085573
TLR9	-0.006163235	-0.075230108	-0.15011	-0.04179	0.112721	-0.25919
TLX1	-0.038093176	-0.040672327	-0.16421	0.001899	0.000802	0.057271
TLX2	-0.050939363	-0.070652384	-0.06727	0.002051	0.005722	0.074861
TLX3	-0.041371576	-0.074136462	-0.07876	-0.00641	0.03103	0.184633
TM2D1	0.005250675	0.055757253	0.065427	-0.05221	0.128922	-0.05224
TM2D2	0.039631862	0.060985543	0.083383	-0.00518	-0.0125	0.09186
TM2D3	0.055592972	0.110354664	-0.16221	0.038565	-0.0886	0.088425
TM4SF1	-0.043293979	-0.076268518	-0.05649	-0.04715	0.020163	-0.08365
TM4SF18	-0.065924315	-0.084560121	0.008336	-0.02449	0.071034	-0.00245
TM4SF20	-0.036423779	-0.040849741	0.006966	0.009835	-0.00814	0.075533
TM4SF4	-0.032284879	-0.028133505	-0.02186	0.025125	-0.06642	-0.04783
TM4SF5	-0.040808955	-0.026035347	-0.03657	0.01013	########	0.1196
TM6SF1	0.004818891	0.086097468	-0.35484	-0.04489	0.118361	0.311457
TM6SF2	0.013459047	-0.07960239	-0.01838	0.014933	-0.04465	-0.07499
TM7SF2	-0.00521987	0.011937624	0.235356	0.1042	-0.27416	-0.25165
TM7SF3	0.053709147	0.015959795	0.002089	0.03853	-0.10203	0.063435
TM9SF1	0.006410976	0.003509621	-0.1928	0.01896	-0.04464	0.195984
TM9SF2	0.07895406	0.001251526	-0.42584	0.049058	-0.09353	0.118112
TM9SF3	0.045440953	0.064208737	0.166638	-0.05436	0.11286	-0.07059
TM9SF4	0.021846577	-0.080196663	-0.21975	-0.00408	0.010274	0.53664
TMA16	-0.012042761	-0.016134347	-0.08623	0.098786	-0.25362	0.109057
TMA7	0.108643654	0.120968995	-0.12032	0.170863	-0.29243	-0.28114
TMBIM4	0.088509097	0.036745645	-0.15969	0.001049	0.043719	0.084308
TMBIM6	0.079823411	0.125088454	-0.19497	0.063092	-0.11362	-0.06863
TMC1	-0.027100875	-0.065029952	0.025798	0.00063	0.011401	-0.08264
TMC2	-0.027978369	-0.051254875	0.033796	-0.00851	-0.05388	-0.07735
TMC4	-0.019197453	0.060842417	-0.06951	0.002884	-0.02503	0.184818
TMC5	-0.038592806	-0.074692493	-0.07957	-0.01709	0.075253	0.059075
TMC6	0.025915836	0.079361391	0.29987	-0.05214	0.109157	-0.20158
TMC7	-0.097866996	-0.049003559	-0.14233	-0.0025	0.049362	0.014831

TMC8	0.00900302	0.013452095	0.370621	-0.02349	0.037468	-0.13116
TMCC1	0.080858842	0.041258504	-0.12453	0.032016	0.029374	-0.01331
TMCC1-AS1	-0.057776541	-0.005068725	-0.0295	-0.01983	0.043027	-0.063
TMCC2	-0.028498485	-0.08658685	-0.03322	-0.02119	-0.01414	-0.0884
TMCC3	0.002649541	0.090917817	-0.29215	-0.07078	0.206789	0.028362
TMCO1	0.086673161	0.106530079	0.001186	0.046584	-0.20937	0.088829
TMCO2	-0.036936454	-0.001086256	-0.02354	0.014584	-0.03782	-0.04702
TMCO3	-0.029421205	-0.013574886	-0.10542	0.057181	-0.11521	0.163357
TMCO4	-0.001611349	-0.030703984	0.111274	0.022045	-0.09155	0.041003
TMCO5A	-0.096349856	-0.067283632	-0.05305	-0.03127	-0.003	0.041811
TMCO5B	-0.034859196	-0.048159265	-0.00261	-0.00307	0.029221	-0.01386
TMCO6	0.001826793	-0.012070336	-0.02034	-0.00393	-0.02262	0.21996
TMED1	0.028117389	0.107368862	-0.22006	0.03996	-0.18001	0.351745
TMED10	0.086208764	0.139619431	-0.04264	0.04575	-0.12953	0.040041
TMED2	0.022707396	0.124160284	0.033753	0.026132	-0.10695	-0.18835
TMED3	-0.037718662	-0.075222866	-0.03784	0.03588	-0.08992	0.010984
TMED4	0.025875042	-0.033098787	0.2601	0.008397	-0.08025	-0.29717
TMED5	0.025746674	0.093890642	-0.11118	-0.07746	0.155128	0.19422
TMED6	-0.024631543	-0.016030658	0.097358	0.000396	0.0141	0.037246
TMED8	0.0151608	-0.039158388	-0.27455	0.003324	-0.03666	-0.03483
TMED9	0.094618396	0.134621968	-0.09196	0.068811	-0.17279	0.155317
TMEFF2	-0.046288458	-0.050639406	-0.04566	0.18958	-0.05879	0.003228
TMEM100	-0.039221542	-0.052421232	-0.09841	0.059501	-0.14216	0.057934
TMEM101	0.024113104	-0.017670904	0.046154	0.02803	-0.12361	0.134454
TMEM102	-0.016179406	0.053851553	0.034593	0.014418	0.002851	0.226113
TMEM104	0.015643392	-0.044834568	-0.07417	-0.02555	0.044569	0.217936
TMEM105	-0.001370093	-0.066670967	0.003343	-0.01338	-0.06933	-0.1225
TMEM106A	0.038096319	-0.008711157	-0.20508	0.007101	0.096935	0.176199
TMEM106B	0.006143054	0.044254763	0.302086	-0.0109	0.072037	0.037956
TMEM106C	0.005031457	0.067096323	0.24823	0.083303	-0.26342	-0.26169
TMEM107	-0.013114648	-0.005659232	-0.08529	0.074651	-0.05929	0.164026
TMEM108	-0.044761581	-0.052093978	0.007237	0.009145	0.001795	-0.04617
TMEM108-A	-0.017507665	-0.035744461	0.034162	-0.013	0.014773	-0.0532
TMEM109	0.03239248	0.051744915	0.089811	0.028631	-0.07324	-0.16868
TMEM11	0.045015683	0.009658439	-0.22709	0.020753	-0.09411	0.438697
TMEM110	0.006117	-0.033714514	-0.17034	0.027338	-0.12873	0.406989
TMEM114	-0.03043494	-0.066719832	-0.16321	-0.01176	0.034292	0.062153
TMEM115	0.015527812	0.020760728	-0.07644	0.018275	-0.04452	0.184092
TMEM116	0.098557374	0.083597997	0.185475	0.036174	0.040339	-0.03914
TMEM117	-0.013251806	0.097557262	0.174617	0.006425	-0.02284	-0.30987
TMEM119	-0.032084013	-0.049575036	-0.117	0.003208	0.004755	0.088067
TMEM120A	0.016653195	-0.009821758	-0.28102	0.029312	-0.09886	0.237541
TMEM120B	-0.02995288	-0.034620532	0.118892	0.014594	-0.12504	-0.10904
TMEM121	-0.026697444	-0.06383348	0.040654	0.050895	-0.12108	0.010074
TMEM123	0.073809525	-0.041233974	0.317146	0.039507	-0.04486	-0.23595
TMEM125	-0.030650448	-0.029910315	0.014325	0.009871	-0.0539	-0.11618
TMEM126A	0.038464283	0.083980397	0.170787	0.049984	-0.13332	0.158164
TMEM126B	0.031122889	0.160898073	0.218665	0.08029	-0.2257	-0.0456
TMEM127	0.051458519	-0.061201623	-0.19683	-0.03392	0.117192	0.251546
TMEM128	0.028335144	0.046645302	0.135043	0.025807	-0.09493	0.009032
TMEM129	0.027311463	-0.038259107	0.046352	0.011793	-0.02742	0.098472

TMEM130	-0.037878407	-0.060050774	0.054278	0.01018	0.003625	0.063569
TMEM131	0.034845245	0.011075678	-0.17448	0.012805	-0.00585	-0.00467
TMEM132A	-0.031161755	-0.096118234	-0.14448	0.01362	-0.1206	0.000832
TMEM132B	0.011211792	-0.084644911	-0.02123	0.110494	0.014747	-0.02639
TMEM132C	-0.027051128	0.00117187	0.027973	-0.00465	-0.02617	-0.05116
TMEM132D	-0.03330469	-0.062594601	-0.03066	0.052156	-0.13185	0.027668
TMEM132E	-0.034128687	-0.056083265	-0.02188	########	0.021673	-0.03005
TMEM133	-0.021128071	0.129291175	-0.14633	-0.01727	0.058808	-0.24941
TMEM134	-0.074940598	-0.029150962	-0.10398	-0.05141	-0.04542	0.01045
TMEM135	0.001361589	0.024272709	0.116048	0.042145	-0.13671	-0.03469
TMEM136	-0.021880954	-0.086265986	-0.13333	0.045576	-0.10326	0.037461
TMEM138	0.033521094	0.054198523	0.095578	0.012546	-0.03559	0.119024
TMEM139	-0.052753631	-0.089676534	-0.11123	0.05002	-0.12646	-0.02797
TMEM140	0.019492228	0.073712978	-0.11948	-0.08845	0.222808	-0.08528
TMEM141	0.020768915	-0.058858985	-0.01993	0.084881	-0.3007	0.128095
TMEM143	-0.006130607	-0.090634884	0.198404	0.014014	-0.15816	-0.21778
TMEM144	-0.011328936	-0.10482113	-0.22669	0.010673	0.00478	0.608918
TMEM145	-0.037382385	-0.015577302	-0.18343	0.033065	-0.06644	0.136234
TMEM147	0.036760748	-0.019451295	0.091609	0.060418	-0.15377	0.256334
TMEM14A	0.008604803	0.035639508	0.24583	-0.00972	0.017223	-0.4484
TMEM14B	0.051867343	-0.063303198	0.19049	0.076468	-0.21718	0.024758
TMEM14C	0.02179351	-0.16684681	0.013411	0.027203	-0.0679	0.221401
TMEM150A	0.006015731	-0.015066196	-0.0844	-0.03831	0.126558	0.290782
TMEM150B	-0.02496271	-0.052380608	-0.14659	0.016397	-0.03012	0.617179
TMEM150C	-0.039286006	-0.025202502	-0.01322	-0.02032	0.059594	-0.0845
TMEM151A	-0.025724579	-0.02779508	-0.05362	0.002587	-0.02613	0.034081
TMEM151B	-0.013322535	-0.132086127	-0.04248	0.060642	-0.1128	-0.02526
TMEM154	0.011546995	-0.166034378	-0.2113	-0.0319	0.08966	0.137228
TMEM155	-0.033796223	-0.092221087	0.347037	-0.00525	0.044491	-0.15783
TMEM156	-0.003081799	0.009002631	0.009924	-0.01749	0.056576	-0.4178
TMEM158	-0.011532457	-0.013560966	-0.04785	-0.18121	0.50224	0.135729
TMEM159	0.007372475	-0.035157206	-0.0808	-0.06451	0.205745	0.206867
TMEM160	0.008296933	0.061067048	0.068122	0.081829	-0.20868	0.137665
TMEM161A	0.042674941	-0.037573283	0.151131	0.06937	-0.24972	-0.21942
TMEM161B	-0.00755408	0.081630029	0.254257	0.046617	-0.11309	-0.33462
TMEM161B-	-0.005029272	0.016219592	0.239605	0.022813	-0.04143	-0.15904
TMEM163	-0.009039448	0.125554646	-0.02222	0.080942	-0.21849	-0.05712
TMEM165	-0.059237928	-0.131556374	-0.14549	0.004918	-0.1408	0.181475
TMEM167A	0.058235195	0.012234543	-0.34784	0.005813	-0.00622	0.630353
TMEM167B	0.101716088	0.053809791	-0.32235	-0.02854	0.142143	0.089744
TMEM168	0.014270341	0.036698637	-0.01116	-0.0995	0.271575	0.081913
TMEM169	-0.025480006	0.041861156	-0.02921	0.011808	-0.02941	0.029788
TMEM17	-0.022642259	-0.06312048	0.121462	-0.12173	0.369101	-0.14582
TMEM170A	0.004864629	0.073356833	0.059936	0.019933	-0.10588	0.035067
TMEM170B	0.000692555	-0.122619126	-0.31896	-0.06879	0.216504	0.566993
TMEM171	-0.059553792	0.019807881	0.128826	-0.03265	0.082688	-0.12733
TMEM173	0.015443144	0.018730179	0.152753	0.011711	-0.01379	0.322713
TMEM174	-0.03648393	-0.057549327	0.096575	0.044723	-0.1292	0.027224
TMEM175	0.012551967	-0.068965131	-0.00296	-0.04748	0.091323	-0.00221
TMEM176A	0.011358484	-0.062932444	-0.22081	-0.07886	0.197585	0.612138
TMEM176B	8.02E-05	-0.074773825	-0.2477	-0.11006	0.30515	0.656752

TMEM177	-0.023100445	-0.018829538	0.161984	0.076275	-0.19366	-0.17626
TMEM178A	-0.025311148	-0.04827657	-0.0189	8.01E-05	-0.00104	-0.19535
TMEM178B	-0.050880856	-0.044237178	0.018437	0.038144	-0.01563	-0.11171
TMEM179	-0.022712293	-0.091047976	-0.06584	0.050081	-0.12707	-0.01219
TMEM18	0.03329445	-0.013806691	0.184003	0.008511	-0.06369	0.105373
TMEM180	-0.012207575	-0.00581974	-0.00171	-0.00049	-0.01363	0.266372
TMEM181	0.036789921	0.246443692	0.218281	0.004978	########	-0.20316
TMEM182	-0.016298503	0.054044889	0.135388	0.113173	-0.25945	-0.26184
TMEM184A	-0.004284566	-0.115635037	0.004609	0.005468	-0.07935	-0.13025
TMEM184B	0.04079066	0.177600644	-0.15147	0.031898	-0.09537	-0.15107
TMEM184C	0.051716611	0.119253603	-0.14392	0.01176	-0.04377	0.190578
TMEM185A	0.020445559	0.137347938	0.043949	-0.0474	0.112899	-0.02601
TMEM185B	0.010532956	0.029938842	-0.13449	0.052662	-0.15826	-0.00581
TMEM186	0.01768374	0.030795665	0.057947	0.026524	-0.13467	0.011051
TMEM187	0.009525792	-0.000673154	0.087851	0.027809	-0.04888	0.010404
TMEM19	0.00484151	0.033432195	0.015385	0.013677	0.011244	-0.11377
TMEM190	-0.03089955	-0.095404821	0.004526	0.006829	-0.03941	0.118543
TMEM191A	0.000181325	0.026269027	0.074463	-0.04307	0.156112	0.053385
TMEM192	0.040644002	0.037728638	0.007164	-0.01528	0.037193	0.139398
TMEM194A	-0.004681796	0.007294463	0.005736	0.041108	-0.15561	-0.15776
TMEM194B	-0.007922349	0.098045756	0.002217	0.074422	-0.20599	-0.12708
TMEM196	-0.08122535	0.022253315	-0.0788	-0.03213	0.090851	0.017886
TMEM198	-0.025798071	-0.048160346	-0.04233	0.054134	-0.22305	-0.09023
TMEM198B	0.005463303	-0.061000927	0.2134	-0.04393	0.084439	-0.02104
TMEM2	0.011367401	0.123816669	-0.10529	-0.00903	-0.00255	0.039898
TMEM200A	-0.012505364	0.046858738	0.066036	0.110715	-0.23076	-0.12529
TMEM200B	0.008067742	0.054830371	0.147704	-0.03072	0.123002	-0.2417
TMEM200C	-0.055271249	-0.085096736	-0.0476	0.011795	0.006604	0.178195
TMEM201	-0.011955449	-0.033994293	-0.04715	0.072471	-0.24704	-0.09602
TMEM203	0.046464071	0.085960222	0.200695	0.022443	-0.0659	0.067605
TMEM204	0.063519127	-0.029587347	0.305071	-0.08086	0.132853	-0.31909
TMEM205	0.02853035	-0.028856817	-0.18227	0.026849	-0.06556	0.639927
TMEM206	0.022757083	-0.009957039	-0.09718	0.013076	-0.03262	0.191723
TMEM207	-0.027486397	-0.030617914	-0.02549	0.061654	-0.14884	0.023987
TMEM208	0.047479663	0.068748457	-0.05503	0.059307	-0.17591	0.295822
TMEM209	0.006981007	0.083015903	0.211667	0.099503	-0.26761	-0.07207
TMEM212	-0.02037255	-0.045500467	0.008507	-0.02729	0.045489	-0.06896
TMEM213	-0.06209454	-0.041289794	0.143076	0.012137	-0.00661	-0.0423
TMEM214	0.048975523	0.034637819	-0.00639	0.054654	-0.19188	0.13315
TMEM216	0.025485195	0.059069135	-0.05256	-0.0934	0.213502	-0.06374
TMEM217	-0.026753468	-0.005548876	-0.04779	0.009643	-0.00841	0.051386
TMEM218	-0.010631886	0.002965713	0.041913	-0.03336	0.118881	0.117935
TMEM219	0.020672708	-0.05745311	-0.04269	-0.02502	0.070662	0.26075
TMEM220	-0.011961925	-0.064128515	0.155617	-0.0352	0.117797	0.044769
TMEM221	-0.039245758	-0.05533512	0.002063	-0.02599	0.028346	-0.11741
TMEM222	0.049631287	0.028891469	0.126195	0.029653	-0.14626	-0.30844
TMEM223	-0.012163539	-0.038155222	0.002456	0.024831	-0.03643	0.060677
TMEM225	-0.03395385	-0.072316678	-0.04307	0.017621	-0.0362	-0.1313
TMEM229B	-0.027199961	0.029690738	0.035223	-0.07194	0.206114	-0.02234
TMEM230	0.051537765	0.07411631	0.203991	0.030291	-0.07237	0.000552
TMEM231	-0.024658904	-0.078423823	0.03791	-0.02363	0.064664	-0.20925

TMEM232	-0.020324637	-0.02099394	-0.06179	0.014553	-0.04722	0.019063
TMEM234	-0.026034398	-0.029195075	-0.06329	-0.02976	-0.00456	-0.07162
TMEM235	-0.039046265	-0.082658401	-0.02082	0.002017	0.023648	0.03462
TMEM237	-0.027130224	-0.007821215	0.26874	0.17072	-0.41234	-0.22381
TMEM238	-0.033157237	-0.039658122	-0.0437	-0.01325	0.051344	0.119522
TMEM239	-0.045381297	-0.090716966	-0.0854	0.008677	-0.06878	-0.05861
TMEM241	0.032185631	-0.006605839	0.038342	0.024881	-0.07279	-0.14338
TMEM242	-0.008994225	0.013424109	0.071399	-0.04739	0.139775	0.079345
TMEM243	0.034560549	-0.06593237	0.189017	0.007958	-0.0245	-0.13667
TMEM245	-0.026092769	0.068357256	0.273564	0.033017	-0.03979	-0.10561
TMEM246	-0.043159489	-0.068726429	-0.00926	0.013851	-0.01985	0.006905
TMEM248	0.091149272	0.072175518	-0.05616	0.007672	-0.00718	-0.32575
TMEM25	-0.032293006	-0.05293653	0.25008	-0.03924	0.103921	-0.19116
TMEM251	0.023098872	0.059481401	-0.15859	0.001569	-0.02792	0.258514
TMEM252	-0.028546198	-0.035345679	-0.11011	-0.00145	0.008837	-0.11377
TMEM253	-0.028423063	0.002455595	-0.02053	0.020593	-0.05888	-0.05511
TMEM254	-0.001049465	-0.014221164	0.000308	-0.05947	0.150618	0.138714
TMEM254-/	-0.025616987	-0.069167185	0.108436	0.001727	-0.07968	-0.11172
TMEM255A	-0.019255462	0.038371476	0.086646	-0.03291	0.060225	0.021089
TMEM255B	-0.030493011	-0.12632805	-0.16825	-0.0222	0.087133	0.185617
TMEM256	0.018581387	-0.018595643	0.118406	0.03939	-0.09684	0.176795
TMEM257	-0.04064449	-0.031822698	0.05674	0.003511	0.011758	-0.10529
TMEM258	0.083177767	0.064275581	0.091945	0.089489	-0.21369	0.085501
TMEM259	-0.007976525	-0.038750337	0.197568	-0.04301	0.096535	-0.01553
TMEM26	-0.060876109	-0.04881516	-0.01925	-0.00393	0.006903	0.039445
TMEM260	0.025699023	-0.027350377	-0.05123	0.002655	-0.01866	-0.18063
TMEM261	0.018866349	0.026381495	0.292302	0.044787	-0.11856	-0.10313
TMEM262	-0.025697511	-0.068879657	0.011387	0.002373	-0.01638	-0.01809
TMEM263	0.009400325	0.112479839	0.360815	0.007374	-0.02939	-0.51097
TMEM27	-0.059982552	-0.042261691	0.041722	-0.03204	0.069385	-0.02283
TMEM30A	0.104699071	0.176329618	-0.2308	-0.00728	0.018855	0.237138
TMEM30B	-0.026395545	-0.093533259	0.335747	-0.03729	0.128141	-0.19419
TMEM31	-0.038915938	-0.033579113	0.019857	-0.00637	0.03765	-0.0369
TMEM33	0.025989226	0.050401739	-0.22475	0.034987	-0.14082	0.286533
TMEM35	-0.037210286	-0.043391361	0.055652	0.000847	0.020142	-0.00841
TMEM37	-0.027969232	-0.093139097	-0.11803	-0.01111	-0.00943	-0.10701
TMEM38A	-0.021127771	-0.080834826	-0.1334	-0.02757	0.041868	0.174765
TMEM38B	0.008448981	-0.055491981	-0.04357	0.057193	-0.21758	-0.16964
TMEM39A	-0.013958759	0.006883422	-0.10146	0.019413	-0.12413	0.050695
TMEM39B	0.008367866	-0.06066659	-0.10997	0.003076	0.018704	0.237777
TMEM40	-0.016384489	-0.039487101	-0.05926	-0.25777	0.711426	0.063933
TMEM41A	0.022371603	0.092272986	0.148556	-0.01867	0.030132	-0.23102
TMEM41B	0.031774979	0.095794203	0.17678	0.03474	-0.11112	-0.35532
TMEM42	0.023317158	-0.032773292	0.23086	-0.04187	0.083319	-0.08379
TMEM43	0.02477464	0.036382007	-0.16988	-0.00173	0.059567	0.032192
TMEM44	-0.026316347	-0.018131412	0.048717	0.015029	-0.06226	-0.15404
TMEM44-AS	0.003340796	-0.059568978	-0.10635	0.033917	-0.06142	0.253592
TMEM45A	-0.068521904	-0.011960578	0.031787	-0.08833	0.25571	0.064177
TMEM45B	-0.016581796	-0.048483703	-0.13037	-0.03166	0.039705	-0.14422
TMEM47	-0.049949508	-0.045606545	-0.00198	-0.0147	0.035558	-0.1121
TMEM5	0.020977893	0.027443662	0.119212	0.077764	-0.19781	-0.09049

TMEM50A	0.091043635	0.151360489	-0.10953	0.021968	-0.02376	0.089695
TMEM50B	0.035625846	0.049795308	0.18525	0.015144	0.050457	-0.23325
TMEM51	-0.022746001	-0.057744902	-0.07524	-0.03654	0.066394	0.304931
TMEM51-AS	-0.077262778	-0.056024948	-0.03926	-0.03298	0.082083	0.062765
TMEM52	-0.026031465	-0.032807028	-0.05939	0.0152	-0.02299	-0.03148
TMEM52B	-0.020468107	-0.016014773	0.000652	0.001899	0.016573	0.058762
TMEM53	-0.005952898	-0.043864035	-0.17822	0.03062	-0.10113	0.145632
TMEM54	-0.027760603	-0.028432396	-0.03911	0.011181	-0.03592	-0.05143
TMEM55A	0.002980535	-0.112800749	-0.23626	-0.07194	0.214612	0.293799
TMEM55B	0.040664415	-0.055523701	-0.01705	-0.01647	0.034968	-0.26504
TMEM56	-0.047365422	-0.100904319	-0.07484	0.04576	-0.08447	-0.09469
TMEM57	0.007672101	0.014437678	0.053088	0.036847	-0.05994	-0.09203
TMEM59	0.038128657	0.081825422	-0.05527	0.009727	0.012276	-0.05934
TMEM59L	-0.024034229	-0.118108938	0.051134	0.032433	-0.12871	0.018051
TMEM60	0.030238075	0.014704439	-0.1626	0.001514	-0.02399	0.104659
TMEM61	-0.032765362	-0.015645646	0.039662	-0.00123	-0.01014	-0.08743
TMEM62	-0.003489822	0.013742539	-0.11356	0.031891	-0.0775	0.034464
TMEM63A	0.007066725	-0.076503346	0.188955	-0.06033	0.196102	-0.18678
TMEM63B	-0.000542402	-0.082720134	-0.00954	-0.0003	-0.08864	-0.04115
TMEM63C	-0.029414498	-0.139998965	-0.07754	-0.04473	0.156646	0.382088
TMEM64	-0.037088978	0.125400205	0.023074	-0.06824	-0.01722	-0.11654
TMEM65	-0.002484963	0.031330188	-0.06715	0.05329	-0.18592	0.088451
TMEM67	-0.151452357	-0.077731023	0.132551	-0.01846	0.054673	-0.15208
TMEM68	0.003452963	0.068642055	-0.03793	0.098237	-0.27307	0.081499
TMEM69	0.032375713	0.001626576	-0.04965	0.013134	-0.02697	0.396084
TMEM70	0.022806582	-0.051221276	0.001031	-0.0219	4.55E-05	-0.01921
TMEM71	0.020420554	0.072385786	-0.03196	-0.03712	0.107417	0.180541
TMEM72	-0.053528068	-0.065878685	0.011536	0.00666	0.007944	-0.02088
TMEM72-AS	-0.027064803	-0.025916111	0.02041	-0.00164	-0.07122	-0.04482
TMEM74	-0.01136071	-0.184668366	-0.00133	0.078301	-0.06438	-0.04672
TMEM74B	-0.035961435	-0.013158407	0.000629	0.017489	-0.02921	0.017058
TMEM75	-0.042141803	-0.057417726	-0.06089	-0.00338	0.005842	-0.0228
TMEM79	-0.004112234	-0.003824398	0.059917	0.027612	-0.08929	-0.02385
TMEM80	0.021300694	-0.024756436	-0.01868	-0.05044	0.159742	0.169285
TMEM86A	-0.04962407	-0.058435651	-0.02031	-0.02957	0.117623	0.182982
TMEM86B	-0.007608155	-0.042539949	-0.01523	-0.01927	0.081106	0.114447
TMEM87A	-0.013028179	0.129018355	0.012866	-0.03281	0.028884	-0.116
TMEM87B	0.021502656	-0.028396697	0.08613	-0.04552	0.136415	0.095678
TMEM88	-0.012187988	-0.080713865	-0.06666	-0.01096	-0.00773	0.056511
TMEM8A	0.031369312	-0.026870122	-0.08042	-0.02359	0.026578	0.087281
TMEM8B	-0.003188148	-0.106155776	-0.09586	-0.00546	0.011821	0.009286
TMEM9	0.011684652	-0.017856027	0.099841	-0.11561	0.314377	0.057423
TMEM91	0.00580292	-0.034358751	-0.12585	-0.09709	0.271446	0.281936
TMEM92	-0.030877619	-0.061541382	0.084525	0.023676	-0.11165	-0.07668
TMEM95	-0.027589329	-0.06194313	0.045611	0.007357	-0.0377	0.014387
TMEM97	0.017791571	0.048589722	0.103224	0.088293	-0.3298	-0.25324
TMEM98	-0.020835971	-0.038063058	0.009982	0.209376	-0.51475	-0.05011
TMEM99	-0.008348354	0.045645511	0.223963	0.029909	-0.15774	-0.17762
TMEM9B	0.04972771	0.117662733	0.009863	-0.01597	0.036629	0.091785
TMF1	-0.000717979	0.047181992	-0.13294	-0.03443	0.128995	-0.00368
TMIE	-0.045582121	-0.13513328	0.121482	-0.05644	0.194946	-0.06682

TMIGD2	0.037000433	0.091580534	0.147365	-0.0391	0.053603	-0.38843
TMLHE	-0.018552098	-0.062904116	-0.10588	-0.10406	0.217542	0.197597
TMOD1	-0.012996088	0.0882751	0.001207	-0.04639	0.085527	-0.1462
TMOD2	-0.014660722	-0.156322933	-0.04503	-0.07978	0.235282	0.406825
TMOD3	-0.016998547	0.072634783	-0.13775	0.023995	-0.06969	0.16634
TMPO	0.012738358	0.014826909	0.098242	0.029182	-0.15725	0.180006
TMPO-AS1	-0.021428913	-0.03276474	0.045953	0.129338	-0.32687	0.226876
TMPRSS11I	-0.022718496	-0.017180907	-0.00567	0.116091	-0.13192	0.003287
TMPRSS11I	-0.024788147	-0.052839388	0.12478	0.021953	-0.12687	-0.04682
TMPRSS11I	-0.017062789	-0.041618228	0.029011	0.015528	-0.06211	-0.06222
TMPRSS12	-0.049935631	-0.052348976	-0.12792	-0.00313	0.018809	0.050148
TMPRSS13	-0.031968798	-0.042777347	0.04754	0.013287	-0.0738	-0.15182
TMPRSS15	-0.021934851	-0.017065717	0.019715	0.103028	-0.15231	-0.05304
TMPRSS2	-0.021452461	-0.037800452	0.050859	0.025993	-0.06425	-0.13007
TMPRSS3	-0.026755831	-0.045973937	0.05007	0.164795	-0.27706	-0.06163
TMPRSS4	-0.038370119	-0.130586318	-0.0542	0.030961	-0.08006	-0.0103
TMPRSS5	-0.029222876	-0.009392436	-0.02766	-0.01504	-0.02719	-0.08448
TMPRSS6	0.005568601	-0.116968	0.124196	0.004755	0.016277	-0.16683
TMSB10	0.029499734	0.073354927	-0.02509	0.076055	-0.08307	0.197258
TMSB15B	-0.056582757	0.005408011	0.088846	0.021141	-0.07235	-0.15937
TMSB4X	0.075595215	0.163789645	-0.28935	0.117223	0.004795	0.110868
TMSB4Y	0.000528113	-0.092271655	-0.10855	-0.06644	0.080101	0.088188
TMTC1	0.01120707	-0.059390481	-0.09948	-0.02329	0.069868	0.300428
TMTC2	-0.012709115	-0.096484137	-0.15636	-0.14052	0.406784	0.659259
TMTC3	-0.008684049	0.138457804	0.139419	0.026287	-0.10429	-0.07183
TMTC4	-0.005665424	0.038855119	0.022382	0.084511	-0.21164	-0.00353
TMUB1	0.024074667	-0.055654874	0.121603	0.031655	-0.20486	-0.17575
TMUB2	0.028110745	-0.085823419	-0.1947	-0.03877	0.093592	0.145082
TMX1	0.058518038	0.0955567	-0.04123	0.072019	-0.207	0.112255
TMX2	0.039381922	0.05530741	0.048398	0.052663	-0.14124	0.056221
TMX3	0.02218125	0.04980055	0.100108	0.032108	-0.07743	-0.00674
TMX4	0.02585597	0.113285592	-0.03842	0.021815	-0.04667	-0.09019
TNAP	-0.046062173	-0.03688083	0.142982	0.010013	-0.00024	-0.16648
TNC	0.000322509	-0.005643521	0.033074	0.109333	-0.18146	-0.01095
TNF	0.002807555	-0.005866614	-0.06824	-0.00288	-0.01809	0.076284
TNFAIP1	0.001372999	0.004198619	-0.07374	0.008068	-0.07488	-0.00426
TNFAIP2	0.00331608	-0.1144997	-0.32028	-0.07159	0.190949	0.443232
TNFAIP3	0.041379226	0.058474017	0.094889	0.042134	-0.09946	-0.05018
TNFAIP6	-0.001983032	-0.064488309	-0.19131	-0.02703	0.075123	0.219923
TNFAIP8	0.045135313	0.064628904	0.135673	0.01238	-0.0207	-0.2445
TNFAIP8L1	0.025361008	0.022000091	0.082438	-0.00068	-0.02609	0.074484
TNFAIP8L2	0.024492322	0.047604209	-0.2154	-0.06735	0.178297	0.343708
TNFAIP8L3	-0.020116952	-0.004862547	0.055088	-0.00411	0.008841	-0.11134
TNFRSF10A	0.019264312	-0.090037761	0.214515	-0.00585	-0.08469	-0.05319
TNFRSF10I	-0.021572178	-0.111946782	-0.0809	-0.00365	-0.05871	0.234034
TNFRSF10C	-0.048913951	-0.107165722	-0.16335	-0.0891	0.132978	0.063351
TNFRSF10I	-0.011976798	-0.186459177	-0.0044	-0.08053	0.251648	0.380151
TNFRSF11A	0.004275851	0.096716126	-0.05297	0.067579	-0.18595	-0.08194
TNFRSF11I	-0.021162682	-0.040153509	-0.00024	7.54E-05	0.005941	-0.03051
TNFRSF12A	-0.010962131	-0.07351993	-0.03579	0.011894	-0.09804	0.025665
TNFRSF13I	-0.013334398	-0.09835749	-0.07295	-0.05079	0.084543	-0.01019

TNFRSF13(	-0.015318659	-0.062655415	-0.03723	-0.03628	0.004427	-0.19693
TNFRSF14	0.044040839	0.005802987	0.067135	-0.05179	0.12592	0.010797
TNFRSF17	-0.00482345	-0.079536964	-0.09068	-0.12001	0.343239	-0.2168
TNFRSF18	-0.003029815	0.165542992	-0.07988	0.001026	-0.0386	-0.06752
TNFRSF19	-0.047386204	-0.027176482	-0.13604	0.132025	-0.0622	-0.00597
TNFRSF1A	0.029673603	0.071684769	-0.20313	-0.03198	0.095604	0.342015
TNFRSF1B	0.039560773	0.088905583	-0.35277	-0.01492	0.054164	0.499372
TNFRSF21	-0.001321411	-0.068509958	-0.1043	-0.04204	0.119545	-0.13775
TNFRSF25	0.004457178	-0.029702509	0.389966	-0.0485	0.120376	-0.21671
TNFRSF4	-0.010677315	0.091394013	-0.0589	0.008107	-0.03823	-0.08771
TNFRSF8	-0.012877979	-0.060162146	-0.14322	0.070145	-0.22949	0.294568
TNFRSF9	-0.014535807	0.005789651	-0.03993	0.035159	-0.16707	-0.00224
TNFSF10	0.008198076	0.044313559	-0.26175	-0.00142	0.008244	0.433768
TNFSF11	-0.019186695	0.088923003	-0.04481	-0.02116	0.026075	-0.05494
TNFSF13	0.008488606	-0.126096254	-0.35076	-0.12059	0.331288	0.549909
TNFSF13B	0.017924285	-0.110199338	-0.43535	-0.08523	0.262301	0.446655
TNFSF14	0.001787224	0.1226755	-0.06679	0.028744	-0.09165	-0.04003
TNFSF15	-0.019621073	-0.035846636	-0.05793	-0.02803	0.043917	0.064571
TNFSF18	-0.027603805	-0.053538626	0.001008	0.018049	-0.03808	-0.02398
TNFSF4	0.002941122	0.041852594	-0.01569	0.009195	0.011238	-0.07706
TNFSF8	-0.013839192	-0.143131541	0.226869	-0.05276	0.117338	0.169124
TNFSF9	-0.008322028	-0.042732582	-0.01089	0.04943	-0.16738	-0.05541
TNIK	-0.012665964	0.168120142	0.305954	-0.01822	0.128596	-0.28623
TNIP1	0.042379639	0.075475624	0.094237	-0.01126	0.041712	-0.20157
TNIP2	0.041722926	0.026911982	0.026815	0.025224	-0.08005	-0.03914
TNIP3	-0.016377887	-0.02453912	0.076879	0.050939	-0.16799	-0.01608
TNK1	-0.021393483	-0.202993578	0.298062	-0.02251	0.031519	-0.17196
TNK2	-0.045916775	-0.109023421	-0.08622	-0.02871	0.021799	0.033827
TNK2-AS1	-0.027841209	-0.023335001	-0.0952	-0.01213	0.049768	0.088436
TNKS	-0.040871848	-0.068436498	-0.02352	0.021479	-0.04464	0.087348
TNKS1BP1	-0.02552499	-0.039188281	-0.04965	-0.01187	0.052474	0.061012
TNKS2	0.006937976	0.058228569	0.031993	-0.02982	0.078045	-0.02175
TNMD	-0.049890537	0.024381752	0.03067	-0.01293	0.046566	0.060321
TNN	-0.036292757	-0.055348608	-0.0466	0.011985	-0.01741	0.001594
TNNC1	-0.026429366	-0.087376313	0.01194	0.049359	-0.1206	-0.03432
TNNC2	-0.025087304	-0.09570478	-0.08491	-0.06749	0.208954	0.077696
TNNI1	-0.029690515	-0.072778589	-0.00815	0.051284	-0.11537	0.035184
TNNI2	-0.007546749	-0.156384011	-0.24509	-0.04113	0.12898	0.239602
TNNI3	-0.033693237	-0.073639003	-0.19981	-0.00845	0.03523	0.158561
TNNI3K	-0.030398282	-0.023869689	-0.01969	-0.04398	0.123748	0.275616
TNNT1	-0.009882043	-0.105655838	-0.18897	-0.01785	0.060886	0.557754
TNNT2	-0.024911756	-0.036682843	-0.06445	0.008173	-0.04633	-0.01408
TNNT3	-0.029799403	-0.129774738	0.031526	0.002477	-0.05544	-0.0833
TNP1	-0.024586757	-0.008218179	-0.13798	0.024219	-0.06871	0.051232
TNP2	-0.071523188	-0.06028028	-0.01479	-0.00677	0.030023	0.061379
TNPO1	0.060081483	0.122239149	-0.01919	0.052841	-0.09526	-0.13529
TNPO2	0.025473161	-0.021720362	0.174569	0.040569	-0.1242	-0.19414
TNPO3	0.123207815	0.049745696	0.076507	0.072901	-0.30802	-0.1335
TNR	-0.034872345	0.050562569	0.040061	-0.06948	-0.1002	-0.14051
TNRC18	-0.05284545	-0.022185587	-0.039	-0.08134	-0.0567	0.043836
TNRC6A	0.041616609	0.014342684	0.174874	0.028905	0.033886	-0.10485

TNRC6B	0.021246421	0.037685515	0.138003	-0.02075	0.092055	-0.2226
TNRC6C	-0.010658328	0.118442604	0.320218	-0.019	0.174684	-0.32866
TNRC6C-AS	0.00754443	0.035611338	0.366436	-0.06147	0.154639	-0.27131
TNS1	-0.008010784	-0.050479144	-0.12813	-0.00833	0.256223	0.250561
TNS3	0.005116205	-0.14079449	-0.32584	-0.04011	0.125435	0.432034
TNS4	-0.026382939	-0.097666432	0.066083	0.056988	-0.24446	-0.06265
TNXB	-0.035471655	-0.088658146	0.062194	-0.02811	0.069618	-0.00039
TOB1	0.030205092	-0.074824417	0.290192	-0.0017	0.026956	-0.1575
TOB1-AS1	-0.026468263	-0.072056729	0.062289	0.037009	-0.07459	-0.037
TOB2	0.019430691	0.043228467	-0.07285	-0.01176	0.024122	0.156073
TOE1	0.03793432	-0.068304377	0.155597	0.040873	-0.15162	-0.14697
TOLLIP	0.000914398	-0.033195408	-0.24723	-0.0707	0.117163	0.254935
TOLLIP-AS	-0.0150345	-0.066726566	0.228847	-0.04371	0.152516	0.035072
TOM1	0.006397237	-0.088672237	-0.13377	-0.03657	0.097217	0.385805
TOM1L1	-0.058295474	-0.042639407	0.038268	-0.03556	0.119253	-0.06843
TOM1L2	0.081814166	-0.074043545	0.06774	-0.0348	-0.02872	0.035101
TOMM20	0.047859554	-0.00650007	0.201258	0.023168	-0.06413	0.119721
TOMM22	0.044819511	0.053729024	0.16047	0.071293	-0.16887	0.142653
TOMM34	0.019563457	-0.048312561	-0.03945	0.084529	-0.27893	-0.18961
TOMM40	0.021177118	-0.040607203	0.059664	0.057593	-0.20417	-0.03019
TOMM40L	0.023144428	0.066033293	-0.0594	0.078878	-0.19832	0.21022
TOMM5	0.029286091	0.096273041	0.152648	0.056407	-0.15123	0.05564
TOMM70A	0.0310232	0.078884035	0.208263	0.045807	-0.13611	-0.04124
TONSL	-0.022180152	0.041803402	-0.03033	0.185422	-0.34602	0.078399
TOP1	0.048888388	0.061050565	-0.13879	0.058666	-0.13902	0.085626
TOP1MT	-0.001851277	-0.198939778	0.103455	0.00226	0.00954	-0.00592
TOP1P2	-0.071243054	-0.060299833	-0.10304	0.00468	-0.01028	0.002644
TOP2A	-0.015078557	0.032344422	0.060292	0.171658	-0.24352	-0.07157
TOP2B	0.090388337	0.205813694	0.149771	0.058446	-0.13291	-0.41555
TOP3A	0.003174536	-0.024898461	-0.29511	0.001208	-0.07609	0.239218
TOP3B	-0.012291432	-0.012087478	0.067943	-0.02501	0.108456	0.030861
TOPBP1	0.057957569	0.129809559	-0.21374	0.114133	-0.30088	-0.15408
TOPORS	0.030573361	0.011726451	-0.09685	0.006864	-0.00169	-0.14146
TOPORS-AS	0.029228837	-0.04577694	0.182254	-0.00682	-0.02161	-0.34508
TOR1A	0.11298189	0.16616519	-0.24341	0.078578	-0.12863	0.192617
TOR1AIP1	-0.026919149	0.0502662	-0.18736	0.02976	-0.10586	0.073928
TOR1AIP2	-0.052543358	0.039392392	-0.08907	0.115438	-0.14435	-0.00706
TOR1B	0.047973777	0.147810576	-0.13243	-0.01283	0.026529	0.144851
TOR2A	0.003479623	-0.030366775	-0.21396	0.017759	-0.05194	0.326363
TOR3A	0.027284923	-0.05851338	-0.12508	0.023832	-0.07451	0.206157
TOR4A	0.014489336	0.039996659	-0.27482	-0.03821	0.036673	0.257227
TOX	-0.000513382	0.226837462	0.199714	0.00767	-0.00081	-0.2962
TOX2	-0.010479118	-0.037350935	-0.06797	0.116747	-0.23174	-0.1513
TOX3	-0.131281478	-0.105985221	-0.03686	-0.04762	0.056341	-0.07401
TOX4	0.114509554	0.092257768	0.03846	0.072402	0.003544	-0.08833
TP53	0.016362829	0.003623019	-0.02155	0.076457	-0.22014	0.116071
TP53AIP1	-0.042377585	-0.061263444	-0.09696	0.066851	-0.02745	0.020082
TP53BP1	-0.012146607	-0.076082941	0.181222	0.028322	-0.11206	-0.39965
TP53BP2	0.04236385	0.042726632	-0.0987	0.077146	-0.21344	0.11985
TP53I11	-0.001715733	-0.042212643	-0.03983	-0.08396	-0.00179	-0.10055
TP53I13	0.013959288	0.052265833	0.063135	-0.02948	0.059386	-0.27427

TP53I3	-0.003896204	-0.136449717	-0.29125	-0.04284	0.10721	0.563812
TP53INP1	-0.023090492	-0.051429711	-0.10299	-0.00364	-0.03098	-0.11528
TP53INP2	0.015420479	-0.056355462	-0.143	0.009121	-0.03751	-0.1322
TP53RK	0.024732967	0.145108865	0.082261	0.060396	-0.19267	-0.18706
TP53TG1	0.006184973	0.009026444	0.197648	0.049494	-0.0948	-0.12209
TP53TG5	-0.032137517	-0.067203079	0.016107	0.02016	-0.07217	-0.04661
TP63	0.005087414	-0.080536254	-0.02674	0.028722	0.027111	-0.04714
TP73	-0.047114966	-0.018471222	-0.00544	0.066584	-0.05965	0.069255
TP73-AS1	-0.025506739	-0.060749924	0.039977	-0.03647	0.083956	-0.0118
TPBG	-0.030795211	-0.050354628	0.026769	-0.11274	0.307209	0.193236
TPBGL	-0.028584566	0.01042338	-0.05126	0.00183	-0.02471	-0.03052
TPCN1	0.073640262	-0.165366021	-0.10305	-0.05121	0.143756	0.362353
TPCN2	-0.00778285	-0.178464028	-0.16294	-0.07007	-0.0133	0.49732
TPD52	0.004908669	0.075073879	0.111746	0.017274	-0.0327	-0.40221
TPD52L1	-0.04449037	0.108373074	0.018799	-0.00064	0.010481	-0.1165
TPD52L2	0.044879503	-0.033743052	-0.31462	0.010142	-0.0019	0.423768
TPD52L3	-0.040749547	-0.065418233	-0.0392	0.005422	-0.02051	0.072795
TPGS1	-0.015592768	-0.0320984	0.024705	0.042356	-0.08283	0.127971
TPGS2	0.010764809	0.106529599	-0.01544	0.083381	-0.09591	-0.02398
TPH1	-0.05415509	-0.030826721	0.152775	-0.07367	0.065916	-0.08756
TPH2	-0.047122597	-0.004408469	0.039788	0.007868	-0.01608	-0.08899
TPI1	0.066930635	0.081634854	-0.08528	0.104891	-0.2486	0.2357
TPK1	0.018733006	-0.013353051	-0.09961	-0.02322	0.095973	0.251582
TPM1	0.045757572	0.102637606	-0.03266	-0.09595	0.303612	0.174243
TPM2	0.009270763	-0.017067793	0.035468	0.066035	0.022254	-0.16775
TPM3	0.010203241	-0.049966798	-0.12001	-0.1027	-0.10779	0.225045
TPM4	0.014388464	0.074202708	-0.1597	0.024918	-0.09767	0.080984
TPMT	0.014808368	0.037678382	-0.02019	0.046238	0.012771	0.181678
TPO	-0.036751842	-0.04606828	-0.03848	0.049112	-0.11914	-0.03291
TPP1	0.032058693	-0.057415953	-0.27183	-0.1109	0.247055	0.472328
TPP2	0.021038507	0.098498095	0.370776	0.019212	-0.07942	0.019857
TPPP	-0.063111671	-0.058019177	0.01261	0.049738	0.020689	-0.13149
TPPP2	-0.049116549	-0.069984326	-0.05266	0.04037	-0.08294	-0.01731
TPPP3	-0.004498516	-0.084875998	-0.18504	-0.0988	0.290891	0.571762
TPR	-0.005858643	0.025056906	0.102683	0.013473	-0.10362	0.048597
TPRA1	0.006481956	0.021728763	-0.03067	0.03037	-0.09342	0.126954
TPRG1	-0.008773785	-0.033401535	0.102827	0.010921	-0.05587	-0.01804
TPRG1L	0.048420423	0.04289634	-0.02959	-0.02598	0.082626	-0.29169
TPRKB	0.036086746	0.029138457	0.142245	0.039811	-0.12412	0.125666
TPRN	-0.010916209	-0.047740562	-0.08262	0.036452	-0.19519	0.159067
TPRXL	-0.026300517	-0.034700043	-0.01089	-0.00806	-0.04763	-0.09902
TPSAB1	-0.041955021	0.002937402	-0.13843	-0.01545	0.031736	-0.02091
TPSB2	-0.020416149	-0.034158484	-0.18597	-0.03793	0.099662	-0.04088
TPSD1	-0.024610839	-0.058572102	-0.02815	0.027677	-0.05536	-0.0231
TPSG1	-0.010974611	-0.057818537	0.032273	0.001177	-0.11751	-0.09879
TPST1	-0.015282654	-0.064641338	-0.09201	-0.02368	0.031071	-0.12533
TPST2	0.040863422	0.288243673	0.226619	-0.04391	0.115443	-0.08834
TPT1-AS1	-0.024765683	0.103145432	0.16607	0.02735	-0.0487	-0.23006
TPT1P8	-0.024704916	-0.129058887	0.125654	-0.00317	0.031135	0.155262
TPTE	-0.031674993	-0.050140175	0.070744	0.002412	-0.05161	-0.07021
TPTE2P5	-0.041860367	0.017453529	0.020061	-0.03488	0.094501	-0.20314

TPTE2P6	-0.017616557	-0.027260219	0.012722	0.041429	-0.08177	-0.01996
TPTEP1	-0.028865513	-0.065488725	0.08929	-0.09008	0.255089	0.106365
TPX2	-0.008751233	0.017117117	0.021085	0.150814	-0.32029	-0.07033
TRA2A	0.024593244	0.100565205	0.14167	-0.00016	0.089914	-0.32243
TRA2B	0.054084584	0.042167734	0.172187	0.052828	-0.15925	-0.11343
TRABD	0.071317781	-0.096886246	0.071222	-0.01014	-0.06481	-0.04284
TRABD2A	-0.002950296	-0.135107854	0.474933	-0.05222	0.148227	-0.22714
TRABD2B	-0.060885463	-0.057986827	-0.08976	-0.03975	0.035082	0.073559
TRAC	0.012713213	-0.049437214	0.605807	-0.03407	0.076554	-0.36526
TRADD	0.033941465	-0.052499749	0.102848	0.000485	0.006073	-0.17222
TRAF1	0.003126844	-0.016080918	0.159598	0.049701	-0.15784	-0.12248
TRAF2	-0.003852433	-0.000322206	0.022926	0.043987	-0.20917	-0.20395
TRAF3	0.018183026	-0.04216603	-0.06542	0.026554	-0.10885	0.100227
TRAF3IP1	-0.005093711	0.042304892	0.098794	-0.03521	0.099986	0.003201
TRAF3IP2	-0.007359143	-0.065235957	0.20495	-0.07596	0.165615	-0.19362
TRAF3IP2-	-0.038853049	0.035377618	0.11109	0.063666	-0.23861	-0.15127
TRAF3IP3	0.015383592	0.040428307	0.282116	-0.03274	0.097519	-0.14262
TRAF4	-0.028800316	-0.075871764	-0.07181	0.014895	-0.07038	-0.14113
TRAF5	0.010580101	0.09057074	0.289818	-0.02376	0.063262	-0.42398
TRAF6	0.028477766	0.058882841	-0.11033	-0.01061	0.00675	-0.08313
TRAF7	0.005697765	-0.135028221	-0.10449	0.048905	-0.19328	0.098138
TRAFD1	0.011292247	-0.010472297	-0.17744	-0.03396	0.042061	0.145948
TRAIP	-0.019166392	-0.014245513	-0.01909	0.09412	-0.28966	-0.01937
TRAK1	-0.032028202	-0.127670115	-0.24314	-0.02931	0.103654	0.027196
TRAK2	0.028489384	0.044731279	0.126357	-0.02466	0.043756	-0.06233
TRAM1	0.053694281	0.130756432	0.135591	0.005968	-0.01666	-0.08003
TRAM1L1	-0.02991555	-0.076134593	0.012007	-0.00038	0.009591	0.011084
TRAM2	0.008478357	0.06358943	0.003412	-0.02453	0.076416	0.056718
TRAM2-AS1	-0.005953681	-0.01978791	-0.13066	0.034136	-0.05455	0.210742
TRANK1	0.015285048	0.091270937	0.013135	-0.04495	0.104451	-0.23434
TRAP1	0.013153225	-0.01759113	0.229364	0.082863	-0.2278	-0.05621
TRAPPC1	0.029262694	0.013678522	-0.01219	-0.01395	0.032531	0.193917
TRAPPC10	0.028255865	0.124716064	-0.02785	0.000854	-0.01179	-0.25461
TRAPPC11	0.011890189	0.048910344	-0.17423	0.015575	0.062756	0.324415
TRAPPC12	0.032646455	-0.067974017	-0.10167	-0.02703	0.056349	0.054846
TRAPPC13	0.031637558	0.114281781	0.014244	-0.03326	-0.01299	0.136192
TRAPPC2	0.011138214	0.144089777	0.263307	-0.01021	0.017885	-0.2894
TRAPPC2L	0.006028871	-0.06578388	0.163117	0.013349	-0.09165	-0.15211
TRAPPC3	0.072515864	-0.01433839	-0.08571	0.041888	-0.13729	0.267111
TRAPPC3L	-0.028177387	-0.044752068	-0.02153	-0.18684	0.551988	-0.05395
TRAPPC5	0.032751601	-0.10089521	-0.14192	0.003266	-0.01171	0.420075
TRAPPC6A	0.02464355	0.022889959	0.211555	0.003579	-0.00994	-0.00421
TRAPPC6B	0.030305426	0.112589163	-0.07107	0.016278	-0.03457	0.078261
TRAPPC8	0.108554923	0.118405139	-0.1489	0.003512	0.062621	-0.21353
TRAPPC9	-0.00783434	-0.075957894	-0.09063	-0.00978	-0.00741	0.012112
TRAT1	7.26E-05	-0.053344852	0.523445	0.002909	-0.00483	-0.30317
TRAV12-2	-0.025146591	-0.134050668	0.399312	-0.03719	0.134933	-0.12579
TRAV8-3	-0.010474524	-0.133177895	0.469329	-0.08094	0.245605	-0.26339
TRBC1	0.017981914	0.153917862	0.465123	-0.03011	0.078587	-0.36808
TRBV27	-0.00427852	-0.00750287	-0.02962	-0.23487	0.663639	0.137588
TRDMT1	-0.010465753	-0.055883937	0.141859	-0.06093	0.002611	-0.06374

TRDN	-0.034679718	-0.104025559	-0.00605	0.081151	-0.12815	-0.06872
TRDV3	0.00047425	0.394039472	0.114563	0.007989	-0.03433	-0.22768
TREH	-0.038945911	-0.003844717	-0.05469	0.020312	-0.03727	0.018466
TREM1	0.000760444	-0.082513325	-0.31749	-0.06329	0.187494	0.498986
TREM2	-0.001686957	-0.016850762	-0.08799	-0.01452	-0.04402	-0.04232
TREML1	-0.012110052	-0.027726032	-0.05629	-0.23402	0.614521	0.022288
TREML2	-0.006546169	-0.09415197	-0.23659	-0.03138	0.034593	-0.22086
TREML3P	-0.0245352	-0.056824816	-0.23088	-0.0319	0.099567	0.064675
TREML4	-0.040118548	-0.065592796	-0.16708	0.016891	-0.03312	0.000333
TREML5P	-0.034123433	-0.012393646	-0.00158	0.03867	-0.11063	-0.09759
TRERF1	-0.004693264	0.045947955	0.063542	0.02146	-0.1075	-0.11004
TRG-AS1	0.015358042	0.135658941	0.35264	-0.0102	0.028488	-0.11757
TRH	-0.029701383	-0.025532295	-0.0358	0.012969	-0.04637	0.054866
TRHDE	-0.029398245	-0.053056286	-0.02029	0.011858	-0.02597	-0.09413
TRHDE-AS:	-0.060579315	-0.020687712	0.019846	-0.01483	0.067606	-0.01484
TRHR	-0.030250876	-0.073121683	0.00259	0.007474	-0.02634	-0.02835
TRIAP1	0.034632053	0.062209736	0.142584	0.080397	-0.21556	0.143291
TRIB1	-0.024418129	-0.078504806	-0.03953	-0.05673	0.115234	0.023398
TRIB2	0.006862983	0.121184002	0.250847	-0.03555	0.074533	-0.45669
TRIB3	-0.001511543	0.071754906	0.017532	0.086169	-0.27452	-0.08773
TRIL	-0.027729757	-0.067543933	-0.03995	-0.01614	0.046397	-0.01667
TRIM10	0.007460182	-0.026005415	-0.15204	0.126456	0.014004	0.018509
TRIM11	0.01557652	0.087580354	-0.04235	0.072538	-0.18656	0.052578
TRIM13	0.000133848	0.077739137	-0.09511	0.012694	0.035567	-0.11806
TRIM14	0.006669697	0.06972016	0.096593	-0.00761	0.028911	0.142473
TRIM15	-0.039285711	-0.037890355	0.043004	-0.02351	-0.04059	-0.06787
TRIM16	-0.005883274	-0.035126425	0.069218	0.005409	-0.03174	0.01837
TRIM17	-0.026821418	0.009680311	-0.12214	0.019151	-0.04845	0.01162
TRIM2	0.004913866	-0.081543795	0.050855	0.038254	0.043516	0.104301
TRIM21	0.027613181	0.067874949	-0.19351	-0.0042	-0.00745	0.069096
TRIM22	0.054342576	0.084393382	-0.0706	-0.00399	0.030306	-0.17782
TRIM23	0.023623934	0.087899735	0.040102	-0.04443	0.140472	-0.17744
TRIM24	0.037544762	0.013654059	-0.20585	0.085229	-0.19487	0.107281
TRIM25	0.029431793	-0.027265439	-0.21426	-0.01526	0.008588	0.042803
TRIM26	0.056187707	0.050256639	-0.10678	0.043785	-0.1546	0.054177
TRIM27	0.110270942	0.03301123	-0.0984	0.026733	-0.04647	0.168601
TRIM28	0.034969767	0.00583884	0.184582	0.093475	-0.25681	-0.06557
TRIM29	-0.011106075	-0.011250075	-0.0563	-0.00915	-0.18828	0.022621
TRIM3	-0.04371424	-0.012918889	-0.07291	0.005028	0.016101	0.183157
TRIM31	-0.032355684	-0.051303004	-0.02914	0.023447	-0.01835	0.071685
TRIM32	0.000301221	0.150517694	0.192804	0.008157	-0.00029	-0.28775
TRIM33	0.050913181	0.078844919	-0.0813	0.043818	-0.13555	-0.25924
TRIM35	0.020822962	0.04820452	0.359405	0.012232	-0.03077	-0.23503
TRIM36	-0.004788973	-0.116088162	-0.08361	0.06789	0.046137	0.175575
TRIM37	0.022087404	0.104550351	0.23551	0.079405	-0.21115	-0.0911
TRIM38	0.029818128	-0.015447456	-0.2228	-0.04442	0.094151	0.064504
TRIM4	0.049676205	0.042774541	0.078255	0.076034	0.079998	0.012195
TRIM40	-0.040816538	-0.002942722	-0.073	-0.0104	0.06325	-0.00204
TRIM41	0.038601693	-0.020190223	0.135081	0.02898	-0.10464	-0.10695
TRIM42	-0.044961404	-0.085663602	-0.07763	0.008458	0.016923	0.063633
TRIM44	0.032008905	0.036536042	0.031451	-0.11001	0.315127	0.115898

TRIM45	-0.026971608	-0.039771021	-0.08063	0.040567	-0.14441	-0.05609
TRIM46	-0.03028429	-0.054294759	0.059677	0.037257	-0.18149	-0.15298
TRIM47	-0.000477747	-0.049327629	0.10085	-0.0189	0.003761	-0.11887
TRIM5	0.005834888	0.088079064	-0.03108	-0.04633	0.136039	0.066982
TRIM50	-0.009382161	0.021691392	-0.06668	0.038284	-0.02731	0.057928
TRIM51	-0.028821967	-0.007602491	0.062918	-0.00173	0.027623	-0.10644
TRIM52	0.021074121	0.107701722	0.22842	-0.07279	0.216294	-0.25729
TRIM52-AS	-0.000326263	0.002012616	0.311372	-0.01214	0.036405	-0.14364
TRIM54	-0.029785398	-0.004668973	-0.06284	0.010624	0.001728	0.071149
TRIM55	-0.078854887	-0.100500518	0.023738	-0.00342	0.034913	0.053058
TRIM56	0.029096079	0.09358377	0.030088	0.016488	-0.06188	-0.0839
TRIM58	-0.020633317	-0.040803305	-0.07902	-0.21899	0.704416	0.136845
TRIM59	0.005595446	0.107318082	0.249059	0.070159	-0.16769	-0.26903
TRIM6	-0.015361469	-0.053075246	-0.0863	-0.02583	0.048045	-0.01877
TRIM61	-0.023207169	0.033130026	0.166784	-0.01871	0.020514	-0.30251
TRIM62	0.025969744	-0.004365743	0.138041	0.025159	0.013043	-0.00958
TRIM63	-0.035947555	-0.053911885	-0.07248	-0.00562	0.043208	-0.03989
TRIM65	-0.004195072	-0.06655965	0.012598	0.032504	-0.15338	-0.1881
TRIM66	0.012034332	-0.037356254	0.139889	-0.05855	0.052325	-0.05087
TRIM67	-0.023319819	-0.069550598	0.010309	-0.01259	-0.05639	-0.03481
TRIM68	0.012712398	0.082648467	0.179154	-0.01938	0.059397	-0.19916
TRIM69	-0.089434965	-0.146718675	0.084903	0.008196	-0.04006	-0.10488
TRIM7	0.007277257	-0.116212355	0.043614	-0.00777	0.145506	0.214195
TRIM71	-0.034872584	-0.013361675	-0.10457	-0.01836	0.080498	0.177752
TRIM72	-0.037791665	-0.02293026	0.08679	-0.02373	0.047186	-0.03819
TRIM78P	-0.027635474	0.008664485	0.107525	-0.01136	0.030103	0.079975
TRIM8	-0.011714829	-0.124617991	0.053255	-0.01662	-0.03127	0.048582
TRIM9	-0.020735859	-0.013815309	-0.03945	0.097091	-0.17911	-0.02911
TRIML1	-0.045459629	-0.004228123	-0.16274	0.020308	-0.02406	0.160927
TRIML2	-0.030631996	-0.075274588	-0.11916	0.067605	-0.15179	0.106196
TRIO	-0.01242053	-0.008214695	-0.35159	0.057414	-0.03523	0.317586
TRIOBP	-0.022842629	-0.097020198	-0.04618	-0.05562	-0.03411	0.146029
TRIP10	-0.009937367	-0.022566599	-0.06299	0.033825	-0.13187	-0.01267
TRIP11	0.024980518	0.064352989	0.050745	-0.02336	-0.00954	0.061371
TRIP12	0.059795712	0.06369445	-0.19793	-0.06983	0.204663	0.257407
TRIP13	-0.010215251	-0.000266799	-0.03859	0.133529	-0.37295	-0.09903
TRIP4	0.055646383	0.158379889	-0.22808	-0.01786	0.033761	0.073056
TRIP6	0.00235256	-0.179532129	-0.04697	-0.03698	0.088861	-0.02726
TRIQK	-0.031183656	-0.132214036	-0.22195	-0.09142	0.444663	0.680734
TRIT1	0.024544264	-0.054255949	-0.06489	0.035219	-0.08373	0.190633
TRMT1	0.025211165	-0.092945307	0.015289	0.005118	0.037914	0.313738
TRMT10A	-0.027820008	0.007229576	0.043278	0.077054	-0.07448	0.067906
TRMT10B	-0.00200127	0.018039535	0.118939	-0.05847	0.159118	0.014762
TRMT10C	0.025704285	0.055731373	0.133861	0.039756	-0.10749	-0.0458
TRMT11	0.011293341	0.006374495	0.370349	0.034809	-0.09341	-0.33222
TRMT112	0.075443164	0.06736263	0.190567	0.12358	-0.26956	-0.2488
TRMT12	0.039067403	0.030590608	-0.05099	-0.01099	-0.03921	0.042258
TRMT13	0.010357872	0.074400517	0.249602	-0.01691	0.059436	-0.17213
TRMT1L	-0.001591506	-0.001564697	-0.10641	0.029624	-0.06096	-0.00623
TRMT2A	0.022489049	-0.095547397	0.14725	0.014281	-0.1057	-0.01105
TRMT2B	-0.00642526	-0.043975833	0.152816	0.012573	-0.0829	-0.16653

TRMT44	-0.020344848	-0.007678395	-0.02985	0.040252	-0.07259	0.071815
TRMT5	0.035645466	-0.024871572	-0.01153	0.113484	-0.34718	-0.16259
TRMT6	0.019281497	0.075567778	0.025627	0.036406	-0.07533	0.143404
TRMT61A	-0.004368768	-0.07356288	0.052877	-0.00391	-0.08468	-0.02002
TRMT61B	0.017843316	0.021679999	0.194573	0.028832	-0.06242	-0.04405
TRMU	0.014950265	-0.000766543	0.243116	0.011443	-0.05782	-0.15711
TRNAU1AP	-0.03229606	0.099332758	0.180675	-0.03275	0.071005	-0.14698
TRNP1	-0.039966222	-0.106854484	-0.19937	-0.02453	0.091398	0.323924
TRNT1	-7.21E-06	0.051884904	0.033119	0.051313	-0.15244	-0.05652
TRO	-0.048719457	-0.032566694	-0.04271	0.012319	0.034709	-0.14997
TROAP	-0.010568737	-0.003446353	-0.02972	0.182995	-0.58384	-0.02755
TROVE2	0.01655523	0.18605296	0.016086	0.036732	-0.12844	-0.13561
TRPA1	-0.060653036	-0.067699145	-0.0005	0.030707	-0.04995	0.008747
TRPC1	-0.05566247	-0.131058303	0.319565	-0.01874	0.119842	-0.19552
TRPC2	-0.030928001	-0.030077136	-0.17951	-0.03662	0.139097	0.233672
TRPC3	-0.062560341	-0.010372135	0.038159	0.002401	-0.00222	-0.01912
TRPC4	-0.073708793	-0.055315131	-0.02714	0.050972	-0.07021	-0.04521
TRPC4AP	0.023822752	-0.029362437	-0.20164	0.00884	-0.07295	0.025398
TRPC5	-0.053503386	-0.001241537	-0.01866	-0.00639	0.015816	-0.03576
TRPC5OS	-0.057061824	-0.029766923	0.009318	-0.0262	0.057223	-0.03147
TRPC6	-0.031368604	-0.051318746	-0.09674	-0.02315	0.073028	-0.13643
TRPC7	-0.029619103	-0.027280565	-0.01662	-0.02128	0.01746	-0.04613
TRPM1	-0.045991959	0.045270254	0.001014	-0.04557	0.075583	-0.04824
TRPM2	0.001728928	-0.00055065	-0.21392	-0.01457	0.047354	0.369134
TRPM2-AS	-0.032442108	-0.126667374	-0.07658	0.010545	-0.03607	-0.04136
TRPM3	-0.068225546	-0.074308143	0.118663	-0.00259	-0.00844	-0.0352
TRPM4	-0.017654436	-0.10123717	-0.14476	-0.03288	0.121021	0.543645
TRPM5	-0.031680347	-0.019627843	0.02437	0.013602	-0.00632	0.065864
TRPM6	-0.03097359	-0.032292593	-0.07521	-0.00645	-0.02663	-0.15813
TRPM7	-0.024883149	-0.010663383	0.2533	0.0245	-0.03121	0.012811
TRPM8	-0.050347268	-0.006544551	-0.00289	0.015507	-0.03678	0.147189
TRPS1	0.004673308	-0.046817565	-0.17921	-0.01658	0.047519	0.711219
TRPT1	0.017423906	-0.02511918	0.073921	0.035514	-0.11997	-0.12547
TRPV2	0.0194829	0.090876532	0.160656	0.019854	-0.05673	-0.04788
TRPV3	-0.056445431	-0.04657786	-0.05469	-0.00455	0.003273	-0.07312
TRPV4	-0.02948403	-0.123228593	-0.13645	-0.00599	0.004735	0.05069
TRPV5	-0.042486316	-0.098124615	-0.10979	-0.01226	-0.00012	-0.02581
TRPV6	-0.030515012	-0.111562222	0.00197	0.010179	-0.08776	-0.07132
TRRAP	0.008403007	0.034429832	0.192982	0.038292	-0.08757	-0.16472
TRUB1	-0.114250273	-0.105310031	0.011462	-0.00249	0.007142	0.05384
TRUB2	0.024923066	0.052905189	0.123404	0.041702	-0.10865	0.043467
TRY2P	-0.022401203	-0.013965983	-0.0115	0.069768	-0.18588	-0.09386
TSACC	-0.016996971	0.008948526	0.040104	-0.00937	-0.03796	0.020239
TSC1	0.050017624	0.047718768	0.056077	-0.0099	0.006416	-0.06501
TSC2	0.003189261	-0.00918313	0.025476	0.003607	0.007785	-0.01549
TSC22D1	-0.010110298	0.026952877	0.07159	0.032868	-0.05466	-0.03411
TSC22D1-/	-0.034032043	0.076488159	0.057129	-0.02834	0.063543	-0.16608
TSC22D2	0.006533536	0.012930503	0.01839	-0.00115	0.112231	0.003655
TSC22D3	0.006598486	-0.047685268	0.108935	-0.0898	0.214066	-0.13031
TSC22D4	0.010179913	0.033591924	-0.07293	-0.01277	0.026616	0.008163
TSEN15	0.028090893	0.090252338	0.175829	0.077689	-0.29624	-0.17616

TSEN2	-0.016942767	-0.091409835	0.157078	0.034626	-0.10912	-0.05459
TSEN34	0.029434944	-0.06806649	-0.33462	-0.00654	0.044176	0.189703
TSEN54	0.027138731	0.249034334	0.396534	0.005165	-0.04072	-0.40817
TSFM	0.006275395	0.03550746	0.001336	0.051859	-0.08546	-0.04985
TSG101	0.070768224	0.05258591	-0.3797	0.075684	-0.16352	0.129616
TSGA10	-0.047675718	-0.018721636	0.2992	-0.03849	0.068237	-0.13269
TSGA10IP	-0.022121851	-0.06546153	-0.04884	0.028887	-0.08861	0.163924
TSGA13	-0.029676985	-0.007226926	-0.0669	0.008463	-0.00583	0.058193
TSHB	-0.029164084	-0.017148509	-0.00115	0.012653	-0.03528	-0.10921
TSHR	-0.040934202	-0.022297444	-0.05818	0.019421	-0.04567	-0.03164
TSHZ1	0.020602624	0.134023308	0.252799	-0.01941	0.072262	-0.02469
TSHZ2	-0.002559855	-0.094771512	-0.01633	-0.04556	0.161789	-0.19703
TSHZ3	-0.004736726	0.049836952	-0.23851	-0.07114	0.186653	0.509969
TSIX	-0.010019565	-0.001528878	-0.04939	0.00023	-0.01316	0.086134
TSKS	-0.029579622	-0.09213083	0.028563	-0.02561	0.098185	0.227071
TSKU	-0.020775829	-0.078798952	0.041926	-0.01936	-0.06914	-0.13694
TSLP	-0.074589341	-0.031276981	-0.0477	-0.02975	0.067454	0.040348
TSN	-0.01036204	-0.003344615	0.03652	-0.02238	-0.1557	0.184391
TSNARE1	0.07861551	-0.067375442	0.078358	-0.05044	0.131891	-0.04101
TSNAX	0.040079711	0.061195264	-0.05393	0.033374	-0.06719	0.071871
TSNAXIP1	-0.018927391	-0.114286204	-0.01182	0.032167	-0.10174	0.050562
TSPAN1	-0.02613325	-0.043958153	-0.02672	-0.01115	-0.05309	-0.08815
TSPAN10	-0.033255596	-0.06049363	0.027144	0.029529	-0.09176	-0.08983
TSPAN11	-0.02553238	-0.062413964	0.071841	0.004242	-0.00314	0.090424
TSPAN12	-0.030781657	-0.013070777	-0.12984	0.011834	0.006673	-0.0989
TSPAN13	0.00145048	-0.076706277	-0.23681	-0.07775	0.194226	-0.2337
TSPAN14	0.033080156	0.131680595	0.167718	-0.05118	0.122642	0.177557
TSPAN15	-0.021832831	0.005921448	0.050988	-0.00616	0.040121	-0.06232
TSPAN16	-0.057691425	-0.045190396	-0.01318	-0.02251	-0.00582	0.027585
TSPAN17	0.00928135	0.058908046	0.059281	0.069818	-0.24647	0.036442
TSPAN18	-0.005458869	-0.084072059	0.191508	-0.05454	0.144438	-0.12123
TSPAN2	-0.017804283	0.121584191	-0.06146	-0.06187	0.185409	-0.06691
TSPAN3	0.009563732	-0.010928812	-0.02193	0.039356	-0.00283	-0.05471
TSPAN31	0.035010672	0.106214001	0.057294	-0.02227	0.040911	0.020616
TSPAN32	0.011757917	0.080404156	0.343112	-0.06411	0.176954	0.115396
TSPAN33	0.012808614	-0.120230532	-0.08847	-0.07185	0.175857	-0.06244
TSPAN4	0.005117118	-0.010051064	-0.13835	-0.02668	0.091169	0.691133
TSPAN5	-0.005017081	0.179945218	0.202168	0.020676	-0.07042	-0.2747
TSPAN6	-0.030288502	-0.120827467	0.401026	-0.04899	0.156789	-0.19562
TSPAN7	-0.016674585	-0.023733608	0.013099	0.087227	-0.21807	0.026575
TSPAN8	-0.028247452	-0.038372143	0.076993	0.056093	-0.15497	-0.03832
TSPAN9	-0.035887702	-0.043597351	0.007382	-0.06098	0.082101	-0.09364
TSPEAR	-0.062055691	-0.064698988	0.020698	-0.03644	0.104284	-0.05665
TSPO	0.03000654	-0.047464663	-0.12361	-0.09842	0.256489	0.512719
TSPO2	-0.02955542	-0.047362449	-0.14957	0.002803	-0.02971	0.008742
TSPY1	-0.034362452	-0.069865547	-0.10947	0.027641	-0.07952	0.110734
TSPY26P	-0.024138463	-0.132937871	0.111642	-0.00305	0.00521	0.171123
TSPYL1	-0.025608979	-0.013161519	0.06497	-0.02681	0.081422	0.007946
TSPYL2	0.023027788	-0.062700736	0.201459	0.001494	-0.02393	-0.29931
TSPYL4	0.028773066	0.054281737	0.293876	0.032904	-0.08773	-0.25606
TSPYL5	0.001493517	0.049719161	-0.10194	-0.05618	0.172653	-0.24899

TSPYL6	-0.044313003	0.014538524	-0.01461	0.017931	-0.0598	0.017738
TSR1	-0.000214308	0.004044638	-0.00731	0.064891	-0.13633	0.051464
TSR2	0.054777506	0.066777898	0.211437	0.041701	-0.17332	-0.30007
TSR3	-0.00269609	0.024961238	-0.00407	0.06466	-0.21946	0.007725
TSSC1	0.020569452	-0.030472019	0.112914	0.037332	-0.11604	-0.13214
TSSC4	0.014745337	0.037603976	-0.10903	0.016181	-0.10982	0.073974
TSSK1B	-0.040239179	-0.024129994	-0.11948	0.02393	-0.03124	0.055817
TSSK2	-0.035431067	-0.046992549	-0.09752	-0.00479	-0.01779	0.006835
TSSK3	-0.000296416	-0.059151478	-0.01198	-0.01293	-0.07049	-0.09347
TSSK4	-0.045202404	0.04707328	0.028573	0.003711	0.021138	-0.17767
TSSK6	-0.057250267	-0.007026532	0.100152	-0.01199	-0.02998	0.06749
TST	0.00833053	0.0015227	-0.33557	0.006622	-0.02185	0.352925
TSTA3	0.040543075	0.018069156	0.07856	0.094705	-0.30784	-0.17715
TSTD1	0.030036188	0.028348738	0.27073	-0.02895	0.057076	-0.63228
TSTD2	-0.038953276	-0.066176829	0.108559	0.008956	-0.01396	-0.0362
TTBK1	-0.024182717	-0.026007382	-0.0168	-0.00806	-0.05738	-0.08258
TTBK2	-0.013809018	0.004962723	-0.00647	0.006319	-0.01629	0.140735
TTC1	0.069856202	0.062034152	-0.03251	0.078907	-0.22708	-0.11764
TTC12	0.010805061	-0.04788818	0.149188	-0.08772	0.237308	-0.24867
TTC13	0.016950029	0.113156302	0.117615	0.000159	-0.00704	-0.09229
TTC14	0.038681599	0.057774811	0.171353	-0.01238	0.063363	-0.13495
TTC16	-0.005184204	0.094056243	0.477253	-0.0517	0.158093	-0.20303
TTC17	0.02353901	0.006735619	0.054082	-0.06388	0.120506	0.131325
TTC18	-0.014550627	-0.062667257	0.098499	0.012143	0.014937	-0.10506
TTC19	0.039325953	0.07118486	0.173075	0.045984	-0.1125	-0.03387
TTC21A	-0.018959806	-0.136977821	-0.25735	-0.03364	0.114284	0.109475
TTC22	0.065249127	0.149639015	0.136846	-0.04123	0.026604	-0.42316
TTC23	-0.028659316	-0.123785665	0.036763	0.023584	-0.07886	-0.02278
TTC23L	-0.045325052	-0.031170697	-0.06808	0.027128	-0.06948	-0.18939
TTC25	-0.022236097	-0.053713988	-0.06646	0.039904	-0.10307	0.108267
TTC26	-0.05171424	-0.074886651	0.067207	0.057986	-0.12689	-0.16468
TTC27	0.012069738	0.014252429	0.196728	0.006726	-0.03852	-0.13963
TTC28	-0.061661615	0.064662632	0.002016	0.032441	-0.01026	-0.20911
TTC28-AS1	-0.021777663	0.062408557	0.13765	0.003513	0.021193	-0.18032
TTC29	-0.054633707	-0.031727116	0.111932	0.057625	-0.11501	-0.00157
TTC30A	-0.06540631	-0.015820848	-0.12557	-0.02518	0.054605	0.198329
TTC30B	-0.030108525	0.036632049	-0.09636	-0.07076	0.210476	0.167727
TTC31	0.040808001	0.089762889	0.129664	-0.02028	0.037911	0.018922
TTC32	0.03544534	-0.061471192	0.131091	0.025472	-0.08276	-0.04079
TTC33	-0.004422136	0.057776844	-0.05308	0.028181	-0.09124	0.000652
TTC36	-0.026434237	-0.000947121	-0.02703	0.02636	-0.05015	0.088107
TTC37	0.022542323	0.054015418	0.166423	0.027995	0.001029	0.004744
TTC38	-0.009550467	-0.021848173	-0.03165	-0.01206	-0.07112	-0.04946
TTC39A	-0.050134831	-0.051811603	0.001357	-0.00224	0.055065	-0.08731
TTC39B	-0.022847384	0.09592184	0.27794	-0.08129	0.161047	-0.16664
TTC39C	-0.020203919	-0.052148788	0.260166	0.00419	5.36E-05	-0.0774
TTC4	0.016182845	0.015746719	0.030448	0.036532	-0.11638	0.032503
TTC40	-0.06779298	-0.075278509	-0.12499	-0.05426	-0.023	-0.14428
TTC5	-0.020214085	0.013642741	-0.04179	0.052057	-0.14601	-0.11729
TTC6	-0.047755221	-0.103420744	0.028288	-0.00597	0.077904	-0.12122
TTC7A	0.014705751	-0.141485733	-0.21043	0.101936	-0.00058	0.403405

TTC7B	-0.005400789	-0.044880998	-0.12528	-0.10402	0.270413	0.029341
TTC8	-0.016063501	-0.03765824	0.023751	-0.01061	0.044805	-0.08957
TTC9	-0.006404911	-0.077986425	0.269084	-0.07497	0.189339	-0.26092
TTC9B	-0.03083458	-0.081580224	-0.02104	0.048259	-0.12816	-0.03954
TTC9C	0.007625663	0.099096821	-0.04136	0.008488	-0.04893	0.078687
TTF1	0.033751041	0.036037207	-0.0412	0.015032	-0.05761	-0.18424
TTF2	-0.01047792	-0.003647584	0.010643	0.072273	-0.19071	0.125497
TTI1	0.01662587	0.042126502	0.010904	0.059399	-0.17019	-0.03602
TTI2	0.019956304	0.062151199	-0.12162	0.093305	-0.25598	0.023814
TTK	-0.014146403	0.024202257	0.037903	0.145635	-0.28496	-0.06759
TTL	0.034997131	0.016790714	-0.12715	-0.00119	-0.08257	0.252413
TTLL1	0.000791234	-0.007839958	0.091783	0.010564	-0.00425	0.003417
TTLL10	-0.074023974	-0.076255723	0.006812	-0.06444	0.054086	-0.06964
TTLL11	-0.061887348	0.01437222	0.096305	-0.03197	0.081303	0.11177
TTLL11-I	-0.031876155	-0.04358029	-0.11787	-0.00889	0.012476	-0.13819
TTLL12	0.016118568	-0.051526934	0.069199	0.045317	-0.19922	0.078019
TTLL13	-0.03774622	-0.044009959	0.024805	0.012702	-0.01337	-0.03706
TTLL2	-0.031283102	-0.039067747	0.043514	0.021877	-0.05235	-0.05393
TTLL3	-0.037730305	0.044399292	-0.20438	0.054504	-0.04526	-0.00845
TTLL4	0.004127086	-0.027443523	-0.12517	0.024953	-0.04236	0.044669
TTLL5	-0.072712158	-0.111448017	0.072418	0.122423	-0.01241	-0.23878
TTLL6	-0.030303163	-0.00905145	0.010073	-0.00233	0.016963	-0.06157
TTLL7	-0.037816937	-0.039082035	0.040943	-0.02053	0.082408	-0.05727
TTLL7-IT	-0.036794995	-0.033120501	-0.0539	-0.04771	0.161611	0.096563
TTLL9	0.012316108	-0.07443996	-0.12472	0.088568	0.046444	0.019959
TTN	-0.03620193	-0.074635734	0.310425	-0.06379	0.061188	-0.22864
TTN-AS1	-0.010682569	-0.001130474	-0.06108	-0.04437	0.109557	-0.12434
TTPA	-0.03760622	-0.043850075	-0.06734	-0.00438	0.030045	0.027951
TTPAL	0.020229226	0.138645851	0.249675	0.059566	-0.2121	-0.41233
TTR	-0.025507188	-0.07085153	-0.05327	-0.00752	-0.07398	-0.04612
TTTY10	-0.032995412	-0.09121053	-0.08227	0.019479	-0.03599	-0.03289
TTTY11	-0.02765269	-0.001905884	0.018855	-0.01338	-0.01209	-0.0755
TTTY12	-0.029205439	-0.045347799	-0.04678	0.006944	-0.01366	-0.07834
TTTY13	-0.034574402	-0.110221472	-0.00516	-0.00493	0.030883	0.002422
TTTY15	-0.005502335	0.000776657	0.139777	-0.02448	0.080187	-0.13821
TTTY5	-0.0285182	-0.035906021	0.032763	0.050563	-0.1358	0.055552
TTTY7	-0.035592122	-0.007431502	-0.08957	0.015739	-0.03479	-0.02527
TTYH1	-0.039265773	0.030076326	0.005719	0.0116	0.003129	0.010631
TTYH2	0.017444724	-0.101524114	-0.08404	-0.09519	0.273629	0.40506
TTYH3	0.017392518	-0.073488721	-0.17756	-0.0518	0.082397	0.371983
TUB	-0.047649482	-0.06479809	0.00418	-0.01504	-0.05449	-0.08378
TUBA1A	0.040991517	-0.062567346	-0.20929	0.016653	-0.04201	0.095044
TUBA1B	0.063936444	-0.024394616	-0.25581	0.151988	-0.33646	-0.12802
TUBA1C	0.058546203	-0.051817844	-0.22072	0.142315	-0.31276	-0.10969
TUBA3FP	-0.023836346	-0.018615052	0.169001	0.003135	-0.02094	-0.01656
TUBA4A	0.041445299	-0.01025769	0.110834	0.02953	-0.06225	-0.11376
TUBA4B	-0.006096004	-0.075821878	-0.01889	0.016181	-0.13104	-0.16398
TUBA8	-0.033974633	-0.052389215	-0.1054	-0.04041	0.094374	0.048533
TUBAL3	-0.032520079	-0.065097416	-0.00966	0.020481	-0.03488	-0.05624
TUBB	0.060697561	0.101259652	0.074686	0.111169	-0.32278	-0.18687
TUBB1	-0.002137454	-0.025031525	-0.05916	-0.22934	0.646845	0.090859

TUBB2A	0.001810864	-0.047169772	0.13804	0.043037	-0.12695	0.020871
TUBB2B	-0.022957108	-0.02926113	-0.02862	-0.01025	0.005786	0.002651
TUBB3	0.034108523	-0.016315552	-0.07468	0.089555	-0.25439	-0.05001
TUBB4A	-0.026051856	-0.035322068	0.167264	0.011158	-0.01935	-0.08281
TUBB4B	0.052209517	0.057727392	-0.11665	0.10894	-0.29394	-0.00932
TUBB6	0.012028012	-0.117155373	-0.26865	-0.07439	0.192015	0.177177
TUBB7P	-0.026246147	-0.045597648	-0.02752	0.013103	-0.01522	0.17223
TUBBP5	-0.022078656	-0.052043298	-0.15662	-0.00947	0.040643	0.044387
TUBD1	0.000788226	0.046366837	0.193017	-0.00756	0.036628	-0.11206
TUBE1	-0.010390533	0.072256043	0.271384	0.036013	-0.10689	-0.24735
TUBG1	0.009090715	0.036173341	-0.09636	0.125877	-0.32212	0.154624
TUBG2	-0.012846152	-0.043946353	0.254128	-0.00033	0.020739	-0.16004
TUBGCP2	-0.016416752	-0.046754161	0.008963	-0.09255	0.128491	0.339844
TUBGCP3	-0.022083398	0.004563853	-0.22025	0.063502	-0.14513	0.171713
TUBGCP4	0.002086964	-0.006208933	0.037553	0.032596	-0.15899	-0.25419
TUBGCP5	-0.003786437	0.020558563	0.17401	0.08622	-0.17842	-0.19644
TUBGCP6	-0.006678874	-0.030704224	0.21917	-0.03994	0.138082	-0.09665
TUFM	0.031638248	-0.006099517	0.033179	0.063168	-0.18404	0.182295
TUFT1	-0.008712504	-0.019958481	-0.11787	-0.03147	0.071398	0.039726
TUG1	0.048540782	-0.003872711	0.071719	0.001428	0.009181	-0.30032
TULP1	-0.02705471	-0.069101559	-0.01178	0.019433	-0.03341	0.138794
TULP2	-0.017904417	-0.107596779	-0.07706	0.000288	0.015678	-0.0084
TULP3	0.011697226	0.047126248	0.082497	0.017726	-0.08507	-0.16062
TULP4	0.007553077	0.066705266	0.072379	-0.02028	0.08906	-0.06453
TUNAR	-0.023149265	-0.037875203	-0.12037	0.018097	-0.03543	-0.01299
TUSC1	-0.005082846	-0.069590979	-0.27305	-0.06802	0.145836	0.01892
TUSC2	0.035673943	0.027544977	-0.06567	0.023461	-0.11295	-0.00972
TUSC3	0.014025379	0.016473384	-0.00451	0.028715	-0.10077	-0.01497
TUSC5	-0.021960476	-0.087032028	-0.07944	-0.00972	-0.02405	-0.02157
TUSC7	-0.0720553	-0.071427502	-0.03753	0.034023	-0.07158	-0.09346
TUSC8	-0.033809628	0.005400421	-0.0408	-0.00178	0.027954	-0.13712
TUT1	-0.01002554	-0.050855522	0.006633	0.017194	-0.14516	-0.10235
TVP23A	-0.019339669	-0.028991652	-0.03212	-0.00613	0.003926	0.00373
TVP23B	0.026640024	0.117462802	0.113107	0.022153	-0.08122	-0.09996
TVP23C	-0.016566322	-0.047826155	0.021281	0.012145	-0.13994	-0.07245
TWF1	-0.043299813	-0.030255596	-0.00799	0.002031	0.001534	0.101412
TWF2	0.046145199	-0.009789294	-0.06797	0.009596	-0.03728	0.253982
TWIST1	-0.028945907	0.01129018	-0.05053	-0.00921	0.049934	-0.09491
TWIST2	-0.042152937	-0.015706607	-0.12657	-0.00456	0.041158	-0.00129
TWISTNB	0.002970395	0.050358376	0.121977	0.093689	-0.23526	-0.092
TWSG1	0.008785493	0.064162892	-0.02133	0.059672	-0.16095	-0.01128
TXK	0.001400336	0.221263956	0.381691	-0.0625	0.157062	-0.31552
TXLNA	0.006114691	0.059338504	-0.04656	0.057629	-0.19154	0.00045
TXLNB	-0.044360316	-0.033371724	-0.03678	-0.02785	0.070779	-0.12584
TXLNG	0.01674078	0.019045548	0.216266	0.035157	-0.06236	-0.1506
TXLNGY	-0.020431453	-0.040539963	0.233866	-0.07596	0.18344	-0.06962
TXN	0.025398756	-0.040579916	-0.17239	0.08573	-0.23362	0.044583
TXN2	0.043476375	0.004649446	0.126006	0.056528	-0.17166	0.254423
TXNDC11	0.044492281	-0.024626781	0.06896	-0.01657	0.03028	-0.00022
TXNDC12	0.122742987	0.017848506	-0.12668	0.029692	-0.03053	0.161762
TXNDC15	0.05071124	0.141800366	-0.06339	0.052786	-0.16706	0.358221

TXNDC16	0.003580063	0.040581847	-0.17175	0.018926	-0.05163	0.17512
TXNDC17	0.042627265	0.012976963	-0.21293	0.077184	-0.20609	0.290739
TXNDC2	-0.041139947	-0.092165082	-0.0418	-0.00769	-0.00429	-0.12083
TXNDC8	-0.040147727	-0.050560162	0.171063	0.033749	-0.0836	-0.09445
TXNDC9	0.045345701	0.163926136	0.01611	0.057883	-0.15669	-0.05081
TXNIP	0.032722331	0.061819708	0.032121	-0.05172	0.186568	-0.12978
TXNL1	-0.016316599	0.010499512	-0.10079	0.007291	-0.02866	0.031907
TXNL4A	-0.032379894	0.019048268	0.028746	0.034119	-0.03121	0.100696
TXNL4B	0.00571317	-0.011190225	-0.09843	0.023495	-0.1195	0.042592
TXNRD1	0.0258716	0.063236527	-0.20715	0.068725	-0.18619	0.03065
TXNRD2	-0.010820161	-0.107250741	0.002877	-0.0312	0.063337	0.37872
TYK2	0.054306493	-0.009907736	-0.27704	0.022122	-0.02683	0.101549
TYMP	0.007925375	-0.123131162	-0.3224	-0.06163	0.15929	0.468862
TYMS	-0.010232514	0.096741358	0.099052	0.203297	-0.33561	-0.15422
TYMSOS	-0.025788341	-0.031224132	-0.08075	0.123238	-0.33256	0.110843
TYR	-0.037001226	-0.024769887	-0.07039	0.008471	-0.00108	-0.0508
TYRO3	-0.049806712	-0.078013906	0.074104	0.000394	-0.01763	-0.01061
TYRO3P	-0.040019204	-0.047158557	0.014302	0.004725	-0.01525	-0.13672
TYROBP	0.020134403	0.11136556	-0.42215	-0.06878	0.208474	0.36713
TYRP1	-0.053254403	-0.040525553	0.105168	0.026393	-0.02659	-0.0425
TYSND1	0.015898435	-0.135840218	0.166089	0.000227	0.025218	-0.01797
TYW1	0.037888287	-0.016054345	0.245544	0.012659	-0.07635	-0.26069
TYW3	0.031005276	0.019704739	0.118869	0.047182	-0.13302	0.240001
TYW5	0.005789063	0.021614452	0.125483	0.047376	-0.16007	-0.16601
U2AF1	0.022355574	-0.01526787	-0.05567	-0.01529	0.052608	-0.23173
U2AF1L4	0.021676607	-0.121732713	0.251629	-0.03654	0.061259	-0.20336
U2AF2	-0.010575172	-0.068399035	0.074762	0.022082	-0.12826	-0.01266
U2SURP	0.02346809	0.032607779	0.170429	0.029338	-0.11897	-0.05328
U47924.27	-0.039252778	-0.0145997	-0.06122	-0.0049	0.040164	0.176719
U47924.29	-0.04046247	-0.102474366	-0.01283	-0.02337	0.074754	0.031743
U91328.2	-0.021408633	0.032767746	-0.07584	-0.00242	0.014494	0.070453
U91328.20	-0.025178846	-0.059784387	-0.14125	-0.01521	-0.01571	0.025314
UACA	-0.064574412	-0.095900532	-0.16813	-0.06013	0.080897	0.321386
UAP1	0.019209749	0.18889487	0.153987	0.074	-0.20442	-0.32832
UAP1L1	-0.004500197	-0.13716046	0.09419	-0.02656	0.08312	0.42004
UBA1	0.02314929	0.012811711	-0.20267	0.068692	-0.17155	0.075595
UBA2	0.035706243	0.069396469	0.206948	0.062391	-0.18077	-0.01446
UBA3	0.093848285	0.031602872	-0.20427	0.032944	-0.06234	0.134678
UBA5	0.026943292	0.089013795	0.101177	0.085088	-0.1834	-0.12935
UBA52	0.098123386	0.082857153	0.049755	0.123368	-0.20768	0.065431
UBA6	-0.03337482	0.084674378	-0.04632	0.068454	-0.19317	-0.15307
UBAC1	0.027424392	-0.046949338	-0.19395	0.027102	-0.08663	0.450743
UBAC2	0.053070422	-0.031023204	0.233355	-0.0195	0.050866	0.144807
UBAC2-AS1	-0.037012378	-0.026735228	0.098565	0.032945	-0.11602	-0.05421
UBALD1	-0.027639679	-0.008030552	-0.06469	-0.06284	0.061751	0.045892
UBALD2	0.043770213	-0.037758218	0.173501	0.047219	-0.14535	-0.21488
UBAP1	0.053804382	0.004752567	-0.13564	0.005902	-0.02095	0.046466
UBAP1L	-0.022695333	-0.10172647	0.11053	-0.01273	0.058287	0.022774
UBAP2	0.034593695	0.036513967	0.215669	0.062968	-0.1816	-0.00399
UBAP2L	0.029380599	-0.053577337	0.063232	0.109096	-0.25786	-0.05896
UBASH3A	-0.001272971	-0.10964255	0.54755	-0.02891	0.064406	-0.21145

UBASH3B	-0.0439309	-0.051581308	-0.01404	-0.0164	-0.01598	0.06529
UBB	0.07213279	0.331561446	-0.18045	0.143951	-0.19119	-0.30131
UBC	0.094793652	0.185957825	-0.3355	0.145642	-0.12719	-0.12232
UBE2A	0.084921299	0.097650678	-0.41911	0.117923	-0.30275	0.201746
UBE2B	-0.040642537	0.027510697	-0.12835	0.024544	-0.00204	-0.0961
UBE2C	-0.003500154	-0.012738721	0.006766	0.167688	-0.49743	-0.12681
UBE2D1	0.001282109	-0.055778207	-0.34121	-0.04499	0.131071	0.402378
UBE2D2	0.105468821	0.112382514	0.188605	0.059054	-0.1662	-0.429
UBE2D3	0.044574346	0.026179231	-0.08146	0.031288	-0.01592	-0.07289
UBE2D4	0.007620689	-0.067309675	0.160481	-0.00529	-0.09541	-0.09587
UBE2DNL	-0.04155329	-0.016836801	-0.0191	-0.00309	0.036188	0.145679
UBE2E1	0.079369985	0.076711753	-0.21768	0.027214	-0.05856	0.402984
UBE2E2	0.012858534	-0.182245929	-0.24988	-0.1033	0.299675	0.370961
UBE2E2-AS	-0.040635837	-0.045553617	-0.18754	0.02099	-0.03527	-0.00674
UBE2E3	0.048133293	0.100978365	-0.15031	-0.05549	0.149685	0.100793
UBE2F	0.051323644	0.282529363	-0.15646	-0.06518	0.140661	0.212656
UBE2G1	0.023798672	0.131005456	-0.00424	0.052049	-0.23764	-0.29126
UBE2G2	0.0364382	0.029876853	0.284604	0.008957	-0.02319	-0.02646
UBE2H	0.013313373	0.013679472	-0.02451	0.010942	-0.04565	-0.08916
UBE2I	-0.046460935	-0.016800076	-0.03977	-0.07856	0.083244	0.00903
UBE2J1	0.03915534	-0.045060628	-0.34745	-0.05214	0.079388	-0.09821
UBE2J2	-0.013414985	-0.035632126	-0.02462	0.01105	-0.11423	0.047833
UBE2K	0.061089654	-0.028699685	-0.0476	0.050046	-0.11764	0.150896
UBE2L3	0.062866866	0.159538423	-0.04169	0.020946	-0.09851	-0.0763
UBE2L6	0.086127559	0.126594024	-0.09498	0.052554	-0.10492	0.098173
UBE2M	0.02360605	0.022964084	-0.07417	0.115533	-0.34714	0.073536
UBE2N	0.018550125	0.027414968	0.124623	0.046845	-0.16303	-0.07791
UBE2NL	0.06379873	0.005116167	0.043434	0.0838	-0.21382	-0.16945
UBE2O	-0.001715861	-0.053782439	0.131665	-0.02188	-0.04246	-0.24619
UBE2Q1	-0.026659992	0.116875692	0.027303	0.052521	-0.16231	-0.03095
UBE2Q2	0.036158587	0.174332539	0.133713	-0.01804	0.050777	-0.03614
UBE2Q2L	-0.020561609	0.086934779	0.164701	-0.07505	0.20354	-0.23613
UBE2QL1	-0.055896424	-0.126522754	-0.07982	-0.02555	0.103419	-0.0098
UBE2R2	0.066447908	-0.018611988	-0.35324	-0.00408	0.043072	0.271508
UBE2S	0.00781874	0.025380743	0.028849	0.164298	-0.46921	-0.00493
UBE2T	-0.006137776	0.021079565	0.108953	0.169893	-0.39115	-0.16391
UBE2U	-0.08557958	-0.091420523	-0.04397	-0.02489	0.057633	-0.0144
UBE2V2	-0.001276282	0.106120984	-0.04646	0.055314	-0.12465	0.081481
UBE2W	-0.017787249	-0.043908109	-0.25123	-0.01156	-0.05839	0.118515
UBE2Z	0.058187825	0.114731313	0.077024	0.066589	-0.15119	-0.04175
UBE3A	0.036643954	0.075315173	0.127197	0.06895	-0.14173	0.026617
UBE3B	-0.082762584	-0.011925733	-0.11998	0.05359	-0.0318	-0.00414
UBE3C	0.005278751	-0.021150157	0.092216	-0.01713	-0.11152	0.028724
UBE3D	-0.073417713	-0.032740577	0.157692	-0.04634	-0.00923	-0.12067
UBE4A	0.123295801	0.131110238	-0.03627	0.052922	-0.08729	0.000731
UBE4B	-0.004687046	0.041198643	-0.17506	0.013676	-0.09644	0.117655
UBFD1	0.012411534	0.07699564	-0.03868	0.083351	-0.28706	-0.09878
UBIAD1	0.010720354	-0.031072499	0.11222	0.018426	-0.04539	-0.12256
UBL3	0.053919523	0.034512318	-0.01275	-0.11289	0.309053	-0.01503
UBL4A	0.058488981	0.136974625	0.115373	-0.00377	-0.03827	-0.06612
UBL4B	-0.020211284	-0.078746816	-0.09748	0.007604	-0.08723	0.054894

UBL5	0.089888682	0.062902834	0.04678	0.115397	-0.28049	0.042687
UBL7	0.030750181	-0.044841842	-0.05211	0.027793	-0.09864	0.03477
UBL7-AS1	-0.001274872	0.015445232	0.062933	0.070173	-0.1638	-0.00018
UBLCP1	0.057902625	0.227222958	0.148108	-0.00495	0.008533	-0.26261
UBN1	0.055407873	0.082856206	-0.18691	0.019642	-0.04134	-0.08598
UBN2	-0.0098969	0.076561926	0.025146	-0.00982	0.058815	0.065087
UBOX5	-0.001180043	-0.063784581	0.069411	-0.03333	-0.03689	-0.15355
UBP1	0.057720717	0.084445451	0.227111	0.01621	-0.03444	-0.03854
UBQLN1	0.035268829	0.085594558	-0.01924	0.006009	-0.12536	0.03592
UBQLN2	0.028538278	0.033232445	0.192011	0.00285	0.010042	-0.0303
UBQLN3	-0.040249789	-0.036939627	0.052767	-0.00203	0.025843	0.070547
UBQLN4	0.03087763	-0.052898691	0.121532	0.051119	-0.20241	-0.15331
UBQLNL	-0.033980364	-0.086135912	0.121729	-0.02373	0.093955	-0.09817
UBR1	0.031796333	0.210025512	0.187703	0.051232	-0.14989	-0.25905
UBR2	0.023914984	0.041790868	-0.2037	-0.02689	0.029101	-0.07844
UBR3	0.002588282	0.024254416	-0.05156	0.03136	-0.04555	0.057029
UBR4	-0.018373767	-0.02153397	0.028036	0.041547	0.018935	-0.10883
UBR5	0.064232194	0.143473009	0.002948	0.055175	-0.10541	-0.15503
UBR7	0.029574702	0.101317929	-0.03045	0.062341	-0.17241	-0.13413
UBTD1	-0.023995082	-0.067796729	-0.14076	-0.03382	0.022284	0.13062
UBTD2	0.018055702	-0.04128913	-0.16273	0.01348	-0.07989	0.151888
UBTF	-0.030869053	0.012608291	0.185215	-0.03247	-0.04751	-0.1899
UBXN1	0.054155331	-0.024151472	0.033735	0.036447	-0.07347	0.050732
UBXN10	-0.069281807	-0.051870713	0.104265	0.008057	-0.02245	0.022174
UBXN10-AS	-0.019717738	-0.030485497	0.205273	0.000829	-0.08542	-0.15835
UBXN11	0.004224412	-0.155349264	0.018051	-0.07227	0.197509	0.59762
UBXN2A	0.063482129	0.10408655	0.15318	0.037126	-0.05081	-0.25258
UBXN2B	0.02589312	-0.010495498	-0.29884	0.005527	0.004248	0.249708
UBXN4	0.085566015	0.149983465	0.07461	0.062359	-0.10421	-0.24676
UBXN7	0.020763415	0.070774224	0.003762	-0.016	0.018716	-0.00519
UBXN7-AS	-0.02353546	0.006946633	0.089906	0.015607	-0.04812	-0.14147
UBXN8	0.013156337	0.089536583	0.081185	0.034804	-0.07553	0.12472
UCA1	-0.025530565	-0.040139066	0.042798	0.003006	-0.00296	0.07016
UCHL1	-0.031659097	-0.096107952	-0.0438	0.000923	-0.02659	-0.01175
UCHL3	0.030812454	-0.006475138	-0.05735	0.065153	-0.16063	0.265004
UCHL5	-0.004045342	0.173794386	0.058297	0.094441	-0.23311	-0.01308
UCK1	0.01862786	0.003632717	0.000193	0.01558	-0.12936	0.037495
UCKL1	-0.047596924	-0.05387816	0.160721	0.003353	-0.08156	0.101434
UCMA	-0.032143054	-0.036222545	-0.07892	0.006865	-0.01079	-0.01722
UCN	-0.014999199	-0.111139928	0.034	0.008214	0.004521	0.158637
UCN2	-0.029410371	-0.042054692	-0.0738	0.035541	-0.08348	-0.02031
UCN3	-0.027621006	-0.095703023	0.047779	0.02419	-0.07054	-0.08387
UCP1	-0.037285258	-0.064104801	-0.01502	0.012093	-0.01496	-0.0945
UCP2	0.036847297	0.034155017	0.074888	-0.03292	0.115105	-0.02795
UCP3	-0.030376973	0.019411185	-0.01069	-0.01519	0.056985	0.038223
UEVLD	-0.011829584	0.152574407	-0.18449	-0.04169	0.127722	0.242969
UFC1	0.056217077	0.108873541	0.217326	0.034472	-0.09051	0.027089
UFD1L	0.104197734	0.144876639	0.065577	0.124162	-0.3217	-0.11642
UFL1	0.022117752	0.048082459	0.133583	0.055462	-0.00533	-0.04857
UFM1	-0.072172412	-0.113191849	-0.05022	-0.0214	0.010599	-0.03043
UFSP1	-0.021760842	-0.08168689	0.000624	0.011933	-0.03229	0.011464

UFSP2	0.019565941	0.047757341	0.141521	0.01782	-0.05062	-0.07599
UG0898H09	-0.172962733	-0.125394923	-0.01728	0.044067	0.093621	-0.1093
UGCG	0.026837783	-0.057853946	-0.00949	-0.00281	0.045372	-0.3891
UGDH	0.016472226	0.086967245	0.000892	0.007474	-0.0493	-0.00083
UGGT1	0.020596221	0.104655252	-0.16111	0.053944	-0.28646	0.117293
UGGT2	-0.026928093	-0.13682465	-0.17993	-0.0674	0.31339	0.559209
UGP2	0.038816721	0.121936578	0.151899	0.000614	0.008082	-0.22652
UGT1A1	-0.034554527	-0.040452732	0.021646	0.031557	-0.06731	-0.0269
UGT1A6	-0.043136395	-0.05621983	-0.01378	0.02649	-0.05199	0.031219
UGT2A3	-0.082887089	-0.076880898	0.032429	-0.01016	0.046908	0.039749
UGT2B15	-0.025477258	-0.092985161	0.047192	0.050518	-0.14458	-0.13061
UGT2B17	-0.018595438	-0.036709139	-0.09647	-0.00104	0.024195	-0.19124
UGT2B28	-0.023542837	-0.078063733	0.070202	-0.00991	-0.00096	-0.09162
UGT2B4	-0.127931481	-0.090072964	-0.07114	0.026044	0.048893	-0.0343
UGT3A1	-0.002173429	-0.059084672	-0.05739	-0.01041	-0.03311	0.080179
UGT8	-0.027487334	-0.065562698	-0.14339	-0.03208	0.088287	-0.20832
UHMK1	0.037736414	0.220923804	0.1696	0.019965	-0.06672	-0.12845
UHRF1	-0.004390407	0.014612917	0.001143	0.107626	-0.29117	-0.1661
UHRF1BP1	-0.017867647	0.045126038	0.0581	0.035848	-0.06344	0.068005
UHRF1BP1I	-0.02692042	-0.032496599	-0.19054	0.04161	-0.0454	0.141128
UHRF2	0.057323244	0.059294214	0.186628	-0.00039	0.004796	-0.47621
UIMC1	0.058477282	-0.020812797	-0.20786	0.041255	-0.08587	-0.1721
ULBP1	-0.036354912	-0.033467056	0.038588	0.066	-0.16511	0.077683
ULBP2	-0.017852434	0.030266657	0.025098	0.168075	-0.38535	-0.05197
ULBP3	-0.037474977	-0.048412348	-0.09174	0.026435	-0.04678	-0.03327
ULK1	0.013317907	-0.065425177	-0.22484	-0.02038	0.058516	0.059348
ULK2	0.020688678	0.021379224	0.028099	-0.08985	0.244015	0.414989
ULK3	0.029662822	0.109972792	0.338867	0.008359	-0.03575	-0.09736
ULK4	0.028655019	0.080528647	0.104223	0.001314	0.130574	0.044277
UMOD	-0.032094148	-0.024451007	0.033669	-0.01065	0.040474	-0.03989
UMODL1	-0.056592523	-0.040951404	0.067526	0.040386	-0.11919	0.043221
UMPS	0.00436355	0.069891596	0.08698	0.064587	-0.2174	0.048636
UNC119	0.04256326	-0.082320374	-0.31618	-0.03143	0.079721	0.060177
UNC119B	0.024548165	0.005932974	0.180331	0.072872	-0.20155	-0.20222
UNC13A	-0.032587624	-0.105861061	0.05051	0.017921	-0.04685	0.069463
UNC13B	-0.018994314	-0.08243764	-0.07644	-0.05897	0.146949	0.195327
UNC13C	-0.030819766	-0.028742603	-0.01381	0.122605	-0.10804	-0.01047
UNC13D	-0.027242759	-0.010635067	0.020399	-0.05369	0.133299	0.004965
UNC45A	0.043498612	0.000839524	0.092172	0.018035	-0.13317	-0.04555
UNC45B	-0.033830961	0.160653142	0.212176	-0.00476	0.041581	-0.12179
UNC50	0.065222038	0.188505551	-0.165	0.037239	-0.10412	0.186517
UNC5A	-0.043929213	-0.019464237	-0.0361	0.019051	-0.03763	0.050129
UNC5B	-0.036886692	-0.028574571	-0.10166	-0.01372	0.00412	0.069872
UNC5B-AS1	-0.02789346	-0.037135518	-0.00674	0.004191	0.001886	0.121708
UNC5C	-0.070583941	-0.082706239	-0.11081	-0.06349	0.034407	0.148542
UNC5CL	-0.006811804	-0.023609686	0.103975	-0.02269	0.083943	-0.17023
UNC5D	-0.036123581	-0.015980919	0.096234	0.086154	-0.23901	-0.05208
UNC79	-0.030453336	-0.082002003	-0.10654	0.025745	-0.08435	0.067984
UNC80	0.000959548	-0.028193021	0.039122	-0.01212	-0.02874	-0.00707
UNC93A	-0.070461322	-0.030501776	0.127083	-0.02848	0.091709	0.082963
UNC93B1	-0.07770675	-0.110280313	-0.27378	-0.15894	0.258414	0.20629

UNG	0.012123758	0.109830934	0.189719	0.024746	-0.05596	-0.26069
UNK	0.057313646	0.118661661	0.147627	0.093512	-0.03093	-0.13238
UNKL	0.018006114	0.022002532	-0.02522	0.002063	0.010279	-0.13222
UPB1	-0.021774864	-0.100519766	-0.05005	0.005694	-0.02054	-0.03342
UPF1	0.01721812	0.003168621	-0.13257	0.028555	-0.09725	-0.02781
UPF2	0.061623219	0.075413913	-0.1028	0.047902	-0.11786	-0.51127
UPF3A	0.012434749	0.079697094	0.192341	0.012291	-0.00795	-0.09681
UPF3B	0.021120246	0.078044414	0.063356	0.086593	-0.22428	-0.14523
UPK1A	-0.029063491	-0.024733831	0.105663	0.020613	-0.05393	-0.07502
UPK1B	-0.019046675	-0.004093135	0.007513	0.063335	-0.15087	-0.02197
UPK2	-0.040407173	-0.008672966	-0.02911	-0.01511	0.071304	0.079374
UPK3A	-0.007815639	-0.107206536	-0.26008	-0.02978	0.091566	0.376298
UPK3B	-0.029449679	0.007189132	0.055295	0.029969	-0.08793	-0.10306
UPP1	0.019806587	0.095314929	0.082279	0.006027	-0.02612	0.318865
UPP2	-0.053710394	-0.056049958	0.002256	0.02602	-0.06701	-0.07181
UPRT	0.02144411	0.055175693	0.068885	-0.01123	0.038561	0.102849
UQCC1	-0.054456907	-0.022698669	0.194939	0.031151	-0.0904	-0.12201
UQCC2	-0.026295964	-0.003963675	0.036197	0.034237	-0.0899	-0.06499
UQCR10	0.063250378	0.040339619	0.044429	0.082372	-0.18938	0.223414
UQCR11	0.086813646	0.035951066	0.171585	0.03728	-0.11227	0.112484
UQCRB	0.052791146	0.016258207	0.229543	0.083952	-0.19117	0.139068
UQCRC1	0.038775546	0.0117442	-0.15518	0.078452	-0.21532	0.402656
UQCRC2	0.064349313	0.064029663	0.054446	0.048914	-0.13962	0.146412
UQCRFS1	0.070976267	0.07043977	0.083251	0.085745	-0.2079	0.219942
UQCRQ	0.076265778	0.072365364	0.074074	0.119985	-0.3282	0.249993
URB1	0.00111023	0.010522803	0.080666	0.062915	-0.08075	-0.04858
URB2	-0.000599976	-0.011837084	0.080089	0.049943	-0.16126	-0.08851
URGCP	0.017251686	-0.029646032	0.351717	0.004822	-0.03648	-0.26418
URI1	0.012602881	0.071468946	0.277379	0.013007	-0.00121	-0.18501
URM1	0.026726863	-0.027538387	0.160278	0.008081	-0.04053	0.098953
UROC1	-0.051647293	-0.141321674	-0.05916	0.00774	0.011224	-0.02157
UROD	0.036728486	0.006638625	0.023335	0.04326	-0.11494	0.227402
UROS	-0.035205492	0.004677467	0.03373	0.144339	-0.13211	-0.01557
USB1	0.050973311	0.123276366	-0.15908	0.053279	-0.03607	0.098108
USE1	0.025583782	-0.066713971	0.20328	0.0466	-0.1228	0.068562
USF1	0.002704615	-0.112983518	-0.25304	0.021708	-0.0733	0.186095
USF2	0.056451027	-0.042955255	-0.04345	-0.00487	-0.0131	-0.17011
USH1C	-0.070051677	-0.041192037	3.23E-06	-0.04329	0.028587	-0.01212
USH1G	-0.007406264	-0.058300896	0.026032	-0.0177	-0.07633	-0.10953
USH2A	-0.023838478	-0.013784804	0.178779	0.023619	-0.03209	-0.05479
USHBP1	-0.022595564	-0.01244602	0.000799	0.01963	-0.03733	0.178295
USMG5	0.085275862	0.108077296	0.065136	0.126635	-0.27608	0.020415
USO1	0.044968582	0.129183375	-0.01197	0.022958	-0.07156	-0.0416
USP1	0.066252075	0.183924361	0.020153	0.079503	-0.22867	-0.25009
USP10	0.035831585	0.002320574	-0.12708	0.029182	-0.07813	0.030476
USP11	0.030926198	0.107107144	0.37922	0.030324	-0.09473	-0.4835
USP12	0.003344621	0.01059001	0.004588	0.022099	-0.07925	-0.0368
USP13	-0.028805651	-0.098372792	0.115194	0.134132	-0.27393	-0.21643
USP14	0.016367113	0.06945163	0.281564	0.019867	-0.13458	-0.17391
USP16	0.044008828	0.097530559	0.266959	0.007557	-0.10198	-0.11155
USP18	-0.000152047	0.04162039	0.012136	0.019734	-0.06073	-0.00513

USP19	0.035981903	0.003529408	-0.1253	0.046089	-0.10854	0.038439
USP2	0.000136338	-0.062405612	0.024989	0.000746	-0.11425	-0.06021
USP2-AS1	-0.044453587	-0.03165947	0.052731	-0.02066	0.087261	-0.00356
USP20	0.012157988	0.074726241	0.277703	-0.00635	-0.00052	-0.25046
USP21	0.020231524	0.013130346	0.161648	0.039173	-0.09944	-0.0464
USP22	-0.107065909	0.006011381	0.108905	0.058152	-0.05852	-0.23231
USP24	0.043890267	0.127566782	0.042111	0.005088	0.05613	-0.19912
USP25	-0.018328112	-0.003620217	0.017308	-0.00932	0.064095	0.053754
USP26	-0.040998435	-0.035675917	-0.07098	0.012573	-0.01985	-0.04915
USP27X	-0.014311392	0.018402485	0.017712	-0.01167	0.06077	0.048923
USP27X-AS	-0.031090318	-0.094009273	0.015235	-0.03401	0.121687	0.176492
USP28	0.006808656	0.211354795	0.026888	-0.01776	-0.02915	-0.15929
USP29	-0.036748696	-0.026377974	0.131111	0.020333	-0.03941	-0.07455
USP3	0.04844226	-0.046801251	-0.14623	-0.05126	0.165715	0.405169
USP3-AS1	-0.02582186	-0.037543525	0.003315	-0.04789	0.151926	0.089421
USP30	0.013255403	0.029963245	0.01025	0.010464	0.018641	0.089658
USP30-AS	-0.002805882	0.115507063	0.188877	0.0555	-0.12816	-0.16504
USP31	-0.040006375	0.102447596	0.096533	0.005737	0.008111	0.001801
USP32	-0.032684165	-0.114423502	-0.28785	-0.0041	0.018692	0.082045
USP32P2	0.030683728	-0.05070224	-0.12816	-0.00382	0.043559	0.19641
USP33	0.05437488	0.090999957	0.08685	-0.09706	0.257201	-0.16421
USP34	0.013046345	0.040397882	0.097876	-0.02027	0.027843	-0.29306
USP35	-0.002347103	-0.04776621	0.0299	-0.01145	0.018478	0.139014
USP36	0.035970532	-0.049213302	0.304169	-0.00679	-0.02374	-0.16234
USP37	0.010921919	0.071141276	0.043874	0.056353	-0.18807	-0.0882
USP38	0.019399947	0.15353863	-0.15887	0.053369	-0.12026	-0.13559
USP39	0.059282083	0.022416079	0.026884	0.090507	-0.30437	-0.14059
USP4	0.089618771	0.02642528	-0.29357	-0.02646	0.10755	0.015773
USP40	-0.004571281	-0.027651001	0.069076	-0.07594	0.209688	0.000137
USP42	-0.008953933	-0.00685608	0.073845	-0.01911	0.064494	-0.0692
USP43	-0.019478266	0.035792768	-0.14143	0.048392	-0.14412	-0.1386
USP44	-0.018498251	-0.079244861	0.379571	-0.11017	0.333768	-0.28896
USP45	-0.019244722	-0.035298747	0.119256	0.083605	-0.21655	-0.23994
USP46	0.028051962	0.001290922	-0.07662	0.051965	-0.03168	0.001055
USP46-AS	-0.022650898	-0.003727193	-0.06094	0.004591	-0.01401	-0.00614
USP47	0.015484129	0.132706917	0.272753	-0.01164	-0.01289	-0.10242
USP48	-0.007595225	-0.03902453	-0.21082	0.096001	-0.13261	0.19254
USP49	0.050908989	0.05464054	-0.17008	0.096469	-0.30256	0.114889
USP5	0.007564969	-0.05450185	0.040307	0.033036	-0.17373	-0.09496
USP51	-0.027823225	-0.057523468	0.308548	-0.07398	0.219899	-0.24301
USP53	0.007054349	0.030180846	0.249274	-0.0289	0.124812	-0.37678
USP54	-0.004716013	0.017414541	0.067293	0.035899	-0.12259	-0.05785
USP7	0.11851968	0.036814746	0.099709	0.009877	-0.04484	-0.32173
USP8	0.061192832	0.014144376	-0.32593	-0.03388	0.096787	0.138561
USP9X	0.06453278	0.073305255	-0.05967	0.035297	-0.08887	-0.12685
USP9Y	0.002504648	0.024871752	0.211568	-0.04851	0.141207	-0.26893
USPL1	0.016685461	0.024749303	-0.03426	-0.0151	0.047741	-0.08153
UST	-0.017699599	0.127031352	-0.0353	-0.05147	0.156222	-0.37153
UTF1	-0.027150367	-0.078342021	-0.07269	-0.00682	0.048332	0.27335
UTP11L	0.013004724	0.095203093	0.030551	0.067382	-0.20271	0.165665
UTP14A	0.007947841	0.013062786	0.058529	0.034593	-0.08774	0.074635

UTP15	-0.010774568	0.014380808	0.091329	0.065379	-0.18722	-0.09547
UTP18	0.028650702	0.049099921	0.126252	0.048858	-0.13172	0.10668
UTP20	0.005009907	0.032291961	0.19123	0.048947	-0.14474	-0.15436
UTP23	0.011227883	0.153190013	0.118805	0.026445	-0.07533	-0.12262
UTP3	0.030704111	0.064272399	0.078081	0.004915	-0.00229	0.153859
UTP6	0.037079103	0.056239065	-0.22316	0.044993	-0.10289	-0.11304
UTRN	0.014741979	0.180313789	-0.10539	-0.03238	0.088792	0.2529
UTS2	-0.005821586	0.112857699	0.126393	-0.01428	0.160438	0.007852
UTS2B	-0.017961263	-0.032467277	-0.07096	0.138549	-0.15888	-0.08555
UTS2R	0.006333337	-0.078335302	0.021357	-0.00539	-0.0842	0.004516
UTY	0.000286415	0.018515081	0.003154	-0.01875	0.076948	-0.12191
UVRAG	0.028735934	-0.044136605	-0.25514	-0.02237	0.078361	-0.01392
UVSSA	-0.040031746	-0.097470228	-0.01212	0.004147	-0.0876	-0.0702
UXS1	0.023794767	0.005039631	0.354109	-0.02344	0.03967	-0.08291
UXT	0.061101103	-0.005348365	0.313842	0.055044	-0.1399	-0.07862
VAC14	-0.001244683	-0.06307547	0.002053	0.010586	0.002959	0.004724
VAC14-AS	-0.059066753	-0.077074595	0.048479	-0.01601	0.054824	0.018533
VAMP1	0.002763733	-0.060516358	0.225237	-0.0456	0.067534	-0.46009
VAMP2	0.037517546	0.010773161	0.266763	-0.0155	-0.07909	-0.31013
VAMP3	0.031453876	0.037637439	-0.30043	-0.08533	0.243222	0.346662
VAMP4	-0.00549243	0.106846153	0.011244	0.127488	-0.16187	0.276146
VAMP5	0.010811555	-0.010120658	0.077146	-0.04097	0.102639	0.284905
VAMP7	0.093142623	0.11922996	-0.29888	-0.01083	0.042806	0.348212
VAMP8	0.025880677	0.054677913	0.019442	-0.07406	0.216289	0.176488
VANGL1	-0.01066482	0.188751898	-0.08554	0.069972	-0.13659	-0.09808
VANGL2	-0.032677044	-0.097194479	-0.04966	0.00502	0.005903	-0.20492
VAPA	-0.006489546	-0.067878999	0.020188	-0.06924	0.276125	-0.12463
VAPB	-0.001460126	0.056655212	0.20741	-0.01523	0.036019	-0.25825
VARS	0.008108193	-0.080992497	-0.0374	0.074921	-0.2355	0.168373
VARS2	0.035951385	-0.041470688	0.151989	0.060501	-0.18108	-0.09674
VASH1	-0.024326272	-0.057159054	-0.19046	0.066896	0.226256	0.039651
VASH2	-0.011118559	-0.080481471	-0.09438	0.012972	0.001527	-0.16623
VASN	-0.031489577	-0.041064305	-0.07658	0.039061	-0.11305	-0.06792
VASP	0.032396447	0.019287385	-0.29381	0.003118	0.002333	0.21125
VAT1	0.055528929	-0.023306641	0.029013	-0.05203	0.106981	-0.08139
VAT1L	-0.031688139	-0.102271138	0.06428	0.013181	-0.02922	-0.03407
VAV1	0.034398766	0.014879292	-0.09374	-0.02398	0.063446	0.055071
VAV2	0.039665267	-0.175740593	-0.16326	-0.02752	0.078158	0.163268
VAV3	0.004267824	0.129816624	-0.22192	-0.10827	0.330273	-0.03137
VAX2	-0.031750395	-0.016393056	-0.10815	0.01749	-0.03132	-0.00786
VBP1	0.092007113	0.108143278	0.093734	0.096619	-0.25877	-0.28163
VCAM1	-0.014798123	-0.007820208	0.030556	0.092972	-0.24325	-0.07716
VCAN	-3.30E-05	-0.082474834	-0.26073	-0.12139	0.329045	0.688184
VCL	0.028237238	0.08627907	-0.18097	-0.14578	0.437187	0.106565
VCP	0.075606411	0.105655676	-0.1413	0.093811	-0.24807	-0.09532
VCPIP1	0.032224683	0.13579306	-0.16175	-0.00281	0.05828	0.039956
VCPKMT	0.021708652	0.013124725	-0.10883	-0.00848	0.03328	-0.17922
VCX2	-0.033909183	-0.083527161	-0.05359	0.032742	-0.06452	0.19338
VDAC1	0.025796295	0.094351211	0.04582	0.088796	-0.2533	0.164284
VDAC2	0.077685418	0.044962143	0.047865	0.086167	-0.22771	0.085202
VDAC3	0.060327809	0.144668951	0.084732	0.069928	-0.1956	-0.04965

VDR	-0.004412544	-0.08096255	-0.1536	-0.0173	0.00235	0.250559
VEGFA	0.033571564	-0.040642722	-0.22536	0.017714	-0.05172	0.242614
VEGFB	0.003956086	-0.04368568	0.019266	0.011968	-0.02831	-0.27257
VEGFC	-0.040218312	-0.072077338	0.041401	-0.06704	0.226873	0.033701
VENTX	-0.013354906	-0.094735429	-0.20107	-0.03656	0.110979	0.561267
VENTXP1	-0.082619654	-0.106423417	0.02206	0.02011	-0.03837	0.045597
VEPH1	-0.00932995	-0.038438511	-0.03838	-0.2707	0.535441	0.065893
VEZF1	0.104526679	0.120161052	-0.06082	-0.11054	0.160497	-0.13043
VEZT	0.010755853	0.018684177	0.025784	0.050174	-0.06965	0.156817
VGF	-0.024388989	-0.0656647	0.095129	-0.00942	-0.07444	-0.03331
VGLL1	-0.029030698	-0.02786032	-0.03111	-0.03273	0.091337	0.094281
VGLL2	-0.03194539	-0.040748868	0.021248	0.00332	-0.01629	-0.04736
VGLL3	-0.060974765	-0.070617013	0.012663	0.082142	-0.06177	-0.01681
VGLL4	0.025820504	0.156683424	0.133582	0.00097	-0.01133	0.027256
VHL	-0.009994441	-0.00244358	-0.03444	-0.0066	-0.03245	0.181574
VIL1	-0.037128241	0.011342958	-0.00638	-0.19066	0.479209	-0.06511
VILL	0.011731868	-0.000491524	0.180994	-0.04861	0.159555	0.079843
VIM	0.016378433	-0.209600462	-0.14437	0.026481	-0.03426	0.396987
VIMP	0.047203121	0.089070051	-0.07632	0.025925	-0.08812	0.097626
VIP	-0.093806318	-0.077171137	-0.04801	-0.02792	0.080732	-0.00884
VIPAS39	0.011045342	-0.039325368	-0.11256	0.037089	-0.15945	0.09006
VIPR1	0.002888517	-0.16836623	0.187534	-0.09442	0.251426	0.246244
VIPR2	-0.009083298	-0.025666623	-0.04198	0.034603	-0.0374	0.02392
VIT	-0.012691857	0.247790176	0.105055	-0.02377	0.082858	-0.13822
VKORC1	0.04938602	0.049739833	-0.01535	0.038997	-0.14185	0.35396
VKORC1L1	0.006269543	0.072682825	-0.03622	0.015095	-0.02346	0.031469
VLDLR	-0.011026866	0.02996708	-0.07033	-0.00699	-0.02601	-0.14922
VMA21	-0.010677646	-0.042169232	-0.1211	0.034072	-0.05353	0.414594
VMO1	-0.021132458	-0.041042049	-0.10525	-0.02348	0.051173	0.091121
VMP1	0.00968287	-0.046786675	-0.10893	0.001266	-0.04609	0.308913
VN1R1	-0.036444339	-0.019991932	-0.00522	-0.01032	0.051558	-0.12884
VN1R10P	-0.019676707	-0.018774097	0.082527	0.001595	-0.0171	-0.07177
VN1R2	-0.032477206	-0.04305426	-0.04322	0.007857	-0.00844	-0.16759
VN1R3	-0.028213045	-0.070212046	-0.091	0.013028	-0.02364	-0.05392
VN1R4	-0.033584317	0.037109921	0.039412	0.001856	0.010031	-0.13826
VN1R5	-0.034327597	0.003206463	-0.06664	-0.0019	0.028641	-0.08958
VNN1	-0.003803534	-0.094212023	-0.30845	-0.07248	0.220583	0.588049
VNN2	0.015934538	-0.022605916	-0.18382	-0.07057	0.202186	0.332722
VNN3	-0.009926009	-0.052014557	-0.20998	-0.00229	0.00168	0.068674
VOPP1	0.065028963	0.129484039	-0.1116	0.050442	-0.11445	-0.44861
VPRBP	-0.002318523	0.072088533	0.010068	0.06604	-0.09415	-0.05453
VPREB1	-0.049238396	-0.016764784	-0.10885	-0.01166	0.055145	0.024069
VPREB3	-0.00588716	-0.070076497	-0.08675	-0.05264	0.140664	-0.12656
VPS11	0.045546815	-0.023821487	0.004228	-0.03716	0.075353	0.035084
VPS13A	0.014107245	0.102077795	0.243309	0.009728	-0.02874	-0.43611
VPS13A-AS	-0.022635113	-0.059900676	-0.00948	0.018945	-0.10425	-0.05173
VPS13B	0.039145525	0.094494016	0.023402	-0.00044	0.135485	-0.12079
VPS13C	0.021820784	0.067935634	0.044689	-0.0225	0.081947	0.071538
VPS13D	0.061829982	0.091988476	0.130718	0.015356	0.12195	-0.02044
VPS16	0.031952298	0.189524677	0.027991	0.031388	-0.07773	-0.02778
VPS18	0.00906019	0.014366363	-0.19345	0.005304	-0.04606	0.122371

VPS25	0.044582867	0.007627888	-0.10454	0.057411	-0.18985	0.321087
VPS26A	0.048846584	0.262864499	-0.14303	0.018723	-0.03511	0.119485
VPS26B	0.023293655	0.086282259	0.047687	-0.00607	0.007137	0.105284
VPS28	0.062277695	0.001857953	-0.02102	0.012716	-0.01011	0.070073
VPS29	0.067882904	0.006795939	-0.06521	0.018298	-0.04571	0.208677
VPS33A	0.014586934	-0.057491526	-0.00156	0.043463	-0.15928	0.063306
VPS33B	0.04281974	-0.05194971	0.135246	0.039929	-0.13524	0.058152
VPS35	0.041999028	-0.031875838	-0.24189	0.042469	-0.04063	0.180964
VPS36	0.021227765	0.036650335	0.038417	-0.02923	0.102976	0.023948
VPS37A	0.016727404	0.126052246	-0.12729	0.012409	-0.05463	0.164016
VPS37B	0.016419709	0.171078982	-0.05541	0.006127	-0.02761	0.010514
VPS37C	0.024690263	-0.077602479	-0.33751	-0.01877	0.055953	0.46991
VPS37D	-0.021076303	-0.076866138	0.038504	-0.00898	-0.05211	-0.08087
VPS39	0.04973373	-0.011147572	-0.09171	-0.01115	-0.00388	-0.03968
VPS41	0.009666586	0.020957074	-0.17068	-0.02638	0.094008	0.265368
VPS45	0.008333602	0.023026576	-0.00475	0.015997	-0.0231	0.195982
VPS4A	0.074376524	0.045889115	0.066422	0.084775	-0.27096	0.043864
VPS4B	0.091470834	0.273602508	-0.19779	-0.01461	0.076419	-0.21012
VPS51	0.046540387	0.016913863	0.267744	0.011622	-0.03247	-0.1514
VPS52	0.077032868	0.010650717	0.192756	0.004263	-0.02423	-0.18916
VPS53	-0.036328745	-0.021335488	-0.06205	-0.05857	0.085221	0.262197
VPS54	0.036813504	0.02855437	-0.10974	0.040509	-0.10458	0.374787
VPS72	0.028585966	0.092909238	0.014281	0.039637	-0.19535	-0.02006
VPS8	-0.055018944	-0.061226033	-0.15329	-0.06423	-0.03668	0.149365
VPS9D1	0.004027746	-0.013594342	-0.05252	-0.03381	0.099426	0.156161
VPS9D1-AS	-0.008383203	-0.08445994	0.070744	-0.00313	-0.06629	-0.08369
VRK1	0.030797882	0.0640184	0.152154	0.112448	-0.31809	-0.29329
VRK2	0.033334006	0.010715586	-0.27353	0.028321	-0.0857	0.137268
VRK3	-0.006219664	-0.007351431	-0.04899	-0.00659	-0.03278	-0.08389
VRTN	-0.016802477	-0.096363747	-0.01618	-0.00534	-0.06281	-0.06755
VSIG1	-0.008839806	-0.091980526	0.344464	-0.06168	0.184567	-0.23635
VSIG10	-0.034932857	-0.078961755	-0.13728	0.019054	-0.0389	-0.02384
VSIG10L	-0.020406077	-0.010574694	-0.06877	0.085995	-0.23115	0.204098
VSIG2	-0.026907209	-0.122640325	-0.01057	-0.02448	0.008661	-0.0257
VSIG4	-0.002648343	-0.095926802	-0.11774	-0.07988	0.231331	0.490958
VSNL1	-0.05124822	-0.066974722	0.008008	0.006296	-0.05009	-0.084
VSTM1	-0.005626543	-0.108365254	-0.18869	-0.05999	0.169423	0.462747
VSTM2A	-0.108461032	-0.019349523	0.10018	0.007209	-0.0148	-0.02094
VSTM2L	-0.031571687	-0.041083382	-0.0105	-0.00534	-0.01264	-0.09605
VSTM4	-0.030483485	-0.067719548	-0.04972	0.092244	-0.03962	-0.00011
VSX1	-0.058474033	-0.020929484	-0.03943	0.00088	-0.00523	-0.03544
VTA1	0.039810064	0.082335159	-0.08447	0.019366	-0.0694	0.035732
VTCN1	-0.035914735	-0.075334801	0.056423	0.02063	-0.04208	0.051496
VTI1A	-0.083325626	-0.104536843	-0.11881	0.035848	-0.07676	0.128759
VTI1B	-0.009925199	0.03479425	-0.09475	-0.08958	0.179596	0.498178
VWA1	-0.010139242	0.016298934	0.009393	0.084985	-0.06636	-0.06842
VWA3A	-0.019562857	-0.068640098	0.011699	0.002926	-0.07922	-0.10557
VWA3B	-0.05954558	-0.017217507	-0.09047	0.004078	0.051435	-0.07071
VWA5A	0.010990643	0.067102248	-0.18645	0.011995	-0.02275	0.210172
VWA5B1	-0.037748464	-0.07793292	-0.15296	0.008786	0.008502	0.17128
VWA5B2	-0.038040493	-0.082458373	-0.00335	0.030962	-0.10027	-0.05249

VWA7	-0.034338252	-0.061430424	-0.00739	0.004861	-0.00663	0.079072
VWA8	0.008927762	0.032236405	0.162844	0.008777	0.013762	-0.05756
VWA8-AS1	-0.10098647	-0.082042111	-0.01368	-0.02695	0.046698	-0.072
VWA9	0.001181702	0.030731957	0.26602	0.080173	-0.22672	-0.21012
VWC2	-0.108295239	-0.018695784	0.039677	-0.00489	0.084744	-0.01869
VWCE	-0.022800095	-0.066172536	-0.02632	0.0206	-0.09438	-0.01458
VWDE	-0.068695314	-0.104199004	0.182369	-0.04232	0.099602	-0.09955
VWF	-0.038211636	-0.111564204	-0.14856	-0.10249	0.274296	0.165986
WAC	0.063464231	0.142617508	-0.01058	-0.00675	0.043654	-0.24378
WAC-AS1	0.074553295	0.132683365	0.071932	0.015603	-0.05403	0.069269
WAPAL	0.066731319	0.110038878	-0.13403	0.03892	-0.0876	-0.03379
WARS	0.029790676	-0.058806603	-0.39264	0.021445	-0.04325	0.419788
WARS2	0.020629716	0.01778122	-0.02978	-0.06179	-0.09283	0.203489
WAS	0.047090931	-0.031088393	-0.05003	0.029875	-0.03595	0.090374
WASF1	-0.01051324	-0.077027868	-0.05968	-0.02221	0.072874	-0.05591
WASF2	0.087815921	-0.014055939	-0.25768	-0.05304	0.192083	0.182601
WASF3	-0.018964476	-0.037309563	-0.00708	-0.12131	0.314692	-0.06038
WASL	-0.042528755	0.018768728	0.016736	0.023801	-0.09258	0.286621
WBP11	0.038831752	0.032095133	0.179788	0.021565	-0.06627	-0.05583
WBP1L	0.058215039	0.123597319	-0.1896	-0.04305	0.137111	0.130138
WBP2	0.021952434	-0.051905436	-0.17261	-0.02726	0.074733	0.045399
WBP2NL	-0.025125371	0.013455814	-0.01527	-0.00852	0.022532	-0.01091
WBP4	0.02275459	-0.004120818	-0.092	0.034679	-0.14038	0.228419
WBP5	-0.00241334	-0.081972493	0.112632	-0.12882	0.379136	0.043617
WBSCR16	-0.013637755	0.01792082	0.137695	0.032182	-0.09861	-0.11141
WBSCR17	-0.037524284	0.005727303	-0.09142	0.009915	-0.06337	-0.01915
WBSCR22	0.023465924	0.011747702	0.174049	0.057203	-0.15431	-0.08963
WBSCR27	-0.040936319	-0.051415009	0.032911	0.000487	-0.01446	0.108404
WBSCR28	-0.03834199	-0.065134627	-0.02625	0.013217	-0.03124	0.0014
WDFY1	0.036293491	0.156874728	-0.15586	-0.02223	0.052787	0.315072
WDFY2	0.023197279	-0.06281581	-0.20915	-0.04783	0.140598	0.167292
WDFY3	-0.019425943	-0.088711762	-0.26715	-0.04563	0.080417	0.421904
WDFY3-AS2	-0.027890684	-0.090349949	-0.12767	-0.07209	0.031454	0.299128
WDFY4	0.00353927	-0.181465046	-0.34338	-0.05968	0.156245	-0.02884
WDHD1	-0.024830701	0.063182122	0.006646	0.217873	-0.29539	-0.08303
WDPCP	0.022728456	-0.019990176	0.206877	-0.05673	0.136119	-0.16184
WDR1	-0.013049984	0.027820834	-0.06426	-0.03355	-0.02572	0.039464
WDR11	0.029764922	0.048179246	-0.12567	-0.03804	0.122339	0.186059
WDR11-AS1	-0.033774036	-0.047099204	-0.0309	-0.1993	0.570666	0.085455
WDR12	-0.015562993	0.010465628	0.086017	0.041492	-0.0816	0.016935
WDR13	0.01739939	-0.049597763	-0.19217	-0.007	0.02575	0.405202
WDR16	-0.048463542	0.009471057	-0.02506	0.066434	-0.18473	-0.01477
WDR17	-0.085041328	-0.132174719	-0.06146	-0.05238	0.182454	0.224821
WDR18	0.008982715	-0.029791773	-0.03899	0.100308	-0.30378	0.145748
WDR19	-0.037376016	-0.035604898	0.122021	-0.05526	0.093223	-0.09394
WDR20	0.073668982	0.062879618	-0.04578	0.078131	-0.03674	0.040876
WDR24	-0.012546221	-0.080886448	0.018404	0.040664	-0.10516	0.20373
WDR25	-0.013561296	-0.002102377	-0.13212	0.066482	-0.14579	0.283102
WDR26	0.059975864	0.097558289	-0.19332	0.010575	-0.04459	0.054432
WDR27	-0.027953328	-0.065251398	0.104261	-0.00305	0.000337	-0.30909
WDR3	0.003950443	0.0084587	0.149616	0.0437	-0.1461	-0.03837

WDR31	-0.044635212	-0.097827896	-0.06881	-0.01159	-0.03467	-0.08526
WDR34	0.003609008	-0.015352094	-0.06749	0.122291	-0.38329	-0.15951
WDR35	-0.013067787	0.107587196	0.048517	-0.04748	0.140002	-0.35666
WDR36	0.011086624	0.049438004	0.102656	0.061009	-0.14987	0.026458
WDR37	-0.055843264	-0.002090645	0.037064	-0.07043	-0.00912	-0.10337
WDR38	-0.041966154	-0.078514882	-0.05741	0.021844	-0.05981	0.042914
WDR4	-0.012224207	0.011629216	-0.0114	0.037698	-0.11388	-0.0834
WDR41	0.024565114	0.022849298	-0.37988	0.083612	-0.08253	0.25646
WDR43	0.023322946	-0.023180709	0.125152	0.069374	-0.17065	0.024024
WDR44	0.01517108	0.038674898	-0.03401	0.006828	-0.01696	-0.10753
WDR45	0.04687512	0.100647108	-0.16877	-0.0557	0.144719	0.202003
WDR45B	0.096885268	0.087298007	0.0097	0.061225	-0.14185	0.200749
WDR46	0.019848516	0.036683331	0.063879	0.032596	-0.0984	0.06469
WDR47	0.03086736	0.101044365	-0.25049	0.028921	-0.06386	0.089085
WDR48	0.014754137	0.066878745	0.006389	0.007237	-0.07948	-0.38685
WDR49	-0.055230388	-0.087147022	-0.20163	-0.03835	0.166755	0.406974
WDR5	0.006319471	0.012598184	0.047032	0.046039	-0.1651	0.147434
WDR52	-0.013284531	-0.033748216	0.120974	-0.00986	0.038262	-0.19353
WDR53	0.002518193	-0.002384149	-0.06542	0.036786	-0.10387	-0.0547
WDR54	0.014694638	0.042666775	0.22653	0.073651	-0.20993	-0.24894
WDR55	0.034298496	-0.036136145	0.173694	0.001755	-0.08312	-0.15207
WDR59	-0.064156826	0.04478456	0.310531	-0.07003	0.147725	-0.17572
WDR5B	-0.005055008	0.105791254	0.031932	-0.01459	0.065003	-0.13024
WDR6	0.027123369	-0.053444431	0.268959	0.000301	-0.00113	-0.32004
WDR60	-0.02311496	0.049862177	0.13479	0.087835	-0.05888	-0.13595
WDR61	0.0157553	-0.015438081	0.153651	0.060709	-0.09209	0.267964
WDR62	-0.03837343	0.029480185	-0.03537	0.043542	-0.15231	-0.00765
WDR63	-0.027703489	-0.025655235	0.044802	0.113671	-0.21664	-0.02745
WDR64	-0.019388332	-0.063495872	0.014153	0.011424	-0.01868	-0.13158
WDR65	-0.019949581	-0.016408555	0.112436	0.11547	-0.30793	-0.02011
WDR66	-0.013558303	-0.10425813	-0.08425	0.044457	0.005945	-0.12236
WDR7	0.020296787	0.100558249	-0.00495	0.012387	-0.02221	0.19383
WDR70	0.016776414	-0.053447745	8.15E-05	0.023024	-0.07396	0.091056
WDR72	-0.044033267	0.005903652	0.061413	-0.03902	0.053017	-0.11381
WDR73	0.034325425	-0.051780475	0.25197	-0.03406	0.044044	-0.08693
WDR74	0.042222798	0.022887643	0.11019	0.078138	-0.12414	-0.04133
WDR75	0.021317302	-0.008766999	0.15181	0.057906	-0.13661	-0.00686
WDR76	-0.014732847	0.064027892	0.057121	0.116221	-0.27847	-0.15258
WDR77	0.030443965	-0.015064433	0.066327	0.038041	-0.15135	-0.0855
WDR78	-0.064348121	-0.029624727	0.034029	0.062636	0.022779	-0.05771
WDR81	0.052803956	-0.03900155	0.052163	-0.03518	0.060334	-0.04103
WDR82	0.104981672	0.217957799	0.363431	0.029691	-0.02896	-0.40057
WDR83	0.002265933	0.029764798	-0.13056	0.03714	-0.22172	-0.00196
WDR83OS	0.084354555	0.07249514	-0.01579	0.061519	-0.14696	0.311037
WDR86	-0.048011847	-0.038958928	0.122891	-0.00167	0.02878	-0.0679
WDR86-AS	-0.029868018	-0.058073748	0.228184	-0.03271	0.099391	-0.1966
WDR87	-0.029452315	-0.051600762	-0.06276	0.033715	-0.09532	-0.00643
WDR88	-0.053574545	-0.066007127	-0.05774	-0.00439	0.046007	0.063792
WDR89	-0.004197564	0.082190384	0.279427	0.040854	-0.10663	-0.18501
WDR90	0.040109733	-0.072305759	0.211404	-0.03762	-0.2575	0.009421
WDR91	-0.049309922	-0.066344685	-0.12909	-0.04498	0.112047	0.212403

WDR92	0.01374214	0.043043782	0.070358	0.010114	-0.05646	-0.05292
WDR93	-0.027522416	-0.019856103	0.002319	-0.0003	-0.00884	-0.01013
WDR96	-0.072780102	0.00175131	-0.13647	0.032426	0.053795	0.113699
WDSUB1	0.020529157	0.123534372	-0.0775	-0.04895	0.15465	0.145889
WDTC1	-0.013025473	-0.074152727	0.119035	-0.0343	-0.00374	-0.1837
WDYHV1	0.007201759	0.078536221	0.067618	0.076466	-0.20532	0.139625
WEE1	-0.000860606	0.074785615	-0.00612	0.073795	-0.25655	-0.36272
WEE2-AS1	-0.033320105	-0.024074192	0.149547	0.03184	-0.06612	-0.02443
WFDC1	-0.030826353	-0.038204012	0.004648	-0.00925	0.020186	-0.06257
WFDC10A	-0.028411033	0.01193538	0.027032	0.029869	-0.06102	-0.03069
WFDC10B	-0.038732197	-0.052969662	-0.00925	0.03664	-0.07944	0.02808
WFDC11	-0.047321703	-0.007925296	-0.14543	0.069139	-0.17148	-0.06371
WFDC12	-0.023212931	-0.039245653	0.022091	-0.01132	-0.07023	-0.14346
WFDC13	-0.05552388	0.011654339	-0.11729	-0.01276	0.060156	0.092377
WFDC2	-0.052394353	0.028267723	0.060069	0.015637	-0.07483	0.050355
WFDC21P	0.003780667	-0.084447803	-0.13327	-0.01431	-0.01151	-0.26704
WFDC3	-0.031215299	-0.049653589	-0.12334	-0.00733	-0.02822	0.091628
WFDC5	-0.044387496	-0.037436214	-0.0339	0.009358	-0.00119	-0.01106
WFDC6	-0.031643954	-0.07072907	-0.1525	-0.01404	0.046578	-0.03206
WFDC8	-0.039099427	-0.0834209	-0.08362	0.051834	-0.05847	0.128068
WFDC9	-0.039024744	-0.03724234	-0.00139	0.022197	-0.04749	-0.12856
WFIKKN1	-0.029110596	-0.091895372	-0.07244	-0.01996	-0.00349	0.095932
WFIKKN2	-0.040910895	-0.010963532	-0.0513	0.002501	0.012886	0.035566
WFS1	-0.020929734	0.006079575	0.021527	0.132946	-0.42317	-0.13051
WHAMM	0.034666619	0.085941744	0.272023	-0.02826	0.078468	-0.16528
WHAMMP2	0.000547892	-0.014685213	0.077046	-0.08753	0.166367	-0.13334
WHSC1	-0.03392493	-0.013756753	0.03813	0.183452	-0.4158	-0.12751
WHSC1L1	0.002903446	-0.025892555	-0.08052	0.053303	-0.19743	-0.11811
WI2-8903	-0.040198785	-0.007703456	9.43E-05	0.016924	-0.02463	-0.08252
WIBG	0.000224819	-0.037783636	0.101567	0.062926	-0.2307	-0.02303
WIF1	-0.041994671	-0.031685354	-0.09989	0.003808	-0.00385	0.029354
WIPF1	0.020224891	0.162375178	0.024243	0.041533	-0.05443	-0.22328
WIPF2	0.041912023	0.129840873	-0.21505	0.003729	-0.00842	-0.07744
WIPF3	-0.04612634	-0.02850408	0.075945	0.005066	-0.08828	-0.07573
WIPI1	0.012184942	0.124131754	-0.24106	0.004427	-0.03003	0.136214
WIPI2	0.033812284	-0.000962351	-0.13981	-0.05465	0.127115	0.0691
WISP1	-0.056757768	0.003088947	-0.08521	0.024681	-0.03369	-0.01668
WISP2	-0.031592033	-0.031793567	-0.03121	-0.0161	0.039368	0.055914
WISP3	-0.036919853	0.019487596	-0.02174	0.022475	-0.05205	0.012876
WIZ	0.002468091	-0.084869648	0.151356	0.021504	-0.09434	-0.03849
WLS	-0.006809864	-0.077683842	-0.26828	-0.05475	0.150805	0.536174
WNK1	-0.007375422	-0.002765297	0.167886	-0.00736	-0.01368	-0.043
WNK2	-0.039619039	-0.073753611	-0.02471	0.003052	-0.09288	-0.09722
WNK3	-0.058838427	-0.052851335	0.019697	0.025283	-0.08423	-0.08857
WNK4	-0.052035952	-0.035939933	-0.16962	0.028336	-0.06335	-0.08923
WNT1	-0.024185933	-0.058461961	0.059979	0.003936	0.003302	0.020927
WNT10A	-0.014692581	-0.120197796	-0.08513	0.01623	-0.02405	-0.16899
WNT10B	-0.004186918	-0.007046818	0.327102	-0.03518	0.040474	-0.20165
WNT11	-0.02930139	-0.00230705	-0.09176	0.02328	-0.05178	0.0824
WNT16	-0.042319196	-0.094636629	-0.08722	-0.02436	0.042416	-0.13147
WNT2	-0.031373984	-0.011276091	0.049554	0.013755	-0.04046	0.037941

WNT2B	-0.006605511	-0.051728074	0.014541	0.000437	0.041706	-0.21728
WNT4	-0.022556016	0.001406785	-0.11863	0.03066	-0.01451	-0.0209
WNT5A	-0.02516864	-0.031792142	0.024598	0.013767	-0.06792	-0.06093
WNT5B	-0.055306691	-0.049043167	-0.14574	-0.07111	0.123304	0.17781
WNT6	-0.056608269	-0.049301133	-0.09375	0.049108	-0.06206	0.173385
WNT7A	-0.03294321	-0.158445253	0.03042	-0.00944	0.035455	-0.1499
WNT7B	-0.026963409	-0.085102362	-0.07407	-0.0214	-0.02326	-0.071
WNT8A	-0.038596856	-0.070240263	0.056382	0.014505	-0.01288	0.005569
WNT8B	-0.030449089	-0.031165036	0.089739	0.008114	-0.01121	-0.0268
WNT9A	-0.04016801	-0.063485775	-0.0664	0.053193	-0.13357	0.064121
WNT9B	-0.031327492	-0.066080139	-0.05129	-0.02026	0.065848	-0.11714
WRAP53	0.020164571	-0.040670423	0.065378	0.133478	-0.47384	-0.18026
WRAP73	-0.003980976	0.054811592	0.066678	0.048776	-0.11159	0.10805
WRB	0.028081287	0.036871649	-0.06306	0.008513	-0.03809	0.084096
WRN	0.011379379	0.039489353	0.033916	0.104008	-0.25392	-0.09146
WRNIP1	-0.026056973	-0.088002054	0.029156	0.009475	-0.09361	-0.07039
WSB1	-0.004497534	0.006962043	-0.13427	-0.02382	0.029378	0.126946
WSB2	0.046229521	0.065637314	-0.13818	0.028889	-0.06979	0.265248
WSCD2	-0.032751942	-0.053609364	0.017747	-0.00037	-0.05424	-0.18749
WT1	-0.050337457	-0.118476693	0.005532	-0.01092	0.051835	-0.13175
WT1-AS	-0.028406409	-0.032813556	0.000112	-0.01927	0.052374	0.055616
WTAP	-0.048359793	0.104735092	0.058476	0.010204	-0.02064	-0.10757
WTIP	-0.031063415	-0.084821399	0.012917	0.019901	-0.0894	-0.00942
WWC1	-0.095897234	-0.054666854	0.15195	-0.07304	0.011952	-0.06138
WWC2	-0.038953292	-0.000225951	0.067899	0.000739	0.018348	-0.05505
WWC2-AS2	-0.032690055	0.038245773	0.001591	0.041094	-0.11065	0.081196
WWC3	0.019672075	0.038126749	-0.01099	0.024514	-0.08073	-0.07306
WWOX	-0.063115971	-0.040469469	0.141484	0.084164	-0.15837	0.06856
WWP1	0.029146437	0.071961391	0.343246	-0.01932	0.058463	-0.06641
WWP2	-0.003120079	-0.039017056	-0.00562	-0.02999	-0.07912	-0.07225
WWTR1	-0.007264324	-0.023419053	0.008481	0.025564	-0.02581	-0.04601
WWTR1-AS	-0.05042553	-0.071667372	-0.03988	0.0377	-0.0669	-0.02523
XAB2	0.006621384	-0.049957388	0.064597	0.042694	-0.07464	0.020303
XAF1	0.018450418	0.099947274	-0.01732	-0.09506	0.225696	0.095222
XAGE-4	-0.015089663	-0.027513049	0.026805	0.008116	-0.11776	-0.11357
XAGE2	-0.016967157	-0.092978606	-0.0074	-0.01304	-0.01462	-0.05493
XAGE3	-0.032227899	-0.012516926	-0.08604	0.044018	-0.11215	-0.14145
XBP1	0.020154725	0.201032291	0.178872	0.010083	-0.02572	-0.02161
XCL1	-0.005813468	0.182370197	0.145577	0.007381	-0.06124	-0.15629
XCR1	-0.019659009	-0.074147293	-0.05844	-0.01315	0.048339	-0.10931
XDH	-0.0302905	-0.026015147	0.024612	0.096392	-0.26253	0.028696
XG	-0.07707251	-0.129192385	0.030915	-0.00188	0.04275	0.127917
XIAP	0.039994351	0.139510584	0.016094	-0.04978	0.095573	-0.12672
XIRP1	-0.007560816	-0.014883299	-0.01799	-0.00474	-0.02737	-0.03898
XIRP2	-0.042429259	-0.044707179	-0.02642	-0.0152	0.05068	-0.06156
XIST	0.014933788	0.056521384	-0.07443	0.009527	0.049668	0.035253
XK	-0.013397888	-0.039409762	-0.11561	-0.16155	0.436979	-0.11094
XKR4	-0.050887407	0.00371196	0.029384	-0.01421	0.04072	0.065301
XKR6	-0.053555857	0.016676439	0.212022	-0.06367	0.161977	-0.30525
XKR7	-0.029751432	-0.034003921	-0.07112	0.002007	0.003634	-0.09232
XKR8	0.051426462	-0.018191263	-0.10733	0.003028	0.006961	-0.19026

XKRX	-0.037831039	-0.041499494	-0.03692	-0.0221	0.097013	0.023794
XPA	0.039603366	0.0552527	0.101741	-0.00548	0.006453	-0.00262
XPC	0.046114978	0.011960379	0.12628	0.027999	-0.07832	-0.23193
XPNPEP1	0.022382285	0.093957569	-0.10755	-0.00804	-0.01108	0.111126
XPNPEP2	-0.023251008	0.141789392	0.095758	-0.02012	0.072806	-0.03378
XPNPEP3	-0.024711136	0.008175399	0.11777	0.073874	-0.06275	0.058178
XPO1	0.040664088	0.087343981	0.110106	0.054943	-0.16043	-0.18103
XPO4	0.010983895	-0.000354946	0.151574	0.052919	-0.12759	0.006377
XPO5	-0.050284856	-0.028803154	0.033557	0.087582	-0.34926	0.015692
XPO6	0.045064657	-0.024640394	0.019187	0.022706	-0.05335	-0.26586
XPO7	-0.025146388	-0.062259702	-0.07529	0.004725	-0.03217	0.008941
XPOT	0.035528393	0.047595833	0.241138	0.071728	-0.2147	-0.02544
XPR1	-0.006597336	0.048398164	-0.13984	0.055096	-0.0844	0.295869
XRCC1	0.032628575	-0.03609524	0.036313	0.032921	-0.11858	0.080934
XRCC2	0.019010203	-0.02756356	0.105754	0.019839	-0.10615	-0.19636
XRCC3	-0.022587282	-0.021181915	-0.11072	0.112473	-0.33975	-0.01622
XRCC4	-0.021013109	0.033537915	-0.26536	0.025618	-0.03867	0.507086
XRCC5	0.106404709	0.130283941	0.088236	0.079559	-0.17498	-0.37788
XRCC6	0.041890055	0.034287569	0.145504	0.089412	-0.21899	-0.08966
XRCC6BP1	-0.002961186	-0.018361193	-0.00475	0.038272	-0.0862	0.197098
XRN1	0.013612329	0.120947967	-0.03658	0.023939	-0.07577	-0.1925
XRN2	0.041395538	0.013308593	-0.18837	0.019884	-0.04415	0.140206
XRRA1	-0.029293584	-0.035747837	0.030231	0.026195	-0.0635	-0.11958
XXbac-B47	-0.028570788	-0.030916797	0.034084	0.044648	-0.10851	0.137218
XXyac-YX	-0.031374798	0.006087003	0.091911	0.01469	-0.0279	-0.14833
XXYLT1	0.011023589	-0.016574014	-0.02797	0.007757	0.015046	0.269437
XYLB	0.015146624	-0.037602585	-0.00185	0.036524	-0.12779	0.056919
XYLT1	0.012352376	0.081012773	-0.17801	0.010075	-0.03549	0.122618
XYLT2	-0.015750148	-0.019770635	0.172774	0.038951	-0.09006	0.006912
Y16709	0.078927095	0.112630178	0.024245	0.053887	-0.11074	-0.1847
YAE1D1	0.014355798	0.053633736	0.217879	0.009357	0.01007	-0.09783
YAF2	-0.006856107	0.107073277	0.010962	0.020028	-0.05872	-0.04912
YAP1	-0.068493563	-0.033790351	0.014508	0.053745	-0.11898	-0.04565
YARS	0.035207095	0.194054424	0.105991	0.067824	-0.21039	-0.05288
YARS2	0.031816334	0.016064215	0.095443	0.029343	-0.13871	-0.02656
YBEY	0.006150003	-0.021005613	0.132921	-0.0245	0.021532	0.030989
YBX1	0.042511156	0.064000416	0.068648	0.072258	-0.14833	0.060674
YBX2	-0.023993651	-0.033543226	0.001475	-0.02398	-0.01121	-0.09689
YBX3	0.019301011	-0.182600949	0.04265	0.013922	-0.00421	0.326636
YDJC	0.022452395	-0.007917436	0.078439	0.049033	-0.13671	0.037825
YEATS2	0.033514498	-0.076150106	-0.01634	0.028361	-0.14429	0.112019
YEATS4	0.017797847	0.044049107	0.137638	0.06891	-0.17002	-0.08331
YES1	-0.002724352	0.291626895	0.134419	0.058226	-0.15219	-0.23524
YIF1A	0.020501745	-0.007686877	-0.02859	0.068373	-0.19798	0.27685
YIF1B	-0.019821011	-0.068661991	-0.08757	-0.02333	-0.09945	0.291415
YIPF1	0.032054246	0.000390008	-0.27876	0.018825	-0.05322	0.157365
YIPF2	0.012061631	-0.027801741	0.001906	0.027413	-0.15125	-0.012
YIPF3	0.032406098	-0.034255961	-0.28219	0.020688	-0.04033	0.306495
YIPF4	0.010508098	-0.01570788	-0.08087	-0.05217	0.078384	0.106519
YIPF5	-0.003665113	0.129488931	0.101586	-0.01674	0.013357	0.012501
YIPF6	-0.027448187	0.023284168	-0.08534	0.030313	-0.11136	0.030355

YJEFN3	-0.026434364	-0.051298162	0.054391	0.048715	-0.11596	-0.0419
YKT6	0.018042825	0.01585256	-0.10819	0.046632	-0.15092	0.130028
YLPM1	0.008124886	0.04071304	0.061582	0.029386	-0.15813	-0.05828
YME1L1	0.062279402	0.056173774	0.358897	0.175689	0.003994	-0.3678
YOD1	-0.006641387	0.018778483	-0.08014	0.017332	-0.06699	0.013002
YPEL1	-0.00755896	0.261419484	0.033203	-0.0376	0.072684	-0.2789
YPEL2	-0.0062133	-0.098914207	0.040327	-0.07352	0.214265	0.3782
YPEL3	0.017285709	0.009912612	-0.08661	-0.07227	0.20457	-0.15083
YPEL4	-0.016302843	-0.043039807	0.031749	-0.00032	0.011692	0.022471
YPEL5	0.052835166	-0.042640117	-0.19216	-0.07672	0.231749	-0.15843
YRDC	0.01776211	-0.009944439	0.047459	0.081548	-0.24779	-0.115
YTHDC1	0.01296806	0.015295887	0.045945	-0.02936	0.087457	-0.1443
YTHDC2	0.008124723	0.026508803	0.12951	0.038963	-0.04138	0.04681
YTHDF1	0.100313025	0.069267247	0.044691	0.066611	-0.16162	-0.07239
YTHDF2	0.096461152	0.149757588	0.123633	0.054615	-0.13714	-0.08095
YTHDF3	0.034439329	0.04742829	-0.21326	0.015037	-0.03542	0.135015
YTHDF3-AS	-0.023991239	-0.069022803	-0.23862	0.048853	-0.137	0.095347
YWHAB	0.178699466	0.1485981	0.075284	-0.02373	-0.03667	0.187461
YWHAE	0.022010496	-0.046813733	-0.16951	0.073362	-0.21998	0.354491
YWHAEP7	-0.029440115	-0.03547052	-0.03774	0.004586	-0.07355	-0.17655
YWHAG	0.0549753	0.014436491	-0.21887	0.071001	-0.18578	0.363705
YWHAH	-0.004761325	0.002870035	-0.04142	0.017876	-0.05038	0.016772
YWHAQ	0.086118855	0.196510685	0.231384	0.059435	-0.19793	-0.17393
YWHAZ	0.053193751	0.344700395	-0.00096	0.116868	-0.18697	-0.48028
YY1	0.062880018	0.065984852	0.178486	-0.0219	0.061878	-0.17656
YY1AP1	0.095767092	0.046142509	-0.16621	0.060616	-0.14271	-0.16217
ZACN	-0.024514885	0.020462535	-0.10284	-0.01775	0.007849	0.030981
ZADH2	-0.001338714	0.013338861	0.007677	0.003947	0.014209	0.173822
ZAK	-0.013389747	-0.092751527	-0.22424	0.03616	-0.03447	0.468153
ZAN	-0.039456999	-0.057355256	0.063129	0.029311	-0.01714	-0.14743
ZAP70	0.010820922	0.241936972	0.440164	-0.0089	0.014097	-0.29094
ZAR1	-0.06005685	-0.071057973	-0.06914	0.006689	0.015964	0.093508
ZBBX	-0.064416418	-0.115844104	0.007208	0.003261	-0.01066	-0.1347
ZBED1	0.03225109	0.009101918	-0.35134	-0.05339	0.153073	0.280419
ZBED2	-0.020700905	-0.020426066	-0.03886	0.046003	-0.15856	-0.05419
ZBED3	0.003003681	-0.073968887	0.077496	-0.09274	0.287813	0.180578
ZBED3-AS	-0.028897294	-0.086901052	-0.0571	-0.02435	0.044112	-0.05569
ZBED4	-0.015362793	0.037958778	0.090364	0.009952	-0.0736	-0.08555
ZBED5	0.033996662	-0.043488488	0.164807	-0.01358	0.05853	-0.13546
ZBED5-AS	0.010290885	-0.172662872	0.082396	-0.06922	0.148983	0.078264
ZBED6	0.001227269	0.031738471	-0.07428	0.04819	-0.0979	0.116792
ZBED6CL	0.014137722	0.02812478	0.222887	0.064748	-0.16525	-0.31204
ZBED8	-0.025477053	-0.007644303	0.007546	0.000689	-0.00038	-0.01062
ZBED9	-0.062447774	-0.04408691	0.160343	-0.02315	0.076805	-0.04709
ZBP1	0.00236875	0.129675546	0.048693	-0.04452	0.115353	-0.17134
ZBTB1	0.032709043	0.117919391	0.092319	0.021391	-0.02015	-0.09369
ZBTB10	0.022979292	0.047440663	0.047813	-0.06276	0.091904	-0.08174
ZBTB11	0.023006151	0.087914231	0.057952	-0.00195	0.017297	0.05744
ZBTB11-AS	-0.04036629	-0.031840084	-0.02459	0.057052	-0.13369	0.093667
ZBTB12	-0.034572051	-0.01440612	-0.06171	0.031057	-0.05594	0.102614
ZBTB14	0.011522229	0.046220887	0.152897	-0.02412	0.08326	-0.08786

ZBTB16	0.006553285	0.221309364	-0.03442	-0.01736	0.043332	0.114275
ZBTB17	0.004745784	-0.108827965	-0.05096	-0.01664	-0.02166	0.022227
ZBTB18	0.015705915	-0.178774509	-0.06674	-0.11059	0.320834	-0.06825
ZBTB2	0.033041232	0.147142081	0.096729	0.019551	-0.04774	-0.10838
ZBTB20	-0.023279197	0.038276858	0.147577	-0.06994	0.23472	-0.19069
ZBTB20-AS	-0.028256611	0.002444943	0.123867	-0.00027	-0.02175	-0.13963
ZBTB21	0.017772574	0.028954451	0.023072	0.015596	-0.04993	0.033609
ZBTB22	0.014914241	-0.057282587	0.024131	-0.01845	-0.03126	-0.06848
ZBTB24	0.079737425	-0.029190142	0.047853	-0.00806	0.033863	0.060045
ZBTB25	0.007875359	-0.011167625	0.448197	0.007333	-0.0197	-0.2694
ZBTB26	0.005199047	0.049074363	-0.05952	0.035721	-0.05224	0.078856
ZBTB3	-0.004218712	0.085973514	0.008104	-0.04534	0.126106	0.13283
ZBTB32	-0.005096741	-0.033833522	-0.00198	0.073816	-0.22909	-0.00896
ZBTB33	0.027262274	0.084102155	0.041843	0.0023	-0.01083	-0.04809
ZBTB34	0.020179565	-0.001961312	-0.41341	-0.03164	0.108053	0.294318
ZBTB37	-0.007032667	0.133312345	0.078462	0.081871	-0.13042	-0.07841
ZBTB38	-0.006906957	0.11695476	0.359536	-0.03893	0.109193	-0.25636
ZBTB39	0.007808368	-0.000137857	0.058592	0.053841	-0.12101	0.013116
ZBTB4	0.028727329	0.127071706	0.314093	-0.0199	0.046157	-0.1889
ZBTB40	-0.027459102	-0.007706919	0.157844	-0.09034	0.13829	-0.05353
ZBTB41	0.012662975	0.099888892	0.094651	0.017711	-0.02173	-0.02449
ZBTB42	-0.016566585	-0.02836238	-0.03714	0.01992	-0.05992	-0.06698
ZBTB43	0.027615121	0.129623388	0.03667	0.036828	-0.11624	0.086881
ZBTB44	-0.041111652	0.217081105	0.167799	-0.07848	0.10575	-0.26485
ZBTB45	-0.015511761	-0.078596488	-0.0038	0.016135	-0.09021	0.045574
ZBTB46	0.004438847	-0.071496969	-0.15589	0.020304	0.004563	-0.01415
ZBTB46-AS	-0.028700767	0.016658837	-0.05981	0.008557	0.002716	0.043134
ZBTB47	0.061395103	-0.179214929	-0.19046	0.066753	0.023942	0.246303
ZBTB48	0.014488295	-0.05486407	-0.21878	-0.0094	0.018547	0.193477
ZBTB49	-0.000284354	0.085310871	-0.00614	-0.01853	0.044177	-0.09989
ZBTB5	0.02192443	0.005144389	0.147372	0.004682	-0.08374	-0.31735
ZBTB6	-0.024747565	0.113983381	0.016126	0.019522	-0.03658	-0.03782
ZBTB7A	-0.031200923	0.052038509	0.03246	-0.02792	0.065583	0.145986
ZBTB7B	0.00271902	-0.107123302	-0.03742	-0.06038	0.07782	0.068702
ZBTB7C	-0.045256194	-0.026496505	-0.03244	-0.0222	0.019351	-0.01305
ZBTB8A	-0.013392836	0.047349543	-0.04906	-0.01693	0.061749	-0.11266
ZBTB8B	-0.052251606	-0.012232544	0.027812	-0.01049	0.050126	-0.07286
ZBTB8OS	0.066094302	0.082421337	-0.02264	0.107461	-0.31792	0.286416
ZBTB9	-0.008615359	0.037786794	0.112422	0.01437	-0.11637	-0.1538
ZC2HC1A	-0.02758271	-0.117694893	-0.15623	0.094802	-0.02477	0.324142
ZC2HC1B	-0.040758445	-0.013297551	-0.0728	0.011902	-0.02409	-0.04277
ZC2HC1C	-0.020842308	-0.062549162	-0.00602	0.106505	-0.28742	0.04536
ZC3H10	-0.033090285	0.014133603	-0.13107	-0.0137	-0.09968	0.116559
ZC3H12B	-0.035190309	-0.039026396	0.16746	0.014632	-0.02955	-0.0639
ZC3H12C	-0.02387237	-0.068993057	-0.05174	0.017078	-0.12904	0.06901
ZC3H12D	0.001030959	-0.174425533	0.110592	-0.07312	0.187863	-0.09154
ZC3H13	0.046573088	0.134332961	0.092678	0.029585	-0.06989	-0.16908
ZC3H14	0.021575508	0.105587867	0.081109	0.046497	-0.10164	0.03448
ZC3H15	0.060410827	0.094962265	0.101674	0.068505	-0.14365	0.078666
ZC3H18	0.005350648	0.089074368	-0.03014	0.02997	-0.07993	0.011844
ZC3H3	-0.019505673	-0.116320458	0.010443	0.010971	-0.00829	0.118648

ZC3H4	0.044991816	0.06616677	0.008588	0.068496	-0.17797	-0.14914
ZC3H6	1.27E-05	0.014441397	0.182533	-0.0052	0.026752	-0.12129
ZC3H7A	0.057743071	-0.010691195	-0.09913	-0.01008	0.030044	0.192283
ZC3H7B	-0.000229692	0.000169551	0.110515	0.062424	-0.18313	-0.09913
ZC3H8	0.00967842	0.072159556	0.220518	0.018197	-0.04376	-0.12982
ZC3HAV1	0.065358681	0.108752149	0.339832	0.014234	-0.02111	-0.45855
ZC3HAV1L	-0.01635269	-0.025799669	0.055487	-0.0359	0.054666	-0.10849
ZC3HC1	0.02848025	0.023577637	0.051585	0.077	-0.21114	0.03106
ZC4H2	-0.012965619	-0.060885307	0.184356	-0.0457	0.099909	-0.13115
ZCCHC10	0.007702256	0.048916199	-0.1206	0.013414	-0.0696	0.111077
ZCCHC11	0.003817176	0.022446112	0.187157	0.002611	-0.00513	-0.4036
ZCCHC12	-0.037343513	-0.084908353	0.235488	-0.0189	0.083814	-0.02988
ZCCHC13	-0.037734371	-0.057586436	-0.10041	0.029746	-0.05488	0.104997
ZCCHC14	-0.021196224	-0.013008975	0.084105	-0.02561	0.096726	0.065026
ZCCHC17	0.049884274	0.234299757	0.035309	0.002439	-0.05174	-0.04874
ZCCHC18	-0.003493915	0.046704213	0.198617	-0.0022	0.007417	-0.299
ZCCHC2	0.052566548	0.075938805	0.01105	-0.01693	0.001579	-0.02826
ZCCHC24	0.005092206	0.006277879	-0.31829	-0.00995	0.051566	0.391599
ZCCHC3	-0.063830299	0.00964024	-0.00537	-0.03371	0.042277	0.266567
ZCCHC4	-0.050300247	0.019948948	0.0957	0.044565	-0.1784	-0.05078
ZCCHC5	-0.031841662	0.013319849	-0.13179	0.00222	-0.04707	-0.03651
ZCCHC6	0.00957122	0.068666219	-0.28227	0.010369	0.019812	0.179307
ZCCHC7	0.007551313	0.010199991	0.063472	-0.03923	0.010758	-0.28548
ZCCHC8	0.042969464	0.088352354	-0.05191	0.0228	-0.04901	0.033967
ZCCHC9	0.036911339	-0.013151758	0.023842	0.053953	-0.18189	0.070389
ZCRB1	0.048728165	0.040913547	0.136723	0.070786	-0.21745	-0.06601
ZCWPW1	-0.013003573	-0.116049909	0.021646	-0.01763	-0.04051	-0.32911
ZCWPW2	-0.058791232	-0.0232309	-0.1165	-0.04055	0.103864	0.034198
ZDBF2	-0.008766218	-0.011715155	0.123874	-0.00629	0.020956	-0.16179
ZDHHC1	-0.023727518	-0.12247542	-0.2077	-0.03284	0.121206	0.447067
ZDHHC11	-0.009475108	-0.041936636	0.097615	-0.03051	0.053083	-0.16712
ZDHHC12	0.023758835	-0.063990079	-0.08021	0.063428	-0.22883	0.204179
ZDHHC13	-0.004074202	0.064546602	0.019852	0.069303	-0.21552	-0.24379
ZDHHC14	-0.021343454	0.014758999	-0.0687	0.028759	-0.03221	-0.07641
ZDHHC15	-0.032763866	-0.013596419	0.014259	0.000806	0.008092	-0.06903
ZDHHC16	0.021579729	-0.004447687	0.013305	0.027368	-0.10782	0.005721
ZDHHC17	0.01879176	-0.026270498	-0.13106	-0.01625	0.02956	-0.32191
ZDHHC18	-0.013826025	-0.058097131	-0.08722	-0.00845	-0.02347	-0.17563
ZDHHC19	-0.034253175	-0.100135588	-0.10465	-0.00027	-0.00663	-0.10962
ZDHHC2	0.017084703	0.010406632	-0.02968	-0.01261	0.054024	-0.06134
ZDHHC20	0.036939032	0.1804177	-0.00254	0.01153	-0.01382	0.415689
ZDHHC21	0.036734708	0.119077538	-0.09114	-0.04517	0.080645	-0.1386
ZDHHC22	-0.033851919	-0.054388057	-0.02382	-0.01284	-0.01042	0.00988
ZDHHC23	-0.004812584	-0.098906731	-0.08006	-0.0534	0.156533	-0.23893
ZDHHC24	0.027548429	0.072666308	-0.00146	0.031822	-0.22747	-0.0258
ZDHHC3	0.012526692	0.018271522	-0.24015	0.006614	-0.0577	0.341321
ZDHHC4	0.004867702	-0.017301648	0.049128	0.045571	-0.09217	0.056834
ZDHHC5	0.045176542	0.042354725	-0.1256	0.038649	-0.1058	0.042862
ZDHHC6	0.082825328	-0.01033901	0.116047	0.000302	-0.00228	-0.19522
ZDHHC7	0.063571234	-0.01778767	-0.27563	-0.0557	0.188472	0.505326
ZDHHC8	-0.000848875	-0.043447783	0.120826	-0.00232	-0.03849	0.084047

ZDHHC8P1	-0.039831516	-0.058649024	0.023634	-0.00293	0.019664	-0.03633
ZDHHC9	-0.008438444	-0.083567972	0.075202	-0.04644	0.100962	-0.02875
ZEB1-AS1	-0.008204291	-0.019174756	0.056237	0.049805	-0.13577	-0.37529
ZEB2	0.023322785	0.072521221	-0.23646	-0.0564	0.189685	0.349622
ZER1	-0.023057823	-0.095955201	0.003979	-0.02827	0.03502	-0.08131
ZFAND1	0.01992344	-0.037052692	0.211592	0.010885	-0.03246	0.076087
ZFAND2A	0.062019797	0.045013079	-0.0153	0.002941	-0.01578	-0.05586
ZFAND2B	0.034179503	0.023141098	0.121233	-0.0241	0.023859	-0.16084
ZFAND3	0.023359368	-0.019298638	-0.12782	-0.06589	0.134017	0.197505
ZFAND4	-0.017468397	0.051178665	0.147705	-0.03009	0.122457	-0.0847
ZFAND5	-0.071773908	-0.04579119	-0.10469	-0.01971	-0.01539	0.353116
ZFAND6	0.065401506	0.054764274	-0.18362	0.009725	0.019349	-0.23775
ZFAS1	0.066186118	-0.154452145	0.188828	0.056185	-0.13432	0.093272
ZFAT	-0.001849753	-0.099963489	-0.02046	-0.02904	0.02753	-0.1594
ZFC3H1	0.012421425	-0.027154028	-0.04523	-0.00599	0.002128	-0.16236
ZFHX2	-0.020964926	-0.074722669	-0.01441	-0.01465	-0.04818	-0.01949
ZFHX3	0.020540142	-0.002095924	-0.31764	-0.00846	0.179006	0.271197
ZFHX4	-0.049823848	-0.031320108	0.04258	-0.03071	0.027718	0.002621
ZFHX4-AS1	-0.048914332	-0.061471393	-0.03553	0.00988	-0.02717	-0.0404
ZFP1	-0.047107654	-0.041849496	-0.04446	0.007364	0.015958	0.026663
ZFP14	-0.025333704	0.04921034	0.145044	-0.00478	0.015802	-0.15159
ZFP2	-0.038747523	0.024225704	0.105595	-0.05301	0.173771	-0.08627
ZFP28	-0.048758623	0.097294988	0.160749	-0.09131	0.19251	-0.27354
ZFP3	0.000297686	0.098979215	0.057261	-0.08816	0.266487	-0.04978
ZFP30	0.00415667	0.08843326	0.187432	0.000546	0.031058	-0.21137
ZFP36	0.040245654	0.02397807	-0.20282	-0.04725	0.141854	-0.0056
ZFP36L1	-0.012927556	-0.011554596	-0.08437	-0.06514	0.160981	0.088487
ZFP36L2	-0.004165352	0.009122114	0.175602	-0.08321	0.313271	-0.0221
ZFP37	-0.024504821	0.093104118	0.094579	-0.00953	0.051451	-0.17937
ZFP41	-0.02176653	-0.067980947	0.322093	0.085218	0.014164	-0.11764
ZFP42	-0.02902977	-0.004905835	-0.04713	0.094135	-0.23317	0.000761
ZFP57	-0.015112389	0.001244855	-0.02899	-0.01939	-0.00305	-0.04264
ZFP62	0.021428588	0.040332318	0.272544	0.028201	-0.07382	-0.16915
ZFP64	-0.030349337	-0.066342453	-0.0214	0.011964	-0.14948	0.151047
ZFP69	-0.004456207	0.100516394	0.054774	-0.03565	0.125569	0.08589
ZFP69B	-0.01693291	0.07710301	0.265752	-0.03847	0.133153	-0.08349
ZFP82	-0.008107355	0.099059597	0.088606	-0.06388	0.189512	-0.31231
ZFP90	-0.016323788	0.057019714	0.206792	-0.02328	0.048122	-0.10431
ZFP91	0.081418134	0.123073656	-0.13713	0.083725	-0.2377	-0.13471
ZFP92	-0.003476793	0.024996387	0.00148	-0.0547	0.036712	0.0275
ZFPL1	-0.002107059	-0.137204703	-0.05891	-0.00285	0.004119	0.04393
ZFPM1	0.003925614	0.020893059	0.085052	-0.00437	-0.02598	-0.02404
ZFPM2	-0.030401711	-0.010221628	0.034647	-0.00826	-0.00229	0.021689
ZFR	0.019260465	0.066333578	0.01543	0.034837	-0.11857	0.089892
ZFR2	-0.040458323	0.021624801	0.093456	0.001848	-0.00175	-0.16633
ZFX	0.000748669	0.090816753	-0.1222	0.072596	-0.14584	-0.06039
ZFY	-0.000900981	0.028627231	0.031896	-0.03846	0.128151	-0.02221
ZFYVE1	0.021787748	0.043871829	-0.13594	0.000677	-0.03695	0.06843
ZFYVE16	0.023812467	0.056660983	-0.29255	0.083513	0.079586	0.142611
ZFYVE19	0.018724849	0.017933331	-0.00343	-0.00722	0.002571	0.084835
ZFYVE20	-0.033879493	-0.113806103	0.061849	0.039997	0.005197	-0.00764

ZFYVE21	0.005549806	0.029713243	-0.19687	-0.02822	0.077715	0.370089
ZFYVE26	0.02657518	-0.003028394	-0.0698	-0.02312	0.077636	0.094967
ZFYVE27	0.03454911	-0.038213987	0.026669	-0.0532	0.07925	-0.16308
ZFYVE28	-0.042919366	0.237095668	0.160424	0.004695	-0.10831	-0.10892
ZFYVE9	-0.032357227	-0.055086225	0.076179	-0.01208	-0.00853	-0.0489
ZG16	-0.02693033	-0.137429985	-0.04219	0.018979	-0.05718	0.07037
ZG16B	0.001628408	-0.05704626	-0.12691	0.005256	0.010217	0.581274
ZGLP1	-0.013463274	-0.116468491	0.027944	-0.03629	0.040333	0.278263
ZGPAT	0.042000468	-0.005947351	0.115742	-0.02238	0.046372	0.10162
ZGRF1	-0.010513824	0.085252262	0.031741	0.105483	-0.29164	-0.13097
ZHX1	0.013944743	0.118303221	-0.13852	0.043322	-0.07639	0.163627
ZHX1-C8oı	-0.00204142	0.036914695	-0.07788	0.077966	-0.20209	0.147779
ZHX2	0.008360881	0.03393411	0.114822	-0.07227	0.183974	-0.43218
ZHX3	-0.026278564	-0.061931678	0.057192	0.031358	-0.06278	0.033237
ZIC1	-0.03099973	-0.075819623	-0.02871	0.021084	-0.05584	0.022731
ZIC2	-0.021165514	-0.022404997	-0.02767	-0.05732	0.041793	-0.0048
ZIC3	-0.040429241	-0.003768112	-0.0207	-0.01645	0.07524	0.017261
ZIC4	-0.058664198	-0.031872788	0.013983	0.006211	0.015137	0.107203
ZIC5	-0.034870805	-0.034210657	-0.1369	0.002379	0.014363	0.006226
ZIK1	-0.008585101	0.039324315	0.078905	-0.05812	0.180602	-0.19395
ZIM3	-0.056965814	-0.026055436	-0.02686	-0.0158	0.02299	0.075198
ZKSCAN1	0.028727688	0.080679329	0.105103	-0.01696	0.054575	0.046996
ZKSCAN2	-0.02479952	-0.034503956	-0.08194	0.092386	-0.26532	-0.09187
ZKSCAN3	-0.01313706	0.051133323	0.046121	0.004547	-0.01586	-0.13019
ZKSCAN4	0.015592849	0.031242527	-0.01243	-0.09251	0.248683	-0.09305
ZKSCAN5	-0.000814868	0.055884381	-0.05836	0.063873	-0.18608	-0.05447
ZKSCAN7	-0.013142277	0.17825122	0.143976	-0.07313	0.231923	-0.20576
ZKSCAN8	0.002505802	0.096373778	0.134246	-0.03702	0.122182	-0.21538
ZMAT1	-0.009739065	-0.04890809	0.210766	-0.11798	0.338015	-0.28455
ZMAT2	0.102114701	0.091392661	-0.02332	0.037341	-0.05991	-0.1227
ZMAT3	-0.004452962	0.130223907	0.145298	0.058243	-0.21681	0.070076
ZMAT4	-0.022580337	0.136313972	-0.12872	-0.04363	0.064369	-0.05866
ZMAT5	-0.000420119	-0.06492215	-0.16674	0.047574	-0.14438	0.272909
ZMIZ1	-0.041912582	-0.15699404	-0.13161	-0.07044	0.076375	0.133781
ZMIZ1-AS:	-0.021425431	-0.075904443	0.009188	-0.0123	0.017839	-0.03507
ZMIZ2	-0.000250132	-0.094568818	-0.03325	-0.02707	-0.02127	-0.17571
ZMPSTE24	0.066276596	0.16451727	-0.26626	0.078246	-0.18164	0.061868
ZMYM1	0.005289859	0.095674997	0.102258	0.01537	-0.00505	-0.00371
ZMYM2	-0.002617282	-0.071480967	0.097425	-0.02633	0.087908	0.006324
ZMYM3	-0.003961099	-0.044113557	-0.00379	0.006553	0.005521	0.058733
ZMYM4	0.027961534	0.042198544	-0.00903	0.004287	0.072759	0.133329
ZMYM5	0.000825806	0.058664267	0.089319	0.054056	0.009373	0.042381
ZMYM6	0.00705068	-0.037636142	-0.14973	-0.02419	0.129509	0.201138
ZMYND10	-0.040229259	-0.005487932	-0.02682	0.001965	-0.00333	-0.0412
ZMYND11	-0.000154396	0.147555684	0.238086	-0.04364	0.08134	-0.32292
ZMYND12	-0.0279736	-0.050232532	-0.04726	-0.15028	0.447987	0.101105
ZMYND15	-0.021292632	-0.132532037	-0.09947	-0.01313	0.055109	0.210661
ZMYND19	0.016787775	-0.005696223	0.080243	0.067208	-0.18837	-0.00825
ZMYND8	0.064972008	0.08368652	0.089574	0.062083	-0.03129	-0.14052
ZNF10	-0.0090198	0.085946544	0.223922	-0.04881	0.189754	-0.35892
ZNF100	-0.023271473	-0.00301218	-0.19854	-0.00513	0.036915	0.173496

ZNF101	0.009831242	-0.011027714	0.475335	-0.03547	0.058465	-0.27516
ZNF106	0.041463246	-0.01237984	-0.37484	-0.03576	0.11414	0.421523
ZNF107	0.015412009	-0.009897236	-0.0296	0.043617	-0.09584	-0.3579
ZNF112	-0.024335325	-0.049414296	-0.07068	-0.02668	0.085284	-0.04488
ZNF114	-0.051043087	-0.0154702	0.096663	0.001806	0.013319	-0.09483
ZNF117	-0.020698527	0.040050005	-0.25594	-0.03248	0.105023	-0.04447
ZNF12	0.052373373	0.066618876	0.131451	-0.01893	0.074502	-0.16502
ZNF121	0.028520713	0.063467935	0.262815	0.037954	-0.0884	-0.01759
ZNF124	0.010478638	-0.017814593	-0.06025	-0.00212	0.031032	0.043229
ZNF132	-0.026045068	0.025501004	0.055454	-0.02104	0.066029	-0.03622
ZNF133	0.008266416	0.012407073	0.070136	-0.02181	0.06297	-0.01219
ZNF134	0.007246685	0.057807041	-0.02413	-0.06092	0.113243	-0.09318
ZNF135	-0.032541208	0.000153708	0.03507	-0.03514	-0.00844	-0.34993
ZNF136	0.032150112	0.080960139	0.125307	-0.04588	0.106015	-0.1908
ZNF137P	-0.004561283	0.138231228	0.064686	-0.06473	0.185926	-0.17802
ZNF138	-0.018248157	0.047986499	0.14795	0.015722	-0.01151	-0.30347
ZNF14	0.015861506	0.034997901	0.320775	-0.02751	0.083955	-0.43421
ZNF140	0.01520557	0.041414258	0.016462	-0.03928	0.071331	-0.13196
ZNF141	-0.014045588	0.030467289	0.121251	-0.06245	0.197376	-0.18415
ZNF142	0.024914135	0.065256061	-0.04959	0.039167	-0.12034	-0.10821
ZNF143	0.037988324	0.031577823	-0.19457	-0.00934	0.012425	0.118539
ZNF146	0.027421069	0.079905941	0.183274	0.02276	-0.05158	-0.08022
ZNF148	-0.010535925	0.069090447	-0.15361	-0.00383	0.070034	-0.0522
ZNF154	-0.014077952	0.065861642	0.109776	-0.02515	0.096309	-0.28636
ZNF155	-0.014779637	0.065315834	-0.03804	-0.04688	0.1553	-0.14109
ZNF157	-0.042873256	-0.048941428	0.00282	0.02698	-0.07164	-0.22026
ZNF16	0.007538567	0.059510522	0.008347	0.014101	-0.03662	0.044043
ZNF160	-0.015923933	0.020003758	0.124384	0.001959	0.051217	-0.09814
ZNF165	-0.013906836	-0.027010244	-0.03689	-0.02473	0.05797	-0.08567
ZNF169	0.005190045	0.022823387	0.019956	-0.01092	0.125959	0.049408
ZNF17	-0.033866501	0.068856675	0.026627	-0.00274	0.025755	-0.06922
ZNF174	0.019614556	-0.00979324	0.06947	0.095057	-0.03638	0.020513
ZNF175	-0.002203946	-0.029111224	-0.02391	-0.02625	0.075518	-0.17588
ZNF18	0.028443085	0.043062867	-0.01666	0.009151	-0.0107	-0.1531
ZNF180	-0.009255555	0.087496165	0.025132	-0.03425	0.143966	-0.07664
ZNF182	0.013557575	0.053397048	0.057992	-0.00749	0.031657	-0.09222
ZNF184	0.014866499	0.121459438	0.136507	0.038096	-0.08125	-0.17521
ZNF185	0.006177738	-0.115054595	-0.30863	-0.11825	0.330035	0.44228
ZNF189	0.027284499	0.088926595	0.038189	-0.01685	0.017661	-0.16349
ZNF19	0.015773222	0.073966366	0.074482	0.011397	0.139391	-0.23581
ZNF195	0.011117505	0.064947402	0.157749	0.031247	-0.04376	0.011965
ZNF197	-0.024361261	0.118586864	-0.01172	-0.0227	0.078454	-0.0932
ZNF2	-0.011577592	0.078317206	0.038546	0.000759	-0.04671	-0.11185
ZNF200	0.010302639	0.121932902	-0.0536	0.016751	-0.07898	-0.23623
ZNF202	0.003121123	0.029525668	0.133322	0.034704	-0.0868	-0.14074
ZNF204P	-0.008634425	-0.055293974	0.508618	-0.09225	0.261529	-0.31732
ZNF205	-0.012346648	-0.136783685	-0.0246	0.002002	-0.03859	-0.02546
ZNF205-AS	-0.036361685	0.041284636	0.099596	0.006099	0.002289	-0.06657
ZNF207	0.003268334	0.072180157	0.100245	-0.03208	0.162834	-0.28009
ZNF208	-0.045688035	-0.079764852	0.063672	-0.02682	0.021272	-0.07455
ZNF211	0.027310153	0.129657743	0.074034	-0.10413	0.255584	-0.1983

ZNF212	0.040523375	0.029583741	0.122661	0.007042	-0.08923	-0.11851
ZNF213	0.010134204	-0.032332118	-0.23122	0.018898	-0.12304	0.152126
ZNF213-AS	-0.022303208	0.034556161	0.210355	-0.01836	0.073059	-0.32506
ZNF214	0.003273426	0.048343159	-0.03512	0.116117	-0.11252	-0.0423
ZNF215	-0.062068447	-0.122944644	0.046934	-0.01761	0.046744	0.01004
ZNF217	0.041637433	0.027404076	-0.03047	-0.01108	0.040324	0.029931
ZNF219	-0.028569187	-0.078740885	0.024702	0.013872	-0.09354	0.004932
ZNF22	0.025605347	0.126237811	0.277437	0.02958	-0.07885	-0.11084
ZNF221	-0.042525948	0.009086025	-0.02002	0.003194	-0.00073	-0.19754
ZNF222	0.001024989	0.095951351	0.036324	-0.10537	0.287297	-0.17818
ZNF223	-0.017612366	0.119464233	-0.06879	-0.03989	0.125465	0.092945
ZNF224	-0.001018161	0.10956608	0.052209	-0.00815	0.103329	-0.09606
ZNF225	-0.01344966	0.103443041	0.036645	-0.05811	0.185847	-0.04728
ZNF226	0.06088996	0.120858011	0.101939	-0.07742	0.179926	-0.13834
ZNF227	0.010486615	0.135935159	0.021307	-0.03459	0.121842	-0.09043
ZNF229	-0.052247738	-0.086017588	-0.0139	-0.04478	0.117264	-0.11189
ZNF23	0.007858994	0.036996052	0.176488	-0.00643	0.039455	-0.15294
ZNF230	-0.03826806	0.107445877	-0.09018	-0.03039	0.126952	0.003899
ZNF232	0.015951332	0.055526342	0.086133	0.019537	-0.05034	-0.09902
ZNF233	-0.057807932	-0.061298203	0.006764	-0.04862	0.147513	-0.05608
ZNF234	-0.012158134	0.0702887	0.034937	-0.05633	0.164848	0.020915
ZNF235	-0.015031343	0.072005423	0.009789	-0.05459	0.148875	-0.23016
ZNF236	0.003608377	0.046637052	0.110975	0.003153	-0.02855	-0.20609
ZNF239	-0.021233674	0.044780352	0.164039	-0.12184	0.360075	-0.22773
ZNF24	0.009592697	-0.007654426	0.013423	0.002523	-0.06286	-0.07632
ZNF248	0.001587589	-0.008538495	0.119742	-0.00649	0.028531	-0.21694
ZNF25	0.009636722	0.074086036	0.023066	-0.0396	0.132736	0.162825
ZNF250	-0.02012455	0.103602047	-0.06859	0.027334	-0.06746	-0.08217
ZNF251	-0.002895219	0.03643676	0.144412	0.034102	-0.05106	-0.27391
ZNF252P	-0.004336606	0.03289946	-0.04283	-0.10029	0.275485	0.054984
ZNF252P-/	-0.036619407	-0.064296355	0.102295	-0.02306	0.084209	-0.08908
ZNF253	-0.004089629	0.133442368	0.199249	0.001998	0.021362	-0.37255
ZNF254	-0.02366372	0.00881316	0.104408	-0.02636	0.131911	-0.17809
ZNF256	-0.002323688	0.079684052	0.076601	-0.04941	0.155886	-0.25194
ZNF257	-0.026952865	0.064043814	0.133481	-0.015	0.044925	-0.26153
ZNF259P1	-0.037725653	-0.003815479	-0.0168	0.031339	-0.0647	0.198801
ZNF26	0.008251265	0.035911638	0.114071	-0.00742	0.044997	-0.08695
ZNF260	0.010539857	0.108085258	0.101434	-0.02075	0.065777	-0.19296
ZNF264	0.034475456	0.127320976	0.040412	0.010042	0.149892	-0.07863
ZNF266	0.049778378	0.150703918	0.214312	-0.0593	0.158348	-0.34593
ZNF267	0.022865262	0.064721313	-0.22928	0.068459	-0.17407	0.118811
ZNF268	-0.030400316	0.061475136	0.137101	0.026799	0.047255	-0.14164
ZNF271	-0.006759737	0.0360052	-0.00522	-0.06075	0.19079	0.193709
ZNF273	-0.003720292	0.033246586	0.22369	0.016229	-0.00581	-0.27413
ZNF274	0.02333683	-0.050300929	0.151709	-0.07455	0.136858	-0.51109
ZNF275	0.018965073	0.031016035	0.268885	-0.03973	0.094512	-0.23646
ZNF276	0.013124255	0.141658642	0.126287	-0.06392	0.189067	-0.14758
ZNF277	0.0170878	0.039835498	0.12277	0.029002	0.008643	0.267421
ZNF28	-0.040036948	0.010943914	0.050066	-0.01482	0.04744	0.009437
ZNF280A	-0.041634869	-0.006815276	-0.13464	0.027707	-0.06356	-0.02397
ZNF280B	0.054188679	0.077321522	0.24116	-0.02204	-0.04355	-0.35995

ZNF280C	-0.016997492	0.076301846	0.090928	0.141802	-0.37868	-0.10231
ZNF281	0.047419883	0.027450785	-0.12505	0.041329	-0.1182	0.034107
ZNF282	-0.002385621	0.03844428	-0.02688	0.062492	-0.25804	0.056209
ZNF283	-0.020850068	0.054055869	0.095765	-0.03358	0.105879	-0.00755
ZNF284	-0.006146392	0.101065017	0.154552	-0.06641	0.159294	-0.1762
ZNF286A	0.032423398	0.129779575	0.108723	0.128141	-0.14391	-0.20853
ZNF287	-0.006818773	-0.008860755	0.113844	0.033676	0.162614	-0.1399
ZNF292	-0.000221381	0.035905291	0.025068	0.012372	-0.06673	-0.32617
ZNF295-AS	-0.027691302	-0.006539186	0.040279	0.001	-0.00745	-0.07124
ZNF296	-0.006821122	-0.052506995	-0.21453	-0.02065	0.079004	0.044153
ZNF29P	-0.055665347	-0.123127726	-0.0011	-0.02152	0.054848	-0.12411
ZNF3	-0.062469263	-0.029850749	-0.01187	-0.05617	-0.0058	-0.08272
ZNF30	-0.004865873	0.127574635	0.270833	-0.06825	0.214874	-0.36464
ZNF300	-0.012518975	0.207435876	-0.02874	-0.06123	0.190135	-0.07057
ZNF300P1	-0.052622395	-0.006559381	-0.02214	-0.05205	0.167786	-0.01228
ZNF302	0.00827938	0.08271739	0.141932	-0.1319	0.241785	-0.1315
ZNF304	-0.001050908	0.118792583	0.031877	0.032055	-0.05477	-0.10039
ZNF311	-0.05633272	-0.04673316	0.032548	-0.00801	0.026253	-0.05749
ZNF316	-0.021597098	-0.104553664	0.002403	-0.00173	-0.0672	0.103884
ZNF317	0.014398562	0.009815756	-0.00526	0.024914	-0.08604	-0.04673
ZNF318	0.0144194	0.06331298	-0.03201	0.004112	0.026748	0.045642
ZNF319	0.010534764	0.107907667	-0.00541	-0.01144	0.001982	-0.00614
ZNF32	0.021214682	0.074967902	0.224821	-0.11142	0.32536	-0.12386
ZNF32-AS	-0.065965836	-0.064813391	-0.0882	0.019435	-0.04947	0.035317
ZNF320	0.001880122	0.096260932	0.127347	-0.07075	0.201087	-0.10223
ZNF322	0.005696292	0.111888007	-0.06113	-0.01724	0.053707	-0.02107
ZNF324	0.014694362	0.053564685	0.14156	0.001017	-0.04162	-0.30163
ZNF324B	-0.031990819	-0.081469874	-0.14286	0.035608	-0.08069	0.078252
ZNF326	-0.022621244	-0.029273234	0.006169	-0.01645	-0.01174	0.139874
ZNF329	0.003324124	0.092805879	0.227916	-0.09872	0.285621	-0.4227
ZNF330	0.041340019	-0.014001007	-0.03476	0.071573	-0.21073	0.336323
ZNF331	0.014202321	-0.008877556	0.216182	-0.04494	0.131324	-0.1241
ZNF333	0.006707062	-0.036090807	-0.19366	-0.0483	0.170351	0.199708
ZNF334	-0.032825812	-0.077652706	-0.03702	0.000934	########	-0.09722
ZNF335	-0.014088988	-0.062344524	0.235846	-0.04622	0.122735	-0.11195
ZNF337	0.021763756	0.007371243	0.296497	0.02674	-0.09371	-0.32183
ZNF33A	-0.026204933	0.004783087	0.034087	-0.06878	0.063013	-0.12943
ZNF33B	0.00370641	-0.02916087	0.070051	-0.01975	0.101795	0.009895
ZNF34	0.000922065	0.03455402	-0.08046	-0.01089	0.070496	0.210054
ZNF341	-0.022222057	0.008147742	-0.02813	0.029366	0.062304	0.08632
ZNF343	-0.027056019	-0.024417468	0.042707	-0.00773	-0.05197	0.052605
ZNF345	-0.016681958	0.076004906	0.188648	-0.0601	0.167541	-0.25711
ZNF346	0.055189581	0.013820937	0.152875	0.09197	-0.2357	-0.22432
ZNF347	-0.021307367	0.143156365	0.019079	-0.04669	0.14187	-0.2288
ZNF35	-0.009575641	0.106648852	-0.20751	-0.03792	0.081669	0.13959
ZNF350	-0.003008307	0.064279829	-0.11435	-0.12416	0.351713	-0.02782
ZNF354A	0.027247141	0.060842514	-0.11712	-0.05222	0.147105	0.103955
ZNF354B	-0.012976788	0.047987947	-0.02511	-0.0824	0.234679	0.059948
ZNF354C	-0.030325521	0.088998808	0.161364	-0.07501	0.161355	-0.30062
ZNF358	-0.015336743	-0.097676322	0.000415	-0.04616	0.091886	0.049588
ZNF362	-0.032108307	-0.070971553	0.018908	0.028604	-0.09047	0.094565

ZNF365	-0.02178782	0.051665382	0.24115	-0.02156	0.080043	-0.12838
ZNF366	-0.033721862	0.036964814	-0.09988	-0.06436	0.214635	0.014098
ZNF367	-0.000598327	0.056315307	0.252195	0.057156	-0.14974	-0.2372
ZNF37BP	0.035970081	0.127444016	0.144021	-0.10583	0.113301	-0.11391
ZNF382	-0.012457464	-0.015866164	0.208292	-0.04586	0.148218	-0.3191
ZNF383	0.000494602	0.077711158	0.063789	-0.0361	0.132956	-0.10031
ZNF384	0.03668547	-0.042354117	0.068853	-0.00521	-0.02194	-0.27595
ZNF385A	0.000720045	-0.091665924	-0.23074	-0.06422	0.175337	0.508132
ZNF385B	-0.084131459	-0.073227643	-0.12932	0.004253	-0.03368	-0.11729
ZNF385C	-0.014524222	-0.043252973	-0.13004	0.010469	-0.00379	-0.03582
ZNF385D	-0.036797337	-0.070670158	-0.0139	-0.16722	0.488067	-0.05076
ZNF391	-0.038491102	-0.057989433	0.130335	0.020539	0.006709	-0.27063
ZNF394	0.047439613	0.087376419	0.15648	-0.02575	0.055755	-0.25046
ZNF395	0.017490104	0.024962546	0.109986	-0.03631	0.080743	-0.19498
ZNF396	-0.125995157	-0.057282987	0.040236	-0.11703	0.072973	0.09588
ZNF397	-0.039383815	0.015364067	-0.02983	-0.00273	0.105982	0.026758
ZNF398	0.010632369	0.116688931	0.010273	0.000772	0.033752	-0.3134
ZNF404	-0.007296942	0.070443339	0.075966	-0.10119	0.30574	-0.27605
ZNF407	-0.058469908	-0.060439474	0.009933	-0.01774	-0.02011	0.069086
ZNF408	0.039266006	-0.051139784	-0.01692	-0.00584	-0.06114	-0.069
ZNF41	-0.027391632	0.05468612	-0.0256	-0.01671	0.056731	0.047318
ZNF414	-0.03297453	-0.046008992	0.007939	-0.00754	0.061597	0.249462
ZNF415	-0.019067889	0.084480243	-0.01251	-0.04988	0.148146	-0.23065
ZNF416	-0.016687653	0.046619542	0.104847	-0.02819	0.075834	-0.11256
ZNF417	-0.04369412	-0.014313733	0.008441	-0.0495	0.140058	0.055198
ZNF418	-0.014107188	0.11189166	-0.15824	-0.03377	0.082886	-0.28713
ZNF419	0.021132668	0.091457757	0.236147	-0.04765	0.088687	-0.44715
ZNF420	0.00974318	0.105522026	0.226547	-0.04297	0.13071	-0.22559
ZNF423	-0.049110826	0.026284882	0.079643	-0.00472	0.027338	-0.05734
ZNF425	-0.042333545	0.019671266	-0.04982	0.028623	-0.06102	-0.13798
ZNF426	-0.002307364	0.080439287	0.041513	-0.13381	0.381506	-0.00605
ZNF428	-0.006455717	-0.037408721	0.283248	0.052314	-0.16006	-0.20193
ZNF429	-0.03296707	-0.087337353	0.003355	-0.00727	-0.02342	-0.07222
ZNF43	0.008796485	0.047324904	0.185007	-0.0605	0.20216	-0.23248
ZNF430	0.01902833	0.080592694	0.152812	-0.04244	0.136725	-0.26046
ZNF431	-0.033805466	0.12798524	0.102735	-0.01961	0.058562	-0.22303
ZNF432	0.013634373	0.118381401	0.098731	-0.0374	0.100103	-0.15736
ZNF436	-0.001739668	0.091618075	-0.00211	0.012698	-0.01724	0.081424
ZNF438	0.002351578	-0.046919356	-0.23023	-0.03456	0.095224	0.399506
ZNF439	-0.002983898	0.043909749	0.024815	-0.08461	0.246788	-0.43393
ZNF44	-0.016549601	0.142390293	0.087847	-0.10235	0.274842	-0.1243
ZNF440	-0.055397918	0.160876772	-0.00168	-0.03417	0.175073	0.072301
ZNF441	-0.017750388	0.147785864	0.070218	-0.03989	0.130167	-0.20993
ZNF442	-0.049777011	0.083825284	0.03052	-0.05006	0.1453	-0.09892
ZNF443	-0.054295536	-0.058892707	0.098528	-0.06092	-0.00849	-0.07885
ZNF444	0.011312043	-0.082060127	0.183194	0.025736	-0.18045	-0.22316
ZNF445	0.011112436	0.000492178	0.029535	0.007871	-0.0972	-0.18029
ZNF446	0.004101573	-0.079872259	0.101307	-0.0235	-0.056	-0.19294
ZNF449	0.0032573	-0.001991502	-0.01872	-0.00922	0.027999	-0.00508
ZNF45	0.014610049	0.127345532	0.010556	-0.01051	0.060518	-0.07321
ZNF451	0.00266053	0.01501308	0.117173	0.008079	-0.01374	-0.0723

ZNF460	-0.086090566	-0.005381096	0.05487	-0.08342	-0.08213	-0.00538
ZNF461	-0.038957106	0.004364419	0.111005	########	0.018979	-0.13634
ZNF462	0.014915125	-0.018801631	-0.01645	0.043344	-0.0223	-0.1018
ZNF467	0.000115643	-0.1287991	-0.28059	-0.08504	0.251932	0.436949
ZNF468	0.015900547	0.170528029	0.016023	-0.07017	0.19375	-0.05092
ZNF469	0.014326603	0.049640785	-0.16696	-0.01014	-0.01851	-0.21756
ZNF470	-0.011695682	0.103832347	0.115686	-0.08434	0.251624	-0.20413
ZNF471	-0.126160713	-0.088097681	0.014092	-0.07092	0.177689	-0.10482
ZNF473	-0.004459824	0.032320382	-0.02839	0.071921	-0.21776	-0.07821
ZNF474	-0.043072917	-0.035123332	0.031911	0.009531	0.001544	0.028828
ZNF48	0.023662324	-0.066375433	0.082889	0.055294	-0.26132	-0.25548
ZNF480	-0.016151985	0.094945454	0.181805	-0.06462	0.198194	-0.13143
ZNF483	0.002065125	-0.133063921	0.133594	0.047308	0.020935	-0.17472
ZNF484	-0.029087554	0.045612221	0.000541	0.036057	-0.09333	-0.03165
ZNF485	-0.024448064	0.038314568	0.1549	-0.02361	0.061477	-0.14675
ZNF486	-0.025274272	0.022556129	0.017571	-0.02777	0.100212	-0.11423
ZNF488	-0.029821451	-0.036993482	0.086386	-0.00869	0.005687	-0.05293
ZNF490	-0.013492004	-0.00159504	0.07282	-0.00714	-0.06364	-0.16514
ZNF491	-0.022532187	-0.016006668	0.019037	0.003261	-0.03004	-0.12197
ZNF492	-0.030899979	-0.041113921	-0.0074	-0.00365	0.028628	-0.11062
ZNF493	-0.002625958	0.071055838	0.178126	-0.06723	0.155082	-0.42089
ZNF496	-0.047613919	-0.040928938	0.316595	-0.00717	-0.07184	-0.16719
ZNF497	-0.039471548	0.036500648	-0.027	0.055261	-0.13669	0.018815
ZNF500	-0.019673714	-0.026741378	0.192827	-0.03048	0.112224	-0.10484
ZNF501	-0.033773122	0.138537331	-0.04068	-0.01934	0.071291	-0.2301
ZNF502	-0.007642721	0.13705539	0.060202	-0.06738	0.203289	-0.22391
ZNF503	0.034914435	0.062706215	-0.11613	-0.07535	0.108328	0.319546
ZNF503-AS	-0.0293091	-0.034181343	-0.0128	-0.04099	0.076015	0.013489
ZNF503-AS	-0.010647137	-0.106298191	0.07065	0.088866	-0.09257	0.238907
ZNF506	-0.006263798	0.118258017	0.023263	-0.06482	0.150478	-0.19268
ZNF507	-0.011322275	0.07531647	0.174453	0.017219	-0.10267	-0.40933
ZNF510	-0.005650884	0.043936511	0.183617	-0.02512	0.096609	-0.22881
ZNF511	0.043378712	-0.065171855	-0.12626	0.009439	-0.06243	0.199311
ZNF512	0.026034092	-0.016242511	0.37019	-0.01698	0.01794	-0.38334
ZNF512B	0.002845356	-0.003948711	0.161683	-0.05895	0.188313	-0.17164
ZNF513	0.001929637	-0.041820359	-0.06647	-0.07328	0.210377	0.169059
ZNF514	0.003198754	0.034976377	0.135414	-0.06834	0.198367	-0.08152
ZNF516	-0.003309104	-0.040077954	-0.30089	-0.11614	0.314755	0.251253
ZNF517	-0.033991278	-0.047407645	-0.14065	0.005798	0.008603	-0.0116
ZNF518A	-0.005842792	0.049617289	-0.01672	-0.05829	0.15395	0.077928
ZNF518B	-0.052506421	-0.062684658	0.082155	-0.0656	0.155548	-0.02241
ZNF519	-0.027402527	-0.027707818	0.064949	0.156718	-0.29615	-0.19031
ZNF521	-0.01188281	-0.059788439	-0.04433	-0.01163	0.049979	-0.19454
ZNF524	-0.004852648	-0.102770799	-0.0234	-0.00611	-0.01631	0.241553
ZNF526	-0.016067913	0.061777326	0.133358	0.048937	-0.12046	-0.0646
ZNF527	-0.019714963	-0.019074078	0.086334	-0.01354	-0.04444	-0.12168
ZNF528	-0.013904883	-0.025342925	0.000941	-0.00649	-0.0354	-0.15262
ZNF529	-0.047941644	-0.026222561	0.335347	-0.113	0.268318	-0.28585
ZNF530	-0.022757618	0.030328423	-0.00945	-0.01008	0.050252	-0.05222
ZNF532	-0.012262768	-0.069075657	-0.10126	-0.04873	0.104862	0.089601
ZNF536	-0.062025459	-0.006051778	0.070481	-0.03967	-0.00887	0.03616

ZNF540	-0.014355456	0.088321678	0.386218	-0.05825	0.174124	-0.40019
ZNF541	-0.023368986	0.038740537	0.07549	-0.01915	0.035453	0.002786
ZNF542P	-0.001595833	0.095338174	0.015393	-0.14942	0.441856	-0.18138
ZNF543	-0.044492437	0.004124592	-0.01702	-0.00831	0.030249	-0.03309
ZNF544	0.004212089	0.034004794	0.244018	0.038544	-0.1308	-0.36152
ZNF546	-0.026937909	0.010476023	0.151405	-0.03152	0.098017	-0.0144
ZNF547	-0.048214083	-0.033948431	0.004736	0.026108	-0.07515	-0.12404
ZNF548	-0.002012649	0.078059959	0.302651	-0.00044	0.022823	-0.29471
ZNF549	-0.032934313	0.059806894	0.032714	-0.02561	0.079701	-0.09885
ZNF550	-0.003734998	0.041209308	0.3621	-0.0439	0.079345	-0.45718
ZNF551	0.012410766	0.004172887	0.096078	-0.17794	0.149226	-0.0532
ZNF552	-0.015840098	-0.033072202	-0.0319	-0.00614	-0.04506	-0.17438
ZNF554	-0.018919084	-0.026773997	0.178359	-0.01593	-0.04097	-0.22626
ZNF555	-0.001524649	0.113897388	0.009207	-0.04304	0.105204	-0.0701
ZNF556	-0.01660438	-0.042008821	-0.02323	0.003209	0.019041	-0.06945
ZNF557	-0.013183795	0.114768796	-0.03017	0.023803	-0.03715	0.049332
ZNF558	0.027631161	0.008941888	0.279352	0.001436	-0.01423	-0.14502
ZNF559	0.019191697	0.078268564	0.215411	-0.00943	0.026794	-0.2325
ZNF560	-0.07624633	-0.03003866	-0.0512	-0.06056	0.163638	-0.02182
ZNF562	0.014597587	0.055200376	0.004788	-0.00435	0.007971	0.050459
ZNF563	-0.038490069	-0.075810712	0.277324	-0.05832	0.188102	-0.19808
ZNF564	0.014746922	0.119774296	-0.00046	-0.01285	0.050401	-0.13733
ZNF565	-0.000702697	0.116441777	0.018576	-0.04382	0.174756	0.130748
ZNF566	-0.010612517	0.137407388	0.204313	-0.03907	0.129825	-0.25305
ZNF567	-0.01136312	0.087951165	0.230074	-0.03249	0.099756	-0.23042
ZNF568	-0.133307228	0.16169169	0.129694	-0.0554	0.201524	-0.11891
ZNF569	-0.007210161	0.107362238	0.112027	-0.0352	0.133737	-0.17393
ZNF57	-0.00032853	0.063569534	-0.05378	-0.00092	0.023517	0.017662
ZNF570	-0.010327223	0.164250549	0.116223	-0.04166	0.143925	-0.15386
ZNF571	-0.001266922	0.101198845	0.227661	-0.09012	0.27199	-0.34773
ZNF571-AS	-0.023789301	0.041386208	-0.02077	-0.00878	0.039905	-0.15882
ZNF572	-0.030437768	0.065372394	0.014609	-0.01742	0.064824	-0.24915
ZNF573	-0.014166506	0.051809577	0.233204	-0.08188	0.241785	-0.18188
ZNF574	0.015711795	-0.079062457	-0.05963	0.032219	-0.16391	-0.18341
ZNF575	0.009970623	0.025094735	0.134278	-0.07195	0.168821	-0.11571
ZNF576	0.00708392	-0.003026424	0.041957	0.060829	-0.20728	-0.01796
ZNF577	-0.025740899	-0.022235588	0.111393	-0.02244	0.055674	-0.07315
ZNF578	-0.016894229	0.23536195	0.084587	-0.09379	0.289113	-0.29219
ZNF579	0.007399012	-0.12672783	0.006931	0.00433	-0.11212	-0.12719
ZNF580	0.028236166	-0.135223179	-0.05786	0.001433	-0.04308	-0.04309
ZNF581	0.0314819	-0.152454711	0.021026	0.012875	-0.04478	0.216131
ZNF582	-0.014553274	0.009455627	0.095406	-0.05399	0.076522	-0.07754
ZNF582-AS	-0.020509907	0.02996854	0.157172	-0.01893	0.027557	-0.42604
ZNF583	-0.025269483	0.004312703	0.074464	-0.03807	0.09941	-0.09031
ZNF584	-0.009052736	-0.042944636	-0.03381	0.01662	-0.12743	-0.09024
ZNF585A	-0.027364874	0.155820398	0.08445	-0.03046	0.150125	-0.20112
ZNF585B	-0.008209817	0.122704847	0.165628	-0.00564	0.018702	-0.32149
ZNF586	0.005828901	-0.065738579	0.025221	-0.0759	0.187644	-0.23848
ZNF587	0.023335466	0.034587035	0.031307	0.010183	-0.05228	-0.30904
ZNF587B	0.017596653	-0.022964046	0.087862	0.032241	-0.08844	-0.23663
ZNF589	-0.006208535	-0.078250675	0.073717	-0.04053	0.10979	0.013369

ZNF592	0.067130757	-0.094128988	-0.10898	-0.03584	-0.03319	0.012298
ZNF593	0.014954117	0.004952153	-0.0665	0.069248	-0.16963	0.253429
ZNF594	-0.033161632	0.016838256	-0.013	0.079052	-0.20086	-0.06352
ZNF595	-0.015401432	0.025276444	-0.04463	-0.01115	-0.01011	-0.07746
ZNF596	-0.023595446	0.063802826	0.069206	-0.0735	0.218848	-0.03331
ZNF597	-0.014505715	0.023881573	-0.0187	-0.06067	0.158511	-0.1083
ZNF598	0.00837308	-0.047629452	0.135834	0.031606	-0.16076	-0.03652
ZNF599	-0.081631931	0.074403814	0.204752	-0.03153	0.118225	-0.24065
ZNF600	0.015100493	0.228482298	0.19199	-0.05825	0.176101	-0.24082
ZNF605	-0.034872239	0.021970441	0.093309	-0.05354	0.171452	-0.07615
ZNF606	0.000659039	0.010986128	0.066626	-0.06957	0.187383	-0.27014
ZNF607	-0.0208259	0.098245157	0.097137	-0.0808	0.245292	-0.2143
ZNF608	-0.024453063	-0.096918082	-0.13108	0.049006	-0.14095	-0.28994
ZNF609	-0.02224993	-0.058402973	0.216978	0.038871	-0.14085	0.024798
ZNF610	-0.030427252	-0.035349389	0.156166	-0.02599	0.0969	-0.26701
ZNF611	0.032280131	0.055133214	0.119771	-0.01505	0.04209	-0.29794
ZNF613	-0.030239588	0.0964656	-0.0399	-0.02303	0.081958	-0.06401
ZNF614	0.013206411	0.051815649	-0.00643	0.017972	0.002261	-0.02681
ZNF615	0.012054398	0.113054271	0.011957	-0.08942	0.189517	-0.12688
ZNF616	-0.027147768	0.111865969	0.122798	-0.04307	0.12211	-0.05822
ZNF618	-0.017955536	0.112135516	-0.2	-0.03497	0.183931	0.065207
ZNF619	-0.028323697	-0.024717387	-0.05129	-0.01998	-0.0337	-0.09023
ZNF620	-0.099755496	-0.049198762	-0.02326	0.001267	-0.00172	-0.00858
ZNF621	0.00191375	0.144973668	0.037368	-0.02868	0.090755	-0.03355
ZNF622	0.069577889	0.031061127	0.061846	0.07329	-0.20657	-0.0377
ZNF623	-7.59E-05	0.085573299	0.123533	-0.00633	0.045842	-0.00141
ZNF624	-0.014335614	0.126977181	0.01537	-0.05012	0.161312	-0.01329
ZNF625	-0.034813482	0.014263242	-0.02146	-0.00622	0.052716	0.085989
ZNF626	-0.04445525	0.063885323	0.091478	-0.06192	0.097425	-0.12434
ZNF627	0.003336553	0.042443839	-0.00461	-0.0648	0.192343	-0.04683
ZNF628	-0.000827893	-0.039491268	-0.05727	0.057306	-0.11808	0.111891
ZNF629	0.006598465	-0.119047288	-0.02619	-0.04719	0.071456	0.1347
ZNF630	-0.01980578	0.077870407	0.040591	-0.10737	0.319414	-0.08971
ZNF638	0.049987326	-0.021264143	0.017053	-0.01406	0.045003	-0.19459
ZNF639	-2.47E-05	-0.012337137	0.225649	0.04274	-0.09931	-0.08263
ZNF641	0.015566018	0.081535903	-0.16396	-0.0686	0.208675	0.082841
ZNF644	0.014398752	0.128757768	0.086168	0.039604	-0.08288	-0.02756
ZNF645	-0.032037932	-0.080182235	0.145347	0.022901	-0.05062	-0.00703
ZNF646	-0.0039246	-0.124011222	-0.03038	0.016953	-0.08331	-0.00342
ZNF649	-0.018998707	0.165894178	-0.05109	-0.07157	0.208948	-0.0453
ZNF652	0.027392226	0.021879911	-0.0448	-0.04495	0.118003	-0.01364
ZNF653	-0.027980017	-0.014429725	-0.0104	0.03188	-0.05852	0.072619
ZNF654	0.00784245	0.059629407	0.047063	0.052092	0.010864	-0.11893
ZNF655	0.058176309	0.105902961	0.137878	0.027889	-0.05181	-0.08889
ZNF658	-0.012195911	-0.006149479	0.197663	-0.08413	0.251277	-0.10564
ZNF660	-0.029076183	0.127375523	0.032584	-0.01042	0.060071	-0.07126
ZNF662	-0.025866665	-0.072240131	0.4223	-0.03331	0.118921	-0.26825
ZNF663P	-0.045373513	-0.086175545	-0.01497	0.017947	-0.02838	-0.00981
ZNF664	0.004698619	0.071564502	0.102366	0.037393	-0.07192	-0.13288
ZNF665	0.011330427	0.069303641	0.171136	-0.10189	0.249946	-0.3157
ZNF667	-0.024300941	-0.091091192	0.114749	-0.05445	0.17209	-0.37857

ZNF667-AS	-0.00180626	-0.061909859	0.190961	-0.1019	0.286095	-0.42795
ZNF668	-0.011434816	-0.007045889	-0.09397	0.041191	-0.09737	0.279494
ZNF669	0.012298482	0.089300444	0.201312	-0.01005	0.070983	-0.16226
ZNF670	-0.022574166	0.033236164	0.004386	0.060503	-0.1559	-0.04487
ZNF671	0.011728997	0.022290175	0.10157	-0.07741	0.165792	-0.28115
ZNF672	0.100995272	-0.10447619	-0.01156	-0.01104	-0.02862	0.008042
ZNF674	-0.099645152	-0.057918839	-0.03512	-0.01705	0.102372	0.037339
ZNF674-AS	-0.014769575	0.008995385	0.034911	0.000694	0.012661	0.005336
ZNF675	-0.008480465	0.049816102	0.238469	-0.01434	0.077061	-0.27865
ZNF677	-0.002901884	0.130852675	0.100339	-0.15497	0.311533	-0.28289
ZNF678	-0.014782713	0.128112394	0.102244	-0.00406	-0.07076	-0.20274
ZNF680	0.002365935	0.029587341	0.114386	-0.00922	0.006178	-0.3395
ZNF681	-0.036242052	-0.007196907	0.353244	-0.00795	0.047732	-0.31283
ZNF682	-0.018731986	0.109213786	0.196758	-0.04248	0.134448	-0.25772
ZNF683	0.003688964	0.007386047	0.264045	-0.02664	0.033559	-0.09982
ZNF684	-0.018438689	0.193355671	-0.04297	0.006775	0.005025	-0.01341
ZNF687	-0.002864716	-0.088695615	-0.01896	-0.01426	-0.06548	-0.08924
ZNF688	-0.017296155	0.026137301	-0.09154	0.003928	0.020316	0.014065
ZNF689	0.032695202	0.023817878	0.060402	0.012841	0.001758	0.250442
ZNF691	-0.004447919	0.081167513	-0.03652	0.044	-0.14381	-0.0936
ZNF692	0.017842659	-0.014753896	0.131315	0.025025	-0.11299	-0.29426
ZNF696	-0.015483374	-0.000673726	0.040559	-0.00463	-0.045	-0.13635
ZNF697	-0.011682827	-0.080055704	-0.23411	-0.08854	0.247363	0.543741
ZNF7	-0.046745846	0.005770114	-0.12138	-0.01565	0.035581	0.026884
ZNF70	-0.030180947	-0.04038939	-0.03093	-0.00279	-0.07805	-0.08216
ZNF700	0.020564497	0.038195404	-0.03369	0.011035	-0.00265	-0.11698
ZNF701	-0.012985607	0.104418826	-0.13075	-0.08156	0.198977	-0.11827
ZNF702P	-0.032513765	0.044760364	0.119137	-0.05737	0.17714	-0.0072
ZNF703	-0.002172828	-0.108069221	-0.16431	-0.11559	0.350556	0.554933
ZNF704	-0.124494684	0.011873468	0.10831	-0.045	-0.01118	-0.01565
ZNF705G	-0.045779718	-0.050819893	-0.02018	-0.00107	0.015697	-0.06944
ZNF706	0.023232139	-0.048081489	0.078787	0.008285	-0.03606	0.135458
ZNF708	0.00153857	0.102254772	0.15676	-0.03045	0.129645	-0.09459
ZNF709	-0.048989405	0.011236358	-0.044	-0.07057	0.119589	-0.08869
ZNF71	-0.049495361	-0.036362878	0.032473	-0.00991	-0.05497	-0.01687
ZNF710	-0.055324613	-0.023670269	-0.13085	-0.10272	0.170978	0.290966
ZNF711	-0.013441433	-0.053193327	0.027969	-0.05478	0.147808	-0.32607
ZNF713	-0.03303797	0.007547278	-0.02684	-0.02709	0.063316	0.006108
ZNF717	-0.020532031	-0.026963142	0.141481	-0.05492	0.148066	-0.11116
ZNF718	-0.018980622	0.176276972	-0.00362	0.002465	0.059731	-0.14479
ZNF721	0.064218326	0.116624515	0.183919	0.013176	-0.0158	-0.40287
ZNF732	-0.08136927	-0.01145004	-0.10853	-0.02816	0.079697	-0.03849
ZNF736	-0.008799622	-0.005176468	0.030677	-0.06512	0.00232	-0.22143
ZNF738	-0.002755934	0.08862639	0.078724	0.006112	-0.01178	-0.1065
ZNF74	-0.023903768	0.115263134	0.090756	0.002066	-0.02759	-0.09396
ZNF740	-0.035131539	-0.103640492	0.194426	-0.03349	0.066758	-0.26176
ZNF746	0.007021123	-0.002413591	-0.19881	0.007692	-0.03839	0.041285
ZNF747	-0.012599539	-0.075576706	0.018214	-0.00725	-0.03775	0.018328
ZNF749	-0.028669025	0.05759152	0.051987	-0.00485	0.017313	-0.07372
ZNF750	-0.162403715	-0.091069474	-0.00338	0.039576	0.079336	-0.08003
ZNF75A	0.033010979	0.05561782	0.220155	-0.05689	0.141839	-0.37308

ZNF75D	-0.010005692	0.126033561	0.170988	-0.01619	0.086305	-0.15694
ZNF76	0.01940426	-0.042182524	0.249924	0.006093	-0.11215	-0.29366
ZNF763	-0.028358157	0.071176193	-0.08246	-0.1068	0.31172	-0.11973
ZNF764	0.010277008	0.099820764	0.118016	0.030657	-0.04708	-0.11316
ZNF765	0.041750623	0.141585844	0.02416	-0.03648	0.032453	-0.12084
ZNF766	0.030875301	0.062952606	0.233398	0.00669	-0.00686	-0.20375
ZNF767P	0.012548027	0.00300996	0.059985	-0.0243	0.083042	-0.21034
ZNF77	-0.006476794	0.110919225	0.10218	0.015089	-0.01597	-0.06297
ZNF770	0.014128772	0.044712612	0.088227	0.006627	-0.00564	0.152613
ZNF771	-0.019963197	-0.074743476	-0.09955	0.041736	-0.06471	-0.04582
ZNF775	-0.034939923	-0.062880834	-0.19492	0.04009	-0.09359	0.100619
ZNF776	0.02420647	0.037846686	0.00177	-0.04818	0.104454	-0.25686
ZNF777	0.009544199	-0.05799303	-0.00461	0.036614	-0.08165	0.057118
ZNF778	-0.025572978	0.045111236	-0.06434	-0.00382	0.017093	-0.0485
ZNF780A	-0.012306107	0.083113328	0.001817	-0.00526	0.033473	0.076861
ZNF780B	0.018246959	0.100881072	0.137072	-0.00978	0.092665	-0.05295
ZNF781	-0.057213817	0.085079311	0.25332	-0.06288	0.193045	-0.19898
ZNF782	-0.014577439	0.061595746	0.11353	0.003314	0.006796	-0.12668
ZNF783	0.018078493	-0.031719099	0.18775	-0.07177	0.123783	-0.19527
ZNF784	-0.007175965	-0.10604107	-0.00528	0.000962	-0.01317	0.125569
ZNF785	-0.022837408	0.027094635	0.044761	-0.08405	0.189157	0.053282
ZNF786	0.013653905	0.144431699	0.198304	0.014548	0.015602	-0.13085
ZNF787	-0.014333027	-0.060378136	-0.17588	0.049005	-0.12933	0.214647
ZNF789	0.033302745	-0.049320145	0.087863	0.116274	-0.14135	-0.21406
ZNF79	0.00791032	0.033196546	0.02395	0.082634	-0.21985	-0.02767
ZNF790-AS	-0.021523383	0.004262914	0.059396	-0.01648	0.069342	-0.29062
ZNF791	0.05120636	0.054246229	0.129971	-0.01391	0.03677	-0.13106
ZNF792	0.004536928	0.079151049	0.218129	-0.03667	0.127553	-0.15759
ZNF793	-0.013886195	0.048157046	0.123953	-0.02837	0.098362	-0.11281
ZNF8	-0.001566901	-0.029768629	0.120873	0.013819	-0.00586	-0.07406
ZNF80	-0.061468411	-0.064318324	0.119473	-0.06329	0.146502	-0.06602
ZNF800	0.005706149	0.114575701	0.211142	-0.00452	0.013851	-0.16028
ZNF804A	-0.016001052	-0.097784845	-0.16064	-0.09584	0.283244	0.323785
ZNF804B	-0.058380616	-0.052110621	0.076609	0.004585	-0.02054	0.059424
ZNF805	-0.000964291	0.119060637	0.103119	-0.00523	0.021452	-0.08454
ZNF808	-0.015360967	0.167965071	-0.09494	-0.03904	0.134041	-0.10902
ZNF81	-0.035219496	0.027608651	-0.03699	0.013316	-0.11695	-0.0151
ZNF813	-0.001911149	0.10986159	0.111114	-0.03353	0.11822	-0.07106
ZNF814	-0.043590631	0.051739531	0.141003	-0.06149	0.210436	-0.21593
ZNF818P	-0.007910194	0.066116605	0.026195	-0.04555	0.137944	-0.2607
ZNF821	-0.021064625	-0.027771301	0.008673	-0.01353	-0.09376	-0.08879
ZNF823	-0.005930984	0.058814584	0.038605	-0.0318	0.103747	-0.16188
ZNF826P	-0.030975224	-0.035756961	0.00166	0.014723	-0.04996	-0.2113
ZNF827	-0.016000733	0.089839243	0.334293	-0.06017	0.119584	-0.37345
ZNF829	-0.017080532	0.133448078	0.168386	-0.02849	0.105055	-0.24254
ZNF83	0.005522348	0.134731554	0.154625	-0.03159	0.128295	-0.17699
ZNF830	0.040706508	0.144640502	0.181976	-0.00141	-0.04144	-0.4022
ZNF831	-0.000872408	0.217433879	0.361185	-0.03763	0.128414	-0.32477
ZNF835	-0.017876617	-0.040787511	-0.02151	-0.01172	-0.01369	-0.13741
ZNF836	0.004179781	0.065631277	0.245095	-0.03632	0.091405	-0.19074
ZNF837	-0.041621638	-0.092618648	-0.01167	0.048096	-0.10833	0.025013

ZNF839	-0.001433809	-0.069363023	0.084235	0.013336	-0.02371	-0.23314
ZNF84	0.011534034	0.03680528	0.20447	-0.07492	0.200429	-0.31977
ZNF843	-0.007390901	-0.035480669	0.00475	-0.00792	-0.06839	-0.14509
ZNF844	-0.017085288	0.071493359	-0.05455	-0.11018	0.290862	-0.09829
ZNF85	-0.003550204	0.001076347	0.010875	-0.0221	0.053263	-0.33618
ZNF850	-0.023404662	0.105148123	0.1042	-0.02895	0.104328	-0.29231
ZNF852	-0.024247679	0.035491696	0.094206	-0.05112	0.150276	-0.11469
ZNF853	-0.023016896	0.178874774	0.210344	-0.07434	0.221332	-0.26186
ZNF862	0.014787029	-0.063115505	0.304333	-0.00769	0.012397	-0.17764
ZNF865	-0.015569681	-0.057100379	0.042143	0.071208	-0.19335	0.046385
ZNF876P	-0.062727047	-0.157530641	-0.00942	0.002103	0.055863	0.025321
ZNF879	-0.006363676	0.124296444	0.06583	-0.09579	0.281463	-0.06981
ZNF880	-0.005661007	0.059998958	0.157958	-0.05636	0.159926	-0.19858
ZNF883	-0.022528308	0.054939852	0.342275	-0.03344	0.102968	-0.19108
ZNF891	-0.03756368	0.029808419	0.110624	-0.01165	0.051467	-0.09128
ZNF90	-0.047242057	-0.027612357	-0.02219	-0.0184	0.042473	-0.05528
ZNF91	-0.001426127	0.114655271	0.141907	-0.08327	0.294245	-0.23803
ZNF92	-0.024682462	0.003080839	-0.00919	0.003105	0.010298	-0.14014
ZNF93	-0.033450889	-0.074511001	0.043796	-0.01333	0.106422	-0.08104
ZNF99	-0.036510232	-0.070191617	-0.05232	-0.0211	0.03189	-0.06721
ZNFX1	0.043575618	0.01084722	-0.00043	0.001857	0.004775	-0.10083
ZNHIT1	0.045758112	0.057507732	-0.08341	0.054428	-0.15323	0.255811
ZNHIT2	0.006149779	-0.049159206	0.019321	-0.02266	-0.0259	0.095348
ZNHIT3	0.040664002	0.053928372	0.318559	0.042978	-0.13657	-0.01311
ZNHIT6	0.009529376	0.062363316	0.315143	0.016688	-0.03903	-0.25619
ZNRD1	-0.056586088	-0.027068647	0.005593	-0.00168	0.023246	-0.00838
ZNRD1-AS	0.031377449	0.017149926	0.066309	-0.0934	0.008227	0.010567
ZNRF1	0.006080441	-0.023125491	-0.1648	0.099062	-0.20346	0.257235
ZNRF2	0.004829849	0.01629187	-0.21548	-0.02267	0.056258	0.011243
ZNRF2P1	-0.043298968	-0.075514145	-0.01542	-0.01663	0.071118	-0.09629
ZNRF3	-0.026372149	-0.021626625	0.005647	-0.04973	0.149603	-0.20114
ZNRF4	-0.03405065	-0.096271257	-0.11545	0.000465	-0.03867	0.084118
ZP1	-0.018987034	-0.022526031	-0.02264	0.018093	-0.02814	-0.04891
ZP2	-0.039442615	-0.049139185	-0.15575	0.032228	-0.0542	0.076053
ZP4	-0.032275518	-0.052202633	0.060403	0.024211	-0.06374	0.007787
ZPBP	-0.031725946	-0.009993431	0.091613	-0.00316	0.033044	-0.22884
ZPBP2	-0.034570186	-0.042078492	-0.06993	-0.02681	0.103906	-0.02442
ZPLD1	-0.05061089	-0.094079569	0.05349	0.015962	-0.02828	-0.06517
ZPR1	0.026709784	0.035560276	-0.05387	0.060563	-0.19175	0.123927
ZRANB1	0.027156365	0.078850552	-0.03484	-0.00311	-0.01941	-0.32276
ZRANB2	0.053549176	0.071399621	0.24855	0.037698	-0.10007	-0.29863
ZRANB2-AS	-0.034398969	-0.028086462	-0.02883	-0.00567	0.051381	0.086006
ZRANB3	-0.011428234	0.041998188	0.118166	-0.06178	-0.01935	-0.21601
ZRSR2	0.044353683	0.00552998	0.05079	-0.02181	0.030608	0.100989
ZSCAN1	-0.026383531	-0.04156995	-0.00046	5.60E-05	-0.0098	0.092745
ZSCAN10	-0.042974706	-0.059374919	-0.07346	0.016542	-0.0351	0.12269
ZSCAN12	-0.032041073	0.086715106	-0.17787	0.006672	0.005689	-0.03527
ZSCAN12P	-0.02875297	-0.054447578	-0.0708	-0.01439	0.034806	0.133295
ZSCAN16	0.017178265	0.032365591	-0.06674	-0.06643	0.222297	-0.00512
ZSCAN16-A	-0.023225344	-0.02970147	0.112977	0.026402	-0.0541	-0.08017
ZSCAN18	0.001007223	0.029621891	0.249801	-0.12954	0.310296	-0.47294

ZSCAN2	-0.016016205		-0.099586806	0.080233	0.020054	-0.07805	-0.04901
ZSCAN20	-0.044794041		-0.034254821	0.114362	-0.06816	0.048136	-0.00732
ZSCAN21	0.028455671		0.109729347	-0.00817	0.024388	-0.08221	0.045844
ZSCAN22	-0.003441756		0.103324512	-0.0512	0.04389	-0.10322	-0.11753
ZSCAN23	-0.064221523		-0.05622521	0.096825	0.002666	0.013989	-0.05834
ZSCAN25	0.005964781		-0.025213333	0.327594	-0.00706	-0.01483	-0.11968
ZSCAN26	0.009383219		0.115087366	0.09007	-0.04484	0.159306	-0.09532
ZSCAN29	0.03468994		0.083490534	-0.02924	-0.00288	-0.01001	-0.10546
ZSCAN30	0.087903635		0.011714463	0.134977	-0.05288	0.009444	-0.17793
ZSCAN31	-0.035629525		0.044205281	0.048515	-0.01135	0.06927	-0.0319
ZSCAN32	0.007041032		0.056898395	-0.07881	0.008821	-0.05029	-0.15364
ZSCAN4	-0.074349299		0.013902836	0.006917	-0.0194	0.062559	0.032439
ZSCAN5A	-0.022391444		-0.028160996	-0.02791	0.027729	-0.04528	0.26366
ZSCAN9	-0.005946597		0.082098409	-0.31751	-0.05166	0.134831	0.164238
ZSWIM1	-0.010775178		-0.127275338	0.072626	-0.01317	-0.0407	-0.03768
ZSWIM2	-0.065996733		-0.095529654	0.149721	-0.00245	0.010436	-0.00174
ZSWIM3	-0.009155882		0.047994645	-0.13332	0.021759	-0.08326	-0.15933
ZSWIM4	-0.030759426		-0.021155997	0.004921	-0.00529	0.033024	0.086265
ZSWIM5	-0.019961145		-0.003909455	0.194687	-0.02791	0.070113	-0.14571
ZSWIM6	0.033872452		-0.050549383	-0.32623	########	0.037795	0.397988
ZSWIM7	0.026956108		-0.029149364	0.060995	-0.03689	0.120362	0.306361
ZSWIM8	0.028258012		0.02790734	-0.13067	-0.02104	0.061303	-0.00052
ZUFSP	0.022182361		0.103400249	-0.07734	0.057204	-0.12964	0.10955
ZW10	0.019644986		0.065890058	-0.06267	0.074114	-0.29056	-0.00488
ZWILCH	0.019526082		0.128149693	0.091789	0.126475	-0.34851	-0.16401
ZWINT	0.006051932		0.02352939	0.163988	0.104711	-0.31054	-0.07181
ZXDA	-0.006831121		0.068161644	0.036444	-0.04787	0.147255	-0.10781
ZXDB	-0.008149623		0.038993797	0.208293	-0.02817	0.110618	-0.15
ZXDC	-0.010308599		-0.065146436	-0.06501	-0.04129	0.124028	0.039324
ZYG11A	-0.029990103		0.020027819	-0.12038	-0.00066	0.011384	0.041814
ZYG11B	0.022190444		-0.062881109	-0.09285	-0.01244	0.074649	0.128152
ZYX	0.023207982		-0.068624608	-0.12414	-0.02866	0.096716	0.345449
ZZEF1	-0.041434201		-0.003842331	-0.03181	-0.01591	0.003592	-0.09386
ZZZ3	0.022279298		-0.015470037	0.307442	-0.01786	-0.02039	-0.32921

GS.B cel	GS.CD4 T	GS.Eosin	GS.Myelo	GS.Neutr	GS.Plasma	GS.Natura	GS.Dendr	p.GS.Imma	p.GS.Natu
-0.08824	-0.02037	-0.18863	0.069787	-0.24897	0.264858	-0.00554	0.024972	0.958656	0.004559
0.011051	-0.08485	-0.15327	-0.05496	-0.01175	0.238708	0.018121	0.037558	0.914886	0.117192
0.060794	-0.02193	0.17454	-0.09147	-0.08862	0.031907	0.074024	-0.03118	0.478462	0.668269
-0.04771	-0.02134	0.195483	0.099264	0.178029	-0.05772	-0.00814	-0.00115	0.83328	0.606269
-0.2106	0.049744	-0.17998	-0.03543	-0.10204	0.049406	0.073516	0.005618	0.977023	0.162072
-0.08482	-0.02296	0.194338	-0.13797	0.142267	0.019121	0.040426	-0.03531	0.439889	0.793455
0.008361	-0.03377	0.21123	-0.08638	0.154539	-0.00584	0.038821	-0.00452	0.744692	0.726214
0.116349	-0.06565	0.222812	-0.00281	0.306955	-0.08928	-0.01934	-0.00115	0.86278	0.348153
0.095735	-0.06337	-0.0436	-0.03029	-0.11621	-0.03137	0.032387	0.000998	0.553596	0.598496
0.024366	-0.00313	0.145562	-0.10674	0.165249	-0.05485	0.028133	0.000688	0.650027	0.288414
0.159647	0.002226	0.212373	0.023582	0.067128	-0.04541	-0.07521	-0.01273	0.907194	0.246468
-0.24428	-0.01462	0.171098	0.025866	-0.22977	0.327647	-0.05164	0.01636	0.816793	0.729457
0.030391	0.002638	-0.03416	0.0758	0.137071	0.076569	0.012682	-0.04752	0.47606	0.516352
0.127636	-0.08635	0.238292	-0.05298	0.143077	-0.10004	0.075353	-0.01893	0.59021	0.999572
-0.09159	-0.05711	0.054197	0.161414	-0.03503	0.151067	0.032172	-0.07572	0.364067	0.789777
0.005087	0.035596	0.506959	-0.03514	0.096326	-0.14147	0.043425	-0.03038	0.615678	0.197921
-0.09291	-0.04201	0.09825	0.081394	0.19866	-0.02801	0.018925	-0.01332	0.581609	0.640994
0.0256	-0.01816	0.239467	0.008148	-0.25273	-0.09167	-0.03322	0.011119	0.781669	0.956744
-0.02762	-0.04749	0.137539	-0.03772	0.122459	0.049529	0.045454	-0.00665	0.74247	0.888056
-0.12295	0.15178	0.133488	-0.13213	-0.00049	-0.14848	0.010904	-0.00413	0.619529	0.246051
-0.17286	0.009726	-0.00695	0.0207	-0.0269	-0.01971	-0.02703	0.026793	0.955214	0.384483
0.018581	-0.01542	0.079494	0.002517	0.00885	-0.08149	-0.04354	-0.01628	0.5112	0.819922
-0.10383	-0.05529	-0.09037	0.090072	0.073612	0.092539	0.001284	-0.02434	0.619025	0.758924
0.022674	0.017337	-0.28306	0.110175	-0.34608	0.115965	-0.05605	-0.00485	0.574709	0.937049
0.115455	0.057975	-0.05894	0.029261	-0.10613	0.024389	-0.06039	0.008822	0.72498	0.462241
0.206401	0.088719	-0.2904	0.03408	-0.23624	0.109829	-0.06994	0.013544	0.674432	0.720642
0.195086	0.150678	-0.29114	0.016525	-0.50479	-0.01191	-0.05974	0.016006	0.856983	0.526024
0.216484	0.0904	-0.13548	-0.14672	-0.37192	-0.10521	-0.04391	-0.02001	0.638458	0.05634
0.385238	0.034023	-0.18997	-0.04996	-0.15363	-0.01356	0.026872	-0.01479	0.726229	0.472372
0.078675	-0.01492	-0.0614	-0.11809	0.067113	0.042969	-0.06538	-0.01591	0.376703	0.628736
-0.10249	-0.04236	0.064384	-0.07015	0.385069	-0.18236	-0.03036	-0.00587	0.997695	0.730807
-0.03077	0.101359	-0.07511	-0.04264	-0.20508	0.040091	-0.04089	0.022588	0.969071	0.919569
-0.15912	-0.09923	0.162421	-0.05849	0.301561	-0.16414	-0.01534	0.016742	0.705911	0.730663
0.167488	-0.1258	0.399068	-0.15207	0.326189	-0.07387	0.014387	-0.01441	0.923313	0.100877
0.335203	0.09235	-0.26782	-0.0012	-0.18561	-0.02789	0.050688	0.007079	0.869192	0.836066
-0.02902	-0.00055	-0.10456	0.136389	-0.01775	0.118817	-0.02544	-0.04451	0.571471	0.659787
-0.05808	-0.0558	-0.08986	0.049544	-0.07592	0.08084	0.012353	-0.09272	0.375039	0.446544
0.020956	0.127066	0.034643	-0.02659	0.010084	-0.13737	0.05846	-0.0164	0.736357	0.400501
0.011055	-0.07662	0.003955	0.04131	0.084014	0.017744	-0.02731	0.02931	0.844103	0.376014
-0.01612	0.00951	-0.11265	-0.02222	0.14203	-0.07102	-0.05548	0.025688	0.951302	0.998357
0.02471	0.005496	0.366708	-0.0748	0.137461	-0.08399	0.015266	-0.01573	0.570479	0.529844
0.012995	0.055455	0.135893	-0.14666	0.052622	0.064565	0.080422	0.010299	0.906203	0.330134
-0.0448	0.006665	-0.1121	-0.01346	-0.0661	0.279149	0.028245	-0.00847	0.783372	0.52358
-0.02686	-0.06139	0.238857	-0.04437	0.269661	0.106625	0.037325	-0.00626	0.853288	0.123536
0.036659	0.011676	0.18876	0.02485	0.181119	-0.10615	0.036356	0.044661	0.397132	0.270695
-0.12191	0.007971	0.006794	0.053757	0.015261	0.169933	-0.05105	-0.07899	0.602098	0.71604
0.054264	-0.15024	-0.1406	-0.09546	-0.12052	-0.09004	0.164915	0.009502	0.980284	0.000427
-0.18344	0.02953	0.267112	0.066653	-0.4424	0.144985	-0.03652	0.008564	0.684859	0.488063

-0.01325	-0.0444	-0.0288	0.145737	-0.01373	0.040243	0.056545	-0.04076	0.375059	0.299108
0.709889	-0.06708	-0.11678	0.13425	-0.0881	-0.10933	0.007568	-0.00433	0.894324	0.286989
-0.07438	0.108486	0.600539	0.032175	0.08733	-0.084	-0.01622	-0.07315	0.353184	0.123365
0.039535	-0.0499	-0.07981	-0.06029	0.202973	0.120188	0.001661	0.021289	0.87731	0.378966
0.204462	0.037246	-0.28382	0.104287	-0.59232	0.092309	-0.03103	0.002498	0.698418	0.749201
-0.08626	0.107557	-0.1154	0.030155	-0.00803	0.049079	-0.08236	0.02589	0.744492	0.799663
-0.01774	-0.09433	-0.02895	0.069014	0.126173	0.01478	0.014763	0.005767	0.180169	0.56425
0.004432	0.050586	0.460271	-0.02198	-0.15786	-0.07674	-0.00414	-0.00101	0.739964	0.074884
-0.20969	0.063397	0.232722	-0.00632	-0.27551	-0.10398	-0.01367	0.015255	0.793692	0.48151
0.046775	0.047408	0.411424	-0.17005	0.17649	-0.07566	-0.01894	-0.02623	0.580987	0.497202
-0.10161	-0.00348	0.004582	0.095129	0.097	0.134867	-0.03922	-0.04542	0.402429	0.530179
0.004589	-0.01108	0.046314	0.008927	0.069457	-0.04874	0.024786	-0.02538	0.925256	0.208044
-0.05555	-0.0637	-0.11736	-0.02488	0.167111	0.211611	0.008337	-0.00125	0.712426	0.931821
-0.05622	-0.05548	0.05986	-0.03864	0.058717	-0.13825	-0.0025	0.042124	0.690842	0.224753
-0.03363	0.049379	-0.06233	0.032589	-0.11811	-0.02708	0.075905	0.002144	0.830809	0.985054
-0.06555	-0.04545	0.183372	-0.04424	0.284851	-0.20506	-0.02256	0.009008	0.872704	0.968708
-0.01503	0.010812	0.092312	-0.0369	0.009436	0.034408	0.028113	-0.04939	0.432359	0.551054
0.025143	-0.00939	0.07565	0.07102	0.15701	-0.02325	0.007094	0.002594	0.556635	0.088672
-0.0873	-0.01473	0.1739	-0.04884	0.204099	-0.14139	0.07714	0.014621	0.971733	0.335772
-0.08031	-0.21158	0.174043	0.087446	0.022462	0.018507	0.008522	0.016981	0.875864	0.10628
-0.09609	0.112453	0.007967	-0.04511	-0.05579	-0.00777	0.036751	-0.00891	0.881137	0.276669
-0.02564	0.136522	0.03085	-0.14512	-0.28673	-0.12913	-0.05002	-0.01089	0.881161	0.162662
0.148022	0.038564	0.042713	-0.14009	-0.23594	0.107635	-0.02713	0.00394	0.836088	0.845321
0.153201	0.094406	-0.39153	0.084409	-0.4434	0.127453	-0.01214	0.001811	0.770536	0.733154
0.042027	0.006754	-0.20451	0.066174	-0.20717	0.045241	-0.04924	-0.00851	0.491419	0.057364
0.085693	0.07295	-0.25218	0.076392	-0.22177	0.004655	0.112587	0.137885	0.993061	0.617615
0.153206	-0.06738	-0.03586	0.059169	-0.00509	-0.05256	-0.02608	-0.00674	0.746032	0.941633
0.07491	-0.09696	0.190606	-0.11861	0.284252	-0.01895	0.143157	0.006076	0.76049	0.314567
-0.09615	-0.13297	0.214713	-0.04798	0.100116	-0.0498	0.143536	-0.00059	0.815281	0.579877
-0.05465	0.010508	0.304769	-0.07491	0.296985	-0.09614	-0.01603	-0.00269	0.710719	0.336849
-0.00455	0.017856	0.161056	-0.02177	0.131659	0.004816	0.06298	-0.02596	0.601592	0.628011
0.047411	-0.03607	-0.118	0.112332	0.144125	-0.16496	-0.03337	-0.00577	0.689561	0.431157
-0.05679	0.01595	0.25764	0.013095	-0.02578	-0.02682	-0.05078	-0.02016	0.393018	0.771084
0.290864	-0.01999	0.099003	0.007557	-0.44732	0.142043	0.004501	-0.00748	0.742722	0.684735
-0.02027	-0.05669	0.055861	0.040455	0.171038	-0.12728	-0.09032	-0.0038	0.083573	0.479682
-0.12463	0.00971	-0.1408	0.007302	-0.13361	0.072345	-0.05733	0.021535	0.822975	0.225668
-0.08932	-0.01495	-0.08025	-0.02935	0.183076	0.003597	0.00688	0.008647	0.977278	0.579037
0.12621	0.129261	-0.38365	0.108095	-0.42895	0.098021	-0.06262	0.004722	0.764296	0.613022
0.002793	-0.02623	-0.15726	0.016516	-0.20127	0.420021	-0.03602	-0.0011	0.891026	0.263894
-0.16128	-0.17871	0.082097	-0.01766	0.379808	0.007941	########	0.003506	0.684206	0.689502
-0.04893	0.007319	-0.24903	0.124026	0.103435	0.019031	0.000883	0.002999	0.708375	0.897371
-0.02208	0.032942	-0.17634	-0.06816	-0.20182	-0.038	-0.03058	-0.00816	0.715857	0.000424
0.105999	0.003259	0.321846	-0.15394	0.20093	-0.12733	-0.04588	-0.00088	0.889441	0.185344
-0.11425	-0.0053	0.187846	-0.20677	0.192209	0.072417	0.000421	-0.00206	0.886599	0.407954
-0.34512	-0.1094	0.442513	-0.01148	0.333322	-0.08708	-0.05291	0.000144	0.639464	0.108998
-0.23925	-0.02304	-0.02873	-0.07574	0.397305	-0.09124	0.009766	-0.00844	0.547891	0.494852
-0.1061	-0.09747	0.27864	-0.0752	0.422609	-0.14442	-0.02085	0.00354	0.870516	0.432894
-0.29022	-0.14467	0.073262	-0.10777	0.538996	-0.13201	0.045187	0.013388	0.922289	0.836713
0.002744	-0.0768	-0.24808	0.171867	-0.23336	0.213052	0.00421	0.028035	0.941296	0.894725
-0.07907	-0.02151	-0.1868	0.032563	-0.11929	0.008943	0.008985	0.016304	0.775704	0.873088
0.131116	-0.08089	0.166823	-0.14611	0.110881	0.093753	-0.05226	0.007478	0.352838	0.058508

0.046589	-0.0066	-0.18722	-0.05322	-0.06365	0.141593	0.037665	0.05571	0.434418	0.886903
-0.12508	-0.11901	-0.0949	0.121645	-0.0102	-0.24094	0.028858	-0.01137	0.68963	0.048705
-0.03723	0.042517	0.153865	-0.08959	0.122787	0.052251	0.034009	-0.12109	0.231599	0.609821
-0.05488	-0.00956	-0.21856	0.104931	-0.22722	0.106192	-0.03084	-0.00033	0.7408	0.319517
0.071889	0.030027	-0.11812	0.031009	-0.02895	-0.09649	-0.00683	0.017154	0.84473	0.940649
0.274897	0.293019	-0.19146	-0.08542	-0.14693	-0.18321	-0.0889	0.003851	0.909985	0.346768
0.076893	-0.03355	0.417428	-0.05415	0.297458	-0.08079	-0.05026	-0.04184	0.613471	0.257246
-0.08012	-0.00195	-0.08373	0.197931	0.028586	-0.01058	-0.01872	0.009909	0.887128	0.309337
0.030536	0.0917	0.107354	-0.03564	0.215325	-0.05357	-0.04058	0.009665	0.624293	0.293358
-0.12729	-0.07108	0.163997	0.029571	0.288948	-0.1382	-0.05742	0.010496	0.73972	0.809337
0.078499	0.046133	0.005808	0.095859	-0.04479	0.026393	0.02447	-0.06748	0.317069	0.876999
-0.21911	0.15336	-0.26637	0.029653	-0.49573	0.040459	-0.02951	-0.01178	0.550217	0.399168
0.174275	-0.02718	-0.03915	-0.09878	0.021581	-0.12874	0.002903	-0.01791	0.556982	0.208522
-0.09958	-0.01415	0.190347	0.01945	0.343184	-0.05505	-0.05646	0.002996	0.593795	0.773743
-0.03688	-0.02225	0.210454	0.019496	0.107049	0.03818	-0.00332	0.000123	0.89642	0.459605
0.020976	0.081129	-0.03369	0.024601	-0.06262	0.218487	0.066539	-0.14112	0.027658	0.340093
0.100666	-0.00811	0.333744	-0.07026	0.158619	-0.13762	0.020852	-0.0054	0.715604	0.458666
-0.0201	-0.13394	0.421176	0.049187	-0.05183	-0.01665	0.067572	-0.02115	0.658166	0.606063
-0.04889	0.145081	0.032072	0.046735	-0.02189	0.008156	0.00948	-0.04222	0.500733	0.738547
0.034502	0.053669	-0.0369	-0.017	0.111332	0.062892	-0.017	-0.00133	0.63369	0.457196
0.338675	0.152043	-0.22872	0.138826	-0.36072	-0.02061	-0.05018	-0.00105	0.820273	0.161254
-0.04078	0.023475	-0.00413	-0.02847	0.32145	-0.02074	-0.04649	0.01412	0.704581	0.653601
-0.02345	-0.00153	0.240605	-0.015	0.247481	-0.14285	-0.00586	-0.00412	0.69414	0.414188
0.0423	0.050367	0.018219	-0.03663	-0.10517	0.019117	-0.01452	-0.00636	0.801343	0.788626
-0.03063	0.019033	0.088335	-0.00082	0.089032	-0.02673	0.004502	0.014461	0.796151	0.13333
0.040478	-0.06686	-0.0095	0.020517	0.263169	0.072594	0.04393	0.026379	0.969237	0.118776
0.015926	0.033665	-0.01895	-0.06285	-0.05848	-0.04889	0.017373	-0.00739	0.651033	0.532461
-0.04386	-0.02449	0.111825	0.033646	-0.05976	-0.09909	0.00138	0.004104	0.886397	0.953517
0.098608	0.020315	0.167493	-0.0463	0.151837	-0.20192	0.005739	0.001026	0.717038	0.612182
-0.02815	-0.02507	0.297685	-0.16174	0.238775	-0.03548	-0.00364	-0.02257	0.667484	0.810573
0.050311	0.042636	0.4215	-0.17558	0.142414	-0.19406	0.013966	-0.02331	0.759041	0.634233
-0.04946	-0.05585	0.017834	0.114694	-0.02491	0.044581	0.038151	-0.01832	0.655616	0.741547
0.107721	-0.11169	0.237854	-0.00741	0.071541	-0.07432	0.068236	-0.00469	0.702417	0.20269
-0.04834	-0.03208	0.177813	-0.00688	-0.02406	-0.04143	0.038827	-0.01091	0.710458	0.825946
0.119246	0.036969	0.118724	-0.07868	0.080696	-0.11246	0.042972	-0.00034	0.756336	0.633045
0.323069	0.274534	-0.13512	-0.11954	-0.25398	-0.02326	-0.01735	0.006496	0.951764	0.354914
0.167178	0.030102	-0.07021	0.053592	0.001206	0.003875	-0.00248	-0.02656	0.593919	0.682822
-0.06977	-0.1098	-0.08446	0.110776	-0.0463	0.0698	0.000525	0.027628	0.876273	0.743126
-0.08625	0.033662	0.007033	-0.00177	0.014389	-0.05653	0.003035	-0.02886	0.630426	0.179822
-0.17758	-0.08641	0.07855	-0.07746	0.137802	0.067105	0.100361	0.063032	0.562164	0.649457
-0.076	-0.10089	0.103904	0.180693	0.044979	-0.05744	0.067702	0.012424	0.720694	0.377661
-0.01236	0.050092	0.150382	-0.01268	-0.02171	-0.05288	-0.03932	0.003781	0.763373	0.711515
-0.11825	-0.10061	-0.12286	0.062118	-0.14713	0.36072	-0.01709	0.005536	0.872824	0.981068
0.036995	0.007784	0.378553	-0.06963	0.295994	-0.08419	0.029625	-0.01184	0.658072	0.699647
-0.12584	0.027596	0.36066	0.009986	0.061938	0.006815	-0.00075	-0.00438	0.643517	0.246706
0.151495	0.155795	0.134165	-0.07689	0.080072	-0.09924	0.030486	-0.01302	0.623143	0.707777
0.002322	0.010463	-0.06956	-0.14802	0.077844	0.09688	0.04859	0.00278	0.791302	0.549229
0.016137	-0.01992	0.066228	-0.10351	0.320776	-0.20624	-0.03956	0.012277	0.780684	0.557281
0.039164	-0.08516	-0.08707	0.015163	0.064962	0.109794	0.04753	0.018726	0.669966	0.932553
-0.03265	0.010536	-0.26459	0.194681	-0.13368	0.148013	-0.03184	0.008003	0.794096	0.144293
0.055563	0.009961	0.503283	-0.12144	0.259945	-0.17647	-0.03806	-0.01617	0.71951	0.626855

-0.04104	-0.0601	0.259357	-0.04131	0.15031	-0.13117	0.004058	-0.00765	0.714565	0.434841
0.046383	-0.00847	-0.19369	0.270596	-0.28699	0.070018	-0.03825	0.003606	0.766636	0.401536
0.692652	-0.03502	0.064469	0.005599	0.008073	-0.11202	-0.01365	-0.00154	0.817949	0.249747
-0.05346	-0.03587	0.058934	0.057801	0.128436	0.000672	-0.04622	0.001147	0.738812	0.476795
0.063825	0.021929	0.18454	-0.00335	-0.02612	0.033302	0.001049	-0.03715	0.541297	0.378068
-0.00068	-0.05483	0.399102	-0.06151	0.137881	-0.06608	0.035224	-0.00665	0.736638	0.502616
0.150819	-0.01319	-0.01296	-0.12459	0.207563	0.058198	0.051762	-0.00686	0.686384	0.382546
-0.02247	-0.08313	-0.09935	0.030536	0.079868	0.190063	0.070723	-0.07011	0.313038	0.378208
-0.13532	0.063416	0.015674	0.058773	-0.02363	0.03175	-0.07305	-0.00166	0.758373	0.515348
-0.04312	-0.00937	0.18312	-0.077	0.069094	-0.06812	0.027196	-0.00299	0.683704	0.157691
-0.19105	-0.0075	-0.01504	0.071039	0.129008	0.02543	0.011158	0.009204	0.96901	0.69104
0.248697	-0.05116	0.274087	-0.11069	0.252845	-0.13713	-0.0326	-0.01078	0.958409	0.612978
0.071951	-0.01691	-0.23627	0.087544	-0.00834	-0.07184	-0.00751	0.025443	0.934134	0.769512
-0.02677	-0.01811	-0.09632	0.228472	0.094976	0.10749	0.006306	-0.04516	0.509273	0.552603
-0.0277	-0.04202	0.075907	0.079087	0.105829	0.033984	-0.03284	-0.0006	0.64898	0.896019
-0.10089	0.175758	0.058082	-0.01096	0.124014	-0.19876	-0.02462	0.014801	0.855738	0.764806
0.050479	0.112724	-0.12173	0.036577	-0.02618	-0.01164	-0.01042	0.005523	0.767762	0.379022
0.033929	-0.03666	-0.19869	0.127486	-0.06599	0.084438	-0.02977	0.016328	0.680585	0.214179
0.202288	0.025237	0.061638	0.008263	-0.00569	-0.00726	0.002727	-0.04021	0.514333	0.519738
-0.0787	-0.06178	0.10517	-0.0036	-0.00759	0.141678	0.116661	-0.0493	0.494898	0.416826
0.119218	0.031564	-0.00241	-0.01222	-0.0943	0.050217	-0.04252	0.002824	0.829394	0.969338
0.029226	0.020618	0.297624	-0.07479	0.189876	-0.14774	0.005911	-0.00885	0.744885	0.465057
0.088016	0.038935	-0.19004	-0.10755	0.016711	0.101537	-0.01853	0.007645	0.718156	0.812273
0.100748	-0.14483	-0.00098	0.048482	0.022638	0.210274	-0.03641	0.005136	0.953083	0.206053
-0.06234	-0.03131	-0.00627	0.228745	0.035294	-0.04099	0.004684	0.004457	0.666332	0.536953
-0.10656	-0.03139	-0.05632	0.127866	0.2632	-0.06845	-0.0052	0.017536	0.578033	0.099833
-0.11613	-0.09972	-0.07218	-0.07016	0.074801	0.185834	0.120876	-0.00143	0.86414	0.554705
0.061014	0.031381	-0.14269	0.104005	-0.1593	0.126587	0.001821	-0.03161	0.622754	0.315191
0.873824	-0.11361	-0.11932	-0.04364	-0.1661	-0.06855	-0.00798	0.001282	0.950819	0.439832
-0.05554	-0.032	0.014292	0.028426	0.02286	0.031881	0.045818	-0.03959	0.497349	0.894939
-0.01576	-0.05832	-0.10055	-0.06352	-0.01518	0.185334	-0.00141	-0.00039	0.745415	0.950025
0.19223	0.027788	-0.03917	-0.10191	-0.05032	-0.17805	0.13448	0.015588	0.956501	0.515736
-0.02263	-0.0943	-0.15026	0.036121	-0.10728	0.13125	0.035197	-0.01517	0.60086	0.780254
0.122966	0.022883	-0.02482	0.096546	0.073051	0.001523	0.001625	-0.04722	0.499809	0.805204
0.035781	0.053867	-0.02973	-0.00442	0.066897	-0.10787	0.09908	-0.02102	0.695818	0.770387
0.08107	-0.04912	-0.12203	0.064572	0.085612	-0.05641	-0.01926	0.036462	0.966764	0.875792
-0.02264	-0.03754	0.034175	0.012091	-0.05916	0.043821	0.077011	-0.03184	0.650603	0.888556
0.088957	0.111171	0.274557	-0.15739	0.047997	-0.14429	0.00879	-0.01493	0.799153	0.69364
-0.07306	-0.16792	0.289487	0.125633	0.358621	0.043885	-0.03006	-0.00541	0.986829	0.241186
-0.14341	0.009926	0.300958	-0.00064	0.135929	-0.08589	-0.00967	-0.00526	0.720997	0.883771
-0.05506	0.0767	-0.18561	0.216785	-0.11239	0.047684	########	-0.0228	0.593177	0.546082
-0.12581	0.082336	0.094383	-0.0363	-0.24167	-0.07363	-0.00427	0.011738	0.792211	0.432078
-0.04118	0.023091	0.215513	-0.12777	0.134051	-0.05615	-0.03608	0.012545	0.62357	0.659856
0.017951	-0.02461	0.341335	-0.02727	0.124249	-0.09161	0.001345	-0.01013	0.735888	0.586129
0.086728	-0.08142	0.07606	0.011341	-0.00249	0.082566	0.029349	-0.03701	0.540816	0.751994
0.022613	-0.03432	-0.02896	0.087171	0.101426	-0.05405	0.028299	-0.00608	0.675184	0.435772
-0.09853	-0.03121	-0.23032	0.105858	-0.09496	0.20332	-0.00914	-0.04398	0.458583	0.794361
0.113702	-0.14794	-0.07463	0.046932	-0.03735	0.096878	0.043779	-0.04181	0.522695	0.849216
-0.0437	-0.03353	-0.04688	0.233565	0.094429	0.100032	0.013215	-0.05593	0.425963	0.773536
0.042731	0.021628	-0.05593	0.021856	0.075322	0.057564	0.018764	-0.04388	0.538764	0.903305
-0.02687	-0.14763	0.003204	0.130528	-0.09494	-0.0063	0.103019	-0.03574	0.537453	0.511494

-0.01324	-0.00308	0.402479	-0.06042	0.210349	-0.1068	0.017289	-0.00218	0.595404	0.305195
0.047809	-0.04794	-0.0621	0.024998	0.087116	-0.05699	0.084202	0.008002	0.947181	0.776218
0.055573	0.00336	0.002841	-0.02846	0.097788	-0.06951	0.016071	-0.0038	0.681913	0.832341
-0.18569	-0.08475	0.207544	0.146859	0.160008	0.033414	-0.05593	-0.01005	0.557575	0.341133
-0.03215	0.001558	0.009551	0.030479	-0.01694	-0.10416	0.067825	0.032729	0.87549	0.596673
-0.04852	0.031926	-0.06478	0.029773	-0.11898	0.040809	-0.02926	0.009315	0.975575	0.685613
-0.05491	0.046564	0.585104	-0.03276	-0.05755	-0.02418	-0.0219	0.012343	0.996931	0.122522
0.063889	0.016779	-0.14973	-0.03954	-0.13187	0.050216	0.030445	0.013284	0.768589	0.345388
-0.10322	0.003018	-0.07212	-0.04137	-0.19528	0.084034	-0.00752	0.013903	0.830777	0.509558
-0.03159	-0.00701	-0.40172	0.187164	-0.39979	0.132135	-0.0062	0.026448	0.746176	0.547601
0.06175	-0.0159	-0.047	-0.09905	0.008575	0.004708	0.092363	-0.04609	0.409489	0.77499
0.263028	0.005498	-0.08301	0.08224	-0.55121	0.116263	-0.00318	0.000607	0.748015	0.650671
-0.11311	-0.00457	-0.0271	-0.0043	0.152604	0.017214	-0.02861	0.014748	0.884195	0.648959
0.043165	0.086669	-0.14187	-0.04084	-0.18172	0.046775	-0.02772	0.012396	0.978805	0.442181
-0.22518	-0.20824	0.261061	-0.02015	-0.02266	0.101903	0.019298	-0.00135	0.426175	0.854289
-0.02889	-0.10057	0.237373	0.087309	0.148092	-0.03983	0.024455	0.002142	0.471616	0.31968
0.018424	0.100389	0.092581	-0.07874	0.07466	-0.00298	-0.01123	-0.03115	0.897917	0.924211
-0.04945	-0.11037	0.19349	-0.13156	0.382207	-0.09312	-0.00453	0.002074	0.771188	0.97328
-0.10016	-0.14288	-0.03652	0.026824	-0.0986	0.003918	0.086813	0.016645	0.928242	0.166511
0.197424	0.03686	0.311205	-0.084	0.048033	-0.04982	-0.02082	-0.06172	0.397393	0.616504
0.098105	0.106413	-0.10901	0.067618	-0.18997	0.054594	-0.04893	-0.00286	0.709549	0.361327
0.503623	-0.08276	0.104892	-0.14514	-0.06556	-0.16033	-0.0102	-0.00255	0.619064	0.479596
0.246824	0.061985	0.083704	-0.09587	0.102081	-0.24704	-0.04686	0.007779	0.93725	0.293734
-0.20071	-0.0813	-0.21741	0.008883	-0.1916	0.100945	-0.03582	0.014188	0.711613	0.01322
0.016915	0.053357	-0.41055	0.199797	-0.20951	0.073925	-0.03019	-0.00091	0.736111	0.727781
-0.00359	-0.01427	0.380809	0.10311	-0.19899	0.120592	-0.04901	-0.00405	0.875921	0.106426
0.175785	0.034946	-0.21442	0.047649	0.043949	-0.0947	-0.04624	-0.0005	0.607905	0.254554
-0.03725	0.1449	0.116675	0.011264	0.202467	-0.07981	-0.19132	########	0.817563	0.206676
-0.02338	-0.02039	0.296987	0.053868	0.12581	-0.02204	0.027343	-0.0386	0.433764	0.502039
-0.12921	-0.01975	0.041735	0.07956	0.071205	0.072443	-0.00113	0.00352	0.69578	0.125633
-0.28711	-0.07363	0.071996	0.237961	-0.20965	-0.04274	-0.02239	0.009896	0.93102	0.322501
-0.0586	0.037168	-0.0038	0.076931	0.100218	-0.03238	0.04745	0.034848	0.798446	0.072546
0.100236	0.018508	0.117047	-0.11181	0.222593	-0.01742	-0.06913	-0.0096	0.651097	0.913842
0.041206	-0.07353	0.381037	0.02944	0.233216	-0.17216	0.021809	-0.0058	0.63912	0.456962
-0.01092	-0.10048	0.309467	-0.06091	0.139939	0.029797	0.064188	0.012837	0.862679	0.424737
0.02944	0.105497	-0.04491	-0.07058	0.055645	-0.20854	-0.01364	-0.00087	0.891977	0.12295
0.071272	0.058494	0.131279	-0.10399	0.019376	-0.03168	-0.01218	0.031217	0.779289	0.592666
0.020703	-0.07644	0.072184	0.0091	0.023241	0.026776	-0.04659	-0.00836	0.463284	0.142908
-0.12889	-0.03657	0.001688	0.013351	-0.03724	0.196167	0.033328	-0.04547	0.464867	0.66702
0.05246	0.130732	-0.31947	0.035444	-0.29838	0.147432	-0.1007	0.012282	0.93009	0.825146
-0.08332	-0.15449	-0.19863	0.064328	-0.18162	-0.01311	0.043032	0.023791	0.915668	0.311808
0.157204	-0.09979	-0.42029	0.199303	-0.25068	0.082783	-0.03964	-0.00581	0.725076	0.74132
-0.1417	0.031912	0.38594	0.129257	0.121556	-0.09893	-0.00869	-0.00309	0.583204	0.256448
-0.01497	-0.01195	-0.01404	0.047329	-0.02847	0.034625	-0.02193	-0.02915	0.736096	0.824356
-0.08033	-0.02404	-0.33802	0.051438	-0.04738	-0.04104	-0.00078	0.011456	0.76007	0.6632
-0.09713	0.055156	-0.02163	-0.06562	-0.04418	-0.06811	-0.01238	0.011323	0.962177	0.000714
-0.11835	-0.12005	-0.19123	0.194264	0.050585	0.164196	0.070587	-0.03409	0.594054	0.502224
-0.14253	-0.00069	-0.19258	0.12131	-0.03552	0.222544	-0.01659	0.002104	0.948147	0.087565
-0.12585	-0.08989	0.064213	-0.05374	-0.06315	-0.00253	-0.04299	0.011844	0.93358	0.50343
-0.17934	-0.12387	0.046446	0.155068	0.010422	-0.03481	0.013344	0.003659	0.557039	0.699875
-0.31091	-0.12539	-0.24144	0.039262	0.386698	0.029009	0.04052	-0.01441	0.961251	0.585609

-0.1566	-0.03217	-0.0762	0.024061	-0.06908	-0.03524	0.002231	0.001973	0.81547	0.367093
-0.09344	-0.04104	-0.1666	0.095147	-0.14797	0.281275	-0.00095	0.027256	0.991972	0.421434
-0.12824	-0.02163	-0.16799	0.099074	0.099505	0.027055	0.046373	-0.03734	0.443081	0.759865
0.227633	0.093376	-0.15012	-0.05386	-0.47064	-0.04906	-0.02303	-0.00926	0.66574	0.121025
-0.17487	0.053126	-0.08368	0.035838	-0.00849	-0.25706	-0.04546	0.006998	0.77226	0.617449
0.358815	-0.07751	0.082837	-0.07376	-0.02465	0.082447	-0.01	0.000838	0.807772	0.168812
-0.08888	0.050676	0.032568	0.163573	-0.03438	-0.03449	0.064752	-0.00053	0.97667	0.263206
-0.29723	-0.10792	0.453693	0.134596	0.220729	-0.22263	0.038369	-0.00415	0.759691	0.163756
0.063692	0.002766	0.316089	-0.1478	0.264921	-0.08493	-0.05476	-0.00707	0.865102	0.480031
0.018133	-0.1072	0.167623	0.004312	0.05497	-0.02358	0.057481	0.000559	0.660232	0.575014
-0.02534	-0.08898	-0.08572	0.19569	-0.00191	-0.03528	-0.07801	0.020608	0.421648	0.125903
-0.04846	-0.00331	-0.22038	-0.08831	0.023029	0.086485	0.07417	0.018964	0.900084	0.249843
0.026029	-0.05881	0.406996	-0.14032	0.122718	-0.06678	0.030832	-0.01589	0.787835	0.690068
-0.07198	-0.03108	-0.15116	-0.01302	-0.09214	0.483915	0.008724	-0.00522	0.747765	0.352201
-0.05744	-0.02366	0.1775	0.078706	0.031948	-0.12313	-0.02806	-0.00371	0.701245	0.810973
0.072703	-0.08466	0.260114	0.218282	-0.13616	0.062474	-0.04046	-0.00734	0.931323	0.126013
0.15805	0.004792	0.061401	-0.07011	0.040663	0.150744	-0.06073	-0.08908	0.112844	0.507499
-0.25581	-0.10541	0.013486	-0.02508	0.504746	-0.0801	0.004015	0.005414	0.845086	0.486364
-0.01403	-0.11858	0.106376	-0.0817	-0.12104	0.278444	-0.03021	0.01611	0.728553	0.496354
-0.18502	-0.03915	0.394498	0.074815	0.270428	0.019376	-0.08081	0.025036	0.825818	0.257709
0.071629	-0.01817	-0.09123	0.282576	-0.39959	-0.01627	-0.0225	-0.00453	0.607884	0.228794
-0.1739	0.316737	-0.09444	-0.02566	-0.08401	-0.09304	0.032456	-0.03306	0.731687	0.263842
-0.04543	-0.07828	0.020547	0.150168	0.010082	0.121126	0.04645	-0.01156	0.50641	0.179703
-0.02937	0.085625	-0.02071	0.028598	-0.17028	0.102582	-0.01198	-0.05041	0.427683	0.61458
0.020596	0.057988	0.133434	0.071546	-0.10059	-0.02345	-0.00539	-0.03628	0.51412	0.266691
-0.17481	0.011325	0.39557	-0.1063	-0.10476	0.046271	0.022767	-0.00264	0.85723	0.456366
0.199896	-0.00908	0.213246	0.014625	-0.03888	-0.08752	0.004043	-0.0197	0.572741	0.472811
0.003201	-0.08762	0.181443	0.002249	0.172757	-0.0421	-0.06599	-0.00627	0.674227	0.841245
-0.29424	-0.01324	0.066533	0.216588	0.248259	-0.05509	-0.06675	-0.00099	0.837206	0.027888
0.025156	0.011753	0.371004	-0.21597	0.247241	-0.10032	0.018132	-0.02705	0.682809	0.749492
-0.06199	-0.04524	-0.10411	0.06146	0.050529	0.084841	0.025553	-0.01281	0.668967	0.97977
-0.24002	-0.06748	0.053772	0.046939	0.070829	-0.16862	-0.01496	0.001881	0.949987	0.383309
0.00352	-0.15042	0.110344	0.224502	0.009271	0.078874	0.006394	-0.002	0.740595	0.595025
-0.42505	-0.17505	0.157437	0.06327	0.065657	0.017032	0.026146	0.072398	0.425913	0.057351
-0.12759	-0.08872	0.339369	0.04332	0.114769	-0.16702	-0.05727	0.006633	0.6868	0.868612
0.002384	-0.00537	0.238652	-0.01596	0.169288	-0.13059	-0.01403	0.005064	0.533756	0.539134
0.033776	-0.05388	0.004258	0.037945	0.140265	-0.02398	0.027252	0.017184	0.886461	0.75341
0.104262	0.136646	-0.27498	0.089684	-0.49545	0.0197	-0.03918	0.018716	0.835906	0.457015
0.069531	0.005444	-0.09238	0.065833	0.043772	0.002451	-0.00047	-0.00921	0.564345	0.599299
-0.14724	-0.12923	0.259595	0.123434	0.025095	-0.00573	0.058265	0.002156	0.662785	0.600114
-0.00998	0.009864	0.033045	0.029485	0.198667	-0.02121	-0.00919	0.000826	0.663117	0.224482
0.034256	0.011446	0.303502	-0.13747	0.202231	-0.05146	-0.03367	0.002956	0.726643	0.458282
-0.00539	-0.0542	-0.14783	0.085567	-0.18634	0.061506	0.039817	-0.02726	0.647413	0.724103
-0.46803	0.027147	0.209097	0.041309	0.367612	-0.16761	0.003933	0.005076	0.955744	0.054525
0.004428	0.076825	0.261929	-0.01226	0.177923	-0.09156	0.03491	-0.04789	0.434927	0.498054
-0.10367	-0.12668	0.184582	-0.11713	0.180908	0.038077	0.043328	-0.00112	0.658363	0.297939
-0.27284	-0.20375	0.187652	-0.08371	0.228917	0.052648	-0.02147	0.005464	0.512059	0.006833
-0.06303	-0.04627	0.088684	-0.12861	-0.03946	0.036652	-0.07598	0.01029	0.224404	0.017945
-0.36204	-0.17341	0.117017	0.047157	0.239108	-0.06137	-0.02615	0.002164	0.453185	0.082135
0.021443	0.090654	0.071279	0.018377	-0.02806	-0.1306	-0.05754	-0.01149	0.571754	0.784151
0.097105	-0.1365	0.159343	-0.11162	0.129917	-0.07258	-0.02853	-0.02744	0.344337	0.104796

-0.15154	-0.1103	0.264712	0.07828	0.1535	-0.10861	-0.07469	0.026309	0.294312	0.009838
0.053426	0.07609	-0.15611	0.015293	-0.05491	-0.04225	-0.07028	0.024174	0.924738	0.868759
0.079485	0.085919	0.264821	-0.17713	-0.06389	-0.07806	0.017369	-0.00799	0.799015	0.821099
0.104429	0.015676	-0.21094	-0.10261	-0.01587	0.038083	0.051096	-0.00263	0.720781	0.618378
0.067184	0.058631	-0.27383	-0.00042	-0.18567	0.04326	0.042944	0.010219	0.855625	0.575844
0.204923	0.157103	-0.20061	-0.03928	-0.39833	0.09895	-0.03863	0.004826	0.884728	0.331827
0.06799	-0.02819	0.284957	-0.0103	0.019125	-0.05831	-0.05136	0.005648	0.758519	0.344309
-0.01416	-0.13654	0.165693	-0.09667	0.098834	-0.01003	0.095884	0.002857	0.630618	0.851071
0.018321	-0.06296	-0.1068	0.126325	0.130939	-0.03695	0.023496	-0.01636	0.694421	0.836494
-0.34278	0.211217	-0.09874	0.018455	-0.03254	0.106257	-0.05514	0.013963	0.862724	0.035143
-0.19654	-0.13	0.025332	0.14414	-0.00081	0.086082	0.050589	-0.02164	0.511658	0.133909
-0.11412	0.319412	-0.03129	-0.10613	-0.06777	-0.13857	0.03219	-0.00509	0.838587	0.153268
-0.19251	0.087146	-0.33472	-0.0302	-0.18079	0.156473	-0.03097	0.020943	0.906636	0.272568
-0.1521	0.353201	-0.13936	0.049587	-0.18886	-0.08715	-0.08937	0.069254	0.994962	0.346316
0.052504	0.05401	0.451473	-0.12497	0.143738	-0.18645	-0.01548	-0.01449	0.780397	0.724188
-0.1007	-0.01598	-0.03963	0.216327	0.067168	0.038603	-0.02728	0.002873	0.645417	0.042567
0.030567	-0.15797	0.006448	0.131538	0.14632	-0.05683	0.046067	2.33E-05	0.68807	0.448285
-0.10481	-0.00572	-0.08143	0.049075	0.111945	0.259619	-0.00844	0.002116	0.621549	0.222316
0.206147	0.174209	-0.20885	-0.00992	-0.37673	0.040178	-0.04606	0.011144	0.848683	0.422378
-0.26198	0.093312	-0.29356	0.043773	-0.18614	0.236178	-0.00231	0.01692	0.807858	0.122882
0.002999	0.118166	0.285444	-0.10104	0.019028	-0.03057	0.01931	-0.02596	0.534198	0.281466
0.125642	0.029424	-0.11186	0.12657	0.176914	-0.04995	-0.06912	0.01286	0.790887	0.460062
0.030955	0.071951	0.34672	-0.08145	0.011232	-0.10222	-0.033	0.025665	0.162399	0.892952
-0.20968	-0.15795	-0.10763	-0.09629	0.333802	0.005764	0.043746	0.011473	0.849243	0.10411
-0.05699	-0.11631	0.223637	-0.05634	0.206285	0.139864	-0.01465	0.00891	0.626488	0.378419
-0.0865	0.070726	-0.03036	-0.03563	0.018804	-0.14809	0.048005	0.060627	0.905288	0.888102
-0.12936	-0.11508	0.188576	0.120926	0.138902	-0.14902	-0.01302	-0.00126	0.969576	0.392683
-0.05937	-0.18855	-0.14008	0.082761	0.217739	0.052301	0.026846	0.009895	0.502146	0.747235
0.006275	-0.01882	0.319224	-0.14433	0.212923	-0.06295	0.034196	-0.03632	0.536092	0.449772
0.269584	-0.06081	0.329429	-0.05557	0.205219	0.113752	0.007112	0.013786	0.928294	0.024443
-0.01714	-0.05683	-0.00691	0.099102	0.006081	0.11539	0.047583	-0.0591	0.356442	0.544047
-0.10005	-0.02627	0.348977	-0.00111	0.291863	-0.12488	-0.00028	-0.00313	0.594772	0.243877
0.053724	0.002596	0.328625	-0.12133	0.21761	-0.11101	-0.01278	-0.01936	0.572773	0.945598
-0.07957	-0.07256	0.061327	-0.03088	0.097951	0.118892	0.100588	-0.05421	0.921434	0.451548
-0.03185	0.208811	0.240945	-0.09548	0.142975	-0.06568	-0.01429	-0.0817	0.846952	0.377168
0.602568	-0.14948	0.194782	0.155834	-0.02435	-0.04912	0.023299	-0.04588	0.959797	0.847908
-0.0356	-0.05265	0.029634	0.161379	0.163765	0.148304	0.080616	-0.03227	0.160628	0.192201
0.081339	-0.02495	0.081065	-0.02604	0.052537	0.041644	0.0354	-0.03639	0.501884	0.269582
-0.03392	0.032238	-0.00712	0.16292	0.036157	0.164023	-0.05739	-0.01312	0.799113	0.380173
0.013361	0.031659	-0.10742	-0.01024	-0.05952	0.060172	0.005035	-0.04839	0.482464	0.78288
-0.03956	0.046058	0.238587	-0.10549	0.09432	-0.12367	0.02893	-0.0253	0.528769	0.980236
0.076885	-0.02766	0.454455	-0.0626	0.15974	-0.14751	-0.0093	-0.01076	0.68466	0.717428
-0.01794	0.067902	0.109692	-0.03171	0.189377	-0.2277	0.053101	0.045427	0.560252	0.714016
-0.16205	-0.12882	0.559442	0.077726	0.31685	-0.22151	5.64E-05	0.003997	0.869141	0.272246
-0.1019	-0.0369	0.052887	-0.07022	0.187761	-0.05177	0.035179	0.06017	0.847216	0.539415
0.075142	-0.10685	0.28498	-0.17266	0.157367	-0.13712	0.047979	-0.00831	0.815294	0.341065
-0.07852	-0.10865	-0.11205	0.005858	-0.02202	-0.03284	0.067384	0.002656	0.922663	3.30E-06
-0.03463	0.01916	-0.06205	0.012119	0.08782	-0.06787	-0.05735	0.015314	0.835668	0.597975
0.058216	-0.03967	0.379702	-0.05542	0.218596	-0.15058	-0.01504	-0.00821	0.606076	0.472848
0.107607	-0.01073	0.221039	-0.03835	0.329406	-0.1327	-0.03029	0.000539	0.638428	0.484834
-0.06722	0.068052	0.276226	0.019988	0.249271	-0.07378	-0.09087	0.002438	0.702228	0.270827

0.03123	-0.07864	-0.06503	0.125305	0.125696	0.009844	0.043971	0.001873	0.595148	0.387962
-0.04068	-0.14412	0.327455	-0.02868	0.281611	-0.13385	0.041088	-0.00648	0.721843	0.504334
-0.0839	0.192672	0.154581	-0.09621	0.066539	-0.32584	0.041473	0.0022	0.91268	0.845413
-0.01119	0.050734	0.317235	-0.14886	0.097367	-0.02464	-0.02126	-0.01027	0.56904	0.994277
-0.0432	-0.00558	0.013782	-0.00883	0.14399	0.115473	0.080673	-0.04463	0.048804	0.155415
-0.04067	-0.05899	0.067094	0.167196	0.150442	0.022929	-0.00075	-0.00496	0.661188	0.430043
0.059126	0.061474	0.456487	-0.15292	0.191093	-0.13955	0.012419	-0.03351	0.644328	0.406799
0.081536	0.013098	0.314503	-0.11757	0.289748	-0.12605	0.015884	-0.03432	0.661739	0.315797
-0.0689	-0.10082	0.293004	-0.0602	0.21849	-0.04573	0.078188	0.003295	0.630005	0.491797
-0.17841	-0.09912	-0.04601	-0.1307	-0.0695	-0.10885	0.052927	0.008041	0.87732	0.2731
0.200445	-0.02828	0.121541	-0.10958	-0.03745	-0.16008	0.006199	-0.04771	0.651416	0.578268
-0.0467	-0.06519	0.18145	0.014178	0.1234	0.06216	0.003272	-0.00198	0.574533	0.282934
0.046974	0.057414	-0.02447	-0.06258	0.088519	-0.0547	-0.00173	0.013465	0.767111	0.874639
0.13312	0.023166	0.327047	-0.04497	0.045896	-0.04029	0.05648	-0.04385	0.443235	0.314427
0.025754	0.028535	0.116174	0.000583	-0.00509	0.097029	0.034625	-0.09155	0.273861	0.978288
0.003736	-0.01507	0.006996	0.051982	0.130602	0.036015	-0.00199	-0.0252	0.128606	0.172401
-0.1475	-0.10708	-0.20474	0.181672	0.131056	0.017856	0.047154	0.001727	0.687707	0.562521
-0.01784	-0.05367	0.359594	-0.04556	0.226563	-0.08842	0.05984	-0.01824	0.562245	0.135258
-0.16444	-0.09891	0.078587	-0.02171	0.095026	-0.15524	0.013885	0.005454	0.846569	0.402126
-0.06401	-0.04369	0.147564	0.167929	0.187758	-0.05767	-0.01741	0.006811	0.645514	0.118275
-0.18037	-0.1021	0.099371	0.044467	0.114997	-0.25684	-0.02086	-0.00074	0.766943	0.395971
-0.0805	-0.05261	-0.15681	0.232492	-0.153	-0.11541	-0.00892	0.011113	0.823275	0.128255
-0.07901	-0.07374	-0.00249	-0.06872	0.423285	0.043336	-0.05528	0.026056	0.389864	0.148223
0.175198	-0.0109	0.286448	-0.32791	0.132805	-0.10006	0.089155	0.039553	0.780467	0.285691
0.067392	-0.04275	0.134061	-0.14894	0.126452	-0.02137	0.050729	-0.00456	0.517487	0.861753
0.040884	0.025543	0.476144	-0.10977	0.2011	-0.09079	-0.02108	-0.013	0.694478	0.58046
-0.07291	0.100979	-0.0097	-0.01238	-0.05937	0.088384	-0.10852	0.016934	0.764572	0.897227
0.034169	0.228979	-0.27792	0.035339	-0.07845	0.041936	-0.07668	0.001545	0.873906	0.793041
-0.03913	0.022015	0.070392	0.035464	0.071638	0.14239	-0.03816	0.005385	0.889147	0.393557
-0.07936	-0.03501	-0.13546	0.141437	-0.03715	0.090225	-0.04859	-0.00108	0.848578	0.16638
0.058745	-0.13927	-0.09484	0.062914	-0.0332	0.152427	0.023336	0.005103	0.933073	0.472564
0.08967	-0.09881	0.098957	0.01375	0.164685	-0.12304	0.046935	-0.0083	0.712875	0.384817
-0.03502	-0.06842	0.146796	0.034518	0.2604	-0.1392	-0.04125	-0.01498	0.994802	0.957237
-0.02699	0.009929	-0.27436	0.033398	-0.09598	0.099736	-0.01724	0.021848	0.937513	0.412387
0.073347	0.069931	0.395737	-0.08664	0.058872	-0.12405	-0.05596	-0.02056	0.728066	0.115198
-0.00673	-0.05318	0.206972	0.083362	0.050999	-0.11454	0.122765	-0.04438	0.397696	0.722753
0.085199	0.064232	-0.10165	-0.19658	0.089497	0.152482	0.04832	0.017016	0.948733	0.284244
0.044434	-0.03564	0.260203	0.037368	0.210581	-0.16194	0.000381	0.004663	0.810055	0.268737
0.13295	0.044464	-0.16332	0.09687	-0.08095	0.24223	-0.01535	0.015124	0.928009	0.030306
-0.11485	-0.13877	0.454317	0.04864	0.34148	-0.09558	-0.03304	-0.00472	0.998003	0.239193
0.017692	-0.02997	0.313583	-0.05897	0.17542	-0.07788	0.04915	-0.00561	0.575045	0.472381
0.050569	-0.06743	0.297321	-0.04583	0.232814	-0.04898	0.018105	-0.00273	0.682681	0.529496
-0.22766	-0.08844	-0.1855	0.11612	-0.03802	0.069547	0.017607	-0.0041	0.739583	0.117179
0.033059	0.034086	-0.13793	0.016823	0.116095	-0.16014	0.023498	-0.00802	0.713487	0.759521
-0.07678	-0.02982	-0.03074	-0.00996	0.040716	-0.10616	-0.14699	0.04146	0.968391	0.301355
0.060312	-0.00692	0.383607	-0.07613	0.149789	-0.13182	0.030132	-0.03058	0.470519	0.367369
-0.08708	-0.0484	0.29334	-0.01116	0.095758	-0.10451	0.036884	-0.00636	0.536892	0.654965
0.241008	-0.11986	0.135038	-0.10658	0.051517	0.06451	-0.06018	0.001711	0.614381	0.195611
0.335439	0.026477	0.173408	-0.11703	0.061922	-0.07709	0.026726	0.013816	0.874835	0.248769
0.153504	0.109746	0.11566	-0.10338	-0.06053	-0.07417	-0.02893	0.010284	0.689795	0.235875
0.013351	0.152099	0.129078	-0.18326	0.00562	-0.18175	-0.03632	0.042012	0.9574	0.992214

-0.01471	0.07496	-0.09931	-0.03144	-0.09547	0.221132	-0.02034	-0.07693	0.311423	0.62754
-0.045	0.008495	0.159613	0.001762	0.136829	-0.00124	0.007838	-0.03016	0.592033	0.409153
-0.09152	-0.10458	0.179712	0.114859	0.107077	0.012605	0.062261	-0.0702	0.312896	0.321824
-0.09794	-0.01357	0.228929	-0.04049	0.190273	0.04857	-0.01693	-0.03787	0.597868	0.334931
-0.00247	-0.05526	-0.0037	0.213843	-0.04273	0.2263	0.044932	-0.05579	0.188981	0.259551
0.098984	0.063631	-0.02532	0.006743	-0.42907	0.047308	-0.01934	-0.00061	0.734546	0.219608
-0.03187	0.011469	0.261372	0.092553	0.247278	-0.11451	-0.0081	-0.02452	0.572516	0.199707
-0.05218	-0.0516	0.050658	0.08721	0.093122	0.018578	0.059547	-0.02555	0.517798	0.539241
-0.30047	0.010607	0.047919	0.096223	-0.12755	-0.19347	-0.00743	-0.00309	0.856476	0.110827
-0.07858	0.063666	0.262482	-0.02979	-0.06053	0.105089	-0.06856	########	0.639745	0.607611
0.092682	-0.03635	0.413466	-0.08357	0.252259	-0.16244	-0.00016	-0.01107	0.714651	0.627079
0.066286	-0.01499	0.578421	-0.14472	0.075991	-0.13151	0.030089	-0.02193	0.751608	0.559429
-0.24581	-0.17682	0.101288	0.094468	0.501442	0.080655	-0.04954	-0.00373	0.464859	0.43059
-0.24619	-0.20092	0.19441	0.000362	0.009657	0.02464	-0.0635	-0.00658	0.372086	0.000232
-0.1369	-0.05779	-0.06231	0.277507	-0.0454	0.059254	-0.00229	-0.00455	0.747821	0.164608
-0.07191	0.086634	0.112017	-0.02071	-0.02565	-0.07517	-0.01735	0.005114	0.741388	0.068883
0.48244	0.013306	-0.30839	0.055055	-0.21346	0.104347	-0.08696	0.003118	0.863491	0.155698
-0.2022	-0.06731	-0.1467	-0.06984	0.496797	-0.08238	0.008076	0.007519	0.976019	0.395852
0.044267	-0.05403	0.356046	-0.00563	0.261548	-0.06239	-0.01452	-0.00287	0.900208	0.235922
0.086097	0.107667	-0.28321	0.168452	-0.06386	0.09474	-0.05562	0.011953	0.816496	0.09775
0.222056	0.004459	-0.26168	-0.00808	-0.17185	0.168069	-0.04366	-0.01138	0.315784	0.084145
-0.04446	0.112227	-0.12896	-0.08134	0.032011	0.027585	-0.03302	-0.0123	0.644253	0.683495
0.052977	-0.04871	-0.2704	0.047339	-0.26935	-0.0119	0.033261	0.013932	0.79381	0.198755
-0.02827	-0.06996	0.134071	0.18122	0.278185	0.009582	-0.00998	0.007412	0.71484	0.091649
-0.04251	0.019071	0.230832	-0.08739	0.226849	-0.02329	0.014759	-0.00821	0.581756	0.450163
-0.11579	-0.02452	-0.04343	0.250388	-0.11251	-0.07268	-0.01261	0.007034	0.929462	0.437256
-0.2061	-0.13959	0.836242	0.028987	0.072046	-0.08352	0.013193	-0.00577	0.889162	0.248978
0.070364	-0.13046	0.037612	-0.064	0.122889	0.190461	-0.02878	0.000993	0.558421	0.504551
0.114553	-0.10052	0.232016	0.016057	-0.05544	-0.00274	-0.07144	0.003096	0.886501	0.951756
0.0304	-0.02963	0.240128	-0.08531	0.218426	-0.15332	-0.03873	0.001593	0.589394	0.949853
-0.18478	-0.10652	0.020796	0.172782	0.121177	-0.03341	-0.04796	0.005299	0.447424	0.979567
0.354608	0.30485	-0.21245	-0.08776	-0.33816	-0.11988	-0.0421	0.004192	0.870894	0.133395
-0.02181	-0.03374	-0.06549	0.079265	0.004897	0.188772	0.080003	-0.06707	0.255393	0.199551
-0.06467	-0.00037	-0.22003	0.219565	-0.06787	0.055982	-0.02182	-0.02374	0.630596	0.974685
-0.01323	-0.07184	-0.13659	0.01092	0.116267	0.089534	0.068675	-0.02344	0.47336	0.428882
-0.09747	-0.02024	-0.01878	0.23945	-0.04189	0.026957	-0.00358	-0.03384	0.578008	0.752424
-0.05601	-0.10132	0.030644	0.082125	0.161816	0.030611	-0.00622	0.026671	0.913975	0.227166
0.166744	-0.06451	0.118799	0.043309	0.254885	-0.06119	0.001123	0.008415	0.739349	0.172119
0.016864	-0.11178	-0.04734	-0.03139	0.008428	-0.05497	-0.02907	-0.13484	0.774429	0.039256
-0.07913	-0.21188	-0.04932	-0.05724	-0.06553	0.051219	0.081136	0.00261	0.825309	0.004536
0.032634	-0.02562	0.241273	-0.00944	0.300797	-0.10828	-0.0225	0.002943	0.530978	0.572427
-0.04712	-0.0822	0.202038	-0.07382	0.221274	0.005143	-0.06191	-0.06935	0.395356	0.828623
0.291671	-0.12838	0.096278	0.118005	-0.2081	0.025482	0.005444	0.036346	0.687801	0.01867
-0.07898	-0.10127	-0.21779	0.119936	0.045995	0.01466	-0.02488	-0.01037	0.470601	0.440318
-0.00655	0.237105	-0.34316	0.136341	-0.51341	0.020188	-0.08141	-0.00853	0.577249	0.769718
-0.21604	-0.00577	-0.07508	0.003451	-0.04459	-0.08194	0.002241	-0.0182	0.788463	0.076759
-0.16455	-0.05747	-0.20701	0.115216	0.045964	-0.08836	0.026836	0.006796	0.686636	0.258831
-0.10153	0.464624	-0.0631	-0.07985	-0.09437	-0.0639	-0.0057	0.055826	0.89607	0.246808
0.126507	0.016347	-0.08203	-0.11447	0.024786	0.458242	-0.01195	0.005569	0.977524	0.239024
-0.01804	0.015959	-0.15483	-0.01693	-0.1771	-0.04237	0.042342	-0.00155	0.914731	0.127049
0.210679	0.010742	-0.01202	0.037938	0.115308	-0.10833	0.033745	-0.01005	0.853064	0.52988

0.176253	0.081538	-0.09055	-0.0712	-0.25046	0.012308	0.00644	-0.00676	0.700939	0.18968
-0.08501	-0.04624	0.190288	-0.0595	0.334177	-0.0985	-0.00362	0.015226	0.98141	0.587885
-0.16193	-0.03307	0.208337	0.012019	0.394814	-0.09357	0.034177	-0.00396	0.823093	0.964051
-0.0206	0.081796	0.055598	-0.00229	-0.00153	0.038061	-0.04236	-0.02515	0.51771	0.936697
-0.05066	0.109247	0.031576	0.104241	0.064612	0.007614	0.013241	-0.04514	0.553603	0.26614
0.361688	-0.01766	0.105642	-0.09594	0.137897	-0.1245	-0.01367	-0.01244	0.693554	0.560178
0.034778	0.02966	0.013156	-0.0345	-0.0356	-0.00601	0.045748	-0.00262	0.889368	0.722918
0.232357	0.162678	-0.02991	-0.20247	-0.11919	-0.08797	-0.03577	-0.00469	0.855091	0.186992
0.157772	0.037474	0.017547	-0.02854	-0.05317	-0.00065	-0.05532	-0.02797	0.657954	0.452879
0.064156	-0.09514	0.085152	0.14045	0.077896	-0.09801	0.040055	0.002975	0.657608	0.648337
-0.01038	-0.09785	0.030641	0.055196	0.000885	0.032244	0.014143	-0.0428	0.571677	0.726575
0.047848	-0.02755	0.194761	0.028263	0.136528	-0.09646	0.009416	-0.00977	0.837836	0.492014
0.022802	-0.02335	-0.04126	0.135593	0.011314	0.083997	0.019915	-0.0344	0.561785	0.554863
-0.06252	0.075514	-0.05998	-0.14413	-0.13508	0.085807	-0.04023	0.02207	0.954648	0.001074
-0.10603	-0.07314	0.17149	-0.08643	0.354731	0.099236	-0.00277	0.006717	0.988532	0.818642
-0.11913	0.203929	-0.02228	0.029785	-0.0649	-0.00672	-0.02083	-0.02301	0.782724	0.496013
-0.00678	-0.01689	0.089138	0.079074	-0.00978	-0.04555	0.010293	-0.0073	0.794165	0.713842
0.017745	-0.10761	0.281685	-0.05292	0.206009	-0.1462	0.060984	-0.01443	0.515558	0.499279
0.093494	0.128656	0.062958	-0.01093	-0.06164	-0.01793	-0.13866	-0.00561	0.851597	0.934259
0.107298	-0.0732	0.127186	-0.01993	0.380724	-0.0132	0.048617	-0.03154	0.575047	0.501559
0.026692	0.014887	-0.0507	0.155154	0.079957	0.01963	-0.05254	0.008049	0.454532	0.256572
0.724162	-0.0828	-0.19781	-0.02351	-0.11133	0.143384	0.033535	0.009632	0.845453	0.18894
0.151449	-0.05628	-0.08267	-0.03171	-0.02903	0.147126	0.084882	0.018985	0.838131	0.523974
0.122846	0.130131	-0.13234	0.065258	-0.35895	0.039222	-0.03406	0.022099	0.89323	0.871862
0.055501	-0.04058	-0.08262	0.13617	-0.39006	0.07165	0.021801	0.007781	0.543913	0.777671
-0.04417	-0.03195	-0.01041	-0.05166	0.009375	0.127639	0.038797	-0.04288	0.524399	0.202198
-0.08153	-0.06409	0.082532	0.096352	-0.03315	-0.10046	-0.00452	0.002038	0.791766	0.23557
-0.05815	-0.11977	0.015889	-0.09315	0.176736	0.095785	0.084069	-0.00109	0.593151	0.702926
0.38369	-0.22569	0.257167	0.043589	0.208216	-0.05238	0.00675	-0.00257	0.250201	0.92214
0.04439	0.102583	0.087841	-0.0582	-0.48034	0.021838	0.042521	-0.01305	0.891517	0.814584
-0.15291	-0.06454	-0.13863	-0.01463	-0.10754	-0.12355	0.042682	0.043869	0.96542	2.36E-05
-0.07715	0.038622	-0.00612	-0.06073	0.012409	0.026176	0.08078	-0.00757	0.519438	0.768329
-0.00536	-0.06473	0.044804	-0.03439	0.254307	-0.09181	0.001925	0.021315	0.912477	0.689499
-0.01469	-0.09815	0.196096	0.05802	0.036113	-0.00654	0.034346	0.002546	0.682039	0.619706
0.025511	0.000453	0.318887	-0.05483	0.21434	-0.16487	-0.02487	0.000707	0.981856	0.587611
0.079945	0.110444	0.073563	-0.20511	0.054682	0.018117	-0.05192	0.006613	0.885753	0.633127
0.138391	0.150453	0.054799	-0.13794	-0.05618	-0.0768	-0.04182	0.011442	0.798454	0.714319
-0.02196	0.212452	-0.2935	0.064547	-0.29012	0.017792	-0.06477	0.012954	0.917407	0.671993
0.131924	-0.00055	0.117508	0.083635	-0.0135	-0.0681	0.020408	-0.01503	0.674227	0.508595
0.337647	0.155038	-0.0376	-0.18456	-0.09138	-0.06425	-0.00792	-0.0079	0.830885	0.97765
0.084506	-0.03669	-0.07869	0.09797	0.048894	0.318577	-0.17737	-0.01795	0.283694	0.443052
0.015989	0.093056	0.152599	-0.00631	-0.00713	-0.01218	-0.02436	-0.02245	0.528991	0.296277
0.163327	-0.02098	-0.11548	0.024495	-0.03744	-0.12063	0.040189	-0.02345	0.90248	0.414151
-0.19425	-0.07228	0.08717	0.034169	0.415057	0.079755	0.006459	0.016142	0.868381	0.041533
-0.01489	0.063686	-0.29537	0.029388	-0.08538	-0.00459	-0.00777	0.033243	0.750601	0.174118
-0.01119	0.034406	0.227678	-0.07022	0.130085	-0.01739	-0.00687	0.00526	0.810178	0.872643
-0.03489	-0.00666	0.103893	-0.00376	-0.07219	-0.11358	0.025266	-0.00367	0.945087	0.423202
-0.00538	0.08045	-0.37405	0.056375	-0.31746	0.058784	-0.01599	0.022164	0.932441	0.006273
0.19417	0.098879	0.180771	-0.07742	-0.22782	0.005652	-0.04047	-0.00228	0.764243	0.234505
0.123419	0.330858	-0.12936	-0.1378	-0.09939	-0.18049	-0.00303	0.059421	0.848605	0.210308
-0.15345	-0.15054	-0.11465	0.002319	0.187909	0.011724	-0.03005	0.002171	0.966599	0.923546

-0.05868	0.007106	-0.00881	-0.08922	0.25215	-0.14258	-0.0143	0.004669	0.768269	0.492548
-0.04087	0.023501	-0.07047	-0.03335	-0.04321	0.009221	-0.00378	0.010653	0.930825	0.635811
-0.23623	-0.18897	0.208979	0.016889	0.364441	-0.0874	-0.02948	0.01604	0.947217	0.996101
-0.18523	-0.08917	-0.03227	0.290264	0.191827	0.167798	-0.07655	-0.00873	0.64115	0.371716
-0.07919	-0.13168	0.392172	0.002247	0.257325	-0.00888	-0.02796	-0.01114	0.890714	0.093451
-0.29854	0.142271	-0.27975	0.210559	-0.27883	-0.11297	-0.00757	0.047535	0.852018	0.165813
-0.21647	-0.08088	-0.18221	-0.05347	-0.10491	-0.03461	0.061064	0.020309	0.939824	0.008566
-0.16295	-0.11252	-0.17722	0.035037	-0.10403	0.036163	0.030141	0.017646	0.931253	0.070241
0.5623	0.030205	-0.29741	0.019258	-0.36703	0.15611	-0.06738	0.004116	0.899595	0.796795
0.06356	0.03534	-0.05188	-0.03533	-0.09022	-0.06699	-0.02863	0.022722	0.848552	0.855022
-0.35025	-0.05853	-0.1162	0.220315	0.258279	-0.1414	0.06957	0.005011	0.861543	0.111546
-0.34906	-0.02116	0.133605	0.179095	-0.15578	-0.25773	-0.09286	-0.00703	0.708665	0.123898
-0.01667	-0.04886	0.00941	0.25287	-0.04332	-0.05399	0.044414	-0.012	0.561395	0.924854
-0.07533	0.061148	0.236025	0.036347	-0.00966	0.037582	-0.02908	-0.06937	0.336121	0.891977
-0.00226	-0.06299	0.141735	0.109908	0.196242	-0.07204	-0.01133	0.009091	0.825193	0.236545
-0.04622	-0.00563	0.048708	0.053604	0.074527	-0.05857	0.000768	0.00779	0.595573	0.492707
-0.03176	-0.06162	0.091734	0.003502	0.148471	-0.06175	0.022845	-0.00275	0.703862	0.444756
-0.42128	-0.1126	0.059265	0.024943	0.229913	-0.10046	-0.02693	0.000287	0.739295	0.291473
0.032123	0.021049	0.388443	-0.07863	0.061302	0.03759	-0.00686	-0.03699	0.457262	0.454719
-0.00258	-0.0884	0.084666	0.094454	0.13472	-0.01338	0.078114	-0.04112	0.403064	0.445585
-0.31801	-0.08337	0.243249	-0.11805	0.133925	-0.23564	0.014151	0.004722	0.903323	0.374976
0.044844	-0.02112	-0.17875	0.079678	-0.02373	0.091842	0.014228	0.003764	0.714621	0.493328
-0.06614	-0.04	0.19604	-0.11675	0.091245	-0.05943	0.003521	-0.0082	0.65475	0.637683
-0.04471	0.076386	-0.08102	-0.09362	0.52051	-0.06381	-0.01597	0.007541	0.782472	0.830948
0.047156	0.022048	-0.24517	0.111202	-0.27078	0.243231	-0.05719	-0.00454	0.809698	0.128311
0.000603	-0.10529	-0.07065	0.029441	-0.01112	-0.0397	-0.05145	-0.01298	0.385434	0.457626
-0.11042	-0.06536	-0.02762	0.150358	-0.02372	-0.08724	-0.01444	0.009912	0.970069	0.630543
-0.06445	-0.0575	-0.25669	0.143541	-0.08398	0.10888	0.035693	0.062853	0.718532	0.108999
0.02989	0.010125	-0.09086	0.013242	-0.05055	0.098573	-0.01893	0.025914	0.843769	0.829092
-0.20633	-0.16845	-0.09776	0.124733	-0.1162	-0.10847	0.111929	0.016388	0.612533	0.925601
-0.10463	-0.12782	-0.13101	0.122226	-0.00397	0.322316	0.043008	-0.00401	0.642938	0.17046
-0.24972	0.020381	0.080202	0.210613	-0.28898	-0.05597	-0.01857	0.012306	0.797966	0.256593
-0.01446	0.01564	0.03605	-0.01565	0.05998	-0.00107	-0.07302	0.08277	0.865433	0.816886
0.121095	0.049964	-0.09422	0.020125	-0.19151	0.040673	-0.11573	-0.00227	0.51263	0.160182
0.118115	0.089445	-0.12474	-0.05357	-0.15578	0.10163	-0.02698	0.00542	0.77208	0.746133
-0.01542	0.08084	0.011108	0.158639	-0.00347	0.06519	-0.07076	-0.0181	0.458858	0.308575
-0.00807	-0.11624	0.058372	-0.07802	0.264248	-0.0574	0.021057	0.000406	0.695877	0.639008
0.07479	-0.04124	-0.01903	0.049104	-0.02085	0.016473	0.025581	-0.01547	0.754951	0.657267
0.483727	0.057089	-0.32232	-0.00972	-0.35148	0.148331	-0.03058	0.005759	0.709114	0.565307
-0.36929	0.057977	-0.19749	0.11504	0.167975	-0.07691	-0.0119	-0.00274	0.794334	0.028182
0.001908	-0.06127	0.313246	-0.06586	0.230913	-0.11893	0.029606	-0.00078	0.617665	0.651569
-0.04234	0.036068	-0.24362	0.158839	-0.29685	0.116349	-0.06994	0.00628	0.706969	0.539823
0.136843	-0.01618	0.16657	-0.03996	0.037781	-0.10716	-0.00585	0.013537	0.892414	0.755405
-0.11912	-0.038	-0.11247	0.290562	-0.09816	-0.048	-0.01209	0.003842	0.887066	0.126493
-0.00442	0.034194	-0.01919	0.013554	0.015658	-0.06892	0.008644	-0.07777	0.694441	0.586189
0.202754	0.089803	-0.45945	0.111101	-0.06726	-0.31661	-0.0129	-0.00328	0.622698	0.91158
-0.01571	0.03796	-0.04396	0.056827	0.200329	-0.12878	0.068702	0.007888	0.724055	0.321399
0.507663	-0.03393	-0.24547	0.062832	0.069604	-0.13645	-0.01248	-0.00342	0.959164	0.108752
0.108334	0.150229	-0.37817	0.080454	-0.53372	0.120414	-0.03682	-0.00316	0.846741	0.843281
-0.01416	0.002857	-0.3039	0.128444	-0.36945	-0.04239	-0.0263	0.006784	0.632391	0.325017
0.094658	0.146882	-0.09699	-0.05899	-0.27737	-0.14138	-0.0333	-0.01897	0.58291	0.798589

0.035197	0.024239	0.292601	-0.00056	0.163103	-0.09994	-0.00641	-0.00832	0.614037	0.2556
0.012179	0.161011	-0.06596	0.004594	0.039671	0.061251	-0.16817	0.017854	0.719489	0.129559
-0.13397	-0.06959	0.129109	-0.06186	-0.03115	0.497175	-0.00213	0.026446	0.987028	0.23909
-0.06424	0.052973	-0.00263	0.071908	0.022137	0.003201	0.018068	-0.07432	0.595362	0.790021
-0.06224	-0.01196	-0.08876	-0.0926	-0.11812	-0.03472	0.074736	-0.01616	0.75634	0.002553
0.095672	0.22013	0.243206	-0.03929	-0.17104	-0.08418	-0.08195	0.005903	0.76016	0.455803
-0.06094	0.052819	0.131442	-0.00459	0.090296	-0.02817	0.025224	-0.03844	0.535942	0.531025
0.13604	0.057521	0.253997	-0.17248	0.181093	-0.19572	0.007448	-0.02103	0.723584	0.985361
0.036397	0.122568	0.197583	-0.04796	-0.01272	-0.07086	-0.05506	-0.00891	0.842191	0.70384
0.043386	-0.04408	-0.13765	0.100805	-0.24187	0.183832	0.062104	-0.04542	0.510262	0.852377
-0.23819	-0.08824	-0.19752	-0.10818	0.179534	0.199773	0.094077	0.013126	0.958846	0.67121
-0.29463	-0.09827	-0.22544	0.141714	0.406278	-0.09402	0.02904	0.010528	0.939975	0.841228
-0.03477	0.015323	-0.11859	0.071887	-0.03068	0.203511	0.054848	-0.05209	0.53115	0.529669
0.154525	0.124011	0.214973	-0.14678	-0.02385	-0.13519	0.013606	-0.00755	0.661129	0.855879
-0.07254	0.105495	-0.18354	0.091689	-0.10698	0.017392	-0.08475	0.01882	0.989941	0.344328
0.066853	0.026783	0.037355	-0.02	0.180496	-0.03569	0.009347	-0.00241	0.598803	0.161861
-0.10354	-0.00567	0.229203	-0.16402	0.101829	0.337455	-0.00222	0.000106	0.838957	0.475774
-0.10966	0.008118	0.034663	0.036822	-0.00931	0.109569	0.039658	-0.01447	0.124431	0.468175
0.083454	0.019108	0.348988	-0.05427	0.261894	-0.07073	-0.0646	-0.00401	0.889217	0.803111
-0.05711	-0.02243	-0.01529	-0.02218	0.031829	0.26564	0.010884	0.010836	0.792275	0.796254
-0.05846	-0.1077	0.001186	0.152698	-0.01333	0.134668	0.092433	-0.06262	0.364314	0.1857
0.08491	0.018497	0.248389	-0.12031	0.174699	-0.07371	0.018172	-0.01121	0.718559	0.831248
0.085657	0.06586	0.320069	-0.19143	0.114893	-0.14906	0.051099	-0.02229	0.76057	0.852008
0.030624	-0.10777	0.01463	-0.06813	0.226188	0.074213	0.034659	-0.00199	0.733211	0.351829
-0.11571	-0.04102	-0.01563	0.128657	0.590061	-0.06246	-0.03344	-0.00619	0.787563	0.626113
0.003865	0.041981	-0.09186	0.175023	-0.0853	0.18807	-0.09428	-0.07324	0.210076	0.530837
-0.00665	0.110271	-0.23463	-0.06222	-0.11066	0.016032	0.014335	0.021572	0.897373	0.087248
0.076976	0.028075	-0.05759	0.03157	-0.00534	-0.04926	0.015073	0.014994	0.663017	0.783566
-0.01222	-0.02571	0.313133	-0.02821	0.19091	-0.06709	0.005915	-0.00933	0.711223	0.624786
0.008305	-0.00873	-0.01331	0.015917	-0.29689	0.078735	-0.02592	0.008019	0.662516	0.544773
0.093924	0.126876	0.017501	-0.02193	-0.42752	0.114043	-0.02154	########	0.784747	0.245371
-0.17352	0.287859	-0.14567	-0.02779	-0.10967	-0.11238	-0.03317	0.008796	0.975389	0.09058
0.188425	0.060931	-0.08792	0.026949	-0.4549	0.00075	-0.03096	-0.00428	0.73082	0.419923
0.095743	0.008805	0.155912	-0.09661	0.158883	-0.13407	0.024577	-0.02156	0.392374	0.702885
0.175791	-0.02609	0.321956	-0.08572	0.180509	-0.14412	-0.02294	-0.00862	0.784149	0.570504
0.234332	-0.03318	-0.00378	-0.17976	-0.02505	0.011216	0.050052	-0.01256	0.636636	0.22257
0.209673	0.204469	-0.42943	0.035574	-0.31201	-0.00401	0.0019	0.003276	0.736732	0.225192
-0.12855	0.035698	-0.05732	-0.01942	0.294799	0.007384	-0.00039	0.010371	0.768383	0.711229
0.304496	0.162661	-0.20262	0.062796	-0.48421	0.08115	-0.0319	0.004242	0.817707	0.841553
-0.03289	-0.02307	0.049377	-0.01563	-0.03246	-0.01204	0.074219	-0.00445	0.344794	0.550655
-0.08514	-0.12869	0.35764	-0.19328	0.050516	-0.17824	0.133025	-0.01358	0.824322	0.056435
-0.01416	0.027053	-0.05195	0.180545	-0.09248	0.057634	0.005507	-0.08316	0.305914	0.513715
0.076379	-0.00338	0.141739	-0.20063	0.166518	0.033841	0.018412	0.002288	0.867804	0.866571
0.071574	-0.00442	0.348653	-0.11579	0.111723	-0.0949	-0.01699	-0.0056	0.651221	0.304646
-0.03381	0.056813	0.005805	0.044555	0.129258	-0.13812	-0.00115	-0.0114	0.58883	0.655788
-0.01898	-0.07497	-0.10614	-0.03272	-0.02827	-0.05261	0.03476	0.00716	0.884382	7.76E-06
0.092566	-0.07745	0.029205	0.089246	0.050653	0.037413	-0.00348	-0.01607	0.53739	0.353711
0.064576	0.092763	-0.10186	-0.00887	-0.16926	-0.19646	0.050829	-0.00204	0.937395	0.509012
0.111994	-0.02402	-0.09432	0.038744	-0.08195	0.033366	-0.01751	-0.01536	0.480602	0.951462
0.032429	0.046376	-0.03036	-0.08991	0.020172	-0.10972	0.063101	0.057228	0.483615	0.324744
-0.0516	0.077616	-0.2114	0.06104	-0.03854	0.161589	-0.0584	0.00888	0.967135	0.545114

-0.17121	0.00514	-0.10915	0.029531	-0.05232	0.02432	-0.01701	0.004964	0.789528	0.445145
-0.24716	0.062895	0.009217	-0.08017	0.319748	-0.04071	-0.05199	0.013623	0.495938	0.917196
-0.19055	-0.21388	-0.14883	0.131116	0.301441	0.24359	0.005516	0.006354	0.627562	0.353902
-0.14487	-0.07527	0.108656	-0.0494	0.173031	-0.08685	0.030944	-0.0048	0.655109	0.108244
0.096495	-0.10175	-0.01215	0.139512	0.033783	-0.03516	0.090285	-0.02171	0.298682	0.153981
0.09434	-0.01124	-0.30899	0.159381	-0.53938	0.110453	-0.02934	-0.00632	0.641932	0.801813
0.215464	0.076906	-0.36076	0.086237	-0.39985	-0.15056	-0.04015	-0.01316	0.621315	0.164415
0.008631	-0.0016	0.209968	0.059168	0.057627	-0.04176	0.006596	-0.01305	0.621619	0.175924
0.055134	0.042222	0.26463	0.004279	0.185262	0.011824	-0.04945	-0.11711	0.449439	0.539264
-0.1705	-0.00373	-0.09777	0.005529	-0.1037	0.101745	-0.00221	0.013489	0.925441	3.36E-05
-0.10376	0.057767	-0.0245	0.086072	0.036425	0.024843	0.029222	-0.03488	0.555039	0.645257
0.005328	-0.09275	0.099626	-0.01779	0.142931	-0.13536	0.066649	0.004349	0.615262	0.760711
0.018752	0.017447	0.109771	0.008104	-0.13908	0.126375	0.011518	-0.06507	0.421621	0.710586
-0.13249	0.145119	-0.18574	0.049522	0.083541	0.046496	0.006942	-0.00206	0.746529	0.321399
0.015666	-0.00119	-0.26751	0.137818	-0.33048	0.118239	-0.04011	-0.00292	0.732285	0.549793
-0.09718	-0.13499	-0.07034	0.121919	-0.15104	0.13134	-0.02536	0.023154	0.917278	0.814971
-0.36724	-0.15009	0.44399	-0.07931	0.326336	-0.01607	-0.01409	-0.00309	0.655853	0.316679
-0.12778	-0.04129	-0.19585	0.07556	-0.10309	-0.02061	-0.01086	0.011085	0.778859	0.236397
0.011939	0.028363	-0.1192	-0.01311	0.148586	-0.01317	0.052573	-0.00235	0.888478	0.824239
0.021198	0.205017	-0.17663	-0.13239	-0.19482	-0.07995	0.03966	-0.0491	0.497361	0.001739
-0.08336	0.16224	0.063459	-0.11969	0.121547	-0.13255	-0.05075	-0.00087	0.929052	0.674803
0.01418	0.051528	0.061596	-0.06306	0.195787	-0.07429	-0.02522	0.006473	0.755369	0.12121
0.06934	0.041546	0.457677	-0.16928	0.174578	-0.21596	0.008186	-0.01744	0.793992	0.571179
-0.12854	0.002802	0.085783	-0.07952	0.43222	-0.05192	0.024088	-0.02767	0.642122	0.790726
0.004063	-0.08002	-0.09304	0.013259	0.029966	0.110552	0.033469	-0.04415	0.555563	0.974649
-0.02994	-0.00435	0.105576	-0.0039	0.023212	0.043476	0.002487	-0.01903	0.787707	0.451082
0.047857	-0.04104	-0.05882	0.075028	0.016502	-0.01734	-0.04499	0.026243	0.821059	0.172198
-0.01074	0.015688	0.295411	-0.10018	0.120365	0.04527	0.028128	-0.08563	0.240702	0.799714
0.451609	-0.12021	-0.20783	0.080839	-0.12047	0.336815	0.012971	0.00363	0.937113	0.064037
-0.00614	0.006226	-0.04882	0.137694	0.165692	-0.09642	-0.01393	0.007012	0.707148	0.446922
-0.04527	-0.02464	0.069355	0.008556	-0.19807	0.008809	-0.02873	0.004621	0.873552	0.810762
-0.26926	-0.13675	0.441508	0.031781	0.032859	-0.00858	-0.00657	0.006646	0.642601	0.503294
0.003689	-0.03707	-0.03457	-0.03908	0.373087	-0.05432	-0.01524	-0.00744	0.66065	0.596677
-0.01183	-0.0051	0.193327	-0.02808	0.174459	0.062003	0.013057	-0.11548	0.192226	0.520793
0.06195	-0.17261	0.010957	0.203224	0.1014	0.238754	0.081484	0.008991	0.93118	0.009155
0.194612	-0.08949	-0.04822	0.047286	-0.13421	0.070666	-0.05047	0.00751	0.804721	0.393192
0.261753	0.148062	-0.26444	0.021365	-0.2313	0.032746	-0.03017	0.009712	0.900036	0.167418
-0.22044	-0.03745	-0.16374	0.008701	-0.12109	-0.11094	0.005403	0.004397	0.994611	0.330589
-0.03469	-0.06068	-0.04388	0.112631	0.070865	-0.08668	0.018979	0.010314	0.661431	0.23836
-0.03885	-0.07481	-0.02829	-0.00135	0.026134	-0.0098	0.072153	-0.02677	0.514003	0.55228
0.03257	0.024887	0.32606	-0.01318	0.212453	-0.16766	-0.01999	-0.00724	0.773039	0.519506
0.093433	0.043538	0.263108	0.057418	0.157283	-0.13222	-0.04629	-0.00184	0.699877	0.524204
-0.05144	-0.0092	0.201435	0.046989	0.193178	-0.06371	-0.00066	-0.00272	0.652413	0.281166
-0.05134	-0.06402	-0.08933	0.124976	-0.01948	0.098768	-0.01182	0.240881	0.506796	0.214707
-0.23849	-0.01143	0.023207	0.196469	-0.1123	0.137539	-0.01502	0.000457	0.828001	0.016755
-0.05428	-0.07795	-0.12324	0.158542	0.05354	0.105828	0.004993	0.005403	0.715761	0.709793
-0.07791	0.031861	0.139122	0.040575	-0.1119	-0.28616	0.005122	-0.00785	0.847864	0.874169
-0.16474	-0.11511	0.498695	0.073145	0.085142	-0.13783	-0.08037	0.050359	0.475073	0.040472
-0.06129	0.042618	-0.02548	0.119641	0.059497	0.138869	0.039004	-0.04924	0.421626	0.513551
-0.05353	-0.11578	0.372624	0.023251	0.152607	-0.0321	-0.00055	0.010283	0.799946	0.343681
0.289597	0.096271	-0.26789	-0.08698	-0.20715	0.252638	0.022001	0.01204	0.999996	0.622947

-0.04188	0.03662	0.347894	-0.03704	-0.14775	-0.02537	0.019287	-0.05869	0.844896	0.997146
-0.05985	-0.01812	-0.12413	0.148502	-0.02275	0.347682	0.024132	0.008889	0.871297	0.206894
-0.10358	0.089352	-0.17029	-0.10871	-0.09948	0.11466	-0.02854	-0.00388	0.874709	0.495753
0.145456	-0.026	-0.07559	-0.0468	-0.04413	0.026763	-0.09167	-0.01717	0.226823	0.043785
-0.31547	-0.07106	0.135834	0.023723	0.172886	-0.11783	-0.05494	0.011536	0.449042	0.877988
0.249806	-0.03952	0.036893	-0.04277	-0.11249	0.008141	0.009988	-0.00953	0.681193	0.681024
-0.22841	-0.10011	-0.17358	0.156215	0.051131	0.23993	0.009826	-0.00887	0.950934	0.14033
0.07818	0.132991	-0.0625	-0.00924	0.069135	-0.09288	-0.06116	0.003178	0.971961	0.727015
-0.06564	0.002296	-0.3253	-0.00276	-0.22981	0.113288	-0.0277	0.026159	0.982864	0.273017
-0.01996	-0.00069	0.254897	-0.0705	0.102377	-0.06943	0.021943	-0.00823	0.775382	0.986769
-0.08494	-0.13671	-0.15721	0.206425	0.062968	-0.00067	0.041633	0.034622	0.985002	0.623169
0.216319	0.08787	-0.15299	-0.1033	-0.34251	0.062231	-0.08536	0.045834	0.645623	0.696143
0.074062	-0.00079	-0.27486	0.058816	-0.34832	0.126663	-0.02033	0.013508	0.997097	0.799591
-0.05607	-0.09896	-0.01485	0.035759	-0.17121	0.2471	0.038556	0.008313	0.77968	0.552527
-0.08063	0.041752	0.058894	0.08781	-0.23739	0.02434	-0.05338	-0.0106	0.757373	0.41251
-0.01175	-0.0873	-0.03018	-0.05042	-0.16835	0.060036	0.11808	0.002101	0.935304	0.808236
0.049376	0.061684	0.043191	-0.06799	-0.16348	0.017854	-0.05409	-0.00509	0.742228	0.333103
0.124257	0.052746	-0.45309	0.119542	-0.43992	0.146717	-0.026	0.005087	0.744029	0.98778
-0.01952	0.099758	-0.31459	0.043046	-0.30382	0.251278	-0.02742	0.024177	0.939443	0.578721
-0.08113	-0.0487	0.090706	0.140888	0.197654	-0.07503	-0.01297	-0.00668	0.620231	0.248379
0.069029	-0.00619	0.238354	-0.16052	0.026386	-0.13393	0.031233	-0.00645	0.95679	0.705604
0.152003	0.117377	-0.33413	0.112322	-0.27136	0.057803	-0.06395	0.013433	0.942434	0.570036
-0.0839	0.147762	-0.39526	0.042903	-0.32978	0.142472	-0.09806	0.020815	0.79786	0.301599
-0.01002	-0.0787	0.410962	-0.05636	0.137187	-0.10903	0.019773	-0.02539	0.52418	0.492539
-0.08667	-0.09811	-0.08956	-0.10664	-0.02849	0.107227	-0.01459	0.038887	0.964286	0.74021
0.151458	0.035909	0.279476	-0.21331	0.071636	-0.10169	-0.04974	-0.01276	0.697069	0.767778
0.000454	-0.00126	-0.10522	0.02912	-0.21509	0.060407	-0.00692	0.011994	0.860115	0.375481
-0.03174	0.097702	-0.54417	0.085368	-0.29715	0.027326	-0.04104	0.010033	0.847625	0.616547
0.157193	0.077662	-0.30145	-0.03665	-0.2129	0.011089	0.000262	0.016313	0.993823	0.138108
0.124047	0.131048	0.04554	0.012107	-0.09722	-0.07395	-0.04372	-0.01099	0.623261	0.099066
-0.05636	0.02393	-0.13459	0.069265	-0.25844	0.196383	0.00287	-0.00057	0.887432	0.682132
-0.06333	-0.01534	0.262756	-0.15285	0.066476	-0.0468	0.047049	-0.0069	0.829111	0.836459
0.07167	-0.08893	0.101619	-0.02976	0.126444	0.09297	0.035661	0.004585	0.64751	0.705719
-0.10017	-0.02021	-0.15363	0.108957	0.010387	-0.00133	0.001111	0.00345	0.875742	0.965894
-0.02844	-0.08635	-0.14435	0.139048	-0.07254	0.048052	0.048	-0.0116	0.68611	0.670226
0.052584	-0.05345	0.395743	0.062817	0.078931	-0.03098	-0.0237	0.001659	0.731083	0.840269
-0.10239	-0.10291	0.841952	0.009107	0.16455	-0.03628	-0.00026	-0.00207	0.81734	0.488332
-0.07694	-0.01239	-0.02953	0.045601	-0.04452	-0.02778	-0.00121	0.00398	0.864592	0.738557
0.171633	-0.08794	0.260934	-0.03858	0.280235	-0.22317	-0.00015	0.001192	0.948874	0.023042
-0.07306	-0.10676	0.335887	-0.22607	0.245651	0.192506	0.047397	0.009018	0.749684	0.319974
-0.05653	0.025023	0.354988	-0.02496	0.153651	-0.05622	-0.03682	-0.02462	0.498501	0.093885
-0.07479	0.010764	-0.12938	0.194272	0.092422	0.078293	-0.06573	0.004982	0.639243	0.686422
-0.11136	0.009093	-0.09391	-0.00871	0.490728	0.022838	-0.07558	-0.00713	0.686291	0.116441
-0.03959	0.005321	-0.01301	0.01584	-0.01955	0.010396	-0.02526	0.108033	0.777969	0.629359
0.103454	-0.02961	0.018251	0.00066	-0.04815	0.043313	0.055761	-0.01729	0.548933	0.97426
-0.1046	-0.07222	-0.05834	-0.04758	0.675238	-0.05701	-0.00023	-0.00355	0.833239	0.636792
0.062364	-0.02633	-0.11356	-0.01778	-0.01699	0.028355	-0.04664	-0.00538	0.672312	0.953362
0.046012	-0.05799	0.216473	-0.04488	0.243321	-0.08949	-0.00197	0.009289	0.702664	0.098828
-0.10667	0.0281	0.09464	-0.06827	-0.03533	-0.01788	0.018775	-0.01116	0.762891	0.216376
-0.123	0.383492	0.050891	-0.01435	-0.04315	-0.15359	-0.06613	-0.00366	0.735134	0.043625
-0.0221	0.01914	-0.10382	0.175874	-0.08979	0.131512	0.080385	-0.08081	0.245416	0.764247

0.058233	0.119765	-0.2831	-0.00472	-0.09154	-0.05562	0.020657	-0.01187	0.656946	0.470095
-0.02815	-0.05142	0.377478	-0.04682	0.290478	-0.17141	0.037011	-0.00993	0.742237	0.932272
-0.10783	0.067097	0.114641	0.065515	0.264792	-0.02051	-0.04169	9.46E-05	0.692566	0.229668
-0.0082	-0.04408	0.063444	-0.03747	0.138369	-0.0394	0.012232	0.005699	0.678683	0.580092
-0.1117	-0.02051	-0.15038	0.135426	-0.17796	0.074576	-0.04906	-0.00145	0.620847	0.365988
-0.00962	0.044483	-0.14796	0.144896	0.01029	-0.03079	-0.00397	-0.02018	0.601662	0.958605
-0.08529	0.003891	0.038682	0.053983	0.089944	-0.0245	0.00141	0.002574	0.799973	0.771341
-0.07261	-0.04892	0.320496	0.026537	0.162755	-0.01991	0.005961	-0.00132	0.489739	0.20831
-0.07205	0.042367	0.074981	-0.08826	0.150592	0.008361	-0.01163	-0.00794	0.747254	0.636287
-0.11549	-0.04524	-0.02236	0.108363	0.261701	-0.01161	-0.03421	0.009863	0.519618	0.796079
-0.09898	-0.00826	-0.16372	-0.07748	-0.1159	-0.05464	-0.02734	-0.01034	0.804094	0.050168
-0.00364	0.073828	0.073083	-0.06624	0.167028	-0.01369	0.008341	-0.0391	0.542229	0.911742
0.11294	-0.06026	0.105197	-0.0788	0.085996	-0.03601	0.028159	-0.00434	0.590654	0.452711
0.129227	-0.19744	0.023241	-0.01468	0.102445	-0.07805	0.095333	-0.00052	0.71204	0.362265
0.012535	0.028172	0.411755	-0.03036	0.100695	-0.07011	-0.0055	-0.11502	0.214533	0.900469
0.025848	0.094002	0.166934	-0.14956	0.120808	-0.01508	-0.02499	-0.01788	0.585075	0.583967
0.344968	-0.13198	-0.15674	0.092894	-0.19704	0.067575	-0.00052	0.017435	0.864705	0.32265
-0.04691	0.028434	0.313133	-0.13315	0.262365	-0.11157	-0.03479	-0.00232	0.705416	0.214655
-0.43533	-0.03515	0.329937	0.100757	0.230844	0.08985	-0.02436	-0.00467	0.817335	0.012025
-0.17245	0.283763	-0.08521	-0.15155	-0.05198	-0.18055	-0.01507	0.010481	0.962001	0.785221
0.103473	0.167438	-0.29327	-0.08504	-0.21034	0.224988	-0.02757	0.007492	0.976978	0.764591
-0.01849	0.288642	-0.23507	-0.07281	-0.25228	-0.01494	-0.02746	0.001309	0.997883	0.222474
0.050114	0.004206	0.322004	-0.12623	0.114906	-0.12166	-0.01652	0.010961	0.697112	0.294873
0.245354	-0.01286	-0.06924	-0.05807	-0.02969	0.0809	0.089428	-0.00225	0.807575	0.051593
0.454094	0.027588	-0.27296	-0.12255	0.09624	0.096186	0.000636	0.016185	0.98328	0.987326
0.019679	0.051687	0.161129	-0.01842	-0.01992	-0.02953	0.005737	-0.02128	0.606462	0.626457
0.038289	-0.13953	0.048938	-0.03321	0.24701	-0.11465	0.01765	0.005657	0.962236	0.689292
-0.00917	-0.09537	0.169821	-0.0353	0.094761	-0.02858	0.097324	-0.01636	0.656462	0.313927
0.048683	0.096743	0.065512	-0.05729	0.046894	-0.09453	0.006061	-0.03052	0.539957	0.976507
-0.2864	-0.07165	0.152707	0.067146	0.428622	-0.12384	-0.02079	-0.00853	0.694024	0.290245
0.148359	-0.08918	0.288677	0.060246	0.122256	-0.10935	-0.00328	0.011544	0.871204	0.056212
-0.01912	-0.01214	0.048117	-0.03255	0.133912	-0.09162	0.026491	-0.00677	0.669945	0.472993
0.177827	0.023903	0.07492	0.010836	-0.12573	-0.00554	-0.05409	-0.00649	0.967529	0.839463
0.050079	-0.03741	0.311103	0.004584	0.16886	-0.09549	-0.00246	-0.00046	0.746457	0.321598
0.1208	-0.00674	-0.25726	-0.05174	-0.16924	0.103118	0.017737	-0.01248	0.380199	0.005502
-0.1697	0.045343	-0.10897	-0.12176	-0.03599	-0.03515	0.046772	-0.00287	0.766981	0.074712
0.072712	0.081562	-0.22196	-0.03882	-0.46242	0.035931	0.006088	0.017161	0.922313	0.291894
-0.1894	-0.08828	-0.03064	0.038312	-0.26673	0.124534	-0.07023	-0.00919	0.539444	0.359139
-0.11715	-0.00969	-0.23528	0.091187	0.145427	0.091504	-0.07521	-0.03601	0.513396	0.667661
-0.18545	0.04797	0.249104	-0.00218	-0.10471	-0.03617	-0.0639	-0.01329	0.662573	0.090699
0.328278	0.095619	0.043359	-0.08689	-0.3677	0.052113	-0.08238	0.016522	0.369221	0.481082
-0.14467	-0.00793	-0.32463	0.024798	0.106424	0.078838	-0.04214	0.033145	0.882534	0.666717
0.065034	0.085863	0.059721	-0.01046	-0.22681	0.101321	-0.09085	-0.01403	0.481278	0.427601
0.125467	0.087474	-0.16221	0.012015	-0.5091	0.154121	-0.07265	-0.00071	0.68479	0.399865
0.036784	0.052182	-0.31751	0.166834	-0.19262	0.17209	-0.01566	0.016485	0.556824	0.301526
-0.16605	-0.07036	-0.16929	0.064938	-0.15677	-0.03619	-0.00065	0.008102	0.899607	0.201813
0.075287	0.066661	-0.17492	-0.00384	0.057603	-0.07469	-0.04766	0.021894	0.83348	0.373265
0.026248	-0.03013	-0.12052	0.00886	-0.09519	0.198829	0.019792	0.023721	0.91113	0.619278
-0.19378	-0.09956	-0.10738	0.060493	0.412999	-0.05121	-0.00039	-0.02045	0.782013	0.400497
0.053759	-0.05876	0.154384	0.070313	-0.07837	0.025856	0.18693	0.005854	0.738177	0.130474
-0.10269	0.090909	0.217391	-0.06861	0.126827	-0.12482	-0.03335	0.004833	0.731304	0.728439

0.072922	-0.09145	-0.04988	0.303727	-0.07661	0.174053	0.029347	-0.118	0.616633	0.461159
0.003109	-0.07344	-0.05263	0.123961	-0.00753	0.111499	-0.05061	-0.02363	0.955397	0.43013
0.002471	-0.01314	-0.05174	0.104117	-0.083	0.11581	0.100136	-0.0218	0.231943	0.125165
0.025722	-0.11369	0.100146	-0.02277	0.160717	-0.04781	0.149202	0.000408	0.770448	0.333828
0.132235	-0.08538	0.058145	-0.00024	0.067285	-0.15316	0.059361	0.002822	0.651956	0.366303
0.062958	-0.00144	0.206859	-0.11762	0.240103	-0.12157	0.025154	-0.00934	0.636322	0.632516
0.066296	-0.02724	0.373415	0.013747	0.226286	-0.13919	-0.01656	-0.01649	0.891241	0.549467
0.222494	-0.02859	0.002619	-0.08055	0.024179	-0.06186	0.147207	-0.00272	0.928513	0.244323
0.167989	-0.00058	0.258056	-0.16322	0.181375	-0.12339	-0.00116	-0.05496	0.571876	0.958969
0.061221	0.30355	-0.07069	-0.05045	-0.08824	-0.10492	0.051201	-0.03403	0.705061	0.376725
0.173663	0.024369	-0.00756	-0.13405	0.010682	0.043855	0.063735	-0.05604	0.410344	0.827117
-0.06902	-0.00053	-0.08052	0.073701	0.074497	-0.15355	-0.01203	0.001356	0.937268	0.68818
0.079078	0.08444	-0.20164	0.006308	-0.11028	0.092897	-0.01556	0.005944	0.874647	0.315167
0.064773	0.023535	0.183056	0.067588	0.091212	-0.1308	0.013712	-0.00405	0.734806	0.501166
-0.00042	0.031136	-0.05561	0.02341	0.102341	-0.14977	-0.03244	0.028869	0.867354	0.975756
-0.07678	0.17358	0.04535	-0.15456	0.170455	-0.07811	-0.07225	0.011323	0.630721	0.681429
-0.08453	0.109281	-0.18255	0.012228	0.071977	0.110737	-0.07413	0.015062	0.876805	0.749205
-0.01508	0.049239	-0.13207	0.046136	-0.43327	0.087566	0.002987	0.022064	0.728437	0.115554
0.014648	0.040435	0.009271	0.095402	-0.09494	0.027519	-0.05895	-0.00062	0.804837	0.219355
0.007663	0.0391	-0.07173	-0.11061	-0.00907	0.095513	0.048819	0.016387	0.933353	0.912746
0.118376	-0.0035	-0.09819	-0.00668	0.058067	0.121931	0.008651	0.012088	0.354067	0.088287
0.068309	-0.05738	0.21651	-0.0143	-0.17124	0.149029	-0.00855	-0.01468	0.628449	0.068669
0.142703	0.069842	-0.01703	-0.13202	-0.01244	-0.04004	-0.03908	-0.01109	0.709951	0.47877
0.021822	-0.02717	0.188669	-0.0728	0.142691	0.009305	0.02525	-0.04149	0.512034	0.460659
0.302408	0.147209	-0.10125	-0.05277	-0.05623	0.139053	-0.23732	0.016424	0.491293	0.733697
0.219078	0.120955	0.060185	-0.01174	-0.11382	0.079783	-0.15365	0.004032	0.568057	0.848034
-0.00105	-0.00596	0.134267	-0.07529	0.121781	0.207534	0.027205	-0.04801	0.436804	0.698949
0.090337	0.100778	0.081168	-0.12152	0.177605	0.131292	0.000275	-0.0327	0.474766	0.519386
0.10949	-0.0825	0.413607	-0.0654	0.339596	-0.10347	-0.05856	0.00404	0.734726	0.711335
-0.00478	0.032124	0.062017	0.170021	0.079344	-0.03786	-0.05434	0.002503	0.843624	0.260805
-0.02895	0.169479	-0.24783	-0.09349	-0.29109	0.049203	0.229092	0.00433	0.693689	0.252814
-0.20558	-0.09053	0.285454	0.01447	0.255091	-0.0975	-0.04843	-0.00731	0.818492	0.296134
0.280549	0.105054	-0.16738	-0.03456	-0.20154	0.03178	-0.03818	0.013154	0.802838	0.526363
0.081184	0.073577	-0.30366	-0.07704	-0.26209	0.03325	-0.04492	0.002766	0.437886	0.024613
-0.0003	0.182777	0.428411	-0.19252	0.145989	-0.1967	-0.00187	-0.02383	0.69382	0.398401
0.102627	-0.00115	0.148978	-0.04112	0.150772	-0.1858	-0.01194	-0.00147	0.710247	0.960687
0.040606	-0.01436	0.160963	0.139029	0.115232	-0.0922	0.023276	-0.00134	0.934517	0.169975
-0.04647	-0.15987	-0.06941	0.131194	0.038382	-0.01392	0.04252	0.003795	0.741636	0.484484
0.02348	0.003115	0.262276	-0.06435	0.077372	-0.03736	-0.01146	-0.01067	0.884192	0.031648
0.108084	0.010903	0.40692	-0.16454	0.158595	-0.20397	0.025846	-0.00813	0.764593	0.533299
-0.19754	-0.05314	0.075611	-0.06362	0.226685	-0.14089	0.003075	-0.00242	0.91229	0.374321
-0.02627	0.047425	-0.16716	-0.0731	-0.09813	0.230704	0.000436	0.010142	0.961573	0.786199
0.019753	0.025462	-0.09046	0.074608	0.101938	-0.08671	-0.03143	-0.00142	0.949881	0.125776
0.030977	-0.03091	0.028021	0.051478	-0.08557	0.010347	0.004924	0.005915	0.792195	0.871949
-0.03862	0.051832	0.496766	-0.19327	0.26104	-0.14846	-0.01132	-0.01129	0.702357	0.499869
-0.14652	-0.04379	0.22609	0.083119	-0.1335	-0.03603	-0.00956	0.003146	0.461431	0.089133
0.03333	0.087336	0.096545	0.027609	-0.01258	0.002964	-0.01122	-0.00922	0.956912	0.95738
-0.07491	0.02191	0.026859	-0.15501	0.100555	0.27023	0.044624	-0.07323	0.191132	0.538508
-0.00569	-0.0347	0.055513	0.167143	0.094807	-0.02088	-0.03561	-0.05209	0.483084	0.459981
-0.01408	-0.05421	0.045095	-0.05594	0.143359	0.151688	0.076555	-0.05719	0.445872	0.85147
-0.09267	-0.02025	0.034266	-0.04572	0.003482	-0.06685	-0.00773	-0.03802	0.501075	0.671515

0.017129	0.020636	0.167206	-0.10961	0.176398	-0.21495	0.074215	0.045066	0.845552	0.611068
-0.01052	0.008064	0.253238	-0.08696	-0.01669	-0.04692	0.047392	-0.01446	0.738149	0.24487
0.054764	-0.02187	-0.12625	-0.05027	0.087178	-0.01625	0.045897	0.000706	0.939456	0.586251
-0.09599	-0.06022	-0.06037	0.253708	0.059606	-0.0255	0.014032	-0.01045	0.685835	0.558177
0.134116	-0.02847	0.405581	-0.02241	0.089217	-0.1009	-0.01313	-0.00065	0.942954	0.667596
0.09207	-0.0438	0.096168	0.024126	0.037616	-0.04363	-0.01031	0.007246	0.761129	0.502254
0.060065	0.029454	0.344761	-0.14787	0.213147	-0.10002	-0.01011	-0.02494	0.68668	0.93301
-0.10276	-0.14691	-0.04563	-0.14812	0.044349	0.004955	0.035778	0.009064	0.854623	0.612616
0.113836	0.228074	-0.17768	-0.17283	-0.24102	-0.00239	0.021883	0.007069	0.92074	0.211608
0.185024	0.222302	-0.27515	-0.08063	-0.33925	0.111646	0.001072	0.003774	0.881237	0.334627
0.18338	0.255916	-0.02582	-0.09424	-0.03989	0.076641	-0.04727	-0.06756	0.382956	0.093823
-0.01351	0.092361	0.035612	-0.13153	0.071132	0.266701	0.0238	-0.00331	0.812221	0.804825
0.046903	-0.04263	0.107065	-0.03692	0.022843	0.015666	0.006212	-0.00758	0.928671	0.828231
-0.03975	0.014854	-0.21477	-0.04744	-0.23337	0.107055	0.020775	0.020689	0.89986	0.282122
-0.04133	0.032966	-0.02407	-0.01009	-0.10425	-0.02731	-0.00094	0.031053	0.852362	0.591978
0.042235	-0.11969	0.123705	0.02266	0.27643	-0.0919	0.03617	0.000201	0.638608	0.855565
0.131672	-0.10551	-0.04561	-0.07369	0.405164	0.013363	0.098903	-0.00716	0.800978	0.732657
-0.04169	0.084534	-0.11944	0.056687	-0.06376	0.191078	0.029753	-0.04763	0.463245	0.736487
0.071359	0.070062	-0.17527	-0.00279	-0.31049	0.120206	0.010134	0.019336	0.832539	0.089817
0.336645	0.13267	-0.11774	-0.00539	-0.16482	-0.06905	-0.0408	-0.01035	0.470525	0.285234
-0.3676	0.00356	-0.25973	0.115548	-0.06591	0.109164	0.092432	0.030789	0.608406	0.964579
0.073176	0.059228	0.161574	-0.00239	0.216047	-0.10094	-0.07947	0.007823	0.791212	0.842871
0.014013	-0.05234	0.098273	-0.12824	0.088457	0.377018	0.007844	-0.01105	0.818075	0.180899
-0.02548	-0.13284	-0.12139	0.040976	-0.02971	0.161354	0.042302	0.011147	0.964339	0.813426
-0.21268	0.424974	0.579211	-0.06976	0.052293	-0.11124	0.003118	-0.0046	0.890606	0.23788
-0.07212	0.031533	0.509917	-0.17074	0.254253	-0.3348	-0.02469	-0.00594	0.78874	0.653259
0.012436	-0.04894	0.02916	0.084281	0.098731	-0.0362	0.027272	0.000889	0.647286	0.348542
-0.01168	-0.08694	0.238131	0.109829	0.172281	0.043304	0.019854	-0.00024	0.616168	0.526249
-0.02134	-0.01555	0.391442	-0.16581	0.283342	-0.10225	0.006849	-0.03478	0.659462	0.244052
-0.10448	-0.01518	-0.17319	0.095122	0.146382	0.003292	0.067946	0.060801	0.60566	0.677916
-0.29441	-0.13044	0.144462	0.095916	0.160559	0.122153	-0.03848	0.00406	0.759579	0.495943
-0.13457	0.079035	-0.00354	0.013222	0.092276	-0.05804	0.034891	0.022265	0.973585	0.954869
0.097399	0.048914	-0.03351	-0.27638	0.037652	0.096937	-0.00761	0.019107	0.937146	0.439872
-0.02757	0.034306	0.030203	-0.01201	-0.01569	-0.00545	0.02029	0.00365	0.750496	0.839161
0.111272	0.00015	-0.25992	-0.00392	-0.09472	0.129394	0.054231	-0.03126	0.529668	0.240475
-0.11934	-0.03958	0.098482	0.018011	0.010893	0.040358	0.047531	-0.03525	0.575674	0.611737
0.13457	0.111181	-0.15939	0.007358	0.076074	-0.06514	-0.05103	-0.00604	0.809849	0.515839
-0.03724	-0.01014	-0.07029	0.063787	-0.0448	0.027386	2.71E-05	-0.01388	0.830055	0.998081
0.02342	0.004747	0.014385	0.195997	0.12522	0.048195	-0.0075	-0.0351	0.451387	0.269794
-0.22487	-0.213	-0.10674	0.204115	0.188831	-0.08946	-0.00651	0.006559	0.906578	0.354237
0.004977	-0.02666	0.017401	0.015278	0.086929	-0.01635	0.023478	-0.0096	0.689055	0.695053
-0.05981	0.041959	0.0741	-0.01739	0.099682	-0.02222	-0.00178	-0.02865	0.717581	0.36769
0.032402	0.014765	0.216404	0.008586	0.002239	-0.09316	0.029334	-0.01604	0.589842	0.112644
-0.0359	-0.04705	0.340828	-0.18014	0.185756	-0.19598	0.035577	-0.01476	0.765453	0.568355
-0.1604	0.013049	-0.21365	0.023133	-0.39238	0.039337	0.015541	-0.00342	0.763759	0.677903
0.025974	-0.03317	0.460952	-0.08705	0.161079	-0.10342	-0.01335	-0.01008	0.840732	0.386285
-0.26435	-0.01204	-0.26743	0.221961	-0.042	0.139201	-0.00072	0.029554	0.984926	0.577147
0.098638	0.303971	-0.22063	-0.03599	0.041414	-0.0752	-0.06564	0.015917	0.975543	0.093048
-0.12558	-0.1842	0.189216	-0.02532	0.346253	0.035825	0.010378	-0.01662	0.116187	0.695777
-0.17145	-0.15666	-0.12642	0.15561	0.188333	0.209144	0.006192	0.001482	0.906389	0.259148
0.118934	0.170969	-0.06961	-0.00219	-0.56118	-0.02399	-0.05777	0.009625	0.345054	0.677891

-0.06567	-0.02074	-0.04055	-0.04085	0.20147	-0.0084	0.086669	-0.02504	0.519434	0.507967
-0.02158	-0.02883	0.008065	0.139313	-0.06087	0.022714	0.013729	-0.04101	0.529363	0.880271
0.106634	0.001638	-0.02836	-0.06638	-0.2666	-0.20069	0.060804	0.007483	0.4258	0.20917
-0.12186	-0.05652	0.147171	0.224565	0.084084	-0.01427	0.048732	-0.08004	0.640155	0.926378
0.002141	-0.0349	0.346947	-0.11823	0.156453	-0.03291	0.027805	-0.01465	0.70934	0.764902
-0.05888	-0.12656	0.178878	0.08642	0.240578	0.213481	0.026814	0.013289	0.876096	0.018275
-0.57592	0.00694	0.265623	0.057021	-0.13484	-0.23799	0.063602	0.002144	0.845839	0.781486
-0.0985	0.017797	0.009872	0.142195	-0.04074	0.089298	0.023539	-0.0116	0.721524	0.885691
-0.11927	-0.00357	0.43952	-0.11207	0.277974	-0.02592	-0.03477	-0.00049	0.66162	0.953997
-0.0673	-0.07202	0.025409	0.271326	0.103178	-0.02688	0.001658	-0.00524	0.665797	0.605
-0.09632	0.032477	-0.17823	0.157671	-0.53498	-0.03366	-0.02548	0.011989	0.652941	0.563271
-0.15365	0.032376	-0.06591	0.156617	-0.12811	-0.0407	-0.01935	0.010629	0.81176	0.970497
-0.15767	0.012141	-0.13604	0.220409	-0.36932	-0.05263	-0.04629	0.004683	0.510677	0.823373
-0.13216	-0.02264	-0.15598	0.246968	-0.15567	-0.02524	-0.01385	0.028567	0.928047	0.680877
0.201578	0.143603	-0.19861	-0.07651	-0.21611	-0.0786	0.00705	0.002421	0.845418	0.473691
-0.17674	-0.04306	-0.08905	-0.04484	0.574528	-0.05124	0.012213	0.000804	0.978501	0.460704
0.154453	-0.13056	-0.13406	0.124945	-0.28382	0.023214	-0.04206	-0.00474	0.673483	0.152956
-0.17423	0.027521	0.083893	0.190336	-0.17842	-0.05711	-0.03171	0.008033	0.618642	0.00253
0.190796	0.051574	-0.08931	-0.02269	-0.34713	-0.12111	0.013723	0.001599	0.846565	0.07078
-0.09552	-0.00941	0.102248	0.030781	-0.03568	-0.01761	-0.08369	0.016045	0.182567	0.947848
-0.13081	-0.06831	0.078942	0.187568	0.056092	-0.00196	0.058575	-0.00441	0.663408	0.124273
-0.4248	-0.11775	0.007318	0.129488	0.137167	-0.21279	0.017485	-0.00167	0.826201	0.127093
-0.12853	-0.08133	0.573167	0.057368	0.160749	-0.05734	0.001771	-0.00745	0.76941	0.376139
-0.13078	-0.08774	-0.00048	-0.03023	0.683163	-0.04106	-0.00393	-0.00798	0.814368	0.828567
-0.1162	-0.10876	-0.07825	-0.01717	0.653497	-0.03028	0.001226	-0.0133	0.800468	0.322588
0.106611	0.007885	0.251105	0.061169	0.163468	-0.15317	-0.03144	0.001257	0.680386	0.222345
-0.00994	-0.04355	0.213639	-0.04367	0.103431	0.005105	0.040509	-0.01564	0.579963	0.876189
0.080386	-0.1129	0.143665	0.082661	0.031798	-0.00656	0.065273	-0.00522	0.692027	0.667028
-0.00865	-0.08187	0.168989	-0.07234	0.238735	-0.00898	0.026396	-0.00459	0.717765	0.442277
-0.03812	0.015466	0.061403	-0.04868	-0.05649	0.001008	0.057679	-0.04116	0.563364	0.877961
0.0061	0.050085	-0.02386	0.012172	-0.03457	0.107949	-0.03291	-0.04107	0.548838	0.586723
-0.0209	0.087258	-0.04663	-0.04633	-0.05506	0.161884	-0.04874	0.024363	0.989849	0.925674
0.035743	0.038947	-0.09211	0.018817	0.111792	-0.01407	0.037273	-0.01555	0.584549	0.692258
0.020172	-0.0103	0.373443	-0.16669	0.173074	-0.1119	0.0056	-0.00991	0.750975	0.738391
0.104662	0.080066	0.366027	-0.12657	0.128569	-0.13817	-0.04543	-0.01115	0.804714	0.647569
0.038612	0.050706	0.124507	-0.08422	0.19569	-0.08473	-0.05446	-0.00236	0.658591	0.566957
0.033705	-0.02685	0.322485	-0.07691	0.188656	-0.14959	0.046294	-0.00219	0.620131	0.138882
-0.0327	-0.04067	0.176701	0.156202	0.076382	-0.13667	-0.00277	0.004338	0.708747	0.593244
-0.04906	-0.04777	-0.08404	0.149338	-0.02393	0.079375	0.0289	-0.00795	0.795512	0.672918
0.039498	-0.02443	-0.28818	-0.18292	-0.02539	0.108868	0.073221	0.020491	0.958053	0.396923
0.003943	0.00021	0.574855	-0.09243	0.085431	-0.14936	-0.00254	-0.01042	0.712526	0.590924
-0.01222	-0.02507	0.072625	0.132583	0.163681	-0.00224	0.019421	0.010992	0.647777	0.123478
-0.01148	-0.03736	-0.12309	0.029202	-0.06144	0.068809	0.064636	-0.02079	0.642625	0.384151
-0.03163	-0.07136	-0.12499	0.037011	0.395326	-0.06349	-0.00344	0.008323	0.852583	0.192045
0.051719	-0.00921	0.416873	0.046938	0.183168	-0.13224	-0.00517	-0.00943	0.778865	0.215204
-0.0776	-0.00564	0.031408	0.030665	0.043826	0.036107	0.064412	-0.05382	0.469176	0.920009
0.175171	-0.1358	0.063941	0.073162	0.045051	-0.10292	-0.02981	-0.01565	0.72459	0.211942
-0.00097	-0.09876	-0.06199	0.082045	0.188997	-0.02906	0.02859	0.018867	0.62961	0.215107
0.184541	-0.02559	0.137161	0.057329	-0.00026	0.02959	0.016649	-0.01315	0.7774	0.515029
-0.16765	-0.05465	0.259145	-0.12793	0.031102	-0.00384	-0.08564	0.002155	0.643255	0.699542
0.01198	-0.03325	0.36724	-0.06157	0.301401	-0.18377	0.027686	-0.00685	0.647644	0.397971

0.035742	-0.03654	-0.11895	-0.02109	0.062746	-0.02284	-0.02678	0.009009	0.782169	0.39412
-0.07982	-0.06704	-0.21648	0.144864	-0.10803	0.00817	0.015247	-0.00679	0.929771	0.114286
0.31272	-0.04535	-0.17522	0.052034	-0.10302	0.171842	-0.03778	-0.0213	0.652911	0.794288
-0.04517	-0.06129	0.037739	-0.11235	0.310296	-0.12231	-0.0491	0.033282	0.651714	0.432838
-0.27082	-0.03291	-0.13741	0.078147	0.022709	-0.00014	-0.05467	0.020193	0.445742	0.917455
-0.1083	-0.09416	-0.20818	0.155587	-0.16804	0.135185	0.01561	0.012141	0.606469	0.202111
-0.13864	-0.11042	-0.01589	0.078319	-0.0681	0.006973	-0.05777	-0.00186	0.475273	0.544124
-0.2168	-0.0362	-0.05815	0.155916	-0.27993	0.011369	-0.04995	-0.01288	0.720389	0.613616
0.423547	-0.25202	-0.16874	0.153003	0.096257	0.080414	0.006525	0.009677	0.602744	0.935599
-0.13674	-0.05996	0.162178	0.020362	0.113091	-0.00079	0.026887	-0.02499	0.4746	0.33981
-0.09923	-0.03888	-0.23212	0.114701	-0.05828	0.044755	-0.05353	-0.01844	0.41133	0.215022
0.066903	-0.03552	-0.41949	0.025281	-0.27572	0.193178	-0.07296	0.019636	0.589828	0.029918
-0.09439	0.316673	-0.16844	0.162221	-0.09264	0.069586	0.022446	0.001061	0.839896	0.771246
-0.18306	-0.20494	0.386786	-0.05893	0.072827	0.248777	-0.08172	0.010891	0.112	0.872304
0.068878	-0.03427	0.270271	-0.06313	-0.05337	0.046674	0.119416	-0.05585	0.401956	0.720684
-0.08726	-0.06286	0.076438	-0.05345	-0.01796	0.050267	0.052133	-0.02797	0.460803	0.844153
-0.06651	-0.1435	-0.15425	0.218002	-0.20595	0.044281	0.034017	0.027275	0.889944	0.497549
0.137046	0.073587	-0.07023	0.028723	-0.22469	-0.03554	-0.00081	0.054594	0.837715	0.85768
-0.24688	-0.04722	-0.09176	-0.04261	0.495522	-0.07387	-0.05363	0.010504	0.946702	0.186658
0.072426	0.075473	-0.12716	-0.00238	0.019467	0.010613	-0.05361	0.020557	0.926339	0.72599
-0.30993	-0.03165	-0.0722	0.03031	0.02952	-0.01283	-0.07169	0.031024	0.692736	0.644369
-0.15659	-0.17407	0.274581	-0.00575	0.36338	-0.08289	-0.14871	0.020472	0.846879	0.894168
-0.01061	-0.09139	0.116935	0.182331	0.024333	-0.04881	0.041377	-0.01084	0.80046	0.4598
-0.19137	0.236625	-0.13222	-0.12412	-0.08162	-0.14582	-0.01195	0.006855	0.971102	0.262785
0.023785	0.030398	-0.13968	-0.00752	0.099956	0.004483	-0.01901	0.026402	0.735117	0.621496
-0.01175	0.091812	-0.10469	-0.05399	0.078377	-0.18595	-0.01714	0.021144	0.952193	0.689682
-0.13387	-0.00687	-0.07046	-0.08731	0.279838	0.063165	-0.02669	-0.01653	0.893625	0.928626
0.160996	-0.09524	0.040561	-0.00376	-0.05615	-0.03083	-0.02909	-0.05734	0.598888	0.585894
-0.0605	-0.04556	0.072564	-0.03508	0.296572	-0.0796	-0.01746	-0.02998	0.973867	0.566454
-0.0366	-0.08856	-0.04799	0.161853	0.127125	-0.06432	0.063177	-0.00096	0.611293	0.056411
-0.11617	-0.157	-0.21042	0.042503	-0.0995	0.445235	0.036242	0.001183	0.920773	0.213844
0.063278	-0.02708	0.130529	0.099527	-0.00642	-0.05327	-0.01715	0.018598	0.634916	0.22826
0.077945	0.044285	0.480098	-0.04958	0.2066	-0.0672	-0.04579	-0.09219	0.296686	0.11176
0.167434	-0.02258	0.066611	0.034635	0.09245	-0.14669	0.008996	-0.00206	0.629825	0.093829
-0.08419	0.003676	-0.18941	0.091241	-0.30392	0.119527	-0.06166	0.003544	0.82896	0.832299
-0.16749	0.060956	-0.13624	0.138071	-0.07938	0.138996	0.022062	-0.05818	0.353614	0.26993
0.196325	0.080989	-0.55288	0.129127	-0.48897	0.143924	-0.033	-0.00842	0.645937	0.891872
0.087808	-0.08665	-0.17696	0.036238	-0.11965	0.023016	-0.02872	0.021247	0.851053	0.341157
0.115528	-0.02389	0.214805	-0.14566	0.121918	-0.15993	-0.00843	-0.00055	0.856545	0.875934
0.022236	-0.07203	0.202877	-0.12261	0.208948	-0.1466	-0.0482	0.010142	0.914683	0.096359
0.112702	0.065661	-0.13044	0.011868	-0.31184	0.128727	0.008376	0.044983	0.872375	0.927504
-0.00849	0.025374	-0.19029	0.240362	-0.38471	0.027	-0.01464	-0.02483	0.79977	0.935264
-0.13466	-0.09441	0.131818	-0.03335	-0.0077	0.297526	0.028414	-0.02572	0.625839	0.433062
-0.0481	-0.09398	0.195986	-0.01138	0.25869	-0.04783	0.040023	0.002643	0.749866	0.298151
0.020411	-0.03066	0.199275	0.002586	0.081433	0.086484	0.045312	-0.00379	0.736889	0.874705
-0.02142	0.010321	-0.01983	-0.00911	0.107434	-0.05078	-0.00351	0.002716	0.744619	0.79038
-0.05129	-0.13444	0.052532	-0.01068	0.206721	-0.17881	0.003316	0.023657	0.660069	0.78886
-0.47046	-0.19433	-0.00319	0.00181	0.380424	-0.06256	-0.02394	0.0052	0.464717	5.07E-06
-0.00831	-0.02179	-0.16684	0.196418	0.009491	0.085584	0.043672	0.005932	0.633082	0.230448
0.151762	0.048839	-0.25708	0.080772	-0.42016	0.103951	-0.05156	-0.0162	0.633121	0.227446
0.027529	0.001292	-0.00791	0.097424	-0.01145	0.056816	0.044033	-0.02152	0.490165	0.819911

0.042607	0.007176	-0.08633	0.108389	0.038285	0.062962	-0.03931	0.02795	0.801434	0.057564
0.093457	-0.03829	0.298102	-0.00139	0.239424	-0.13563	-0.01976	-0.00616	0.735218	0.143292
-0.04881	-0.00265	0.154151	-0.01314	0.151116	-0.12788	-0.02102	-0.02457	0.512628	0.629947
-0.0166	-0.03869	0.036467	0.132865	0.075757	0.026484	0.10738	-0.0085	0.590492	0.297596
0.016898	-0.02158	0.321013	-0.12944	0.227065	-0.05533	0.030056	-0.00026	0.734965	0.291164
-0.07413	-0.05537	-0.06226	0.006185	0.029443	-0.02409	0.016194	0.003642	0.967345	0.829172
0.323717	-0.07648	0.084214	-0.0364	-0.3852	-0.06021	-0.01466	-0.01488	0.681358	0.04011
0.031483	-0.12681	0.23625	-0.01448	0.216082	-0.08413	0.057899	-0.00561	0.652396	0.639906
0.041504	-0.09653	0.166766	0.101764	0.242319	-0.1365	0.03666	0.001857	0.821773	0.390538
0.114786	-0.16142	-0.2657	-0.00846	-0.14118	-0.07287	0.098083	0.001122	0.690853	0.001844
-0.0465	-0.05198	0.436645	-0.19694	0.209973	-0.03529	0.040735	-0.01042	0.751757	0.758202
-0.36653	-0.13456	0.284576	0.087451	0.250113	-0.13249	0.004045	-0.00201	0.749895	0.348488
0.012374	0.03858	0.139118	0.203926	0.10797	-0.11475	-0.05136	-0.00741	0.967249	0.134144
0.054297	-0.02389	0.021274	0.230266	0.106776	-0.0373	-0.04655	-0.00743	0.587569	0.152616
0.007279	-0.0354	0.053103	0.003308	0.256751	-0.06165	0.023293	-0.00451	0.595686	0.801933
0.017352	-0.15089	0.254766	0.068492	0.030306	-0.00713	0.073001	-0.00277	0.657249	0.443496
-0.04135	0.002358	0.043617	-0.01165	0.240264	-0.03844	-0.07232	0.013197	0.676658	0.917976
-0.04597	-0.06751	-0.10582	0.190587	-0.07769	0.18861	0.033293	-0.04196	0.554718	0.781958
-0.0408	0.048564	0.263268	-0.06352	0.218033	-0.07223	-0.01938	-0.00831	0.576956	0.803279
-0.18444	0.007972	0.004081	0.087609	0.236777	-0.21143	-0.04619	0.013484	0.826562	0.066539
-0.05192	-0.01689	0.130001	-0.01815	0.294551	-0.16242	-0.04346	0.007327	0.672595	0.733614
-0.04562	0.071037	-0.14307	0.112555	-0.23555	-0.25124	-0.02003	0.003012	0.827633	0.898255
-0.00525	-0.01885	0.086626	-0.03971	0.210473	-0.14258	0.068477	0.051443	0.176375	0.616009
0.057241	0.030214	0.252876	0.054283	0.234236	-0.17425	0.022264	-0.00559	0.64492	0.56525
-0.24464	0.110774	-0.07426	-0.08952	0.113278	-0.17526	-0.07141	0.011009	0.937552	0.047889
0.019384	0.009068	-0.09301	0.039821	0.062048	0.017189	-0.02586	0.005514	0.879974	0.980867
0.142362	-0.04161	0.062587	0.009977	-0.14374	-0.12319	0.000294	0.008799	0.846667	0.050224
-0.0432	0.068038	-0.30816	0.142853	-0.26355	0.065856	-0.03225	0.001323	0.751082	0.560586
-0.01781	-0.05091	-0.22198	0.080186	-0.35206	0.072344	0.073797	0.026684	0.882739	0.762683
0.052302	-0.01929	0.174681	0.070717	-0.12288	-0.06813	-0.09647	-0.00549	0.384491	0.739002
-0.06515	-0.01805	-0.23451	-0.01961	-0.16048	0.410381	0.030572	0.001801	0.977779	0.023721
0.046789	0.064565	-0.24323	-0.04577	-0.02063	-0.03147	-0.04651	0.016591	0.945443	0.37478
0.430248	-0.0005	-0.35989	0.064645	-0.47752	0.106146	-0.02839	0.011356	0.842598	0.506729
-0.08875	-0.044	-0.16925	-0.023	0.228977	0.054522	0.011628	0.022494	0.889029	0.336284
0.040569	0.11517	-0.29618	0.113687	-0.51603	0.079174	-0.0765	-0.01381	0.580999	0.715174
-0.14079	-0.09642	-0.26246	0.031416	0.40193	0.093148	0.031033	0.016371	0.877762	0.789299
-0.06433	0.13563	-0.18043	-0.04207	-0.19238	0.064803	0.006022	-0.00639	0.505943	0.909018
0.061398	0.025221	0.236354	-0.08709	0.209762	-0.10683	-0.00684	-0.00891	0.548032	0.6996
-0.05944	0.02148	-0.02412	0.012434	0.036043	0.009295	0.02087	-0.00548	0.720016	0.880998
0.012368	0.01725	0.306259	-0.13725	0.085168	-0.12519	0.009172	-0.00429	0.773845	0.445113
0.043201	-0.07326	0.332144	-0.08798	0.322325	-0.15021	0.044238	0.019752	0.952956	0.257848
-0.1293	0.292458	0.02133	-0.0711	0.000661	-0.03743	-0.00311	-0.02689	0.738902	0.347521
-0.11497	-0.04498	0.081728	0.206149	-0.15237	0.234571	-0.0034	-0.08452	0.168107	0.571803
0.048483	-0.0722	0.106874	0.069575	-0.11289	0.043473	0.118378	-0.07297	0.248974	0.840619
-0.03807	-0.05736	0.236839	0.053677	0.313382	-0.07756	-0.01973	-0.00502	0.658948	0.420509
0.047787	0.030684	0.213139	-0.02916	0.139827	-0.11873	-0.0391	0.000495	0.640679	0.389489
-0.01997	-0.12538	0.262299	0.066858	0.13708	-0.05944	0.010057	-0.00698	0.663671	0.476776
-0.20361	-0.0534	-0.0072	-0.10811	0.440467	-0.10861	-0.00328	0.011423	0.94883	0.41014
0.023379	-0.04419	0.231853	0.011783	0.124017	-0.0597	0.059722	-0.03237	0.620442	0.76927
0.035887	0.128986	-0.31438	0.024954	-0.26665	0.068776	-0.05381	0.032016	0.790231	0.371653
-0.09561	0.025966	-0.10117	-0.07468	0.012714	0.456991	-0.01675	0.029891	0.957087	0.509794

-0.16158	-0.05479	-0.12131	0.076339	0.106101	0.065181	-0.02709	-0.00101	0.771379	0.506579
-0.18806	-0.15273	0.244713	0.042845	0.33736	-0.12194	-0.02482	-0.00687	0.759369	0.823644
-0.28696	-0.18995	0.440938	-0.11638	0.389674	-0.14934	0.08583	0.007143	0.98096	0.9698
-0.04329	-0.0164	-0.01783	-0.00282	0.049151	0.041023	0.000817	-0.00182	0.862696	0.797915
-0.13444	-0.0388	-0.29942	0.084922	-0.14504	0.307202	-0.06049	0.000526	0.299126	0.007658
-0.14546	-0.09931	0.521311	0.061233	-0.00436	0.004386	0.006994	-0.04055	0.660377	0.058342
-0.05641	-0.08925	0.268416	-0.08233	0.185453	-0.04873	0.009609	-0.03522	0.497592	0.414196
-0.2313	-0.14571	0.077181	0.016906	0.27644	0.259827	0.045164	-0.00954	0.609136	0.460651
-0.33412	-0.18163	-0.04504	0.229237	0.132636	0.110286	-0.02081	0.009612	0.473886	0.529573
-0.22403	-0.07198	-0.0608	-0.00169	0.012835	0.132225	-0.05508	0.014937	0.356661	0.206714
-0.3069	-0.07026	0.076684	0.105159	0.315342	-0.08695	-0.0599	-0.00932	0.50506	0.667007
0.145174	-0.09897	0.129412	0.029841	-0.21984	-0.06791	-0.02187	0.027944	0.092174	0.00403
0.022171	0.020387	0.145679	-0.04714	0.125977	-0.07032	-0.06607	0.005507	0.790501	0.461636
0.07609	-0.10877	0.028717	-0.00262	0.002853	-0.04577	0.005091	-0.00734	0.610147	0.966826
-0.27515	-0.17301	-0.20329	0.056549	0.157925	0.294033	0.008684	-0.00391	0.382694	0.011974
-0.28164	0.008091	0.132902	-0.1017	0.179279	0.020943	-0.01693	0.000785	0.625592	0.280011
0.218719	0.080322	-0.20068	0.010958	-0.48445	0.093813	0.040675	-0.00081	0.952171	0.976745
-0.04994	-0.09444	0.022117	-0.05024	0.379618	-0.19649	-0.04191	0.008774	0.883669	0.352867
-0.00678	0.033552	-0.1679	0.096574	-0.3015	0.048776	-0.02467	0.006875	0.637911	0.909513
0.025935	0.149021	-0.04621	-0.11344	0.094889	-0.12962	-0.07104	0.017903	0.889455	0.555974
-0.26987	0.014463	-0.0978	0.04451	0.298381	-0.05443	0.091123	0.109397	0.37299	0.94033
-0.0418	-0.11091	0.141242	0.082767	0.090526	-0.04251	0.025634	0.000463	0.867509	0.992635
0.180482	0.070377	-0.13889	-0.03931	-0.14769	-0.00337	-0.05384	-0.0261	0.510269	0.631485
-0.11039	-0.02561	0.393129	-0.06111	0.292725	0.019541	-0.0754	0.000712	0.843219	0.403056
-0.17998	-0.05032	0.407187	0.012202	0.026262	-0.18449	0.021671	0.010332	0.816403	0.9764
-0.08296	0.066386	0.15912	-0.07497	0.104763	-0.07238	0.012962	-0.01504	0.781084	0.767678
0.115532	0.100718	-0.40557	-0.07334	-0.14467	0.125341	-0.06271	0.009871	0.915827	0.418639
0.533623	-0.12752	-0.15375	0.124098	-0.27234	0.302468	-0.05514	-0.01039	0.564014	0.40748
-0.23571	-0.12449	0.219859	0.029562	-0.12095	0.12158	0.045027	0.030842	0.642037	0.254932
-0.06039	0.002965	-0.10152	-0.04305	0.244341	-0.00158	0.048461	-0.01575	0.894059	0.770302
0.158505	-0.0569	0.311575	-0.03231	0.21665	-0.15842	0.01815	-0.01291	0.69253	0.459862
-0.12871	-0.04117	-0.22839	0.126045	-0.17375	0.058949	0.068636	0.00411	0.955396	0.780502
-0.01534	-0.09568	-0.12131	0.320889	-0.0326	-0.06577	-0.00045	0.042451	0.787857	0.256473
0.073293	0.021942	0.352352	-0.17362	0.08896	-0.1704	0.017944	-0.02235	0.533926	0.746773
-0.02967	-0.05269	0.207633	-0.05126	0.2269	0.017211	0.021814	-0.04134	0.677363	0.263904
0.615259	-0.16235	-0.18441	-0.08913	-0.12804	0.199327	-0.00227	0.037228	0.785271	0.055892
0.081443	-0.16413	0.225129	-0.17821	0.308815	-0.047	0.07122	-0.00413	0.572305	0.153112
-0.45481	-0.21307	-0.11588	0.032172	0.387728	-0.10136	0.038282	-0.00366	0.907668	0.264094
-0.05303	-0.06086	-0.17172	0.116273	0.098201	-0.00194	-0.04495	0.00107	0.666135	0.837132
-0.17814	-0.11624	-0.12644	-0.0655	0.239464	0.133467	-0.08284	0.055865	0.54037	0.844539
-0.06052	-0.116	-0.04095	0.151938	0.086953	0.172849	0.005085	-0.08375	0.299487	0.247336
0.008263	-0.03809	0.139463	-0.26185	0.091542	-0.04159	0.063285	-0.08289	0.941066	0.823651
-0.09008	-0.03663	0.170594	-0.0479	0.214255	-0.17464	-0.03785	-0.00588	0.598747	0.504632
-0.11266	-0.14124	-0.20475	0.247717	-0.14583	0.202515	0.107898	0.012875	0.953431	0.404262
0.066864	0.000365	-0.30033	0.18945	-0.12339	0.151848	0.018547	-0.05836	0.840416	0.193223
0.089045	-0.1031	0.363322	-0.02464	0.223559	-0.1293	0.0286	0.030061	0.612568	0.257903
-0.07604	-0.13433	0.02506	-0.08238	0.0091	-0.04708	0.033146	0.054244	0.976392	0.51376
-0.06789	-0.05439	-0.06177	0.057329	0.171526	0.13968	-0.03427	-0.06401	0.400434	0.508047
-0.00298	-0.01991	-0.18783	0.134112	-0.15813	0.056499	0.036318	-0.0038	0.55331	0.377972
-0.04149	0.046747	0.108591	-0.03762	0.072648	0.068161	-0.06136	-0.01596	0.622515	0.292154
0.085686	-0.08061	-0.13653	0.126399	-0.09269	0.221641	0.070249	-0.01582	0.72535	0.401981

0.037896	0.024023	0.151474	-0.01416	0.139269	-0.09684	-0.01943	-0.00943	0.558356	0.305499
0.140958	0.226217	-0.27252	0.150388	-0.20562	0.160455	-0.09757	0.100113	0.579572	0.339673
0.021168	0.045474	-0.02943	0.130541	-0.03516	0.031279	-0.00843	0.004181	0.789269	0.230891
-0.01165	0.032514	0.151985	0.022291	0.006913	-0.15677	0.008111	-0.01644	0.758426	0.437561
-0.16224	-0.11636	0.208099	-0.06902	0.322533	0.066977	0.012869	-0.01093	0.602927	0.364608
-0.14003	-0.06048	-0.03931	0.073728	0.053477	0.053022	-0.04496	0.01881	0.642388	0.734624
0.030866	0.009077	0.157496	-0.09395	0.073185	-0.17768	0.005524	0.00254	0.779945	0.595709
-0.04635	-0.01138	0.026199	0.104617	0.27209	-0.11654	0.003195	0.011275	0.680133	0.206574
-0.03114	0.072007	-0.18016	0.165161	-0.13351	0.117696	-0.07064	0.000514	0.644371	0.933272
0.067726	0.108267	-0.02046	-0.09187	-0.10094	-0.01188	-0.01676	0.000803	0.696868	0.218306
-0.18812	-0.03972	-0.21921	0.124667	-0.15308	-0.1118	0.003222	0.007541	0.97055	0.156303
-0.03799	-0.10148	-0.03032	0.017858	0.204702	-0.07853	-0.00106	-0.05597	0.967445	0.120609
0.008053	-0.0142	0.265466	0.01749	0.20938	-0.10737	0.017861	-0.03819	0.501308	0.402073
0.058418	-0.03982	0.379246	-0.00615	0.138437	-0.13088	0.01883	-0.00411	0.674604	0.335372
0.074196	-0.10413	0.301804	-0.09729	0.282284	-0.0736	-0.0073	-0.00653	0.831994	0.727478
0.020885	-0.07456	0.371193	-0.0275	0.30712	-0.15447	-0.02641	-0.01229	0.932852	0.445868
0.280672	0.102505	0.182592	-0.16214	-0.03534	-0.12762	-0.04991	-0.00306	0.632669	0.726395
0.089333	-0.03346	-0.0337	0.03178	0.063438	-0.10644	0.06427	0.015551	0.961446	0.744265
0.064366	-0.13781	0.066049	-0.02062	0.273046	-0.05323	-0.00181	0.005733	0.69983	0.46036
0.111148	0.034904	0.356294	-0.15465	0.171282	-0.22362	-0.0134	0.000989	0.711076	0.795753
0.044	-0.02106	0.073864	0.014144	-0.06332	0.014016	0.071367	-0.03149	0.796407	0.286956
-0.02112	-0.03915	0.054441	0.127144	-0.0396	0.116041	0.039805	-0.04867	0.403919	0.592256
0.043245	0.029586	0.264781	0.000971	-0.01795	-0.21385	0.002246	0.040642	0.989351	0.516567
-0.03518	0.097447	-0.44273	-0.03893	-0.34924	-0.09483	0.027568	-0.00497	0.694164	0.003366
-0.04876	-0.07294	-0.05842	0.210626	-0.05546	0.15516	0.065027	-0.06023	0.452308	0.658063
-0.03733	-0.15051	0.065554	0.101769	0.123978	0.123469	0.05225	0.006079	0.609058	0.264609
-0.07624	0.096117	0.323969	-0.11847	0.071792	-0.10859	-0.05392	-0.00652	0.641773	0.915901
-0.06817	0.008108	-0.0119	0.120223	-0.01581	-0.01942	0.00127	0.017244	0.738317	0.563635
-0.08532	-0.02776	0.293767	-0.10328	0.191438	-0.03481	0.004548	-0.00213	0.640601	0.459565
-0.3205	-0.12738	0.176453	0.065713	0.446922	-0.10573	0.014529	-0.0237	0.718607	0.274626
0.260968	0.071288	0.04979	-0.15191	0.123158	-0.10601	-0.00053	-0.00047	0.700162	0.620242
-0.14625	-0.00288	0.347457	0.042021	-0.0877	-0.04537	0.008221	-0.01408	0.634168	0.956838
0.16572	0.025513	-0.00969	0.013249	-0.01181	0.009323	-0.1087	-0.00406	0.526293	0.895922
-0.05359	-0.0313	-0.02423	0.156615	-0.04499	0.141662	0.021137	-0.07246	0.306002	0.636104
-0.07496	-0.0561	-0.00752	0.071256	-0.04938	0.165929	0.00993	-0.02738	0.618544	0.793981
0.05998	-0.00581	-0.27209	-0.01983	-0.18322	0.005914	0.009978	0.018103	0.956351	0.037038
-0.05891	-0.13854	-0.11833	0.024293	0.17715	-0.00109	-0.00526	-0.00077	0.376312	0.459386
0.408379	0.040476	-0.16861	-0.04587	-0.06501	0.056514	-0.03084	0.076842	0.730564	0.735138
-0.27618	-0.05339	-0.07314	0.130486	-0.18835	0.224342	0.029645	0.043235	0.857125	0.33012
-0.19587	-0.21298	0.3325	-0.09266	0.277309	0.241595	-0.0101	0.013214	0.888896	0.131601
-0.15002	-0.02944	0.393692	-0.06916	0.330108	-0.09215	0.016376	-0.01387	0.753395	0.873147
0.015397	-0.02623	0.094542	-0.0954	0.281894	-0.06661	-0.01768	0.008288	0.889161	0.907411
0.135171	0.057392	0.264416	-0.20591	0.198757	-0.16111	-0.0021	-0.00789	0.856971	0.386227
-0.23132	-0.08545	0.137081	-0.02007	0.250241	0.045243	-0.00629	-0.00812	0.651308	0.317528
0.199309	0.017865	-0.16752	-0.00395	-0.11444	-0.04624	0.16738	-0.03735	0.994127	0.292736
-0.02282	-0.0024	-0.10048	-0.14809	0.139362	-0.03545	0.038064	0.009645	0.47731	0.452156
0.053877	0.046206	-0.21391	0.085641	-0.29571	0.120426	-0.04734	0.021319	0.965248	0.164308
0.046141	0.031787	-0.2633	-0.05912	-0.23973	0.117107	0.004097	0.007173	0.922588	0.380921
0.176484	0.078506	-0.23874	0.013636	-0.53457	0.121453	-0.01031	-0.01086	0.301736	0.20368
-0.00209	0.073821	-0.17525	0.157963	0.03742	0.090933	0.104471	0.00736	0.494356	0.144325
0.062635	-0.15944	0.024616	-0.01148	0.261781	0.048587	0.0064	-0.00395	0.868385	0.052135

-0.0765	-0.10888	-0.00564	-0.10049	0.062729	0.040278	0.0925	0.022928	0.976201	0.453769
0.200064	-0.03908	-0.08683	0.100854	-0.06612	0.083644	0.039571	-0.06439	0.462266	0.736326
0.199351	0.096384	-0.5141	0.09449	-0.40405	-0.00277	-0.00314	0.003726	0.631715	0.332005
-0.10949	-0.07152	-0.0742	-0.02376	0.047203	-0.16568	0.012086	7.83E-05	0.896496	0.377777
0.131449	0.136953	-0.33208	0.001264	-0.06543	0.051884	-0.1139	0.015883	0.753088	0.890603
0.114245	0.061824	-0.19507	0.102437	-0.31334	0.029186	-0.07939	-0.00093	0.775379	0.659654
0.095346	0.108273	-0.21567	-0.00025	-0.39004	-0.0584	-0.05569	0.001154	0.55918	0.189764
-0.03691	-0.04027	0.451957	0.026036	0.251074	-0.08398	-0.06546	-0.03161	0.559194	0.73478
0.302118	0.001796	-0.08928	-0.08615	-0.01943	-0.09714	-0.001	-0.00744	0.696668	0.098869
-0.18884	0.152986	0.142541	-0.00665	-0.29594	0.037061	-0.00292	-0.00265	0.700798	0.001344
0.146474	0.003236	0.213499	-0.00627	0.246991	-0.17998	-0.00548	-0.00406	0.604202	0.157281
-0.03642	-0.04667	-0.00311	0.021078	0.019393	0.034937	0.042941	0.030381	0.947616	0.757878
-0.09429	-0.05676	0.342046	-0.06467	0.080436	0.055407	0.002243	0.00432	0.610479	0.475785
0.104457	0.061681	0.038133	-0.15654	-0.04817	-0.05952	0.043967	-0.01452	0.77921	0.475369
0.075505	0.00842	-0.21975	-0.0258	-0.26263	0.244414	-0.05274	0.014323	0.30649	0.004289
-0.03852	0.072935	-0.04064	0.176835	-0.30421	-0.03273	-0.08402	-0.01652	0.510696	0.596449
0.013976	0.02598	0.137861	-0.09029	-0.26762	-0.10452	0.032562	0.019245	0.485583	0.152889
-0.10908	-0.10363	0.505071	-0.03525	0.051472	0.112478	-0.00814	0.030111	0.944899	0.491887
-0.15274	-0.19465	0.351607	0.026131	0.246993	0.023048	0.011042	-0.0004	0.694078	0.900303
-0.10759	-0.06091	-0.1107	0.029968	-0.13179	0.032378	-0.01832	0.018637	0.586687	0.474526
0.033436	-0.05888	0.083024	0.124979	0.044571	-0.05288	0.024932	0.007038	0.712462	0.32123
0.209515	0.111864	-0.16342	-0.01577	-0.61972	0.080917	-0.03621	0.001367	0.668114	0.159558
-0.07763	0.007489	-0.19263	0.1269	-0.24262	0.19516	-0.04222	-0.00473	0.62665	0.792824
0.084969	0.02089	-0.06713	-0.07861	0.030148	0.121216	0.031127	0.006672	0.655201	0.206736
-0.01037	-0.08912	-0.07229	-0.13887	0.215194	0.022074	0.110747	-0.01842	0.696854	0.994574
-0.03855	0.182947	-0.03358	-0.02378	-0.0111	0.038037	-0.06339	-0.03944	0.53492	0.384715
0.01273	0.004643	0.435186	0.020857	0.197801	-0.06386	0.022607	-0.03394	0.512559	0.284069
0.112614	-0.00667	0.222822	0.006857	0.25078	-0.02608	-0.04547	-0.00236	0.940747	0.279711
0.038107	-0.02888	-0.13278	0.05278	0.113936	-0.03685	-0.03084	0.006897	0.977008	0.546911
-0.02402	-0.15842	0.221556	-0.02846	0.234905	0.007128	-0.0311	0.013388	0.873597	0.820708
0.043894	0.067256	0.191355	-0.06292	-0.00084	-0.02994	0.018189	-0.00403	0.505727	0.731137
-0.02798	-0.02797	-0.05567	0.032799	-0.02936	0.012337	0.064085	0.071484	0.783877	0.650925
-0.25818	-0.08562	-0.23454	0.205648	-0.16737	0.251024	0.004906	0.006921	0.971434	0.044827
0.379747	-0.01022	-0.23637	-0.01335	-0.20976	0.081527	0.034264	0.008871	0.943622	0.440197
0.020587	0.025981	-0.17741	0.014455	-0.28681	0.127292	0.01124	0.011569	0.765788	0.516387
-0.15883	-0.03803	0.056852	-0.07752	-0.05382	0.305163	-0.01498	-0.12288	0.273159	0.554136
-0.06146	0.082349	-0.34505	-0.02847	-0.31483	0.191779	-0.02062	0.018746	0.945919	0.842426
0.176139	0.042888	-0.0484	0.053326	-0.44094	0.13874	-0.00183	-0.00331	0.695346	0.648917
-0.05021	-0.133	0.151135	-0.00528	-0.03923	-0.0179	-0.06464	0.054695	0.759193	0.71021
0.036557	-0.04884	-0.12846	0.077199	-0.03385	0.073851	0.028677	-0.01387	0.631167	0.500725
-0.41609	-0.0592	0.223933	-0.06165	0.311055	-0.17234	0.088754	0.004625	0.787034	0.0078
0.007781	-0.03039	0.120643	-0.0594	0.110365	-0.03786	-0.01874	0.013225	0.723895	0.69274
0.025648	-0.06862	-0.08759	0.073334	-0.02209	0.059173	0.042687	-0.04798	0.52321	0.954143
0.019865	-0.04535	0.180381	-0.08474	0.058965	-0.0555	-0.04391	0.006881	0.560732	0.722137
-0.27532	-0.03519	0.045784	0.035863	0.029038	-0.04127	-0.03632	-0.00106	0.470672	0.725572
-0.28854	-0.19103	0.299301	0.023638	0.375241	-0.05387	-0.03652	0.046529	0.201315	0.102485
0.004536	-0.17035	0.385137	-0.03729	0.146646	-0.08531	-0.04758	0.002674	0.283126	0.720576
-0.17676	-0.07207	0.285944	-0.09554	0.278339	-0.09818	-0.07879	0.001006	0.385676	0.316062
-0.17004	-0.02743	0.295203	-0.12565	0.200357	-0.17725	-0.0412	0.009868	0.520137	0.249988
0.151863	-0.08766	-0.2807	0.041514	-0.18714	0.167657	0.000277	-0.01256	0.626801	0.000572
-0.18382	-0.08552	-0.15516	0.155448	0.000419	-0.10296	-0.06607	0.08428	0.977718	0.072462

0.019489	-0.06998	0.170059	-0.09467	0.114057	0.011752	-0.00501	0.009026	0.584648	0.055137
-0.075	0.03003	0.095868	-0.09873	0.195218	0.308127	0.014492	-0.01166	0.588414	0.208464
-0.05199	-0.03836	0.378387	-0.09092	0.292943	-0.01814	-0.00347	0.003557	0.807725	0.6025
-0.34276	-0.13911	0.189981	0.111571	0.123677	-0.16366	0.005177	-0.01685	0.907946	0.844439
-0.31982	-0.09529	0.20314	-0.05404	0.315582	-0.16232	-0.01146	-0.0006	0.680771	0.68174
-0.15202	-0.10051	0.529	-0.02507	0.212285	-0.16093	0.02975	-0.00666	0.967511	0.388243
0.052573	-0.01885	0.011126	0.068408	0.036281	-0.22835	0.045491	-0.01728	0.542077	0.650758
0.004538	-0.07101	-0.10384	-0.09846	0.315049	-0.09711	0.044944	-0.00143	0.827532	0.38242
0.025325	-0.0717	0.196245	0.07895	0.108949	-0.08925	0.019058	0.006533	0.68541	0.371745
-0.26845	-0.12028	0.113711	0.090732	0.149179	-0.05711	-0.01222	0.015347	0.997114	0.767141
-0.22402	-0.1124	-0.07918	0.070128	0.356853	0.127678	-0.0211	0.019671	0.961664	0.227598
-0.23681	-0.15537	0.308315	0.063853	-0.04287	0.046754	0.069222	0.020543	0.948713	0.43277
-0.16997	-0.15252	-0.09082	0.1645	-0.07388	0.064682	0.011731	-0.00184	0.928903	0.84807
-0.15682	0.127619	0.065294	0.154974	0.012571	-0.00883	-0.00789	-0.00261	0.632406	0.316087
-0.06201	0.073672	0.087131	0.078768	0.178646	-0.07473	-0.01054	0.004624	0.661195	0.856665
-0.3025	-0.0019	-0.06271	0.064491	0.225572	-0.24189	-0.03464	0.003781	0.988217	0.170028
-0.01386	-0.01349	0.055825	-0.10039	0.09215	-0.02995	0.017724	-0.00381	0.618619	0.62191
-0.05678	-0.09013	0.001683	0.22957	-0.05446	0.128616	0.038772	-0.05383	0.49368	0.982528
0.157001	0.146675	-0.19092	-0.16393	-0.25185	0.014898	-0.09389	0.071136	0.617329	0.083476
-0.0461	0.005787	0.320477	-0.11293	0.135858	-0.0401	-0.02297	0.010107	0.860189	0.911556
-0.05393	0.013067	0.054911	0.084607	-0.04167	0.019862	0.017789	-0.03569	0.63976	0.621671
0.095001	-0.00189	0.26199	-0.12706	0.092469	-0.07354	0.052659	-0.0445	0.471033	0.270144
0.032735	-0.00697	0.473	-0.06036	0.211316	-0.10005	-0.01937	-0.01011	0.71119	0.340916
-0.09682	-0.04639	0.035923	0.005905	-0.0407	0.091928	-0.01116	-0.01066	0.45524	0.630263
0.255765	0.092665	-0.19039	0.014832	-0.44333	0.045342	-0.03992	-0.00412	0.869037	0.799524
0.108411	-0.04795	0.319583	-0.17541	0.249314	-0.10341	-0.00389	0.005247	0.783609	0.631304
-0.10957	-0.00461	0.021527	0.032312	0.13868	0.036159	0.026394	-0.01455	0.577845	0.282058
0.009449	0.105564	0.20275	-0.30576	0.056315	-0.15476	0.019097	-0.00605	0.772064	0.705709
-0.1108	-0.06834	0.090091	-0.04666	0.218902	-0.10655	-0.00506	-0.02291	0.819366	0.806758
-0.04791	0.103267	0.096344	-0.12779	0.030014	0.107393	0.022129	-0.00397	0.764437	0.919065
-0.20056	-0.09222	0.032575	0.182998	0.36609	-0.13276	-0.00306	-0.00489	0.746173	0.303292
-0.13251	-0.0962	0.149173	0.076341	0.336966	-0.16567	0.063859	-0.00051	0.876859	0.581398
-0.18972	-0.03983	-0.10322	0.195025	0.323947	-0.1327	0.004347	0.006366	0.946439	0.381419
-0.15698	-0.07535	-0.17941	0.16884	-0.06939	0.310173	0.000664	0.0062	0.970096	0.212779
0.087458	-0.00102	0.355597	-0.11026	0.262875	-0.18506	-0.006	-0.00488	0.6737	0.535624
0.03217	0.110166	-0.37437	-0.0954	-0.09644	0.115555	-0.01619	0.008339	0.83462	0.024606
-0.05885	-0.00681	-0.02559	0.002418	-0.00522	0.196541	0.02591	-0.08304	0.623656	0.740236
-0.01848	-0.06615	0.111018	-0.13449	0.12268	-0.02346	0.02338	-0.03387	0.574309	0.731918
0.099345	-0.00556	0.213645	-0.07828	0.065031	-0.07331	-0.00347	-0.00216	0.848491	0.593795
-0.11427	0.123447	-0.16029	0.149209	-0.23964	-0.13409	-0.07551	-0.00116	0.961576	0.099074
0.200579	-0.02464	0.164578	0.055692	-0.01016	-0.07027	-0.0084	-0.00227	0.524358	0.255488
0.08566	0.063294	0.200562	-0.09831	0.189974	-0.22767	-0.00016	-0.00476	0.674964	0.923402
0.058042	-0.01602	0.30094	-0.02829	0.098044	-0.08486	-0.00952	-0.00891	0.822517	0.721106
0.158619	0.108373	-0.0687	-0.03316	-0.08296	0.141925	-0.08949	0.020997	0.959655	0.634545
-0.13377	-0.0099	0.081887	-0.09627	-0.09094	0.095095	0.057076	-0.05037	0.403875	0.59448
-0.06529	-0.08176	0.228248	0.202085	-0.01295	-0.02694	0.004622	0.001302	0.960466	0.809451
0.035707	0.009034	-0.00609	0.082961	0.078625	0.069864	0.018939	-0.05632	0.646726	0.517882
-0.08657	-0.07022	-0.02224	0.182819	-0.05259	0.192901	0.058326	-0.0542	0.016566	0.334009
-0.1208	-0.02737	0.117832	-0.11391	0.120014	0.017619	0.021748	0.002056	0.742191	0.961101
-0.06412	0.01066	0.025054	-0.04522	0.149485	-0.01367	-0.00791	0.017737	0.750098	0.779159
-0.05172	0.004274	0.326785	-0.10977	0.101775	-0.06039	-0.07413	0.001378	0.765256	0.500693

-0.08411	0.058699	-0.07296	0.082408	-0.13617	0.004094	0.001764	-0.02597	0.626633	0.539949
0.334097	-0.0136	-0.09589	-0.09289	-0.25885	0.138779	-0.02594	0.01419	0.752232	0.187484
-0.1583	-0.00912	-0.01764	0.14606	-0.26138	0.093193	-0.01767	0.011687	0.74045	0.718479
0.043518	-0.00837	-0.48857	0.103709	-0.39374	0.131203	0.044853	0.025951	0.813057	0.410034
0.027859	-0.02835	0.059758	-0.06022	-0.02357	-0.0423	0.112499	-0.02982	0.64789	0.50034
-0.203	-0.12099	-0.11512	0.104134	-0.03146	-0.14915	0.008602	-0.00513	0.84491	0.018295
0.007243	0.003495	0.252841	-0.00771	0.086313	-0.07615	0.00674	-0.00451	0.735509	0.573679
-0.06774	0.020831	-0.04986	0.100838	0.039184	0.045775	-0.00789	-0.01191	0.623587	0.483024
0.088508	0.170462	-0.07502	0.056282	-0.52331	0.009648	-0.01045	0.001421	0.772211	0.625665
-0.09667	-0.05596	0.180805	-0.09728	0.357681	-0.06905	-0.02145	-0.00051	0.921534	0.805157
-0.22528	-0.02911	-0.18778	0.061581	-0.1135	-0.13218	-0.00158	0.007479	0.961532	0.223841
-0.21423	-0.01619	-0.21735	0.161799	-0.13357	-0.10925	-0.00304	-0.00173	0.965012	0.248621
0.081624	0.059519	0.049324	-0.10156	0.128891	-0.1003	-0.0175	0.010451	0.584055	0.614688
0.042517	0.114479	-0.13869	-0.1673	-0.02283	-0.04866	0.031907	-0.00085	0.834552	0.615129
0.066683	-0.09573	0.283074	0.030591	0.006144	0.083094	-0.0941	-0.01361	0.255571	0.327807
-0.09273	-0.03454	0.345406	-0.0408	0.273983	-0.17434	0.026689	-0.00456	0.66858	0.316838
-0.10501	-0.03955	0.085221	0.059453	0.1353	-0.08327	0.018581	-0.00622	0.65247	0.402566
0.034078	-0.08745	0.175435	-0.0285	0.22071	-0.04814	0.094794	-0.00059	0.644633	0.15785
-0.04283	0.01077	0.390959	0.044636	0.080065	-0.13279	0.042918	-0.02199	0.62552	0.098343
-0.04979	-0.01575	-0.10681	-0.06536	-0.00186	0.538312	-0.00021	0.006149	0.901859	0.320766
-0.16048	-0.1213	0.175911	0.110547	-0.02013	-0.26719	-0.02177	0.012708	0.888058	0.044025
0.082698	-0.11419	-0.04652	0.152047	0.108412	-0.02068	0.073937	-0.01416	0.532329	0.991989
-0.01589	-0.02004	0.073291	-0.10078	0.100582	0.145767	-0.0811	0.001089	0.599051	0.428446
0.003974	-0.03189	-0.27134	-0.14688	0.05026	0.031798	0.098466	0.022206	0.535472	0.374738
-0.17073	-0.09898	-0.17812	0.063367	-0.02215	0.035443	-0.04557	0.007288	0.696507	0.336745
0.216616	0.209204	-0.24865	0.004641	-0.12202	0.074385	-0.07172	0.00646	0.944673	0.5779
0.005476	0.151493	0.178571	-0.13336	-0.04843	-0.03668	-0.07875	-0.01532	0.32864	0.242127
0.04372	0.014709	0.438588	-0.16484	0.158576	-0.16234	0.046351	-0.02696	0.633243	0.787607
0.000482	-0.01841	0.0256	-0.0589	0.300762	-0.05708	0.018409	0.011076	0.959578	0.603811
-0.01719	-0.00783	0.197689	0.044003	0.059334	-0.11143	-0.04313	0.006741	0.50711	0.812418
-0.09583	-0.12101	-0.17775	0.022693	0.09863	0.485241	0.066186	-0.04878	0.904075	0.139927
0.012302	-0.01088	0.230906	0.041537	0.195572	-0.0723	0.011457	-0.00361	0.584766	0.311375
-0.00218	-0.07406	-0.19211	0.292588	-0.07107	0.122218	-0.03255	-0.00403	0.855428	0.162998
-0.05807	-0.04638	-0.01116	-0.00861	-0.02039	0.126136	-0.00672	0.028412	0.884925	0.726333
0.098701	0.058563	0.159298	-0.04457	-0.0949	-0.05812	0.043758	-0.04129	0.495447	0.581985
-0.13698	-0.07264	0.176435	0.009383	0.584328	-0.02688	-0.04483	0.002324	0.880291	0.513373
-0.02491	0.061785	-0.35472	0.128944	-0.24692	0.031415	-0.06351	0.029761	0.871268	0.96463
-0.28396	-0.08951	0.562072	-0.00546	-0.01974	-0.12367	-0.0156	2.18E-05	0.991397	0.214595
0.048295	-0.01068	0.031614	0.014803	-0.01366	-0.03389	0.08071	0.008642	0.904211	0.820826
0.08794	0.074928	-0.08111	-0.0006	-0.27997	0.005013	-0.01528	0.019289	0.779614	0.384807
0.063892	-0.04062	-0.03136	0.032874	0.090879	0.023359	0.038013	-0.04915	0.474391	0.415217
-0.01627	0.020896	0.117219	-0.06305	0.088868	-0.16077	0.014535	-0.01922	0.689656	0.630513
0.113422	0.158712	-0.27217	0.062295	-0.52891	0.015968	-0.02498	-0.00095	0.708366	0.168776
0.185335	0.143417	-0.10212	-0.10662	-0.12648	0.034469	0.044104	-0.0133	0.626737	0.627619
-0.18323	0.120318	0.147609	-0.04994	0.083463	-0.03889	0.042073	-0.00506	0.625359	0.495532
0.001883	-0.01959	0.255147	-0.06048	0.290131	-0.13589	-0.01853	-0.03388	0.427099	0.96747
0.078712	-0.08283	0.285859	-0.05573	0.283335	0.0051	0.016657	-0.05891	0.261859	0.243281
0.107194	0.186887	-0.27972	-0.00073	-0.33514	0.1506	-0.05922	0.009732	0.779545	0.07363
0.055207	0.047448	-0.05508	0.015043	-0.16121	-0.09545	-0.00991	0.012469	0.943468	0.278649
-0.07735	-0.07224	-0.04905	0.097603	-0.02132	-0.03067	0.068532	0.030576	0.669633	0.793325
0.013144	0.045325	0.113143	0.005815	0.168707	-0.04995	-0.04743	-0.00614	0.82862	0.583284

0.039448	0.027991	0.170478	-0.0186	0.125641	-0.06566	-0.02393	-0.00052	0.717667	0.587146
-0.03056	0.056988	-0.04571	0.059077	0.10462	-0.03956	-0.04229	0.016538	0.794345	0.530666
0.189463	0.010793	0.039942	-0.06201	-0.19869	-0.02161	-0.05312	0.012845	0.938638	0.528007
0.091611	-0.01604	0.034105	0.171408	-0.15457	0.057536	-0.05756	0.012625	0.724562	0.280668
0.012953	0.062647	0.234136	-0.03282	0.002614	-0.10578	-0.01808	0.002759	0.672057	0.841583
0.098938	-0.03781	0.147152	0.058577	-0.18021	-0.04292	0.08488	-0.01969	0.916217	0.766321
0.101626	0.119107	-0.09161	0.041598	-0.14795	-0.10021	-0.0515	-0.00427	0.759207	0.246605
0.124528	-0.00572	-0.07092	-0.07053	0.042278	-0.07184	0.062199	0.02001	0.935666	0.327701
0.014434	-0.0922	0.138502	0.017362	0.126266	-0.00986	0.024041	-0.09522	0.920808	0.984825
0.025484	-0.0163	0.007313	0.122184	-0.20289	0.034826	-0.04758	0.030863	0.296966	0.934221
-0.03388	-0.04925	0.039485	0.055809	0.052732	0.085657	0.01352	0.016822	0.683848	0.151144
-0.22901	-0.00018	-0.23064	-0.0549	0.400524	-0.10707	-0.10005	-0.02579	0.391652	0.874121
0.140447	0.106902	0.030012	-0.18999	0.177807	-0.07043	0.08047	-0.0433	0.160771	0.419761
-0.17023	-0.02589	-0.21221	-0.01489	-0.1214	-0.1061	0.016562	0.077666	0.308815	0.791646
0.356613	-0.08196	0.031855	-0.10747	0.077531	-0.00262	-0.00121	0.002003	0.573307	0.233611
0.187636	0.260986	-0.31233	0.016979	-0.27563	0.070205	-0.05207	0.004153	0.850619	0.554916
-0.171	-0.02918	-0.03845	0.075943	-0.02838	-0.04447	-0.07709	0.007965	0.686886	0.627691
-0.03255	-0.06955	-0.04757	0.070861	-0.1765	0.378736	0.005187	-0.00892	0.73324	0.980557
0.098968	-0.06192	0.097335	-0.11503	-0.08	-0.07335	0.010486	-0.00572	0.536036	0.614008
-0.11938	-0.12252	0.157718	-0.12592	0.287381	0.164683	0.009068	-0.01957	0.791709	0.215944
0.045942	0.074295	-0.29495	0.00035	-0.40967	0.099224	0.040945	0.00408	0.678345	0.666218
-0.0693	0.047112	-0.06297	0.020403	-0.03114	-0.04397	0.028508	0.006862	0.922766	0.581602
-0.14714	-0.15412	0.391267	0.003306	0.434222	-0.11744	-0.00737	0.000649	0.750469	0.433079
-0.14862	-0.09264	0.114928	-0.04959	0.33966	0.038434	-0.00852	-0.00693	0.644389	0.811979
-0.05709	0.010869	0.031785	-0.19048	0.158485	0.005268	0.101166	0.018203	0.784526	0.101942
-0.29161	-0.09723	-0.13698	0.213575	0.292141	-0.08383	-0.0208	0.001151	0.649619	0.523584
-0.02619	-0.11211	0.073773	0.06351	-0.17331	-0.04527	0.009673	0.004656	0.767797	0.079867
-0.14664	-0.10069	0.388507	-0.17108	0.16782	-0.02188	0.002438	0.005869	0.535754	0.968916
0.135591	-0.08104	-0.02687	0.064265	-0.13119	0.097239	-0.01027	0.007688	0.93929	0.660711
-0.04629	-0.1743	-0.01042	-0.03901	0.095692	0.250901	0.030666	0.004461	0.706042	0.142397
0.019888	0.028132	0.136656	-0.14929	-0.18874	-0.04851	0.008349	0.007986	0.878175	0.106802
-0.24424	-0.13204	0.070433	-0.00275	0.148161	-0.18127	0.010381	0.028733	0.847159	0.537407
-0.06864	-0.07223	-0.09336	-0.02782	0.253946	-0.02193	-0.07702	########	0.636094	0.767697
-0.04281	0.217079	0.151849	-0.08414	0.041386	-0.00425	-0.04787	-0.00424	0.677925	0.224995
-0.08554	0.240412	-0.20911	-0.11847	0.245193	-0.02333	-0.10865	-0.00648	0.81808	0.994823
0.213515	0.134105	-0.49421	0.095659	-0.39025	0.101355	-0.0426	-0.0042	0.73498	0.730311
0.063159	-0.06608	0.043802	0.103993	-0.09697	0.148692	0.023268	0.010743	0.969441	0.603918
0.015958	0.024166	0.324518	-0.11659	0.218145	-0.0866	0.023664	-0.0088	0.75901	0.729689
-0.1446	-0.06095	-0.11725	0.099666	-0.16383	0.071162	0.034228	0.029392	0.821341	0.915775
0.09104	0.190897	-0.09197	-0.15479	-0.07882	-0.10917	-0.04542	-0.00521	0.926447	0.2068
0.114421	0.03616	-0.42282	0.037481	-0.47933	0.154892	-0.10151	0.010887	0.197744	0.006707
0.03082	0.008497	0.425963	-0.08543	0.226298	-0.11872	0.014464	-0.01172	0.684813	0.256319
0.030836	0.021739	-0.10975	-0.04967	-0.14194	0.027699	-0.04375	0.005759	0.73727	0.824723
-0.14963	-0.00804	0.167819	-0.00848	0.128054	0.20511	0.104614	-0.11048	0.165776	0.33496
-0.05125	-0.1195	0.052028	-0.10147	0.144978	-0.06909	0.119894	0.021183	0.874134	0.343665
-0.22864	-0.14514	-0.0717	0.126312	-0.14725	0.063319	-0.02962	-0.00925	0.552359	0.581097
-0.2585	0.090921	0.062551	0.055921	0.057758	0.023395	-0.07257	0.034103	0.900382	0.825836
0.132673	-0.06291	0.409074	-0.10576	0.144415	-0.14495	0.042991	-0.00677	0.662027	0.392963
0.362289	-0.27558	-0.37026	0.12311	-0.21062	0.082261	0.120499	0.011151	0.7997	0.383897
-0.05354	-0.09416	-0.07171	0.103715	0.150072	-0.10082	0.019858	-0.03482	0.806364	0.046819
-0.05557	-0.0725	0.048112	0.069784	0.026775	0.070213	0.049113	-0.03468	0.650548	0.812384

-0.25507	-0.22395	0.095287	-0.03194	0.317356	0.132807	0.071327	0.068801	0.87301	0.354293
0.218792	-0.06336	-0.22079	0.041808	-0.24478	0.067897	0.086431	0.008982	0.890176	0.756893
-0.06374	-0.06895	0.37357	0.016089	0.276378	-0.09792	-0.00122	-0.01175	0.738812	0.460006
0.080682	0.090795	-0.25754	0.06874	-0.38557	0.179891	-0.05729	0.000649	0.602272	0.192533
-0.08996	0.005311	-0.23558	-0.13842	-0.07665	0.43848	-0.00749	0.029052	0.995203	0.420383
-0.04804	0.010527	0.054554	-0.00605	-0.12215	0.007901	-0.00246	0.009385	0.811061	0.674614
-0.05894	-0.02721	0.036669	0.003637	0.014664	0.201983	-0.02545	-0.01216	0.545533	0.659147
0.077187	-0.05445	-0.07867	0.030229	-0.05002	0.193877	0.070929	-0.0899	0.223754	0.367185
-0.0004	-0.05738	0.03309	0.211687	-0.02268	-0.01556	0.019259	-0.02034	0.491136	0.225776
-0.29752	0.195118	-0.36362	0.136796	-0.28097	0.071131	-0.04055	0.002925	0.619932	0.91915
0.171299	-0.00062	0.001262	-0.04269	0.05059	0.013741	-0.02588	0.021594	0.781284	0.482983
-0.09731	-0.0017	-0.08349	0.17846	-0.07358	0.086604	0.093439	-0.03184	0.529021	0.421168
-0.0792	0.024052	0.177003	-0.0468	0.017875	-0.05087	0.079906	-0.03961	0.37831	0.387561
-0.11099	-0.05491	-0.0951	0.291953	-0.08001	-0.1521	0.070501	0.002027	0.87311	0.578299
0.054208	-0.02255	0.271346	-0.14375	0.164135	-0.09702	0.032915	-0.00066	0.728858	0.333913
-0.0836	0.045234	-0.04367	0.104836	-0.03844	0.0129	0.149362	-0.02242	0.610988	0.969578
0.066506	-0.12321	0.066336	0.130182	0.043904	-0.06007	0.067541	-0.0532	0.356924	0.622733
-0.05808	-0.05609	-0.0116	0.018486	0.144084	0.013245	0.008062	-0.00896	0.555604	0.349687
-0.08594	0.003093	-0.42174	0.246677	-0.2905	0.090006	-0.08025	-0.04606	0.59233	0.649845
0.150693	-0.04661	0.330485	-0.04976	0.218716	-0.07147	0.057612	-0.09182	0.452542	0.552124
0.240161	-0.01136	-0.22752	0.102855	0.212484	-0.31295	-0.02803	0.014733	0.454658	0.688617
-0.00212	-0.07633	0.069368	0.236958	0.180689	-0.00553	0.011667	-0.06525	0.526306	0.355148
-0.15788	-0.03887	-0.10708	0.027926	0.013894	0.166605	0.031115	-0.10128	0.434127	0.818939
-0.43335	0.027876	-0.42309	0.034693	0.037836	0.145123	0.039866	-0.04009	0.697347	0.008112
-0.15088	-0.05283	-0.20079	0.064507	-0.19991	0.226748	0.007844	0.034493	0.742161	0.551663
-0.05393	-0.14991	0.113794	0.11312	0.322476	0.079281	0.052581	-0.07175	0.284459	0.103654
0.141439	-0.05205	0.146825	-0.05915	0.273815	-0.06664	-0.01104	0.001906	0.647878	0.310876
-0.00992	-0.0668	0.127321	0.108591	0.155713	0.056173	0.025315	0.001756	0.618234	0.228159
0.084018	0.060278	-0.35657	0.123373	-0.57792	0.102179	-0.0008	0.023166	0.566049	0.295704
-0.06328	-0.01462	-0.23494	0.183155	-0.49005	0.152823	-0.06303	0.01234	0.290998	0.428402
-0.041	0.010643	-0.11913	0.174833	-0.56354	0.098904	-0.07619	0.006348	0.320752	0.570805
-0.02567	0.091019	-0.33473	0.17105	-0.32107	0.017904	-0.08012	-0.01384	0.543341	0.549215
-0.15819	-0.0254	0.070205	0.03352	-0.31066	0.181642	-0.02248	0.044931	0.394205	0.811801
-0.01871	0.005536	-0.33602	0.04549	-0.47539	0.066675	-0.02571	0.011412	0.483287	0.012578
0.120136	0.008845	-0.15558	0.115581	-0.44939	0.065565	-0.03424	-0.01526	0.578262	0.831979
0.156543	0.103338	-0.33476	0.056564	-0.41196	0.031257	-0.06437	-0.01146	0.656586	0.966048
0.010176	0.038449	-0.14816	0.093438	-0.6475	0.082482	-0.06056	-0.00189	0.517992	0.421972
-0.0845	0.139698	0.013844	0.091142	-0.34267	-0.08862	-0.11143	0.003941	0.486196	0.609278
-0.04956	0.013092	-0.16216	0.169878	-0.41227	0.026991	-0.10536	0.001529	0.201025	0.19366
-0.06967	0.026528	-0.12046	0.072666	-0.40493	0.058985	-0.11078	0.006522	0.248378	0.285453
-0.06014	-0.03992	-0.03952	-0.02935	0.344432	-0.09345	0.045065	0.004495	0.686072	0.561438
-0.05749	0.090178	-0.0216	0.045409	-0.53761	0.032942	-0.06135	0.01701	0.271816	0.438828
0.141445	0.098378	0.124685	0.001056	-0.29246	-0.13221	-0.07892	-0.01629	0.329864	0.639559
0.110377	0.018714	-0.11208	-0.05382	-0.37925	0.120433	-0.05406	0.033943	0.767354	0.625662
-0.06183	-0.10209	-0.12258	0.261512	-0.26656	0.127832	-0.06564	-0.00758	0.495134	0.825283
0.123307	-0.02578	0.039817	0.043936	-0.44882	-0.11845	0.01516	0.081747	0.455171	0.189053
-0.31322	-0.12871	0.103912	0.084263	0.145882	0.081243	-0.04157	-0.0021	0.437776	0.930622
0.081489	-0.05321	0.10379	-0.01614	0.074294	0.036056	-0.00115	-0.00364	0.708736	0.650419
-0.24721	-0.02445	0.170555	-0.17475	-0.05321	-0.12303	-0.0319	-0.00059	0.391861	0.24412
0.044828	-0.10388	0.243143	-0.01169	0.329207	-0.02428	-0.03605	-0.01153	0.958563	0.088254
0.035452	0.04189	0.58424	-0.04097	-0.14761	0.118254	-0.03468	-0.02529	0.670352	0.96203

-0.03341	0.058029	0.104428	-0.00855	0.052652	-0.06271	-0.0301	-0.0107	0.749426	0.822792
-0.25476	-0.17644	0.190766	-0.00721	0.422429	0.039768	-0.0269	0.003453	0.541839	0.404018
-0.26081	-0.14264	0.114301	-0.05184	0.036882	0.08563	-0.03345	-0.00158	0.41648	0.523388
-0.10881	-0.20559	0.093669	0.03966	0.242648	-0.03498	-0.03369	0.005496	0.621626	0.752661
0.119701	0.070425	0.320408	-0.16317	0.163719	-0.15342	-0.01323	-0.01241	0.763855	0.838745
-0.09492	-0.11293	0.079338	0.081349	0.138269	0.040015	-0.0008	0.004174	0.31964	0.645661
-0.05755	0.194563	-0.18599	-0.02463	-0.06712	-0.0441	-0.0398	-0.00215	0.883966	0.900898
-0.15087	0.056639	-0.10055	-0.01664	-0.01294	0.002067	0.070488	0.005524	0.795269	0.158538
-0.21096	-0.11266	-0.04083	0.008901	0.378846	0.006479	-0.07797	0.016607	0.649046	0.776703
-0.04045	-0.01764	0.028188	0.163501	-0.09747	0.030169	0.012594	-0.00614	0.518636	0.148385
-0.33397	-0.10472	0.111087	0.002133	0.351675	0.01549	-0.01602	0.007623	0.679457	0.679127
-0.1411	-0.10698	-0.09267	0.021606	0.179064	-0.02175	-0.04219	0.006918	0.598368	0.335843
-0.12114	0.011638	0.241575	-0.03794	0.000168	-0.03882	0.009867	-0.02776	0.512444	0.904369
-0.29767	-0.12117	0.047514	-0.05921	0.027171	0.015744	0.003986	0.00479	0.711199	0.001183
-0.24631	-0.13861	0.335413	-0.07593	0.230259	0.022733	-0.10239	0.01553	0.099657	0.143173
0.033701	0.071549	-0.29933	0.092404	-0.16005	0.039731	-0.03793	0.013862	0.962833	0.52547
0.269893	-0.017	0.081969	-0.18645	-0.12037	-0.02555	-0.02923	0.02191	0.420562	0.546448
0.078253	0.046426	0.109767	-0.1481	0.120094	-0.09149	0.055065	0.000184	0.760263	0.701674
0.017604	0.099698	-0.00256	0.045509	0.206821	-0.13227	0.034073	-0.01993	0.629905	0.692517
0.038977	-0.03058	0.047799	-0.0178	-0.06536	0.115142	-0.02224	0.014874	0.981572	0.618129
-0.106	-0.10847	0.263818	0.025354	0.193161	-0.07207	-0.00603	0.007958	0.890643	0.245235
-0.09495	0.091519	0.065814	0.083784	0.144258	0.012469	-0.12412	0.000569	0.736809	0.900406
0.115538	-0.1349	0.024039	-0.02542	0.116135	0.023001	0.053223	0.00576	0.857342	0.44035
-0.01747	0.048342	0.03392	-0.06958	-0.02001	-0.05385	0.022034	0.015673	0.767843	0.423652
0.046004	0.027937	-0.06569	0.133357	-0.15078	0.195065	-0.06037	-0.02396	0.768842	0.857732
-0.05336	0.262476	0.307816	-0.24417	-0.05746	0.006459	-0.05699	0.006976	0.952675	0.985255
-0.07569	-0.07009	0.030919	0.010067	0.080268	-0.36265	0.095052	0.067403	0.43869	0.300407
-0.26595	-0.12829	0.033433	0.179829	-0.04784	-0.17158	0.054607	0.009705	0.964555	0.016416
-0.05059	-0.09003	-0.05109	0.210223	-0.07426	0.142419	0.06036	-0.09542	0.313584	0.156142
-0.01067	-0.08087	-0.03552	-0.02704	0.009642	-0.07743	0.09705	0.006237	0.909329	0.055423
-0.04344	0.172015	0.041203	0.01359	-0.04587	-0.04731	-0.12701	0.045916	0.930367	0.686245
-0.04512	0.062319	-0.10898	0.025559	-0.19147	0.012909	-0.03206	0.011774	0.854924	0.923628
0.043896	-0.10759	-0.14668	0.175271	-0.14779	0.224306	-0.0277	0.006665	0.963248	0.105329
0.015768	0.030369	-0.05372	-0.02777	-0.1639	-0.03047	-0.03211	-0.01682	0.604563	0.395611
0.101023	0.072578	-0.49514	0.145651	-0.38899	0.126364	-0.05327	0.016676	0.778693	0.41981
0.112263	0.061549	-0.08817	0.001067	-0.46358	0.055239	-0.05905	-0.01318	0.476214	0.700627
-0.18444	-0.0684	-0.30069	0.045665	-0.2552	0.034954	0.005086	0.034221	0.902121	0.963976
0.09217	0.059951	0.27148	-0.1918	0.121466	-0.09785	0.013975	0.00605	0.81648	0.103349
0.227954	0.065626	-0.05329	-0.09111	0.014298	-0.01087	-0.0363	-0.02343	0.769964	0.157302
-0.29875	0.004485	0.165157	-0.1208	0.29997	-0.18415	-0.00495	0.025365	0.721961	0.87615
0.09264	0.054608	-0.09907	0.177626	-0.61091	0.096082	-0.07141	0.002311	0.572602	0.720745
-0.01993	-0.14395	0.037192	-0.06845	0.098614	-0.06794	-0.00121	-0.0065	0.296816	0.078153
-0.02834	0.043353	-0.11184	0.080902	-0.15388	-0.04991	-0.02505	0.023814	0.691353	0.786429
-0.03402	-0.09048	-0.07746	-0.01923	0.000762	0.022517	0.057965	0.015196	0.929525	0.294676
-0.13625	0.081773	-0.21575	0.138065	-0.21524	0.017703	0.064591	-0.0431	0.754724	0.709438
-0.03292	-0.10844	-0.06213	0.140782	-0.03404	0.23937	0.035186	-0.04997	0.450511	0.3494
-0.31234	0.095049	-0.10202	0.006228	0.225316	-0.00811	-0.00877	0.007375	0.303309	0.661213
-0.21128	0.024286	-0.22666	0.073338	-0.15612	0.097055	-0.05627	0.002692	0.591659	0.837762
-0.06155	0.068052	-0.23312	0.025152	-0.11279	0.048847	-0.00823	-0.00343	0.729733	0.65015
0.008965	-0.07257	0.216529	-0.06087	0.278097	-0.08488	-0.00051	-0.01403	0.579649	0.100516
0.23676	0.058511	-0.16785	-0.0455	-0.44171	-0.00368	-0.02059	-0.00051	0.640382	0.012673

-0.03836	-0.08678	0.070698	0.061938	0.147909	-0.04664	0.095459	-0.00648	0.588903	0.542394
-0.04187	-0.06295	0.140696	-0.07513	0.062937	-0.01644	-0.01126	-0.00669	0.790532	0.23009
0.027061	0.008686	0.306591	-0.00649	0.114966	-0.16244	-0.01711	-0.01015	0.761133	0.706314
0.11324	-0.077	-0.12208	0.163338	-0.2656	0.082937	-0.10591	-0.01645	0.318857	0.653034
-0.02498	-0.01343	0.168006	-0.01751	0.05251	-0.08688	0.026674	0.003055	0.935249	0.507265
-0.06835	-0.03764	-0.11561	0.086116	-0.04496	-0.02404	-0.02752	-0.03499	0.880258	0.192527
-0.01772	0.011438	-0.00534	0.029574	0.027358	-0.04417	0.00106	-0.00019	0.861865	0.872559
0.043125	0.016686	0.437202	########	0.222927	-0.15095	-0.0296	-0.01238	0.711091	0.570664
0.253854	-0.15868	-0.20266	0.217241	-0.00062	0.298391	-0.02568	0.083438	0.806266	0.362032
-0.13704	-0.01257	-0.25848	0.091982	-0.19934	0.326509	-0.05797	0.019955	0.787506	0.317008
-0.01701	-0.03305	-0.08352	0.023612	0.637615	-0.0446	0.023684	-0.04709	0.453089	0.28582
-0.13063	-0.06273	0.066971	-0.0178	0.400412	-0.01748	0.081279	-0.00338	0.663471	0.269261
-0.00181	-0.0613	0.110968	-0.08132	0.127114	-0.07529	0.026901	0.015786	0.671296	0.913847
-0.08169	-0.05001	0.097834	0.166553	0.015284	-0.04604	-0.00065	-0.02271	0.939334	0.413944
0.088913	-0.12019	0.227148	0.06112	0.096657	0.000584	0.027953	-0.09613	0.255514	0.867004
0.072447	-0.04031	0.345559	-0.03418	0.181971	-0.13377	-0.04742	-0.01249	0.851179	0.438028
-0.04871	-0.01025	0.246476	-0.03631	0.309861	-0.16407	0.023783	-0.0026	0.650364	0.439897
-0.01669	-0.02541	0.137462	0.025334	0.083013	-0.04713	0.034062	-0.01491	0.604396	0.500389
0.010993	-0.06352	-0.2061	0.102203	-0.20543	0.208764	0.062441	-0.04665	0.532282	0.950316
-0.02522	-0.00334	0.188128	0.024015	0.141058	-0.06885	0.042608	-0.03054	0.644769	0.290131
0.02255	0.103486	0.234137	-0.1775	0.05474	-0.01861	0.027662	-0.06394	0.42454	0.712186
-0.2411	0.096598	0.028251	0.09524	-0.05953	0.132928	-0.04079	0.02021	0.896113	0.620462
-0.11912	-0.00918	0.078783	0.041738	-0.13412	0.136126	0.097681	-0.05839	0.419052	0.232538
0.019669	-0.00115	0.249638	0.014957	0.115068	-0.09098	0.029907	-0.0049	0.75016	0.232145
0.055429	-0.00543	0.285445	-0.14439	0.21691	-0.19395	0.026617	-0.00423	0.669664	0.816421
-0.03429	-0.03424	0.129152	-0.18105	0.109916	-0.15862	0.05019	-0.00039	0.796579	0.475267
-0.05658	0.052451	-0.19	0.21173	-0.07819	-0.00828	-0.06107	-0.02392	0.582614	0.773315
0.233846	0.010924	0.183698	-0.05992	0.079659	-0.14253	0.036705	-0.01795	0.633842	0.697865
0.084962	-0.01229	0.277572	-0.08577	0.108169	-0.1333	0.027172	-0.00706	0.641196	0.313607
0.045275	0.020133	0.041376	-0.03081	-0.04273	0.085448	0.025079	-0.02054	0.691614	0.341089
0.049377	-0.01561	0.063965	0.164511	-0.04898	0.058394	0.026528	-0.02924	0.52098	0.650306
-0.14213	-0.08696	-0.10405	-0.04508	-0.08612	0.109107	0.130284	-0.01405	0.779091	0.846394
[truncated: 699,998 more chars]
